# Universal Ready-to-Use Immunotherapeutic Approach for the Treatment of Cancer: Expanded and Activated Polyclonal γδ Memory T Cells

**DOI:** 10.3389/fimmu.2019.02717

**Published:** 2019-11-22

**Authors:** Vinicia A. Polito, Rosaria Cristantielli, Gerrit Weber, Francesca Del Bufalo, Tamascia Belardinilli, Claudia M. Arnone, Andrea Petretto, Laura Antonucci, Ezio Giorda, Nicola Tumino, Angela Pitisci, Biagio De Angelis, Concetta Quintarelli, Franco Locatelli, Ignazio Caruana

**Affiliations:** ^1^Department of Paediatric Haematology and Oncology, Cellular and Gene Therapy, IRCCS Bambino Gesù Children's Hospital, Rome, Italy; ^2^Core Facilities, Proteomics Laboratory, Istituto Giannina Gaslini, Genoa, Italy; ^3^Core Facilities, IRCCS Bambino Gesù Children's Hospital, Rome, Italy; ^4^Immunology Research Area, IRCSS Bambino Gesù Children's Hospital, Rome, Italy; ^5^Department of Gynaecology/Obstetrics and Paediatrics, Sapienza University of Rome, Rome, Italy

**Keywords:** γδ T-cells, immunotherapy, adoptive T-cell transfer, anti-tumour effect, universal and ready-to-use cell product, anti-viral efficacy, engineered T-cells

## Abstract

In the last years, important progresses have been registered in the treatment of patients suffering from oncological/haematological malignancies, but more still needs to be done to reduce toxicity and side effects, improve outcome and offer new strategies for relapsed or refractory disease. A remarkable part of these clinical benefits is due to advances in immunotherapy. Here, we investigate the generation of a novel, universal and ready-to-use immunotherapeutic product based on γδ-T lymphocytes. These cells are part of the innate immune system, exerting potent natural cytotoxicity against bacteria, viruses and tumours. This ability, coupled with their negligible alloreactivity, makes them attractive for adoptive immunotherapy approaches. To achieve a cell product suitable for clinical use, we developed a strategy capable to generate polyclonal γδ-T cells with predominant memory-Vδ1 phenotype in good manufacturing practice (GMP) procedures with the additional possibility of gene-modification to improve their anti-tumour activity. Irradiated, engineered artificial antigen-presenting cells (aAPCs) expressing CD86/41BBL/CD40L and the cytomegalovirus (CMV)-antigen-pp65 were used. The presence of CMV-pp65 and CD40L proved to be crucial for expansion of the memory-Vδ1 subpopulation. To allow clinical translation and guarantee patient safety, aAPCs were stably transduced with an inducible suicide gene. Expanded γδ-T cells showed high expression of activation and memory markers, without signs of exhaustion; they maintained polyclonality and potent anti-tumour activity both *in vitro* (against immortalised and primary blasts) and in *in vivo* studies without displaying alloreactivity signals. The molecular characterisation (phophoproteomic and gene-expression) of these cell products underlines their unique properties. These cells can further be armed with chimeric antigen receptors (CAR) to improve anti-tumour capacity and persistence. We demonstrate the feasibility of establishing an allogeneic third-party, off-the-shelf and ready-to-use, γδ-T-cell bank. These γδ-T cells may represent an attractive therapeutic option endowed with broad clinical applications, including treatment of viral infections in highly immunocompromised patients, treatment of aggressive malignancies refractory to conventional approaches, bridging therapy to more targeted immunotherapeutic approaches and, ultimately, an innovative platform for the development of off-the-shelf CAR-T-cell products.

## Introduction

Although in the last decades remarkable progress has been recorded in the outcome of patients affected by oncological and haematological malignancies, many of them still either suffer from relevant toxicities or have relapsed/refractory disease. Innovative and less toxic treatment strategies are therefore urgently needed (1, 2). Emerging evidences highlight the possibility to strengthen the ability of the immune system to identify, seek out and destroy malignant and/or virally infected cells. Due to their well-known intrinsic immune-surveillance properties, components of the innate immune system might represent promising platforms for innovative immunotherapy approaches. Innate T cells are a heterogeneous population expressing a T-cell receptor (TCR) composed of γ- and δ-chains, exerting a cytotoxic activity against bacteria, viruses and tumours. Several clinical trials emphasised their therapeutic potential (3–6). For example, long-term remission of leukaemia amongst allogeneic haematopoietic stem cell transplantation (HSCT) recipients transplanted from a human leucocyte antigen (HLA)-disparate donor correlates with increased frequency of donor-derived γδ-T cells in patient peripheral blood (7). A potent *ex-vivo* anti-tumour activity of isolated γδ-T cells has also been clearly shown in patients after HLA-haploidentical αβ-T-cell depleted HSCT (8). Moreover, γδ-T cells have the peculiar ability of recognizing antigens in a MHC-independent manner (9) and are capable to activate macrophages and dendritic cells (10, 11). Their negligible alloreactivity makes them optimal candidates for the generation of a third-party, off-the-shelf and ready-to-use, T-cell bank.

Although these characteristics render γδ-T cells extremely attractive as platform for immunotherapies, their low frequency in peripheral blood (PB) poses a relevant limitation for clinical exploitation (12). Expansion protocols conventionally used to propagate αβ-T cells fail at initiating and sustaining γδ-T-cell growth (13, 14). To date, large-scale *ex-vivo* γδ-T-cell expansion is limited to the Vδ2^+^ population, particularly Vγ9Vδ2, which can be expanded through the administration of Zoledronic Acid (15, 16). The adoptive transfer of these cells showed clinical responses in the treatment of both solid and haematological malignancies (16–19). Other studies demonstrated the expansion of γδ-T cells using a particular cytokine cocktail supplemented with either anti-CD3 mAb (20) or plant-derived T-cell mitogens (21, 22) or artificial antigen-presenting cells (aAPC) engineered to express costimulatory ligands (23). However, these approaches showed a high variability in the expansion of the different γδ-T cell subpopulations (CD4, CD8, CD4–/CD8–, Vδ1, Vδ2, and Vδ1–/Vδ2–) and feasibility of gene-modification in order to improve persistence and efficacy against a broad range of tumours and viral infections. This was also observed in studies using aAPCs with the same costimulatory domains and protocols (23, 24). Moreover, a safety mechanism to eliminate aAPCs *in-vivo* has not been previously investigated.

Here, we describe a method to efficiently expand polyclonally-activated γδ-T cells using aAPCs expressing costimulatory molecules and the CMV-encoded protein pp65. In addition, in order to guarantee an optimal safety profile, aAPCs were stably engineered with an inducible safety switch. In a translational perspective, an automated protocol based on the use of the closed-system Clinimacs Prodigy has been developed. The polyclonal γδ-T cell products obtained harness a broad antigenic affinity and can serve as off-the-shelf, stand-alone treatment or as bridging therapy to more targeted immunotherapeutic approaches.

## Materials and Methods

### Cell Lines

All tumour cell lines except CHLA255 (25) were purchased from ATCC or DSMZ. The leukaemia and lymphoma cell lines, Daudi, BV173, MV4:11, RS4:11, 697, OCI-AML3, Raji, Karpas299, HDLM2 and K562 were cultured in RPMI 1640 supplemented with 10% foetal bovine serum (FBS) and 2 mM Glutamax (Thermo Scientific, Pittsburgh, PA, USA). The neuroblastoma (SHSY5Y and IMR32), glioblastoma (U87 and U373), and medulloblastoma (Daoy) cell lines were cultured in DMEM (Thermo Scientific) supplemented with 10% FBS and 2 mM Glutamax. The CHLA255 and the 293T cell lines were cultured in IMDM (Thermo Scientific), supplemented with 10% FBS and 2 mM Glutamax. In selected experiments, freshly-isolated B-cells were obtained from healthy donors (HD) using CD19 microbeads (Miltenyi). Cells were maintained in a humidified atmosphere containing 5% CO_2_ at 37°C. All cell lines were routinely tested for mycoplasma infection and authenticated by means of short tandem repeat analysis (Eurofins Genomic, Ebersberg, Germany). De-identified, frozen primary Acute Lymphoblastic Leukaemia (ALL)-B and Acute Myeloid leukemia (AML) primary blasts were obtained at diagnosis as approved by institutional review board of Bambino Gesù Children's Hospital (OPBG).

### aAPC Generation

aAPCs were generated using the K562 cell line. These cells were first transduced with lentiviral vectors encoding either human pp65/Enhanced Green Fluorescent Protein (eGFP) or CD40L/pp65. Then, cells were additionally transduced with a retrovirus vector encoding human CD86, 4-1BBL and inducible caspase-9 (iC9). After transduction, single cell clones were obtained. aAPCs were used as feeder cells at a ratio of 1:2 (γδ-T:aAPCs) and irradiated at 100 Gy before use.

### Vector Design and Transient Transfection

Retroviral and lentiviral vectors were generated to stably transduce K562, an immortalised leukaemia cell line. The retroviral vector was designed to encode the cDNA of CD86, 41BBL and the iC9 suicide gene using SFG retroviral backbone (25), while the lentiviral vectors encode the cDNA for the CMV-pp65 with or without the costimulatory molecule CD40L. An additional retroviral vector encoding eGFP-Firefly-Luciferase (eGFP-FFLuc) was used to label tumour cells for *in-vitro* and *in-vivo* studies, as previously described (26, 27). For gene-modification, 1 × 10^6^ expanded γδ-T cells/well in a non-tissue culture treated 24 well plate were transduced 4–5 days after II° restimulation using retronectin-coated plates (1 μg/well, Takara Bio, Shiga, Japan) with 1 mL/well of a retroviral vector encoding for a third-generation chimeric antigen receptor (CAR) (28) specific for GD2 (CAR-GD2.CD28.4-1BBζ) (29) (Supplementary Figures 1B,C). After removal from the retronectin-coated plates, transduced γδ-T cells were stimulated with aAPCs in bioreactors in the presence of IL2 and IL15 as described below. The transduction efficiency was determined by anti-CAR idiotype staining (1A7) as previously described (30).

### *Ex-vivo* Isolation and Expansion of Polyclonal αβ- and γδ-T Cells

PB mononuclear cells (PBMC) were isolated from buffy coats (BC) obtained from HD at OPBG after informed consent was signed, in accordance with the rules set by our Institutional Review Board (Bambino Gesù Children's Hospital—Ethical committee, Rome, with prot. N°969/2015), using Lymphoprep^TM^ density gradient medium (Eurobio, Courtaboeuf, France). αβ-T lymphocytes were activated with immobilised OKT3 (1 μg/ml, e-Bioscience, San Diego, CA, USA) and anti-CD28 (1 μg/ml, BD Biosciences, San Jose, CA, USA) monoclonal antibodies (mAbs), while γδ-T cells were isolated using γδ isolation kit (Miltenyi, Bergisch-Gladbach, Germany) and stimulated weekly with irradiated aAPCs at a ratio of 1:2 (γδ-T cells:aAPCs) in G-Rex bioreactors (Wilson Wolf, Saint Paul, MN, USA). T cells were expanded in medium containing 45% RPMI 1640 and 45% Click's medium (Sigma-Aldrich, St. Louis, MO, USA), supplemented with 10% FBS and 2 mM Glutamax in a humidified atmosphere in the presence of IL2 (100 U/ml, R&D, Minneapolis, MN, USA) for αβ-T lymphocytes; for γδ-T cells IL2, IL12, IL15, and IL21 were added during the initial stimulation with aAPCs, while only IL2 and IL15 were used for the following stimulations with aAPCs (weekly) and intermittent cytokine feeds (every 3–4 days) carried out as half-media changes. [IL2: 50 U/ml (R&D); IL12: 30 U/ml (Miltenyi); IL15: 250 U/ml (Miltenyi); and IL21: 0,6 U/ml (Miltenyi)]. For re-stimulations, the aAPC:γδ-T cell ratio was recalculated taking into account the number of expanded live cells. In selected experiments, on day+4 from the stimulation, the dimerising agent AP1903, kindly provided by Bellicum Pharmaceuticals, able to activate the iC9 safety switch, was added to the co-culture at a concentration of 100 nM (26). To prove the stability of expanded γδ-T cells after freezing, these cells were frozen in aliquots of 20 × 10^6^ cells/mL/vial and cryopreserved using cryostore solution (Sigma). Cells were then thawed in CTL media and cultured as previously described. In selected experiments, Vγ9Vδ2 T cells were generated from total PBMC derived from buffy coats. PBMCs were re-suspended in RPMI medium and cultured with 5 μM Zoledronic acid (Enzo Life Sciences, Farmingdale, NY) and 50 U/mL IL2 (Miltenyi). Proliferating T cells were maintained in IL2-containing medium for 15–18 days (8). Since we never obtained a purity higher than 60%, a γδ-T cell selection was performed before carrying out the functional assays.

### Co-culture Assay

For co-culture experiments, αβ- or γδ-T cells were plated at the indicated E:T ratios with or without supplementation of IL2/15 in the presence of eGFP-FFLuc tumour cells. Following 3 and 6 days of co-culture, cells were collected and residual tumour evaluated by flow-cytometry (25, 31).

### Phenotypic Analysis

Expression of cell surface molecules was determined by flow-cytometry using standard methodology. Anti-human mAbs were purchased from BD Biosciences, Miltenyi, eBiosciences. Samples were analysed with a BD LSR-Fortessa X-20 and analysed by FACS-Diva software (BD Biosciences). For each sample, a minimum of 20,000 events were acquired.

### Chromium-Release Assay

The cytotoxic activity was evaluated using a 4 or 6 h ^51^Cr-release assay, as previously described (25).

### Degranulation Assay

Effector and target cells were co-cultured at a 1:1 ratio with anti-CD107a (1.2 μg/ml) mAb and Brefeldin A (1 μl/ml according to the manufacturer's instructions) for 4 h. After incubation, cells were stained with extracellular mAb (32), washed and acquired by flow-cytometry.

### Cytokine Profile

Supernatants collected after 24 h of co-culture were analysed using immunoassays incorporating magnetic microsphere technology (Merck, Darmstadt, Germany), according to the manufacturer's instructions, as previously described (29).

### IFNγ Enzyme-Linked Immunospot Assay (ELISpot)

The IFNγ ELISpot assay was performed as previously described (33). Briefly, 5 × 10^4^ T cells were plated, stimulated with peptide or allogeneic normal or EBV-infected CD19^+^ cells and incubated for 16 h at 37°C. Plates were developed and spots counted with an Elispot reader (Aelvis, Hanover, Germany).

### Phosphoproteome Analysis

The phosphoproteomic samples were prepared as previously described (29) with minor changes in the detection system and the method to interpret the quantitative data. The detailed description of the methods and analysis can be found in the Supplemental Method file.

### Facs Cell Sorting

Expanded γδ T-cells were stained with Vδ2 Pe-Vio770 (Miltenyi) and Vδ1 FITC (Miltenyi). Cells were sorted to a purity of ≥94% with the MoFlo Astrios sorter (Beckman Coulter, Brea, CA, USA) using the Summit software (Beckman Coulter). The purity of cell separation was controlled by flow-cytometry (BD LSR-Fortessa X-20) and analysed by FACS-Diva software (BD Biosciences) using the same mAb used for cell sorting. After sorting, Vδ1, Vδ2, and Vδ1^neg^Vδ2^neg^ T-cells were rested overnight and used for functional studies.

### OpenArray

Pre-designed TaqMan OpenArray human inflammation and signal transduction panels were used according to the manufacturer's instructions (Life Technologies, Camarillo, CA, USA). cDNAs were loaded onto the OpenArray card and run on the QuantStudio 12K Flex Real-Time PCR system (Life Technologies). RQ of gene expression values were calculated using Thermo Fisher Cloud Resources. RQ values of the target genes in the different samples were normalised against a pool of their respective endogenous control genes and the fold induction calculated against the mRNA levels in the respective controls. RQ minimum and maximum values were calculated with a confidence level of 95%, using Benjamini-Hochberg false discovery rate to adjust *P*-values. Maximum allowed Ct included in calculations is 35 and Cq confidence >0.8. Multivariate Student's *t*-test was used and *p* < 0.05 were considered to be statistically significant.

### Automated γδ-T Cell Expansion

In order to transfer the protocol to an automated system, feasibility tests were performed combining the Automacs pro-separator and the Clinimacs Prodigy (Miltenyi) using a modified MoDC program. γδ-T lymphocytes were isolated from BC by depletion on the Automacs pro-separator using anti-CD19/CD14/CD56/αβ-T cell beads (Miltenyi). The negative fraction was transferred to the Clinimacs Prodigy for the culture adapting the manual protocol. The Clinimacs Prodigy tubing set (Miltenyi) was set up before starting the procedure. At day 0 isolated γδ-T cells and irradiated aAPCs (100 Gy) were co-cultured in a MACS GMP cell differentiation bag (Miltenyi) in 75 mL of TexMacs medium (Miltenyi) supplemented with IL2, IL15, IL12, and IL21 premium grade cytokines (Miltenyi) (at the same concentration used for the *in-vitro* expansion). At day +4, medium supplemented with IL2 and IL15 was added to the culture. At day +8, a second stimulation with irradiated aAPCs was performed adding fresh medium supplemented with IL2 and IL15. At day +12, fresh medium supplemented with IL2 and IL15 was added to the culture. At day +18, the culture ended and γδ-T cells were re-suspended in 100 mL of medium. Clinimacs PBS/EDTA (Miltenyi) supplemented with 0.5% of human serum albumin was used for the entire procedure as washing buffer.

### Xenograft Mouse Model

*In-vivo* experiments were performed in accordance with national and international ethical requirements and were approved by the Italian Ministry of Health (N°88/2016-PR). A previously described NSG mouse model (25, 26) was used to assess the *in-vivo* anti-tumour effect of αβ- and γδ-T cells. Seven-ten week-old mice were injected intravenously (i.v.) with Daudi-FF.Luc^+^ cells (2 × 10^5^/mouse). After tumour engraftment, T cells were injected i.v. (5 × 10^6^ or 2.5 × 10^7^ cells/mouse). Tumour growth was monitored by *in-vivo* bioluminescence using the Xenogen-IVIS Imaging System (PerkinElmer, Waltham, MA—USA), as previously described (25, 34). Mice received IL2 and IL15 administrations every 3/4 days for the entire duration of the treatment. Mice were euthanised when the veterinarian detected signs of discomfort or graft-vs.-host disease (GvHD), such as weight loss >15%.

### Statistical Analysis

Unless otherwise noted, data are expressed as average ± standard error mean (SEM). One-way ANOVA with correction for multiple corrections was used to determine statistical differences between samples, with *p* < 0.05 indicating a significant difference. Mice survival data were analysed using the Kaplan-Meier survival curve and Fisher's exact test. No samples were excluded from the analysis. Neither randomisation nor blinding was done during the *in-vivo* study. To compare the growth of tumours over-time, bioluminescence signal intensity was assessed blindly. For phosphoproteomic studies, label-free quantification experiments were statistically evaluated with Perseus software (http://www.perseus-framework.org) (35) as previously described (29). All *t*-test FDR values < 0.05 and S0 > 0.3 were considered to be statistically significant. Graph generation and statistical analyses were performed using Prism version 6.0d software (GraphPad, San Diego, CA, USA).

## Results

### Potent *ex-vivo* γδ-T-Cell Expansion Is Driven by the Combination of CD86/41BBL With CD40L and pp65 CMV Co-stimulation

**Figure 1:**
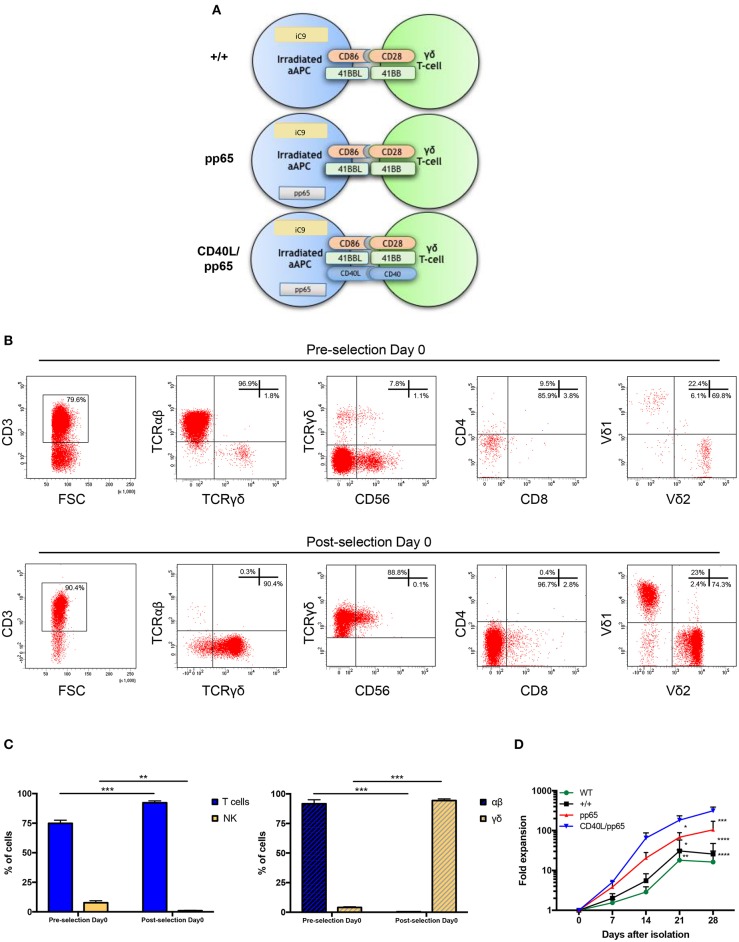
Generation and expansion of γδ-T cells from healthy donors by stimulation with aAPCs. The K562 cell line was engineered to express a combination of different costimulatory molecules and the iC9 safety switch and used as aAPC **(A)**. Human γδ-T cells were isolated from PBMCs using magnetic bead negative selection; the cellular composition was analysed by flow-cytometry both before and after isolation (pre- and post-selection day 0) **(B)**. The percentage of pre- and post-selection T cells and NK cells, as well as of αβ and γδ-T cells, is shown **(C)**. The **(D)** shows the fold expansion of isolated γδ-T cells co-cultured with each of the generated engineered aAPCs (+/+, pp65 and CD40L/pp65) and with WT aAPC. Data from 4 donors are expressed as average ± SEM. *p < 0.05; **p < 0.01; ***p < 0.001; ****p < 0.0001.

Both polyclonality and an activated phenotype of γδ-T cells, together with an efficient expansion, are essential to achieve a functional and effective T-cell product. For this reason, we engineered aAPCs to express different costimulatory molecules, including CD86, 41BBL, CD40L, and the CMV-pp65 antigen. In a translational perspective, we improved the safety profile by stably transducing aAPCs with the iC9 suicide gene. After generating stable and clonal aAPCs (Figure 1A and Supplementary Figure 1A), γδ-T cells were isolated from PBMCs of HD using the Miltenyi magnetic beads negative selection system. Purity was assessed after selection, showing 96.9% ± 1.4% γδ-T cells, 0.3% ± 0.2% TCR-αβ^+^, and 0.9% ± 0.3% Natural Killer (NK) cells (Figures 1B,C). The isolated cells were then stimulated with irradiated aAPC, either wild-type (WT), +/+ (iC9, CD86, and 41BBL), pp65 (+/+ and pp65), or CD40L/pp65 (+/+, pp65, and CD40L); aAPCs were used as feeder cells at a ratio of 1:2 (γδ-T:aAPCs) and cultured with sequential cytokine administrations. The most efficient expansion was achieved when γδ-T cells were stimulated with CD40L/pp65_aAPCs (313 ± 70-fold increase at day+28), compared to WT, +/+ and pp65 [16 ± 10 (*p* < 0.0001), 25 ± 15 (*p* < 0.0001) and 104 ± 50 (*p* < 0.001), respectively]. Moreover, only γδ-T cells stimulated with aAPCs including pp65 (CD40L/pp65 and pp65) showed a sustained proliferation rate until day+28, while those stimulated with +/+ and WT aAPCs reached a plateau at day+21, followed by a decrease until day+28 (Figure 1D).

### CD40L/pp65 and pp65 aAPCs Induce the Expansion of Polyclonal γδ-T Cells With Predominant Vδ1 Phenotype

**Figure 2:**
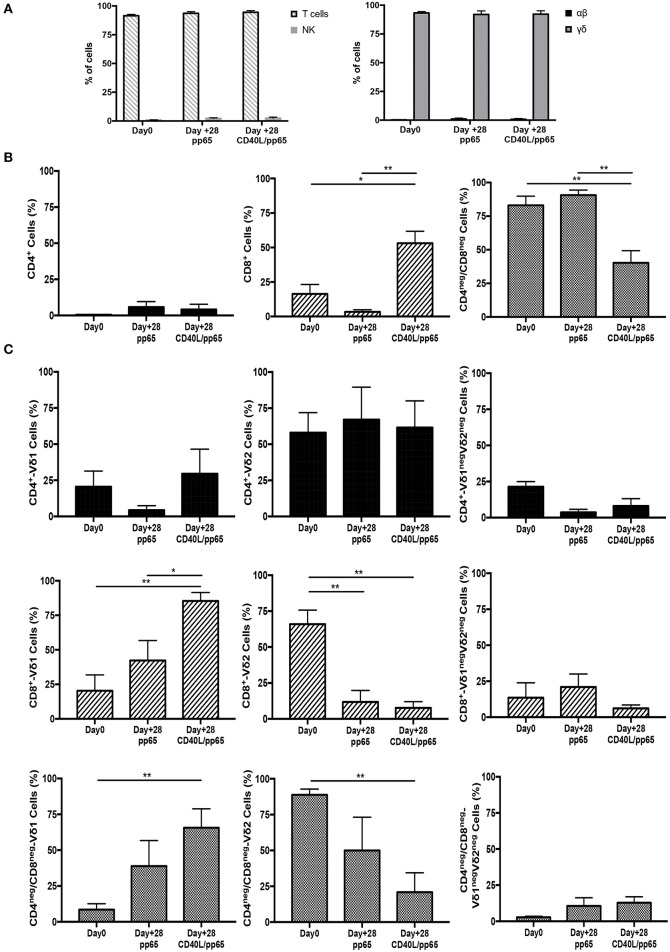
Phenotype of freshly isolated and expanded γδ-T cells stimulated with aAPCs/pp65 and CD40L/pp65. Evaluation of T cells, NK cells, as well as of αβ and γδ-T cell subset distribution, in freshly isolated and expanded γδ-T cells (*n* = 7) **(A)**. **(B)** displays the flow-cytometry analysis of the CD4^+^, CD8^+^, and CD4^neg^/CD8^neg^ cell content within the freshly isolated and expanded γδ-T cells (*n* = 4). Distributions of Vδ1, Vδ2, and Vδ1^neg^Vδ2^neg^ subpopulations within the CD4^+^, CD8^+^, and CD4^neg^/CD8^neg^ in freshly isolated and expanded γδ-T cells are displayed in panels (*n* = 4) **(C)**. Data summarised as average ± SEM of 4–7 donors. *p < 0.05; **p < 0.01.

γδ-T-cell products with the most efficient expansion (pp65 and CD40L/pp65) were phenotypically characterised at day+28. As shown in Figure 2A, both products maintain purity (CD3/TCR-γδ^+^) at the end of the expansion, with a maximum of 0.8% ± 0.6% and 1.9% ± 0.2% (*n* = 7) residual TCR-αβ T and NK cells, respectively; no statistical differences were observed between the two groups. At day+28, pp65-stimulated γδ-T cells maintained the same cell composition compared to that observed at the beginning of the culture (90.6% ± 3.7% of CD4^neg^/CD8^neg^ and 3.4% ± 1.4% of CD8^+^), whereas CD40L/pp65-stimulated cells significantly reduced the percentage of CD4^neg^/CD8^neg^, increasing the CD8^+^ subset (40.2% ± 9% and 53.1% ± 8.6%, respectively; *p* < 0.001) (Figure 2B). We then evaluated the polyclonality of our products, analysing the percentage of Vδ1, Vδ2, and Vδ1^neg^Vδ2^neg^ subsets within the CD4^+^, CD8^+^, and CD4^neg^/CD8^neg^ subpopulations (Figure 2C and Supplementary Figure 2): both CD8^+^ and CD4^neg^/CD8^neg^ γδ-T cell populations revealed a significant enrichment of the Vδ1 compartment, especially in presence of CD40L costimulation (*p* < 0.001). Only the CD4^+^ subset, although fairly underrepresented, maintained a polyclonal phenotype (Figure 2C).

### CD40L/pp65 Costimulation of γδ-T Cells Maintains the Memory Phenotype Without Inducing Overexpression of Exhaustion Markers

**Figure 3:**
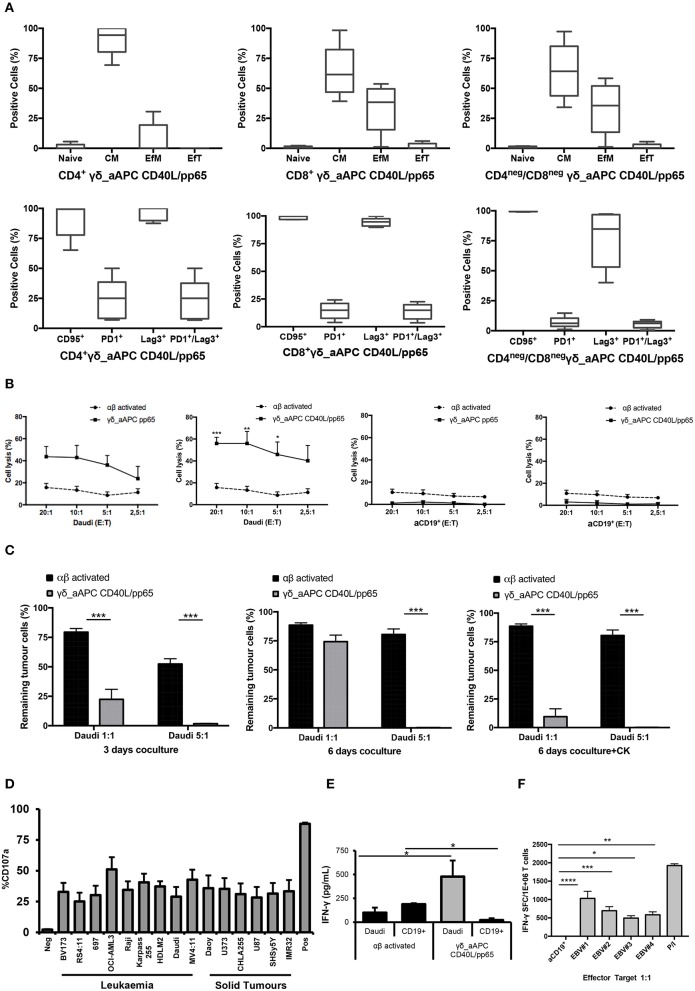
Phenotype and functional characterisation of expanded γδ-T cells. characterisation of the effector/memory, activation, and exhaustion **(A)** profile of CD40L/pp65 expanded γδ-T cells in the CD4^+^, CD8^+^, and CD4^neg^/CD8^neg^ subpopulations. **(B)** show the cytolytic activity by ^51^Cr release assay at different E:T ratios of γδ aAPC/pp65 or aAPC/CD40L/pp65 (black line) compared to polyclonally activated αβ-T cells (black dotted line) against Daudi tumour cells and aCD19^+^ B cells. **(C)** shows long-term cytotoxic activity of γδ CD40L/pp65 T cells against Daudi tumour cells in 3 or 6 days of co-culture at E:T ratios of 1:1 and 5:1 compared to polyclonally activated αβ-T cells. 6 day co-culture with Daudi tumour cells and γδ CD40L/pp65 T cells in presence of cytokines (IL2/IL15). Degranulation assay evaluating the CD107a expression of γδ CD40L/pp65 in co-culture with a panel of different tumour cell lines **(D)**. ELISA assay measuring the IFN-γ release in the supernatant collected from co-culture experiments **(E)**. **(F)** shows the number of spot forming units per 1 × 10^6^ γδ-T cells in IFN-γ Elispot when co-cultured with aCD19^+^ B cells or EBV infected B cells. Data are summarised as average ± SEM of 4 donors. *p < 0.05; **p < 0.01; ***p < 0.001; ****p < 0.0001.

Memory and exhaustion immunophenotypes of CD4^+^, CD8^+^, and CD4^neg^/CD8^neg^ γδ-T cells were analysed at day+28. In both pp65- and CD40L/pp65-expanded γδ-T cells, a predominant percentage of central memory (CM, CD45RO^+^/CD27^+^) and effector memory (EfM, CD45RO^+^/CD27^−^) cells was observed (Figure 3A). To further characterise these γδ-T-cell populations, the expression of the activation marker CD95 and the activating/inhibitory receptors, PD1 and Lag3, were analysed to define the exhaustion profile. All the γδ-T-cell subsets showed high levels of CD95 expression, underlining their activated status. The analysis of PD1 and Lag3 revealed, instead, that although Lag3 levels were extremely high and homogeneous in the CD4^+^ and CD8^+^ subsets, in CD4^neg^/CD8^neg^ the expression was more heterogeneous. On the other hand, the level of PD1 was homogeneously low in the CD8^+^ and CD4^neg^/CD8^neg^ subpopulations. Taken together, these results suggest the presence of an activated, but not exhausted phenotype in these subpopulations. In the CD4^+^ subset, however, the higher and heterogeneous expression of PD1 on the cells, which co-express Lag3, indicates an activated, but also more exhausted phenotype (Figure 3A).

### Expanded γδ-T Cells Show Anti-tumour Activity in Both Short- and Long-Term *in-vitro* Assays

**Figure 4:**
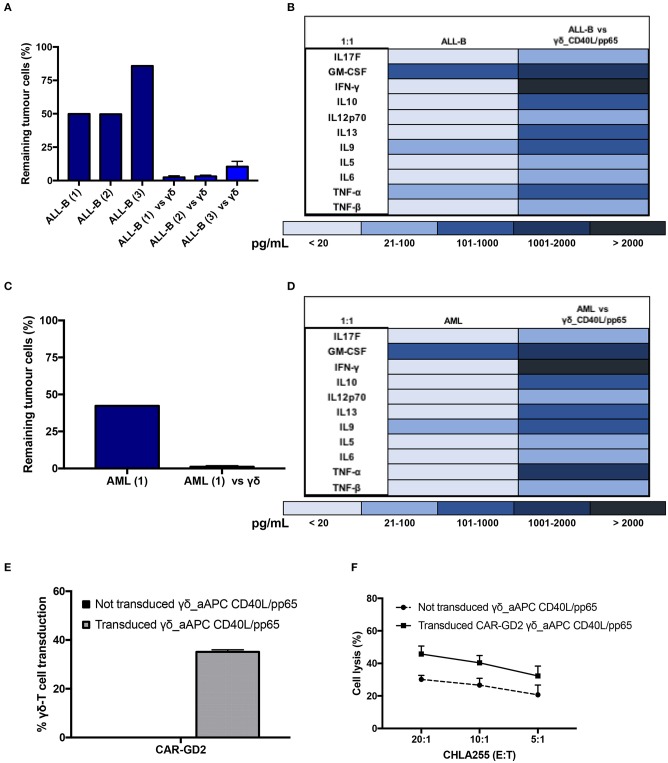
Functionality study of CD40L/pp65 expanded γδ-T against primary leukaemia blasts. **(A)** shows the long-term cytotoxic activity of 4 different γδ CD40L/pp65 T cell products against three primary acute leukaemia blasts. Panel of cytokines produced after 24 h of co-culture by multiplex analysis **(B)**. Data from 4 donors are expressed as average ± SEM. Long-term cytotoxic activity of 4 different γδ CD40L/pp65 T cell products against primary AML blasts **(C)**. Panel of cytokines produced after 24 h of co-culture with primary AML blasts, detected by multiplex analysis **(D)**. **(E)** demonstrates the transduction efficiency of CD40L/pp65 γδ-T cell products with a retrovirus vector encoding a third generation GD2-CAR. Short-term cytotoxic activity by ^51^Cr-release assay against the CHLA255 neuroblastoma cell line is reported in **(F)**. Data from 3 donors is expressed as average ± SEM.

Expanded γδ-T cells were analysed for their functional anti-tumour activity in both short and long-term cytotoxicity assays. In the short-term ^51^Cr release assay, only pre-activated γδ-T cells, stimulated with either pp65 or CD40L/pp65_APCs showed activity against the ALL-B CD19^+^-Daudi cells, but not against allogeneic B-cells (aCD19^+^). An effect of the same magnitude was not observed when we used freshly isolated γδ-T cells, γδ-T cells expanded with zoledronic acid or canonically polyclonally-activated and expanded αβ-T cells (Supplementary Figure 3 and Figure 3B). Moreover, only the cytotoxicity induced by γδ-T cells stimulated with CD40L/pp65 reached significant difference against Daudi. For this reason, we further explored the *in-vitro* anti-tumour activity of these expanded γδ-T cells by performing long-term co-culture assays. In detail, polyclonally-activated and expanded αβ-T cells or CD40L/pp65-stimulated γδ-T cells were co-cultured for 3 and 6 days with Daudi-GFP^+^ cells at an E:T ratio of 1:1 and 5:1. After 3 days of co-culture, expanded γδ-T cells showed a significantly higher cytotoxic activity at both E:T ratios, compared to expanded αβ-T cells (*p* < 0.001) (Figure 3C). However, on day+6, only at an E:T ratio of 5:1 γδ-T cells could significantly kill the tumour (*p* < 0.001). We hypothesised that this effect could be related to cytokine consumption, and therefore IL2/15 were added at day+3, leading to a re-acquisition of killing capacity on day+6 also at 1:1 E:T ratio (*p* < 0.001) (Figure 3C). Based on these results, we extended the evaluation of the anti-tumour activity in additional long-term co-culture experiments against a wider range of tumour targets, including the AML cell line MV4:11, the neuroblastoma SHSY5Y and the glioblastoma U87 tumour cell lines (Supplementary Figure 4A). To fully validate our findings, several other tumours, including B- and T-ALL, Non-Hodgkin Lymphoma, medulloblastoma, glioblastoma, and neuroblastoma cells, were tested in a degranulation assay (Figure 3D). The results underline the broad re-activity of the expanded γδ-T cells. Furthermore, we tested γδ-T cell activation after 24h of co-culture with Daudi or aCD19^+^ by analysing the IFNγ secretion in the supernatants, by ELISA, observing a specific production upon Daudi, but not aCD19^+^ cell exposure (*p* < 0.05) (Figure 3E and Supplementary Figure 4B). In addition, the virus specificity has been tested in IFNγ Elispot using B cells infected with EBV observing a significant high recognition by expanded γδ-T cells (Figure 3F). In order to address the contribution of the single γδ-T cell subpopulation to the anti-tumour and anti-viral activity, we performed cytotoxicity assays using the FACS sorted subpopulations. We observed only a statistical difference between the Vδ1 and Vδ1^neg^Vδ2^neg^ subpopulation in the response towards CMV. No other statistical differences were observed in the anti-virus or anti-tumour response although the Vδ2 subpopulation showed a slightly greater anti-tumour activity (Supplementary Figures 4C–E). The potency of expanded γδ-T cells was also tested against human primary patient-derived leukaemia blasts in co-culture assays (3 AML and 3 ALL). Expanded γδ-T cells almost completely eliminated primary blasts (Figures 4A,C), producing Th1/Th2 cytokines, but not Th17 (Figures 4B,D).

Lastly, to improve the anti-tumour capacity of the expanded cells and to provide the basis for a more potent immunotherapy product, we engineered γδ-T cells to express a CAR (Supplementary Figure 1C). As shown in Figure 4E, expanded γδ-T cells were efficiently transduced with a retroviral vector encoding a third-generation GD2.CAR (29). The CAR-transduced γδ-T cells showed a significant improvement of their anti-tumour activity compared to the untransduced γδ-T cells (Figure 4F), paving the way for the use of this potent, third-party, cell platform for more advanced gene-therapy approaches.

### Identification of a Peculiar Phospho-Proteomic Profile of αβ- and γδ-T Lymphocytes

**Figure 5:**
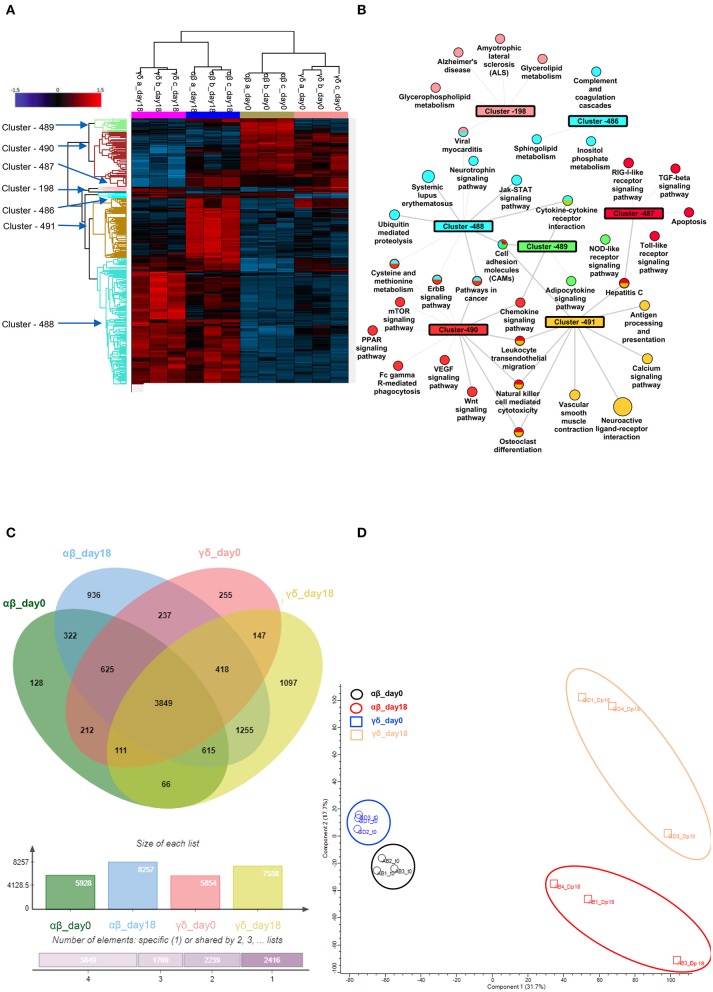
Phospho-proteomic profile of γδ CD40L/pp65 and polyclonally activated αβ-T cells. Anova graphical representation of the differential phosphoproteome pattern in the two populations on day 0 and day+18 after co-culture. **(A)** shows the heatmap Zscore Signalling Score values of proteins identified as relevant after Photon analysis, as well as the significant clusters on which is calculated a Fisher Test enrichment, using KEGG annotation, downloaded from Uniprot, with a significance threshold of Benjamini-Hochberg FDR < 0.02. The output file is converted into an alluvial plot where different colours are associated with different data sets and the width of flow line for each biological process is related to –log10 (Benjamini-Hochberg FDR < 0.02) of the enrichment. In **(B)**, the data table obtained from the Fisher Test applied on each protein cluster highlighted in the Heatmap is summarised in the network, where the main enriched Kegg annotations are reported. The node size is proportional to –log10 (Benjamini-Hochberg FDR), the edge width is related to Enrichment Factor and the colour identifies the belonging cluster. When a node is shared between different clusters all the reference colours are reported. Venn diagrams summarizing the phosphorylation sites detected in the two populations are reported in **(C)**. Numbers represent the distinct phosphorylation sites in the respective overlapping and non-overlapping areas; the histograms below show the total number of phosphorylation sites involved in the analysed groups. **(D)** shows the difference between the populations both before and after co-culture by PCA analysis.

In order to further define the different functional characteristics of αβ- and γδ-T cells, we performed a phospho-proteomic analysis in both freshly-isolated and polyclonally-expanded populations, using a new and accurate tool for interpreting the signalling data. Photon software is an interactive tool for the identification of functional proteins and reconstruction of signalling pathways through integrated analysis of phosphoproteomic data and protein-protein interaction networks, as String database (36). It was applied to our experimental dataset of 10,273 phosphorylation sites with high localisation probability (>0.75), integrated with a high-confidence PPI String network, removing high-degree nodes (degree < 700). We performed Photon analysis with default parameters and subsequently one-way ANOVA with a permutation-based FDR < 0.01, identifying 498 proteins. The analysis showed a clearly different phospho-protein distribution in αβ- and γδ-T cells already in the freshly-isolated populations, which becomes even more pronounced after activation for the significant increase of protein levels and activated pathways (Figures 5A,B and Supplementary Tables 1–3). As shown in Figures 5A,B, seven different clusters discriminating these populations were identified (cluster 198, 486–91). Although both freshly-isolated and polyclonally-expanded αβ- and γδ-T cells share more than 74% of their proteins and biological processes, each population shows a peculiar profile. The two freshly-isolated populations share 4,797 phosphorylation sites and, despite the moderate differences, the overall analysis reveals their strong proximity, implying important phenotypical and functional similarities (Figure 5B). However, when the expanded products are compared, the two populations markedly diverge, highlighting that different mechanisms/pathways are activated in response to activation (Figures 5C,D). The kinome analysis confirmed the difference between the two activated subgroups (Supplementary Figures 5A,B and Supplementary Tables 4, 5). In order to validate these data, dissecting the difference between αβ- and γδ-T cells, and to explore the conserved innate property of our expanded γδ-T cells, we performed a targeted gene expression profiling investigating signal transduction and inflammation pathways. The results confirmed what was observed in phosphoproteomic and kinome analysis showing that there is a similarity between freshly isolated and expanded γδ-T cells with the acquisition of pathways that indicate their activation (Supplementary Figures 6A,B).

### Expanded γδ-T Cells Show a Remarkable Anti-tumour *in-vivo* Activity in an Established Leukaemia Xenograft Mouse Model

**Figure 6:**
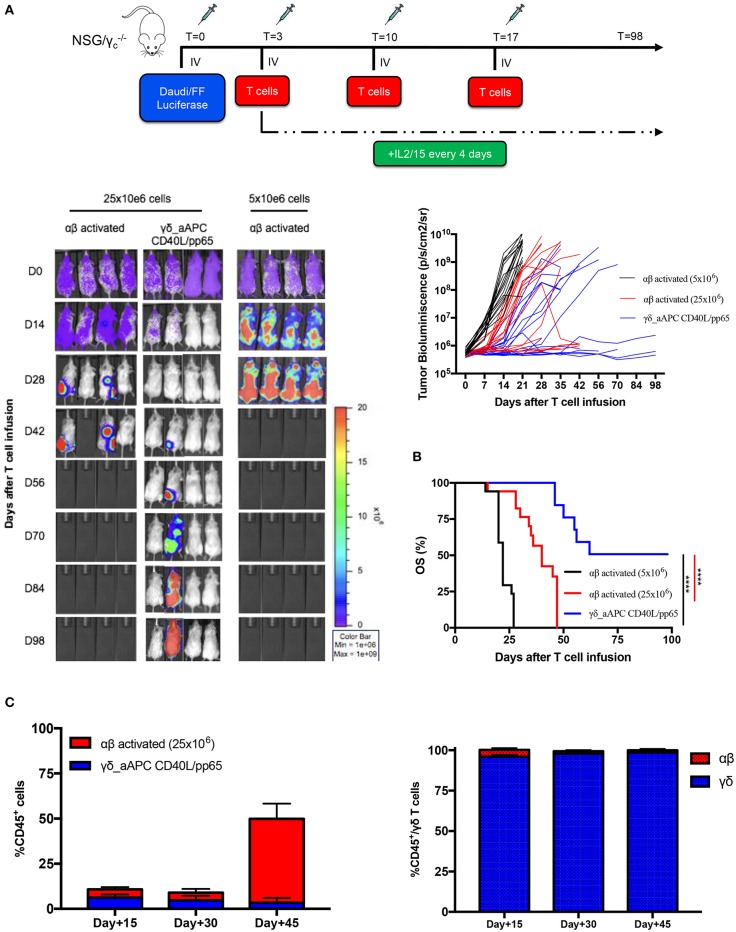
*In vivo* anti-tumour analysis of expanded γδ-T cells. **(A)** shows the experimental design and the *in vivo* bioluminescence imaging of NSG mice engrafted with Daudi leukaemia cell line and treated with either polyclonally activated αβ-T cells or γδ CD40L/pp65 T cells. Four representative mice per group are reported; bioluminescence of each single mouse treated with polyclonally activated αβ-T cells (5 × 10^6^ cells) (black line; 17 mice), polyclonally activated αβ-T cells (25 × 10^6^ cells) (red line; 17 mice) and γδ CD40L/pp65 T cells (blue line; 17 mice) **(A)**. Kaplan-Meier estimate of OS in tumour-bearing mice treated with either polyclonally activated αβ-T cells (5 × 10^6^ cells) (black line; 17 mice), polyclonally activated αβ-T cells (25 × 10^6^ cells) (red line; 17 mice), or γδ CD40L/pp65 T cells (blue line; 17 mice) **(B)**. Circulating CD45^+^, γδ-T, and αβ-T cells overtime persistence evaluation in treated mice **(C)**. Data summarised as average ± SEM. Log-rank (Mantel-Cox). ****p < 0.0001.

In order to assess the safety of the proposed expansion protocol, we first evaluated the presence of remaining aAPCs in the γδ-T cell preparations. As shown in Supplementary Figures 7A,B, in none of the products, aAPCs were detected by neither flow-cytometry nor more sensitive molecular (short tandem repeat) analysis. Then, we established a systemic xenograft leukaemia mouse model by infusing i.v. Daudi-FFLuc^+^ cells. Mice subsequently received i.v. either activated αβ-T cells or CD40L/pp65_γδ-T cells (γδ_CD40L/pp65), for 3 administrations, each infusion at the dose of 25 x 10^6^ T cells/mouse. As shown in Figures 6A,B, mice treated with CD40L/pp65 γδ-T cells had a significantly improved overall survival (OS) by day+100 compared to mice treated with αβ-T cells (*p* < 0.0001). The group treated with αβ-T cells showed a partial tumour control up to day+42, but also significant signs of xenograft GvHD. In contrast, mice treated with γδ_CD40L/pp65 displayed significant long-term tumour control without signs of toxicity. To evaluate whether the anti-tumour effect observed in αβ-T cell treated mice was based on alloreactivity, a cohort of mice was treated with a conventional dose of 5 × 10^6^ αβ-T cells/mouse. These mice experienced rapid tumour growth as demonstrated by the increasing bioluminescence signal over-time, suggesting that the tumour control observed at the higher dose, combined with the development of signs of GvHD in the mice, might represent an unspecific, alloreactive activation of the cells (Figure 6A). All together, these data translated into a significant improvement of OS of mice treated with CD40/pp65 γδ-T cells (*p* < 0.001, Figure 6B). In order to characterise the infused T-cells, we periodically analysed blood samples, documenting a strong expansion of T cells only in mice treated with αβ-T lymphocytes; the presence of γδ-T cells could be detected in mice infused with CD40/pp65 γδ-T cells until day+45 (Figure 6C). None of the mice treated with CD40/pp65 γδ-T cells showed expansion of aAPCs (data not shown).

### Validation of a GMP-Grade Protocol for the Expansion of γδ-T Cells

**Figure 7:**
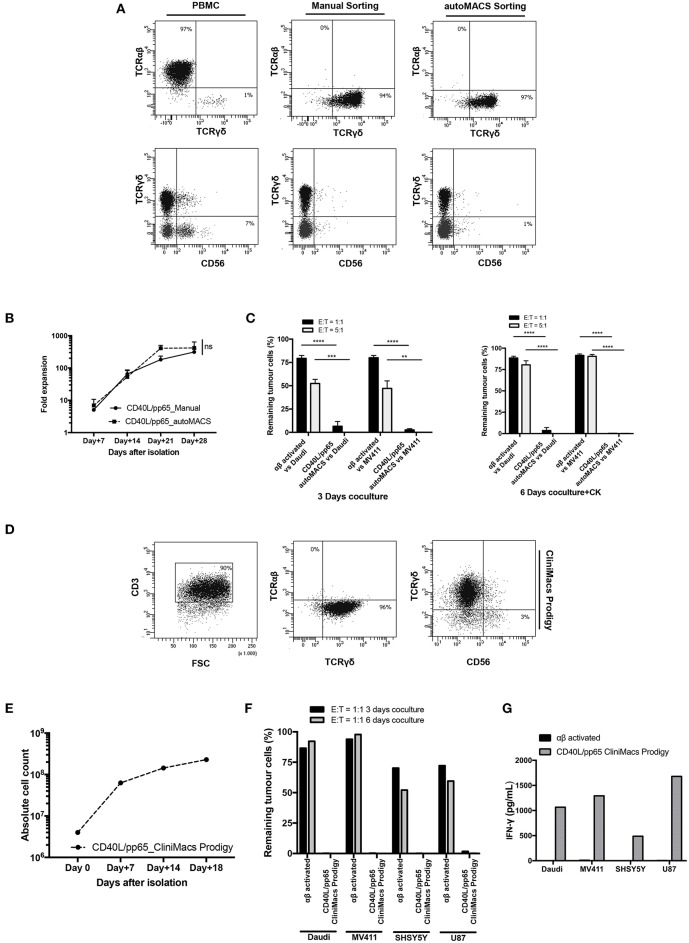
Translational GMP grade γδ-T cell production. Identification of a GMP grade protocol for γδ-T cell isolation and expansion for the manufacturing of polyclonal γδ-T cell products to use in third-party setting. In **(A)**, the purity of T-cell products after standard (manual) isolation and automatised/automated sorting (autoMACS pro separator) of γδ-T cells is shown. **(B)** shows the expansion capacity of these cells using the optimised protocol identified in this study. The potency of these cells was verified by long-term co-culture at day+3 and day+6 **(C)** at 1:1 and 5:1 E:T ratios; data are summarised as average ± SEM of three donors. **p < 0.01; ***p < 0.001; ****p < 0.0001. **(D–G)** display the characteristics of polyclonal γδ-T cell products obtained using Clinimacs Prodigy; purity **(D)**, expansion **(E)**, functionality by long-term co-culture at day+3 and day+6 and IFN-γ release **(F,G)**. Data summarises the average of two donors.

Based on the encouraging results obtained, in a translational perspective, we established a clinical-grade protocol using the closed system Clinimacs Prodigy, to generate a third-party γδ-T cell bank. Therefore, we optimised the selection protocol using the autoMACS Pro-separator, which faithfully reproduces the separation procedure used by the Clinimacs Prodigy. γδ-T cells isolated through this approach exhibited a similar phenotype compared to those manually isolated (Figure 7A). γδ-T cells (purity > 92.3% ± 4.1%) were then co-cultured with CD40L/pp65_aAPCs in bioreactors as described above. We did not observe any difference in the expansion rate between automatically and manually isolated γδ-T cells (Figure 7B). We then verified the functionality through 3 and 6 day co-cultures with either Daudi or MV4:11. Figure 7C shows a significant difference in the γδ-T-cell killing activity compared with activated, matched control αβ-T cells. Then, we transferred the optimised protocol on the Clinimacs Prodigy, extending the expansion up to 18 days. Purity, expansion, ability to kill tumour cells and IFNγ-release were evaluated at the end of the culture. As represented in Figures 7D–G, the automation with Clinimacs Prodigy resulted in an effective expansion of a pure population of polyclonal γδ-T cells as demonstrated by FACS analysis with an expansion-rate comparable to those generated with the manual protocol. Furthermore, we confirmed the functional capacity by co-culture and cytokine release assays.

A stability study was then carried out on frozen expanded γδ-T cell products. After 30 ± 6 months of cryostorage, γδ-T cells were thawed and co-cultured with Daudi and SHSY5Y, without cytokine addition. Thawed cells preserved their capacity to kill tumour cells also at an E:T ratio of 1:1 and to release a significant level of IFNγ (Supplementary Figures 8A–C).

## Discussion

An emerging and promising option for treatment of patients affected by chemo-resistant and refractory haematological/oncological diseases is represented by immunotherapeutic approaches (37). Impressive results have been observed in patients with CD19^+^ ALL and B-cell non-Hodgkin lymphoma treated with CD19.CAR-T cells (28, 38). However, similar results still need to be shown in the context of solid tumours (39, 40). Due to antigen-independent mechanisms, αβCAR-T cells produce an extensive array of side effects and treatment-related morbidity, such as cytokine-release syndrome and neurotoxicity (41). Furthermore, manufacturing of αβCAR-T cells is time-consuming, representing sometimes a limiting factor, especially for patients with rapidly evolving disease (42). Finally, many patients are heavily pre-treated and, thus, their lymphocyte count in peripheral blood is low, making the production of autologous CAR-T cells difficult to achieve. Considering all these hurdles, we speculated that γδ-T cells could represent a potentially novel and effective immunotherapeutic approach. These cells are naturally primed for innate cytotoxicity (43), displaying both anti-viral and anti-tumour activity and physiologically infiltrate the microenvironment of solid tumours (44). Therefore, focusing also on their function as professional APCs, several studies underline the role of γδ-T cells in boosting the response of the immune system (45).

In this study, we report a clinical-grade protocol to isolate and efficiently expand, both manually and automatically, high numbers of polyclonal γδ-T cells, providing also evidence of the possibility of further enhancing the anti-tumour efficacy through gene-modification. The cellular products generated were highly stable over-time, also after thawing, maintaining a memory phenotype and an excellent and broad anti-tumour response in the absence of alloreactivity. Thanks to their polyclonal composition, an extensive activity against several malignancies and virus was observed. In fact, the Vδ1 subset, although never directly infused in a patient, has previously been correlated with complete responses in patients with leukaemia after mismatched allogeneic HSCT, activity against glioblastoma and found to be expanded in patients experiencing CMV reactivation (7, 46). This population has also been associated with clinical benefits due to its more *naïve* memory phenotype (12), the reduced susceptibility to activation-induced cell death (47) and its natural residency in tissues. Vδ2, which instead have been directly infused in patients, elicited responses against several malignancies (48). Little is known about the Vδ1^neg^Vδ2^neg^ population, and it has been suggested that this subset has a role in both anti-viral and anti-tumour immunity (23, 49). We believe that the presence of all these populations in our final product is relevant to determine the anti-tumour activity observed.

The introduction of the costimulatory molecules CD86, 4-1BBL, CD40L, and pp65-CMV antigen on aAPCs allowed a great expansion of γδ-T cells. Moreover, while maintaining a polyclonal phenotype, a major representation of the memory Vδ1 subgroup was observed in our system. The polyclonal cellular product obtained contains, therefore, a long-lived population and is able to provide an anti-tumour activity against a broad range of malignancies. Importantly, all these characteristics are maintained after gene-modification with a GD2.CAR. Furthermore, considering the presence of an uncommonly high quantity of CD8^+^ cells in our product, we envisage the possibility of a further gene-modification with exogenous, transgenic TCRs (50, 51). Based on the gene-expression profile, these expanded γδ-T cells could be used as platform for the expression of the CD5.CAR for the treatment of refractory T-ALL, with the theoretical advantage of avoiding a fratricide effect as opposed to transduced αβ-T cells (52).

Considering the large evidence already published on the anti-viral activity of γδ-T cells, we confirmed that this activity is conserved also in our expanded γδ-T cell product (53).

In view of potential safety concerns associated with the use of irradiated tumour cells as feeders, we further engineered the aAPCs with the iC9 suicide gene as safety switch, whose efficacy has already been validated in clinical trials (54). Moreover, we proved that thanks to the irradiation, no traces of aAPCs neither at cellular nor at molecular level were observed, both *in-vitro* and *in-vivo*, confirming observations previously reported by our group (26). The possibility of using tumour cell lines as feeder cells is also supported by several on-going clinical studies (NCT00694330, NCT00361296, NCT00363649) (55).

Beside the possibility of safely obtaining large numbers of expanded γδ-T cells with potent and broad *in-vitro* and *in-vivo* anti-tumour activity, we could prove some relevant features of these cells that make their clinical translation extremely appealing. Importantly, despite the relevant tumour control observed, neither allogeneic nor xenogeneic effects of the expanded γδ-T cells, as well as αβ-T cell expansion, were detected *in-vitro* or *in-vivo*, confirming their suitability for developing third-party cell banks. Some concerns regarding the possible plasticity of a particular subset of Vδ1^+^/CD4^+^ γδ-T cells, which are capable to convert into αβ-T cells increasing the risk of GvHD, needs to be taken into consideration (56). However, the percentage of total Vδ1^+^/CD4^+^ in our products is very low and we did not observe any expansion of αβ-T cells neither *in vitro* nor *in vivo*. Furthermore, γδ-T cells are being kept in culture for more than 21 days before being frozen and stored until administration which makes the persistence or expansion of this precursor T-cell population very unlikely; moreover we confirmed by immune-phenotype analysis in our expanded γδ-T cell products the absence of these precursors (data not shown). Then, for the clinical translation, release criteria need to defined, indicating a threshold level of purity of the product. In case the number of αβ T cells exceeds this threshold, an αβ-depletion procedure can be carried out before freezing the product. Moreover, no production of Th17 cytokines was observed, excluding the potential concern of promoting proliferation and dissemination of tumour cells, induce myeloid-derived suppressor cells and macrophages in the tumour microenvironment, and impairing the tumour immunosurveillance (57, 58). Furthermore, based on the gene-expression data and phosphoproteomic analysis, we can speculate that these expanded γδ-T cells could work as APCs and have different migration capacity compared to αβ-T cells.

Lastly, the phospho-proteomic and gene-expression characterisation of the γδ-T cell products revealed a peculiar behaviour of γδ-T cells, as compared to αβ-T cells (metabolism, pathways activated upon stimulation, phenotype, migration). Further studies are needed, however, to obtain deeper insight into the differences observed between the two populations and to better define the relevance of these findings.

In conclusion, we provide evidence supporting a robust and solid protocol for expanding, both manually and automatically, high and clinically-relevant numbers of polyclonal γδ-T cells under GMP-grade conditions, with the possibility of further gene-engineering to improve their potency. This approach supports the possibility of generating a third-party γδ-T cell bank from unrelated HD, which can be administered as an off-the-shelf product for treatment of several malignancies. Moreover, these cell products could also be administered after HSCT in patients at high-risk of relapse, in place of conventional donor-derived lymphocyte infusions, maintaining the graft-vs.-tumour effect, and possibly the protection against viral infections, while avoiding the risk of severe GvHD. Further potential clinical implications include the use of expanded/activated γδ-T cells as bridging treatment for patients with refractory/relapsed malignant disease who undergo personalised immunotherapies, like autologous CAR αβ-T cell infusions, offering tumour control during the manufacturing time, until the gene-modified product becomes available. Lastly, but nonetheless importantly, these cells could serve as an innovative platform for the establishment of off-the-shelf CAR-T cell product to be used in an allogeneic setting.

## Data Availability Statement

The datasets generated for this study can be found in the ProteomeXchange Consortium via the PRIDE partner repository with the dataset identifier PXD015506 (http://proteomecentral.proteomexchange.org/cgi/GetDataset?ID=PXD015506).

## Ethics Statement

The studies involving human participants were reviewed and approved by Ethical committee—Bambino Gesù Children's Hospital: protocol 969/2015. Written informed consent to participate in this study was provided by the participants' legal guardian/next of kin. The animal study was reviewed and approved by Ministero della Salute-88/2016_PR.

## Author Contributions

IC and FL supervised the project conduction. VP, RC, FL, and IC designed experimental studies, analysed the data, and wrote the manuscript. IC, GW, and VP designed and cloned the vectors. VP, RC, FD, and GW performed the *in vitro* experiments and the development of the translational protocol. VP, RC, and CA performed the *in vivo* experiments. VP, LA, and TB performed gene expression analyses. FD and APi collected primary patient blasts. APe conducted LC-MS/MS experiments and analysed data. VP, RC, GW, and EG conducted the immunofluorescence experiments. NT conducted FACS cell sorting. FD, BD, and CQ contributed to the analysis of experimental data and edited the paper. All the authors reviewed and approved the final version of the manuscript.

### Conflict of Interest

The authors declare that the research was conducted in the absence of any commercial or financial relationships that could be construed as a potential conflict of interest.

## Abbreviations

ALL: Acute Lymphoblastic Leukaemia
AML: Acute Myeloid Leukemia
aCD19^+^: allogeneic B-cells
aAPCs: artificial Antigen-Presenting Cells
OPBG: Bambino Gesù
Children's Hospital
BC: Buffy Coats
CM: Central Memory
CAR: Chimeric Antigen Receptors
CMV: Cytomegalovirus
EfM: Effector Memory
eGFP-FFLuc: eGFP-Firefly-Luciferase
eGFP: Enhanced Green Fluorescent Protein
FBS: Fetal Bovine Serum
GMP: Good Manufacturing Practice
GvHD: Graft-vs.-Host Disease
HSCT: Haematopoietic Stem Cell Transplantation
HD: Healthy Donors
HLA: Human Leucocyte Antigen
iC9: inducible Caspase-9
i.v.: intravenous injection
mAbs: monoclonal Antibodies
NK: Natural Killer
PBMC: PB Mononuclear Cells
PB: Peripheral Blood
SEM: Standard Error Mean
TCR: T-Cell Receptor
WT: Wild-Type.
