# Supplementary figures and images for: Universal Ready-to-Use Immunotherapeutic Approach for the Treatment of Cancer: Expanded and Activated Polyclonal γδ Memory T Cells
Source: Front Immunol. 2019 Nov 22;10:2717. doi: 10.3389/fimmu.2019.02717 (PMC6883509; doi:10.3389/fimmu.2019.02717)

A

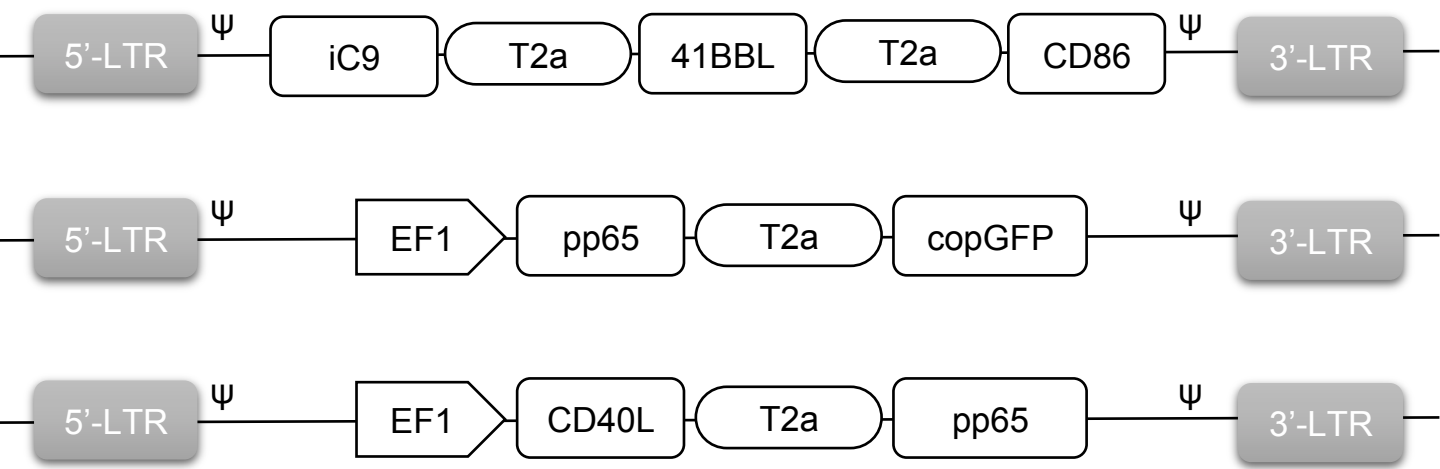

B

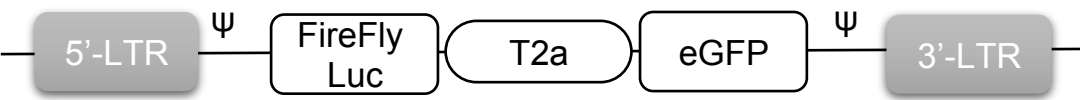

C

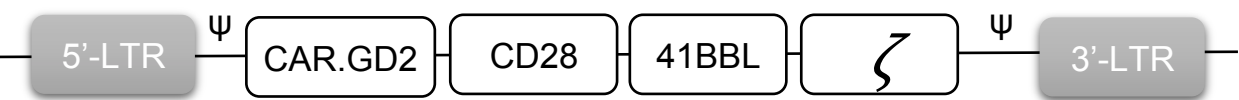

Supplement: Supplementary file 1 [file Image_1.pdf]

A

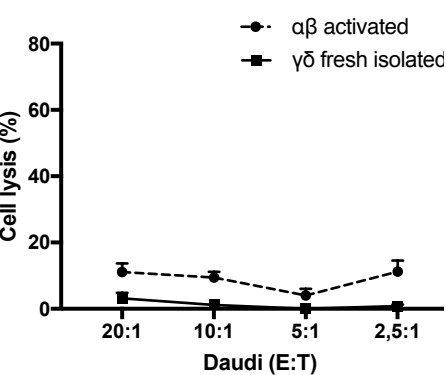

B

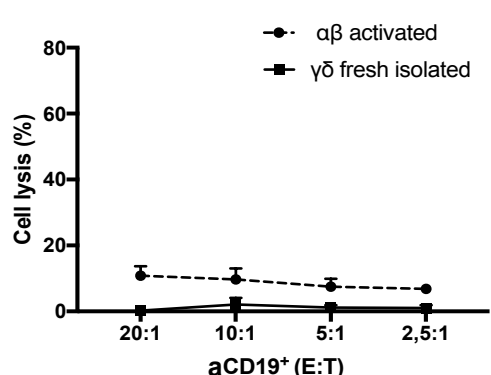

C

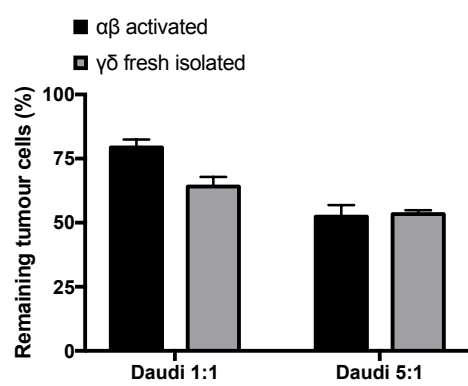

D

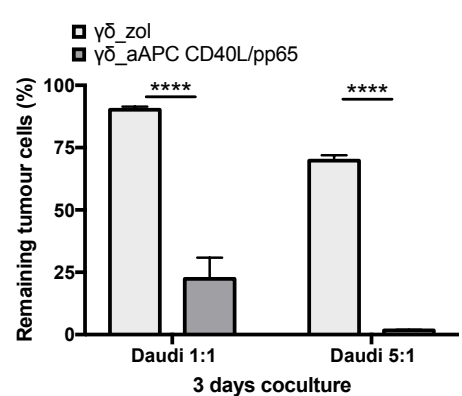

E

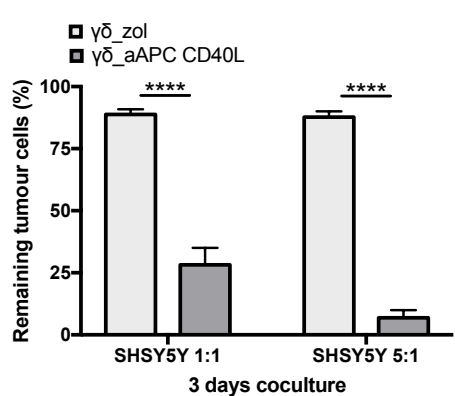

Supplement: Supplementary file 3 [file Image_3.pdf]

**A**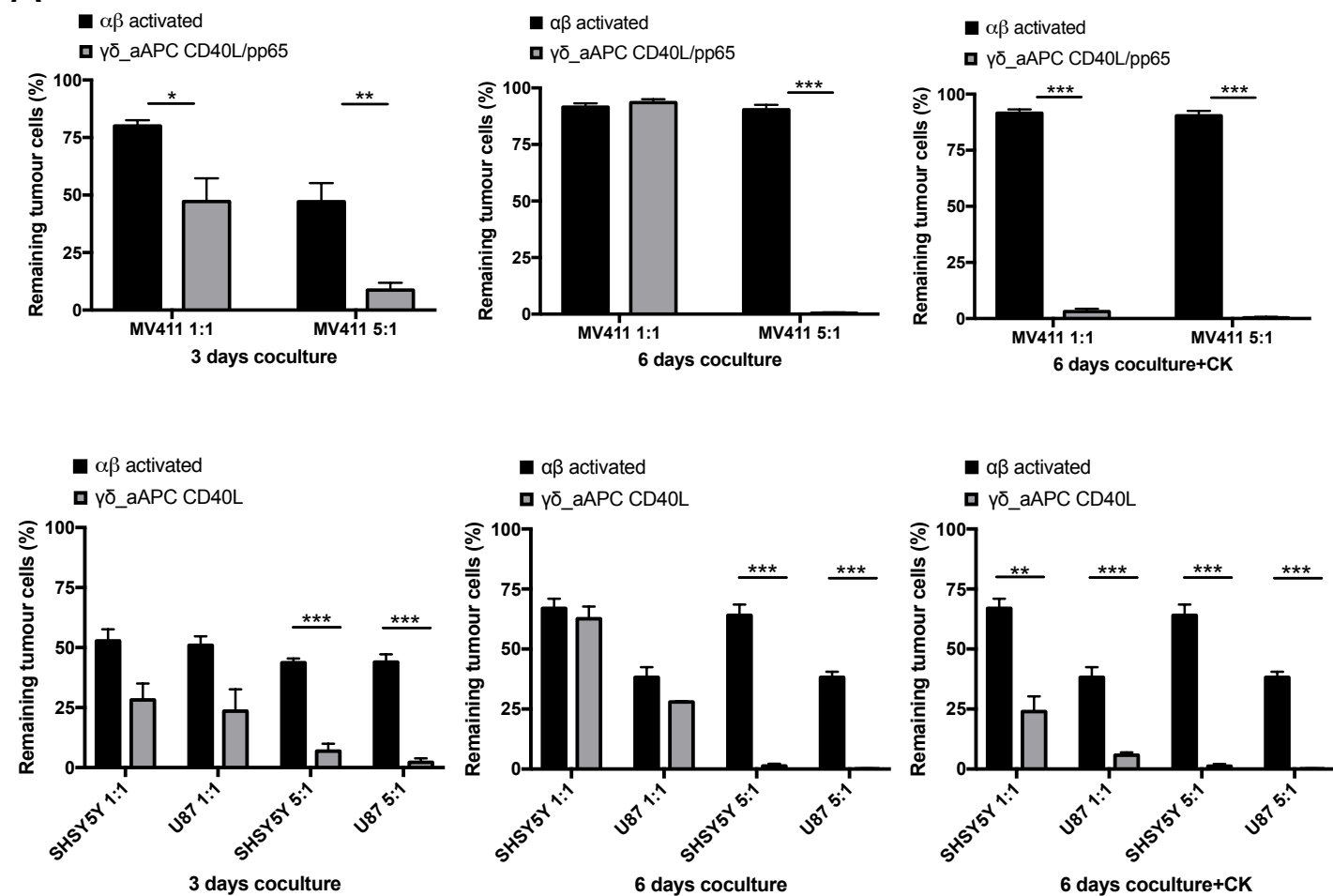**B**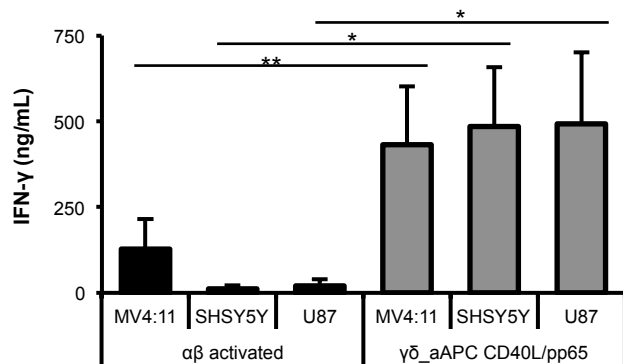**C**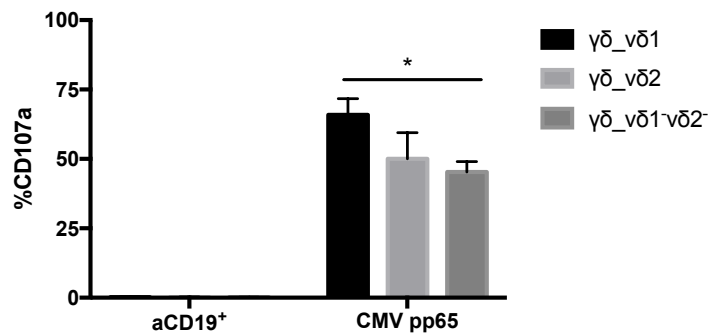**D**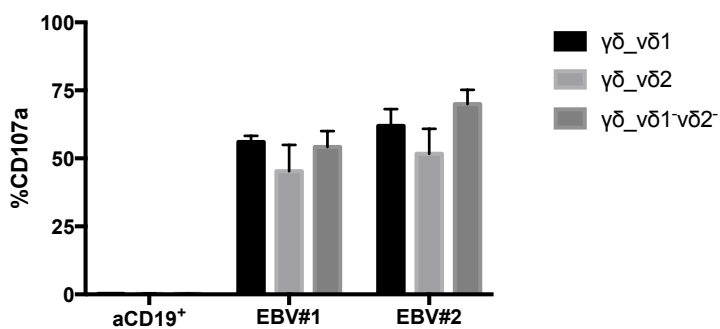**E**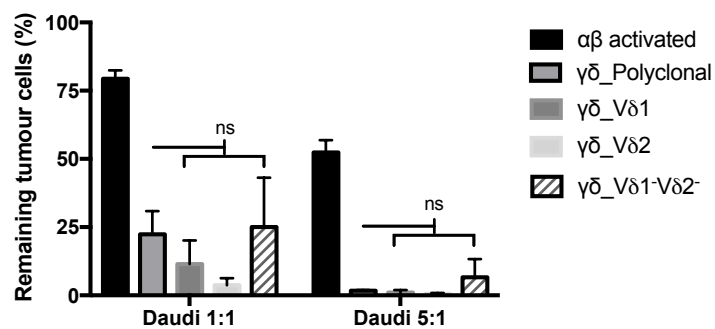

Supplement: Supplementary file 4 [file Image_4.pdf]

A

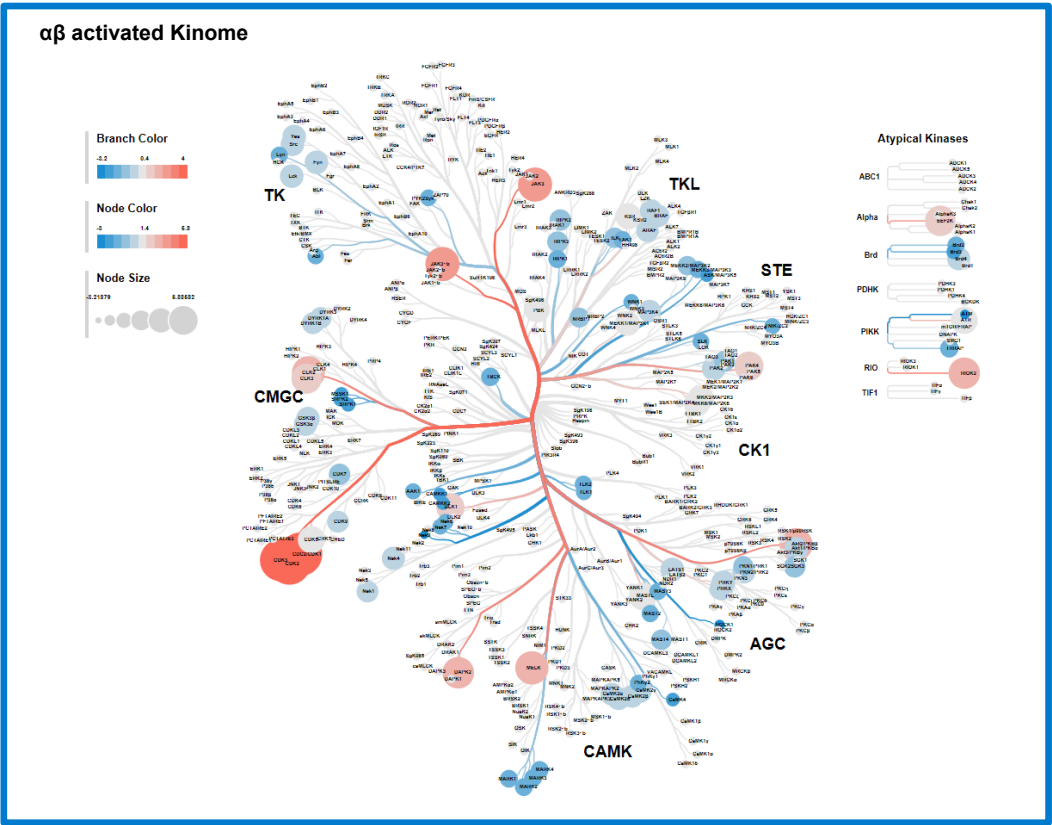

B

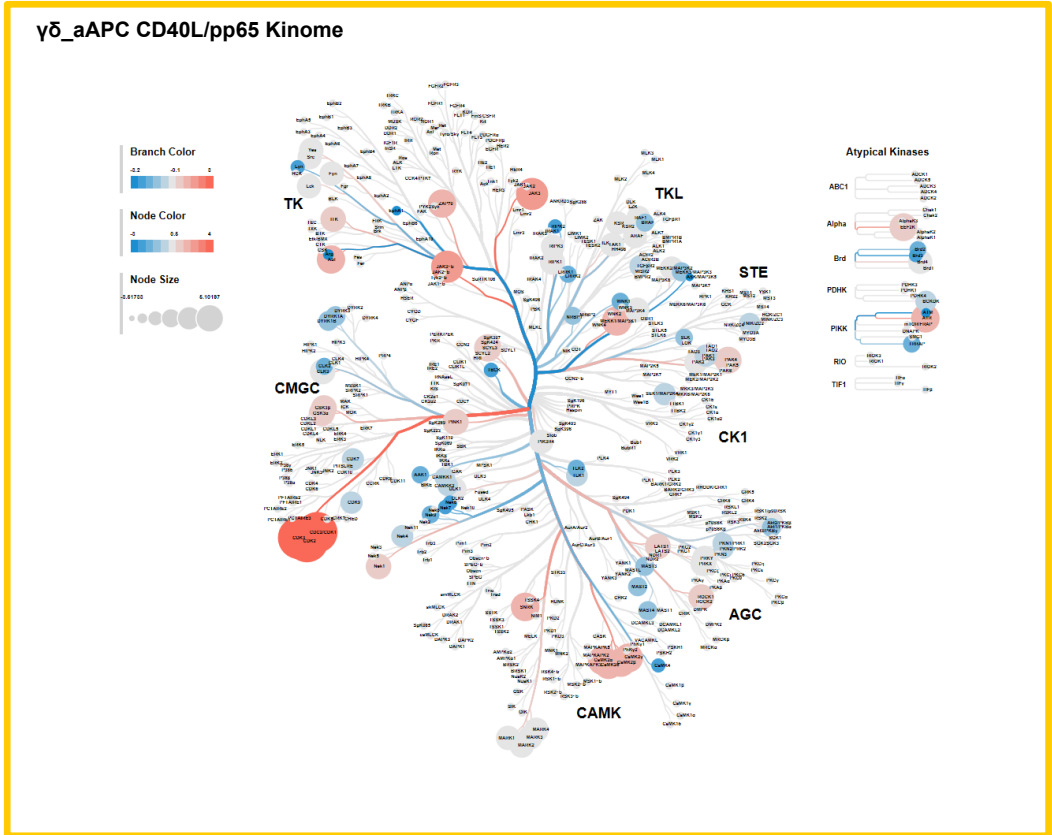

Supplement: Supplementary file 5 [file Image_5.pdf]

A

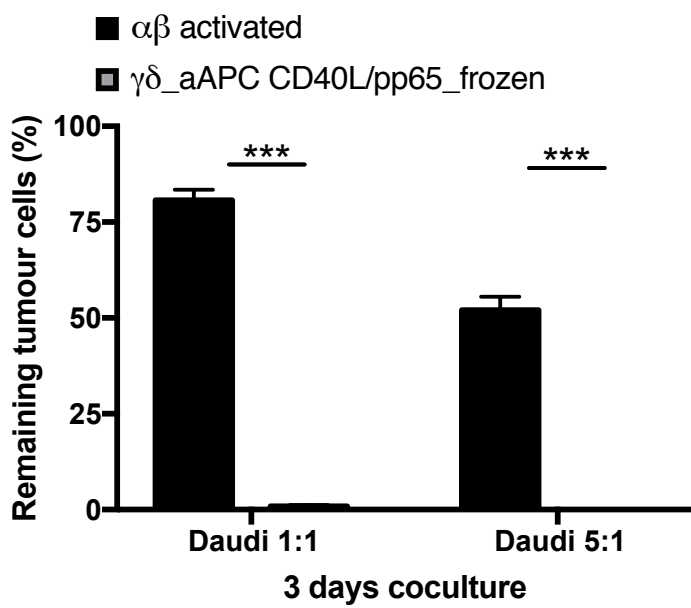

B

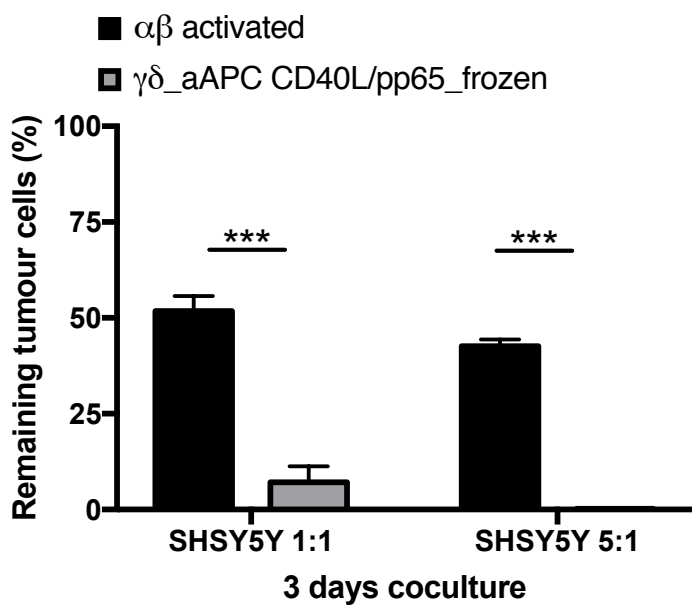

C

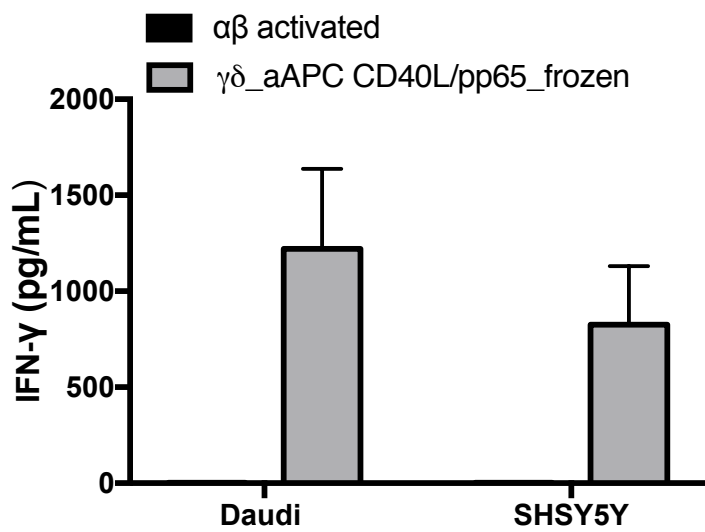

Supplement: Supplementary file 8 [file Image_8.pdf]
