# Supplementary material for: Universal Ready-to-Use Immunotherapeutic Approach for the Treatment of Cancer: Expanded and Activated Polyclonal γδ Memory T Cells
Source: Front Immunol. 2019 Nov 22;10:2717. doi: 10.3389/fimmu.2019.02717 (PMC6883509; doi:10.3389/fimmu.2019.02717)
Supplement: Supplementary file 2 [file Image_2.pdf]

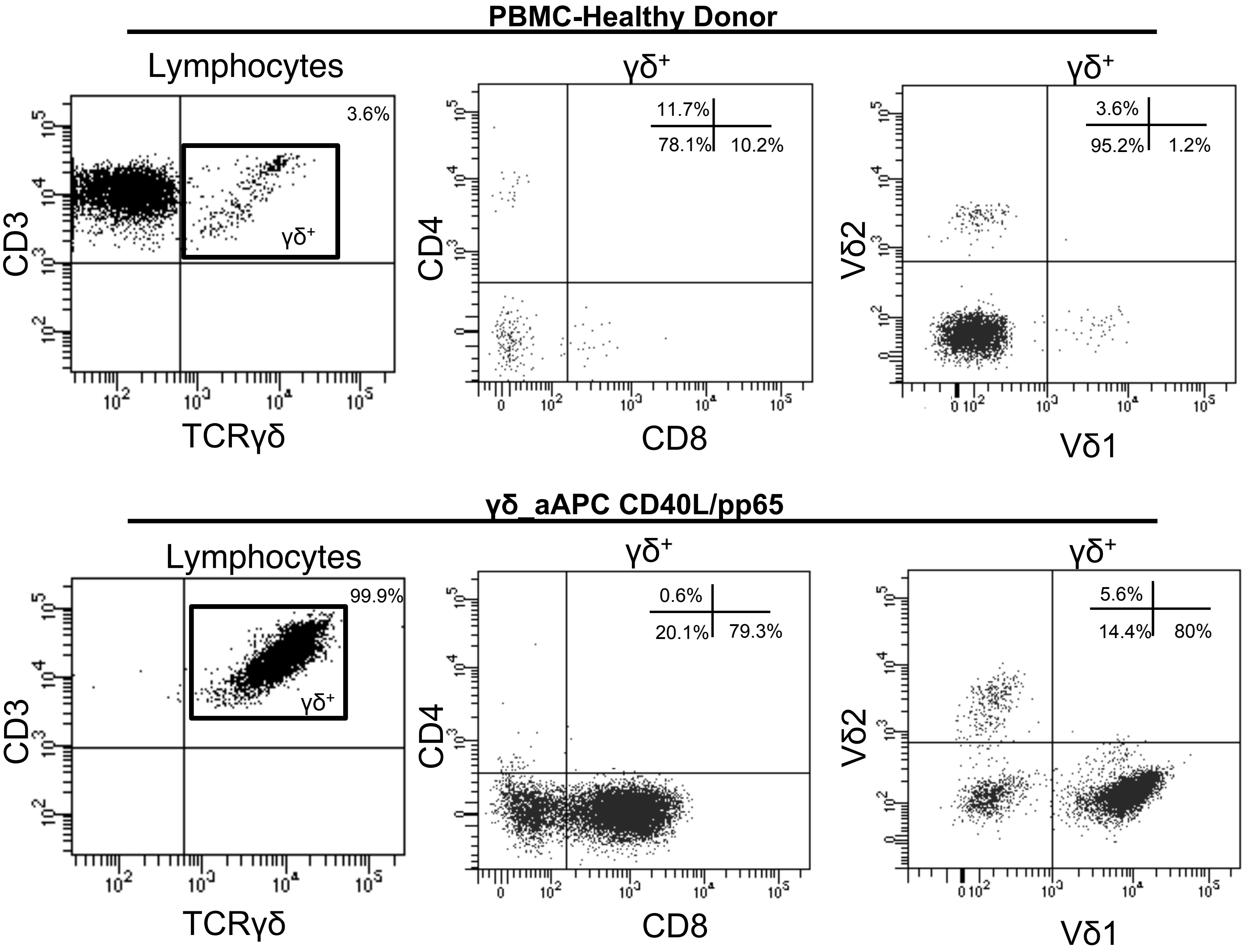

PBMC-Healthy Donor
Lymphocytes
3.6%
γδ⁺
CD3
TCRγδ
γδ⁺
11.7%
78.1%
10.2%
CD4
CD8
γδ⁺
3.6%
95.2%
1.2%
Vδ2
Vδ1
γδ_aAPC CD40L/pp65
Lymphocytes
99.9%
γδ⁺
CD3
TCRγδ
γδ⁺
0.6%
20.1%
79.3%
CD4
CD8
γδ⁺
5.6%
14.4%
80%
Vδ2
Vδ1
