# Supplementary material for: Universal Ready-to-Use Immunotherapeutic Approach for the Treatment of Cancer: Expanded and Activated Polyclonal γδ Memory T Cells
Source: Front Immunol. 2019 Nov 22;10:2717. doi: 10.3389/fimmu.2019.02717 (PMC6883509; doi:10.3389/fimmu.2019.02717)
Supplement: Supplementary file 6 [file Image_6.pdf]

A

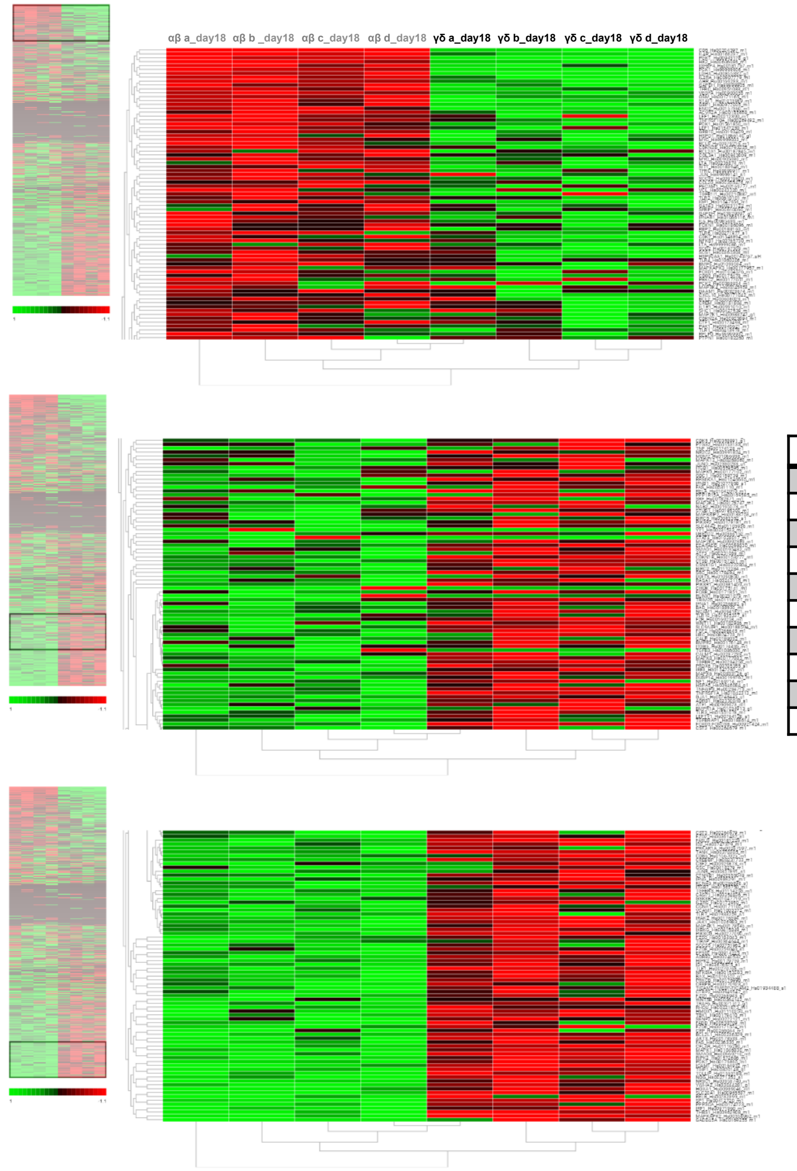


| Modulated Pathways |
| --- |
| Immune System |
| Metabolism of Proteins |
| Programmed Cell Death |
| Gene Expression (Transcription) |
| Extracellular Matrix Organisation |
| Signal Transduction |
| Disease |
| Cellular Responses to External Stimuli |
| Hemostasis |
| Metabolism |

B

| Down-Regulated | | | | |
| --- | --- | --- | --- | --- |
| ALOX5 | FLT3LG | IFNGR2 | LIF | RPLP0 |
| CCL25 | GPI | IGFBP4 | LTA | STAT1 |
| CCR4 | GRB10 | IL1A | LTA4H | TNFSF13B |
| CCR7 | GRN | IL2RA | LY86 | TRAP1 |
| CD4 | HDAC4 | IL4R | NFRKB | VEGFA |
| CD5 | HK2 | IL6R | NLRP3 | VEGFB |
| CFP | HSPD1 | IL7R | OSM | |
| CXCR4 | ICOSLG | LDHA | PGK1 | |

| Up-Regulated | | | | |
| --- | --- | --- | --- | --- |
| ABCB1 | CMKLR1 | ICAM1 | IL18RA | TGIF1 |
| CCR9 | CX3CR1 | IL12A | IL2RB | THBS1 |
| CD86 | HMOX1 | IL12RB2 | RIPK2 | XCR1 |
| CFLAR | | | | |
