# Supplementary material for: Universal Ready-to-Use Immunotherapeutic Approach for the Treatment of Cancer: Expanded and Activated Polyclonal γδ Memory T Cells
Source: Front Immunol. 2019 Nov 22;10:2717. doi: 10.3389/fimmu.2019.02717 (PMC6883509; doi:10.3389/fimmu.2019.02717)

A

DNA-System	K562 wt	γδ1-T_T0	γδ1-T_T28	γδ2-T_T0	γδ2-T_T28	γδ3-T_T0	γδ3-T_T28	γδ4-T_T0	γδ4-T_T28
AM	X, X	X, X	X, X	X, Y	X, Y	X, Y	X, Y	X, Y	X, Y
D6S1043	11, 15	11, 11	11, 11	12, 13	12, 13	12, 12	12, 12	13, 13	13, 13
Penta E	5, 14	10, 11	10, 11	7, 11	7, 11	11, 17	11, 17	7, 13	7, 13
D16S539	11, 12	12, 13	12, 13	9, 9	9, 9	9, 12	9, 12	11, 13	11, 13
D18S51	15, 16	13, 17	13, 17	12, 20	12, 20	17, 18	17, 18	15, 18	15, 18
Penta D	9, 13	11, 13	11, 13	7, 14	7, 14	10, 13	10, 13	13, 14	13, 14
D21S11	29, 31	30, 31.2	30, 31.2	26, 32.2	26, 32.2	29, 30	29, 30	31.2, 32.2	31.2, 32.2
D7S820	9, 11	10, 11	10, 11	9, 12	9, 12	9, 10	9, 10	9, 10	9, 10
D19S433	14, 14.2	13, 14.2	13, 14.2	13, 14	13, 14	14, 16	14, 16	13, 14	13, 14

B

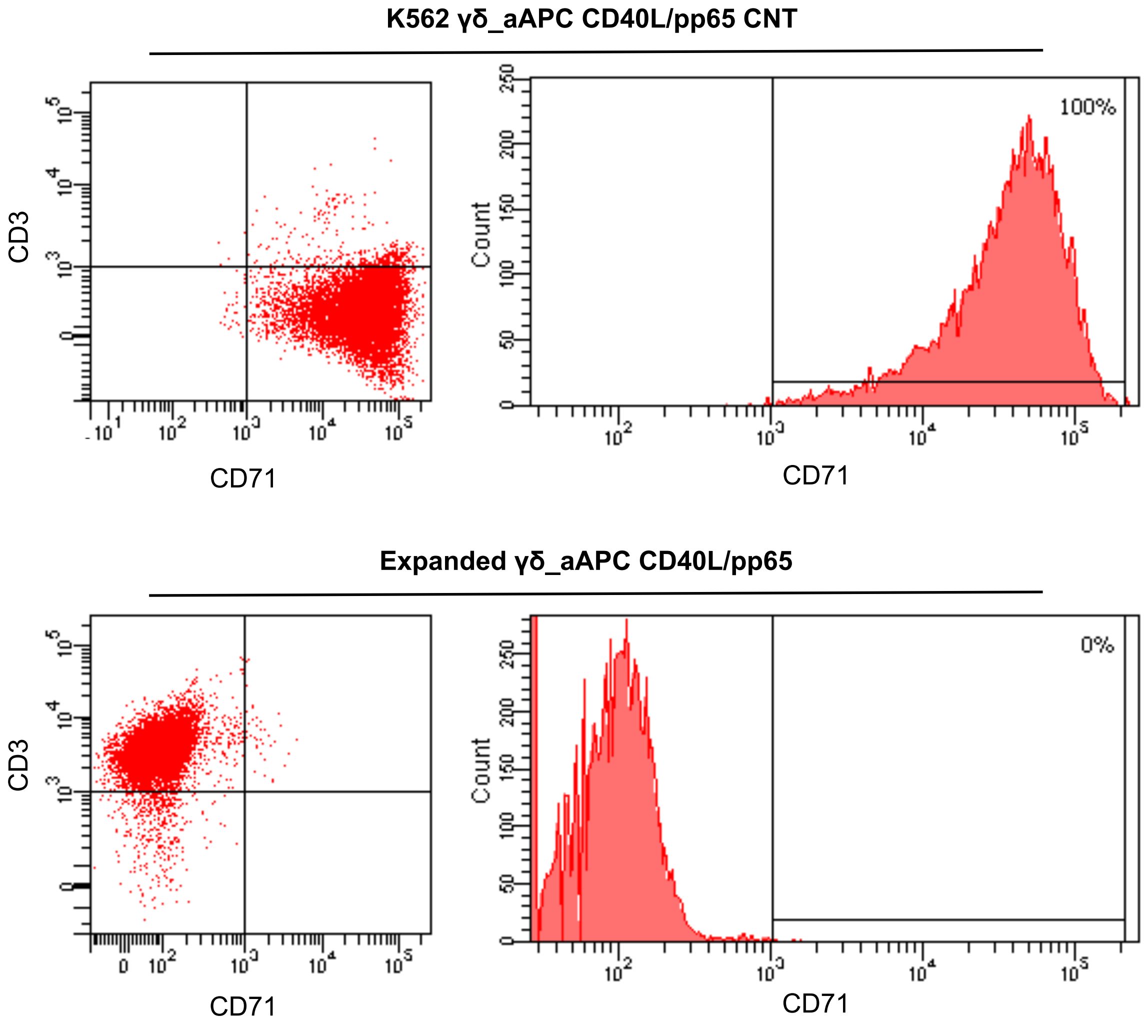

Supplement: Supplementary file 7 [file Image_7.pdf]
