# Supplementary material for: Universal Ready-to-Use Immunotherapeutic Approach for the Treatment of Cancer: Expanded and Activated Polyclonal γδ Memory T Cells
Source: Front Immunol. 2019 Nov 22;10:2717. doi: 10.3389/fimmu.2019.02717 (PMC6883509; doi:10.3389/fimmu.2019.02717)
Supplement: Supplementary file 10 [file Table_2.PDF]

Polito et al. Universal ready-to-use immunotherapeutic approach for the treatment of cancer: expanded and activated polyclonal γδ memory T cells.
Anova Photon

AB1_t0 Signaling score	AB2_t0 Signaling score	AB3_t0 Signaling score	AB1_Dp18 Signaling score	AB3_Dp18 Signaling score	AB4_Dp18 Signaling score
-0,886482	-0,513104	0,00846228	1,32252	1,70772	1,51633
-0,139164	-0,519856	-0,88991	1,03694	1,25087	0,994572
-1,61308	-0,340411	-1,33485	0,300681	-0,0368168	0,408752
-1,02909	0,0513723	0,0465398	1,33181	1,69135	1,52091
-0,800767	-0,380819	-0,470859	1,85338	1,43491	1,61968
-1,08254	-0,661037	-0,0384358	0,649662	0,72357	-0,0384358
-1,12336	0,142955	-0,159596	-1,15839	-1,09172	-1,2512
0,455538	-0,179135	0,673114	-0,969705	-0,777214	-0,969705
-1,20269	-0,473053	-0,823874	1,39609	0,521229	0,562114
-0,994099	-0,619495	-0,821999	1,38155	0,554445	0,593135
-1,31314	-1,46799	-1,73694	0,191562	1,08153	0,0294724
-0,731801	-0,153073	-0,600734	-0,279663	-0,584276	-0,249362
-0,926974	-0,663437	-1,0786	1,28104	1,71178	1,50759
-1,09887	-0,514043	-0,166833	0,0559544	0,570882	0,241576
-0,991767	-1,13845	-0,708238	1,39842	0,530197	0,568806
-0,044891	0,383064	-0,257386	-0,359109	0,049742	0,137106
0,80952	0,484203	0,80952	-0,429078	-0,103761	0,80952
-0,306852	-0,248178	-0,0286312	0,717498	1,28931	-0,122654
1,03598	0,469693	0,305781	-0,255398	-0,400116	0,196278
-0,298772	0,178428	0,0672807	0,324835	1,3371	0,190595
-1,07286	-0,367839	-0,524248	0,739432	0,679631	0,106006
-0,921498	-0,967815	-0,760095	1,21887	1,34066	0,323173
-1,65446	-1,66392	-1,43776	0,888573	0,990223	0,61121
0,727203	0,49628	0,156762	0,74705	0,327044	0,353336
-0,23484	0,349102	0,52037	1,35456	1,43705	-0,00634774
-0,72788	-0,864933	-1,39193	1,41939	0,919514	0,601966
0,59121	0,417695	1,20578	1,32099	0,316002	1,45481
0,299555	-1,15536	-0,446672	1,31129	1,66067	1,49505
-1,125	-0,431533	0,325881	0,515192	0,569121	-0,286556
-0,282423	-0,200457	-0,427774	1,30945	1,70864	1,5194
-1,43117	-1,20418	-0,766747	0,555248	1,14178	1,18222
-0,807628	-0,475531	-0,439138	-0,0566025	0,471588	0,219944
-0,685365	-0,148926	-1,04185	1,30878	1,73197	1,53655

Polito et al. Universal ready-to-use immunotherapeutic approach for the treatment of cancer: expanded and activated polyclonal γδ memory T cells.

Anova Photon

1,05096	-0,0242991	0,864969	-0,28441	-0,484807	0,0295452
-0,81601	-0,81601	-0,81601	0,262718	0,177064	-0,174469
0,553476	0,371447	1,20308	1,32789	0,263093	1,46746
-0,982629	-0,982629	-0,347598	0,52669	0,587785	0,266814
-1,03684	-1,02895	-1,05939	1,38854	0,537978	0,557215
0,571324	0,388973	1,22322	1,34711	0,280427	1,48692
0,599994	0,439134	1,18208	1,29894	0,341149	1,41729
-0,614928	-0,869091	-0,822131	1,53068	1,15637	-0,0924745
-1,23174	-0,775348	-0,276226	1,26336	2,04482	1,20073
-0,416965	0,437051	0,425616	1,0004	0,892645	0,583985
-0,870405	-0,212877	-0,505936	1,13821	0,636105	0,0945579
0,99551	0,513047	0,709749	-1,2358	-0,308559	-1,54489
0,574533	0,985322	0,749298	0,808701	0,357002	-0,418066
-0,487206	0,145187	0,329308	1,47288	1,23121	0,684573
0,277997	0,829817	0,6282	-0,434934	-0,323197	0,325269
-0,33193	-0,455261	-0,354424	1,32224	1,70986	1,52611
-0,415942	-0,967374	-0,415621	1,36425	1,73346	1,55284
-1,09323	-0,300606	-0,85463	0,649484	0,925228	0,149767
-1,20878	-0,393655	-0,511272	1,23344	1,6874	1,4722
-0,89929	-0,89929	-0,711257	0,726711	0,387883	0,139318
-1,33523	-0,479562	0,14092	1,05122	0,918122	0,542441
-0,983744	-0,983744	-0,349987	0,525726	0,599033	0,270198
-1,13106	-0,438471	0,302882	0,525767	0,577469	-0,236216
-0,346234	-0,666047	-0,688889	1,40532	1,80615	1,61613
-0,990001	-1,00491	-0,76974	0,928203	1,34225	0,313175
-0,402818	0,443351	0,424309	1,00479	0,893374	0,588935
-0,277997	0,188477	0,0839481	0,336647	1,32377	0,22222
-0,303805	1,18576	1,00064	-1,05288	-0,914481	-0,966251
0,159076	0,427139	-1,18951	0,293898	0,863653	0,428364
-0,639095	-0,0820005	0,0924676	1,32066	1,73409	1,54318
-0,867513	-0,519056	0,219534	1,27962	1,70178	1,50684
-1,12096	-0,44227	0,236419	0,543643	0,645958	-0,228167
0,673595	0,45249	0,673595	0,45249	-0,784482	-0,391875
0,404033	0,57798	0,552878	0,265832	-0,227065	0,706316

Anova Photon

-0,949797	-0,949797	-0,203984	0,482592	-0,203984	0,793143
-1,37275	-0,753843	-0,834147	1,09505	1,03285	0,517879
-1,01377	-1,01377	-1,01377	0,82413	1,41034	0,510734
-1,00589	-0,0322595	-0,937837	1,27612	1,66162	1,15366
0,486387	0,0720167	0,981112	1,07709	0,0520389	0,183806
-0,333566	-0,267288	-0,628496	0,211587	0,228402	0,133717
-0,3322	-0,985044	-0,271119	1,29363	1,70538	1,50079
-0,53906	-0,770787	-0,880667	1,08274	1,48336	0,187342
-0,783316	-0,289441	-0,0177915	1,32273	1,70495	1,51503
-0,388319	0,2211	0,407215	1,5136	1,09721	0,746796
-1,14989	0,317499	0,436386	-1,44774	-1,04845	-1,42917
0,343965	0,206979	1,25332	0,511836	-0,0470106	1,15302
0,645815	1,18671	0,630803	-0,638354	-1,18192	-0,598469
-1,04571	-0,567289	-0,332911	0,621765	0,699839	-0,203508
0,810646	0,439836	0,474854	-0,942312	-0,998976	0,0108994
-0,769053	0,250858	-0,459311	0,458599	1,12041	-0,0498557
1,15362	0,38663	0,420513	-0,357908	-0,497818	0,032017
0,615459	0,435423	-0,464582	0,783638	0,632727	0,769076
-1,1617	-0,816776	-0,929831	0,605738	1,17444	1,06914
-0,246086	0,262837	0,134595	0,275161	1,34395	0,0246144
-1,21664	-0,770727	-0,502982	-0,100636	-0,164384	-0,17472
-1,15488	-0,749735	-0,563733	1,19882	1,05961	1,08436
0,979228	0,620705	0,238172	-0,285944	-0,398453	-0,0586343
-0,708824	-0,407167	-0,203272	0,657284	1,19699	0,495014
1,51537	0,686877	0,906904	0,0602228	-0,055487	-0,038759
-1,25858	-0,172979	-0,386366	0,566042	0,628937	0,217819
0,370725	0,0193701	-0,877717	1,51373	1,39713	1,56487
0,584121	1,04184	0,475913	0,851492	0,394186	0,128229
-0,223002	-0,131401	0,0364401	0,749143	1,18677	0,0265794
0,471935	0,322525	0,397675	-1,47554	-1,01664	-1,93402
-0,45631	0,335939	0,569833	1,50872	1,17637	0,307842
-0,892794	-0,920547	-0,747157	1,42323	1,45895	0,368421
-0,309141	-0,49474	-0,475028	-0,767487	-0,439527	-1,71633
-0,968071	-0,122242	-0,626569	1,2129	0,611767	-0,0595462

Anova Photon

-0,607478	-0,327689	-0,24004	1,20401	1,57801	1,40531
-0,987844	-0,203093	-0,646502	1,06748	0,951237	-0,229
-1,22742	-0,657269	-0,637711	1,3019	1,27135	0,992502
1,23285	-0,0152881	0,138806	-0,330758	-0,53798	0,0559845
1,18181	0,436561	0,291914	-0,474996	-0,589168	0,0889568
-0,832369	-0,548728	-1,12603	0,187187	0,035469	-0,475484
-0,42299	0,461855	0,443373	1,00969	0,906744	0,556582
-0,211765	0,64933	0,089898	-1,57	-0,612834	-1,49607
0,18362	0,0859319	0,674571	-0,238837	0,674571	0,18362
-0,989032	-0,989032	-0,429126	0,778734	0,522131	0,45232
-0,905424	-1,48602	-0,888391	-0,50097	-0,702282	-1,04121
-0,252258	-0,184559	-1,058	1,32073	1,2768	1,4983
0,625353	0,464179	1,19721	1,30475	0,371395	1,42753
-0,986042	-0,455303	-0,610101	1,27376	1,66707	1,47989
-0,838557	-0,317148	-0,289385	1,16776	1,53492	1,3553
-1,27186	-0,303958	-0,986954	0,902973	1,29876	1,0221
-0,949426	-0,163831	-0,367459	0,194723	0,821767	0,105824
-0,639961	-0,529976	0,198767	1,28972	1,74275	1,52112
-0,626984	-0,00110429	0,0090551	1,26416	1,62112	1,45629
-1,20427	-0,677298	-0,662788	1,0743	1,57873	0,219965
-0,374184	0,268809	0,461142	1,62207	0,974512	0,511766
-0,504689	0,240884	-0,49184	1,32414	1,80346	1,56523
-1,00886	-0,843009	-0,794563	1,2582	1,61873	1,37563
-0,976445	-0,656315	-0,411757	1,73431	1,69774	1,19596
1,18021	0,3257	-0,152038	1,08699	0,98554	0,501931
-1,11669	-0,460527	-1,05321	0,792498	1,37044	0,492659
-0,994361	-0,240348	-0,68993	1,05251	0,934852	0,0840634
-0,829932	-0,504931	-0,772956	-0,154443	-0,161564	-0,548041
0,716353	0,388645	0,716353	-0,266771	0,196948	0,716353
-0,276375	0,178198	0,0855112	0,33817	1,32417	0,201549
-0,788046	-0,235184	-1,02152	1,20349	1,63604	1,42112
1,15512	0,323584	-0,152816	1,09492	1,00486	0,500466
1,15866	0,309231	-0,16042	1,09837	1,0082	0,499049
-0,687059	-0,972442	-0,699906	0,766268	-0,33952	-0,639547

Anova Photon

-0,720732	-1,14416	-0,355505	0,75322	1,33944	0,442475
0,370725	0,0193701	-0,877717	1,51373	1,39713	1,56487
0,393292	0,970423	0,151932	-1,52437	-1,4732	-0,530583
0,436658	0,571975	1,0143	-1,95685	-0,994799	-1,09909
1,06452	1,11042	-0,28137	-0,794804	-0,555749	-0,860369
-1,19668	-0,132032	-0,960575	1,29979	1,68233	1,50284
-0,236446	1,00404	-0,129563	-1,1511	-0,573376	-1,26082
-1,30983	-0,746277	-0,292409	0,761612	1,33505	0,457647
-0,861878	-0,387173	-0,192647	1,5438	1,61039	1,61975
-1,20167	-0,937196	-0,656746	0,770773	1,39446	0,450137
-0,965966	-0,137243	-0,358937	0,267689	0,844925	0,131111
0,556231	0,36477	1,23555	1,36149	0,254551	1,50915
0,0888979	0,176233	1,04677	-0,491724	-0,0910229	-0,724034
-0,928151	-0,775702	-0,532401	1,03318	1,35461	0,494482
-1,12272	-0,840026	0,143016	1,79419	1,64	1,08478
-0,994206	-0,240036	-0,689711	1,05309	0,933947	0,0823451
-0,155476	-0,28826	-0,795341	1,59897	1,48224	1,65018
0,675638	0,384525	0,393573	0,597666	0,442058	1,20795
1,17859	-0,0215338	0,349805	-0,225502	-0,900149	0,0342273
-0,838462	0,0589088	0,0373094	1,50334	1,74973	1,41415
-0,983221	-0,983221	-0,348651	0,521791	0,591314	0,27233
0,955958	0,308446	0,998046	0,0221775	-0,639557	0,249714
-0,0405516	0,638372	-0,150089	0,134468	1,07905	-0,129978
-0,90564	-0,90564	-0,90564	0,70591	0,524847	0,534674
1,43551	0,297357	0,277152	-0,23499	-0,193951	0,153917
-1,07333	-0,888417	-0,959891	1,37085	1,30098	0,18907
1,05023	0,501527	0,779448	0,219524	-1,16795	0,302489
1,1816	0,324749	-0,154296	1,08813	0,986394	0,501462
-0,961175	-0,362011	0,301016	0,22729	-0,145839	0,649313
-0,792762	-1,14218	0,413153	1,31292	1,74276	1,54427
0,731936	0,359648	0,141874	0,359648	0,359648	0,731936
-1,16795	-0,876892	-0,591408	0,0955101	0,0236188	-0,0748724
-1,00058	-0,140633	-0,607523	1,21775	0,651421	-0,234286
0,617557	0,808413	0,651057	0,840253	0,413081	-0,442076

Anova Photon

0,612642	0,451898	1,18911	1,29512	0,35753	1,41846
-0,295853	-0,246633	-0,0156137	0,680085	1,26313	-0,119692
1,06469	0,337373	0,167099	-0,147742	-0,612158	0,142986
0,0813302	-0,436579	-0,544609	1,35528	1,75254	1,5647
0,695384	0,695384	0,343058	-0,361593	0,695384	0,695384
-1,19474	-0,509482	-0,157845	0,168519	0,508378	0,530055
-0,470396	-0,681066	0,370453	1,23883	1,58756	1,41967
-0,878122	-0,15954	0,0890546	-0,469325	0,296238	-0,917777
-0,592509	-1,14587	-0,680474	1,65066	1,00329	0,863009
-1,10878	-0,656913	0,295727	1,30884	1,69199	1,51614
-0,751328	-0,366498	-0,0267092	1,30215	1,67015	1,50345
-1,08996	-0,559784	0,29819	0,453117	0,615006	-0,354685
-0,782026	-0,372633	-0,554577	1,56433	1,62955	1,63796
-0,952187	-0,566182	-0,342347	1,42998	1,80873	1,27729
1,15939	0,325819	-0,154917	1,08506	1,00877	0,494962
-1,01124	-1,01124	-1,01124	0,886596	1,3592	0,68375
0,21039	0,931237	0,0329779	0,0184079	0,825582	-0,637983
-1,04278	-1,02961	-0,829208	0,707954	1,21606	0,753335
-0,805714	0,269072	-0,410807	1,39817	1,94512	1,22928
-1,07695	-0,689274	-0,171836	0,167424	0,615378	0,258406
-1,09457	-0,00262749	-0,318738	1,27219	1,66966	1,48612
-1,02377	-0,972942	-0,556914	0,584161	1,17322	1,05872
-0,419386	0,494363	-0,686055	1,25245	1,20508	1,44395
-0,922	-0,00918886	-0,170228	-0,785604	-0,0742734	-0,693683
1,19616	0,329842	-0,156251	1,08509	0,983268	0,497919
-0,854363	-0,854363	-0,124456	-0,00696737	-0,48941	0,57147
-0,810691	-1,16031	-1,04479	1,39508	1,81362	0,355986
-0,95915	-0,95915	-0,333059	0,476151	-0,0200138	0,816576
-1,15119	-0,23918	-0,23918	0,224953	0,727217	-0,08031
-0,0659401	0,381728	0,805514	0,121935	-0,0927902	-1,19459
0,851854	0,809608	0,809608	0,601547	0,474706	0,567611
-0,965788	-0,136388	-0,622257	1,2162	0,620271	-0,0695866
-0,429927	0,451602	0,436931	0,994051	0,892048	0,563305
-1,00092	0,137143	-0,891627	1,03245	1,30496	0,634576

Anova Photon

0,862463	1,13842	-0,323957	-0,901851	-0,267641	-1,17437
1,14389	0,815324	0,742253	-0,691133	-0,194437	-0,25597
-0,465729	-0,823165	-0,71522	1,13598	1,51612	0,172974
-0,609321	-0,24265	0,642874	0,0779926	-0,339097	0,242063
-1,12622	0,0718208	-0,580267	0,361588	0,731887	0,0892372
-1,31003	-0,783923	-0,411312	1,00532	1,29746	0,955364
-1,25736	-0,481556	-0,156953	0,394592	0,317457	0,296909
-1,26171	-0,119879	0,317888	0,485393	0,350558	0,296555
-0,628922	-0,632147	-0,848482	0,797153	1,55029	0,765626
0,628584	0,430017	-0,464647	0,786229	0,634229	0,778045
0,862393	0,577031	0,622894	0,615628	0,594296	0,0758308
1,73045	1,20857	1,72443	-0,671267	0,0210475	-0,549402
0,574414	0,395416	1,21895	1,34671	0,286801	1,47968
-0,765221	0,423917	-0,454564	0,340939	1,11387	-0,12204
-0,825782	-1,11197	-1,02406	0,96386	1,15354	0,351904
0,658094	0,600251	1,20456	0,664874	0,71047	0,577006
1,17035	-0,0320729	0,346509	-0,227752	-0,901	0,0214082
-0,131083	-0,560034	0,46379	1,23085	1,65141	1,45721
-1,0366	-1,0366	-1,0366	0,88038	1,36067	0,576161
1,52138	1,44742	0,602122	-0,713643	-1,30827	-1,7294
-0,765046	-0,780231	-1,38943	1,50258	0,928702	0,623411
-0,627423	-0,0092557	-0,951345	1,33731	0,687648	0,28303
0,402706	0,816029	0,402706	-0,867568	0,198467	-0,225557
-0,109499	-0,216823	-0,909689	1,38699	1,80829	1,60218
-0,305009	-0,248421	-0,021869	0,672628	1,27216	-0,12211
1,06676	1,11303	-0,271526	-0,802337	-0,56092	-0,863485
-0,96174	-0,204065	0,0172002	1,28127	1,67769	1,48976
-0,752501	-0,81023	-1,08358	0,74377	1,26682	0,614502
0,359001	0,441646	0,449124	0,563738	0,270507	0,733062
1,1818	0,324627	-0,154598	1,08829	0,98652	0,501406
-0,35909	0,1403	0,42048	1,37113	1,08853	0,640624
-1,11202	-0,540956	-0,201918	0,205641	0,668227	0,268888
-1,02256	-0,302075	-1,20109	0,264997	1,53773	0,234419
-1,01446	-0,240545	-0,283526	0,233953	0,819687	-0,149516

Anova Photon

1,47732	0,395	0,753091	-0,187886	-0,207911	0,0390134
0,622211	0,432043	-0,463556	0,784549	0,636468	0,776284
0,355302	-0,714694	-0,948826	1,28377	1,70246	1,49442
-1,04938	-0,675209	-0,549258	1,15867	0,629939	0,572716
-0,508513	-0,891673	-1,15939	1,23392	1,32325	0,872628
-0,686469	-0,160019	-0,728968	1,35724	1,75521	1,57144
-1,08372	-0,0491274	-0,726074	0,522662	0,74596	0,0828687
0,162006	-0,30841	-0,895185	1,1199	1,63052	1,37805
-0,982629	-0,982629	-0,347598	0,52669	0,587785	0,266814
1,30149	0,0903744	0,640405	-0,867045	-0,999574	-0,0219119
-1,12216	-0,442225	-0,225922	0,12014	0,575942	0,408995
1,07069	0,862891	0,380608	-0,059954	-0,713145	-1,13492
-1,35236	-0,677537	-0,870906	0,909874	1,31244	1,02774
-0,730438	-0,730438	-0,730438	0,0957374	0,162618	-0,507174
-0,908058	-0,908058	0,0600492	0,302076	0,608672	-0,524455
0,53909	-0,733176	-0,750316	1,27432	1,23269	0,482361
0,861253	-0,178389	0,95378	-0,125171	-0,559486	-0,703979
-0,798747	0,228911	-0,419969	1,28772	1,27926	1,9817
-1,09224	-0,600711	-0,182801	0,158081	0,608177	0,26526
-1,12987	0,0379952	-0,709287	1,32615	1,75241	1,55034
-0,670544	-0,0399398	-0,0940252	0,125286	0,786341	0,111423
-0,0055668	0,626615	-0,0737815	0,131568	1,05916	-0,249072
-1,07224	-0,828843	0,0287909	0,503949	0,716436	0,133607
-0,742191	-0,868219	-0,517292	1,71797	1,26318	1,26318
0,139308	-0,582878	-0,335805	1,25067	1,71314	1,49959
-0,553449	-0,250975	-0,43212	1,35338	1,98263	1,15909
-0,972805	-0,283775	-0,379307	-0,20572	0,994449	-0,379307
0,451619	0,793791	0,740264	-0,734511	-0,255831	-1,5082
0,370725	0,0193701	-0,877717	1,51373	1,39713	1,56487
-0,751865	-0,661551	-0,352039	1,40562	1,79197	1,53501
0,633037	0,57398	0,742377	1,33377	0,365888	1,5452
-0,98587	-0,74512	-1,14668	1,07423	1,00511	0,526828
-0,989386	-0,674267	-0,476585	0,834229	0,836604	0,754875
-0,975328	-0,975328	-0,975328	0,638817	1,11954	0,295988

Anova Photon

0,755085	0,649021	0,853131	-1,48519	-0,712028	-1,15221
0,584938	0,453713	1,09025	1,38552	0,300206	1,54388
1,06529	1,1112	-0,280628	-0,794076	-0,560296	-0,849696
-1,00116	-0,658006	-0,771863	0,227086	-0,0261623	-0,830152
-1,25077	-0,544101	-0,625985	1,19907	1,07922	1,06354
-1,25063	-0,834391	-0,362802	1,25576	1,69635	1,47743
-0,451275	0,578555	-0,547792	-0,649335	-0,517841	-0,978109
1,21388	0,587732	0,652552	0,0264091	-0,534914	-0,863267
-0,964474	-0,964474	-0,596288	0,830717	0,748743	0,197023
-0,994347	-0,239299	-0,689498	1,05317	0,933869	0,083457
-1,12056	-0,786036	-0,702471	0,618555	1,15704	1,04895
1,31297	0,103224	0,274458	-0,243451	-0,902825	0,046695
0,312704	0,767421	0,312704	-0,467115	0,0880109	0,183242
0,585256	0,527886	1,10789	1,29923	0,312683	1,509
0,370725	0,0193701	-0,877717	1,51373	1,39713	1,56487
-0,411109	-0,0304311	0,956917	-0,657371	-0,517337	-0,93061
-0,956611	-0,173117	-0,350206	0,224783	0,815581	0,125383
1,06668	1,10864	-0,275681	-0,794827	-0,564784	-0,868053
-1,34366	-0,723157	-0,503349	1,33294	1,7876	1,57765
-0,0563103	-0,20302	0,235684	1,30868	1,33107	0,42925
-0,587519	0,575577	-0,846727	-0,655949	-0,657957	-0,519583
-0,865279	-0,660473	-0,919026	1,203	-0,358204	0,464913
1,17432	0,308543	-0,154829	1,09795	0,996493	0,512867
0,370725	0,0193701	-0,877717	1,51373	1,39713	1,56487
-0,808146	0,0405087	-0,359823	-0,110537	-0,0287879	-0,40411
-0,374569	-0,223756	-0,238403	1,31294	1,71995	1,527
-0,843615	-0,843615	-0,843615	0,632312	0,353406	0,100358
1,16823	0,319494	-0,155087	1,09189	1,00204	0,498803
-1,16452	0,442723	-0,76069	0,348197	0,571931	0,607905
-0,0753065	-0,0753065	-0,351648	-0,351648	-0,824057	-0,351648
-1,07642	-0,388419	-0,333942	0,631794	0,58964	-0,00568429
0,684558	0,750997	0,448458	0,621062	0,752061	0,672045
-0,569133	-0,521371	-1,56665	0,174252	0,337378	-0,754251
-0,830296	-0,830296	-0,830296	0,448469	0,579587	-0,160224

Anova Photon

-1,26675	-0,0572566	-0,425635	1,334	1,72267	1,53842
-1,1837	-0,967011	-0,861745	0,581641	0,908216	0,24416
-0,952876	0,0405723	-0,0175301	0,641228	1,37775	-0,161508
-0,968955	-0,124056	-0,627829	1,21135	0,613035	-0,0501877
-0,902623	-0,0430615	-0,626645	1,23558	0,723819	-0,252763
-0,854363	-0,477093	-0,616332	0,385646	-0,099824	-0,378302
-1,02496	-0,256296	-0,817239	1,10172	0,541898	-0,203585
-0,773624	-0,53242	-0,300103	0,0650962	0,723521	-0,378687
-0,830695	-0,830695	-0,830695	0,455814	0,578582	-0,160911
0,559057	0,072094	-0,181454	1,18326	1,21207	0,895316
0,671669	0,605201	0,77178	1,34301	0,418237	1,54554
-0,830306	-0,830306	-0,830306	0,448574	0,579704	-0,160173
-1,12254	-0,177094	-0,26154	0,358945	0,132987	0,116197
-0,908239	-0,0758564	-0,5777	1,33539	1,76756	1,55282
-0,829883	-0,829883	-0,829883	0,44093	0,580612	-0,15952
1,17518	1,02531	1,22282	0,0200997	-0,0735162	-0,175222
1,54687	0,673746	0,809085	-0,0796778	-0,915851	-0,0150294
-0,574773	-0,611977	-1,11826	-0,410712	-0,0611277	-0,844181
-0,447912	0,162986	0,342683	1,44713	1,21347	0,673481
-1,09274	-0,860405	-0,123919	0,666005	0,683197	0,616679
-0,685672	-0,240019	-0,499372	-0,0476428	-0,0334816	-0,870398
-1,89348	-1,35259	-1,54014	0,403015	0,403864	0,748173
-0,988368	-0,646423	-0,430679	0,29621	0,174989	-0,145482
-1,14411	-1,12048	-0,673411	1,21645	1,63236	1,44031
-1,08243	-0,519619	-0,584098	0,582021	1,27597	-0,188326
0,943295	-0,182436	0,970667	-0,317286	-0,910695	-0,406287
-0,961767	-0,133029	-0,38075	0,242117	0,842905	0,148801
0,908437	1,09628	1,04709	-0,763058	-0,89003	-1,32
-0,270088	-1,11326	-0,511697	-0,136187	-0,146908	-0,850827
-1,24148	-0,733041	-0,945321	-0,00286257	0,927797	-0,0680975
-0,983612	-0,107639	-0,388879	0,334233	-0,337713	-0,29339
-0,598732	-1,67485	-0,389572	1,35594	1,35594	1,35594
-1,68199	-0,891185	-2,03236	0,568581	0,631706	0,513309
0,780175	0,901322	0,992052	-0,870333	-1,07384	-1,54463

Polito et al. Universal ready-to-use immunotherapeutic approach for the treatment of cancer: expanded and activated polyclonal γδ memory T cells.

Anova Photon

-0,677066	0,258974	-0,0854746	-0,398765	-1,17252	-1,12039
0,0772831	-1,01025	-0,0998344	1,3053	1,7498	1,53908
-1,19054	0,0684674	-0,525136	1,04556	1,02757	-0,0658768
-0,0606715	0,214882	0,184636	1,59088	0,75974	0,427561
1,20218	0,223334	0,357083	-0,231461	-0,515559	0,0434543
0,934343	1,02264	1,54006	1,01127	-0,240248	0,829208
-0,332067	-0,601677	-0,710435	1,30104	1,69438	1,51565
-1,0845	-0,770115	0,0425486	1,14256	1,10662	0,724732
0,967897	-0,356942	0,995947	-0,508757	-1,21817	-0,609647
0,596082	0,620991	1,05996	0,655538	-0,704281	0,314611
-0,832784	-0,832784	-0,832784	0,607962	0,453694	-0,112411
-0,699806	-0,699806	-0,699806	0,204284	0,0653722	-0,699806
0,0987724	0,290708	-0,990653	1,53703	0,974007	1,59089
-0,696026	-0,696026	-0,696026	0,0389014	0,168316	-0,696026
-0,994565	-0,238907	-0,689469	1,05461	0,933764	0,0841106
-0,265679	-0,516613	-1,26476	1,09762	1,81529	1,69705
0,732215	0,572883	0,505312	-0,297897	-0,017029	-0,185482
-0,968977	-0,968977	-0,602695	0,877196	0,741355	0,243811
-0,9887	-0,320883	-0,350714	-0,523023	-0,225478	-1,83998
-0,96164	-0,727925	-0,998897	1,30519	0,720258	0,0114692
-1,59537	-0,997033	-1,74687	0,635712	-0,152726	-0,257579
-0,187776	-0,232968	0,622196	-1,39353	-0,241314	-1,17276
-0,35278	0,219343	0,388249	1,42345	1,28527	0,318815
-0,682613	-0,388184	-0,363346	1,34298	2,07904	1,30271
0,604401	0,316205	1,0829	0,525229	0,580947	0,500416
-0,875185	-0,875185	-0,875185	0,443564	0,598908	0,157415
-0,757159	-0,522925	-0,372663	1,4279	1,82511	1,62775
-1,11187	-1,11187	-0,317295	0,408655	0,672561	0,0521591
-2,17768	-1,44244	-1,14925	0,131071	0,618921	0,374606
1,63836	0,808906	0,709609	-1,36566	-0,326423	-0,753322
-1,10425	-0,743977	-0,708183	0,591713	0,730872	0,644369
2,18785	1,09733	1,01374	-0,0678332	0,0902135	-0,529888
-0,98067	-0,98067	-0,620228	0,891771	0,79189	0,39391
-0,924813	-0,924813	-0,924813	0,158455	1,111	0,0482261

Anova Photon

-1,10186	-1,10186	-1,10186	0,80478	1,38796	0,621684
-0,962867	-0,962867	-0,593419	0,820562	0,74594	0,180909
-0,307116	-0,24844	-0,0263329	0,716042	1,28907	-0,122914
-0,782703	-0,0495888	-0,836729	-1,09103	-0,249335	-0,722843
-1,04558	-0,56718	-0,332811	0,621827	0,697376	-0,203414
-0,669756	-1,15628	-0,947128	1,14849	1,56224	0,31519
-0,737673	-0,501726	-1,11958	0,684052	1,25384	0,514114
-0,85192	-0,734326	-0,546467	1,28257	0,560611	0,394318
-1,24519	-0,687403	-0,618859	0,965877	1,05212	0,397766
-0,426001	-0,795574	-0,563358	0,519173	-0,174431	1,1266
-0,479239	0,527847	0,459867	0,74021	1,18745	0,157539
-0,905421	-0,200017	-0,905421	0,778198	0,947704	0,132144
-0,529066	-0,601397	0,0187573	-0,277786	-0,308524	-0,30406
1,22063	0,14229	0,410934	-0,0619356	-0,83136	0,0576417
-0,910491	0,258242	-0,696113	1,30518	1,79922	1,57109
0,176561	0,615686	-0,400434	0,176561	1,02065	0,213444
-1,27311	-0,414355	-0,253526	-0,279615	-0,0505898	-0,545797
-1,12873	-0,480735	-0,304973	1,21666	1,60056	1,41497
-0,733799	-0,733799	-0,733799	0,229095	0,0906321	-0,511006
-0,919716	-0,261802	-0,19426	0,20144	0,671596	-0,0280666
1,13059	0,0392531	0,234275	-0,186411	-0,443573	0,391758
0,523936	1,28574	0,464856	-0,246915	-1,00499	-0,213001
0,625935	0,427494	-0,466607	0,779409	0,634372	0,771159
-0,741779	-0,697594	-0,570483	1,37971	0,506925	0,516034
1,27254	0,369104	-0,112246	0,916691	0,970423	0,380622
-0,782292	-0,229373	-0,358105	0,0908183	0,612673	0,0336426
-0,76089	0,16053	-0,535673	0,031528	0,00592934	-0,830068
0,81262	0,713831	0,940139	1,49431	0,670082	0,770234
-0,928649	-0,980072	-0,0695482	2,03654	1,05845	0,801369
-1,24168	0,200391	-0,706414	1,25608	0,883582	0,772316
1,3116	0,111636	0,269692	-0,240289	-0,911444	0,0601933
-1,01599	-0,49221	0,232394	1,2667	1,67413	1,48492
1,16973	-0,0261118	0,345556	-0,231301	-0,901733	0,0231347
-1,11677	-0,449237	-0,755857	0,749243	1,37248	0,426931

Anova Photon

-0,947115	-0,947115	-0,673855	0,710503	0,233894	0,799648
-0,602414	-0,540855	-0,828064	0,881142	0,159388	0,508337
-1,02063	-0,837981	-0,715169	1,23774	0,467354	1,39157
1,10587	0,833082	0,261826	0,0153509	-0,155064	0,161623
-1,15761	-0,847637	-0,0168596	0,519157	0,404754	0,325797
0,564464	0,056805	-0,186654	1,18315	1,21267	0,924729
0,500702	0,353903	1,07252	1,41014	0,178128	1,58008
-0,800151	-0,096197	-0,622832	1,39546	2,00878	1,20268
-1,03055	-0,660082	-0,381761	1,39361	1,75943	1,57767
-0,478549	-0,0844023	-1,1915	-0,2828	-0,2828	-1,3616
-0,495414	0,0667251	-0,393235	-0,264938	0,537112	-0,245183
-1,08687	-0,676148	-0,745152	0,904659	1,3393	0,165712
-1,12357	-0,43016	0,312081	0,506815	0,570423	-0,285193
-0,916425	-0,282286	-0,74258	1,08768	0,941486	-0,18789
0,812986	0,812986	0,504969	-0,446021	-0,163404	0,812986
-0,970518	-1,09316	-0,336732	1,21242	1,08538	1,06876
-0,687617	-0,0450103	-0,260762	0,0598224	0,838966	0,0778963
-1,08367	-1,08367	-1,08367	0,938693	1,46261	0,594488
0,370725	0,0193701	-0,877717	1,51373	1,39713	1,56487
-0,963489	-0,671975	-0,237318	1,29275	1,67516	1,49387
-0,897799	-0,355476	-0,238701	1,38462	1,76857	1,58655
-0,84295	-0,276429	-0,0516262	1,37692	1,76453	1,57194
0,592941	0,428055	1,18562	1,30435	0,328003	1,42683
1,28794	1,17139	0,406132	0,86533	0,335464	1,03681
0,59434	0,59434	1,29726	-0,476716	-0,834774	-0,586671
-0,457773	-0,714104	-0,28889	-0,309615	1,12065	-0,507428
-0,751231	-1,44439	-1,2472	1,13391	-0,20508	0,943488
-1,0478	-0,73005	-0,155755	0,631655	-0,0405469	0,736294
-1,20861	-0,0933347	-0,201416	1,11427	1,02194	0,28856
-1,13187	-0,851226	0,00345982	1,39101	1,48572	1,62795
0,555546	0,42069	1,07325	1,38094	0,263547	1,53668
-0,96721	-0,174258	-0,720452	1,05953	0,921976	-0,020407
-1,19585	-0,374959	-0,214895	0,956036	1,11891	0,61414
0,370725	0,0193701	-0,877717	1,51373	1,39713	1,56487

Anova Photon

-0,994359	-0,240483	-0,689984	1,05214	0,935971	0,0838688
-0,607826	-0,434681	-0,227127	1,05126	0,923408	0,0550659
1,17979	0,334181	-0,152229	1,08659	0,985153	0,501628
1,19441	0,330338	-0,154491	1,08362	0,982064	0,497978
0,0206873	0,137926	0,156901	-0,23975	-0,211678	-0,734667
1,18814	-0,0607238	0,245947	-0,239705	-0,902067	0,0257447
-0,306995	-0,248333	-0,0288353	0,717129	1,28881	-0,122837
1,1564	0,327552	-0,154221	1,09597	1,00559	0,499338
-1,2819	-1,37652	-1,48047	1,44778	0,0331064	0,684522
-0,988894	-0,988894	-0,988894	0,897222	1,49881	0,394481
-1,62898	-0,755702	-0,827945	-0,517977	-0,841068	-0,81351
1,1818	0,324627	-0,154598	1,08829	0,98652	0,501406
-0,596448	-1,1928	-0,633727	0,7706	1,38561	0,444599
1,16957	0,311978	-0,155601	1,09294	0,991148	0,505909
-0,307134	-0,248478	-0,0290027	0,716884	1,28851	-0,122995
-1,32652	-0,940306	-1,63114	-0,168313	0,363716	-0,222
-0,39987	0,262636	0,459305	1,65885	0,990472	0,52114
1,33285	1,43506	1,04242	-0,656611	0,286584	-0,765758
-1,1807	-0,641847	-0,693942	1,20251	1,10856	0,647434
0,205933	0,238832	0,100427	1,0414	1,09622	1,19425
-1,08522	-0,126404	-0,425145	0,300382	0,819552	0,240007
-0,972656	-1,10922	-0,146432	0,340247	1,18081	0,854275
0,594467	0,42182	1,21579	1,34094	0,318656	1,46806
0,166489	0,261054	0,735968	0,16118	-0,0773756	-1,18887
1,17003	-0,0351198	0,345907	-0,227357	-0,899148	0,0263453
-0,987566	-0,825965	-0,815388	0,913371	1,235	0,247569
-0,915704	-0,542818	-0,247908	1,35097	1,79918	1,57991
-1,59093	-1,02771	-1,26208	0,952018	0,874523	0,730257
-0,813541	-0,531995	0,292612	-0,14982	-0,873469	-0,537676
-1,01216	-1,01216	-0,2407	0,961943	1,37872	0,0577411
-0,942053	-0,000652623	0,341549	1,05557	0,940393	0,601019
-0,7143	-0,399548	-0,501336	-1,0581	0,527341	-0,428776
0,811697	0,811697	0,811697	0,0412469	-1,08744	0,325597
0,140702	0,481695	0,213867	0,25088	0,763073	0,4663

Polito et al. Universal ready-to-use immunotherapeutic approach for the treatment of cancer: expanded and activated polyclonal γδ memory T cells.

Anova Photon

-1,17147	-0,691808	-0,688935	-0,890822	0,21629	-0,205051
-1,76647	-1,58823	-1,5736	0,625797	0,681988	0,449552
-0,781927	-0,781927	-0,781927	1,03891	0,436031	-0,781927
-1,22791	-0,315512	-0,454566	1,32824	1,75416	1,54579
-0,51558	-0,87235	-0,972002	0,794313	1,39147	0,467276
-0,671334	-0,0221228	-0,502141	2,03296	0,445471	1,8995
0,81514	0,809523	0,539132	0,441673	0,373633	-0,0800777
0,954084	-0,199672	0,978554	-0,328299	-0,953603	-0,419394
-1,12752	-0,195168	-0,154276	0,309153	0,194663	-0,141775
-0,730512	-0,730512	-0,730512	0,095783	0,162674	-0,507216
-0,851571	-0,381429	-0,851571	0,291193	0,233222	-0,649092
-1,44948	-0,45435	-0,644994	1,56546	1,18586	1,58812
-1,65712	-0,564579	-0,877027	1,28873	1,80675	0,809966
-1,28717	-1,12067	-0,498736	1,21409	1,61094	1,42769
-0,758016	-0,69025	-0,341083	-1,11922	-0,18758	-0,769964
-0,349967	0,0933214	-0,574604	1,2833	1,688	1,50112
-1,08881	-0,196057	-1,20635	1,31498	1,27971	0,297445
-1,01242	0,326256	-0,767622	1,09265	1,62834	1,69495
0,56086	-0,227536	0,902778	0,616455	1,08136	0,686869
-0,999717	-0,0465896	0,211982	-0,218029	0,330323	-0,515069
-1,04367	-0,519789	-0,0712104	1,31655	1,78228	1,55087
-1,11878	-1,03708	-0,726907	1,14776	1,39125	0,186693
1,25558	-0,303868	-0,346991	-1,24875	-1,13814	-0,860466

Polito et al. Universal ready-to-use immunotherapeutic approach for the treatment of cancer: expanded and activated polyclonal γδ memory T cells.

Anova Photon

GD1_t0 Signaling score	GD2_t0 Signaling score	GD3_t0 Signaling score	GD1_Dp18 Signaling score	GD2_Dp18 Signaling score	GD3_Dp18 Signaling score
-0,673565	-1,00749	-0,974413	-0,269975	-0,599051	0,369046
-1,13942	-1,09206	-1,50514	0,576766	0,410637	1,01576
-0,485394	-0,905988	0,0801835	1,18916	1,46045	1,27732
-0,628325	-0,614124	-1,13282	0,021568	-0,894352	-0,364833
-0,461868	-0,712527	-0,708439	-0,345708	-0,563137	-0,463847
-1,08254	-1,08254	-1,08254	1,62888	1,29712	0,768835
1,54135	1,23941	1,19182	0,191967	0,472982	0,0037702
1,186	1,78328	1,07991	-1,19727	-0,697055	-0,387754
1,30989	0,828368	0,804577	-0,810899	-1,3539	-0,757843
1,29956	0,845097	0,822583	-0,895276	-0,801209	-1,36429
1,02749	0,333327	0,169179	0,190279	0,224712	1,27053
-0,267669	-0,571424	-1,06372	2,42655	0,920584	1,15459
-0,569247	-0,168699	0,0893123	-0,0655705	0,034808	-1,152
-0,847001	-1,09887	-1,09887	2,00156	1,15645	0,798078
1,31494	0,834404	0,812404	-0,543046	-1,28374	-0,79392
-1,88068	-1,47468	-0,345041	1,20096	1,24384	1,34708
0,80952	0,484203	0,80952	-1,91076	-1,50976	-1,06264
-1,40013	-1,82563	-0,863337	1,00812	0,815208	0,965275
0,857333	1,03598	0,857333	-0,486694	-1,68495	-1,93121
-1,46897	-1,46897	-1,46897	0,987582	0,506827	1,11304
-1,07286	-1,07286	-1,07286	1,68385	1,28807	0,686556
-1,05257	-0,697432	-1,10913	1,16197	0,694028	0,769842
0,139124	0,142002	0,657481	0,618672	0,652331	0,0565266
0,896608	0,0121527	0,952276	-1,16936	-2,07325	-1,4261
-1,47476	-1,37217	-1,45768	0,545462	0,408931	-0,0696765
-0,9617	-0,786724	-0,796061	0,577316	0,884848	1,12619
-0,605535	-1,06929	-0,876854	-0,538492	-1,24197	-0,974344
-0,522613	-1,31857	-0,54031	-0,204539	-0,0148847	-0,563617
-1,125	-1,125	-1,125	1,51491	1,30442	0,988554
-1,133	-0,0569501	-1,39482	-0,0844326	-0,311058	-0,646577
-0,512216	-0,790758	-0,64672	1,06411	0,21734	1,1911
-0,495153	-0,982815	-1,58271	1,74683	1,24745	1,15377
-0,682256	-1,19421	-0,662347	-0,0672457	0,0220776	-0,117171

Polito et al. Universal ready-to-use immunotherapeutic approach for the treatment of cancer: expanded and activated polyclonal γδ memory T cells.

Anova Photon

1,05096	1,05096	0,864969	-1,01561	-1,66624	-1,43698
-0,81601	-0,602163	-0,81601	1,8374	1,77672	0,80278
-0,729943	-1,24373	-0,409727	-0,531327	-1,05792	-1,2138
-0,982629	-0,982629	-0,982629	1,56103	1,50048	0,817943
1,30491	0,829438	0,806768	-0,443418	-1,28511	-0,571146
-0,56056	-0,766457	-1,20833	-0,891738	-1,02116	-0,849726
-0,945066	-1,24551	-1,14104	-1,00922	-0,597189	-0,340558
-0,723667	-1,09421	-1,09376	1,23131	0,919308	0,472594
-0,534218	-0,221063	-1,05543	0,0838323	-0,307048	-0,191677
-1,03007	-1,7692	-1,7692	0,786311	0,486941	0,372488
-0,793682	-0,895761	-1,81928	1,10709	0,9808	1,14118
1,08349	1,17631	0,668101	-1,055	-1,055	0,0530442
0,648092	0,388382	0,459119	-1,02427	-2,01586	-1,51226
-1,45883	-1,40936	-1,40863	0,564987	0,217422	0,11846
1,14937	0,681232	1,14937	-1,47663	-1,73128	-1,07522
-1,35364	-1,14209	-0,140315	0,060673	-0,565652	-0,275569
-0,568845	-1,12662	-0,221875	-0,592045	-0,612654	0,270423
-1,09323	-0,85463	-1,09323	0,905091	1,65099	1,00901
-0,0117169	-1,38205	-0,717603	-0,357555	0,00690218	0,182689
-0,89929	-0,89929	-0,89929	1,70385	1,52391	0,726033
-0,810579	-1,33523	-1,33523	1,02126	0,542441	1,07942
-0,983744	-0,983744	-0,983744	1,5572	1,49177	0,824782
-1,13106	-1,13106	-1,13106	1,50831	1,29833	0,986146
-0,846529	-0,489559	-0,927863	-0,212536	-0,46527	-0,184671
-1,09102	-1,01677	-0,687453	0,99596	0,931723	1,04858
-1,11182	-1,74263	-1,74263	0,789403	0,489425	0,366318
-1,4796	-1,4796	-1,4796	0,97827	0,486221	1,09725
0,897329	0,831173	1,51364	-0,394856	-1,07923	-0,71703
-1,36164	-1,30334	-1,32782	0,934883	0,942187	1,13311
-0,421293	-1,14983	-1,11335	-0,597719	-0,107723	-0,579382
-0,841753	-1,35665	-0,595598	-0,271372	-0,36836	0,112533
-1,12096	-1,12096	-1,12096	1,55112	1,38982	0,787314
-1,62885	-0,784482	-1,62885	0,904248	0,875241	1,18687
0,74236	1,02137	0,653745	-1,41684	-1,92807	-1,35254

Polito et al. Universal ready-to-use immunotherapeutic approach for the treatment of cancer: expanded and activated polyclonal γδ memory T cells.

Anova Photon

-0,949797	-0,949797	-0,949797	1,71151	1,44621	0,723502
-0,666741	-1,00178	-0,877145	0,525684	1,38836	0,946576
-1,01377	-0,163273	-1,01377	0,68723	1,53773	0,261978
0,272833	0,129709	0,451893	-0,687005	-1,0575	-1,22534
0,531538	0,661513	0,603354	-1,5133	-1,17474	-1,96081
-1,44356	-1,17007	-0,66021	1,89296	0,401167	1,63536
-1,31862	-0,966349	-0,472449	0,209589	-0,127885	-0,23573
-1,25876	-1,00968	-0,954467	1,08274	0,768043	0,809199
-1,2801	0,00169138	-0,788746	-0,986009	-0,337227	-0,0617678
-1,51013	-1,26297	-1,5079	0,592613	0,241954	-0,151179
0,946638	1,09749	1,22523	0,659492	0,0639248	0,328579
0,468125	0,364698	0,337661	-1,47825	-1,8517	-1,26264
1,22987	1,01024	0,764004	-0,597584	-1,4865	-0,964608
-1,04571	-1,04571	-1,04571	1,47446	1,25582	1,23467
1,11269	1,28662	1,01778	-0,46355	-1,61563	-1,13286
-1,17851	-1,17851	-1,48825	1,14327	0,909602	1,24075
0,965973	1,15362	0,856209	-0,911734	-1,51677	-1,68435
-0,924646	-1,76508	-1,76508	0,476595	0,0698762	1,1366
-0,770356	-0,917684	-0,972262	1,07397	0,409368	1,23596
-1,46305	-1,46305	-1,46305	0,92678	0,446816	1,22049
-1,06488	-0,581528	-0,0421397	1,46393	1,49986	1,65484
-1,12272	-0,96159	-0,778037	-0,130125	1,06787	1,05015
0,787962	1,28238	0,787962	-0,408265	-1,67692	-1,86819
-1,29791	-0,903314	-1,67287	1,19598	1,03983	0,608252
-1,50855	-1,41694	-1,56251	0,446404	0,553855	0,412623
-1,04519	-1,04519	-1,25858	1,30478	1,25988	1,18943
0,0809294	-0,877717	-0,877717	-0,877717	-0,877717	-0,558168
0,671609	0,235137	0,377323	-1,29095	-1,97747	-1,49142
-1,50404	-1,50404	-1,38051	1,01095	0,92743	0,805684
0,0639172	0,0388345	0,322525	1,35255	0,196816	1,25943
-1,7739	-1,15221	-1,26185	0,509464	0,468323	-0,232228
-0,778649	-1,19824	-0,830475	1,12504	0,590111	0,402104
1,46916	1,45521	1,50141	-0,296269	-0,17011	0,242857
-0,831192	-1,06519	-1,43606	0,653005	1,19198	1,43922

Polito et al. Universal ready-to-use immunotherapeutic approach for the treatment of cancer: expanded and activated polyclonal γδ memory T cells.

Anova Photon

-1,30852	-1,47168	-0,551711	-0,259913	0,0317184	0,547988
-0,861865	-0,987844	-1,41857	0,946174	1,05476	1,31506
-0,935563	-0,585641	-0,983414	0,854803	1,09648	-0,490013
1,23285	1,23285	0,799082	-0,680097	-1,45317	-1,67512
1,12371	0,776107	1,00133	-0,479874	-1,58794	-1,76842
1,62872	1,35277	1,60083	-0,457658	-1,06293	-0,301774
-0,957667	-1,78878	-1,78878	0,779737	0,486553	0,313686
1,14879	1,53688	1,22165	-0,0469562	-0,452271	-0,256659
0,674571	1,04596	1,04596	-2,09882	-1,5743	-0,656848
-0,989032	-0,989032	-0,989032	1,73901	1,09271	0,789373
0,885004	0,860538	0,749778	1,37753	0,885992	0,765454
-0,633971	-0,961687	-1,50264	0,699346	-0,287171	0,0851204
-1,08279	-0,960433	-1,17743	-0,62394	-0,864405	-0,681413
-1,11728	-0,439677	-0,74579	0,730447	-0,336812	-0,460157
-0,842422	-1,08726	-1,3976	0,811856	-0,202108	0,104654
-0,852209	-0,852107	-1,10943	0,991823	0,117634	1,04323
-0,949426	-1,10261	-1,36447	1,82593	0,981838	0,967142
-0,136317	-0,911534	-1,32015	-0,680629	-0,0970361	-0,436751
-0,427616	-0,622358	-1,71424	0,021669	-0,857724	-0,122256
-1,10212	-0,640824	-1,08764	1,03718	0,731813	0,732955
-1,29747	-1,12321	-1,81875	0,601819	0,31288	-0,139396
-0,835384	-0,304718	-1,06723	-0,666486	-0,373136	-0,690237
-1,26481	0,239851	-0,714533	0,376735	0,19859	-0,441958
-0,323735	-0,420506	-1,19511	0,145702	-0,19159	-0,598258
0,158811	0,169136	0,248395	-1,24841	-1,40743	-1,84884
-0,826539	-1,08443	-0,966502	1,29489	0,777765	0,779651
-0,87234	-1,08094	-1,41155	0,914334	0,955301	1,3484
-1,14276	-0,572667	0,000450586	1,29507	1,54522	1,84655
0,716353	0,716353	0,716353	-2,02249	-1,61658	-0,977874
-1,47779	-1,47779	-1,47779	0,98379	0,4997	1,09865
-0,751849	-1,44187	-0,388332	-0,106069	0,566091	-0,0938672
0,15688	0,171179	0,252289	-1,24698	-1,4131	-1,8464
0,159114	0,173431	0,254647	-1,24657	-1,40693	-1,84677
1,71031	1,13193	1,55871	-0,33195	-0,987759	-0,509035

Anova Photon

-1,62287	-0,786111	-0,685855	1,28204	0,748058	0,750005
0,0809294	-0,877717	-0,877717	-0,877717	-0,877717	-0,558168
1,15131	1,19493	1,10014	-0,740686	-0,766359	0,0731807
0,648344	1,04491	0,890019	-0,999687	0,33195	0,112261
1,21292	0,890483	1,22957	-0,828003	-0,995327	-1,19229
-0,665452	0,327475	-0,940061	-0,183429	-0,129781	-0,604428
1,49071	1,18019	1,42261	-0,465772	-0,649084	-0,631383
-0,998171	-1,37985	-0,621697	1,27889	0,756563	0,758467
-0,574725	-0,801273	-0,978977	0,0253334	-0,635275	-0,367325
-1,08067	-0,441468	-1,12655	1,31319	0,756859	0,758887
-0,965966	-1,11889	-1,38033	1,80657	0,917972	0,959071
-0,755852	-0,832844	-1,20906	-0,87286	-0,961257	-0,649859
1,29621	1,08787	1,1206	-1,81818	-1,18864	-0,502984
-1,26764	-0,824477	-1,20925	0,818002	0,904396	0,932953
-0,950427	-0,658268	-0,634358	-0,370761	-0,197316	0,111889
-0,87216	-1,0808	-1,41148	0,914886	0,955079	1,34905
0,164457	-0,795341	-0,795341	-0,795341	-0,795341	-0,475408
-1,4195	-1,35578	-1,81674	0,646013	0,682686	-0,438087
1,38135	1,05998	0,857218	-0,85526	-1,28502	-1,57371
-1,01253	-0,914141	-0,615955	-0,60887	-0,560752	-0,212736
-0,983221	-0,983221	-0,983221	1,55939	1,49612	0,823811
0,955958	0,656004	0,619546	-0,602029	-2,09555	-1,42872
-1,11262	-1,63176	-1,63176	1,17026	0,967851	0,706768
-0,90564	-0,90564	-0,90564	1,75893	1,18096	0,72852
1,05754	0,781721	0,592733	-0,712432	-1,50122	-1,95334
-0,478482	-0,891439	-0,993163	1,23112	0,979009	0,213698
0,466965	1,16572	0,701782	-1,37658	-1,51728	-1,12588
0,157403	0,169854	0,249707	-1,25367	-1,41018	-1,84115
-1,29269	-0,961175	-1,29269	1,51713	0,93771	1,38311
-0,302589	-0,486519	-0,420932	-0,680727	-0,397826	-0,78957
0,359648	0,731936	0,731936	-2,43245	-1,39027	-0,685495
-0,668802	-0,229337	-1,00198	1,29168	1,92289	1,27754
-0,783105	-1,21806	-1,21806	0,643207	1,21117	1,47871
0,697156	0,442188	0,510034	-1,04589	-2,15656	-1,33521

Polito et al. Universal ready-to-use immunotherapeutic approach for the treatment of cancer: expanded and activated polyclonal γδ memory T cells.

Anova Photon

-1,09635	-1,14025	-1,23043	-0,704348	-0,499518	-0,653865
-1,40123	-1,82771	-0,863206	1,11529	0,751767	0,959666
1,0074	0,960599	1,06469	-0,548742	-1,5922	-1,84399
-0,487137	-1,04654	-0,865262	-0,146998	-0,997042	-0,229698
0,695384	0,343058	0,695384	-2,07537	-1,70754	-0,713918
-0,950649	-0,950649	-1,19474	1,97014	0,985208	0,795809
-1,03313	-1,29174	-0,934882	0,498998	-0,342427	-0,361866
-0,557254	-0,600993	-1,24487	1,11978	1,81845	1,50436
-1,03406	-1,03285	-0,977222	0,548545	0,817984	0,57949
-0,6475	-0,850765	-0,758693	-0,201293	-0,765829	0,177075
-0,678671	-1,03514	-1,33705	0,238666	-0,111767	-0,407259
-1,08996	-1,08996	-1,08996	1,46141	1,28567	1,16089
-0,273798	-0,378223	-1,15252	-0,429888	-0,568607	-0,31956
-0,355092	0,426053	-1,10102	-0,932181	-0,545035	-0,148008
0,160728	0,17304	0,25435	-1,24859	-1,40913	-1,84948
-1,01124	-0,495543	-1,01124	0,68375	1,28078	0,657665
-1,62257	-0,966178	-1,62257	0,678483	1,18375	0,968474
-0,736576	-1,10031	-0,853843	1,1128	0,531697	1,27048
-0,946761	-0,572602	-0,770919	-0,2423	-0,985624	-0,10692
-0,832354	-1,07695	-1,07695	2,00732	1,1125	0,763285
-0,682404	-0,594714	-1,48687	-0,234853	-0,0153425	0,00215982
-0,983582	-1,23225	-0,737012	1,06863	0,380453	1,24128
-1,38177	-1,50884	-0,621677	-0,240786	0,595918	-0,133244
1,7875	1,24875	1,64236	-0,487729	-0,496188	-1,03971
0,153564	0,166027	0,245948	-1,2566	-1,41243	-1,83253
-0,854363	-0,854363	-0,854363	1,9138	1,52643	0,880949
-0,454725	-0,836659	-0,843349	0,54934	0,311231	0,725276
-0,95915	-0,95915	-0,95915	1,68517	1,40095	0,769975
-0,747979	-1,00238	-1,40559	1,65348	0,85955	1,40061
1,38556	1,18862	1,03586	-1,12829	-1,44349	-0,994125
-1,57047	-0,385165	-2,32635	0,0183634	-0,0150103	0,163705
-0,828096	-1,06348	-1,43656	0,658586	1,19586	1,43125
-0,962599	-1,7906	-1,7906	0,777301	0,489701	0,36879
-1,023	-1,33796	-1,07889	0,536623	0,454881	1,23177

Anova Photon

1,12808	0,984689	1,32815	-0,809598	-0,976731	-0,987658
0,901748	0,573183	0,856569	-2,102	-0,896318	-0,893105
-1,23749	-0,945893	-1,15583	1,08946	0,663072	0,765712
-1,19048	-1,19048	-1,19048	1,92176	1,30558	0,572237
-1,12622	-1,12622	-1,12622	1,24293	1,38673	1,20098
-0,937456	-0,941059	-1,04894	0,900646	0,186401	1,08753
-0,677662	-1,0331	-1,25736	1,81992	1,42219	0,612912
-0,596532	-1,48343	-1,48343	1,26773	1,14984	1,07702
-0,485275	-1,1412	-1,42132	-0,0203051	0,740519	1,32406
-0,924188	-1,76367	-1,76367	0,467519	0,0597385	1,13181
0,688109	0,336779	0,414658	-1,13841	-2,02977	-1,61944
-0,760922	-0,626941	-0,854851	0,104795	-0,998768	-0,327136
-1,32203	-0,793473	-0,896894	-0,840091	-0,677184	-0,772287
-1,17589	-1,17589	-1,48654	1,17456	0,922564	1,2043
-0,404181	-1,03382	-1,13195	1,09636	0,959131	1,00697
0,0236394	-0,117148	-0,00811719	-0,952898	-2,35351	-1,00722
1,3719	1,05245	0,903912	-0,854429	-1,27856	-1,57272
-1,47778	-1,03922	-0,0806783	-0,371948	-0,687786	-0,454725
-1,0366	-0,0781113	-1,0366	0,650701	1,45852	0,334688
0,47328	0,476204	0,0266589	0,120229	-0,713643	-0,202339
-0,96027	-0,78601	-0,774499	0,459525	0,714353	1,22691
-1,08525	-1,08525	-1,31416	0,443774	0,720591	1,60034
1,04472	1,04472	1,04472	-1,70935	-1,6485	-0,503093
-0,856598	-0,875142	-0,4622	-0,462324	-0,54243	-0,362754
-1,39729	-1,82165	-0,861934	1,1225	0,751706	0,959283
1,21115	0,889272	1,22784	-0,82711	-1,00751	-1,17516
0,00280966	-0,933991	-1,38976	-0,0872321	-0,1737	-0,71824
-0,814179	-0,972986	-1,19609	1,08671	0,898092	1,01968
0,665095	0,789275	0,587391	-1,70786	-1,93224	-1,21874
0,157218	0,169674	0,249557	-1,25439	-1,40802	-1,84208
-1,52793	-1,13358	-1,7478	0,646367	0,210453	0,250523
-0,866078	-1,11202	-1,11202	1,99372	1,03707	0,771477
-0,934254	-1,02093	-0,704869	1,18132	0,677007	1,29031
-0,731696	-1,01446	-1,46263	1,82918	0,95303	1,06098

Anova Photon

0,991227	0,557792	0,42128	-0,841582	-1,87744	-1,51991
-0,923577	-1,76393	-1,76393	0,465558	0,0613778	1,13651
-0,612364	-0,964913	-0,775982	-0,154426	-0,847149	0,182399
-0,678032	-1,09114	-1,40852	1,15174	1,3115	0,626958
-1,09662	-1,00427	-0,853274	0,955484	0,762059	0,366394
-0,423806	-1,03343	-0,0662478	-0,495383	-1,03903	-0,0505353
-1,08372	-1,08372	-1,08372	0,866757	1,48784	1,40399
-0,968986	-1,21797	-1,32185	0,062712	0,296498	0,062712
-0,982629	-0,982629	-0,982629	1,56103	1,50048	0,817943
1,09294	1,21493	0,817253	-0,699544	-1,34765	-1,22167
-0,879962	-1,12216	-1,12216	2,00442	1,04582	0,759275
-0,404994	-1,13492	-1,6818	0,975167	1,2659	0,574479
-1,10324	-0,811807	-0,601082	0,997222	0,120607	1,04904
-0,730438	-0,730438	-0,730438	1,84586	1,72368	1,06191
-0,908058	-0,908058	-0,908058	1,95725	1,17604	0,960655
-1,04033	-1,45517	-1,35967	0,361686	0,677554	0,770965
1,36833	0,82326	1,05374	-0,857356	-0,759979	-1,876
-0,838524	-0,798324	-1,09199	-0,534238	0,0090751	-0,304883
-0,846477	-1,09224	-1,09224	2,01123	1,10911	0,754859
-1,0043	-0,693699	-0,47858	0,00435969	-0,488875	-0,166654
-1,12742	-1,12742	-1,58431	1,76752	0,916622	0,936476
-1,10319	-1,62688	-1,62688	1,21344	0,983692	0,670896
-1,07224	-1,07224	-1,07224	1,67082	1,08755	0,97666
-0,645323	-1,04573	-0,552576	0,154658	0,836307	-0,863959
-1,1348	-1,24845	-0,911205	-0,310193	-0,0857222	0,00634141
-1,50535	-0,240194	-0,70799	-0,528228	0,174404	-0,451186
-0,972805	-0,972805	-0,972805	1,53874	1,1428	1,46334
1,61742	0,895708	0,740264	-0,498287	-1,19149	-1,05075
0,0809294	-0,877717	-0,877717	-0,877717	-0,877717	-0,558168
-0,682731	0,249793	-0,93564	-0,490921	-0,364671	-0,742975
-1,31021	-1,06586	-1,17669	-0,818834	-0,704604	-0,118059
-0,736987	-0,930895	-1,10116	0,937444	1,00885	1,09424
-1,18879	-1,49559	-0,505717	1,56951	0,429592	0,905527
-0,975328	-0,607835	-0,975328	1,05454	1,49713	0,878451

Polito et al. Universal ready-to-use immunotherapeutic approach for the treatment of cancer: expanded and activated polyclonal γδ memory T cells.

Anova Photon

0,987621	0,822619	1,1293	0,262275	-1,15221	-0,957424
-0,888915	-0,849409	-1,14185	-0,776234	-0,892285	-0,809812
1,2137	0,894006	1,22474	-0,833573	-0,999093	-1,19157
1,58643	1,43294	1,62831	-0,375035	-0,861609	-0,350775
-0,503925	-1,07123	-1,1343	-0,320025	1,07158	1,03694
-0,420928	-1,10403	0,37031	-0,212772	0,011888	-0,626191
1,60779	1,6388	1,29329	-0,871431	-0,231563	-0,871088
-1,42459	-0,863267	-1,42459	1,04092	0,259379	1,32976
-0,964474	-0,964474	-0,964474	1,11894	1,62801	0,895228
-0,872159	-1,08104	-1,41211	0,913562	0,955645	1,34875
-0,959217	-1,27032	-0,694954	1,05962	0,430014	1,21938
1,31297	0,994588	0,84655	-0,901615	-1,30337	-1,54019
1,01901	1,01901	1,01901	-1,77975	-1,79044	-0,68381
-1,09186	-1,10117	-1,0115	-0,41176	-0,895818	-0,829838
0,0809294	-0,877717	-0,877717	-0,877717	-0,877717	-0,558168
1,28452	1,3906	1,5353	-0,592313	-1,02174	-1,00642
-0,956611	-1,10938	-1,37055	1,82019	0,970245	0,960298
1,21141	0,888782	1,23385	-0,831593	-0,99398	-1,18045
-0,425719	-0,470853	-0,646938	-0,111883	-0,160977	-0,311651
-1,49923	-0,99166	-1,71186	0,258394	-0,0977695	0,996777
1,746	1,67929	1,15821	-0,500981	-0,846727	-0,543634
-0,716359	-0,407699	-1,41062	1,27189	1,10682	1,29104
0,15763	0,161631	0,247166	-1,24823	-1,43011	-1,82343
0,0809294	-0,877717	-0,877717	-0,877717	-0,877717	-0,558168
-0,478596	-1,33538	-1,01363	1,29872	1,91108	1,28871
-1,54486	-0,737613	-0,28058	-0,336364	-0,784166	-0,0395843
-0,843615	-0,843615	-0,843615	2,01304	1,31062	0,651955
0,156004	0,17027	0,251195	-1,24676	-1,41339	-1,84268
-1,16452	-1,16452	-1,16452	1,10043	1,26466	1,08293
-0,824057	-0,824057	-0,824057	2,3733	1,1069	1,02159
-1,07642	-1,07642	-1,07642	1,71377	1,3788	0,719711
-1,99613	-1,01698	-1,64005	0,00891239	-0,0891271	0,804195
1,38356	1,24654	1,65547	-0,136589	-0,286097	-0,963117
-0,830296	-0,830296	-0,830296	1,99044	1,26724	0,85627

Anova Photon

-0,899702	-0,902044	0,0387375	-0,387431	-0,548409	-0,14661
-0,268346	-0,698452	-1,19661	0,777827	0,806136	1,85789
-1,27464	-1,27464	-1,27464	0,76532	0,955449	1,17553
-0,832226	-1,06597	-1,43643	0,65103	1,19116	1,43908
-1,00053	-1,00053	-1,37441	0,649838	1,24369	1,34762
-0,854363	-0,616332	-0,854363	1,94053	1,85098	0,57382
-0,750367	-1,02496	-1,23268	1,07408	1,19625	1,39614
-0,897619	-0,598861	-1,37114	1,29787	0,749454	2,01651
-0,830695	-0,830695	-0,830695	1,98882	1,26672	0,855145
0,259211	0,153402	0,302372	-1,30698	-1,31718	-1,83117
-1,16957	-0,596043	-1,04711	-0,600012	-1,24793	-0,694773
-0,830306	-0,830306	-0,830306	1,99068	1,26664	0,856412
-1,12254	-0,907287	-1,12254	1,57652	1,74075	0,788124
-0,415202	-0,400873	-1,16047	0,153232	-0,579325	-0,691332
-0,829883	-0,829883	-0,829883	1,99207	1,26778	0,857415
0,780927	-0,0228096	0,0504406	-0,745824	-2,15851	-1,09889
0,760649	0,341878	0,62718	-0,554683	-1,31829	-1,87588
-0,383354	-0,34752	-0,307161	1,89005	1,82693	0,942081
-1,29845	-1,53066	-1,48964	0,562611	0,228443	0,135859
-1,09274	-1,09274	-1,09274	1,61093	0,909615	0,868854
-0,779126	-0,96008	-0,552654	1,32153	2,04783	1,29908
0,576623	0,735907	0,699575	0,767715	0,595283	-0,143949
-0,988368	-0,646423	-0,988368	1,73599	1,78496	0,841959
-0,83946	-0,77849	-0,460323	0,257971	0,413403	0,0557823
-0,670521	-1,23818	-0,993006	1,54383	1,16747	0,706891
1,41782	0,841157	1,08679	-1,01263	-0,930651	-1,49974
-0,961767	-1,1147	-1,37614	1,81436	0,920498	0,959467
0,951407	0,681179	0,874126	-1,25164	-0,375071	-0,958719
1,53835	1,62768	1,55892	-0,915656	-0,58104	-0,199291
-0,297283	-0,303377	-1,26609	1,06301	1,169	1,69775
-0,983612	-0,618953	-0,983612	1,88215	1,79708	0,683947
-0,678429	-0,603098	-0,67608	-0,534731	0,471699	0,615981
0,36549	0,469287	0,03264	0,988188	0,0754618	0,960878
0,856835	0,703649	0,871046	0,13554	-0,260496	-1,49132

Polito et al. Universal ready-to-use immunotherapeutic approach for the treatment of cancer: expanded and activated polyclonal γδ memory T cells.

Anova Photon

1,16693	1,64188	1,66892	-0,479846	-0,777468	-0,0251715
-0,71131	-1,06724	-0,812103	-0,697758	-0,120953	-0,152013
-1,19054	-0,990234	-1,19054	1,11071	0,567108	1,33345
-0,901194	-1,38403	-2,04571	0,720755	0,0576212	0,435538
1,00978	1,00978	1,00978	-0,917282	-1,41637	-1,77472
-1,14535	-1,08762	-1,16836	-0,352451	-0,837487	-0,50601
-1,30724	-0,844402	-0,405881	0,435258	-0,60563	-0,138987
-1,00437	-1,39366	-1,11329	1,0583	0,767451	0,523714
1,51705	0,829714	1,12493	-0,789616	-0,826778	-1,12563
-1,71903	-1,14796	-1,492	0,803133	0,0301196	0,982835
-0,832784	-0,832784	-0,832784	2,00496	1,29048	0,752015
-0,699806	-0,699806	-0,699806	1,84594	1,74204	1,04101
-0,632843	-1,07527	-1,55252	0,0469452	0,0459528	-0,333016
-0,696026	-0,696026	-0,696026	1,83662	1,73514	1,09321
-0,872278	-1,08133	-1,41267	0,914209	0,957784	1,34473
-0,487923	-0,302152	-0,662916	0,173028	-0,466603	-0,816344
0,762378	0,886555	1,11857	-2,27648	-1,30935	-0,49167
-0,968977	-0,968977	-0,968977	1,09965	1,60706	0,878506
1,41262	1,38615	1,45066	0,0327732	-0,195086	0,161652
-1,05012	-0,856843	-0,736657	1,4968	1,10906	0,689291
1,04027	0,39961	-0,00619661	1,16887	0,468302	1,04302
1,31523	1,74163	1,08599	-0,459797	-0,993458	-0,083446
-1,4418	-1,16849	-1,73781	0,526426	0,341126	0,198197
-0,590258	-0,603878	-0,578512	0,109579	-0,665248	-0,962269
0,390314	0,146024	0,345495	-1,03818	-2,4895	-0,96426
-0,875185	-0,875185	-0,875185	1,5913	1,59482	0,865114
-0,164401	-0,794158	-0,511473	-0,645155	-0,414724	-0,698102
-0,529853	-0,861206	-1,11187	1,84778	1,07757	0,985229
0,786205	0,737388	0,524	0,557027	0,401719	0,638442
0,767922	0,670877	0,715414	-0,618423	-0,841055	-1,4062
-1,16841	-1,38274	-0,271631	1,31549	0,777741	1,319
-0,549349	-0,634034	-1,55602	-0,549349	-0,511025	0,00836013
-0,98067	-0,98067	-0,98067	1,05564	1,5543	0,836069
-0,505748	-0,924813	-0,924813	1,32035	1,66913	0,822657

Anova Photon

-0,660706	-0,0775303	-1,10186	0,701344	1,4933	0,136618
-0,962867	-0,962867	-0,962867	1,12432	1,63544	0,900585
-1,40041	-1,82592	-0,863611	1,00222	0,819852	0,967559
1,64893	1,38362	1,62211	-0,00746628	-0,12092	-0,794042
-1,04558	-1,04558	-1,04558	1,47592	1,25654	1,23407
-0,797229	-1,03973	-0,846422	0,99085	0,660193	0,779576
-0,607034	-0,993809	-1,46355	1,2517	1,08657	0,633095
-0,77646	-0,570902	-1,6815	1,66428	0,66135	0,598441
-0,878306	-1,03986	-1,06889	1,35585	0,909452	0,85745
-0,825349	-1,03977	-1,45469	1,3185	1,19468	1,12022
-1,04489	-1,71018	-1,71018	0,865001	0,278148	0,728433
-1,15669	-0,905421	-1,15669	0,74155	1,69912	0,930945
-0,529539	-0,898136	-1,21359	1,07747	1,69703	1,86883
1,11363	0,930695	0,890013	-0,774995	-1,14495	-1,95259
-0,368605	-0,388505	-0,371399	-0,463495	-0,686126	-1,049
-1,03387	-1,69825	-1,69825	1,11861	0,959237	0,550046
1,9009	1,24671	1,34575	0,104042	-1,19846	-0,581956
-1,02673	-0,846325	-1,13148	0,527003	0,170628	-0,0108451
-0,733799	-0,733799	-0,733799	1,84875	1,73401	1,01131
-0,615833	-1,07907	-1,62392	1,67033	0,765644	1,41366
0,971148	1,25714	0,82445	-1,17873	-1,70326	-1,33664
1,03181	1,03181	0,83741	-1,01757	-1,69842	-0,994667
-0,925858	-1,76481	-1,76481	0,472908	0,07618	1,13463
1,29072	0,806813	0,778073	-0,587255	-0,922632	-1,75853
0,215802	0,228541	0,310092	-1,24865	-1,43214	-1,87077
-0,902273	-1,08442	-1,47617	1,56631	1,23705	1,29214
-0,815073	-1,00427	-0,78509	1,29761	1,94263	1,29284
-1,44554	-0,637282	-1,34064	-0,467293	-0,65815	-0,852313
-0,923865	-1,00007	-0,943238	0,446686	0,561954	-0,0595526
-1,22262	-1,13096	-1,03051	0,426749	0,485045	1,30802
1,3116	0,992178	0,843656	-0,907818	-1,30612	-1,53489
-0,499631	-1,36328	-0,543912	0,265881	-0,698787	-0,31022
1,37136	1,05178	0,903182	-0,850985	-1,28017	-1,57444
-0,383199	-1,46811	-1,13946	1,29127	0,735339	0,737366

Polito et al. Universal ready-to-use immunotherapeutic approach for the treatment of cancer: expanded and activated polyclonal γδ memory T cells.

Anova Photon

-0,947115	-0,947115	-0,947115	1,48596	1,46308	0,716343
-1,50951	-0,739733	-0,986177	0,974096	0,988316	1,69548
-0,845498	-1,12219	-0,982419	0,886429	1,04026	0,500537
0,638623	0,798888	0,727066	-0,728586	-1,54161	-2,11707
-0,766467	-1,15761	-1,15761	1,61496	1,21735	1,02178
0,247137	0,148018	0,287402	-1,29941	-1,30531	-1,833
-0,959587	-0,856591	-1,37953	-0,782608	-0,843814	-0,27335
-0,823933	-1,19782	-0,107764	-0,446074	-0,453859	-0,058303
-0,897269	-0,391362	-0,889977	-0,124702	-0,198109	-0,15689
-0,473442	-0,0817197	-0,325172	1,32857	1,64032	1,5931
-1,26939	-0,493394	-1,52056	1,67297	1,60153	0,803772
-0,62234	-1,10232	-1,19636	1,25625	0,893279	0,869989
-1,12357	-1,12357	-1,12357	1,51708	1,31024	0,993005
-0,670609	-0,966144	-1,54405	0,934397	1,04844	1,29798
0,812986	0,504969	0,812986	-1,88469	-1,48716	-1,09358
-0,682547	-0,795941	-1,20769	-0,397805	1,07728	1,04056
-1,03213	-1,19699	-1,47882	1,77818	1,00789	0,938573
-0,630168	-0,176665	-1,08367	0,594488	1,46261	0,0886165
0,0809294	-0,877717	-0,877717	-0,877717	-0,877717	-0,558168
-1,4985	-0,104537	-0,63622	0,162693	-0,12236	-0,390069
-0,503399	-0,565799	-0,93196	0,112772	-0,866378	-0,493
-0,45764	-0,713047	-0,961177	-0,757747	0,0615154	-0,714285
-1,16704	-0,681382	-1,34072	-0,475439	-0,596961	-1,00425
-1,16465	-0,741113	-1,77011	-0,247009	-0,401079	-0,7791
1,29726	0,052215	1,29726	-0,552077	-1,15241	-1,53003
-1,00472	-0,959568	-0,964969	1,61805	1,41016	1,05821
1,23978	0,814133	1,18677	-0,668932	-0,597767	-0,403482
-1,0478	-1,0478	-1,0478	1,64411	1,35996	0,745556
-1,20861	-1,20861	-1,20861	0,527095	0,876097	1,30123
-0,802639	-0,866348	-0,897404	0,00532765	0,040075	-0,00405198
-1,06577	-1,13884	-1,21857	-0,704918	-0,584598	-0,517964
-0,91015	-0,96721	-1,4709	0,93467	0,980086	1,33433
-1,19585	-1,19585	-1,19585	0,626043	0,887771	1,17036
0,0809294	-0,877717	-0,877717	-0,877717	-0,877717	-0,558168

Anova Photon

-0,872361	-1,08092	-1,41148	0,914673	0,954948	1,34798
-0,783042	-1,40645	-1,6373	1,02757	0,914087	1,12504
0,154498	0,166807	0,248135	-1,24842	-1,4074	-1,84873
0,154519	0,166949	0,246662	-1,25409	-1,40741	-1,84054
1,53294	1,2907	1,32824	-1,79121	-1,07313	-0,416956
1,38743	1,07157	0,924705	-0,809012	-1,24384	-1,58819
-1,40003	-1,82543	-0,863356	1,00769	0,814817	0,967375
0,156512	0,168839	0,250248	-1,2524	-1,4123	-1,84153
-0,164226	-0,0578258	-0,167322	1,43485	0,743914	0,184091
-0,988894	-0,297207	-0,988894	0,716097	1,49881	0,236253
0,522184	0,446037	0,918599	1,32773	1,18072	0,989921
0,157218	0,169674	0,249557	-1,25439	-1,40802	-1,84208
-0,804332	-0,9718	-1,2595	1,32538	0,765185	0,767228
0,161631	0,17409	0,253994	-1,25034	-1,40989	-1,84543
-1,40006	-1,82542	-0,863438	1,00457	0,819448	0,967105
1,39262	1,20119	1,2401	0,529258	-0,526905	0,0883046
-1,49351	-1,47201	-1,29252	0,608246	0,308244	-0,150982
-0,523026	-1,72925	-1,18071	0,0489912	0,173294	0,536158
-0,920366	-1,26842	-0,831841	1,23524	0,817014	0,526358
0,196663	0,0519335	0,30901	-1,37068	-2,01594	-1,04804
-0,935846	-1,08522	-1,34057	1,81407	0,897547	0,926838
-0,661267	-1,16226	-1,27073	0,83493	0,715991	1,3963
-1,0298	-0,845622	-1,0735	-1,00534	-0,634462	-0,771016
0,933964	0,915291	1,65146	-1,19345	-1,5414	-0,824312
1,37165	1,05209	0,903497	-0,854206	-1,27971	-1,57396
-0,636604	-1,14353	-1,10813	1,235	0,86628	1,01997
-0,589816	-0,548071	-0,539069	0,208523	-0,944113	-0,611079
0,805542	0,604452	0,819535	-0,509365	-1,09021	0,693969
1,73723	1,73723	1,11507	-0,218045	-0,770308	-0,987296
-1,01216	-1,01216	-1,01216	1,27337	0,957527	0,6722
-1,06691	-1,54372	-1,54372	1,02516	0,705244	0,42813
1,53491	1,59667	1,42966	-1,02837	-0,604576	-0,353568
0,811697	0,811697	0,811697	-1,47319	-1,28919	-1,38721
0,51064	1,3295	0,516007	-1,96734	-1,44034	-1,26498

Anova Photon

1,56275	1,53633	1,57079	-0,326342	-0,460296	-0,45144
0,405891	0,436978	0,461675	0,859944	0,443205	0,563273
-0,781927	-0,781927	-0,781927	1,40069	1,53801	1,05985
-0,539051	-0,622864	-0,639552	0,236186	-0,182534	-0,882392
-0,680785	-1,1004	-1,3297	1,25945	0,772428	0,785888
-0,631008	-1,00947	-0,823385	-0,0892079	-0,482088	-0,147172
0,786429	0,626111	0,468331	-1,22732	-1,74511	-1,80746
1,42972	0,830619	1,08878	-0,990767	-0,928491	-1,46153
-1,12752	-0,791081	-1,12752	1,63467	1,75381	0,772556
-0,730512	-0,730512	-0,730512	1,8452	1,7233	1,06332
-0,851571	-0,649092	-0,851571	1,6278	1,56432	1,36936
-0,95055	-0,383992	-0,436916	-0,0804397	0,599543	-0,538255
-0,223333	-0,916461	-0,619893	0,366912	0,339836	0,246219
-1,05313	0,338049	-0,607411	-0,309022	0,17879	0,106578
1,59291	1,64671	1,54733	-0,359908	-0,385014	-0,175904
-1,34393	-1,05535	-0,704261	0,140702	-0,587496	-0,0908446
-0,876167	-1,00993	-0,876151	1,11543	1,00367	0,242243
-0,572647	-0,721702	-1,11342	0,261234	-0,59722	-0,218404
0,607959	0,519284	-0,138081	-1,42407	-1,45829	-1,7276
-0,795025	-1,20608	-1,08628	1,29801	1,52266	1,50382
-0,961614	-0,321739	-0,530523	-0,994461	-0,0880399	-0,11866
-1,01486	-0,999714	-0,512405	1,20259	0,637898	0,843543
1,5076	1,12527	1,21397	-0,66472	-0,357614	-0,181872

Polito et al. Universal ready-to-use immunotherapeutic approach for the treatment of cancer: expanded and activated polyclonal γδ memory T cells.
Anova Photon

C: KEGG name
Bile secretion;Neuroactive ligand-receptor interaction;Pancreatic secretion
Adherens junction
Alzheimer's disease;Valine, leucine and isoleucine degradation
Neuroactive ligand-receptor interaction;Pancreatic secretion
Cell adhesion molecules (CAMs);Intestinal immune network for IgA production
Meiosis - yeast
ing pathway;Epithelial cell signaling in Helicobacter pylori infection;Hepatitis C;Leishmaniasis;Measles;Neurotrophin signaling pathway;NOD-like receptor signaling pathway;Osteoclast differen
Bladder cancer;GnRH signaling pathway;Leukocyte transendothelial migration;Pathways in cancer
Chemokine signaling pathway;Cytokine-cytokine receptor interaction
Chemokine signaling pathway;Cytokine-cytokine receptor interaction
Sphingolipid metabolism
GnRH signaling pathway;Neuroactive ligand-receptor interaction
Cysteine and methionine metabolism;Glycolysis / Gluconeogenesis;Propanoate metabolism;Pyruvate metabolism
;Cell adhesion molecules (CAMs);Graft-versus-host disease;Intestinal immune network for IgA production;ko05152;Leishmaniasis;Phagosome;Rheumatoid arthritis;Staphylococcus aureus infe
SNARE interactions in vesicular transport
Colorectal cancer;Endometrial cancer;Mismatch repair;Pathways in cancer
Complement and coagulation cascades
Collecting duct acid secretion;Epithelial cell signaling in Helicobacter pylori infection;Oxidative phosphorylation;Phagosome;Rheumatoid arthritis;Vibrio cholerae infection
Cell cycle;Cell cycle - yeast;Meiosis - yeast
Neuroactive ligand-receptor interaction
Maturity onset diabetes of the young
Acute myeloid leukemia;Cytokine-cytokine receptor interaction;Hematopoietic cell lineage;Pathways in cancer
Neuroactive ligand-receptor interaction

Polito et al. Universal ready-to-use immunotherapeutic approach for the treatment of cancer: expanded and activated polyclonal γδ memory T cells.

Anova Photon

Ribosome biogenesis in eukaryotes
Osteoclast differentiation
Cell cycle - yeast
MAPK signaling pathway;Osteoclast differentiation
Carbon fixation pathways in prokaryotes;Citrate cycle (TCA cycle)
Vascular smooth muscle contraction
GnRH signaling pathway;Neuroactive ligand-receptor interaction
Vascular smooth muscle contraction
Systemic lupus erythematosus
Autoimmune thyroid disease;Neuroactive ligand-receptor interaction
Neuroactive ligand-receptor interaction
Apoptosis;Cytosolic DNA-sensing pathway;Hepatitis C;RIG-I-like receptor signaling pathway;Toll-like receptor signaling pathway
Wnt signaling pathway

Polito et al. Universal ready-to-use immunotherapeutic approach for the treatment of cancer: expanded and activated polyclonal γδ memory T cells.
Anova Photon

mTOR signaling pathway
Cell cycle;Cell cycle - yeast;Meiosis - yeast;Oocyte meiosis;Progesterone-mediated oocyte maturation;Ubiquitin mediated proteolysis
Neuroactive ligand-receptor interaction
Cell cycle;Oocyte meiosis;Progesterone-mediated oocyte maturation
Neuroactive ligand-receptor interaction
Lysine degradation
Base excision repair
Cytokine-cytokine receptor interaction;Hematopoietic cell lineage;Jak-STAT signaling pathway
SNARE interactions in vesicular transport
Adherens junction;Cell adhesion molecules (CAMs)
Ribosome biogenesis in eukaryotes
ithelial cells;Chemokine signaling pathway;Chronic myeloid leukemia;ErbB signaling pathway;Focal adhesion;Glioma;Insulin signaling pathway;Natural killer cell mediated cytotoxicity;Neurotro
Cysteine and methionine metabolism
Cell adhesion molecules (CAMs);Hepatitis C;Leukocyte transendothelial migration;Tight junction
Amoebiasis;Endocytosis;ko05152;Phagosome
Glycerolipid metabolism;Glycerophospholipid metabolism
Cell cycle
Purine metabolism

Polito et al. Universal ready-to-use immunotherapeutic approach for the treatment of cancer: expanded and activated polyclonal γδ memory T cells.
Anova Photon

Wnt signaling pathway
Ribosome biogenesis in eukaryotes
rens junction;Bladder cancer;Calcium signaling pathway;Endometrial cancer;ErbB signaling pathway;Focal adhesion;Non-small cell lung cancer;Pancreatic cancer;Pathways in cancer;Prostate c
Valine, leucine and isoleucine degradation
Cysteine and methionine metabolism
Ubiquitin mediated proteolysis
pithelial cells;Chronic myeloid leukemia;Endocytosis;ErbB signaling pathway;Insulin signaling pathway;Jak-STAT signaling pathway;Pathways in cancer;T cell receptor signaling pathway;Ubiquiti
Cytokine-cytokine receptor interaction;Jak-STAT signaling pathway
Neuroactive ligand-receptor interaction
Neuroactive ligand-receptor interaction
Cell cycle;p53 signaling pathway;Progesterone-mediated oocyte maturation
Long-term depression;Neuroactive ligand-receptor interaction
Vascular smooth muscle contraction
Cytokine-cytokine receptor interaction;Jak-STAT signaling pathway
RNA degradation
Cysteine and methionine metabolism;Glycolysis / Gluconeogenesis;Propanoate metabolism;Pyruvate metabolism
Neuroactive ligand-receptor interaction
Carbon fixation in photosynthetic organisms;Pentose phosphate pathway

Polito et al. Universal ready-to-use immunotherapeutic approach for the treatment of cancer: expanded and activated polyclonal γδ memory T cells.

Anova Photon

Chemokine signaling pathway;Regulation of actin cytoskeleton
Axon guidance
Calcium signaling pathway;Neuroactive ligand-receptor interaction
American trypanosomiasis);Cytokine-cytokine receptor interaction;Jak-STAT signaling pathway;ko05152;Leishmaniasis;Measles;Natural killer cell mediated cytotoxicity;Osteoclast differentiatio
Neuroactive ligand-receptor interaction;Vascular smooth muscle contraction
Osteoclast differentiation
alpha-Linolenic acid metabolism;Biosynthesis of unsaturated fatty acids;Fatty acid metabolism;Peroxisome;PPAR signaling pathway
Basal cell carcinoma;Hedgehog signaling pathway;Melanogenesis;Pathways in cancer;Wnt signaling pathway
Cell adhesion molecules (CAMs);Hepatitis C;Leukocyte transendothelial migration;Tight junction
RNA transport
Systemic lupus erythematosus
Insulin signaling pathway;mTOR signaling pathway
Basal transcription factors
Neuroactive ligand-receptor interaction
Pyruvate metabolism
Aldosterone-regulated sodium reabsorption;Taste transduction

Polito et al. Universal ready-to-use immunotherapeutic approach for the treatment of cancer: expanded and activated polyclonal γδ memory T cells.

Anova Photon

Phagosome
Cysteine and methionine metabolism;Glycolysis / Gluconeogenesis;Propanoate metabolism;Pyruvate metabolism
Neuroactive ligand-receptor interaction
Cysteine and methionine metabolism
Neuroactive ligand-receptor interaction
Systemic lupus erythematosus
Neuroactive ligand-receptor interaction
Focal adhesion
Protein processing in endoplasmic reticulum;Ubiquitin mediated proteolysis
Amoebiasis;Hematopoietic cell lineage;ko05152;MAPK signaling pathway;Pathogenic Escherichia coli infection;Phagosome;Regulation of actin cytoskeleton;Toll-like receptor signaling pathway
Jak-STAT signaling pathway
Calcium signaling pathway;Neuroactive ligand-receptor interaction
Cytokine-cytokine receptor interaction;Hematopoietic cell lineage;Jak-STAT signaling pathway;Primary immunodeficiency
Measles
Pentose phosphate pathway
Axon guidance
Gastric acid secretion

Polito et al. Universal ready-to-use immunotherapeutic approach for the treatment of cancer: expanded and activated polyclonal γδ memory T cells.

Anova Photon

Axon guidance
Acute myeloid leukemia;Pathways in cancer;PPAR signaling pathway;Wnt signaling pathway
Cell cycle - yeast;Pathways in cancer;Small cell lung cancer
Huntington's disease
Endocrine and other factor-regulated calcium reabsorption
Pathways in cancer;Prostate cancer
Circadian rhythm - fly;Circadian rhythm - mammal
Osteoclast differentiation
Ribosome biogenesis in eukaryotes;RNA transport
Neuroactive ligand-receptor interaction
Cell cycle;Cell cycle - yeast;Meiosis - yeast;Oocyte meiosis;Progesterone-mediated oocyte maturation;Ubiquitin mediated proteolysis
Alzheimer's disease;Amyotrophic lateral sclerosis (ALS);Apoptosis;ko05152;Natural killer cell mediated cytotoxicity;p53 signaling pathway;Pathways in cancer;Viral myocarditis
Cell cycle - yeast;RNA transport
Oocyte meiosis
Neuroactive ligand-receptor interaction
Plant-pathogen interaction

Anova Photon

Ribosome biogenesis in eukaryotes
Neuroactive ligand-receptor interaction
MAPK signaling pathway
Glycerolipid metabolism;Glycerophospholipid metabolism;Phosphatidylinositol signaling system
Huntington's disease;TGF-beta signaling pathway
Cytokine-cytokine receptor interaction;Jak-STAT signaling pathway
Adipocytokine signaling pathway;Cytokine-cytokine receptor interaction;Jak-STAT signaling pathway;Neuroactive ligand-receptor interaction
Vascular smooth muscle contraction
Non-small cell lung cancer;Pathways in cancer;Small cell lung cancer
Systemic lupus erythematosus
Neuroactive ligand-receptor interaction
Cell adhesion molecules (CAMs);Hepatitis C;Leukocyte transendothelial migration;Tight junction
Calcium signaling pathway;Neuroactive ligand-receptor interaction;Vascular smooth muscle contraction
Antigen processing and presentation;Natural killer cell mediated cytotoxicity
Bacterial invasion of epithelial cells
Cell cycle;Cell cycle - yeast;Meiosis - yeast;Oocyte meiosis;Progesterone-mediated oocyte maturation;Ubiquitin mediated proteolysis

Chemokine signaling pathway;Endometrial cancer;Neurotrophin signaling pathway;Non-small cell lung cancer
Antigen processing and presentation;Natural killer cell mediated cytotoxicity
Axon guidance;Fc gamma R-mediated phagocytosis;Regulation of actin cytoskeleton
Adherens junction
Neuroactive ligand-receptor interaction
Adipocytokine signaling pathway;Apoptosis;Hepatitis C;ko05152;RIG-I-like receptor signaling pathway
Insulin signaling pathway;Jak-STAT signaling pathway;Type II diabetes mellitus
Antigen processing and presentation;Natural killer cell mediated cytotoxicity
Cell adhesion molecules (CAMs);Hepatitis C;Leukocyte transendothelial migration;Tight junction
Calcium signaling pathway;Endocytosis;Neuroactive ligand-receptor interaction;Salivary secretion
Cytokine-cytokine receptor interaction;Rheumatoid arthritis
Cell adhesion molecules (CAMs);ECM-receptor interaction;Malaria
p53 signaling pathway
Cell adhesion molecules (CAMs)
Tight junction
Aldosterone-regulated sodium reabsorption;Taste transduction
Systemic lupus erythematosus
Mismatch repair
Bladder cancer;Non-small cell lung cancer;Pathways in cancer

Neuroactive ligand-receptor interaction
Systemic lupus erythematosus
Antigen processing and presentation;Natural killer cell mediated cytotoxicity
Systemic lupus erythematosus
Systemic lupus erythematosus
Neuroactive ligand-receptor interaction;Osteoclast differentiation
Systemic lupus erythematosus
Circadian rhythm - fly;Circadian rhythm - mammal;Hedgehog signaling pathway;Wnt signaling pathway
Aldosterone-regulated sodium reabsorption;Taste transduction
Calcium signaling pathway;Neuroactive ligand-receptor interaction
Huntington's disease
Purine metabolism;Thiamine metabolism
Systemic lupus erythematosus
Regulation of actin cytoskeleton
Fructose and mannose metabolism

p53 signaling pathway
Neuroactive ligand-receptor interaction
mRNA surveillance pathway
Neuroactive ligand-receptor interaction
Systemic lupus erythematosus
Systemic lupus erythematosus
Calcium signaling pathway;Dilated cardiomyopathy;Endocytosis;Gap junction;Neuroactive ligand-receptor interaction;Salivary secretion
Carbon fixation in photosynthetic organisms;Pyruvate metabolism
Purine metabolism
ancer;Focal adhesion;Glioma;Inositol phosphate metabolism;Melanoma;p53 signaling pathway;Pathways in cancer;Phosphatidylinositol signaling system;Prostate cancer;Small cell lung cancer
Bladder cancer;Leukocyte transendothelial migration;Pathways in cancer
DNA replication;Homologous recombination;Mismatch repair;Nucleotide excision repair
Neuroactive ligand-receptor interaction
Systemic lupus erythematosus
Neuroactive ligand-receptor interaction
Other types of O-glycan biosynthesis
Pathways in cancer;Small cell lung cancer
Protein processing in endoplasmic reticulum;Ubiquitin mediated proteolysis

Anova Photon

Cell cycle;Cell cycle - yeast;Meiosis - yeast;Oocyte meiosis;Progesterone-mediated oocyte maturation;Ubiquitin mediated proteolysis
Adipocytokine signaling pathway;Carbohydrate digestion and absorption;Galactose metabolism;Glycolysis / Gluconeogenesis;Insulin signaling pathway;Starch and sucrose metabolism
ithelial cells;Chemokine signaling pathway;Chronic myeloid leukemia;ErbB signaling pathway;Focal adhesion;Glioma;Insulin signaling pathway;Natural killer cell mediated cytotoxicity;Neurotro
Adherens junction;Circadian rhythm - plant;Measles;Ribosome biogenesis in eukaryotes;Tight junction;Wnt signaling pathway
Lysine degradation
Ribosome biogenesis in eukaryotes;RNA degradation
Calcium signaling pathway;Gastric acid secretion;Neuroactive ligand-receptor interaction
Systemic lupus erythematosus
Cytokine-cytokine receptor interaction
Systemic lupus erythematosus
Ribosome biogenesis in eukaryotes;RNA transport
Base excision repair
Chemokine signaling pathway;Cytokine-cytokine receptor interaction
Cell adhesion molecules (CAMs)
Maturity onset diabetes of the young
RIG-I-like receptor signaling pathway
Prion diseases
Ribosome biogenesis in eukaryotes
Ribosome biogenesis in eukaryotes;RNA transport
Arachidonic acid metabolism

Polito et al. Universal ready-to-use immunotherapeutic approach for the treatment of cancer: expanded and activated polyclonal γδ memory T cells.
Anova Photon

Proteasome
Ribosome biogenesis in eukaryotes
Antigen processing and presentation;Natural killer cell mediated cytotoxicity
Neuroactive ligand-receptor interaction;Vascular smooth muscle contraction
B cell receptor signaling pathway
Citrate cycle (TCA cycle);Pyruvate metabolism
Leukocyte transendothelial migration
Cell cycle;Cell cycle - yeast;Meiosis - yeast;Oocyte meiosis;Progesterone-mediated oocyte maturation;Ubiquitin mediated proteolysis
Cell adhesion molecules (CAMs);Hepatitis C;Leukocyte transendothelial migration;Tight junction
Neuroactive ligand-receptor interaction
Osteoclast differentiation
Protein processing in endoplasmic reticulum
pathway;Gastric acid secretion;Glioma;GnRH signaling pathway;ko05152;Long-term potentiation;Melanogenesis;Neurotrophin signaling pathway;Olfactory transduction;Oocyte meiosis;Photo
Pentose phosphate pathway;Purine metabolism
Cell adhesion molecules (CAMs);Hepatitis C;Leukocyte transendothelial migration;Tight junction

Hepatitis C;PPAR signaling pathway
Ribosome biogenesis in eukaryotes;RNA transport
e;Cell adhesion molecules (CAMs);Graft-versus-host disease;Intestinal immune network for IgA production;ko05152;Leishmaniasis;Phagosome;Rheumatoid arthritis;Staphylococcus aureus infe
Cell cycle;Cell cycle - yeast;Meiosis - yeast;Oocyte meiosis;Progesterone-mediated oocyte maturation;Ubiquitin mediated proteolysis
e;Cell adhesion molecules (CAMs);Graft-versus-host disease;Intestinal immune network for IgA production;ko05152;Leishmaniasis;Phagosome;Rheumatoid arthritis;Staphylococcus aureus infe
Cytosolic DNA-sensing pathway;Hepatitis C;Measles;RIG-I-like receptor signaling pathway
Cell cycle
Alanine, aspartate and glutamate metabolism;Arginine and proline metabolism;Nitrogen metabolism
Focal adhesion;MAPK signaling pathway
Osteoclast differentiation
oma;Leukocyte transendothelial migration;Long-term depression;Long-term potentiation;MAPK signaling pathway;MAPK signaling pathway - yeast;Melanogenesis;Natural killer cell mediated c
RNA transport
Cell cycle
Neuroactive ligand-receptor interaction
p53 signaling pathway
Lysine degradation
Cell adhesion molecules (CAMs);Epithelial cell signaling in Helicobacter pylori infection;Leukocyte transendothelial migration;Tight junction
Hedgehog signaling pathway;Wnt signaling pathway

Polito et al. Universal ready-to-use immunotherapeutic approach for the treatment of cancer: expanded and activated polyclonal γδ memory T cells.

Anova Photon

Systemic lupus erythematosus
Chemokine signaling pathway;Endocytosis
Alzheimer's disease;Amyotrophic lateral sclerosis (ALS);Apoptosis;Huntington's disease;ko05152;p53 signaling pathway;Parkinson's disease;Small cell lung cancer
Melanogenesis;Neuroactive ligand-receptor interaction
NOD-like receptor signaling pathway
Neuroactive ligand-receptor interaction
Bacterial invasion of epithelial cells
Neuroactive ligand-receptor interaction
osis;Focal adhesion;Glioma;Hepatitis C;Insulin signaling pathway;Jak-STAT signaling pathway;Leukocyte transendothelial migration;Measles;Melanoma;mTOR signaling pathway;Natural killer ce

Polito et al. Universal ready-to-use immunotherapeutic approach for the treatment of cancer: expanded and activated polyclonal γδ memory T cells.
Anova Photon

C: ANOVA Significant	N: -Log ANOVA p value	N: ANOVA q-value	T: Node
+	3,73899	0,00881119	ENSP00000019103
+	4,62251	0,008	ENSP00000020945
+	3,05974	0,00816092	ENSP00000168216
+	3,16871	0,00796129	ENSP00000176195
+	6,18228	0,002	ENSP00000215637
+	3,36514	0,00773109	ENSP00000216024
+	3,67316	0,00834783	ENSP00000216797
+	3,72645	0,00868493	ENSP00000219070
+	3,57461	0,00817204	ENSP00000219235
+	4,10577	0,00847619	ENSP00000219244
+	2,68929	0,00965145	ENSP00000219334
+	2,7016	0,00951782	ENSP00000220429
+	3,38348	0,00786147	ENSP00000221421
+	3,23083	0,00742456	ENSP00000222718
+	3,75346	0,00898551	ENSP00000225245
+	2,9523	0,00832821	ENSP00000227322
+	3,3066	0,00731818	ENSP00000229319
+	3,05308	0,00811299	ENSP00000229829
+	2,87861	0,00845411	ENSP00000230340
+	3,60344	0,0082905	ENSP00000231668
+	3,75155	0,00892086	ENSP00000231790
+	4,03654	0,00804301	ENSP00000232458
+	4,94602	0,00693333	ENSP00000234071
+	3,38616	0,00787826	ENSP00000234396
+	2,74424	0,00922944	ENSP00000234626
+	4,27222	0,00941538	ENSP00000235090
+	3,11978	0,00830864	ENSP00000235933
+	2,89323	0,00828362	ENSP00000237527
+	2,92936	0,00831313	ENSP00000240488
+	3,37807	0,00781116	ENSP00000240652
+	3,66874	0,00821951	ENSP00000241453
+	3,75878	0,00915556	ENSP00000242480
+	4,4415	0,00718367	ENSP00000243673

+	3,73036	0,00874483	ENSP00000244230
+	4,14934	0,00843373	ENSP00000244661
+	2,72806	0,00932479	ENSP00000245620
+	4,48746	0,00713043	ENSP00000249440
+	3,72031	0,00862585	ENSP00000250151
+	3,10578	0,00827964	ENSP00000251372
+	3,37205	0,00779574	ENSP00000251390
+	2,7247	0,00933617	ENSP00000251496
+	3,29458	0,0072809	ENSP00000251527
+	3,7421	0,00894286	ENSP00000251757
+	3,1956	0,00795973	ENSP00000252037
+	2,6823	0,00965914	ENSP00000252818
+	3,23469	0,00743463	ENSP00000253099
+	4,16503	0,0086	ENSP00000253462
+	4,05492	0,00808889	ENSP00000253792
+	3,59534	0,00817582	ENSP00000253796
+	3,48372	0,00809662	ENSP00000254122
+	3,78379	0,00939535	ENSP00000254605
+	3,00374	0,00816086	ENSP00000254661
+	4,31155	0,01	ENSP00000254810
+	2,9901	0,00816887	ENSP00000254846
+	4,51775	0,00761905	ENSP00000255040
+	3,00457	0,00815054	ENSP00000256246
+	5,81216	0,00533333	ENSP00000256592
+	4,48094	0,00691667	ENSP00000256925
+	3,93529	0,00826415	ENSP00000257118
+	3,68524	0,00859355	ENSP00000257700
+	2,74112	0,00926998	ENSP00000257745
+	2,87562	0,00846265	ENSP00000257915
+	4,03272	0,00779167	ENSP00000258034
+	3,57099	0,00810638	ENSP00000258456
+	2,87924	0,00846489	ENSP00000259791
+	3,25512	0,00737455	ENSP00000259808
+	4,34189	0,007	ENSP00000260227

+	3,1957	0,00798653	ENSP00000261497
+	4,05697	0,00836782	ENSP00000261669
+	2,83856	0,00857009	ENSP00000261819
+	3,90598	0,00843636	ENSP00000261942
+	3,26835	0,00739706	ENSP00000261991
+	2,91497	0,00825871	ENSP00000262013
+	4,03602	0,00795745	ENSP00000262178
+	3,70284	0,00862162	ENSP00000262300
+	2,92095	0,00826	ENSP00000262441
+	3,86416	0,00845217	ENSP00000262460
+	2,71004	0,0094346	ENSP00000262519
+	3,03351	0,00808864	ENSP00000262878
+	3,93284	0,00818692	ENSP00000262887
+	4,17452	0,00865823	ENSP00000263187
+	3,17181	0,008	ENSP00000263239
+	3,26624	0,00736996	ENSP00000263360
+	3,96839	0,00827723	ENSP00000263657
+	2,84757	0,00859574	ENSP00000263663
+	4,5321	0,0075122	ENSP00000263851
+	3,18048	0,008	ENSP00000263897
+	4,15954	0,00849383	ENSP00000264018
+	3,48319	0,00805769	ENSP00000264025
+	2,82538	0,00872222	ENSP00000264279
+	4,00888	0,00816327	ENSP00000264554
+	5,38186	0,00857143	ENSP00000264670
+	4,18204	0,00883117	ENSP00000264709
+	3,06755	0,0083787	ENSP00000264734
+	4,18268	0,00894737	ENSP00000264995
+	4,28032	0,00987097	ENSP00000265062
+	3,33267	0,00747826	ENSP00000265276
+	2,67827	0,00963599	ENSP00000265728
+	3,51943	0,0081592	ENSP00000265748
+	3,69018	0,00838961	ENSP00000266395
+	2,89972	0,00822549	ENSP00000266743

+	3,86039	0,00830769	ENSP00000267015
+	3,00522	0,00817251	ENSP00000267415
+	2,74814	0,00923747	ENSP00000268459
+	2,80387	0,00894064	ENSP00000268802
+	2,8266	0,00871462	ENSP00000268854
+	4,22493	0,00935211	ENSP00000269097
+	3,48383	0,00813592	ENSP00000269385
+	3,60171	0,00824444	ENSP00000269571
+	2,80842	0,00891034	ENSP00000269980
+	4,31285	0,0101695	ENSP00000270172
+	4,66798	0,00733333	ENSP00000270225
+	3,23673	0,00746099	ENSP00000270279
+	3,75817	0,0090219	ENSP00000270452
+	3,322	0,00737984	ENSP00000270590
+	3,87112	0,00856637	ENSP00000270631
+	3,53194	0,00832821	ENSP00000270800
+	3,46549	0,008	ENSP00000272369
+	3,19732	0,00798649	ENSP00000272847
+	2,8455	0,00858491	ENSP00000275198
+	3,29682	0,00732075	ENSP00000276014
+	3,04365	0,00808914	ENSP00000276533
+	4,0764	0,0084186	ENSP00000276571
+	2,96065	0,00824806	ENSP00000278175
+	3,66631	0,00819394	ENSP00000278193
+	3,33378	0,00749206	ENSP00000278947
+	4,02851	0,00775258	ENSP00000279804
+	3,61297	0,00841618	ENSP00000280358
+	3,84747	0,00966102	ENSP00000280665
+	3,61144	0,00834091	ENSP00000280704
+	3,67797	0,00845283	ENSP00000281513
+	3,35582	0,00770124	ENSP00000282091
+	3,36886	0,00774684	ENSP00000283254
+	3,33855	0,00765854	ENSP00000283256
+	3,0299	0,00807735	ENSP00000283646

+	3,15879	0,0080127	ENSP00000284154
+	3,06755	0,00835398	ENSP00000286809
+	3,16452	0,00792308	ENSP00000286827
+	2,74724	0,00922609	ENSP00000287239
+	3,16366	0,00791083	ENSP00000288699
+	2,8585	0,00857143	ENSP00000289753
+	3,3514	0,00767078	ENSP00000290219
+	3,23147	0,00743662	ENSP00000290974
+	4,84291	0,00647619	ENSP00000291294
+	3,6136	0,00846512	ENSP00000291526
+	3,41269	0,00794643	ENSP00000291547
+	3,06326	0,00819653	ENSP00000291759
+	2,92555	0,00827136	ENSP00000293217
+	4,72839	0,00696296	ENSP00000293406
+	3,28487	0,00731599	ENSP00000293549
+	3,60913	0,00829213	ENSP00000293695
+	3,92456	0,00811009	ENSP00000295522
+	3,3164	0,00736923	ENSP00000295756
+	2,96225	0,0082487	ENSP00000295959
+	4,25587	0,00923529	ENSP00000295987
+	4,51462	0,00744186	ENSP00000296682
+	2,67984	0,00964754	ENSP00000296802
+	3,14088	0,00805031	ENSP00000297012
+	4,80865	0,00636364	ENSP00000297229
+	2,72017	0,00933051	ENSP00000297579
+	2,97773	0,0082199	ENSP00000298048
+	2,74021	0,00926724	ENSP00000298552
+	3,33661	0,00759677	ENSP00000299333
+	3,24907	0,00749275	ENSP00000299424
+	3,06862	0,00839169	ENSP00000299766
+	2,68486	0,00964948	ENSP00000300051
+	4,27974	0,00971429	ENSP00000300061
+	2,66332	0,00974442	ENSP00000300086
+	3,0147	0,00817486	ENSP00000300151

+	3,78067	0,00925191	ENSP00000301219
+	3,04751	0,0081236	ENSP00000301336
+	2,88799	0,0083309	ENSP00000301364
+	3,96226	0,00819417	ENSP00000301917
+	2,6897	0,00967152	ENSP00000302393
+	3,12619	0,00827245	ENSP00000302548
+	3,43385	0,00816744	ENSP00000302846
+	2,78434	0,00916479	ENSP00000302898
+	4,74363	0,00752	ENSP00000303147
+	3,01429	0,00815259	ENSP00000303149
+	4,27011	0,00927273	ENSP00000303248
+	3,02088	0,00813223	ENSP00000303373
+	4,73188	0,00723077	ENSP00000303424
+	2,77096	0,00917595	ENSP00000303476
+	3,35456	0,00766942	ENSP00000303540
+	4,49135	0,00728889	ENSP00000303709
+	2,65349	0,00981891	ENSP00000304236
+	4,79545	0,00626087	ENSP00000305133
+	3,22694	0,0074007	ENSP00000305632
+	3,22822	0,00742657	ENSP00000305973
+	3,43657	0,00816822	ENSP00000306129
+	4,04302	0,00813043	ENSP00000306157
+	2,90847	0,00822716	ENSP00000306190
+	3,58137	0,00817391	ENSP00000306817
+	3,31669	0,00738224	ENSP00000307599
+	3,13686	0,0083	ENSP00000307684
+	3,24375	0,00756679	ENSP00000307939
+	3,70021	0,0085906	ENSP00000308022
+	3,16989	0,008	ENSP00000308252
+	3,04415	0,00810056	ENSP00000308369
+	2,65397	0,00983065	ENSP00000308461
+	2,90423	0,00823645	ENSP00000308699
+	3,54165	0,00812435	ENSP00000308750
+	3,1689	0,00798706	ENSP00000309504

+	2,86166	0,00855502	ENSP00000309539
+	3,37588	0,00779487	ENSP00000310928
+	3,5208	0,00818	ENSP00000311083
+	2,67283	0,00965041	ENSP00000311513
+	3,88528	0,00842857	ENSP00000311816
+	3,90385	0,00836036	ENSP00000311857
+	3,0148	0,00819726	ENSP00000313581
+	2,69897	0,00958664	ENSP00000314151
+	2,7869	0,00912217	ENSP00000314420
+	2,83348	0,00863256	ENSP00000314971
+	4,40536	0,00744	ENSP00000315017
+	3,63103	0,0084497	ENSP00000315569
+	3,21302	0,00788889	ENSP00000315997
+	2,92948	0,00833418	ENSP00000316578
+	4,36734	0,00732075	ENSP00000316589
+	3,09345	0,00825378	ENSP00000317376
+	2,99936	0,0081383	ENSP00000317691
+	2,88947	0,00833171	ENSP00000318077
+	2,98791	0,00818947	ENSP00000318775
+	3,06131	0,00817291	ENSP00000318822
+	3,82667	0,00935484	ENSP00000319169
+	2,79501	0,00906606	ENSP00000319318
+	2,97044	0,00823958	ENSP00000319531
+	4,69747	0,00703448	ENSP00000320180
+	3,03376	0,00811111	ENSP00000320291
+	3,18741	0,00797333	ENSP00000321606
+	2,73905	0,00925591	ENSP00000321724
+	5,40292	0,00933333	ENSP00000322341
+	5,36614	0,00666667	ENSP00000322439
+	3,33461	0,007552	ENSP00000322460
+	3,98554	0,008	ENSP00000323663
+	3,19409	0,00794649	ENSP00000324884
+	2,75854	0,00927753	ENSP00000325819
+	3,00313	0,00813904	ENSP00000326519

+	3,24237	0,00751254	ENSP00000326981
+	2,835	0,00859674	ENSP00000327072
+	2,914	0,00823821	ENSP00000327246
+	4,03278	0,00787368	ENSP00000327255
+	4,85869	0,00711111	ENSP00000327465
+	3,4093	0,00791111	ENSP00000327652
+	3,77893	0,00918182	ENSP00000328269
+	4,18885	0,00913514	ENSP00000328405
+	4,48746	0,00697872	ENSP00000328598
+	2,96665	0,00823896	ENSP00000328690
+	3,17297	0,00802614	ENSP00000328928
+	2,69261	0,00961667	ENSP00000329357
+	3,79305	0,00932283	ENSP00000329384
+	4,59273	0,00694737	ENSP00000329554
+	2,72636	0,00933902	ENSP00000330307
+	2,90407	0,00821622	ENSP00000330393
+	2,70453	0,00947059	ENSP00000330694
+	3,68304	0,00858974	ENSP00000330721
+	3,20776	0,00787586	ENSP00000331741
+	3,69745	0,00856	ENSP00000331746
+	3,5466	0,008125	ENSP00000332296
+	3,05572	0,00817143	ENSP00000332790
+	3,59432	0,00813115	ENSP00000332995
+	2,7812	0,00915315	ENSP00000333657
+	5,02691	0,00769231	ENSP00000333821
+	3,05868	0,00816046	ENSP00000334382
+	3,11085	0,00829358	ENSP00000335320
+	3,01383	0,00811924	ENSP00000335560
+	3,06755	0,00832941	ENSP00000336571
+	3,75836	0,00908824	ENSP00000336630
+	3,5991	0,00822099	ENSP00000336769
+	5,66345	0,004	ENSP00000336923
+	3,33077	0,00741961	ENSP00000339004
+	4,57216	0,00687179	ENSP00000339109

+	3,04554	0,00812325	ENSP00000339527
+	3,47764	0,008	ENSP00000339634
+	3,17437	0,00802623	ENSP00000339850
+	4,05495	0,00817978	ENSP00000339916
+	2,77391	0,00919911	ENSP00000340279
+	2,7934	0,00906364	ENSP00000340943
+	3,42436	0,00807306	ENSP00000341170
+	3,13527	0,00827414	ENSP00000341268
+	4,64305	0,007375	ENSP00000341280
+	3,60942	0,00831638	ENSP00000341282
+	4,23114	0,00942857	ENSP00000341327
+	2,9296	0,00835533	ENSP00000341874
+	3,48736	0,00817561	ENSP00000342071
+	3,53119	0,00824365	ENSP00000342215
+	3,06755	0,00830499	ENSP00000342445
+	3,42708	0,00809174	ENSP00000342848
+	3,4905	0,00819608	ENSP00000343246
+	3,18227	0,00801329	ENSP00000343690
+	4,87816	0,00775	ENSP00000343782
+	3,06582	0,0082087	ENSP00000344192
+	3,16691	0,00794855	ENSP00000344468
+	2,77885	0,00917753	ENSP00000344635
+	3,36177	0,00773222	ENSP00000344786
+	3,06755	0,0082807	ENSP00000345259
+	3,67909	0,00853503	ENSP00000345751
+	3,48135	0,00801914	ENSP00000346017
+	3,67745	0,0084	ENSP00000346509
+	3,34346	0,00767347	ENSP00000346534
+	3,14396	0,00806309	ENSP00000347602
+	3,28102	0,00727675	ENSP00000347733
+	3,61411	0,00851462	ENSP00000348020
+	3,73909	0,00887324	ENSP00000348510
+	2,72374	0,00932484	ENSP00000349547
+	3,84135	0,00956667	ENSP00000349873

+	3,0786	0,00835928	ENSP00000350005
+	3,13695	0,00832602	ENSP00000350009
+	2,69953	0,00956485	ENSP00000350028
+	2,91364	0,00821782	ENSP00000350162
+	2,68694	0,0096646	ENSP00000350249
+	3,00798	0,00816216	ENSP00000350275
+	3,28348	0,00728889	ENSP00000350719
+	2,74823	0,00925764	ENSP00000351446
+	3,83837	0,00944262	ENSP00000351589
+	4,86626	0,00752941	ENSP00000352035
+	3,53019	0,00820202	ENSP00000352064
+	3,84076	0,00952066	ENSP00000352119
+	3,57509	0,00819459	ENSP00000352252
+	3,41708	0,00801802	ENSP00000352561
+	3,84429	0,00964706	ENSP00000352627
+	3,07665	0,00833433	ENSP00000352839
+	2,67577	0,00962118	ENSP00000352929
+	3,69574	0,00850331	ENSP00000353048
+	4,197	0,00927778	ENSP00000353098
+	4,04628	0,00813187	ENSP00000353151
+	3,78128	0,00932308	ENSP00000353292
+	4,37335	0,00752941	ENSP00000353508
+	3,96472	0,00827451	ENSP00000353581
+	5,48652	0,0088	ENSP00000353915
+	2,80646	0,00892661	ENSP00000354497
+	3,18001	0,008	ENSP00000355077
+	3,41582	0,00798206	ENSP00000355140
+	4,59723	0,00713514	ENSP00000355228
+	3,83649	0,00936585	ENSP00000355330
+	2,9804	0,00823097	ENSP00000355587
+	2,91673	0,00825935	ENSP00000355778
+	2,84086	0,00861176	ENSP00000355809
+	3,29626	0,00730827	ENSP00000356022
+	2,86284	0,00857554	ENSP00000356047

+	3,3819	0,00782759	ENSP00000356150
+	3,69444	0,00844737	ENSP00000356213
+	2,65514	0,00985051	ENSP00000356224
+	2,93708	0,00836735	ENSP00000356480
+	3,64181	0,00838095	ENSP00000356548
+	3,40084	0,00789427	ENSP00000356811
+	3,62165	0,00854118	ENSP00000356908
+	3,77775	0,00920301	ENSP00000357726
+	2,78801	0,00913379	ENSP00000358019
+	2,7696	0,00917333	ENSP00000358131
+	3,662	0,00816867	ENSP00000358155
+	4,05565	0,00827273	ENSP00000358160
+	2,87359	0,00845192	ENSP00000358301
+	4,26433	0,00919403	ENSP00000358452
+	3,61251	0,00836571	ENSP00000358531
+	3,20729	0,0078488	ENSP00000358595
+	2,85253	0,00856057	ENSP00000358719
+	4,69799	0,00728571	ENSP00000359321
+	2,75713	0,00928352	ENSP00000360502
+	3,39553	0,00784279	ENSP00000360540
+	2,95632	0,00827763	ENSP00000361021
+	2,73657	0,00925054	ENSP00000361405
+	3,41798	0,00809091	ENSP00000362131
+	3,69406	0,0084183	ENSP00000362183
+	2,76829	0,00915929	ENSP00000362208
+	5,15513	0,00763636	ENSP00000362352
+	5,37969	0,0075	ENSP00000362353
+	3,42992	0,0081106	ENSP00000362410
+	4,2905	0,00990164	ENSP00000362690
+	3,21275	0,00786159	ENSP00000362824
+	3,79983	0,00939683	ENSP00000362979
+	2,70662	0,00945684	ENSP00000362994
+	4,9996	0,00714286	ENSP00000363003
+	3,53281	0,00837113	ENSP00000363019

+	2,80568	0,00893364	ENSP00000363322
+	4,61653	0,00754286	ENSP00000363640
+	3,05427	0,00814773	ENSP00000363976
+	3,55523	0,00810471	ENSP00000364512
+	4,17721	0,00876923	ENSP00000364855
+	3,76531	0,00922388	ENSP00000364976
+	3,67807	0,00850633	ENSP00000364995
+	2,68557	0,00966942	ENSP00000365025
+	4,50689	0,00745455	ENSP00000365107
+	3,56241	0,00810582	ENSP00000365806
+	2,93163	0,00836641	ENSP00000365851
+	3,39587	0,00787719	ENSP00000365877
+	4,15054	0,00853659	ENSP00000365991
+	2,73775	0,00925322	ENSP00000366396
+	3,67226	0,0082963	ENSP00000366506
+	3,16406	0,00792332	ENSP00000366679
+	2,76364	0,00918322	ENSP00000366729
+	4,556	0,0069	ENSP00000366970
+	4,35232	0,00712727	ENSP00000366999
+	3,07298	0,00834524	ENSP00000367203
+	3,35991	0,0077	ENSP00000367439
+	3,52808	0,0081809	ENSP00000367787
+	2,84972	0,0085782	ENSP00000367802
+	3,19866	0,00789153	ENSP00000367832
+	3,20013	0,00790444	ENSP00000368314
+	4,65653	0,00735484	ENSP00000368401
+	3,40784	0,0079115	ENSP00000368411
+	3,98998	0,00808081	ENSP00000368632
+	2,99082	0,00819048	ENSP00000368699
+	2,88597	0,00832039	ENSP00000368752
+	2,92115	0,0082807	ENSP00000369162
+	2,77133	0,0091875	ENSP00000369344
+	3,00112	0,00813867	ENSP00000369391
+	3,08421	0,00837349	ENSP00000369530

+	4,6196	0,00776471	ENSP00000369681
+	3,86907	0,00849123	ENSP00000370222
+	4,33162	0,0103158	ENSP00000370293
+	3,20412	0,00786301	ENSP00000370589
+	3,79175	0,00928125	ENSP00000370710
+	4,84741	0,0066	ENSP00000373648
+	2,77569	0,00918386	ENSP00000375608
+	3,29037	0,00726866	ENSP00000376162
+	5,16997	0,0084	ENSP00000376177
+	3,04983	0,00814648	ENSP00000376544
+	2,94583	0,00832737	ENSP00000376946
+	3,65183	0,00833533	ENSP00000377298
+	2,95865	0,00826804	ENSP00000377384
+	2,92894	0,00829219	ENSP00000377496
+	3,32428	0,00740856	ENSP00000377527
+	2,6828	0,00967901	ENSP00000377771
+	3,55687	0,00812632	ENSP00000377947
+	2,82285	0,00874827	ENSP00000378350
+	3,06755	0,00825656	ENSP00000378577
+	3,11487	0,00832	ENSP00000379086
+	3,94048	0,00830476	ENSP00000379213
+	3,8621	0,00837931	ENSP00000379514
+	3,17736	0,008	ENSP00000379651
+	3,19974	0,00789116	ENSP00000380024
+	2,67702	0,00963265	ENSP00000380252
+	2,75431	0,0092807	ENSP00000381412
+	3,15329	0,00803797	ENSP00000381504
+	3,49752	0,00819704	ENSP00000381590
+	3,1337	0,00827329	ENSP00000381840
+	4,32453	0,0102759	ENSP00000383303
+	3,43306	0,00814815	ENSP00000383672
+	3,33154	0,00744882	ENSP00000384015
+	4,10159	0,00837647	ENSP00000384490
+	3,06755	0,00823256	ENSP00000384554

+	3,61281	0,00841379	ENSP00000385122
+	3,66956	0,00826994	ENSP00000385616
+	3,3367	0,00762753	ENSP00000386306
+	3,31461	0,007341	ENSP00000386796
+	3,53152	0,00828571	ENSP00000387946
+	2,86086	0,00853461	ENSP00000389182
+	3,05363	0,00812465	ENSP00000390020
+	3,37148	0,00777966	ENSP00000390600
+	2,76947	0,00915299	ENSP00000392617
+	2,83908	0,0085808	ENSP00000394394
+	4,35653	0,00725926	ENSP00000394791
+	3,33461	0,00752191	ENSP00000396320
+	3,80069	0,009472	ENSP00000396774
+	3,34691	0,00768852	ENSP00000396915
+	3,05569	0,00814815	ENSP00000398890
+	3,73557	0,00877778	ENSP00000399511
+	3,24311	0,00753957	ENSP00000401018
+	2,97397	0,00822977	ENSP00000401980
+	4,372	0,00738462	ENSP00000402084
+	4,84903	0,00694737	ENSP00000402608
+	3,32599	0,00742188	ENSP00000405890
+	3,24128	0,00748571	ENSP00000405963
+	3,41789	0,0080543	ENSP00000406478
+	2,75188	0,00926915	ENSP00000408695
+	2,99275	0,00819098	ENSP00000409799
+	4,27572	0,0095625	ENSP00000411552
+	3,74061	0,00887943	ENSP00000411658
+	2,71833	0,0093277	ENSP00000414287
+	3,09991	0,00825455	ENSP00000415430
+	3,10699	0,00830488	ENSP00000417980
+	3,30707	0,00734601	ENSP00000420294
+	3,01384	0,0081413	ENSP00000420419
+	3,57456	0,00812834	ENSP00000420927
+	3,95678	0,00819231	ENSP00000421689

+	4,19533	0,00915068	ENSP00000421922
+	7,11851	0,004	ENSP00000422753
+	2,7446	0,00924078	ENSP00000423563
+	3,45121	0,00807547	ENSP00000428824
+	3,92529	0,00818519	ENSP00000430690
+	2,6571	0,00982186	ENSP00000433511
+	5,11538	0,00766667	ENSP00000434045
+	3,112	0,00831902	ENSP00000435619
+	3,2569	0,00738686	ENSP00000439493
+	4,59774	0,00733333	ENSP00000439660
+	4,24936	0,00933333	ENSP00000444823
+	3,08219	0,00834835	ENSP00000448165
+	3,23767	0,00748754	ENSP00000448888
+	3,01481	0,00821978	ENSP00000451605
+	4,74756	0,00766667	ENSP00000452746
+	4,18558	0,00901333	ENSP00000453076
+	2,84057	0,00859155	ENSP00000453403
+	2,82179	0,00877419	ENSP00000459962
+	3,49909	0,00823762	ENSP00000460441
+	3,33657	0,00756627	ENSP00000463376
+	3,30757	0,00737405	ENSP00000467494
+	3,44427	0,00805634	ENSP00000471505
+	2,65032	0,00985542	ENSP00000471914

Polito et al. Universal ready-to-use immunotherapeutic approach for the treatment of cancer: expanded and activated polyclonal γδ memory T cells.
Anova Photon

T: UniProt
P47872;Q12961;Q13213;Q53T00
B2R6P6;O43623;Q53FC1
Q5H927;Q6IBS9;Q8TCV9;Q96HD5;Q99714
P09683
A5PKV4;B2RPL9;O60222;O75867;Q13477;Q5UGI7
A8K9A2;B4DMW6;Q08AI1;Q14565;Q99498;Q9UH11
B2R8L6;P25963
B2R6U1;B4DWH3;E9PE45;P08253;Q9UCJ8
A0N0Q6;B2R4W2;O00626
A0N0Q9;Q2M287;Q92583
B7ZL82;Q2M1S8;Q9NY59
A8IE52;Q06545;Q06547;Q12940;Q12941;Q12942;Q8IYD0
P01229;Q9UDI0
A1L4K3;B2RMW3;O43364
O75312;Q2TAA0
P07195
P06340;Q58HU0;Q58HU1;Q5STC7;Q9TQC6;Q9TQC7;Q9TQC8;Q9TQC9;Q9TQD0;Q9TQD1;Q9TQD2;Q9TQD3
Q13895;Q6P5W4;Q86W44;Q96IP8
D3DQM3;D3DQM4;D3DQM5;D3DQM6;O75622;O75623;O75624;Q12981;Q6K044;Q96FG4
B4DI13;B4DQ11;E9PCU2;P40692
Q0MT80;Q2M269;Q6U836;Q9H8V3;Q9NSV8;Q9NVW9
B4DPQ7;P04070;Q15189;Q15190;Q16001;Q53S74;Q9UC55
P15313;Q53FY0;Q6P4H6
D3DT31;O00311;O00558;Q5T5U5
B3KMW6;B4DP38;Q3LID2;Q53FU2;Q6JZZ5;Q96GK4;Q9BQA1;Q9BWY3
O95971
P01286;Q4KN10;Q5JYR1
B2R5F9;Q9BWT6
P10997;Q0ZD87;Q14598
A0AVG9;B7ZLT7;B7ZLT8;F5H0A0;P36888;Q13414
B2R724;B3KRD7;P11161;Q68CZ5;Q8IV26;Q9UNA6
B0M0K5;Q6NWR4;Q9NYM4;Q9P1Y8

Anova Photon

A0AVJ8;O00566
C9J1P3;C9JIP1;O15471;O75022;Q86U49
P31249;Q99955;Q9BSC5
O75018;O75019;Q3MJA6
Q3MJE0;Q96SV9;Q9BPX3;Q9BUR3;Q9BVY1;Q9H914;Q9H9Z6;Q9HBI9
A0FGR8;A4D229;Q69YJ2;Q6UKI4;Q6ZTU0;Q6ZVU1;Q9BQS0;Q9NW47;Q9ULJ2
B4DXT7;G3V0I2;O75344;Q7Z4T4;Q9UDS0
P17535;Q53EK9
A6NNV7;Q9BW07;Q9BYD3;Q9H4N2;Q9Y317
D3DUM5;Q6IAG9;Q9Y248
B4DIM0;B4E3P0;P53396;Q13037;Q9BRL0
A7L9S6;K7EMD3;O60895;Q8N1F2
A2TF08;A5JVV3;P01225;Q14D61
O43159;Q7KZ78;Q9BVM6
O60894;Q6FGS5
P06351;P33155;P84243;Q5VV55;Q5VV56;Q66I33;Q9V3W4
C9IZ40;O15054;Q96G33
P02743
Q9BXT5
B1AKP0;P01222;Q16163
B4DK60;Q8N3Y8;Q8NA22;Q8TDN4;Q9BTG1
A1L4Q1;A8KA40;D3DPR2;Q2TAL3;Q5T0C1;Q6NUJ6;Q6ZQR1;Q8IXK0;Q8N306;Q8TAG8;Q96BL4;Q9Y4Y7
Q6NUQ1;Q75MG9;Q75MH0;Q96IW8;Q9H229;Q9HAD9
B6ZDE4;B6ZDM3;M4K8J3;Q6P5Y2;Q6PKG4;Q6T316;Q86TI3;Q86W12;Q86WG0;Q86WL2;Q8IV78;Q8IWR5;Q8IZD2;Q8NFF8;Q9NWE7
A8K5E9;Q12800;Q12801;Q9UD75;Q9UD77
D8KZS1;O14804;Q2M1V1;Q4VBL1;Q5VUQ3;Q6NTA8
Q6NWS4;Q6NXU6;Q9Y5Y3
A0AV89;B2RAG1;B4E3F9;Q13180;Q13546;Q59H33
P09237;Q9BTK9

Polito et al. Universal ready-to-use immunotherapeutic approach for the treatment of cancer: expanded and activated polyclonal γδ memory T cells.
Anova Photon

A0JNS3;Q2NLE2;Q6MZY4;Q8TBS8;Q96IW5;Q9UPT9
Q5TAW6;Q6WG71;Q96FG1;Q9BZ33;Q9H9S4
E9PFB2;Q8N4H7;Q9BQD4;Q9UJX4
O94963;Q8IUF2;Q96CS3;Q9BRP2;Q9BVM7
A0A0A0MQV2
A6H8U5;A8MSX0;B4DHH2;O60271;O60905;Q3KQU8;Q3MKM7;Q86WC7;Q86WC8;Q8IZX7;Q96II0;Q9H811
P41587;Q13053;Q15870;Q53Y09;Q6ZN22;Q9UCW0
B3KUN8;B4DXD4;D3DUA4;F8W164;I3L1V2;O14731;Q7LE24;Q8TCM9;Q99640
O95838;Q4VAT3
Q14691;Q9NQE2;Q9NQI7
A6NP62;O15047;Q6PIF3;Q8TAJ6
B4E2A5;E1P5V2;Q5JXG8;Q8N491;Q9H004;Q9H005;Q9H3U9;Q9Y3Z3
P18887;Q6IBS4;Q9HCB1
O15457;Q5T4U6;Q8NEB3;Q9UNP8
Q6GTZ9;Q6IAU4;Q92732;Q9BQB7;Q9NVP1
A8K7V5;O00149;O75530;Q6NTH2;Q7LDA5;Q7LDG8;Q86VV2;Q9UNY7
A8K6Q0;Q53G13;Q8WVB8;Q9NRX1
B4DI42;F8WD72;Q15574;Q53T94;Q8WVC3
A0N0L3;P13232;Q5FBY5;Q5FBY9
Q8NCK1;Q9BRT4;Q9NZ43
O75465;Q15223;Q2M3D3;Q9HBE6;Q9HBW2
Q53SA4;Q6PK08;Q9P036;Q9UFN3;Q9Y2X3
O60230;P98077;Q9NPL5;Q9UCX4
A8K529;B2RNR4;B3KP09;B4DQW2;G3V1R4;Q08J23;Q9BVN4;Q9H858;Q9NXD9
E9PEB8;Q86TE8;Q86XF5;Q8IZV0;Q8WXU9;Q9Y6K1
Q9Y5I7
P09001;Q6IBT2
A8K3V6;P51149;Q9NWJ0;Q9UPB0
Q5VW51;Q86TA3;Q9HCL2
A4D1D8;A8K954;O75226;Q75MS6;Q75N01;Q9UBU7;Q9Y2M6
Q5CZ78;Q6NSK5;Q9H8Y4;Q9NQW6;Q9NVN9;Q9NVP0
Q13956;Q52LY7
Q8IZU3

B6V9G7;Q9NQS5
B3W5Q7;Q9BSI4;Q9H904;Q9UHC2
B2RC39;Q8WZ08;Q969G9
Q7L6B7;Q7M4M4;Q7Z4B5;Q9NWB0;Q9ULX3
Q8WU15;Q9BUM1
Q96H39;Q9HC52;Q9NR07
B2RZG3;B4DHN3;P04626;Q14256;Q6LDV1;Q9UMK4;X5D2V5
B4DP47;E7EW46;P12694;Q16034;Q16472
E9PB42;Q9BUJ4;Q9UJW3
B2RDP5;B3KMQ2;F5GXX7;G3XAK6;O95717;Q9P020;Q9UBE0
Q8N1E5;Q9ULV8;Q9Y5Z2;Q9Y5Z3
A0A0A0MQW7
O75388;Q502U7;Q6NWS5
Q96A98;Q96DJ4
A8K839;B2R9Y9;Q8N6P7;Q9HB22
A8MV50;O00470
P49190;Q8N429
Q5VUQ4;Q96RI8
B1AQI5;B1AQI6;Q8WWL7;Q96SB5;Q96SB6;Q96SB7;Q9NT38
B2R8H5;D3DSY0;Q8N648;Q9BRT9
B3KQS4;P06850
B2R793;D3DQV3;P35318;Q6FGW2
Q9NUP9
O60939;O75302;Q9UNN3
A8MVX4;Q16619;Q5U5Y7
A6NDL9;B0YIX3;Q9BXU0
B4DRD1;Q86XH9;Q8IZD4;Q96BP8;Q96MZ8
D3DQY4;P07864;Q6GSG8;Q7Z7J4
A2RRP1;O95790;Q2VPJ7;Q53TK6;Q86V39;Q8NFY8;Q9Y3W5
P01270;Q4VB48;Q9UD38
Q16142;Q53SX0;Q9BZB3;Q9C006;Q9NY46;Q9NYK2;Q9P2J1;Q9UPD1;Q9Y6P4
A6NC14;A6NIQ5;Q14472;Q53T77;Q99250;Q9BZC9;Q9BZD0
P49247;Q541P9;Q96BJ6

Anova Photon

Q13588
B7ZLR6;F5GZ53;Q13009;Q17RT7
O15087;Q86Y05;Q8WU81;Q8WYB5;Q9UKW2;Q9UKW3;Q9UKX0
Q8TCL6;Q9BPU6;Q9NQC4;Q9NRY9
P50406;Q13640;Q5TGZ1
P38484;Q9BTL5
B2RP83;B3KX50;B7Z1Q7;B7Z2G9;B7Z2M2;B7ZB19;E9PB54;E9PB64;E9PG77;Q7Z6J3;Q9HCC9
P43119
Q03403;Q15854
O00528;P55347;Q8IWT7
P59901;Q32MC4
A8K6X8;A8KAA0;B4DK61;F5GYQ8;Q12863;Q15067;Q15068;Q15101;Q16131;Q7Z3W5;Q9UD31
Q3MHD2;Q86YB1;Q96NL5
P04628;Q5U0N2
B4DYD3;Q6PIF2
O95832
Q6PIZ9;Q9NZX5
O95059;Q53X97
B1AJQ1;O75825;P17600;Q5H9A9
B4DX22;Q27Q50;Q9NQV7
A0A0A0MQZ6
Q96QV6
A0A087WT20
A6P3A7;A6P3A8;B1AMQ6;B7Z1E6;B7Z5M5;B7Z6Q7;B7Z6R8;B7Z6Y0;B7Z7Q1;D3DRP8;F5H0Y0;F5H2R4;F5H689;Q14680;Q7L3C3
B7Z897;Q5VVN5;Q92574
A5H1I5;Q17RL3;Q9NY72;Q9ULR2
O00703;Q12962;Q13175;Q6FH13
B2RAC3;P32245;Q16317;Q3MIJ6
Q86WU2;Q8IZK5
P51170;P78437;Q6PCC2;Q93023;Q93024;Q93025;Q93026;Q93027;Q96TD2
B4DQN4;Q4W4Y2;Q8WYZ3;Q9NWR2;Q9NYB0
Q9BYD0;Q9HB70;Q9NX20

B2RBQ8;Q71RE6;Q96NA2;Q9BSL3;Q9BYS3
Q2NL82;Q8WUY5;Q9NVT0;Q9P2E6
A0AVJ5;O00155
Q6DUY4;Q96LI2;Q9BYZ2
B2R9R3;P09016;Q96AU0
P35408;Q3MJ87
O60681;O75442;Q6AZY8;Q6DLZ0;Q9UPK5;Q9UQB9
A8K511;B4DN45;D6W5L1;P31153;Q53SP5
Q9NS67
B4DHD1;B4DTV2;Q2M215;Q3KU24;Q3KU25;Q3KU26;Q9HBX9
P04908;P28001;Q76P63
G3V5L3;Q13250;Q13251;Q13258;Q1ZZ52
A6NLB8;Q9Y4G6
E9PG49;P35498;Q16172;Q585T7;Q8IUJ6;Q96LA3;Q9C008
B2RBX4;C9J8K2;K4DI90;P51965
P08571;Q53XT5;Q96FR6;Q96L99;Q9UNS3
O75159;Q53SD4;Q8IYZ4
O95269;O95349;Q9NSB6;Q9NSB7;Q9NSB8;Q9UNT7
P09017
B2R9S3;P21918;Q8NEQ8
B2RCS6;B4DVT1;P16871;Q05CU8;Q6NSP4;Q6NWM0;Q6NWM1;Q6NWM2;Q6NWM3;Q6SV45;Q9UPC1
Q13291;Q5W172;Q9HBE8
A9UK04;B4DV96;Q9H477
A6NN05;C9JD48;C9JR31;Q68K15;Q8NDX3;Q9UHE0;Q9UHM0;Q9UI33
O75528;Q6FI83;Q9UFS2
A6H8X8;O15045;Q4ZG46;Q8IWJ2;Q8TDH3;Q9H2G8
A0AUJ8;A4QMR7;B3KSN0;B3KU86;Q6MZG9;Q86TJ2
A8K567;B7Z5N8;B7Z5P8;B7ZAD0;D3DTV3;O95615;P14651;P17484
Q9NT47;Q9NYA5;Q9Y2U8
A8K9P7;Q92730
Q0VDA5;Q14781;Q9BTB1
Q24JS8;Q8TE53;Q8TE54;Q96RN2

Anova Photon

A8K5H2;B4DR31;D3DSS7;O00424;Q16555
A8K6J6;B4E3V3;B6ZGS1;B7Z3W1;E9PE18;Q03181;Q5D1P0;Q7Z5K0;Q9BUD4
P33551;P61024
Q86X86;Q96T23;Q9H3L8;Q9NVZ8;Q9NYU0
A2RUE0;B9EGJ0;Q12956;Q12957;Q13127;Q13134;Q59ER1;Q8IWI3
A8K955;A8MXU3;K7ENG3;P17706;Q96AU5;Q96HR2
B4DU93;B4DUB0;F5H8L3;P20151;Q15946;Q9UJZ9
C9JXH3;G3V0H4;G3XAE3;P07288;Q15096;Q16272;Q86TG8;Q8IXI4
B2RPA8;B4DI49;D3DTR3;O15534
Q6I9Y6;Q9H5J8
A6NG03;Q4W5B8;Q6IAG4;Q96BW3;Q9BYD6;Q9H793;Q9NRL5
Q53S79;Q580W6;Q5XKH0;Q69YG5;Q6AW99;Q7Z4T6;Q8IYB7;Q8N9K9
A0A087WSV6
Q15022;Q96BD9
A4FTY2;B4DID0;B4DQH9;E7EW27;Q6VN20;Q9P264
B2RD52;P82912;Q969D7;Q96GI3;Q9BYC3
D3DW70;Q9BUL9;Q9NX88
B0YJ34;P33032;Q502V1
A8K8H1;E9PCR4;Q6PCC6;Q9NSH6;Q9UJX5
P55957;Q549M7;Q71T04;Q7Z4M9;Q8IY86
A8MTP3;A8MZ91;B4DX49;B4DY30;B5BU10;D3DS33;E2QRE7;O14744;Q6IBR1;Q9UKH1
A6H8Z1;B4DZ10;D6W5U8;H7BYK9;Q8NDP3;Q9UJ98
P23434;Q9H1E9
Q02643;Q99863
B7Z7D3;Q9BXW6;Q9BZF5;Q9NW87
A0EJG6;Q13024;Q14194;Q4W5F1;Q96TC8
B4DZ72;G3XAG0;P51460;Q3KPI5;Q3KPI6;Q6YNB5;Q9UEA2;Q9UPH6
P57723;Q96AH7
O15276;P49411
E9PPT5;Q6PIG5;Q8IWT1
D3DX56;Q8NFT6;Q8TEX0;Q96B19;Q9H912
A4D181;O43365
P10589
A2ICR6;Q9BTK6

Polito et al. Universal ready-to-use immunotherapeutic approach for the treatment of cancer: expanded and activated polyclonal γδ memory T cells.
Anova Photon

B3KQ67;Q53G10;Q9NV31
B2RDZ8;D3DTB7;Q15573;Q9NWA1
A5JUT9;B3KPV1;B4DEB5;B4DGI4;F5H1F5;G3V0I1;P32241;Q15871;Q6P2M6
B5BU11;J3KNM0;O75551;P35813
E1P5H0;F5H7M8;O94922;Q01538;Q7Z5W2;Q9UPV2
A6NMS5;D6W616;Q6IBP8;Q8NBD5;Q9HD21;Q9P0W2;Q9Y491;Q9Y4A2
O75481;O75482;O75483;O95217;P23743;Q3ZE25;Q8IZ56;Q8N5Q2
B2RAB4;Q9GZZ0
E7EMJ4;Q4V9L5;Q9GZR7
P17483;Q9NTA0
E4Z9M7;G5E9M8;P08047;Q86TN8;Q9H3Q5;Q9NR51;Q9NY21;Q9NYE7
Q9GZX6
P48357;Q13592;Q13593;Q13594;Q92919;Q92920;Q92921
Q8IW16;Q8TEI6;Q96MG7;Q9H214
Q8N4P3
P10913;P14652;P17485
B7ZL39;P01258;Q13935;Q13937;Q52LX7
P10826;P12891;Q00989;Q15298;Q9UN48
Q8IUE6
A0A0C4DFR3
B7Z5D3;D3DSI7;P20592
A8K016;Q01718;Q3MI45;Q504X6
B2R688;O96003;Q86YM5;Q86YM7
A1A4C1;Q67FY1;Q6ZWJ0;Q6ZWK2;Q86UU0
A6NJH5;A9CQZ6;F8W7Z4;Q86VW2;Q8WV84;Q96E63
B2R6B9;P57739
B2R7E0;P29274
Q6H2H3
A1A4S6;A1L0S5;Q2VPC4;Q2VPC5;Q96EV3;Q96S75
A6NFY2;P55315;P55316;Q14488;Q86XT7
Q2M3H8;Q9BSE6;Q9H1A4;Q9H8D0

B4DVZ6;E1P5E6;O15171;O43524;Q5T2I7;Q9BZ04
A8W795;O14621;O14622;O14623;O14624;O43534;P78400;P78401;Q8N738;Q99559;Q99560;Q99561;Q99562;Q99706;Q9UQJ7
B2RMQ1;D3DRG5;O00240;O14531;Q5T0Q7
A8K6H5;P53671;Q7KZ80;Q7L3H5;Q96E10;Q99464;Q9UFU0
A8K8W0;B4DFE3;D3DT13;J3KR02;Q6P2P8;Q6ULS1;Q7L168;Q9UIX0;Q9Y2D8
B2R967;B3SXS6;B3SXS7;B3SXS8;B3SXT0;F8WA81;O43461;Q13324;Q4QRJ4;Q99431
P21246;Q5U0B0;Q6ICQ5;Q9UCC6
B2RDS3;B3KQZ9;Q15628;Q52NZ1
A8K9Z6;B7Z5S5;B7Z8L0;Q5U5L5;Q86X63;Q92833
B2RC80;Q8N0S2;Q9BWU3;Q9BWU4
Q8WXH5
O14730;Q8IXN9
O43472;P43628;P78402;Q14944;Q14945;Q9UM51;Q9UQ70
O14493
O60533;Q29VU9;Q29VV0;Q2TAM1;Q86UR1;Q8IUS3
A4D184;B2R8U7;O43363;P49639
B3SXQ8;Q14195;Q93012
P13945;Q4JFT4
Q16552;Q5T2P0
O75056;Q5T1Z6;Q5T1Z7;Q96CT3;Q96PR8
B2R7B2;B4DLW7;D3DQK7;P51959;Q15757;Q96L32
NN8;B3KQZ1;B5MDP6;B5MDR6;B7ZMD8;O94856;Q149P5;Q5T2F0;Q5T2F1;Q5T2F2;Q5T2F3;Q5T2F4;Q5T2F5;Q5T2F6;Q5T2F7;Q5T2F9;Q5T2G0;Q5W9F8;Q68DH3;Q6ZQV6;Q7Z3K1;Q96HT1;Q96
B1ALH8;B1ALH9;D3DPM9;D3DPN0;P11171;P11176;Q14245;Q5TB35;Q5VXN8;Q8IXV9;Q9Y578;Q9Y579
C5HTZ2;O60891;P51168;Q96KG2;Q9UJ32;Q9UMU5
A8K573;D3DQX4;P10092;Q569I0;Q9UCN9
A1L4E4;P0C5Z0;P98176;Q5TZB2;Q6FG78;Q96PR7
P29374;Q15991;Q15992;Q15993
A4D265;O75218;Q9Y4A5;Q9Y631;Q9Y6H4
P49751;Q56DK9;Q9P292;Q9UHC0;Q9UHC1
B2RP21;Q12789;Q12838;Q6DKN9;Q9Y4W9
B7ZLL1;O14571;O60539;O60710;Q0VGC6;Q5TZT2;Q9HB04;Q9HB18;Q9NS22;Q9NS23;Q9UND4;Q9UND5

P09681;Q4VB42;Q6NTD3
Q8TEY6;Q8TEY7;Q96AV6;Q9H9F0;Q9UPQ5
Q5JR03;Q8TEF0;Q9BSY3;Q9BUJ9;Q9HCE1
A2RUE5;O75763;Q5JX11;Q9BX26;Q9NTX8;Q9UG27
O95018;Q5MJ36;Q9H662;Q9NUX5;Q9NW19;Q9UG95
B4DND7;B4DPR6;I6XXQ5;Q540G1;Q86YP9;Q8N1S3;Q8NF49;Q8TER7;Q8WWW3;Q8WWW4;Q8WWW5;Q8WXH0;Q8WXH1;Q9NU50;Q9UFQ4;Q9Y2L4;Q9Y4R1
P61964;Q91VA5;Q9NWX7;Q9UGP9
P02261;P0C0S8;Q2M1R2;Q76PA6
O43526;O43796;O75580;O95845;Q4VXP4;Q4VXR6;Q5VYT8;Q96J59;Q99454
P26715
P02261;P0C0S8;Q2M1R2;Q76PA6
A0PJT7;A5PLR1;P02295;P02296;P16106;P68431;Q6ISV8;Q6NWP8;Q6NWP9;Q6NXU4;Q71DJ3;Q93081
A0A0A0MRG0
P02261;P0C0S8;Q2M1R2;Q76PA6
A2RTZ4;Q2XP58;Q56H76;Q56H77;Q56H78;Q6JSL4;Q6JSL5;Q6JSL6;Q6JSL7;Q6JSL8;Q6W5P3;Q6W5P4;Q6ZMB8
P49674
A4QPH3;Q96SF2;Q9UJS3
B3KSP7;Q6IA56;Q9BTE3;Q9BVT9;Q9H916
A4D180;O43366;Q00056
A5X2U9;B4E2Q5;C5HTZ0;O43271;P37088;Q6GSQ6;Q9UM64
P11137;Q17S04;Q8IUX2;Q99975;Q99976
C4WYH4;Q13639;Q546Q1;Q684M0;Q712M9;Q96KH9;Q96KI0;Q9H199;Q9NY73;Q9UBM6;Q9UBT4;Q9UE22;Q9UE23;Q9UQR6
A8K4M2;Q86WU8;Q9Y2M0
D3DWF6;Q8WV22;Q9P045;Q9P049
P14653;Q4VB03
P05204;Q0VGD5;Q6FGI5;Q96C64
E1P5V9;P21980;Q16436;Q6B838;Q9BTN7;Q9UH35
Q9BSD7
P06351;P33155;P84243;Q5VV55;Q5VV56;Q66I33;Q9V3W4
D0PQI2;Q502W5;Q5T5M7;Q5VTQ9;Q5VTR0;Q8N8S7;Q9NVF3;Q9UFB8
A0A0C4DFU2
O60824;O60825;Q5VVQ3;Q5VVQ4;Q9H3P1

Polito et al. Universal ready-to-use immunotherapeutic approach for the treatment of cancer: expanded and activated polyclonal γδ memory T cells.

Anova Photon

O15151;Q2M2Y2;Q32SL2;Q6GS18;Q8IV83
P01282;Q5TCY8;Q5TCY9;Q96QK3
W695;E7EQI5;H0Y4C0;O94890;Q3ZCV0;Q5JV19;Q5JV22;Q8N9P7;Q8NF91;Q8TCP1;Q8WWW6;Q8WWW7;Q8WXF6;Q96N17;Q9C0A7;Q9H525;Q9H526;Q9NS36;Q9NU50;Q9UJ06;Q9UJ07;Q9UL
B2RBS7;B3KRH1;Q5TEN1;Q5TEN2;Q99496
Q96GA3;Q96JX8
B7Z365;Q4VX89;Q4VX90;Q5T7G3;Q8NDW9;Q9UPW3;Q9Y450
Q5QD02;Q6NWS1;Q6NWS2;Q6NWS3;Q9P1P5
P80511;P83219;Q5SY66;Q7M4R1
Q5SQQ5;Q6P673;Q8WY66;Q9BS90;Q9NXX6
B2R5F0;P20670;Q6FI13
A0PJT7;A5PLR1;P02295;P02296;P16106;P68431;Q6ISV8;Q6NWP8;Q6NWP9;Q6NXU4;Q71DJ3;Q93081
B0LPE2;P08588;Q5T5Y4;Q9UKG7;Q9UKG8
O14963;Q15431;Q5VXJ6
B4DZ70;P48163;Q16797;Q16855;Q53F72;Q5VWA2;Q9BWX8;Q9H1W3;Q9UIY4
A6NGQ9;A8K2Q3;B1AKT5;B1AKT6;Q9UES9;Q9UP40;Q9Y483
A6NCR6;P51160;Q5VY29
B2RDG8;Q32WF5;Q3MV20;Q53EZ4;Q5VY28;Q6N034;Q96H32;Q9NVS7
B2R904;F2YHV0;O00633;O02679;P60484;Q6ICT7
B2R7V9;P14780;Q3LR70;Q8N725;Q9H4Z1;Q9UCJ9;Q9UCL1;Q9UDK2
Q13156;Q3SY03
A9Z1Z8;B1AMS6;Q13003;Q13004;Q16136
B2RD82;P82914;Q9H2K1
Q5SQT2;Q9P0M6
P43220;Q2M229;Q99669
B3KNE1;Q9H1F1;Q9Y5Q3
O15196;Q13285;Q5T6F5
O15294;Q7Z3K0;Q8WWM8;Q96CC1;Q9UG57
B2R9U3;E1P5T9;H0YNI0;Q9GZN2
B4DJ77;Q13077;Q658U1;Q8NF13
Q32NF2;Q5T6S3;Q5T6S4;Q6N038;Q8TBL6;Q9UFS9
A6NLF6;A8K786;P51668

Polito et al. Universal ready-to-use immunotherapeutic approach for the treatment of cancer: expanded and activated polyclonal γδ memory T cells.
Anova Photon

Q8NHZ8
B1AZX2;B1AZX3;O43189;O60929;Q5SU07;Q5SU08;Q96KM7
P28067;Q29639;Q29640
E9PAX2;Q6AHZ0;Q9NQR9
B0V033;B0V034;O43196;O60586;Q5BLU9;Q5SSR1;Q8IW44;Q9BQC7
P33552;Q6FGI9;Q6LET5
Q5T7I7;Q8TAP2;Q92529;Q9UCX5
B0UXA9;P07312;P13862;P67870;Q4VX47
Q5T8D5;Q5T8D7;Q8NCD6;Q96GX5;Q96SJ5
B0S838;P36915;Q96CT5
P35226;Q16030;Q5T8Z3;Q96F37
B2R6E8;B4DST0;O43463;Q53G60;Q6FHK6
X6R9L0
Q3L8N4;Q6KGZ9;Q9BQL1;Q9H0D6;Q9NTW0;Q9NXS6;Q9UL53
B5BUP7;P25021;Q14464;Q7Z5R9
Q96KK5
Q07011
Q17R96;Q99678
A0PJT7;A5PLR1;P02295;P02296;P16106;P68431;Q6ISV8;Q6NWP8;Q6NWP9;Q6NXU4;Q71DJ3;Q93081
O15550;Q52LL9;Q5JVQ7
B3KPY0;D3DRT8;P78345;Q53F71;Q8NHS8
E5KLB5;E5KLB6;P49916;Q16714;Q6NVK3
P80098;Q569J6
A4D0S3;E9PDA4;O15051;O15179;Q14BM2;Q92823;Q9UHI3;Q9UHI4
P26367;Q6N006;Q99413
A6NNF7;B3KSD7
P23771;Q5VWG7;Q5VWG8;Q96J16
P05161;Q5SVA4;Q7Z2G2;Q96GF0
O60489;P04156;P78446;Q15216;Q15221;Q27H91;Q5QPB4;Q8TBG0;Q96E70;Q9UP19
B2RB28;Q8NDC8;Q96NV9;Q9BRS2
Q8NGU9
O75818;Q5VX97;Q8WVK8
D3DTR2;O15296;Q8IYQ2;Q8TEV3;Q8TEV4;Q8TEV5;Q8TEV6;Q9UKM4

Anova Photon

B4DVU5;F5H1A6;Q8WVD0;Q9Y6I4
A5HKJ2;D6MXU3;Q9HB69;Q9Y244
Q5T4Z2;Q8NHY5
O00567;Q2M3T6;Q9NQ05
B4E1Y4;B7ZL46;F5H347;F5H7P0;O94877;Q2M3M0;Q5JUC9;Q5VYJ2;Q5VYJ3;Q9H3R0
A2VCT8;B4DJY4;E7EQ89;O43525
O43473;P43629;Q14946;Q16541
E9PCW9;O14580;O95350;Q9NSB9;Q9NSC0;Q9NSC1;Q9NSC5
A8K6G5;A8KAD3;Q16602;Q53S02;Q53TS5
P40259;Q53FS2;Q9BU06
Q8TAM1;Q96CW2;Q9H5D2
H0YH75;Q14980;Q14981;Q9BTE9
C5ILB7;Q14458;Q8WXG2;Q96HA2;Q9P2W1
Q562H5;Q96AP0;Q9H8F9
B4DN00;P11498;Q16705
O14518;O60484;O60618;Q2YD83;Q96FS4
B7Z492;B7Z4F1;B7ZAE4;J3KNP6;P13631;P22932;Q15281;Q52LZ8;Q9BYX8;Q9H1I3;Q9UJ38
A8K6E5;B4E3A2;B7WP05;D3DQB7;O75433;Q53FN2;Q9BS73;Q9UJX2
O15551
Q3LFQ0;Q7Z4H4
P12272;Q15251;Q6FH74
A8K1Y1;B7ZLA7;B8ZZK3;P41586;Q17S10
Q6PI73
A4KYM4;A4KYM6;D3DUR8;Q0EF62;Q0EF63;Q4VBQ6;Q96E15;Q9H3J0;Q9UNL4
B2RBU2;B4E338;E9PGJ7;Q16236;Q53RW6;Q59HH2;Q96F71
Q9UL21;Q9UQM7;Q9Y2H4;Q9Y352
P11908;Q0VDH9;Q0VDI0;Q15245;Q2TAK7
Q59FS5;Q6ZN18;Q6ZN62;Q96BG3
O60468;O60469
A5PL20;B3KMV7;B4DZF7;B7WNY4;B7WP53;E9PDU4;E9PF23;F8WD13;O94901;Q96CZ7;Q9HA14;Q9UH98
A8KAH0;O95503;Q96EM5
D3DX19

A1L190
B0QY62;O75156;Q2NKN8;Q2T9F7;Q504T5;Q6B4H1;Q7Z3E3;Q9UH99
A1BUH5;Q15858;Q6B4R9;Q6B4S0;Q6B4S1;Q70HX1;Q70HX2;Q8WTU1;Q8WWN4
Q01118
A8K3J9;D3DQR1;Q13133;Q8IW13;Q96H87
B2R799;E9PB02;P78346
B0V0Y0;P13765;Q29746;Q29825;Q6FHC2
A6NDQ1;Q9Y5Y9
A5PLL5;A6NNQ7;B4DV56;B4E1R6;Q7L2I6;Q92925;Q9UHZ1
Q96AC4;Q96GF4;Q9BU24;Q9NT16;Q9UJX3
A8K7P5;Q86XC8;Q9P0U3
P35499;Q15478;Q16447;Q7Z6B1
C9JJE7
Q07699;Q5TZZ4;Q6TN97
O77936;P28068;Q13012;Q29751;Q58ZE2;Q5SNZ8;Q5STC4;Q9XRX2
B2RDP3;E9PB21;Q9BRX5;Q9H870
A8K6X0;B2BD33;B2BD34;F5H6C8;M1P2Z0;Q2HWT5;Q3I0Y2;Q5T7I6;Q7Z434;Q86VY7;Q9H1H3;Q9H4Y1;Q9H8D3;Q9ULE9
B3KVE1;D3DQV0;P30291
B7Z818;J3KQL0;O43774;P31327;Q53TL5;Q59HF8;Q7Z5I5
B4DSC1;F5H4U9;P40424;Q5T488
F8VPA5;H0YKF2;J3KQP9;Q13972;Q16027
O75023;Q8N760
B5BU22;P17252;Q15137;Q32M72;Q96RE4
A2AAZ8;B0S834;B0S835;Q5JPL9;Q5JPM1;Q5STF8;Q5STF9;Q5STG2;Q5SU41;Q5SU42;Q86Y49;Q86Y50;Q86Y51;Q96F16;Q9H633
P49918
B2R7N4;P18509;Q52LQ0
G3XAK1
B0QYM3;Q20WK2;Q53GX5;Q5R3I6;Q6DHX4;Q9BRE0;Q9NYZ3;Q9UGZ9;Q9Y557
B1AQ58;B1AQ59;Q86X08;Q8TCN7;Q96F53;Q96JF1;Q96KH4;Q9H9B1
A2RRP9;Q5HYF0;Q6NSG2;Q8NDX5;Q8NFT7;Q8NFZ1;Q8TBM2;Q9H971;Q9H9I4
B2R6T9;B4DGT9;P57087;Q6UXG6;Q6YNC1
A8K3A7;B3KNW0;Q15119;Q6P515;Q9BS05
D3DQG0;D3DQG1;P48729;Q4JJA0;Q5U046;Q5U047;Q6FGA2;Q71TU5;Q96HD2;Q9UDK3

D3DVR9;P30533;Q2M310;Q53HQ3;Q53HS6
B2RDC8;Q96BK6;Q96K05;Q9NT96;Q9NW04;Q9UJV9
O75367;O75377;Q503A8;Q7Z5E3;Q96D41;Q9H8P3;Q9UP96
B3KV35;Q8TDU6
A0A0A6YYJ4
O60541;P43250;Q13652
Q13334;Q15743;Q4VBB4;Q6IX34
Q8N140
B2RMX8;O14727;O43297;Q7Z438;Q9BXZ6;Q9UBZ5;Q9UGN8;Q9UGN9;Q9UGP0;Q9UJ58;Q9UJ59;Q9UJ60;Q9UJ61;Q9UJ62;Q9UJ63;Q9UJ64;Q9UJ65;Q9UJ66;Q9UJ67;Q9UNC9
Q01726;Q66K38;Q6UR93;Q8WWX6;Q8WWX7;Q96I33;Q96RU4;Q9UBF7;Q9UN58;Q9UN59;Q9UN60;Q9UN61;Q9UN62
B5BU74;B5BUK4;O43585;O43586;O95657
Q14439;Q6NXF6
B4DDF1;E7ERR5;J3KN21;Q53GW2;Q8TBT4;Q96E58;Q96FJ1;Q9BXS6;Q9GZM9;Q9NZ85;Q9UI70
A2RUA9;Q3LU47;Q8NER1;Q9H0G9;Q9H303;Q9H304;Q9NQ74;Q9NY22
B4DVE6;Q658T1;Q8NEZ1;Q8WYJ6;Q96EL4;Q9H285
J3QL48
B7WP14;B7ZKQ0;P48546;Q14401;Q16400;Q52M04;Q9UPI1
O00459;Q5EAT5;Q9UPH9

Polito et al. Universal ready-to-use immunotherapeutic approach for the treatment of cancer: expanded and activated polyclonal γδ memory T cells.
Anova Photon

T: Gene name
SCTR
SNAI2
HSD17B10
SCT
MADCAM1
DMC1
NFKBIA
MMP2
CCL22
CCL17
SMPD3
GABPB1
LHB
HOXA2
ZPR1
LDHB
HLA-DOA
BYSL
BNIP1
MLH1
ECT2
PROC
ATP6V1B1
CDC7
WDR77
CD160
GHRH
MND1
IAPP
FLT3
EGR2
GPR83

MPHOSPH10
LILRB3
HOXD3
LILRA1
NCAPG
ESYT2
FKBP6
JUND
MRPL4
GINS2
ACLY
RAMP2
FSHB
RRP8
RAMP1
H3F3A
KDM6B
APCS
TEX15
TSHB
CABLES1
PHC2
RINT1
KMT2E
TFCP2
TAAR5
GPR45
RIPK1
MMP7

Polito et al. Universal ready-to-use immunotherapeutic approach for the treatment of cancer: expanded and activated polyclonal γδ memory T cells.

Anova Photon

USP22
CAB39L
ANAPC5
FAF2
RPS6KA5
SPAG9
VIPR2
PKMYT1
GLP2R
GINS1
SETD1A
SAMHD1
XRCC1
MSH4
DDX18
EED
PNO1
TAF1B
IL7
USE1
PVRL1
NOP58
SHC2
NSUN2
DNMT3A
CLDN16
MRPL3
RAB7A
GPAM
DBF4
ANLN
PDE6H
SYCP3

Polito et al. Universal ready-to-use immunotherapeutic approach for the treatment of cancer: expanded and activated polyclonal γδ memory T cells.

Anova Photon

GPR84
TINF2
NKD1
NOB1
G6PC3
CBX8
ERBB2
BCKDHA
DNMT3L
SAE1
CBLC
LILRB4
GPR32
PTH2
IL22RA1
MEIS1
PTH2R
TAAR6
CCNB3
GINS4
CRH
ADM
LIN7C
SCN2B
CTF1
TEX12
DCP1B
LDHC
NBAS
PTH
SCN3A
SCN2A
RPIA

Anova Photon

GRAP
TIAM1
KAT6B
DPYSL5
HTR6
IFNGR2
ZFYVE28
PTGIR
TFF2
PKNOX1
LILRA4
ACOX1
LSM12
WNT1
SYCE2
CLDN1
TRAT1
RPP14
SYN1
PRDM9
NSA2
HIST1H2AA
DCAF13
MELK
TSC1
SCN3B
TAF10
MC4R
LDHD
SCNN1G
TERF2IP
MRPL16

Anova Photon

RILP
TSR1
GPR25
LDHAL6B
HOXD4
PTGER4
AURKC
MAT2A
GPR27
RXFP1
HIST1H2AB
PTGDR
TLN2
SCN1A
UBE2E1
CD14
SOCS5
HOMER2
HOXC4
DRD5
IL7R
SLAMF1
RBKS
SCN11A
TADA3
GCC2
TADA2B
HOXB3
LEMD3
RND1
CBX2
SLC26A7

Polito et al. Universal ready-to-use immunotherapeutic approach for the treatment of cancer: expanded and activated polyclonal γδ memory T cells.

Anova Photon

DPYSL2
PPARD
CKS1B
RSF1
REST
PTPN2
KLK2
KLK3
PER1
TAF1D
MRPL1
DIS3L2
LILRB1
SUZ12
RANBP10
MRPS11
RPP25
MC5R
ANAPC4
BID
PRMT5
STAG3
GCSH
GHRHR
OSBPL1A
CRMP1
INSL3
PCBP4
TUFM
SCN4B
DBF4B
HOXA3
NR2F1
PAGR1

Polito et al. Universal ready-to-use immunotherapeutic approach for the treatment of cancer: expanded and activated polyclonal γδ memory T cells.

Anova Photon

IMP3
TAF1A
VIPR1
PPM1A
MYT1
HMG20B
DGKA
HOXD1
DDX24
HOXB4
SP1
IL22
LEPR
NDNL2
HDDC3
HOXB2
CALCA
RARB
HIST2H2AB
SETD8
MX2
MC2R
HOMER1
BCL9L
ARHGEF25
CLDN2
ADORA2A
KIR2DL1
ARHGAP10
FOXG1
ANAPC1

Anova Photon

FOXO3
KIR2DL4
DPYSL4
LIMK2
SSX2IP
CRHR2
PTN
TRADD
JARID2
SYCE1
SOCS4
RIOK3
KIR2DL3
CLDN4
NOXA1
HOXA1
DPYSL3
ADRB3
IL17A
SDC3
CCNG1
NFASC
EPB41
SCNN1B
CALCB
H2AFB1;H2AFB2
ARID4A
TRRAP
MLH3
GTF3C1
RASSF1

Polito et al. Universal ready-to-use immunotherapeutic approach for the treatment of cancer: expanded and activated polyclonal γδ memory T cells.

Anova Photon

GIP
USP33
MOV10
SYCP2
POT1
SYNE2
WDR5
HIST1H2AG
KCNQ2
KLRC1
HIST1H2AG
HIST1H3A
CALCR
HIST1H2AG
NPSR1
CSNK1E
CCT8L2
MCMBP
HOXA4
SCNN1A
MAP2
HTR4
FAN1
NSMCE1
HOXB1
HMGN2
TGM2
NTPCR
H3F3A
ENAH
SOD2
PFKFB2

Polito et al. Universal ready-to-use immunotherapeutic approach for the treatment of cancer: expanded and activated polyclonal γδ memory T cells.

Anova Photon

MDM4
VIP
SYNE1;SYNE2
RNF2
LTV1
HBS1L
TAAR2
S100A12
NSMCE4A
HIST2H2AA3
HIST1H3A
ADRB1
SYCP1
ME1
MTF2
PDE6C
CEP55
PTEN
MMP9
RPA4
GRIK3
MRPS15
H2AFY2
GLP1R
MAFB
NR5A1
OGT
TGIF2
TRAF1
PHF19
UBE2D1

Anova Photon

CDC26
PHF1
HLA-DMA
G6PC2
MSH5
CKS2
SHC3
CSNK2B
MASTL
GNL1
BMI1
SUV39H1
DNAJC3
XRN2
HRH2
HIST1H2AH
TNFRSF9
GPR20
HIST1H3A
KDM6A
RPP38
LIG3
CCL7
NRCAM
PAX6
SCNN1D
GATA3
ISG15
PRNP
RIOK1
GPR150
RPP40
ALOX15B

Polito et al. Universal ready-to-use immunotherapeutic approach for the treatment of cancer: expanded and activated polyclonal γδ memory T cells.
Anova Photon

USP3
POMP
HUS1B
NOP56
KDM4C
KCNQ3
KIR3DL1
HOMER3
CALCRL
CD79B
BBS10
NUMA1
PSMC3IP
ACD
PC
SIPA1
RARG
CDC23
CLDN3
ADM2
PTHLH
ADCYAP1R1
LILRA6
ING4
NFE2L2
CAMK2A
PRPS2
AEBP2
DSCAM
SUN1
CBX6
CLDN5

SYCE3
SUN2
SCN9A
SCN7A
NR1H3
RPP30
HLA-DOB
SCN10A
SMARCD2
ANAPC7
SENP1
SCN4A
MUC20
SCN1B
HLA-DMB
GINS3
MAVS
WEE1
CPS1
PBX1
RASGRF1
LILRB5
PRKCA
RPP21
CDKN1C
ADCYAP1
MST1
GTSE1
EHMT1
PHC3
JAM2
PDK2
CSNK1A1

Anova Photon

LRPAP1
DDX41
H2AFY
GPBAR1
TFF3
GRK6
GPR68
EID3
APAF1
MC1R
PSTPIP1
GPR176
NUSAP1
TRPV1
01.Sep
GIPR
PIK3R2
