# Supplementary material for: Universal Ready-to-Use Immunotherapeutic Approach for the Treatment of Cancer: Expanded and Activated Polyclonal γδ Memory T Cells
Source: Front Immunol. 2019 Nov 22;10:2717. doi: 10.3389/fimmu.2019.02717 (PMC6883509; doi:10.3389/fimmu.2019.02717)
Supplement: Supplementary file 11 [file Table_3.PDF]

Polito et al. Universal ready-to-use immunotherapeutic approach for the treatment of cancer: expanded and activated polyclonal γδ memory T cells.

KEGG Cluster Enrichment

C: Selection value	C: Category column	C: Category value	N: Total size	N: Selection size	N: Category size	N: Intersection size	N: Enrichment factor
Cluster -198	KEGG	Glycerophospholipid metabolism	5995	2	2	1	1498,8
Cluster -198	KEGG	Glycerolipid metabolism	5995	2	2	1	1498,8
Cluster -198	KEGG	Amyotrophic lateral sclerosis (ALS)	5995	2	2	1	1498,8
Cluster -198	KEGG	Alzheimer's disease	5995	2	3	1	999,17
Cluster -487	KEGG	TGF-beta signaling pathway	5995	8	1	1	749,38
Cluster -487	KEGG	Pathogenic Escherichia coli infection	5995	8	1	1	749,38
Cluster -198	KEGG	Viral myocarditis	5995	2	4	1	749,38
Cluster -486	KEGG	Sphingolipid metabolism	5995	10	1	1	599,5
Cluster -486	KEGG	Inositol phosphate metabolism	5995	10	1	1	599,5
Cluster -486	KEGG	Complement and coagulation cascades	5995	10	1	1	599,5
Cluster -487	KEGG	Cytosolic DNA-sensing pathway	5995	8	3	2	499,58
Cluster -487	KEGG	RIG-I-like receptor signaling pathway	5995	8	5	3	449,62
Cluster -487	KEGG	Toll-like receptor signaling pathway	5995	8	4	2	374,69
Cluster -489	KEGG	Pentose phosphate pathway	5995	23	3	3	260,65
Cluster -489	KEGG	NOD-like receptor signaling pathway	5995	23	2	2	260,65
Cluster -487	KEGG	Apoptosis	5995	8	6	2	249,79
Cluster -489	KEGG	Purine metabolism	5995	23	4	3	195,49
Cluster -487	KEGG	Hepatitis C	5995	8	12	3	187,34
Cluster -489	KEGG	Epithelial cell signaling in Helicobacter pylori infection	5995	23	3	2	173,77
Cluster -487	KEGG	ko05152	5995	8	10	2	149,88
Cluster -489	KEGG	Adipocytokine signaling pathway	5995	23	4	2	130,33
Cluster -489	KEGG	Chemokine signaling pathway	5995	23	10	4	104,26
Cluster -489	KEGG	Cytokine-cytokine receptor interaction	5995	23	13	4	80,201
Cluster -490	KEGG	Vibrio cholerae infection	5995	99	2	2	60,556
Cluster -490	KEGG	VEGF signaling pathway	5995	99	2	2	60,556
Cluster -490	KEGG	Pyruvate metabolism	5995	99	6	6	60,556
Cluster -490	KEGG	Propanoate metabolism	5995	99	3	3	60,556
Cluster -490	KEGG	PPAR signaling pathway	5995	99	3	3	60,556
Cluster -490	KEGG	Pancreatic cancer	5995	99	2	2	60,556
Cluster -490	KEGG	Citrate cycle (TCA cycle)	5995	99	2	2	60,556
Cluster -490	KEGG	Base excision repair	5995	99	2	2	60,556

KEGG Cluster Enrichment

Cluster -491	KEGG	Neuroactive ligand-receptor interaction	5995	105	44	43	55,798
Cluster -490	KEGG	Ribosome biogenesis in eukaryotes	5995	99	12	11	55,509
Cluster -490	KEGG	RNA transport	5995	99	7	6	51,905
Cluster -491	KEGG	Vascular smooth muscle contraction	5995	105	8	7	49,958
Cluster -491	KEGG	Endocytosis	5995	105	5	4	45,676
Cluster -490	KEGG	Glycolysis / Gluconeogenesis	5995	99	4	3	45,417
Cluster -490	KEGG	Bladder cancer	5995	99	4	3	45,417
Cluster -490	KEGG	Non-small cell lung cancer	5995	99	6	4	40,37
Cluster -490	KEGG	mTOR signaling pathway	5995	99	3	2	40,37
Cluster -490	KEGG	Hedgehog signaling pathway	5995	99	3	2	40,37
Cluster -490	KEGG	Fc gamma R-mediated phagocytosis	5995	99	3	2	40,37
Cluster -490	KEGG	Chagas disease (American trypanosomiasis)	5995	99	3	2	40,37
Cluster -490	KEGG	Acute myeloid leukemia	5995	99	3	2	40,37
Cluster -491	KEGG	Calcium signaling pathway	5995	105	10	7	39,967
Cluster -491	KEGG	Salivary secretion	5995	105	3	2	38,063
Cluster -491	KEGG	Pancreatic secretion	5995	105	3	2	38,063
Cluster -491	KEGG	Tight junction	5995	105	11	7	36,333
Cluster -490	KEGG	Endometrial cancer	5995	99	5	3	36,333
Cluster -491	KEGG	Antigen processing and presentation	5995	105	8	5	35,685
Cluster -489	KEGG	Cell adhesion molecules (CAMs)	5995	23	15	2	34,754
Cluster -491	KEGG	Osteoclast differentiation	5995	105	10	6	34,257
Cluster -490	KEGG	Wnt signaling pathway	5995	99	9	5	33,642
Cluster -490	KEGG	Phosphatidylinositol signaling system	5995	99	4	2	30,278
Cluster -490	KEGG	Cysteine and methionine metabolism	5995	99	6	3	30,278
Cluster -490	KEGG	Axon guidance	5995	99	4	2	30,278
Cluster -490	KEGG	Amoebiasis	5995	99	4	2	30,278
Cluster -491	KEGG	Melanogenesis	5995	105	4	2	28,548
Cluster -491	KEGG	Leukocyte transendothelial migration	5995	105	12	6	28,548
Cluster -491	KEGG	Hepatitis C	5995	105	12	6	28,548
Cluster -491	KEGG	GnRH signaling pathway	5995	105	4	2	28,548
Cluster -488	KEGG	Type I diabetes mellitus	5995	219	3	3	27,374
Cluster -488	KEGG	Taste transduction	5995	219	3	3	27,374

KEGG Cluster Enrichment

Cluster -488	KEGG	Systemic lupus erythematosus	5995	219	19	19	27,374
Cluster -488	KEGG	Staphylococcus aureus infection	5995	219	3	3	27,374
Cluster -488	KEGG	SNARE interactions in vesicular transport	5995	219	2	2	27,374
Cluster -488	KEGG	Oocyte meiosis	5995	219	9	9	27,374
Cluster -488	KEGG	Mismatch repair	5995	219	3	3	27,374
Cluster -488	KEGG	Meiosis - yeast	5995	219	8	8	27,374
Cluster -488	KEGG	Graft-versus-host disease	5995	219	3	3	27,374
Cluster -488	KEGG	Cell cycle - yeast	5995	219	10	10	27,374
Cluster -488	KEGG	Cell cycle	5995	219	12	12	27,374
Cluster -488	KEGG	Asthma	5995	219	3	3	27,374
Cluster -488	KEGG	Allograft rejection	5995	219	3	3	27,374
Cluster -491	KEGG	Cell adhesion molecules (CAMs)	5995	105	15	7	26,644
Cluster -491	KEGG	Natural killer cell mediated cytotoxicity	5995	105	11	5	25,952
Cluster -490	KEGG	ErbB signaling pathway	5995	99	7	3	25,952
Cluster -488	KEGG	Progesterone-mediated oocyte maturation	5995	219	9	8	24,333
Cluster -490	KEGG	Focal adhesion	5995	99	8	3	22,708
Cluster -488	KEGG	Ubiquitin mediated proteolysis	5995	219	10	8	21,9
Cluster -488	KEGG	Rheumatoid arthritis	5995	219	5	4	21,9
Cluster -488	KEGG	Adherens junction	5995	219	5	4	21,9
Cluster -488	KEGG	Viral myocarditis	5995	219	4	3	20,531
Cluster -488	KEGG	Intestinal immune network for IgA production	5995	219	4	3	20,531
Cluster -488	KEGG	Hematopoietic cell lineage	5995	219	4	3	20,531
Cluster -488	KEGG	Autoimmune thyroid disease	5995	219	4	3	20,531
Cluster -490	KEGG	Pathways in cancer	5995	99	18	6	20,185
Cluster -490	KEGG	Leukocyte transendothelial migration	5995	99	12	4	20,185
Cluster -488	KEGG	Jak-STAT signaling pathway	5995	219	11	8	19,909
Cluster -488	KEGG	Phagosome	5995	219	7	5	19,553
Cluster -490	KEGG	Osteoclast differentiation	5995	99	10	3	18,167
Cluster -490	KEGG	Chemokine signaling pathway	5995	99	10	3	18,167
Cluster -488	KEGG	Cytokine-cytokine receptor interaction	5995	219	13	8	16,846
Cluster -490	KEGG	Natural killer cell mediated cytotoxicity	5995	99	11	3	16,515
Cluster -488	KEGG	Leishmaniasis	5995	219	5	3	16,425

KEGG Cluster Enrichment

Cluster -488	KEGG	Aldosterone-regulated sodium reabsorption	5995	219	5	3	16,425
Cluster -488	KEGG	Toxoplasmosis	5995	219	6	3	13,687
Cluster -488	KEGG	Neurotrophin signaling pathway	5995	219	6	3	13,687
Cluster -488	KEGG	ko05152	5995	219	10	5	13,687
Cluster -488	KEGG	Glioma	5995	219	6	3	13,687
Cluster -488	KEGG	Cysteine and methionine metabolism	5995	219	6	3	13,687
Cluster -488	KEGG	Bacterial invasion of epithelial cells	5995	219	6	3	13,687
Cluster -488	KEGG	Insulin signaling pathway	5995	219	7	3	11,732
Cluster -488	KEGG	ErbB signaling pathway	5995	219	7	3	11,732
Cluster -488	KEGG	Pathways in cancer	5995	219	18	5	7,604
Cluster -488	KEGG	Cell adhesion molecules (CAMs)	5995	219	15	4	7,2998

Polito et al. Universal ready-to-use immunotherapeutic approach for the treatment of cancer: expanded and activated polyclonal γδ memory T cells.

KEGG Cluster Enrichment

N: P value	N: -log10(Benj. Hoch. FDR)
0,00066711	2,0158
0,00066711	2,0158
0,00066711	2,0158
0,0010005	1,90752
0,0013344	1,80134
0,0013344	1,80134
0,0013338	1,80134
0,0016681	1,74834
0,0016681	1,74834
0,0016681	1,74834
4,6706E-06	3,84043
1,5576E-08	6,0398
9,3318E-06	3,57461
4,9342E-08	5,62202
0,000014081	3,41764
0,000023283	3,22992
1,9671E-07	5,09104
3,4068E-07	4,88356
0,000042096	3,00369
0,000069568	2,85146
0,000083897	2,78563
3,3933E-08	5,76531
1,1444E-07	5,29288
0,00026999	2,35466
0,00026999	2,35466
1,7422E-11	8,66663
0,00000437	3,85708
0,00000437	3,85708
0,00026999	2,35466
0,00026999	2,35466
0,00026999	2,35466

KEGG Cluster Enrichment

6,2464E-79	75,1579
1,6701E-19	16,2079
1,2006E-10	7,99319
3,2549E-12	9,34406
4,3704E-07	4,79011
0,0000172	3,35144
0,0000172	3,35144
1,0172E-06	4,45127
0,00079687	1,96593
0,00079687	1,96593
0,00079687	1,96593
0,00079687	1,96593
0,00079687	1,96593
4,7238E-11	8,32061
0,000896	1,95059
0,000896	1,95059
1,2778E-10	7,99319
0,000042311	3,00369
7,9801E-08	5,4317
0,0014125	1,78123
4,9157E-09	6,51718
1,3131E-07	5,25019
0,0015679	1,75835
0,000083266	2,78563
0,0015679	1,75835
0,0015679	1,75835
0,0017612	1,73299
2,092E-08	5,95519
2,092E-08	5,95519
0,0017612	1,73299
0,000048107	3,00369
0,000048107	3,00369

KEGG Cluster Enrichment

2,2582E-28	24,9008
0,000048107	3,00369
0,0013286	1,80134
9,8667E-14	10,7375
0,000048107	3,00369
2,7996E-12	9,3515
0,000048107	3,00369
3,4614E-15	12,1132
4,2015E-18	14,9321
0,000048107	3,00369
0,000048107	3,00369
2,3324E-09	6,78982
6,2591E-07	4,64836
0,00014338	2,56045
2,4308E-11	8,56775
0,00022573	2,39926
1,1726E-10	7,99319
8,3597E-06	3,6111
8,3597E-06	3,6111
0,00018549	2,47757
0,00018549	2,47757
0,00018549	2,47757
0,00018549	2,47757
0,000000268	4,97253
0,000030494	3,1225
4,1479E-10	7,51177
1,2152E-06	4,38738
0,00046832	2,14629
0,00046832	2,14629
3,0112E-09	6,70523
0,00063361	2,02092
0,00044701	2,15444

KEGG Cluster Enrichment

0,00044701	2,15444
0,00086178	1,95762
0,00086178	1,95762
0,000013074	3,43916
0,00086178	1,95762
0,00086178	1,95762
0,00086178	1,95762
0,0014537	1,7778
0,0014537	1,7778
0,00033213	2,27105
0,001583	1,75835
