# Supplementary material for: Universal Ready-to-Use Immunotherapeutic Approach for the Treatment of Cancer: Expanded and Activated Polyclonal γδ Memory T Cells
Source: Front Immunol. 2019 Nov 22;10:2717. doi: 10.3389/fimmu.2019.02717 (PMC6883509; doi:10.3389/fimmu.2019.02717)
Supplement: Supplementary file 12 [file Table_4.PDF]

Polito et al. Universal ready-to-use immunotherapeutic approach for the treatment of cancer: expanded and activated polyclonal γδ memory T cells.

GD kinome

GD1_t0	GD2_t0	GD3_t0	GD1_Dp18	GD3_Dp18	GD4_Dp18
-1,95672	-1,97583	-2,56157	-2,83697	-1,76756	-2,3601
-3,40634	-2,78361	-2,38568	0,133181	0,481522	0,190094
0,345713	0,0691204	0,231829	-0,88875	-1,06049	-1,03262
-2,48815	-1,72712	-2,43457	-1,47883	-1,71416	-1,70749
0,433287	0,475772	0,461868	0,223625	-0,256229	-0,580853
1,26505	-0,325258	1,1857	1,23472	1,10123	1,1579
-0,359681	0,272857	-0,317739	-1,8803	-2,2761	-2,17684
-1,60501	-2,26264	-1,6488	-2,34201	-1,88847	-1,63393
-0,198915	0,699953	0,102732	-1,82113	1,14801	0,412964
-1,33252	-2,11143	-2,11707	-2,4156	-0,316551	-2,51165
-2,6329	-2,87713	-2,53325	-1,66534	0,423632	-1,12163
-1,67767	-1,91097	-1,72257	-0,776028	-0,942186	-1,30128
-0,793041	-0,326836	2,25735	3,52405	0,467815	3,61051
-0,203329	0,603476	-0,0947456	-2,64438	1,73116	-2,34786
-0,0742397	0,20165	0,628321	0,362403	1,92646	1,15786
0,920206	1,31618	0,95465	0,474524	2,41329	1,0108
-0,327946	-0,836176	-0,984877	-0,333448	0,345631	-0,000530243
-1,63965	-1,47849	-0,812374	-3,73621	-1,53854	-2,01459
-1,90437	0,543037	-3,58772	0,173553	0,143665	-1,19069
0,302938	-1,37327	0,457721	1,50802	2,7596	2,37419
-2,33262	-2,66674	-2,36167	-0,673317	-0,528172	-2,88769
0,825207	0,639202	0,436966	-0,46806	-3,5878	-2,16894
-2,0807	-2,33917	-3,17922	-0,712814	-1,47555	-0,593874
-0,562964	-1,09659	-1,32198	-1,2779	-1,70215	-2,37971
-2,50043	-2,67268	-2,92905	0,755316	-0,129444	0,960176
2,37469	2,13627	2,66827	1,97637	3,06932	-2,90693
0,104698	-0,127146	-0,066906	0,123833	1,56353	-0,0596542
-0,536007	-0,892318	-0,961264	-1,55819	-1,84484	-1,88366
-1,07974	-1,1727	-1,10356	-2,93276	-2,33655	-2,46425
0,0762329	-0,0783863	0,23946	0,441769	1,95009	1,70618
0,979961	1,88991	0,974319	-1,80871	-1,53319	-2,47028

Polito et al. Universal ready-to-use immunotherapeutic approach for the treatment of cancer: expanded and activated polyclonal γδ memory T cells.
GD kinome

-1,98447	-1,71157	-1,49789	-3,51575	-1,74701	-1,86346
-1,85638	-1,83268	-2,41811	-1,78764	-1,6464	-2,17932
-2,62678	-3,32808	-2,00716	-1,99198	-0,619404	-0,0434351
-0,203876	-0,49855	-0,744397	-0,486697	0,693866	-0,979636
-3,25734	-2,35562	-2,33148	-0,0487232	0,273838	0,049715
-2,63533	-2,95636	-3,05562	1,044	2,34023	0,953621
-1,47259	-1,88372	-1,63698	1,0389	2,10707	0,192678
2,31935	1,73525	1,93353	0,331392	1,77428	-0,200485
0,960234	0,534277	0,890671	-0,238598	-0,309356	-0,897202
0,00101566	0,566358	-0,0376358	-0,998407	0,32731	-0,0233345
-0,952869	-0,417017	-1,34025	-2,75344	1,12029	-1,07326
1,41514	1,65342	1,68342	1,68602	-0,0511503	0,924093
-0,413713	-0,250408	-0,310472	-2,47443	-2,2822	-1,55128
-0,641714	-0,920549	-1,07847	-1,60188	-1,1584	-3,1151
-2,86627	-2,42961	-3,10618	1,5817	-0,747541	0,185991
-0,826447	-0,746519	0,05159	-0,238466	-1,44405	0,878408
2,52262	2,30569	2,2759	4,0811	2,78695	2,55397
0,719061	0,0669079	0,719496	-2,90202	-2,4486	-0,121881
-2,60173	-1,79706	-2,92465	-2,26226	-0,761044	0,619125
1,60498	1,05657	1,00628	2,23869	2,51631	2,29364
1,37443	1,99807	1,2555	-2,98395	0,676506	-2,03088
-1,27807	-1,47996	-1,26188	0,0874863	-2,68053	-0,0381203
4,01855	3,88152	4,9234	5,52162	2,63809	4,26157
1,91401	1,63815	2,02421	1,99096	2,22667	0,534737
-0,468605	-0,880106	-0,930096	0,364481	1,01702	-1,27738
2,84068	2,70852	2,6832	3,11324	4,16494	2,87165
4,71814	4,33073	4,07125	3,85327	5,13364	4,74259
1,19206	1,46705	1,51201	1,43456	-1,36558	1,02463
0,662105	0,418766	0,136325	0,262453	-2,10969	-1,79889
-2,30537	-2,75492	-2,99028	-0,111481	0,314941	-0,864954
-0,0847645	0,247506	0,160209	-2,29904	-0,182081	-1,79803
-1,55818	-1,42741	-0,872404	-1,8536	-2,49168	-2,38008

Polito et al. Universal ready-to-use immunotherapeutic approach for the treatment of cancer: expanded and activated polyclonal γδ memory T cells.
GD kinome

-1,33544	-2,76699	-2,60991	1,45611	1,75848	0,603437
1,86077	1,39173	1,21184	0,633892	-1,5806	-0,242022
-1,2105	-1,42827	-1,26079	-2,65633	-2,16196	-2,31369
-2,34292	-2,75184	-1,61966	-1,41492	-1,56362	0,372755
2,37008	1,84958	1,49265	1,57652	-0,521069	-1,49045
-0,758039	-0,4356	-1,08544	-0,612726	-2,037	-2,31371
0,517063	0,702908	0,271818	-0,317471	-2,09101	-0,110636
1,28905	1,29162	0,831982	0,0155153	2,8792	1,22501
-0,307081	-0,869528	0,331675	-0,625782	0,108915	-1,19327
-2,24596	-3,0186	-2,50476	3,54259	2,94813	2,94038
-3,06765	-2,83377	-2,59296	3,54259	2,94813	2,78898
0,386173	-0,305801	0,451565	-0,0345402	1,33784	0,877993
0,639292	0,703297	0,253252	1,38224	-2,75701	-1,72887

Polito et al. Universal ready-to-use immunotherapeutic approach for the treatment of cancer: expanded and activated polyclonal γδ memory T cells.
GD kinome

C: KEGG name
Bacterial invasion of epithelial cells;Endometrial cancer;Focal adhesion;PPAR signaling pathway
Axon guidance;Chemokine signaling pathway;Epithelial cell signaling in Helicobacter pylori infection;ErbB signaling pathway;Fc gamma R-mediated phagocytosis;Focal adhesion;MAPK signalin
GnRH signaling pathway;MAPK signaling pathway
Adipocytokine signaling pathway
Adherens junction;Leishmaniasis;MAPK signaling pathway;Measles;NOD-like receptor signaling pathway;Osteoclast differentiation;RIG-I-like receptor signaling pathway;T cell receptor signalin
ko05152;Neurotrophin signaling pathway;NOD-like receptor signaling pathway;Shigellosis
mTOR signaling pathway;Regulation of autophagy
Axon guidance;ErbB signaling pathway;Focal adhesion;Regulation of actin cytoskeleton;Renal cell carcinoma;T cell receptor signaling pathway
Axon guidance;Cell cycle;Chronic myeloid leukemia;ErbB signaling pathway;Neurotrophin signaling pathway;Pathogenic Escherichia coli infection;Pathways in cancer;Shigellosis;Viral myocardi
Acute myeloid leukemia;B cell receptor signaling pathway;Bladder cancer;Chemokine signaling pathway;Chronic myeloid leukemia;Colorectal cancer;Endometrial cancer;ErbB signaling pathwa
Acute myeloid leukemia;Bladder cancer;Chemokine signaling pathway;Chronic myeloid leukemia;Colorectal cancer;Endometrial cancer;ErbB signaling pathway;Focal adhesion;Glioma;Hepatiti
Acute myeloid leukemia;Adipocytokine signaling pathway;Apoptosis;B cell receptor signaling pathway;Carbohydrate digestion and absorption;Chagas disease (American trypanosomiasis);Che
B cell receptor signaling pathway;Fc epsilon RI signaling pathway;Fc gamma R-mediated phagocytosis;ko05152;Natural killer cell mediated cytotoxicity;Osteoclast differentiation
Amyotrophic lateral sclerosis (ALS);Fc epsilon RI signaling pathway;GnRH signaling pathway;MAPK signaling pathway;Osteoclast differentiation;Toll-like receptor signaling pathway;Toxoplasmo
GnRH signaling pathway;MAPK signaling pathway;Neurotrophin signaling pathway;RIG-I-like receptor signaling pathway;Ubiquitin mediated proteolysis
Axon guidance;Chemokine signaling pathway;Focal adhesion;Leukocyte transendothelial migration;Pathogenic Escherichia coli infection;Regulation of actin cytoskeleton;Shigellosis;TGF-beta s
Cell cycle;p53 signaling pathway
Apoptosis;Cytosolic DNA-sensing pathway;Hepatitis C;RIG-I-like receptor signaling pathway;Toll-like receptor signaling pathway

Ribosome biogenesis in eukaryotes
MAPK signaling pathway;Tight junction
Bladder cancer;Pathways in cancer
Gap junction;GnRH signaling pathway;MAPK signaling pathway
Cytosolic DNA-sensing pathway
Calcium signaling pathway;Insulin signaling pathway
Acute myeloid leukemia;Adipocytokine signaling pathway;Apoptosis;B cell receptor signaling pathway;Carbohydrate digestion and absorption;Chagas disease (American trypanosomiasis);Che
Amoebiasis;Apoptosis;Bile secretion;Calcium signaling pathway;Chemokine signaling pathway;Dilated cardiomyopathy;Endocrine and other factor-regulated calcium reabsorption;Gap junctio
Alzheimer's disease;Axon guidance
Axon guidance;ErbB signaling pathway;Focal adhesion;MAPK signaling pathway;Regulation of actin cytoskeleton;Renal cell carcinoma;T cell receptor signaling pathway
Apoptosis;Cell cycle;p53 signaling pathway
Calcium signaling pathway;ErbB signaling pathway;Gastric acid secretion;Glioma;GnRH signaling pathway;ko05152;Long-term potentiation;Melanogenesis;Neurotrophin signaling pathway;Olf
Adherens junction;Axon guidance;Bacterial invasion of epithelial cells;Endocytosis;Epithelial cell signaling in Helicobacter pylori infection;ErbB signaling pathway;Fc epsilon RI signaling pathwa
Alzheimer's disease;Axon guidance;B cell receptor signaling pathway;Basal cell carcinoma;Cell cycle;Chemokine signaling pathway;Circadian rhythm - fly;Colorectal cancer;Endometrial cancer;
B cell receptor signaling pathway;Chemokine signaling pathway;Epithelial cell signaling in Helicobacter pylori infection;Fc epsilon RI signaling pathway;Fc gamma R-mediated phagocytosis;Lon

Chemokine signaling pathway;Jak-STAT signaling pathway;ko05152;Measles
Calcium signaling pathway;Long-term potentiation;Neurotrophin signaling pathway;Osteoclast differentiation
Amyotrophic lateral sclerosis (ALS);MAPK signaling pathway;Neurotrophin signaling pathway;Protein processing in endoplasmic reticulum
GnRH signaling pathway;MAPK signaling pathway;Neurotrophin signaling pathway
Oocyte meiosis
Basal transcription factors;Cell cycle;Nucleotide excision repair
Cell cycle;Measles;Oocyte meiosis;p53 signaling pathway;Pathways in cancer;Progesterone-mediated oocyte maturation;Prostate cancer;Small cell lung cancer
Cell cycle;Gap junction;Oocyte meiosis;p53 signaling pathway;Progesterone-mediated oocyte maturation

Polito et al. Universal ready-to-use immunotherapeutic approach for the treatment of cancer: expanded and activated polyclonal γδ memory T cells.
GD kinome

C: Student's T-test Significant GD_Dp18_GD_t0	N: Localization prob	T: Protein
	1	A0A0A0MTH3
+	0,999995	B1AVT0
+	0,999575	B3KNX7
+	0,975569	E7EX48
+	0,999999	F2Z2U4
	0,999152	J3KNB8
+	1	Q96RR4-5
	0,99996	J3QT34
+	1	O43318
	0,999999	O43353-2
+	0,909668	O75385
+	0,99981	O95835
	1	O96013
itis	1	P00519
+	0,995502	P04049;P10398
is C;Insulin signaling pathway;Long-term depressi	0,997814	P15056
mokine signaling pathway;Chronic myeloid leuke	0,998899	P31749
	0,999171	P43405
osis	1	P46734
	0,99943	P49761-1
	0,990106	Q13233
+	1	Q13464
+	0,936592	Q13535-2
+	0,999786	Q13546
+	1	Q14680-5
	1	Q15059
	0,997317	Q16513-2
	0,999695	Q6P0Q8
+	0,99307	Q86YV5
	0,999986	Q8IVT5-2
+	0,999531	Q8TD19

	0,999785	Q8TEA7-3
	0,995664	Q96BR1-2
	0,946334	Q96GX5-2
	0,999986	Q96PY6-4
+	0,997965	Q9BVS4-2
+	0,999938	Q9NYL2
+	0,999993	Q9UIK4
+	0,999999	Q9UKE5-6
	0,876333	Q9UKI8-4
	0,999994	Q9Y2U5
	0,998744	Q9Y572
	1	A0A0A0MT23;E7ETY4
+	0,999998	H3BLV9
	1	J3KNN3
+	0,998232	P31751
	0,999985	P50750
n;Gastric acid secretion;GnRH signaling pathway;	0,999981	P51817
+	1	P61160
	0,999561	Q00535
+	1	Q13177
+	0,999957	Q13315
factory transduction;Oocyte meiosis;Phototransd	0,99503	Q13557-8
	1	Q16512-2
	0,999999	Q9UHY1
+	1	P06239;P06241-2
ErbB signaling pathway;Focal adhesion;Hedgeho	0,986567	P49840
	0,999987	Q13627-2
	0,999786	H0Y6K2
+	0,999995	F5GWT4
+	0,999976	O00418
+	0,996036	O60307
+	1	P07948-2

+	0,998356	P52333
+	1	Q16566
+	0,992748	Q86UE8-2
	0,999733	Q96KB5
	1	Q99683
+	0,999997	Q99759
+	0,999992	Q9H2G2-2
+	1	P50613
+	0,999999	A0A096LP25;Q2M2I8
+	1	
+	1	P06493
+	0,933365	O60885
	0,82313	Q8TDX7

Polito et al. Universal ready-to-use immunotherapeutic approach for the treatment of cancer: expanded and activated polyclonal γδ memory T cells.
GD kinome

T: Protein names
Integrin-linked protein kinase
Dual specificity protein kinase CLK2
Non-specific serine/threonine protein kinase;Serine/threonine-protein kinase PAK 1
Serine/threonine-protein kinase Nek4
Transformation/transcription domain-associated protein
Mitogen-activated protein kinase kinase kinase 4
Calcium/calmodulin-dependent protein kinase kinase 1;Calcium/calmodulin-dependent protein kinase kinase 2
Microtubule-associated serine/threonine-protein kinase 4
Mitogen-activated protein kinase kinase kinase 7
Receptor-interacting serine/threonine-protein kinase 2
Serine/threonine-protein kinase ULK1
Serine/threonine-protein kinase LATS1
Serine/threonine-protein kinase PAK 4
Tyrosine-protein kinase ABL1
RAF proto-oncogene serine/threonine-protein kinase;Serine/threonine-protein kinase A-Raf
Serine/threonine-protein kinase B-raf
RAC-alpha serine/threonine-protein kinase
Tyrosine-protein kinase SYK
Dual specificity mitogen-activated protein kinase kinase 3;Dual specificity mitogen-activated protein kinase kinase 6
Dual specificity protein kinase CLK3
Mitogen-activated protein kinase kinase kinase 1
Rho-associated protein kinase 1
Serine/threonine-protein kinase ATR
Receptor-interacting serine/threonine-protein kinase 1
Maternal embryonic leucine zipper kinase
Bromodomain-containing protein 3
Serine/threonine-protein kinase N2
Microtubule-associated serine/threonine-protein kinase 2
Tyrosine-protein kinase SgK223
Kinase suppressor of Ras 1
Serine/threonine-protein kinase Nek9

TBC domain-containing protein kinase-like protein
Serine/threonine-protein kinase Sgk3
Serine/threonine-protein kinase greatwall
Serine/threonine-protein kinase Nek1
Serine/threonine-protein kinase RIO2
Mitogen-activated protein kinase kinase kinase MLT
Death-associated protein kinase 2
TRAF2 and NCK-interacting protein kinase
Serine/threonine-protein kinase tousled-like 1
Mitogen-activated protein kinase kinase kinase 2
Receptor-interacting serine/threonine-protein kinase 3
MAP/microtubule affinity-regulating kinase 3;MAP/microtubule affinity-regulating kinase 4;Serine/threonine-protein kinase MARK1;Serine/threonine-protein kinase MARK2
SRSF protein kinase 3;SRSF protein kinase 1;SRSF protein kinase 2;SRSF protein kinase 2 N-terminal;SRSF protein kinase 2 C-terminal
Phosphorylase b kinase gamma catalytic chain, liver/testis isoform
RAC-beta serine/threonine-protein kinase
Cyclin-dependent kinase 9
cAMP-dependent protein kinase catalytic subunit PRKX;Putative serine/threonine-protein kinase PRKY
Actin-related protein 2
Cyclin-dependent-like kinase 5
PAK-2p27;PAK-2p34;Serine/threonine-protein kinase PAK 2
Serine-protein kinase ATM
Calcium/calmodulin-dependent protein kinase type II subunit delta
Serine/threonine-protein kinase N1
Nuclear receptor-binding protein
Non-specific protein-tyrosine kinase;Proto-oncogene tyrosine-protein kinase Src;Tyrosine-protein kinase Fyn;Tyrosine-protein kinase Lck;Tyrosine-protein kinase Yes
Glycogen synthase kinase-3 alpha;Glycogen synthase kinase-3 beta
Dual specificity tyrosine-phosphorylation-regulated kinase 1A;Dual specificity tyrosine-phosphorylation-regulated kinase 1B
Bromodomain-containing protein 2
Serine/threonine-protein kinase WNK1
Eukaryotic elongation factor 2 kinase
Microtubule-associated serine/threonine-protein kinase 3
Tyrosine-protein kinase Lyn

Polito et al. Universal ready-to-use immunotherapeutic approach for the treatment of cancer: expanded and activated polyclonal γδ memory T cells.
GD kinome

Tyrosine-protein kinase JAK3
Calcium/calmodulin-dependent protein kinase type IV
Serine/threonine-protein kinase tousled-like 2
Lymphokine-activated killer T-cell-originated protein kinase
Mitogen-activated protein kinase kinase kinase 5
Mitogen-activated protein kinase kinase kinase 3
STE20-like serine/threonine-protein kinase
Cyclin-dependent kinase 7
AP2-associated protein kinase 1;Uncharacterized protein FLJ45252
Cyclin-dependent kinase 2;Cyclin-dependent kinase 3
Cyclin-dependent kinase 1
Bromodomain-containing protein 4
Serine/threonine-protein kinase Nek6;Serine/threonine-protein kinase Nek7

Polito et al. Universal ready-to-use immunotherapeutic approach for the treatment of cancer: expanded and activated polyclonal γδ memory T cells.
GD kinome

T: Gene names
ILK
CLK2
PAK1
NEK4
TRRAP
MAP3K4
CAMKK1;CAMKK2
MAST4
DKFZp586F0420;MAP3K7
RIPK2
ULK1
LATS1
PAK4
ABL1
ARAF;RAF1
BRAF
AKT1
SYK
MAP2K3;MAP2K6
CLK3
MAP3K1
ROCK1
ATR
RIPK1
MELK
BRD3
PKN2
MAST2
SGK223
KSR1
NEK9

Polito et al. Universal ready-to-use immunotherapeutic approach for the treatment of cancer: expanded and activated polyclonal γδ memory T cells.

GD kinome

TBCK
SGK3
MASTL
NEK1
RIOK2
ZAK
DAPK2
TNIK
TLK1
MAP3K2
RIPK3
MARK1;MARK2;MARK3;MARK4
SRPK3;SRPK1;SRPK2
PHKG2
AKT2
CDK9
PRKX;PRKY
ACTR2
CDK5
PAK2
ATM
CAMK2D
PKN1
NRBP1
FYN;LCK;SRC;YES1
GSK3A;GSK3B
DYRK1A;DYRK1B
BRD2
WNK1
EEF2K
MAST3
LYN

Polito et al. Universal ready-to-use immunotherapeutic approach for the treatment of cancer: expanded and activated polyclonal γδ memory T cells.

GD kinome

JAK3
CAMK4
TLK2
PBK
MAP3K5
MAP3K3
SLK
CDK7
AAK1
CDK2;CDK3
CDC2;CDK1
BRD4
NEK6;NEK7

Polito et al. Universal ready-to-use immunotherapeutic approach for the treatment of cancer: expanded and activated polyclonal γδ memory T cells.
GD kinome

T: Sequence window
GTTRTRPRNGTLNKHSGIDFKQLNFLTKLNE
WSSSSDRTRRRRREDSYHVRSRSYDDRSSDR
EFYNSKKTSNSQKYMSFTDKSAEDYNSSNAL;LNVKAVSETPAVPPVSEDEDDDDDDATPPPV;PLPVTPTRDVATSPISPTENNTTPPDALTRN
GEKRQVRRDLFAFQESPPRFLPSHPIVGKVD;SQEEMSSSGPSVRKASLSVAGPGKPQEEDQP
GEGVNSVSSSIKRGLSVDSAQEVKRFRTATG;RTATGAISAVFGRSQSLPGADSLLAKPIDKQ
IQSRDCISKKLERLESEDDSLGWGAPDWSTE;SIAAELQFRSLSRHSSPTEERDEPAYPRGDS
SRLLPARPSLSARKLSLQERPAGSYLEAQAG;TSGSQARPHLSGRKLSLQERSQGGLAAGGSL;VPEIKILVKTMIRKRSFGNPFEGSRREERSL
APKLGGQRYRSGRRKSAGNIPLSPLARTPSP;AQPAGEGRTHMTKSDSLPSFRVSTLPLESHH
ATTGNGQPRRRSIQDLTVTGTEPGQVS;SSVESLPPTSEGKRMSADMSEIEARIAATTA;SSVESLPPTSEGKRMSADMSEIEARIAATTG;TTAYSKPKRGHRKTASFGNILDVPEIVISGN;VPEIVISGNGQPRRRSIQDLTVTGTEP
GLQPYPEILVVSRSPSLNLLQNKSM______
LPDFLQRNPLPPILGSPTKAVPSFDFPKTPS
ENVDSGDKEKKQITTSPITVRKNKKDEERRE;GNVQQSVNRKQSWKGSKESLVPQRHGPPLGE;HEIPTWQPNIPVRSNSFNNPLGNRASHSANS
FGFCAQVSKEVPRRKSLVGTPYWMAPELISR
KGQGESDPLDHEPAVSPLLPRKERGPPEGGL
SALSSSPNNLSPTGWSQPKTPVPAQR;IELLQHSLPKINRSASEPSLHRAAHTEDINA;IELLQRSLPKIERSASEPSLHRTQADELPAC;SPSSEGSLSQRQRSTSTPNVHMVSTTLPVDS;TVCVDMSTNRQQFYHSVQDLSGGSRQ
DEDHRNQFGQRDRSSSAPNVHINTIEPVNID;IELLARSLPKIHRSASEPSLNRAGFQTEDFS;SEDRNRMKTLGRRDSSDDWEIPDGQITVGQR;SSEDRNRMKTLGRRDSSDDWEIPDGQITVGQ
GLKKQEEEEMDFRSGSPSDNSGAEEMEVSLA
ATWSAGGIISRIKSYSFPKPGHRKSSPAQGN
HVKMCDFGISGYLVDSVAKTMDAGCKPYMAP;QVKMCDFGISGYLVDSVAKTIDAGCKPYMAP
________MHHCKRYRSPEPDPYLSYRWKRRR;HDRLPYQRRYRERRDSDTYRCEERSPSFGED
GNRASSSGFPGARATSPEAGGGGGALKASSA;LEKTGKGLCATKLSASSEDISERLASISVGP
RASPRTLSTRSTANQSFRKVVKNTSGKTS__
LSSNSDGISPKRRRLSSSLNPSKRAPKQTED
KKEYSNENAVVKRMQSLQLDCVAVPSSRSNS;LQSKLQDEANYHLYGSRMDRQTKQQPRQNVA
HMEETPKRKGAKVFGSLERGLDKVITVLTRS
TTTPTTSAITASRSESPPPLSDPKQAKVVAR
FDLEPEPPPAPPRASSLGEIDESSELRVLDI
EASNLVRMRNQSLGQSAPSLTAGLKELSLPR;QDVVTGVSPLLFRKLSNPDIFSSTGKVKLQR
QPPPLPQKKIVSRAASSPDGFFWTQGSPKPG;RIEEEEEVEQELLSHSWGRETKNGPTDHSNS;SGQNSKVGTGMSKSASFAFEFPKDRSGIETF;SSDLEKVSQGSAESLSPSFRGVHVSFTTGST
CRISFLPLTRLRRTESVPSDINNPVDRAAEP
DSDSWCLLGTDSCRPSL_______________

GGINKIKPTGLLTIPSPQI_____________
RAFLQMDSPKHQSDPSEDEDERSSQKLHSTS
HLGKRSLKRNFELVDSSPCKKIIQNKKTCVE;IETKGFNKKDLELALSPIHNSSALPTTGRSC
ASMEQLLREQPGEEYSEEEESVLKNSDVEPT;TGLFDANNPKMLRTCSLPDLSKLFRTLMDVP
DDKNIETKEGSEFSFSDGEVAEKAEVYGSEN
IKYQQITPVNQSRSSSPTQYGLTKNFSSLHL;RMRQIASNTSLQRSQSNPILGSPFFSHFDGQ
KKVHLRPDEDLRNCESDTEEDIARRKALHPR
TSHRVEMPRQNSDPTSENPPLPTRIEK;EEDIPPKVPQRTTSISPALARKNSPGNGSAL;GSALGPRLGSQPIRASNPDLRRTEPILESPL;PQRTTSISPALARKNSPGNGSALGPRLGSQP;SSERTRVRANSKSEGSPVLPHEPAKV
GHKISDYFEYQGGNGSSPVRGIPPAIRSPQN;HKISDYFEYQGGNGSSPVRGIPPAIRSPQNS
DLDNTVFGAERKKRLSIIGPTSRDRSSPPPG;LDGESYPKSRMPRAQSYPDNHQEFSDYDNPI
PPQTPETSTFRNQMPSPTSTGTPSPGPRGNQ
NEFTFGNKLDTFCGSPPYAAPELFQG;DFGFSNEFTVGGKLDTFCGSPPYAAPELFQG;KSSGSAVGGKGIAPASPMLGNASNPNKADIP;RESGRKASSTAKVPASPLPGLERKKTTPTPS;STNSVLSTSTNRSRNSPLLERASLG
GNACWVHKHFTEDIQTRQYRAVEVLIGAEYG;GNACWVHKHFTEDIQTRQYRSIEVLIGAGYS;GNACWVHKHFTEDIQTRQYRSLEVLIGSGYN
________________MTLDVGPEDELPDWAAA
TRYFDDEFTAQSITITPPDRYDSLGLLELDQ
ARAFSLAKNSQPNRYTNRVVTLWYRPPELLL
LTDFGFAKKLVDRTWTLCGTPEYLAPEVIQS
TRNCKILLTEPPMNPTKNREKIVEVMFETYQ
QKYEKLEKIGEGTYGTVFKAKNRETHEIVAL
________________MSDNGELEDKPPAPPVR;TPALNAKGTEAPAVVTEEEDDDEETAPPVIA;VLKFYDSNTVKQKYLSFTPPEKDGFPSGTPA
FTSCLRHFSQTSRSTTPANLDSESEHFFRCC
KKPDGVKESTESSNTTIEDEDVKARKQEIIK
EGAENLRRATTDLGRSLGPVELLLRGSSRRL;FGLCKEGMGYGDRTSTFCGTPEFLAPEVLTD;VSNFDEEFTGEAPTLSPPRDARPLTAAEQAA
________________MSEGESQTVLSSGSDPK;EEVTSPVVPPSVKTPTPEPAEVETRKVVLMQ
GFIPFNFVAKANSLEPEPWFFKNLSRKD;KIADFGLARLIEDNEYTAREGAKFPIKWTAP;KIADFGLARLIEDNEYTARQGAKFPIKWTAP;KVADFGLARLIEDNEYTARQGAKFPIKWTAP;LRSVLEDFFTATEGQYQPQP_____
FGSAKQLVRGEPNVSYICSRYYRAPELIFGA
VDFGSSCQLGQRIYQYIQSRFYRSPEVLLGM
ADTTTPTPTAILAPGSPASPPGSLEPKAARL;KATKTAPPALPTGYDSEEEEESRPMSYDEKR;TTPTPTAILAPGSPASPPGSLEPKAARLPPM
DVDDGSGSPHSPHQLSSKSLPSQNLS;ASLQNFNISNLQKSISNPPGSNLRTT______;PEAAFLSRDVDDGSGSPHSPHQLSSKSLPSQ;SLTQVVHSAGRRFIVSPVPESRLRESKVFPS;TEDKITDTKKEGPVASPPFMDLEQAV
EPREHGHSYSNRKYESDEDSLGSSGRVCVEK;HGHSYSNRKYESDEDSLGSSGRVCVEKWNLL;RDAVNQNTKLLQSAKTILRGTEEKCGSPQVR
LNTISLDTMPKF;HFRSSENVLDEEGGRSPRLRPRSRSLSPGRA;IPQFSSCSHRFSKVYSSSEFLAVQPTPTFAE;LNTISLDTMPKFAFSSEDEGVGPGPAGPKRP;PLACPPISAPPPRSPSPLPGHPPAPARSPRL;PQFSSCSHRFS
______MGCIKSKGKDSLSDDGVDLKTQPVPE;___MGCIKSKGKDSLSDDGVDLKTQPVPESQ

Polito et al. Universal ready-to-use immunotherapeutic approach for the treatment of cancer: expanded and activated polyclonal γδ memory T cells.
GD kinome

APPSEETPLIPQRSCSLLSTEAGALHVLLPA;SEETPLIPQRSCSLLSTEAGALHVLLPARGP;THAFTAHPEGKHHSLSFS______________
SASSSHGSIQESHKASRDPSPIQDGNEDMKA;SHGSIQESHKASRDPSPIQDGNEDMKAIPEG
ISDYFEFAGGSAPGTSPGRSVPPVARSSPQH;PGRSVPPVARSSPQHSLSNPLPRRVEQPLYG;PRGHKISDYFEFAGGSAPGTSPGRSVPPVAR;RSVPPVARSSPQHSLSNPLPRRVEQPLYGLD
KKSVLCSTPTINIPASPFMQKLGFGTGVNVY;VNVYLMKRSPRGLSHSPWAVKKINPICNDHY
LGIPDENFEDHSAPPSPEEKDSGFFMLRKDS;RTLFLGIPDENFEDHSAPPSPEEKDSGFFML
QERNVPTKS;HQGNLFTLVPSSRSLSTNGENMGLAVQYLDP;MGLAVQYLDPRGRLRSADSENALSVQERNVP;PRSRHLSVSSQNPGRSSPPPGYVPERQQHIA;SENALSVQERNVPTKSPSAPINWRRGKLLGQ;SSPHSGV
GVSAKNTRTIQRRDSFIGTPYWMA;DLNLSISSFLSKTKDSGSISLQETRRQKKTL;DVAQKVDEDSAEDTQSNDGKEVVEVGQKLIN;FGVSAKNTRTIQRRDSFIGTPYWMAPEVVMC;GTCEAADVAQKVDEDSAEDTQSND
DFGLAKSFGSPNRAYTHQVVTRWYRAPELLF;GVLKLADFGLAKSFGSPNRAYTHQVVTRWYR
GQKVGSLTP;PAVQGQKVGSLTPPSSPKTQRAGHRRILSDV;PGKLGGAVPFAPPEVSPEQAKTVQGGRKNQF;PPAVQGQKVGSLTPPSSPKTQRAGHRRILSD;PSSPKTQRAGHRRILSDVTHSAVFGVPASKS;SLSKYSRH
__MDMFQKVEKIGEGTYGVVYKAKNRETGQL;__MENFQKVEKIGEGTYGVVYKARNKLTGEV;_MDMFQKVEKIGEGTYGVVYKAKNRETGQLV;_MENFQKVEKIGEGTYGVVYKARNKLTGEVV
__MEDYTKIEKIGEGTYGVVYKGRHKTTGQV;_MEDYTKIEKIGEGTYGVVYKGRHKTTGQVV;KTTGQVVAMKKIRLESEEEGVPSTAIREISL
TKVVAPPSS;FAKMPDEPEEPVVAVSSPAVPPPTKVVAPPS;HKSDPYSTGHLREAPSPLMIHSPQMSQFQSL;IHSPQMSQFQSLTHQSPPQQNVQPKKQELRA;STGHLREAPSPLMIHSPQMSQFQSLTHQSPP;VQPQPL
GVVKLGDLGLGRFFSSKTTAAHSLVGTPYYM;TGVVKLGDLGLGRFFSSKTTAAHSLVGTPYY;VKLGDLGLGRFFSSKTTAAHSLVGTPYYMSP

Polito et al. Universal ready-to-use immunotherapeutic approach for the treatment of cancer: expanded and activated polyclonal γδ memory T cells.
GD kinome

T: Phospho (STY) Probabilities
HS(1)GIDFK
REDS(1)YHVR
AVSETPAVPPVS(1)EDEDDDDDDATPPPVIAPRPEHTK;DVATSPIS(0.983)PT(0.017)ENNTTPPDALTR;YMS(1)FTDK
AS(0.951)LS(0.049)VAGPGKPQEEDQPLPAR;DLFAFQES(1)PPR
GLS(1)VDSAQEVK;SQS(1)LPGADSLLAKPIDK
HS(0.002)S(0.998)PTEERDEPAYPR;LES(1)EDDSLGWGAPDWSTEAGFSR
LS(1)LQER;RS(1)FGNPFEGSR
S(1)AGNIPLSPLAR;SDS(1)LPSFR
RMS(1)ADMSEIEAR;S(1)IQDLTVTGTEPGQVSSR;T(0.142)AS(0.858)FGNILDVPEIVISGNGQPR
SPS(1)LNLLQNK
NPLPPILGS(0.91)PT(0.09)K
GS(1)KESLVPQR;QITT(0.007)S(0.991)PIT(0.003)VR;SNS(1)FNNPLGNR
S(1)LVGTPYWMAPELISR
GQGESDPLDHEPAVS(1)PLLPR
QQFYHS(1)VQDLSGGSR;SAS(1)EPSLHR;SHSESAS(0.001)PS(0.001)ALS(0.118)S(0.697)S(0.183)PNNLS(0.948)PT(0.051)GWS(0.001)QPK;STS(0.991)T(0.008)PNVHMVSTTLPVDSR
RDS(0.128)S(0.872)DDWEIPDGQITVGQR;RDS(0.997)S(0.003)DDWEIPDGQITVGQR;SAS(1)EPSLNR;SSS(0.999)APNVHINTIEPVNIDDLIR
S(0.001)GS(0.999)PS(0.001)DNSGAEEMEVSLAKPK
S(0.001)YS(0.999)FPKPGHR
MCDFGISGYLVDS(1)VAK
RDS(0.999)DT(0.001)YR;YRS(1)PEPDPYLSYR
AT(0.012)S(0.988)PEAGGGGGALK;LSAS(0.992)S(0.008)EDISER
STANQS(1)FRK
RLS(0.937)S(0.032)S(0.032)LNPSK
LQDEANYHLYGS(1)RMDR;MQS(1)LQLDCVAVPS(0.587)S(0.413)R
VFGS(1)LER
SES(1)PPPLSDPK
AS(0.003)S(0.997)LGEIDESSELR
KLS(1)NPDIFSSTGK;NQSLGQS(0.999)APS(0.001)LTAGLK
AAS(0.997)S(0.003)PDGFFWTQGSPK;IEEEEEVEQELLS(0.011)HS(0.989)WGR;SAS(1)FAFEFPK;VSQGSAES(0.007)LS(0.956)PS(0.037)FR
TES(1)VPSDINNPVDR
EEMEMDPKPDLDSDSWCLLGTDSCRPS(1)L

Polito et al. Universal ready-to-use immunotherapeutic approach for the treatment of cancer: expanded and activated polyclonal γδ memory T cells.
GD kinome

IKPTGLLTIPS(1)PQI
HQS(0.004)DPS(0.996)EDEDER
DLELALS(1)PIHNSSALPTTGR;NFELVDS(0.893)S(0.107)PCK
EQPGEEYS(1)EEEESVLK;TCS(1)LPDLSK
EGSEFS(0.002)FS(0.998)DGEVAEK
SQS(1)NPILGSPFFSHFDGQDSYAAAVR;SSS(1)PTQYGLTK
NCES(1)DTEEDIAR
KNS(1)PGNGSALGPR;LGSQPIRAS(1)NPDLR;QNS(1)DPTSENPPLPTR;SEGS(1)PVLPHEPAK;T(0.001)T(0.002)S(0.997)IS(1)PALAR
ISDYFEYQGGNGS(0.065)S(0.935)PVR;ISDYFEYQGGNGS(0.818)S(0.182)PVR
AQS(1)YPDNHQEFSDYDNPIFEK;RLS(1)IIGPTSR
NQMPS(0.999)PT(0.001)STGTPSPGPR
GIAPAS(1)PMLGNASNPNK;LDT(1)FCGSPPYAAPELFQGK;RNT(1)YVCSER;S(0.164)T(0.836)FHAGQLR;SRNS(1)PLLER;VPAS(1)PLPGLER
HFTEDIQT(1)R
T(1)LDVGPEDELPDWAAAK
YFDDEFTAQSIT(0.001)IT(0.998)PPDRYDS(0.001)LGLLELDQR
NSQPNRYT(1)NR
TWT(1)LCGTPEYLAPEVIQSK
ILLTEPPMNPT(1)KNR
IGEGTYGT(1)VFK
GTEAPAVVT(1)EEEDDDEETAPPVIAPRPDHTK;S(1)DNGELEDKPPAPPVR;YLS(1)FTPPEK
STT(1)PANLDSESEHFFR
ESTESSNT(0.005)T(0.995)IEDEDVK
S(1)LGPVELLLR;T(0.001)S(0.001)T(0.998)FCGTPEFLAPEVLTDTSYTR;TDVSNFDEEFTGEAPT(1)LS(1)PPR
S(1)EGESQTVLSSGSDPK;TPT(1)PEPAEVETR
ANS(1)LEPEPWFFK;DGS(1)LNQSSGYR;DPLVTYEGSNPPAS(1)PLQDNLVIALHSYEPSHDGDLGFEK;ILEQS(1)GEWWK;LIEDNEY(0.891)T(0.109)AR;NLDNGGFY(0.994)IS(0.006)PR;SVLEDFFTATEGQY(1)QPQ
GEPNVS(0.012)Y(0.987)ICS(0.001)R
IYQY(1)IQSR
ADTTTPTPTAILAPGS(1)PAS(0.984)PPGS(0.016)LEPK;ADTTTPTPTAILAPGS(1)PAS(0.999)PPGS(0.001)LEPK;TAPPALPTGYDS(1)EEEEESRPMSYDEKR
DVDDGSGS(0.997)PHS(0.003)PHQLSSK;DVDDGSGSPHS(1)PHQLSSK;FIVS(1)PVPESR;KEGPVAS(1)PPFMDLEQAVLPAVIPK;SIS(1)NPPGSNLR
KYES(1)DEDS(1)LGSSGR;KYESDEDS(1)LGSSGR;LLQSAKT(1)ILR
(1)PSLLNTIS(0.999)LDTMPK;HFRS(0.845)S(0.155)ENVLDEEGGR;RLS(1)ADIR;S(0.002)PS(0.998)PLPGHPPAPARS(1)PR;SSENVLDEEGGRS(1)PR;VY(0.029)S(0.845)S(0.889)S(0.237)EFLAVQPTPTFAER;V
GKDS(1)LS(1)DDGVDLK

Polito et al. Universal ready-to-use immunotherapeutic approach for the treatment of cancer: expanded and activated polyclonal γδ memory T cells.
GD kinome

HHSLS(0.998)FS(0.001);S(0.001)CS(0.999)LLS(0.853)T(0.147)EAGALHVLLPAR;S(0.101)CS(0.899)LLS(0.987)T(0.013)EAGALHVLLPAR
AS(1)RDPS(1)PIQDGNEDMK;DPS(1)PIQDGNEDMK
ISDYFEFAGGS(0.002)APGT(0.222)S(0.776)PGR;ISDYFEFAGGS(0.991)APGT(0.008)S(0.001)PGR;S(0.467)S(0.544)PQHS(0.995)LS(0.994)NPLPR;S(0.484)S(0.532)PQHS(0.99)LS(0.995)NPLPR
GLSHS(1)PWAVK;SVLCS(0.442)T(0.557)PT(0.001)INIPAS(1)PFMQK
TLFLGIPDENFEDHS(1)APPS(1)PEEK
[illegible]S(0.016)AGDINTIYQPPEPR;DYSDGRRT(1)FPR;HLS(0.999)VS(0.002)S(0.001)QNPGRS(0.771)S(0.226)PPPGYVPER;LRS(1)ADS(1)ENALSVQER;NVPT(0.011)KS(0.937)PS(0.051)APINWR;SLS(0.996)T(0.[illegible]
[illegible]QETR;LS(1)QNACILESVSEK;NTRT(1)IQR;RDS(1)FIGTPYWMAPEVVMCETSK;T(0.001)KDS(0.999)GS(0.994)IS(0.006)LQETR;VDEDS(1)AEDT(0.001)QS(0.999)NDGKEVVEVGQK;VDEDSAEDTQS(1)NDGK[illegible]
AYT(1)HQVVTR;SFGS(1)PNR
[illegible]PSPEAQPIAAYK;HYSPEDEPS(1)PEAQPIAAYK;ILS(1)DVTHSAVFGVPASK;LGGAVPFAPPEVS(1)PEQAK;VGSLT(1)PPS(0.888)S(0.112)PK;VGSLT(1)PPS(0.891)S(0.109)PK;VGSLT(1)PPSS(1)PKTQR;VQTT(1)[illegible]
IGEGT(1)Y(1)GVVYK
IGEGT(1)Y(1)GVVYK;LES(1)EEEGVPSTAIR
[illegible]PPQQNVQPK;EAPS(1)PLMIHS(1)PQMSQFQS(0.045)LT(0.125)HQS(0.83)PPQQNVQPK;EAPSPLMIHS(0.97)PQMS(0.03)QFQS(0.013)LT(0.155)HQS(0.833)PPQQNVQPK;IHS(1)PIIR;MPDEPEEPVVAVS([illegible]
FFS(0.506)S(0.506)KT(0.822)T(0.221)AAHS(0.945)LVGTPYYMSPER;FFS(0.823)S(0.834)KT(0.501)T(0.475)AAHS(0.363)LVGT(0.002)PY(0.001)YMSPER

Polito et al. Universal ready-to-use immunotherapeutic approach for the treatment of cancer: expanded and activated polyclonal γδ memory T cells.
GD kinome

T: Position in peptide	T: Unique identifier
2	UID400
4	UID1040
12;3;8	UID1044;UID1045;UID1047
2;8	UID1521;UID1522
3	UID1700;UID1701
3	UID1761;UID1762
2	UID2283;UID9525
1;3	UID2360;UID2361
1;3	UID2794;UID2795;UID2796
3	UID2797
9	UID3082
2;3;5	UID3429;UID3430;UID3431
1	UID3448
15	UID3456
19;3;6	UID16801;UID3496;UID3499;UID3736
3;4	UID2182;UID2183;UID2184;UID3901
3	UID4409
3	UID4720
13	UID4801
3	UID4922;UID4926
3;4	UID6085;UID6087
6	UID6189
3	UID6241
12;3	UID6245;UID6246
4	UID6498
3	UID6644
3	UID6817
3;7	UID7490;UID7492
10;15;3	UID8157;UID8160;UID8161;UID8163
3	UID8197
27	UID8744

11	UID8780
6	UID9205
7	UID9322;UID9323
3;8	UID9485;UID9486
8	UID9881
3	UID10762;UID10763
4	UID11062
3;4;5;9	UID11130;UID11131;UID11132;UID11133;UID11135
13;14	UID11141;UID11142
3	UID11580;UID11581
5	UID11713
2;3;4;6	UID11931;UID11932;UID12053;UID1466;UID1471;UID385
8	UID11989
1	UID12137
14	UID12423
8	UID12512
3	UID12515
11	UID12552
8	UID12584
1;3;9	UID12655;UID6065;UID6067
3	UID12661
9	UID12678
1;18;3	UID12739;UID6815;UID6816
1;3	UID11039;UID13132
14;3;5;7;8	UID13241;UID13252;UID13253;UID3571;UID3573;UID3574;UID3576
7	UID13276
4	UID13285
12;16;19	UID13870;UID13871;UID567;UID568;UID569
11;3;4;7;8	UID15015;UID15016;UID1713;UID1714;UID1716;UID1718
4;7;8	UID12168;UID15831;UID15832
13;3;4;5	UID16232;UID16233;UID2918;UID2919;UID2921;UID2922;UID2925;UID2927
4;6	UID16946;UID16947;UID3644

3;5;6	UID18395;UID18396;UID5091
2;3	UID20127;UID20128;UID6825
11;16;6;8	UID21340;UID8034;UID8035;UID8036
14;5	UID22727;UID9423
15;19	UID22994;UID22995;UID9692
12;2;3;6;8	UID13009;UID23015;UID23016;UID9708;UID9709;UID9711;UID9713;UID9714;UID9716
11;2;3;4;5	UID10195;UID10199;UID10203;UID10204;UID13064;UID23499;UID23500;UID23506;UID23507
3;4	UID12511;UID18297;UID25814;UID4994
13;3;4;5;8;9	UID12754;UID20203;UID236;UID237;UID239;UID26056;UID6898;UID6900;UID6901
5;6	UID12099;UID13239;UID25402;UID26542
3;5;6	UID12297;UID13255;UID25600;UID26558;UID3590
10;13;14;23;3;4	UID29610;UID29611;UID29612;UID3007;UID3008;UID3009
3;4;6	UID35372;UID35373;UID39529
