# Supplementary material for: Universal Ready-to-Use Immunotherapeutic Approach for the Treatment of Cancer: Expanded and Activated Polyclonal γδ Memory T Cells
Source: Front Immunol. 2019 Nov 22;10:2717. doi: 10.3389/fimmu.2019.02717 (PMC6883509; doi:10.3389/fimmu.2019.02717)
Supplement: Supplementary file 13 [file Table_5.PDF]

Polito et al. Universal ready-to-use immunotherapeutic approach for the treatment of cancer: expanded and activated polyclonal γδ memory T cells.

AB Kinome

AB1_t0	AB2_t0	AB3_t0	AB1_Dp18	AB3_Dp18	AB4_Dp18
-2,04646	-2,31765	-1,16187	-1,86122	-0,895568	-1,11195
-0,426383	-0,364763	-1,24099	-2,42158	0,204733	-3,63411
-0,535666	-1,08634	0,828053	-0,863751	-0,771685	-0,43364
-1,25259	-2,45397	-1,77322	-1,45406	-1,69874	-2,35858
0,507176	0,506329	1,55246	-0,217505	-0,881661	0,156281
-0,586539	-1,11643	-0,0906754	-1,38372	-1,26023	-0,245433
-0,347023	-0,165841	-0,981476	-0,973847	-0,56359	-1,34444
-0,329976	-3,38468	0,939112	0,970246	0,715994	0,428828
-0,496854	-0,989098	-1,01182	-2,55161	-2,51209	-3,16598
-1,91297	-3,01794	-1,17631	-2,02794	-0,973351	-1,68184
-2,33562	-1,12831	-0,742335	-0,899028	0,0289726	-0,649441
-1,27024	-0,462106	-0,445401	0,213326	0,9471	0,313664
0,824491	-0,597055	-3,22325	0,502564	1,10281	0,0252447
1,19764	1,33099	0,344642	1,01321	1,79315	0,875967
0,357469	0,995043	0,849827	-1,72545	-0,224974	-1,77626
2,13571	2,01175	1,69505	1,66749	2,28087	0,480077
0,00217724	1,00809	-0,348534	-3,23224	-2,44001	-2,96065
-0,0153322	0,183844	-0,386549	-1,21548	-1,02931	-0,366054
-3,24455	-2,79125	-2,73257	-2,16787	-1,86631	-2,9044
-1,807	-1,91803	-1,61125	-1,84589	-1,51775	-2,31443
0,448735	1,68327	1,01279	-2,1102	-3,13947	-0,113727
-2,45018	-2,88608	-2,08918	-1,09032	-1,32812	-0,435138
0,0202684	-2,33977	0,59049	-0,838717	0,127626	-0,765557
0,274805	-0,350492	0,475916	0,504174	1,37327	0,961863
-1,83245	-2,02989	-2,23152	-0,524043	-1,25824	-1,80984
-2,31307	-3,11164	-1,99869	-0,842	-0,741615	-0,955081
-0,352345	-0,516813	0,545429	0,482549	1,09746	0,872025
-2,96628	-2,96133	-3,05739	-1,865	-0,99869	-1,43311
-2,11127	-2,59118	-1,26036	-1,46787	-0,160284	-2,05674
2,99262	2,49657	2,36403	1,39099	-2,21862	2,24626
0,844382	0,407347	0,346832	-0,314572	0,76528	-0,172877

AB Kinome

-0,0698919	-0,163382	-0,662294	-1,39405	-2,66771	-1,27053
-1,31604	-1,42582	-0,783631	-1,09112	-0,734255	-0,882876
-2,29821	-1,68216	-1,8924	-0,519893	-0,0897388	-1,34013
-0,785684	-1,26822	-1,31689	-2,39049	-2,76636	-3,75083
0,149156	-1,73785	-0,381347	-0,0606394	0,751069	-0,132585
-2,15274	-2,24485	-1,57107	-2,20754	-1,34365	-0,960546
-1,67168	-2,74873	-1,00561	0,493644	0,104403	-1,28282
-1,79147	-2,4358	-1,77036	0,209595	0,716743	-1,03086
2,0609	1,6369	1,74746	1,2473	2,06594	1,80132
-0,669034	0,658982	0,612765	0,38993	-0,078845	-0,0362244
0,0430021	0,345135	0,00765038	-0,867532	0,488106	0,0707369
-0,226226	-0,706718	0,656639	0,39297	0,498367	0,0473547
0,660641	0,697417	0,814485	0,858257	1,43484	1,19865
2,70178	2,23179	2,47751	1,97591	2,64479	2,12707
-2,91692	0,691635	-1,39887	-1,301	-2,0898	-0,116225
3,32651	2,85564	2,37477	3,79956	3,71207	3,14447
0,595284	1,16469	0,798225	-1,25805	-0,920357	-0,140964
0,00884151	2,60878	1,98775	-1,33014	-0,240091	-2,267
-1,64948	-2,16007	-2,28625	0,212598	0,285643	1,67655
-2,26561	-1,51603	0,555489	-1,55452	-1,60231	-1,64069
1,21015	-0,364617	1,01917	-0,0827866	-1,66462	0,241118
-2,10285	-2,41844	-2,13112	-1,87075	-0,200962	-1,58579
2,21761	3,55318	2,80182	1,82381	1,64219	2,03512
-1,09825	-0,918694	-0,990746	-0,651212	-0,603851	-0,882777
-1,9025	-2,49609	1,57811	1,48062	2,67346	-1,64668
3,90098	4,39928	4,71992	3,75538	4,09812	3,47788
2,35185	2,73919	1,51983	1,60965	-1,01434	1,87762
1,23366	1,04622	0,946693	0,75491	0,314757	-1,97888
-0,754581	-0,721396	0,55345	-0,367103	0,333869	-0,0119486
-1,60469	-1,78498	-1,78773	-0,256419	0,168903	-0,698985
-2,80538	-2,30717	-2,17257	0,923638	0,994553	-0,539159
-1,61932	-1,54039	0,394747	-0,0785913	0,579641	0,324159

AB Kinome

3,10927	2,43541	3,06068	2,15954	-2,50888	3,01702
-1,74418	-2,29253	-0,823549	-2,60437	-2,70576	-3,28703
-0,920063	-0,895438	-1,61745	-2,8583	-1,79416	-2,30482
3,76522	3,27906	1,74053	-0,692655	-0,85511	-0,219413
-0,400067	-0,155949	-1,13724	-0,840142	-0,587019	-0,992913
0,616731	0,556398	0,712358	0,426888	-2,29804	-0,112591
2,77005	2,24345	1,89177	2,01682	3,42965	1,51806
0,720931	0,812351	0,137657	0,0623045	1,02143	-0,263505
-2,87701	-2,7769	-2,38394	2,9937	3,28755	1,5705
-2,36047	-2,11666	-2,01412	2,9937	3,28755	1,5705
1,67958	-2,41624	-0,461826	0,195291	0,628819	-0,962882
-0,705622	3,52283	-0,272005	-1,39186	-0,504143	-1,32335

Polito et al. Universal ready-to-use immunotherapeutic approach for the treatment of cancer: expanded and activated polyclonal γδ memory T cells.

AB Kinome

C: KEGG name
Calcium signaling pathway;ErbB signaling pathway;Gastric acid secretion;Glioma;GnRH signaling pathway;ko05152;Long-term potentiation;Melanogenesis;Neurotrophin signaling pathway;Olf
Axon guidance;Chemokine signaling pathway;Epithelial cell signaling in Helicobacter pylori infection;ErbB signaling pathway;Fc gamma R-mediated phagocytosis;Focal adhesion;MAPK signalin
Adipocytokine signaling pathway
Adherens junction;Leishmaniasis;MAPK signaling pathway;Measles;NOD-like receptor signaling pathway;Osteoclast differentiation;RIG-I-like receptor signaling pathway;T cell receptor signalin
ko05152;Neurotrophin signaling pathway;NOD-like receptor signaling pathway;Shigellosis
mTOR signaling pathway;Regulation of autophagy
Axon guidance;ErbB signaling pathway;Focal adhesion;Regulation of actin cytoskeleton;Renal cell carcinoma;T cell receptor signaling pathway
Axon guidance;Cell cycle;Chronic myeloid leukemia;ErbB signaling pathway;Neurotrophin signaling pathway;Pathogenic Escherichia coli infection;Pathways in cancer;Shigellosis;Viral myocardi
Acute myeloid leukemia;B cell receptor signaling pathway;Bladder cancer;Chemokine signaling pathway;Chronic myeloid leukemia;Colorectal cancer;Endometrial cancer;ErbB signaling pathwa
B cell receptor signaling pathway;Chemokine signaling pathway;Epithelial cell signaling in Helicobacter pylori infection;Fc epsilon RI signaling pathway;Fc gamma R-mediated phagocytosis;Lon
Acute myeloid leukemia;Bladder cancer;Chemokine signaling pathway;Chronic myeloid leukemia;Colorectal cancer;Endometrial cancer;ErbB signaling pathway;Focal adhesion;Glioma;Hepatiti
Axon guidance
Acute myeloid leukemia;Adipocytokine signaling pathway;Apoptosis;B cell receptor signaling pathway;Carbohydrate digestion and absorption;Chagas disease (American trypanosomiasis);Che
Acute myeloid leukemia;Adipocytokine signaling pathway;Apoptosis;B cell receptor signaling pathway;Carbohydrate digestion and absorption;Chagas disease (American trypanosomiasis);Che
Adherens junction;Chagas disease (American trypanosomiasis);Chronic myeloid leukemia;Colorectal cancer;Cytokine-cytokine receptor interaction;Endocytosis;MAPK signaling pathway;Osteo
ErbB signaling pathway;Viral myocarditis
Natural killer cell mediated cytotoxicity;Primary immunodeficiency;T cell receptor signaling pathway
Chagas disease (American trypanosomiasis);Epithelial cell signaling in Helicobacter pylori infection;ErbB signaling pathway;Fc epsilon RI signaling pathway;GnRH signaling pathway;MAPK signa
Chemokine signaling pathway;Leukocyte transendothelial migration;T cell receptor signaling pathway
GnRH signaling pathway;MAPK signaling pathway;Neurotrophin signaling pathway;RIG-I-like receptor signaling pathway;Ubiquitin mediated proteolysis
Axon guidance;Chemokine signaling pathway;Focal adhesion;Leukocyte transendothelial migration;Pathogenic Escherichia coli infection;Regulation of actin cytoskeleton;Shigellosis;TGF-beta s
Cell cycle;p53 signaling pathway
Apoptosis;Cytosolic DNA-sensing pathway;Hepatitis C;RIG-I-like receptor signaling pathway;Toll-like receptor signaling pathway

Polito et al. Universal ready-to-use immunotherapeutic approach for the treatment of cancer: expanded and activated polyclonal γδ memory T cells.

AB Kinome

Regulation of autophagy
MAPK signaling pathway;Tight junction
Gap junction;GnRH signaling pathway;MAPK signaling pathway
Cytosolic DNA-sensing pathway
Amoebiasis;Apoptosis;Bile secretion;Calcium signaling pathway;Chemokine signaling pathway;Dilated cardiomyopathy;Endocrine and other factor-regulated calcium reabsorption;Gap junctio
Apoptosis;Cell cycle;p53 signaling pathway
Calcium signaling pathway;ErbB signaling pathway;Gastric acid secretion;Glioma;GnRH signaling pathway;ko05152;Long-term potentiation;Melanogenesis;Neurotrophin signaling pathway;Olf
Parkinson's disease
Adherens junction;Axon guidance;Bacterial invasion of epithelial cells;Endocytosis;Epithelial cell signaling in Helicobacter pylori infection;ErbB signaling pathway;Fc epsilon RI signaling pathwa
Alzheimer's disease;Axon guidance;B cell receptor signaling pathway;Basal cell carcinoma;Cell cycle;Chemokine signaling pathway;Circadian rhythm - fly;Colorectal cancer;Endometrial cancer;
GnRH signaling pathway;MAPK signaling pathway
Chemokine signaling pathway;Jak-STAT signaling pathway;ko05152;Measles
Axon guidance;ErbB signaling pathway;Focal adhesion;MAPK signaling pathway;Regulation of actin cytoskeleton;Renal cell carcinoma;T cell receptor signaling pathway

AB Kinome

Calcium signaling pathway;Long-term potentiation;Neurotrophin signaling pathway;Osteoclast differentiation
Parkinson's disease
Amyotrophic lateral sclerosis (ALS);MAPK signaling pathway;Neurotrophin signaling pathway;Protein processing in endoplasmic reticulum
GnRH signaling pathway;MAPK signaling pathway;Neurotrophin signaling pathway
Oocyte meiosis
Basal transcription factors;Cell cycle;Nucleotide excision repair
Cell cycle;Measles;Oocyte meiosis;p53 signaling pathway;Pathways in cancer;Progesterone-mediated oocyte maturation;Prostate cancer;Small cell lung cancer
Cell cycle;Gap junction;Oocyte meiosis;p53 signaling pathway;Progesterone-mediated oocyte maturation

Polito et al. Universal ready-to-use immunotherapeutic approach for the treatment of cancer: expanded and activated polyclonal γδ memory T cells.

AB Kinome

C: Student's T-test Significant AB_Dp18_AB_t0	N: Localization prob	T: Protein
+	0,999998	A0A0A0MS52;Q13557-8
	0,999995	B1AVT0
g pathway;MAPK signaling pathway - yeast;Natu	0,999575	B3KNX7
+	0,975569	E7EX48
	0,999999	F2Z2U4
	1	Q96RR4-5
+	0,999975	J3QT34;O60307;Q6P0Q8
ng pathway;Toll-like receptor signaling pathway;T	1	O43318
+	0,999999	O43353-2
	0,909668	O75385
	0,995304	O95835
	0,999998	O96013
itis	1	P00519
+	0,995502	P04049;P10398
+	1	P07948-2
+	0,997814	P15056
+	0,994926	P21709
mokine signaling pathway;Chronic myeloid leuke	0,998899	P31749
mokine signaling pathway;Chronic myeloid leuke	0,988824	P31751
oclast differentiation;Pancreatic cancer;Pathways	0,959255	P37173
	0,99974	P42684-4
+	0,999988	P43403
aling pathway;Toll-like receptor signaling pathwa	0,986035	P45985
	0,99943	P49761-1
	0,996869	Q08881
+	0,991762	Q13233
signaling pathway;Vascular smooth muscle contr	1	Q13464
+	0,936592	Q13535-2
	1	Q13546
	1	Q15059
	0,997317	Q16513-2

AB Kinome

+	0,95582	Q86YV5
	0,998524	Q8IVT5-2
	1	Q8IZE3-2
+	0,999785	Q8TEA7-3
	0,999986	Q96PY6-4
	1	Q99570
	0,987095	Q9NRH2
+	0,999938	Q9NYL2
+	0,999999	Q9UKE5-6
	0,876333	Q9UKI8-4
+	0,999989	Q9Y2U5
	0,998744	Q9Y572
+	1	A0A0A0MT23;E7ETY4
	0,886158	O14874
	0,999985	P50750
n;Gastric acid secretion;GnRH signaling pathway	0,999981	P51817
+	1	P61160
+	0,996623	Q13315
+	1	Q13557-8
	0,999878	Q16512-2
	0,98082	Q8TD19
	1	Q9BXM7
	1	Q9UHY1
y;Focal adhesion;Gap junction;GnRH signaling pa	0,993644	P06239;P06241-2
ErbB signaling pathway;Focal adhesion;Hedgeho	0,986567	P49840;P49841
	0,999987	Q13627-2
	0,999786	H0Y6K2
+	0,999922	F5GWT4
	0,999994	J3KNB8
+	0,999988	O00418
+	0,998356	P52333
+	0,999999	Q13177

Polito et al. Universal ready-to-use immunotherapeutic approach for the treatment of cancer: expanded and activated polyclonal γδ memory T cells.

AB Kinome

	1	Q16566
	0,989602	Q5S007
+	0,994565	Q86UE8-2
+	1	Q99683
+	0,999559	Q99759
+	0,99999	Q9H2G2-2
	1	P50613
+	0,999999	A0A096LP25;Q2M2I8
+	1	
+	1	P06493
	0,999607	O60885
	0,828678	Q8TDX7

Polito et al. Universal ready-to-use immunotherapeutic approach for the treatment of cancer: expanded and activated polyclonal γδ memory T cells.

AB Kinome

T: Protein names
n/calmodulin-dependent protein kinase type II subunit beta;Calcium/calmodulin-dependent protein kinase type II subunit delta;Calcium/calmodulin-dependent protein kinase type II subunit g
Dual specificity protein kinase CLK2
Non-specific serine/threonine protein kinase;Serine/threonine-protein kinase PAK 1
Serine/threonine-protein kinase Nek4
Transformation/transcription domain-associated protein
Calcium/calmodulin-dependent protein kinase kinase 1;Calcium/calmodulin-dependent protein kinase kinase 2
Microtubule-associated serine/threonine-protein kinase 2;Microtubule-associated serine/threonine-protein kinase 3;Microtubule-associated serine/threonine-protein kinase 4
Mitogen-activated protein kinase kinase kinase 7
Receptor-interacting serine/threonine-protein kinase 2
Serine/threonine-protein kinase ULK1
Serine/threonine-protein kinase LATS1
Serine/threonine-protein kinase PAK 4
Tyrosine-protein kinase ABL1
RAF proto-oncogene serine/threonine-protein kinase;Serine/threonine-protein kinase A-Raf
Tyrosine-protein kinase Lyn
Serine/threonine-protein kinase B-raf
Ephrin type-A receptor 1
RAC-alpha serine/threonine-protein kinase
RAC-beta serine/threonine-protein kinase
TGF-beta receptor type-2
Abelson tyrosine-protein kinase 2
Tyrosine-protein kinase ZAP-70
Dual specificity mitogen-activated protein kinase kinase 4
Dual specificity protein kinase CLK3
Tyrosine-protein kinase ITK/TSK
Mitogen-activated protein kinase kinase kinase 1
Rho-associated protein kinase 1
Serine/threonine-protein kinase ATR
Receptor-interacting serine/threonine-protein kinase 1
Bromodomain-containing protein 3
Serine/threonine-protein kinase N2

Polito et al. Universal ready-to-use immunotherapeutic approach for the treatment of cancer: expanded and activated polyclonal γδ memory T cells.

AB Kinome

Tyrosine-protein kinase SgK223
Kinase suppressor of Ras 1
Protein-associating with the carboxyl-terminal domain of ezrin
TBC domain-containing protein kinase-like protein
Serine/threonine-protein kinase Nek1
Phosphoinositide 3-kinase regulatory subunit 4
SNF-related serine/threonine-protein kinase
Mitogen-activated protein kinase kinase kinase MLT
TRAF2 and NCK-interacting protein kinase
Serine/threonine-protein kinase tousled-like 1
Mitogen-activated protein kinase kinase kinase 2
Receptor-interacting serine/threonine-protein kinase 3
MAP/microtubule affinity-regulating kinase 3;MAP/microtubule affinity-regulating kinase 4;Serine/threonine-protein kinase MARK1;Serine/threonine-protein kinase MARK2
[3-methyl-2-oxobutanoate dehydrogenase [lipoamide]] kinase, mitochondrial
Cyclin-dependent kinase 9
cAMP-dependent protein kinase catalytic subunit PRKX;Putative serine/threonine-protein kinase PRKY
Actin-related protein 2
Serine-protein kinase ATM
Calcium/calmodulin-dependent protein kinase type II subunit delta;Calcium/calmodulin-dependent protein kinase type II subunit alpha
Serine/threonine-protein kinase N1
Serine/threonine-protein kinase Nek9
Serine/threonine-protein kinase PINK1, mitochondrial
Nuclear receptor-binding protein
Non-specific protein-tyrosine kinase;Proto-oncogene tyrosine-protein kinase Src;Tyrosine-protein kinase Fyn;Tyrosine-protein kinase Lck;Tyrosine-protein kinase Yes
Glycogen synthase kinase-3 alpha;Glycogen synthase kinase-3 beta
Dual specificity tyrosine-phosphorylation-regulated kinase 1A;Dual specificity tyrosine-phosphorylation-regulated kinase 1B
Bromodomain-containing protein 2
Serine/threonine-protein kinase WNK1
Mitogen-activated protein kinase kinase kinase 4
Eukaryotic elongation factor 2 kinase
Tyrosine-protein kinase JAK3
PAK-2p27;PAK-2p34;Serine/threonine-protein kinase PAK 2

Polito et al. Universal ready-to-use immunotherapeutic approach for the treatment of cancer: expanded and activated polyclonal γδ memory T cells.

AB Kinome

Calcium/calmodulin-dependent protein kinase type IV
Leucine-rich repeat serine/threonine-protein kinase 2
Serine/threonine-protein kinase tousled-like 2
Mitogen-activated protein kinase kinase kinase 5
Mitogen-activated protein kinase kinase kinase 3
STE20-like serine/threonine-protein kinase
Cyclin-dependent kinase 7
AP2-associated protein kinase 1;Uncharacterized protein FLJ45252
Cyclin-dependent kinase 2;Cyclin-dependent kinase 3
Cyclin-dependent kinase 1
Bromodomain-containing protein 4
Serine/threonine-protein kinase Nek6;Serine/threonine-protein kinase Nek7

Polito et al. Universal ready-to-use immunotherapeutic approach for the treatment of cancer: expanded and activated polyclonal γδ memory T cells.

AB Kinome

T: Gene names
CAMK2B;CAMK2D;CAMK2G
CLK2
PAK1
NEK4
TRRAP
CAMKK1;CAMKK2
MAST2;MAST3;MAST4
DKFZp586F0420;MAP3K7
RIPK2
ULK1
LATS1
PAK4
ABL1
ARAF;RAF1
LYN
BRAF
EPHA1
AKT1
AKT2
TGFBR2
ABL2
ZAP70
MAP2K4
CLK3
ITK
MAP3K1
ROCK1
ATR
RIPK1
BRD3
PKN2

Polito et al. Universal ready-to-use immunotherapeutic approach for the treatment of cancer: expanded and activated polyclonal γδ memory T cells.

AB Kinome

SGK223
KSR1
SCYL3
TBCK
NEK1
PIK3R4
SNRK
ZAK
TNIK
TLK1
MAP3K2
RIPK3
MARK1;MARK2;MARK3;MARK4
BCKDK
CDK9
PRKX;PRKY
ACTR2
ATM
CAMK2D;CAMK2A
PKN1
NEK9
PINK1
NRBP1
FYN;LCK;SRC;YES1
GSK3A;GSK3B
DYRK1A;DYRK1B
BRD2
WNK1
MAP3K4
EEF2K
JAK3
PAK2

Polito et al. Universal ready-to-use immunotherapeutic approach for the treatment of cancer: expanded and activated polyclonal γδ memory T cells.

AB Kinome

CAMK4
LRRK2
TLK2
MAP3K5
MAP3K3
SLK
CDK7
AAK1
CDK2;CDK3
CDC2;CDK1
BRD4
NEK6;NEK7

AB Kinome

T: Sequence window
QETVECLKKFN;ILTTMLATRNFSAAKSLLKKPDGVKESTESS;LKGAILTTMLATRNFSAAKSLLKKPDGVKES;QALKHPWVCQRSTVASMMHRQETVECLRKFN;QTTVVHNATDGIKGSTESCNTTTEDEDLKVR;TVVHNATD
WSSSSDRTRRRRREDSYHVRSRSYDDRSSDR
EFYNSKKTSNSQKYMSFTDKSAEDYNSSNAL;LNVKAVSETPAVPPVSEDEDDDDDDATPPPV;PLPVTPTRDVATSPISPTENNTTPPDALTRN
GEKRQVRRDLFAFQESPPRFLPSHPIVGKVD;SQEEMSSSGPSVRKASLSVAGPGKPQEEDQP
GEGVNSVSSSIKRGLSVDSAQEVKRFRTATG;RTATGAISAVFGRSQSLPGADSLLAKPIDKQ
ERSLSAPGNLLTKQGSEDNLQGTDPPPVGEE;SRLLPARPSLSARKLSLQERPAGSYLEAQAG;TSGSQARPHLSGRKLSLQERSQGGLAAGGSL
AVQPTPTFAERSFSEDREEGWERSEVDYG;GLAGLKGRDRSWVIGSPEILRKRLSVSESSH;GSSCQSSSSQPERGPSPSLLNTISLDTMPKF;IPQFSSCSHRFSKVYSSSEFLAVQPTPTFAE;LGQSAPSLTAGLKELSLPRRGSFCRT
ATTGNGQPRRRSIQDLTVTGTEPGQVS;SSVESLPPTSEGKRMSADMSEIEARIAATTA;SSVESLPPTSEGKRMSADMSEIEARIAATTG;TTAYSKPKRGHRKTASFGNILDVPEIVISGN;VPEIVISGNGQPRRRSIQDLTVTGTEP
GLQPYPEILVVSRSPSLNLLQNKSM_______
LPDFLQRNPLPPILGSPTKAVPSFDFPKTPS
ENVDSGDKEKKQITTSPITVRKNKKDEERRE;HEIPTWQPNIPVRSNSFNNPLGNRASHSANS
FGFCAQVSKEVPRRKSLVGTPYWMAPELISR;GSGGPQESSRDKRPLSGPDVGTPQPAGLASG
KGQGESDPLDHEPAVSPLLPRKERGPPEGGL
SALSSSPNNLSPTGWSQPKTPVPAQR;IELLQHSLPKINRSASEPSLHRAAHTEDINA;IELLQRSLPKIERSASEPSLHRTQADELPAC;SPSSEGSLSQRQRSTSTPNVHMVSTTLPVDS;TVCVDMSTNRQQFYHSVQDLSGGSRQ
___MGCIKSKGKDSLSDDGVDLKTQPVPESQ
DEDHRNQFGQRDRSSSAPNVHINTIEPVNID;IELLARSLPKIHRSASEPSLNRAGFQTEDFS;SEDRNRMKTLGRRDSSDDWEIPDGQITVGQR;SSEDRNRMKTLGRRDSSDDWEIPDGQITVGQ
NFDPRMTLRLPSLSGSDGIPYRTVSEWLESI
GLKKQEEEEMDFRSGSPSDNSGAEEMEVSLA
KQRAPGEDPMDYKCGSPSDSSTTEEMEVAVS
CLCDFGLSLRLDPTLSVDDLANSGQVGTARY
ASSGSPALPRKQRDKSPSSLLEDAKETCFTR
TLNSDGYTPEPARITSPDKPRPMPMDTSVYE
PTGVQNPHIERLRTHSIESSGKLKISPEQHW
________MHHCKRYRSPEPDPYLSYRWKRRR;HDRLPYQRRYRERRDSDTYRCEERSPSFGED
EVFSEGKIPYENRSNSEVVEDISTGFRLYKP
GNRASSSGFPGARATSPEAGGGGGALKASSA;LEKTGKGLCATKLSASSEDISERLASISVGP;VPFQSGRITPPRRAPSPDGFSPYSPEETNRR
RASPRTLSTRSTANQSFRKVVKNTSGKTS__
LSSNSDGISPKRRRLSSSLNPSKRAPKQTED
KKEYSNENAVVKRMQSLQLDCVAVPSSRSNS
TTTPTTSAITASRSESPPPLSDPKQAKVVAR
FDLEPEPPPAPPRASSLGEIDESSELRVLDI

Polito et al. Universal ready-to-use immunotherapeutic approach for the treatment of cancer: expanded and activated polyclonal γδ memory T cells.

AB Kinome

PQKKIVSRAASSPDGFFWTQGSPKPG;SGQNSKVGTGMSKSASFAFEFPKDRSGIETF;SRTCSDGGPSSELAHSPTNSGKKLFAPVPFP;SSDLEKVSQGSAESLSPSFRGVHVSFTTGST;SYSLSPGNRHHPVFSSSDPLEKAFKGS
CRISFLPLTRLRRTESVPSDINNPVDRAAEP;RIDDVSSMRFDLSHGSPQMVRRDIGLSVTHR
LPELRTEMVPKKDDVSPVMQFSSKFAAAEIT
GGINKIKPTGLLTIPSPQI_____________
ASMEQLLREQPGEEYSEEEESVLKNSDVEPT;TGLFDANNPKMLRTCSLPDLSKLFRTLMDVP
QEPDDKRARKHVKQDSNVNEEWKSMFGSLDP
DDLTATPLSHATVPQSPARAADSVLNGHRSK
GKFYRVSQSALNPHQSPDFKRSPRDLHQPNT;IKYQQITPVNQSRSSSPTQYGLTKNFSSLHL;RMRQIASNTSLQRSQSNPILGSPFFSHFDGQ;SRSSSPTQYGLTKNFSSLHLNSRDSGFSSGN
TSHRVEMPRQNSDPTSENPPLPTRIEK;EEDIPPKVPQRTTSISPALARKNSPGNGSAL;GSALGPRLGSQPIRASNPDLRRTEPILESPL;PQRTTSISPALARKNSPGNGSALGPRLGSQP;SSERTRVRANSKSEGSPVLPHEPAKVK
GHKISDYFEYQGGNGSSPVRGIPPAIRSPQN;HKISDYFEYQGGNGSSPVRGIPPAIRSPQNS
DLDNTVFGAERKKRLSIIGPTSRDRSSPPPG;IFTPEYDDSRIRRRGSDIDNPTLTVMDISPP;LDGESYPKSRMPRAQSYPDNHQEFSDYDNPI
PPQTPETSTFRNQMPSPTSTGTPSPGPRGNQ
SNEFTVGGKLDTFCGSPPYAAPELFQG;KSSGSAVGGKGIAPASPMLGNASNPNKADIP;KVQRSVSANPKQRRFSDQAGPAIPTSNSYSK;KVQRSVSSSQKQRRYSDHAGPAIPSVVAYPK;RESGRKASSTAKVPASPLPGLERK
LRPLLGPALALRARSTSATDTHHVEMARERS
ARAFSLAKNSQPNRYTNRVVTLWYRPPELLL
LTDFGFAKKLVDRTWTLCGTPEYLAPEVIQS
TRNCKILLTEPPMNPTKNREKIVEVMFETYQ
FTSCLRHFSQTSRSTTPANLDSESEHFFRCC;SMDDQEKRSLAFEEGSQSTTISSLSEKSKEE;VQGFFTSCLRHFSQTSRSTTPANLDSESEHF
ICQRSTVASMMHRQETVDCLKKFNARRKLKG
LRRATTDLGRSLGPVELLLRGSSRRL;FGLCKEGMGYGDRTSTFCGTPEFLAPEVLTD;LLRRLIPNATGTGTFSPGASPGSEARTTGDI;TDVSNFDEEFTGEAPTLSPPRDARPLTAAEQ;VSNFDEEFTGEAPTLSPPRDARPLTAA
DSDSWCLLGTDSCRPSL_______________;KVTLLNAPTKRPRSSTVTEAPIAVVTSRTSE
LGRGLQLGRALLLRFTGKPGRAYGLGRPGPA
EEVTSPVVPPSVKTPTPEPAEVETRKVVLMQ
KLTEERDGSLNQSSGYRYGTDPTPQHYP;GQEGFIPFNFVAKANSLEPEPWFFKNLSRKD;KIADFGLARLIEDNEYTAREGAKFPIKWTAP;KIADFGLARLIEDNEYTARQGAKFPIKWTAP;KVADFGLARLIEDNEYTARQGAKF
________MSGRPRTTSFAESCKPVQQPSAFG;FGSAKQLVRGEPNVSYICSRYYRAPELIFGA;PSGGGPGGSGRARTSSFAEPGGGGGGGGGGP
VDFGSSCQLGQRIYQYIQSRFYRSPEVLLGM
ADTTTPTPTAILAPGSPASPPGSLEPKAARL;KATKTAPPALPTGYDSEEEEESRPMSYDEKR;TTPTPTAILAPGSPASPPGSLEPKAARLPPM
NFNISNLQKSISNPPGSNLRTT_____;EKPELSEPSHLNGPSSDPEAAFLSRDVDDGS;PEAAFLSRDVDDGSGSPHSPHQLSSKSLPSQ;SDPEAAFLSRDVDDGSGSPHSPHQLSSKSLP;SLTQVVHSAGRRFIVSPVPESRLRESK
DCISKKLERLESEDDSLGWGAPDWSTEAGFS;IQSRDCISKKLERLESEDDSLGWGAPDWSTE;SIAAELQFRSLSRHSSPTEERDEPAYPRGDS
DEDLIFRLEGVDGGQSPRAGHDGDSDGDSDD;EPREHGHSYSNRKYESDEDSLGSSGRVCVEK;HGHSYSNRKYESDEDSLGSSGRVCVEKWNLL;RDAVNQNTKLLQSAKTILRGTEEKCGSPQVR
APPSEETPLIPQRSCSLLSTEAGALHVLLPA;SEETPLIPQRSCSLLSTEAGALHVLLPARGP;THAFTAHPEGKHHSLSFS_______________
ELEDKPPAPPVR;KKNPQAVLDVLKFYDSNTVKQKYLSFTPPEK;QKYLSFTPPEKDGFPSGTPALNAKGTEAPAV;TPALNAKGTEAPAVVTEEEDDDEETAPPVIA;VLKFYDSNTVKQKYLSFTPPEKDGFPSGTPA;VPEEKKPRH

Polito et al. Universal ready-to-use immunotherapeutic approach for the treatment of cancer: expanded and activated polyclonal γδ memory T cells.

AB Kinome

SASSSHGSIQESHKASRDPSPIQDGNEDMKA;SHGSIQESHKASRDPSPIQDGNEDMKAIPEG
SLRSSKLQSHMRHSDSISSLASEREYITSLD
RSVPPVARSSPQHSLSNPLPRRVEQPLYGLD
LGIPDENFEDHSAPPSPEEKDSGFFMLRKDS;RTLFLGIPDENFEDHSAPPSPEEKDSGFFML
MGLAVQYLDP;INTIYQPPEPRSRHLSVSSQNPGRSSPPPGY;MGLAVQYLDPRGLRSADSENALSVQERNVP;NSLSGSCQSLDRSADSPSFRKSRMSRAQSFP;PRSRHLSVSSQNPGRSSPPPGYVPERQQHIA;SENALSVQ
FIGTPYWMA;DVAQKVDEDSAEDTQSNDGKEVVEVGQKLIN;EETENSLPIPASKRASSDLSIASSEEDKLSQ;FGVSAKNTRTIQRRDSFIGTPYWMAPEVVMC;GTCEAADVAQKVDEDSAEDTQSNDGKEVVEV;NLSISSFL
DFGLAKSFGSPNRAYTHQVVTRWYRAPELLF;GVLKLADFGLAKSFGSPNRAYTHQVVTRWYR
QGQKVGSLTP;PGKLGGAVPFAPPEVSPEQAKTVQGGRKNQF;PKTQRAGHRRILSDVTHSAVFGVPASKSTQL;PPAVQGQKVGSLTPPSSPKTQRAGHRRILSD;PSSPKTQRAGHRRILSDVTHSAVFGVPASKS;SLSKYSRHY
__MDMFQKVEKIGEGTYGVVYKAKNRETGQL;__MENFQKVEKIGEGTYGVVYKARNKLTGEV;_MDMFQKVEKIGEGTYGVVYKAKNRETGQLV;_MENFQKVEKIGEGTYGVVYKARNKLTGEVV
__MEDYTKIEKIGEGTYGVVYKGRHKTTGQV;_MEDYTKIEKIGEGTYGVVYKGRHKTTGQVV
PPPTKVVAPPS;HKSDPYSTGHLREAPSPLMIHSPQMSQFQSL;IHSPQMSQFQSLTHQSPPQQNVQPKKQELRA;KEEKIHSPIIRSEPFSPSLRPEPPKHPESIK;STGHLREAPSPLMIHSPQMSQFQSLTHQSPP;VQPQPLVV
GVVKLGDLGLGRFFSSKTTAAHSLVGTPYYM;LGLGRFFSSKTTAAHSLVGTPYYMSPERIHE;TGVVKLGDLGLGRFFSSKTTAAHSLVGTPYY;VKLGDLGLGRFFSSKTTAAHSLVGTPYYMSP

Polito et al. Universal ready-to-use immunotherapeutic approach for the treatment of cancer: expanded and activated polyclonal γδ memory T cells.

AB Kinome

T: Phospho (STY) Probabilities
GS(0.091)T(0.909)ESCNTTTEDEDLK;GST(0.013)ES(0.985)CNT(0.001)TTEDEDLK;NFS(1)AAKS(1)LLK;QET(1)VECLRK;S(1)LLKKPDGVK;STVAS(1)MMHR
REDS(1)YHVR
AVSETPAVPPVS(1)EDEDDDDDDATPPPVIAPRPEHTK;DVATSPIS(0.983)PT(0.017)ENNTTPPDALTR;YMS(1)FTDK
AS(0.951)LS(0.049)VAGPGKPQEEDQPLPAR;DLFAFQES(1)PPR
GLS(1)VDSAQEVK;SQS(1)LPGADSLLAKPIDK
LS(1)LQER;QGS(1)EDNLQGTDPPPVGEEEVLL
S(1)NPDIFSSTGK;LHLS(1)PPLGR;NQSLGQS(0.999)APS(0.001)LTAGLK;RLS(1)ADIR;S(0.001)FS(0.999)EDREEGWER;S(1)AGNIPLSPLAR;SDS(1)LPSFR;SWVIGS(1)PEILR;VY(0.029)S(0.845)S(0.889)S(0.23
RMS(1)ADMSEIEAR;S(1)IQDLTVTGTEPGQVSSR;T(0.142)AS(0.858)FGNILDVPEIVISGNGQPR
SPS(1)LNLLQNK
NPLPPILGS(0.91)PT(0.09)K
QITT(0.007)S(0.991)PIT(0.003)VR;SNS(1)FNNPLGNR
DKRPLS(1)GPDVGTPQPAGLASGAK;S(1)LVGTPYWMAPELISR
GQGESDPLDHEPAVS(1)PLLPR
QQFYHS(1)VQDLSGGSR;SAS(1)EPSLHR;SHSESAS(0.001)PS(0.001)ALS(0.118)S(0.697)S(0.183)PNNLS(0.948)PT(0.051)GWS(0.001)QPK;STS(0.991)T(0.008)PNVHMVSTTLPVDSR
GKDS(1)LS(1)DDGVDLK
RDS(0.128)S(0.872)DDWEIPDGQITVGQR;RDS(0.997)S(0.003)DDWEIPDGQITVGQR;SAS(1)EPSLNR;SSS(0.999)APNVHINTIEPVNIDDLIR
LPSLS(0.005)GS(0.995)DGIPYR
S(0.001)GS(0.999)PS(0.001)DNSGAEEMEVSLAKPK
CGS(0.989)PS(0.011)DSSTTEEMEVAVSK
LDPT(0.041)LS(0.959)VDDLANSGQVGTAR
DKS(1)PSSLLEDAK
ITS(1)PDKPR
LRT(0.014)HS(0.986)IESSGK
RDS(0.999)DT(0.001)YR;YRS(1)PEPDPYLSYR
S(0.003)NS(0.997)EVVEDISTGFR
AT(0.012)S(0.988)PEAGGGGGALK;LSAS(0.992)S(0.008)EDISER;RAPS(1)PDGFSPYSPEETNR
STANQS(1)FRK
RLS(0.937)S(0.032)S(0.032)LNPSK
MQS(1)LQLDCVAVPS(0.587)S(0.413)R
SES(1)PPPLSDPK
AS(0.003)S(0.997)LGEIDESSELR

Polito et al. Universal ready-to-use immunotherapeutic approach for the treatment of cancer: expanded and activated polyclonal γδ memory T cells.

AB Kinome

AAS(0.997)S(0.003)PDGFFWTQGSPK;HHPVFS(0.02)S(0.937)S(0.043)DPLEK;SAS(1)FAFEFPK;TCSDGGPS(0.001)S(0.001)ELAHS(0.935)PT(0.062)NS(0.001)GK;VSQGSAES(0.007)LS(0.956)PS(0.037)FR
FDLS(0.003)HGS(0.997)PQMVR;TES(1)VPSDINNPVDR
KDDVS(1)PVMQFSSK
IKPTGLLTIPS(1)PQI
EQPGEEYS(1)EEEESVLK;TCS(1)LPDLSK
QDS(1)NVNEEWK
IDVPQDLEDDLTATPLS(0.001)HAT(0.012)VPQS(0.987)PAR
NFS(0.998)S(0.002)LHLNSR;SQS(1)NPILGSPFFSHFDGQDSYAAAVR;SSS(1)PTQYGLTK;VSQSALNPHQS(1)PDFK
KNS(1)PGNGSALGPR;LGSQPIRAS(1)NPDLR;QNS(1)DPTSENPPLPTR;SEGS(1)PVLPHEPAK;T(0.001)T(0.002)S(0.997)IS(1)PALAR
ISDYFEYQGGNGS(0.065)S(0.935)PVR;ISDYFEYQGGNGS(0.818)S(0.182)PVR
AQS(1)YPDNHQEFSDYDNPIFEK;RGS(1)DIDNPTLTVMDISPPSR;RLS(1)IIGPTSR
NQMPS(0.999)PT(0.001)STGTPSPGPR
GIAPAS(1)PMLGNASNPNK;LDT(1)FCGSPPYAAPELFQGK;RFS(1)DQAGPAIPTSNSYSK;RNT(1)YVCSER;RYS(1)DHAGPAIPSVVAYPK;S(0.164)T(0.836)FHAGQLR;SRNS(1)PLLER;VPAS(1)PLPGLER
S(0.099)T(0.886)S(0.015)ATDTHHVEMAR
NSQPNRYT(1)NR
TWT(1)LCGTPEYLAPEVIQSK
ILLTEPPMNPT(1)KNR
HFSQT(0.029)S(0.971)R;SLAFEEGS(0.997)QS(0.003)T(0.001)TISSLSEK;STT(1)PANLDSESEHFFR
QET(1)VDCLKK
LIPNATGTGT(0.001)FS(0.998)PGAS(0.001)PGSEAR;S(1)LGPVELLLR;T(0.001)S(0.001)T(0.998)FCGTPEFLAPEVLTDTSYTR;TDVSNFDEEFTGEAPT(1)LS(1)PPR
EEMEMDPKPDLDSDSWCLLGTDSCRPS(1)L;S(0.019)S(0.019)T(0.962)VTEAPIAVVTSR
ALLLRFT(1)GK
TPT(1)PEPAEVETR
QSSGYR;DGSLNQS(0.051)S(0.949)GYR;ES(0.011)ES(0.114)T(0.875)AGSFSLSVR;ILEQS(1)GEWWK;LIEDNEY(0.891)T(0.109)AR;LTEERDGS(0.017)LNQS(0.909)S(0.075)GYR;NLDNGGFY(0.994)IS(0.006)
GEPNVS(0.012)Y(0.987)ICS(0.001)R;T(0.024)S(0.024)S(0.952)FAEPGGGGGGGGGPGGSASGPGGTGGGK;TTS(0.999)FAESCKPVQQPSAFGSMK
IYQY(1)IQSR
ADTTTPTPTAILAPGS(1)PAS(0.984)PPGS(0.016)LEPK;ADTTTPTPTAILAPGS(1)PAS(0.999)PPGS(0.001)LEPK;TAPPALPTGYDS(1)EEEEESRPMSYDEKR
.996)PHS(0.004)PHQLSSK;DVDDGSGS(0.997)PHS(0.003)PHQLSSK;DVDDGSGSPHS(1)PHQLSSK;FIVS(1)PVPESR;KEGPVAS(1)PPFMDLEQAVLPAVIPK;KEKPELSEPSHLNGPS(0.018)S(0.982)DPEAAFLSR;
HS(0.002)S(0.998)PTEERDEPAYPR;LES(1)EDDS(1)LGWGAPDWSTEAGFSR;LES(1)EDDSLGWGAPDWSTEAGFSR
KYES(1)DEDS(1)LGSSGR;KYESDEDS(1)LGSSGR;LEGVDGGQS(1)PR;LLQSAKT(1)ILR
HHSLS(0.998)FS(0.001);S(0.001)CS(0.999)LLS(0.853)T(0.147)EAGALHVLLPAR;S(0.101)CS(0.899)LLS(0.987)T(0.013)EAGALHVLLPAR
DGFPS(0.933)GT(0.067)PALNAK;FYDS(0.964)NT(0.036)VK;GTEAPAVVT(1)EEEDDDEETAPPVIAPRPDHTK;IISIFS(0.893)GT(0.106)EK;S(1)DNGELEDKPPAPPVR;YLS(1)FTPPEK

AB Kinome

AS(1)RDPS(1)PIQDGNEDMK;DPS(1)PIQDGNEDMK
HS(0.008)DS(0.99)IS(0.654)S(0.346)LAS(0.002)ER
S(0.484)S(0.532)PQHS(0.99)LS(0.995)NPLPR
TLFLGIPDENFEDHS(1)APPS(1)PEEK
PEPR;DYSDGRRT(1)FPR;HLS(0.999)VS(0.002)S(0.001)QNPGRS(0.771)S(0.226)PPPGYVPER;HLS(1)VSSQNPGR;LRS(1)ADS(1)ENALSVQER;NVPT(0.011)KS(0.937)PS(0.051)APINWR;SADS(0.994)PS(0.0
DSGS(1)ISLQETR;NTRT(1)IQR;RAS(0.973)S(0.026)DLS(0.001)IASSEEDK;RDS(1)FIGTPYWMAPEVVMCETSK;VDEDS(1)AEDT(0.001)QS(0.999)NDGKEVVEVGQK;VDEDSAEDTQS(1)NDGKEVVEVGQK
AYT(1)HQVVTR;SFGS(1)PNR
AAYK;HYSPEDEPS(1)PEAQPIAAYK;ILS(1)DVTHSAVFGVPASK;LGGAVPFAPPEVS(1)PEQAK;RILS(0.23)DVT(0.764)HS(0.006)AVFGVPASK;VGSLT(1)PPS(0.888)S(0.112)PK;VGSLT(1)PPS(0.891)S(0.109)PK
IGEGT(1)Y(1)GVVYK
IGEGT(1)Y(1)GVVYK
88)PPQQNVQPK;EAPS(1)PLMIHS(1)PQMSQFQS(0.045)LT(0.125)HQS(0.83)PPQQNVQPK;EAPSPLMIHS(0.97)PQMS(0.03)QFQS(0.013)LT(0.155)HQS(0.833)PPQQNVQPK;IHS(1)PIIR;MPDEPEEPVVA
FFS(0.506)S(0.506)KT(0.822)T(0.221)AAHS(0.945)LVGTPYYMSPER;FFS(0.823)S(0.834)KT(0.501)T(0.475)AAHS(0.363)LVGT(0.002)PY(0.001)YMSPER;TTAAHS(1)LVGTPYYMSPER

Polito et al. Universal ready-to-use immunotherapeutic approach for the treatment of cancer: expanded and activated polyclonal γδ memory T cells.

AB Kinome

T: Phospho (STY) Score diffs
57.02)CNT(-107.87)T(-116.8)T(-125.46)EDEDLK;GS(-38.56)T(-18.77)ES(18.77)CNT(-28.79)T(-36.23)T(-45.18)EDEDLK;NFS(78.81)AAKS(78.81)LLK;QET(40.72)VECLRK;S(49)LLKKPDGVK;S(-61.74)T(-5
REDS(52.99)Y(-52.99)HVR
71.92)ET(-71.59)PAVPPVS(69.43)EDEDDDDDAT(-69.43)PPPVIAPRPEHT(-103.05)K;DVAT(-67.34)S(-50.65)PIS(17.74)PT(-17.74)ENNT(-49.16)T(-57.15)PPDALT(-119.07)R;Y(-60.3)MS(33.72)FT(-33.
AS(12.89)LS(-12.89)VAGPGKPQEEDQPLPAR;DLFAFQES(117.93)PPR
GLS(59.34)VDS(-59.34)AQEVK;S(-63.68)QS(63.68)LPGADS(-65.58)LLAKPIDK
LS(91.52)LQER;QGS(91.31)EDNLQGT(-91.31)DPPPVGEEEVLL
S(72.43)PPLGR;NQS(-82.18)LGQS(32.29)APS(-32.29)LT(-46.79)AGLK;RLS(107.21)ADIR;S(110.41)AGNIPLS(-110.41)PLAR;S(-31.52)FS(31.52)EDREEGWER;S(-41.08)DS(41.08)LPS(-61.42)FR;S(-46.37
RMS(74.27)ADMS(-74.27)EIEAR;S(93.61)IQDLT(-93.61)VT(-153.75)GT(-176.48)EPGQVS(-212.22)S(-217.61)R;T(-7.83)AS(7.83)FGNILDVPEIVIS(-188.63)GNGQPR
S(-59.94)PS(59.94)LNLLQNK
NPLPPILGS(10.03)PT(-10.03)K
QIT(-60.75)T(-21.77)S(21.77)PIT(-25.67)VR;S(-39.72)NS(39.72)FNNPLGNR
DKRPLS(52.73)GPDVGT(-52.73)PQPAGLAS(-107.14)GAK;S(73.83)LVGT(-73.83)PY(-191.97)WMAPELIS(-213.31)R
GQGES(-101.82)DPLDHEPAVS(101.82)PLLPR
)R;S(-36.51)T(-36.51)S(20.7)T(-20.7)PNVHMVS(-94.8)T(-100.21)T(-105.03)LPVDS(-131.61)R;S(-49.22)HS(-43.06)ES(-37.13)AS(-30.47)PS(-29.43)ALS(-7.73)S(5.82)S(-5.82)PNNLS(12.8)PT(-12.8)GW
GKDS(80.74)LS(80.74)DDGVDLK
RDS(24.67)S(-24.67)DDWEIPDGQIT(-144.21)VGQR;RDS(-8.34)S(8.34)DDWEIPDGQIT(-68.63)VGQR;S(-33.14)S(-33.14)S(33.14)APNVHINT(-66.34)IEPVNIDDLIR;S(-50.75)AS(50.75)EPS(-54.64)LNR
LPS(-67.02)LS(-22.92)GS(22.92)DGIPY(-81.57)R
S(-32.59)GS(32.59)PS(-32.59)DNS(-60.64)GAEEMEVS(-140.64)LAKPK
CGS(19.65)PS(-19.65)DS(-34.29)S(-44.71)T(-44.71)T(-45.48)EEMEVAVS(-64.47)K
LDPT(-13.72)LS(13.72)VDDLANS(-64.27)GQVGT(-74.01)AR
DKS(36.83)PS(-36.83)S(-42.8)LLEDAK
IT(-49.04)S(49.04)PDKPR
LRT(-18.51)HS(18.51)IES(-41.97)S(-59.44)GK
RDS(29.55)DT(-29.55)Y(-45.18)R;Y(-56.87)RS(56.87)PEPDPY(-81.12)LS(-79.44)Y(-79.44)R
S(-25.03)NS(25.03)EVVEDIS(-116.45)T(-122.79)GFR
AT(-19.32)S(19.32)PEAGGGGGALK;LS(-56.76)AS(20.81)S(-20.81)EDIS(-99.83)ER;RAPS(45.63)PDGFS(-45.63)PY(-73.53)S(-74.05)PEET(-88.24)NR
S(-132.79)T(-121.74)ANQS(121.74)FRK
RLS(14.71)S(-14.71)S(-14.71)LNPS(-45.83)K
MQS(172.77)LQLDCVAVPS(1.52)S(-1.52)R
S(-69.77)ES(69.77)PPPLS(-87.59)DPK
AS(-25.7)S(25.7)LGEIDES(-108.6)S(-114.56)ELR

AB Kinome

.02)QGS(-258.92)PK;HHPVFS(-16.81)S(13.34)S(-13.34)DPLEK;S(-45.83)AS(45.83)FAFEFPK;T(-56.71)CS(-44.51)DGGPS(-29.24)S(-29.24)ELAHS(11.76)PT(-11.76)NS(-30.31)GK;VS(-85.18)QGS(-54.5
FDLS(-25.3)HGS(25.3)PQMVR;T(-48.54)ES(48.54)VPS(-91.13)DINNPVDR
KDDVS(96.72)PVMQFS(-96.72)S(-105.33)K
IKPT(-39.07)GLLT(-40.4)IPS(39.07)PQI
EQPGEEY(-63.31)S(45.65)EEEES(-45.65)VLK;T(-61.68)CS(61.68)LPDLS(-96.61)K
QDS(80.22)NVNEEWK
IDVPQDLEDDLT(-62.84)AT(-55.48)PLS(-32.59)HAT(-19.02)VPQS(19.02)PAR
)LHLNS(-84.02)R;S(-44.37)QS(44.37)NPILGS(-139.96)PFFS(-172.49)HFDGQDS(-210.31)Y(-233.58)AAAVR;S(-51.03)S(-41.96)S(41.96)PT(-47.8)QY(-99.71)GLT(-91.33)K;VS(-170.33)QS(-127.25)ALN
S(51.24)PGNGS(-51.24)ALGPR;LGS(-62.84)QPIRAS(62.84)NPDLR;QNS(98.02)DPT(-98.02)S(-118.1)ENPPLPT(-201.76)R;S(-105.07)EGS(105.07)PVLPHEPAK;T(-28.41)T(-27.72)S(27.72)IS(59.55)PAL
IS(-152.6)DY(-146.41)FEY(-98.93)QGGNGS(6.53)S(-6.53)PVR;IS(-208.26)DY(-194.34)FEY(-144.13)QGGNGS(-11.56)S(11.56)PVR
AQS(92.33)Y(-92.33)PDNHQEFS(-118.81)DY(-159.39)DNPIFEK;RGS(42.73)DIDNPT(-42.73)LT(-50.01)VMDIS(-76.48)PPS(-82.13)R;RLS(50.62)IIGPT(-50.62)S(-55.67)R
NQMPS(30.46)PT(-30.46)S(-35.32)T(-42.16)GT(-55.57)PS(-63.74)PGPR
-193.1)AAPELFQGK;RFS(96.66)DQAGPAIPT(-96.66)S(-103.61)NS(-116.88)Y(-132.92)S(-139.74)K;RNT(68.49)Y(-68.49)VCS(-85.89)ER;RY(-33.56)S(33.56)DHAGPAIPS(-82.2)VVAY(-102.28)PK;S(-7.0
S(-9.53)T(9.53)S(-17.71)AT(-56.89)DT(-72.4)HHVEMAR
NS(-67.24)QPNRY(-48.34)T(48.34)NR
T(-47.18)WT(47.18)LCGT(-110.24)PEY(-264.68)LAPEVIQS(-306.64)K
ILLT(-76.72)EPPMNPT(76.72)KNR
HFS(-55.78)QT(-15.26)S(15.26)R;S(-101.22)LAFEEGS(25.58)QS(-25.58)T(-32.86)T(-40.1)IS(-54.45)S(-61.55)LS(-75.58)EK;S(-46.72)T(-46.72)T(46.72)PANLDS(-154.49)ES(-169.33)EHFFR
QET(94.41)VDCLKK
S(28.67)PGAS(-28.67)PGS(-41.46)EAR;S(143.79)LGPVELLLR;T(-100.07)DVS(-98.87)NFDEEFT(-39.14)GEAPT(39.14)LS(74.8)PPR;T(-29.09)S(-29.09)T(29.09)FCGT(-80.83)PEFLAPEVLT(-222.4)DT(-22
EEMEMDPKPDLDS(-136.54)DS(-137.11)WCLLGT(-55.28)DS(-33.32)CRPS(33.32)L;S(-17.06)S(-17.06)T(17.06)VT(-51.34)EAPIAVVT(-98.93)S(-98.93)R
ALLLRFT(75.11)GK
T(-68.44)PT(68.44)PEPAEVET(-136.74)R
.43)S(-168.92)GY(-204.58)R;ES(-18.92)ES(-8.87)T(8.87)AGS(-33.19)FS(-47.27)LS(-58.45)VR;ILEQS(131.17)GEWWK;LIEDNEY(9.11)T(-9.11)AR;LT(-67.42)EERDGS(-17.35)LNQS(10.85)S(-10.85)GY(
(-19.16)Y(19.16)ICS(-28.28)R;T(-16.01)S(-16.01)S(16.01)FAEPGGGGGGGGGPGGS(-131.25)AS(-141.88)GPGGT(-163.66)GGGK;T(-35.27)T(-35.27)S(35.27)FAES(-54.34)CKPVQQPS(-92.64)AFGS(
IY(-54.03)QY(50.33)IQS(-50.33)R
)PT(-92.52)AILAPGS(58.82)PAS(17.87)PPGS(-17.87)LEPK;ADT(-99.96)T(-91.8)T(-92.7)PT(-78.05)PT(-70.01)AILAPGS(54.09)PAS(28.3)PPGS(-28.3)LEPK;T(-86.52)APPALPT(-44.73)GY(-53.22)DS(40.0
)PHS(-25.05)PHQLS(-50.02)S(-58.37)K;DVDDGS(-84.02)GS(-52.94)PHS(52.94)PHQLS(-82.83)S(-91.86)K;FIVS(54.03)PVPES(-54.03)R;KEGPVAS(100.95)PPFMDLEQAVLPAVIPK;KEKPELS(-69.85)EPS(
HS(-27.73)S(27.73)PT(-49.1)EERDEPAY(-141.91)PR;LES(50.98)EDDS(50.98)LGWGAPDWS(-50.98)T(-56.34)EAGFS(-67.45)R;LES(73.04)EDDS(-73.04)LGWGAPDWS(-246.57)T(-253.5)EAGFS(-270)R
KY(-198.27)ES(-105.86)DEDS(46.8)LGS(-46.8)S(-54.54)GR;KY(-79.71)ES(79.71)DEDS(36.36)LGS(-36.36)S(-50.89)GR;LEGVDGGQS(94.78)PR;LLQS(-36.91)AKT(36.91)ILR
HHS(-36.85)LS(28.41)FS(-28.41);S(-31.37)CS(31.37)LLS(7.64)T(-7.64)EAGALHVLLPAR;S(-9.49)CS(9.49)LLS(18.91)T(-18.91)EAGALHVLLPAR
)PALNAK;FY(-81.13)DS(14.31)NT(-14.31)VK;GT(-105.84)EAPAVVT(94.01)EEEDDDEET(-94.01)APPVIAPRPDHT(-143.59)K;IIS(-38.72)IFS(9.24)GT(-9.24)EK;S(159.13)DNGELEDKPPAPPVR;Y(-132.37)L

Polito et al. Universal ready-to-use immunotherapeutic approach for the treatment of cancer: expanded and activated polyclonal γδ memory T cells.

AB Kinome

AS(106.94)RDPS(106.94)PIQDGNEDMK;DPS(56.55)PIQDGNEDMK
HS(-21.16)DS(21.16)IS(2.78)S(-2.78)LAS(-26.41)ER
S(-0.42)S(0.42)PQHS(17.14)LS(19.78)NPLPR
T(-149.44)LFLGIPDENFEDHS(149.44)APPS(168.16)PEEK;T(-163.63)LFLGIPDENFEDHS(163.63)APPS(185.95)PEEK
5.89)FPR;HLS(34.26)VS(-27.09)S(-27.98)QNPGRS(5.32)S(-5.32)PPPGY(-35.49)VPER;HLS(34.61)VS(-34.61)S(-40.21)QNPGR;LRS(136.97)ADS(86.66)ENALS(-86.66)VQER;NVPT(-19.18)KS(12.62)PS(-
51.24)IQR;RAS(15.65)S(-15.65)DLS(-30.6)IAS(-49.16)S(-52.95)EEDK;RDS(38.12)FIGT(-38.12)PY(-59.81)WMAPEVVMCET(-158.29)S(-160.73)K;VDEDS(-133.24)AEDT(-49.27)QS(49.27)NDGKEVVEV
AY(-91.55)T(91.55)HQVVT(-124.83)R;S(-99.34)FGS(99.34)PNR
36)PEAQPIAAY(-243.31)K;ILS(63.08)DVT(-63.08)HS(-121.48)AVFGVPAS(-257.84)K;LGGAVPFAPPEVS(56.34)PEQAK;RILS(-5.21)DVT(5.21)HS(-21.13)AVFGVPAS(-85.9)K;VGS(-51.27)LT(51.27)PPS(9.
IGEGT(172.84)Y(110.73)GVVY(-110.73)K;IGEGT(198.67)Y(129.09)GVVY(-129.09)K
IGEGT(172.84)Y(110.73)GVVY(-110.73)K;IGEGT(198.67)Y(129.09)GVVY(-129.09)K
.14)PLMIHS(34.13)PQMS(-34.13)QFQS(-12.65)LT(-8.24)HQS(8.24)PPQQNVQPK;EAPS(66.51)PLMIHS(24.46)PQMS(-24.46)QFQS(-13.06)LT(-1.87)HQS(1.87)PPQQNVQPK;IHS(80.79)PIIR;MPDEPEE
(11.56)LVGT(-50.6)PY(-74.59)Y(-79.51)MS(-87.73)PER;FFS(7.1)S(7.5)KT(0.43)T(-0.43)AAHS(-2.09)LVGT(-31.91)PY(-38.55)Y(-44.45)MS(-54.59)PER;T(-39.63)T(-39.63)AAHS(39.63)LVGT(-63.39)PY(

Polito et al. Universal ready-to-use immunotherapeutic approach for the treatment of cancer: expanded and activated polyclonal γδ memory T cells.

AB Kinome

T: Position in peptide	T: Unique identifier
1;3;5	UID11927;UID11928;UID19558;UID19559;UID341;UID342
4	UID1040
12;3;8	UID1044;UID1045;UID1047
2;8	UID1521;UID1522
3	UID1700;UID1701
2;3	UID2283;UID9524
1;2;3;4;6;7	UID16232;UID16233;UID2356;UID2358;UID2360;UID2361;UID2919;UID2925;UID2926;UID2930;UID7490;UID7491;UID7492;UID7493
1;3	UID2794;UID2795;UID2796
3	UID2797
9	UID3082
3;5	UID3430;UID3431
1;6	UID3447;UID3448
15	UID3456
19;3;6	UID16801;UID3496;UID3499;UID3736
6	UID3644
3;4	UID2182;UID2183;UID2184;UID3901
7	UID4122
3	UID4409
3	UID4412
6	UID4540
3	UID4699
3	UID4719
5	UID4732
3	UID4922;UID4926
3	UID5740
3;4	UID6085;UID6087;UID6088
6	UID6189
3	UID6241
3	UID6246
3	UID6644
3	UID6817

AB Kinome

10;14;3;7	UID8157;UID8159;UID8161;UID8162;UID8163
3;7	UID8196;UID8197
5	UID8310
11	UID8780
3;8	UID9485;UID9486
3	UID9668
24	UID10588
11;3	UID10761;UID10762;UID10763;UID10764
3;4;5;9	UID11130;UID11131;UID11132;UID11133;UID11135
13;14	UID11141;UID11142
3	UID11580;UID11581;UID11582
5	UID11713
2;3;4;6	UID11931;UID11932;UID12053;UID1466;UID1467;UID1471;UID385;UID387
2	UID12180
8	UID12512
3	UID12515
11	UID12552
3;6;8	UID12661;UID6127;UID6128
3	UID12679
1;12;16;18;3	UID12738;UID12739;UID6812;UID6815;UID6816
27;3	UID12922;UID8744
7	UID13039
3	UID13132
12;14;3;5;7;8	UID12290;UID13241;UID13252;UID13253;UID16879;UID16880;UID3571;UID3574;UID3576;UID3577;UID3578
3;7	UID13276;UID4965;UID4966
4	UID13285
12;16;19	UID13870;UID13871;UID567;UID568;UID569
11;17;3;4;6;7;8	UID15014;UID15015;UID1712;UID1713;UID1714;UID1715;UID1716;UID1718
3;7	UID15065;UID15066;UID1761;UID1762
4;7;8;9	UID12168;UID15831;UID15832;UID2525
3;5;6	UID18395;UID18396;UID5091
1;3;4;5;6;9	UID12655;UID19362;UID19370;UID6060;UID6062;UID6065;UID6067

AB Kinome

2;3	UID20127;UID20128;UID6825
4	UID20420
8	UID21340;UID8037
15;19	UID22994;UID22995;UID9692
12;2;3;4;6;8	UID13009;UID23013;UID23014;UID23015;UID23016;UID9708;UID9709;UID9713;UID9714;UID9715;UID9716
11;3;4;5	UID10195;UID10197;UID10200;UID10203;UID10204;UID13064;UID23506;UID23507
3;4	UID18297;UID25814;UID4994
13;3;4;5;7;8;9	UID12753;UID12754;UID20203;UID236;UID237;UID239;UID26055;UID26056;UID6898
5;6	UID12099;UID13239;UID25402;UID26542
5;6	UID12297;UID13255;UID25600;UID26558
10;13;23;3;4;5	UID16308;UID16309;UID29610;UID29611;UID3007;UID3008;UID3011
3;4;6	UID35372;UID35373;UID35374;UID39529
