# Supplementary material for: Universal Ready-to-Use Immunotherapeutic Approach for the Treatment of Cancer: Expanded and Activated Polyclonal γδ Memory T Cells
Source: Front Immunol. 2019 Nov 22;10:2717. doi: 10.3389/fimmu.2019.02717 (PMC6883509; doi:10.3389/fimmu.2019.02717)
Supplement: Supplementary file 14 [file Data_Sheet_1.docx]

**SUPPLEMENTARY FIGURE LEGENDS**

**SUPPLEMENTARY FIGURE 1: Schematic representation of the viral vectors used in the study.**

Retroviral and lentiviral vectors used to transduce aAPCs (**A**). Retroviral vector encoding GFP and Firefly luciferase used to transduce tumour cell lines (**B**). Retroviral vector used to transduce primary expanded γδ-T cells: third generation CAR.GD2 (**C**).

**SUPPLEMENTARY FIGURE 2: Comparison of γδ-T cell subpopulations in PBMC from HD and CD40L/pp65 expanded γδ-T cells.**

Percentage of CD4^+^, CD8^+^ and CD4CD8^--^ cells, as well as vδ1^+^, vδ2^+^ and vδ1^-^ vδ2^-^ cells in the γδ-T cell population of a representative example of healthy donor PBMC compared to in vitro expanded CD40L/pp65 γδ-T cells.

**SUPPLEMENTARY FIGURE 3: Short- and long-term *in vitro* assays for the evaluation of the functionality of freshly isolated γδ-T cells and zoledronic acid expanded γδ-T cells.**

The *in vitro* cytolytic activity was evaluated by ^51^Cr release assay at different E:T ratios of polyclonally activated αβ-T cells (black dotted line) and freshly isolated γδ-T cells (black line), versus Daudi tumour cell line (**A**) or allogeneic B cells (**B**). *In vitro* long-term anti-tumour assay by 3 days co-culture of polyclonally activated αβ-T cells and freshly isolated (**C**) or zoledronic acid expanded γδ-T cells (**D-E**) at E:T ratios of 1:1 and 5:1. Daudi and SHSY5Y cell lines were used as target. Data are summarized as average ± SEM of 3-4 donors.

**SUPPLEMENTARY FIGURE 4: *In vitro* functional assays of γδ-T cells stimulated with CD40L/pp65.**

*In vitro* long-term 3- and 6-day co-culture assays with activated αβ-T cells and CD40L/pp65 γδ-T cells at E:T ratio 1:1 and 5:1. MV411, U87 and SHSY5Y tumour cell lines were used as targets. Six-day co-culture experiments were also performed in presence of IL2/15 (**A**). Evaluation of IFN-γ secretion in supernatants of co-culture experiments by ELISA assay (**B**). Degranulation assay evaluating the CD107a expression of γδ_vδ1, γδ_vδ2 and γδ_vδ1^-^/vδ2^-^ in co-culture with a panel of CMV-pp65 pepmix (**C**) and EBV infected B cells (**D**) *In vitro* long-term anti-tumour assay by 3 day co-culture with polyclonally activated αβ-T cells, γδ_vδ1, γδ_vδ2 and γδ_vδ1^-^/vδ2^-^ T cells at E:T ratios of 1:1 and 5:1 against Daudi cells (**E**). Data are summarized as average ± SEM of 4 donors. *p<0.05; **p<0.01; ***p<0.001.

**SUPPLEMENTARY FIGURE 5: Kinoma analysis.**

Expanded activated αβ-T cells (**A**) and CD40L/pp65 γδ-T cells (**B**) were analysed for their kinoma profile at day+18 of expansion.

**SUPPLEMENTARY FIGURE 6: Real-Time PCR OpenArray data analysis of γδ CD40L/pp65 and polyclonally activated αβ-T cells.**

Panel **A** shows the heatmap of the significant modulated pathways emerging from the analysis. Tables in panel **B** show the down- and up-regulated pathways arising from the OpenArray data. Data from 4 donors are shown.

**SUPPLEMENTARY FIGURE 7: Identity of the expanded γδ-T cell products.**

At day+28 of expansion, CD40L/pp65 γδ-T cell products have been characterized in order to detect residual aAPCs. Short tandem repeat analysis has been carried out on 4 different γδ-T cell products (**A**). Representative phenotype analysis for the detection of aAPC markers is shown in panel **B**.

**SUPPLEMENTARY FIGURE 8: Functional characterization of frozen expanded γδ-T cells.**

*In vitro* long-term 3-day co-culture assay with activated αβ-T cells and thawed CD40L/pp65 expanded γδ-T cells at E:T ratio 1:1 and 5:1. Daudi and SHSY5Y tumour cell lines were used as targets (**A** and **B** respectively). IFN-γ release assay is shown in panel **C**. Data from 3 donors are expressed as average ± SEM. ***p<0.001.
