# Supplementary material for: Universal Ready-to-Use Immunotherapeutic Approach for the Treatment of Cancer: Expanded and Activated Polyclonal γδ Memory T Cells
Source: Front Immunol. 2019 Nov 22;10:2717. doi: 10.3389/fimmu.2019.02717 (PMC6883509; doi:10.3389/fimmu.2019.02717)
Supplement: Supplementary file 15 [file Data_Sheet_2.docx]

**Supplemental Method**

**Phosphoproteome analysis**.

Cells were lysed, solubilized, denatured and reduced using a solution of 6M GdmCl, 10 mM TCEP, 40 mM CAA, 100 mM Tris pH 8.5. Afterward, the samples were loaded into a 30 kDa filtration device and mixtures of sequencing grade of Lys C and Trypsin were added at a ratio of 1:50 and 1:100 (µg enzyme: µg protein), respectively. After overnight digestion at 37°C, peptides were collected with one wash of 50% CH_3_OH, 45% H_2_O, 5% TFA. Of each sample, a 5 µg aliquot was preserved for proteome analysis. After this step, on the remaining amount, the phosphopeptides were enriched and then purified, following the guidelines outlined in Titansphere Phos-TiO kit (GL Sciences Cat. No. 5010–21312). The enrichment samples were acidified and desalted on C18 StageTips before injection to mass spectrometry. The peptide mixtures were separated on an Easy-Spray C18 LC column (75-µm ID×50 cm, 2 µm, 100 Å) thermostated at 55°C with a non-linear gradient of 2–60% solution B (80% CAN and 20% H_2_O, 5% DMSO, 0.1% FA) in 180 min with a flow rate of 250 nl/min. Eluting peptides were analyzed using an Orbitrap Fusion Tribrid mass spectrometer (Thermo Scientific Instruments). Orbitrap detection was used for both MS1 and MS2 measurements at resolving powers of 120 K and 50 K (at m/z 200), respectively. Data-dependent MS/MS analysis was performed in top speed mode with a 1.5 sec. cycle time, during which precursors detected within the range of m/z 375−1500 were selected for activation in order of abundance. Quadrupole isolation with a 1.8 m/z isolation window was used, and dynamic exclusion was enabled for 20 s. Automatic gain control targets were 4×10^5^ for MS1 and 5×10^4^ for MS2, with 50 and 86 ms maximum injection times, respectively. The signal intensity threshold for MS2 was 1×10^4^. HCD was performed using 28% normalized collision energy. Data analysis and phosphopeptide quantification were done using the Maxquant v1.6.10 software([32](#_ENREF_32)), according to the basic settings, using a human protein database (UniProt release 08/2017). The data were subsequently filtered accepting only high confidence quantitative identifications (high localization probability >0.75). In order to reconstruct the altered pathways in our experimental conditions, we used the Photon plugin implemented in the Perseus v1.6.22 software([33](#_ENREF_33)) platform, following the workflow described in the reference paper([34](#_ENREF_34)). The data obtained were then processed using a One-way ANOVA with an FDR <0.01 and the resulting significant proteins used, via Fisher exact test with a Benjamini-Hochberg FDR <0.02, to determine the enrichment in the KEGG pathway for each group of samples. The data was then plotted using alluvial plot([35](#_ENREF_35)). Coral was the tools for visualizing kinase data, extrapolated from the quantified phosphosite, in the human kinome([36](#_ENREF_36)). The mass spectrometry proteomics data have been deposited to the ProteomeXchange Consortium via the PRIDE([37](#_ENREF_37)) partner repository with the dataset identifier PXD015506.
