# Supplementary material for: Universal Ready-to-Use Immunotherapeutic Approach for the Treatment of Cancer: Expanded and Activated Polyclonal γδ Memory T Cells
Source: Front Immunol. 2019 Nov 22;10:2717. doi: 10.3389/fimmu.2019.02717 (PMC6883509; doi:10.3389/fimmu.2019.02717)
Supplement: Supplementary file 9 [file Table_1.PDF]

Polito et al. Universal ready-to-use immunotherapeutic approach for the treatment of cancer: expanded and activated polyclonal γδ memory T cells.
Phosphorylation sites

AB1_t0	AB2_t0	AB3_t0	AB1_Dp18	AB2_Dp18	AB3_Dp18	AB4_Dp18	GD1_t0	GD2_t0	GD3_t0
-2,19256	-2,05728	-2,024	-1,6361	-2,05769	-1,26469	-0,678276	-1,64223	-1,98785	-1,61967
NaN	NaN	NaN	-2,86992	-2,25814	NaN	-1,82017	-1,91001	-0,57087	-2,03253
-1,51047	-0,59329	-0,976307	-0,111893	-1,30205	-1,10946	-0,578295	-0,888699	-1,23223	-0,971719
NaN	NaN	NaN	NaN	NaN	-1,3083	NaN	NaN	NaN	NaN
-2,58091	NaN	-2,12579	-2,12174	-2,4352	NaN	NaN	-1,90645	-2,14996	-1,73152
1,58289	1,33883	1,22861	2,09629	0,97208	1,89178	0,388697	0,150818	0,535201	0,626445
NaN	NaN	NaN	0,142004	NaN	-1,1995	-0,198769	NaN	NaN	NaN
-0,0301552	-0,372684	0,429108	0,843504	0,637074	-1,15002	1,95026	-0,325888	-0,797595	0,277864
-0,455448	-0,21442	0,181562	0,629436	0,443945	NaN	1,23293	-0,0740528	0,267947	0,329602
NaN	NaN	NaN	-0,892609	NaN	0,242437	-1,44097	NaN	NaN	NaN
NaN	NaN	NaN	-0,802614	-2,94135	0,266945	-3,94244	NaN	NaN	NaN
-2,02241	NaN	-1,0719	-0,992025	NaN	-0,707998	-1,19122	NaN	NaN	NaN
-0,967644	0,177864	NaN	-1,66047	0,631138	NaN	-2,34895	-0,665897	NaN	-0,458623
-1,32872	-0,818808	-1,04039	-1,33574	NaN	-0,605127	-1,14764	NaN	NaN	NaN
NaN	NaN	NaN	-2,52496	-3,33912	-3,43493	-4,56014	NaN	NaN	NaN
NaN	NaN	NaN	NaN	NaN	-0,988588	-2,88656	NaN	NaN	NaN
-1,32928	-1,93644	-1,91295	0,241247	NaN	-0,438335	NaN	NaN	NaN	NaN
NaN	-3,14255	-0,980106	NaN	NaN	NaN	NaN	0,173155	-0,0828085	0,88366
NaN	NaN	0,229427	-1,69833	1,64708	-1,84846	-1,33722	0,975574	0,982877	0,782561
0,1567	0,299049	-1,47686	1,03482	-0,0181122	0,21224	0,225939	0,361366	-0,303588	-0,487418
NaN	NaN	NaN	-1,26256	-1,45836	-1,62111	NaN	NaN	NaN	NaN
NaN	-1,56656	NaN	1,3036	NaN	0,233292	1,20713	NaN	-0,958537	0,0371428
NaN	NaN	NaN	NaN	NaN	NaN	NaN	NaN	NaN	NaN
NaN	NaN	NaN	-1,94332	-2,57913	-1,06508	-2,28015	NaN	NaN	NaN
-0,0979328	-0,590191	0,522242	0,712999	0,703512	0,908161	0,308376	1,1462	0,287858	0,454064
NaN	-1,71035	NaN	NaN	NaN	NaN	NaN	NaN	NaN	NaN
1,27483	0,725155	1,22154	1,5633	0,577694	0,625103	1,10205	1,33531	1,58937	0,593167
NaN	NaN	NaN	NaN	-2,47066	NaN	NaN	-1,65734	-1,55497	-2,29691
-0,885643	NaN	NaN	NaN	NaN	NaN	NaN	-0,0856514	-1,01259	-0,0139856
3,15706	NaN	2,63728	NaN	3,11119	0,313974	2,3404	2,81756	2,71128	2,54489
NaN	NaN	NaN	NaN	-0,553507	-2,77243	-2,09309	NaN	NaN	NaN
NaN	NaN	-1,18152	NaN	NaN	NaN	NaN	-1,37091	-1,42516	-1,88571
-0,171589	1,98159	0,948914	0,0347271	0,832834	NaN	1,54941	0,390409	0,599418	1,93821

Polito et al. Universal ready-to-use immunotherapeutic approach for the treatment of cancer: expanded and activated polyclonal γδ memory T cells.
Phosphorylation sites

NaN	-0,481056	NaN	-3,1136	-1,13688	NaN	-2,39299	NaN	NaN	NaN
0,147781	NaN	-0,298704	0,217588	0,223881	0,904964	-0,0200272	NaN	NaN	NaN
-1,06669	-1,05172	0,0306625	-0,542469	-1,64254	-0,538097	-0,831619	NaN	NaN	-1,10971
NaN	-1,08187	-1,12228	-1,31309	-1,33066	NaN	-1,07043	NaN	NaN	NaN
0,667324	-0,0472507	1,12562	0,110699	0,915998	0,67909	0,714172	-0,337406	1,16347	0,0127153
2,85579	3,8679	1,93579	4,13614	2,85345	3,33184	3,9807	2,93173	3,01738	2,75543
3,66878	3,73253	4,00541	4,56383	0,884081	-1,187	4,21232	3,46264	0,808366	3,11869
4,18835	4,43638	4,23639	4,86815	3,84705	4,91008	4,70498	4,25764	4,22918	4,12883
NaN	-1,80168	-2,5626	-0,503822	NaN	0,218021	-0,743565	NaN	NaN	NaN
NaN	NaN	NaN	-3,49939	NaN	NaN	-3,04975	NaN	NaN	NaN
NaN	NaN	NaN	-2,48448	NaN	-0,912609	-3,13934	NaN	NaN	NaN
NaN	NaN	NaN	-1,41446	-1,9973	-0,750408	NaN	NaN	NaN	NaN
0,50441	0,969791	0,339411	0,56864	-0,217567	0,896042	0,789282	0,208857	-0,267392	-0,0597506
1,5089	2,32191	1,66167	1,97056	0,375175	2,2435	2,51433	1,63607	1,30615	1,18083
NaN	NaN	NaN	-2,59531	NaN	-0,948305	-3,58814	NaN	NaN	NaN
NaN	NaN	NaN	NaN	NaN	NaN	NaN	NaN	NaN	NaN
-2,53504	-1,44164	-1,99879	-1,33616	-1,77376	-2,12007	-0,612566	NaN	NaN	NaN
-1,2998	-0,63422	-1,30655	-1,50693	NaN	-1,96995	-1,3506	-1,109	-0,701081	-0,901404
-0,584148	0,621231	-0,358431	-0,69478	-0,338472	NaN	0,37327	-0,222044	1,6882	0,412856
NaN	NaN	NaN	-2,73604	-3,39542	-2,92207	NaN	NaN	NaN	NaN
-1,70415	-1,82225	-1,92168	-0,505362	-1,33252	0,211205	-0,265244	-0,784491	-0,938079	-0,952495
-3,05912	-4,08099	0,0204372	0,682056	NaN	0,521053	1,01084	0,440876	0,293826	-0,0166292
NaN	NaN	-0,102661	0,861219	NaN	0,374201	1,10704	NaN	NaN	NaN
0,232294	0,075861	0,785875	-0,322418	0,98798	-0,16593	0,577929	0,274004	0,1732	0,214362
-0,0783825	-0,833918	0,160269	0,169903	0,6486	-0,580761	0,951181	-0,403139	-0,0505686	0,130225
-0,115479	-0,0313396	-0,711224	0,316437	0,705135	0,720261	1,02321	0,764292	0,121388	0,296126
0,889254	3,62401	1,04573	1,99649	1,42397	2,48811	1,92376	0,515358	1,88238	1,70697
0,808973	NaN	NaN	1,39366	1,3383	1,45811	1,47073	NaN	NaN	NaN
-0,99332	0,536879	-0,688669	0,276201	-1,30458	-0,694031	0,793543	NaN	0,123424	NaN
NaN	NaN	2,84996	NaN	2,852	2,07191	NaN	2,47083	2,00491	2,04551
4,18528	3,8779	4,18861	3,97025	3,57523	4,27361	4,0047	4,15076	3,89829	3,76391
NaN	1,61976	NaN	1,41229	0,747446	-1,14037	1,73251	-0,316042	0,894401	0,0715303
-0,701818	-0,215868	-0,622726	-0,065834	0,0184479	-0,638502	-0,518587	-0,463663	-0,486503	-0,925
-1,09865	-1,68548	-0,992579	-0,335024	-1,10363	-0,314011	-0,448114	-1,24669	-1,82545	NaN

Polito et al. Universal ready-to-use immunotherapeutic approach for the treatment of cancer: expanded and activated polyclonal γδ memory T cells.
Phosphorylation sites

1,31342	1,35231	1,15027	0,95381	-0,0303707	-1,17145	1,19077	0,674738	1,46398	0,28741
NaN	NaN	NaN	-1,28205	-4,22839	-0,724417	NaN	NaN	NaN	NaN
NaN	NaN	NaN	NaN	NaN	NaN	NaN	0,568666	1,03445	0,851209
0,869232	-0,292271	0,867142	NaN	0,234772	NaN	NaN	0,235054	0,541696	0,469609
0,897785	-0,17692	0,348513	NaN	-0,0131683	NaN	NaN	1,02372	1,18699	0,833802
NaN	0,526426	0,335604	NaN	0,180786	NaN	0,862642	0,216776	-0,0405569	0,218629
-0,674845	-0,370026	-0,170675	-0,73451	-1,41253	-0,00805092	0,188345	-0,32398	-3,47832	-0,802548
0,190363	0,0566387	-0,747631	0,408489	-0,266012	0,801163	0,677055	-0,513102	NaN	-0,941478
NaN	NaN	NaN	-1,27736	-1,68113	NaN	-0,514496	NaN	NaN	NaN
NaN	NaN	-1,34593	NaN	NaN	NaN	NaN	-0,603476	-0,593314	-1,03883
-0,496016	-0,112413	-0,182821	0,786137	-0,0556507	1,39963	-0,03195	0,196394	-0,366181	-0,727218
2,66732	2,94119	2,59381	3,00389	1,98245	3,54139	2,98415	2,72446	2,45253	2,34018
2,60004	2,6312	2,40734	3,04487	2,82593	-2,26403	3,07666	2,71868	2,50986	2,48831
-1,81624	NaN	-1,25697	-0,347647	-1,31756	0,785675	-0,956156	-1,9266	-1,5644	-1,51779
1,44139	1,29207	2,00496	3,02796	1,73469	3,44334	1,28439	1,03265	0,897819	1,27231
3,13878	0,593933	1,90255	4,80787	3,37398	3,24476	4,30206	NaN	NaN	NaN
1,94834	1,8536	1,99431	2,36405	2,50254	2,30285	1,65976	2,66809	2,18235	2,12662
NaN	NaN	-1,58704	-2,61443	-1,36547	NaN	-1,96647	NaN	NaN	NaN
NaN	-2,48258	NaN	-1,58303	-1,53464	-1,33245	-0,0876637	-0,803215	-1,00289	NaN
0,23715	-0,452381	-1,55742	-0,327093	-0,759394	0,760855	0,197515	-1,09561	-1,13318	-1,46678
NaN	NaN	NaN	-1,46873	0,952923	0,19758	0,259687	-0,578381	NaN	-0,0208368
3,45095	1,0602	2,24185	1,74115	2,93706	1,7942	2,53722	2,03174	1,91885	2,46771
-1,19179	NaN	NaN	NaN	-0,696529	1,11191	NaN	NaN	NaN	NaN
NaN	NaN	NaN	NaN	NaN	0,689234	NaN	-0,533716	-0,101985	-2,1215
-1,87931	-1,52638	NaN	-0,883221	-0,746408	-1,87081	NaN	NaN	NaN	NaN
0,555939	-0,366205	0,755186	1,25328	-0,0581646	1,50624	0,631016	NaN	NaN	NaN
NaN	NaN	NaN	-0,916559	NaN	-1,30028	-2,03479	NaN	NaN	NaN
NaN	NaN	NaN	-1,6459	NaN	-1,49237	2,08171	-1,11255	-0,930163	0,0966234
-0,205933	NaN	-0,377001	-0,265717	NaN	0,233088	-0,729935	NaN	NaN	NaN
-2,10526	-1,02272	-1,98354	NaN	NaN	-1,77218	-1,04734	-1,69528	-2,0191	-1,45724
0,348589	-1,54349	0,0332737	0,844835	NaN	-1,50714	0,217987	0,127855	-0,119212	-0,938352
-1,58171	NaN	-2,0897	NaN	-1,52283	NaN	NaN	NaN	NaN	-1,39616
NaN	NaN	NaN	-3,06307	NaN	-3,36227	-3,34445	NaN	NaN	NaN
NaN	NaN	NaN	-2,47076	NaN	-2,12017	-3,08899	NaN	NaN	NaN

Polito et al. Universal ready-to-use immunotherapeutic approach for the treatment of cancer: expanded and activated polyclonal γδ memory T cells.
Phosphorylation sites

2,36996	2,27752	2,7024	4,58482	1,14639	5,4815	3,74776	2,1689	2,93958	2,277
1,9	1,53843	2,51071	4,76335	2,23434	4,49946	5,10972	3,57777	3,44198	3,11544
0,211548	0,901047	1,24668	1,5297	0,137423	0,0066967	1,94771	2,16896	2,04124	1,9188
-2,17612	-1,82197	-0,763412	0,624676	NaN	0,472046	-0,402679	-0,0202446	-0,19473	0,258657
NaN	0,572094	NaN	NaN	1,09634	NaN	NaN	-0,587759	-3,62886	0,970195
1,73995	4,79845	3,11188	1,4979	5,08579	0,244057	4,02365	3,1925	2,92616	4,89586
1,18256	NaN	3,36305	NaN	NaN	NaN	NaN	2,62511	4,47861	3,59522
1,10176	0,681919	3,56811	2,63681	1,87385	2,99535	2,77414	3,33593	2,5259	2,63532
-2,14479	-3,5153	-1,03395	-1,22568	-1,99367	NaN	NaN	-0,252859	-0,776713	-0,543176
1,02706	-0,597504	0,985771	1,87065	-1,52357	2,29861	2,66773	1,67619	-1,23064	1,49317
NaN	NaN	2,58875	NaN	NaN	3,21552	1,73661	NaN	2,89194	NaN
-2,98291	NaN	-2,02892	3,02247	2,1206	-1,25153	-2,58304	2,93346	NaN	2,63299
NaN	NaN	NaN	NaN	NaN	NaN	NaN	NaN	NaN	NaN
NaN	NaN	NaN	-0,820974	NaN	-0,758232	-0,271542	NaN	NaN	NaN
NaN	NaN	NaN	-1,37363	NaN	-1,05543	-0,493799	NaN	NaN	NaN
NaN	NaN	NaN	-0,310812	NaN	-0,665049	-1,62875	NaN	NaN	NaN
-1,46842	NaN	NaN	0,0992813	NaN	0,305466	0,526543	NaN	-2,56786	NaN
NaN	NaN	NaN	-1,92722	NaN	-3,53946	-1,75944	NaN	NaN	NaN
NaN	NaN	NaN	-2,00735	-1,76978	NaN	-1,65722	-2,05235	-2,15617	-2,54605
NaN	NaN	-1,71105	-1,69392	-3,16125	-0,143255	-0,660011	NaN	NaN	NaN
NaN	NaN	1,8014	2,15253	-2,81061	0,238379	2,06835	NaN	NaN	NaN
-0,00769997	-0,154118	0,998877	-4,22182	0,873705	0,51503	0,5725	1,1244	1,34921	1,40388
1,17407	2,13864	0,531187	NaN	0,560715	-1,7061	0,513372	NaN	1,51029	1,66706
NaN	NaN	-1,23166	-1,37587	NaN	-1,11106	-1,51606	NaN	NaN	NaN
-0,997726	NaN	NaN	-0,773508	NaN	-0,418213	NaN	NaN	NaN	-1,28127
0,896465	1,45037	0,605803	NaN	NaN	0,242538	0,00495338	0,502335	0,52255	0,358207
NaN	NaN	NaN	-0,00674248	NaN	-0,21697	-0,324175	-2,07308	-1,56555	-2,07241
NaN	NaN	NaN	1,89774	-1,19422	2,25052	-0,309229	NaN	NaN	NaN
NaN	NaN	NaN	-2,02124	NaN	-1,17035	-1,11519	NaN	NaN	NaN
1,95653	3,18225	1,86138	1,32547	2,08927	1,90771	2,61971	1,4624	0,91467	1,22822
NaN	NaN	NaN	NaN	NaN	NaN	NaN	NaN	NaN	NaN
1,41468	1,67657	1,84996	1,76509	-0,370827	2,12185	2,64651	1,34241	1,1776	1,24836
2,36043	1,43743	1,32229	2,85742	1,40638	2,84275	-1,25315	NaN	NaN	NaN
NaN	NaN	NaN	-0,553326	-1,24608	NaN	-1,20646	NaN	NaN	NaN

Polito et al. Universal ready-to-use immunotherapeutic approach for the treatment of cancer: expanded and activated polyclonal γδ memory T cells.
Phosphorylation sites

NaN	NaN	NaN	0,158354	NaN	0,338158	-0,485546	NaN	NaN	NaN
NaN	NaN	NaN	-0,977221	-1,54572	-1,01557	-1,22326	NaN	NaN	NaN
0,212685	NaN	1,058	NaN	NaN	NaN	NaN	-0,324762	-0,62893	-0,346005
NaN	NaN	NaN	-0,522104	-0,175152	-2,8304	0,285952	NaN	NaN	NaN
NaN	NaN	NaN	NaN	NaN	NaN	NaN	NaN	NaN	NaN
NaN	NaN	NaN	-0,177069	NaN	1,42774	-0,574329	NaN	NaN	NaN
NaN	NaN	NaN	-0,450775	-0,677851	NaN	-1,88547	NaN	NaN	NaN
NaN	NaN	NaN	-1,70311	-2,36975	NaN	NaN	-2,33084	-2,63443	-3,03653
0,188797	0,349684	0,633121	0,141571	0,422297	NaN	0,651674	0,347887	1,0568	0,269454
-0,929096	-0,13855	-1,55076	0,0710869	-0,998381	0,130089	0,441933	-1,41333	-0,416316	-1,20946
-1,54673	-0,661285	-1,45374	-1,33046	-1,41814	-0,221014	-0,438089	NaN	-1,63366	-0,358779
NaN	NaN	NaN	NaN	NaN	NaN	NaN	NaN	NaN	NaN
0,259026	NaN	-0,220186	-0,980663	-0,0355206	NaN	-0,52388	NaN	NaN	NaN
1,96747	2,27521	1,24436	2,02758	0,999687	0,869986	2,17542	2,1143	1,76907	1,79703
-2,88967	-1,34832	-1,9023	-1,22036	-0,891928	NaN	-0,754381	-1,80666	NaN	-1,59492
NaN	NaN	-1,73223	NaN	NaN	NaN	NaN	NaN	NaN	NaN
0,010252	0,782068	0,483713	0,304787	0,721212	-1,08568	1,5609	0,212004	-0,0352926	0,513259
0,771023	1,72911	1,52878	1,82048	1,88497	1,43565	1,65438	2,01486	1,30541	1,52342
1,12252	2,15929	1,25301	1,89146	2,1424	1,45099	1,88756	1,78065	1,47878	2,02293
NaN	NaN	NaN	NaN	NaN	NaN	NaN	-2,10106	-1,98217	-1,9376
0,29092	-1,58485	-0,85445	1,79628	2,54715	1,6444	1,87342	0,969601	0,913363	0,936656
0,930475	0,343313	3,70163	1,74839	1,58881	1,83097	2,91984	NaN	NaN	NaN
-1,20774	-1,18057	-2,97087	NaN	-1,78136	NaN	NaN	NaN	NaN	NaN
0,286663	NaN	-0,249115	-0,725363	NaN	1,68574	0,16663	NaN	NaN	NaN
NaN	NaN	NaN	-1,01919	-4,72473	-0,557913	-2,01478	NaN	NaN	NaN
NaN	NaN	NaN	-3,12401	-0,315786	NaN	-0,510511	NaN	NaN	-1,58745
NaN	NaN	-0,814201	-0,544613	NaN	-0,886808	-1,00009	NaN	NaN	NaN
NaN	-2,86032	NaN	NaN	-1,88246	-1,67764	-1,45285	NaN	NaN	NaN
-1,31371	NaN	1,51853	1,62241	-0,104733	1,62887	1,67426	NaN	NaN	NaN
1,17516	1,40714	1,13983	1,36633	0,651192	NaN	1,23891	0,141735	0,584996	1,33878
-0,203053	0,54619	0,543194	-0,137587	-0,142553	0,110685	0,308809	-0,77836	-0,251803	0,413684
NaN	-1,95113	-2,05935	NaN	NaN	NaN	NaN	-1,3104	-0,485497	-1,59222
-3,88941	NaN	-0,391775	0,803724	-2,87339	NaN	0,731907	-1,34401	-1,91388	-2,46361
NaN	NaN	NaN	NaN	NaN	NaN	NaN	NaN	NaN	NaN

Polito et al. Universal ready-to-use immunotherapeutic approach for the treatment of cancer: expanded and activated polyclonal γδ memory T cells.
Phosphorylation sites

NaN	NaN	NaN	-0,887669	NaN	-0,770834	-1,38702	NaN	NaN	NaN
-0,219305	1,87397	0,492945	0,851778	1,089	2,0268	1,21447	-1,21115	-1,18283	1,3207
1,9369	1,0329	1,95936	0,9396	1,16604	1,42136	2,11712	0,978363	0,882436	0,845344
3,90054	3,16526	3,79636	1,52011	3,26687	1,62848	2,82605	2,52163	2,17396	2,61647
3,82393	-0,974661	3,22318	1,89922	3,64783	2,4567	3,28102	1,65528	0,91554	1,24691
1,77976	1,5372	1,95494	0,459644	2,31237	0,832388	2,21177	1,3245	0,985469	1,54119
4,6918	3,53503	3,86801	2,77337	4,04695	2,46229	4,02283	3,63211	3,32921	3,07317
NaN	NaN	NaN	-2,04744	NaN	-1,36987	-2,78472	NaN	NaN	NaN
NaN	-3,32157	-0,891876	-1,3344	NaN	-2,27211	-1,76665	-2,54496	-2,48125	-2,68287
NaN	NaN	2,09727	1,31184	0,595434	1,66389	1,96956	-0,0360146	-0,499433	-0,645862
0,306417	-0,15905	-0,367727	1,02726	1,02589	0,470491	0,985334	0,587811	0,18344	-0,303355
-0,0396004	0,228603	-1,68478	1,68863	1,76286	1,94404	2,54099	1,40723	NaN	1,18598
-0,725466	0,33531	0,407728	0,0409756	-0,265636	0,300901	1,27565	0,248301	0,718118	0,243539
-0,540609	-0,473661	0,329712	0,348928	1,05834	NaN	0,622404	0,481585	-0,0878572	0,053998
1,16206	0,959013	1,48386	1,92239	NaN	2,73044	1,25511	0,983923	0,664653	NaN
NaN	NaN	NaN	-0,0460949	0,128399	0,102673	-1,20878	-0,684496	-0,774398	-1,02733
NaN	NaN	1,29318	2,34749	0,781059	-1,61456	2,61978	NaN	0,815259	NaN
NaN	NaN	-1,38083	-1,60652	NaN	-0,526985	-0,972025	NaN	NaN	NaN
NaN	NaN	NaN	-0,90667	NaN	-1,89134	-1,09916	NaN	NaN	NaN
-0,600401	-0,18034	-0,198303	-0,771278	0,0294933	NaN	-0,692616	NaN	-0,803096	-1,08249
-1,92324	NaN	NaN	-0,611959	NaN	NaN	NaN	-1,10894	NaN	NaN
-2,21829	0,0721626	-1,94231	NaN	-0,957888	NaN	NaN	-2,26222	-1,55587	-0,162288
NaN	NaN	NaN	NaN	-1,84807	NaN	NaN	NaN	NaN	NaN
NaN	-1,3739	-2,90846	-0,958416	NaN	-1,66057	-1,20393	-2,68856	-2,1903	-2,41031
NaN	-1,50257	-2,16669	-0,588551	-1,27221	-1,4074	-0,748175	-1,20268	-1,17619	-0,719678
-1,26088	-3,21	NaN	-1,21865	-1,17445	-2,78526	NaN	-1,60549	-0,979737	-2,45377
NaN	NaN	NaN	NaN	NaN	NaN	NaN	NaN	NaN	NaN
-1,47474	NaN	0,664219	1,17574	-0,513912	NaN	0,850548	NaN	NaN	NaN
-1,07871	-2,3068	-2,00558	NaN	NaN	-1,24433	-2,39923	-1,69109	NaN	NaN
1,57797	2,10064	1,30034	2,5692	NaN	3,02755	2,83935	1,05034	0,737929	0,774533
NaN	NaN	-1,06514	-0,847059	-2,78738	-1,56075	-1,10712	-2,12469	NaN	-2,94405
NaN	NaN	-0,864681	-1,41406	NaN	-2,03599	NaN	NaN	NaN	NaN
NaN	NaN	-0,095396	NaN	NaN	1,74821	NaN	NaN	NaN	NaN
NaN	NaN	NaN	NaN	NaN	NaN	NaN	NaN	NaN	NaN

Polito et al. Universal ready-to-use immunotherapeutic approach for the treatment of cancer: expanded and activated polyclonal γδ memory T cells.
Phosphorylation sites

NaN	NaN	NaN	-1,06073	NaN	0,278847	0,181288	-0,686743	1,42166	0,638967
NaN	-2,034	NaN	NaN	-1,425	NaN	-2,16583	NaN	NaN	NaN
-0,429033	-0,0522499	0,198084	-0,0670853	0,364815	-0,987043	0,428629	0,0447197	-0,664895	-3,19093
NaN	NaN	NaN	-1,94247	NaN	-1,39052	-3,4005	NaN	NaN	NaN
NaN	NaN	NaN	-0,357458	1,50434	2,67395	1,06643	1,62187	NaN	1,90367
-2,0294	NaN	NaN	NaN	0,166695	NaN	NaN	NaN	NaN	NaN
6,27762	7,43063	7,06409	7,02748	6,90192	7,57727	7,11108	6,79321	7,05649	6,98789
-2,84314	-2,31911	-2,27589	-1,149	NaN	-0,8342	-2,12072	NaN	-2,86429	NaN
-0,661472	NaN	-0,615953	0,439589	-0,955183	-1,9499	0,640394	-0,495167	-0,448783	-0,663457
-0,353714	-0,441191	-1,0719	0,369179	-1,06303	0,927317	-0,104792	NaN	NaN	NaN
NaN	NaN	NaN	NaN	-2,70802	-1,38403	-2,8305	NaN	NaN	NaN
1,87612	2,85919	2,32191	2,25382	1,93995	1,80391	2,1773	NaN	2,0194	2,03821
-0,224173	-0,930731	0,727674	0,499718	0,909111	0,693834	0,571928	0,378237	-7,14201	NaN
0,919069	0,661184	1,90595	1,91339	1,34648	2,24185	1,98499	1,11003	0,820842	0,91479
0,104301	-0,628645	-0,522165	-0,23959	-0,402271	-0,189789	-0,611097	NaN	NaN	NaN
NaN	NaN	NaN	NaN	NaN	NaN	NaN	NaN	NaN	NaN
NaN	NaN	-1,1484	-0,557459	-2,77835	-0,99976	-1,67061	-1,71529	-3,45846	NaN
NaN	NaN	NaN	-1,03349	NaN	-1,20096	-1,1076	NaN	NaN	NaN
NaN	NaN	NaN	NaN	NaN	NaN	NaN	NaN	NaN	NaN
NaN	NaN	NaN	NaN	NaN	NaN	NaN	-2,36435	-2,40443	-2,97032
NaN	NaN	NaN	NaN	NaN	NaN	NaN	NaN	NaN	NaN
NaN	NaN	NaN	NaN	NaN	NaN	NaN	NaN	NaN	NaN
NaN	NaN	NaN	-1,47991	-1,36884	-0,3134	-1,01817	NaN	NaN	NaN
NaN	-2,59555	-1,23022	-1,43643	NaN	-0,527901	-0,879988	NaN	NaN	NaN
NaN	-1,92996	-1,48859	0,324181	-1,10308	-1,54493	0,0743885	-1,30527	-1,3961	-1,70389
-2,01219	NaN	0,0167961	0,990725	NaN	1,66052	2,10751	NaN	NaN	NaN
NaN	NaN	NaN	0,212996	NaN	NaN	0,486174	NaN	NaN	NaN
1,01165	2,30672	1,43938	2,80069	1,47866	3,0581	2,38754	1,35991	0,983085	1,36325
-1,39397	NaN	0,00131416	-0,970793	-1,42332	-0,541267	NaN	-0,686943	-0,685527	-0,570792
1,00488	0,825174	-0,146917	-2,11233	3,07152	NaN	-0,536757	0,16477	-0,19628	0,467902
2,80706	2,2999	1,9055	0,386158	2,58498	0,555464	1,04763	1,55724	1,00798	1,25424
-1,15927	-1,46462	NaN	NaN	-0,555428	-1,93897	NaN	-1,34224	-1,11903	-1,39038
0,0687122	-0,0398026	-0,0480804	-1,99374	0,934608	-1,38325	-0,307678	-1,27786	-1,09277	-0,587523
NaN	NaN	NaN	NaN	NaN	NaN	NaN	NaN	NaN	NaN

Polito et al. Universal ready-to-use immunotherapeutic approach for the treatment of cancer: expanded and activated polyclonal γδ memory T cells.
Phosphorylation sites

1,80438	1,33833	NaN	2,03751	1,5538	2,55799	2,18523	0,980261	0,752124	NaN
NaN	NaN	NaN	NaN	NaN	NaN	NaN	NaN	NaN	NaN
2,35498	2,35746	2,26985	2,65353	3,36081	3,09742	3,20669	2,4585	2,24421	2,12141
1,51751	0,978119	1,12134	1,66152	1,32451	1,39189	1,9585	1,96699	1,91918	2,06566
-2,06726	-3,37466	-0,270433	NaN	-1,15355	-0,705046	-0,719742	-1,44232	-1,51679	-1,11125
2,61206	3,71627	3,12654	1,08498	1,92393	0,974186	1,96074	1,83507	3,68873	1,91248
NaN	NaN	NaN	NaN	-0,574524	NaN	-1,89093	NaN	NaN	NaN
NaN	-3,02199	0,265213	-0,588385	-3,04417	0,97327	0,3076	NaN	-1,51368	-1,64493
NaN	NaN	NaN	-3,0658	-2,64718	-1,63177	NaN	NaN	NaN	NaN
-0,201794	-0,0583534	0,593916	0,729612	0,442692	1,29344	0,417896	0,307606	0,425653	0,681441
-0,151766	-0,440577	-0,742409	-0,00112152	0,214355	0,536173	NaN	NaN	-1,75537	-1,24851
-1,4018	-1,73939	-0,950476	-0,794239	-0,409807	-0,0575752	-0,179886	-0,680374	-1,30167	-0,724181
-0,935827	NaN	-0,318842	-0,826931	-0,36046	-0,254503	-0,210278	-1,21832	-1,10639	-0,828723
NaN	NaN	NaN	NaN	NaN	NaN	NaN	NaN	NaN	NaN
NaN	NaN	NaN	-0,851265	NaN	-0,354376	-1,68915	NaN	NaN	NaN
NaN	NaN	NaN	NaN	NaN	NaN	NaN	NaN	NaN	NaN
NaN	NaN	NaN	-4,24586	-1,97433	NaN	-2,58572	NaN	NaN	NaN
2,00727	1,3316	1,74996	1,74307	1,0776	2,60767	2,12488	1,68024	1,45907	2,00851
NaN	NaN	NaN	NaN	NaN	NaN	NaN	NaN	NaN	NaN
NaN	NaN	NaN	1,67862	0,838474	1,51091	1,26174	1,2083	1,1335	0,752227
0,437918	0,3249	NaN	-0,0692253	0,448183	0,350147	0,40489	0,492741	0,129773	0,86589
NaN	-1,98076	NaN	NaN	-1,00864	NaN	-2,1064	NaN	NaN	NaN
NaN	NaN	NaN	-1,00256	-0,969643	-1,4168	-1,10184	NaN	NaN	NaN
-2,2129	-2,10742	-1,40129	-1,23016	-1,04192	-1,63188	-1,82491	-1,83094	-1,64459	-1,98143
2,1801	1,28044	1,94281	2,44581	1,89922	2,79531	1,68882	2,31816	2,58926	2,23949
NaN	NaN	NaN	-1,33356	-0,349043	-2,29686	-1,78055	NaN	NaN	NaN
0,350201	-1,03397	-0,289995	0,742195	1,02422	-0,200352	0,565857	0,0517883	-0,421224	-0,0845766
-1,58679	-2,42247	-2,64095	NaN	NaN	NaN	NaN	NaN	-2,07988	NaN
1,40637	0,0458279	0,208521	1,23184	1,02673	1,45903	0,881445	0,809608	0,667239	1,54363
-1,02372	-2,62513	0,119114	-0,419706	-0,72374	-1,66817	-0,0479393	-0,297499	-0,019845	0,787223
1,06137	0,82271	NaN	NaN	1,1344	0,0766582	NaN	1,0011	0,746757	0,674989
NaN	NaN	NaN	NaN	NaN	NaN	NaN	NaN	NaN	NaN
2,17393	NaN	0,619942	0,946674	-1,48772	0,606886	-0,974525	1,53167	1,50459	-0,243638
-3,81552	NaN	-0,878527	-0,90481	NaN	-0,547682	-1,21302	-2,59627	-1,94024	-3,17173

-1,86623	-2,13767	-1,26678	-3,04002	-0,753532	-2,7022	-0,502733	NaN	NaN	NaN
0,815329	0,283648	-1,00422	0,0985603	-0,289486	0,310789	1,06899	-1,12072	-0,867791	-0,863124
-1,88095	-2,462	-0,254164	-0,451807	NaN	-1,34023	-1,76232	-1,38186	0,055562	NaN
NaN	NaN	0,415886	1,47599	-1,17772	2,32612	1,36937	NaN	NaN	NaN
NaN	-2,33876	NaN	0,48469	-1,28248	0,167273	0,217237	NaN	NaN	NaN
-1,80411	-2,87167	-1,63147	-1,9077	-1,57949	NaN	-1,20506	-0,909912	-1,46435	-2,04677
NaN	NaN	NaN	NaN	NaN	NaN	NaN	NaN	NaN	NaN
NaN	-0,735991	NaN	-0,898867	-0,822605	-1,3898	1,02024	NaN	NaN	NaN
-2,58921	-2,23054	-2,25773	-1,54773	-2,39885	-1,06573	-1,61966	NaN	NaN	NaN
NaN	NaN	NaN	-1,56835	NaN	-0,656788	-0,790375	NaN	NaN	NaN
-0,462259	1,00484	0,547247	1,12843	NaN	2,03609	0,508087	NaN	1,56184	-0,633777
NaN	NaN	NaN	NaN	NaN	NaN	NaN	-1,83503	-2,05495	-1,83121
NaN	NaN	NaN	-0,652241	-4,2228	-2,02258	-0,874447	NaN	NaN	NaN
NaN	NaN	NaN	-0,297625	NaN	-0,172804	3,53385	-0,567619	-0,424056	-1,49723
NaN	-2,15999	-0,858212	NaN	NaN	NaN	NaN	-1,15181	-1,48883	-1,55062
NaN	NaN	NaN	-0,983095	NaN	-0,358273	NaN	NaN	NaN	NaN
NaN	NaN	NaN	NaN	NaN	NaN	NaN	-2,11677	-2,75426	-2,84739
NaN	NaN	NaN	-2,41588	-3,79387	-0,930153	NaN	NaN	NaN	NaN
NaN	NaN	NaN	3,26237	-0,138731	3,86905	2,79151	NaN	NaN	NaN
NaN	NaN	NaN	NaN	NaN	NaN	NaN	NaN	NaN	NaN
-1,26382	-1,95668	-2,07694	-1,43722	-1,87932	NaN	-1,18166	NaN	NaN	NaN
0,496742	1,87716	NaN	2,00018	NaN	NaN	NaN	0,52939	0,174418	0,97347
0,908764	1,50458	0,00209808	1,48672	0,0347214	2,45311	0,815422	-0,489029	-0,23887	-0,470752
NaN	NaN	NaN	-1,07944	-1,31437	-2,49062	-1,51223	NaN	NaN	NaN
NaN	NaN	NaN	NaN	NaN	NaN	NaN	NaN	NaN	NaN
1,95423	1,79519	0,987055	1,01166	1,92588	-0,0279503	1,76595	2,00917	1,74315	1,31352
NaN	NaN	NaN	-1,522	-2,12803	-1,10522	-0,353712	NaN	NaN	NaN
1,33844	0,09235	1,84294	-0,371658	2,31409	0,735888	0,364983	0,528509	1,42487	0,511779
4,10519	4,52674	3,67423	4,02672	2,50945	4,06821	4,13328	3,39068	3,98671	3,86299
0,901014	-0,918842	2,29093	1,81824	1,86365	3,54714	1,68597	1,54908	1,32919	0,454299
-0,460476	0,525127	-0,308279	-1,13278	-0,0366287	-1,76602	-0,545277	-0,249771	-0,732495	-1,12335
0,695576	0,531069	0,342306	0,329823	0,92893	-0,271688	0,879381	0,562853	0,394116	0,28468
-0,632984	NaN	-2,86331	0,562927	-0,405582	0,55138	-1,91483	NaN	NaN	NaN
NaN	NaN	-2,34571	-0,871302	-3,76962	-1,09607	-1,95438	-3,17936	-3,3923	-3,60831

Polito et al. Universal ready-to-use immunotherapeutic approach for the treatment of cancer: expanded and activated polyclonal γδ memory T cells.
Phosphorylation sites

NaN	NaN	NaN	2,54045	NaN	1,19182	2,51088	NaN	NaN	NaN
0,197174	-0,982456	NaN	-1,13049	0,076252	-1,32604	-0,569513	-1,31909	NaN	-1,3586
0,228603	0,052784	-0,17182	-0,599455	0,472649	NaN	-0,334818	-0,441341	NaN	-0,796796
NaN	NaN	NaN	NaN	NaN	NaN	NaN	NaN	NaN	NaN
NaN	NaN	-1,61534	NaN	NaN	NaN	NaN	-1,23708	-1,45808	-0,953204
3,30308	4,23287	3,77061	4,2124	3,61453	4,55303	4,10434	4,56552	4,3807	4,27705
0,274811	0,595997	-0,667305	0,0934525	NaN	NaN	0,275669	-1,28903	0,14265	-0,0592737
NaN	NaN	NaN	NaN	-0,320713	NaN	NaN	0,723469	0,534209	NaN
0,547747	0,157196	1,47192	1,62032	1,94018	0,86005	1,01571	1,61839	1,45249	1,80697
NaN	NaN	NaN	0,39023	-0,830824	0,825506	-0,304005	NaN	NaN	NaN
-0,349358	0,309898	NaN	-0,268194	-0,986164	1,88232	NaN	-0,998928	-0,22387	-0,95485
2,59266	2,48746	2,536	3,75554	3,00932	3,92574	3,42652	1,85561	1,59481	3,37679
-2,09216	-2,82125	-1,65044	0,581472	NaN	0,582205	-0,4599	-0,945448	-0,588248	-1,24938
-1,88787	-1,87053	3,02609	2,16897	NaN	-1,12226	3,69058	NaN	NaN	NaN
NaN	0,238277	1,17358	0,211632	0,4807	NaN	1,11511	NaN	NaN	NaN
1,5012	0,938595	NaN	1,3162	-2,10156	-1,19439	2,22951	NaN	-0,998176	NaN
NaN	NaN	NaN	NaN	NaN	NaN	NaN	NaN	NaN	NaN
2,01226	0,955322	1,00417	1,37351	1,53832	NaN	1,40963	1,4777	1,16741	1,63992
0,725716	1,14259	0,8536	0,794851	0,967478	NaN	-0,0385456	0,78443	0,453223	0,629563
2,4091	2,31225	2,42145	2,72136	2,15746	1,48939	2,65256	2,18012	2,10103	1,89296
0,448408	0,392681	0,590542	NaN	0,350859	NaN	NaN	0,562801	0,430865	0,084857
-0,99893	-1,664	NaN	1,35207	-0,839855	1,98745	NaN	-1,19684	NaN	NaN
NaN	NaN	NaN	NaN	-0,556179	-1,33723	-1,57776	-2,53374	NaN	NaN
NaN	NaN	NaN	NaN	NaN	NaN	NaN	-1,34135	-1,54344	-1,3566
NaN	NaN	NaN	0,379532	0,283707	-1,21833	-0,139055	NaN	NaN	NaN
-0,252794	-0,418215	-0,136116	0,819851	0,642567	0,116003	1,06451	1,03338	0,269616	0,169524
NaN	-0,933674	NaN	0,69676	NaN	1,60022	-0,0353584	-0,13876	NaN	NaN
NaN	NaN	NaN	-1,62846	-3,82609	-1,34851	NaN	NaN	NaN	NaN
NaN	NaN	NaN	-0,498177	-2,84623	0,148151	-1,04794	-2,37544	-2,15197	-2,91313
3,43497	3,32123	3,63285	2,79228	3,50382	3,21229	2,9879	4,01671	3,72178	3,30007
NaN	NaN	NaN	-0,474485	-1,50755	-0,925888	-0,210924	-1,06278	-1,36729	-0,766068
NaN	NaN	NaN	-0,905119	NaN	-0,612986	-1,07367	NaN	NaN	-2,69727
-1,56174	-0,789215	NaN	NaN	NaN	0,48871	NaN	-0,650623	-0,732903	NaN
-1,42961	-2,79734	-1,32215	0,38179	-0,880995	1,29354	0,615011	-1,04986	-1,8969	0,0152617

Polito et al. Universal ready-to-use immunotherapeutic approach for the treatment of cancer: expanded and activated polyclonal γδ memory T cells.
Phosphorylation sites

NaN	NaN	NaN	0,101612	NaN	NaN	0,0872765	NaN	NaN	NaN
-1,09388	-1,77243	-0,616278	-1,16556	NaN	-2,12513	-0,219017	-1,78819	-0,743234	-1,07232
NaN	NaN	NaN	-2,37059	NaN	-1,21391	-2,63152	NaN	NaN	NaN
2,34755	3,054	2,53559	2,81267	2,89227	3,98816	-2,90191	1,63052	1,06359	2,18603
2,34755	3,054	-2,27445	-1,61483	2,89227	-1,09513	3,12237	1,63052	1,06359	2,18603
NaN	NaN	NaN	-1,52168	-2,21367	-2,88283	NaN	NaN	NaN	NaN
1,0421	1,51862	1,6396	0,418427	1,85385	-0,232935	0,818974	0,687355	0,708989	0,987889
-0,0477676	NaN	-0,189501	-0,0879383	-0,321922	NaN	-0,230114	0,108343	0,0840311	-0,210307
NaN	NaN	0,373859	-0,701252	1,21622	NaN	0,809824	1,03898	NaN	1,41907
1,38311	2,99026	1,90516	2,08849	1,91348	1,49935	2,70361	2,06407	2,12694	2,89481
NaN	-1,55617	-2,08313	NaN	-2,63706	NaN	NaN	NaN	NaN	-2,76063
-1,60834	-2,09879	-2,02616	-1,44506	-1,17419	-0,67099	-1,24339	-1,12115	-1,35738	-1,13721
NaN	-0,453489	NaN	NaN	NaN	NaN	NaN	NaN	NaN	NaN
1,33862	3,19304	2,36661	2,3169	2,19228	2,87658	3,2254	1,83695	1,83896	2,59266
-0,111567	0,272341	0,222397	0,358753	NaN	-0,718451	0,931749	0,875803	NaN	0,537099
-2,00402	NaN	-0,395729	NaN	NaN	NaN	NaN	-0,0462284	-0,531474	-0,481927
NaN	NaN	NaN	NaN	NaN	NaN	NaN	-1,96962	-2,23016	-1,88775
NaN	-0,547535	-0,736216	-0,355576	-1,15197	-0,902605	-0,541206	-0,0779953	-0,649533	-1,01123
2,33415	1,88574	0,933237	1,56093	1,12073	2,46748	1,30895	1,61049	1,32756	1,15756
0,485579	0,484396	-0,11397	0,523188	0,466736	0,410357	0,274986	0,996479	0,966283	0,368608
NaN	NaN	NaN	-2,23391	-2,82839	NaN	-3,15675	NaN	NaN	NaN
NaN	NaN	NaN	-1,75769	-2,26673	NaN	-0,476875	NaN	NaN	NaN
NaN	NaN	-1,70095	NaN	NaN	NaN	NaN	-1,78123	-1,95583	-2,03074
-0,41156	NaN	2,0463	NaN	-0,536829	NaN	NaN	0,0310898	-0,155095	-0,701575
-0,768732	-1,95632	0,531349	NaN	-1,57389	NaN	-2,29751	0,38982	-0,182296	1,20063
NaN	NaN	-2,16822	NaN	NaN	NaN	NaN	-1,58978	-1,83486	-1,51378
2,55065	1,01134	2,88433	-2,07266	2,19122	NaN	-0,131079	2,81032	2,5179	2,14189
NaN	NaN	NaN	NaN	-2,69839	NaN	NaN	NaN	NaN	NaN
-2,95631	NaN	-1,81633	-1,32511	-0,481594	-1,90554	-1,373	-1,73027	-1,89255	-1,96082
NaN	NaN	NaN	NaN	NaN	NaN	NaN	NaN	NaN	NaN
3,88079	4,10001	3,89405	3,02751	4,96855	1,13675	3,31976	4,19909	3,77526	4,03591
NaN	NaN	NaN	NaN	-1,32858	NaN	NaN	-1,67316	-1,78425	-2,41104
NaN	NaN	NaN	NaN	NaN	NaN	NaN	NaN	NaN	NaN
NaN	NaN	NaN	NaN	NaN	NaN	NaN	NaN	NaN	NaN

Polito et al. Universal ready-to-use immunotherapeutic approach for the treatment of cancer: expanded and activated polyclonal γδ memory T cells.
Phosphorylation sites

NaN	NaN	NaN	NaN	NaN	NaN	NaN	NaN	NaN	NaN
NaN	NaN	NaN	-2,52021	-2,25393	NaN	NaN	NaN	NaN	NaN
0,284189	-0,273394	0,451136	-2,31749	1,15113	NaN	-1,17569	0,554029	-1,39367	-0,522292
1,71997	3,39916	2,67222	3,36909	3,28737	1,18414	3,54074	2,32971	1,37395	2,03081
NaN	-1,47425	NaN	-1,90171	-1,65878	NaN	-0,538185	NaN	NaN	-0,247174
NaN	NaN	NaN	NaN	-0,030138	-3,59544	NaN	NaN	NaN	NaN
-0,812202	-2,37369	2,17372	1,15005	0,0747948	2,2338	2,23631	NaN	0,278436	NaN
NaN	NaN	NaN	NaN	-1,9922	NaN	-1,84951	-3,38573	NaN	NaN
NaN	NaN	NaN	NaN	NaN	NaN	NaN	NaN	NaN	NaN
0,647797	0,0258598	1,61628	1,66517	2,36801	0,983246	1,99854	0,184504	-0,0256987	0,364211
NaN	NaN	NaN	-2,97375	-2,63328	-2,17162	NaN	NaN	NaN	NaN
1,48007	0,0623341	1,75481	2,02785	2,31734	2,69925	1,51237	1,52544	1,11657	1,0948
-1,46263	-1,11361	NaN	-0,0488682	-0,378792	0,284859	-0,764938	NaN	-0,421664	-1,13908
NaN	NaN	NaN	-1,85077	NaN	-1,65347	-2,61349	NaN	NaN	NaN
NaN	NaN	NaN	NaN	NaN	NaN	NaN	NaN	NaN	NaN
NaN	NaN	NaN	NaN	NaN	NaN	NaN	NaN	NaN	NaN
NaN	NaN	NaN	NaN	NaN	NaN	NaN	-2,64309	-2,99609	-2,14607
NaN	NaN	NaN	NaN	NaN	-3,05827	-1,17574	-0,177387	-0,0804853	0,490798
-0,272692	0,114666	NaN	-1,56802	NaN	-2,40784	-0,938831	0,450583	0,309665	0,485858
NaN	NaN	NaN	NaN	NaN	NaN	NaN	NaN	NaN	NaN
2,40713	1,13098	2,36715	NaN	2,34905	NaN	1,62504	2,23312	1,99456	1,68587
-2,0315	-1,93293	-1,86391	-0,405905	-1,66643	NaN	-1,63846	-2,57526	-2,81286	-1,66052
NaN	NaN	NaN	-1,19699	-1,76745	-0,53483	-1,75876	NaN	-2,04951	-1,59395
0,405811	1,46393	0,558228	0,921371	NaN	-0,33218	1,21215	0,123281	1,09753	0,926066
-0,709135	-0,573334	-1,21321	-0,342649	-0,591732	-0,969948	0,111101	0,248007	-0,548141	-0,902947
1,39266	0,931995	1,07037	1,63726	1,9566	1,12345	2,00167	1,50599	1,32018	NaN
NaN	NaN	NaN	NaN	NaN	NaN	-0,612907	NaN	NaN	NaN
NaN	NaN	NaN	-0,51119	NaN	-0,637365	NaN	NaN	NaN	NaN
0,812664	-0,39353	NaN	-0,549204	0,361364	-0,835846	-1,04725	-0,321579	-0,790128	-0,221448
5,812	5,33373	5,78211	5,74132	6,39906	5,31962	6,09253	5,56478	5,79274	6,08971
-1,03845	NaN	NaN	1,0005	NaN	NaN	NaN	NaN	NaN	NaN
NaN	NaN	-0,538237	NaN	NaN	NaN	NaN	NaN	NaN	NaN
-0,85957	-1,5835	0,66609	-0,0520859	-0,782047	0,550972	0,990292	-0,49844	-0,652753	-1,14846
-1,44156	-0,0333939	0,00347328	0,891758	1,04311	-0,904129	1,08414	-0,253721	-0,428603	-0,204795

-0,799404	-1,84076	-0,62015	NaN	NaN	NaN	NaN	-1,11385	-1,43422	-1,97158
0,369921	0,236294	0,728569	0,792095	0,28027	1,63343	0,371387	1,57333	1,28594	1,32088
NaN	-2,42523	NaN	-1,63678	-1,81158	-2,42576	NaN	-1,85105	-2,1325	-0,922954
1,50419	1,25626	1,49052	3,55973	1,59146	3,96548	3,68623	2,76785	2,81631	2,82761
NaN	NaN	NaN	1,47758	NaN	2,61526	-0,609938	NaN	NaN	NaN
0,15642	NaN	1,34307	1,03005	0,490223	1,15441	0,109011	1,22674	0,639596	0,972301
4,46035	3,84976	0,348978	4,95676	5,10641	0,366507	4,05478	4,94096	5,71319	4,83854
NaN	NaN	NaN	0,16069	-2,20617	0,918468	0,0323524	-1,24302	-1,19231	-0,89009
NaN	0,151737	0,224157	-0,184111	0,404385	NaN	0,427448	0,551035	0,665559	0,543252
NaN	-2,15505	-1,69531	NaN	-1,43066	-2,54763	NaN	-3,1513	-2,62947	-1,92544
1,28357	1,87317	1,35166	0,685375	1,62411	NaN	0,966587	1,30494	1,03124	1,5034
-3,64617	-4,48565	-2,80078	-2,62222	NaN	-1,61798	NaN	NaN	NaN	NaN
NaN	NaN	NaN	-1,216	-3,24043	-1,10827	-1,88985	NaN	NaN	NaN
NaN	-0,920816	NaN	NaN	NaN	NaN	NaN	NaN	0,0332479	-0,992755
NaN	NaN	NaN	-1,69922	NaN	-1,29595	-0,95174	NaN	NaN	NaN
0,186386	0,485064	0,584333	0,645859	0,756109	-1,13839	1,26667	0,460186	0,804965	0,445376
2,48462	2,56706	1,54347	2,4815	2,58654	3,09574	2,16669	2,06353	1,63161	1,77722
NaN	NaN	NaN	NaN	NaN	NaN	NaN	NaN	NaN	NaN
1,09896	0,801683	0,0244656	1,30278	0,51198	2,44188	0,896891	NaN	0,651788	0,801908
NaN	NaN	NaN	NaN	NaN	NaN	NaN	-1,2167	-2,7167	-1,36102
NaN	NaN	NaN	NaN	NaN	NaN	NaN	NaN	NaN	NaN
-1,63499	-1,73042	-1,53439	NaN	NaN	NaN	NaN	NaN	NaN	NaN
-2,52511	-2,09865	-2,13042	-1,25574	-2,17143	-0,638914	-1,70247	-1,51817	-1,45302	-1,93225
-0,916319	-1,13282	-0,47854	0,496075	-0,0201283	1,05859	0,978317	0,405272	0,832751	1,19973
NaN	NaN	NaN	0,10988	NaN	0,673841	-0,482889	NaN	NaN	NaN
NaN	NaN	NaN	0,742525	-1,33151	1,90276	0,743166	NaN	NaN	NaN
-2,24619	NaN	-2,03048	-2,86456	NaN	NaN	-2,00344	-1,85501	-1,94948	-1,92832
1,23433	0,713898	-0,682941	1,19136	0,931498	1,40244	0,632486	0,24662	NaN	0,117885
NaN	NaN	NaN	-0,743099	-2,88072	-0,616877	-0,94665	NaN	NaN	NaN
-0,873816	NaN	-0,842884	0,0132122	NaN	-0,0348091	-1,5366	-0,337091	-0,105819	NaN
1,01861	1,31873	2,89588	1,82417	1,31277	2,89726	2,96264	0,446835	0,400765	0,970897
NaN	NaN	NaN	-1,04965	-0,722519	-1,77002	NaN	NaN	NaN	NaN
0,373371	0,817593	2,73412	2,54748	-0,0935555	2,61952	2,99232	0,53558	1,44324	0,397712
NaN	NaN	NaN	-1,93936	-0,564144	-0,145639	-1,37416	-2,35577	NaN	-2,39056

0,739017	0,0375195	1,45586	1,66396	-0,452122	2,49525	1,50735	-0,3232	0,790454	-0,55203
-0,761526	-0,864565	-0,698095	-0,580227	0,69837	-2,385	-0,970798	-1,33693	-0,935998	-0,124322
1,89124	2,30852	2,50381	2,65201	2,21824	3,46379	2,46786	1,55749	1,08289	1,57555
NaN	-2,10393	NaN	-1,6628	NaN	NaN	-2,12441	NaN	NaN	NaN
NaN	NaN	NaN	1,61337	-2,45603	2,55073	-0,735357	NaN	NaN	NaN
-0,310032	NaN	2,1822	3,40787	NaN	2,9265	0,90204	NaN	NaN	0,130204
NaN	NaN	1,38734	NaN	NaN	NaN	0,723406	NaN	-0,360061	-0,726344
NaN	NaN	NaN	-2,58105	NaN	-1,01	-2,57038	NaN	NaN	NaN
NaN	NaN	NaN	NaN	-2,28949	-2,51103	-0,690992	NaN	NaN	NaN
4,6065	4,91328	4,56136	4,68075	4,59572	-0,316246	4,96046	4,72887	4,6746	4,55201
NaN	NaN	NaN	NaN	-1,56562	NaN	NaN	NaN	NaN	NaN
NaN	NaN	NaN	-2,38089	-2,50684	-2,07509	-2,2399	NaN	NaN	NaN
3,47478	2,72461	3,79336	2,85803	5,34524	1,94083	3,94381	3,50373	3,58525	3,82997
NaN	NaN	-1,20479	-1,52559	-3,24886	-1,15987	-0,498465	NaN	NaN	NaN
0,151806	-0,0507317	0,938223	1,26908	0,190161	0,747641	1,72645	0,281925	0,875634	0,44955
NaN	-2,00027	NaN	-2,4599	-1,03561	NaN	NaN	-1,21253	-1,29229	-0,594056
-0,483322	1,00321	0,337769	0,650669	2,28317	-1,45398	0,888706	-0,926266	-0,946038	0,461183
NaN	-1,02316	-2,30043	0,402939	-0,458984	-1,26552	NaN	-0,622204	-1,45448	-1,39128
0,0343628	0,562	-0,441916	0,983213	-0,0150909	1,09517	-0,288582	0,422401	-0,513915	-0,876628
-1,19128	-1,1811	NaN	-0,640244	NaN	-0,0272179	-1,61144	-2,50154	-1,985	NaN
-0,51458	-0,673037	NaN	0,621811	0,415663	0,791155	0,466999	0,124746	0,0805445	-0,0110216
-1,41103	-0,0163727	-2,55461	-0,594395	-0,521606	-1,65921	-1,20429	-1,71931	-1,89164	-1,88283
-0,00828362	0,839174	0,2031	1,24345	0,777134	2,25145	1,55893	NaN	NaN	-0,546691
NaN	NaN	NaN	NaN	NaN	NaN	NaN	NaN	-1,15288	NaN
-1,16928	-1,31624	-1,60511	NaN	0,0820122	NaN	-1,01645	-1,51531	-1,56306	0,0292482
NaN	NaN	NaN	NaN	NaN	NaN	NaN	NaN	NaN	NaN
NaN	NaN	NaN	-1,52395	-1,07412	-2,21669	NaN	NaN	NaN	NaN
NaN	NaN	NaN	-1,72999	NaN	-1,41581	NaN	NaN	NaN	NaN
NaN	NaN	NaN	NaN	NaN	NaN	NaN	NaN	NaN	NaN
-4,33588	-3,67687	-3,58469	-6,39543	0,266153	NaN	NaN	-3,01191	-2,72957	-0,0901117
-0,852943	NaN	1,26099	1,03356	0,593212	1,49429	0,274893	-0,756411	-0,792399	-0,0209532
NaN	NaN	NaN	NaN	NaN	NaN	NaN	NaN	NaN	NaN
-1,11606	-0,993704	-1,93864	NaN	-2,16696	NaN	NaN	NaN	NaN	NaN
NaN	NaN	NaN	-1,30313	-1,6111	-0,287413	-0,962587	-1,86415	-1,8921	-1,44045

NaN	NaN	NaN	-2,55943	NaN	-1,26901	-2,92987	NaN	NaN	NaN
-1,27539	NaN	-1,6769	-0,994513	-0,928963	0,0475025	-1,85201	-1,02625	-1,38154	-1,25392
NaN	-3,71804	-2,47421	-2,83882	NaN	-0,900925	-1,61089	NaN	NaN	NaN
-0,548716	NaN	NaN	-1,83928	NaN	NaN	-0,913691	NaN	NaN	NaN
-0,0562363	0,781979	0,00343132	-0,376732	-0,77104	-1,15255	0,44471	-0,427958	-0,439019	-0,731326
NaN	NaN	NaN	-1,47403	-2,84564	NaN	-1,06168	NaN	NaN	NaN
0,98167	-1,15641	0,700279	-0,262779	2,22591	-1,3048	0,274746	NaN	NaN	NaN
-1,6112	-2,57025	-0,38282	0,217302	-0,540257	-0,344681	0,559214	-0,552982	-0,550452	-0,29511
0,0971413	-0,528849	0,0680027	NaN	0,475023	NaN	NaN	-0,756746	-1,50434	-0,175393
-1,38514	-1,07519	-0,763195	-0,8508	-0,366678	-1,08986	-0,0317669	-0,638536	-1,17911	-0,64045
NaN	NaN	-1,10221	-1,71591	NaN	-0,627205	0,730696	NaN	-2,81929	NaN
NaN	NaN	NaN	NaN	NaN	NaN	NaN	NaN	NaN	NaN
0,214495	1,88355	0,85219	NaN	2,74766	-1,60633	-1,28826	0,120831	-0,368323	0,74348
NaN	NaN	0,596161	-0,265362	NaN	-1,80891	-0,197102	-1,60526	-1,56137	-1,64651
-1,53142	-1,87853	-1,53243	-0,731089	0,658325	-1,49517	-1,8891	-2,08689	-2,07633	-1,98733
NaN	NaN	NaN	1,81275	NaN	-0,307129	NaN	NaN	NaN	NaN
-2,22809	-2,409	-2,17644	-0,746641	-0,827541	-2,50749	-2,30301	-2,33448	-2,50644	-2,02093
-0,809734	-2,11915	NaN	1,89171	NaN	2,13413	0,558077	0,354067	0,303746	NaN
-0,612637	-1,07841	-0,220482	-0,54097	-1,26441	-0,680023	-0,532259	-0,73172	-0,229365	-0,857083
2,71062	1,8397	2,99218	3,55233	3,01577	3,55654	2,46248	3,1405	2,80998	3,02921
-0,698627	-2,48572	-0,117668	-0,278568	-0,15659	1,50535	-0,824636	-0,229218	-0,340323	0,27521
3,68747	3,47577	NaN	2,535	3,74372	2,90091	2,43468	3,71238	3,41614	3,4758
NaN	NaN	NaN	0,0258522	-0,811134	-0,0317421	NaN	NaN	NaN	NaN
NaN	NaN	NaN	-1,26733	NaN	-0,586832	-2,70987	NaN	NaN	NaN
-0,789532	NaN	0,292484	NaN	-0,613274	0,0892181	-0,359463	1,15575	NaN	NaN
NaN	NaN	2,66996	NaN	3,15755	NaN	2,28769	NaN	NaN	2,84218
1,71861	1,27762	1,39244	1,62783	1,72173	1,82382	NaN	2,03387	1,72663	1,31693
0,0514565	1,05466	0,660065	0,498419	1,1939	0,383877	0,684862	0,873219	0,532367	0,601752
NaN	-1,35373	NaN	-2,32483	-0,751808	NaN	-2,68182	-1,38309	NaN	NaN
-0,793434	-1,44214	-1,62315	1,29793	-1,19129	NaN	-0,908228	NaN	NaN	NaN
NaN	NaN	NaN	NaN	NaN	NaN	NaN	NaN	NaN	NaN
NaN	NaN	NaN	-0,164797	-0,37355	0,520803	NaN	NaN	NaN	NaN
NaN	NaN	NaN	-0,865461	NaN	NaN	-0,559137	NaN	NaN	NaN
NaN	NaN	NaN	NaN	NaN	NaN	NaN	NaN	NaN	NaN

-0,938263	-0,251068	-0,485231	-0,189238	0,286694	NaN	0,223185	0,260429	-0,367927	-0,436124
NaN	NaN	NaN	NaN	NaN	NaN	NaN	NaN	NaN	NaN
NaN	NaN	NaN	NaN	NaN	NaN	NaN	NaN	NaN	NaN
NaN	-4,06109	0,776007	-0,420912	NaN	-0,0480042	-0,0462322	NaN	NaN	NaN
NaN	NaN	NaN	0,0488338	NaN	-0,0870571	-0,667978	NaN	NaN	NaN
-0,639053	-1,17184	-0,683657	0,11849	-0,926529	NaN	0,0431938	NaN	NaN	NaN
NaN	NaN	-1,92594	-3,00856	NaN	-2,06453	-2,42001	NaN	NaN	NaN
-1,84803	-1,99535	NaN	-0,840921	-1,97076	-1,96882	-2,82537	NaN	NaN	NaN
NaN	NaN	NaN	NaN	NaN	NaN	NaN	NaN	NaN	NaN
NaN	NaN	NaN	-0,426041	NaN	0,911095	-1,10285	NaN	NaN	NaN
-0,027174	NaN	-0,529949	NaN	-0,0732517	NaN	NaN	0,0689144	-0,446769	-0,0682917
NaN	NaN	0,383738	-0,178608	-1,99912	-2,03285	-0,183004	-1,65796	-1,85016	-0,503171
-0,0569096	0,22501	-0,138563	-0,166304	0,099844	0,106016	0,394829	0,586	0,338693	0,406482
NaN	NaN	NaN	-0,943174	-0,396309	NaN	-1,79219	-1,60936	-2,23209	-2,10413
NaN	NaN	NaN	NaN	NaN	NaN	NaN	-1,14815	-1,30501	-2,16674
NaN	NaN	NaN	NaN	NaN	NaN	NaN	-1,73699	-1,95432	-1,78469
-1,15932	-0,165133	-0,463324	0,650785	-0,134573	2,86018	0,445351	-0,258488	-0,656564	-0,564059
1,65731	1,85545	1,89025	1,59523	2,16692	0,494591	2,06781	1,51116	1,38562	1,25289
2,5948	2,66054	2,38856	2,52447	2,64939	2,13692	2,61945	2,61228	2,32347	2,45157
2,53713	1,50984	1,34433	1,04278	1,49842	0,130306	1,4092	1,35699	0,462094	0,374179
NaN	-0,676867	NaN	NaN	NaN	NaN	NaN	NaN	NaN	NaN
5,21971	5,30565	6,20849	5,68039	4,36089	5,48886	5,4097	5,12883	5,57021	4,97432
-0,656082	0,988913	0,385492	0,536177	0,480883	0,358023	0,907293	0,52825	0,379766	1,25799
3,63415	4,04435	4,5349	3,65269	3,86605	3,86461	3,82937	3,52778	3,44424	3,42536
NaN	NaN	NaN	NaN	NaN	NaN	NaN	0,218493	0,561704	-0,293225
NaN	NaN	NaN	NaN	NaN	NaN	NaN	NaN	NaN	NaN
2,80869	5,53355	4,39763	4,55518	1,96194	2,94419	4,92559	3,38285	3,8479	4,66098
-0,799326	NaN	-0,849285	0,0584869	0,16634	-1,11142	-0,943035	NaN	NaN	NaN
NaN	NaN	NaN	NaN	NaN	NaN	NaN	NaN	NaN	NaN
1,31513	2,07069	1,3409	1,53858	0,04669	0,906307	1,48517	1,02605	1,09456	0,0593596
NaN	NaN	NaN	NaN	NaN	NaN	NaN	NaN	NaN	NaN
NaN	NaN	NaN	NaN	NaN	NaN	NaN	NaN	NaN	NaN
-0,0654564	2,08189	0,668758	1,84765	1,79926	3,44466	2,20262	0,894978	0,992908	1,53487
2,79251	3,5361	3,11577	3,21103	1,80342	3,08772	3,43201	3,02722	3,27255	2,93552

Polito et al. Universal ready-to-use immunotherapeutic approach for the treatment of cancer: expanded and activated polyclonal γδ memory T cells.
Phosphorylation sites

-0,866634	0,283775	0,564978	-1,21694	0,0643082	-1,10218	0,466125	0,871584	0,743207	0,665456
NaN	NaN	NaN	NaN	NaN	NaN	-2,45953	NaN	NaN	NaN
NaN	NaN	NaN	NaN	NaN	NaN	NaN	NaN	NaN	NaN
NaN	0,7323	-0,444595	-0,943705	0,372213	NaN	-0,434278	0,190132	-0,201459	-0,540435
NaN	1,5927	NaN	1,3243	-5,55699	1,15296	1,06461	-2,95726	-1,50842	-0,857333
1,22576	-0,313641	1,15875	0,900351	1,19501	1,63606	0,501789	1,15026	0,701945	0,827352
-1,20774	NaN	NaN	0,282108	-0,739983	2,09313	0,662163	0,920767	0,356631	0,603097
-1,87185	-0,90151	-2,47833	-1,87747	-0,741028	-2,0702	-1,64152	-2,23813	-1,98468	-1,36243
NaN	-0,974537	NaN	NaN	NaN	NaN	NaN	NaN	-0,691047	NaN
NaN	NaN	NaN	NaN	NaN	NaN	NaN	NaN	NaN	-0,752318
NaN	NaN	NaN	NaN	NaN	NaN	NaN	NaN	NaN	NaN
NaN	NaN	NaN	1,05345	NaN	NaN	NaN	NaN	NaN	NaN
-0,648575	-1,35345	-1,13214	-1,45626	-1,32616	-0,348871	-0,920824	-0,765583	-0,737342	NaN
2,44307	2,3813	2,69115	3,20127	3,47411	1,20524	3,58875	2,88177	2,80032	3,23699
-1,15053	-2,69564	-0,131557	-0,474972	-1,7406	-0,667692	-1,02057	-0,56146	-0,344092	-1,07831
1,31486	1,24342	1,16736	2,06676	0,748514	2,48867	1,69773	1,22514	1,62936	1,1493
NaN	NaN	NaN	1,20625	0,188465	NaN	0,39607	NaN	NaN	NaN
NaN	-1,94842	-1,90222	-1,82553	NaN	NaN	-1,93673	-1,91913	-2,43206	-1,75642
-1,12864	-1,01373	-1,06266	-0,220251	-1,08283	NaN	-0,349293	-0,579611	-0,654529	-0,839892
-0,165468	0,470072	-1,30178	0,823908	-0,24662	0,859919	0,216545	-0,259264	-0,379672	-0,79349
-0,683815	-1,0232	-1,31275	-0,287628	-1,04332	-0,445873	0,0252762	-0,924561	-0,958577	-1,04294
-0,926506	NaN	-0,381964	NaN	-0,481674	NaN	NaN	-0,860662	-0,757865	-1,52867
NaN	NaN	NaN	NaN	NaN	NaN	NaN	NaN	NaN	NaN
NaN	NaN	NaN	NaN	-1,90326	-1,67753	NaN	NaN	NaN	NaN
NaN	NaN	NaN	0,316156	0,705416	-2,2148	0,441801	0,0120468	-0,207177	-0,648522
-2,3371	NaN	0,426382	0,0150509	-2,20434	-0,543081	0,271862	-1,46638	-1,13273	-1,40252
-1,4369	NaN	0,368862	-0,666145	-0,730801	-0,160429	-0,796961	-0,545317	-1,09654	-0,803309
1,68194	2,97519	2,59648	2,37323	3,75663	1,27345	3,15948	2,61126	2,59433	3,51022
-1,26163	-0,234478	-0,83428	-1,28963	-0,28598	NaN	-0,7554	-0,986469	-1,29134	-1,02368
NaN	-3,25288	0,280092	-0,401188	-2,54611	NaN	-0,0220222	-1,93021	-1,87334	-1,78113
-1,64941	-2,71638	0,425861	-0,402256	NaN	NaN	0,0319138	NaN	-1,03426	-0,408172
NaN	NaN	NaN	NaN	NaN	NaN	NaN	NaN	NaN	NaN
NaN	NaN	NaN	NaN	NaN	NaN	NaN	NaN	NaN	NaN
0,334337	0,689859	0,164661	0,362331	0,610281	-0,495153	0,284466	0,740826	0,684442	0,418946

Polito et al. Universal ready-to-use immunotherapeutic approach for the treatment of cancer: expanded and activated polyclonal γδ memory T cells.
Phosphorylation sites

-1,20244	-1,18442	-1,90722	NaN	-0,844543	-0,10886	NaN	NaN	NaN	NaN
-1,40422	NaN	NaN	NaN	0,286484	0,378725	NaN	0,914825	-0,138293	0,580346
1,98675	0,917223	1,47885	2,10811	0,364174	2,71193	1,56604	0,434338	1,07532	0,944072
-1,31172	-1,906	0,161823	-1,8029	NaN	-1,08528	NaN	-0,0619202	-0,359483	-0,176923
-1,66151	-2,26024	-0,942713	-0,875072	-1,86185	-1,43892	-0,479364	-2,35692	-2,38202	-2,80716
NaN	-0,973345	-1,36377	NaN	NaN	-0,444242	-2,33705	-2,27774	-1,53831	-2,2478
NaN	-2,72965	-1,2412	-2,14513	NaN	-2,28747	NaN	-1,47343	NaN	-1,55109
NaN	NaN	NaN	NaN	NaN	NaN	NaN	NaN	NaN	NaN
NaN	0,719963	0,782429	1,43749	1,61346	1,14349	1,41089	0,281698	0,0296774	NaN
NaN	NaN	NaN	-2,99719	NaN	-1,81186	-2,84366	NaN	NaN	NaN
NaN	NaN	NaN	NaN	NaN	NaN	NaN	NaN	NaN	NaN
NaN	NaN	-0,30904	NaN	NaN	NaN	NaN	0,460024	0,98215	0,656398
NaN	NaN	NaN	NaN	NaN	NaN	NaN	0,610729	-0,131942	1,74334
0,334337	0,689859	0,164661	0,362331	0,610281	-0,495153	0,284466	0,740826	0,684442	0,418946
NaN	NaN	NaN	NaN	NaN	NaN	NaN	NaN	NaN	NaN
-0,0458069	-0,0926437	0,22374	-0,285049	0,724091	-0,72624	-0,161373	0,528526	0,0856161	-0,296857
-0,630873	-1,09113	NaN	-0,0602665	-0,00347328	1,08267	-0,356771	NaN	NaN	-0,82384
NaN	NaN	NaN	0,671246	-1,9234	0,112682	-1,71313	NaN	NaN	NaN
-1,11572	-0,826523	0,359722	1,86607	0,226242	1,6617	0,251375	-0,419725	-0,496276	0,0417871
0,00883293	-1,61388	0,302197	2,8644	0,303057	3,05174	0,366802	-0,162655	-0,606555	-0,740884
1,4544	2,49764	1,51609	1,62175	2,23192	2,26073	2,2809	0,939095	1,38805	1,21622
NaN	NaN	NaN	-2,59353	-2,82015	-1,47265	NaN	NaN	NaN	NaN
0,629446	2,81872	3,14492	4,09037	2,70674	2,7744	2,70344	2,23312	2,15714	4,03969
-2,16958	-1,23263	0,121668	-1,57162	0,267145	-0,998203	-0,903957	-1,08404	-1,68563	-0,0191393
NaN	NaN	NaN	-2,01803	-2,07006	-1,24294	-1,31075	NaN	NaN	NaN
NaN	NaN	NaN	-0,492994	-0,862629	0,180868	-0,522852	-0,766855	-0,587323	-0,50265
-1,08022	-0,131041	0,427799	-0,0943794	1,25235	-1,25814	0,760946	0,308088	-0,260818	0,269187
1,3318	1,5315	NaN	2,35286	2,93706	2,33379	2,19483	1,61741	1,08008	2,17194
NaN	NaN	1,23583	NaN	-2,94264	-0,368385	-1,52279	NaN	NaN	NaN
0,612289	0,285181	0,380257	0,254915	0,762543	-0,156683	-0,0786209	1,21605	0,592904	0,59274
NaN	-1,37333	NaN	-1,60411	-0,581102	-2,78683	NaN	-2,12904	-2,20082	-1,79923
NaN	NaN	NaN	-0,131977	NaN	NaN	-1,34463	NaN	NaN	NaN
-0,227043	NaN	2,03128	2,26461	-0,0394878	1,68044	2,17418	-0,714264	-0,933331	-0,111943
-1,60013	-2,07312	-2,03369	0,275639	-0,727501	0,928577	-1,07217	-1,29251	-1,40748	-1,19783

Polito et al. Universal ready-to-use immunotherapeutic approach for the treatment of cancer: expanded and activated polyclonal γδ memory T cells.
Phosphorylation sites

NaN	NaN	NaN	NaN	NaN	NaN	NaN	NaN	NaN	NaN
-3,04924	NaN	-0,870365	0,00899887	NaN	0,960766	-0,742668	NaN	NaN	NaN
-2,13417	NaN	-0,113054	1,51697	NaN	2,2097	1,26788	NaN	NaN	NaN
NaN	-2,43497	-1,13516	-0,209839	NaN	-0,119236	-0,537764	NaN	NaN	NaN
-2,83697	NaN	1,28528	1,51389	-1,79748	2,40543	1,66245	NaN	NaN	NaN
NaN	-0,973068	-2,04056	-2,32686	-2,18676	-1,38353	NaN	NaN	NaN	NaN
3,22995	2,40767	4,19023	2,49255	3,50847	2,66213	3,46323	2,69083	2,873	2,96964
NaN	NaN	NaN	-2,24424	-3,10322	-1,38169	-0,772362	NaN	NaN	NaN
-1,20681	NaN	-1,22471	-1,17969	-1,07406	NaN	-1,59555	NaN	NaN	NaN
-1,48651	-0,553604	-1,4527	-1,20995	-0,790972	NaN	NaN	-0,936493	-0,752637	-1,01299
NaN	NaN	NaN	NaN	NaN	NaN	NaN	-0,36618	-0,741347	-0,473649
NaN	NaN	NaN	-2,47658	NaN	-2,10592	-2,07226	NaN	NaN	NaN
NaN	NaN	NaN	NaN	NaN	NaN	NaN	NaN	NaN	NaN
1,17931	0,458101	1,5578	0,28614	2,53341	-3,62042	1,27989	0,76482	0,347922	0,616511
-1,81177	NaN	-1,63298	-1,52475	-1,24137	0,706667	-1,26412	NaN	NaN	NaN
NaN	-0,161312	-0,191364	-1,23339	0,74509	-0,747267	-0,628025	-0,111879	0,140084	-0,338094
-0,3449	-1,09809	0,123558	-0,41806	0,713743	0,477652	NaN	0,0810852	-0,375623	0,276671
1,11913	0,672762	0,40028	-0,0131092	0,603653	0,501719	0,653666	-0,693747	0,352412	-0,740193
5,65758	4,82815	5,34421	4,51128	5,87863	5,22932	5,43021	5,49587	4,73891	4,72572
1,26176	0,523829	0,786489	0,101303	1,01496	0,977112	0,684027	0,961811	0,540427	0,51546
NaN	NaN	NaN	NaN	NaN	NaN	NaN	NaN	NaN	NaN
NaN	NaN	NaN	NaN	NaN	NaN	NaN	NaN	NaN	NaN
0,610462	0,404734	0,785137	-0,767487	1,02687	-1,32938	0,980131	0,136843	0,979968	0,745946
1,56904	1,36646	2,49819	3,20638	3,42506	1,99104	2,48401	2,76891	2,65586	3,00902
NaN	NaN	NaN	NaN	NaN	-1,35043	NaN	NaN	NaN	NaN
2,16166	1,48166	0,0946693	1,75145	1,88251	1,58632	1,84471	1,76054	1,80561	1,82236
-0,05299	0,492945	1,08802	0,627533	3,04941	-2,37869	2,11165	0,501242	0,709365	1,22292
-0,656961	-0,213987	-1,32964	-1,22882	NaN	-3,13905	-0,748699	-1,19393	-0,613065	-1,15065
NaN	NaN	NaN	NaN	NaN	NaN	NaN	-0,138073	0,545215	-0,495774
-0,753225	-0,143652	-1,34297	-0,902643	0,185019	-0,66131	-0,828243	-0,843241	-0,259467	0,0344343
2,34513	2,72866	1,98449	0,815054	3,21661	-3,14328	1,13009	2,21712	2,22311	2,67531
-2,22663	NaN	-2,45665	-2,78639	NaN	NaN	-2,07867	-1,59294	-1,82611	-1,75503
NaN	1,40061	NaN	NaN	1,00168	-1,49517	1,44969	NaN	NaN	NaN
NaN	NaN	NaN	NaN	NaN	NaN	NaN	NaN	NaN	NaN

Polito et al. Universal ready-to-use immunotherapeutic approach for the treatment of cancer: expanded and activated polyclonal γδ memory T cells.
Phosphorylation sites

4,35892	3,79817	4,98474	4,90193	4,58202	0,165886	4,92131	4,87551	4,61859	4,34942
1,6681	1,0927	1,74788	2,21071	1,41983	2,01324	1,31552	2,01647	1,71537	1,61354
-0,653095	NaN	-0,20591	-0,0801048	-0,0108738	-3,23847	-0,059351	-0,0723457	-0,116452	-0,42019
NaN	NaN	-1,17013	-0,872612	-2,83051	-0,608467	-2,2028	-1,77533	NaN	-1,53119
NaN	NaN	NaN	-0,0685883	NaN	-1,65166	NaN	-2,28648	NaN	NaN
-1,78714	NaN	3,0878	5,79828	-0,155132	6,02046	5,16627	NaN	NaN	NaN
NaN	NaN	NaN	0,38953	-1,98094	NaN	-0,114485	NaN	NaN	NaN
-1,40398	-1,98058	-2,42488	NaN	-0,449247	-5,19559	NaN	-1,78501	NaN	NaN
-1,32245	-1,46076	-1,45252	-1,08657	-1,14749	-1,07967	-1,07095	-1,26168	-1,30379	-1,5367
NaN	NaN	NaN	NaN	NaN	NaN	NaN	NaN	NaN	NaN
NaN	NaN	NaN	NaN	NaN	NaN	NaN	NaN	NaN	NaN
-1,24302	-0,931206	-0,964428	-0,478373	-0,453951	-0,649776	-0,548803	NaN	NaN	-1,22059
-0,774601	NaN	-1,0874	-0,602215	NaN	-0,307173	-0,665709	-0,737782	-0,401612	-0,690015
NaN	-2,28941	-3,41341	-3,0672	-2,40533	-2,92198	-1,90911	-1,10594	-0,906711	-1,33734
NaN	-0,537529	-0,271379	0,157465	0,518723	-1,058	0,754469	NaN	-1,51327	-0,908021
-2,06408	-1,22926	-0,830393	0,522293	-0,435339	0,28653	-0,20154	-1,39001	-1,32065	-0,791902
NaN	NaN	NaN	-0,476198	NaN	0,124493	-1,71592	NaN	NaN	NaN
NaN	-0,0961189	0,00927544	0,702082	-0,747936	1,2483	0,224888	NaN	NaN	NaN
0,25544	-0,0961189	0,00927544	0,702082	-0,747936	1,2483	0,224888	-0,99033	-0,530557	-1,00238
-1,93199	-2,31051	-0,252167	NaN	NaN	NaN	NaN	-0,770893	-1,57102	-1,32516
-2,34558	-2,49298	-2,0417	-0,921391	-0,236637	-1,76329	-1,8362	-1,59309	-1,8046	-2,05858
1,76348	2,51333	1,9966	2,10106	2,07895	2,40693	1,36616	1,82567	1,72384	2,03177
1,81875	2,29159	1,49992	1,85519	NaN	1,37258	2,73587	NaN	2,01875	-0,234565
NaN	NaN	NaN	-0,382065	NaN	-1,13578	-0,73064	NaN	NaN	NaN
NaN	NaN	NaN	-1,56949	NaN	0,134243	-2,45978	NaN	NaN	NaN
NaN	NaN	NaN	-0,0775623	NaN	-0,191977	-0,875757	NaN	NaN	NaN
-1,06301	-1,13518	NaN	-0,370255	-1,48844	-0,0234375	0,107206	NaN	NaN	NaN
NaN	NaN	NaN	-1,06738	-3,76266	-1,51254	-2,38492	-2,1378	-2,83134	-3,19265
-0,814947	-0,955229	0,340902	0,246262	0,377213	-1,15127	-0,578629	-2,09316	-1,52639	NaN
NaN	NaN	NaN	-2,59298	-1,74774	-2,79521	-2,18552	NaN	NaN	NaN
NaN	-2,06822	-2,71167	-2,19442	-0,946938	NaN	-2,25849	NaN	NaN	NaN
NaN	NaN	NaN	NaN	NaN	NaN	NaN	NaN	NaN	NaN
NaN	-1,84826	NaN	-0,835138	-0,239521	0,317728	-1,60721	NaN	NaN	NaN
0,28809	NaN	-0,592081	-0,628233	1,47948	0,799923	-0,797155	NaN	NaN	NaN

NaN	NaN	NaN	-0,283327	NaN	-0,728918	-1,21147	NaN	NaN	NaN
NaN	NaN	NaN	0,174879	-2,31331	1,68767	0,306028	-1,49598	-1,67184	-2,28938
-0,346771	-0,591881	-0,846996	-0,989359	-0,161064	-0,176184	-0,0716515	-1,04091	-0,672067	-1,02317
NaN	NaN	NaN	0,179123	0,0820389	0,547369	-1,61246	NaN	NaN	NaN
NaN	NaN	NaN	NaN	NaN	NaN	NaN	NaN	NaN	NaN
NaN	NaN	2,26439	2,69696	1,9381	NaN	NaN	2,38572	2,58858	1,90232
NaN	NaN	NaN	-0,962494	NaN	0,43157	-1,06024	NaN	NaN	NaN
NaN	NaN	NaN	NaN	-0,659456	NaN	-0,847013	-1,71014	-1,6105	-2,93873
-0,119545	-0,529749	0,206797	0,0874615	0,132088	-0,227453	NaN	-0,124414	-0,109111	NaN
0,506847	NaN	-0,0301781	NaN	NaN	NaN	NaN	0,258804	0,18382	0,0180292
NaN	NaN	-1,61813	NaN	NaN	NaN	-1,54635	-1,24669	-1,60275	-1,403
-1,48651	-0,170698	-0,283764	0,764225	0,609571	NaN	1,24901	-0,0110416	NaN	-0,16648
NaN	NaN	NaN	-0,896915	NaN	-0,526588	-2,9875	NaN	NaN	NaN
NaN	-0,755688	NaN	-0,183693	-0,0968666	0,603498	0,4711	NaN	-0,177487	NaN
0,11281	-2,00651	0,35144	-0,324835	-1,92297	NaN	-0,290384	0,339573	0,1942	-1,0772
NaN	NaN	NaN	-0,329561	-1,46643	NaN	-0,380779	0,517748	0,100232	0,545991
3,79772	4,45787	2,71807	4,12302	3,87925	1,24264	4,65077	1,15116	1,76111	2,88183
-2,22558	-2,62658	-0,723001	-2,39523	0,290115	-2,54679	NaN	-0,486929	-0,855235	-0,324523
-1,36345	-1,14138	-1,05793	-0,368807	-0,376095	-0,512606	-0,466679	-1,38166	-1,4836	-1,15417
-0,835234	-1,10907	-0,360197	0,31172	-0,763924	0,423641	1,39194	-0,940989	-1,68615	-1,66998
-1,77617	-1,04724	-0,936655	-0,503925	-0,327976	NaN	-1,41559	-0,794016	-0,601132	-0,224904
-1,58968	-1,14149	-1,50498	-0,601212	-0,581995	NaN	-0,207102	-1,23109	-0,939376	-0,299254
NaN	NaN	NaN	-1,43191	NaN	1,06416	NaN	NaN	NaN	NaN
-0,705601	0,123844	-0,0270653	-0,485716	-0,162464	0,443163	0,142902	NaN	NaN	NaN
1,26771	1,1813	1,2379	-0,743931	1,40015	-0,241268	0,972408	1,30918	1,33871	0,876178
NaN	NaN	NaN	0,101526	-1,99006	1,01188	NaN	NaN	NaN	NaN
NaN	NaN	NaN	-0,347412	-2,38681	-0,0440445	-0,0749664	NaN	NaN	NaN
1,24171	0,218195	2,25403	3,23569	0,90476	3,16791	2,1918	0,24184	-0,325341	-0,142825
NaN	-0,833252	NaN	-0,211796	NaN	-1,09241	-0,457811	NaN	NaN	NaN
NaN	NaN	NaN	-0,409954	NaN	-0,698477	-1,83748	NaN	NaN	NaN
-2,86571	NaN	-1,25176	0,391632	-1,02042	-0,19705	0,22823	-1,73135	-1,84728	-0,234241
NaN	1,42373	NaN	0,382214	1,53734	NaN	1,33062	NaN	NaN	NaN
1,17239	1,44968	1,2015	0,350702	1,58484	0,595034	1,73386	-0,563707	-0,291062	-0,941215
1,5562	1,55013	1,25745	2,33115	2,45272	-2,70563	2,49253	1,62333	1,72694	1,53162

Polito et al. Universal ready-to-use immunotherapeutic approach for the treatment of cancer: expanded and activated polyclonal γδ memory T cells.
Phosphorylation sites

0,971575	1,58628	0,302299	1,78843	2,24427	1,72364	1,96161	0,197351	0,366479	1,62795
NaN	NaN	NaN	-1,46263	NaN	-0,451303	-1,95637	NaN	NaN	NaN
NaN	NaN	-0,799185	-1,78164	-1,04655	NaN	-1,08164	NaN	NaN	NaN
-0,460506	-1,11533	1,01648	-1,63884	0,147213	NaN	-1,7574	0,210238	0,0645132	0,247912
0,0432701	-0,945927	-0,314037	0,774363	1,08486	-0,728283	2,12687	0,456337	0,289117	0,624049
NaN	NaN	NaN	NaN	NaN	1,31864	NaN	NaN	NaN	NaN
-1,57	-2,06541	0,880098	0,238586	-1,38027	0,50704	-0,0269775	0,077095	-0,136601	-0,546489
0,00249672	-1,00661	-0,930098	0,93355	-0,245878	NaN	0,391153	0,453554	-0,0427732	0,100287
-0,789648	-1,42543	-1,13497	-0,182962	-0,76156	NaN	-1,09314	-0,84705	-1,14089	-0,282634
NaN	NaN	NaN	NaN	NaN	NaN	NaN	NaN	NaN	NaN
NaN	NaN	NaN	-1,20481	NaN	-1,55913	-1,32944	NaN	NaN	NaN
NaN	NaN	NaN	-0,261326	NaN	-1,78848	-0,18478	NaN	NaN	NaN
0,606026	0,814053	1,02171	1,23707	0,968307	0,680588	0,990967	-0,00812912	0,220859	0,359765
2,59284	1,86053	3,34159	NaN	2,01826	NaN	NaN	2,17366	2,18081	2,26249
-2,17844	-1,512	-1,14605	-2,86912	-0,294987	NaN	-0,980864	-1,66934	-1,75981	-0,698333
0,00153923	-2,04444	-1,12829	NaN	-0,561541	NaN	NaN	-0,139143	-1,10128	-1,23581
NaN	NaN	NaN	-1,03327	-0,849403	NaN	-0,374798	NaN	NaN	NaN
NaN	NaN	NaN	NaN	NaN	NaN	NaN	NaN	NaN	NaN
0,249199	-0,476583	NaN	-1,25605	0,140495	NaN	-0,694569	-0,143105	-0,739053	-0,551862
-0,780571	0,316668	0,247431	NaN	0,415022	-2,05503	NaN	-0,710831	-0,909713	-0,405707
-1,34813	NaN	-1,81767	0,0114803	-0,337788	1,05997	-0,732012	NaN	NaN	NaN
NaN	NaN	NaN	NaN	NaN	NaN	NaN	NaN	NaN	NaN
-2,21694	-2,33011	-1,19636	-1,12587	-0,916512	-2,50789	-1,29866	-1,74759	-1,222	-1,81207
-1,19792	-2,87761	-1,31654	-1,82481	NaN	NaN	NaN	NaN	NaN	NaN
NaN	NaN	-0,949041	-1,49626	NaN	-1,34652	-1,00188	NaN	NaN	NaN
2,29548	2,39016	2,04131	1,49907	1,36271	1,07882	1,64254	2,04068	1,75425	1,44391
2,50415	2,88374	2,75163	3,4698	0,964218	3,56538	3,2472	3,56592	3,82134	3,57386
1,02026	1,40415	0,789192	1,31509	0,782465	NaN	1,48541	0,92182	0,338855	0,784694
-0,618519	1,7494	-1,29225	-0,351675	-0,779018	0,261757	-0,461039	-1,4912	-1,51843	-1,06995
-0,0195065	-0,0946884	0,139059	-1,09908	-0,355614	-0,650322	-0,0993462	-0,0553474	0,0812235	0,0998163
0,305622	0,804541	1,62373	1,1394	2,04325	-2,287	1,44814	0,995102	1,13938	0,837276
NaN	NaN	NaN	-0,556702	NaN	-0,360409	-2,08311	NaN	NaN	NaN
NaN	NaN	NaN	-1,01187	NaN	-0,580564	-2,38352	NaN	NaN	NaN
-0,508503	NaN	-0,407293	1,11822	1,67628	0,172386	0,393164	NaN	-0,38074	0,883162

Polito et al. Universal ready-to-use immunotherapeutic approach for the treatment of cancer: expanded and activated polyclonal γδ memory T cells.
Phosphorylation sites

NaN	NaN	NaN	NaN	NaN	NaN	NaN	-2,2509	-2,9139	-2,69035
-0,960026	NaN	0,130215	0,217777	-2,71072	0,552771	-0,0962372	-0,134373	0,196296	-0,390136
-2,06707	-3,25327	-2,2356	-1,41201	NaN	-1,19132	-1,99674	NaN	NaN	NaN
-1,2294	-1,68243	-0,927837	-1,14777	-0,95866	NaN	-0,578262	-1,64668	-0,831391	-1,36941
-1,37272	-0,953629	-0,396566	-0,208607	0,394403	-0,152948	0,142134	-0,693182	-0,924508	-0,605638
4,63391	4,55696	1,29577	3,95326	3,00643	3,31376	4,35117	1,59711	-0,0990591	2,03193
NaN	NaN	NaN	-2,82703	NaN	-1,66152	-2,4512	NaN	NaN	NaN
NaN	-1,28922	-0,181643	-2,87814	NaN	-2,18812	-2,46347	-1,64636	-1,59499	-0,826402
NaN	NaN	NaN	NaN	NaN	NaN	NaN	NaN	NaN	NaN
NaN	NaN	NaN	NaN	NaN	NaN	NaN	-1,75894	0,194379	0,844451
NaN	NaN	NaN	-1,05163	NaN	-0,617979	-2,38002	NaN	NaN	NaN
0,463913	-0,624964	-1,1875	-1,18779	-0,309429	NaN	-1,07161	-1,71816	-0,830516	-1,55871
NaN	NaN	NaN	-1,79453	NaN	-1,50208	-1,34174	-2,63134	-2,3283	-2,86091
-0,115913	-1,05317	-0,838518	0,52651	-0,60659	NaN	0,861904	NaN	0,0536261	NaN
NaN	NaN	NaN	0,199196	NaN	1,03094	-0,138096	NaN	NaN	NaN
NaN	NaN	0,298306	1,65494	NaN	1,08713	0,459932	0,163679	0,822583	0,709079
NaN	NaN	NaN	-0,817894	-2,18197	NaN	-1,8872	-2,18363	NaN	-1,80997
NaN	NaN	NaN	NaN	NaN	NaN	NaN	NaN	NaN	NaN
NaN	-1,96383	NaN	NaN	NaN	NaN	NaN	NaN	NaN	NaN
NaN	NaN	NaN	NaN	NaN	NaN	NaN	NaN	NaN	NaN
NaN	NaN	NaN	NaN	NaN	0,569582	NaN	NaN	NaN	NaN
-0,815302	-0,659193	NaN	-0,647655	NaN	NaN	-0,768511	-1,30589	-0,725356	-1,68939
NaN	-3,51183	-2,36655	NaN	NaN	-2,70064	-0,454531	NaN	NaN	NaN
2,64437	3,22804	3,12812	3,47056	3,66655	3,53075	3,66998	3,00825	2,79773	3,14809
NaN	NaN	NaN	-0,329746	-2,72446	-0,0476322	NaN	NaN	NaN	NaN
NaN	-1,51953	-1,23022	NaN	-1,91522	NaN	-0,953773	-0,187832	NaN	0,787354
-0,871363	-1,51953	1,16273	0,175091	-0,0237083	0,549335	0,544121	0,717337	0,696008	-0,646113
5,41619	4,32498	4,66492	4,6561	4,03442	3,42939	4,64667	4,79191	5,01622	4,02341
3,23236	3,09644	2,59637	-1,50646	0,458668	NaN	0,168301	-7,44E-05	2,22399	1,70971
3,46663	2,32194	2,87592	0,775223	3,45111	NaN	0,31308	3,20557	3,14697	2,98465
2,77826	2,97267	3,28071	2,66076	2,53405	3,37925	2,9822	2,92862	2,64403	2,55921
3,19694	1,56643	2,52915	3,22825	3,35125	0,471615	1,64719	2,16112	-0,894078	1,38274
NaN	NaN	1,50784	0,352718	NaN	NaN	1,09181	-0,0465107	0,184777	0,0345469
3,87919	6,24689	5,03053	5,2124	5,12803	5,6446	4,93985	4,1132	3,96862	5,0747

Polito et al. Universal ready-to-use immunotherapeutic approach for the treatment of cancer: expanded and activated polyclonal γδ memory T cells.
Phosphorylation sites

-0,999063	-1,77185	-0,878956	NaN	NaN	NaN	NaN	NaN	NaN	NaN
NaN	NaN	NaN	NaN	NaN	NaN	NaN	-0,0589809	NaN	-0,870614
1,19294	-0,0585804	2,66245	2,0947	1,22913	NaN	2,58914	1,05635	0,869922	0,794782
-0,8393	-0,487856	-0,862946	0,381084	-0,348356	-0,81531	0,146784	-0,377672	-0,395503	-0,503789
3,84213	4,37561	3,89619	3,69466	4,19646	4,08292	4,04682	4,44439	3,9387	3,72522
5,85332	6,06676	6,18736	5,07818	6,27589	4,59315	5,40169	6,12812	6,01305	5,86248
NaN	-3,2279	1,14664	1,25628	NaN	-0,317635	0,876585	-2,77227	NaN	NaN
0,569101	-0,664566	0,156202	1,07184	-0,284351	0,513603	1,25791	0,836536	0,830216	0,886022
-1,52075	-1,8558	2,22653	-0,219139	-1,29157	-0,0699749	-0,0556526	2,30913	1,95796	1,66373
1,60639	2,86455	2,82526	2,30635	3,25363	0,847319	3,49249	2,45709	2,16973	2,8256
NaN	NaN	NaN	NaN	NaN	NaN	NaN	NaN	NaN	NaN
1,09447	0,858807	1,32432	0,555191	1,75024	-0,416195	1,2163	1,68708	1,89479	1,35735
-1,43177	-1,64697	-1,36186	-0,634239	-0,945679	-0,359655	-0,289047	NaN	NaN	NaN
-0,527584	0,285694	NaN	-0,0907536	0,00221634	-0,264977	0,430271	-0,47246	-0,489551	0,462182
NaN	NaN	NaN	-1,2634	-0,596367	-2,13721	-0,325491	0,139496	-0,0133924	0,413218
-1,17723	-1,2152	-0,985342	NaN	-1,41313	-2,38431	-0,864241	-0,879629	-0,769843	-0,293506
3,9673	4,23981	4,14422	4,11158	4,63447	4,59488	4,34569	4,12097	4,00713	4,02171
4,24395	4,82387	4,39361	4,29506	4,39926	4,42421	4,53476	4,48382	4,15134	4,09284
NaN	-1,29716	NaN	0,0186481	-1,66391	0,401039	-0,0100002	-0,556646	-0,735394	-1,06021
NaN	NaN	NaN	1,11636	-2,78541	NaN	1,61472	NaN	NaN	NaN
0,938299	1,49012	0,306608	0,890469	0,00357819	-2,40625	-0,0314465	0,307987	0,495194	-0,391429
-1,11853	NaN	-0,548553	-1,06734	-0,339622	-3,6186	-0,215277	NaN	-0,214631	-0,313359
-0,215517	1,15699	-0,121214	0,839399	0,771881	1,41481	1,75314	NaN	NaN	1,53582
NaN	-2,04181	-1,91032	-1,75354	NaN	NaN	-1,87207	-1,83752	-1,43477	-2,24421
-1,02308	-0,888128	-1,07021	-1,25671	-0,737089	-0,864666	-1,04116	-1,40406	-1,89233	-1,10497
NaN	NaN	NaN	-0,121559	NaN	-1,21703	-0,796923	NaN	NaN	NaN
NaN	NaN	NaN	0,28223	NaN	0,231455	0,100719	NaN	NaN	NaN
-0,581675	NaN	NaN	NaN	-0,66946	-2,72681	-0,917528	NaN	NaN	NaN
0,189991	1,36561	0,132441	0,845961	-0,772058	NaN	0,6189	-0,929636	0,725455	0,581943
NaN	NaN	NaN	-0,102922	-1,11486	-0,147894	-0,375813	NaN	NaN	NaN
NaN	NaN	NaN	1,33191	-2,00128	NaN	0,217756	NaN	-0,853381	-1,05067
NaN	NaN	NaN	NaN	NaN	NaN	NaN	-0,535343	-0,654334	-1,02238
NaN	-1,80385	NaN	NaN	NaN	NaN	NaN	-1,41194	-0,322364	-2,4201
NaN	NaN	NaN	NaN	NaN	NaN	NaN	-1,38374	-1,2635	-1,29945

NaN	-1,52526	NaN	-0,74021	-1,55255	2,14352	0,213522	NaN	NaN	NaN
NaN	-1,24805	NaN	-0,0553303	NaN	1,73196	-0,731493	NaN	NaN	NaN
-1,47505	-0,378668	-0,78743	-0,778261	-0,444441	NaN	0,209238	-1,02351	-1,21204	0,305776
-0,697325	-4,00526	-1,27722	-0,795227	NaN	-1,56969	-2,85933	-0,261086	-0,258363	-2,51162
1,91671	3,60775	3,1237	2,58353	3,54162	4,64699	3,073	2,94765	2,82296	3,3195
0,656603	0,209599	0,0429344	1,18789	-0,782095	NaN	0,742498	0,140469	0,61222	NaN
3,73193	4,33188	4,07359	3,00387	3,88565	NaN	4,029	4,45193	4,34118	4,48483
NaN	NaN	NaN	NaN	NaN	NaN	NaN	NaN	NaN	NaN
3,1053	3,70054	3,22408	2,74732	3,3978	0,397133	2,97081	3,87928	3,89277	3,39333
-3,09867	-1,06092	-2,03943	-0,211391	-2,93814	0,711578	-0,0440407	-2,11007	-1,79817	-1,32051
1,18393	0,268208	1,05993	2,11754	1,15803	2,56376	2,20771	2,13489	1,48399	1,93911
NaN	NaN	NaN	-1,71458	NaN	-1,50435	-1,02448	NaN	-0,998626	-1,64931
NaN	NaN	NaN	-0,6327	NaN	NaN	NaN	-2,38909	NaN	NaN
NaN	NaN	NaN	NaN	NaN	NaN	NaN	NaN	NaN	NaN
0,122889	0,669043	-0,0304165	1,25428	0,0835419	1,57305	1,33231	-0,119881	0,965652	0,143603
NaN	NaN	NaN	-1,95182	-3,09195	-2,905	NaN	NaN	NaN	NaN
NaN	NaN	NaN	-0,495064	NaN	NaN	-0,31286	NaN	NaN	NaN
NaN	NaN	NaN	NaN	NaN	NaN	NaN	NaN	NaN	NaN
0,868498	0,646729	1,2457	-0,142448	NaN	1,18156	-1,11577	1,06675	1,52825	1,15055
-0,593885	-1,3073	-0,675001	0,878521	-0,838583	-1,39474	-0,818752	-0,622013	-0,865623	-0,916312
0,324888	1,51998	-0,23403	3,91222	-0,420536	-2,63136	1,92276	NaN	0,865314	0,475976
NaN	NaN	NaN	-1,75329	-1,90188	-1,7701	-2,72329	-1,3757	-2,0354	-1,67987
NaN	NaN	NaN	2,09706	NaN	-0,0472603	NaN	NaN	NaN	NaN
NaN	NaN	NaN	0,865665	NaN	2,46323	0,446499	NaN	NaN	NaN
0,874355	0,157337	0,113934	3,44066	1,43905	3,09644	1,28861	0,208382	-0,0852003	0,151021
NaN	NaN	NaN	NaN	NaN	NaN	NaN	NaN	NaN	NaN
NaN	NaN	NaN	-3,19596	NaN	NaN	-3,72293	NaN	NaN	NaN
NaN	-2,20302	-1,6559	0,673668	NaN	1,41894	0,474834	-0,442764	-1,35727	-1,26568
NaN	NaN	NaN	NaN	NaN	NaN	NaN	NaN	NaN	NaN
0,558517	NaN	NaN	-0,477861	-0,903051	0,328457	-0,377813	NaN	NaN	NaN
NaN	NaN	NaN	NaN	NaN	NaN	NaN	NaN	NaN	NaN
NaN	-0,750462	1,12989	-0,208118	1,64708	0,10323	1,46637	NaN	-0,117898	NaN
-0,0317059	-0,536015	-0,535315	-1,31328	0,329567	NaN	NaN	0,315002	0,0152388	0,0558977
-1,75846	-2,58944	-1,40286	NaN	-0,0180836	NaN	-1,42702	-0,671129	-0,625319	-1,13878

-2,80975	NaN	-1,06625	-2,52464	-0,903156	NaN	-1,72261	-1,03885	-1,60537	0,194886
0,475565	-0,436798	0,0423794	-0,912746	0,17382	-1,31747	-0,977867	0,529804	0,302566	1,09146
-1,0513	-3,89723	0,0164604	-1,16362	-0,877491	NaN	-1,02994	0,877569	0,247138	-0,16558
-1,73494	-2,56619	-2,17807	NaN	NaN	NaN	-1,27424	NaN	NaN	NaN
-1,73494	-2,56619	-2,17807	-1,28778	-2,20867	NaN	NaN	NaN	NaN	NaN
NaN	NaN	NaN	NaN	-2,26632	NaN	NaN	-2,02204	-2,53789	-2,64507
NaN	NaN	NaN	-2,5942	NaN	-0,386253	-2,23305	NaN	NaN	NaN
-1,31505	-2,07476	1,27016	2,13497	0,0476532	2,65252	2,76808	-0,217049	-0,100137	-0,392364
-0,7759	-1,96663	NaN	0,209646	-0,776533	NaN	-0,588306	NaN	-1,4468	NaN
4,03654	3,99606	3,3805	4,34639	3,71024	4,43139	4,5633	3,47875	3,57471	3,76141
NaN	-1,77595	-2,28382	-2,17114	-1,47099	NaN	-1,25007	NaN	NaN	-0,874648
-1,78337	NaN	-0,206913	1,03858	NaN	1,73239	0,848066	NaN	NaN	NaN
-1,21731	-2,57963	-1,59035	NaN	-1,44258	NaN	NaN	-2,22362	-2,04866	-2,56182
NaN	NaN	NaN	-0,456869	-1,53588	-1,3108	-1,10722	NaN	NaN	NaN
NaN	NaN	NaN	3,45472	-0,398556	2,89274	1,79908	NaN	NaN	NaN
3,95238	3,19982	2,60615	3,6596	4,24874	1,20535	3,47026	3,61234	2,86551	3,54577
-1,16497	-1,30657	-1,11468	NaN	-1,05114	-1,99352	NaN	NaN	NaN	NaN
NaN	NaN	NaN	-0,957916	NaN	NaN	-0,799137	-1,18863	-1,0541	-1,71968
-0,711143	-0,638918	-0,793699	-1,27603	-0,173613	NaN	-1,3659	-0,527195	-0,504167	-0,534839
-0,291664	NaN	0,373566	0,223269	0,830118	-0,328173	0,681103	-0,287357	-0,662589	-0,172494
-2,08391	-1,96432	-1,68104	-1,3282	-1,13276	-1,32319	-0,388891	-1,32657	-1,46158	-1,84607
1,14853	-1,07444	0,512913	-0,297647	1,03175	0,400042	0,437681	0,503143	-0,207934	-0,173892
NaN	NaN	NaN	-2,53015	NaN	NaN	NaN	NaN	NaN	NaN
NaN	NaN	NaN	NaN	NaN	NaN	NaN	NaN	NaN	NaN
4,19299	4,02103	3,99946	3,77782	4,42953	4,09721	3,92958	4,34455	4,14983	3,86187
NaN	-1,08808	NaN	-0,936825	NaN	-1,18417	-0,377697	-0,640051	NaN	-0,72479
NaN	NaN	NaN	-2,32659	-2,53375	-2,04531	-2,02148	NaN	NaN	NaN
NaN	NaN	NaN	-0,320765	-3,39237	NaN	-0,357687	NaN	NaN	NaN
NaN	NaN	NaN	NaN	NaN	NaN	NaN	NaN	NaN	NaN
NaN	NaN	NaN	-0,798452	-0,194838	0,275385	-0,0998497	NaN	NaN	NaN
NaN	NaN	NaN	1,29111	NaN	-2,13963	-0,182014	NaN	NaN	NaN
NaN	NaN	NaN	-0,418724	NaN	0,318209	-1,13581	NaN	NaN	NaN
NaN	NaN	NaN	0,53643	NaN	0,786438	-0,453979	NaN	NaN	NaN
NaN	NaN	NaN	-0,0611095	NaN	-1,18221	-0,842999	NaN	NaN	NaN

Polito et al. Universal ready-to-use immunotherapeutic approach for the treatment of cancer: expanded and activated polyclonal γδ memory T cells.
Phosphorylation sites

NaN	NaN	NaN	0,632404	NaN	1,10027	-0,978395	NaN	NaN	NaN
NaN	NaN	NaN	-0,349073	NaN	0,213791	-2,14542	NaN	NaN	NaN
NaN	NaN	NaN	-0,0862389	NaN	0,352308	-2,66855	NaN	NaN	NaN
NaN	NaN	NaN	-0,461164	NaN	-0,0645771	-2,04075	NaN	NaN	NaN
NaN	NaN	NaN	-0,0682812	NaN	0,554647	-1,65941	NaN	NaN	NaN
NaN	NaN	NaN	-1,0399	-2,39868	0,492464	-1,86636	NaN	NaN	NaN
3,81733	2,99887	4,28819	5,32096	4,81399	3,90109	5,01711	NaN	NaN	NaN
NaN	NaN	NaN	0,773073	-3,39581	0,908672	0,0604401	NaN	NaN	NaN
NaN	-0,11046	NaN	-0,426683	0,353243	-0,196363	0,780462	NaN	-1,42571	0,652244
NaN	NaN	NaN	NaN	NaN	NaN	NaN	NaN	NaN	NaN
0,0294285	-1,26581	1,10043	1,79745	0,575043	3,40244	1,6077	0,226402	NaN	0,711324
-1,20882	0,37044	0,122896	1,14689	0,86338	2,12811	0,511698	0,193016	NaN	0,410233
NaN	NaN	NaN	-1,88254	NaN	-1,60011	-2,98233	NaN	NaN	NaN
NaN	NaN	NaN	-2,33927	NaN	-1,69438	-2,52401	NaN	NaN	NaN
-1,24735	-1,63739	-0,513515	-0,039732	-0,773468	0,347513	-0,985413	-1,72631	-0,886817	-1,30829
NaN	-2,23193	-0,800882	-1,44741	NaN	-0,105892	-0,862938	NaN	NaN	NaN
NaN	NaN	NaN	-3,37863	-3,68451	NaN	-1,97391	NaN	NaN	NaN
NaN	NaN	NaN	NaN	-1,32901	-3,94638	-3,07859	NaN	NaN	NaN
0,64794	NaN	-0,977684	NaN	0,1577	NaN	-1,96106	-0,376926	-0,814521	-0,703368
1,35468	2,01169	2,52698	NaN	0,87462	-5,20577	-0,36887	2,55023	2,25069	2,22071
2,26748	1,97539	1,81026	NaN	1,81033	NaN	NaN	1,88623	NaN	NaN
-0,878815	-1,48441	-1,31359	NaN	-0,819641	NaN	-1,4556	-1,31586	-1,64369	-1,62264
-0,653095	1,75024	-0,487221	0,0142498	0,451534	2,76294	0,4809	-0,495729	-0,592261	0,0145121
NaN	NaN	NaN	NaN	NaN	-2,09354	NaN	NaN	NaN	NaN
NaN	NaN	NaN	NaN	NaN	NaN	NaN	NaN	NaN	NaN
NaN	NaN	NaN	NaN	NaN	NaN	NaN	-0,180435	-0,313544	-0,278481
NaN	NaN	NaN	-2,29341	-1,76881	NaN	-0,157331	NaN	NaN	NaN
1,28412	1,47046	1,91873	1,86536	2,04662	1,67785	2,20471	1,77329	1,57938	1,32125
NaN	NaN	NaN	-0,40593	NaN	-1,88437	-0,919344	NaN	NaN	NaN
1,81311	2,67757	2,08942	3,23686	2,47795	2,82405	3,06805	2,12635	2,1475	2,97889
2,00827	3,4847	1,40969	2,25852	3,30382	1,99943	2,7426	2,2318	1,29307	2,98537
4,69232	4,86288	4,68418	4,21981	3,90054	1,62024	4,94073	4,11982	4,61843	4,15354
-0,00377846	-1,31558	NaN	-0,717422	-0,601664	NaN	-0,821592	-0,218964	1,44083	-0,0449018
4,36684	4,48401	4,22963	4,66287	3,90073	3,66652	4,88449	3,89665	3,97411	4,20372

Polito et al. Universal ready-to-use immunotherapeutic approach for the treatment of cancer: expanded and activated polyclonal γδ memory T cells.
Phosphorylation sites

1,3468	1,62159	1,46618	1,84186	2,02366	-0,288889	1,801	1,98247	1,46747	1,59892
3,67339	4,5973	4,2908	4,21537	4,63	4,82178	4,36642	4,28923	4,02438	4,7475
0,271395	-0,283199	0,0787411	-0,277477	-0,184267	-0,261093	-0,236525	NaN	NaN	NaN
NaN	4,04218	2,61984	1,85671	1,99134	2,04962	1,79786	1,99873	2,07925	2,07315
NaN	1,33827	-0,569162	-0,926552	-1,74985	NaN	0,251131	NaN	NaN	0,567676
-1,42175	1,3272	NaN	NaN	-3,9093	-1,31828	1,28906	-0,184088	-0,251412	1,39186
2,10197	2,56319	2,08762	1,15154	1,89472	1,14393	1,72867	2,73608	2,07542	2,29278
-1,10892	NaN	2,62507	0,967882	NaN	NaN	1,59162	0,743124	0,778666	0,0829515
NaN	NaN	NaN	-0,581354	NaN	-2,48162	-1,11819	NaN	NaN	NaN
2,97819	2,31639	2,21959	3,85048	2,78204	3,83217	3,27906	2,45795	2,35203	3,20392
NaN	-0,530895	NaN	1,79071	NaN	NaN	0,530045	NaN	NaN	NaN
NaN	NaN	NaN	2,687	1,37043	2,60289	2,71964	1,50344	1,16256	1,13129
NaN	NaN	NaN	-1,88995	-2,49594	-1,29857	-1,42882	NaN	NaN	NaN
4,79951	3,27687	3,2772	3,25732	1,91273	2,32874	3,07202	3,82299	3,96113	2,20168
-0,911482	0,0530682	-0,0186443	-1,39499	-0,293139	NaN	-0,258009	-0,599388	-0,679669	-0,682887
1,71861	2,57246	1,97163	1,72689	2,20584	2,22596	2,10771	1,96877	2,07556	1,63771
NaN	-0,140076	-0,737558	-0,678038	-0,243587	1,0018	-0,314833	NaN	NaN	-0,20107
0,853754	-0,295708	1,64483	1,78571	0,247042	1,66147	1,78105	-1,32632	-1,21785	-1,36521
NaN	NaN	NaN	0,0995026	NaN	0,239191	-0,977604	NaN	NaN	NaN
-2,06838	NaN	0,469332	0,781652	NaN	2,42587	0,306787	NaN	NaN	NaN
-0,319748	NaN	NaN	-0,661051	-0,0738506	-1,04259	-0,351088	NaN	NaN	NaN
-0,78957	-1,53002	-0,317335	-0,0853024	0,210159	-2,67983	-0,176523	NaN	NaN	NaN
NaN	NaN	NaN	-0,552732	NaN	NaN	-1,71768	NaN	NaN	NaN
1,29298	1,76878	4,10731	2,16574	2,02562	1,5724	1,81491	2,45777	1,57938	2,23453
-0,725391	NaN	0,117102	NaN	NaN	0,684544	0,362289	-1,70818	-1,63589	-2,27564
-1,25676	-0,733786	-0,821749	NaN	NaN	NaN	NaN	-1,61501	-1,3043	-1,39447
NaN	NaN	-0,0311737	0,378119	NaN	-0,273743	0,145531	-3,014	-2,81085	-3,34682
NaN	NaN	NaN	-1,04798	NaN	-0,269129	-0,817333	NaN	NaN	NaN
NaN	NaN	NaN	1,46957	NaN	1,9562	0,018961	NaN	NaN	NaN
NaN	NaN	NaN	-1,7328	NaN	-2,4946	-1,12125	NaN	NaN	NaN
NaN	NaN	NaN	NaN	NaN	NaN	NaN	NaN	NaN	NaN
NaN	NaN	NaN	1,72161	-1,16332	2,32901	1,40119	-1,44592	-1,84735	NaN
0,255627	-1,10423	-1,37022	1,20195	-0,124775	1,35137	0,878897	-0,0212822	0,163289	-0,495774
-1,4372	-1,79826	NaN	0,72027	-1,77873	1,12262	0,785656	-0,817165	NaN	NaN

Polito et al. Universal ready-to-use immunotherapeutic approach for the treatment of cancer: expanded and activated polyclonal γδ memory T cells.
Phosphorylation sites

6,56297	7,33468	5,93097	6,95756	6,8206	4,61473	7,24687	5,9057	5,17117	5,93506
NaN	NaN	-1,5147	-1,99492	NaN	-1,78448	-1,60517	NaN	NaN	NaN
NaN	NaN	NaN	-2,10038	-3,58017	-1,24028	-2,55506	NaN	NaN	NaN
1,40553	NaN	0,60078	2,20287	1,88212	1,07853	2,15233	1,86824	1,69201	1,60376
1,19743	0,282238	-0,580021	1,96431	0,914762	1,83141	1,037	0,666574	-0,115451	0,082387
-2,21042	-0,0463181	1,00913	2,87927	NaN	2,75213	2,94449	-1,99147	NaN	-1,28311
NaN	NaN	NaN	-2,14659	NaN	-1,86172	-0,487959	NaN	NaN	NaN
NaN	-1,7791	-0,476799	-2,01722	0,855043	-2,62624	-1,29235	-1,35093	-1,06895	-1,01243
2,5604	1,15244	2,24927	1,55956	1,54076	0,235735	1,51347	1,40224	1,16329	1,11867
0,992479	0,675983	NaN	2,08216	NaN	2,69822	1,48221	NaN	NaN	NaN
NaN	NaN	NaN	NaN	NaN	NaN	NaN	NaN	NaN	NaN
-0,562166	-1,85659	-0,145416	-0,147184	0,643732	-1,18616	-0,173018	-0,979273	-0,269418	-0,580573
NaN	NaN	NaN	-2,01967	-4,78459	-1,80963	NaN	NaN	NaN	NaN
NaN	NaN	NaN	-1,35527	NaN	-2,33432	-2,50308	NaN	NaN	NaN
0,844336	0,473778	0,350758	0,453617	0,738527	0,944916	0,468647	1,26896	NaN	0,436128
0,640226	-0,129438	1,11048	1,03356	1,08029	1,59757	0,456337	0,680445	0,421784	1,04573
0,540783	-0,020649	0,688753	1,60336	0,651897	-0,0152988	0,949331	0,228661	0,372766	2,23419
1,50301	2,62593	1,26638	1,57741	NaN	3,36188	NaN	NaN	0,745778	0,294631
-1,09155	-1,92208	-1,64678	NaN	-0,917694	-2,19348	-2,22279	-1,16791	-1,41834	-1,63741
-0,752811	NaN	-0,804691	-0,410589	-0,362286	0,0176277	-1,82642	-0,394196	-1,02422	-0,209165
2,20601	1,43846	1,00945	2,82024	1,38781	3,66605	2,80321	1,73821	1,69423	1,42309
1,10176	0,49217	0,199654	1,31265	1,22451	0,279932	1,07037	0,265688	1,07532	1,04127
NaN	NaN	NaN	-1,92756	NaN	-1,66684	-1,98272	NaN	NaN	NaN
-1,81428	-1,37095	-2,26118	NaN	-1,91995	NaN	NaN	-2,39603	-2,47577	-3,02945
NaN	NaN	NaN	NaN	NaN	-0,716599	NaN	1,09855	1,87603	2,12405
NaN	-0,317907	-0,83326	0,437826	NaN	-0,087822	-0,54319	-0,0540791	-0,68893	-1,13393
0,998302	NaN	-0,30673	-0,124943	1,0217	NaN	-0,0195503	0,518671	-0,408347	0,20017
NaN	NaN	NaN	-1,62732	NaN	-1,98372	-1,75581	NaN	NaN	NaN
-1,60135	-1,23567	-1,08636	-1,01046	-0,728914	NaN	-1,04222	NaN	NaN	NaN
NaN	0,48562	NaN	0,584785	NaN	0,582445	0,600876	NaN	NaN	NaN
-0,354143	-0,735342	-0,91703	0,0906582	-0,0723839	0,123283	0,124489	-1,24078	-1,38931	-1,17464
-0,208509	-0,86516	-1,25287	-0,339785	-0,0488415	NaN	-0,00447655	-0,707407	-1,28338	-1,02854
NaN	NaN	NaN	NaN	NaN	NaN	NaN	NaN	NaN	NaN
NaN	NaN	NaN	-2,57239	NaN	-1,03123	-1,44924	NaN	NaN	NaN

NaN	NaN	NaN	NaN	NaN	NaN	NaN	NaN	NaN	NaN
-0,830622	-1,36653	-1,48805	-0,118204	-1,43565	0,342985	-0,346022	-1,18903	-1,79006	-1,82339
NaN	NaN	NaN	-0,0365047	-1,2922	NaN	-0,156061	-1,33826	-1,10053	-1,2792
0,0867958	0,376812	-1,01815	1,31532	0,487143	1,0637	0,229698	0,245127	-0,330129	-0,156981
0,0837021	0,56432	1,12824	0,404882	1,24972	1,24537	1,2566	0,920238	NaN	0,91941
-0,456244	-0,999174	1,29067	1,25852	0,785734	1,6246	1,07409	0,794588	0,753463	0,63448
NaN	NaN	NaN	NaN	-0,918552	NaN	-2,33321	-2,29919	-3,26447	-1,57263
-0,915562	NaN	0,0719204	2,42789	-0,553883	1,78199	1,08814	1,38343	1,5767	1,18406
NaN	NaN	NaN	NaN	NaN	NaN	NaN	NaN	NaN	NaN
2,28888	3,40842	3,25379	1,70473	3,6044	NaN	3,17114	3,70111	3,62351	3,59108
NaN	NaN	NaN	NaN	NaN	NaN	NaN	NaN	NaN	NaN
-1,36419	-0,84004	-1,69614	-0,421652	0,195957	-1,23821	-0,195053	NaN	NaN	NaN
NaN	NaN	NaN	-2,08135	-3,36431	NaN	-2,65061	NaN	NaN	NaN
-0,799637	-0,268356	-0,829903	-0,0647774	NaN	0,852901	-0,245186	-1,34274	-1,92031	-1,37488
NaN	NaN	NaN	NaN	NaN	NaN	NaN	NaN	NaN	NaN
NaN	NaN	NaN	NaN	NaN	NaN	NaN	NaN	NaN	NaN
NaN	NaN	NaN	-0,372871	NaN	NaN	-0,844522	NaN	NaN	NaN
-0,533226	-1,55237	-0,970604	NaN	-0,860821	-1,31291	NaN	-1,19718	-1,09987	NaN
0,00321007	NaN	0,133408	-0,439564	-0,241335	0,480833	-0,643826	0,176537	0,0441103	-0,649421
NaN	NaN	NaN	-2,12984	NaN	0,577635	-1,65673	NaN	NaN	NaN
-2,78634	NaN	-1,29371	0,0517273	-3,2938	0,620823	-0,274918	-1,67085	-1,58062	-0,997191
NaN	NaN	NaN	NaN	NaN	NaN	NaN	-2,05235	-2,4968	-2,83727
NaN	NaN	NaN	NaN	NaN	-2,60502	-1,57543	NaN	NaN	NaN
NaN	NaN	NaN	2,65339	-0,486963	2,40013	1,96527	NaN	NaN	NaN
NaN	NaN	NaN	-0,298462	NaN	-1,31675	-0,594097	NaN	NaN	NaN
0,170252	0,568842	0,666224	1,95831	NaN	1,91974	NaN	1,22173	1,13331	1,30062
1,19762	2,29693	1,93523	2,3502	2,24367	2,94041	2,21056	2,18116	2,52276	1,74547
-0,509615	0,332962	0,712231	1,91432	-0,608713	2,30372	1,26713	-0,130407	0,0495901	-0,390136
NaN	NaN	NaN	NaN	NaN	NaN	NaN	NaN	NaN	NaN
-2,67727	0,449684	-1,2651	-1,03994	NaN	-2,09892	-1,0114	-2,43489	-2,44551	0,238763
-0,693132	-0,346739	-0,507839	0,377693	0,634512	1,04936	0,473452	-0,808315	-0,759356	-0,604554
-0,456305	-0,699659	-0,632452	-1,37946	-0,0449638	NaN	-1,05363	-0,600826	-1,08884	-1,73793
NaN	NaN	NaN	-0,916315	-1,01973	NaN	-0,746712	NaN	NaN	NaN
NaN	NaN	NaN	NaN	-1,38605	-1,08632	NaN	NaN	NaN	NaN

Polito et al. Universal ready-to-use immunotherapeutic approach for the treatment of cancer: expanded and activated polyclonal γδ memory T cells.
Phosphorylation sites

NaN	NaN	NaN	NaN	NaN	NaN	NaN	NaN	NaN	NaN
NaN	NaN	NaN	-1,29453	-3,29386	-2,69869	-1,71123	NaN	NaN	NaN
NaN	NaN	-2,10558	-2,0164	NaN	-1,17895	-2,44547	NaN	NaN	NaN
NaN	NaN	NaN	NaN	NaN	NaN	NaN	NaN	NaN	NaN
NaN	NaN	NaN	NaN	NaN	NaN	NaN	NaN	NaN	NaN
-1,3243	-1,51085	-1,64208	NaN	NaN	NaN	NaN	NaN	NaN	NaN
NaN	NaN	NaN	0,0414219	NaN	0,2055	0,589724	NaN	NaN	NaN
NaN	NaN	NaN	0,056509	-0,445911	0,167593	-0,542931	NaN	NaN	NaN
NaN	NaN	NaN	0,737682	NaN	NaN	-3,85671	NaN	NaN	NaN
-0,664513	-0,349693	-0,148279	0,965906	0,450306	0,404213	0,422489	NaN	NaN	NaN
NaN	NaN	NaN	-0,724573	-0,521729	-1,22205	0,0305157	NaN	NaN	NaN
3,26018	2,64894	2,71391	2,45318	3,45391	0,385895	2,60612	3,24037	2,97648	2,84253
1,52931	2,24746	2,68025	0,86132	2,3717	NaN	2,0854	NaN	NaN	2,09641
2,0224	2,15044	2,15661	2,92498	2,51289	3,21823	2,74253	2,64582	2,84736	2,28291
0,800924	1,44265	1,19956	1,34261	1,76663	-2,52283	1,50373	1,35348	1,50633	1,72194
NaN	NaN	NaN	-1,41529	-2,38748	NaN	-0,922729	NaN	NaN	NaN
0,506313	-0,498051	-0,419205	0,716696	-0,00145149	1,05675	1,12108	0,0506325	-0,220807	-0,676417
0,347185	0,444427	0,556499	0,60656	0,253639	-1,00673	0,480724	NaN	0,51525	NaN
0,784042	1,56353	1,28659	1,0981	1,32201	1,14593	0,359367	-0,113337	-0,405301	0,348939
0,10708	1,49748	1,28737	1,35099	1,98216	-0,234627	2,04439	1,09446	0,732261	1,57038
NaN	NaN	NaN	-0,305214	NaN	0,998442	-0,803459	NaN	NaN	NaN
NaN	NaN	NaN	NaN	NaN	NaN	NaN	NaN	NaN	NaN
NaN	NaN	NaN	NaN	NaN	NaN	NaN	NaN	NaN	NaN
NaN	NaN	NaN	-0,63867	-1,66445	NaN	-0,731529	NaN	NaN	NaN
NaN	NaN	NaN	NaN	NaN	NaN	NaN	NaN	NaN	NaN
NaN	NaN	NaN	NaN	NaN	NaN	NaN	NaN	NaN	NaN
NaN	NaN	NaN	NaN	NaN	NaN	NaN	NaN	NaN	NaN
-0,14904	-1,20105	1,1748	4,95498	-0,0234184	1,47666	3,92389	-0,204519	-0,107211	-0,645431
NaN	NaN	NaN	0,840631	NaN	NaN	NaN	NaN	NaN	NaN
NaN	NaN	NaN	-0,909119	-1,91073	-1,04274	0,556026	-1,45718	-2,09961	-1,88444
-1,27647	0,854506	-1,16081	0,444731	0,154026	5,08822	1,21022	-1,13939	-1,18004	0,379304
1,44426	0,65229	0,949682	2,3357	1,64262	-2,18153	2,05082	0,960915	0,998975	0,706269
2,0159	0,362465	1,86877	1,51941	-1,77347	-0,941246	2,17933	1,50291	1,18212	1,66134
1,00232	0,194023	1,03749	1,12826	1,73904	0,953421	1,80887	NaN	1,26126	1,37046

Polito et al. Universal ready-to-use immunotherapeutic approach for the treatment of cancer: expanded and activated polyclonal γδ memory T cells.
Phosphorylation sites

-2,51563	NaN	0,143881	-0,801558	-0,748079	-0,228857	NaN	NaN	NaN	NaN
NaN	NaN	NaN	-1,83731	NaN	-1,17871	-1,87289	NaN	NaN	NaN
-0,291008	NaN	0,784769	0,511501	NaN	NaN	0,464458	0,164925	-0,0856791	0,755084
-1,07697	-1,07887	-1,06523	NaN	NaN	NaN	NaN	-0,734541	-0,744023	-4,19338
NaN	NaN	NaN	-2,2452	NaN	-0,702078	-1,82468	NaN	NaN	NaN
NaN	NaN	NaN	NaN	NaN	NaN	NaN	NaN	NaN	NaN
NaN	NaN	NaN	NaN	NaN	NaN	NaN	NaN	NaN	NaN
-1,50673	NaN	-0,219889	-1,78886	0,11297	NaN	-0,340408	0,314159	0,471131	0,298835
NaN	NaN	NaN	-0,948025	NaN	-1,14965	0,179197	NaN	NaN	NaN
1,24293	1,18384	1,67269	1,57161	2,31643	1,67954	1,44494	2,01678	1,52005	1,55906
-1,45913	-0,759111	-1,50492	-0,865294	-1,55999	NaN	-0,882334	-1,59279	-1,54955	-1,71915
NaN	NaN	NaN	NaN	NaN	NaN	-1,46532	-2,02762	-2,0997	-1,78801
NaN	-2,72707	-1,00104	-1,88696	-2,25406	-1,14262	-0,485422	NaN	NaN	NaN
3,47931	3,54933	3,45873	0,869896	3,85074	0,302845	2,48369	2,90026	2,75358	2,67847
2,79009	-0,696501	2,39872	NaN	NaN	1,14366	1,75692	2,35373	2,37407	-1,63285
0,823551	NaN	-0,709593	-2,11878	1,02128	-0,197187	-1,03332	0,805618	1,89247	-1,01072
1,48247	1,43524	1,43367	-0,53273	1,51443	NaN	NaN	1,10215	1,27844	0,453427
-2,1732	-2,28911	-1,29506	NaN	NaN	NaN	NaN	-0,889299	-0,377114	-1,69725
-1,76255	-0,829288	-0,866341	NaN	-0,506905	NaN	-1,09805	NaN	NaN	-1,66983
-0,665007	-0,852165	-0,903446	NaN	-1,69699	NaN	NaN	-0,217976	0,031764	-0,167228
NaN	NaN	NaN	-0,546303	-2,55478	0,875156	-1,64201	NaN	NaN	NaN
NaN	-1,71209	-0,00530815	1,8395	-2,1143	2,29805	1,14816	NaN	-0,00573254	NaN
-1,06473	-2,65269	-1,32347	1,01303	-1,39867	1,47679	0,271103	-1,49015	-1,22493	-1,49833
2,23314	2,60168	2,51494	4,28849	2,58829	3,32958	4,19827	2,41618	2,12426	2,29339
-1,28722	NaN	NaN	0,486397	-1,25277	0,130854	0,193104	-1,34788	-0,916432	-0,723424
0,504694	0,539883	1,41009	3,15412	1,80138	3,19669	3,80432	NaN	NaN	1,22508
-1,662	NaN	-1,89731	-0,772221	-0,89785	-0,846214	-0,77076	NaN	NaN	NaN
0,183285	0,330362	0,818573	2,84299	0,922134	2,77198	2,88967	0,828693	0,734113	0,217841
-1,72163	-0,197582	0,632164	1,20833	2,06597	1,78164	0,89809	-0,530039	-1,38165	0,720405
-0,25205	-0,311049	-0,803913	NaN	-1,50448	0,0253029	0,419901	NaN	NaN	-1,23532
NaN	-0,839649	NaN	NaN	NaN	NaN	NaN	NaN	NaN	NaN
NaN	NaN	NaN	NaN	NaN	NaN	NaN	NaN	NaN	NaN
-0,0393696	0,378132	NaN	1,02996	-0,13526	1,96098	0,266354	NaN	NaN	NaN
0,868254	0,677444	0,560099	1,47116	1,16629	NaN	1,26081	1,39968	1,02853	0,764302

Polito et al. Universal ready-to-use immunotherapeutic approach for the treatment of cancer: expanded and activated polyclonal γδ memory T cells.
Phosphorylation sites

1,32923	0,838478	2,11949	1,89487	1,92371	-1,909	2,56146	2,28533	2,25341	1,94341
-1,8288	NaN	-1,79344	-0,29084	-1,53679	NaN	-0,786053	-0,725067	-1,47343	NaN
0,39492	-0,932899	0,914579	1,60638	0,435982	1,66306	1,46556	-0,258516	0,424262	1,66764
-3,14002	-0,779396	NaN	NaN	NaN	NaN	NaN	-2,59745	-2,23913	-1,57845
NaN	NaN	NaN	NaN	-2,49261	NaN	-2,13292	NaN	NaN	NaN
NaN	NaN	NaN	NaN	NaN	NaN	NaN	NaN	NaN	NaN
NaN	-0,212795	NaN	-1,16507	0,687925	-0,506378	NaN	0,0195217	NaN	0,0849428
NaN	NaN	NaN	2,68443	-1,65537	2,03939	2,39903	NaN	-3,4529	NaN
-1,92816	NaN	-1,09662	NaN	-0,204206	NaN	NaN	-1,19793	-1,50527	-0,264354
0,291832	NaN	0,0534115	-1,22878	-0,813782	-2,5316	-0,0455399	-1,31959	-0,825128	-0,790353
NaN	0,0788002	NaN	-0,885155	0,0290756	1,70041	-0,843721	NaN	NaN	NaN
NaN	NaN	NaN	-0,485279	-2,06648	NaN	NaN	-2,2509	-1,91977	-2,29196
0,16795	0,715986	-0,058506	0,17395	-0,00125313	-0,207113	0,397495	-0,194267	0,535058	-0,666331
2,96456	2,54342	3,18476	2,89401	4,07901	0,545071	2,8512	3,44908	2,78689	2,87727
-2,26002	-0,671349	-1,45159	-1,42705	1,28912	-1,17675	-0,707428	-0,505541	-1,27308	-0,272758
3,57002	3,4641	3,70435	1,92936	3,59009	2,73682	NaN	NaN	2,81916	3,89475
NaN	NaN	NaN	-1,54757	NaN	-1,3255	-2,1292	NaN	NaN	NaN
NaN	NaN	NaN	-1,11647	NaN	NaN	-1,81584	NaN	NaN	NaN
-1,46084	-2,00664	-1,26995	0,774794	-1,38494	1,80915	0,481876	-2,63009	NaN	-2,16303
NaN	NaN	NaN	1,81542	-1,28803	NaN	1,88417	-2,14771	-2,79624	NaN
-2,22987	NaN	-0,820812	-0,950758	0,155046	-0,575396	-0,605001	NaN	NaN	NaN
NaN	NaN	NaN	2,67061	-1,1852	2,58823	1,22014	-0,853479	NaN	NaN
NaN	NaN	NaN	2,77729	NaN	1,43028	2,09814	NaN	NaN	NaN
-1,43998	-0,726603	-0,899527	-0,968489	-0,799112	-1,44625	1,25483	-0,923756	-1,43145	-0,589767
-1,59641	-1,08451	-1,07578	-0,574987	-1,00916	-0,0512295	-0,398373	-1,62343	-1,67937	-0,270736
NaN	NaN	NaN	NaN	NaN	NaN	-2,74727	NaN	NaN	NaN
2,64437	3,22804	3,12812	3,47056	3,66655	3,53075	3,66998	3,00825	2,79773	3,14809
NaN	NaN	NaN	NaN	NaN	NaN	NaN	NaN	NaN	NaN
NaN	-1,30877	NaN	-1,32244	-1,82825	-0,696417	-0,679888	NaN	NaN	NaN
NaN	NaN	NaN	-1,98702	NaN	-2,34105	-3,18529	NaN	NaN	NaN
-1,27086	-2,21882	-1,06723	-0,531799	NaN	1,19366	-1,13306	NaN	NaN	NaN
1,93789	0,957972	2,40334	1,46345	2,21021	-1,10409	0,539377	1,534	1,27953	1,33678
0,242838	0,263283	0,0953045	1,15657	0,540058	0,405571	1,21918	0,432268	0,524119	0,6239
NaN	NaN	NaN	NaN	NaN	NaN	NaN	NaN	NaN	NaN

Polito et al. Universal ready-to-use immunotherapeutic approach for the treatment of cancer: expanded and activated polyclonal γδ memory T cells.
Phosphorylation sites

-1,94028	-1,79485	-1,98547	-0,915173	NaN	-1,19975	-0,675848	-1,75187	-1,03057	-1,14729
-2,07081	-1,3638	NaN	-2,11233	-2,05214	-1,62314	-1,83429	NaN	NaN	NaN
-1,34558	-2,63342	-3,14325	NaN	-0,10351	NaN	NaN	-1,35054	-2,95195	-3,06398
1,0143	1,11546	-0,632616	-1,48104	NaN	-0,390001	-1,04429	NaN	1,29324	-3,01348
0,339216	-0,572569	0,772165	-0,233978	0,86058	0,579721	-0,501814	-0,90715	-1,49963	-1,1875
NaN	NaN	NaN	-1,52337	NaN	-2,32379	-1,96734	NaN	NaN	NaN
NaN	NaN	NaN	-0,814528	-0,732027	-0,474327	-1,43655	-0,687626	-0,649629	-0,49989
NaN	NaN	NaN	-2,02909	NaN	-1,34896	-1,63609	NaN	NaN	NaN
NaN	NaN	NaN	-2,69487	NaN	NaN	-1,37173	NaN	NaN	NaN
-0,920748	-0,946285	-0,108065	-1,37435	0,103235	-0,922886	-0,537569	0,0826168	-0,102712	0,277144
2,63585	3,02804	2,35514	2,35631	2,91352	1,87861	2,39708	2,64479	2,03004	2,68405
0,468243	0,30839	0,39213	0,852694	0,724899	-0,632008	0,471212	0,381897	0,476493	1,63054
-1,45606	-1,75235	-0,449942	-0,35807	NaN	-0,0220985	-2,04314	-1,81506	NaN	-1,62781
NaN	NaN	NaN	-1,06322	NaN	-0,931273	-0,743153	NaN	NaN	NaN
0,957047	-0,452168	0,24877	0,741537	1,06134	0,548761	0,87999	1,02642	0,826867	0,540605
0,76473	NaN	1,55354	1,44925	1,46131	1,29085	1,41826	1,78883	1,07921	0,976155
3,15453	3,24434	4,33074	3,06632	4,38286	1,58084	4,2783	4,21503	3,72563	NaN
NaN	-0,594183	-0,372684	-0,701162	1,33998	NaN	0,534834	NaN	-2,96656	0,0585661
1,11615	1,01567	2,3671	0,482323	2,97169	-0,179167	0,513311	1,1562	0,696387	0,889495
-3,32958	-3,08735	-3,28015	NaN	NaN	NaN	NaN	NaN	NaN	NaN
0,0949478	0,534945	0,146048	0,556692	1,11615	0,52747	NaN	NaN	-0,790944	-0,336354
NaN	NaN	NaN	NaN	NaN	NaN	NaN	0,523588	0,943961	0,0148745
NaN	NaN	NaN	NaN	NaN	NaN	NaN	-1,02839	-0,383708	-1,19578
NaN	NaN	NaN	-2,38319	NaN	-1,56497	-1,64851	NaN	NaN	NaN
-0,875738	-1,46381	-0,650639	-0,480757	-1,25264	NaN	-0,250866	-0,768553	-0,915736	-1,55972
-2,86202	-1,38155	-0,631533	-0,67951	-1,15121	-0,456211	0,118305	-1,97716	-1,52644	-1,59937
NaN	NaN	NaN	-1,66629	-2,77226	-0,599995	-2,1016	NaN	NaN	NaN
NaN	NaN	-1,30136	-1,07158	NaN	-2,3439	-0,598455	NaN	NaN	NaN
-1,48776	NaN	4,78226	4,92468	NaN	3,20334	4,31716	0,661095	1,28072	1,00697
2,10856	1,19898	1,75248	2,57136	1,88443	0,994358	1,67349	2,15754	2,28346	2,7244
-1,68989	NaN	-2,08169	NaN	NaN	NaN	NaN	-2,86916	-1,38272	-2,63791
-2,12723	-0,762411	-0,470089	-0,604532	-1,45516	-1,59557	-1,01149	-0,756874	-1,18502	-1,52695
0,646372	1,23332	2,00163	2,04443	0,527134	0,989536	1,42743	2,08008	1,8902	1,79584
NaN	NaN	NaN	-1,01628	-2,15868	-0,959167	-1,44863	NaN	NaN	NaN

-1,73442	NaN	-3,12568	NaN	NaN	NaN	NaN	NaN	-1,59066	NaN
-0,833006	-1,42698	-1,04707	-1,48047	-0,281065	-2,28811	NaN	NaN	NaN	NaN
-0,0466137	-1,74622	1,08703	-0,403521	1,01031	-0,491898	0,619978	0,257908	-0,694428	0,291184
2,86728	3,36886	2,72184	-0,584217	2,70164	-1,33805	0,179514	1,71234	2,82049	1,00045
3,82549	4,58047	3,84309	5,03946	2,60648	4,12034	5,88932	4,00818	5,5743	5,44534
NaN	NaN	-0,156384	NaN	NaN	NaN	NaN	-0,00474739	-0,403998	-0,290977
-1,2294	NaN	2,00206	-2,41049	-1,24932	-0,992374	-1,00802	NaN	NaN	NaN
-2,51283	NaN	-1,2385	-0,546892	NaN	0,0353928	-1,33192	-1,78974	-2,05384	-2,15565
NaN	NaN	NaN	-1,80624	-2,7957	-2,08787	NaN	NaN	NaN	NaN
NaN	NaN	NaN	-0,38106	-0,178656	NaN	-0,60354	NaN	NaN	NaN
NaN	NaN	NaN	-1,93296	NaN	-0,454535	-2,15365	-1,12605	NaN	-1,54465
-2,51947	-4,142	-0,279821	0,119795	-2,41553	1,7436	0,689857	NaN	-1,49125	NaN
NaN	NaN	NaN	-1,29683	NaN	-0,868116	-0,42267	NaN	NaN	NaN
NaN	NaN	NaN	NaN	NaN	NaN	NaN	-0,899174	-1,03091	-0,908795
NaN	NaN	NaN	NaN	NaN	NaN	NaN	-0,235989	-1,09286	-1,91692
2,90841	2,45663	2,90971	4,07888	3,09101	4,86642	2,5155	2,6651	2,53155	2,37422
-0,954609	-1,48271	2,90971	NaN	-1,00738	NaN	-1,42961	-1,36634	-1,75309	-1,40052
-0,200434	-0,184494	-0,144995	0,442568	0,679234	0,661015	0,22966	-0,42417	-0,509995	0,218984
0,237888	NaN	0,134895	-1,36774	0,453196	-1,05409	-2,05312	-0,156275	-0,219924	-0,974196
NaN	NaN	NaN	NaN	NaN	0,996521	NaN	NaN	NaN	NaN
NaN	NaN	NaN	-1,92129	NaN	-2,07792	-1,6244	NaN	NaN	NaN
NaN	NaN	NaN	NaN	-2,37199	-1,14564	-1,33767	NaN	NaN	NaN
-1,46497	-0,869993	0,282377	1,31435	-0,55802	2,34911	1,00696	1,40892	1,06633	1,70021
NaN	NaN	NaN	NaN	NaN	NaN	NaN	NaN	NaN	NaN
NaN	NaN	NaN	-1,21638	-3,60203	-0,10615	-2,09662	NaN	NaN	NaN
NaN	-0,382908	NaN	-0,542013	-1,92459	NaN	-0,0409565	NaN	NaN	NaN
-1,51353	NaN	-1,44731	NaN	-1,3024	NaN	-2,38503	-1,19068	-1,54973	-1,29895
1,3802	2,87172	1,92014	1,87807	1,72302	1,54999	2,70248	2,04588	2,19055	2,09055
-0,0497761	0,681238	-0,0492001	-0,0068531	0,408997	-2,86194	0,488995	-0,342995	0,451425	0,233249
-0,767744	NaN	-0,356665	0,0583096	-0,35071	0,860842	0,477951	NaN	NaN	NaN
-0,342833	-0,289583	NaN	0,114761	0,214674	-0,341032	0,259518	NaN	NaN	NaN
3,54276	2,72652	2,6379	2,58892	3,13167	0,209133	2,69577	2,36646	2,83534	3,16328
NaN	NaN	NaN	-0,606379	-0,399456	-0,995478	0,0351887	NaN	NaN	NaN
NaN	NaN	NaN	NaN	NaN	NaN	NaN	NaN	NaN	NaN

NaN	-2,00808	-1,30189	-0,891653	-1,81909	-0,508474	NaN	NaN	-1,79802	NaN
NaN	NaN	NaN	0,508532	NaN	-0,26512	NaN	NaN	NaN	NaN
NaN	NaN	-2,22774	1,02609	NaN	0,672188	0,00751305	NaN	NaN	NaN
NaN	NaN	NaN	-1,6054	NaN	NaN	NaN	NaN	NaN	NaN
NaN	NaN	NaN	-0,387009	NaN	-0,253788	-1,61801	NaN	NaN	NaN
NaN	NaN	NaN	-0,246922	-2,58581	-1,54868	0,0617256	NaN	NaN	NaN
-0,58378	NaN	-1,08524	-2,93943	NaN	NaN	-1,59305	-1,68129	-0,773555	-0,944346
NaN	-1,33903	NaN	-1,08782	NaN	-3,35747	-0,720846	-1,05784	NaN	-1,6691
2,28828	1,8105	1,89966	2,97152	2,34788	2,30314	2,83438	1,96558	2,14972	1,82217
-0,308428	0,393393	0,781937	1,14922	0,925655	-0,671886	1,56823	0,581059	0,311254	0,852098
-1,6325	-1,20651	-1,11592	-0,946749	-1,15703	-1,11041	-1,51293	-1,14412	-1,22066	-1,37774
-2,81788	-2,06209	-1,53403	-1,76037	-1,50513	NaN	-1,51798	-1,57299	-2,04942	-1,95281
NaN	NaN	NaN	NaN	NaN	NaN	NaN	NaN	NaN	NaN
NaN	NaN	NaN	-0,98415	-1,69708	0,331032	-1,95585	NaN	NaN	NaN
NaN	NaN	NaN	NaN	-1,81069	-2,28507	-2,41989	NaN	NaN	NaN
2,45106	2,27221	1,63503	2,91461	2,15101	3,28773	3,0042	2,21325	1,53003	2,91625
NaN	NaN	NaN	NaN	-2,87779	-0,714867	-2,86242	NaN	NaN	NaN
NaN	NaN	-2,06324	-2,17786	-1,69542	-1,64291	-1,36636	NaN	NaN	NaN
-0,837744	-0,565474	-1,36726	-1,92701	-0,971148	-2,58539	-1,30296	-0,744701	-0,862561	-0,489865
-0,588438	0,156078	-0,139589	NaN	NaN	-0,719002	-1,44394	NaN	NaN	-0,620202
0,377947	0,390543	0,0263176	0,0411911	1,96107	-0,9832	0,601229	0,302679	-0,00973415	1,3628
0,762232	0,574083	-0,411825	-0,326332	1,31277	-0,34477	0,257483	-0,644997	-0,610682	0,0362024
1,04448	-0,147442	0,957781	0,491898	NaN	0,682678	NaN	NaN	NaN	NaN
NaN	NaN	NaN	-2,03067	NaN	-0,891336	-1,28275	NaN	0,0430346	NaN
0,953478	2,023	1,18304	2,09629	0,882046	1,85847	1,63076	-0,0569038	NaN	0,496617
-2,93172	-0,80331	-1,96952	NaN	NaN	NaN	-0,52372	-1,59988	-1,42444	-1,4392
NaN	NaN	NaN	NaN	NaN	NaN	NaN	-1,53058	-2,07261	-1,4147
NaN	NaN	NaN	0,0646858	-2,50149	-0,0362835	-1,55276	NaN	NaN	NaN
NaN	NaN	0,361555	NaN	NaN	1,02742	NaN	NaN	NaN	NaN
0,717461	-0,82448	-0,346226	0,304907	1,53812	1,24502	1,15057	-0,345339	-0,670043	-0,263995
NaN	NaN	NaN	-1,83147	-1,22672	NaN	-1,64907	NaN	NaN	NaN
-0,720528	-0,764261	NaN	0,187992	0,571011	NaN	0,857841	-0,76177	-0,676583	-0,778495
NaN	NaN	NaN	2,68022	1,78084	-0,0745106	1,95689	NaN	NaN	NaN
0,106707	-1,23439	1,11792	1,52114	0,979496	-0,726318	2,09344	-0,575403	-0,48051	-0,77814

Polito et al. Universal ready-to-use immunotherapeutic approach for the treatment of cancer: expanded and activated polyclonal γδ memory T cells.
Phosphorylation sites

NaN	-3,38148	-0,681276	-1,47971	-0,344957	-2,51692	-0,832336	NaN	NaN	NaN
NaN	-0,68536	-1,87273	-1,09683	-0,502266	-0,498875	-0,480238	NaN	-1,1366	-1,09464
NaN	0,353714	-0,396595	0,361473	-0,289381	NaN	-1,11301	-0,116362	-0,480538	-3,43273
NaN	-2,21896	-0,357288	-2,43146	-0,489277	NaN	-1,80987	-0,864109	-1,22052	-1,25671
-2,18787	-3,10534	-0,592527	-0,944235	-2,22772	-0,902718	-0,173018	-1,73707	-1,63061	-1,81804
NaN	NaN	-0,948183	-0,674425	-2,96298	-0,584314	-0,712839	NaN	NaN	NaN
1,09854	0,651794	0,347013	0,681253	1,10205	NaN	1,0074	0,105658	-0,243087	0,23516
NaN	NaN	NaN	0,562553	NaN	0,372721	0,742498	-0,923567	NaN	-0,450164
1,79399	1,40279	1,92478	2,40193	2,51004	-1,11846	2,1358	1,77716	1,76901	1,40726
NaN	NaN	NaN	-3,11835	-2,87091	-1,96451	-1,70999	NaN	NaN	NaN
1,85807	2,30218	2,1393	2,33093	2,43464	-0,772224	2,68215	2,02053	2,1443	2,41803
NaN	NaN	NaN	NaN	NaN	-2,73756	NaN	-1,61493	-1,60437	-3,15805
-2,11984	-2,08888	-3,15957	NaN	NaN	NaN	NaN	-2,35411	-2,34477	-2,89617
1,2703	1,03328	0,704578	1,3966	1,03467	1,82956	0,869019	1,00159	0,796016	NaN
1,44901	1,35091	1,35307	1,91573	1,51304	2,10499	-2,72002	1,55494	0,979136	1,44087
-0,610828	-0,603313	-0,100523	-0,235817	0,333204	-0,590744	-0,000312805	0,0235519	0,308572	-0,349072
-0,640549	-1,38276	1,71656	1,29043	-0,0242004	1,77486	1,82731	-0,0819244	-0,386655	-0,102721
-0,726757	-1,24661	-1,52485	NaN	NaN	NaN	-0,790375	NaN	NaN	-0,986421
-2,69241	-1,94434	NaN	-2,14448	NaN	-0,389593	-2,19582	NaN	NaN	NaN
-0,354143	-0,75745	-1,02478	-0,158525	-0,240931	0,00359154	0,133602	-0,311237	NaN	-0,15195
NaN	NaN	NaN	NaN	NaN	NaN	NaN	NaN	NaN	-2,10344
-0,720823	NaN	-1,26479	NaN	NaN	NaN	NaN	-0,963039	-0,969218	-2,42133
-1,10047	-0,913433	-0,205105	-3,03911	-0,525202	NaN	-1,27267	-0,0249577	-0,479337	-0,387012
0,176565	0,056982	0,737242	1,17989	NaN	NaN	1,87871	0,559624	NaN	NaN
NaN	NaN	NaN	0,0237198	NaN	-0,669273	0,673557	-1,58461	-1,0656	-1,91657
NaN	NaN	NaN	-3,16798	NaN	-1,49122	-1,9417	NaN	NaN	NaN
NaN	NaN	-0,0247993	NaN	-0,403664	0,846851	0,987589	0,750658	0,47199	0,395707
0,0172825	-0,00400925	NaN	0,624914	-0,403664	NaN	NaN	NaN	NaN	NaN
0,443163	-0,41279	-0,279795	1,14822	0,223272	1,34053	0,33124	0,266663	0,591544	-0,0363817
NaN	NaN	NaN	-1,3369	NaN	-0,254932	-2,23832	NaN	NaN	NaN
NaN	NaN	NaN	0,2106	NaN	-0,13718	-0,316612	NaN	NaN	NaN
-0,00689125	-2,64283	-0,997225	-1,29756	NaN	-2,21541	-1,7804	-0,0368328	0,204383	NaN
0,169716	-0,785345	0,201277	0,493595	0,268751	NaN	0,527178	0,0184135	-0,18579	0,076045
NaN	NaN	NaN	-1,31616	-2,72454	-3,82023	-2,46433	NaN	NaN	NaN

NaN	NaN	NaN	0,522293	NaN	NaN	-1,21437	NaN	NaN	NaN
-1,09012	-0,939728	-1,89684	-0,723085	0,286484	-0,914482	-0,387516	-1,28319	-1,51011	-1,91718
-1,79634	-0,443264	-0,857098	-1,51143	0,0243015	-0,177267	-1,00996	NaN	NaN	-1,74438
-1,13441	-1,27155	-0,0696964	NaN	NaN	NaN	NaN	-1,30078	-0,506175	-1,91909
-0,40661	-0,627922	0,650644	0,542627	-0,286257	-0,135468	-0,560719	0,148367	1,08482	0,1925
-0,0425167	0,195656	-0,728434	-1,22495	0,143459	-0,202971	-0,164452	NaN	-0,992902	NaN
-1,26704	NaN	NaN	-2,17886	-1,8987	-1,01758	-2,11683	-1,35143	-1,43882	-1,65573
NaN	NaN	NaN	NaN	NaN	0,975163	NaN	0,530788	0,543244	0,384412
NaN	NaN	NaN	NaN	NaN	NaN	NaN	NaN	NaN	NaN
NaN	NaN	NaN	-0,904224	NaN	-1,4635	-1,87715	NaN	NaN	NaN
NaN	NaN	NaN	0,458574	NaN	0,750635	0,361891	NaN	NaN	NaN
NaN	NaN	NaN	NaN	NaN	NaN	NaN	NaN	NaN	NaN
NaN	NaN	NaN	0,791988	NaN	NaN	NaN	NaN	NaN	NaN
-0,873365	-0,658997	0,0749416	2,67272	0,263693	1,64294	1,62091	-0,42977	-0,52423	-0,772288
-1,75258	NaN	0,146929	1,31139	-0,413929	0,977901	1,40575	-1,21068	-1,85981	-2,24878
NaN	NaN	0,0366879	1,23325	-0,114395	0,983065	1,28017	-1,11574	-1,6179	-1,91683
NaN	NaN	NaN	NaN	NaN	NaN	NaN	NaN	NaN	NaN
NaN	NaN	NaN	-0,459267	NaN	-1,4034	-0,788174	0,234015	-0,118609	0,366757
1,18029	2,9229	1,82205	2,20622	3,29541	0,389925	2,65114	1,80597	1,63683	2,90336
2,86728	3,36886	2,72184	-0,584217	2,70164	-1,33805	0,179514	1,71234	2,82049	1,00045
NaN	-1,74273	-1,03152	-0,499632	-3,02268	-0,408478	1,37317	-0,796181	-1,12091	0,235745
-1,10781	-2,55269	-1,53568	NaN	NaN	NaN	NaN	NaN	NaN	NaN
2,42876	1,86464	2,02088	1,88834	1,59325	2,51419	1,59747	1,39685	1,60475	1,30982
NaN	-0,748158	-0,645092	-1,0013	0,845612	NaN	-0,713902	-0,314459	-0,685824	-0,148715
NaN	-2,64312	-2,22476	-1,70329	-1,73794	NaN	-1,70153	NaN	NaN	NaN
NaN	NaN	NaN	NaN	NaN	NaN	NaN	NaN	NaN	NaN
NaN	-2,59819	NaN	-1,68704	-2,70085	-3,05478	-0,64669	-2,8132	NaN	-1,9257
NaN	NaN	NaN	NaN	NaN	NaN	NaN	-0,482635	-0,900189	-0,672133
-0,326199	-1,15919	0,33906	-1,65317	-0,836805	NaN	-0,0200272	0,119728	0,260554	-0,537435
0,135887	0,156778	1,5172	-0,163315	NaN	0,335882	0,287344	1,14226	2,32338	1,77802
-0,116493	-0,982426	NaN	-0,501141	-0,791168	NaN	NaN	0,764042	0,79448	0,562074
-0,565994	-1,21083	0,38278	-1,66898	-0,294289	-1,19529	-1,72144	-0,140848	-0,89107	-1,23359
1,6723	0,702358	2,31766	0,974165	2,11661	1,18166	0,521444	2,48663	2,20605	1,79346
NaN	NaN	NaN	NaN	NaN	NaN	NaN	NaN	NaN	NaN

Polito et al. Universal ready-to-use immunotherapeutic approach for the treatment of cancer: expanded and activated polyclonal γδ memory T cells.
Phosphorylation sites

-0,809149	-1,552	0,136593	1,14532	0,0403996	1,40697	-0,220865	0,0784416	-0,577741	-0,567149
-1,33214	-2,11827	-1,67534	0,231213	-0,940866	-0,0957394	-1,51747	-1,12648	-1,40253	-1,55223
-0,895864	-0,357197	NaN	1,31102	-0,996508	2,04076	-1,07678	NaN	-1,81575	-1,07923
-0,451902	-1,83242	0,425482	NaN	NaN	NaN	0,612995	-0,134455	-0,0242395	-0,229497
NaN	NaN	NaN	NaN	NaN	NaN	NaN	NaN	NaN	NaN
NaN	NaN	NaN	NaN	NaN	NaN	NaN	NaN	NaN	NaN
-0,05756	-1,61947	1,21142	0,699141	0,647766	1,41585	0,267357	1,49992	1,4225	1,02862
0,0317879	0,0110798	-2,56048	0,110136	-0,12558	1,18662	-0,599199	-1,09242	-0,766316	-0,45189
NaN	NaN	NaN	NaN	NaN	NaN	NaN	NaN	-1,71434	-1,48497
NaN	NaN	NaN	NaN	-1,85948	NaN	NaN	-1,0776	-1,18176	-1,522
NaN	NaN	NaN	-0,355621	NaN	-2,10902	-1,91441	NaN	NaN	NaN
NaN	NaN	NaN	NaN	NaN	NaN	NaN	NaN	NaN	NaN
-1,40658	-0,712799	0,230839	0,817976	-0,441542	-0,0664577	0,968416	-0,848082	-0,283652	-0,293563
NaN	NaN	NaN	0,071106	-2,49	NaN	-0,543875	-0,366278	-0,0616999	-0,810094
0,662537	0,990952	0,674746	0,720047	1,62309	0,635401	0,946667	0,92103	1,21941	2,32776
-1,1533	-1,64142	-0,788565	-2,11161	-0,793631	-1,72785	NaN	-1,17375	NaN	-0,695134
NaN	NaN	NaN	-1,70472	NaN	-1,55398	-1,4043	NaN	NaN	NaN
-0,540512	-1,90676	NaN	-0,632757	NaN	0,276968	-0,282372	0,0820751	-0,0215712	NaN
NaN	NaN	NaN	NaN	NaN	NaN	NaN	NaN	NaN	NaN
-1,7137	-1,92403	-1,77977	-0,612494	-0,102961	-0,664841	-0,866886	-2,65582	-2,14687	-2,18614
-2,32441	-0,78693	-1,52643	-1,67736	-0,812632	NaN	-2,33953	-1,80074	-2,04518	-1,16586
-2,68946	-2,08114	-2,26301	-1,9556	-2,60921	NaN	-1,0842	-1,88888	-1,91744	-1,61257
NaN	NaN	NaN	NaN	NaN	NaN	NaN	NaN	NaN	NaN
NaN	NaN	NaN	NaN	NaN	-2,88654	NaN	NaN	NaN	NaN
-3,27636	NaN	-1,70082	1,55794	-1,52063	-0,0466423	-1,6128	-1,22123	-1,2575	-1,56671
3,31571	5,30277	4,99873	3,41621	4,62747	0,3836	4,89211	4,56256	4,47751	1,56682
NaN	-0,73019	NaN	NaN	NaN	-1,73099	-0,483358	-2,20613	-1,4299	-1,87414
NaN	NaN	NaN	NaN	NaN	NaN	NaN	-0,672958	-1,05602	-0,931071
NaN	NaN	NaN	NaN	NaN	NaN	NaN	NaN	NaN	NaN
-2,48008	-2,8271	-1,89842	-1,19611	-2,18391	-0,783154	-1,53867	-1,10288	-1,12645	-1,43169
-1,69761	-2,09419	-3,20911	-1,69202	-2,40533	-1,74137	-0,649527	-1,64652	-1,55539	-2,1269
1,33658	0,70561	-0,499575	0,441891	1,75959	NaN	-0,399433	0,00773621	-0,217275	-0,111522
0,0371265	-0,0586929	-0,328817	1,74932	0,178123	1,84178	0,381407	-0,405846	-0,554452	-0,848876
NaN	NaN	NaN	0,972105	-1,10681	NaN	0,871952	NaN	NaN	NaN

Polito et al. Universal ready-to-use immunotherapeutic approach for the treatment of cancer: expanded and activated polyclonal γδ memory T cells.
Phosphorylation sites

-2,09645	-1,5347	-1,90531	NaN	-1,45992	-2,82173	NaN	-1,16814	-2,06273	-2,3661
1,21484	0,727793	1,50216	0,96666	1,13154	2,34076	1,92276	1,39315	0,868911	0,8793
0,721798	0,649811	-0,342001	0,0992298	-0,884981	2,24825	0,254169	0,278412	-2,0261	-0,556411
NaN	-1,68523	NaN	0,264786	NaN	NaN	-0,281639	0,0776863	-0,244859	-0,962253
NaN	NaN	NaN	NaN	NaN	NaN	NaN	NaN	NaN	NaN
NaN	NaN	NaN	NaN	NaN	NaN	NaN	NaN	NaN	NaN
2,34886	3,0616	5,13382	3,92074	3,40893	4,7406	6,34431	3,15958	3,29696	5,28231
NaN	NaN	NaN	NaN	NaN	NaN	NaN	NaN	NaN	NaN
NaN	NaN	NaN	-3,0907	-3,5914	-2,77104	-2,03296	NaN	NaN	NaN
NaN	NaN	NaN	NaN	-2,96475	-2,15914	-1,67396	NaN	NaN	NaN
NaN	-3,55015	-1,92973	-2,66064	-2,96875	-0,734529	-1,4681	NaN	NaN	NaN
-0,0775585	-0,221233	0,352121	-2,06464	0,486696	NaN	-1,33614	-1,4075	-2,17448	-1,39303
-0,912155	-1,53272	-1,19248	-0,871771	-0,495716	-0,0498638	-0,741209	-2,61779	-3,208	-2,72208
-2,0757	-1,79027	NaN	-1,18432	-1,31997	-0,596313	NaN	NaN	NaN	NaN
-2,6546	-2,17496	-1,66139	NaN	NaN	NaN	NaN	-1,41586	-1,30419	-1,69272
NaN	NaN	NaN	NaN	NaN	NaN	NaN	NaN	NaN	NaN
NaN	-1,67749	NaN	0,607285	-1,28241	0,294466	NaN	NaN	NaN	NaN
NaN	NaN	NaN	0,0642471	NaN	0,259054	-0,313971	-1,0724	-1,28658	-0,608363
NaN	NaN	NaN	0,132711	-1,57643	-0,307915	0,361855	NaN	NaN	NaN
NaN	NaN	NaN	-1,03244	NaN	NaN	-0,801666	NaN	NaN	NaN
NaN	NaN	NaN	NaN	NaN	NaN	NaN	NaN	NaN	NaN
-0,537724	-0,469557	-1,33156	0,0475178	-0,844749	0,220491	-0,157928	-0,357655	-0,910792	-1,32349
1,01165	0,528156	1,14683	NaN	1,51443	1,57051	-0,195129	1,37989	0,868012	1,08682
-1,7159	NaN	NaN	-2,23894	-0,637926	-2,13758	NaN	-1,52533	-2,35502	-1,67958
NaN	NaN	NaN	-1,35137	-3,20898	NaN	-1,11108	NaN	NaN	NaN
NaN	NaN	NaN	-0,703495	NaN	NaN	-1,48195	NaN	NaN	NaN
NaN	NaN	NaN	-0,875916	-1,62963	NaN	-2,18888	-1,49246	-0,526858	NaN
NaN	NaN	-2,10002	NaN	NaN	NaN	NaN	-1,8133	-1,75108	-2,16849
-0,297632	NaN	-1,56574	NaN	NaN	NaN	NaN	-1,03834	-1,27775	-0,733768
NaN	NaN	NaN	NaN	-2,13614	0,654558	-0,682323	-1,40982	-0,881669	-1,00767
-0,94096	-0,729982	-2,81348	NaN	-0,140171	-1,20947	-2,06607	-0,874727	-2,02493	-1,03591
-0,737547	NaN	NaN	0,676891	-1,27379	-0,235474	NaN	NaN	-0,309649	-1,24231
0,908882	0,791821	0,906799	1,05469	1,75387	1,42288	1,28952	0,565704	0,554879	0,693271
NaN	NaN	NaN	NaN	NaN	NaN	NaN	NaN	NaN	NaN

Polito et al. Universal ready-to-use immunotherapeutic approach for the treatment of cancer: expanded and activated polyclonal γδ memory T cells.
Phosphorylation sites

5,37676	5,2276	4,88282	5,64802	6,5129	5,82314	5,63844	5,32601	5,96891	5,74673
-1,87758	-1,97702	-1,80881	0,210695	-1,24345	-0,210716	0,501551	-1,59144	-1,81402	-1,4544
-0,272125	NaN	NaN	1,15583	NaN	1,98573	NaN	NaN	NaN	NaN
0,0203667	-1,21697	-0,355877	-0,259563	-0,640097	1,73851	-0,19038	-0,560282	-0,532393	-0,748805
NaN	NaN	NaN	NaN	NaN	NaN	NaN	-0,946409	-1,82168	-1,84351
NaN	NaN	NaN	-1,31726	-3,60666	-2,36789	-2,1338	NaN	NaN	NaN
-1,16462	-0,537573	NaN	0,457638	NaN	-1,02149	-0,762775	NaN	-0,481397	-0,915967
-0,220943	-0,298983	-0,872149	-1,96626	-1,21091	NaN	-1,58713	NaN	NaN	NaN
-1,14968	-2,8486	-2,31596	-1,05682	-0,996962	-0,988636	-2,42744	NaN	-2,96815	-1,69435
-1,58632	NaN	0,496374	0,527912	-0,786257	1,6429	0,690966	-1,23996	-1,33853	-1,58009
NaN	-1,87224	NaN	-0,586727	-1,81879	0,0385475	0,241749	-0,27282	-0,76224	-0,854342
-2,78623	NaN	2,81436	2,88891	-3,06927	3,98937	2,57033	-1,37913	-0,664376	-1,20162
NaN	NaN	NaN	-3,73398	NaN	-0,981874	-2,60273	NaN	NaN	NaN
NaN	NaN	NaN	-3,73398	NaN	-0,981874	-2,60273	NaN	NaN	NaN
NaN	NaN	NaN	-0,101915	-0,706888	1,31026	-0,057188	-1,87208	-1,95615	-2,10403
NaN	NaN	0,909849	0,737904	NaN	1,68309	1,03776	NaN	NaN	NaN
NaN	NaN	-0,417902	0,231808	-1,6613	0,908417	1,02552	-2,12828	-1,36994	NaN
-2,88558	-1,11753	-0,909447	-0,256332	-0,779018	-0,770098	0,19845	-1,17207	-1,38406	-0,552602
NaN	NaN	-0,54178	-1,55916	-1,47779	-1,78708	-1,95006	-1,95108	-1,66454	-1,26292
-1,07161	-1,00855	-1,1353	NaN	-0,725901	-2,85282	-0,955463	-1,06215	-1,29551	-0,278314
3,63589	3,56508	3,88948	3,67623	4,11194	4,87959	3,29936	3,77743	3,43964	3,03985
-1,49655	-1,88992	0,268297	-0,084444	-0,156969	-1,42068	-0,716801	-0,659777	-0,910638	-1,04455
4,34373	2,12345	4,84423	3,52458	4,07809	4,68723	4,65308	3,96263	4,09364	3,8709
NaN	NaN	NaN	NaN	NaN	NaN	NaN	NaN	NaN	NaN
NaN	NaN	-1,47156	NaN	NaN	NaN	NaN	NaN	NaN	NaN
NaN	NaN	NaN	-2,10306	NaN	NaN	-0,0815029	-2,7114	-1,35015	-3,05344
-0,656082	0,988913	0,385492	0,536177	0,480883	0,358023	0,907293	0,52825	0,379766	1,25799
NaN	-0,73019	NaN	NaN	NaN	-1,73099	-0,483358	-2,20613	-1,4299	-1,87414
NaN	NaN	NaN	NaN	NaN	NaN	NaN	NaN	NaN	NaN
-2,64159	-2,07733	-0,835451	0,470165	-1,77143	1,19271	0,179533	-0,651445	-1,40927	-0,918523
2,42586	2,88597	2,12309	2,64248	4,19338	-0,953123	3,28819	2,13176	1,60138	2,30216
-2,63964	NaN	-1,10225	1,78752	NaN	1,2883	-0,0245667	-0,898521	0,614097	NaN
NaN	NaN	-1,32879	-2,85921	NaN	-2,3805	-1,39664	NaN	NaN	NaN
0,992031	0,354322	0,535479	-0,347015	2,4836	-0,612436	0,984995	1,2183	0,994967	1,03019

Polito et al. Universal ready-to-use immunotherapeutic approach for the treatment of cancer: expanded and activated polyclonal γδ memory T cells.
Phosphorylation sites

3,26386	2,41207	3,64087	-1,29458	-0,27067	3,12652	-0,608471	-0,178713	3,43621	-0,250993
NaN	NaN	NaN	NaN	NaN	NaN	-1,92769	NaN	NaN	NaN
NaN	NaN	NaN	-2,45596	-1,07215	-1,16365	NaN	NaN	NaN	NaN
-1,91287	-0,888994	-0,736816	1,40544	-0,56259	0,475584	-0,291698	NaN	NaN	NaN
0,827957	1,06683	1,57611	3,07024	2,00381	3,07043	2,41525	1,93949	1,93953	1,78243
NaN	NaN	NaN	NaN	NaN	NaN	NaN	NaN	NaN	NaN
-0,477064	-0,623331	-0,43173	-0,148365	0,863708	-0,149221	0,276407	-0,368273	-0,31635	-0,68484
NaN	NaN	NaN	-2,17271	NaN	-1,22936	-2,02876	NaN	NaN	NaN
NaN	NaN	NaN	NaN	NaN	NaN	NaN	NaN	NaN	NaN
2,45847	0,559677	2,35381	NaN	3,20898	NaN	NaN	3,97502	3,49061	3,34403
NaN	NaN	NaN	NaN	NaN	NaN	NaN	NaN	NaN	NaN
-2,00815	-1,74043	-1,14408	-2,37182	-0,167149	NaN	-1,30892	-2,43098	NaN	-0,993348
NaN	NaN	NaN	-1,19779	NaN	-0,815414	NaN	NaN	NaN	NaN
NaN	NaN	NaN	-1,218	NaN	-0,7005	-0,837278	NaN	NaN	NaN
NaN	NaN	NaN	-1,53217	-3,23167	NaN	-1,85072	-2,1925	-2,23363	-2,19936
NaN	NaN	NaN	NaN	NaN	NaN	NaN	NaN	NaN	NaN
NaN	NaN	-0,565142	1,28101	-2,30626	0,864599	1,23976	-2,35679	-2,75634	-2,19022
NaN	NaN	NaN	-0,606827	-1,39545	-1,715	NaN	NaN	NaN	NaN
NaN	-2,79918	NaN	-2,35753	NaN	NaN	NaN	-0,721731	-1,02468	-0,504506
-0,29574	NaN	-1,57885	NaN	-2,09559	1,81594	-0,116905	NaN	NaN	NaN
-0,144001	NaN	-1,04004	1,72989	-0,581186	-1,01358	0,445055	NaN	NaN	NaN
NaN	NaN	NaN	-1,3516	-2,11012	-0,543659	-1,53374	-2,3508	-2,30693	-2,32217
NaN	-1,75608	-1,62786	-0,0648727	NaN	-0,586815	-0,538218	-1,66633	-1,78178	-1,55513
NaN	NaN	NaN	NaN	NaN	NaN	NaN	NaN	NaN	NaN
NaN	NaN	NaN	NaN	NaN	NaN	NaN	NaN	NaN	NaN
0,0225449	-0,505131	-0,631533	0,103085	0,261772	0,361479	-0,30862	0,187813	0,166327	-0,33644
0,0225449	-0,505131	-0,631533	0,103085	NaN	NaN	-0,30862	0,187813	0,166327	NaN
NaN	NaN	NaN	-0,725727	NaN	-0,776098	-1,33552	NaN	NaN	NaN
NaN	NaN	NaN	NaN	NaN	NaN	NaN	NaN	NaN	NaN
1,79012	1,92995	2,58239	2,4124	3,18729	-1,95305	2,69825	2,81274	2,56361	2,69203
-3,85774	-3,29158	-0,601505	-1,26199	-2,12431	NaN	-0,932718	-1,97804	NaN	-2,4544
NaN	-2,18393	NaN	NaN	NaN	NaN	NaN	NaN	NaN	NaN
-2,24238	-1,26113	-2,23032	-1,28125	-1,58741	-0,658754	-0,810537	NaN	NaN	NaN
2,39423	4,27484	3,54824	4,17197	4,54924	4,31195	5,23493	3,54455	3,00366	3,96165

Polito et al. Universal ready-to-use immunotherapeutic approach for the treatment of cancer: expanded and activated polyclonal γδ memory T cells.
Phosphorylation sites

-0,893625	2,40932	0,689148	1,46045	2,99709	-1,06786	3,10468	-0,104084	-0,561034	2,23331
1,59985	0,516659	0,851839	0,361473	1,94838	0,204149	-0,117779	0,22574	-0,291213	0,40643
-2,41984	NaN	NaN	NaN	-1,03049	-1,7517	-1,54779	NaN	NaN	NaN
NaN	NaN	NaN	NaN	NaN	NaN	NaN	NaN	NaN	NaN
-0,682705	-1,00139	NaN	0,715578	-0,927015	NaN	NaN	-0,804216	-1,07893	0,131293
NaN	NaN	NaN	NaN	NaN	NaN	NaN	NaN	NaN	NaN
NaN	NaN	NaN	NaN	NaN	NaN	NaN	NaN	NaN	NaN
NaN	-3,04997	NaN	-0,964001	-2,67394	-3,43441	-0,140385	NaN	NaN	NaN
NaN	NaN	NaN	0,560686	-1,06654	0,0809708	0,222553	NaN	NaN	NaN
NaN	NaN	NaN	NaN	NaN	NaN	NaN	NaN	NaN	NaN
-2,81697	-1,58635	-2,30189	-0,339622	-2,11221	-1,19466	-2,25635	NaN	NaN	NaN
-0,46586	-0,682882	-0,139637	1,80288	-0,185526	NaN	0,860798	-0,775923	0,31107	0,0139666
-2,81697	-1,58635	-2,30189	-0,339622	-2,11221	-1,19466	-2,25635	NaN	NaN	NaN
-2,47896	NaN	-1,93443	-1,19396	-1,17509	-1,1721	-0,435577	-2,46983	-1,81337	-2,45214
-2,14914	-1,45977	-0,596689	NaN	NaN	NaN	NaN	NaN	NaN	NaN
-2,49384	NaN	-0,95236	NaN	NaN	NaN	NaN	0,161829	-0,114076	-1,71342
NaN	-0,0462532	NaN	-1,74544	0,134447	NaN	-0,644768	NaN	NaN	NaN
1,66176	2,69764	2,07576	2,32356	2,39643	1,564	2,65537	1,37374	1,72843	1,66301
2,32054	3,21328	-1,68158	0,386158	3,49035	1,81754	1,83083	3,17984	NaN	3,43746
NaN	-2,28493	-2,15032	-1,15648	-1,88099	-0,307365	-0,600725	-2,18874	-1,97909	-1,69688
2,99727	3,09944	2,37248	2,71893	1,30853	3,60846	2,61319	1,26896	3,27456	1,02761
NaN	NaN	-0,81558	-0,399733	NaN	-0,435011	0,545975	NaN	NaN	NaN
2,11027	2,13268	2,39981	3,20731	2,34464	2,18717	2,54444	2,46786	2,33984	2,31633
NaN	-1,98958	-1,1468	NaN	-1,55991	NaN	-1,44595	-2,66012	NaN	-2,7191
NaN	NaN	NaN	-1,35795	NaN	-0,740044	-1,78571	NaN	NaN	NaN
-0,524916	NaN	-0,204298	-1,89411	0,403053	NaN	-0,631413	NaN	NaN	NaN
NaN	NaN	NaN	-0,363073	-2,86506	-1,11412	NaN	NaN	NaN	NaN
0,588871	-1,44931	-2,07453	NaN	0,789211	NaN	2,62635	-1,22735	NaN	-2,55628
6,19525	5,69282	5,3588	4,76902	6,08676	2,40952	5,40417	5,63563	5,46618	5,51781
NaN	NaN	-1,18899	NaN	-0,958771	NaN	-1,9906	-0,852623	-0,783582	-1,53285
2,69271	2,57542	3,59	2,44253	3,89349	NaN	3,04392	3,78944	3,73368	3,10766
5,17234	3,26583	4,30019	1,30657	5,01546	NaN	1,16908	4,38211	4,09772	4,19109
1,16066	1,42825	0,238188	NaN	1,54681	NaN	NaN	1,29125	0,760985	2,07805
-2,20424	-0,0759125	NaN	-1,11631	NaN	-0,387037	-1,35528	-0,673594	0,709992	-0,184779

Polito et al. Universal ready-to-use immunotherapeutic approach for the treatment of cancer: expanded and activated polyclonal γδ memory T cells.
Phosphorylation sites

-1,43774	NaN	-0,405804	NaN	-1,9788	NaN	NaN	-0,304228	0,433753	-0,338441
NaN	NaN	NaN	NaN	NaN	NaN	NaN	NaN	NaN	NaN
-1,521	-1,53805	-1,84102	NaN	NaN	NaN	NaN	NaN	NaN	NaN
2,20601	2,85	1,99335	3,50316	3,26471	2,47636	3,08874	2,26938	2,42472	2,90756
NaN	0,328627	NaN	1,39324	0,449703	1,53427	0,780853	NaN	0,107882	NaN
2,40767	1,83254	2,28215	2,52769	2,31111	2,79867	2,62646	1,75249	1,71681	1,56225
0,508825	-0,844145	0,52631	1,29463	0,230682	-1,36018	1,27805	0,0675354	-0,254981	0,236836
-0,513592	0,752785	0,233383	-0,389679	-1,72369	NaN	NaN	0,637848	0,991155	0,0710726
NaN	-1,74116	-1,36628	-0,698774	NaN	-0,0807076	-1,11277	-1,61623	NaN	-1,73709
0,272079	NaN	0,496706	1,9419	NaN	1,48927	2,18268	NaN	NaN	NaN
1,30351	1,11926	0,870972	1,47923	1,62004	NaN	0,8297	1,5929	1,15834	0,550368
NaN	-1,07996	0,202032	0,323093	NaN	-1,78365	-0,0152302	-0,588133	NaN	NaN
NaN	NaN	NaN	NaN	-1,01639	3,04882	-0,537796	NaN	NaN	NaN
2,19913	2,13424	1,70925	NaN	2,23319	0,249914	NaN	2,52449	1,98303	2,6243
NaN	NaN	NaN	0,689713	NaN	-0,299986	0,711718	-0,675545	NaN	NaN
3,47817	3,09093	3,3462	4,23941	3,61715	4,59662	3,8851	3,35775	3,26932	3,2036
NaN	NaN	NaN	-0,262537	NaN	-0,0606976	-1,63811	NaN	NaN	NaN
NaN	NaN	NaN	-0,580996	NaN	-2,20925	-1,75785	NaN	NaN	NaN
-1,1947	-0,845207	NaN	-1,14079	0,234482	-1,62473	-0,597746	NaN	NaN	NaN
NaN	-1,20198	-1,7456	-1,03918	-2,17002	NaN	-0,345226	-1,53368	-0,936625	-0,576907
-0,847189	-2,71454	0,289593	NaN	-0,416508	-0,801041	NaN	-0,762829	-0,781924	-1,03699
-2,17511	0,122271	NaN	1,46145	-0,528275	1,32755	NaN	NaN	NaN	NaN
NaN	NaN	NaN	-2,13468	-3,6863	-2,58546	-2,56534	-2,40195	-2,55937	NaN
NaN	NaN	NaN	-2,78651	NaN	-1,51428	-1,96455	NaN	NaN	NaN
NaN	-1,14506	-2,14516	-0,165747	NaN	NaN	-0,669008	NaN	NaN	NaN
-1,58773	-1,25249	-1,18162	0,368183	NaN	0,0685692	-0,0593052	-2,04378	-1,59598	-1,85003
-1,40936	-0,708694	-0,462038	NaN	NaN	NaN	NaN	NaN	NaN	NaN
NaN	NaN	NaN	-1,93549	-2,02689	-2,41549	NaN	NaN	NaN	NaN
-2,61605	-1,21534	-2,4217	-1,90446	-1,9164	-2,62498	-1,31759	NaN	NaN	NaN
2,71583	1,99393	1,56046	1,75336	1,99677	2,4553	1,74836	1,63358	1,99677	1,51002
-1,01143	-0,844313	-0,173349	-0,483145	-0,543692	-0,294067	-0,448999	-0,694513	-0,893735	-0,707597
-1,37492	-1,47165	NaN	-1,18863	-1,31035	NaN	-0,767673	-2,14683	-1,72943	-1,79405
0,4347	0,0717163	0,229229	NaN	1,07247	NaN	0,031105	0,318827	-0,0166044	1,26497
-0,508566	-0,256306	-0,190372	0,28632	0,146133	-1,80862	-0,160025	-0,252472	-0,474597	-0,0468683

Polito et al. Universal ready-to-use immunotherapeutic approach for the treatment of cancer: expanded and activated polyclonal γδ memory T cells.
Phosphorylation sites

NaN	-1,88444	-1,6341	-1,47449	-1,84961	NaN	NaN	NaN	NaN	NaN
NaN	-0,152403	-2,55898	-1,14079	NaN	0,0989857	-1,09638	NaN	NaN	NaN
NaN	NaN	NaN	-1,64314	-1,12636	NaN	-1,9938	-1,90402	NaN	-1,52265
NaN	NaN	NaN	-0,877434	NaN	-0,555128	-0,975449	NaN	NaN	NaN
NaN	NaN	NaN	NaN	NaN	NaN	NaN	NaN	NaN	NaN
0,775984	1,33889	1,52134	1,56628	1,66503	1,93665	1,72611	1,58685	1,18843	1,62214
3,1644	0,691017	2,20734	3,45175	0,483589	4,4484	2,39117	2,53167	2,50546	2,17046
NaN	-0,667706	0,575399	0,626463	NaN	-0,413639	-0,44446	0,753458	0,464667	0,906489
-1,83691	-2,04881	-1,95671	NaN	-1,71564	NaN	NaN	-0,701715	-1,1658	-1,19184
4,89638	4,58625	5,07693	4,79746	4,91046	1,59007	5,22164	6,0558	5,79062	5,56108
NaN	-0,891102	NaN	NaN	NaN	NaN	NaN	0,431955	-2,38149	-0,0128965
NaN	NaN	NaN	NaN	NaN	NaN	NaN	NaN	NaN	NaN
NaN	NaN	NaN	NaN	NaN	NaN	NaN	NaN	NaN	NaN
NaN	NaN	0,153194	NaN	NaN	NaN	NaN	1,2057	1,7634	1,58812
2,09598	1,43507	1,31447	2,04229	3,36848	0,275187	2,25258	-0,174343	-0,697949	0,51028
NaN	1,00786	NaN	0,796124	1,54125	2,13315	0,350964	NaN	NaN	NaN
0,0542316	NaN	NaN	NaN	0,290209	NaN	NaN	NaN	NaN	NaN
NaN	NaN	NaN	-2,48644	-1,41442	-1,94699	-1,56243	NaN	NaN	NaN
-2,67086	NaN	-0,865839	1,98605	-2,36995	1,16706	-0,076828	NaN	NaN	NaN
0,474199	0,452648	0,408079	0,164501	0,153463	NaN	-1,10554	0,295095	0,52768	1,09092
4,61755	1,75555	3,70943	2,623	4,00939	1,92933	2,45773	4,32041	3,56186	3,83417
NaN	NaN	0,534821	-0,679892	-0,487204	-0,0393581	-0,739567	-0,597576	-1,02268	-1,22246
-1,75484	-2,58233	-0,55057	NaN	NaN	NaN	NaN	-0,21207	0,356198	-0,273812
3,14883	1,19402	2,54819	2,86753	3,39661	3,63465	2,56029	2,11574	1,87104	2,15498
NaN	NaN	NaN	-1,5318	NaN	-0,172939	-1,72202	NaN	NaN	NaN
NaN	NaN	0,395807	-0,697939	0,343145	NaN	-0,564812	NaN	0,0523806	NaN
0,229727	-0,769297	NaN	-0,960419	0,221615	NaN	0,0649128	0,374317	0,467681	0,0101881
NaN	-0,722279	NaN	-0,474817	-1,83726	0,285547	-1,23547	NaN	NaN	NaN
-1,27242	-1,53119	-1,87607	0,303522	NaN	1,38851	-1,46008	-1,91536	-2,78023	-2,52252
-1,94525	-1,09552	-2,0049	-1,14075	-2,55554	-1,23173	-1,4623	NaN	NaN	NaN
NaN	NaN	NaN	NaN	NaN	NaN	NaN	NaN	NaN	NaN
-0,209335	-0,20229	-0,767952	-0,0506382	-0,499973	0,0590515	-0,372335	-0,737656	NaN	-1,18593
-0,859652	-1,33899	-1,13828	-0,667927	-0,813782	-0,935184	-0,807247	-2,09337	-1,36043	-1,66233
-0,491394	-0,415489	-0,579134	-0,183046	NaN	NaN	0,473692	NaN	-0,172347	NaN

Polito et al. Universal ready-to-use immunotherapeutic approach for the treatment of cancer: expanded and activated polyclonal γδ memory T cells.
Phosphorylation sites

NaN	NaN	NaN	NaN	NaN	NaN	NaN	NaN	NaN	NaN
1,94307	2,0039	1,66208	1,22509	1,35117	1,85873	1,49693	1,58552	1,35387	1,34222
-0,878117	NaN	-2,54184	0,757608	-0,921997	NaN	-2,11026	NaN	-2,50842	-2,61145
-0,705053	-0,341242	-1,71633	0,0438137	-0,58972	0,308662	-1,30621	NaN	NaN	NaN
NaN	-1,68699	NaN	NaN	NaN	NaN	NaN	-0,916348	-1,18852	-1,26419
NaN	NaN	NaN	NaN	NaN	NaN	NaN	-2,55802	-2,01404	-3,39804
NaN	NaN	NaN	-2,02191	-2,40572	-1,9633	-2,30898	NaN	NaN	NaN
NaN	NaN	NaN	-1,04089	NaN	-0,719654	-2,19613	NaN	NaN	NaN
0,312719	NaN	NaN	-0,431236	1,91416	0,158388	0,392788	NaN	NaN	NaN
-1,39926	-3,22025	-0,311193	NaN	-1,18159	NaN	-2,29608	NaN	NaN	NaN
NaN	-1,3828	-0,664112	0,69449	0,911684	NaN	1,79502	0,97151	0,681119	0,332339
1,65568	2,23322	1,51044	1,0203	3,38634	-0,438435	1,48517	1,60065	1,26987	2,00285
NaN	NaN	0,90465	0,405855	-2,80859	-0,892136	1,07579	NaN	NaN	NaN
0,593508	-1,12132	-0,00218391	0,599783	-1,03555	-1,66262	1,63112	-0,542736	-0,45341	-0,561448
0,692402	-0,089407	0,916939	0,123697	0,178024	0,105795	1,86694	0,83877	0,234851	0,493947
NaN	NaN	NaN	NaN	0,506306	-0,0889683	-0,820724	NaN	NaN	NaN
NaN	NaN	NaN	NaN	NaN	NaN	NaN	NaN	NaN	NaN
-0,310032	NaN	2,1822	3,40787	NaN	2,9265	0,90204	NaN	NaN	0,130204
NaN	NaN	1,38734	NaN	NaN	NaN	0,723406	NaN	-0,360061	-0,726344
NaN	NaN	NaN	NaN	NaN	NaN	NaN	NaN	NaN	NaN
0,593937	0,864122	-0,346819	1,88206	-0,644979	0,558395	1,26379	-0,569136	-1,2178	-0,6288
-2,11335	-2,2378	-0,332228	-1,50489	NaN	-4,07771	-1,67068	-2,73759	-1,76537	NaN
NaN	NaN	NaN	NaN	NaN	NaN	NaN	NaN	NaN	NaN
NaN	NaN	NaN	-2,1179	NaN	-1,42219	-1,33897	NaN	NaN	NaN
-0,586626	-1,5419	-1,60511	NaN	NaN	NaN	NaN	NaN	NaN	NaN
0,360964	-0,483973	-0,144619	-0,673281	-0,586983	NaN	1,10007	-0,385828	0,339665	0,0193205
1,09833	1,58009	1,13192	1,22878	1,16616	0,895205	1,28632	1,05611	0,655047	0,59936
0,81723	0,181578	0,0826092	1,04554	0,177597	1,34142	1,24222	0,519541	0,484284	0,347714
NaN	NaN	NaN	NaN	NaN	NaN	NaN	NaN	NaN	NaN
NaN	NaN	NaN	NaN	NaN	NaN	NaN	NaN	NaN	NaN
NaN	NaN	-2,29324	-0,417128	-2,20565	NaN	-2,18979	NaN	NaN	NaN
NaN	NaN	NaN	-1,15869	NaN	-0,848995	-1,3558	NaN	NaN	NaN
NaN	NaN	NaN	NaN	NaN	NaN	NaN	-1,43826	0,663487	-0,386682
NaN	-1,61813	-1,44812	-2,58248	-1,20211	NaN	-1,56435	-1,21543	-1,41293	-1,54605

Polito et al. Universal ready-to-use immunotherapeutic approach for the treatment of cancer: expanded and activated polyclonal γδ memory T cells.
Phosphorylation sites

-0,990612	-1,55534	-1,38072	2,53519	0,056921	-0,0920315	-0,41678	NaN	NaN	NaN
NaN	NaN	NaN	NaN	NaN	NaN	NaN	NaN	NaN	NaN
NaN	NaN	NaN	0,929888	NaN	2,94894	NaN	NaN	NaN	NaN
4,49135	3,96866	3,9781	4,26564	4,33337	3,11205	3,80871	NaN	4,00305	4,1476
NaN	NaN	-0,491684	NaN	NaN	NaN	NaN	0,950884	0,391007	0,511004
-0,969261	0,348217	0,0204163	0,488756	0,337149	0,842247	1,14159	NaN	-0,167571	-0,374372
0,992256	0,540739	1,39397	0,921856	2,13866	1,15339	1,02046	1,19822	1,01571	1,70578
NaN	NaN	NaN	NaN	NaN	NaN	NaN	-1,93011	-2,0778	-1,43622
NaN	NaN	NaN	NaN	NaN	NaN	NaN	NaN	NaN	NaN
NaN	NaN	NaN	-1,44581	NaN	-2,83151	-2,25913	-3,32843	NaN	-3,38178
NaN	NaN	-1,80992	-0,819256	NaN	-2,21341	-1,3304	NaN	NaN	NaN
NaN	NaN	NaN	NaN	-2,22799	-1,50558	-1,44073	NaN	NaN	NaN
NaN	NaN	NaN	-1,23556	-1,38605	NaN	-1,63936	-0,984396	NaN	-2,01665
NaN	NaN	NaN	-1,53403	0,515783	NaN	-1,47386	NaN	NaN	NaN
-0,263445	-1,51267	-2,64175	-0,0991955	-0,901297	-0,21516	-0,369822	-0,672918	-0,928367	-0,944566
0,123238	-0,974232	-0,354441	1,45268	0,260445	1,52372	0,965328	0,311087	0,00542545	-0,00180149
-2,43992	-3,03241	0,179317	-1,58133	-0,812433	-0,567825	-1,60742	-1,87555	-1,37866	NaN
0,622488	0,946619	1,00396	1,39541	1,46708	1,29393	0,991978	1,07802	1,45832	1,34881
NaN	NaN	NaN	NaN	NaN	NaN	NaN	NaN	NaN	NaN
NaN	NaN	NaN	NaN	NaN	NaN	NaN	NaN	NaN	NaN
3,0411	3,40562	3,12785	3,06063	1,48883	3,32838	3,25046	2,09633	3,03355	2,29423
NaN	NaN	NaN	-0,636467	-1,97858	-0,178894	-0,911924	-2,65828	-2,41369	-2,57996
-1,10603	NaN	0,343811	1,42337	-0,147364	-1,69217	-1,5242	-0,119474	0,27135	-0,0215368
NaN	NaN	0,272882	0,958925	NaN	0,402401	0,383104	-0,606548	0,36667	-0,786983
-0,102623	0,0377922	-0,241377	0,671938	1,47733	0,704977	0,931868	-0,832586	-1,23425	-0,30602
-1,27291	-1,80876	-1,67805	NaN	NaN	NaN	NaN	-1,74015	-2,54004	-2,52582
NaN	NaN	NaN	0,474539	NaN	0,873522	-1,37897	NaN	NaN	NaN
NaN	NaN	NaN	-1,38569	NaN	NaN	-2,19736	NaN	NaN	NaN
NaN	NaN	NaN	2,35506	-2,54548	2,36045	0,894247	NaN	NaN	NaN
NaN	NaN	NaN	-3,04027	NaN	-2,34725	NaN	NaN	NaN	NaN
NaN	NaN	NaN	NaN	NaN	NaN	NaN	NaN	NaN	NaN
NaN	NaN	NaN	NaN	NaN	NaN	NaN	NaN	-2,82678	-2,19158
1,34416	3,00311	1,7807	2,46704	2,15593	0,407469	2,48633	1,06329	1,27437	2,1555
-2,62264	-1,49057	-2,2397	NaN	-1,45687	NaN	-2,16423	-2,72484	-2,79297	-1,29286

Polito et al. Universal ready-to-use immunotherapeutic approach for the treatment of cancer: expanded and activated polyclonal γδ memory T cells.
Phosphorylation sites

0,534401	0,422396	1,56748	0,808561	0,635372	0,843918	2,37743	0,621874	0,582671	0,42653
0,695438	NaN	6,11283	5,68872	-0,328726	5,97797	5,70412	2,68075	-1,39712	2,35721
NaN	NaN	NaN	-1,74218	NaN	NaN	NaN	NaN	NaN	NaN
NaN	NaN	NaN	-1,59098	NaN	-0,84643	-1,90693	NaN	NaN	NaN
NaN	NaN	NaN	-0,762209	-2,71605	0,206747	-2,11897	NaN	NaN	NaN
NaN	-0,0643024	-1,63699	NaN	-0,578424	NaN	NaN	-1,57025	-2,40487	-1,62497
NaN	NaN	-0,17354	-0,318701	NaN	0,47567	0,094244	NaN	NaN	NaN
-1,46164	NaN	0,810982	1,24687	-0,576685	2,79696	1,55482	0,983166	1,28038	0,788419
3,00051	1,30145	3,84547	3,03598	2,71633	4,59211	2,63623	2,73462	2,55749	2,83644
NaN	NaN	NaN	-0,606966	NaN	-1,15391	-0,888567	-4,16678	NaN	-1,8344
NaN	NaN	NaN	-0,881729	-3,19637	-1,47598	-1,69804	NaN	NaN	NaN
NaN	NaN	NaN	-0,490616	-1,44963	-0,721153	-0,995581	-0,974609	-1,29596	-1,19252
NaN	NaN	NaN	-1,56758	-2,05379	-0,401447	-0,68383	NaN	NaN	NaN
2,7221	3,21838	2,69086	3,08121	2,24109	2,74923	3,33809	2,19817	2,31261	1,88689
NaN	NaN	NaN	-1,01986	-2,05899	-2,04486	0,836851	NaN	NaN	NaN
-0,754128	0,114809	-0,936087	-1,16449	-0,99407	-0,966408	-0,176903	-1,14898	-1,22488	-1,53271
0,986523	3,07438	2,20081	1,83043	4,78901	0,524141	2,82097	1,9503	1,61336	2,89015
-0,93297	2,08952	-1,55132	-1,95952	2,03716	1,21951	-1,37138	-1,20993	-1,35292	-1,40647
NaN	NaN	NaN	-1,03771	NaN	0,0547905	-2,05675	NaN	NaN	NaN
NaN	NaN	NaN	NaN	NaN	NaN	NaN	NaN	NaN	NaN
-1,55851	-0,510202	-0,769144	1,01485	NaN	-0,896324	-0,418333	-0,988646	-1,17165	NaN
NaN	-1,75935	NaN	-1,06073	NaN	-1,41629	-0,118069	0,125341	0,0568838	0,0246992
NaN	-1,53101	1,5702	1,07908	NaN	2,66264	1,80254	NaN	-0,183763	-0,796119
NaN	NaN	NaN	NaN	-0,985657	-0,326414	0,383345	-0,162374	-0,243159	0,485023
-1,19082	-0,0863285	0,664619	-2,04289	0,405458	-1,27497	-1,10997	1,24759	0,741736	2,58403
NaN	NaN	-0,760824	1,42378	NaN	0,906755	1,3393	1,47079	1,07522	1,29932
0,978048	1,04782	0,623798	1,56069	1,22049	0,715889	1,52508	1,34231	0,745044	0,795047
2,97482	3,55239	3,85436	3,65476	3,38694	4,65448	4,15878	2,38811	2,9504	2,291
-0,549599	-2,17835	-1,33055	-2,12254	-1,83391	NaN	-1,79444	-3,86648	-2,94877	-0,976678
NaN	NaN	-2,09205	NaN	-1,5082	NaN	-1,34049	NaN	NaN	-2,03037
NaN	0,0819378	NaN	3,67652	1,89754	-2,4454	3,28003	NaN	-2,02193	-1,24279
NaN	NaN	-0,875723	NaN	NaN	NaN	NaN	-0,334826	-0,678388	-0,207231
0,935736	0,0211086	0,728952	1,73354	0,698072	-1,71792	2,01023	0,686859	1,18528	0,265737
0,0154514	-1,23193	0,175325	1,16717	0,497774	-2,5846	2,54506	0,530426	0,642095	0,351083

Polito et al. Universal ready-to-use immunotherapeutic approach for the treatment of cancer: expanded and activated polyclonal γδ memory T cells.
Phosphorylation sites

2,10576	2,25704	2,24722	4,17909	1,87144	0,375126	2,40077	2,30262	2,39855	1,04751
-0,0457382	-0,667286	0,733805	1,1684	1,59767	-2,5555	1,05711	1,25524	0,944709	0,555406
NaN	NaN	NaN	-0,0175323	NaN	-0,469208	-0,824518	NaN	NaN	NaN
-2,63008	NaN	-2,15485	-1,06084	-2,1616	NaN	-0,852409	-1,86152	-2,0181	-1,67202
0,179012	-0,564207	-1,23425	1,08559	-0,393692	NaN	0,173838	-0,828194	-0,966771	-0,864125
-0,877583	-2,4935	-1,23425	0,27368	NaN	NaN	0,173838	-0,828194	-0,966771	-0,864125
1,18088	1,21243	0,30575	3,08966	0,930944	-0,2799	2,41055	-0,228546	0,0766993	0,745946
4,59056	4,48247	4,19591	6,462	3,55904	2,28889	5,31907	4,09151	4,15514	3,84229
0,0428162	0,449913	1,81472	3,20205	2,13323	3,93604	2,68975	1,54618	0,779263	1,25038
2,88158	3,47675	1,72895	5,16625	1,42546	3,71204	3,25006	1,03106	1,04214	0,73413
0,473976	0,206497	0,796776	0,96929	0,622097	1,31609	1,00484	0,692719	NaN	1,56201
NaN	-2,87133	-2,73774	NaN	NaN	NaN	NaN	NaN	NaN	NaN
NaN	NaN	NaN	NaN	NaN	NaN	NaN	NaN	NaN	NaN
1,49416	0,69256	0,273531	0,374153	1,64435	0,484692	-0,445707	1,50573	0,421891	0,982093
-1,06487	-2,01625	NaN	-0,81893	-1,45555	-1,1992	-1,41744	-0,991421	-1,26419	-1,13767
NaN	NaN	NaN	-1,59553	NaN	-1,26512	NaN	NaN	NaN	NaN
NaN	NaN	NaN	0,0693359	NaN	-2,1965	-0,861599	NaN	NaN	NaN
0,114397	0,363796	-0,726574	0,438232	0,322149	NaN	0,903357	-0,520811	0,406964	-0,545785
1,0588	1,66266	1,11185	2,57849	2,00572	2,24026	2,62193	1,01153	1,05127	2,50421
NaN	NaN	-1,39593	-2,34851	-2,05285	-1,91478	-2,44449	-1,10373	NaN	NaN
NaN	NaN	NaN	NaN	NaN	NaN	NaN	-2,18783	-2,73393	-3,03454
-0,473774	-1,03349	-0,461191	-1,28586	0,529263	NaN	-0,948503	0,035696	-0,0537577	-0,250747
1,57767	1,76553	1,96051	1,94381	3,25232	1,04228	1,60012	1,01918	0,834018	1,37258
NaN	NaN	NaN	NaN	NaN	NaN	NaN	NaN	NaN	NaN
3,59563	2,96115	3,93925	4,61768	3,33345	2,77093	3,71404	3,13762	2,72957	2,64136
NaN	NaN	NaN	NaN	NaN	NaN	NaN	NaN	NaN	NaN
NaN	NaN	-0,201834	-1,14782	NaN	-0,431435	-1,06327	NaN	NaN	NaN
-1,55497	-1,17124	-0,571114	0,671015	-0,155954	0,32626	0,256268	-1,04883	NaN	-1,36693
NaN	NaN	NaN	NaN	NaN	NaN	NaN	-3,1208	-2,57946	-2,97931
NaN	NaN	NaN	-2,03722	-2,4145	NaN	-1,86294	NaN	NaN	NaN
-0,472256	NaN	0,447908	0,318531	-0,442392	1,34501	0,759758	-0,275324	-0,356277	-0,137691
NaN	NaN	NaN	-1,30335	-0,18585	NaN	-0,307707	NaN	NaN	NaN
NaN	NaN	-1,33835	-0,116329	-1,54863	-1,31246	NaN	-0,202452	-1,02606	-1,09033
0,477009	-0,163214	-0,862135	-0,636667	1,34894	-1,65467	0,455214	0,159597	-0,109023	0,638938

Polito et al. Universal ready-to-use immunotherapeutic approach for the treatment of cancer: expanded and activated polyclonal γδ memory T cells.
Phosphorylation sites

NaN	-0,0070591	NaN	NaN	NaN	-2,18316	-0,624403	-0,119392	-0,253171	0,388291
NaN	NaN	-0,724575	NaN	NaN	NaN	0,113644	-0,782343	-0,2774	0,611068
2,19553	1,08838	1,17705	0,00275612	2,59297	0,20092	0,82201	2,28276	1,48406	1,81227
NaN	NaN	1,14002	0,210138	NaN	0,721207	0,967159	NaN	NaN	NaN
NaN	-2,20367	-2,07981	NaN	-2,32423	NaN	-2,67695	-2,16875	-1,94679	-2,2059
1,30504	0,521551	0,827917	NaN	NaN	NaN	NaN	NaN	NaN	0,123393
-0,165092	0,313162	0,180328	0,510597	-0,466154	0,665102	NaN	-0,431452	0,282437	-1,08677
0,932465	0,328627	0,40243	1,00288	1,3178	1,57361	0,671453	1,25273	0,666593	1,43816
NaN	NaN	NaN	NaN	NaN	NaN	NaN	-0,783632	-1,92731	-1,72893
-1,20249	-1,09655	-1,50017	-0,506432	-1,60521	-0,566263	-1,08448	-1,39846	-1,16833	-1,82477
NaN	NaN	NaN	-1,32631	NaN	-1,12377	-1,9204	NaN	NaN	NaN
0,79669	2,48144	NaN	NaN	NaN	NaN	NaN	-0,407995	0,221158	0,253333
NaN	NaN	NaN	NaN	NaN	NaN	NaN	NaN	NaN	NaN
-0,512892	NaN	0,232191	NaN	0,152107	-0,246235	0,705427	NaN	NaN	NaN
-2,3635	-2,26525	NaN	NaN	NaN	-1,03388	NaN	-2,43989	-2,4002	-1,88199
NaN	NaN	NaN	0,420351	NaN	0,036562	-2,18531	NaN	NaN	NaN
3,61102	3,49538	3,50166	2,93227	4,05366	2,27311	2,92069	3,56796	3,21787	3,43708
-1,09798	-2,97665	-2,24361	-0,712023	NaN	0,918913	-1,67953	-0,347717	-0,243451	-1,89448
1,23906	NaN	1,06582	1,51536	1,13659	1,51175	NaN	2,11107	1,62464	1,96268
1,30586	-0,470549	1,01239	1,31716	1,27826	1,32731	1,21841	1,61481	1,12158	1,71069
NaN	NaN	NaN	NaN	NaN	NaN	NaN	NaN	NaN	NaN
0,833229	0,533115	-0,631533	0,754017	1,56518	NaN	0,315128	0,802475	-0,0875292	0,331611
0,705053	0,260815	-0,35417	1,00792	1,44344	NaN	1,86203	0,351685	0,102889	0,408628
NaN	NaN	NaN	-0,396849	NaN	-0,858459	-0,172262	NaN	NaN	NaN
NaN	NaN	NaN	NaN	NaN	NaN	NaN	NaN	NaN	NaN
-1,20578	-0,274223	NaN	-0,410955	-0,663954	NaN	-0,301083	NaN	NaN	NaN
NaN	NaN	1,27104	-0,148405	-2,69015	-0,360964	NaN	NaN	NaN	NaN
NaN	NaN	NaN	NaN	NaN	-0,753578	NaN	NaN	NaN	NaN
NaN	NaN	NaN	-0,719479	-3,07259	0,392574	-1,30539	NaN	NaN	NaN
-3,10246	-3,45568	-2,33316	-1,92052	-1,81019	-4,08652	-1,72136	-3,16698	-2,52899	-2,0073
NaN	NaN	NaN	-2,32714	NaN	NaN	-1,06248	NaN	NaN	NaN
2,13807	2,47912	2,05667	2,1102	-0,982395	3,31496	3,15788	1,4614	1,43234	1,356
NaN	NaN	NaN	-0,481348	-2,5227	0,385162	0,255051	NaN	NaN	NaN
0,574343	0,719868	-0,367672	0,718931	0,54248	1,30808	-0,233789	-0,0321064	-0,249095	-0,645324

Polito et al. Universal ready-to-use immunotherapeutic approach for the treatment of cancer: expanded and activated polyclonal γδ memory T cells.
Phosphorylation sites

NaN	NaN	NaN	-0,0589638	-2,18611	0,191088	1,1563	NaN	NaN	NaN
1,98528	1,78889	2,31259	2,06245	2,65662	2,62846	3,72953	1,53769	1,27412	1,51461
2,52043	1,36682	1,99962	4,41693	3,32306	3,70479	4,44937	2,17173	2,23405	2,22607
1,17783	1,84725	NaN	1,65762	0,0885773	3,92826	NaN	0,890545	0,132264	0,137294
NaN	-0,10206	-0,118912	0,304668	NaN	0,867754	-0,966293	-1,48987	-0,017415	NaN
-1,8789	-1,27981	-0,885794	-0,0635853	NaN	-0,131262	0,727325	-1,06096	-1,09847	-0,766929
-3,39024	-2,52221	-1,19306	NaN	-0,909872	NaN	-1,81568	-0,650154	-0,776469	NaN
2,82645	3,671	3,11609	2,6127	3,96591	1,57466	3,33202	3,57126	3,35874	3,27634
0,969297	2,15023	2,77842	1,25313	1,52445	2,90207	1,11408	1,59116	1,89732	0,849425
-0,0102825	-0,289602	NaN	-0,690859	NaN	NaN	-0,0878067	NaN	-0,588679	NaN
NaN	-0,844955	NaN	-0,774828	-1,16211	NaN	NaN	NaN	NaN	NaN
NaN	-2,32363	-0,661461	NaN	NaN	NaN	NaN	-1,4919	-1,85097	-2,06894
1,97765	0,164305	1,68018	2,4902	2,95708	0,41333	NaN	2,34251	2,51151	1,98922
-2,45196	-2,6439	-2,20988	-2,81262	NaN	NaN	-2,03113	-2,22373	-2,23324	-2,61974
1,95538	0,941761	2,06744	1,87	1,99506	1,13096	1,26314	2,31761	1,68705	1,96579
-0,718616	-0,557152	-1,12806	-1,05369	-0,248882	-1,07628	NaN	-0,648121	-1,21147	-1,10507
1,23793	NaN	1,05555	1,23988	NaN	1,82405	NaN	1,39201	NaN	1,22891
NaN	NaN	NaN	0,183969	NaN	-0,795311	-2,80654	NaN	NaN	-0,842031
-0,69928	NaN	-1,08776	NaN	0,0294647	NaN	NaN	NaN	NaN	NaN
-1,3631	-1,38679	-1,72472	-1,26062	-1,35111	-1,86753	-1,07283	NaN	NaN	NaN
NaN	NaN	NaN	0,153883	NaN	1,03948	-3,3722	NaN	NaN	NaN
NaN	NaN	NaN	NaN	NaN	NaN	NaN	NaN	NaN	NaN
NaN	NaN	-1,47491	NaN	-0,581186	0,997362	0,133785	NaN	NaN	NaN
-0,214039	-1,06424	-0,133997	1,53033	0,172937	2,14387	0,475702	-0,0247803	-0,333381	-0,227212
-2,97653	NaN	-1,99735	-1,19186	-1,68113	NaN	-2,18918	-2,68168	-2,58954	-2,59985
-1,30376	NaN	-0,195557	1,14839	-0,0560341	1,54223	-0,0559788	-0,882799	-1,21971	-1,0855
-0,962416	-0,0702267	NaN	-0,662533	-1,08741	2,93974	-0,908228	NaN	NaN	-1,8903
1,5743	2,06601	1,94803	2,14942	2,6955	2,29229	2,34745	1,7587	1,52747	1,90735
-1,85472	-0,40184	NaN	-2,74267	-0,41181	NaN	-1,56171	-0,537586	-0,673114	0,0835142
NaN	NaN	NaN	NaN	NaN	NaN	NaN	NaN	NaN	NaN
NaN	NaN	NaN	NaN	-0,168842	-1,311	-0,953558	NaN	NaN	NaN
NaN	-1,47048	-1,30534	-1,91053	-0,36492	NaN	-1,50809	-1,76902	-1,67132	-1,2364
-1,09412	-3,02288	-1,33696	-0,0773487	-1,30408	-1,4439	-1,02958	NaN	NaN	NaN
3,74217	3,73242	2,32364	3,23557	3,58915	2,69648	2,93292	3,29458	2,6652	3,64379

Polito et al. Universal ready-to-use immunotherapeutic approach for the treatment of cancer: expanded and activated polyclonal γδ memory T cells.
Phosphorylation sites

NaN	NaN	-0,976683	NaN	-0,844749	NaN	-1,53368	-1,02377	-1,07594	-0,84747
2,7594	2,4869	1,12036	0,868889	1,71031	2,45182	0,701309	1,59083	0,505704	0,901584
0,888893	0,911106	0,956579	-0,460886	1,79951	NaN	-0,931227	1,03436	0,845019	0,763625
NaN	NaN	NaN	-1,21173	NaN	-2,6594	-0,296082	-2,32958	-1,62598	-2,31461
NaN	NaN	NaN	NaN	NaN	NaN	NaN	NaN	NaN	NaN
2,13029	-0,657841	1,7287	-1,29837	1,32235	NaN	-0,458273	1,26399	1,5296	0,158628
-0,565134	-1,03317	-1,38914	-0,575891	-1,47005	-0,649815	-0,772095	-1,04008	-1,70068	-1,54867
NaN	2,71805	NaN	2,51395	0,458937	3,48053	3,32944	0,865437	NaN	0,422153
0,54524	-0,0289211	1,10655	1,05274	0,281651	-0,16741	0,905027	0,797462	0,804261	0,345819
NaN	NaN	NaN	NaN	-2,23882	-1,5734	-1,68836	NaN	NaN	NaN
NaN	NaN	NaN	NaN	-1,87181	-3,13979	-2,73665	NaN	NaN	NaN
NaN	-1,00304	-2,77782	-0,26029	-1,78234	-0,105505	-0,679243	-2,14209	-0,605212	NaN
-1,21824	-0,714869	1,29758	0,955896	-0,202669	1,64585	-0,470451	0,534891	0,815024	0,417262
-1,25863	NaN	-0,82535	NaN	NaN	NaN	NaN	NaN	NaN	NaN
2,74109	3,16488	3,31966	2,63501	4,06403	0,735888	3,14921	3,27534	3,39705	3,85354
NaN	NaN	NaN	-0,155964	-1,08596	-1,11542	0,25621	NaN	NaN	NaN
-0,667522	-0,593573	-0,0797195	NaN	-0,875561	-2,6182	-2,20239	0,519749	0,372291	-0,244426
2,12436	2,62533	2,41912	0,467243	3,05479	1,00317	0,906221	2,67412	2,84334	2,67872
NaN	NaN	NaN	NaN	NaN	NaN	NaN	1,01893	0,427789	0,0199766
0,444641	0,438004	0,692823	1,33228	0,222372	1,44444	1,4628	0,620026	0,595622	0,181067
1,83979	0,453102	0,400583	NaN	0,585047	1,29432	-0,0548401	NaN	NaN	NaN
NaN	NaN	NaN	-1,58872	-2,16696	-0,445353	-1,71365	NaN	NaN	NaN
NaN	NaN	NaN	0,914074	NaN	1,30362	-0,0811481	NaN	NaN	NaN
1,6997	1,34509	1,03128	1,25498	1,07004	1,70729	1,29745	1,01844	1,25448	0,29835
-0,772732	0,696892	0,54187	0,142004	0,750628	-1,17478	1,04925	0,658474	0,875522	0,694972
NaN	0,890162	0,266693	0,0585575	-0,579145	0,0112209	1,24401	NaN	NaN	0,869914
1,49051	1,65427	-0,0473804	1,30412	0,154793	2,27415	1,5016	0,405647	-0,440131	0,261569
NaN	NaN	NaN	-0,85392	-1,80313	1,06857	-0,0711346	NaN	NaN	NaN
0,669851	1,16089	0,356464	0,734592	0,851486	0,677443	0,973888	0,578873	0,803555	0,102727
NaN	NaN	NaN	-1,72895	NaN	-1,68379	-1,70015	NaN	NaN	NaN
3,41965	3,57034	3,26188	4,15236	4,16708	3,66916	3,79935	3,40666	3,25265	4,44608
-2,68902	-3,8376	-1,84254	-2,03926	-2,06517	-1,28694	-1,72757	-3,04054	-3,30574	-2,94201
NaN	NaN	NaN	-2,47433	-2,8583	NaN	-1,7417	NaN	NaN	NaN
NaN	NaN	NaN	-1,4947	NaN	NaN	-2,4075	NaN	NaN	NaN

NaN	NaN	NaN	-1,02808	-3,32002	-3,06347	-1,79716	-1,5439	-1,73488	-2,25567
NaN	NaN	NaN	-0,754032	-0,103083	-2,84781	-2,01505	-2,30645	NaN	-2,14699
3,04278	0,953875	3,42716	0,874414	2,73853	1,65326	1,97332	2,04116	1,45144	1,50008
-1,18528	0,264452	-0,0914154	-0,688843	0,432888	-1,53424	0,33115	-0,614584	-0,538218	0,199111
-0,720198	-2,23347	-0,540331	-2,15026	-1,14453	-2,49852	-1,19122	-0,396887	-0,177882	-0,956853
NaN	NaN	NaN	NaN	NaN	NaN	NaN	NaN	NaN	NaN
NaN	NaN	NaN	-1,07999	NaN	NaN	0,517063	NaN	NaN	NaN
NaN	NaN	NaN	-2,37411	-2,758	NaN	-2,40312	-2,50976	-2,31181	-2,45831
-0,714436	-0,351938	-1,08826	-0,947386	-0,970257	NaN	-0,795099	-0,646206	-0,597651	-1,01012
-0,192709	NaN	0,677929	0,579382	0,735201	0,0149021	0,535158	0,211962	0,11066	0,0290232
-0,755785	0,287098	-0,377937	NaN	0,466858	NaN	-0,960587	-0,285593	NaN	0,0796537
1,68908	1,64434	2,16226	2,85737	1,26873	3,32432	2,50841	1,24095	2,11875	1,82515
1,49851	-0,62158	0,923548	2,48347	0,645622	NaN	0,910507	0,845308	0,737805	-0,12675
NaN	NaN	NaN	NaN	NaN	NaN	NaN	NaN	NaN	NaN
NaN	NaN	NaN	-1,09099	-2,02967	-1,03704	-2,00434	NaN	NaN	NaN
NaN	NaN	NaN	0,64127	NaN	0,718077	0,796635	NaN	NaN	NaN
1,76138	2,35949	2,6876	1,26656	2,50053	-2,7884	1,54941	1,66373	1,53944	1,74731
-2,42271	0,655458	-1,98262	NaN	NaN	NaN	NaN	NaN	NaN	NaN
-1,67956	0,481163	-2,04213	-2,02632	-0,364189	NaN	-1,7179	NaN	-1,9242	-0,427672
2,18251	3,1531	1,59258	2,23622	2,39875	-1,24567	3,13101	0,348984	0,990535	2,14184
NaN	NaN	NaN	NaN	NaN	NaN	NaN	NaN	NaN	NaN
-0,805086	-1,54226	-0,719959	-0,477631	-0,215057	NaN	-0,347158	NaN	-1,17967	-1,28015
-0,807663	-1,48746	-1,93913	-2,63085	NaN	-0,861069	-0,87859	-0,412239	-0,034894	-0,0630312
-1,18285	0,5315	0,862253	0,362761	-0,78146	-1,56894	0,646973	0,688238	0,858882	0,916251
NaN	NaN	NaN	NaN	NaN	NaN	NaN	NaN	NaN	NaN
NaN	NaN	NaN	-2,19476	NaN	-2,17768	-1,63693	-1,84351	-1,64343	-0,934531
-0,214195	0,359552	0,690855	NaN	0,24181	0,467113	0,232365	NaN	NaN	0,323142
NaN	NaN	NaN	-1,70024	-1,84695	-1,75647	-1,26079	NaN	NaN	NaN
NaN	NaN	NaN	-1,36551	NaN	-1,03798	-2,11877	NaN	NaN	NaN
-0,927483	-2,53498	-0,879032	-1,76793	-0,830622	NaN	-0,66979	-0,586111	-0,741519	-1,27754
NaN	NaN	NaN	-3,13095	NaN	-2,55493	-2,77282	NaN	NaN	NaN
NaN	NaN	NaN	NaN	-0,646757	NaN	-0,870396	0,995728	-0,477393	0,21922
-1,50552	-2,452	-3,77715	NaN	1,02114	NaN	NaN	-0,886206	-0,129561	-0,291202
-0,293606	-0,716301	0,667025	-1,65322	-0,245373	NaN	-0,466524	-1,34129	-2,03221	-1,42594

0,578758	0,72751	1,30584	1,6405	0,506927	2,91095	2,38716	0,161472	0,97058	1,12562
NaN	NaN	NaN	NaN	NaN	NaN	NaN	NaN	NaN	NaN
NaN	NaN	NaN	NaN	NaN	NaN	NaN	-2,57415	-1,81849	-2,82085
NaN	NaN	NaN	-0,758877	-0,656855	-0,225632	-0,484764	NaN	NaN	NaN
2,24477	NaN	2,05606	1,2648	1,50695	2,31987	1,61559	1,80925	1,70043	1,5518
0,468695	1,40626	0,914692	1,00398	1,28013	1,43028	1,09139	0,546791	0,119239	-0,0243349
-0,477219	-1,14603	0,286415	0,617264	0,223833	NaN	0,72056	NaN	NaN	-0,50353
NaN	NaN	NaN	-2,18553	NaN	-1,16351	-2,41178	NaN	NaN	NaN
2,46378	2,65031	2,47699	2,27839	3,8844	0,933353	2,80206	3,41777	2,79004	3,16533
NaN	NaN	NaN	NaN	NaN	NaN	NaN	NaN	NaN	NaN
-0,14484	-0,377857	-1,27979	NaN	NaN	NaN	NaN	-0,807835	-0,791797	-1,03239
0,318316	-0,616175	-0,17737	-0,639528	0,169176	-0,420103	-0,166306	-0,546993	-0,823529	-0,930676
-0,65457	0,918461	-0,58386	0,183823	NaN	-0,000839233	0,0726891	0,130911	0,565312	-0,192182
NaN	NaN	NaN	NaN	NaN	NaN	NaN	NaN	NaN	NaN
NaN	NaN	NaN	-0,928789	-1,11917	-2,36394	NaN	NaN	NaN	NaN
NaN	NaN	NaN	0,0822964	NaN	-0,899607	-1,27901	NaN	NaN	NaN
NaN	NaN	NaN	0,0915718	NaN	0,372442	-1,92888	NaN	NaN	NaN
-1,00434	-1,25235	-0,942755	-0,635582	NaN	-1,1215	NaN	NaN	NaN	NaN
NaN	NaN	NaN	-2,14042	NaN	-1,99572	-1,0273	NaN	NaN	NaN
-1,05858	-4,51135	-1,25677	-0,180752	-1,08096	0,693539	0,518421	-1,52239	-0,896634	-0,983333
1,46576	0,266136	0,685593	-0,76708	-0,0228119	NaN	NaN	0,455229	-0,0347261	-0,169652
NaN	NaN	0,686647	1,88446	NaN	NaN	1,82271	-0,923567	-0,198796	-1,17843
5,44275	6,13903	5,50389	5,70977	6,16547	4,18699	5,93756	5,67907	5,27995	7,02672
NaN	NaN	NaN	0,489803	0,907505	2,95297	1,21022	-0,816858	-1,32044	-0,564466
NaN	NaN	NaN	NaN	NaN	-1,55444	NaN	-0,816858	-1,32044	-0,564466
NaN	NaN	NaN	NaN	-2,55903	NaN	-2,93667	NaN	NaN	NaN
-0,0236073	0,817327	NaN	1,16905	1,65973	1,42966	1,60967	0,784401	0,310079	1,1023
-0,282948	0,007164	-0,510403	-0,47298	-1,97187	-0,0733757	-0,446318	-1,3961	-0,928132	-1,89841
-1,51417	NaN	NaN	-0,394045	-0,52087	NaN	-0,744427	-0,995094	-1,66727	-0,278731
-1,10555	NaN	2,00348	0,676315	-0,658335	0,857738	0,472694	NaN	0,221774	NaN
NaN	NaN	NaN	0,885702	NaN	0,710701	-0,0130863	NaN	NaN	NaN
NaN	NaN	0,51104	-0,416441	NaN	-0,601322	0,445498	-1,25697	-1,00031	-1,49497
5,81653	5,43566	5,48153	4,71251	5,3996	4,83562	6,49611	5,30048	5,56376	5,18214
-1,62271	-3,29383	0,170662	1,05318	-1,30542	1,00287	0,85932	NaN	NaN	NaN

Polito et al. Universal ready-to-use immunotherapeutic approach for the treatment of cancer: expanded and activated polyclonal γδ memory T cells.
Phosphorylation sites

-1,81044	NaN	0,509924	-0,233179	NaN	NaN	0,707481	NaN	0,677918	NaN
NaN	NaN	NaN	NaN	NaN	NaN	NaN	NaN	NaN	NaN
4,9735	5,55786	5,67221	5,12507	5,54236	5,27791	5,37695	4,98542	4,81119	4,6799
NaN	NaN	NaN	-2,24259	NaN	NaN	-1,30903	-2,1989	-2,0884	-2,14709
NaN	NaN	NaN	-1,46142	-2,34902	0,117992	-0,347727	-1,03937	-1,15083	-1,28854
NaN	NaN	NaN	-1,5229	NaN	-3,16268	NaN	NaN	NaN	NaN
-0,62439	-0,654625	-0,764383	-0,312113	-0,851505	-1,58741	0,159384	-0,950211	-1,16327	-1,46109
0,181574	-1,09559	0,0421734	NaN	-0,335957	NaN	NaN	-0,567102	-0,455714	-0,703816
NaN	NaN	NaN	NaN	NaN	NaN	NaN	NaN	NaN	NaN
NaN	NaN	NaN	-2,0379	NaN	0,173769	-1,97752	NaN	NaN	NaN
NaN	-1,02494	-1,45293	NaN	-1,10957	NaN	-0,895245	NaN	-2,00289	-1,41623
-1,52916	1,61996	NaN	0,923601	NaN	1,50024	0,870119	NaN	1,90687	NaN
NaN	NaN	NaN	-0,896093	NaN	-0,96283	NaN	NaN	NaN	NaN
NaN	NaN	NaN	0,587112	NaN	1,21735	NaN	NaN	NaN	NaN
0,554541	0,0252476	0,656439	2,12312	-0,361666	2,45816	2,63925	0,0827827	0,0845728	-0,278842
3,59892	2,65028	2,61911	3,21616	3,46224	-0,479063	3,10046	2,84819	2,77735	2,67976
3,78103	2,86884	4,38145	4,00169	4,11713	4,07676	5,07693	4,36757	4,06697	4,89231
-1,47275	NaN	0,241079	0,57803	0,0772762	1,3375	0,245657	-1,14269	-1,13868	NaN
NaN	NaN	NaN	-1,55554	-2,38616	NaN	-1,33012	-0,622013	-1,1193	-1,16093
4,46116	4,31382	3,88476	5,34	5,8371	5,43613	5,88506	4,15174	4,36973	5,41253
-2,34162	NaN	-0,673952	0,589436	NaN	1,60862	-1,00063	-1,09897	0,0755129	-0,23816
-3,53822	NaN	-1,1367	0,524849	-1,282	-0,281603	1,45263	-2,38361	-2,25559	-1,39707
2,20141	1,66232	0,714687	1,60752	2,18298	2,37017	2,48281	2,12297	2,20441	2,1409
-1,9872	-1,24605	0,761576	-0,417767	-1,06904	-1,33487	NaN	-0,193382	-0,281209	0,16883
NaN	NaN	NaN	NaN	NaN	NaN	NaN	NaN	NaN	NaN
NaN	NaN	NaN	NaN	NaN	NaN	NaN	-1,78174	-2,00528	-1,85434
0,468098	NaN	0,106943	NaN	-0,243824	NaN	NaN	-1,01567	-1,34905	-1,32821
NaN	NaN	NaN	NaN	-0,62108	NaN	NaN	-1,82713	-1,76203	-1,09586
NaN	NaN	-0,608856	NaN	NaN	NaN	NaN	1,07896	0,671494	0,854384
0,583801	0,567686	0,258921	1,63578	-0,65995	1,27157	2,14093	-0,341984	0,459247	0,0607901
0,746487	1,39345	0,842884	0,808876	2,02016	NaN	1,25586	0,817698	0,274356	1,62824
-1,85165	NaN	-2,2622	NaN	NaN	NaN	-1,77267	-1,55185	-1,94995	-2,03065
0,834732	0,215649	-1,47934	1,38728	1,07355	1,93279	1,77707	1,07008	0,998256	0,76295
-2,53607	-2,47122	-1,76781	-2,4202	-2,68266	-2,41217	NaN	NaN	NaN	NaN

Polito et al. Universal ready-to-use immunotherapeutic approach for the treatment of cancer: expanded and activated polyclonal γδ memory T cells.
Phosphorylation sites

1,86862	2,37813	2,02827	2,41181	2,33159	2,30005	2,54639	1,8357	2,46614	2,2178
-0,540188	NaN	-0,261641	0,152214	-0,150043	0,0828953	-0,431564	0,00917435	-0,666295	-0,550719
NaN	NaN	NaN	-0,993671	NaN	0,199982	NaN	NaN	NaN	NaN
NaN	NaN	NaN	NaN	NaN	NaN	NaN	NaN	NaN	NaN
NaN	NaN	NaN	-0,953175	NaN	NaN	-1,09954	NaN	NaN	NaN
1,54645	2,40438	2,27042	1,96412	2,842	2,40914	2,00946	2,19632	2,19539	2,2071
NaN	NaN	-0,49679	0,586256	NaN	-0,187876	0,942139	NaN	NaN	NaN
NaN	-1,32328	-0,134323	0,458977	NaN	0,320129	0,655369	-1,71955	-0,48409	NaN
-2,73722	0,323034	-1,91976	-0,722176	-0,885191	NaN	-0,0739307	NaN	NaN	NaN
-0,818407	NaN	1,55636	1,01175	-0,366972	1,47812	0,429375	-0,490324	-0,0326471	-0,71515
-2,71879	-2,3371	NaN	-0,702448	-2,46439	-2,4372	-1,5815	NaN	NaN	NaN
NaN	NaN	-0,37722	0,0580273	-1,07598	-0,0202732	0,18432	-1,82016	-1,7621	-2,10127
NaN	NaN	NaN	NaN	NaN	NaN	NaN	NaN	NaN	NaN
NaN	-2,06118	NaN	-0,565489	-0,355686	2,85459	-0,52816	4,35665	4,40791	4,48963
2,4841	0,873592	1,98631	1,70834	1,64335	1,47149	1,7742	2,64079	2,83479	2,11332
NaN	NaN	NaN	NaN	NaN	NaN	NaN	-1,74441	-2,20744	-1,99399
NaN	NaN	NaN	NaN	NaN	NaN	NaN	NaN	NaN	NaN
2,42448	2,35898	1,51037	2,73166	2,56143	-0,868643	2,94217	2,50972	2,22096	2,30931
NaN	NaN	NaN	-1,38857	-3,21819	NaN	-2,30998	NaN	NaN	NaN
NaN	NaN	NaN	-0,859945	NaN	-0,410372	-1,7991	NaN	NaN	NaN
NaN	NaN	NaN	0,140255	NaN	0,598759	-1,72979	NaN	NaN	NaN
0,0562305	0,418903	-0,686075	NaN	NaN	NaN	-0,173168	0,0374718	-0,609681	-0,149913
NaN	NaN	-1,7749	-0,860413	-2,67576	-0,401495	NaN	NaN	NaN	NaN
-1,34337	-1,08981	-0,900517	-0,926098	-0,959324	-0,885391	-0,973473	NaN	NaN	-1,35437
-0,919861	-2,14008	-1,2409	0,229208	-1,11905	1,02123	1,29563	-0,0970516	-0,481854	-0,688203
0,347748	0,87138	-0,302542	1,73022	-1,21248	3,20412	0,631477	-1,3387	-0,280137	-1,25386
0,821531	2,21246	1,24382	2,45493	1,55554	3,2181	2,13621	0,575394	0,587455	1,09737
0,311228	1,29829	-0,47456	1,3229	-1,74298	NaN	0,88096	-0,415096	0,643409	-0,224555
0,311228	1,29829	-0,47456	1,3229	-1,74298	1,66427	0,88096	-0,415096	0,643409	-0,224555
NaN	NaN	NaN	-0,958239	NaN	NaN	-0,153847	0,22134	-0,340739	-0,274867
0,275457	-0,385164	0,00845528	0,0766411	-0,616932	-1,84514	0,489346	0,187134	0,489917	-0,424743
-2,77562	-1,14796	NaN	NaN	-1,04537	NaN	-1,59204	NaN	NaN	NaN
2,30973	2,35797	1,61268	0,239225	2,23428	0,948647	0,452423	2,36476	1,70591	2,19347
NaN	NaN	1,09548	-2,53846	NaN	0,310017	-0,893873	NaN	NaN	NaN

0,720579	1,42141	-0,972641	-2,47638	NaN	-1,60903	NaN	1,13569	1,0631	0,210896
-0,522446	NaN	NaN	-1,46349	-0,43861	-0,0392342	NaN	NaN	-1,01964	NaN
-2,6683	NaN	1,85025	-0,561604	NaN	0,408281	-0,0491886	-1,85953	-1,69257	-2,22702
1,0275	1,29778	0,792744	2,75706	1,6973	0,404757	2,2798	1,62414	1,71049	NaN
NaN	NaN	-1,0185	-0,54735	-2,84584	NaN	-1,00036	NaN	NaN	NaN
-2,6352	NaN	-0,122391	0,472818	-1,23372	-0,239145	0,377594	NaN	NaN	NaN
NaN	NaN	NaN	-2,09644	NaN	-2,03285	NaN	NaN	NaN	NaN
1,92731	3,16016	1,74788	2,14386	2,45852	2,30029	2,84738	1,64606	2,70355	2,64474
NaN	-2,37184	0,0947876	1,80583	-1,73586	2,01428	1,47266	-0,37479	-0,611842	-0,856544
3,00118	4,19246	3,34122	4,84959	3,91095	3,58708	4,75748	4,10132	3,94298	4,385
6,42377	5,97357	7,24659	6,02782	7,42923	5,73733	6,33294	7,07203	6,67845	6,82448
NaN	-0,474678	NaN	0,0120087	0,514669	0,60334	NaN	NaN	NaN	NaN
0,916471	-0,0186749	1,89345	-2,12414	0,96203	-1,14077	-2,15763	1,19496	0,994761	0,946216
NaN	NaN	-3,40888	-2,48109	NaN	-2,86429	-2,39817	NaN	NaN	NaN
NaN	-1,06929	-1,80525	-2,11193	-0,0686226	NaN	-0,688902	-2,34066	-1,76002	NaN
NaN	NaN	NaN	NaN	NaN	NaN	NaN	-2,96872	NaN	-2,63934
NaN	NaN	NaN	NaN	NaN	-2,24527	-1,16899	NaN	NaN	NaN
NaN	NaN	-0,322998	-1,97483	NaN	-1,69659	-1,36199	-1,7316	-0,957501	-1,30239
NaN	NaN	1,16557	NaN	-1,16453	NaN	-2,02529	0,344002	1,03705	0,50623
-0,257324	NaN	-0,905708	-1,93774	-0,136385	-1,29504	-2,11374	NaN	NaN	NaN
-2,95662	NaN	-2,63792	-1,44261	NaN	-2,04461	NaN	-2,79763	-2,48389	-2,3396
NaN	NaN	NaN	NaN	NaN	NaN	NaN	NaN	NaN	NaN
-0,313133	-0,630404	-0,902456	NaN	NaN	NaN	NaN	NaN	NaN	NaN
-1,16337	-1,83781	-1,32321	-1,64257	-1,16958	NaN	-0,414545	-1,75178	NaN	-1,50021
NaN	NaN	NaN	0,263531	-0,687893	NaN	0,890154	NaN	NaN	NaN
-1,45306	-1,69061	NaN	-0,126728	NaN	-0,726141	-0,45447	NaN	NaN	NaN
-1,43605	-3,0296	-2,66307	NaN	NaN	NaN	NaN	-0,454473	-1,80732	-1,91043
NaN	NaN	NaN	NaN	NaN	NaN	NaN	NaN	NaN	NaN
-2,0597	-2,87624	NaN	NaN	-1,26338	NaN	NaN	-2,87116	-2,05367	-3,28835
NaN	NaN	NaN	-2,30027	NaN	-2,13689	-2,07198	NaN	NaN	NaN
NaN	NaN	NaN	-2,00418	-0,477629	-2,57122	NaN	NaN	NaN	NaN
NaN	NaN	NaN	NaN	NaN	NaN	NaN	-1,38153	-1,76496	-1,74692
NaN	NaN	NaN	0,875618	1,06732	1,02212	0,560745	-0,0298386	-0,515672	NaN
1,44656	NaN	0,61067	3,43349	1,27473	1,77905	0,690966	1,38915	1,04303	1,52302

Polito et al. Universal ready-to-use immunotherapeutic approach for the treatment of cancer: expanded and activated polyclonal γδ memory T cells.
Phosphorylation sites

NaN	NaN	NaN	NaN	NaN	-0,583902	NaN	-3,54531	NaN	NaN
1,35825	2,63142	1,79134	2,21436	2,22384	NaN	2,22989	2,17405	1,91347	1,91028
NaN	3,92122	3,27781	3,31825	3,01788	NaN	2,45043	2,37883	NaN	2,17596
0,150278	0,932976	0,142633	0,0651608	NaN	0,250519	-0,10544	NaN	NaN	NaN
NaN	NaN	NaN	NaN	NaN	-1,59203	-1,27646	NaN	NaN	NaN
NaN	NaN	0,724213	1,15236	0,199774	1,55905	0,897249	-0,221199	-1,77166	-0,94214
-1,87881	-0,939756	-1,27604	-1,42291	NaN	NaN	-0,444004	NaN	-1,9521	-2,03572
NaN	NaN	NaN	-1,22908	NaN	NaN	NaN	NaN	NaN	NaN
NaN	NaN	NaN	-2,12992	-1,6199	-1,74872	-1,87978	NaN	-1,7163	NaN
NaN	NaN	-0,762333	NaN	-0,769682	-0,46707	-0,143564	NaN	NaN	NaN
NaN	NaN	NaN	-3,64258	NaN	3,15142	0,208658	NaN	NaN	NaN
NaN	NaN	NaN	NaN	NaN	NaN	NaN	-3,61857	-3,0548	-1,8555
-0,618107	0,597027	-0,159079	NaN	-0,705357	NaN	NaN	NaN	0,871604	0,845727
NaN	NaN	NaN	NaN	NaN	NaN	NaN	NaN	NaN	NaN
NaN	NaN	NaN	NaN	NaN	NaN	NaN	NaN	NaN	NaN
NaN	NaN	NaN	-1,94261	-1,30373	-1,04664	-1,55473	NaN	NaN	NaN
NaN	0,319042	0,116047	1,13347	1,59908	NaN	-0,634531	-0,883858	-0,100686	0,605075
NaN	NaN	NaN	-2,10797	-2,79845	-2,37142	-2,39346	NaN	NaN	NaN
NaN	NaN	NaN	NaN	-0,938904	NaN	NaN	3,04878	2,45903	3,21259
-0,0618858	-1,99802	-1,36847	-0,320536	-0,855974	-0,592964	0,232496	NaN	NaN	NaN
-1,31972	-0,704983	-0,257072	-1,12571	0,343616	-1,68672	0,140593	0,238293	-0,017705	-0,208846
NaN	NaN	NaN	NaN	NaN	NaN	NaN	-0,875093	-0,986629	0,554376
-0,603382	1,22947	1,13578	-2,13419	1,1716	-1,67649	-0,215874	1,50573	2,2911	1,62296
NaN	NaN	NaN	NaN	NaN	NaN	NaN	-2,08531	-2,33957	-2,13132
-3,77784	-1,66232	NaN	-1,58347	-0,911263	-0,938534	-0,550013	NaN	NaN	-1,71666
-2,44551	-2,23362	-1,09685	-0,237225	-1,43089	0,369293	-0,840317	-1,80666	-2,02243	-2,40543
NaN	NaN	NaN	-2,886	-2,19533	NaN	-2,04148	NaN	NaN	NaN
NaN	0,33568	NaN	-2,03225	NaN	-0,204903	NaN	0,184416	0,0599165	0,294892
-1,25927	-0,221432	-1,69827	NaN	NaN	NaN	NaN	-0,410315	0,302333	0,120568
-0,652464	-0,493702	-1,0403	NaN	NaN	NaN	NaN	1,14372	1,04622	0,807818
NaN	NaN	NaN	-0,899519	NaN	-2,28572	-2,3964	NaN	NaN	NaN
NaN	NaN	NaN	NaN	NaN	NaN	NaN	NaN	NaN	NaN
-1,0068	NaN	NaN	1,01476	NaN	0,946037	1,03362	NaN	NaN	NaN
-2,27842	NaN	1,04028	2,34605	NaN	2,43394	1,62069	-1,06372	-1,31054	-0,865462

Polito et al. Universal ready-to-use immunotherapeutic approach for the treatment of cancer: expanded and activated polyclonal γδ memory T cells.
Phosphorylation sites

-2,18168	-1,42259	-1,22288	-2,28094	-0,759008	-2,64201	-2,51466	-1,43549	-1,80045	-1,85966
NaN	NaN	NaN	-0,814463	NaN	0,742706	-1,21095	NaN	NaN	NaN
-0,850405	1,1227	0,321154	1,21847	1,66566	0,524307	1,74683	-1,20067	-0,755439	1,8486
NaN	NaN	NaN	NaN	NaN	NaN	NaN	NaN	NaN	NaN
-0,755672	-0,31649	-0,0282326	0,258177	0,944733	-1,02066	0,421688	0,050848	-0,109487	0,00877476
NaN	NaN	NaN	NaN	NaN	NaN	NaN	NaN	NaN	NaN
NaN	NaN	NaN	-1,41573	NaN	-0,688206	-0,725052	NaN	NaN	NaN
0,0484371	-1,41672	NaN	0,491243	NaN	0,789215	-0,0204792	-0,610962	0,174872	-0,503497
2,04891	1,73009	2,45296	3,26923	1,6311	2,09526	2,85309	2,18094	2,277	2,64547
NaN	NaN	-0,89109	0,150427	NaN	0,423283	-0,191017	NaN	NaN	NaN
NaN	NaN	NaN	NaN	NaN	NaN	NaN	NaN	NaN	NaN
NaN	NaN	NaN	NaN	NaN	NaN	NaN	NaN	NaN	NaN
NaN	NaN	NaN	NaN	NaN	NaN	NaN	NaN	NaN	NaN
-2,33902	-0,838284	-1,13232	NaN	NaN	NaN	NaN	NaN	NaN	NaN
0,428577	0,963734	1,6557	NaN	2,089	-2,11773	NaN	0,989336	0,874852	0,985343
0,399632	2,23265	1,55744	-0,104265	0,7218	0,261856	1,39602	-0,0185757	0,28579	0,192439
-2,37631	-0,190935	-1,28258	NaN	-1,42813	-2,5196	-1,15365	NaN	NaN	NaN
NaN	-2,77297	-2,3957	-2,49015	-1,90433	NaN	-1,58989	NaN	NaN	NaN
NaN	NaN	NaN	NaN	-1,70142	-0,390112	-0,957718	NaN	NaN	NaN
-0,118214	-1,2909	0,0720215	NaN	-0,145161	-0,974979	-1,34395	-0,950453	NaN	NaN
NaN	NaN	2,98475	NaN	-0,409203	NaN	NaN	0,483816	0,286748	-0,339662
NaN	0,151878	NaN	NaN	NaN	NaN	NaN	-0,293886	-0,574073	0,951922
NaN	NaN	1,35754	NaN	NaN	NaN	NaN	-0,751068	-1,52178	-1,40951
-1,08117	-4,0545	-1,27666	-3,09833	-1,07406	NaN	-2,48295	-0,0817928	-0,493213	-1,16906
3,79062	4,04085	2,82995	2,36569	NaN	4,11225	1,10049	3,84225	3,75352	2,65847
3,98065	3,51008	4,42808	3,87085	4,47549	4,16798	4,14874	4,30699	3,78939	3,7038
1,88865	1,4744	1,54913	2,24419	1,02617	2,91301	2,30805	0,798323	0,949494	1,0032
2,3002	2,03863	2,38125	1,08039	2,44579	NaN	1,15369	2,12429	1,86616	1,51461
-1,1867	2,50125	3,02046	1,83033	2,9072	0,670233	NaN	3,0221	3,05892	2,60361
NaN	NaN	NaN	1,37082	-1,59968	2,10727	-1,8825	-1,47641	-2,01445	-1,33589
-0,208303	0,0741901	NaN	NaN	NaN	NaN	NaN	0,138386	0,65882	0,77641
NaN	NaN	NaN	-1,98819	NaN	NaN	NaN	NaN	NaN	NaN
NaN	NaN	NaN	NaN	NaN	-4,17166	NaN	NaN	NaN	NaN
NaN	NaN	NaN	3,08769	NaN	1,85267	1,84004	NaN	NaN	NaN

NaN	NaN	NaN	-4,20224	NaN	NaN	-4,4811	NaN	NaN	NaN
NaN	NaN	NaN	0,96553	-1,82633	0,387911	NaN	NaN	NaN	NaN
NaN	NaN	NaN	NaN	NaN	NaN	NaN	NaN	NaN	NaN
NaN	NaN	NaN	-0,418036	NaN	-1,75057	0,00279045	NaN	NaN	NaN
NaN	NaN	NaN	NaN	NaN	NaN	NaN	NaN	NaN	NaN
NaN	NaN	NaN	-3,21288	NaN	-2,55233	-1,55835	NaN	-1,63309	NaN
-0,917921	-0,0940399	1,62607	1,48993	0,480127	-1,54952	1,38588	0,150616	-0,0115519	0,637641
0,516117	0,791729	0,354212	0,179544	0,0743084	0,282495	0,607916	0,442436	0,416293	0,348994
NaN	NaN	NaN	NaN	NaN	NaN	NaN	NaN	NaN	NaN
NaN	-2,8019	NaN	NaN	NaN	NaN	NaN	-0,312536	-0,425075	-0,401639
-0,211401	0,500122	-0,341948	-2,18378	0,387596	-1,97314	0,142881	1,32648	1,35572	3,08518
-0,812359	-0,462519	-1,53894	-2,77981	-0,316175	NaN	-0,840197	-0,478739	-1,53581	0,225064
-1,39638	-2,55828	-0,704748	0,355595	-0,415825	1,0052	0,618202	-0,931633	-1,06723	-1,08355
1,03013	0,198509	NaN	1,45737	1,75538	NaN	0,916912	NaN	NaN	NaN
NaN	NaN	NaN	-1,98251	NaN	-0,352232	-2,10045	NaN	NaN	NaN
-2,94122	-2,77105	-2,67534	NaN	NaN	NaN	NaN	-2,62408	-2,77012	-2,25874
0,282026	0,939001	0,740164	NaN	1,18354	NaN	NaN	0,802761	0,285823	0,922923
1,93182	1,89285	2,76017	0,162861	2,53988	NaN	0,720425	2,73582	1,70535	2,33282
1,97555	1,16604	1,97179	0,800034	2,27438	NaN	1,35834	2,01153	NaN	NaN
4,67817	NaN	2,69059	0,511372	4,02126	4,9424	2,06989	4,5614	4,76374	5,64226
2,58338	NaN	4,89363	4,36435	6,28828	3,36711	3,85363	7,12317	6,43129	3,73609
4,67817	NaN	2,69059	0,511372	4,02126	4,9424	2,06989	4,5614	4,76374	5,64226
2,58338	NaN	4,89363	4,36435	6,28828	3,36711	3,85363	7,12317	6,43129	3,73609
NaN	NaN	NaN	NaN	NaN	NaN	NaN	NaN	NaN	NaN
-0,284224	-0,54986	0,620907	1,68749	NaN	1,41813	NaN	NaN	NaN	NaN
-1,00896	-1,59937	NaN	NaN	NaN	NaN	NaN	NaN	NaN	NaN
1,0715	3,34204	-0,451883	1,61954	1,67201	1,36897	2,85383	1,4877	0,829408	2,44888
-0,168301	1,47915	NaN	0,925442	0,331015	1,25916	1,14503	-1,01911	-0,892745	0,316283
-0,905449	NaN	0,122156	0,598448	-3,32216	-0,785589	-0,930204	1,66899	1,39165	0,992166
NaN	NaN	1,51015	0,996817	1,51582	1,47403	-0,605068	2,36471	1,65927	2,23701
6,42989	6,08858	5,8868	5,36587	5,9148	1,09876	5,62038	6,24059	5,93156	5,5958
NaN	-1,43555	NaN	NaN	NaN	NaN	NaN	-2,92582	NaN	-1,62639
-0,447361	-1,19077	-0,45953	-0,162039	0,334152	-0,687628	-0,517405	0,561146	0,587592	0,40598
NaN	-0,630526	NaN	-1,56269	0,236534	-0,583166	NaN	NaN	NaN	NaN

Polito et al. Universal ready-to-use immunotherapeutic approach for the treatment of cancer: expanded and activated polyclonal γδ memory T cells.
Phosphorylation sites

7,33653	7,00001	7,65688	6,27244	7,75804	6,79125	6,40517	7,55059	7,21939	7,22724
-2,42211	-1,22872	-0,314671	0,240906	0,139772	-0,664478	0,149324	-1,83503	-2,02101	-0,880002
-0,226051	-0,426418	-1,19485	1,61852	-0,550838	3,75838	0,769491	NaN	NaN	-1,6683
3,7582	3,93565	3,09176	2,87882	3,97926	2,28493	2,90222	2,42661	2,33774	3,09547
-1,55858	NaN	-1,65697	-0,418135	-0,824568	-0,363459	-0,989799	-0,876238	-1,44613	-1,36243
NaN	NaN	NaN	NaN	NaN	NaN	NaN	NaN	NaN	NaN
NaN	-1,73835	-2,38647	-0,894112	-1,67794	-2,75833	-0,936905	-2,27835	-2,31181	-2,30858
0,172234	1,29664	-0,306206	-0,662533	0,876867	-0,193346	0,174768	1,04709	-0,433774	0,484088
NaN	NaN	NaN	-1,18077	-1,93346	-0,973991	-2,06776	NaN	NaN	NaN
NaN	NaN	-0,607176	0,384468	NaN	-0,917444	-0,121031	NaN	NaN	NaN
NaN	NaN	NaN	NaN	NaN	NaN	NaN	NaN	NaN	NaN
NaN	NaN	NaN	-0,301561	NaN	-0,987305	-1,91846	NaN	NaN	NaN
NaN	NaN	NaN	1,69568	NaN	2,10919	0,0304489	NaN	NaN	NaN
2,26743	NaN	1,57334	4,81924	1,86193	1,94635	NaN	1,54592	1,48392	1,45851
NaN	NaN	NaN	2,45439	-1,8951	2,08434	1,09715	NaN	NaN	NaN
NaN	NaN	NaN	NaN	NaN	NaN	NaN	NaN	NaN	NaN
NaN	NaN	NaN	NaN	NaN	NaN	NaN	NaN	NaN	NaN
0,114027	-0,000452042	0,909622	0,416086	0,442062	NaN	0,493177	0,201262	-0,240056	-0,224475
NaN	NaN	NaN	-0,115274	-3,04395	NaN	-1,01248	NaN	NaN	NaN
-3,58771	-3,4945	-2,91764	-2,71392	NaN	-2,52063	-2,56746	-2,56093	-2,84514	NaN
NaN	NaN	NaN	NaN	NaN	NaN	NaN	-1,77353	-2,29964	-2,38419
1,11862	-0,87447	1,36834	3,0892	1,19948	NaN	2,59739	0,00644493	-0,574927	-0,0129423
NaN	NaN	NaN	-0,831764	1,13582	0,833128	NaN	NaN	NaN	1,6502
-0,694866	NaN	-1,05586	-2,79402	-2,64358	-0,224091	NaN	NaN	NaN	1,02377
NaN	NaN	NaN	-2,11575	-2,16186	-3,68032	-1,89118	-1,78037	-1,97127	NaN
NaN	NaN	3,7488	-0,110245	0,348896	-1,52307	1,71988	0,479511	1,34251	0,663198
NaN	NaN	NaN	NaN	NaN	NaN	NaN	NaN	NaN	NaN
0,665777	0,702358	0,779345	1,69392	1,31848	1,8832	1,8217	1,0193	1,37055	1,10976
NaN	NaN	NaN	NaN	NaN	NaN	NaN	0,362337	-0,422544	-0,170762
NaN	NaN	-1,58876	0,0969791	-0,838888	-0,51211	-1,27857	NaN	NaN	NaN
-0,597731	-0,971846	0,465069	0,0767632	-0,559486	NaN	-0,600115	NaN	NaN	NaN
-4,88485	-2,78709	5,794	3,43387	-0,475927	3,52034	4,7472	-0,505222	0,430514	NaN
-0,934547	-0,0833206	NaN	0,509178	-0,171305	NaN	0,205444	-0,147766	-0,451192	-0,884826
NaN	NaN	NaN	-0,838125	NaN	NaN	-0,955723	NaN	NaN	NaN

Polito et al. Universal ready-to-use immunotherapeutic approach for the treatment of cancer: expanded and activated polyclonal γδ memory T cells.
Phosphorylation sites

NaN	NaN	NaN	NaN	NaN	NaN	NaN	-1,84244	-1,92116	-1,35068
NaN	NaN	NaN	NaN	NaN	NaN	NaN	NaN	NaN	NaN
NaN	NaN	NaN	NaN	NaN	NaN	NaN	NaN	NaN	NaN
1,48271	1,53263	1,50291	NaN	1,80513	NaN	0,46648	0,879604	0,924537	1,0977
NaN	0,844564	-0,894318	-0,292572	0,934713	NaN	0,897009	-1,20176	NaN	NaN
3,10934	2,88059	3,10226	4,53089	3,62032	2,29317	NaN	NaN	3,37471	3,51487
2,07171	0,794428	3,02601	2,23965	3,8194	-1,63548	3,11405	3,76559	3,66229	2,90594
6,86627	6,43685	7,03763	6,64084	8,22851	-0,814339	7,97643	7,50202	6,94252	7,52888
NaN	NaN	NaN	NaN	NaN	NaN	NaN	NaN	NaN	NaN
-0,418585	0,175383	0,183598	-1,73677	0,459061	-0,538567	-1,82199	0,480566	-0,00633144	0,757123
-0,492714	-0,66605	0,145281	-0,895308	-1,40129	-0,340714	-1,55585	-0,829214	-1,14162	NaN
1,35372	0,706566	0,669958	0,284435	0,711349	0,702698	0,800873	0,57546	0,267625	0,0411406
-0,122021	0,658127	0,321934	0,193388	0,71166	-0,188421	0,357536	0,385721	-0,0438089	0,724856
NaN	NaN	NaN	NaN	NaN	NaN	NaN	NaN	NaN	NaN
4,56269	3,00371	6,1774	5,82018	6,76642	0,639021	6,98711	6,45977	6,13284	5,75789
6,44632	6,19622	7,6533	8,23659	8,13774	4,56401	8,23933	7,57355	7,39196	7,32515
NaN	NaN	NaN	NaN	NaN	NaN	NaN	NaN	NaN	NaN
NaN	NaN	NaN	NaN	NaN	NaN	NaN	NaN	NaN	NaN
NaN	NaN	NaN	NaN	NaN	NaN	-0,689478	NaN	NaN	NaN
4,12054	2,78589	3,15704	NaN	5,24309	2,03443	1,36677	3,96322	3,87275	3,84951
3,56667	5,37261	4,52729	4,24223	3,05294	5,18633	4,51447	4,1849	4,08352	4,2996
-2,45318	NaN	0,329308	NaN	NaN	NaN	NaN	-0,397543	-0,69012	NaN
3,85119	3,88561	2,96827	4,23561	4,02884	3,2416	4,62247	3,25309	3,96862	3,81526
-0,00644302	-1,43004	-0,811855	0,311573	0,387291	0,876461	1,11284	0,317686	-0,187705	-0,725205
NaN	NaN	NaN	NaN	NaN	NaN	NaN	NaN	NaN	NaN
-2,2882	-2,06144	-3,20727	NaN	-1,73274	NaN	NaN	NaN	NaN	NaN
-2,43545	-1,80756	-1,75487	NaN	NaN	NaN	NaN	-1,83601	-1,74035	-2,44113
0,442129	-0,751616	0,0787411	2,62848	0,647404	3,01812	1,79329	NaN	-0,47745	-0,831048
4,8939	4,46868	3,70316	7,33562	3,94867	5,08963	6,80252	4,40796	4,51414	4,04984
-0,115501	-0,528624	NaN	0,695627	NaN	-1,47704	NaN	NaN	-2,00825	NaN
-2,62704	-2,54858	-2,83269	NaN	NaN	NaN	NaN	NaN	NaN	NaN
NaN	NaN	NaN	NaN	NaN	NaN	NaN	NaN	-2,28768	NaN
0,456108	-0,516407	1,10871	0,743624	1,35362	1,7486	1,62243	-0,405186	0,132302	0,458396
-0,574677	-1,21169	-0,42065	1,02473	-0,25613	0,857738	0,364357	-0,387688	-0,739121	0,0588961

Polito et al. Universal ready-to-use immunotherapeutic approach for the treatment of cancer: expanded and activated polyclonal γδ memory T cells.
Phosphorylation sites

-0,484976	-0,192144	0,00577545	1,16619	-1,14064	1,29481	-0,546059	NaN	NaN	NaN
5,70019	5,5185	4,6855	2,56318	5,17837	2,58242	2,56883	4,61134	4,71029	4,51515
NaN	NaN	NaN	-1,34398	NaN	-1,1849	-1,76376	NaN	NaN	NaN
NaN	NaN	NaN	NaN	NaN	1,02666	NaN	-2,28952	-1,43161	-1,99134
NaN	NaN	NaN	-1,24568	-1,15773	NaN	-1,00313	NaN	-3,13522	-1,04606
-0,225163	1,11287	0,43442	0,0878239	0,65773	NaN	1,10579	-1,60678	-1,21861	0,0499239
-1,56194	1,5856	5,24508	NaN	NaN	5,33562	NaN	-2,02427	NaN	-1,98533
2,30563	NaN	NaN	4,24921	3,34049	1,50307	4,85251	2,87689	2,43682	2,5757
1,74529	1,28198	NaN	6,80696	-2,49525	7,02902	4,59453	1,76774	1,41768	NaN
NaN	NaN	NaN	NaN	NaN	NaN	NaN	NaN	NaN	NaN
-0,363214	-0,787279	3,18402	2,67827	-1,39007	3,8299	3,84616	1,00209	1,01743	0,70641
NaN	NaN	NaN	-2,26035	-2,48978	NaN	-2,03168	NaN	NaN	NaN
NaN	NaN	0,811344	-0,116529	-0,660219	1,05132	0,298561	-0,528957	NaN	-1,1504
2,59558	1,49979	3,2763	2,9847	-1,07556	3,99284	3,13595	1,15866	1,93299	1,18457
NaN	NaN	NaN	-1,95019	-3,97395	-1,06355	NaN	NaN	NaN	NaN
-1,93532	-2,07673	-0,13216	-0,204556	NaN	0,34676	-1,04822	-1,9322	-1,12175	-2,10907
NaN	NaN	NaN	-1,89726	-4,21777	NaN	-2,45499	NaN	NaN	NaN
NaN	NaN	NaN	NaN	NaN	NaN	NaN	NaN	NaN	NaN
NaN	NaN	-0,0268269	0,179886	NaN	0,00167656	0,601126	-1,00292	0,161605	-0,382726
-1,95716	-2,118	-0,872654	NaN	NaN	NaN	NaN	-0,582783	-0,749978	-0,423173
-1,04355	-0,767307	-0,613129	-0,298641	-0,926313	-0,276035	-0,716543	-1,66285	-1,24734	-1,76202
NaN	NaN	NaN	NaN	NaN	NaN	-1,28902	NaN	NaN	NaN
-1,65981	-3,32514	2,56153	2,18563	-2,60822	1,8712	3,63689	0,696018	0,2604	1,18224
NaN	NaN	NaN	-2,65508	NaN	NaN	-0,682575	NaN	NaN	NaN
NaN	NaN	NaN	-1,8743	NaN	-1,4677	-0,745174	NaN	NaN	NaN
NaN	NaN	NaN	0,790819	0,772064	-1,0905	1,22004	-0,0207767	NaN	NaN
-1,79836	-2,73607	0,948584	2,11152	-0,354267	1,99484	2,76002	1,06258	1,05454	1,08228
NaN	NaN	NaN	NaN	NaN	-1,93287	-1,1335	NaN	NaN	NaN
4,23057	5,43699	4,82574	4,79441	6,14135	4,98699	5,72662	5,59492	5,084	5,7425
NaN	NaN	-0,728504	-0,459469	NaN	-0,687859	0,254189	NaN	NaN	NaN
-0,77854	-0,513935	0,143709	-3,00434	0,2451	-2,59629	-1,5874	-0,0130291	0,33377	0,34526
-1,0191	-1,68318	-1,08938	-1,83514	-1,50126	-3,01143	NaN	NaN	NaN	NaN
NaN	NaN	NaN	0,302553	NaN	0,374662	-0,0281391	NaN	NaN	NaN
-2,5155	-2,83386	-3,82733	-1,95667	-3,85698	-2,23316	NaN	NaN	NaN	NaN

Polito et al. Universal ready-to-use immunotherapeutic approach for the treatment of cancer: expanded and activated polyclonal γδ memory T cells.
Phosphorylation sites

-1,54692	0,802042	-0,213289	0,485085	0,153591	0,927759	0,968872	-0,5221	-0,236354	0,66031
NaN	NaN	NaN	NaN	NaN	NaN	NaN	NaN	NaN	NaN
NaN	NaN	NaN	NaN	NaN	NaN	NaN	NaN	NaN	NaN
NaN	NaN	NaN	NaN	NaN	NaN	NaN	NaN	NaN	NaN
NaN	NaN	NaN	NaN	NaN	NaN	NaN	NaN	NaN	NaN
NaN	NaN	NaN	NaN	NaN	NaN	NaN	NaN	NaN	NaN
-1,04548	-0,45174	NaN	-0,748709	-1,39007	0,0461121	-0,647566	-1,76207	-1,4034	-0,564059
NaN	NaN	NaN	2,28146	-2,37427	2,7626	0,406797	NaN	NaN	NaN
8,48838	9,20622	8,92413	3,73939	9,56212	4,90589	4,35566	9,90121	9,74175	10,5192
NaN	NaN	NaN	NaN	NaN	NaN	NaN	NaN	NaN	NaN
-1,18503	-0,340315	0,130466	-2,24154	-0,829966	NaN	-1,05693	0,329664	0,33986	0,52316
-1,66923	-1,97011	-0,318735	NaN	NaN	NaN	NaN	0,987953	0,179227	0,441901
NaN	NaN	1,44966	-0,895411	NaN	0,83803	-1,41059	1,52544	1,77106	-1,05062
5,31138	0,289013	4,48447	1,52747	-0,284212	6,38964	NaN	6,15195	5,7057	1,80184
3,61245	2,51317	3,2444	3,0674	3,77019	1,96544	3,06416	3,51381	3,62504	2,91543
-0,99777	NaN	-0,824224	1,62575	-0,934875	-0,201866	-0,0313778	-0,59016	-1,10489	-0,721038
0,835983	1,75717	1,72216	2,51947	2,3549	2,54608	2,35109	1,38218	0,810355	1,15189
-0,996389	0,103472	0,30467	-1,61275	1,39864	NaN	-0,126314	-0,583605	-0,623265	0,57293
2,2172	3,25886	1,53801	1,48094	2,14819	0,269234	NaN	NaN	1,64334	1,89092
NaN	-0,17054	1,78876	-0,312294	NaN	NaN	1,24392	-0,270411	-0,257455	0,637832
0,56106	1,10282	2,57785	0,637966	2,2772	-1,78431	2,48129	1,16735	1,31838	1,12573
0,4347	0,206362	0,88056	-1,44736	1,44658	NaN	1,27565	1,14463	0,638015	0,722215
-1,00304	1,70068	0,0103073	1,85605	0,536318	3,08589	1,76956	0,0712662	-0,0913038	0,124194
-0,776855	0,171377	NaN	NaN	-2,21962	-1,46138	-0,851522	-1,1321	0,609534	0,485497
NaN	NaN	NaN	NaN	NaN	NaN	NaN	-2,16081	-1,79731	-1,12765
0,99091	0,436853	0,182871	-0,426659	NaN	0,657068	-0,262211	-0,0486259	0,22676	-0,0963297
-2,38713	NaN	-1,09006	-2,21527	NaN	NaN	-1,97444	-1,93106	-2,27115	-2,21536
-0,807663	-1,48746	-1,93913	-2,63085	NaN	-0,861069	-0,87859	-0,412239	-0,034894	-0,0630312
-1,44071	0,22341	-2,2411	-2,08236	NaN	-3,54113	-0,49572	-0,621975	-0,433414	0,186826
NaN	NaN	NaN	NaN	0,117407	NaN	-0,248766	-1,3341	-0,782453	-3,25982
-0,255671	-1,28223	0,149721	-0,655104	-0,257185	NaN	-1,28177	NaN	NaN	-1,31896
-1,63036	NaN	-2,08646	0,127403	NaN	1,2732	-0,651949	NaN	NaN	NaN
NaN	NaN	NaN	2,4328	-2,48167	0,906628	1,4315	NaN	NaN	NaN
NaN	NaN	NaN	-0,751987	NaN	-1,28876	-2,37329	NaN	NaN	NaN

Polito et al. Universal ready-to-use immunotherapeutic approach for the treatment of cancer: expanded and activated polyclonal γδ memory T cells.
Phosphorylation sites

NaN	NaN	NaN	0,795593	NaN	0,117109	-0,373812	NaN	NaN	NaN
NaN	NaN	NaN	4,56422	-1,77327	5,29919	2,47093	NaN	NaN	NaN
NaN	NaN	-0,258696	2,66911	NaN	-0,189379	0,708986	NaN	NaN	NaN
NaN	NaN	NaN	-0,463545	NaN	0,232067	NaN	NaN	NaN	NaN
NaN	NaN	NaN	-0,792168	NaN	-0,699158	-1,91576	NaN	NaN	NaN
NaN	NaN	NaN	0,547667	NaN	1,12784	-0,933529	NaN	NaN	NaN
NaN	NaN	NaN	NaN	NaN	NaN	NaN	NaN	NaN	NaN
NaN	NaN	NaN	NaN	NaN	NaN	NaN	NaN	NaN	NaN
NaN	NaN	NaN	NaN	NaN	NaN	NaN	NaN	NaN	NaN
NaN	NaN	NaN	-0,547726	-2,85931	-0,894138	-0,0574894	-2,24368	-2,71307	-2,23835
2,07712	2,84124	0,836607	1,56548	1,54027	3,91752	1,01273	2,05533	1,18754	1,52589
2,36116	-1,63952	1,69688	4,1054	3,03543	4,68751	2,78449	3,02225	2,6596	3,0127
NaN	NaN	NaN	NaN	NaN	NaN	NaN	NaN	NaN	NaN
1,64787	2,20234	2,06911	1,5585	1,69502	1,97284	1,75208	1,906	2,04448	2,00583
NaN	NaN	NaN	NaN	NaN	NaN	NaN	NaN	NaN	NaN
NaN	-0,93549	-0,0210304	-1,96339	0,891762	-2,31106	-0,315529	NaN	-0,469705	NaN
NaN	NaN	NaN	NaN	NaN	NaN	NaN	NaN	NaN	NaN
NaN	NaN	NaN	-2,06818	NaN	-1,69617	-2,36901	NaN	NaN	NaN
-0,113569	NaN	-0,292616	-0,0462856	-0,721815	NaN	0,422239	0,202347	-0,353757	-0,0257349
NaN	NaN	NaN	-0,621656	NaN	NaN	-1,54688	NaN	NaN	NaN
NaN	0,366337	NaN	-0,0282516	0,146929	0,339201	0,211304	0,707184	0,229316	0,546823
-0,8403	0,747875	-1,04957	NaN	-0,996735	NaN	NaN	-0,516846	-0,722444	-1,11771
NaN	-0,316956	-1,24507	-3,26325	NaN	-0,436859	0,565027	NaN	-0,814736	NaN
NaN	NaN	NaN	NaN	NaN	NaN	NaN	NaN	NaN	NaN
NaN	NaN	NaN	NaN	NaN	NaN	NaN	-3,61894	-3,6354	-2,95044
NaN	NaN	-1,35631	-2,76574	NaN	-0,990587	-1,99853	-0,431183	NaN	-1,39598
NaN	NaN	NaN	NaN	NaN	NaN	NaN	NaN	NaN	NaN
NaN	NaN	NaN	NaN	NaN	NaN	NaN	NaN	NaN	NaN
NaN	NaN	NaN	1,66396	NaN	2,5988	1,65232	NaN	NaN	NaN
1,35363	3,07227	NaN	NaN	-0,437801	NaN	NaN	4,11133	3,3232	3,24968
2,19206	0,621029	3,40977	1,87632	2,92629	1,662	1,11975	3,4076	3,14237	3,13973
1,55095	-0,409891	2,03004	NaN	NaN	NaN	NaN	2,75283	2,53619	2,81547
2,13289	2,88671	2,12114	4,45632	3,42482	4,33077	4,15477	2,9888	2,65045	3,00828
NaN	NaN	NaN	NaN	NaN	NaN	-1,50049	NaN	NaN	-2,02625

NaN	NaN	NaN	NaN	NaN	NaN	NaN	-0,640402	-0,858912	-1,1735
NaN	-2,99952	-2,07382	-0,659395	NaN	-0,467865	-0,552763	NaN	NaN	NaN
-0,583914	0,526859	0,109303	0,389811	1,12608	0,403397	-0,809088	-0,285351	0,713372	0,174129
2,92933	1,48512	3,55304	2,13254	2,14375	3,42203	1,45687	3,73096	3,14997	2,34507
0,959345	0,74416	4,68747	3,45857	NaN	1,06279	NaN	NaN	3,6488	4,58709
-1,72738	2,19399	1,06653	1,44871	2,94366	-1,76736	2,4206	-0,284742	0,0888414	1,83496
NaN	-2,034	NaN	NaN	-1,425	NaN	-2,16583	NaN	NaN	NaN
-0,810244	NaN	0,802378	-0,775583	1,13918	NaN	-0,435246	0,00704193	-0,297046	0,897155
NaN	NaN	NaN	-0,835236	-1,98859	-2,72309	NaN	NaN	NaN	NaN
-1,03864	-0,730215	-0,733465	-1,31492	-1,27937	-1,34141	-1,68405	-0,215397	-0,569318	-0,79935
NaN	NaN	0,936785	NaN	NaN	NaN	NaN	1,28543	2,2104	1,58108
-3,13699	NaN	-2,57733	-4,63429	NaN	NaN	-1,79088	-1,25203	-1,05027	-0,902947
-1,91978	NaN	-1,45944	-1,61562	-1,9352	NaN	-1,05782	-1,4334	-1,99756	-2,40058
-0,286482	0,0907269	1,00544	-1,26711	0,117722	NaN	-0,00777435	1,65377	1,3064	1,24613
4,45974	4,98469	4,21806	3,47911	3,71219	4,24899	3,83272	4,31741	4,56666	4,2996
-1,69863	-1,41037	0,616903	NaN	-0,291676	NaN	NaN	NaN	NaN	NaN
NaN	-2,69493	NaN	NaN	-2,03084	NaN	-2,7685	-2,44178	-2,53861	-2,18906
-0,238794	-0,588457	-0,358049	0,0638962	-0,505209	0,238277	0,603409	-0,203688	-0,190791	-0,208688
-2,8997	-2,56887	-0,897196	-1,7742	NaN	NaN	-0,711704	NaN	NaN	NaN
NaN	NaN	NaN	-0,365322	NaN	0,748281	-0,665886	NaN	NaN	NaN
4,89974	4,44119	4,25072	4,57282	3,38757	2,49799	3,81553	3,93999	3,77104	4,03241
NaN	-3,16319	-0,390886	-0,444359	NaN	1,94911	-0,590862	NaN	NaN	NaN
NaN	NaN	NaN	NaN	NaN	NaN	NaN	NaN	NaN	NaN
-0,95595	-0,114014	-0,0223217	NaN	0,473877	NaN	NaN	0,109915	0,535058	1,97394
NaN	0,462856	1,67348	NaN	1,45261	NaN	NaN	2,97107	3,01492	3,61467
-0,576706	-0,885565	1,49406	NaN	2,37625	NaN	NaN	2,71298	2,45578	3,10021
0,418175	-0,106749	0,463795	1,78704	0,299734	1,55122	1,80625	-0,17243	0,057435	-0,143104
NaN	NaN	NaN	NaN	NaN	NaN	NaN	NaN	NaN	NaN
NaN	NaN	NaN	NaN	NaN	NaN	NaN	-1,7212	-2,21933	-2,23651
-0,401737	-1,69655	0,0830498	NaN	NaN	NaN	NaN	-0,212648	-0,310082	-0,561143
NaN	-0,496468	-0,892546	NaN	-0,380123	NaN	NaN	NaN	NaN	NaN
NaN	NaN	NaN	NaN	NaN	NaN	NaN	NaN	NaN	NaN
0,221613	-0,464088	NaN	0,375711	0,832932	1,02648	1,16529	NaN	NaN	NaN
NaN	NaN	NaN	NaN	NaN	NaN	NaN	NaN	NaN	NaN

Polito et al. Universal ready-to-use immunotherapeutic approach for the treatment of cancer: expanded and activated polyclonal γδ memory T cells.
Phosphorylation sites

NaN	NaN	-0,223227	1,9946	NaN	0,932602	1,96556	-2,21861	-3,23594	-3,45549
0,303074	-0,179123	0,378595	1,45086	1,45604	0,455013	1,3038	-0,0125751	0,15874	0,374373
0,787022	1,54026	NaN	2,29163	NaN	3,25233	1,2296	-2,59706	NaN	-0,502326
3,13693	2,73621	3,29038	2,20159	3,3584	2,17071	2,00211	NaN	2,90761	2,82893
-1,74553	-2,66805	-1,33594	NaN	-1,98116	-1,72765	-2,06336	-1,62029	-1,55883	-1,87339
0,308491	NaN	NaN	1,52178	0,588455	2,27459	NaN	1,65274	2,24279	1,60986
1,32532	0,199596	2,91343	3,56269	-0,690643	-0,989779	3,14573	1,3148	1,21906	1,69667
1,32532	0,199596	NaN	NaN	2,84366	NaN	NaN	-1,86879	-2,08092	NaN
1,54668	1,59564	1,6088	1,97075	2,93977	1,94473	1,82334	1,39163	0,677148	2,60044
1,22366	1,49499	1,5923	1,34644	1,99527	0,969419	1,22177	1,81529	1,32576	2,35466
NaN	NaN	5,95686	5,10123	-2,0039	4,24691	5,71676	-0,737116	-0,626428	-0,252004
NaN	NaN	NaN	NaN	-3,29902	NaN	NaN	-2,14198	-2,4832	-2,3029
1,43991	-0,328802	0,469378	-0,848217	0,504805	-0,524311	1,04124	0,57127	0,0754728	0,134078
1,96124	2,56329	1,78212	NaN	2,06556	1,42248	NaN	NaN	NaN	NaN
-0,321505	-0,535856	-0,300896	-1,3235	0,242819	NaN	-1,4328	-1,01009	-1,33812	-0,970639
NaN	NaN	NaN	-0,404007	-2,36478	-0,683271	0,090744	NaN	NaN	NaN
2,59842	2,38253	0,859219	1,46751	2,79869	1,13996	1,73083	2,52588	2,13214	2,33683
2,54281	2,30316	2,24869	1,96468	0,519258	2,21066	-2,1395	0,251217	3,05535	1,34276
-1,01377	-0,5114	-1,08776	NaN	NaN	NaN	NaN	NaN	NaN	NaN
-0,321114	-0,0302086	0,0321751	0,320858	0,351593	NaN	0,471985	0,134346	0,0757074	0,0650072
NaN	NaN	NaN	3,84384	NaN	3,77213	0,888102	NaN	NaN	NaN
0,255814	-1,27419	0,834232	-0,544746	-1,02377	-0,39324	0,303967	2,12016	1,87787	0,0784807
-0,107496	-1,46432	0,996223	6,31281	-0,15827	6,15707	5,34931	0,482155	0,288159	-1,02149
8,68096	8,28585	8,42042	5,5781	7,63355	5,20704	6,11805	8,46939	7,88963	7,98155
6,24026	4,73271	6,3861	3,4612	6,01453	2,47558	4,10525	5,15923	4,68604	6,58812
-0,580038	-0,260498	-0,0402565	-0,690948	0,291674	-0,624929	0,393538	0,469818	0,236279	0,503951
-0,208975	-2,7079	2,42501	4,7318	-1,56621	6,22136	4,06389	-1,42387	-2,29843	-2,1942
-1,48189	-1,12512	0,874098	2,59912	-1,13989	4,60013	1,61472	-1,69965	NaN	-1,14516
NaN	NaN	-2,68867	-0,372086	NaN	0,636711	-1,65758	-1,99147	-2,12578	NaN
1,19353	-0,808922	1,98716	5,65018	-0,909605	0,151501	3,23334	0,479797	0,455901	0,864629
1,06586	0,989305	2,47767	3,73318	NaN	NaN	2,47793	1,63237	1,20748	1,71223
1,00299	0,904463	0,790295	-0,111397	1,98848	-0,220875	0,482132	2,60155	2,45563	1,21414
4,33069	5,39932	5,34984	1,82406	5,77366	0,815941	2,08166	5,46458	5,08708	5,72841
-3,98945	NaN	0,758196	-1,9046	-1,24049	-2,00338	0,364115	-0,310123	-0,0528002	-0,612393

Polito et al. Universal ready-to-use immunotherapeutic approach for the treatment of cancer: expanded and activated polyclonal γδ memory T cells.
Phosphorylation sites

NaN	NaN	NaN	-1,80791	NaN	-1,60396	-1,79475	NaN	NaN	NaN
NaN	-2,11949	NaN	-0,503326	-1,35053	-2,8219	-0,486423	NaN	-2,80009	-1,73144
3,48841	3,92153	NaN	2,3844	4,22968	-2,75388	3,25527	4,50177	4,13182	4,87482
NaN	NaN	1,33931	2,3844	1,30899	NaN	NaN	1,49444	1,05513	1,49764
-1,69863	-1,90372	NaN	-0,748802	NaN	-1,17294	-0,580265	-1,33027	NaN	-1,99234
1,71202	1,8325	1,47764	1,523	2,32207	1,55383	1,79599	NaN	-0,726815	NaN
NaN	NaN	NaN	-1,82957	-1,15001	NaN	-1,32261	NaN	NaN	NaN
NaN	NaN	NaN	-0,0814648	-1,56511	-1,00305	-1,8012	-0,948429	-1,59908	0,217326
NaN	0,450825	NaN	1,26977	-0,246553	NaN	-0,051918	NaN	NaN	NaN
-1,36989	-1,33628	NaN	-1,12615	-2,62605	NaN	0,578901	-1,29129	-2,00166	-2,11363
0,324675	-0,274279	0,0185127	-0,968634	1,02785	-0,580189	-0,782475	-1,20619	-0,466102	-0,90239
NaN	NaN	NaN	-0,0305595	-2,9529	-2,74365	-0,236814	NaN	NaN	NaN
1,0633	0,783247	0,575686	2,0336	0,285971	3,03263	0,974342	0,00555038	-0,104451	-0,081708
NaN	NaN	NaN	NaN	NaN	NaN	NaN	NaN	NaN	NaN
NaN	NaN	NaN	-1,70562	-3,39052	-2,41204	-0,870438	NaN	NaN	NaN
-1,7064	NaN	1,63667	1,87346	NaN	1,50649	1,53868	-0,81765	0,211679	-0,669467
NaN	NaN	NaN	2,52836	2,39085	1,85671	2,14219	NaN	NaN	NaN
0,847193	0,0549583	0,856529	0,40835	1,18266	0,511587	-0,972332	0,57506	0,0201826	-0,00991344
NaN	NaN	NaN	0,105291	NaN	0,477221	-0,80991	NaN	NaN	NaN
1,48757	1,73581	1,55765	1,24888	1,85926	-1,14445	2,14931	2,05299	1,62949	1,63254
0,613369	1,3465	1,07309	0,364189	0,0935955	NaN	0,493622	0,209784	0,606708	-0,54107
NaN	NaN	NaN	-1,12956	-2,78934	-1,36554	-0,962196	NaN	NaN	NaN
NaN	NaN	NaN	0,083147	NaN	NaN	NaN	NaN	-1,11716	NaN
-1,58525	-1,50814	1,4254	1,86294	0,438711	1,84221	0,944695	-0,172487	-0,444339	-0,0923605
NaN	NaN	NaN	-1,50473	NaN	-0,541145	-1,63235	NaN	-2,47007	NaN
-0,912575	-0,279999	-0,436483	-1,60288	-0,245575	0,715014	-1,03474	-0,853029	-0,397553	-0,605847
2,17437	1,09751	2,25891	2,32014	0,225292	1,03153	2,05157	1,87465	1,32018	0,545487
0,828461	1,08119	1,27516	NaN	NaN	NaN	NaN	NaN	1,79028	NaN
1,24886	1,46382	1,37709	1,09655	1,07355	1,75983	1,11046	1,53167	1,93342	1,14858
0,14893	NaN	-0,304373	-1,05331	-0,436686	NaN	-0,442789	-0,8013	-0,826653	-0,958013
NaN	NaN	NaN	-4,07209	NaN	-3,54812	-0,332254	NaN	NaN	NaN
NaN	-0,301342	NaN	1,14897	-0,47415	NaN	NaN	0,46426	0,369658	0,623231
NaN	NaN	NaN	NaN	NaN	NaN	-2,48158	NaN	NaN	NaN
1,63858	1,5944	0,65078	0,519859	0,55085	-1,51079	0,339724	0,537952	0,579381	0,0404043

Polito et al. Universal ready-to-use immunotherapeutic approach for the treatment of cancer: expanded and activated polyclonal γδ memory T cells.
Phosphorylation sites

NaN	NaN	NaN	NaN	NaN	NaN	NaN	NaN	NaN	NaN
NaN	NaN	NaN	NaN	NaN	NaN	NaN	NaN	NaN	NaN
-3,836	-2,45558	NaN	NaN	NaN	NaN	NaN	NaN	NaN	NaN
-2,32855	NaN	0,0822678	-0,167149	-1,84429	1,04982	-0,0449619	-1,15369	-1,07123	-1,56399
1,39368	0,415754	2,30541	1,90855	0,737864	2,61963	2,0621	NaN	NaN	NaN
-2,21611	NaN	-2,02745	NaN	-1,63589	-2,73668	-0,52433	NaN	NaN	NaN
NaN	-0,444092	NaN	-1,24991	NaN	-1,99094	NaN	-0,59313	-0,955194	-0,844625
2,12631	1,64319	3,46861	3,21174	2,86115	3,0159	3,08498	3,19893	3,17144	2,56593
-1,70407	NaN	-0,919193	-0,743992	-1,78616	-0,414726	0,464052	-2,0691	-1,74145	-1,54512
NaN	NaN	-2,68812	-0,781858	NaN	NaN	-1,47697	-2,31102	-2,35512	-1,92169
NaN	NaN	NaN	-0,202629	NaN	-0,295242	-0,0778408	NaN	NaN	NaN
NaN	NaN	NaN	-1,49372	NaN	NaN	-2,82666	NaN	NaN	NaN
1,99886	1,44133	0,939329	2,59462	1,61551	2,32955	1,33355	2,26824	2,16338	2,23487
-0,499041	0,221941	-1,17256	2,56053	-0,112734	2,58829	0,579679	-0,499886	-0,495004	-0,504375
-0,396381	0,231127	-0,0803928	NaN	NaN	NaN	NaN	-0,37534	-0,39642	-0,647407
NaN	NaN	NaN	NaN	NaN	NaN	NaN	NaN	NaN	NaN
-1,00864	0,535267	0,300631	2,28263	-0,988701	2,60563	1,26797	-1,31778	-0,337729	-0,300527
0,506769	-0,402582	1,24068	2,08654	0,792135	1,34907	1,60483	0,0903225	0,147323	0,221959
-1,76786	-1,63255	-0,794067	2,63652	-0,963531	0,991165	1,66167	-2,14959	-2,67341	-2,1803
2,01827	1,57837	1,88871	3,26952	3,0866	3,65048	2,92615	1,78499	1,37538	1,5489
-0,382988	-1,42001	-1,18071	1,12414	-0,141361	1,04053	-0,713205	-1,79224	-1,68194	-1,7565
NaN	NaN	NaN	1,14049	-0,959435	1,70531	NaN	NaN	NaN	NaN
NaN	NaN	NaN	NaN	NaN	NaN	NaN	NaN	NaN	NaN
NaN	NaN	NaN	NaN	NaN	NaN	NaN	NaN	NaN	NaN
-0,631184	0,33494	-0,559757	3,11955	-1,55463	3,39004	2,27786	-0,416227	-0,520637	-0,840468
4,57716	4,12691	3,62491	5,89001	2,63248	6,96466	4,42211	4,10892	5,29846	-2,27021
NaN	NaN	NaN	NaN	NaN	NaN	NaN	-2,26678	-2,32645	-2,34893
0,00852203	-0,653522	-0,708345	NaN	1,17953	-1,26596	-1,66486	-0,0962238	0,417323	0,424121
NaN	NaN	NaN	NaN	NaN	NaN	NaN	NaN	NaN	NaN
-1,18036	NaN	-1,48449	NaN	NaN	NaN	NaN	-2,29172	NaN	NaN
-0,892588	-1,6086	-1,07677	-0,997299	-1,23466	-0,775133	0,291222	-0,984692	-1,15997	NaN
1,07849	1,02066	1,27936	1,59389	1,83381	-0,996815	1,63817	1,34801	1,3692	1,32693
-0,622087	NaN	0,739021	0,764225	0,000597	-0,0871849	0,43618	-0,0665874	-0,147351	-0,463014
NaN	-1,75172	-1,10239	-0,354141	-0,878798	-2,26334	-0,801394	-0,671885	-1,30846	-1,06481

NaN	NaN	NaN	-1,89733	NaN	-2,47774	-2,19459	NaN	NaN	NaN
NaN	NaN	NaN	1,27934	-0,271151	NaN	-0,010294	NaN	NaN	NaN
-1,69761	-2,2059	-1,02724	NaN	NaN	NaN	NaN	NaN	NaN	NaN
0,524969	0,456398	0,76395	2,25709	1,47835	2,26101	1,24279	1,09446	0,677148	0,248609
NaN	NaN	1,67919	NaN	NaN	NaN	NaN	2,11758	2,61272	1,90539
-0,662886	-0,475197	NaN	-0,232359	-0,73477	NaN	0,254957	NaN	NaN	NaN
-1,53827	NaN	-1,78641	-1,38929	-1,73378	NaN	-2,36337	-1,31822	-1,51491	-0,986056
0,13962	-1,2435	-0,962816	1,64897	0,524467	1,21555	-0,110474	0,826586	0,358151	0,758481
0,0599613	-0,462519	-1,40079	-0,768082	0,36017	-0,953518	-0,249723	-0,0098362	-0,76691	-0,281043
NaN	NaN	-0,743118	-1,79765	-2,86608	-0,540569	-0,550406	-2,37816	NaN	-1,0472
NaN	NaN	NaN	-1,25469	NaN	-2,56469	NaN	NaN	NaN	NaN
-0,19174	-1,35067	0,0979996	0,886993	0,228014	0,0877533	0,385399	-0,383387	-0,444507	-0,115767
NaN	NaN	-1,80628	-0,970074	-2,11899	-1,27715	-1,24127	NaN	NaN	NaN
0,825064	0,540203	-0,559757	0,719154	NaN	1,7588	1,07558	NaN	NaN	NaN
NaN	NaN	NaN	-2,23981	NaN	-2,87349	NaN	-2,37143	NaN	-2,39913
NaN	NaN	NaN	1,46432	-3,34682	NaN	NaN	NaN	NaN	NaN
NaN	NaN	NaN	-0,641878	-1,10828	0,381578	NaN	NaN	NaN	NaN
0,664509	0,309521	-0,535807	0,839502	NaN	0,0768871	-1,32709	0,439516	-0,142793	0,527864
NaN	NaN	NaN	NaN	NaN	NaN	NaN	NaN	NaN	NaN
-1,75846	-2,58944	-1,40286	NaN	-0,0180836	NaN	-1,42702	-0,671129	-0,625319	-1,13878
NaN	-0,507765	NaN	NaN	NaN	NaN	NaN	-0,102262	-1,12354	-0,213841
NaN	-0,302187	NaN	1,19915	NaN	2,19551	NaN	-0,986174	-1,29154	-0,380065
NaN	NaN	NaN	NaN	NaN	NaN	NaN	-1,49078	-1,42191	-2,13868
NaN	NaN	0,756315	2,20031	NaN	-0,626705	2,19912	NaN	NaN	NaN
-0,169731	0,155519	0,0505695	0,26494	-0,477947	0,850046	0,0615959	0,142256	-0,00814533	-0,162442
-0,250961	0,165554	-0,0504932	1,46844	NaN	1,83188	-0,273405	0,770168	0,326575	NaN
2,407	5,12941	3,7278	2,03446	3,36641	0,499771	2,58154	2,83048	4,31998	3,46224
1,42702	2,3231	1,48923	1,08819	2,3615	1,67008	0,63781	2,33417	2,32072	3,42181
NaN	-0,839956	NaN	NaN	NaN	NaN	NaN	-0,513565	-0,909636	0,62338
-0,773876	0,188429	-1,01476	NaN	-0,8741	NaN	NaN	NaN	NaN	NaN
NaN	NaN	-1,91479	NaN	NaN	NaN	NaN	-1,27962	-1,19213	-1,53617
-0,243042	-1,51067	2,8059	NaN	NaN	NaN	NaN	1,27598	0,775914	0,280644
0,766569	-0,43568	2,76485	NaN	0,628822	NaN	NaN	2,42234	1,86503	1,75298
NaN	NaN	-0,862251	NaN	NaN	NaN	NaN	-2,06075	-3,3297	-3,16839

Polito et al. Universal ready-to-use immunotherapeutic approach for the treatment of cancer: expanded and activated polyclonal γδ memory T cells.
Phosphorylation sites

-1,04585	0,318542	NaN	NaN	NaN	NaN	NaN	0,499796	-0,164212	0,0161238
-0,571257	-0,357815	0,446198	1,10785	0,74682	NaN	2,28943	0,243505	-0,244641	0,394223
NaN	NaN	NaN	-2,62313	NaN	-2,58431	-3,00671	NaN	NaN	NaN
3,8994	3,57871	3,20947	4,08062	4,68188	2,5157	4,39166	3,09043	2,98901	4,40854
-1,94869	-2,05787	-1,79852	-2,77125	-1,56604	NaN	-1,80128	-1,71471	-1,72366	-2,11204
0,407143	-0,529749	0,458729	NaN	0,873814	NaN	0,233181	1,21776	1,21712	0,785493
NaN	NaN	NaN	-1,32221	-0,924692	-1,50164	-1,61664	NaN	NaN	NaN
-0,658863	-0,698193	-0,260166	-1,49237	0,287394	-1,81051	-1,59325	0,0278893	-0,419879	-0,69584
NaN	NaN	NaN	-1,88634	0,717136	NaN	-1,88497	NaN	NaN	NaN
-0,658863	-0,698193	-0,260166	-1,49237	0,287394	-1,81051	-1,59325	0,0278893	-0,419879	-0,69584
NaN	NaN	NaN	NaN	NaN	NaN	NaN	-1,83726	-1,98006	-1,97934
NaN	NaN	1,17123	-0,796217	NaN	NaN	1,2276	NaN	NaN	NaN
0,710793	0,37933	0,14934	3,49811	0,782879	4,14005	3,00696	-0,839264	-0,0776911	-0,00652027
NaN	NaN	NaN	-2,01372	-1,78655	-1,87099	-1,72247	NaN	NaN	NaN
0,99102	0,715321	1,25727	-0,211519	2,29695	0,0718784	1,26071	0,995354	0,602256	0,535071
NaN	NaN	NaN	-2,88614	NaN	NaN	-0,882334	NaN	NaN	NaN
-0,540674	-1,37414	-1,59221	-0,875477	-0,794716	-0,475393	-0,635262	-1,45437	-0,59084	NaN
1,37788	1,82724	0,18742	0,804462	0,331398	1,29618	0,703918	0,0228157	0,576907	0,171298
NaN	NaN	NaN	NaN	NaN	NaN	NaN	NaN	NaN	NaN
NaN	NaN	NaN	-0,870897	NaN	-2,18889	-1,58612	NaN	NaN	NaN
NaN	NaN	NaN	-2,30872	NaN	1,41993	-4,34218	NaN	NaN	NaN
-0,515377	-0,0599918	-0,503084	-0,836187	-0,511763	0,686258	-1,75672	NaN	NaN	NaN
NaN	-0,825859	-0,804543	-2,05521	0,442963	-1,54759	NaN	NaN	NaN	NaN
NaN	NaN	0,056778	-2,27897	NaN	NaN	-0,861435	-2,44517	-2,11614	-2,09204
-1,65236	-2,28599	-1,77729	NaN	NaN	NaN	NaN	-1,08272	NaN	NaN
NaN	NaN	NaN	NaN	NaN	NaN	NaN	NaN	NaN	NaN
NaN	NaN	-2,58914	-2,64274	-4,24975	-2,21502	-2,05071	NaN	NaN	-3,14921
NaN	NaN	NaN	NaN	NaN	NaN	NaN	NaN	NaN	NaN
NaN	NaN	NaN	-1,92059	-1,98352	-2,17616	-2,10698	NaN	NaN	NaN
1,60676	2,77296	2,60431	3,19285	3,61137	0,918343	3,39753	2,53868	2,49971	3,07764
-0,397085	0,326765	NaN	0,245953	1,06393	0,610107	NaN	NaN	NaN	-0,413144
-1,24926	-2,40273	-0,741735	1,09311	-2,0666	-0,36622	0,395102	-0,887867	-0,065814	-1,77798
0,361538	-0,839705	0,350143	0,19684	0,748564	NaN	0,12254	0,682095	0,372718	-1,07294
0,384768	NaN	-0,379866	-1,41127	0,990128	-0,0833721	-0,746223	0,510132	0,0778265	-0,0114851

Polito et al. Universal ready-to-use immunotherapeutic approach for the treatment of cancer: expanded and activated polyclonal γδ memory T cells.
Phosphorylation sites

1,28951	-1,97702	-1,15801	-1,29169	-1,97098	NaN	-2,28951	-0,640129	-0,971549	NaN
NaN	NaN	NaN	NaN	NaN	NaN	NaN	NaN	NaN	NaN
NaN	NaN	NaN	-0,494184	NaN	-0,0727463	NaN	NaN	-1,30729	NaN
-1,75379	NaN	-0,94463	NaN	NaN	NaN	NaN	-1,78828	-2,35313	-4,42384
5,21202	3,34664	5,76397	2,85681	4,5635	3,88089	4,94334	6,10489	5,38674	6,20941
-1,27334	-0,879189	-1,10512	NaN	-0,965528	-0,931911	-2,21313	-0,11655	-0,717242	-0,786468
NaN	NaN	NaN	-0,115952	NaN	0,907139	-0,757929	NaN	NaN	NaN
NaN	-3,06176	-1,06218	-1,66693	-2,40372	-1,21299	-0,609972	NaN	NaN	NaN
NaN	NaN	NaN	-1,37678	-2,14919	NaN	NaN	-2,38257	NaN	-2,33045
-1,96586	NaN	NaN	-2,62607	NaN	NaN	-1,31714	-1,26456	-1,26424	-2,06692
NaN	NaN	NaN	NaN	NaN	NaN	NaN	-1,87454	-2,20886	-1,92187
-1,35657	-0,970137	-0,887047	-0,0779114	-0,0431175	-2,17713	-0,651035	NaN	NaN	NaN
NaN	NaN	NaN	NaN	NaN	0,29759	NaN	NaN	NaN	NaN
1,17891	0,00752068	-0,5082	0,291887	0,154665	1,88528	-0,223028	0,228043	-0,155551	0,679008
-1,04502	-1,74868	-1,93995	-0,717604	-0,480522	0,113569	-0,466925	0,247	-0,567887	-0,509919
-1,51781	-2,94164	-1,34879	-1,66582	-0,68195	-3,1436	NaN	-1,00828	NaN	-1,24274
-1,10921	-0,339191	-1,07471	-1,02005	NaN	NaN	-1,12013	-1,0292	NaN	-1,49451
1,88533	NaN	1,91918	NaN	2,89246	1,9223	NaN	1,87804	NaN	2,28456
NaN	-1,29193	NaN	-1,71863	-2,22665	-1,89418	-1,68678	-2,00312	-2,21171	-2,14973
NaN	NaN	NaN	NaN	NaN	NaN	NaN	-1,22426	-1,63545	-1,77233
2,14923	1,9515	1,86464	1,98089	2,37038	2,66255	2,52977	1,97925	2,18275	1,66141
NaN	0,649811	-0,227066	-0,115334	NaN	0,0260086	0,726109	-0,154552	0,0165586	-0,316012
1,08292	0,648916	0,552082	1,07149	1,15689	1,59198	1,68986	0,934011	0,739158	0,430078
NaN	-1,19113	-2,22387	NaN	-2,27345	-1,94925	-1,78401	NaN	NaN	NaN
NaN	0,221407	NaN	0,0358028	NaN	1,25891	0,879625	NaN	NaN	-0,155089
-0,690285	0,163191	0,0582447	-0,342533	-0,876186	-2,14651	-0,47588	-0,782558	-0,509236	-0,420621
NaN	NaN	NaN	0,279053	-2,03142	0,186672	-2,02602	NaN	NaN	NaN
NaN	NaN	NaN	NaN	NaN	NaN	NaN	NaN	NaN	NaN
NaN	NaN	NaN	NaN	NaN	NaN	NaN	NaN	NaN	NaN
-4,30132	NaN	-1,36962	-0,497269	NaN	NaN	0,274414	NaN	NaN	NaN
2,35048	0,40061	3,29326	5,75683	2,35039	NaN	0,596523	0,218214	0,277894	1,2472
NaN	NaN	NaN	NaN	NaN	NaN	NaN	NaN	NaN	NaN
NaN	NaN	NaN	NaN	2,52331	-0,0244122	0,547855	NaN	NaN	NaN
NaN	NaN	NaN	1,0944	0,203112	-0,655277	-0,643511	NaN	NaN	NaN

Polito et al. Universal ready-to-use immunotherapeutic approach for the treatment of cancer: expanded and activated polyclonal γδ memory T cells.
Phosphorylation sites

2,1881	1,33042	1,7347	1,63353	1,93424	1,30105	1,71076	1,62317	1,68374	1,96562
-1,20835	-1,37297	0,147158	-0,861683	-0,589975	-1,76752	0,37957	0,107672	-0,245906	-0,976724
NaN	NaN	NaN	NaN	NaN	NaN	NaN	NaN	NaN	NaN
NaN	-1,83064	NaN	0,358753	0,621025	NaN	-0,437059	2,12874	2,015	1,71608
NaN	NaN	NaN	NaN	NaN	NaN	NaN	NaN	NaN	NaN
NaN	NaN	NaN	NaN	NaN	NaN	NaN	-1,85935	-2,06007	-1,81626
NaN	NaN	NaN	-0,496412	NaN	-1,52438	-1,11204	NaN	NaN	NaN
-0,942804	-0,995537	-0,936371	NaN	NaN	NaN	NaN	NaN	NaN	NaN
-0,526459	-1,2041	-0,763016	-0,991512	0,815331	NaN	0,00444031	0,890814	0,106112	0,411252
1,40595	1,00499	0,930792	0,905758	1,13284	1,62623	2,08029	0,977983	0,47273	2,51083
NaN	NaN	NaN	NaN	NaN	NaN	NaN	NaN	NaN	NaN
NaN	NaN	NaN	1,90541	-1,68524	2,73724	0,930929	NaN	NaN	NaN
NaN	NaN	NaN	NaN	NaN	NaN	NaN	NaN	NaN	NaN
NaN	NaN	NaN	NaN	NaN	NaN	NaN	NaN	NaN	NaN
-0,896364	0,719301	-0,906504	-0,0550995	-0,122204	0,666914	0,0223312	NaN	NaN	NaN
NaN	NaN	NaN	0,919235	NaN	-1,27956	-1,59359	NaN	NaN	NaN
7,03808	7,43378	7,2726	7,99073	7,33615	4,86202	8,01469	7,23558	6,70206	6,55823
-0,797695	-0,910151	-0,59256	-1,24795	NaN	-0,667788	-0,756191	-0,532705	-0,590501	-0,752009
NaN	NaN	NaN	NaN	0,950983	-0,554266	-4,67185	NaN	NaN	NaN
NaN	NaN	NaN	NaN	NaN	NaN	NaN	NaN	NaN	NaN
NaN	NaN	NaN	NaN	NaN	NaN	NaN	NaN	NaN	NaN
NaN	NaN	NaN	0,115763	NaN	0,91186	0,385878	NaN	NaN	NaN
NaN	-1,37285	-0,557604	NaN	NaN	NaN	NaN	-0,660368	-0,854381	0,398007
NaN	NaN	NaN	0,329983	NaN	0,189827	-0,0884228	NaN	NaN	NaN
NaN	NaN	NaN	NaN	NaN	NaN	NaN	0,0385151	-0,330722	-0,488029
-1,02808	-1,80854	-0,62619	-1,44601	1,21131	-0,967861	-0,932804	-1,00392	-1,20763	-0,533675
-1,12591	-0,998583	-0,839769	-0,851696	NaN	NaN	-0,652054	-1,65647	-2,70449	-0,105756
0,181023	-0,361233	1,10271	1,72689	1,54904	0,462511	2,10304	0,362299	0,238454	0,625628
0,878124	0,828074	0,380877	-1,50218	1,28888	-1,5708	0,0686893	0,0262241	0,275271	0,538806
NaN	NaN	-2,22001	NaN	NaN	NaN	NaN	NaN	NaN	NaN
NaN	NaN	NaN	NaN	NaN	NaN	NaN	NaN	NaN	NaN
NaN	NaN	NaN	0,124287	NaN	1,29168	-1,62958	NaN	NaN	NaN
5,84177	5,23308	5,62377	1,78747	6,32796	7,3984	7,94285	5,99987	5,80665	5,73437
-0,422289	0,844303	0,980947	0,191458	2,14883	NaN	1,85439	0,566294	0,454211	0,972301

Polito et al. Universal ready-to-use immunotherapeutic approach for the treatment of cancer: expanded and activated polyclonal γδ memory T cells.
Phosphorylation sites

4,27656	4,76687	4,34656	4,35603	5,25891	2,12268	4,7131	4,88955	5,7666	5,06958
-2,79174	-3,78187	NaN	-2,40162	-3,03883	-1,54343	-1,8825	NaN	NaN	NaN
NaN	NaN	NaN	-2,54681	-1,92826	NaN	-2,72945	NaN	NaN	NaN
0,295765	0,0504684	0,404032	-1,93429	-0,297955	NaN	NaN	0,00448227	-0,0681353	-0,920911
-2,81444	NaN	-2,5458	-1,7219	-1,83513	-1,92771	-1,69276	-2,39197	-2,07296	NaN
-1,3604	-0,763945	-0,683826	-2,09448	0,33386	-1,74699	-2,07424	NaN	NaN	NaN
NaN	NaN	NaN	NaN	NaN	NaN	NaN	NaN	NaN	NaN
0,183815	0,297657	0,253027	-1,15947	0,73539	NaN	-0,464735	NaN	NaN	NaN
NaN	NaN	NaN	-0,691067	NaN	0,403397	-1,03584	NaN	NaN	NaN
-1,64808	-1,92611	-1,60402	-3,42311	-0,218195	NaN	NaN	0,226978	-0,249703	-0,509561
-0,208897	-1,0311	NaN	0,575071	-0,290388	-0,436844	-1,43939	0,318768	0,479129	0,217662
2,95175	1,35402	2,36134	2,65236	-1,72595	6,17076	-3,24041	2,86119	3,09235	1,69426
NaN	NaN	NaN	NaN	NaN	NaN	NaN	NaN	NaN	NaN
-1,28384	NaN	0,650105	0,457638	NaN	0,810486	0,0738792	NaN	NaN	NaN
NaN	NaN	-0,533104	-0,494055	NaN	-3,5906	-0,770035	NaN	-0,801879	-1,055
NaN	0,424833	NaN	0,551434	0,335144	1,18757	NaN	NaN	NaN	NaN
-0,694757	-1,13107	-1,22635	NaN	0,0886574	NaN	NaN	-1,38759	-1,21747	-1,28882
2,56656	3,2917	3,602	2,6587	4,82417	-0,833879	4,27819	2,30736	2,69819	3,32501
NaN	-0,372524	NaN	0,236481	-0,493193	NaN	-0,0241337	NaN	NaN	-1,18729
NaN	0,248672	NaN	-1,11193	0,157166	NaN	-0,359406	NaN	NaN	NaN
NaN	NaN	NaN	-2,72883	-1,01042	-2,10628	-1,93187	-1,13166	-1,81481	-1,42342
NaN	NaN	NaN	NaN	NaN	NaN	NaN	-0,431721	-2,24069	-2,39272
NaN	NaN	NaN	NaN	NaN	NaN	NaN	NaN	NaN	NaN
-0,367729	0,408848	0,193995	-0,704454	1,04352	-2,27559	-0,293859	0,301424	0,316861	0,613469
-2,74302	-1,71035	NaN	-2,74871	-2,17002	-2,52331	-1,87756	-1,44565	-0,928561	-1,30256
-1,22741	-0,275391	-0,0867481	0,355309	-0,66851	0,332935	-0,0868568	0,196524	0,771836	0,333869
NaN	NaN	NaN	-1,93226	NaN	NaN	-2,00344	-0,700541	-1,64247	-1,5128
-1,17109	-1,33636	0,0093174	-2,52084	-0,198193	-0,885302	-0,323755	0,628683	0,433282	0,511954
1,58155	2,57563	1,30404	1,49249	2,70617	0,585163	2,38263	-0,575516	-0,245687	-0,319932
-0,367729	0,408848	0,193995	-0,704454	1,04352	-2,27559	-0,293859	0,301424	0,316861	0,613469
-0,367729	0,408848	0,193995	-0,704454	1,04352	-2,27559	-0,293859	0,301424	0,316861	0,613469
NaN	NaN	NaN	-0,722691	-2,74832	-0,738344	-1,18414	NaN	NaN	NaN
NaN	NaN	NaN	NaN	NaN	NaN	NaN	NaN	NaN	NaN
-2,74302	-1,71035	NaN	-2,74871	-2,17002	-2,52331	-1,87756	-1,44565	-0,928561	-1,30256

-1,22741	-0,275391	-0,0867481	0,355309	-0,66851	0,332935	-0,0868568	0,196524	0,771836	0,333869
4,37955	5,81913	4,42434	4,42893	5,04185	3,10746	5,56872	4,05368	3,83818	5,24219
-1,43099	-1,20795	-0,993929	NaN	NaN	NaN	NaN	-1,13363	NaN	0,71943
NaN	NaN	NaN	NaN	NaN	NaN	NaN	0,628296	0,610475	0,372512
-2,89171	NaN	-0,746563	NaN	NaN	NaN	NaN	1,3161	0,503985	0,110356
-0,243702	-1,14358	3,35191	NaN	0,737743	-3,91421	-0,245293	1,82405	1,9636	1,84208
NaN	NaN	NaN	NaN	NaN	NaN	NaN	NaN	NaN	NaN
NaN	NaN	NaN	NaN	NaN	NaN	NaN	NaN	NaN	NaN
NaN	NaN	NaN	-2,09864	NaN	-0,398191	-1,05931	NaN	NaN	NaN
3,57345	3,54774	3,77311	1,6127	1,9831	0,900991	2,04021	1,96826	2,51794	1,69724
1,25121	2,4596	1,97152	NaN	1,67984	-2,02234	NaN	NaN	NaN	NaN
NaN	0,815294	NaN	0,184811	0,804464	-2,07828	NaN	NaN	NaN	NaN
-1,75093	-1,3018	-0,600412	-0,781796	-1,47328	-1,62358	-1,27316	-1,23162	-1,51984	-1,37375
0,374079	0,364161	0,440882	0,867682	0,888233	NaN	0,919041	0,414919	0,423223	0,219201
NaN	-3,00714	NaN	-2,08946	-1,74689	-2,90842	NaN	NaN	NaN	NaN
NaN	NaN	NaN	NaN	NaN	NaN	NaN	NaN	NaN	NaN
-1,69674	-1,5515	-1,26159	-1,25543	NaN	-1,45907	-2,75619	NaN	-1,76287	-2,35045
-2,29235	-1,34464	-1,76226	0,221455	-0,426943	-2,76451	0,107788	-0,765879	-0,795671	-1,23122
NaN	NaN	NaN	-0,466032	-2,7473	-1,60396	NaN	NaN	NaN	NaN
-0,635061	-0,112211	-1,88795	-0,901302	-2,96551	NaN	-2,11519	-0,752161	-0,1914	-1,39719
NaN	NaN	NaN	-2,5702	-2,49965	-0,694477	NaN	NaN	NaN	NaN
-0,985565	-2,62091	-1,43643	-1,24808	NaN	NaN	-1,11301	NaN	NaN	NaN
NaN	NaN	NaN	NaN	NaN	NaN	NaN	-0,154247	-0,252315	0,355412
-0,312746	0,296389	0,0975437	-2,20312	-0,266422	-1,0212	-1,06654	-0,0908489	0,00591564	0,107591
-2,2185	NaN	-1,16517	5,35049	-1,71676	3,92298	4,82959	NaN	NaN	NaN
NaN	0,0127182	NaN	-1,10761	NaN	NaN	-0,288364	0,420298	0,98878	0,676425
NaN	NaN	NaN	-1,43872	NaN	-2,0762	-0,960629	NaN	NaN	NaN
NaN	NaN	NaN	-0,216764	0,478027	-0,195129	0,825672	NaN	NaN	NaN
NaN	NaN	NaN	-0,475584	NaN	NaN	0,625696	NaN	NaN	NaN
-1,4622	-0,910765	NaN	-1,83101	NaN	-0,544743	-1,68211	NaN	NaN	NaN
-0,791386	-0,635817	-1,05278	-1,2268	-0,185883	-1,3429	-0,411777	-0,735579	-1,26478	-1,39363
NaN	NaN	NaN	-2,17919	-1,99356	-2,1799	-2,95012	NaN	NaN	NaN
NaN	NaN	NaN	NaN	NaN	NaN	NaN	NaN	NaN	NaN
NaN	NaN	NaN	NaN	NaN	NaN	NaN	NaN	NaN	NaN

Polito et al. Universal ready-to-use immunotherapeutic approach for the treatment of cancer: expanded and activated polyclonal γδ memory T cells.
Phosphorylation sites

-2,89715	NaN	-1,89282	-1,8513	-2,0654	-1,78048	-1,62965	NaN	NaN	NaN
NaN	NaN	NaN	NaN	-2,87175	-2,67065	NaN	NaN	NaN	NaN
1,00443	0,391851	-2,93303	-0,40391	-0,0115051	0,526972	NaN	0,959633	0,8301	0,630467
-3,80903	-1,63371	-3,14139	NaN	NaN	NaN	NaN	-3,94252	NaN	-2,40556
0,617289	0,0104923	-0,737558	NaN	NaN	NaN	NaN	NaN	NaN	NaN
NaN	NaN	NaN	2,94865	NaN	NaN	NaN	0,425413	0,691951	-2,45654
NaN	NaN	0,872593	-2,05483	NaN	NaN	0,849928	-0,820068	-0,680621	-0,401366
2,58382	1,66315	3,49804	2,76731	1,69581	2,38415	2,36854	1,92682	2,85815	2,32776
1,23205	-0,436882	2,25736	1,82816	1,87764	2,37453	2,14442	0,3727	-0,0640345	NaN
4,4959	4,60615	4,67151	3,83046	3,53118	3,67855	5,02694	6,01229	5,78364	5,77358
5,38629	5,61562	6,1191	4,73325	5,13371	4,26653	4,75583	6,52939	6,32705	6,1358
1,63413	0,669729	2,55506	2,72345	1,55824	3,03898	2,31161	2,63579	2,41625	2,46514
-0,2798	-1,42401	0,612612	0,765739	-0,421488	NaN	-0,282372	-0,239105	-0,00203228	-0,279651
3,58826	2,70465	5,98942	4,87918	2,39572	0,730047	4,99668	3,95271	3,76885	3,68376
1,31234	0,166803	2,29495	2,5075	1,99656	1,70997	2,7252	2,87336	2,40216	2,29946
3,74336	4,30691	4,30373	4,9716	4,4037	4,87849	4,68009	4,00679	3,59876	4,91485
NaN	NaN	NaN	-3,401	NaN	2,87228	-4,36645	NaN	NaN	NaN
-3,58053	NaN	-2,16384	-2,02715	NaN	-0,835054	-2,30942	NaN	NaN	NaN
-1,18614	-0,864082	NaN	-1,96031	-0,828903	NaN	-0,696053	-0,655605	-0,972274	NaN
-2,10805	NaN	-0,197889	3,56656	NaN	NaN	2,71747	NaN	NaN	NaN
NaN	NaN	NaN	NaN	NaN	NaN	NaN	NaN	NaN	NaN
NaN	NaN	NaN	NaN	NaN	NaN	NaN	NaN	NaN	NaN
NaN	NaN	NaN	-1,0145	NaN	-1,1518	-1,38066	NaN	NaN	NaN
NaN	NaN	NaN	NaN	NaN	NaN	NaN	NaN	NaN	NaN
NaN	NaN	NaN	NaN	NaN	NaN	NaN	NaN	NaN	NaN
NaN	NaN	NaN	NaN	NaN	NaN	NaN	NaN	NaN	NaN
NaN	NaN	NaN	NaN	NaN	NaN	NaN	NaN	NaN	NaN
NaN	NaN	NaN	-3,88728	NaN	NaN	-2,25603	NaN	NaN	NaN
1,42053	0,728355	0,86307	2,37953	1,71568	1,13942	1,30986	1,5095	1,16622	0,933295
-2,4887	-3,16907	-1,94925	NaN	NaN	NaN	NaN	-2,29257	-2,64081	-2,53351
NaN	NaN	NaN	NaN	NaN	NaN	NaN	NaN	NaN	NaN
NaN	NaN	NaN	NaN	NaN	NaN	NaN	NaN	NaN	NaN
2,83848	1,61672	1,57041	1,81698	NaN	-0,321793	1,5812	1,67635	1,40418	1,39291
-0,464012	-0,219055	-0,336637	0,848003	0,278324	0,98361	1,21524	-0,737947	-1,32851	-0,951074

Polito et al. Universal ready-to-use immunotherapeutic approach for the treatment of cancer: expanded and activated polyclonal γδ memory T cells.
Phosphorylation sites

NaN	NaN	NaN	-0,237183	-2,40166	0,236855	-0,376856	NaN	NaN	NaN
NaN	NaN	NaN	0,0271149	NaN	-1,22048	-1,04038	NaN	NaN	NaN
NaN	NaN	-2,03438	-1,07828	NaN	-3,11736	-1,00654	NaN	NaN	NaN
NaN	NaN	NaN	-0,505648	NaN	NaN	NaN	NaN	NaN	NaN
NaN	NaN	-1,38897	-0,450449	-1,28345	0,177164	-0,750353	-1,45697	NaN	-1,61524
0,544905	-0,00169945	0,982342	2,38763	0,992846	-0,00829124	1,95844	0,215294	0,0166998	-0,0698328
-0,543957	NaN	-1,60608	0,36319	-0,130148	-0,309221	-0,471752	NaN	NaN	NaN
NaN	NaN	NaN	-1,99426	-2,1018	-0,385424	-1,52916	-1,72994	-0,814124	-1,51765
-1,47977	-2,05813	-1,39844	-2,64469	-1,55689	NaN	-2,05833	NaN	NaN	-1,47269
NaN	NaN	-1,39844	NaN	NaN	NaN	NaN	-0,328798	-0,259049	-1,47269
NaN	NaN	NaN	NaN	NaN	NaN	NaN	1,29227	0,756015	0,555984
1,32059	-0,108091	1,42501	0,44581	0,668505	3,99254	0,621227	1,58169	1,7198	0,569316
NaN	NaN	NaN	NaN	NaN	NaN	NaN	NaN	NaN	NaN
NaN	NaN	NaN	NaN	NaN	NaN	NaN	NaN	NaN	NaN
1,87222	2,81099	2,44617	3,04866	2,58895	1,26151	3,5561	0,0305042	0,379718	1,11845
1,06778	1,48323	2,09385	1,9568	2,66525	0,468414	2,14472	0,993851	0,833903	1,55461
3,3387	2,16486	4,56199	3,30648	4,27792	0,479029	3,75883	4,8756	4,73075	4,01605
1,68479	1,23265	1,24981	1,12473	0,00147629	-1,64989	0,578917	0,869947	-0,714009	0,367007
3,37923	2,95921	2,58023	3,54023	2,56128	3,45037	2,42194	2,70321	3,19467	2,08114
-2,07297	-1,51957	0,505915	-0,269012	-0,636511	NaN	-0,0268402	-0,276623	-0,323365	-0,783069
2,25594	2,11344	2,91812	2,38939	3,38822	-0,345837	1,91585	2,95337	3,34069	2,7916
-2,52382	-2,36267	-1,78904	NaN	NaN	NaN	NaN	NaN	NaN	NaN
NaN	NaN	NaN	-1,71573	NaN	NaN	-1,80003	NaN	NaN	NaN
-1,10238	-0,887119	-0,61927	0,659075	-0,549381	1,20493	0,273083	-0,674908	-0,496102	-1,04839
NaN	NaN	NaN	NaN	-3,57189	-2,27437	-2,94595	NaN	NaN	NaN
NaN	NaN	-1,05062	-0,869385	-0,84623	-0,187468	-0,39543	-0,817122	-0,907134	-1,16591
NaN	NaN	NaN	NaN	NaN	NaN	NaN	NaN	NaN	NaN
1,51258	0,314039	2,13636	3,05396	0,155277	4,3958	1,0485	1,21088	1,04572	0,275193
NaN	NaN	-1,96786	-1,74236	NaN	NaN	1,08519	NaN	NaN	NaN
NaN	NaN	NaN	NaN	NaN	NaN	-0,0113964	NaN	NaN	NaN
NaN	NaN	NaN	NaN	NaN	NaN	NaN	NaN	NaN	NaN
NaN	-1,64473	NaN	-1,72701	NaN	NaN	NaN	NaN	NaN	NaN
NaN	-1,07894	NaN	-0,316181	-0,113657	NaN	-1,15544	NaN	NaN	-1,28764
NaN	-1,32769	-1,24281	0,278219	-1,7631	0,526556	-0,245001	NaN	NaN	NaN

Polito et al. Universal ready-to-use immunotherapeutic approach for the treatment of cancer: expanded and activated polyclonal γδ memory T cells.
Phosphorylation sites

3,55699	4,89266	4,98695	4,04521	5,36027	3,09012	4,52828	4,34209	3,9479	4,65288
3,16746	NaN	2,57792	2,78795	3,30672	1,48473	1,09976	2,72012	2,45376	3,59452
1,97351	2,61063	1,97222	1,40911	2,81256	1,17201	1,88441	1,72929	1,48465	1,84637
NaN	NaN	NaN	-1,85721	NaN	-1,83134	-2,36015	NaN	NaN	NaN
NaN	-0,260721	-0,838177	NaN	-0,863611	-1,14225	-1,48245	NaN	NaN	NaN
NaN	NaN	NaN	0,586622	NaN	NaN	1,10963	NaN	NaN	NaN
2,64833	2,62679	1,87051	4,28699	2,89853	3,62836	2,09835	2,55308	2,31628	2,26707
NaN	NaN	-2,37016	NaN	NaN	NaN	NaN	-2,95709	-2,79283	-2,68037
0,956013	NaN	-2,58024	2,06597	2,74516	2,44169	2,22516	NaN	NaN	NaN
-0,359716	0,14879	-0,761578	0,945816	-0,253349	0,0990963	1,01284	-0,131172	-0,293323	0,0199308
-1,89982	-3,1241	NaN	-1,32005	NaN	-0,846279	-1,34406	-2,27847	-2,27422	-3,09545
0,193996	-0,376907	NaN	0,481138	0,657118	0,226444	0,693731	NaN	NaN	NaN
NaN	NaN	NaN	NaN	-1,95601	0,719751	-1,59359	NaN	NaN	NaN
NaN	NaN	NaN	NaN	NaN	NaN	NaN	NaN	NaN	NaN
NaN	NaN	NaN	NaN	NaN	NaN	NaN	NaN	NaN	NaN
NaN	NaN	NaN	NaN	NaN	NaN	NaN	NaN	NaN	NaN
4,09033	3,60826	3,86154	4,44977	4,19291	3,97976	4,82447	3,83804	4,01185	3,51368
-0,042057	-1,50301	NaN	0,0309715	NaN	NaN	0,762001	0,129429	NaN	0,416919
1,38883	0,52902	0,332981	2,04959	1,48375	2,44098	1,84247	0,987827	1,06829	0,787753
NaN	NaN	NaN	-0,511007	NaN	NaN	-0,447048	NaN	NaN	NaN
NaN	NaN	NaN	-0,918468	NaN	-1,70512	-2,03323	NaN	NaN	NaN
NaN	NaN	NaN	-0,786608	NaN	-1,29169	-1,90492	NaN	NaN	NaN
-1,37776	-1,47775	-1,26801	0,0763626	-0,539057	-1,15591	NaN	-1,45075	-0,872552	NaN
NaN	NaN	NaN	NaN	NaN	NaN	NaN	NaN	NaN	NaN
NaN	NaN	-0,871607	-1,39842	NaN	-0,214743	-1,60762	-2,7211	-2,54577	-2,64479
-1,15823	1,0512	1,43578	NaN	-1,49564	NaN	NaN	-0,454201	0,156203	-1,21493
NaN	NaN	NaN	NaN	NaN	NaN	NaN	NaN	NaN	NaN
NaN	NaN	NaN	NaN	NaN	NaN	NaN	NaN	NaN	NaN
NaN	-0,648703	-4,67158	NaN	-0,498768	NaN	NaN	-0,237839	-0,394721	-0,394563
NaN	NaN	NaN	NaN	NaN	NaN	NaN	NaN	NaN	NaN
NaN	NaN	NaN	-1,30294	NaN	-1,06922	-2,02184	NaN	NaN	NaN
NaN	NaN	NaN	NaN	NaN	NaN	NaN	NaN	NaN	NaN
NaN	NaN	NaN	0,860004	-0,779116	0,260157	1,32001	NaN	NaN	NaN
NaN	NaN	NaN	2,18321	0,1017	NaN	2,50927	-0,411476	0,208346	0,334233

Polito et al. Universal ready-to-use immunotherapeutic approach for the treatment of cancer: expanded and activated polyclonal γδ memory T cells.
Phosphorylation sites

NaN	NaN	NaN	NaN	NaN	NaN	NaN	NaN	NaN	NaN
-1,50425	-0,335648	-0,435852	0,87862	-0,383049	1,53704	0,536098	NaN	NaN	-0,698667
NaN	-1,4052	NaN	-1,52279	-0,509331	NaN	-0,758533	NaN	NaN	NaN
-0,364824	NaN	-1,70668	-0,738184	-0,468317	0,488113	-1,34412	NaN	NaN	NaN
NaN	NaN	NaN	NaN	NaN	NaN	NaN	NaN	NaN	NaN
2,62663	2,15153	2,62435	3,10917	2,54164	1,65768	2,95522	2,90197	2,31162	2,78503
NaN	NaN	-3,99923	-2,50792	-3,63111	-2,53876	-3,03598	NaN	NaN	NaN
NaN	NaN	NaN	0,0528984	NaN	0,476274	-1,30909	NaN	NaN	NaN
3,92929	4,58839	3,98922	2,73061	3,8057	-1,73099	3,78051	3,57071	4,46688	4,13563
0,942951	1,61799	1,22063	1,94075	1,71733	1,87897	1,36686	0,502846	0,633132	0,454398
NaN	NaN	NaN	-0,943068	-1,2171	-2,43895	NaN	NaN	NaN	NaN
0,367172	0,267042	0,839455	0,482191	2,28077	0,765938	2,42673	1,52189	1,56379	1,38722
2,11016	3,05412	2,64351	1,21808	NaN	0,765938	2,42673	NaN	NaN	1,11104
NaN	NaN	NaN	-0,726336	-1,92448	NaN	-0,15099	NaN	NaN	NaN
-0,534616	-0,934597	-0,926991	0,315123	-1,03136	0,53104	0,204979	0,230555	0,212794	0,20007
0,683813	0,880293	0,22912	0,295021	1,6653	0,210688	NaN	0,437897	0,227408	NaN
5,00108	5,32058	5,81851	4,24233	5,81096	4,29221	4,52314	5,48555	5,39914	5,09988
-0,326031	0,00958061	0,851839	-0,255016	NaN	NaN	NaN	NaN	NaN	0,0678568
1,06905	1,48362	1,70711	1,84846	1,27861	1,9466	1,86276	1,26492	1,92518	1,5996
1,00488	0,980173	2,13973	1,55043	NaN	2,49693	1,70248	1,26274	1,92767	1,22253
NaN	NaN	NaN	-0,113184	-3,7765	-0,552437	-1,08292	NaN	NaN	NaN
NaN	-1,62523	3,02307	2,49503	0,128555	2,31893	2,53649	1,48983	1,12685	1,0813
NaN	-1,48589	-0,148443	0,672169	-0,54303	-1,33865	-0,0257263	NaN	NaN	-0,87074
-0,326031	0,00958061	0,851839	-0,255016	NaN	NaN	NaN	NaN	NaN	0,0678568
-1,67029	-2,58336	NaN	-0,197182	-2,58144	-1,15468	-1,42624	NaN	NaN	NaN
NaN	-1,07549	-0,0806828	0,130028	NaN	1,48542	0,343691	-2,0263	0,111154	-1,04905
-1,08051	NaN	-1,2907	1,64068	-0,204927	1,5424	-0,140926	NaN	NaN	NaN
0,998413	-0,265938	0,0990086	-0,984512	2,65116	-1,86919	-0,0779839	1,59207	0,94875	1,25433
NaN	NaN	NaN	-2,56236	NaN	-1,84019	-3,62972	NaN	NaN	NaN
NaN	NaN	NaN	-1,28071	-2,45645	1,29681	NaN	NaN	NaN	NaN
NaN	NaN	NaN	-0,667139	NaN	NaN	-1,46489	NaN	NaN	NaN
NaN	NaN	NaN	NaN	NaN	NaN	NaN	NaN	NaN	NaN
2,94356	2,12592	3,73927	5,23751	4,0608	5,41261	3,89824	2,17648	1,91663	1,9168
1,03286	1,0889	2,93415	2,01266	2,12152	0,304205	1,95758	2,56725	3,1728	2,10283

Polito et al. Universal ready-to-use immunotherapeutic approach for the treatment of cancer: expanded and activated polyclonal γδ memory T cells.
Phosphorylation sites

-1,29733	-1,3107	0,358187	-1,09884	-0,127848	-2,28315	NaN	1,42052	1,10021	0,807689
NaN	NaN	NaN	-2,66635	NaN	-3,18659	-1,54786	NaN	NaN	NaN
1,11636	0,595274	3,17238	2,21966	1,85133	2,79951	3,38752	2,72462	3,19225	2,70806
0,210487	-0,832308	2,67458	2,07917	1,53322	3,34841	2,93389	1,3901	0,392148	0,121202
-0,49548	-3,22162	3,92703	3,64201	-0,413816	3,52764	0,43877	1,3242	0,81129	0,990317
0,255571	1,26601	-0,867424	0,815784	0,487427	1,81093	0,501472	-0,427622	NaN	-0,0854292
NaN	-2,22864	NaN	-3,87849	-1,96508	-3,55079	NaN	NaN	NaN	NaN
NaN	-2,74638	NaN	-2,74291	NaN	-2,04116	-2,62062	-1,09385	-1,52591	NaN
-0,0348816	0,859753	-0,460958	1,74784	1,32019	0,680811	0,634935	-0,132484	-0,243451	0,534945
2,5354	2,95963	3,93412	NaN	3,43555	NaN	NaN	2,6527	-0,932353	2,72794
-1,26527	-1,54176	-0,402298	-0,781702	0,161976	NaN	0,00475311	-0,0427303	0,111627	-0,0508337
NaN	NaN	NaN	-0,213543	NaN	NaN	0,169355	-1,58237	NaN	-0,456511
0,18462	0,522528	1,29188	-0,838026	1,22281	-1,47551	-0,432076	0,910055	0,876529	0,870542
NaN	NaN	NaN	NaN	NaN	NaN	NaN	NaN	NaN	NaN
NaN	NaN	NaN	0,530207	-0,44216	-1,26368	0,105837	NaN	NaN	NaN
0,297691	0,443741	0,798119	1,29561	1,11759	NaN	1,52958	-0,180351	0,295047	0,0503683
-0,327318	0,863609	0,346563	NaN	0,827879	-3,45306	-0,159451	0,276827	0,673682	NaN
NaN	NaN	NaN	0,818705	NaN	-1,41696	-0,767141	NaN	NaN	NaN
0,844709	-0,0618763	0,448685	0,0484066	0,469141	NaN	0,313835	0,636116	0,691823	1,19041
NaN	NaN	NaN	-1,43359	NaN	-0,473278	-1,98687	NaN	NaN	NaN
1,64387	1,88987	1,8658	0,605955	2,10535	0,988995	-0,469955	1,73896	1,50387	1,78735
-2,58184	NaN	-1,85752	-2,47097	-0,653585	-2,44677	0,132931	-2,15747	-1,17475	-0,3147
NaN	NaN	NaN	1,78139	NaN	1,62879	1,59998	NaN	NaN	NaN
NaN	NaN	NaN	-0,646475	NaN	-2,20825	-1,20692	NaN	NaN	NaN
2,83848	1,61672	1,57041	1,81698	NaN	-0,321793	1,5812	1,67635	1,40418	1,39291
0,712564	-0,117661	-0,202639	-2,66239	0,91008	NaN	NaN	0,933882	0,624244	0,171359
NaN	NaN	NaN	-1,10164	-2,18533	-1,64172	-0,480862	NaN	NaN	NaN
NaN	-4,11941	-1,16788	NaN	NaN	NaN	NaN	-2,04729	-1,9071	-1,22113
-1,82421	-1,45004	-0,573351	-1,17533	-0,354013	NaN	0,509705	-1,30484	-1,54937	-0,921172
NaN	NaN	NaN	NaN	NaN	NaN	NaN	NaN	NaN	NaN
NaN	NaN	NaN	NaN	NaN	-0,0114288	-0,370024	NaN	NaN	NaN
-0,680807	-0,69146	-0,388691	-0,246159	0,210602	0,12097	0,0884972	-0,414364	-0,811566	-0,291566
-0,755785	-3,15943	-0,0757389	-0,850039	NaN	-0,185966	-0,811281	-1,26834	-0,950589	NaN
NaN	NaN	NaN	1,68314	-3,63538	-0,246235	-0,671747	NaN	NaN	NaN

Polito et al. Universal ready-to-use immunotherapeutic approach for the treatment of cancer: expanded and activated polyclonal γδ memory T cells.
Phosphorylation sites

NaN	NaN	NaN	NaN	NaN	NaN	NaN	NaN	NaN	NaN
NaN	NaN	NaN	2,73575	NaN	3,1607	2,35131	NaN	NaN	NaN
NaN	-3,47825	-2,07293	-0,0569725	-2,95588	-0,716026	-1,83413	NaN	NaN	NaN
NaN	NaN	NaN	-0,944483	NaN	-0,0591984	NaN	NaN	NaN	NaN
NaN	NaN	NaN	0,771889	NaN	-0,0372658	-1,18567	NaN	NaN	NaN
NaN	NaN	NaN	-0,287651	NaN	0,208614	-2,44559	NaN	NaN	NaN
NaN	NaN	NaN	-0,150322	NaN	0,221313	-1,00829	NaN	NaN	NaN
NaN	NaN	NaN	NaN	NaN	NaN	NaN	NaN	NaN	NaN
NaN	NaN	NaN	-0,635868	NaN	NaN	NaN	NaN	NaN	NaN
NaN	NaN	NaN	NaN	NaN	NaN	NaN	NaN	NaN	NaN
NaN	NaN	NaN	0,571859	NaN	NaN	NaN	NaN	NaN	NaN
NaN	NaN	NaN	-1,15844	NaN	-0,965496	-2,61897	NaN	NaN	NaN
NaN	NaN	NaN	-0,499268	NaN	-0,164049	-2,0283	NaN	NaN	NaN
NaN	NaN	NaN	NaN	NaN	0,54318	NaN	NaN	NaN	NaN
NaN	NaN	NaN	1,48758	NaN	1,69724	NaN	NaN	NaN	NaN
NaN	NaN	NaN	1,03374	NaN	1,68853	0,0401096	NaN	NaN	NaN
NaN	NaN	NaN	1,17216	NaN	-0,756996	-0,599031	NaN	NaN	NaN
NaN	NaN	NaN	2,94204	-0,50836	2,55974	0,733521	NaN	NaN	NaN
NaN	NaN	NaN	0,426517	NaN	-0,791468	-1,25715	NaN	NaN	NaN
NaN	NaN	NaN	0,808142	NaN	-1,48249	0,0622387	NaN	NaN	NaN
1,12774	NaN	0,179169	-0,65308	1,56249	-1,95807	0,0592175	-0,423969	-0,559583	-0,581088
NaN	NaN	-0,848866	NaN	NaN	NaN	NaN	-1,63478	-2,06633	-1,93444
NaN	-0,784431	-0,223845	NaN	-0,953091	-0,385517	NaN	-1,75759	NaN	-1,93155
NaN	NaN	NaN	-2,17911	-0,86838	NaN	-1,23305	NaN	NaN	NaN
0,589048	-0,0631618	-3,01551	0,189009	1,83731	0,535677	0,216757	-0,479712	0,520549	0,111377
NaN	NaN	NaN	NaN	NaN	NaN	NaN	NaN	NaN	NaN
1,57662	1,57099	1,62029	1,05575	1,97841	-3,15242	1,52353	1,77897	1,77765	1,61069
NaN	NaN	NaN	-0,90505	NaN	NaN	NaN	NaN	NaN	NaN
NaN	NaN	NaN	0,169756	-1,42416	-0,892736	-0,329332	-2,12664	NaN	-1,21845
NaN	-0,274166	-2,15494	-1,15345	-0,158016	-2,22911	-0,644663	-2,98013	-1,54919	-0,919738
NaN	1,61621	NaN	2,25825	1,25842	2,40197	1,66082	NaN	NaN	1,87299
1,20025	1,0599	1,66783	1,5755	0,962118	NaN	NaN	NaN	1,99687	1,53637
-1,06198	-1,15304	-1,00835	-2,76074	-0,529957	NaN	-0,0540047	-0,406342	-1,03254	-0,642276
2,06132	1,79191	2,08528	0,239769	2,06325	0,383049	1,85439	2,58515	2,67952	0,142335

Polito et al. Universal ready-to-use immunotherapeutic approach for the treatment of cancer: expanded and activated polyclonal γδ memory T cells.
Phosphorylation sites

NaN	NaN	NaN	NaN	2,12171	-1,78815	-0,620205	NaN	0,114434	NaN
-0,809422	0,266525	-0,569855	-1,47137	-0,453835	1,38782	-2,21832	-1,66911	-1,51239	-1,34776
NaN	NaN	-0,79113	-2,06096	NaN	0,00860214	NaN	-1,85673	-2,12452	-3,58114
NaN	NaN	0,352253	-1,00138	-2,77882	-2,44182	-0,785089	-0,300169	-0,380313	-0,457551
NaN	NaN	NaN	NaN	NaN	NaN	NaN	NaN	NaN	NaN
-1,48195	NaN	-0,893726	-1,79842	-1,27889	-0,351528	-0,948719	-0,285593	-0,206586	-0,142446
NaN	NaN	NaN	-0,986769	-0,712234	NaN	NaN	-1,3816	-1,73727	-1,16047
-1,82057	-0,582823	-1,25981	-0,838816	0,16073	NaN	-0,552338	-2,00542	-1,5172	-0,938924
NaN	NaN	NaN	-2,0324	NaN	NaN	NaN	NaN	NaN	NaN
-1,25229	-1,23857	-1,37571	-1,27083	-1,1978	-0,638708	-1,61014	-2,10954	-1,82161	-1,8371
1,20277	1,02794	1,63018	0,572105	1,01665	0,887711	1,32706	1,78926	2,46235	1,98441
-2,07006	NaN	0,810862	-0,57548	-2,09098	0,339581	0,0139008	0,10568	0,257144	0,0800238
-1,46935	NaN	-1,88552	-0,552086	-0,936018	0,0880909	-0,765242	NaN	NaN	NaN
-3,70395	NaN	-1,0616	0,171732	NaN	1,52631	-0,526291	NaN	NaN	NaN
-0,420883	0,311153	0,224394	-0,266003	-0,719566	0,537165	0,828192	0,457645	0,0936155	0,791736
NaN	NaN	NaN	-0,80769	-2,01443	NaN	-1,65539	NaN	NaN	NaN
NaN	NaN	0,14599	0,444324	0,182615	NaN	0,343691	NaN	NaN	NaN
-0,91535	-2,22231	-1,50293	NaN	-1,31784	NaN	NaN	-1,92461	-2,01428	-2,4995
NaN	NaN	NaN	-1,27238	-3,67431	-0,0649529	-1,31775	NaN	NaN	NaN
-1,57172	-1,72712	NaN	-0,125364	-1,8213	0,376049	0,781372	NaN	-1,34196	-1,15274
-1,40913	NaN	-1,89534	0,0211506	-2,16555	0,586599	-0,200769	-2,35999	-2,52049	NaN
NaN	NaN	NaN	-2,52348	NaN	-2,08121	-1,29017	NaN	NaN	NaN
-2,01797	-2,85006	-2,44829	NaN	NaN	NaN	-1,87248	-1,56264	-1,61294	-2,4826
-2,02631	NaN	NaN	-1,72208	-1,60261	-2,55409	-1,39176	NaN	-0,963048	-1,41092
NaN	NaN	NaN	-2,39707	-1,89732	-0,561958	-0,824795	NaN	NaN	NaN
-0,701746	-1,97524	NaN	1,22344	-0,939013	2,81204	0,525068	-1,6118	-1,47617	-1,27476
NaN	NaN	NaN	-0,927984	-2,23707	-2,64493	-0,0396919	-2,81555	-3,21467	-2,01712
NaN	NaN	NaN	-1,17221	NaN	0,378263	-0,253101	NaN	NaN	NaN
NaN	NaN	NaN	NaN	NaN	NaN	NaN	NaN	NaN	NaN
0,122396	NaN	-1,63818	NaN	NaN	NaN	NaN	-0,122972	-1,20559	-1,5898
NaN	NaN	NaN	NaN	NaN	NaN	NaN	NaN	NaN	NaN
0,847937	0,524477	1,39122	3,56314	1,60349	3,66018	2,86933	1,55121	1,298	1,40154
NaN	NaN	-0,356827	2,29905	-3,14985	2,16364	1,54099	NaN	NaN	NaN
NaN	NaN	NaN	-0,800789	-2,22932	-1,59329	NaN	NaN	NaN	NaN

Polito et al. Universal ready-to-use immunotherapeutic approach for the treatment of cancer: expanded and activated polyclonal γδ memory T cells.
Phosphorylation sites

1,02191	2,08008	3,40718	2,87231	1,89709	3,97451	2,849	2,09627	1,90315	1,94365
NaN	-1,22722	-1,91567	-1,71652	NaN	-2,09466	NaN	-1,89852	-2,26631	-2,43132
NaN	-2,44817	-1,19855	NaN	NaN	NaN	NaN	-2,11536	-1,78687	-2,66715
1,56656	2,58876	1,31974	NaN	2,81535	0,786577	NaN	2,32494	2,56302	2,64753
NaN	NaN	NaN	-2,04145	NaN	NaN	-2,08065	NaN	-3,33563	NaN
NaN	NaN	NaN	-0,402426	NaN	NaN	-2,34429	NaN	NaN	NaN
NaN	NaN	NaN	0,740656	NaN	NaN	0,2929	NaN	NaN	NaN
NaN	NaN	NaN	-1,96612	NaN	-1,44279	-2,58144	NaN	NaN	NaN
NaN	NaN	NaN	NaN	NaN	NaN	NaN	NaN	NaN	NaN
NaN	NaN	NaN	0,696987	-1,18094	1,25057	-0,0699615	NaN	NaN	NaN
NaN	NaN	NaN	NaN	NaN	NaN	NaN	NaN	NaN	NaN
1,2639	2,43161	1,41558	2,32331	1,74669	-1,9127	1,78462	1,02495	0,762921	0,112927
NaN	NaN	NaN	-2,17653	NaN	-3,66219	-3,57831	NaN	NaN	NaN
NaN	NaN	NaN	-1,61258	-3,07388	-0,721411	-1,31569	NaN	NaN	NaN
-0,448153	0,467821	0,213594	NaN	NaN	-0,450796	NaN	NaN	0,716496	0,545157
0,542484	0,85812	0,0287724	-0,0422478	1,00026	0,248804	0,947943	0,980135	1,194	0,655248
2,42107	2,79967	2,43257	2,73	3,32664	NaN	3,05445	2,46321	2,24062	2,11486
NaN	NaN	NaN	-1,19687	NaN	-1,14029	-1,72646	NaN	NaN	NaN
0,3957	NaN	0,608864	NaN	1,07868	0,294271	0,885323	0,295868	NaN	1,49333
NaN	-1,04736	NaN	NaN	0,741693	-3,10288	-0,669365	-0,463766	NaN	-0,618968
-1,24725	-1,53398	-0,761003	2,53373	-0,975334	2,20071	1,16918	NaN	NaN	NaN
NaN	NaN	NaN	1,6175	NaN	2,30956	-0,986431	NaN	NaN	NaN
NaN	NaN	NaN	NaN	NaN	NaN	NaN	NaN	NaN	NaN
1,22002	2,32996	2,48227	6,03613	1,45407	6,89739	5,40285	0,338232	0,540851	0,673118
-0,419748	0,166943	0,544182	3,78984	NaN	NaN	NaN	NaN	NaN	NaN
0,155678	0,999937	1,53508	4,85671	NaN	4,38351	3,95384	-0,445852	-1,1996	0,0766764
NaN	0,509783	2,37067	4,38974	1,48039	3,05392	3,82242	NaN	NaN	NaN
-0,937794	-0,160877	-1,74703	-0,758162	0,706478	-2,18485	-0,193239	-0,704151	-1,19344	-0,865546
3,01048	2,77655	2,56017	3,24598	3,71447	1,91132	3,14604	2,80323	2,51622	2,36832
NaN	-0,198191	NaN	-0,628489	-1,18004	2,81381	-1,35752	NaN	NaN	NaN
-1,82642	NaN	-1,46183	-1,73274	-2,15868	NaN	-2,37816	NaN	NaN	NaN
-0,766832	NaN	-0,22533	-0,824585	0,182365	-0,807213	0,26639	-0,4659	NaN	-0,739465
NaN	NaN	NaN	NaN	NaN	NaN	NaN	NaN	NaN	NaN
-0,235664	1,86844	-0,155296	1,81029	-4,05547	2,92656	1,56589	0,25651	0,568496	0,837403

Polito et al. Universal ready-to-use immunotherapeutic approach for the treatment of cancer: expanded and activated polyclonal γδ memory T cells.
Phosphorylation sites

-0,474922	-0,527861	-1,72689	0,541365	NaN	0,654102	-0,362534	-0,945736	NaN	-0,598531
NaN	NaN	NaN	0,951151	0,653706	0,331697	0,694008	NaN	NaN	NaN
-2,98137	-1,78731	NaN	NaN	NaN	NaN	NaN	NaN	NaN	NaN
NaN	NaN	NaN	-0,921076	NaN	-1,20096	-2,36798	NaN	NaN	NaN
NaN	NaN	NaN	-1,99367	NaN	-2,07787	-3,81989	NaN	NaN	NaN
-0,793705	-1,21965	-0,447107	0,0523129	-1,62761	0,370035	-0,895493	-1,23068	-2,11507	NaN
-1,91169	-2,14448	NaN	NaN	-2,04227	NaN	NaN	-4,7671	-1,78171	-2,1719
NaN	NaN	NaN	0,034008	-1,91984	-0,40905	-1,27408	-1,39033	NaN	-1,48903
-0,603825	-1,63086	-1,14826	-0,0564003	-0,476402	-0,607079	-0,123562	-0,92712	-1,69975	-1,48485
-2,63714	-3,33657	-3,07754	-0,78401	-1,75636	-2,64583	-1,01203	-1,73475	-1,85869	-2,49574
NaN	NaN	-3,76321	-1,40148	NaN	-3,06417	-2,9535	NaN	NaN	NaN
-0,673384	-0,587824	0,130526	NaN	-0,792744	NaN	NaN	-1,72812	-0,621686	-1,25655
2,02767	1,06928	-0,356773	1,79034	0,614187	NaN	1,08614	0,246641	0,325562	-0,489543
NaN	NaN	NaN	-0,697283	-1,37761	1,4104	-1,59123	NaN	NaN	NaN
-0,696384	-2,21087	2,04583	2,78454	0,621782	2,2435	2,553	0,0583649	-0,331239	-0,445531
0,553158	0,957409	NaN	1,96995	0,153515	1,96927	-3,50318	-0,947178	NaN	-0,110778
4,36132	3,55	3,82303	3,95919	5,66258	4,3572	4,1357	4,0401	3,87973	3,85049
NaN	NaN	-2,41852	-0,620695	-2,56624	NaN	-1,07254	NaN	NaN	NaN
-0,340286	-1,09619	-0,406872	NaN	-0,437571	NaN	NaN	1,62212	1,126	0,806376
-0,500998	-2,22908	-2,56223	NaN	-1,14353	NaN	NaN	0,156982	0,24579	-1,0015
NaN	NaN	NaN	NaN	NaN	NaN	NaN	0,0331154	-2,01495	0,00422764
1,06841	2,46105	2,26536	1,38637	0,436653	1,77843	0,63364	2,70712	2,52234	2,36396
NaN	NaN	0,911879	1,4693	1,55341	2,35987	-1,6924	NaN	0,698411	NaN
NaN	NaN	NaN	-1,49475	NaN	0,058363	-1,73811	NaN	NaN	NaN
-2,52537	-1,18623	-2,80106	-1,64492	-1,13233	NaN	NaN	-3,31519	NaN	-2,125
NaN	NaN	NaN	NaN	NaN	NaN	NaN	NaN	NaN	NaN
-1,33659	NaN	-0,960093	-1,01354	-0,491993	-0,525467	-0,525841	-0,825893	-0,768271	-0,890344
-0,965637	NaN	-1,52271	-0,41951	-2,10816	NaN	0,098362	-1,60518	-0,899577	-1,79453
NaN	NaN	NaN	0,406271	NaN	-2,41096	-0,533907	NaN	-3,08612	-2,95099
-0,0855408	NaN	0,311193	0,375004	0,422085	0,152687	NaN	1,1189	0,799672	0,844193
-0,671143	0,386856	-1,28144	1,60571	0,31691	0,433968	1,00807	0,418749	0,00118923	0,0995378
NaN	NaN	NaN	NaN	NaN	NaN	NaN	NaN	NaN	NaN
-1,47102	-1,07585	NaN	-0,536484	-0,296207	-1,77075	-1,86978	NaN	NaN	NaN
-0,996033	NaN	-0,747454	-0,733194	-0,337214	-1,13221	-0,639328	NaN	NaN	NaN

Polito et al. Universal ready-to-use immunotherapeutic approach for the treatment of cancer: expanded and activated polyclonal γδ memory T cells.
Phosphorylation sites

NaN	NaN	NaN	-0,0110149	NaN	NaN	0,665548	-1,55654	-0,310693	-0,896653
0,0360603	0,699677	1,07751	0,87682	1,56758	-0,400799	1,88937	0,562109	0,0505991	0,631209
1,71929	1,52231	4,14901	2,69619	3,60241	3,74333	3,49573	3,27606	3,24679	3,10144
NaN	NaN	NaN	NaN	NaN	NaN	NaN	-1,62405	-2,38138	-2,56413
-0,406197	0,770973	0,630384	0,483244	1,55525	-0,0491199	1,67103	0,91337	0,188003	1,26974
-1,49913	-0,762939	-1,15456	-1,2148	-0,828903	NaN	-0,474482	-0,210077	-0,661031	-0,365977
-0,413998	-0,979567	-1,49903	0,470963	0,128736	NaN	1,50994	-0,157888	0,0944986	-0,0766649
NaN	-1,77985	-1,88905	0,588335	NaN	NaN	0,0673237	NaN	NaN	NaN
-1,50774	-0,99386	-1,92449	-0,286417	NaN	0,832926	-0,467388	-1,05971	NaN	NaN
0,867765	0,127415	0,88871	1,18134	1,26873	0,815329	0,806507	0,115269	-0,423064	0,0379248
NaN	NaN	NaN	-0,375849	-2,4068	NaN	-0,151958	NaN	NaN	NaN
-2,02813	NaN	NaN	NaN	NaN	NaN	NaN	-1,39774	-0,884658	-2,90441
-0,3904	1,81343	1,0445	1,59444	1,98805	0,505184	0,746632	-0,225306	-0,219684	0,822688
0,776505	1,47142	0,514961	NaN	2,10898	NaN	0,14357	1,07067	1,78778	1,08834
-0,852863	-0,642075	-0,753695	-1,04028	NaN	NaN	-0,893789	-0,49844	-0,612689	-0,996688
-1,83507	-1,32207	NaN	-0,378977	-2,12555	NaN	-1,28172	-0,49844	0,422091	NaN
0,629257	1,79824	1,20352	1,81369	1,25735	NaN	1,17404	1,43443	1,42227	1,43239
-0,84306	-1,56813	-1,09675	NaN	0,306118	NaN	NaN	-0,698231	-0,532987	-0,567592
1,04037	1,62174	1,50254	NaN	1,39561	NaN	-2,01487	1,63984	2,11667	NaN
0,15826	0,230862	0,917387	-1,96856	0,22765	NaN	-1,76756	-0,97942	0,940333	3,50641
5,38715	5,63066	5,68912	2,68937	5,70582	0,528467	3,18513	3,87591	6,44887	6,19372
NaN	NaN	NaN	NaN	NaN	NaN	NaN	-1,38264	-2,00891	-1,8431
NaN	0,400728	NaN	NaN	NaN	NaN	NaN	0,130226	0,455527	-0,426839
0,000513077	0,591454	1,60699	NaN	1,06461	NaN	-1,89433	2,03472	1,47833	1,48655
-0,198769	0,0422764	-0,0618649	NaN	NaN	NaN	NaN	-0,0324135	0,748832	0,0148745
0,437126	0,996428	NaN	-0,206442	0,529184	-0,155081	-0,645536	0,646606	1,20098	0,122951
1,30315	1,7462	1,45555	-2,40737	1,09607	NaN	-1,39687	2,17538	1,49284	1,56853
NaN	NaN	NaN	NaN	-2,39668	NaN	NaN	NaN	NaN	NaN
0,605074	2,1603	1,20214	NaN	0,978083	NaN	NaN	1,27041	0,601445	1,66749
0,267616	0,600523	-0,0629063	1,01112	0,0927162	1,13686	-0,169796	NaN	NaN	-0,278871
NaN	NaN	NaN	NaN	NaN	NaN	NaN	NaN	NaN	NaN
-0,0887413	-0,0465603	-0,0412598	0,710194	0,506767	1,08905	1,32099	-0,738071	-0,824872	-0,356515
NaN	NaN	NaN	-0,981243	-2,65064	-2,21463	-0,33699	NaN	NaN	NaN
-0,136816	-1,0238	1,11293	1,53561	NaN	NaN	1,48629	NaN	NaN	NaN

Polito et al. Universal ready-to-use immunotherapeutic approach for the treatment of cancer: expanded and activated polyclonal γδ memory T cells.
Phosphorylation sites

3,69828	3,26999	3,99893	3,4507	5,00673	2,40835	3,27394	3,99238	3,53755	3,81861
2,02794	1,87885	2,2791	1,89917	1,85887	NaN	2,52263	1,81401	2,25633	1,64441
-0,876148	-0,809332	-1,0376	0,461916	-0,430004	0,666161	0,317677	-0,766939	-0,249362	-0,451261
-0,972103	-0,316374	0,126083	NaN	1,32031	NaN	-1,89499	-0,407633	-0,432029	0,679868
1,78573	2,91053	2,64381	1,755	3,22616	0,388918	2,92013	2,63285	3,19232	2,88779
1,64537	-0,138927	2,58607	-0,621628	0,545639	NaN	-0,54998	3,29143	3,19593	2,51573
-1,70487	-1,34128	-1,20983	-1,01636	NaN	NaN	-2,32883	NaN	NaN	NaN
-1,70487	-1,34128	-1,20983	-1,01636	NaN	NaN	-2,32883	NaN	NaN	NaN
-1,47915	NaN	-1,83458	NaN	NaN	NaN	NaN	-1,48665	-0,730077	-0,939189
-1,26511	-1,7211	-2,15797	-1,66064	NaN	-1,07704	NaN	NaN	-2,38117	NaN
NaN	NaN	NaN	NaN	NaN	NaN	NaN	NaN	NaN	NaN
5,62272	5,529	5,08987	6,20676	5,92808	5,70473	6,27109	5,66512	5,51203	5,48943
0,629215	0,713993	NaN	0,275017	0,759254	0,657906	0,797277	0,890409	0,589637	0,720126
NaN	NaN	2,16088	1,62955	0,849957	NaN	NaN	2,11516	2,4547	1,65487
NaN	-1,57364	-1,77461	-1,54526	-0,725525	NaN	-0,95438	-1,65098	-1,72536	-1,42323
-1,39497	-1,12087	NaN	-1,41971	0,770615	0,559454	NaN	-0,633497	-1,02043	-0,590596
NaN	-2,0393	NaN	NaN	NaN	NaN	NaN	-1,71202	-0,639403	-1,10684
NaN	NaN	-0,219494	0,347021	NaN	-0,164318	-0,070713	NaN	NaN	NaN
NaN	NaN	NaN	0,271091	NaN	0,507883	-1,1707	NaN	NaN	NaN
NaN	NaN	NaN	-1,1481	-1,89605	NaN	-0,531096	-2,69192	-3,02772	-2,73466
NaN	NaN	NaN	-2,33974	-4,17072	NaN	-2,10573	NaN	NaN	NaN
NaN	NaN	NaN	-0,115871	NaN	0,605154	-0,266775	NaN	NaN	NaN
2,33358	2,14083	1,96909	1,36925	2,28556	2,29464	1,76299	2,43161	2,09537	2,13537
0,948746	0,903963	1,64652	1,19474	1,594	NaN	2,26118	1,02984	0,890645	1,79531
1,24839	-0,783627	0,166098	0,971729	1,19687	0,939741	2,00529	1,01066	0,803555	0,551528
NaN	NaN	NaN	-0,487597	NaN	0,936739	-2,52324	-1,95514	-1,40275	-1,95183
NaN	NaN	NaN	NaN	NaN	NaN	NaN	NaN	NaN	NaN
NaN	NaN	NaN	NaN	NaN	NaN	NaN	NaN	NaN	NaN
NaN	NaN	NaN	-0,978994	NaN	NaN	-0,186026	NaN	NaN	NaN
-3,47685	NaN	-2,09848	0,105171	-1,60971	-3,55867	-0,119793	NaN	NaN	NaN
1,95503	1,01173	1,48545	2,54234	2,58242	-0,183243	2,3263	1,69457	2,06005	1,24216
NaN	NaN	NaN	-0,963642	NaN	NaN	-2,07584	NaN	NaN	NaN
NaN	0,355053	NaN	NaN	NaN	NaN	NaN	0,125021	0,552363	0,0896311
1,66141	1,63649	2,02578	1,68171	2,54739	0,666161	2,14639	2,27093	1,62151	2,02123

Polito et al. Universal ready-to-use immunotherapeutic approach for the treatment of cancer: expanded and activated polyclonal γδ memory T cells.
Phosphorylation sites

1,71976	2,60537	1,81062	2,5605	2,64581	3,21074	2,62058	1,77745	1,0151	2,56061
-1,40103	-2,32183	0,763325	0,475861	-0,281342	1,26973	0,66991	-0,0259476	0,489245	0,0290022
NaN	NaN	NaN	NaN	NaN	NaN	NaN	NaN	NaN	NaN
-3,17028	-1,86235	-2,04458	-2,05125	-2,62172	-1,18736	-1,31208	NaN	NaN	NaN
NaN	NaN	NaN	NaN	NaN	NaN	NaN	NaN	NaN	NaN
NaN	NaN	NaN	NaN	NaN	NaN	NaN	NaN	NaN	NaN
2,25561	2,17783	2,28071	2,88605	2,74117	3,336	3,27719	2,39727	2,07512	2,3954
NaN	NaN	NaN	-0,927391	NaN	-1,09448	-2,25252	NaN	NaN	NaN
0,317331	0	0,495443	0,937969	0,191931	0,482462	0,366734	0,158213	0,203635	-0,325385
3,22211	3,3923	3,0597	3,52165	3,57652	0,694649	3,72047	3,0489	3,10723	3,11
NaN	NaN	NaN	NaN	NaN	-2,7585	NaN	NaN	NaN	NaN
NaN	NaN	NaN	NaN	NaN	NaN	NaN	NaN	NaN	NaN
NaN	NaN	NaN	NaN	NaN	NaN	NaN	NaN	NaN	NaN
-0,71983	-0,902122	NaN	-0,454397	-1,07059	-0,645477	NaN	NaN	NaN	NaN
-1,19802	-3,17905	1,3648	1,52823	-0,353832	NaN	0,94179	-0,341351	-0,336875	0,0900621
0,634342	2,29178	1,17715	1,37988	1,37855	2,02412	2,45088	1,59941	1,88699	2,33123
0,148426	-0,444262	1,85378	1,63312	2,29498	0,991648	1,86626	1,96507	1,59569	1,78862
NaN	NaN	NaN	-0,691097	-1,42889	-1,63418	-2,53213	NaN	NaN	NaN
NaN	NaN	NaN	1,66146	1,59089	0,594398	NaN	0,583624	NaN	1,20352
NaN	NaN	NaN	-0,712715	-1,41268	-0,654108	-0,911041	NaN	NaN	NaN
NaN	NaN	-0,890972	NaN	-0,585831	-1,78741	0,947247	NaN	NaN	NaN
-0,72757	-0,805895	-0,298861	0,173918	-0,501019	NaN	1,06739	0,431362	0,281426	0,261052
-3,16751	NaN	-0,0629501	-1,64665	-2,57014	NaN	0,819101	-0,210741	-0,434161	-0,659608
-0,723179	-0,0297165	-0,0445137	-3,21623	-0,80695	NaN	-0,924551	0,0365467	NaN	-0,705276
5,6905	5,2582	4,80647	5,67461	5,34412	5,90547	5,87527	4,63392	4,43129	4,60813
NaN	NaN	NaN	NaN	NaN	-2,46122	-1,75197	NaN	NaN	NaN
NaN	-1,47598	NaN	NaN	NaN	-0,46755	-0,412968	NaN	NaN	NaN
-1,88762	0,120983	-0,93228	0,0652847	-0,371857	0,912752	1,25557	-0,702244	-0,408509	-0,156497
NaN	NaN	NaN	0,113283	NaN	NaN	-0,158403	NaN	NaN	NaN
NaN	NaN	NaN	-1,6207	-3,19361	NaN	-2,27354	NaN	NaN	NaN
NaN	NaN	NaN	NaN	NaN	NaN	NaN	NaN	NaN	NaN
-0,712971	-0,448612	-1,25515	-0,860079	-0,197475	-1,08	-0,662521	-0,764565	-0,114141	-1,19625
-2,58988	NaN	-3,0582	NaN	-3,33828	NaN	NaN	NaN	NaN	NaN
NaN	0,130121	-1,76831	-2,88424	-0,139732	NaN	-0,82432	NaN	NaN	-1,52761

Polito et al. Universal ready-to-use immunotherapeutic approach for the treatment of cancer: expanded and activated polyclonal γδ memory T cells.
Phosphorylation sites

NaN	NaN	NaN	NaN	NaN	NaN	NaN	NaN	NaN	NaN
0,995729	NaN	2,9767	-0,514523	NaN	NaN	1,55786	NaN	NaN	NaN
1,85128	3,09204	1,62531	3,24559	2,83249	3,07924	2,71886	-0,781528	1,03805	2,60497
NaN	NaN	NaN	NaN	NaN	NaN	NaN	-2,02387	-2,42059	-2,29129
NaN	NaN	NaN	0,216639	NaN	1,50168	-0,574097	NaN	NaN	NaN
NaN	NaN	NaN	-0,535126	-2,55778	-0,102028	-1,78347	NaN	NaN	NaN
NaN	NaN	NaN	NaN	-2,63421	NaN	-3,80554	-2,99813	-2,72251	-2,86547
1,35729	1,29683	1,19651	-0,541023	0,350702	-0,920185	NaN	0,437952	-0,0195951	-0,0453043
NaN	-1,32351	NaN	NaN	NaN	NaN	NaN	-0,798956	-1,11614	-0,0453043
NaN	NaN	NaN	NaN	NaN	NaN	NaN	-0,968885	-0,665189	-0,772248
NaN	-0,267193	0,134296	1,12067	-0,254129	-0,883909	0,677895	0,0122204	NaN	-0,170066
3,09859	3,19679	2,31251	3,02631	4,22787	3,49123	3,23879	2,24106	2,50492	3,76202
-1,60304	-1,35238	NaN	-1,00495	-1,65439	NaN	-1,2632	NaN	NaN	NaN
2,15185	2,06795	1,57519	1,8185	2,57476	2,29747	1,3586	1,28481	1,33782	1,29371
1,82387	1,77321	1,91024	1,75173	3,62018	-1,42824	2,54701	2,23403	2,51151	3,34068
-0,956512	0,121412	NaN	0,177383	NaN	NaN	0,547335	-0,63908	0,506455	0,0260077
0,233585	-0,143341	-0,158724	-3,0078	0,361786	-1,52852	NaN	-0,393213	-0,0934477	-0,163085
NaN	NaN	NaN	0,521397	NaN	1,29295	-1,04291	NaN	NaN	NaN
NaN	NaN	-1,24406	0,190718	NaN	0,508389	-0,872276	NaN	NaN	NaN
NaN	NaN	NaN	-1,19148	NaN	0,624481	-0,584984	NaN	NaN	NaN
0,614201	0,285566	0,0827885	1,18773	-1,32858	NaN	1,13631	0,453079	0,302382	0,0469942
0,217972	-0,395721	-0,574455	0,748884	NaN	1,60421	0,341471	NaN	-0,450772	NaN
0,676989	1,13033	1,53267	0,251677	NaN	0,842047	NaN	NaN	NaN	NaN
NaN	NaN	NaN	2,96162	1,74295	NaN	3,35821	NaN	2,78499	NaN
NaN	-0,0417252	-0,862406	0,248198	0,472424	NaN	0,920931	NaN	NaN	NaN
NaN	NaN	NaN	-2,67231	NaN	-1,58022	-1,65934	NaN	NaN	NaN
NaN	0,196743	NaN	-0,901405	-0,425339	-1,06601	0,861782	-0,762068	-0,266185	0,102941
-1,28379	-0,929039	NaN	-1,38175	-0,645824	-1,8834	-0,700874	NaN	NaN	NaN
0,368557	1,28665	-0,0925446	1,3561	NaN	1,2582	NaN	-0,808228	-0,6163	0,844321
NaN	NaN	NaN	-1,08982	-2,81358	-0,975168	-0,941919	NaN	NaN	NaN
NaN	NaN	NaN	NaN	NaN	NaN	NaN	NaN	NaN	NaN
NaN	NaN	NaN	-1,24363	-0,957445	-2,75931	-0,911461	-2,61038	NaN	-1,72589
0,195557	NaN	1,67322	2,22076	0,361055	3,06757	1,58105	0,84058	0,652179	0,142564
-3,04822	-1,13655	NaN	NaN	-2,00973	NaN	NaN	NaN	NaN	NaN

Polito et al. Universal ready-to-use immunotherapeutic approach for the treatment of cancer: expanded and activated polyclonal γδ memory T cells. Phosphorylation sites

-1,88095	-2,42506	NaN	-1,51884	-1,8602	-0,535593	-2,28962	NaN	NaN	NaN
NaN	NaN	NaN	-2,09432	NaN	-1,88699	-2,65469	NaN	NaN	NaN
0,773113	-0,898632	2,0579	0,285114	0,580662	3,01418	1,06044	1,05046	NaN	0,14541
NaN	NaN	NaN	NaN	NaN	NaN	NaN	NaN	NaN	NaN
NaN	NaN	NaN	NaN	-0,33646	NaN	NaN	NaN	NaN	NaN
1,75259	1,5248	-1,10293	NaN	1,93133	NaN	NaN	NaN	NaN	NaN
NaN	0,569578	0,0411644	1,32401	0,228014	6,09624	1,26397	0,120668	-0,483115	1,28724
NaN	NaN	NaN	-2,38032	-3,52984	-2,02517	NaN	NaN	NaN	NaN
0,096096	-0,617702	-0,562134	-0,651865	0,00167656	-0,175777	-1,01433	0,334261	0,00591564	-0,22947
NaN	NaN	NaN	NaN	NaN	NaN	NaN	NaN	NaN	NaN
0,168009	0,467371	1,51661	0,266836	0,793039	-4,60648	0,110357	0,590149	1,0883	0,539596
NaN	NaN	NaN	NaN	NaN	NaN	NaN	NaN	NaN	NaN
-0,092474	-1,82175	0,616766	-2,00772	-0,114241	NaN	0,535975	1,09271	0,980487	0,966676
NaN	NaN	-1,09268	-1,66822	NaN	-1,16456	-1,75529	-1,72713	-2,68075	-2,44213
NaN	NaN	-0,06814	1,84514	NaN	0,911987	-0,148535	NaN	NaN	NaN
-1,63381	NaN	2,37656	3,96708	-0,343801	2,44431	3,34696	NaN	-0,0136614	0,336089
NaN	NaN	NaN	NaN	1,10059	NaN	NaN	1,47429	1,09734	1,49805
-1,08178	-1,67709	-1,24281	-2,37726	-0,863611	NaN	-1,9726	-0,310896	-1,11179	-1,14734
-0,78614	0,809793	-0,308567	-1,2903	0,662319	3,2656	-0,154692	-0,715574	-0,648246	0,16302
NaN	NaN	NaN	NaN	NaN	NaN	NaN	NaN	NaN	NaN
NaN	NaN	NaN	-1,70209	NaN	-0,0793133	-2,61513	NaN	NaN	NaN
NaN	NaN	NaN	NaN	NaN	NaN	NaN	NaN	NaN	NaN
NaN	NaN	NaN	NaN	NaN	NaN	NaN	NaN	NaN	NaN
NaN	NaN	NaN	NaN	NaN	NaN	NaN	NaN	NaN	NaN
NaN	NaN	NaN	NaN	NaN	NaN	NaN	NaN	NaN	NaN
2,30097	1,55258	2,10206	2,73749	3,09134	2,58246	2,99675	3,27755	2,58387	2,64698
5,18436	4,6344	4,2259	3,04888	5,93213	2,21276	3,12052	4,3358	3,99921	4,89347
NaN	NaN	NaN	-1,10991	NaN	-3,40289	-0,36331	NaN	NaN	NaN
4,47247	3,60135	3,80192	5,90595	4,71981	6,33564	5,0863	3,64038	3,69724	4,91827
0,249519	0,476357	0,834826	2,89752	0,96755	1,9824	2,46503	2,58992	2,28846	2,50202
0,145645	NaN	-0,185326	-0,766298	-1,38294	0,807201	-2,49474	0,893232	0,322301	-0,482663
-2,11296	-2,34231	NaN	-1,19442	-2,13314	-1,54298	-1,8575	-1,93907	-1,88906	-2,1258
-0,0771332	NaN	0,528852	0,463383	0,715284	NaN	1,24675	-0,10063	-0,59257	0,270005
NaN	NaN	1,39647	0,820059	NaN	0,952862	1,03471	0,903803	NaN	0,452672

Polito et al. Universal ready-to-use immunotherapeutic approach for the treatment of cancer: expanded and activated polyclonal γδ memory T cells.
Phosphorylation sites

NaN	NaN	NaN	NaN	NaN	NaN	NaN	NaN	NaN	NaN
-1,11456	-0,961828	-0,187906	-0,672197	-1,22852	-0,685621	-0,232029	-0,74057	-0,611497	-0,89644
0,569328	-2,42958	-2,17845	NaN	NaN	NaN	NaN	-1,01416	NaN	NaN
-1,9505	-0,801952	-1,55424	-0,50252	NaN	-2,56767	-2,1842	-1,07214	-0,617148	-0,918393
0,981331	0,731644	0,831375	1,68846	0,442146	-0,289505	0,924822	1,79897	1,66834	1,17118
NaN	NaN	NaN	NaN	NaN	NaN	NaN	NaN	NaN	NaN
NaN	NaN	NaN	-2,12166	NaN	NaN	-1,96638	-2,41161	-2,35691	NaN
-2,23018	-0,411175	-1,06718	NaN	-0,810188	-0,942835	NaN	-0,768724	-1,08919	-0,843801
NaN	NaN	NaN	NaN	NaN	NaN	NaN	NaN	NaN	NaN
2,06873	3,93565	2,73708	0,89245	2,45407	2,64	2,63659	2,51667	2,84875	2,95085
NaN	NaN	NaN	2,33399	-1,7508	-2,04397	2,40166	2,91704	NaN	3,46403
4,9314	5,29212	3,62725	1,84263	3,53974	2,10228	1,64075	4,28834	4,10608	3,87863
NaN	NaN	NaN	NaN	NaN	NaN	NaN	NaN	NaN	NaN
NaN	NaN	NaN	NaN	NaN	NaN	NaN	NaN	NaN	NaN
0,395599	NaN	-0,460171	NaN	-0,323273	NaN	-1,12671	-1,04734	-1,18558	-1,02205
0,415483	-0,124657	1,07982	-0,327393	0,213694	0,219257	-0,363569	0,564844	-0,132751	-0,829089
NaN	NaN	NaN	NaN	NaN	NaN	NaN	NaN	NaN	NaN
-1,10757	NaN	0,38559	-0,301357	0,0779495	-0,706629	0,824032	0,739588	0,87597	-0,746455
NaN	NaN	NaN	NaN	NaN	NaN	NaN	NaN	NaN	NaN
-2,59096	-2,11078	-1,53108	-1,94948	-1,60668	NaN	-1,39605	-1,83592	-1,83413	-1,4561
NaN	-0,0279045	NaN	NaN	NaN	NaN	NaN	0,38048	0,404178	-5,95699
NaN	NaN	NaN	0,591145	-0,170856	1,07262	2,57454	NaN	NaN	NaN
NaN	-1,14924	-2,30074	-2,09495	-1,64058	-1,75663	-0,0322475	-2,08976	-2,62011	-1,94016
NaN	NaN	NaN	-2,35809	-1,19793	-2,72349	-0,761713	-1,26186	-1,45718	-1,21659
-0,571854	-0,652739	-0,183832	-0,035223	1,0298	-0,271544	1,3531	0,621469	0,489085	0,575288
NaN	NaN	NaN	-2,35809	-1,19793	-2,72349	-0,761713	-1,26186	-1,45718	-1,21659
2,95526	2,61634	3,21366	1,7144	3,65348	-2,13215	2,65505	3,12874	3,07481	3,66315
-1,43201	NaN	NaN	-0,529011	-0,821148	NaN	0,158783	NaN	NaN	NaN
-1,27194	-1,9836	NaN	-0,402645	-0,360313	NaN	-0,374073	-0,450344	-0,735667	-0,840304
0,924019	0,850536	-0,0120888	2,23965	2,1952	-3,17854	2,77453	1,43876	0,965232	1,20013
NaN	NaN	NaN	-1,6011	NaN	1,49943	-1,79552	NaN	NaN	NaN
NaN	NaN	NaN	NaN	NaN	NaN	NaN	-0,606777	-1,51035	-1,27626
-0,961199	-1,24879	1,83221	0,806145	-0,643246	-0,529734	0,979338	2,2147	1,93315	2,03765
NaN	NaN	4,32017	NaN	-0,151274	NaN	NaN	0,119362	-0,32583	0,732196

Polito et al. Universal ready-to-use immunotherapeutic approach for the treatment of cancer: expanded and activated polyclonal γδ memory T cells.
Phosphorylation sites

NaN	NaN	NaN	NaN	NaN	NaN	NaN	NaN	NaN	NaN
-3,54144	NaN	-3,5867	-1,66676	-2,45814	-1,91311	-1,4322	NaN	NaN	NaN
NaN	NaN	-0,440304	0,153652	-3,28885	0,3666	-0,474947	NaN	NaN	NaN
NaN	NaN	NaN	NaN	NaN	-0,882008	NaN	NaN	NaN	NaN
2,95989	2,2138	1,75694	1,97762	1,31048	0,386078	2,23137	1,62826	1,73325	1,33332
-2,07119	-1,13781	-1,39911	-0,13785	-0,760212	0,562216	0,271677	-0,8419	-1,69436	-0,824937
-2,50103	-2,51962	-2,77641	-2,25948	-1,67068	-2,79263	-1,91576	NaN	NaN	NaN
NaN	NaN	NaN	NaN	-3,03363	-1,2017	-1,81042	NaN	NaN	NaN
NaN	NaN	NaN	-1,93472	NaN	-2,40492	-1,97417	NaN	NaN	NaN
-0,0363007	-0,637777	-1,53396	-1,80309	0,100348	-1,43237	NaN	-0,807442	-1,14606	-0,986739
1,14671	1,40797	0,563257	NaN	1,17349	NaN	-2,05034	1,66491	1,36769	1,43469
0,773897	1,37849	0,205914	NaN	NaN	NaN	NaN	0,653852	0,990535	1,70557
NaN	NaN	NaN	-2,39474	NaN	-1,54605	-2,5153	NaN	NaN	NaN
NaN	NaN	NaN	0,333912	NaN	-0,118456	-0,250441	NaN	NaN	NaN
NaN	NaN	NaN	NaN	NaN	NaN	NaN	NaN	-3,12497	-3,01024
-0,389757	-0,273544	0,659662	-0,843887	0,870583	-0,794022	-0,819304	1,02666	NaN	0,389028
NaN	NaN	NaN	NaN	NaN	NaN	NaN	NaN	NaN	NaN
NaN	NaN	NaN	NaN	NaN	NaN	NaN	NaN	NaN	NaN
NaN	NaN	NaN	NaN	-1,98678	-3,27942	-1,51249	-2,64338	-2,35932	-2,55938
-2,57997	-1,9753	-1,35338	NaN	-2,38884	-2,49271	-1,28379	-0,779345	-0,719064	-1,018
NaN	NaN	NaN	NaN	NaN	NaN	NaN	NaN	NaN	NaN
NaN	NaN	NaN	-0,223295	NaN	-0,480064	0,720154	NaN	NaN	NaN
NaN	NaN	NaN	NaN	NaN	0,556524	-0,0491409	NaN	NaN	NaN
NaN	NaN	NaN	NaN	NaN	NaN	NaN	NaN	NaN	NaN
-2,43617	0,238012	0,435907	NaN	-1,61075	NaN	1,39559	-0,349464	NaN	-0,0509043
-2,85117	-3,15693	-2,7956	-0,0203819	-2,19207	-0,0345631	-0,756645	NaN	NaN	NaN
0,369869	0,20933	0,60441	1,73735	0,544664	1,04152	1,6396	0,760895	0,0271826	0,456658
NaN	1,50907	NaN	NaN	NaN	NaN	NaN	2,79847	3,10418	3,02326
-3,78539	NaN	0,130835	-0,790545	-3,48853	NaN	-1,19608	-0,297623	0,549283	0,218827
0,653341	1,72067	1,36858	0,771244	-0,782536	NaN	1,20403	0,927076	0,469829	1,56022
NaN	NaN	NaN	-1,75026	-3,21957	NaN	-2,56878	NaN	NaN	NaN
-0,1364	-7,62933	2,56773	0,925539	0,951262	0,68096	1,53984	1,63261	2,27068	1,69745
-0,0307713	-1,23321	-0,0610905	-1,33941	-0,273794	-0,594049	-0,822422	0,200914	-0,451978	-0,11465
-0,194828	-0,368416	-1,06634	-1,36745	-0,423393	NaN	-0,49465	-1,23596	-1,91938	-1,05243

Polito et al. Universal ready-to-use immunotherapeutic approach for the treatment of cancer: expanded and activated polyclonal γδ memory T cells.
Phosphorylation sites

3,22712	3,2327	2,86455	2,6118	2,62917	3,00655	2,68965	2,81554	2,731	2,34209
NaN	NaN	NaN	-0,67942	-1,18935	-2,06784	0,0792618	NaN	NaN	-1,26535
0	-0,122944	-0,00447845	NaN	-0,611622	NaN	NaN	-0,734415	-0,61567	-0,91458
1,12712	1,60974	1,04871	1,52925	0,713396	1,42172	1,99691	1,4108	1,60165	1,33414
NaN	NaN	NaN	NaN	NaN	NaN	NaN	NaN	NaN	NaN
1,14571	-0,416613	0,970276	-0,0599022	0,667269	-0,68923	-0,117632	0,963602	0,95543	0,640027
NaN	NaN	NaN	-1,93233	NaN	-1,29763	-1,33891	NaN	NaN	NaN
-1,57152	-1,78119	-2,31586	NaN	-1,43303	NaN	-1,79374	-2,51531	-1,82946	-2,66693
NaN	NaN	NaN	-0,977257	NaN	-0,736006	NaN	-2,08351	-2,77503	-1,83497
-1,73227	0,328999	-0,0871315	0,728172	NaN	-0,941845	1,50562	-0,0730553	0,711997	0,529693
NaN	NaN	NaN	NaN	NaN	NaN	NaN	NaN	NaN	NaN
1,89802	1,07676	1,85834	2,16057	2,12699	2,36418	2,60185	2,35177	1,38977	2,24884
-1,25735	NaN	-1,97385	-0,456768	-0,269264	-0,531483	0,635511	NaN	NaN	NaN
NaN	0,694679	-0,261412	0,427202	NaN	-0,237873	NaN	-1,26587	-0,127095	-1,07729
NaN	-1,67098	-1,63278	0,31135	NaN	-1,04118	-0,0185547	NaN	-1,88216	NaN
NaN	NaN	NaN	NaN	NaN	-2,04892	-1,83468	NaN	NaN	NaN
-1,4002	NaN	-3,17883	-0,707483	-1,7029	-0,141006	NaN	NaN	NaN	NaN
0,208847	0,223677	0,766319	0,0852451	-0,718584	0,678492	0,761473	0,699001	0,709239	0,293639
0,141928	-0,731173	0,922541	0,454422	0,356911	0,341757	1,13162	-0,162403	-0,217774	-0,327025
NaN	NaN	NaN	NaN	NaN	NaN	-0,559664	-2,99869	-1,66233	-1,64113
-1,30189	-0,761221	-1,08039	-1,56112	-0,582718	NaN	0,0590038	-1,05576	-1,65379	-0,723765
0,7272	1,01667	1,15361	-0,179253	NaN	NaN	-0,0228157	-0,457348	0,498712	0,499347
0,280262	2,34494	1,39663	0,24378	1,67637	-0,371946	1,38194	1,74701	0,78618	1,76065
1,74102	1,59672	2,15032	1,85269	2,13769	2,65648	0,978771	3,6042	2,45716	2,60712
1,71644	0,999937	0,231903	NaN	1,46059	1,29437	-0,883116	0,212843	0,533643	0,162835
0,0345573	NaN	-1,07261	-0,00971985	0,193075	0,571278	-0,230062	NaN	NaN	NaN
1,26074	1,39298	1,03377	2,03742	1,35329	3,32624	0,597853	1,33669	1,32682	0,348128
-2,43641	-0,592796	-1,67223	NaN	NaN	NaN	NaN	NaN	NaN	NaN
-1,26061	-0,742935	-1,62139	NaN	NaN	NaN	NaN	NaN	NaN	NaN
-1,68744	-2,00908	-0,757881	NaN	-1,48326	NaN	NaN	-0,729362	-0,9311	NaN
2,70149	2,39066	2,77288	NaN	2,96442	NaN	1,00473	1,89054	1,77436	1,79141
1,52962	2,07069	1,57973	3,21743	1,9836	0,61614	1,75447	1,39248	1,54134	1,3062
NaN	NaN	-1,50504	-1,58226	NaN	0,476446	-1,99549	NaN	NaN	NaN
1,72497	3,55146	1,82786	2,13656	3,73929	3,00723	2,50711	1,63253	1,16923	2,34953

Polito et al. Universal ready-to-use immunotherapeutic approach for the treatment of cancer: expanded and activated polyclonal γδ memory T cells.
Phosphorylation sites

0,217108	0,154819	0,0979404	NaN	NaN	NaN	NaN	0,592848	0,244855	-0,00228405
5,73749	4,3562	5,13677	5,30644	5,56746	5,95304	4,92021	NaN	3,76275	3,64184
2,33318	NaN	NaN	NaN	2,13174	2,42652	NaN	1,69434	1,37197	1,49805
-1,40788	-1,22635	-1,05758	NaN	-0,565027	NaN	NaN	NaN	NaN	NaN
NaN	NaN	NaN	NaN	NaN	NaN	NaN	NaN	NaN	NaN
NaN	NaN	NaN	-2,6011	NaN	-1,97861	3,03102	0,216261	0,171216	-0,535571
NaN	NaN	NaN	-2,46974	NaN	NaN	-0,265055	NaN	NaN	NaN
-0,127716	-1,17268	2,18565	1,20235	-0,629148	0,790394	1,28127	0,847528	0,70308	0,904897
4,01004	5,46163	5,24999	3,96497	5,07064	3,03099	4,84055	4,69172	4,22151	4,89763
-0,279692	-1,03441	3,19998	3,91026	1,18629	4,46531	3,90868	2,02911	2,90805	2,3748
0,704916	-0,00056076	0,321203	1,2119	0,29888	1,67478	1,27427	0,934534	0,516971	0,33026
NaN	NaN	NaN	-0,69758	NaN	-0,341396	-1,18623	NaN	NaN	NaN
NaN	NaN	NaN	-1,25767	NaN	-0,474277	-1,32776	NaN	NaN	NaN
-0,439167	-0,446169	-3,53438	-1,39093	0,388531	NaN	NaN	-0,551409	-0,38915	NaN
1,87733	1,62755	NaN	1,02347	2,02555	1,29266	1,97315	NaN	NaN	1,41167
0,95854	NaN	0,353449	0,226351	1,71352	0,329697	0,91573	NaN	-0,924663	0,844321
NaN	-0,382908	NaN	-1,85981	0,539511	NaN	0,0756607	-1,08108	-1,51192	-0,708047
NaN	NaN	0,881136	-1,33876	NaN	-0,711056	-1,07494	-1,2696	-1,0881	-1,76873
1,18747	1,89612	1,37145	2,2159	2,65093	NaN	0,862028	1,79394	1,56691	1,65378
NaN	NaN	2,87615	NaN	-1,26093	1,65646	2,14214	0,122892	0,105273	-0,255067
0,896826	5,41886	2,88076	3,22456	4,94086	NaN	5,5077	2,47214	2,79678	4,69681
0,344254	1,25051	0,318996	0,603659	0,914009	0,835749	0,608429	0,505325	0,611817	0,248261
NaN	-1,07018	NaN	-1,39079	NaN	-0,129686	-1,37555	NaN	-1,79212	NaN
NaN	NaN	NaN	NaN	NaN	NaN	NaN	NaN	NaN	NaN
NaN	NaN	NaN	-1,14623	NaN	NaN	NaN	NaN	NaN	NaN
NaN	NaN	NaN	NaN	NaN	NaN	NaN	NaN	NaN	NaN
NaN	NaN	NaN	-3,17523	NaN	1,0482	0,535589	NaN	NaN	NaN
NaN	NaN	-0,208775	NaN	0,449724	NaN	NaN	0,010191	-0,282331	0,208138
NaN	NaN	NaN	NaN	NaN	NaN	NaN	NaN	NaN	-3,1872
0,845579	NaN	0,559811	2,05632	1,4066	NaN	1,20539	2,91353	1,33725	1,69936
NaN	NaN	NaN	NaN	NaN	-1,189	NaN	NaN	NaN	NaN
-0,878363	-2,2304	0,182161	-0,145494	0,105326	2,04457	0,451426	0,152479	-0,459235	0,132298
0,569569	0,408026	0,989727	NaN	NaN	NaN	NaN	0,904869	0,374965	1,10966
-1,49403	-1,2308	-0,541101	-0,527792	-0,568777	NaN	-0,247015	-0,233404	-0,214963	-0,581569

Polito et al. Universal ready-to-use immunotherapeutic approach for the treatment of cancer: expanded and activated polyclonal γδ memory T cells.
Phosphorylation sites

-1,73524	NaN	-0,970064	NaN	NaN	NaN	NaN	-2,31623	-1,57047	-3,06859
1,26232	0,862753	0,403088	2,32635	1,18642	3,32743	2,53028	0,720943	1,55474	0,699643
NaN	NaN	NaN	-1,9611	-1,39351	-2,70329	-1,7193	NaN	NaN	NaN
1,69413	1,68153	0,640211	0,391212	1,40241	NaN	0,777336	0,158861	0,0888987	0,443927
0,915998	1,80713	0,322916	0,933743	2,06032	1,23212	0,910746	0,144129	-0,549972	-0,41052
-0,707676	NaN	-1,57146	-0,192804	-2,03223	-1,36403	-2,52697	-3,0995	-3,20522	-2,75503
NaN	-0,100172	NaN	0,000459671	0,704124	1,54893	-0,0456543	NaN	NaN	0,232017
-1,93404	-1,94896	-2,21489	-1,2065	-1,71676	-0,496151	-0,612873	NaN	NaN	NaN
-1,09683	-1,6032	-1,84292	-1,41656	-1,01059	-1,56178	NaN	-2,23414	-1,93828	-1,87751
NaN	NaN	NaN	-1,53558	NaN	NaN	-1,7067	NaN	NaN	NaN
NaN	NaN	NaN	2,53618	-2,67631	2,99248	1,46061	NaN	NaN	NaN
NaN	NaN	NaN	NaN	NaN	NaN	NaN	NaN	NaN	NaN
3,88222	3,21818	2,67461	3,27906	3,0078	3,71048	3,29556	3,5907	3,68898	NaN
0,29591	0,578264	0,267399	0,0646858	0,499706	NaN	-0,274431	1,04128	0,626903	0,774802
-1,93557	-2,42891	-1,69463	-1,49766	-0,616495	-1,42139	-1,18592	-0,986273	-1,11152	-0,787379
NaN	-1,80805	NaN	-1,61511	0,0632477	-3,04654	-1,48627	-1,40123	NaN	-1,07444
NaN	NaN	NaN	NaN	NaN	NaN	NaN	NaN	NaN	NaN
NaN	NaN	NaN	NaN	NaN	NaN	NaN	NaN	NaN	NaN
NaN	-2,22056	-1,41694	NaN	-0,0118198	NaN	-1,30555	-1,45424	-1,62991	-1,23765
0,644802	-0,448782	0,745358	-0,138193	1,10997	-1,65739	NaN	1,25784	1,17769	1,04773
4,27702	4,20467	3,10588	3,23477	3,63404	-0,453697	2,10808	2,99498	2,65534	2,9692
5,0955	4,72772	4,5984	4,64865	4,91612	-0,374523	4,29251	4,75682	4,35086	4,16995
NaN	NaN	NaN	NaN	NaN	NaN	NaN	-1,25226	-1,39324	-1,38108
5,95954	5,65782	1,38945	5,9205	1,52642	6,43978	4,93153	5,67834	2,0092	1,26536
-0,240059	0,463421	NaN	-0,303148	0,655584	-0,630671	-0,258652	0,37245	NaN	NaN
NaN	NaN	-0,324696	NaN	NaN	NaN	NaN	-0,255953	-0,154159	-0,105165
NaN	-2,4213	0,264931	-2,68964	-1,64751	NaN	-0,882128	0,872538	0,629821	0,547027
1,60837	1,75154	1,3892	0,861118	2,36454	NaN	0,971724	1,34781	0,812108	1,50768
NaN	-1,09	-1,33541	0,340054	NaN	NaN	0,577181	0,0479565	-0,127543	NaN
2,20296	1,52377	-1,14938	NaN	0,868078	NaN	NaN	NaN	NaN	NaN
NaN	NaN	-0,993675	NaN	NaN	NaN	NaN	-1,01497	-1,26291	-1,65364
0,649931	0,514589	0,307909	-1,05254	0,479782	-1,5048	-1,30754	0,59164	0,0721998	-0,118106
-0,149561	-0,483253	NaN	-0,147001	-0,291954	-0,459503	NaN	NaN	NaN	NaN
-0,624252	-0,531796	-1,58018	-0,262714	-0,952595	0,112238	-0,821474	-1,1062	-1,55334	-1,22713

0,472818	-0,0422211	0,357409	-0,0879765	0,866692	0,104458	-0,258892	-0,101727	-0,157563	-0,291034
0,468065	NaN	0,769804	NaN	1,07895	-0,371063	NaN	0,261574	0,223163	0,234145
0,369404	NaN	0,613859	0,274773	0,953217	-0,0922871	0,851788	0,800329	0,739158	1,30398
1,16096	1,90771	1,31071	0,987112	2,09753	0,743208	0,77198	1,02446	0,857184	1,10507
-2,58505	-3,98914	-2,91276	NaN	-2,90992	NaN	-2,46211	NaN	-1,74145	-3,24715
0,0981617	NaN	-1,621	NaN	0,161087	NaN	NaN	-1,31474	-0,983949	-0,609658
NaN	NaN	NaN	NaN	NaN	NaN	NaN	NaN	NaN	NaN
1,11769	1,94654	0,948584	1,0862	-0,00153732	NaN	1,76779	0,300777	0,992702	1,13756
-0,156559	-1,4651	-1,40191	NaN	-0,67186	NaN	NaN	NaN	NaN	NaN
NaN	NaN	NaN	NaN	-3,36031	-0,483076	-2,10534	NaN	NaN	NaN
NaN	-0,66778	-1,21681	NaN	NaN	0,352215	0,274561	-0,306664	-0,593932	-0,688941
NaN	NaN	NaN	-0,414625	NaN	-0,513288	-1,18496	NaN	NaN	NaN
-2,17683	-3,23549	-0,634886	-0,925749	-2,10107	-0,467583	-1,71394	-0,7817	-0,853269	-1,1259
NaN	NaN	-0,592688	NaN	-2,42504	-2,9949	-3,37038	-1,32996	-1,28079	-1,05252
-1,77098	-1,55155	-1,74248	NaN	-1,09735	NaN	NaN	-1,57084	-2,26158	-1,27071
NaN	NaN	NaN	0,248091	NaN	1,09876	-0,277571	NaN	NaN	NaN
-2,26602	-3,03687	-3,2494	-3,0861	-2,71398	-3,53528	-2,17822	-2,38713	-2,13881	-2,14414
NaN	NaN	NaN	NaN	NaN	NaN	NaN	NaN	NaN	NaN
NaN	-3,08273	-1,79278	-1,09939	-2,24406	NaN	-2,20806	NaN	NaN	NaN
0,531315	0,253908	NaN	NaN	0,884033	-0,487406	-0,334564	NaN	0,0250483	0,689717
0,24732	0,51655	-0,0978203	-0,510746	1,00791	-0,090498	0,00270271	1,01289	0,254413	0,418843
NaN	NaN	NaN	-1,40649	-2,81586	-0,307856	-2,78872	NaN	NaN	NaN
-0,287027	1,18631	0,650644	1,53738	1,92782	-0,419975	1,53105	0,726744	0,0807772	1,27714
NaN	-3,03266	-2,8843	-1,91289	-1,99866	NaN	-1,67296	NaN	NaN	NaN
1,43224	1,37669	1,25701	1,62938	1,57705	1,9786	1,45516	1,33995	1,49872	0,945621
1,48335	0,33358	1,93767	0,475729	0,676157	0,820698	0,519669	2,1644	2,03059	1,97166
1,13771	NaN	-0,694353	NaN	NaN	NaN	NaN	1,7881	1,67766	1,56069
4,98101	3,04925	4,94166	NaN	4,77205	NaN	NaN	5,69927	5,19633	NaN
NaN	NaN	0,623247	1,56852	1,00621	2,25499	1,08319	0,29471	-0,0193663	0,00884342
4,82808	4,78213	5,18033	4,93775	5,24181	2,12727	5,16838	5,28662	5,06972	4,97037
-0,288879	-0,172207	-0,288002	0,905365	-0,334057	-0,566406	0,192478	7,44E-05	-0,245442	-0,578139
3,16848	3,4492	3,54438	3,63525	3,50045	2,89845	3,52739	3,30789	3,09942	2,91704
0,22324	0,271049	0,579945	-0,12118	-1,52561	-0,0306416	-0,180773	-0,722389	0,110507	-0,315127
3,88993	NaN	NaN	NaN	NaN	1,10083	3,40315	NaN	NaN	NaN

NaN	4,02304	5,28336	3,5484	5,14332	NaN	NaN	5,09398	4,7996	5,12706
1,39572	1,05773	1,71578	0,373869	2,75761	-1,87987	0,518343	1,72929	1,3753	2,52282
-0,121099	-0,949532	0,836845	-0,177818	-1,28879	-0,720739	-1,06154	NaN	-0,10476	-1,05381
3,26681	2,18877	2,78729	1,1666	3,20081	NaN	0,0625591	3,03983	2,45884	2,25289
-1,70786	-0,143686	-1,8028	-1,61663	NaN	-0,414038	-1,02511	-2,38778	-1,17004	-2,4645
NaN	NaN	-1,51585	-0,692997	NaN	2,29102	-0,921501	NaN	NaN	NaN
3,57559	3,59685	4,24749	4,80399	4,15913	3,9611	4,03692	3,60682	3,37658	3,68235
NaN	NaN	NaN	NaN	NaN	NaN	NaN	NaN	NaN	NaN
NaN	NaN	NaN	-1,08271	-2,28921	-2,16693	-1,0272	-2,46638	NaN	-2,32137
-1,61626	-0,748604	-1,22813	-1,99734	-1,03905	-0,726181	-1,24392	-1,69399	-1,08313	-1,34624
NaN	NaN	NaN	-0,68541	-1,36833	NaN	0,0375004	NaN	NaN	NaN
-0,339918	-0,887148	-0,593742	-0,759872	-1,19298	1,2125	NaN	NaN	NaN	NaN
NaN	NaN	NaN	NaN	NaN	NaN	NaN	NaN	NaN	NaN
3,17936	3,23065	2,4393	4,00513	2,8964	3,70111	4,10707	2,77205	2,39594	4,0819
-1,05575	-2,07542	0,515244	0,534401	-0,904913	1,31455	0,998928	-0,763634	-1,28009	-1,17801
-0,110626	-0,619785	0,231163	0,413052	-1,23399	1,18583	0,312233	-0,671844	-0,0293779	-0,52414
-1,32123	0,471645	0,231144	0,673206	0,829607	2,98449	0,898928	-0,836454	-0,680621	0,183576
NaN	NaN	NaN	NaN	NaN	NaN	NaN	NaN	NaN	NaN
NaN	NaN	NaN	NaN	NaN	NaN	NaN	-1,94214	-2,48596	-0,893283
NaN	NaN	NaN	-0,752266	-2,66943	NaN	-1,72188	-2,12828	-2,5026	-1,24339
4,29657	4,28766	5,01992	3,05851	5,17585	3,05044	2,89932	5,38743	5,04658	5,04942
NaN	NaN	NaN	NaN	NaN	NaN	NaN	NaN	NaN	NaN
-1,4415	-0,347015	-0,423918	-0,379049	-0,499289	-0,84919	-1,01374	-1,00547	-1,73604	-1,7896
1,84843	1,86865	NaN	0,389669	NaN	0,541616	0,393333	NaN	NaN	NaN
NaN	NaN	2,30215	NaN	1,90318	NaN	NaN	2,16901	1,76967	1,91211
NaN	NaN	1,01386	0,0286484	NaN	-0,10615	-0,188723	0,0654144	-0,242188	-2,07193
NaN	2,21505	NaN	NaN	NaN	NaN	NaN	NaN	NaN	NaN
2,49444	0,165833	2,14021	NaN	NaN	NaN	NaN	1,09388	3,09696	NaN
NaN	NaN	NaN	-2,17337	NaN	-1,39887	NaN	NaN	NaN	NaN
3,11236	2,20815	2,91421	3,81461	NaN	3,784	3,57336	NaN	1,64832	2,24609
NaN	NaN	NaN	-0,718269	-2,44152	0,0654793	0,189816	NaN	NaN	NaN
NaN	NaN	NaN	-1,27487	-1,28034	NaN	-1,01176	NaN	NaN	NaN
NaN	NaN	NaN	-2,70418	-1,57991	NaN	-2,03974	NaN	NaN	NaN
0,602062	-0,980211	0,0264015	1,30672	-1,3143	-1,0218	0,647829	-0,33341	0,36888	-0,734569

-3,03909	-2,72428	-0,724297	NaN	NaN	NaN	NaN	-0,607157	-0,547631	-0,56416
NaN	NaN	-0,21076	NaN	NaN	NaN	NaN	-1,02997	-0,852269	-1,0893
-1,26806	-0,625469	0,41374	-2,98227	-0,510344	-2,10984	-2,40798	1,0357	0,962287	0,763492
0,697504	0,636744	0,681501	-0,193266	1,12465	0,941177	0,113852	1,2478	0,973716	0,421655
NaN	NaN	NaN	-1,7049	-2,98316	-2,29963	-1,32602	-2,02143	-3,05959	NaN
NaN	NaN	NaN	-1,45364	NaN	-1,95268	-0,846771	NaN	NaN	NaN
NaN	NaN	NaN	-0,535364	NaN	-1,57183	-1,85556	NaN	NaN	NaN
NaN	NaN	NaN	-1,61162	NaN	-2,43402	-0,938704	-2,80557	-3,10421	-1,24214
NaN	-1,40343	NaN	0,114166	NaN	1,15209	NaN	NaN	NaN	NaN
NaN	NaN	NaN	-1,5459	-2,02909	-1,38063	-1,28651	NaN	NaN	NaN
NaN	NaN	NaN	-2,29089	NaN	1,07194	-1,31219	NaN	NaN	NaN
NaN	NaN	NaN	-1,77143	NaN	-0,391258	-2,5729	NaN	NaN	NaN
NaN	NaN	NaN	-0,52623	NaN	-0,180117	-0,43078	NaN	NaN	NaN
-0,844662	-1,13761	-0,810888	-0,425646	-0,373772	NaN	-0,0426369	-0,679335	-1,0135	-1,17475
-0,968824	NaN	-0,23134	0,48941	-0,460041	0,654863	0,740358	-0,748133	-0,81387	NaN
NaN	NaN	NaN	2,25004	NaN	2,37812	2,9345	NaN	NaN	NaN
NaN	NaN	NaN	-1,74082	NaN	-0,835226	-1,61808	NaN	NaN	NaN
NaN	NaN	NaN	-2,23452	NaN	-3,40975	-1,91171	NaN	NaN	NaN
NaN	NaN	NaN	-2,4596	NaN	-1,14654	-1,45634	NaN	NaN	NaN
2,62366	2,96094	1,87288	2,21123	2,59447	2,92415	1,44961	2,17062	2,18162	2,46725
0,399937	0,120983	-0,950026	-0,0129242	0,110128	1,03352	-0,365267	-0,250631	-0,779176	-0,324437
1,37642	1,96245	1,52068	1,6468	1,80733	-0,365973	2,18744	1,76803	1,37277	1,54402
NaN	NaN	NaN	NaN	NaN	NaN	NaN	NaN	NaN	NaN
NaN	NaN	NaN	NaN	-3,91173	NaN	-1,99389	NaN	NaN	NaN
2,57505	1,68473	1,42034	-0,138964	1,59165	NaN	-0,826855	-0,667599	NaN	0,153602
NaN	0,12084	NaN	-1,29435	3,5701	-0,9832	-1,14038	NaN	NaN	NaN
NaN	NaN	NaN	NaN	NaN	NaN	NaN	NaN	NaN	NaN
0,150259	1,04925	0,464762	1,14847	1,2394	-0,482056	1,25726	0,271114	NaN	0,0263453
-0,307487	NaN	-2,05529	NaN	NaN	2,39481	0,901203	NaN	NaN	NaN
0,568621	-0,605303	0,602037	1,47149	0,282701	-1,07944	1,94144	0,740856	NaN	0,543315
NaN	NaN	-1,00716	-2,84039	NaN	-3,88867	-0,280552	NaN	NaN	NaN
1,751	1,94103	0,569414	0,854628	1,29874	-0,994307	0,201233	1,22536	0,618379	1,1039
3,28563	2,87346	3,14755	1,3882	2,71478	2,29764	1,42035	3,45559	3,64775	3,15872
-1,56055	NaN	-1,12861	NaN	NaN	-0,0261192	NaN	NaN	-0,181575	NaN

Polito et al. Universal ready-to-use immunotherapeutic approach for the treatment of cancer: expanded and activated polyclonal γδ memory T cells.
Phosphorylation sites

-0,827766	-0,269989	-0,796642	0,917095	-1,00122	2,76259	0,47097	NaN	NaN	-1,19368
NaN	NaN	NaN	-0,946146	NaN	-1,16214	-2,65384	NaN	NaN	NaN
NaN	NaN	2,6151	2,59489	2,4906	1,70634	2,67321	3,07988	2,68268	3,01204
-0,0247192	-1,26813	-0,655872	-0,831013	-0,281998	-0,987875	NaN	0,429142	-0,46786	-0,127627
1,36883	0,0501976	0,753302	NaN	0,00813675	NaN	NaN	0,714193	1,3035	-4,83335
NaN	-3,89274	3,51918	2,85211	-2,71342	1,92008	3,43551	-0,96027	-1,72896	-1,29996
NaN	NaN	1,51861	0,350559	NaN	0,11722	1,14674	NaN	NaN	NaN
NaN	NaN	2,08787	-0,0414333	NaN	0,657297	1,62243	NaN	NaN	NaN
5,63256	4,29736	4,8323	3,6192	3,80729	1,07927	3,60731	5,1483	5,39262	4,79787
NaN	-3,49117	1,91929	0,867178	NaN	-1,50452	1,10382	NaN	NaN	NaN
NaN	NaN	-1,28325	-1,0572	NaN	1,09276	-0,763685	NaN	NaN	NaN
NaN	NaN	0,0567169	0,399179	NaN	-0,14776	0,391188	NaN	NaN	NaN
2,29703	2,13548	2,50579	2,75546	3,29622	0,681782	2,29723	3,083	2,91432	3,10444
-0,919271	-0,292957	-0,128263	-0,381298	-1,00601	NaN	0,710762	-0,196838	-0,506961	-0,152282
4,99108	4,25841	5,42951	4,89959	5,51253	-0,933651	5,00561	6,1037	5,61914	5,48113
NaN	-2,91085	3,38324	1,76395	NaN	NaN	2,62388	-0,988596	-0,559007	-1,11985
-0,0257416	NaN	1,88968	1,3145	-0,441736	1,59285	-0,30056	1,26492	0,98495	0,808213
1,94591	0,273373	2,36208	-0,252802	2,1318	0,0818768	NaN	0,993977	0,883548	NaN
NaN	-1,81949	-0,831787	-1,43752	NaN	NaN	-1,94687	-2,0986	-3,87786	-1,89747
-2,26141	-0,519819	-2,00933	-2,36745	NaN	NaN	-1,34299	NaN	1,31401	-1,05944
1,44164	1,00212	1,90782	0,746037	1,46688	-0,443981	0,512732	2,05881	0,223374	0,614234
-1,44611	NaN	1,96143	0,870096	-1,25372	1,43756	0,99848	-0,874086	-0,988215	-0,83166
-2,08088	NaN	-0,535929	NaN	-1,29359	NaN	-3,15305	-2,75461	-2,51684	-1,92762
-1,71136	-3,35818	-1,66354	NaN	-3,16298	NaN	NaN	NaN	NaN	-2,87504
1,07033	NaN	-0,0214386	0,99691	-1,47715	3,58228	0,453108	0,0702209	-0,609712	-0,343587
NaN	NaN	NaN	-0,421232	-1,55246	0,649296	0,754734	NaN	NaN	NaN
-0,753941	-2,21947	1,33805	-0,971874	-0,479095	-1,70446	-0,132547	-0,764099	-0,96161	-0,538934
0,323677	0,181852	-0,812599	0,409042	NaN	0,879917	-1,12256	-1,54492	-1,42268	-1,38036
NaN	NaN	NaN	NaN	NaN	NaN	NaN	NaN	NaN	NaN
3,14946	2,1142	4,01357	4,17034	2,39545	4,64307	3,59136	2,65848	2,72756	2,15235
NaN	NaN	2,77965	1,49803	NaN	NaN	2,01533	-0,102852	-0,410525	-0,299989
1,44476	3,77047	3,52685	-1,22585	2,58891	-1,5504	-1,69139	2,95462	2,69901	NaN
2,69664	0,654766	2,0744	-2,07646	NaN	-0,608137	NaN	NaN	NaN	-0,697253
3,5146	4,15167	3,93204	4,17157	4,28479	3,8228	4,78344	4,73113	4,65682	4,53479

Polito et al. Universal ready-to-use immunotherapeutic approach for the treatment of cancer: expanded and activated polyclonal γδ memory T cells.
Phosphorylation sites

-3,82191	-4,1666	0,460363	-0,637354	-2,4993	-1,89574	0,457851	NaN	NaN	NaN
-1,38719	-2,35524	-0,418043	-0,840988	-1,41298	-0,860634	NaN	0,544466	-0,222362	-0,536635
NaN	-0,430191	0,310818	0,230101	-1,00413	-0,128508	1,26369	-0,636402	-0,395637	NaN
-2,98116	NaN	-0,736782	-3,65114	-1,40235	NaN	-1,45004	NaN	NaN	NaN
-1,30928	NaN	-0,48624	0,266483	-0,211596	0,732357	0,740358	-0,856413	-1,21071	-1,14257
NaN	NaN	NaN	NaN	NaN	NaN	NaN	-1,93125	-2,48114	-1,85235
NaN	NaN	-1,14291	-1,26062	NaN	NaN	-2,01207	-0,509335	-0,983827	-1,40903
-1,93225	-1,90297	-0,386833	-0,0638733	-2,09973	NaN	-1,42048	0,266249	0,112937	-0,00638103
2,0481	0,328007	2,39025	2,07472	2,21866	0,047039	2,31727	2,68572	2,49858	2,5406
-0,215569	-0,278429	-0,401821	0,105412	NaN	-1,74133	-2,07358	-1,82554	0,286984	-1,66335
-4,02898	-4,26447	-2,02478	NaN	NaN	NaN	NaN	NaN	NaN	NaN
-0,00866508	-0,551685	0,245251	0,543762	-0,319897	1,14138	0,385929	0,93675	0,649304	0,461032
0,529182	-1,40326	3,32673	2,64133	-0,845617	3,78479	3,10434	-0,0749454	-0,354098	0,00273991
-3,77295	NaN	-2,29106	-1,36064	-2,56882	NaN	NaN	-1,69528	-1,11912	-1,85244
-2,5228	NaN	0,199524	-0,244808	-1,34034	NaN	-0,714855	1,31931	1,48773	0,947644
NaN	NaN	-1,74952	NaN	NaN	NaN	NaN	-0,276834	-1,4288	-0,299876
NaN	NaN	NaN	NaN	NaN	-0,978613	NaN	-0,868248	-1,0947	-0,937341
-0,725391	NaN	0,846897	-0,35313	NaN	0,158926	-0,982496	-0,225334	1,23152	-0,155652
NaN	NaN	NaN	NaN	NaN	NaN	NaN	-2,00923	-1,92794	-0,529397
NaN	NaN	NaN	NaN	NaN	NaN	NaN	NaN	NaN	NaN
NaN	NaN	NaN	0,664883	NaN	-1,17548	0,123606	NaN	NaN	NaN
NaN	NaN	NaN	NaN	NaN	NaN	NaN	NaN	NaN	NaN
NaN	NaN	NaN	NaN	NaN	-1,93736	-2,61609	NaN	NaN	NaN
NaN	0,514589	NaN	1,04242	NaN	-0,117935	2,36759	-0,103039	-0,153176	NaN
NaN	NaN	NaN	-2,51001	-2,48282	-2,24101	-2,60395	NaN	NaN	NaN
NaN	NaN	-2,618	NaN	NaN	NaN	NaN	NaN	NaN	NaN
-0,946543	-1,578	-1,24286	-1,03037	-1,29974	-1,57582	-1,25774	-2,43907	-1,4538	-1,1203
NaN	NaN	NaN	0,387705	-2,41501	0,0401802	-1,57051	NaN	NaN	NaN
NaN	NaN	NaN	NaN	NaN	NaN	NaN	-1,83006	-2,28758	-1,85202
-0,0672779	-0,824894	-1,29693	-0,481476	0,124126	NaN	-1,05104	NaN	NaN	NaN
0,0175705	0,00300407	0,687963	1,15855	3,82513	-0,364199	1,71499	1,10273	-0,284852	1,35502
0,0532646	-1,06206	0,169815	-0,296179	0,737608	0,491356	0,293831	0,418413	0,398309	0,455905
NaN	NaN	NaN	-2,21263	NaN	-1,73956	-0,280607	NaN	NaN	NaN
NaN	0,35615	NaN	0,322727	0,821152	0,633625	0,714853	NaN	NaN	NaN

Polito et al. Universal ready-to-use immunotherapeutic approach for the treatment of cancer: expanded and activated polyclonal γδ memory T cells.
Phosphorylation sites

NaN	0,394815	NaN	1,1227	NaN	NaN	0,157923	1,03509	NaN	0,782294
NaN	NaN	NaN	NaN	NaN	NaN	NaN	NaN	NaN	NaN
-0,892546	NaN	NaN	NaN	0,0321655	-1,94514	NaN	-1,20119	-0,12383	-0,4528
-0,445353	NaN	0,25034	-0,566469	1,34021	NaN	0,209682	NaN	NaN	NaN
NaN	NaN	-0,548244	-2,08275	-4,66934	-1,32331	-1,52266	NaN	NaN	NaN
0,393511	-0,183132	-0,462769	1,30813	0,971746	1,18846	1,27768	1,03229	0,349502	0,163758
0,657026	0,169437	0,544823	0,357893	0,86474	1,20545	1,1431	0,24416	0,385194	0,445226
NaN	-1,91931	-1,10416	NaN	NaN	-2,38531	-1,16057	NaN	NaN	NaN
0,934334	0,306501	3,43494	2,6832	-1,71005	3,12144	3,14199	NaN	2,074	1,85686
NaN	NaN	NaN	NaN	NaN	NaN	NaN	NaN	NaN	NaN
NaN	NaN	NaN	-2,44992	-0,841076	-1,22213	NaN	NaN	-1,87899	-1,80035
-1,81829	-3,2867	0,176882	-1,00647	-0,313559	0,694872	-0,0676422	-0,353155	-1,07322	-0,906772
-2,55956	-0,69375	NaN	-0,490822	-0,439806	NaN	-0,320431	-0,584949	-0,573707	-0,487611
NaN	NaN	NaN	NaN	NaN	NaN	NaN	NaN	NaN	NaN
-0,0180588	-0,911234	-0,178881	-0,681011	0,418835	-1,20112	-0,606327	-0,440123	-0,435132	-0,356017
NaN	NaN	NaN	-3,66565	-2,30542	NaN	-2,40029	NaN	NaN	NaN
-1,90349	-1,0688	-1,812	-1,68941	-1,98307	NaN	-1,93255	-2,27847	-2,11152	-2,31085
NaN	NaN	NaN	-1,41681	NaN	-0,776098	-1,54877	NaN	NaN	NaN
NaN	NaN	-1,20914	-1,00197	-1,1854	-0,631804	-0,998838	-0,763166	-1,60132	NaN
0,791155	1,22607	1,25443	1,22108	1,31071	1,46776	1,16808	1,36757	1,56052	1,47371
NaN	NaN	NaN	-1,9586	-3,22809	NaN	-1,39699	NaN	NaN	NaN
-0,413521	-0,953207	-0,956839	-0,147511	-0,0544701	0,105349	-0,262802	-1,47371	-1,17239	-1,29782
1,17803	1,30164	0,979765	1,40238	1,65441	1,37328	0,650963	0,538658	-0,238265	0,216043
NaN	NaN	NaN	NaN	1,39454	2,66597	1,88223	0,956423	NaN	0,863873
-2,95214	-1,45827	-1,15627	-1,08185	-0,1397	NaN	-0,517052	-2,32883	-2,35397	-1,36008
-0,278446	-1,05075	-1,24225	-1,49896	NaN	NaN	-1,1394	-1,05043	-1,36608	-1,77893
1,14943	1,72836	1,17104	0,861523	NaN	0,881609	1,79631	0,88839	1,28055	1,61564
0,128984	0,58897	-0,552217	-0,934189	0,495819	-0,8512	-0,737478	-0,356855	-0,98326	-0,571134
NaN	NaN	0,399155	-1,11667	-2,39233	-0,786995	-2,01957	0,616158	-0,117742	-0,380126
-0,487917	-0,291203	-0,925581	-1,92442	NaN	NaN	NaN	-1,31592	-1,19227	-0,997283
NaN	NaN	NaN	1,53288	-1,83797	NaN	NaN	NaN	NaN	NaN
NaN	1,15594	0,697536	1,20458	NaN	-1,50255	1,21985	NaN	0,384282	0,295659
-2,32676	-1,18467	-1,15225	NaN	NaN	NaN	NaN	NaN	NaN	NaN
1,17566	1,06861	1,0923	1,27441	1,4244	1,44668	1,39568	0,912706	0,731645	0,973937

Polito et al. Universal ready-to-use immunotherapeutic approach for the treatment of cancer: expanded and activated polyclonal γδ memory T cells.
Phosphorylation sites

-0,662708	-0,539541	-0,555983	0,187832	-0,162846	-0,113392	-0,166056	-0,516809	-0,790944	-1,2767
NaN	NaN	NaN	0,0779476	NaN	-2,09943	-0,156908	NaN	NaN	NaN
-2,7374	-2,00382	NaN	NaN	NaN	NaN	NaN	-2,73842	-2,7163	-2,99158
2,3012	1,99873	2,41145	2,18244	2,78493	NaN	2,46507	2,4242	2,44975	2,19448
1,43174	0,88435	1,20775	1,0956	1,24265	-0,769976	0,803181	0,744076	0,854806	0,352252
-0,105862	-0,588737	-0,096777	-0,158197	-0,000597	0,207163	-0,387487	-0,280737	-0,569713	-0,279065
NaN	0,160267	NaN	0,219467	NaN	0,505014	-1,57689	-0,133688	-0,954478	-0,572873
3,17896	3,1978	3,19718	3,04242	3,37655	2,59793	3,33182	3,16929	2,92459	2,97633
-0,412956	0,897957	0,377079	1,00636	1,77977	0,761349	-0,409431	-0,0650997	-0,147918	NaN
NaN	NaN	NaN	-3,12973	-2,83726	-3,24675	NaN	NaN	NaN	NaN
NaN	NaN	NaN	-2,43941	-2,32808	NaN	-2,74262	NaN	NaN	NaN
6,03759	4,61119	5,2462	4,29852	5,41607	2,54815	4,858	5,7086	5,42935	5,0431
-1,04853	-1,89889	-2,70185	-0,743683	-0,0829754	NaN	1,08993	NaN	-0,125709	0,0675287
1,30838	1,23623	1,78446	4,43055	1,28142	3,28675	3,17565	0,474403	0,646949	0,188678
NaN	0,105341	NaN	NaN	NaN	NaN	NaN	-0,637955	-0,618155	-0,0180693
NaN	NaN	NaN	-1,99206	NaN	-2,10236	-1,98025	NaN	NaN	NaN
NaN	NaN	NaN	-1,8352	NaN	-1,83861	-1,86221	NaN	NaN	NaN
-1,67499	-2,93567	-1,92096	NaN	-0,513952	NaN	NaN	NaN	NaN	NaN
-0,0980282	0,6887	-0,386112	-0,153877	-0,507471	NaN	0,986349	-0,228487	-0,392241	-0,106175
-1,69913	-1,69943	-1,11969	-1,1764	-0,111904	-2,55339	-1,62702	NaN	NaN	NaN
-0,221411	-1,7382	-0,664179	1,5628	-1,29673	1,5944	-1,4553	0,166527	-0,365388	NaN
3,15969	1,23616	NaN	NaN	2,22318	NaN	-1,55499	1,22386	0,61958	1,32483
-2,37735	-1,56485	-2,54741	NaN	NaN	NaN	NaN	-1,80893	-1,42984	-1,97194
-1,0945	-2,66341	0,617456	-0,0150585	1,13414	NaN	0,693316	0,257971	0,526114	0,180177
-2,27799	-2,05463	-1,36573	0,342072	-0,948914	1,84555	-0,437452	NaN	NaN	NaN
NaN	NaN	-0,648411	-1,6816	NaN	-2,37459	-0,250652	NaN	NaN	NaN
-1,70429	NaN	2,27279	1,53592	-2,06006	-0,200077	2,54861	NaN	NaN	NaN
NaN	NaN	NaN	-0,890427	NaN	0,479115	-1,69775	NaN	NaN	NaN
-0,167322	NaN	-1,55082	-0,430244	-0,0856285	-2,53591	-0,335691	NaN	-1,24724	-1,93287
-2,58626	-1,92008	NaN	-1,7135	-2,08337	-2,38438	-1,96202	-2,14727	-0,53688	-1,56094
-1,48276	-0,967056	-1,01613	-1,38242	-1,30142	-1,02346	-0,646936	-1,51795	-0,985654	-1,54137
-2,31006	-1,60699	-2,35306	-0,4506	NaN	-0,666323	-0,873831	NaN	NaN	NaN
NaN	NaN	NaN	0,96176	0,146698	-1,46996	0,461231	NaN	-0,0932283	0,262105
NaN	NaN	NaN	-1,94827	-2,90331	-0,778584	NaN	NaN	NaN	NaN

Polito et al. Universal ready-to-use immunotherapeutic approach for the treatment of cancer: expanded and activated polyclonal γδ memory T cells.
Phosphorylation sites

NaN	NaN	NaN	-1,04706	NaN	-1,51853	-2,05442	NaN	NaN	NaN
-1,90775	NaN	-0,300608	-1,77345	NaN	-0,684927	-2,06542	-1,70509	-1,87914	-2,29815
NaN	NaN	NaN	1,88281	-0,715406	2,14939	-0,255873	-0,897221	NaN	-1,21211
-0,242592	NaN	NaN	1,16725	NaN	NaN	1,7587	-0,507631	NaN	0,433513
NaN	NaN	NaN	-0,663523	-2,8327	-1,10234	-1,1813	NaN	NaN	NaN
NaN	NaN	NaN	1,03311	NaN	-0,0189381	-1,99425	NaN	NaN	NaN
NaN	NaN	NaN	1,13673	-0,270189	1,62666	0,256342	NaN	NaN	NaN
-0,957077	NaN	NaN	1,43891	NaN	NaN	NaN	-1,24427	-0,900189	-1,55264
-1,75778	NaN	-2,02099	0,436197	-1,53671	0,00765038	1,74437	-1,84297	-2,02618	-1,5805
2,68479	0,290287	1,29049	3,09151	2,06841	3,46553	3,95701	1,73926	2,37051	1,0557
0,383537	-0,649656	0,238565	-0,56963	0,175131	NaN	-0,608709	0,103569	NaN	-0,203473
NaN	NaN	NaN	-1,99191	-2,32196	NaN	-1,35941	NaN	NaN	NaN
NaN	NaN	NaN	-0,931627	NaN	-2,07691	-1,58297	NaN	NaN	NaN
-2,85582	-1,40141	-2,06999	1,68492	-1,71433	2,05372	0,708029	NaN	NaN	NaN
NaN	NaN	NaN	0,87932	-2,95831	0,459726	-0,658918	NaN	NaN	NaN
-0,580072	NaN	1,78735	0,896307	0,747988	0,456411	0,922112	-0,168165	-0,466271	-0,527112
NaN	NaN	NaN	-0,919477	-4,44754	-0,975309	-2,50131	NaN	NaN	NaN
NaN	NaN	NaN	NaN	NaN	NaN	NaN	NaN	NaN	NaN
NaN	NaN	-0,677271	0,277491	NaN	0,628906	0,485664	-0,608562	NaN	NaN
NaN	-0,35367	-0,384645	-0,0451279	NaN	-1,99744	1,01859	0,330339	0,416247	0,0962782
NaN	NaN	-0,83814	-2,04691	-2,52463	-1,25522	-0,836321	NaN	NaN	NaN
NaN	NaN	0,156279	-0,0317612	-2,47096	-1,13808	0,8297	-0,329048	-0,742959	-0,613095
NaN	NaN	NaN	-4,14368	NaN	-1,23203	-3,64158	NaN	NaN	NaN
-1,03219	NaN	NaN	-0,618746	-0,344055	NaN	-0,397696	-1,3529	-1,22925	-0,958056
NaN	NaN	NaN	-0,0899124	-0,493753	-0,301403	0,388117	-2,17753	-2,33563	NaN
-3,25066	NaN	-0,271482	-0,886581	-3,62198	-1,9179	-0,808266	NaN	NaN	NaN
1,35275	1,39955	1,35116	2,23031	1,21622	2,26577	2,38202	1,19735	0,479556	0,597526
3,23833	2,49844	2,79717	3,9194	2,80293	1,24749	4,14407	2,59393	2,44614	2,24927
3,76035	1,7338	2,43229	1,43007	3,59927	0,642097	0,0385437	3,85399	3,7921	3,14731
NaN	NaN	NaN	-1,52655	NaN	-0,129032	-1,96962	NaN	NaN	NaN
0,834482	0,9951	4,21965	2,61675	1,22403	3,07356	3,08224	3,03116	2,9417	3,26313
-2,55877	-0,750174	-2,86923	-1,8894	-1,19	-2,21574	NaN	NaN	NaN	-1,34164
-2,01526	NaN	-1,25652	-1,25714	-1,79442	-0,105762	-0,116034	-0,889622	-1,40161	-0,786508
-1,35548	1,7977	-1,46985	-0,000972748	-0,265499	-1,49105	1,46434	-1,37997	0,0199804	-0,296998

-1,97856	-2,1178	-1,77099	-1,15047	-0,144815	NaN	-0,425909	NaN	NaN	NaN
NaN	NaN	NaN	-0,853321	-1,71816	-1,05521	-1,30704	NaN	NaN	NaN
NaN	NaN	NaN	0,34091	0,0103683	0,443604	-0,610687	-2,27677	-2,14433	-2,38899
NaN	NaN	NaN	-1,2091	NaN	-1,25908	-1,0008	NaN	NaN	NaN
NaN	NaN	NaN	NaN	NaN	-0,649927	NaN	-1,02925	-1,09689	-1,06438
-1,71876	-2,4879	NaN	-0,536564	-0,657436	NaN	0,149244	NaN	NaN	NaN
0,751135	2,03913	-0,920076	1,63714	1,60433	0,127018	2,68142	1,12452	0,959126	1,33342
3,12585	3,60621	1,1763	3,6049	2,41398	4,35948	3,33166	2,49192	1,56198	2,56783
-2,16507	-2,11085	NaN	NaN	NaN	-0,534864	-2,3644	NaN	NaN	NaN
-0,54048	-0,502016	-0,492907	0,068531	0,948038	-1,06352	1,06226	0,592054	0,150617	0,429122
-3,01135	NaN	-2,14123	-2,86215	NaN	NaN	-1,2653	-2,06795	-2,8779	NaN
NaN	NaN	NaN	NaN	NaN	NaN	NaN	NaN	NaN	NaN
-0,732124	1,36724	-0,150019	NaN	1,51731	NaN	-0,0822353	0,781603	-0,360562	0,907592
0,484686	0,529667	0,57597	1,01921	0,588964	0,700117	1,38673	1,01301	1,48846	0,0977373
4,20321	3,47367	3,55428	4,25355	3,70646	2,57289	3,76118	3,96227	3,77313	3,27736
NaN	NaN	NaN	NaN	NaN	NaN	-1,72601	NaN	NaN	NaN
NaN	NaN	NaN	NaN	NaN	NaN	NaN	-1,33021	-1,49079	-1,03633
-3,14469	-2,49263	-1,82892	NaN	-0,479055	NaN	-1,59879	-1,24734	-2,05019	NaN
NaN	NaN	NaN	-0,747568	-0,521116	0,286333	0,103905	-0,152527	-0,831609	-0,449662
NaN	NaN	NaN	NaN	NaN	NaN	NaN	NaN	NaN	NaN
NaN	NaN	NaN	NaN	NaN	NaN	NaN	NaN	NaN	NaN
1,49986	0,487511	1,00248	-0,148344	1,64317	-1,04012	0,193495	1,14553	0,73965	1,14992
NaN	NaN	NaN	1,12708	2,00961	2,98232	0,0768909	NaN	NaN	NaN
0,329018	-0,699785	1,35431	1,10135	1,93936	1,07108	1,20568	0,555422	-0,169068	1,02637
5,48762	5,68179	4,86023	7,3509	5,4162	8,16186	7,04792	5,34706	4,87541	4,97592
NaN	NaN	NaN	-1,22169	NaN	-1,25402	-1,19408	NaN	NaN	NaN
3,7472	3,04927	2,54521	3,88037	3,18714	3,81013	3,74176	2,84573	1,98702	2,96397
NaN	NaN	NaN	NaN	-1,97377	NaN	-1,93485	-1,15491	-1,51873	-1,54672
-1,53103	-1,4073	-2,06812	1,86157	-0,73562	2,4965	1,40963	-0,953491	-0,930749	-1,00938
0,821152	NaN	3,2438	1,53027	NaN	3,30578	2,10626	1,18632	1,26519	0,968671
-2,59716	-1,85309	NaN	-0,686859	-1,41495	-0,568163	-0,978264	NaN	-1,97449	-1,192
0,0908031	0,436853	0,062067	0,526638	0,81925	-0,968893	1,52935	1,00396	1,41137	1,58675
-0,767212	3,33688	2,73826	NaN	NaN	NaN	NaN	NaN	2,68387	3,15111
-1,86403	-2,34104	-1,57588	-1,34146	-1,91705	-0,553684	-1,76444	-1,33196	NaN	-1,13056

Polito et al. Universal ready-to-use immunotherapeutic approach for the treatment of cancer: expanded and activated polyclonal γδ memory T cells.
Phosphorylation sites

NaN	NaN	NaN	-2,16502	-2,02273	-2,12581	NaN	NaN	NaN	NaN
NaN	NaN	NaN	-1,55765	-1,48279	NaN	-2,52093	NaN	NaN	NaN
-0,225685	NaN	-1,58373	NaN	-1,34944	NaN	NaN	-0,253365	-0,291263	-0,640986
-1,0797	-0,455881	0,440493	0,407103	NaN	NaN	1,46443	-1,24273	0,276727	-0,997695
-0,92621	-1,03675	-0,472382	-2,68585	-0,0583725	-2,52152	-0,946003	NaN	NaN	NaN
NaN	-2,00839	-1,63107	-1,0803	-0,626688	-0,919958	-0,658283	NaN	NaN	NaN
1,06372	0,0187893	1,44881	3,83601	1,36966	4,66982	3,25931	1,25168	1,03825	2,16725
NaN	NaN	NaN	-0,73889	NaN	-0,66975	-0,757401	NaN	NaN	NaN
NaN	NaN	NaN	-2,31191	-3,82932	-2,66904	-2,14898	NaN	NaN	NaN
NaN	NaN	-1,97293	-1,7497	NaN	-2,07691	NaN	-1,74082	-2,36862	NaN
0,0435524	-0,310045	0,500126	-0,310835	1,2919	-0,376934	0,954531	0,347202	-0,194354	0,214423
-2,03772	-3,23709	-0,630941	-0,578497	-1,09808	0,834944	0,287527	-1,85438	-1,9757	-2,23802
-0,266933	0,320915	0,830423	0,759455	1,3564	0,281313	1,74136	1,10064	0,784869	1,32419
-2,20208	-0,285761	-1,28412	-1,31424	0,0774097	-2,27977	-0,324175	-0,790648	-0,997193	0,2179
NaN	NaN	NaN	NaN	NaN	NaN	NaN	-1,78114	-3,3892	-3,16767
NaN	NaN	NaN	-2,24598	-1,78685	NaN	-1,49563	NaN	NaN	NaN
NaN	NaN	NaN	-1,37583	-1,35256	-1,74703	-0,468563	NaN	NaN	NaN
4,53821	5,34593	4,91802	4,66541	5,27824	2,38077	5,03494	4,73207	4,27408	4,72685
1,03024	1,86387	0,620768	1,28523	0,713274	-0,183243	1,34925	0,769539	0,558225	0,521388
1,32121	1,38107	0,740164	1,77297	1,12073	-2,8663	2,22808	0,580742	1,31599	-0,0889387
0,210718	-0,119995	-0,854258	0,124254	0,0439625	-4,93447	1,06258	0,304296	0,278943	-0,587661
NaN	0,792089	0,563402	1,01494	-0,274172	1,71975	-0,850353	0,80904	0,446422	-1,33358
1,72112	-1,95535	1,82289	NaN	NaN	NaN	NaN	NaN	NaN	NaN
0,901493	1,1314	NaN	1,72923	1,32995	1,84445	1,23303	NaN	1,5141	1,74595
-0,414413	-1,17282	-0,243635	-0,649038	-0,548384	-1,93562	-0,320906	0,485918	0,642753	-0,0368052
0,793989	NaN	0,775141	1,21039	1,16375	1,71749	0,982283	1,8892	1,61758	-3,08784
-1,53542	-1,12746	-0,755196	-0,659946	0,23962	-1,44618	-0,212555	-1,37796	-1,34968	-0,360341
0,660984	-0,0235596	-0,762512	0,742416	0,250177	1,26405	-0,0449162	0,226786	-0,295611	-0,204452
-0,496267	0,993849	0,02952	NaN	-1,48964	-1,83484	1,31507	0,427658	0,542823	-0,826808
0,0774326	0,582014	0,59223	-0,517677	0,0393772	-1,64239	0,150734	0,206005	0,585681	-0,00610447
NaN	NaN	-0,627924	-1,65536	NaN	NaN	-1,04577	-1,05966	-1,41785	NaN
1,58036	1,56916	NaN	0,918943	2,088	NaN	1,45891	1,58386	1,34308	1,35205
-1,1284	-1,33647	-0,1208	0,471228	0,879667	0,783867	0,826302	0,417574	0,210599	0,0282145
-0,367989	-1,53692	NaN	-2,51316	-0,826885	NaN	NaN	-0,52314	-1,17175	-0,632245

Polito et al. Universal ready-to-use immunotherapeutic approach for the treatment of cancer: expanded and activated polyclonal γδ memory T cells.
Phosphorylation sites

NaN	NaN	NaN	NaN	NaN	NaN	NaN	-2,42641	-1,77405	-2,45705
0,676151	1,98155	1,57362	2,09142	NaN	1,43135	2,93119	1,57718	1,92912	2,70985
1,38713	2,33676	0,314503	3,42011	2,00423	3,69991	3,40261	2,38314	1,96192	2,17169
1,66852	1,68323	0,537817	2,33508	1,89563	2,78876	2,83406	1,81536	1,56677	1,31674
-0,738924	0,979147	-0,100773	-1,14883	1,64816	-1,86077	0,822769	-1,12518	-0,62279	1,76646
2,12221	2,95386	1,47092	1,82053	2,20387	1,84895	NaN	2,51684	2,93688	2,90679
NaN	NaN	-2,21331	-2,82	-3,08571	-1,68144	-1,94566	NaN	NaN	NaN
3,32512	2,58251	2,94943	4,0695	3,25848	3,16685	3,29789	-0,390268	-0,634046	-0,243964
0,177612	NaN	NaN	1,38369	NaN	1,05658	1,51566	0,39459	0,250831	NaN
-0,701601	-1,12906	-0,704714	NaN	-0,146923	-2,51103	-1,48127	-0,325169	-1,07451	0,189141
-0,885603	-1,12906	-1,47226	NaN	-0,222204	-2,51103	-1,48127	-0,325169	-1,07451	0,189141
NaN	NaN	NaN	-0,380198	NaN	-0,0509815	-0,00519371	NaN	NaN	NaN
NaN	NaN	NaN	0,383764	NaN	0,713991	-0,521729	NaN	NaN	NaN
NaN	NaN	NaN	NaN	NaN	NaN	2,67117	NaN	NaN	NaN
-1,0674	-0,820513	-0,463703	-0,989759	-0,0509605	-1,71449	-0,0497894	-0,57946	-0,40685	-0,712214
NaN	NaN	NaN	NaN	NaN	NaN	NaN	NaN	NaN	NaN
0,932346	0,587521	1,21187	0,900843	1,53135	0,437689	1,00183	0,467348	1,53916	1,54991
NaN	-1,86434	-0,857136	-2,70754	-1,92502	NaN	-0,115911	-1,63145	-0,879291	-1,24911
NaN	1,6564	0,95789	NaN	0,612234	NaN	0,679569	0,130432	-0,0294619	0,0492811
4,61436	5,15927	5,19348	4,49702	5,45678	NaN	4,8266	5,03183	5,20831	4,7633
-1,61141	-1,0644	-0,985216	-1,16486	-0,563347	-2,64959	-0,898907	-1,44701	-1,42197	-2,39562
-0,25815	-0,310411	0,078701	-3,68122	0,385355	-2,25313	NaN	NaN	NaN	NaN
-1,57033	-0,456415	-0,167028	NaN	-0,0619888	-0,416643	NaN	-0,196209	-0,424497	0,248801
NaN	NaN	NaN	NaN	1,54807	0,0769997	0,0269222	NaN	NaN	NaN
NaN	NaN	NaN	NaN	NaN	NaN	NaN	NaN	NaN	NaN
3,09444	4,19875	2,96494	2,95836	NaN	NaN	2,86801	3,19594	3,08905	2,65749
NaN	NaN	NaN	NaN	NaN	NaN	NaN	-1,63578	NaN	-1,89747
NaN	NaN	NaN	-1,87632	-0,62937	-1,31228	-1,47884	NaN	NaN	NaN
NaN	NaN	NaN	-1,70353	NaN	-1,23344	-1,25518	NaN	NaN	NaN
NaN	NaN	NaN	-0,161402	-0,45438	-0,0334587	NaN	0,242325	-0,475339	-1,70419
NaN	NaN	-1,92659	-1,47919	-2,58505	-0,473877	-2,10246	-2,18352	-2,57505	-1,75728
-1,6909	-0,560181	-1,95597	-0,462355	-0,507431	-0,130606	-0,0346947	-0,815891	-0,937804	-0,809009
NaN	NaN	NaN	NaN	NaN	NaN	NaN	NaN	NaN	NaN
-2,17895	-0,724232	NaN	-1,75298	0,512718	-0,355602	-1,80143	NaN	NaN	NaN

Polito et al. Universal ready-to-use immunotherapeutic approach for the treatment of cancer: expanded and activated polyclonal γδ memory T cells.
Phosphorylation sites

0,745556	2,48389	-0,272276	0,854525	3,16016	2,10451	0,776815	0,406324	-0,340713	1,0377
NaN	NaN	NaN	-0,473516	NaN	-0,208498	-1,74986	NaN	NaN	NaN
NaN	1,22508	-0,62129	0,813589	0,422489	1,97659	1,40895	-0,562565	0,491815	0,66995
-0,955042	-0,449059	-0,655905	NaN	-1,05291	-2,9584	-1,20398	-1,12746	-0,934272	-1,18155
-1,77747	-0,773046	-0,462242	-0,864256	-0,89679	-0,326233	-0,110281	-0,810896	-1,04071	-0,922215
NaN	NaN	NaN	-0,845406	-1,41192	NaN	-0,792658	-0,309536	-0,351428	-1,05562
2,88207	0,807215	3,5297	2,57167	3,45697	2,65292	2,86798	2,78181	2,63158	2,59075
3,33539	3,49883	3,02098	4,02197	4,19053	3,97313	2,88451	3,9381	3,54379	3,54786
0,840227	-1,45806	-3,61618	-0,585733	0,188517	0,0502834	NaN	NaN	NaN	NaN
-1,908	-3,26526	NaN	-2,33324	-0,489157	-0,411932	-1,46952	-1,02473	-1,54165	-1,07497
2,12221	2,05417	1,07691	2,32117	1,83109	2,72614	1,74116	2,75072	2,22592	2,74225
3,33572	3,81973	1,79751	3,3047	3,20155	4,55027	3,41431	1,87397	2,24309	2,21785
-0,101257	-0,422384	-1,55891	0,29604	0,47027	-2,2537	0,257763	1,08767	1,11307	2,14085
NaN	-0,876842	NaN	NaN	-0,75248	NaN	-1,72549	NaN	NaN	NaN
3,22521	3,13557	2,79458	3,87799	3,28057	4,09124	3,96247	3,69826	3,54187	3,46586
2,1218	NaN	2,15723	NaN	3,00625	-1,53827	NaN	2,4354	1,98033	2,34163
-2,56273	-2,40867	-1,80243	-1,91753	-1,43465	-1,48504	-1,03731	-1,30879	-1,25461	-1,65306
NaN	NaN	NaN	NaN	NaN	NaN	NaN	NaN	NaN	NaN
-0,915476	-1,92705	-0,096529	NaN	0,156782	-1,42566	NaN	-0,702364	0,024806	-0,307837
1,28531	1,36313	1,30197	1,55619	1,7404	1,90564	1,93847	2,03606	1,65002	1,40916
-0,805477	-1,99312	0,00214005	-4,21273	-0,88393	NaN	-1,42055	0,404009	NaN	-1,3363
0,0667744	1,78519	0,827917	0,0586281	2,61829	-0,99861	0,879261	0,0378113	-0,896634	1,99896
0,456921	0,208385	0,879522	-0,290794	0,806416	0,670683	1,06994	0,318167	0,635245	0,240279
NaN	NaN	NaN	NaN	NaN	NaN	NaN	NaN	NaN	NaN
-1,9268	NaN	NaN	-1,70239	-0,742359	NaN	-1,97514	NaN	NaN	NaN
NaN	NaN	NaN	NaN	NaN	NaN	NaN	NaN	NaN	NaN
NaN	NaN	NaN	-0,37948	-1,05603	-2,12869	NaN	NaN	NaN	NaN
-1,25101	0,114378	NaN	-1,25258	-0,942232	-1,12226	NaN	NaN	NaN	NaN
NaN	NaN	NaN	NaN	NaN	NaN	-0,683149	NaN	NaN	NaN
NaN	NaN	NaN	NaN	NaN	NaN	NaN	-1,79987	-0,619605	-1,11279
-1,83627	NaN	-1,56204	0,296999	-1,72623	0,960888	0,584799	NaN	-0,882802	-0,673121
-0,810009	-0,759243	-0,873663	-0,699461	0,157574	NaN	-1,31208	0,289312	-0,645839	0,113097
NaN	1,43955	1,26752	NaN	2,12406	2,09896	NaN	NaN	-3,10153	0,784959
NaN	NaN	NaN	0,092659	NaN	-0,111448	-0,948977	NaN	NaN	NaN

Polito et al. Universal ready-to-use immunotherapeutic approach for the treatment of cancer: expanded and activated polyclonal γδ memory T cells.
Phosphorylation sites

NaN	NaN	NaN	NaN	NaN	NaN	NaN	NaN	NaN	NaN
-0,510569	-0,528332	0,324373	-0,0613594	1,23071	0,472393	1,11645	0,976717	0,34681	0,561126
0,195206	NaN	-0,74358	NaN	NaN	NaN	NaN	-0,341951	-0,330207	-0,824977
NaN	NaN	NaN	-1,81592	NaN	-1,44994	-0,859447	-0,92802	-1,02743	-0,683993
-1,23706	-0,0777206	-0,829941	-1,45995	0,434803	NaN	-0,262882	-0,586672	-0,501433	-0,169265
0,820267	0,193069	1,35977	NaN	1,49711	NaN	NaN	0,741871	0,697021	0,155169
NaN	NaN	NaN	-2,95503	-4,18917	-1,75639	-2,55231	NaN	NaN	NaN
NaN	NaN	NaN	NaN	NaN	NaN	NaN	0,725069	0,402427	1,12562
-1,55523	-1,10229	-0,655605	-0,256266	-0,303102	-0,270458	0,275412	-1,12921	-1,0884	-0,713267
NaN	NaN	NaN	0,75565	NaN	-0,810509	0,669489	-0,448845	-0,85542	0,235219
0,44813	0,321413	0,258743	NaN	0,76425	NaN	NaN	0,159956	-0,336745	-0,29404
2,48654	2,83197	2,04162	2,78195	2,66274	1,78758	2,82567	3,28353	2,94553	3,47726
0,955437	1,11582	-0,0495071	1,31509	1,03758	1,54495	1,34309	0,423536	0,502625	0,397868
-3,16685	-0,9659	NaN	-2,27318	NaN	-2,06628	-1,94102	NaN	NaN	NaN
-1,18894	-1,34904	-1,88239	-0,610582	-0,35849	-3,32745	0,214602	-1,03763	-1,23875	NaN
0,737411	-0,512579	1,12465	1,77485	-0,988644	NaN	NaN	-0,277529	0,100652	-0,34653
1,18844	1,35949	0,484106	1,47262	0,466961	1,11689	2,25464	1,43055	1,93985	0,791471
-0,394003	NaN	0,692562	0,0606728	NaN	NaN	0,397512	1,35358	1,12609	0,951447
1,27686	0,0483456	2,27704	2,06869	0,848774	NaN	2,24383	-0,169146	-0,215726	0,00308323
-1,1538	-2,2657	2,06232	1,61924	-0,767839	2,75797	1,24788	0,933359	0,891198	0,35955
-1,79409	0,398838	1,50657	1,22391	NaN	1,65338	1,50373	-1,98672	0,195275	-0,880425
NaN	NaN	0,213209	-1,30272	NaN	-1,19066	-0,674099	NaN	NaN	NaN
NaN	NaN	NaN	0,373301	NaN	0,949701	1,19917	NaN	NaN	NaN
-1,99181	-0,42374	0,525368	-3,48043	0,659027	-2,81059	-1,27208	0,399855	-0,0735636	NaN
1,323	2,31037	2,10817	2,60487	1,87563	4,90845	NaN	NaN	NaN	1,98093
-0,137897	2,15412	3,02957	1,17044	NaN	-0,265697	3,56521	2,80939	2,89831	2,88385
NaN	NaN	NaN	NaN	NaN	NaN	NaN	NaN	NaN	NaN
-0,705891	-2,73089	-0,918272	-2,48602	NaN	NaN	-0,894371	-2,42896	-1,30283	-1,75093
1,15546	0,0694618	0,733805	-0,337345	1,23192	-1,62093	0,207922	0,364645	0,256354	0,602311
-1,81868	-2,7513	-1,6938	-2,59598	-2,14768	-2,73445	-2,26578	-0,963476	-1,81099	-1,04881
-4,55242	NaN	3,40053	1,6526	3,41254	2,17648	2,927	2,72616	3,10005	2,63469
-0,59746	-0,141176	2,90762	1,45295	1,52207	0,987967	NaN	-1,45786	2,63627	1,77875
-2,65784	3,07277	-2,91324	2,3485	NaN	NaN	NaN	NaN	NaN	NaN
1,614	1,95548	2,0317	0,0930729	1,84232	-1,32094	0,892443	1,53081	1,25766	1,29222

Polito et al. Universal ready-to-use immunotherapeutic approach for the treatment of cancer: expanded and activated polyclonal γδ memory T cells.
Phosphorylation sites

-1,51582	-1,39279	NaN	-1,73788	NaN	NaN	-1,29164	-1,36641	-1,29818	-1,22874
5,34669	5,05282	4,6066	1,48679	1,17614	4,6062	4,94581	5,29566	4,83667	1,27278
0,651922	-1,33651	-1,02979	-0,0245075	0,0758476	0,359051	0,888102	0,081604	0,365731	-0,302251
-0,314882	NaN	NaN	-1,41186	-2,15805	-1,29757	-1,1178	-0,4137	-0,305665	-0,544513
NaN	NaN	NaN	-4,53801	-3,30688	-1,67906	-4,1813	NaN	NaN	NaN
NaN	NaN	NaN	-1,17321	-3,77588	NaN	0,0946617	NaN	NaN	NaN
NaN	NaN	NaN	NaN	-3,15409	-0,777534	-1,39669	NaN	NaN	NaN
NaN	NaN	NaN	NaN	-0,545227	-2,4593	NaN	NaN	NaN	NaN
NaN	NaN	NaN	-2,24511	-2,804	-1,43312	-2,03818	NaN	NaN	-2,87667
-1,24937	-2,27909	-1,35306	-1,51117	NaN	NaN	-1,01908	-0,542336	-0,659574	0,00367832
-1,20013	-2,72336	-1,42142	-2,46689	-1,31593	-1,82678	-1,38714	-1,41853	-1,63093	-1,37363
-2,14726	-1,48114	-2,594	-2,82586	-1,43973	NaN	-1,36924	-1,20947	-0,966531	-1,13087
0,288673	0,942572	1,06845	0,452005	0,286623	0,76149	0,445366	1,50476	2,00062	0,976739
-1,45484	-3,14173	NaN	0,668589	-1,36569	0,831512	-1,40496	NaN	NaN	NaN
NaN	NaN	NaN	-2,23236	NaN	NaN	-1,61534	NaN	NaN	NaN
NaN	NaN	NaN	1,3924	NaN	1,7402	0,576925	NaN	NaN	NaN
NaN	NaN	NaN	0,501797	NaN	0,428547	NaN	NaN	NaN	NaN
NaN	NaN	0,968218	1,47282	0,288208	NaN	1,76358	NaN	NaN	NaN
-0,37298	-1,25722	-1,9038	NaN	0,0955391	NaN	NaN	-0,940557	-0,604341	-0,456857
NaN	NaN	NaN	0,109489	-0,898911	2,04338	0,547243	NaN	NaN	NaN
NaN	NaN	NaN	NaN	-0,532257	NaN	-0,272352	NaN	NaN	-1,99435
NaN	NaN	NaN	NaN	NaN	NaN	NaN	NaN	NaN	NaN
NaN	NaN	NaN	NaN	NaN	NaN	NaN	NaN	NaN	NaN
NaN	NaN	NaN	-0,586727	NaN	NaN	-0,505426	NaN	NaN	NaN
NaN	NaN	NaN	NaN	-2,12679	NaN	NaN	-1,83334	-2,1964	-0,871327
0,461693	0,711044	0,872826	2,03697	1,1189	3,63841	1,88429	0,275921	0,303863	0,417296
NaN	NaN	NaN	NaN	NaN	NaN	NaN	NaN	NaN	NaN
-0,472567	-1,02913	-0,382101	-0,393513	-0,0514011	-0,677242	-0,129587	-0,34132	-0,803166	-0,801629
-2,14558	-0,365602	-2,38415	NaN	NaN	NaN	NaN	2,2371	1,54886	1,44307
-0,734758	NaN	-0,71442	0,342144	0,196974	-0,286417	0,556101	-0,0803146	0,135777	-0,512765
1,56188	1,71826	2,57831	1,38003	2,38591	1,2571	0,889069	1,23142	1,42136	1,6798
NaN	NaN	NaN	NaN	NaN	NaN	NaN	NaN	NaN	NaN
NaN	NaN	NaN	-1,92631	NaN	-1,73401	-2,59744	NaN	NaN	NaN
NaN	NaN	NaN	NaN	NaN	NaN	NaN	-1,48595	-0,420619	0,15729

Polito et al. Universal ready-to-use immunotherapeutic approach for the treatment of cancer: expanded and activated polyclonal γδ memory T cells.
Phosphorylation sites

7,56422	7,76856	7,66901	7,86114	6,10826	6,51574	7,92144	7,61193	8,6846	7,54372
0,155659	-1,11952	2,33381	3,40792	1,55544	3,67461	3,77786	0,999725	0,937444	0,670381
NaN	NaN	NaN	NaN	NaN	NaN	NaN	-3,47904	-3,13306	-2,57229
NaN	0,0746346	NaN	NaN	-1,33187	-1,86718	NaN	NaN	NaN	NaN
-1,94122	-1,53945	NaN	NaN	-1,5468	NaN	-0,56736	NaN	NaN	NaN
NaN	NaN	NaN	-1,71924	NaN	-0,987423	-1,84782	NaN	NaN	NaN
NaN	NaN	NaN	NaN	NaN	NaN	NaN	NaN	NaN	NaN
4,11076	4,3714	3,81829	4,47586	4,99179	4,4632	4,75715	4,04526	3,95427	4,10195
1,67734	2,57194	1,74175	1,95234	2,49333	1,4672	1,41248	2,11913	1,85905	2,16735
NaN	NaN	NaN	-1,73176	NaN	NaN	-3,35866	NaN	NaN	NaN
NaN	NaN	NaN	2,01608	NaN	1,53179	-0,0504837	NaN	NaN	NaN
NaN	NaN	NaN	2,12658	NaN	1,79251	0,183237	NaN	NaN	NaN
NaN	NaN	NaN	NaN	NaN	NaN	NaN	NaN	NaN	NaN
NaN	NaN	NaN	1,59316	NaN	1,86318	-0,592815	-2,4543	-2,90859	NaN
NaN	NaN	NaN	-0,685911	NaN	-0,697718	NaN	NaN	NaN	NaN
NaN	NaN	NaN	NaN	NaN	NaN	NaN	NaN	NaN	NaN
-1,29722	NaN	-1,37077	0,701403	-1,99583	-0,137575	-0,225065	NaN	NaN	NaN
-1,86631	-1,7955	-1,0949	-2,31437	-1,63254	NaN	-1,89002	-0,0367813	-0,740216	-2,39852
NaN	NaN	NaN	-0,92963	-1,61049	-1,21269	-1,39581	NaN	NaN	NaN
-0,807976	-1,21882	-1,81141	NaN	-0,0331039	NaN	-1,80471	NaN	NaN	-1,60871
-1,30591	-1,44485	-1,26306	-1,1927	-1,26577	-1,05927	-1,12714	-0,847004	-1,51474	-1,8082
0,494938	-0,486546	1,40169	0,0501842	1,09074	0,160643	0,0110645	0,850159	0,550265	0,696673
NaN	NaN	NaN	NaN	NaN	NaN	NaN	-2,92198	-3,29742	-3,24186
1,34855	2,055	2,34754	1,35027	3,40804	0,866112	2,54479	1,43996	1,35924	2,34877
-1,61653	-1,97231	-2,04274	-3,17288	-0,043293	NaN	-2,57263	NaN	NaN	NaN
NaN	-1,30707	NaN	NaN	0,10844	0,401403	NaN	-0,448811	-0,802701	-0,389594
-0,512922	-0,475544	0,648212	0,387001	0,727924	0,183615	1,44609	NaN	NaN	NaN
0,0955334	-0,108797	0,164169	-1,45934	1,62448	NaN	-0,503113	0,0449848	-0,407149	0,542039
NaN	NaN	NaN	-0,347506	-2,17824	-0,193758	NaN	NaN	NaN	NaN
NaN	NaN	NaN	NaN	-3,60933	-1,76451	-2,12588	NaN	NaN	NaN
0,599634	0,318918	0,301302	0,14332	0,268515	-1,64314	0,0710087	0,756325	0,961341	-0,0120173
-0,156237	0,983801	0,547972	0,923893	1,7001	2,18956	1,34318	1,31239	1,67169	1,34168
NaN	NaN	NaN	NaN	NaN	1,61653	1,12313	1,20538	1,45996	1,23165
NaN	NaN	NaN	-1,34123	-3,19639	-0,602177	-3,40677	NaN	NaN	NaN

Polito et al. Universal ready-to-use immunotherapeutic approach for the treatment of cancer: expanded and activated polyclonal γδ memory T cells.
Phosphorylation sites

2,40325	1,56784	1,48144	2,04256	3,03011	-0,00191879	2,85806	1,89893	1,81968	1,52198
1,35825	0,656052	1,34541	1,26572	1,52623	1,37965	0,626116	0,701826	0,426049	0,644263
-1,48976	-2,19573	-0,299278	-0,0179596	-1,7958	NaN	0,250568	-0,866791	-1,04891	-1,07744
NaN	NaN	-0,320271	-1,83154	-0,504242	NaN	-1,15539	NaN	-0,148463	-1,25567
NaN	-1,21871	-0,280979	-0,344398	NaN	-0,43404	-0,594807	NaN	NaN	NaN
0,991806	2,54891	1,11616	1,31981	1,52089	1,77426	2,42727	NaN	NaN	NaN
-0,631634	0,306124	NaN	0,0678291	NaN	0,252028	0,0541248	NaN	NaN	NaN
NaN	NaN	0,147217	0,742086	NaN	3,00474	0,106211	NaN	NaN	NaN
3,68934	2,92619	2,98121	2,46887	3,39045	1,57788	2,60406	2,80573	2,47708	2,40998
-0,613285	-0,569006	NaN	-0,752266	0,461872	NaN	0,208328	0,429752	0,18802	0,0164862
-2,77048	-1,60855	-2,09567	NaN	NaN	NaN	NaN	NaN	NaN	NaN
NaN	NaN	NaN	NaN	NaN	NaN	NaN	NaN	NaN	NaN
NaN	NaN	NaN	NaN	NaN	NaN	NaN	NaN	NaN	NaN
NaN	NaN	NaN	NaN	NaN	NaN	NaN	NaN	NaN	NaN
NaN	-0,791021	0,773405	-0,264656	NaN	0,290358	-0,476128	NaN	-2,29813	-0,651872
NaN	NaN	NaN	-3,02559	NaN	-3,23722	NaN	NaN	NaN	NaN
NaN	NaN	NaN	-0,0835876	-1,71415	0,374386	-0,925655	NaN	NaN	NaN
NaN	NaN	NaN	-1,58916	-3,64246	NaN	-2,64599	NaN	NaN	NaN
-0,941647	NaN	-1,09444	0,164976	NaN	1,14377	-0,948719	-0,909584	-0,908711	-2,16447
NaN	NaN	NaN	-0,400873	NaN	NaN	-0,0339165	NaN	NaN	NaN
1,09989	-0,83898	0,811224	-0,0593071	1,38553	-2,0257	0,855249	1,15777	1,9391	1,37409
NaN	NaN	NaN	-0,670441	NaN	0,306532	-0,38813	NaN	NaN	NaN
NaN	NaN	NaN	NaN	NaN	NaN	NaN	NaN	NaN	NaN
NaN	NaN	NaN	NaN	NaN	NaN	NaN	NaN	NaN	NaN
-1,33653	NaN	NaN	-0,658583	NaN	0,106907	NaN	NaN	NaN	NaN
NaN	-0,795881	NaN	1,37217	0,597071	1,78146	-0,636789	NaN	NaN	NaN
-1,39867	-1,35214	-1,19364	-0,100792	-0,870924	NaN	-0,218706	-1,25935	NaN	-1,60787
NaN	NaN	NaN	NaN	NaN	NaN	NaN	NaN	NaN	NaN
NaN	NaN	-0,270357	0,73625	NaN	-0,609489	0,862028	NaN	NaN	NaN
NaN	NaN	NaN	NaN	NaN	NaN	NaN	-0,582596	0,0957098	1,00937
-1,15733	NaN	NaN	-0,362719	NaN	-0,6488	0,350788	NaN	NaN	NaN
1,52489	1,82187	4,10793	4,69047	1,59259	5,34841	4,19095	2,10413	2,83056	2,89349
NaN	NaN	-0,119902	-1,84812	NaN	NaN	NaN	1,93773	1,76359	2,487
0,438181	0,276848	1,07762	0,799295	1,64952	-0,0202732	0,664139	2,00328	1,37269	1,33478

Polito et al. Universal ready-to-use immunotherapeutic approach for the treatment of cancer: expanded and activated polyclonal γδ memory T cells.
Phosphorylation sites

3,88797	2,55865	4,45346	4,26841	4,19615	4,05306	4,91666	5,12354	4,9675	4,36276
NaN	NaN	NaN	NaN	NaN	NaN	NaN	NaN	1,86987	1,68144
0,75034	0,37837	1,38019	1,46452	1,27943	-1,13231	0,360115	1,09598	1,49872	0,810569
NaN	NaN	NaN	-0,019413	NaN	0,972843	0,292828	NaN	NaN	NaN
NaN	-0,960342	-0,358267	0,904579	NaN	1,23523	0,583248	NaN	NaN	NaN
-0,708004	-2,7007	-1,32289	0,577784	NaN	0,463207	-1,16473	-0,0576038	-0,858317	-0,828519
2,82727	2,24121	2,6903	NaN	3,87396	NaN	NaN	3,28689	2,23283	2,54803
NaN	NaN	NaN	2,41136	0,378942	2,47351	NaN	-0,499004	NaN	NaN
NaN	NaN	NaN	0,879419	NaN	0,319939	-0,522179	NaN	NaN	NaN
-0,822704	0,011713	-0,519581	-0,589518	NaN	NaN	NaN	-1,05141	-1,09224	0,424958
NaN	NaN	NaN	-1,06795	NaN	-1,18118	-1,38544	NaN	NaN	NaN
-0,681416	-0,705948	-2,15353	-1,65618	-1,14793	-1,06017	-0,836161	-0,203083	-0,0349989	-0,56243
NaN	NaN	NaN	-1,03967	-0,767986	-0,439442	-0,0183277	0,0663433	-0,0716219	-0,115867
1,03067	0,392206	NaN	NaN	NaN	NaN	NaN	NaN	NaN	NaN
2,39839	2,25508	2,70844	2,03947	2,54934	1,67478	2,06797	3,49568	2,98793	3,68073
-3,17752	NaN	-1,05282	-0,269146	NaN	-1,67068	-0,357086	NaN	-2,85992	-2,67208
NaN	NaN	NaN	-1,142	-2,74323	NaN	-1,67189	NaN	NaN	NaN
NaN	NaN	NaN	NaN	-0,36503	NaN	NaN	NaN	NaN	NaN
NaN	NaN	NaN	-0,501713	0,565392	-0,155081	-0,939604	NaN	NaN	NaN
-1,89541	1,59311	NaN	-0,342976	-1,81939	0,303816	0,433451	-0,733835	-0,885907	-1,5373
NaN	0,593418	NaN	NaN	-1,23593	NaN	NaN	NaN	NaN	NaN
NaN	NaN	NaN	-1,05049	-0,739128	-3,46254	-0,412106	NaN	NaN	NaN
0,504929	3,18865	NaN	2,05968	2,07389	4,2343	2,80641	1,74916	2,21681	2,56492
NaN	NaN	NaN	-2,90957	-0,176565	NaN	-2,1466	-1,08361	-1,50743	-1,36888
NaN	2,57002	NaN	1,47268	NaN	NaN	1,83171	NaN	NaN	NaN
-0,22822	-0,214783	0,0262566	0,50724	-0,452473	1,00168	1,03874	-0,882753	-1,08679	-1,02261
1,97072	1,3427	0,78686	-1,717	1,60217	-0,902828	-0,204988	0,780123	0,773396	1,03008
0,597187	-3,37973	-0,29207	0,749868	1,6418	0,701591	0,058403	0,250463	NaN	-0,441848
-1,50419	-3,84492	1,2105	-0,329954	-1,63245	1,59785	-0,0771351	-0,594334	0,245946	-0,867011
2,228	1,20677	2,18565	0,71143	3,40085	NaN	0,851044	3,7912	4,72254	4,33061
3,0529	1,92709	0,968544	2,96795	2,58489	-0,757988	0,768181	1,64519	1,34768	1,65996
0,698605	0,965967	-0,489986	NaN	-0,522423	NaN	-0,79735	NaN	-0,447162	NaN
1,62663	0,162218	1,67342	NaN	1,72569	NaN	-1,69725	2,63362	1,84239	NaN
NaN	NaN	NaN	NaN	NaN	NaN	NaN	-1,5352	-1,33496	-5,25173

Polito et al. Universal ready-to-use immunotherapeutic approach for the treatment of cancer: expanded and activated polyclonal γδ memory T cells.
Phosphorylation sites

-1,338	-1,44885	NaN	-1,21865	-0,342142	-1,23801	-2,00973	NaN	NaN	NaN
NaN	NaN	-2,29646	-2,59698	-2,8275	-2,38738	-1,85985	NaN	NaN	NaN
NaN	NaN	NaN	-0,479706	0,221054	NaN	-0,49317	0,28133	NaN	-0,954627
4,52091	4,58813	5,15705	4,9174	5,5665	4,31424	4,70481	4,50705	4,27344	4,44271
-1,86306	NaN	-3,11723	-1,97751	NaN	NaN	-2,91587	-2,75676	-2,99547	-2,69636
NaN	NaN	NaN	NaN	NaN	NaN	NaN	NaN	NaN	NaN
NaN	-1,65362	NaN	-1,25644	-1,25081	-1,05461	-1,04084	NaN	NaN	NaN
NaN	NaN	NaN	-1,216	NaN	-2,03638	-0,252941	NaN	NaN	NaN
1,21974	-0,037674	2,28054	3,14484	1,12725	3,1735	2,53244	0,337896	0,36398	0,0741491
-1,40339	NaN	NaN	-0,878786	-2,68546	NaN	0,244865	NaN	NaN	NaN
NaN	NaN	NaN	0,197594	-3,6086	0,528633	-0,621923	NaN	NaN	NaN
NaN	NaN	NaN	3,69948	2,52771	4,05587	3,29832	NaN	NaN	NaN
NaN	NaN	NaN	NaN	NaN	NaN	NaN	NaN	NaN	NaN
1,79476	1,94476	1,90459	2,24508	2,18985	1,10027	2,03422	2,0677	1,85747	1,33278
0,101828	-1,17265	-0,592016	-0,538244	-0,888451	-2,06032	-0,693882	0,339573	0,106399	0,191314
NaN	NaN	NaN	NaN	NaN	NaN	NaN	NaN	NaN	NaN
0,236145	1,06162	-0,103617	NaN	0,37763	NaN	NaN	-0,0494499	-0,668315	0,0496359
NaN	NaN	NaN	-0,957382	-2,48814	-1,88592	-0,814934	NaN	NaN	NaN
-2,15529	-4,01052	0,190283	1,03392	-2,35521	2,0583	0,639246	NaN	NaN	NaN
-0,275661	-0,42255	-1,07578	2,35567	0,485113	NaN	0,339458	-1,66088	-1,40949	-1,70352
NaN	NaN	NaN	-0,175968	NaN	NaN	-1,04522	-1,15458	NaN	-1,5062
-0,79595	-0,238405	-1,20743	-0,242044	-0,44417	-1,01624	-1,22092	-1,63408	-1,39934	-1,8385
-1,79162	-2,44214	NaN	-2,74205	-1,12872	NaN	NaN	-1,7814	-1,8097	-0,916097
0,947588	0,437658	1,42343	0,750851	1,01144	0,923847	1,17473	-1,12305	0,0506983	0,11159
3,86202	4,0929	4,42552	4,38147	4,73174	5,05411	4,13009	4,28591	3,64318	3,56593
NaN	NaN	-1,07551	-1,44326	NaN	-0,481821	-1,27993	NaN	NaN	NaN
1,74981	0,745462	2,90734	1,68497	2,99819	1,55799	2,52838	1,72808	1,90183	1,97913
1,68194	2,97519	2,59648	2,37323	3,75663	1,27345	3,15948	2,61126	2,59433	3,51022
NaN	NaN	NaN	-0,734785	NaN	-0,342094	-2,80876	NaN	NaN	NaN
NaN	-1,02452	-1,11914	0,574453	-0,829206	2,18788	-0,314749	-2,41639	NaN	-1,79079
NaN	NaN	NaN	-2,55878	NaN	-1,58151	-2,61089	NaN	NaN	NaN
NaN	NaN	NaN	-0,191523	NaN	0,926813	-0,238737	NaN	NaN	NaN
-3,88941	NaN	-0,403139	1,71893	-2,43434	1,74235	1,74663	NaN	NaN	NaN
NaN	NaN	NaN	-0,977257	-2,86002	-0,115208	-0,519836	NaN	NaN	NaN

Polito et al. Universal ready-to-use immunotherapeutic approach for the treatment of cancer: expanded and activated polyclonal γδ memory T cells. Phosphorylation sites

1,24745	2,15083	2,23889	1,76303	3,23304	NaN	2,29021	2,5962	1,9096	3,0539
2,21657	1,67691	1,6978	1,37967	2,53297	NaN	2,20053	1,96705	1,70767	2,25952
-1,92808	-2,52463	-2,52992	NaN	-2,66142	NaN	NaN	-2,69223	-2,98194	-3,24817
3,80644	NaN	4,16493	5,10235	5,31962	4,08179	4,99842	4,93135	4,83646	4,80733
-0,759029	-2,42071	-0,839581	NaN	-0,805758	NaN	-2,72792	NaN	NaN	NaN
1,96129	1,5261	4,47726	3,77353	1,68241	1,84382	3,29388	1,88927	1,72688	1,5784
1,01043	-0,115988	4,50562	NaN	NaN	NaN	NaN	3,72541	4,38463	3,41926
1,75683	-2,81182	1,56053	1,20091	-2,19103	1,52872	-1,17645	1,22855	1,53986	-2,58946
NaN	NaN	NaN	-1,89822	NaN	NaN	-3,14651	NaN	NaN	NaN
NaN	NaN	NaN	-2,59776	-0,214331	-2,24902	-1,86554	-1,78819	-2,22047	-2,15596
1,9286	1,24144	NaN	0,178425	2,31751	0,367901	NaN	NaN	0,887212	0,916129
NaN	NaN	NaN	0,328375	NaN	0,803705	0,397665	NaN	NaN	NaN
0,323891	0,690632	1,68592	NaN	1,5431	0,626114	NaN	0,413177	0,18784	0,502948
4,37708	4,94609	4,66968	2,11894	5,47223	2,67954	3,62598	3,84986	3,53224	3,60233
NaN	NaN	NaN	NaN	-1,75167	-1,27052	-1,71159	-1,94281	NaN	-2,1654
-3,73872	-3,21213	-2,89569	NaN	NaN	NaN	NaN	NaN	NaN	NaN
-0,261332	-0,557978	0,438601	0,0669003	0,615467	0,749851	-0,25638	0,461039	-0,519341	0,433055
2,70433	3,93788	2,88295	3,04298	4,71267	4,22285	2,80119	1,44381	0,850945	1,805
2,07928	-0,107588	NaN	NaN	1,50033	-2,50388	1,34045	NaN	NaN	NaN
NaN	-2,46984	NaN	NaN	NaN	NaN	NaN	NaN	NaN	NaN
NaN	3,56087	2,08922	1,87667	3,80407	NaN	2,85809	1,00694	0,797904	4,29411
NaN	NaN	NaN	NaN	NaN	NaN	NaN	NaN	NaN	NaN
-1,02059	-0,988842	-1,29912	-2,63941	0,165302	-1,37486	-1,72114	-0,593958	-0,984761	-0,926875
0,0939445	0,121841	-0,0338631	NaN	-0,169353	-1,19738	-0,206896	NaN	0,122388	0,33183
NaN	NaN	NaN	NaN	-3,36783	-1,08446	NaN	NaN	NaN	NaN
-2,16958	NaN	NaN	-0,931311	-3,39709	NaN	-0,447079	NaN	NaN	NaN
NaN	NaN	NaN	-1,15067	NaN	-0,24297	-0,360554	NaN	NaN	NaN
1,80272	1,81689	1,05831	1,39135	1,00692	2,24946	1,35877	1,78492	0,776393	0,871419
NaN	NaN	NaN	NaN	NaN	NaN	NaN	NaN	NaN	NaN
NaN	NaN	NaN	NaN	NaN	NaN	NaN	-1,27249	-1,7667	-2,95457
NaN	NaN	NaN	-1,94927	-2,05485	-1,59945	NaN	NaN	NaN	NaN
-0,583212	-0,714512	-0,91547	0,448507	0,169884	0,330936	-2,17136	-0,632338	-1,31416	0,592908
2,94707	NaN	2,7144	3,65902	2,99727	4,04742	3,64252	2,92908	2,63706	2,96973
NaN	-1,25945	NaN	-0,869284	-0,149822	-1,60746	0,62999	NaN	NaN	NaN

Polito et al. Universal ready-to-use immunotherapeutic approach for the treatment of cancer: expanded and activated polyclonal γδ memory T cells.
Phosphorylation sites

-2,41305	-2,24435	NaN	-2,2715	-2,52587	-2,00367	-2,88105	NaN	NaN	NaN
3,69828	3,26999	3,99893	3,4507	5,00673	2,40835	3,27394	3,99238	3,53755	3,81861
NaN	NaN	NaN	0,964588	-2,90769	NaN	0,845209	NaN	NaN	NaN
NaN	NaN	1,38174	2,88236	-0,36536	2,04588	1,83616	NaN	NaN	NaN
NaN	NaN	NaN	0,714905	NaN	0,956264	0,372391	NaN	NaN	NaN
NaN	NaN	NaN	NaN	NaN	NaN	NaN	-0,00599861	-0,358274	-1,09165
NaN	1,54053	0,991646	1,25482	2,28655	0,917072	1,65827	NaN	NaN	NaN
1,28595	NaN	0,101875	0,666389	1,06637	NaN	1,18941	1,00855	0,788436	0,697804
2,98889	0,828337	1,86778	1,70411	1,21927	-3,53389	2,20897	2,09212	2,44452	1,90845
-3,33221	NaN	0,296961	-0,659395	-2,24851	0,739408	-0,0863094	-1,12523	-1,22325	-0,856917
-1,7195	-0,654552	-0,0953045	-1,20943	0,352329	NaN	0,0019207	-0,553934	-1,31799	-1,26265
NaN	NaN	-0,955523	-1,43682	NaN	NaN	-1,26766	-0,489658	-0,911023	-0,619849
-2,94274	-2,41572	-2,44413	NaN	NaN	-1,19708	NaN	NaN	NaN	NaN
NaN	NaN	NaN	-1,43508	-2,14089	0,241728	1,08698	1,29867	NaN	1,42352
3,5365	3,76005	3,76643	3,13679	4,03058	3,51228	3,71572	3,3057	3,00195	2,75933
-0,0165672	-0,31045	-0,181812	-0,264257	-0,215849	-2,4833	-0,0896587	0,812168	0,829638	-0,588662
0,4825	NaN	0,29039	3,22011	0,947567	3,32384	2,45345	0,671499	-0,186234	-0,237483
NaN	NaN	NaN	-2,13742	-1,49893	-2,6353	-2,00775	-1,95562	-1,7238	-2,19693
NaN	-1,27701	-1,07186	-0,791914	-0,916245	-0,304686	-0,787943	-0,587646	-0,892745	-0,60431
-1,51098	-1,83247	-1,35653	-1,65086	-1,76784	-1,10192	-1,04038	-1,24557	-1,58917	-1,60557
-2,00474	-2,3562	-2,02211	NaN	NaN	NaN	NaN	-1,96679	-2,06814	NaN
NaN	NaN	NaN	0,843811	NaN	-0,150021	-0,764103	-0,653093	-1,13687	-0,71259
-1,79386	-2,39952	-1,09123	0,226915	NaN	-0,835632	0,34763	-1,95243	-1,25261	-1,89073
NaN	NaN	NaN	NaN	NaN	NaN	NaN	NaN	NaN	NaN
NaN	NaN	NaN	-2,58866	-2,30416	-2,1418	-1,96246	NaN	NaN	NaN
NaN	NaN	NaN	-1,54092	-2,95066	-0,888981	NaN	NaN	NaN	NaN
-2,04876	-1,1027	-1,35539	-1,54767	0,097002	-3,89201	-0,310005	-1,18772	-2,00109	-0,378364
NaN	0,497587	NaN	-1,03994	0,171171	NaN	-0,415291	-0,675983	-0,403998	-0,634667
NaN	NaN	NaN	-0,437744	NaN	-1,0598	-2,48971	NaN	NaN	NaN
NaN	NaN	-0,785748	NaN	NaN	NaN	NaN	1,31981	1,19122	1,38731
NaN	NaN	NaN	NaN	NaN	NaN	NaN	0,474152	0,536191	0,0776758
-1,21073	-1,88813	-1,64712	-1,28873	-1,04972	-1,77879	-1,70568	-1,71112	-1,8654	-2,01795
-0,615198	NaN	-0,507206	-1,05866	-1,21506	-1,9268	NaN	-0,194324	-1,11343	NaN
-1,8757	-1,11216	-2,58685	NaN	-1,37311	NaN	-2,5459	NaN	NaN	-2,78229

0,906504	0,353592	0,578953	-1,98317	0,911728	NaN	-0,769312	NaN	NaN	NaN
-2,22422	-2,30873	NaN	NaN	NaN	NaN	NaN	1,30726	1,10746	1,52262
NaN	NaN	NaN	3,49928	-1,12984	2,74144	0,45562	NaN	NaN	NaN
-3,6225	NaN	-1,40017	0,229004	-1,6104	-1,01777	0,617168	-2,1597	-2,51567	-1,96664
NaN	-1,89286	-2,39726	-0,0722752	-0,468985	0,354092	0,144115	-1,46838	-1,20786	-1,41685
-0,933439	-0,65509	-1,51609	-0,749729	NaN	NaN	-0,621099	-1,1062	-1,11223	-1,48793
NaN	NaN	NaN	NaN	NaN	NaN	NaN	NaN	NaN	NaN
-1,74239	-1,92356	-1,48212	NaN	NaN	NaN	NaN	NaN	NaN	NaN
1,43397	0,415403	NaN	3,98507	-1,59272	NaN	4,18186	1,35767	2,17101	1,27069
-2,02604	-1,89281	-1,45653	1,12582	-2,07125	-0,780991	0,162481	0,167082	0,0835676	-0,106572
-0,0831738	-0,0253811	0,253668	1,6431	-0,0451679	-1,21269	1,43828	1,52354	1,58855	1,35977
-0,190237	NaN	NaN	0,946674	NaN	NaN	0,982847	0,271299	0,0937891	-0,571406
0,260778	1,70399	-0,0855999	1,64168	2,10429	-1,51189	-0,00120735	0,181097	0,00305271	1,83438
-0,786526	1,40061	1,3455	1,05478	1,55234	NaN	1,01262	1,93068	1,6722	2,81345
NaN	NaN	NaN	-1,20164	NaN	-2,12817	-3,17454	NaN	NaN	NaN
-2,2833	-2,06463	-0,0419369	-0,20373	-0,963642	NaN	-1,67524	-0,545792	-0,903562	-0,940289
NaN	-1,62845	-1,28211	-1,29733	NaN	NaN	NaN	NaN	NaN	NaN
NaN	-2,59075	-1,88356	-0,898525	NaN	NaN	-1,16914	NaN	NaN	-2,21461
NaN	NaN	NaN	NaN	NaN	NaN	NaN	NaN	NaN	NaN
NaN	NaN	NaN	NaN	NaN	NaN	NaN	NaN	NaN	NaN
NaN	NaN	NaN	-1,56584	NaN	-1,9159	-2,55756	NaN	NaN	NaN
NaN	NaN	NaN	1,555	NaN	0,733223	NaN	NaN	NaN	NaN
-3,25981	-2,33569	-2,55275	-3,34054	-1,09286	-2,02288	-1,68642	-1,72038	-3,12893	-0,59274
3,27987	2,92223	2,83738	3,9979	4,77662	1,93774	3,30947	3,61091	3,45292	4,51465
0,0683289	-0,412313	-0,319397	-0,325893	0,361521	0,451687	-0,218601	-0,302935	-0,555749	-0,475657
0,577173	1,06169	0,360498	-0,0892467	-0,231821	-0,371281	0,77355	0,804192	0,402705	1,03244
NaN	-1,76537	1,26232	NaN	-0,856386	0,834204	1,5862	0,822641	1,56205	0,733991
3,44871	2,58004	2,4049	4,24068	4,5683	4,42024	3,55608	3,05158	3,49465	3,84069
1,12447	2,35213	0,222469	-0,162945	1,2022	0,432547	0,17651	-0,843061	-0,457234	-0,868937
NaN	NaN	NaN	-0,810717	NaN	-0,271383	-1,82499	NaN	NaN	NaN
NaN	NaN	NaN	NaN	NaN	NaN	NaN	NaN	NaN	NaN
0,00907707	-0,592539	NaN	0,776081	0,834749	0,987362	0,134151	-0,663366	-0,690187	NaN
NaN	NaN	NaN	1,07026	NaN	NaN	2,63097	NaN	1,84268	1,92401
0,654194	NaN	0,0663643	0,479952	NaN	-0,215578	0,352959	0,936621	-5,22541	1,17799

NaN	-0,14608	NaN	0,479952	NaN	NaN	0,352959	NaN	NaN	NaN
NaN	NaN	NaN	NaN	NaN	NaN	NaN	NaN	NaN	NaN
-0,775328	-0,359373	0,206135	-0,755613	-0,558649	-0,550312	1,5584	0,747557	0,326772	0,536212
-4,38951	-2,01027	-2,26505	-2,71079	-3,30708	-2,96821	0,418598	-1,30818	-1,65192	-1,69852
-2,67827	0,217392	-1,48425	-1,80483	-0,462505	NaN	-1,31831	NaN	NaN	NaN
2,55669	3,32526	3,09885	4,0766	2,38537	4,58228	3,86452	4,80696	4,58822	4,48943
NaN	0,0516109	-0,47677	-3,52765	-0,187532	NaN	NaN	1,27938	0,922265	1,3415
NaN	NaN	NaN	-1,3382	-3,41773	-1,44354	-1,23431	NaN	NaN	NaN
-3,45011	-2,59875	-1,6696	-0,636353	-0,685192	0,439898	-0,672138	-2,17111	-1,78814	-2,1238
1,41685	2,20312	1,40618	1,91505	3,05708	1,89614	2,2034	2,50459	2,00808	1,88354
NaN	NaN	NaN	1,66737	0,675571	NaN	0,43745	-0,0019722	0,286916	0,517978
0,26461	-0,151884	NaN	1,39429	1,76896	-1,36647	0,69055	-0,309349	0,611817	0,412391
NaN	NaN	4,40676	3,42549	0,883402	3,78104	3,85627	2,45473	2,32707	2,00028
NaN	NaN	NaN	NaN	NaN	NaN	NaN	NaN	NaN	NaN
-0,918089	-0,77515	-1,73273	NaN	NaN	NaN	NaN	NaN	NaN	NaN
NaN	NaN	NaN	-3,29893	-2,46299	-2,45365	-3,91225	-1,67061	-2,32244	-1,90295
-1,15534	-1,35456	1,03419	-1,16222	-0,706331	NaN	-0,834885	1,8121	1,28838	1,72915
NaN	NaN	NaN	NaN	NaN	NaN	NaN	NaN	NaN	NaN
NaN	NaN	NaN	NaN	NaN	-1,90658	-0,6273	NaN	NaN	NaN
1,7274	2,53094	2,60598	1,81479	3,23422	-0,214048	2,52085	1,9826	1,53619	1,75216
1,59166	2,00126	2,24958	1,01075	2,52913	3,43572	2,07648	2,38648	1,93369	2,35815
NaN	NaN	NaN	-2,97818	-2,1478	NaN	NaN	-1,67754	-1,65812	-2,39695
2,31495	3,56073	3,23293	3,54097	3,48441	4,51065	3,48137	2,63863	2,69376	3,7116
4,98707	4,59916	5,18808	3,76558	5,87078	NaN	5,33709	5,56325	5,65177	5,10355
NaN	NaN	NaN	-1,15914	-3,02455	-1,38497	-1,33096	NaN	NaN	NaN
NaN	NaN	NaN	1,22383	NaN	-1,71489	-0,972332	NaN	NaN	NaN
-0,23144	-0,0259514	-1,6899	1,2096	NaN	NaN	NaN	NaN	NaN	NaN
-1,40097	0,49604	-0,767231	0,649496	1,56355	1,72864	1,1981	-0,833429	-0,665189	0,210105
-1,1193	NaN	-0,832241	-1,68184	-0,611925	-2,21135	-2,87694	NaN	0,0389891	NaN
NaN	NaN	NaN	-1,09668	-0,65793	-1,17957	-1,52375	-1,1465	-0,773767	-1,41918
-1,79812	-1,35768	NaN	-0,085165	NaN	NaN	-1,80026	-1,37486	-1,31278	-2,22327
-0,635027	-1,3273	-0,310247	-1,70203	0,494326	-1,51367	-0,625782	-0,533356	-0,624148	-0,832763
NaN	NaN	NaN	-0,933908	-2,5619	NaN	-0,324959	NaN	NaN	NaN
2,00832	1,05383	1,71437	0,518063	2,15171	0,707842	0,567863	1,53089	0,566005	0,909303

Polito et al. Universal ready-to-use immunotherapeutic approach for the treatment of cancer: expanded and activated polyclonal γδ memory T cells.
Phosphorylation sites

NaN	NaN	NaN	NaN	NaN	NaN	NaN	-1,7844	-1,54703	-1,21249
NaN	NaN	NaN	NaN	NaN	NaN	NaN	-1,7844	-1,54703	-1,21249
NaN	NaN	NaN	-0,695644	NaN	0,335312	-2,5027	NaN	NaN	NaN
NaN	NaN	NaN	-1,79109	-2,70692	-2,39039	-2,21707	-2,69746	NaN	-3,24447
NaN	NaN	NaN	0,465115	-2,26427	1,26674	-0,499348	NaN	NaN	NaN
NaN	NaN	NaN	-1,02539	NaN	-2,16655	-0,909697	NaN	NaN	NaN
NaN	-2,59141	-2,24461	-2,30063	-2,11751	NaN	-1,4933	-1,42173	-1,74653	-2,20771
NaN	0,152018	NaN	1,03733	NaN	0,153009	0,178249	NaN	-1,21556	-0,106275
NaN	NaN	NaN	0,428295	NaN	0,443075	-1,19936	NaN	NaN	NaN
NaN	NaN	NaN	0,0765362	NaN	-0,596947	-1,65934	NaN	NaN	NaN
1,07638	1,52735	1,53281	1,62103	0,205259	1,70872	1,2016	1,36418	1,78255	1,31895
NaN	NaN	NaN	1,232	-1,16332	NaN	-0,0387516	NaN	NaN	NaN
1,58832	1,23431	1,35016	1,36847	1,04021	NaN	0,671734	1,44757	1,63861	1,16013
NaN	NaN	-0,334927	0,411531	NaN	2,25077	0,724352	NaN	NaN	NaN
NaN	NaN	NaN	1,18669	-1,56377	NaN	1,1559	NaN	NaN	NaN
0,26409	1,35384	0,00790596	1,42255	0,965164	-1,8823	1,91104	0,337168	0,198585	0,24503
NaN	NaN	NaN	-2,46061	-3,25838	-1,84191	NaN	NaN	NaN	NaN
-0,902693	0,0956783	NaN	0,726728	-0,657526	NaN	-0,358747	-0,554886	0,0925589	-0,606965
NaN	NaN	NaN	1,40467	NaN	1,80576	-2,32311	NaN	NaN	NaN
NaN	-0,883322	NaN	0,0617657	NaN	0,436714	-1,1528	-2,33335	-1,76761	-0,43889
0,987873	0,767683	0,917164	0,763035	0,881643	-0,212519	1,90985	-0,469452	0,373384	-0,368554
NaN	NaN	NaN	0,546282	NaN	-1,31631	0,844711	NaN	NaN	NaN
NaN	NaN	NaN	NaN	NaN	NaN	NaN	NaN	NaN	NaN
-2,13937	-2,34018	-1,83594	-1,20379	-2,40233	0,779963	-0,691172	NaN	NaN	NaN
1,47839	NaN	NaN	1,24965	NaN	1,53042	0,983187	NaN	NaN	NaN
NaN	NaN	NaN	-1,00584	-1,52996	-1,50194	-1,56171	-1,21097	-1,61545	NaN
-0,931522	-0,373249	-1,5668	0,1747	-0,123814	0,275385	-0,244207	-1,9808	-1,78659	-0,691831
NaN	NaN	NaN	-2,73115	NaN	-1,6013	-3,04082	NaN	NaN	NaN
NaN	NaN	NaN	NaN	NaN	NaN	NaN	NaN	NaN	NaN
NaN	NaN	NaN	-0,384943	NaN	-1,53751	NaN	NaN	NaN	NaN
0,922136	2,2206	1,99346	-1,93072	2,98884	0,489565	-0,793936	NaN	NaN	NaN
-2,44757	NaN	-1,73724	NaN	-1,59727	-2,36387	NaN	-1,69278	-1,73987	-2,43914
-2,11799	-2,96578	-2,06821	-0,822926	-2,08856	-0,458168	0,0199528	-1,13199	-1,09583	-1,15852
0,297945	0,82667	-0,223324	NaN	1,15778	NaN	NaN	1,16312	0,562537	0,597709

Polito et al. Universal ready-to-use immunotherapeutic approach for the treatment of cancer: expanded and activated polyclonal γδ memory T cells.
Phosphorylation sites

-1,66809	-0,923355	NaN	-1,16387	NaN	NaN	NaN	-1,27599	-1,83041	-1,32045
2,03558	1,7837	2,23997	1,78747	2,14305	-3,2158	1,97224	1,96941	1,82125	1,75413
NaN	NaN	NaN	-0,589794	-1,15951	0,430504	0,482388	NaN	NaN	NaN
-0,848434	0,749081	-0,275581	0,724728	0,573229	-0,687126	1,08435	-0,453722	-0,654529	0,712165
-4,38526	NaN	1,03956	1,75625	1,40079	1,93304	1,68833	NaN	NaN	0,785226
NaN	NaN	NaN	-1,56182	-2,63717	NaN	-1,63908	-2,08785	-2,15398	-3,2387
-0,428883	-1,38272	-1,18018	-0,593369	-1,41268	0,928703	-0,260605	-1,75405	-0,910331	-1,37601
NaN	NaN	NaN	-0,593313	-0,573339	-1,26532	-0,631136	-1,64091	-2,34165	-1,40513
NaN	NaN	NaN	0,65686	NaN	0,873064	NaN	NaN	NaN	NaN
-0,676056	NaN	-1,57701	0,276762	-0,193373	0,921888	-0,143663	-2,00382	-2,74943	-0,303214
NaN	NaN	NaN	-0,580364	NaN	-0,123016	-1,44048	NaN	NaN	NaN
NaN	NaN	NaN	-1,33927	NaN	-0,150152	-3,30026	NaN	NaN	NaN
-0,90905	-0,525213	-1,38952	-2,79083	-1,33187	-2,04634	-1,58364	NaN	NaN	NaN
-2,06903	-1,24118	-2,21321	-1,91109	NaN	NaN	-1,68017	NaN	NaN	NaN
0,0940075	1,85528	1,39437	0,851675	-1,25121	-2,73717	1,48939	-1,20211	1,74883	-0,455598
NaN	NaN	NaN	NaN	NaN	NaN	NaN	NaN	NaN	NaN
1,47759	1,93953	1,94462	3,20179	NaN	3,55573	2,99085	NaN	NaN	NaN
NaN	NaN	NaN	-1,46425	NaN	-0,572113	-1,38556	NaN	NaN	NaN
1,69757	0,0390358	-0,87479	-0,832058	NaN	NaN	-1,53582	0,419775	0,215304	-0,449286
-0,778463	-0,985723	-1,46927	0,178164	0,303679	-0,143387	0,222744	-0,149813	-0,33302	0,00754261
NaN	NaN	NaN	-2,38492	NaN	-0,48152	-2,32132	NaN	NaN	NaN
-1,67499	-1,94392	-1,32046	-1,63022	-1,59822	-1,48129	-1,52922	NaN	NaN	NaN
0,87472	0,221272	0,563545	0,944574	1,33402	1,69934	1,62627	0,224247	0,218499	-0,0130358
-1,36574	-0,683531	-1,35718	NaN	NaN	NaN	-1,32619	-0,231031	-1,16898	-0,492414
1,66655	0,562843	NaN	-3,25284	1,94624	NaN	-0,00977516	1,15306	0,728925	1,64294
-0,232067	0,629814	0,911766	NaN	0,0433273	NaN	NaN	0,0250969	0,077651	1,18315
0,059618	-1,14575	1,47931	1,87692	2,65842	3,15559	2,61362	NaN	NaN	NaN
-0,00174332	-3,05826	0,41296	-1,43394	-0,179783	NaN	-0,104263	-0,217134	0,0640421	-0,366244
3,00081	1,84057	1,84908	1,65733	1,58493	-0,606915	1,21985	1,68281	-1,2631	0,507426
3,00674	1,9392	1,77947	1,92074	0,787432	3,67517	1,32054	2,94985	3,50806	NaN
1,05762	0,196743	NaN	1,48961	-1,73226	2,5787	0,215141	NaN	NaN	0,114497
NaN	NaN	NaN	-1,67648	NaN	-0,519341	-2,22123	NaN	NaN	NaN
0,466597	0,860439	1,17817	1,34572	2,03293	1,46668	0,868284	-0,367758	-0,370391	-1,02382
1,86642	1,10224	1,96778	1,30181	1,59683	0,32061	1,44404	2,01862	1,7599	1,54544

Polito et al. Universal ready-to-use immunotherapeutic approach for the treatment of cancer: expanded and activated polyclonal γδ memory T cells.
Phosphorylation sites

NaN	NaN	NaN	-1,75769	NaN	-0,971052	-1,23295	NaN	NaN	NaN
1,48861	2,03909	1,25966	1,11966	NaN	NaN	1,54712	1,37826	NaN	0,959495
NaN	NaN	NaN	1,2989	NaN	1,14246	0,788895	NaN	NaN	NaN
NaN	NaN	NaN	-0,753815	-2,78775	-0,661007	-1,39229	NaN	NaN	NaN
NaN	NaN	NaN	-0,640072	NaN	-1,31449	-1,32211	NaN	NaN	NaN
-0,0788059	1,27865	0,229284	0,687431	1,57676	-0,243679	1,10942	1,01252	0,925616	0,943116
-0,111132	1,09036	1,16944	0,951721	2,15969	0,483063	1,50247	1,35378	0,608054	1,41503
-1,0841	-1,10036	-1,65617	NaN	0,266933	NaN	-2,59893	-1,41022	-1,58874	-0,862454
NaN	NaN	NaN	0,514593	NaN	0,780521	-0,605545	NaN	NaN	NaN
2,28613	2,90051	2,59015	3,7232	3,34617	2,41594	4,17804	1,47609	1,9368	1,75161
3,93203	-0,927853	3,13884	3,90266	3,62805	NaN	3,87672	3,30027	2,01185	1,98163
1,82538	-0,673086	0,402414	0,737793	1,40649	-0,409494	0,744499	0,567287	-0,134799	0,00486851
2,00098	1,63439	0,825407	1,96119	2,87633	1,22516	2,68069	1,54933	1,00471	1,40301
1,0395	NaN	1,10004	1,28629	2,32718	NaN	1,5108	1,52657	1,2851	2,7701
1,83179	0,320042	1,30883	0,151567	2,13853	NaN	1,12897	1,39505	1,16631	1,00033
NaN	NaN	NaN	-1,8013	NaN	NaN	NaN	NaN	NaN	NaN
NaN	NaN	NaN	NaN	NaN	NaN	NaN	NaN	NaN	NaN
NaN	NaN	NaN	NaN	NaN	NaN	NaN	NaN	NaN	NaN
-0,833204	-2,81697	0,575686	-0,159819	1,38095	NaN	1,38571	0,884207	0,993629	0,163758
1,62011	1,33858	1,45995	3,14042	1,56892	-1,66216	3,50841	1,55503	1,45249	0,987775
NaN	NaN	NaN	-1,0227	-1,02573	-0,56628	-0,532227	NaN	NaN	NaN
NaN	NaN	NaN	NaN	NaN	NaN	NaN	NaN	NaN	NaN
NaN	2,23851	2,99683	2,46055	1,12478	2,61011	2,76437	1,85023	2,89272	1,90857
NaN	NaN	-1,51167	-3,29092	NaN	-2,91536	-1,39505	NaN	NaN	NaN
-1,49977	-3,19866	-0,149784	-2,05468	-1,39582	-2,31309	-1,06444	-1,35029	-2,23643	-2,03009
-1,25282	-1,54185	-1,03926	0,225739	-0,557686	-0,0736275	0,672575	-0,0281849	0,0268011	0,154119
0,599825	0,932241	1,19808	2,41471	NaN	1,07022	2,38412	1,56011	1,45024	1,24623
0,819889	0,521008	1,71746	1,15385	2,52514	NaN	1,82409	2,17643	2,00292	1,8849
-2,4951	NaN	-0,60022	-1,42089	-2,80813	-0,233076	-1,01623	-0,851318	-1,00544	-2,57489
3,607	NaN	3,67126	4,41624	4,00166	NaN	4,31503	4,22092	NaN	4,50184
-0,180239	-0,0282688	2,1763	1,97309	1,19078	NaN	2,26695	NaN	0,00501537	0,749227
2,02207	1,81923	2,50186	NaN	2,85589	2,4556	1,48061	2,44152	2,20129	2,49394
NaN	NaN	NaN	NaN	-2,16939	NaN	NaN	-2,23473	-2,7897	-2,91784
1,40409	1,97356	1,10881	1,50297	2,10277	0,288395	1,94196	1,56981	1,8489	2,19131

Polito et al. Universal ready-to-use immunotherapeutic approach for the treatment of cancer: expanded and activated polyclonal γδ memory T cells.
Phosphorylation sites

-0,576805	-0,0799255	-0,247856	-0,853687	0,429493	NaN	-0,941532	-1,01734	-0,977958	0,0523825
1,27989	0,764568	0,849016	-3,49522	1,9122	NaN	-1,65413	1,19037	1,39048	1,43799
-1,98986	NaN	0,182814	-1,68166	NaN	-1,2199	-0,864809	0,789301	0,767867	0,418946
4,20937	4,54534	4,39693	4,21824	4,73772	2,02915	4,87035	-0,251671	4,24408	4,33653
NaN	NaN	NaN	-1,20422	NaN	-1,75344	-1,12388	NaN	NaN	NaN
NaN	NaN	NaN	-2,14912	NaN	NaN	-2,44595	NaN	NaN	NaN
-2,06651	-2,43294	-2,01027	-0,698267	NaN	NaN	-1,59043	NaN	NaN	NaN
NaN	NaN	NaN	-0,442158	NaN	NaN	NaN	NaN	NaN	NaN
-1,70458	-0,0131149	-0,573162	0,415672	0,501776	3,05753	1,14856	-1,87509	-1,62858	-0,248046
NaN	NaN	NaN	NaN	NaN	NaN	NaN	-2,17685	-2,55382	-2,65792
NaN	NaN	NaN	NaN	NaN	NaN	NaN	0,66954	0,819448	0,832257
2,36099	1,79281	1,20839	1,07044	1,31825	1,19789	3,40554	1,13421	1,31977	1,23711
NaN	NaN	NaN	NaN	NaN	NaN	NaN	NaN	NaN	NaN
NaN	-4,0916	-2,6936	-3,0301	-3,25944	-2,05623	NaN	-2,48442	-2,99011	-2,6952
-1,62815	-1,391	-1,46815	-0,760494	-0,940866	NaN	-0,277435	-1,43031	-1,81466	-1,33722
NaN	NaN	NaN	NaN	NaN	NaN	NaN	NaN	NaN	NaN
NaN	NaN	1,96876	NaN	2,09527	0,414139	NaN	1,19354	1,02642	0,747041
NaN	NaN	-0,38448	NaN	NaN	NaN	NaN	-1,56139	-1,78595	-1,31421
0,25757	0,192524	0,395905	-0,253569	0,348963	-1,41594	0,416306	0,586582	0,394959	-0,299369
0,534355	0,275175	1,15247	0,763144	-0,00829887	1,29359	0,447908	0,732248	1,30217	0,364461
-0,05299	0,492945	1,08802	0,627533	3,04941	-2,37869	2,11165	0,501242	0,709365	1,22292
2,34513	2,72866	1,98449	0,815054	3,21661	-3,14328	1,13009	2,21712	2,22311	2,67531
-1,57411	-0,678661	NaN	NaN	-0,112764	NaN	0,466076	NaN	NaN	0,383513
-1,65996	-0,815157	-0,101637	-0,151793	-0,0931911	0,446157	0,507034	0,103964	-0,491712	NaN
NaN	NaN	NaN	NaN	NaN	NaN	NaN	NaN	NaN	NaN
0,178085	-0,416571	0,692823	-0,223919	0,342766	0,434145	0,0600109	0,819818	0,69284	0,566157
1,28127	0,108408	2,86415	-0,792677	1,42111	-0,355494	NaN	4,12769	3,98826	3,87129
NaN	NaN	NaN	-0,475355	NaN	NaN	-2,528	NaN	NaN	NaN
-1,08528	NaN	-0,983749	-0,856247	-0,847509	-1,72769	NaN	NaN	NaN	NaN
-1,44654	NaN	-2,53544	-0,757135	-1,84541	NaN	-1,0096	-1,04682	-0,939101	-1,5128
NaN	NaN	NaN	-1,19354	-1,20683	-0,284782	-0,209452	NaN	NaN	NaN
NaN	NaN	NaN	-0,429056	-0,215487	NaN	NaN	NaN	NaN	NaN
0,308868	0,963573	0,420534	0,235592	1,73623	NaN	1,6429	0,691624	0,629556	1,04873
NaN	NaN	NaN	NaN	NaN	NaN	-1,10165	0,192776	0,427743	-0,374461

Polito et al. Universal ready-to-use immunotherapeutic approach for the treatment of cancer: expanded and activated polyclonal γδ memory T cells.
Phosphorylation sites

NaN	NaN	NaN	NaN	NaN	NaN	NaN	NaN	NaN	NaN
2,24679	NaN	1,33973	3,06818	NaN	3,1858	2,12994	2,35572	2,02753	3,28867
-1,1229	-1,71843	-1,51973	-0,934856	NaN	-0,263105	-0,913271	NaN	NaN	NaN
2,70403	1,77458	2,75506	2,75592	2,82886	2,3926	2,62123	1,7847	1,42548	1,62765
0,990234	0,1091	0,2334	NaN	1,2502	-0,0178452	NaN	0,0787964	-0,338326	-0,408751
NaN	NaN	NaN	NaN	NaN	NaN	NaN	NaN	NaN	NaN
0,222361	2,70272	-0,19323	0,48469	0,602474	0,187723	1,57094	0,639387	1,58807	0,979186
NaN	NaN	-1,39381	-1,75894	0,384506	-1,95821	-2,65019	NaN	-1,46656	NaN
0,581148	0,427153	0,894196	0,927666	0,837074	0,54137	1,55292	1,10447	0,677918	0,663733
NaN	NaN	NaN	-1,12663	NaN	-1,92657	-2,34793	NaN	NaN	NaN
NaN	NaN	NaN	NaN	NaN	NaN	NaN	NaN	NaN	NaN
NaN	NaN	NaN	-0,628574	NaN	-3,84688	0,234341	NaN	NaN	NaN
NaN	NaN	NaN	0,578152	NaN	NaN	NaN	NaN	NaN	NaN
-0,0569553	1,08824	0,307447	1,24128	1,26447	2,21992	1,37101	0,0400944	-0,389472	0,561219
0,547029	0,713802	0,365986	0,280703	NaN	0,285351	0,209566	0,838909	0,677533	0,506537
0,458693	0,268208	0,45154	1,02618	0,374628	1,28928	0,698421	0,344002	0,136674	0,35706
-0,485008	0,294611	-0,880085	NaN	-1,64413	NaN	-0,339956	-0,828726	-0,753016	-0,879706
2,09974	2,76844	1,73349	2,38101	2,089	1,9146	2,38754	2,03563	2,37387	1,89906
0,363308	NaN	0,408527	1,04777	-0,668827	NaN	0,734863	NaN	NaN	NaN
-1,03745	-0,0315151	-0,728434	-0,691364	-0,854534	-0,0413303	-0,646761	-1,53816	-0,964728	-1,73434
-0,861839	1,21283	0,143383	0,0222912	0,425879	NaN	0,216276	-0,478529	-0,871427	0,440177
NaN	NaN	NaN	-1,71037	NaN	NaN	-1,17877	NaN	NaN	NaN
1,12508	3,44592	1,67541	1,86723	0,973269	0,842047	2,33128	0,669964	1,73497	1,77239
NaN	-1,33931	NaN	-0,521128	-0,0472832	NaN	-0,823055	-0,391968	-0,332761	-0,579305
0,627352	0,64394	0,199375	0,914658	0,59144	1,24193	1,06055	1,19681	1,36012	0,799273
-1,09512	-0,219473	-0,644993	NaN	-0,440886	NaN	NaN	-0,030756	-0,465083	NaN
NaN	NaN	NaN	-2,68727	-2,47172	-1,7555	NaN	NaN	NaN	NaN
-1,12246	-1,08434	NaN	-0,230438	NaN	-0,892202	-1,51561	NaN	NaN	NaN
-1,90683	-2,19366	3,61279	3,57937	NaN	NaN	NaN	3,15651	3,18911	3,13897
3,12595	3,32796	-1,91407	3,57937	3,53405	3,27268	3,42027	3,15651	3,18911	-2,29804
-1,4837	-1,58145	-3,13395	NaN	-0,642624	NaN	-1,50943	NaN	NaN	NaN
0,146008	0,753523	0,715075	-1,55074	0,471951	0,797789	0,063715	0,411003	0,287454	0,349336
0,496759	-2,05262	-0,138096	-1,28147	NaN	NaN	-0,64365	NaN	NaN	NaN
-1,80606	-2,0914	-1,07923	NaN	-1,21863	-2,90393	NaN	-0,434954	-0,719537	-0,807038

Polito et al. Universal ready-to-use immunotherapeutic approach for the treatment of cancer: expanded and activated polyclonal γδ memory T cells.
Phosphorylation sites

-0,922863	NaN	0,275442	-0,723661	0,176037	0,821106	-0,01548	1,08379	0,874963	1,21345
0,906027	0,789299	0,928011	1,26281	-0,00578308	1,71333	1,0446	0,794014	0,582534	0,0459948
NaN	NaN	NaN	NaN	NaN	NaN	NaN	NaN	NaN	NaN
NaN	NaN	NaN	-0,542202	-2,68436	0,374109	-0,589617	NaN	NaN	NaN
-1,77174	NaN	-0,826065	-0,246834	-0,00324631	-0,179167	-0,448877	NaN	-2,10684	-1,93024
NaN	NaN	NaN	NaN	NaN	NaN	NaN	NaN	NaN	NaN
-1,91767	-3,13077	-2,57089	-1,2445	-2,40248	-1,48052	-1,40774	NaN	NaN	NaN
-0,0968819	-1,13864	-0,309355	1,026	0,571642	0,712969	1,32742	-0,764141	-0,466187	-0,697997
-2,91267	NaN	-2,25581	-2,86965	-2,02851	-1,84613	NaN	-2,97641	NaN	-2,24323
NaN	NaN	NaN	NaN	NaN	NaN	NaN	NaN	NaN	NaN
NaN	NaN	NaN	-1,47965	NaN	-1,61743	-2,0073	NaN	NaN	NaN
NaN	NaN	NaN	-1,04213	NaN	NaN	-2,00766	NaN	NaN	NaN
NaN	-0,801435	-0,941328	-0,167479	-0,768953	0,428635	0,12496	NaN	NaN	NaN
-1,04784	-2,19644	-2,22466	1,94849	-0,888346	1,64528	-1,25849	-1,5277	-1,36407	-1,43089
NaN	NaN	NaN	-0,896471	-1,13482	-4,44991	-1,72875	NaN	NaN	NaN
NaN	NaN	NaN	NaN	NaN	NaN	NaN	NaN	NaN	NaN
-1,5979	-2,36179	-0,942429	0,209503	-1,4575	-0,256649	0,38278	NaN	NaN	NaN
NaN	NaN	-0,645456	0,0689163	NaN	-1,04999	0,00448608	NaN	NaN	NaN
NaN	NaN	NaN	-1,49641	-0,838024	0,828209	NaN	NaN	NaN	NaN
3,59729	3,34274	3,36905	2,63959	3,82274	2,90114	3,37293	3,77836	3,36828	3,31872
3,00039	0,543308	1,63851	0,754452	1,67504	NaN	0,482485	2,57576	2,18528	1,46218
-0,96172	-0,645239	NaN	-0,235319	-2,36795	NaN	-1,03919	-1,51488	-1,62522	-1,89712
-1,77472	-1,85107	NaN	-1,93212	NaN	-1,04751	-3,69418	NaN	NaN	NaN
-0,491898	NaN	-0,446184	-0,273605	-0,47747	NaN	0,0778217	0,18582	-1,24953	NaN
0,631464	0,465567	0,774397	0,184454	1,19538	-1,18769	1,18233	1,02519	0,392352	0,967614
NaN	NaN	-0,345875	0,188429	NaN	0,146528	0,426933	NaN	NaN	NaN
0,0944462	0,527399	0,516119	0,625032	0,522093	0,746712	0,509029	0,288433	0,29061	-0,257205
-0,735018	-0,02174	-0,141342	-0,777756	NaN	-0,916653	0,209528	NaN	NaN	NaN
1,05118	0,862068	1,17066	1,01102	1,09753	1,46486	1,11552	1,17863	0,568082	0,794782
-0,564375	-0,222885	-0,839695	-4,35328	-0,353577	NaN	NaN	NaN	NaN	NaN
-2,08013	-1,78897	-1,81208	NaN	NaN	NaN	NaN	NaN	NaN	NaN
NaN	NaN	NaN	-1,12944	0,876495	-2,25296	-0,640547	NaN	0,254722	-0,0819502
NaN	2,77485	NaN	2,34698	2,2183	NaN	3,85581	0,346731	1,16237	2,13422
1,0865	2,32225	2,48504	1,23676	2,29544	NaN	NaN	1,73574	1,58438	1,29633

Polito et al. Universal ready-to-use immunotherapeutic approach for the treatment of cancer: expanded and activated polyclonal γδ memory T cells.
Phosphorylation sites

-1,65018	-1,82771	-3,7216	NaN	NaN	NaN	NaN	-0,625395	-0,999406	-1,25737
2,22815	2,14019	1,54049	2,22427	2,37521	2,95268	2,42261	1,87933	1,53414	2,0391
1,75398	-0,143549	1,34966	0,595531	NaN	NaN	1,04146	NaN	NaN	NaN
NaN	NaN	NaN	NaN	NaN	NaN	NaN	NaN	NaN	NaN
0,342001	0,106615	0,389931	-1,40274	0,15571	NaN	0,103279	0,629473	0,515824	0,124825
2,09258	1,6398	1,19975	1,08845	2,38662	0,826656	0,913832	1,1816	1,27937	1,70718
-2,00501	-0,952454	-2,10549	-3,49914	-1,52602	NaN	-2,38924	-2,00192	NaN	-2,22627
-0,389055	NaN	-2,05062	NaN	NaN	NaN	NaN	-1,70436	-1,86279	-2,75312
-1,50647	NaN	-0,924816	-1,0789	NaN	-1,23071	-0,612156	-1,44314	-0,879855	0,0612516
-0,662426	NaN	NaN	-1,97577	-1,30268	NaN	-1,62392	NaN	NaN	NaN
NaN	NaN	NaN	NaN	NaN	NaN	NaN	NaN	NaN	NaN
NaN	NaN	NaN	0,643272	-0,459532	0,755398	0,0353642	NaN	NaN	NaN
1,60015	0,226074	0,727417	1,00819	1,60853	NaN	0,613054	1,37816	1,01591	0,856542
NaN	NaN	NaN	-0,790195	NaN	-0,87709	-1,77366	NaN	NaN	NaN
-1,45484	-1,05392	NaN	-0,450298	-0,886978	NaN	-0,536951	-2,815	-2,06753	-1,32091
-0,556673	NaN	NaN	-0,709555	NaN	-0,148956	-1,10891	NaN	NaN	NaN
0,197447	0,297403	0,310081	-0,124903	-0,928963	0,194965	0,0630512	-0,155857	0,422076	0,0176439
NaN	NaN	NaN	-1,9566	NaN	-3,27269	-1,29038	-1,48952	-1,77664	-1,38323
NaN	NaN	NaN	-0,0065403	-0,941031	-1,06666	-1,18481	-1,40472	NaN	-1,38701
NaN	-3,62133	NaN	-2,35894	-2,64415	-1,95482	-1,89292	NaN	NaN	NaN
0,995171	-0,0262699	0,313601	NaN	1,63193	NaN	-0,593018	0,722652	0,470761	0,388852
NaN	NaN	NaN	-2,07197	NaN	NaN	NaN	NaN	NaN	NaN
NaN	NaN	NaN	NaN	NaN	NaN	NaN	-2,65927	-2,21561	-2,57475
NaN	NaN	NaN	NaN	NaN	NaN	NaN	NaN	NaN	NaN
-1,34672	-0,211044	-1,70433	-0,599232	NaN	-1,03882	-0,624678	-1,1636	0,253638	-0,793412
-0,436626	0,628304	-1,2002	-0,680952	NaN	-0,502111	0,734863	-0,23033	-0,349936	-0,423541
0,380676	1,05451	-0,986181	0,6724	-1,01851	1,98343	0,16663	-0,0924168	0,135293	-0,700681
-0,25075	-0,101507	-1,08722	-0,804956	0,938353	NaN	NaN	0,0401173	0,0961695	-0,513553
NaN	NaN	NaN	-0,688427	-2,80911	NaN	-1,06992	-1,45341	-1,71246	-1,69391
NaN	NaN	-2,48224	-1,69785	NaN	NaN	-1,99389	-2,25007	-2,0256	-2,77172
NaN	NaN	NaN	-0,743931	-2,23306	-0,283964	NaN	NaN	NaN	NaN
3,16949	3,11143	2,97449	2,81518	-2,27634	3,28638	3,12057	3,95732	4,28485	3,8413
0,109793	-1,01662	1,81857	0,910166	NaN	-0,0912647	1,11851	NaN	1,02046	0,656209
1,41443	-1,17159	0,388491	0,0982513	0,979065	1,1824	0,555782	0,335033	0,287824	0,230668

Polito et al. Universal ready-to-use immunotherapeutic approach for the treatment of cancer: expanded and activated polyclonal γδ memory T cells.
Phosphorylation sites

-0,772007	0,476131	0,968868	0,95248	-1,75416	1,91285	1,55908	-0,499216	0,0663157	-0,237103
-0,443743	0,832279	-1,16047	-0,137062	0,894167	0,18351	-0,722431	0,123648	0,273049	0,292197
-1,83747	-1,92694	-2,05379	NaN	NaN	NaN	NaN	NaN	NaN	NaN
0,519468	NaN	-0,747561	-1,68349	-0,271151	-0,69875	NaN	NaN	NaN	NaN
0,678244	0,317293	-0,29093	-2,66869	0,556313	NaN	-0,802641	-0,659895	-0,83878	-0,801429
NaN	NaN	NaN	-0,781385	-0,591003	-0,827784	-0,958109	NaN	NaN	NaN
NaN	NaN	NaN	-2,15181	NaN	-2,41613	-2,43153	NaN	NaN	NaN
-1,26865	-1,0195	-0,849859	-0,265959	-0,218792	-1,60856	1,18882	-0,444019	NaN	NaN
NaN	NaN	-0,638708	0,0105686	0,516003	0,46286	0,166887	-0,441814	-0,272363	-0,595511
2,64319	1,63102	2,05213	3,12641	2,3312	3,25019	2,34058	1,91045	1,32625	1,53074
NaN	NaN	NaN	-0,553326	-1,24608	NaN	-1,20646	NaN	NaN	NaN
NaN	NaN	NaN	NaN	-0,75997	-1,06267	-1,04535	NaN	NaN	-1,87389
NaN	NaN	NaN	-0,258881	NaN	-0,892559	-2,03305	NaN	NaN	NaN
-1,19644	0,874357	0,132828	NaN	NaN	0,400312	0,0683041	NaN	1,49116	-0,695765
0,396685	-0,576653	-0,724192	NaN	-0,909819	NaN	NaN	-1,14153	-1,05214	-1,49775
NaN	-0,435785	-0,6294	NaN	-0,260689	-1,00632	NaN	-1,5741	-0,780162	-1,0991
NaN	NaN	-1,49443	-1,03229	-2,48312	0,240511	-2,15355	NaN	NaN	NaN
-1,92468	-1,50177	-1,40611	-0,900377	-0,0820122	-1,10625	-0,784397	-0,760588	-1,33527	-0,842031
1,07298	1,91471	1,86964	0,737352	2,70006	0,288004	1,73755	2,17813	1,19068	2,16413
-0,395998	-0,0384254	-0,800587	-1,20786	-0,337896	NaN	NaN	0,0911179	-1,29954	-0,65335
-0,319693	1,45162	-0,517576	NaN	1,53841	0,992792	NaN	0,0783005	-0,499984	0,708238
-0,686903	-1,46686	0,183636	-2,8213	0,335236	NaN	-2,51581	0,231424	-0,101039	-0,309514
2,74941	3,36613	3,60071	2,89285	3,55607	2,77426	3,08235	3,33662	3,29297	3,17186
-0,672815	0,186514	-0,482563	-0,306667	-0,629633	-1,13528	-0,51086	-0,79991	-0,606805	-0,900418
-1,07349	NaN	NaN	NaN	-0,718023	NaN	-1,49474	-0,690966	-0,246659	-1,0369
NaN	NaN	NaN	-0,317806	NaN	0,155594	-0,350744	-1,46494	-0,930633	-1,5753
NaN	NaN	NaN	NaN	0,542421	NaN	NaN	-2,65826	-3,01135	1,3315
1,76131	1,57764	2,65054	2,27011	2,59094	2,49952	2,35109	3,66099	2,85219	2,76146
NaN	-1,40904	-1,53753	-1,88139	-0,389441	NaN	NaN	-0,668947	-1,08509	-0,897252
-1,89143	NaN	0,135956	-0,665125	-0,490755	-1,76032	-1,52665	0,166548	-0,0231123	0,728461
NaN	NaN	NaN	-1,40917	NaN	-1,24445	-1,5039	NaN	NaN	NaN
-0,163342	0,575653	0,476341	NaN	0,821697	NaN	0,359907	0,772228	0,337623	0,774802
-0,634645	-0,891996	-0,0412388	NaN	NaN	NaN	NaN	-0,520704	NaN	NaN
NaN	0,214844	NaN	-2,26857	-1,06154	0,141975	-2,6857	NaN	NaN	NaN

Polito et al. Universal ready-to-use immunotherapeutic approach for the treatment of cancer: expanded and activated polyclonal γδ memory T cells.
Phosphorylation sites

0,0673923	0,0431843	-0,611477	0,436876	-0,141298	0,860909	0,467791	0,070055	-0,484033	-0,671256
1,95992	2,82542	1,79525	1,89012	1,43116	1,67666	2,34208	1,75486	3,19653	2,15251
0,915998	1,7837	0,234068	1,07751	-0,125177	1,2783	1,37851	-0,451639	0,821771	-0,207044
-2,64717	NaN	-2,66562	NaN	NaN	3,14737	NaN	-2,5007	-2,62605	-2,47072
2,87691	2,71456	2,72222	2,87969	3,10839	NaN	3,47365	3,1224	3,60637	2,76356
-2,07213	0,14851	-2,06634	-1,54864	NaN	NaN	-0,305302	NaN	NaN	-1,70322
NaN	NaN	NaN	NaN	NaN	NaN	NaN	NaN	NaN	NaN
-2,02531	-2,90139	-1,16827	NaN	NaN	NaN	NaN	-1,4036	-1,51708	-1,95005
NaN	NaN	NaN	-1,34412	-0,976116	-1,4993	NaN	NaN	NaN	NaN
NaN	NaN	NaN	-1,34412	-0,976116	-1,4993	-1,87003	NaN	-1,68194	-0,476329
NaN	NaN	NaN	-0,19207	-2,23747	0,417374	-0,697102	NaN	NaN	NaN
-0,480019	-0,295019	-0,555546	0,739996	-0,136415	0,231762	0,835974	-0,41184	-0,990983	-0,831578
-1,81091	NaN	1,1685	1,26801	NaN	2,05589	1,59399	NaN	NaN	NaN
1,40384	1,66005	1,56017	2,68186	1,97777	2,52051	3,64471	1,5052	1,45884	1,44425
-0,184011	-0,551182	-0,199543	-0,632017	0,393539	0	-0,0901585	-0,555143	-0,62197	-1,20951
-0,63822	-1,21531	-1,3337	-0,954599	0,0617771	-0,517862	-0,0457935	-1,36005	-1,29879	-0,847388
2,64876	2,94148	2,07535	2,36323	3,2327	2,31948	2,69245	1,76789	1,90638	1,6521
NaN	NaN	NaN	0,292067	NaN	0,809254	0,644545	-1,39269	-0,765758	-0,387521
-2,68888	-2,51454	-0,927999	-0,506327	NaN	0,76495	-0,0912056	NaN	NaN	NaN
-1,5085	-1,77047	NaN	-1,0559	NaN	-0,721983	-1,3522	-2,25697	-2,33978	NaN
NaN	NaN	NaN	-1,14965	NaN	0,0602016	-1,52632	NaN	NaN	NaN
NaN	NaN	NaN	-0,283394	NaN	0,645548	-1,18181	NaN	NaN	NaN
NaN	NaN	NaN	NaN	NaN	NaN	NaN	NaN	NaN	NaN
-0,72879	-1,61675	-1,80621	-1,49191	-0,767115	-1,3717	-1,01027	-1,35386	-2,07063	-1,7196
0,175875	0,402262	-0,460287	0,78731	1,87787	-0,376125	-0,334311	0,756636	0,324924	0,277579
-0,793781	-1,17254	0,361687	-0,654062	NaN	-1,87173	-1,26015	0,463446	0,328277	1,26555
NaN	NaN	NaN	-0,364611	-2,84311	0,423462	-1,13076	NaN	NaN	NaN
-1,13216	-1,81587	-1,73957	-1,00016	-1,833	-1,40232	NaN	-0,842838	-1,75032	-3,40029
NaN	NaN	NaN	0,338383	-1,8533	0,503578	NaN	NaN	NaN	NaN
NaN	NaN	NaN	0,845858	NaN	0,972721	-1,5525	NaN	NaN	NaN
0,511536	-0,257143	-0,319714	0,396111	-0,135759	1,43312	-0,148708	0,28133	0,56545	-1,46058
-1,90165	-0,95892	-2,31248	-3,24085	-1,31508	NaN	NaN	-1,78836	-2,18834	-1,32959
-1,06441	NaN	-1,61839	-1,39746	-1,58741	-1,05912	-2,22352	-1,64706	-0,636882	NaN
NaN	NaN	NaN	NaN	NaN	NaN	NaN	NaN	NaN	NaN

Polito et al. Universal ready-to-use immunotherapeutic approach for the treatment of cancer: expanded and activated polyclonal γδ memory T cells.
Phosphorylation sites

NaN	NaN	NaN	NaN	-1,8689	-3,14752	-2,45524	NaN	NaN	NaN
NaN	0,683083	NaN	0,370602	0,10117	NaN	0,841347	NaN	NaN	-0,0765448
NaN	NaN	NaN	-1,96511	NaN	-1,08246	-2,00022	NaN	NaN	NaN
1,03188	1,24526	0,564119	0,997278	NaN	1,0493	1,53028	1,02287	1,6059	0,743342
3,66033	3,77458	4,08974	4,83748	4,38095	4,53608	4,21786	4,21543	4,21158	4,32753
3,80419	1,12763	3,50568	3,51673	2,91408	4,10049	3,43491	3,4208	3,68693	4,22155
4,50321	4,83515	4,25993	4,50238	3,29958	4,77449	4,18039	4,13324	4,42281	4,48257
1,48231	1,6791	2,54202	2,16758	1,06461	1,40941	1,72293	1,43701	1,54991	1,12521
0,711065	2,64279	0,0577755	1,91495	0,555771	3,15182	1,18666	0,289087	0,262179	1,22783
0,557713	0,174002	0,726778	2,67113	0,379772	1,64781	0,958445	1,48948	0,929067	0,901092
0,780279	-0,32593	NaN	NaN	0,837854	NaN	NaN	0,574055	0,252933	0,936418
3,64445	2,2096	4,98474	4,6649	3,30428	4,93596	4,35391	4,70153	4,13041	4,20671
NaN	NaN	NaN	NaN	NaN	NaN	NaN	NaN	NaN	NaN
-0,117582	-0,027523	0,857231	3,53993	-0,218063	-0,769117	1,77473	1,31861	1,23814	1,4514
-0,412838	-0,854502	0,590401	3,39449	-0,444094	2,05141	1,1632	1,30656	2,08279	1,7961
NaN	NaN	0,265795	-1,77634	NaN	NaN	-1,42702	0,964882	1,24741	1,23282
NaN	NaN	NaN	NaN	NaN	NaN	NaN	NaN	NaN	NaN
-0,255911	-2,26584	-0,516636	-0,10553	-0,756844	-4,44096	-1,03026	-0,585999	NaN	-1,38587
2,93422	2,79339	3,20339	3,0569	3,2211	3,211	2,85618	3,18832	2,83407	2,55023
0,596699	-0,929632	-0,40794	0,48797	0,0112991	0,727879	-0,279766	-0,77426	-0,740766	-0,555331
-0,61496	NaN	-1,16508	-0,113703	0,432108	0,441488	0,686251	-2,13659	-1,66721	NaN
NaN	NaN	NaN	-2,48171	NaN	-1,49835	NaN	-1,82263	-1,48808	-2,69505
NaN	-0,695971	-1,83081	-1,16733	-0,824919	-1,76093	-1,14759	-1,6042	-1,73981	NaN
NaN	-0,625011	NaN	NaN	-0,633068	-2,71685	-1,83676	NaN	NaN	NaN
-0,344872	0,104862	-0,396622	-0,233503	0,626228	-0,730986	-0,260389	-0,731804	-0,844514	-0,141788
NaN	NaN	NaN	-1,28832	NaN	0,394125	-2,03974	NaN	NaN	NaN
NaN	-2,05404	-1,25111	-1,71007	-2,37249	-1,34503	-2,51057	-1,9879	-2,35428	-3,0377
NaN	NaN	NaN	-1,82358	NaN	-1,34521	-2,41047	NaN	NaN	NaN
NaN	NaN	NaN	NaN	NaN	NaN	NaN	NaN	NaN	NaN
NaN	NaN	NaN	0,928728	NaN	0,756535	-0,54254	NaN	NaN	NaN
-1,83635	-2,68178	-1,96753	NaN	-1,19865	NaN	-3,17182	-1,68875	-1,80295	-2,06365
NaN	NaN	NaN	NaN	NaN	NaN	NaN	-0,910755	-1,71886	-1,04284
0,292961	0,688602	0,216717	NaN	0,773678	-0,817568	0,638386	-0,191332	0,0402269	NaN
-2,12684	-1,3554	-2,09105	-0,894966	-1,90912	-0,0847683	-1,02325	-1,27201	-1,51808	-1,7624

Polito et al. Universal ready-to-use immunotherapeutic approach for the treatment of cancer: expanded and activated polyclonal γδ memory T cells.
Phosphorylation sites

NaN	NaN	NaN	-2,234	NaN	-1,79342	-2,18806	NaN	NaN	NaN
-1,334	-0,615601	-1,36568	-0,601294	-0,947432	NaN	-0,883652	-0,615273	-1,12376	-0,977626
NaN	NaN	NaN	-1,07537	NaN	-1,01984	-1,44419	NaN	NaN	NaN
NaN	NaN	NaN	-1,62744	NaN	-2,37378	-1,10265	NaN	NaN	NaN
-0,983534	-1,16414	-2,11914	-0,932961	-1,3485	-1,64722	NaN	-2,48735	-2,58547	-2,82728
-1,02204	-1,2752	-1,05414	-1,69713	-0,70545	-0,99247	-1,43727	-0,952816	-0,740251	-0,472947
NaN	NaN	NaN	-1,32755	NaN	-1,26394	-2,30876	NaN	NaN	NaN
NaN	NaN	-0,608597	-0,559435	NaN	-0,370831	-0,541922	-2,31474	-2,26335	-2,52358
NaN	NaN	NaN	-0,956097	-0,484097	-1,36736	-0,545212	NaN	-0,865884	NaN
2,90352	1,86053	1,63973	3,18521	3,05414	3,23002	2,41734	2,98937	2,14003	2,27482
NaN	NaN	NaN	-0,786575	-2,94353	-0,929239	-0,860826	NaN	NaN	NaN
1,35572	0,836037	1,08313	1,59157	1,16388	3,03973	1,20345	0,343098	1,25834	2,31282
NaN	NaN	NaN	1,30427	-1,56975	0,618952	0,472034	NaN	NaN	NaN
NaN	NaN	NaN	NaN	NaN	NaN	NaN	-0,547029	-1,12443	-0,351354
-1,52267	NaN	-2,03421	-0,464863	NaN	-0,614105	-0,974791	NaN	NaN	NaN
NaN	NaN	NaN	NaN	NaN	NaN	NaN	NaN	NaN	NaN
NaN	-0,759031	NaN	NaN	0,24354	-1,31124	NaN	-0,399055	-0,292293	-0,504407
NaN	NaN	NaN	-2,59921	NaN	-0,879515	-2,52131	NaN	NaN	NaN
NaN	NaN	NaN	-0,90206	-2,26208	-1,47424	-0,663193	NaN	NaN	NaN
NaN	NaN	NaN	NaN	NaN	NaN	NaN	NaN	NaN	NaN
-0,0780296	-0,563124	-0,673378	-0,298822	0,108784	0,344307	-0,242302	-0,0620251	-0,663823	-0,826035
1,60265	1,08237	1,29188	0,329851	2,31803	NaN	0,903833	1,98411	1,57924	2,97986
NaN	NaN	NaN	-2,66507	-0,498043	NaN	-2,38807	-2,66428	-3,16758	-1,50574
-0,336758	0,0879021	NaN	0,0232868	0,00221634	-2,11233	-0,0881386	NaN	NaN	NaN
-0,171564	-0,026762	NaN	-0,45314	0,486961	-0,527401	0,154072	0,0850639	0,448316	-0,0652151
0,670551	1,62401	0,44615	1,42268	0,128582	1,9413	1,71587	-0,596746	0,753097	0,149697
NaN	NaN	NaN	-1,05939	NaN	-0,995094	-0,9914	NaN	NaN	NaN
-2,4832	-2,74879	NaN	-1,80753	-1,11295	-2,6227	-1,44753	NaN	-2,52084	-2,24519
2,70375	3,2773	2,29214	2,38851	2,70216	-1,29049	3,20993	3,04757	2,05597	3,06464
0,997408	0,574188	0,330606	1,30583	0,607895	1,9697	1,42635	0,723816	0,439404	0,453761
0,0421429	0,946135	0,583061	-0,743006	0,238079	-0,649908	1,1644	-0,225393	0,412446	0,696248
-0,536394	-0,19594	0,857933	0,754234	1,45573	-0,0688438	0,806633	1,05347	0,974029	1,10561
NaN	-0,937876	NaN	NaN	NaN	NaN	NaN	NaN	NaN	NaN
-0,16057	0,12413	-0,202686	0,697441	0,23366	0,517878	0,7124	-0,51264	-0,422378	-0,285316

Polito et al. Universal ready-to-use immunotherapeutic approach for the treatment of cancer: expanded and activated polyclonal γδ memory T cells.
Phosphorylation sites

-2,6747	NaN	NaN	NaN	-1,72163	-2,09502	NaN	-2,5977	-2,31273	-1,89729
-1,18924	-1,55901	-1,04953	0,236824	-0,814381	-0,153881	1,04276	-0,0379333	0,363614	0,482118
-3,27643	NaN	-0,23605	-0,0995312	-2,07173	0,19486	0,0661087	NaN	NaN	NaN
NaN	NaN	NaN	NaN	NaN	NaN	NaN	NaN	NaN	NaN
NaN	NaN	NaN	-0,556133	NaN	0,377064	-0,397814	NaN	NaN	NaN
2,99212	3,20098	2,69377	2,95172	2,9083	3,14967	3,36978	2,95169	2,62926	3,2324
-0,4687	0,686668	-0,161423	-0,87821	0,558496	NaN	0,345556	-0,439209	0,156056	0,287654
NaN	NaN	NaN	-2,76199	-4,14735	-1,20609	-2,12315	NaN	NaN	NaN
NaN	NaN	NaN	-1,07015	NaN	-1,60054	NaN	NaN	NaN	NaN
-1,52858	-1,86536	-1,7765	NaN	NaN	NaN	NaN	NaN	NaN	NaN
2,34262	3,89998	2,09046	3,36283	3,39315	3,07413	3,93966	1,08002	2,10542	2,6233
4,80942	4,6589	4,50319	5,74981	4,88912	6,42242	5,40454	4,57977	4,28127	5,17027
-1,43817	-1,60628	NaN	-0,747536	-0,968252	1,45833	-0,853378	NaN	NaN	NaN
-0,265215	-0,351421	NaN	0,690168	NaN	1,16562	0,0773144	-0,502569	-1,10622	-0,680532
-1,84779	NaN	-0,251284	-0,209904	-1,43119	-0,26757	0,739691	NaN	NaN	NaN
NaN	-0,0882816	NaN	3,58267	1,01158	4,34982	3,80215	0,343864	-0,0392084	-0,0771246
-0,707531	-0,643753	-0,867115	2,07634	NaN	3,22157	1,24562	NaN	NaN	NaN
-1,28983	-0,729982	-1,93799	-2,47474	-0,151875	NaN	-0,289566	-2,93481	NaN	NaN
-2,34887	-2,1453	-1,74603	-0,205381	-1,23687	0,871035	-0,830307	NaN	NaN	NaN
NaN	NaN	NaN	0,0446701	NaN	-1,1673	-0,052845	NaN	NaN	NaN
NaN	NaN	NaN	NaN	NaN	NaN	NaN	NaN	NaN	NaN
NaN	NaN	NaN	NaN	NaN	NaN	NaN	-0,793455	-1,24778	-0,987512
0,343092	1,25221	-1,79396	-1,40479	0,220079	-0,372568	-0,24143	0,157251	-0,0487013	0,612464
0,63463	NaN	NaN	0,360186	0,637495	1,13479	-1,11383	NaN	NaN	NaN
NaN	NaN	NaN	-1,30599	NaN	-0,183786	-0,164576	NaN	NaN	NaN
-1,80006	-1,0544	-0,883093	-0,535152	-0,443977	-1,1604	0,254807	-1,65466	-1,62934	-1,01331
0,108385	-2,36645	-1,0441	NaN	NaN	NaN	NaN	-0,499146	-0,823783	-1,13762
NaN	NaN	NaN	NaN	NaN	NaN	NaN	1,19179	0,82293	1,43307
NaN	NaN	NaN	0,254776	-0,761223	-0,115986	NaN	NaN	NaN	NaN
0,241196	-0,268433	0,0317822	1,24229	-0,106628	1,67208	1,1589	-0,118769	-0,119412	-0,443345
NaN	-0,937368	NaN	-1,30068	NaN	-0,950226	-1,79328	NaN	NaN	NaN
-1,38818	NaN	-2,01566	-1,52475	-1,56511	-0,83621	-1,10505	-1,83343	-2,61319	-2,4711
NaN	NaN	NaN	-2,43344	NaN	-1,94874	NaN	NaN	NaN	NaN
NaN	NaN	NaN	NaN	NaN	NaN	NaN	NaN	NaN	NaN

Polito et al. Universal ready-to-use immunotherapeutic approach for the treatment of cancer: expanded and activated polyclonal γδ memory T cells.
Phosphorylation sites

-2,22266	-2,20978	-1,95121	-1,66664	NaN	-1,54668	-1,40295	-1,89824	-1,87274	-1,98469
-2,48532	-2,5536	-2,90268	-1,02853	-3,77253	-0,499552	-2,1291	NaN	NaN	NaN
0,396481	0,365852	0,380844	0,946198	0,575634	0,661697	1,14351	0,38637	0,639201	0,147359
2,42069	2,43449	2,00078	2,39328	1,7352	1,33806	2,33634	1,53993	1,63848	1,76612
2,04443	NaN	0,809048	1,94925	NaN	NaN	2,98821	NaN	NaN	NaN
0,487507	1,26568	-1,12163	1,66396	-0,184525	3,02975	1,56966	-0,7796	-0,663304	-1,07415
NaN	NaN	NaN	NaN	-1,49788	0,735239	-0,272863	NaN	NaN	NaN
-0,758577	0,163609	-1,52229	-0,00259399	0,918665	-1,13881	-0,184832	-1,62959	-1,59809	-0,768685
0,89958	NaN	0,657917	1,09896	-0,372667	2,88109	1,12805	NaN	NaN	NaN
-1,88334	-2,04136	-0,81126	-1,81534	-1,10394	0,0238895	-2,01225	NaN	-2,55454	-1,85094
-0,338902	NaN	NaN	NaN	-0,135103	NaN	NaN	1,03849	0,826867	0,836761
-0,990967	0,365128	0,0588951	0,611622	-0,979702	0,273899	0,89208	-1,44036	-0,960412	0,257583
NaN	NaN	-0,628613	-0,469082	NaN	-0,859177	-1,15893	NaN	NaN	NaN
-0,348391	NaN	NaN	0,803936	0,190037	-0,577217	NaN	NaN	NaN	NaN
1,28695	NaN	0,52903	0,0703354	0,254189	NaN	0,422771	1,09551	0,304692	0,463614
NaN	NaN	NaN	NaN	NaN	NaN	NaN	0,469385	-0,150239	-1,17045
-2,87301	-2,26937	NaN	NaN	-2,79211	-2,13294	-2,67753	NaN	NaN	NaN
NaN	-1,80772	-1,03044	-0,465422	-0,719473	NaN	-0,812145	-1,00077	-0,853269	-1,56555
-1,09559	0,571779	-0,857788	-0,320833	-0,218594	NaN	0,0993214	NaN	-1,90441	-0,347289
2,13111	1,6509	2,19498	1,31827	2,26707	-2,01826	1,03645	1,79459	1,70037	2,51417
NaN	-1,23442	NaN	0,0151787	-0,0158958	-1,04288	-0,307928	NaN	NaN	NaN
NaN	NaN	-0,389051	-0,608984	NaN	-0,309978	-0,224203	NaN	NaN	NaN
-0,169554	-1,42276	2,55769	0,741756	NaN	1,58847	0,935026	-0,449322	0,920316	-0,670817
NaN	0,225809	0,276701	0,0870628	0,363379	0,639944	NaN	0,671875	NaN	0,678721
NaN	NaN	NaN	-1,02206	NaN	-0,662903	-1,09395	NaN	NaN	NaN
-1,09966	-2,13077	-1,59087	NaN	NaN	NaN	NaN	NaN	NaN	NaN
NaN	NaN	NaN	0,346746	1,66386	NaN	-0,0540047	NaN	NaN	NaN
NaN	NaN	NaN	-0,655394	NaN	0,600023	NaN	NaN	NaN	NaN
-0,353971	-0,430359	-0,786442	0,650669	-0,774925	-0,206699	0,485376	-1,00522	-1,03439	-0,523908
0,614565	0,567476	0,890198	3,65425	1,40166	3,61072	2,66442	0,0151348	-0,378179	0,710202
NaN	-1,6889	NaN	-1,10452	NaN	0,190142	-1,36994	-2,07318	-1,98079	-2,63791
-1,05682	NaN	-1,05612	1,18555	-0,804266	0,899771	1,56128	NaN	-1,33315	-1,17599
NaN	NaN	NaN	1,42337	-0,706934	0,784143	NaN	-0,0701447	0,986089	NaN
-0,449005	-1,15034	-1,9596	0,131353	NaN	1,54277	0,653522	NaN	NaN	NaN

Polito et al. Universal ready-to-use immunotherapeutic approach for the treatment of cancer: expanded and activated polyclonal γδ memory T cells.
Phosphorylation sites

NaN	NaN	NaN	NaN	NaN	NaN	NaN	-0,835609	-1,08788	-1,52246
-1,00842	NaN	-1,25702	1,92926	-0,862991	2,50917	1,65345	-0,94689	-1,04125	-1,16129
-2,51155	-2,99927	0,00740051	1,29306	-1,16332	1,51658	1,08646	-1,0646	-1,16373	-1,10595
0,318279	-0,66358	1,25016	2,32195	1,53734	2,12798	2,07414	0,571238	0,416186	0,348472
1,94614	2,28262	2,31575	3,48086	1,62954	3,48749	3,15045	2,79326	2,93146	2,40071
NaN	NaN	NaN	-0,609938	NaN	NaN	-0,702366	NaN	NaN	NaN
-2,01815	NaN	-2,54939	-2,43712	-0,975615	-2,0798	-1,66132	-1,99207	-2,57358	-0,829946
1,86213	1,20461	2,34224	2,98721	0,89357	3,47935	2,54804	2,39992	2,37082	2,26134
0,0337734	NaN	0,783661	NaN	-0,0822544	NaN	NaN	0,575863	0,197406	1,0568
NaN	NaN	NaN	NaN	NaN	NaN	NaN	-1,20119	-3,0902	-1,01861
NaN	NaN	NaN	NaN	NaN	NaN	NaN	NaN	NaN	NaN
-1,77977	-1,43442	-1,56273	0,0657406	NaN	0,551136	-0,574097	-1,77345	-1,51931	-1,66211
-0,800102	-0,321095	-1,214	NaN	NaN	NaN	NaN	NaN	-1,14107	-1,68068
NaN	NaN	NaN	-2,74661	-3,11728	NaN	-2,31397	-2,50536	-2,79667	-2,97544
-1,11282	0,646729	-2,09694	-1,18211	0,323717	-0,132574	-1,43649	-0,619404	-0,389499	-0,261353
NaN	NaN	NaN	-1,77842	-3,32056	-2,47501	-1,5046	NaN	NaN	NaN
NaN	NaN	NaN	NaN	NaN	NaN	NaN	NaN	NaN	NaN
-1,47871	NaN	-1,71933	0,211552	-1,80114	2,04695	-0,315195	-1,56795	-1,09141	-1,58037
NaN	-0,691635	NaN	NaN	-0,0546455	NaN	NaN	NaN	NaN	NaN
NaN	-2,42698	-1,06253	-1,04232	NaN	0,0569801	-1,88596	NaN	NaN	NaN
-2,72305	NaN	-0,908451	-3,4808	-3,2928	-1,52255	-3,57362	NaN	NaN	NaN
1,48438	0,45549	0,206007	-0,213245	0,929125	-0,912609	0,324795	-0,500557	-0,394666	0,000218391
-1,40871	-0,80764	NaN	-0,636496	-0,703505	-1,53466	-0,672958	-1,15192	NaN	NaN
NaN	NaN	NaN	NaN	NaN	NaN	NaN	NaN	NaN	NaN
NaN	NaN	NaN	-2,42128	NaN	-3,00921	-2,9475	NaN	NaN	NaN
NaN	NaN	NaN	NaN	NaN	-0,879824	-0,32762	NaN	NaN	NaN
NaN	NaN	NaN	-1,49346	NaN	0,278648	-1,28629	NaN	NaN	NaN
-2,2427	-1,99622	-2,89391	-2,42118	NaN	NaN	-1,56965	NaN	NaN	NaN
NaN	NaN	NaN	NaN	NaN	NaN	NaN	NaN	NaN	NaN
-1,36098	-1,03822	NaN	-0,615786	-1,12177	-1,39209	NaN	-1,48044	-1,73543	-1,17324
-0,620028	-0,19129	-0,630711	-1,15124	-0,134886	NaN	-0,455481	-1,21879	-1,26685	-0,967629
NaN	-0,404499	NaN	-1,15803	2,74529	-1,66197	-2,07546	0,664688	NaN	1,27629
-2,46423	-2,57201	-2,5724	-2,30799	-0,944365	-2,29663	-1,32597	-1,89453	-1,77419	-1,88241
NaN	NaN	NaN	NaN	NaN	NaN	NaN	NaN	NaN	NaN

Polito et al. Universal ready-to-use immunotherapeutic approach for the treatment of cancer: expanded and activated polyclonal γδ memory T cells.
Phosphorylation sites

NaN	NaN	NaN	NaN	NaN	NaN	NaN	NaN	NaN	NaN
0,40139	0,863438	-0,0433769	0,0887394	1,27579	0,401403	1,70988	1,14879	-0,0745134	1,06516
-2,24969	-1,39865	-2,35003	-1,53889	NaN	-0,946526	-1,61623	NaN	NaN	NaN
-2,24969	-1,39865	-2,35003	-1,53889	NaN	NaN	-1,61623	NaN	NaN	NaN
NaN	NaN	NaN	NaN	NaN	NaN	NaN	-3,11594	-1,23141	-0,839974
NaN	-0,805405	-0,114958	-0,235298	-0,413929	NaN	-0,454746	-2,16842	-0,20995	-2,30733
NaN	NaN	NaN	-0,861147	-1,16352	NaN	-0,732458	NaN	NaN	-1,88733
NaN	NaN	NaN	NaN	NaN	NaN	NaN	NaN	NaN	NaN
NaN	NaN	NaN	-1,91309	-1,56789	-1,86111	-1,96394	-1,84817	NaN	-2,32573
0,372871	NaN	-0,354549	0,947723	0,930244	0,536669	0,642256	-0,105103	-0,700582	-0,509429
0,496632	-0,642975	-1,88889	-1,37917	-0,350094	-0,650379	-0,0332527	-1,15564	-1,20971	-0,291398
2,54542	2,84913	2,864	2,73341	2,99947	-0,996098	2,73735	2,75567	2,57226	2,52549
2,94999	2,88738	3,15171	3,3573	3,23639	1,27201	3,14348	3,76862	3,35618	3,41716
NaN	NaN	NaN	-1,54049	-1,14743	-2,65313	-2,18034	NaN	NaN	NaN
-0,940104	-2,49114	NaN	0,160772	NaN	0,804596	-0,327368	NaN	NaN	NaN
NaN	NaN	NaN	NaN	NaN	NaN	NaN	NaN	NaN	NaN
NaN	NaN	NaN	-1,98382	-3,13301	-2,17086	-2,27354	NaN	NaN	NaN
-1,07283	-0,667879	-1,12903	-0,372585	NaN	0,924353	-0,696739	-1,50983	-1,78454	-2,21909
1,3986	0,437542	1,24722	-0,00237274	1,46544	-0,0214901	0,298798	0,431141	0,749686	0,414821
-1,78483	-0,916489	-0,760141	-2,03391	0,0868912	-2,10731	-0,58217	-2,33046	-2,32933	-0,404645
1,00732	1,26413	-0,235327	NaN	1,13478	NaN	-1,65265	NaN	NaN	0,100844
-0,107857	-2,42347	-0,202394	NaN	-1,43511	NaN	NaN	NaN	NaN	NaN
-1,3552	0,696507	-0,717518	NaN	-0,284941	NaN	-0,511497	NaN	NaN	NaN
NaN	NaN	NaN	NaN	NaN	NaN	NaN	NaN	NaN	NaN
2,01723	1,44592	1,26954	2,00577	2,06012	2,36304	NaN	0,941694	0,629556	1,39606
0,0698395	0,0481205	0,540792	0,261862	1,83851	0,156992	1,56006	0,0468941	0,602527	0,363889
NaN	-2,26435	NaN	-0,0611668	-1,90934	-0,961407	-1,74484	-2,34179	NaN	-2,6305
2,02712	1,99635	0,0596886	3,51487	2,07672	3,73643	2,68371	1,46917	0,574843	1,10997
NaN	NaN	NaN	-1,05251	-4,01938	NaN	-1,22781	NaN	NaN	NaN
NaN	NaN	NaN	NaN	NaN	NaN	NaN	NaN	NaN	NaN
-0,693314	NaN	-0,556076	NaN	-0,243587	0,406023	1,24203	-0,542372	0,102181	-0,333142
0,116087	NaN	NaN	-1,50301	NaN	NaN	NaN	NaN	NaN	NaN
1,80693	-0,147116	1,48326	2,89295	1,77055	3,32707	3,56432	1,35602	1,1914	1,24255
NaN	NaN	NaN	NaN	NaN	NaN	NaN	NaN	NaN	NaN

Polito et al. Universal ready-to-use immunotherapeutic approach for the treatment of cancer: expanded and activated polyclonal γδ memory T cells.
Phosphorylation sites

NaN	NaN	NaN	NaN	NaN	NaN	NaN	NaN	NaN	NaN
NaN	NaN	NaN	NaN	NaN	NaN	NaN	NaN	NaN	NaN
-2,05774	-1,10618	-1,36661	-0,767426	NaN	-1,8504	-1,29279	-2,12981	-1,74481	-2,00196
NaN	NaN	NaN	NaN	NaN	NaN	NaN	NaN	NaN	NaN
NaN	NaN	NaN	-2,37573	NaN	-2,12518	-2,62723	NaN	NaN	NaN
NaN	-1,22514	-1,78407	NaN	-2,37533	-2,178	-1,47933	-2,50494	NaN	NaN
-0,27593	-1,25655	0,593494	-0,16523	0,623795	-3,22429	-0,00964165	0,125937	-0,458671	-0,492961
NaN	NaN	NaN	NaN	NaN	NaN	NaN	NaN	NaN	NaN
NaN	NaN	NaN	0,340343	NaN	-0,329016	-0,693773	NaN	NaN	NaN
NaN	NaN	NaN	-0,746025	-0,718723	-1,7315	-1,24699	NaN	NaN	NaN
NaN	-3,11044	NaN	0,630625	NaN	1,04466	-0,965334	NaN	NaN	NaN
NaN	NaN	NaN	NaN	NaN	NaN	NaN	NaN	NaN	NaN
NaN	NaN	-0,858019	-1,1086	-2,34549	0,0363293	-0,396637	NaN	NaN	NaN
-0,217306	-1,29536	-0,215994	-1,36712	0,431686	NaN	-0,92812	-0,763378	-0,590964	-0,525559
-0,675592	-0,473942	-1,3398	-0,704124	0,236439	-0,791613	-0,560158	0,211573	-0,378047	0,106845
NaN	NaN	NaN	-0,74729	-2,35153	-0,484518	-0,759176	NaN	NaN	NaN
1,71032	1,94767	1,67202	2,03311	1,82789	0,613792	2,72523	2,1674	2,97387	2,30337
-0,355169	-0,159414	NaN	0,473612	NaN	-0,0263634	0,53673	NaN	NaN	NaN
-1,01381	-1,90128	-2,75323	-1,17728	-1,03444	-1,23302	-1,97146	-2,00883	-1,6574	-2,14414
NaN	-0,360292	-1,03695	NaN	-0,440577	NaN	-0,351942	-1,53744	-1,50457	-1,31062
-0,806881	-2,47433	-2,06954	NaN	NaN	NaN	NaN	-0,830544	NaN	-1,7218
-1,11282	-1,73882	NaN	NaN	0,279076	NaN	-0,504475	NaN	NaN	NaN
0,867887	1,72591	1,88671	1,55744	NaN	NaN	1,16898	1,42471	1,03885	1,0425
NaN	NaN	NaN	NaN	-0,300789	0,215031	-0,46603	NaN	NaN	NaN
0,474747	0,386497	0,607195	1,38644	1,00508	1,4786	0,529703	1,17742	0,985364	0,756715
NaN	NaN	-1,1557	NaN	-3,00634	1,1186	0,228802	-0,212244	NaN	-0,542272
-1,21042	NaN	-2,18066	-0,757694	-1,3765	-0,623655	0,0210533	-0,9492	-0,756894	-1,22955
-1,71495	NaN	-0,596336	-0,45397	-1,52014	-0,899763	-1,27338	-1,28071	-1,14383	-1,04072
NaN	NaN	NaN	0,980606	NaN	NaN	0,604584	NaN	0,693727	1,45425
NaN	NaN	NaN	2,96569	NaN	3,07952	2,1632	-0,554483	-1,3	-1,10408
NaN	NaN	NaN	-2,29855	-3,2006	-2,23085	-2,67939	NaN	NaN	NaN
NaN	0,0449219	0,19175	1,65014	-0,523647	1,94675	0,315739	-0,279919	-0,436353	-0,708234
NaN	NaN	NaN	-0,502441	-2,65674	-0,255075	0,0242214	NaN	NaN	NaN
NaN	NaN	NaN	-0,252407	NaN	0,337873	-0,834406	NaN	NaN	NaN

Polito et al. Universal ready-to-use immunotherapeutic approach for the treatment of cancer: expanded and activated polyclonal γδ memory T cells.
Phosphorylation sites

-1,16938	2,51421	NaN	0,574207	NaN	2,11202	0,158323	NaN	0,916627	NaN
0,316488	1,83097	NaN	0,773396	NaN	0,355965	-0,190737	-0,731306	0,194648	-0,544747
NaN	-1,09975	NaN	-0,267796	NaN	0,600418	-0,688541	-0,808752	-0,75492	-0,726079
-0,525656	-0,664171	NaN	NaN	NaN	0,347136	-0,61161	NaN	NaN	NaN
-0,832409	0,339132	1,10103	0,0985088	1,42641	0,749922	0,890636	0,600304	0,471072	0,968202
NaN	NaN	NaN	0,611862	NaN	NaN	-1,20409	NaN	-2,36017	NaN
NaN	-1,35947	NaN	0,264648	0,445805	0,485548	0,496412	-0,5348	0,0827723	-0,611831
NaN	NaN	NaN	-1,36248	NaN	NaN	NaN	NaN	NaN	NaN
NaN	NaN	NaN	2,36301	NaN	2,76041	1,70871	1,04007	0,63366	0,589984
NaN	NaN	NaN	-0,0573559	-1,64778	-0,565306	-1,28815	NaN	NaN	NaN
NaN	NaN	NaN	1,8866	0,989126	0,25444	1,61836	NaN	-1,22143	1,49308
1,71983	0,788307	0,829111	1,16685	0,952335	NaN	2,13717	1,06853	1,58636	0,920865
-0,795912	-0,944336	0,699495	1,10144	0,396414	1,48666	1,15269	0,245296	0,285605	-0,0657682
-1,01472	-0,344877	-1,13051	-1,09056	-0,662243	-1,54361	-0,829554	-1,06638	-1,47914	-0,365594
0,252558	0,0956631	0,422354	0,107271	0,0493031	-1,23742	0,445187	0,667658	0,652049	0,401851
3,12042	3,59955	3,19406	2,94822	3,12259	-0,79837	3,37153	2,966	2,97406	2,44875
0,527357	-0,0116215	0,476723	0,767576	1,33458	NaN	0,875124	0,182724	0,193572	0,859832
-1,90725	-1,60107	NaN	-2,43434	-1,96165	-4,37097	-2,02429	NaN	NaN	NaN
NaN	NaN	NaN	-2,49284	-2,78755	-1,43889	NaN	NaN	NaN	NaN
NaN	-2,42021	-2,42136	-2,82338	-2,2945	-2,71379	-2,27939	-2,95616	NaN	-2,08833
NaN	NaN	-0,16729	-0,701283	NaN	0,404846	0,566053	NaN	NaN	NaN
-0,418196	-0,536963	NaN	1,09741	NaN	1,71066	0,84968	NaN	NaN	-1,29793
NaN	NaN	NaN	-1,11543	NaN	-1,25977	-1,56455	NaN	NaN	NaN
-1,99421	-2,01972	-1,14779	NaN	NaN	NaN	NaN	-0,245655	-0,392753	-0,835217
2,94254	3,0263	2,14285	4,24911	2,77176	3,44014	3,15291	3,06508	3,12396	4,3519
-2,1283	NaN	-0,171223	1,49098	NaN	2,63382	1,32795	-1,23461	-1,32193	-0,401761
-0,532032	0,0476379	-0,672464	-0,0285511	NaN	0,840977	-0,0915871	-0,243763	-0,218492	0,242646
-2,82498	-0,837921	-4,52276	-0,701462	-1,17496	-0,956491	0,410971	-3,38049	-3,09405	-1,20501
0,0759296	-0,153858	-0,133207	-0,522448	-0,241367	0,0643349	-0,757324	-0,27487	-0,427333	-0,998521
-0,48354	0,909615	NaN	1,51729	NaN	2,80424	NaN	1,22962	1,23239	1,1138
-0,484259	NaN	-0,763376	-0,0529823	-0,606289	-0,590546	-0,922432	-0,410713	-0,245175	-0,697141
NaN	NaN	NaN	-0,192322	NaN	0,711067	0,207691	NaN	NaN	NaN
NaN	NaN	NaN	1,99119	NaN	2,84395	2,28719	NaN	NaN	NaN
NaN	NaN	NaN	NaN	-1,57194	-3,25702	-2,30832	-2,28453	-1,95671	-1,52397

Polito et al. Universal ready-to-use immunotherapeutic approach for the treatment of cancer: expanded and activated polyclonal γδ memory T cells.
Phosphorylation sites

NaN	NaN	NaN	-0,719721	NaN	-1,41853	-1,51574	NaN	NaN	NaN
NaN	NaN	NaN	-1,61005	-2,44235	-1,35575	NaN	-2,31052	-3,00497	-2,68417
NaN	NaN	NaN	0,123005	-1,8633	1,2096	-0,109653	0,51745	0,373874	-1,17123
NaN	NaN	NaN	-0,889915	-1,93216	-1,9439	-0,211029	NaN	NaN	NaN
NaN	NaN	NaN	NaN	NaN	NaN	NaN	-2,76916	NaN	NaN
NaN	NaN	-1,79447	-0,734694	NaN	0,708282	0,0558681	NaN	NaN	NaN
-1,4919	-0,477842	-0,696585	0,892351	NaN	0,71443	0,675657	0,643448	1,14077	0,00235081
-0,166094	1,28441	0,0987129	2,42583	-0,25379	2,07535	1,30462	1,4734	1,59989	1,52589
-1,31738	-1,51783	-1,62799	-1,19564	-1,37909	NaN	NaN	NaN	-0,346778	-0,509006
NaN	NaN	NaN	NaN	NaN	NaN	NaN	-1,75751	-2,25755	-1,9291
-1,09321	-0,787252	-1,08358	-0,108421	-1,41026	-1,05615	-0,615507	-0,788233	-1,08858	-0,873681
3,4281	3,56482	3,41427	2,81024	3,42807	1,5676	3,23804	3,2469	3,06761	2,97869
-1,87553	NaN	-3,60273	-2,67619	NaN	NaN	-1,71695	-2,79933	-2,50167	-2,122
NaN	NaN	NaN	NaN	NaN	NaN	NaN	NaN	NaN	NaN
4,61918	4,171	3,98092	4,39604	4,40007	4,95122	4,09522	4,09381	4,19834	3,62005
NaN	NaN	NaN	0,413605	NaN	0,954842	-0,638422	NaN	NaN	NaN
-2,00662	-0,0668449	NaN	-1,47081	-2,46965	NaN	-0,495531	-2,43408	-1,062	-1,5766
2,47871	2,66146	2,20564	3,01928	2,20996	2,89871	2,80465	2,43595	1,96712	2,54355
4,82714	3,7095	6,22825	5,32435	5,63092	5,13108	5,68727	4,99921	4,81301	4,6954
NaN	NaN	NaN	-3,14454	0,0107632	NaN	-1,8666	NaN	NaN	NaN
2,08823	2,4133	1,92695	2,37224	0,663324	0,81934	2,08013	1,82137	1,87698	1,38353
-2,30046	0,399666	NaN	0,972572	-2,68698	0,344402	1,20393	NaN	NaN	NaN
1,45529	0,88789	0,851017	1,6103	0,198313	1,57968	1,71431	1,43904	1,0001	0,792001
1,6453	1,60621	-0,917311	1,78736	NaN	-0,430401	1,69007	1,46276	1,75996	1,30398
NaN	-2,46932	-2,06412	-1,58584	-2,23359	-1,19636	-2,03799	-2,96661	-2,24428	-2,93297
0,279287	NaN	NaN	0,0219288	NaN	NaN	NaN	-2,34015	-2,82013	-4,39835
1,66514	-0,784674	-0,209631	-0,0442562	0,858826	NaN	NaN	0,406513	0,286799	0,358851
-3,14351	-1,68013	-1,80858	-2,84053	-0,0701733	NaN	-2,39722	NaN	NaN	NaN
-1,00626	-1,00014	-1,4331	-0,875746	-1,9797	0,094614	NaN	NaN	NaN	NaN
1,9854	2,27505	2,14065	1,92984	3,48258	1,06754	NaN	2,73937	NaN	NaN
NaN	NaN	NaN	NaN	NaN	NaN	NaN	-0,06847	0,135964	0,231725
-0,953442	-1,86184	1,17367	1,99852	-0,610622	1,46073	1,47644	0,103615	0,238212	1,25231
-1,82959	-0,145357	-1,28868	0,155287	1,08727	NaN	1,08245	NaN	NaN	0,127475
-0,711216	-1,32289	-1,76161	-0,347109	-1,00636	-2,13489	-0,651737	0,445919	0,122445	0,38186

Polito et al. Universal ready-to-use immunotherapeutic approach for the treatment of cancer: expanded and activated polyclonal γδ memory T cells.
Phosphorylation sites

NaN	NaN	NaN	-2,74144	-1,35773	-2,57793	NaN	NaN	NaN	NaN
-0,953224	-0,396151	-0,782938	0,400015	0,488503	0,122292	0,886047	-0,642815	-0,98907	-0,638127
NaN	-0,0998039	-0,618847	0,96242	0,165985	1,22342	1,22798	0,179205	0,0131483	-0,0904036
NaN	NaN	-1,52552	-0,771938	-0,878067	-0,317366	-0,723759	NaN	NaN	NaN
NaN	NaN	NaN	0,972666	1,17374	NaN	1,12262	-0,932156	-0,57215	NaN
-3,02402	-3,66155	-2,27466	-0,891994	-3,03388	-0,531223	-2,28929	-3,07558	-2,74626	NaN
2,80108	2,82954	3,09213	3,57601	3,32187	3,91672	4,03692	2,84152	2,54735	2,68826
-2,11383	-3,28654	-2,84443	-2,38252	-2,24177	-1,77883	NaN	NaN	NaN	NaN
-1,02145	0,180065	NaN	1,26381	-0,123659	-0,0269737	1,69863	NaN	-1,17888	-0,269601
-2,89035	NaN	-2,41626	NaN	-2,73438	-1,25762	-1,65603	-1,44402	-1,92342	-1,75348
-2,89035	NaN	-2,41626	NaN	-2,73438	-1,25762	-1,65603	-1,44402	-1,92342	-1,75348
NaN	NaN	NaN	-0,357128	-0,322134	-0,584244	-0,190737	NaN	NaN	NaN
NaN	NaN	NaN	-2,22856	NaN	-1,8429	-2,43383	NaN	-2,37201	-2,43827
-1,40003	-1,38997	-1,35485	-1,09801	-2,10485	-0,453796	-0,688181	-1,37279	-1,23228	-1,19001
-1,09359	-1,26098	-1,67561	-2,00094	NaN	-0,811497	-1,66983	-0,868702	-1,22958	-1,14668
0,522984	-0,310547	-0,838594	-0,740118	0,159559	0,427567	-1,28004	-0,548597	-1,14324	-1,43535
-2,64452	-1,98804	NaN	-1,02359	NaN	-0,558672	-0,975317	NaN	NaN	NaN
-2,10641	-1,74424	-2,48722	-0,344515	-1,16651	-1,35628	-1,16488	-1,8321	-1,68906	-1,87869
-0,390898	0,017004	-0,333029	0,542753	0,785784	0,970766	0,376097	NaN	NaN	NaN
NaN	NaN	NaN	NaN	NaN	NaN	NaN	-1,43671	-0,479222	-1,86626
2,41179	2,43757	2,80932	2,50089	2,95448	2,43421	2,39168	2,31033	2,39481	2,24182
NaN	1,1257	1,41041	1,84125	1,69502	2,53809	NaN	0,939224	NaN	0,592055
1,67013	NaN	0,764074	0,445541	-0,148972	1,01674	0,531591	NaN	NaN	0,363551
NaN	NaN	1,90787	2,21966	NaN	0,789076	2,44982	NaN	NaN	NaN
-2,47201	NaN	2,25696	1,52255	NaN	2,49123	1,9908	NaN	NaN	NaN
-1,36776	-1,21585	-1,08219	-0,426311	-0,731413	-1,80807	-0,201206	-1,14231	-1,42147	-0,733195
0,684368	1,43513	0,90159	2,06518	0,453756	1,76202	2,19127	1,15452	0,814559	1,87674
NaN	NaN	NaN	NaN	NaN	NaN	NaN	NaN	NaN	NaN
2,037	1,83878	3,59707	2,17248	NaN	2,35031	2,54674	3,55018	3,1635	3,53124
-2,37029	-1,40141	NaN	-1,9576	NaN	-1,93892	-1,38375	NaN	NaN	NaN
-2,14815	NaN	-1,41349	-1,82703	-1,34836	-2,0839	-1,77871	-1,3237	-1,55213	-1,42662
NaN	1,21229	NaN	1,37493	0,0105934	1,57341	-0,126558	-0,556683	-0,264285	NaN
NaN	NaN	NaN	-2,16733	NaN	NaN	-1,08373	-2,07025	-2,41271	-1,51378
NaN	-1,37361	0,272882	NaN	NaN	NaN	NaN	NaN	NaN	NaN

Polito et al. Universal ready-to-use immunotherapeutic approach for the treatment of cancer: expanded and activated polyclonal γδ memory T cells.
Phosphorylation sites

-1,51563	-1,4788	-1,93054	-2,57436	-1,066	NaN	-1,68584	-1,59761	-1,5001	-1,69792
NaN	NaN	NaN	-0,669008	0,318073	-1,03682	-0,826498	NaN	NaN	NaN
NaN	NaN	NaN	-0,206102	NaN	0,0898933	-0,190073	-1,67722	-0,460334	-2,14861
-0,577105	-1,4163	-1,95064	-1,46568	-0,199987	NaN	-0,209219	-0,634775	-0,802773	-1,64077
-0,509329	-0,0817699	0,328817	1,49646	0,988983	-0,765589	0,0873814	0,708069	0,509498	0,313109
NaN	NaN	NaN	-0,732492	NaN	NaN	NaN	NaN	NaN	NaN
NaN	NaN	NaN	1,28877	NaN	-0,764835	1,20635	NaN	NaN	NaN
3,60551	4,14869	3,83386	3,61134	4,26546	0,279736	4,00052	3,80539	3,71893	3,62908
-1,57232	NaN	NaN	NaN	NaN	NaN	NaN	-1,74458	-2,16221	-1,57804
NaN	-0,924715	NaN	NaN	NaN	NaN	NaN	-1,49169	-1,55153	-1,74361
NaN	NaN	NaN	NaN	NaN	NaN	NaN	-2,76826	-2,64094	-2,60013
NaN	NaN	NaN	-2,311	-1,57881	-1,78122	-1,21842	NaN	NaN	NaN
-1,27853	-1,01062	-1,09245	-0,692997	-0,413893	1,16994	-0,722799	-1,15647	-1,39507	-1,2968
-2,02504	-1,409	-1,06954	NaN	NaN	NaN	NaN	-1,12278	NaN	NaN
NaN	NaN	NaN	0,108192	-1,55923	-0,303841	-0,373262	NaN	NaN	NaN
NaN	NaN	NaN	-1,48958	-2,43651	-0,596621	NaN	NaN	NaN	NaN
0,874111	1,79106	1,11254	-0,0169735	1,01918	0,0523643	1,01836	1,00496	NaN	NaN
-0,577871	-1,41809	-0,393806	-0,911709	-1,56091	-0,603596	-0,607689	-1,00632	NaN	-0,0499773
NaN	NaN	NaN	-3,76501	-1,68177	NaN	-3,01782	NaN	NaN	NaN
-2,63673	-2,46441	-1,71508	-2,58292	0,320374	NaN	-0,877275	-1,32964	-1,40971	-0,352935
NaN	-1,35847	NaN	1,32914	-1,013	-2,41985	0,702959	-0,957071	-1,34842	0,00541782
-1,44617	-0,494097	-0,678627	0,758804	-0,286362	0,407106	0,439711	-0,229744	-0,250411	0,407813
NaN	NaN	-1,21927	-1,92184	NaN	-1,36823	-0,136276	NaN	NaN	NaN
1,84197	-0,140781	NaN	2,06087	NaN	2,21689	0,810844	1,26896	NaN	0,916615
-1,12474	-0,372463	-1,95622	-0,561142	-0,941359	NaN	-0,802406	-0,519917	-2,65275	-1,70359
NaN	-1,22441	NaN	NaN	NaN	NaN	NaN	-0,0469494	-0,0282907	-0,751508
NaN	NaN	NaN	NaN	NaN	NaN	NaN	NaN	NaN	NaN
NaN	NaN	NaN	-1,0065	NaN	-0,727549	-1,11921	NaN	NaN	NaN
-1,50895	-2,05417	NaN	-1,03696	NaN	-1,08614	-1,26734	-2,94348	-1,66051	-2,0604
-1,66158	-0,1462	-0,484461	NaN	NaN	NaN	NaN	0,17691	-0,299012	-0,992116
-0,356541	-0,112951	0,0190144	-0,375944	-0,191618	-0,513735	-0,164526	0,300331	0,396689	-0,380574
2,25603	1,29968	1,52826	2,13007	1,58408	2,06785	1,41156	1,92537	2,06369	1,96103
0,691711	1,18151	1,22481	0,544392	1,33863	-1,26081	0,757381	1,56172	1,44663	1,2087
-2,92813	NaN	-2,07034	-3,11206	-2,22121	NaN	NaN	-1,77328	-2,42499	-1,46602

Polito et al. Universal ready-to-use immunotherapeutic approach for the treatment of cancer: expanded and activated polyclonal γδ memory T cells.
Phosphorylation sites

0,139032	-0,386263	-0,460375	-0,204386	-0,88945	-0,162975	-0,531225	-0,0573444	-0,103923	-0,385182
NaN	-0,196869	NaN	0,019083	-1,42951	-0,00684547	-0,590593	NaN	-0,477708	NaN
-0,506916	-0,534254	0,313377	-0,512817	0,897329	-1,17841	-1,7525	0,243675	-0,164075	0,0842295
0,988773	1,72671	-0,501373	0,742744	0,90251	0,965191	0,290545	0,672361	-0,514441	-1,09234
3,45065	3,49081	2,85729	1,71737	3,68398	2,35383	3,62198	3,57988	3,97671	3,92077
NaN	NaN	NaN	-1,01982	NaN	-1,32688	NaN	NaN	NaN	NaN
NaN	NaN	NaN	-2,48109	-4,24549	-2,11471	-3,17488	NaN	NaN	NaN
NaN	NaN	NaN	0,188251	-0,617891	0,636711	1,49234	NaN	NaN	NaN
-1,50489	NaN	NaN	NaN	-1,59865	-2,73573	NaN	-1,26318	-1,61357	-2,15218
-0,804773	1,43668	-0,455076	-1,587	0,113995	-0,901396	-0,638771	-1,01456	-1,32964	-0,707896
-0,10841	-1,05579	-1,46224	0,11376	NaN	0,180763	0,127253	-1,44009	0,88577	NaN
-1,71005	0,457874	-1,38936	-1,60529	-0,706007	0,833599	-1,31937	-2,49366	-2,60381	-2,28434
NaN	NaN	-1,38936	NaN	-0,706007	NaN	NaN	-2,49366	-2,60381	-2,28434
0,439251	1,06095	1,07027	-0,141314	NaN	0,855686	0,451687	-0,312784	0,793889	-0,330167
2,96456	2,95398	2,2684	3,27807	2,73597	3,73948	3,13201	2,50019	2,54606	NaN
NaN	NaN	NaN	NaN	NaN	NaN	NaN	NaN	NaN	NaN
NaN	-2,04579	NaN	-0,860079	-1,6158	-0,193073	-0,778061	NaN	NaN	NaN
NaN	NaN	NaN	-2,9057	NaN	NaN	-2,2399	NaN	NaN	NaN
NaN	NaN	NaN	NaN	NaN	NaN	NaN	NaN	NaN	NaN
1,78838	0,458328	1,26276	1,8709	2,18892	0,747355	1,77074	NaN	NaN	NaN
2,47691	2,59597	1,79121	2,35786	2,40316	3,76342	1,73318	1,32519	1,50791	1,38582
NaN	NaN	NaN	-0,257496	NaN	-0,54221	-1,70575	NaN	NaN	NaN
NaN	NaN	NaN	NaN	NaN	NaN	NaN	NaN	NaN	NaN
-1,02708	-1,44371	-0,884501	0,199917	-2,3308	0,437334	0,149202	-0,137442	NaN	-0,582223
NaN	NaN	NaN	NaN	NaN	NaN	NaN	NaN	NaN	NaN
-0,68561	-2,90046	-0,230593	0,405022	0,669739	-0,699371	1,0015	-0,338415	-0,381488	0,886518
NaN	-0,103649	0,495878	0,872408	0,79558	NaN	0,968645	0,380806	-0,000144005	-3,43993
0,0553284	-2,39894	-0,332067	1,42255	0,689108	-0,345684	1,23597	NaN	NaN	NaN
-0,565201	NaN	0,253685	0,262276	0,903454	0,112461	0,514015	-0,0995884	NaN	0,67427
0,557865	-0,17247	1,53946	1,25905	0,687254	NaN	2,02607	NaN	1,09004	NaN
NaN	NaN	NaN	NaN	NaN	NaN	NaN	NaN	NaN	NaN
0,0155182	-0,357716	-0,24263	0,191248	-1,92049	1,42333	0,154533	-0,204405	NaN	-0,140977
0,63822	1,13942	0,731892	2,04238	NaN	1,6697	1,74683	1,11614	1,11676	1,42711
0,2869	-1,25569	2,47665	1,74192	1,54992	2,07376	2,52131	1,54284	1,25946	0,887635

Polito et al. Universal ready-to-use immunotherapeutic approach for the treatment of cancer: expanded and activated polyclonal γδ memory T cells.
Phosphorylation sites

3,67695	3,74153	3,95914	3,97072	4,12538	4,89478	4,01442	4,38319	4,0454	3,80329
0,592783	-0,821476	-0,6912	-0,866835	0,855515	-1,14965	0,248217	1,05371	0,371449	0,504615
0,600325	NaN	NaN	0,0960331	-2,23185	NaN	0,700348	NaN	0,329011	NaN
-0,543047	-1,88444	0,640211	0,0447235	0,154997	-0,28038	0,652245	0,393927	-0,0907793	NaN
-0,221724	-2,28941	0,775883	0,128212	0,219028	1,20529	1,23274	0,281616	0,543668	-0,139688
NaN	NaN	NaN	NaN	NaN	NaN	NaN	NaN	NaN	NaN
NaN	NaN	NaN	NaN	NaN	NaN	NaN	-0,989983	-1,11303	-1,07725
1,83254	0,381723	2,79958	2,5884	2,51388	3,33695	3,26962	2,7115	2,78591	2,8376
2,47522	3,07623	3,85197	3,5344	4,6512	1,52984	4,77562	4,04472	3,86545	4,87176
-2,66788	-3,4316	-0,347681	-0,804539	-0,797283	-2,07969	0,140694	-1,6306	-1,67223	-1,28674
1,0733	0,546618	2,24668	2,10439	3,15526	1,02058	2,8401	1,96769	1,77046	2,75566
NaN	-3,28798	-1,04742	-2,18361	NaN	-1,43073	-1,4599	NaN	NaN	NaN
-0,308924	1,44872	0,8489	NaN	1,80757	NaN	NaN	-1,45465	-1,84816	-0,652087
NaN	2,21716	0,571411	0,783791	-0,71163	1,35287	-0,785976	NaN	0,234189	NaN
0,374853	-0,842804	NaN	NaN	0,00751495	NaN	NaN	-0,583605	-0,726884	-0,807883
-0,0674419	-0,983042	-1,65825	-1,36111	NaN	NaN	-1,31625	-1,5201	-1,61294	-2,63492
-2,5028	-1,58925	-2,71279	NaN	NaN	NaN	NaN	NaN	NaN	NaN
-0,845945	-1,45387	-1,32852	-1,40537	-0,768469	NaN	-1,18135	-1,68731	-1,58967	-2,21675
-2,72538	-3,61151	-1,53458	-0,510038	-2,55799	0,304399	-1,33062	-2,82379	-4,01908	-3,08019
-0,472288	1,66747	1,38263	NaN	1,78514	NaN	NaN	1,57635	1,43651	2,26898
-1,05478	-0,665209	-0,584272	-0,530392	-0,956673	-0,887585	-0,313526	-0,570728	-1,61501	-0,797914
NaN	NaN	NaN	NaN	NaN	NaN	NaN	NaN	NaN	NaN
NaN	NaN	NaN	-0,364799	-1,36269	-0,332602	-0,617048	-1,5082	-1,45302	-1,94802
NaN	NaN	NaN	NaN	-1,66067	-0,661501	NaN	NaN	-2,67406	-2,50158
NaN	NaN	NaN	0,726839	NaN	0,336357	0,950026	0,155775	-0,170615	-0,85958
2,2451	1,92717	NaN	1,41602	2,65129	1,30919	NaN	1,3221	1,17941	1,47479
NaN	-1,85642	-0,872499	-0,877806	-2,10192	-0,29567	-1,07029	-1,12485	-1,27695	-1,60326
-3,22972	-1,82324	-1,43786	NaN	-0,616322	NaN	NaN	-1,9342	-1,86286	-0,595822
NaN	NaN	NaN	NaN	NaN	NaN	NaN	NaN	NaN	NaN
-0,75033	-2,89408	-1,31776	-2,54167	NaN	NaN	NaN	-1,69609	-1,84242	-2,22434
0,228811	-0,241423	0,576679	1,51169	0,636654	1,25941	1,8341	1,05983	0,690045	0,811223
NaN	NaN	NaN	-1,71344	-3,74658	-0,595552	-1,15574	NaN	NaN	NaN
NaN	NaN	NaN	-0,931206	-2,99987	1,04994	-0,672068	NaN	NaN	NaN
0,329372	0,321289	0,726265	2,09925	0,809387	0,185617	1,52493	0,692118	0,835513	1,01959

0,599442	NaN	NaN	NaN	0,613165	0,707254	NaN	NaN	NaN	NaN
NaN	NaN	NaN	NaN	NaN	NaN	NaN	NaN	NaN	NaN
NaN	NaN	NaN	NaN	NaN	NaN	NaN	NaN	NaN	NaN
NaN	NaN	NaN	-1,8509	3,43003	0,0363293	2,19654	3,80562	3,71184	3,11941
1,47116	1,71708	0,901249	1,48081	2,15471	0,881023	1,88326	1,25136	0,990535	1,14785
NaN	NaN	NaN	-3,33087	NaN	-3,99996	-2,02329	NaN	NaN	NaN
-0,854557	-0,401655	-0,384977	1,12599	-0,0548229	1,90426	0,166092	-0,3314	-0,506989	-0,169291
NaN	-1,41248	-1,84948	-0,460178	NaN	NaN	-1,30511	NaN	NaN	NaN
NaN	-0,674129	NaN	0,288805	NaN	-0,922932	NaN	NaN	NaN	NaN
-1,859	-2,64079	NaN	-0,394793	NaN	-0,293552	-1,38037	-3,1664	-2,7934	-2,49678
0,321177	-0,148651	-0,684576	-0,261038	-0,121986	NaN	-0,101263	-0,741112	-0,912992	-1,00348
-1,80708	-1,51351	-1,61034	-0,560247	-1,59736	0,18372	-0,914576	-1,1001	-1,72862	-2,38048
NaN	1,33568	NaN	1,95902	-0,280237	2,10983	1,36234	NaN	NaN	-0,992297
-1,65685	-2,96383	0,891113	0,45871	0,0286579	1,0365	0,898449	-0,528454	-0,410281	-0,104646
-1,61434	-1,62211	-1,41462	NaN	-0,432343	NaN	NaN	-1,52418	-1,5775	-1,00855
NaN	NaN	NaN	-0,0893841	NaN	-0,940809	-1,09748	NaN	NaN	NaN
NaN	NaN	NaN	-0,338203	NaN	-0,397118	-1,24662	NaN	NaN	NaN
0,946547	1,63995	1,25887	1,60166	1,68886	1,61434	1,67454	1,39144	0,657519	1,09844
NaN	NaN	NaN	-2,52432	-2,27098	-1,67634	NaN	-2,38205	-2,83888	NaN
NaN	NaN	NaN	-1,91524	-2,09486	-1,86395	-1,64334	-2,09838	-2,08736	-2,1225
NaN	NaN	NaN	NaN	NaN	NaN	NaN	NaN	NaN	-0,802829
NaN	NaN	NaN	NaN	NaN	NaN	NaN	-1,76114	-2,03615	-2,0675
0,861513	1,01049	1,10566	NaN	NaN	NaN	NaN	NaN	NaN	1,29137
NaN	NaN	NaN	NaN	NaN	NaN	NaN	NaN	NaN	NaN
NaN	-1,64473	NaN	NaN	NaN	NaN	NaN	NaN	NaN	NaN
NaN	NaN	NaN	NaN	NaN	NaN	NaN	NaN	NaN	NaN
NaN	NaN	NaN	NaN	NaN	NaN	NaN	NaN	NaN	NaN
NaN	NaN	NaN	NaN	NaN	NaN	NaN	NaN	NaN	NaN
NaN	NaN	NaN	-1,80528	NaN	NaN	-2,56878	NaN	NaN	NaN
-2,02713	-2,82258	-1,49568	-2,70466	NaN	-1,57629	-2,21468	-2,2916	-2,3315	-2,9422
-2,56947	-3,13912	-2,61319	-1,91809	-1,85886	-2,16193	-2,92725	-2,16003	-1,85594	-2,37917
-1,24492	-0,243486	-1,38155	-1,18721	-1,52226	-1,85729	-1,17943	-1,20188	-1,53047	-0,494222
0,383999	0,676958	0,784891	-0,273739	NaN	-1,67857	-0,95529	1,03655	0,429875	0,702043
NaN	NaN	NaN	NaN	NaN	NaN	NaN	NaN	NaN	NaN

Polito et al. Universal ready-to-use immunotherapeutic approach for the treatment of cancer: expanded and activated polyclonal γδ memory T cells.
Phosphorylation sites

-2,17421	-0,546875	-1,48141	-1,44611	-0,972486	NaN	-0,624266	-1,96114	-3,58196	-1,008
NaN	0,466129	0,419821	-0,285475	-1,02851	-0,202419	0,639532	NaN	0,408742	-1,21755
NaN	-0,395269	-0,8494	-1,62279	NaN	NaN	0,503429	-1,35679	-0,475026	NaN
-1,88638	NaN	-1,57562	-2,11042	-0,701607	NaN	-1,37967	-1,27617	-1,45487	-1,48388
1,42278	0,00925446	1,60378	1,1307	1,21205	-1,87366	1,23369	1,62963	NaN	0,857554
-0,355255	NaN	NaN	NaN	NaN	NaN	NaN	0,33914	0,103633	0,550244
NaN	NaN	NaN	NaN	NaN	NaN	NaN	NaN	NaN	NaN
NaN	NaN	NaN	NaN	NaN	NaN	NaN	NaN	NaN	NaN
NaN	-1,06662	-1,64029	-0,483017	-0,0280495	-0,207668	-0,850031	NaN	NaN	NaN
NaN	NaN	NaN	-2,48037	-1,54456	NaN	-1,73528	-2,1941	-1,65553	-2,93803
NaN	NaN	NaN	NaN	NaN	NaN	NaN	NaN	NaN	NaN
NaN	NaN	NaN	NaN	NaN	NaN	NaN	NaN	NaN	NaN
0,75034	-0,12459	0,924664	-0,309605	0,487123	NaN	0,0116405	-0,0709038	-0,42932	0,644732
-0,511332	-0,724104	-1,18615	0,431025	-0,886978	1,45864	0,0609322	NaN	NaN	NaN
NaN	NaN	NaN	NaN	NaN	NaN	NaN	NaN	NaN	NaN
NaN	NaN	NaN	NaN	NaN	NaN	NaN	NaN	NaN	NaN
-1,48089	NaN	NaN	0,277597	-1,48748	-0,123278	-1,57823	NaN	NaN	NaN
NaN	NaN	NaN	-2,13097	-3,14733	-2,89499	-2,31976	NaN	NaN	NaN
NaN	NaN	NaN	-0,956882	NaN	-1,50133	-2,43649	NaN	NaN	-2,33057
NaN	NaN	NaN	-2,34216	NaN	NaN	NaN	NaN	NaN	NaN
NaN	NaN	NaN	NaN	NaN	NaN	NaN	-2,76826	NaN	NaN
-0,913961	1,62669	-1,35019	0,152512	NaN	-0,310677	0,80113	NaN	-0,293422	-0,0851851
NaN	NaN	NaN	NaN	NaN	NaN	NaN	NaN	NaN	NaN
NaN	NaN	NaN	NaN	NaN	NaN	NaN	NaN	NaN	NaN
-0,451597	-0,43223	NaN	NaN	NaN	NaN	NaN	-0,994797	-1,30947	-1,66618
NaN	NaN	0,0653725	-0,933979	-0,685741	-0,827953	-1,11707	-0,633072	-0,314334	-0,0277929
0,21755	0,480494	1,16292	-0,435108	0,980793	-0,30102	0,0232544	1,7834	2,10742	1,50906
2,10436	2,08041	0,4601	1,63749	1,74787	1,32664	NaN	0,269997	0,193107	1,97983
-2,81741	-1,20582	-0,84201	-3,12655	-0,581909	NaN	-2,10122	-1,1471	-0,817662	-1,29731
NaN	NaN	NaN	0,0691433	NaN	-4,03898	-0,334085	NaN	NaN	NaN
NaN	NaN	NaN	-1,49968	NaN	-1,64944	-0,994823	NaN	-0,759288	-1,39206
NaN	NaN	NaN	-0,426041	NaN	NaN	-0,864077	NaN	NaN	NaN
NaN	-1,95765	NaN	-1,31982	-1,80263	-1,55303	-1,5803	-2,1236	-2,38598	-2,61594
1,95129	1,68546	2,53571	2,43959	2,09714	2,22898	1,93923	2,68258	2,60849	1,87955

Polito et al. Universal ready-to-use immunotherapeutic approach for the treatment of cancer: expanded and activated polyclonal γδ memory T cells.
Phosphorylation sites

-1,02972	-0,51576	-0,799259	-0,0707874	-0,631922	-0,523071	-0,114435	-1,32852	-0,765758	-1,40726
NaN	NaN	NaN	NaN	-2,85429	NaN	NaN	-1,57433	-1,87252	-1,88309
-1,66405	-0,87896	-1,22808	-0,634554	NaN	0,953915	-0,82527	-1,20056	-1,03936	-1,00265
NaN	NaN	NaN	-1,19169	-1,53943	-0,475443	-1,06182	NaN	NaN	NaN
-2,91755	NaN	-2,1151	NaN	-1,63192	NaN	-2,77215	NaN	NaN	NaN
-1,23658	-1,72769	-0,510885	0,00574303	-1,63554	NaN	1,44198	-0,0819759	-0,151763	-0,249492
NaN	NaN	NaN	0,0565796	0,235739	0,60034	-0,203777	NaN	NaN	NaN
-0,599319	0,899378	-0,930502	-0,48819	0,245268	-0,693701	0,494526	-0,158278	0,228845	NaN
0,0237522	-1,3923	1,71074	1,6042	1,66646	1,59603	1,90318	1,54729	1,06868	1,21967
NaN	NaN	NaN	-0,531214	-2,74382	-0,403332	-1,38585	NaN	NaN	NaN
-2,18462	-5,02404	-4,2637	-1,94841	-2,47587	-2,60772	-1,97216	-2,21456	-3,00732	-3,14544
NaN	NaN	NaN	NaN	-0,508482	NaN	-1,59785	-1,50671	-1,56652	-1,83473
NaN	NaN	NaN	-2,00352	NaN	-1,87024	-3,1367	NaN	NaN	NaN
NaN	NaN	NaN	NaN	NaN	NaN	NaN	NaN	NaN	NaN
NaN	NaN	NaN	NaN	NaN	NaN	NaN	NaN	NaN	NaN
NaN	NaN	NaN	NaN	NaN	NaN	NaN	NaN	NaN	NaN
0,673075	0,179653	0,0143642	0,129875	0,635391	0,521805	NaN	-0,201162	-0,0581713	-0,497912
-2,25266	NaN	-1,10111	-0,678272	-1,22732	-0,718391	-0,881346	-1,99514	-2,32573	-2,73184
NaN	NaN	NaN	NaN	NaN	NaN	NaN	NaN	NaN	NaN
-1,8445	-1,82092	-1,40107	-2,70754	-0,713772	NaN	-2,4501	-1,62913	-2,3247	-2,06327
-1,74793	-2,31872	-2,61086	NaN	NaN	NaN	NaN	NaN	NaN	NaN
-0,630493	-0,902849	-1,26271	-1,02812	-0,237274	-2,40536	-0,926674	NaN	NaN	NaN
NaN	NaN	NaN	-1,06869	NaN	-1,53563	-0,79258	NaN	NaN	NaN
NaN	-0,676668	-1,1202	0,29085	-1,25576	NaN	-0,284412	NaN	NaN	NaN
1,52171	1,54694	1,13665	1,31753	3,07399	1,40358	1,81878	0,926945	1,16503	0,787753
0,296455	-0,99172	-0,00581932	0,973789	1,19687	0,0311775	1,60152	0,0926399	-0,0796633	-0,2035
1,52217	2,03678	1,47817	1,57889	1,76955	1,9088	1,4519	1,38868	1,86142	2,14956
NaN	NaN	NaN	-2,85402	NaN	-1,84574	-2,86165	-3,40768	-3,09009	-3,73942
NaN	NaN	NaN	NaN	NaN	NaN	NaN	NaN	NaN	NaN
1,69798	2,57782	1,16207	2,00897	1,90569	1,88878	2,45406	1,42257	1,03285	1,32666
0,716238	0,395409	1,05157	-1,43752	2,05547	NaN	0,79393	1,01078	1,14708	1,45349
NaN	NaN	NaN	NaN	NaN	NaN	NaN	NaN	NaN	NaN
NaN	NaN	NaN	NaN	NaN	NaN	NaN	NaN	NaN	NaN
NaN	NaN	NaN	NaN	NaN	NaN	NaN	NaN	NaN	NaN

Polito et al. Universal ready-to-use immunotherapeutic approach for the treatment of cancer: expanded and activated polyclonal γδ memory T cells.
Phosphorylation sites

NaN	NaN	NaN	NaN	NaN	NaN	NaN	NaN	NaN	NaN
NaN	NaN	NaN	NaN	NaN	NaN	NaN	NaN	NaN	NaN
-0,595705	-0,869024	0,354229	-0,323799	0,229082	0,060318	NaN	0,24975	-0,127566	-0,738355
2,87286	NaN	2,13129	2,9001	NaN	NaN	2,40798	2,14711	1,72614	1,51854
NaN	NaN	NaN	NaN	NaN	NaN	NaN	NaN	NaN	NaN
-0,969786	0,919285	-0,264591	0,473745	0,582638	-1,45845	0,353378	-1,45	-1,16958	-0,49377
-1,73904	-1,01845	NaN	-1,08899	-1,55405	-1,76146	NaN	NaN	NaN	NaN
NaN	NaN	NaN	-0,12306	-1,05527	-0,454288	-0,61849	NaN	NaN	NaN
-1,98331	-2,7994	-2,63541	-1,98965	-2,24406	-3,18485	-1,81466	-2,74245	-2,48998	-2,4771
-2,52203	-2,24111	-1,88051	-0,90505	-1,97009	NaN	-1,1065	-3,3711	-3,07337	-1,98551
NaN	NaN	NaN	NaN	NaN	0,20113	NaN	NaN	NaN	NaN
1,40384	1,75195	1,65166	1,17566	2,84141	NaN	NaN	1,44088	1,40557	2,12899
NaN	NaN	NaN	NaN	NaN	NaN	NaN	NaN	NaN	NaN
3,11697	2,21598	1,60935	4,22965	3,62618	3,15129	3,80496	2,61436	2,25804	2,17976
NaN	NaN	NaN	0,740326	NaN	1,67662	-1,50049	NaN	NaN	NaN
3,11697	2,21598	1,60935	4,22965	3,62618	3,15129	3,80496	2,61436	2,25804	2,17976
-0,496078	-0,311281	-0,221693	-0,637411	0,76609	NaN	-0,0666351	-0,299738	-0,633155	-0,46219
NaN	NaN	NaN	NaN	NaN	NaN	NaN	NaN	NaN	NaN
-0,474735	-0,473034	-1,67988	-1,04009	-0,70022	-1,04875	-1,32049	NaN	NaN	NaN
-0,391863	0,32378	-0,639236	-0,943209	0,650595	NaN	-0,773623	-0,0253887	-0,461463	-0,692534
3,4894	3,71674	3,22061	2,42638	4,15317	NaN	3,13743	2,99742	2,63795	3,24647
NaN	NaN	NaN	0,0854702	NaN	1,0058	-1,31219	-1,398	-1,29365	NaN
NaN	NaN	NaN	-1,79657	-3,40211	-1,74032	-1,96804	NaN	NaN	NaN
-0,664513	NaN	-1,11735	0,681484	-1,6911	0,872803	0,135149	-0,426348	-0,674619	-0,573522
-0,858152	NaN	-0,222929	-0,440212	-0,301279	-0,428352	-0,396284	-0,424204	-1,38432	-0,586867
1,56701	0,786861	NaN	2,12628	2,21272	2,08817	1,13631	NaN	NaN	NaN
0,906979	NaN	0,104914	0,195379	0,711349	NaN	-0,101479	-0,375826	-0,810199	-0,529893
-0,0538483	-0,361334	-1,02404	-0,605762	-0,70318	-0,978117	-0,741098	-0,526011	-0,886552	-0,49109
NaN	NaN	NaN	NaN	NaN	NaN	NaN	NaN	NaN	NaN
NaN	NaN	NaN	NaN	0,714523	-0,0908813	0,995234	NaN	NaN	NaN
0,657593	-0,876385	-0,26347	0,19202	0,442377	1,02907	0,78488	0,617622	0,491071	0,597087
-1,40984	NaN	NaN	-0,750843	-1,50319	-0,00528145	-0,67164	NaN	NaN	NaN
0,102846	NaN	-0,232784	-0,213266	0,401396	0,582605	-0,585823	NaN	NaN	NaN
-0,149313	0,158175	NaN	0,842274	0,620581	0,846319	NaN	0,20607	0,274863	-0,162827

Polito et al. Universal ready-to-use immunotherapeutic approach for the treatment of cancer: expanded and activated polyclonal γδ memory T cells.
Phosphorylation sites

0,520479	0,041975	-0,422894	-0,328339	0,112366	-0,186647	-0,164076	0,049427	-0,836215	-0,139712
1,02301	1,22953	1,75003	0,185425	1,09926	-2,2951	1,55908	2,32738	1,57071	1,03871
NaN	NaN	NaN	0,000514984	-0,395	NaN	-0,279657	NaN	NaN	NaN
-1,25048	-1,26686	-0,723421	-0,233028	-1,6405	-0,0364056	0,0457935	-1,4134	-1,03443	-0,453553
1,15265	0,808817	1,3699	0,767145	1,34268	-4,17101	0,640106	1,23577	1,0721	1,05238
NaN	NaN	0,510832	0,648325	NaN	1,16951	0,816185	NaN	NaN	NaN
0,905073	1,62366	1,5209	1,15987	0,529341	1,72903	1,2739	NaN	1,64859	NaN
0,993826	-0,218021	-0,366442	NaN	0,377455	NaN	-0,64669	-0,366888	-0,233434	-0,0901346
-0,764555	-2,14475	-0,362375	-0,663931	NaN	NaN	-0,238264	-0,416094	NaN	NaN
NaN	NaN	NaN	-1,44802	-1,96875	NaN	-0,418571	-1,08066	NaN	NaN
NaN	NaN	NaN	NaN	-1,96875	-2,14312	-0,418571	NaN	-1,19457	NaN
NaN	NaN	NaN	NaN	NaN	NaN	NaN	NaN	NaN	NaN
NaN	NaN	NaN	NaN	NaN	NaN	NaN	NaN	NaN	NaN
NaN	NaN	NaN	NaN	NaN	NaN	NaN	NaN	NaN	NaN
NaN	NaN	NaN	NaN	NaN	NaN	NaN	NaN	NaN	NaN
2,96796	2,93406	2,79115	1,30091	2,93517	1,7565	1,90132	3,15438	2,89732	3,02617
-1,24677	-1,52275	-1,60931	-1,3604	NaN	-1,45391	0,342493	NaN	-0,984153	NaN
NaN	NaN	NaN	NaN	NaN	NaN	NaN	NaN	NaN	NaN
-3,0397	NaN	-1,97277	-1,44101	-0,859324	-0,783463	-1,29071	NaN	NaN	NaN
-1,12105	-2,21202	-0,0302429	-0,0987244	NaN	0,00526428	-0,208599	NaN	NaN	NaN
NaN	NaN	-0,467655	-0,710577	NaN	-0,193483	0,0703926	NaN	NaN	NaN
NaN	NaN	NaN	NaN	NaN	NaN	NaN	NaN	NaN	NaN
-1,2097	-3,11813	-1,5714	-2,1967	-1,85742	NaN	-2,63083	-0,1999	-1,01271	-1,43982
-1,66109	-1,46622	NaN	-1,46928	-1,67295	-1,84428	NaN	NaN	NaN	NaN
3,51153	3,70365	3,8969	3,91636	3,57442	2,51604	3,78621	3,62608	3,24216	3,81116
-0,373819	-0,769987	-1,4153	0,481401	NaN	1,05963	1,00851	-0,874544	-0,0440207	NaN
NaN	NaN	NaN	1,18151	-0,741171	1,74052	0,960283	-0,213486	0,328292	-0,289378
-0,890186	NaN	NaN	0,532368	-0,316423	1,68961	NaN	-0,156052	0,316335	NaN
NaN	NaN	NaN	-1,30181	NaN	-1,38094	-1,99238	-0,882616	NaN	-1,39604
1,07001	0,414234	0,964531	2,54971	0,559269	3,0247	2,44711	1,69226	2,23226	1,94031
-2,3474	-2,83586	0,319048	-1,12643	-1,26755	-1,07618	-1,03777	-2,4079	-2,13457	-2,04573
1,82676	NaN	1,21471	NaN	1,3981	0,886673	0,970697	NaN	NaN	NaN
0,162813	0,475573	-0,386917	-0,17996	0,877811	-0,605381	0,688332	0,390923	-0,135407	0,0390196
NaN	NaN	NaN	-0,300112	-1,22759	NaN	-3,35303	NaN	NaN	NaN

Polito et al. Universal ready-to-use immunotherapeutic approach for the treatment of cancer: expanded and activated polyclonal γδ memory T cells.
Phosphorylation sites

-4,51806	NaN	1,19734	1,97276	NaN	2,45173	1,44756	-2,96324	-2,98325	-2,34228
-0,302084	-0,317888	-0,0307407	1,70225	0,125561	NaN	1,21128	-0,166708	-0,68151	-0,879157
NaN	NaN	NaN	-1,22203	-4,31453	-0,487438	-1,41089	NaN	NaN	NaN
1,19499	1,54122	1,75951	1,87832	NaN	3,1607	1,7475	1,47492	1,22776	1,23905
-2,71713	-1,71331	-1,12764	-2,58039	-0,3095	-2,1311	NaN	-1,42789	-2,45055	NaN
NaN	NaN	NaN	NaN	NaN	-0,640575	NaN	NaN	NaN	NaN
NaN	NaN	NaN	-2,40046	NaN	-1,34129	-3,28471	NaN	NaN	NaN
-0,81377	-1,25986	-0,278843	-0,0430622	NaN	-0,866238	-0,37248	-1,08393	-0,410825	-0,978486
1,16544	0,184732	0,793966	1,17997	NaN	0,353434	-0,41544	0,116396	0,233039	-0,515979
-0,545424	-0,559423	-0,111931	NaN	-0,0547047	-0,686085	-0,966251	NaN	-1,18988	NaN
-1,84587	-1,33616	-1,74461	-0,899691	-0,772738	-1,31255	-0,597948	-1,91903	-2,00759	NaN
NaN	NaN	NaN	NaN	-2,20041	NaN	-1,68721	NaN	NaN	NaN
1,2182	NaN	0,0542068	1,06606	NaN	2,01193	-0,233158	0,212391	NaN	NaN
4,34548	4,05432	4,12163	4,16215	3,43179	3,99186	4,47753	4,62929	4,2645	4,21365
NaN	NaN	NaN	NaN	NaN	NaN	NaN	NaN	NaN	NaN
1,48446	2,60209	0,683615	0,735477	1,28142	-0,963297	1,80504	0,0094471	-0,171792	2,14853
0,152489	1,45469	NaN	0,439182	1,96551	1,25825	NaN	2,15888	1,58752	1,4407
NaN	NaN	NaN	-2,03466	NaN	-1,20731	-2,72577	NaN	NaN	NaN
-2,15639	-1,73783	-2,94565	NaN	NaN	NaN	NaN	NaN	NaN	NaN
NaN	NaN	NaN	0,680796	-2,14554	NaN	0,247087	NaN	NaN	NaN
NaN	-0,943497	-1,05273	-1,63233	-0,704662	-1,80959	-0,737629	-1,20533	-1,23827	-1,58911
-0,170258	0,000249863	-0,241753	NaN	-0,197899	NaN	0,00194359	-0,287418	-0,662135	-0,721494
1,89262	2,93289	1,7451	2,30482	2,88509	2,0243	2,51585	2,26071	1,75929	1,88646
NaN	NaN	NaN	NaN	NaN	NaN	NaN	NaN	NaN	NaN
2,53656	2,22508	2,05892	3,03313	2,73601	1,14306	3,01883	1,42415	1,83442	2,66655
NaN	NaN	NaN	0,151535	NaN	NaN	NaN	NaN	NaN	NaN
NaN	NaN	NaN	-0,230049	-1,77541	-0,0916481	-0,8085	NaN	NaN	NaN
NaN	NaN	NaN	NaN	-0,358053	-0,882229	NaN	-1,46742	NaN	-0,922563
0,781708	0,683569	0,815928	1,42768	2,10681	0,151716	1,36017	0,08144	0,322122	1,33651
-1,06604	-1,03512	-0,781807	2,13757	NaN	-0,455702	0,763449	-1,60048	-1,68873	NaN
NaN	NaN	NaN	NaN	NaN	NaN	NaN	NaN	NaN	NaN
0,387159	0,592281	-0,138657	2,31228	1,58209	1,83887	1,66456	-0,00790405	0,599147	0,678147
-0,404512	-0,865416	0,663416	1,2977	0,920483	-0,422108	2,31386	-0,479259	-0,292392	0,222253
-0,522318	-1,24771	2,38243	0,884706	-0,973434	2,18928	1,85309	NaN	0,107902	NaN

Polito et al. Universal ready-to-use immunotherapeutic approach for the treatment of cancer: expanded and activated polyclonal γδ memory T cells.
Phosphorylation sites

NaN	NaN	NaN	NaN	NaN	NaN	NaN	NaN	NaN	NaN
NaN	NaN	NaN	NaN	-4,38436	-0,394012	-1,00179	NaN	NaN	-1,33161
0,943764	1,3303	0,582071	1,56324	1,21315	2,04956	1,88005	2,09025	1,49203	1,97872
NaN	NaN	NaN	NaN	NaN	NaN	NaN	NaN	NaN	NaN
NaN	NaN	NaN	NaN	NaN	NaN	NaN	NaN	NaN	NaN
NaN	NaN	NaN	NaN	NaN	NaN	NaN	NaN	NaN	NaN
NaN	NaN	NaN	-3,34246	NaN	-2,02776	-2,24794	NaN	NaN	NaN
NaN	NaN	NaN	-1,48212	NaN	-1,38807	-2,19521	NaN	NaN	NaN
NaN	NaN	NaN	NaN	NaN	NaN	NaN	NaN	NaN	NaN
0,64423	1,02939	0,417917	0,208134	0,814522	-0,579615	0,407404	0,277651	0,763162	0,557528
NaN	NaN	NaN	-0,190662	NaN	-0,631729	-2,30942	NaN	NaN	NaN
NaN	NaN	NaN	NaN	NaN	NaN	NaN	NaN	NaN	NaN
2,21272	1,65551	0,953955	3,72278	1,42926	1,26315	2,7279	1,70562	1,6034	1,42232
NaN	NaN	NaN	NaN	NaN	NaN	NaN	-2,00693	-2,06093	-2,04119
0,0701599	-0,203077	-0,388191	-2,26432	-0,610319	NaN	-1,17913	7,44E-05	-0,211564	-0,643243
0,999531	0,0902157	0,931904	-0,0872345	-0,372961	-0,053093	-0,245584	0,167149	0,272388	0,908568
-2,8891	-1,69786	NaN	-1,50239	-1,05953	NaN	-0,654057	NaN	NaN	NaN
NaN	NaN	NaN	-1,41509	-3,45934	NaN	-2,02229	NaN	NaN	NaN
NaN	NaN	NaN	-0,222738	0,0850697	NaN	-1,20888	NaN	NaN	NaN
-0,74202	-2,60917	-1,03373	-1,00469	-0,0993614	NaN	-1,298	NaN	NaN	NaN
NaN	NaN	NaN	0,560934	NaN	0,311176	-1,07824	NaN	NaN	NaN
1,38601	NaN	0,44046	1,86294	1,38716	0,238379	0,349508	NaN	NaN	NaN
NaN	-0,346323	-1,12029	NaN	-0,960651	NaN	-2,92945	NaN	-2,24856	-0,650645
1,37607	2,78239	1,63653	1,78005	0,956356	1,85754	2,82929	1,36689	1,71905	1,18234
-0,565168	2,08056	0,781073	0,570498	1,83173	-2,20902	2,0895	0,353462	0,451859	1,3113
-1,27929	NaN	1,25096	-0,0888367	-0,916136	-1,54514	0,768705	-0,785263	-1,35664	NaN
0,99102	2,08853	1,87635	1,11177	2,51468	2,67574	2,01383	1,92044	1,30291	1,98256
0,446165	-0,283503	0,553888	0,706707	-0,931671	1,30634	-0,280146	0,421247	0,411952	0,4281
0,988098	0,246048	1,04645	4,18466	0,0364952	3,83486	2,46163	0,414133	0,182952	0,21058
NaN	NaN	NaN	-1,76087	-2,61946	NaN	-0,99469	NaN	NaN	NaN
0,788572	1,16055	0,616903	2,12144	1,66315	1,39381	1,7358	0,832621	0,566421	1,4283
-0,898525	NaN	-0,745106	-0,701372	-0,116674	-0,803192	-0,181559	-0,052578	-0,628423	-0,19625
-0,935741	NaN	NaN	-1,25447	NaN	-1,58957	-0,738075	NaN	NaN	NaN
1,26493	1,18535	1,50171	0,748337	2,12667	-0,232513	1,28136	NaN	NaN	NaN

Polito et al. Universal ready-to-use immunotherapeutic approach for the treatment of cancer: expanded and activated polyclonal γδ memory T cells.
Phosphorylation sites

0,678104	1,70126	0,447115	0,567276	0,0395699	1,58712	1,50207	1,16379	1,07862	0,867652
-0,898027	-1,99212	-2,12931	-0,199692	-1,88593	0,306337	-0,411777	-2,711	NaN	NaN
0,142029	0,452307	-0,343641	0,201612	0,870211	0,5417	0,718525	0,903135	0,44806	0,419631
NaN	NaN	NaN	NaN	NaN	NaN	NaN	NaN	NaN	NaN
-1,80966	-0,376684	-2,34291	0,65406	NaN	0,587238	NaN	NaN	NaN	NaN
-1,00299	-1,00585	NaN	1,66645	-0,0232449	1,07433	NaN	-1,79147	-1,52402	0,543506
4,19397	3,67247	4,13037	5,31607	4,42291	5,23223	4,21569	4,06258	4,12184	3,89852
NaN	-0,37075	-2,15645	3,19389	NaN	NaN	2,03045	0,871311	-2,62162	-4,3875
-0,954176	0,507483	-1,68983	0,755758	-0,0248642	-0,974463	-0,0467854	NaN	-1,72604	-0,418994
0,426004	0,657337	-0,35737	-0,270187	NaN	NaN	-0,00912285	0,203104	0,332305	-0,365239
NaN	NaN	NaN	NaN	NaN	NaN	NaN	NaN	NaN	NaN
2,35633	2,01947	2,27077	1,08533	2,65901	0,729902	2,31525	2,20635	1,99938	2,15853
NaN	NaN	NaN	NaN	NaN	NaN	NaN	NaN	NaN	NaN
4,79695	5,37659	4,07887	3,85468	4,4839	4,77006	5,34228	4,49559	4,65877	4,30077
NaN	NaN	NaN	1,07053	NaN	NaN	NaN	NaN	NaN	NaN
NaN	NaN	NaN	-0,808559	-2,29673	NaN	-0,668617	NaN	NaN	NaN
NaN	NaN	NaN	NaN	NaN	NaN	NaN	NaN	NaN	NaN
NaN	NaN	NaN	NaN	NaN	NaN	NaN	NaN	NaN	NaN
NaN	NaN	NaN	NaN	NaN	NaN	NaN	NaN	NaN	NaN
-0,220943	NaN	NaN	NaN	-0,395075	NaN	NaN	-1,08743	-0,616897	-1,18353
-0,25831	-2,27652	3,79601	3,33259	0,119999	3,60659	3,47425	2,05221	1,05345	1,9245
0,586002	1,14288	1,8654	1,93278	0,968569	-1,54865	1,87244	NaN	NaN	NaN
-1,79999	-1,62192	-2,33167	-0,32127	-1,6668	-1,33611	-0,706005	NaN	-3,02966	-2,14272
NaN	NaN	NaN	-1,64866	-0,811335	-2,36993	-0,565771	NaN	NaN	NaN
2,35179	2,53999	2,24302	3,31755	2,86579	1,14447	2,55737	2,83612	2,84847	2,38582
1,21599	0,649115	1,19707	1,80425	1,89563	-2,3508	1,79445	1,22898	0,665299	1,13934
-0,734461	-0,972702	-0,394754	-2,45344	-0,827188	NaN	NaN	NaN	-0,348943	NaN
0,849424	NaN	NaN	-0,86764	0,677349	NaN	-0,289757	NaN	NaN	NaN
NaN	-1,05972	-1,40505	-1,41455	-0,895361	-0,584135	-0,46307	NaN	NaN	NaN
NaN	NaN	0,0441074	NaN	-0,170185	NaN	NaN	0,15461	-0,192595	-0,240272
0,0742779	-0,152229	-0,636564	-0,796408	-0,33069	-1,00163	-1,04914	0,262676	-0,0207186	-0,0734262
-1,80809	-2,08709	-1,54834	-0,728193	NaN	-0,18733	-0,477715	NaN	NaN	NaN
-1,54888	-0,921082	-1,58895	-1,45566	0,0263424	NaN	-0,733721	-1,68305	NaN	-0,929408
NaN	NaN	NaN	-0,340553	NaN	0,0377312	-1,46421	NaN	NaN	NaN

Polito et al. Universal ready-to-use immunotherapeutic approach for the treatment of cancer: expanded and activated polyclonal γδ memory T cells.
Phosphorylation sites

-0,0309772	-0,302282	-1,06488	-0,899897	0,505264	-0,29829	-0,401941	-1,0607	-1,40064	-0,26913
NaN	NaN	NaN	NaN	NaN	NaN	NaN	NaN	NaN	NaN
NaN	NaN	NaN	-1,0831	-1,74565	-2,86578	-2,46396	-0,0205479	-0,761476	0,578242
NaN	NaN	NaN	-1,24935	NaN	-0,490364	-1,89441	NaN	NaN	NaN
NaN	NaN	NaN	NaN	NaN	-0,576574	NaN	NaN	NaN	NaN
NaN	NaN	NaN	NaN	NaN	NaN	NaN	NaN	NaN	NaN
NaN	NaN	NaN	NaN	NaN	NaN	NaN	NaN	NaN	NaN
NaN	-2,4543	-0,529062	-0,326241	NaN	-0,281151	-0,305855	NaN	NaN	NaN
-0,076498	0,619812	-0,655304	NaN	-0,954468	-0,448177	NaN	NaN	-0,3789	-2,11224
NaN	NaN	NaN	-1,13359	-2,02284	-1,23457	-1,20223	NaN	NaN	NaN
NaN	-1,06544	NaN	-0,999098	-0,440886	NaN	-0,772209	NaN	NaN	NaN
NaN	-0,709688	NaN	-1,18629	-0,797283	1,66056	-1,37799	-1,46631	-1,92202	-1,49257
-1,57644	-3,57322	-2,22149	-1,51484	-0,223465	-1,37966	-2,27592	-1,74893	-1,29546	-0,323977
NaN	NaN	NaN	0,359325	NaN	0,942862	-1,34384	NaN	NaN	NaN
0,62871	0,298162	0,391144	1,11135	-0,221142	NaN	0,67033	0,294304	0,11523	0,0110769
NaN	NaN	-1,0032	-1,46634	NaN	-0,657904	-1,6509	-1,93993	-1,76531	-1,39846
-0,44256	NaN	NaN	-0,149935	NaN	-0,0341949	0,253983	NaN	-0,455348	NaN
-1,13692	-0,204729	-1,75287	-1,18478	NaN	NaN	-0,541628	-2,57505	NaN	-0,313901
-2,79708	-3,59384	-1,24441	-1,43657	-2,45563	NaN	-0,418064	-1,32545	-1,65237	NaN
0,890097	0,514479	NaN	-0,506954	NaN	-0,0621967	0,802795	2,13971	2,81278	1,04272
0,426353	-0,0581264	0,933237	0,345461	0,638226	-1,59713	0,23806	0,575813	0,406655	0,198192
3,44615	3,40002	3,71323	3,17391	3,75513	0,146095	3,49378	3,00334	2,82449	2,84962
-1,50692	-0,437853	-0,334257	-0,816372	-0,183071	-1,56023	-1,01962	-1,02113	-0,952453	-0,62951
-1,99057	-1,77558	-2,21292	NaN	NaN	NaN	NaN	NaN	NaN	NaN
2,28237	3,44033	1,13646	2,64565	2,8672	3,28035	3,48547	0,815714	1,18492	3,20427
NaN	NaN	NaN	0,90399	NaN	1,02807	1,52322	NaN	NaN	NaN
1,15185	0,323158	0,135666	1,62181	NaN	1,99008	NaN	0,388086	-0,0804205	NaN
NaN	NaN	NaN	-2,1762	NaN	-1,33188	-1,65265	NaN	NaN	NaN
NaN	NaN	NaN	0,193035	-3,25941	0,207058	0,257799	NaN	NaN	NaN
NaN	NaN	NaN	NaN	NaN	NaN	NaN	NaN	NaN	NaN
0,611443	-0,592915	-0,765141	-1,1241	0,458109	-2,62558	0,433502	-0,23781	-0,51661	-0,343703
1,60647	-0,314804	1,09469	1,53675	1,82155	NaN	1,28485	1,00259	0,715123	0,387923
NaN	-0,669811	NaN	-1,59542	-0,822956	-0,833366	-0,898489	-1,52267	-1,83779	-1,92404
NaN	NaN	NaN	NaN	NaN	NaN	NaN	NaN	NaN	NaN

Polito et al. Universal ready-to-use immunotherapeutic approach for the treatment of cancer: expanded and activated polyclonal γδ memory T cells.
Phosphorylation sites

-1,00671	-0,698294	-0,885597	-1,32968	-0,332945	-2,24499	-0,0624046	-0,909349	-1,18946	0,188497
-0,20105	0,689859	-0,498047	-0,807175	-0,443319	NaN	-0,115597	-0,481417	-0,422351	-0,040246
3,67994	3,16237	1,74302	3,65693	3,03654	3,34935	3,34208	2,15771	1,85066	2,15163
NaN	NaN	NaN	-1,29539	NaN	-2,00367	NaN	NaN	NaN	NaN
-2,61592	-1,68298	-1,62989	-0,790449	-2,82843	-0,593435	-1,10534	-2,00723	-1,92684	-2,375
0,280243	-0,00329018	-0,132998	NaN	-0,16667	NaN	-0,760767	-0,180634	-0,390492	-0,477414
NaN	NaN	NaN	-1,8909	NaN	-0,473677	NaN	NaN	NaN	NaN
-0,220709	1,2511	-0,0356865	-2,00469	NaN	NaN	-1,11432	-0,223789	0,00118923	-0,401942
1,31351	1,36113	2,30317	1,78491	2,59202	2,96198	2,27473	0,605412	0,601041	0,825667
-0,909008	-0,50429	-1,00695	1,31295	0,00428581	2,23657	1,99254	-0,300045	-0,746533	-0,67895
NaN	NaN	NaN	-2,01016	-3,35099	-1,95789	-0,906719	NaN	NaN	NaN
1,61429	2,3644	1,41701	3,77555	2,12465	3,95748	3,30059	2,07943	1,84113	2,89888
-2,09017	0,568527	NaN	NaN	NaN	2,43711	-1,11161	NaN	NaN	NaN
-0,706038	NaN	NaN	-2,00396	0,487873	NaN	-0,431021	0,447548	0,316548	-0,280402
NaN	NaN	NaN	-1,27563	NaN	0,499855	-1,43383	NaN	NaN	NaN
-1,26564	-2,87716	1,04645	1,09638	-0,16326	0,380474	0,662727	0,374298	0,115949	0,714543
NaN	-2,2949	NaN	-2,60546	-2,19467	NaN	-2,10756	NaN	NaN	NaN
1,56097	0,885025	1,70171	1,69415	1,56758	-0,665316	1,48021	0,572329	1,4613	0,565568
NaN	0,71608	-1,33172	0,880119	-0,115011	-0,203384	NaN	-1,63966	-1,54769	-0,0254087
-2,05933	-2,88634	-2,5479	-0,639957	-1,69046	-4,16446	NaN	NaN	NaN	NaN
NaN	NaN	NaN	-3,61624	NaN	-1,57983	NaN	NaN	NaN	NaN
1,2988	1,80494	0,865747	2,77721	1,3679	3,93971	2,65753	1,82911	1,40557	2,22665
-0,993097	-2,08451	-0,298782	0,695059	0,291023	0,277859	-0,0756264	-1,39564	-1,32696	-1,41973
1,07288	-0,403261	1,06095	2,51443	1,97278	-1,00444	2,13336	1,19539	0,85049	1,25846
NaN	-0,91843	NaN	1,01285	NaN	0,455362	0,371906	NaN	NaN	NaN
0,903404	2,28038	2,30652	1,49959	3,09521	1,648	3,08703	3,07471	2,72394	2,50073
0,116705	-0,192091	NaN	-0,183903	NaN	-0,334867	0,248857	NaN	-0,00232029	-0,672425
NaN	NaN	NaN	NaN	NaN	NaN	NaN	NaN	NaN	NaN
NaN	NaN	NaN	-0,983532	-0,789154	NaN	-0,977999	-0,873631	-1,6394	-1,00339
-1,05084	-1,1036	-2,07016	-0,55125	NaN	NaN	-0,829355	NaN	NaN	NaN
-1,1947	-0,845207	NaN	-1,14079	0,234482	-1,62473	-0,597746	NaN	NaN	NaN
-1,92927	NaN	-0,209362	NaN	-1,41419	-1,68657	-2,26944	-0,709934	-1,80776	NaN
-0,989592	NaN	0,987268	0,349203	-1,21704	0,58947	1,27353	-1,11774	-0,299214	-0,873975
NaN	-1,59038	-1,75695	NaN	-0,546181	NaN	-2,01397	-2,7412	-2,36438	-1,44794

-1,56174	-2,08788	-1,98312	NaN	NaN	NaN	NaN	-2,3921	-2,14017	-1,90664
NaN	-0,0406189	1,02317	NaN	1,25223	1,07955	0,402407	1,44702	1,32682	1,54615
NaN	NaN	NaN	NaN	NaN	NaN	NaN	NaN	NaN	NaN
NaN	NaN	1,28954	0,389669	-2,23306	1,4701	0,809187	NaN	NaN	NaN
NaN	-2,38724	-1,71752	NaN	NaN	NaN	NaN	-1,30214	-1,68966	-1,76771
NaN	NaN	NaN	-0,790133	-0,7169	-1,32439	-0,447475	-0,655409	-0,759391	-0,856999
0,345518	0,480381	0,490685	NaN	1,16083	-2,23119	0,802412	0,521297	0,08002	0,236154
NaN	NaN	NaN	-2,46172	NaN	-0,400183	-2,40976	NaN	NaN	NaN
NaN	NaN	NaN	NaN	NaN	NaN	NaN	NaN	NaN	NaN
NaN	NaN	NaN	-1,20252	NaN	NaN	-2,58037	NaN	NaN	NaN
NaN	NaN	NaN	NaN	NaN	NaN	NaN	NaN	NaN	NaN
NaN	NaN	NaN	NaN	NaN	NaN	NaN	NaN	NaN	NaN
NaN	NaN	NaN	-1,15337	NaN	-0,638391	NaN	NaN	NaN	NaN
NaN	NaN	NaN	-0,0666046	NaN	NaN	-0,538023	NaN	NaN	NaN
NaN	NaN	NaN	NaN	NaN	NaN	NaN	NaN	NaN	NaN
-0,562956	-2,42958	-2,03195	-0,214567	-1,68899	NaN	-1,22928	-0,893	-1,05333	-2,34158
-1,24159	NaN	-0,843454	-0,529808	-0,380974	NaN	-0,509493	NaN	NaN	NaN
NaN	NaN	NaN	-2,95303	-2,46362	-1,85963	-1,76391	NaN	NaN	NaN
NaN	NaN	NaN	-1,78988	-1,24191	NaN	NaN	NaN	NaN	NaN
NaN	NaN	NaN	NaN	-1,42637	-2,8807	-1,92803	-1,31741	-2,81344	-2,15147
NaN	NaN	NaN	-0,60705	NaN	-0,976065	-0,468006	NaN	NaN	NaN
-1,90993	-2,35007	0,325506	NaN	NaN	NaN	NaN	0,0258083	0,222196	NaN
NaN	NaN	NaN	0,627176	NaN	-0,197187	0,0175266	NaN	NaN	NaN
NaN	NaN	NaN	-2,42276	NaN	-0,940004	NaN	NaN	NaN	NaN
NaN	NaN	NaN	-0,43924	-1,19656	0,0261269	NaN	-0,817957	-0,832961	-0,989015
1,23366	2,14746	1,00375	1,48987	2,14311	0,637943	1,85901	1,31099	0,239429	1,42035
-0,892422	-3,38329	-0,850393	-1,53595	-0,526388	-1,13034	-0,847576	-0,798	-0,470529	-0,970143
NaN	NaN	NaN	-1,11743	NaN	-0,723507	-0,530256	NaN	NaN	NaN
-1,17305	0,921095	-0,554705	-0,35833	-1,31876	0,610815	-0,265028	-1,27098	NaN	-0,640307
-0,848757	1,36543	-0,641947	-0,103634	-0,740601	-1,42325	0,63106	-2,2645	-0,653689	0,304662
NaN	-1,49206	NaN	-2,28067	NaN	NaN	-1,88324	-2,0182	-1,65863	-1,31907
NaN	NaN	NaN	-1,48289	-2,21104	-1,53765	-1,04845	NaN	NaN	NaN
1,49083	0,814939	0,798119	0,821932	0,745565	-0,570599	0,909674	0,669369	0,389865	0,689576
NaN	NaN	NaN	NaN	NaN	NaN	NaN	-2,51332	-2,60893	-1,98615

Polito et al. Universal ready-to-use immunotherapeutic approach for the treatment of cancer: expanded and activated polyclonal γδ memory T cells.
Phosphorylation sites

NaN	NaN	NaN	-1,48943	NaN	-1,23032	NaN	NaN	NaN	NaN
NaN	NaN	NaN	NaN	NaN	NaN	NaN	NaN	NaN	NaN
1,37573	1,16187	-2,25106	2,29845	2,1595	0,860182	2,88937	1,92471	1,17986	2,06544
2,5655	2,07188	1,42382	3,28616	2,40515	0,77507	2,03945	1,51431	1,07122	1,35788
NaN	NaN	NaN	-1,5967	-1,35155	-2,94182	-0,479364	-1,16087	-1,45797	-1,30177
2,4855	3,1319	2,66672	2,7403	3,88949	1,76907	3,40415	2,35485	2,17524	2,85689
-0,825035	-0,406914	-0,435366	-0,0965996	0,666643	-1,45431	0,749294	-0,157137	-0,606275	0,0292711
NaN	-1,15728	NaN	-1,06514	NaN	2,51442	-1,97787	NaN	NaN	NaN
-0,818407	-0,731121	-0,192284	1,32364	-0,467018	-0,624634	0,92823	-1,35922	-1,15174	-0,950719
-0,833561	-1,84742	-2,12912	-1,25714	-2,18857	-1,97399	-0,466463	-0,365828	-0,894345	-1,59118
NaN	NaN	NaN	-2,90364	-3,17914	NaN	-0,32152	-0,62863	-0,729398	-1,17444
0,728548	1,39137	2,05188	1,98386	2,02973	1,34958	2,12447	1,79408	1,30042	1,47792
3,1811	3,06639	3,31176	3,56311	3,01091	2,39029	3,10714	3,68205	3,58676	2,94839
NaN	-2,54167	NaN	-1,91573	-2,51878	NaN	-0,722578	-0,986668	-0,658633	-1,01781
NaN	NaN	-1,353	-0,425646	-3,20838	-1,58633	1,03656	-2,18704	NaN	-2,60627
NaN	NaN	NaN	-1,84679	NaN	-2,3713	-1,86709	NaN	NaN	NaN
0,330811	0,887468	-0,409544	0,866673	0,908173	0,853699	-0,305662	0,584206	0,391633	0,0739317
NaN	NaN	NaN	-1,71374	NaN	-1,33052	-1,59454	NaN	NaN	NaN
NaN	NaN	-0,0991364	0,108175	-3,07223	-0,464041	0,371769	-1,55134	NaN	-2,04006
NaN	NaN	-1,66099	-0,616772	NaN	0,41279	-0,761902	NaN	NaN	NaN
NaN	NaN	NaN	-0,185783	NaN	-0,231951	-0,0155239	NaN	NaN	NaN
NaN	NaN	NaN	NaN	NaN	NaN	0,855619	NaN	NaN	-0,964267
3,5558	3,43342	2,77427	3,09676	2,96311	3,16618	2,85398	3,07228	2,74926	2,90634
-3,44317	0,282751	NaN	NaN	NaN	NaN	NaN	0,149672	0,25171	0,914548
NaN	NaN	NaN	-1,81294	NaN	-1,16827	-1,75574	NaN	NaN	NaN
NaN	NaN	NaN	1,0499	NaN	0,790255	0,191696	-2,56005	NaN	-2,49471
-1,62456	NaN	NaN	0,58049	-2,54388	1,59813	0,138889	-0,840916	-1,07819	-1,84574
NaN	NaN	NaN	0,0279446	-2,66189	NaN	-0,529902	NaN	-2,61306	-1,69101
NaN	-1,71474	-0,496759	1,78997	NaN	2,25359	1,30841	NaN	-0,297978	NaN
0,157501	-0,101324	0,872246	2,54848	0,708097	2,98459	2,15082	0,70616	0,277133	0,52209
NaN	NaN	NaN	NaN	-1,11135	1,80997	0,633207	NaN	NaN	NaN
0,0618229	0,408495	NaN	1,73039	0,475044	2,08029	-0,634428	NaN	NaN	NaN
2,1634	2,14819	2,31588	1,48508	2,62839	-0,137312	0,980925	1,87743	1,94529	2,33987
NaN	NaN	NaN	NaN	-0,73818	NaN	NaN	NaN	NaN	NaN

Polito et al. Universal ready-to-use immunotherapeutic approach for the treatment of cancer: expanded and activated polyclonal γδ memory T cells.
Phosphorylation sites

NaN	NaN	NaN	NaN	NaN	NaN	NaN	NaN	NaN	NaN
2,04702	1,96145	2,34504	2,1262	2,75197	0,989176	2,50329	2,05034	2,04522	1,9692
NaN	NaN	NaN	NaN	NaN	NaN	NaN	NaN	NaN	NaN
NaN	NaN	NaN	NaN	NaN	NaN	NaN	NaN	NaN	NaN
2,16713	2,23464	3,23502	3,61212	3,74333	2,26747	3,1895	3,2637	2,63937	3,62497
1,30964	1,61834	NaN	2,44057	3,05865	1,55665	2,20233	NaN	2,15493	2,9902
NaN	NaN	NaN	NaN	NaN	NaN	NaN	-2,4079	-3,38027	-2,99234
-1,89582	-0,0891762	-1,39097	-1,25622	0,593363	NaN	-0,661602	-1,10095	-0,99957	1,00823
-2,21943	-2,53263	-2,65985	-2,83488	-2,3683	-3,08701	-2,0082	NaN	NaN	NaN
NaN	NaN	NaN	-1,56291	NaN	-1,55684	-1,5729	NaN	NaN	NaN
NaN	0,957729	NaN	0,510984	0,232765	0,69339	NaN	NaN	-0,418124	-0,540568
0,175894	-0,212433	0,476585	0,417187	0,228451	1,16027	0,668222	0,0299911	-0,130595	-0,289826
-0,815144	-1,32382	-0,708242	-0,256882	-1,93173	0,580442	0,401731	-0,220705	-0,323596	-0,751275
0,165842	-1,23453	1,24632	-0,08745	0,671774	NaN	0,450052	0,0764332	-0,298306	-0,710599
1,74542	1,9302	1,7331	0,743404	1,92018	-0,446184	1,37988	1,71485	1,52946	1,5747
NaN	-0,00243187	NaN	-0,777	NaN	-2,65698	-1,08999	NaN	NaN	NaN
-1,13192	-0,934477	-0,703955	-0,493149	-1,09044	0,0880909	-0,85957	NaN	NaN	NaN
0,555149	-0,029604	0,311535	-0,512686	0,573935	NaN	0,376837	0,381994	0,708611	0,105268
1,21974	3,40326	1,223	0,667084	2,70233	NaN	2,23098	0,977476	0,232497	2,02901
0,663664	1,86237	2,00338	1,98228	2,67806	3,88722	2,84928	0,0730934	0,459367	3,98515
1,56528	2,38322	2,55174	2,83299	2,21217	2,93686	3,15092	2,07701	2,12002	2,80003
NaN	NaN	NaN	-2,71597	NaN	NaN	-1,9898	NaN	NaN	NaN
NaN	0,292196	0,454914	0,00779152	0,965689	NaN	0,653522	1,04274	-0,084939	0,651309
-1,03132	-0,219925	0,00571251	0,14784	NaN	0,427656	0,463308	1,03277	0,566421	0,193685
NaN	NaN	NaN	NaN	NaN	NaN	NaN	NaN	NaN	NaN
NaN	NaN	NaN	-2,25552	-2,78375	-1,98814	NaN	NaN	NaN	NaN
NaN	NaN	NaN	NaN	NaN	NaN	NaN	NaN	NaN	NaN
NaN	-1,60803	-2,62021	-2,14359	NaN	-1,08879	-3,24474	NaN	NaN	NaN
0,624691	0,590317	1,05178	1,24206	1,55931	0,509148	1,35196	2,2043	1,67843	1,42232
0,167969	NaN	-0,93552	-0,117805	NaN	0,468586	-1,88398	-0,647142	-0,871728	-0,616714
-0,467279	0,162634	-1,63147	-0,669622	0,555037	-0,150818	-1,61808	-1,51083	-1,26828	0,388747
-2,73112	-0,47353	-2,23361	NaN	NaN	NaN	NaN	-2,40935	-2,11427	-2,73816
NaN	NaN	NaN	NaN	NaN	NaN	NaN	NaN	NaN	NaN
-0,214247	-0,896339	-0,394056	NaN	-3,71081	-0,324522	0,699797	0,461708	0,113486	1,13098

Polito et al. Universal ready-to-use immunotherapeutic approach for the treatment of cancer: expanded and activated polyclonal γδ memory T cells.
Phosphorylation sites

NaN	NaN	NaN	NaN	NaN	NaN	NaN	NaN	NaN	NaN
1,88678	2,80182	2,81448	3,8324	3,27535	5,27166	4,45773	2,01548	1,93573	2,8157
1,88678	2,80182	2,81448	3,8324	3,27535	5,27166	4,45773	2,01548	1,93573	2,8157
-0,114584	-0,185751	-0,0921383	-0,501816	-0,1397	0,662758	0,480371	-0,223207	-0,468398	-0,153737
NaN	NaN	NaN	NaN	NaN	NaN	NaN	-1,37046	-1,96301	-1,62412
0,669991	2,34205	1,36283	1,38348	0,320374	1,58245	0,78371	1,18127	0,968171	1,15591
-1,65334	-1,25212	-1,57607	1,60644	-0,951056	1,23253	0,109072	-1,23808	-1,43029	-1,60452
2,28512	1,80258	2,33696	-0,0627403	1,62972	0,313299	-0,273756	2,83542	2,40379	3,53451
3,21631	2,77268	2,62483	3,76274	3,11083	3,83435	3,2815	2,77785	2,87862	2,56957
NaN	NaN	0,214764	NaN	NaN	NaN	-0,454746	-0,869522	-1,72393	-1,21084
3,38725	3,14312	2,67425	3,00701	2,98852	4,12851	3,3298	2,97966	2,62281	3,02417
NaN	NaN	NaN	NaN	NaN	-1,13671	NaN	NaN	NaN	NaN
NaN	NaN	NaN	-1,2032	-0,709536	NaN	-0,366331	NaN	NaN	NaN
-0,763002	-1,4932	-1,69518	-0,641935	-1,17618	NaN	-1,21255	NaN	NaN	NaN
-0,511076	-1,9299	-0,450928	-1,30131	0,286833	-2,43551	-0,550274	0,671719	0,749808	-0,260034
-1,30917	-1,9105	-0,80299	-0,86352	-0,605335	-1,45463	-0,355284	-0,55485	-0,757241	-0,856791
-0,9202	-1,96529	-1,5395	NaN	NaN	NaN	NaN	NaN	-1,02093	0,397153
-0,795912	-0,583616	-0,881021	-0,251312	-1,06833	0,132385	-0,217327	-0,669422	-0,92821	-1,03393
0,828461	NaN	1,4418	NaN	1,48406	NaN	NaN	-0,132511	0,0729418	0,150607
0,295164	1,24223	-3,50011	-1,05927	0,984535	NaN	-0,874897	NaN	NaN	NaN
NaN	-2,26952	-2,40859	0,0381851	-1,64707	0,790878	-0,406733	NaN	NaN	NaN
NaN	NaN	NaN	-1,3258	-1,39373	-0,27331	1,06803	NaN	NaN	NaN
-2,45209	NaN	0,385363	0,326485	-1,19924	-2,45503	1,06803	NaN	NaN	NaN
-0,0448399	-0,592937	NaN	-0,032814	-0,261404	1,15586	0,56554	-1,06398	-1,61275	-1,34519
0,44965	-0,196655	-0,51594	2,58012	2,09134	2,76779	2,05319	0,892159	-1,49882	0,548818
-1,7064	-1,54072	-0,593103	0,556067	0,117983	-1,6597	-0,606565	-0,611609	-1,03397	NaN
NaN	NaN	NaN	NaN	-2,18004	-2,82972	-2,09461	NaN	NaN	NaN
2,6365	3,62956	3,00261	4,23229	3,29246	5,87255	3,93065	2,38915	2,17296	2,5676
3,50047	1,00087	1,09041	2,895	1,93877	-0,106667	2,54804	2,79077	0,949389	2,16827
-0,558643	-0,827269	-0,600668	0,80772	-0,766775	-0,438532	0,371319	-0,664907	-0,212775	-0,503139
3,94662	2,50921	4,71105	4,32545	4,2625	3,53158	4,53418	4,31101	3,94883	3,94183
-1,40954	0,573561	-1,48384	0,357462	NaN	0,725126	1,10143	-2,83405	-0,928678	-2,34998
-0,138069	NaN	2,75343	2,92152	1,85156	3,16484	3,05883	1,91304	1,57965	1,59611
4,29714	3,22762	5,0521	5,06137	5,13469	5,33876	4,50278	4,4999	4,2946	4,33721

Polito et al. Universal ready-to-use immunotherapeutic approach for the treatment of cancer: expanded and activated polyclonal γδ memory T cells.
Phosphorylation sites

-0,984505	-2,74554	-1,3483	0,413742	-0,567722	-1,21608	0,517361	-1,12768	-1,04045	-0,0148668
0,336319	-0,14884	-0,374002	1,32965	0,788914	1,49101	-0,21777	0,0831833	-0,224492	-0,474158
0,665495	0,295628	0,193605	2,13116	0,932123	1,0882	0,0382175	-0,278648	0,0686255	-0,842525
NaN	-2,85975	-2,12625	-2,21955	-2,27965	-1,57329	-1,91998	-2,27762	-2,15855	NaN
NaN	0,229668	NaN	-0,750904	NaN	-0,24382	NaN	NaN	NaN	NaN
NaN	0,404852	NaN	-0,0184441	NaN	0,740198	-0,673565	NaN	NaN	NaN
NaN	NaN	NaN	NaN	NaN	NaN	NaN	NaN	NaN	NaN
-0,714144	-0,556717	-1,17629	-0,388403	-1,1151	1,29739	-0,215458	-0,909069	-1,25109	-1,10902
NaN	NaN	-1,96257	NaN	-0,840975	NaN	-1,90149	-0,803606	-0,241994	-0,335485
0,754845	0,79002	0,611086	2,35247	1,04848	0,64838	2,25117	0,815714	1,20009	0,505439
-1,26511	NaN	NaN	-0,82312	-1,34337	NaN	-0,537407	-1,76562	-1,81322	-2,32746
NaN	NaN	NaN	NaN	NaN	NaN	NaN	NaN	NaN	NaN
NaN	NaN	NaN	-0,92914	-3,1722	0,0657082	NaN	NaN	NaN	NaN
3,29866	3,35432	2,7917	4,15288	4,81753	5,22074	4,99282	3,57848	3,40236	4,03269
NaN	NaN	-0,964304	-0,639299	0,767628	0,0353928	-0,406614	-0,653917	-1,29702	-0,35106
2,21301	1,94144	1,22109	1,26235	2,01292	2,33367	2,20228	2,30902	1,47339	1,72998
-1,75877	-0,971357	-0,253733	0,0222359	0,0626488	0,972843	-0,0302372	-1,22601	-1,26591	-0,979163
4,87805	3,9895	4,7221	2,76731	5,4172	NaN	4,13009	4,5221	3,97411	4,7379
NaN	-0,0982533	NaN	1,03589	1,33323	1,1314	0,633783	0,721609	0,317981	1,25674
5,88615	4,41959	5,87742	5,27754	5,38932	NaN	5,6663	5,0934	5,01032	5,18062
NaN	-0,182848	-3,11667	0,265108	NaN	NaN	0	NaN	NaN	NaN
-0,44059	1,82148	1,42926	0,752272	3,54868	NaN	2,75248	-0,158722	-0,726409	2,66984
0,970209	1,12384	3,22438	2,75581	2,38575	3,25271	-3,73591	1,37595	1,75687	1,46327
2,39151	0,715321	2,38178	NaN	3,77975	4,81081	4,18985	3,19841	4,81425	NaN
-1,60345	-2,20561	-2,67358	-2,00706	-1,47494	-2,44775	-2,71633	-2,8427	-3,44974	-2,58422
-1,32257	-2,15936	-1,55973	NaN	-1,52586	NaN	-2,2882	-1,46887	NaN	NaN
0,611691	-0,492298	0,327585	-0,148895	0,119764	-0,0810871	-0,282726	-0,253216	-0,423394	-0,906472
NaN	NaN	NaN	NaN	NaN	NaN	NaN	NaN	NaN	NaN
-2,1542	-2,00114	-1,85361	-0,714766	-1,64618	0,107685	-1,07499	NaN	NaN	NaN
NaN	NaN	NaN	-2,05155	-3,33573	NaN	-2,37005	NaN	NaN	NaN
NaN	NaN	NaN	-0,188965	-1,37665	NaN	NaN	NaN	NaN	NaN
1,06746	2,60389	0,804321	0,193773	0,429979	-0,897305	1,57821	0,832479	1,02067	0,812531
3,63539	3,89654	-0,387859	3,51137	3,22479	2,651	-0,329275	3,10279	-1,83011	3,67723
NaN	NaN	NaN	0,91836	-0,251589	NaN	0,22263	-0,170269	0,000594139	0,440515

Polito et al. Universal ready-to-use immunotherapeutic approach for the treatment of cancer: expanded and activated polyclonal γδ memory T cells.
Phosphorylation sites

NaN	-0,0661755	NaN	-0,981098	-0,257217	NaN	-0,362219	-1,33203	-1,2266	-0,862454
NaN	NaN	NaN	NaN	NaN	NaN	NaN	NaN	NaN	NaN
0,139072	2,68359	0,196253	1,11839	1,33447	3,0464	0,367426	-0,341446	-0,74097	0,348849
1,00299	0,243681	0,513582	0,034977	0,407948	0,627277	-0,0341682	1,11119	0,267573	-0,0532598
NaN	NaN	NaN	NaN	-1,44189	NaN	NaN	NaN	NaN	NaN
3,08592	1,70289	1,62015	1,56771	1,90591	NaN	1,57859	1,34722	1,7083	1,22587
-0,444836	-0,431599	0,388815	-0,564646	NaN	0,0562897	-0,50419	-0,584688	-0,29493	-0,312789
2,15956	1,96764	2,50455	0,333504	2,10806	0,836756	1,32804	1,26565	1,16439	1,65713
-1,55693	-0,0794964	-1,69367	-2,11344	-1,25325	-3,68161	-1,20625	-0,93368	0,0330286	-0,886054
NaN	NaN	NaN	-1,1207	NaN	0,189512	-0,342703	NaN	NaN	NaN
0,736473	1,96517	1,20867	1,26816	1,04572	-0,466408	1,30597	1,9426	1,69335	1,93594
0,964157	NaN	1,17874	1,08879	1,76269	2,50986	NaN	NaN	NaN	NaN
-1,01156	0,701689	-1,30796	-1,0115	-0,232456	-2,19656	-1,16043	-0,3272	-0,310922	-0,364116
-1,9919	-2,48075	NaN	-1,67384	-0,764601	-1,56026	-1,79762	NaN	NaN	NaN
NaN	NaN	NaN	-1,27194	-1,50666	-2,17632	NaN	NaN	NaN	NaN
3,35212	3,6386	3,03729	3,77366	3,48368	4,45928	3,84971	2,91188	3,83502	3,22646
-1,32273	-1,02541	-1,48485	-0,71401	-0,494875	-1,58428	0,698421	-0,275414	-0,270308	-0,172727
-0,0376282	0,0829239	0,767815	2,03284	1,08994	0,180552	2,02552	1,00172	1,03675	0,855906
NaN	NaN	NaN	-2,55133	NaN	-1,54012	NaN	NaN	NaN	NaN
-0,423994	2,92249	1,13646	1,0546	2,17984	-0,502451	1,92441	-0,0359879	-0,492837	1,93191
NaN	NaN	NaN	1,09173	-1,09098	1,64455	-0,592916	NaN	NaN	NaN
2,19172	2,03172	2,12285	3,10264	2,90762	-1,77022	2,28403	1,77118	1,87993	3,07113
-2,78434	-3,00852	0,0792027	-0,447914	-2,13152	NaN	-2,60994	-0,501616	-0,654721	-0,501708
NaN	NaN	-0,632847	-0,113842	-3,06374	0,343363	-0,178293	NaN	NaN	NaN
NaN	NaN	NaN	NaN	NaN	NaN	NaN	-2,03486	-2,76468	-2,33682
NaN	NaN	NaN	NaN	NaN	NaN	NaN	NaN	NaN	NaN
NaN	NaN	NaN	-2,33028	NaN	-1,72025	-2,88192	-2,36307	NaN	-2,56033
NaN	NaN	NaN	-0,0310478	-2,09656	-1,30984	-0,196692	NaN	NaN	NaN
-0,209671	-0,710581	0,501369	1,70749	NaN	1,08736	1,29153	-0,704315	-0,516229	-0,164292
NaN	NaN	NaN	-1,05014	NaN	NaN	-0,861071	-1,85835	-2,34373	-2,20495
NaN	NaN	NaN	-2,05812	NaN	-1,37322	-1,48614	NaN	NaN	NaN
2,80824	2,44826	2,05412	0,781012	2,06127	1,08927	1,3183	2,71591	2,67159	2,85939
-1,76376	-1,92913	0,754683	1,24787	0,519419	3,66576	1,57289	-0,798521	-0,987199	-0,453145
NaN	NaN	NaN	-1,02367	-1,98329	-0,424374	-1,02652	NaN	NaN	NaN

Polito et al. Universal ready-to-use immunotherapeutic approach for the treatment of cancer: expanded and activated polyclonal γδ memory T cells.
Phosphorylation sites

NaN	NaN	NaN	-1,01398	-2,72319	-1,87573	-1,28449	NaN	NaN	NaN
NaN	-3,34847	-1,91599	-1,80701	-1,61728	NaN	-2,00488	-1,66578	-2,2933	-2,23846
NaN	NaN	NaN	-0,198172	NaN	0,985064	-0,211494	NaN	NaN	NaN
0,535664	1,5856	-0,396065	1,78742	1,21193	1,23426	1,43133	1,11499	1,38719	1,52724
NaN	-2,82059	-0,281006	-0,112509	NaN	1,11788	-0,0839157	NaN	NaN	NaN
NaN	-1,12859	NaN	NaN	-1,39142	-1,61938	-1,75831	NaN	NaN	-0,834971
-0,427261	0,106556	-0,48963	-1,88336	-2,06958	-3,89098	-2,69083	NaN	-1,2063	-0,683183
NaN	NaN	NaN	-1,2445	NaN	0,0956249	-1,93195	NaN	-1,44417	NaN
-2,26613	NaN	-0,622366	-2,02408	NaN	-1,20637	-1,77994	NaN	NaN	NaN
0,960262	0,945406	0,890884	1,07394	0,959328	1,59321	1,60498	1,39191	1,21589	0,802434
NaN	NaN	0,307362	-2,27763	NaN	-0,393887	0,742899	NaN	NaN	NaN
2,39436	1,83027	2,07314	2,79641	3,11119	3,16658	2,29358	1,96954	1,87714	1,53193
NaN	NaN	NaN	-0,863922	NaN	0,689903	NaN	-0,615654	0,112102	-2,79582
-1,7873	-1,24613	-1,98858	0,822348	0,0414219	0,882	0,140268	-1,58446	-1,87823	-0,833989
NaN	-1,70943	-0,2992	-1,47873	NaN	-0,222971	NaN	0,110771	-0,0940828	0,492448
1,21041	1,53827	1,56304	0,568392	NaN	NaN	0,365068	NaN	NaN	1,24942
NaN	NaN	NaN	-3,24618	-3,21048	-1,51798	-3,25629	NaN	NaN	NaN
1,60647	0,600216	2,30819	0,699253	1,73392	1,35713	-0,515135	2,25252	1,91581	1,32602
NaN	NaN	NaN	NaN	NaN	NaN	NaN	NaN	NaN	NaN
0,465982	-0,231255	1,33335	-1,6806	1,68108	-1,97145	-0,608608	0,353559	0,498551	0,942758
0,62114	-0,259546	-0,522501	0,826086	0,238079	0,981426	0,75102	NaN	NaN	NaN
2,4312	1,61616	1,75078	1,21269	2,49927	NaN	1,97826	1,6502	1,34332	3,14765
NaN	NaN	NaN	NaN	NaN	NaN	NaN	2,45241	2,51772	2,61144
NaN	NaN	NaN	NaN	NaN	NaN	NaN	NaN	NaN	NaN
-1,46743	-2,10514	-1,68485	-2,4852	-0,339083	-1,34421	-1,59393	-0,890778	-1,14869	-1,10709
-1,46743	-2,10514	-1,68485	-2,4852	-0,339083	-1,34421	-1,59393	-0,890778	-1,14869	-1,10709
1,92043	2,75903	1,53113	NaN	1,59711	NaN	0,139498	2,40355	2,42827	2,23282
1,31369	1,57309	1,58094	NaN	1,26837	NaN	-0,427441	1,75693	1,93974	1,90066
-0,258738	-1,09682	-1,02284	-1,16017	-0,342251	NaN	-0,298002	0,125776	-0,467576	-0,781489
NaN	NaN	NaN	NaN	NaN	NaN	NaN	NaN	NaN	NaN
-0,573547	0,411777	-0,315435	0,391773	-0,587154	0,305079	0,614763	-1,05721	-0,695523	NaN
-0,82464	-0,612263	-0,306101	NaN	NaN	NaN	NaN	NaN	NaN	NaN
NaN	NaN	NaN	1,56994	NaN	1,48079	1,02957	NaN	NaN	NaN
0,951517	0,227539	-0,844444	2,46015	1,35418	2,69053	1,09349	-0,629633	-0,347611	-0,0188608

Polito et al. Universal ready-to-use immunotherapeutic approach for the treatment of cancer: expanded and activated polyclonal γδ memory T cells.
Phosphorylation sites

NaN	-1,52615	NaN	-2,03165	NaN	-3,77202	-3,09137	NaN	NaN	NaN
0,106998	1,03966	NaN	0,749868	0,106436	0,958609	0,523031	NaN	NaN	NaN
1,95204	-0,208288	0,53945	NaN	1,60526	NaN	NaN	1,08391	0,566142	0,186442
1,99091	1,98534	0,895792	0,549299	2,4471	1,53564	1,38519	-0,0437069	-0,0380487	0,33212
NaN	NaN	NaN	-0,611818	NaN	NaN	-1,71768	NaN	NaN	NaN
NaN	NaN	NaN	-2,6653	0,070116	-3,94619	-3,81944	NaN	NaN	NaN
0,890699	3,03732	1,44367	1,98985	1,33627	3,48386	2,46447	1,07601	0,957332	2,13443
NaN	NaN	0,927231	1,54496	NaN	2,05496	1,06664	-1,90243	-2,62023	-1,2207
0,843216	1,42199	0,266659	1,94872	0,59536	3,34688	2,391	0,0922661	1,0246	0,346885
NaN	NaN	NaN	NaN	-1,98397	0,075182	-0,708778	NaN	NaN	NaN
NaN	NaN	NaN	-0,774639	NaN	-1,08652	-0,951351	NaN	NaN	NaN
NaN	NaN	NaN	0,553564	-1,27848	-0,116116	NaN	NaN	NaN	NaN
NaN	NaN	NaN	NaN	NaN	NaN	NaN	NaN	NaN	NaN
4,53186	3,29892	3,61329	3,96013	4,17475	3,57938	4,47502	4,27501	4,1998	3,69354
0,930357	2,16826	NaN	1,18256	2,33334	-0,0393581	1,10849	-0,260876	-0,551353	0,277694
0,522594	-0,0110569	-0,869745	0,0173416	0,370829	-1,54644	-0,707647	-0,606398	-0,51946	-0,746417
1,04783	1,52599	1,19753	2,55507	0,924429	3,05417	2,84032	1,40355	1,37838	2,85667
0,465658	-0,451508	0,589132	-0,841877	0,195509	-1,53403	-0,530869	-0,792202	-0,965369	-0,943463
-1,8849	NaN	-0,909605	-1,12968	-1,71293	-1,22712	NaN	-0,467588	-0,704486	-1,04091
2,01441	0,113226	1,75619	1,06676	0,324467	1,54388	NaN	2,51702	1,94481	0,183049
NaN	NaN	NaN	-0,796057	-2,19026	-0,760931	-0,981834	NaN	NaN	NaN
4,02589	3,75382	4,73894	4,88118	4,31392	4,43089	5,27085	4,43657	4,21091	4,45741
NaN	NaN	-0,17256	0,149548	NaN	-0,46813	-1,82143	NaN	NaN	NaN
0,849672	2,82273	0,508196	NaN	-2,82407	NaN	1,42485	NaN	1,29441	0,823596
NaN	NaN	-1,41129	0,552311	NaN	1,78209	0,0256939	NaN	NaN	NaN
0,513788	-0,589512	-0,859863	-1,129	-0,0752563	NaN	-0,59339	-0,459507	-0,872853	-0,923215
NaN	NaN	NaN	NaN	NaN	NaN	NaN	NaN	NaN	NaN
NaN	NaN	-3,9714	NaN	NaN	NaN	NaN	-2,26402	-2,26128	-2,82686
NaN	NaN	NaN	-1,49937	NaN	-1,51939	-1,15295	NaN	NaN	NaN
NaN	NaN	NaN	-0,875746	NaN	-0,573734	-1,77756	NaN	NaN	NaN
-2,15738	NaN	NaN	-2,40825	-1,31381	NaN	-2,3834	-3,21426	-2,75274	-2,99816
-1,84482	-1,48991	0,756315	2,44088	NaN	2,57174	2,52853	-0,861389	-0,545415	-0,483914
NaN	NaN	NaN	0,546408	NaN	1,53754	0,744766	NaN	NaN	NaN
-3,15633	NaN	-1,3611	-0,0660267	-1,9234	-0,155882	-0,36495	NaN	NaN	NaN

Polito et al. Universal ready-to-use immunotherapeutic approach for the treatment of cancer: expanded and activated polyclonal γδ memory T cells.
Phosphorylation sites

NaN	NaN	NaN	-1,05151	-0,846434	NaN	-0,406673	NaN	NaN	NaN
-0,271936	NaN	0,768312	2,58031	-0,914474	2,63981	3,01392	0,667517	-0,214368	0,353078
NaN	NaN	-0,939579	-0,962852	-2,57794	-0,718292	-1,02361	-0,835342	-0,872928	0,0467958
0,0417957	0,667768	NaN	0,188784	0,159458	0,568209	1,314	0,836676	1,15171	1,05183
NaN	NaN	NaN	NaN	NaN	NaN	NaN	NaN	NaN	NaN
NaN	NaN	NaN	NaN	NaN	1,12493	NaN	NaN	NaN	NaN
NaN	NaN	1,80487	NaN	NaN	2,05107	2,42194	1,07541	NaN	NaN
-2,0225	-1,92972	-0,790983	-1,96353	0,0517178	-1,43069	-1,65546	-2,6729	-1,39378	-1,62689
-0,241457	-0,349157	0,613998	1,68045	-0,0911903	1,55289	2,30561	0,795019	0,527253	0,390447
NaN	NaN	-1,81416	0,814217	0,651192	NaN	0,326775	NaN	NaN	NaN
1,11934	0,666101	-0,253708	0,491636	0,532509	-0,219339	0,698971	-0,0712452	-0,217393	0,0833616
0,563238	-0,897791	2,28415	NaN	NaN	NaN	NaN	NaN	NaN	NaN
-1,44538	-2,26442	-1,2022	-1,08384	-0,234629	-1,80669	-0,883942	-0,689274	-0,929225	-0,758323
1,96804	0,743603	3,12916	3,31583	0,932539	2,63875	3,58652	1,56889	1,25285	1,5021
2,62536	2,31989	2,48553	1,72534	2,84049	1,02306	2,62193	1,79782	1,87681	2,13537
NaN	NaN	NaN	NaN	NaN	NaN	NaN	-0,164888	0,325284	NaN
NaN	0,630116	NaN	-2,7817	0,538923	NaN	NaN	NaN	NaN	NaN
-0,00107193	-0,885853	0,603851	-0,0325699	0,726309	0,0740433	0,790316	0,0670586	0,331344	-0,13754
NaN	NaN	NaN	NaN	NaN	NaN	NaN	-1,93125	-0,559764	-0,767123
-0,394941	0,609842	0,0869255	-0,345123	0,952673	-1,15652	0,139519	0,051836	0,468141	-0,253972
-1,71275	NaN	NaN	-1,91019	-1,93444	-3,26575	NaN	-3,07506	-2,93978	-2,4374
NaN	NaN	NaN	-1,95931	NaN	NaN	NaN	NaN	NaN	NaN
0,877153	0,17745	1,01962	0,874214	-1,67703	1,07808	0,535944	0,589983	0,31956	0,305963
-1,84635	-0,71912	NaN	-0,401989	NaN	-1,14288	-1,04794	NaN	NaN	NaN
2,51805	2,34613	1,61739	1,71714	1,66018	1,68018	1,26425	1,6904	1,11193	1,55391
NaN	NaN	NaN	NaN	1,55506	NaN	1,3961	NaN	NaN	1,86721
0,193996	-0,376907	NaN	0,481138	0,657118	0,226444	0,693731	NaN	NaN	NaN
-1,06585	NaN	NaN	-2,2707	-1,8661	-1,86137	-1,94205	NaN	NaN	NaN
0,181042	0,537416	0,207586	-0,377855	0,783737	NaN	0,320864	0,142187	-0,152789	-0,0029974
0,559259	0,539883	0,0664444	0,582579	1,16933	-0,172262	1,05851	0,412579	-0,0210104	0,478498
NaN	-0,470247	NaN	NaN	0,082737	NaN	-0,0301914	-1,64785	NaN	-0,733653
0,112087	-0,279261	1,53377	0,667664	1,53596	-0,473795	0,736073	0,419552	0,480615	1,04283
NaN	NaN	NaN	NaN	NaN	NaN	NaN	-2,6729	-1,3761	-2,45465
NaN	NaN	NaN	NaN	NaN	NaN	NaN	1,60361	2,4106	1,41589

0,597408	-0,378689	1,65462	-1,13524	1,225	0,148045	0,448841	-0,0427303	-0,586368	-0,467481
1,01949	0,562948	0,808683	2,09689	1,07085	2,13536	1,73755	0,0144939	0,227654	2,0372
2,40767	1,96305	1,32136	1,93013	0,97031	2,78267	2,01516	1,0501	1,76431	1,18012
2,98528	2,62729	2,66656	2,94646	3,14421	1,21565	3,32438	2,84781	2,51762	4,09593
-0,654711	0,237219	NaN	-0,0764179	-0,331156	-0,290648	-0,312084	NaN	NaN	NaN
-0,927399	-2,08339	-1,32459	-0,424757	-0,68195	-0,0954838	-0,913774	-0,425007	-1,19851	-1,07203
NaN	NaN	NaN	NaN	NaN	NaN	NaN	NaN	NaN	-1,25266
NaN	NaN	NaN	-3,3772	NaN	-0,314144	-4,0917	NaN	NaN	NaN
NaN	NaN	NaN	-0,0505047	1,01482	0,457983	1,30118	NaN	-0,588033	0,114921
NaN	NaN	NaN	-1,73512	-3,12975	-2,26293	-1,87527	NaN	NaN	NaN
-2,35617	-1,906	-2,70461	-0,380436	-1,32609	-2,52083	-0,290085	NaN	NaN	NaN
-0,489294	-0,955078	-0,179241	-0,342113	0,409382	-0,190334	-0,335297	-0,538925	0,475299	0,231412
NaN	0,672663	0,326132	-0,880545	1,06909	NaN	0,602804	-0,347494	-0,789525	1,50121
1,77259	0,843349	2,66258	1,7121	1,78868	3,13664	1,56951	2,36935	2,55927	2,34331
0,319639	-0,0384884	NaN	-1,84244	0,945841	-1,41712	-1,5969	1,15082	1,02188	0,729707
-1,43153	-0,456671	-1,36164	-0,321524	NaN	0,0867367	-0,997364	0,206049	NaN	-1,45541
2,07224	2,26283	2,05239	1,8173	1,65405	2,69903	0,746099	2,48025	2,46357	2,23944
-3,37182	NaN	-1,23965	NaN	-2,88324	-1,26244	-2,52118	-2,23191	-1,34441	-2,44638
-1,40765	-0,181435	-1,22214	-2,28693	0,538727	NaN	-3,3394	-0,925413	-1,27016	0,157311
NaN	NaN	NaN	NaN	NaN	NaN	NaN	NaN	NaN	NaN
NaN	NaN	NaN	NaN	-2,09936	-0,194305	-0,143492	NaN	NaN	NaN
NaN	NaN	NaN	-1,09538	NaN	0,147394	-1,06397	NaN	NaN	NaN
NaN	-1,59216	-0,453798	-0,00881195	0,449745	0,116667	-0,554863	-1,03706	-1,11538	-0,465514
NaN	NaN	NaN	NaN	NaN	NaN	NaN	NaN	NaN	NaN
-1,9635	NaN	-0,0272598	0,627653	NaN	0,605547	0,798306	-0,30691	-0,568679	-1,16088
-2,33902	-2,34848	-1,80377	NaN	-1,03502	NaN	-1,97778	-2,15525	-1,23155	-1,35578
NaN	NaN	NaN	NaN	NaN	NaN	NaN	NaN	NaN	NaN
NaN	NaN	NaN	NaN	NaN	NaN	NaN	NaN	NaN	NaN
NaN	NaN	NaN	NaN	NaN	-1,58187	NaN	NaN	NaN	NaN
NaN	NaN	NaN	NaN	NaN	NaN	NaN	NaN	NaN	NaN
NaN	1,9055	0,678062	0,846983	1,13828	1,15748	0,34429	-0,352646	0,392837	0,07129
NaN	NaN	NaN	-2,37668	NaN	-2,67424	NaN	NaN	NaN	NaN
NaN	-0,691788	NaN	-1,17093	-0,928747	NaN	-0,35009	-1,21288	-0,728004	-0,852269
NaN	NaN	NaN	-2,18453	NaN	NaN	-0,245743	NaN	NaN	NaN

Polito et al. Universal ready-to-use immunotherapeutic approach for the treatment of cancer: expanded and activated polyclonal γδ memory T cells.
Phosphorylation sites

2,4212	2,34076	2,36283	2,15079	2,71728	1,8397	2,39666	2,62005	2,64426	2,65472
-0,913877	-3,01102	-1,19748	NaN	NaN	NaN	NaN	NaN	NaN	NaN
NaN	NaN	NaN	NaN	NaN	NaN	NaN	NaN	NaN	NaN
-2,33405	NaN	NaN	0,22814	NaN	NaN	0,772112	-1,51026	NaN	NaN
NaN	NaN	NaN	-0,796343	-1,87066	NaN	-1,51408	NaN	NaN	NaN
NaN	NaN	NaN	NaN	NaN	NaN	NaN	NaN	NaN	NaN
1,40906	1,39553	0,46513	1,88067	2,06461	-1,22611	0,568707	1,44738	0,994761	NaN
NaN	NaN	NaN	NaN	NaN	NaN	NaN	NaN	NaN	NaN
NaN	NaN	NaN	NaN	NaN	NaN	NaN	NaN	NaN	NaN
NaN	NaN	NaN	NaN	-2,37709	0,735815	-0,670609	NaN	NaN	NaN
1,30405	0,93265	1,36151	1,05707	1,91454	NaN	1,58344	1,76598	1,32297	1,24071
2,05268	1,38965	1,54761	1,09328	1,81817	NaN	1,73829	1,7648	1,70169	0,956542
1,06948	NaN	-0,00471306	0,456701	0,395508	0,436272	0,991978	1,08802	NaN	NaN
NaN	NaN	NaN	-0,995796	NaN	-1,13586	-1,48689	-0,417192	-0,194612	-2,22466
-1,11921	-0,252644	-0,182486	0,18042	0,785255	-0,664953	0,790575	0,221085	0,0970335	0,0592051
-0,04809	-0,635986	-0,913313	-0,5156	-0,19256	-0,252645	0,0716057	-0,0709839	0,329501	-0,71259
NaN	NaN	NaN	-2,91774	-2,64474	NaN	-1,31436	-2,68419	-2,60593	-2,76451
-0,565332	-1,39259	-0,636993	-0,419659	-1,14152	0,0781364	-1,14147	-0,942713	-1,26823	NaN
-1,09798	-0,464174	-0,393528	-0,712534	-0,869625	NaN	NaN	NaN	NaN	NaN
1,01574	-1,21061	-0,671654	NaN	-0,881153	NaN	NaN	-0,691206	-1,00957	-1,22359
-2,19644	-0,563608	NaN	-0,674513	-0,62318	-1,46056	0,27062	NaN	NaN	NaN
1,7084	1,56126	1,79574	1,94376	2,05882	2,20706	1,77388	1,79574	2,11856	1,86053
NaN	NaN	NaN	0,322271	NaN	-0,0091362	-1,51108	NaN	NaN	NaN
0,534832	1,23927	0,290148	0,65954	0,734552	0,371424	0,591475	0,246473	0,706351	0,521338
-3,36778	-1,23244	-2,91549	-2,1243	-3,24051	-2,54889	-3,00233	NaN	NaN	NaN
NaN	NaN	NaN	-1,30131	NaN	NaN	-2,21707	NaN	NaN	NaN
2,6737	2,8121	2,78465	3,0816	2,69533	2,50305	2,65473	2,70853	3,08705	3,04453
0,297672	2,06225	0,113012	-0,026041	-0,577915	1,79751	-0,987185	-0,678896	0,713874	NaN
-0,547314	-0,544487	0,682295	0,553564	-0,817635	0,231251	-0,257421	-0,180182	0,187049	-0,419484
1,29753	1,15342	1,56856	1,34492	0,98884	NaN	1,86104	1,23524	0,999999	0,773057
2,71137	2,32475	2,4664	2,24508	1,43464	2,60753	2,28508	2,3665	2,02944	2,16582
NaN	NaN	NaN	-1,64544	NaN	-0,995333	-2,79594	NaN	NaN	NaN
-1,52684	NaN	-1,0847	-0,953281	-1,67694	-1,60465	-1,72129	NaN	NaN	NaN
NaN	NaN	NaN	-1,24755	-1,83848	-0,50079	-0,859894	NaN	NaN	NaN

Polito et al. Universal ready-to-use immunotherapeutic approach for the treatment of cancer: expanded and activated polyclonal γδ memory T cells.
Phosphorylation sites

-2,53413	NaN	-1,42557	-1,3666	-1,10431	-0,889891	-1,74949	-2,69664	-2,28188	-2,03037
1,41644	0,0960426	0,176882	1,89922	0,273933	2,22232	1,75341	1,19452	1,16485	1,18285
2,25636	0,970665	0,75782	1,77597	1,69792	2,4766	1,84732	2,66805	2,35868	2,39414
NaN	NaN	NaN	-1,00492	NaN	-0,983557	-0,781244	NaN	NaN	NaN
-0,481171	-0,867887	NaN	0,811077	-0,685513	1,68782	0,0426941	-0,196466	-0,250875	NaN
0,149513	-1,10635	-1,79882	0,0638428	-0,310452	0,220388	-2,11732	-1,13912	-1,10067	-0,981835
-0,35903	-0,22868	-0,533903	0,411947	0,543301	0,621136	-0,0480328	1,05275	1,26757	1,04005
NaN	NaN	NaN	NaN	2,01081	NaN	NaN	NaN	2,58007	NaN
2,02559	1,70063	1,14021	2,23664	-0,91721	2,43028	2,22688	2,70719	-0,437768	2,71706
-2,93456	NaN	-0,362812	0,0774784	-3,25433	0,438662	-0,275377	NaN	NaN	NaN
-2,12898	-3,46828	-0,600122	-0,284288	-1,65269	0,674519	-1,76406	-0,492744	-0,753329	-1,03987
2,28828	1,8105	1,89966	2,97152	2,34788	2,30314	2,83438	1,96558	2,14972	1,82217
-1,80139	NaN	-2,00993	NaN	NaN	NaN	NaN	-0,839844	-1,25873	-0,634881
0,323231	1,32689	NaN	NaN	0,145927	-0,300901	NaN	0,399931	NaN	0,460248
0,572874	1,13289	0,70977	0,572971	1,23095	-0,69943	0,369246	1,05443	0,67702	1,17046
0,0512409	0,0503178	-0,353275	0,443514	0,147829	0,00311279	0,339441	0,16939	0,144284	-0,331754
NaN	NaN	NaN	NaN	NaN	-1,98116	-2,40029	NaN	NaN	NaN
NaN	NaN	NaN	NaN	NaN	NaN	NaN	NaN	NaN	NaN
-1,00869	-0,464991	-0,47674	-1,11066	NaN	0,297688	-1,01257	NaN	NaN	NaN
NaN	NaN	NaN	0,173965	-0,742884	2,00837	-0,462177	NaN	NaN	NaN
NaN	NaN	NaN	-2,33167	NaN	-1,56196	-1,78025	NaN	NaN	NaN
0,746088	0,140051	0,336863	1,29411	1,56959	-0,65358	0,953838	0,752762	0,85049	1,07805
NaN	NaN	NaN	0,270422	NaN	0,420242	NaN	NaN	NaN	NaN
-1,12392	-0,101925	-1,13284	-0,134134	-0,490993	-0,187468	0,0705414	-1,09625	NaN	-0,874228
0,956472	1,942	1,33234	0,983864	0,830278	1,17648	1,07748	0,474134	0,538875	-0,00398731
NaN	NaN	NaN	-3,4293	-2,37796	NaN	NaN	-2,1401	-2,90022	-2,90412
-0,0545883	0,753153	0,295389	0,14455	0,655802	-0,330732	0,245543	0,181032	0,00983143	-0,0412378
NaN	-0,920729	-1,11813	0,197161	NaN	0,419525	0,316565	-0,57678	-0,810954	0,325616
-1,21414	-0,825529	-2,49975	-1,71314	-1,35343	-1,39761	NaN	NaN	NaN	NaN
-0,637976	0,258081	-0,359354	-0,242979	0,426451	NaN	0,665407	-0,0405464	-0,297601	-0,147265
NaN	NaN	NaN	-1,56003	NaN	NaN	-1,51977	NaN	NaN	NaN
4,251	4,06106	4,09732	3,67896	3,57755	3,28035	2,96361	3,61621	3,61631	3,57227
NaN	-1,82087	-2,03222	-1,3356	-0,76939	-1,64974	-1,04342	NaN	NaN	NaN
NaN	NaN	0,0677395	0,577784	-1,90731	1,07819	0,15168	NaN	NaN	NaN

Polito et al. Universal ready-to-use immunotherapeutic approach for the treatment of cancer: expanded and activated polyclonal γδ memory T cells.
Phosphorylation sites

3,48785	2,73218	NaN	3,30856	3,35515	3,28478	NaN	3,19209	2,80757	3,01866
NaN	NaN	NaN	NaN	NaN	NaN	NaN	NaN	NaN	NaN
NaN	NaN	NaN	NaN	NaN	NaN	NaN	-0,50905	-0,833728	-1,17056
NaN	NaN	NaN	NaN	NaN	NaN	NaN	-1,92432	NaN	-2,4228
NaN	NaN	NaN	NaN	NaN	NaN	NaN	NaN	NaN	NaN
NaN	NaN	-1,92586	-2,02303	-2,50844	-1,86294	-2,64194	NaN	NaN	NaN
3,39879	2,5443	2,83859	1,65377	0,91511	2,28331	1,03994	2,40011	2,68709	2,60975
-3,51522	NaN	-1,51676	-2,39736	-2,38634	-0,772224	-1,3258	NaN	-1,96069	-2,01498
NaN	NaN	NaN	NaN	NaN	0,583004	NaN	NaN	NaN	NaN
NaN	NaN	NaN	NaN	NaN	NaN	NaN	NaN	NaN	NaN
NaN	NaN	NaN	NaN	NaN	NaN	NaN	NaN	NaN	NaN
3,43373	4,30809	3,45908	3,8501	4,18704	3,38603	4,43962	3,53469	3,78413	3,56211
NaN	NaN	NaN	NaN	NaN	NaN	NaN	NaN	NaN	NaN
0,17267	-0,653326	0,347197	-1,55122	0,746634	-0,893805	-0,37384	0,303307	0,281341	0,191354
NaN	NaN	NaN	NaN	NaN	NaN	NaN	NaN	NaN	NaN
-0,396822	0,0220451	0,971142	-0,955883	0,959312	-0,890335	-0,215044	0,130819	-0,142091	-0,0255022
-1,8429	-2,27117	-0,847034	-3,58409	-1,40935	NaN	-1,79127	-2,00823	-1,82255	-1,9284
NaN	NaN	NaN	NaN	NaN	NaN	NaN	NaN	NaN	NaN
-0,391834	NaN	-0,674189	0,237402	-0,622086	0,0945015	0,156761	-0,537079	-1,00994	-0,877513
-0,861961	-1,00058	-1,42096	-0,381323	-0,611101	0,780451	-0,513378	-0,68594	-0,844367	-1,42878
-1,45392	-1,4193	-0,528267	-1,18186	-0,625942	NaN	-1,47014	-0,596897	-0,574012	-0,654759
NaN	NaN	-0,807953	-1,91344	-2,29422	-2,46135	-1,07975	NaN	NaN	NaN
NaN	NaN	NaN	-2,88342	NaN	-1,00571	NaN	NaN	NaN	NaN
1,0175	0,675495	1,9059	1,39065	0,757202	1,60944	0,689857	1,38352	1,38129	0,826703
NaN	NaN	-0,934223	-1,024	NaN	-1,93164	-1,01695	NaN	NaN	NaN
NaN	NaN	NaN	NaN	NaN	NaN	NaN	NaN	NaN	NaN
NaN	NaN	NaN	NaN	NaN	NaN	NaN	NaN	NaN	NaN
NaN	NaN	NaN	NaN	NaN	NaN	NaN	NaN	NaN	NaN
0,157461	-2,79021	0,855005	NaN	-1,11252	NaN	NaN	-2,36834	NaN	NaN
-0,111855	-0,493196	-0,723106	NaN	-0,919575	NaN	NaN	NaN	NaN	NaN
1,46681	1,37158	2,58642	NaN	0,949511	NaN	NaN	0,506239	0,705472	0,49577
0,77442	1,04208	1,92957	-2,34169	-0,00967216	NaN	NaN	-0,567547	-0,672753	-1,05181
-0,433397	-0,52593	-1,594	NaN	NaN	NaN	NaN	NaN	NaN	NaN
1,58557	1,87325	1,1929	3,22478	2,28451	2,87071	2,9833	2,04938	1,32992	2,5183

Polito et al. Universal ready-to-use immunotherapeutic approach for the treatment of cancer: expanded and activated polyclonal γδ memory T cells.
Phosphorylation sites

NaN	NaN	NaN	NaN	NaN	NaN	NaN	3,122	NaN	3,02758
NaN	NaN	NaN	-2,1988	-1,69395	NaN	-2,22488	-2,17392	-2,4335	-1,72977
NaN	NaN	NaN	NaN	NaN	NaN	NaN	NaN	NaN	NaN
-0,737881	-0,888533	-0,953878	-0,576822	-0,571606	NaN	-1,04905	-0,567213	-0,295234	-1,64435
-1,87193	-2,08405	0,508718	-1,62687	NaN	-2,64284	NaN	-0,8657	-1,2436	-1,69651
-1,51003	0,410021	-0,411713	-0,727552	-0,626074	-1,60432	0,155195	-0,738029	-0,459178	-0,625609
NaN	-2,03975	NaN	-0,113543	NaN	0,0397129	-1,19531	NaN	NaN	NaN
NaN	NaN	NaN	NaN	NaN	NaN	NaN	-1,40017	-0,287253	-1,22348
-0,683243	NaN	-1,45764	-0,103218	-0,665983	0,124163	-0,544428	-1,21711	NaN	NaN
0,0953445	NaN	-0,608339	0,229553	0,12504	1,47326	1,38374	-0,622166	NaN	-0,936419
NaN	NaN	0,0129147	-0,252495	0,634329	NaN	0,0794296	NaN	0,143504	0,661326
NaN	NaN	NaN	NaN	NaN	NaN	NaN	NaN	NaN	NaN
NaN	-2,12437	NaN	-2,21476	NaN	-2,20775	-1,37979	NaN	NaN	NaN
-2,21715	-1,29804	-2,63884	NaN	NaN	NaN	NaN	NaN	NaN	NaN
0,436928	NaN	-0,574581	NaN	1,16159	NaN	NaN	-1,03205	-1,29053	-0,67851
-0,714729	0,361252	0,376507	NaN	1,89815	-1,62019	0,802668	0,138794	-0,0534391	1,31831
-2,0877	-0,928623	-2,07668	-0,529675	-1,08024	-0,271311	-0,62055	-2,02813	-2,48355	-1,48016
2,00949	1,30845	0,385038	0,824738	1,49418	0,524891	0,526884	1,24527	0,466613	0,821649
-0,356541	-0,714001	-0,179913	0,468706	0,77632	0,340715	0,538624	-0,190676	-0,0877695	-0,695245
1,89659	1,95057	1,57355	2,28324	2,28568	3,16991	2,70683	1,82848	2,09119	2,45809
0,699154	1,22308	0,679121	1,4992	1,70203	1,27543	1,84415	NaN	NaN	NaN
NaN	NaN	NaN	0,828157	0,461561	-0,345592	0,671734	NaN	NaN	NaN
NaN	NaN	NaN	-2,19476	NaN	-2,17768	-1,63693	-1,84351	-1,64343	-0,934531
-1,35966	1,29873	-0,909246	1,11839	2,40772	0,283678	3,12997	-0,610466	-1,98095	2,32373
NaN	NaN	NaN	-2,06533	-1,59212	-1,61912	-1,03131	NaN	NaN	NaN
NaN	NaN	NaN	NaN	NaN	NaN	NaN	NaN	NaN	NaN
-1,64934	NaN	-1,0761	-0,654844	-1,10589	NaN	0,426651	-1,30509	-1,39529	-1,56793
-2,62787	NaN	-1,78728	-2,20727	NaN	NaN	-1,60687	-1,85537	-1,53753	-1,91259
-2,95007	-1,70384	-2,44598	NaN	NaN	NaN	NaN	NaN	NaN	NaN
-2,95007	-1,70384	-2,44598	-1,25109	-2,30767	NaN	-1,17771	NaN	NaN	NaN
-0,942162	1,82649	NaN	-0,529516	1,51483	-3,77241	1,46248	NaN	NaN	NaN
0,568575	0,939569	1,72588	1,53928	2,1153	-0,147629	1,96659	1,93871	1,82877	1,52493
NaN	-0,595619	-1,98103	-1,22173	-0,98245	0,8582	-1,47573	-2,33611	-2,62213	-1,83735
0,880308	0,94783	0,572552	-0,343933	1,31586	-0,195267	-0,0681324	0,97291	0,397	0,472407

Polito et al. Universal ready-to-use immunotherapeutic approach for the treatment of cancer: expanded and activated polyclonal γδ memory T cells.
Phosphorylation sites

NaN	-1,35186	-1,03291	-2,50991	-0,86113	NaN	-2,98261	-0,697948	-0,335477	-2,58725
NaN	-2,96493	-1,54413	-2,18829	-1,81198	NaN	-2,49122	-2,11861	NaN	-2,57366
1,00799	1,21384	0,688622	-0,155678	2,1687	-1,21394	0,686666	1,19354	0,948646	1,59269
1,21109	0,936073	1,04028	0,732603	1,21377	-1,93778	1,2374	1,49488	1,2929	1,46725
-1,85214	-0,259491	-0,402437	-0,987171	NaN	NaN	0,0451431	-0,25001	-0,434882	-0,801188
1,98748	2,37546	2,05892	2,14528	2,69467	2,11146	2,32612	2,46393	2,46179	2,48002
NaN	NaN	NaN	-1,22981	NaN	-0,422575	NaN	NaN	NaN	NaN
-1,97688	0,0685558	-0,346657	NaN	NaN	NaN	NaN	-0,351467	NaN	-0,301347
-0,161469	0,553427	-0,0251007	0,0215664	NaN	-0,429337	-0,973516	NaN	NaN	NaN
0,985508	0,328627	-0,282423	0,278734	0,0681915	0,325687	0,0977573	0,76144	-0,0094862	0,0650949
NaN	NaN	NaN	0,346544	-0,637087	NaN	-2,02248	-0,547831	-0,496188	-0,918914
-0,824205	NaN	0,87664	0,721943	-0,217964	-0,0155411	0,683609	NaN	-2,60705	-1,5478
NaN	NaN	NaN	-1,33593	-1,64511	NaN	NaN	-0,98558	-2,20791	-1,8615
NaN	NaN	NaN	-1,33593	-1,64511	NaN	NaN	-0,98558	-2,20791	-1,8615
-1,69147	NaN	-0,870016	-1,71019	-1,43819	NaN	NaN	-1,42675	-1,49824	-2,01174
-1,41549	-1,96317	-1,76651	-1,11858	-1,74803	NaN	-1,567	-1,62082	-2,4272	NaN
NaN	-1,41859	-1,29454	-1,1983	NaN	-0,443884	NaN	-1,59897	-1,70422	-1,11194
-0,959549	0,445343	0,544939	0,469105	NaN	1,6125	1,10932	0,237066	-0,0698557	0,656152
-1,88548	NaN	-1,12062	-0,71706	-0,709768	-0,0252647	-0,843721	-1,46006	-1,27551	-1,32263
0,398617	NaN	-1,43987	-0,309103	-3,72146	-0,080328	-1,42822	0,16029	-0,659606	NaN
NaN	0,0777664	-0,514606	NaN	-1,54988	NaN	-1,84501	-1,29913	-0,974328	-1,10952
-2,29618	-1,95444	-2,72773	-1,29165	-1,85094	-1,09871	NaN	NaN	NaN	NaN
1,43257	0,863354	1,28259	-0,190054	2,0747	-0,504303	-0,328266	1,3363	0,724959	1,20889
-0,658968	-0,343731	0,029707	NaN	NaN	-0,314085	NaN	NaN	NaN	NaN
-0,836229	-0,288555	-0,184795	0,776726	NaN	0,136534	0,523764	-1,50855	-0,495582	NaN
0,249199	0,114954	NaN	0,635128	0,590174	0,911032	0,745434	0,807615	-0,0403681	0,754676
NaN	0,00920868	NaN	0,705805	NaN	0,972477	1,0555	NaN	NaN	NaN
NaN	NaN	NaN	NaN	NaN	NaN	NaN	NaN	NaN	NaN
NaN	NaN	NaN	0,758804	NaN	-0,0610714	-0,0569782	NaN	NaN	NaN
NaN	NaN	NaN	NaN	NaN	NaN	NaN	NaN	NaN	NaN
-0,794672	-0,433912	NaN	3,6745	-0,0977173	4,59692	NaN	NaN	NaN	NaN
1,65639	0,640347	0,923323	4,62112	1,98065	5,18995	3,39038	0,409746	0,606708	0,957368
2,70509	2,82436	3,16524	3,69239	3,52415	3,64723	3,36581	3,83103	3,5041	3,67335
-1,27625	-0,388525	-0,854681	-1,25193	-0,714285	NaN	-1,32451	-0,643436	-0,583354	-0,523545

NaN	NaN	NaN	NaN	NaN	NaN	NaN	-1,3816	-1,81301	-2,67176
0,935036	0,766768	0,922318	1,66691	-0,076067	2,68575	2,05173	0,977222	1,03595	0,673551
-0,5436	-1,42247	2,22907	2,59562	0,0852585	2,88549	3,09056	0,928911	0,0945177	0,206012
NaN	NaN	NaN	-0,99851	NaN	-0,0819759	-1,27863	NaN	NaN	NaN
-1,55182	-1,57164	-1,44945	-1,72112	0,286064	-0,741766	0,00236702	-1,96611	-2,17161	-1,18415
0,0192013	-0,217062	NaN	1,68692	0,137552	1,61348	-3,28704	NaN	NaN	NaN
NaN	NaN	NaN	NaN	NaN	NaN	NaN	NaN	NaN	NaN
0,353401	-0,895731	0,286694	NaN	NaN	NaN	0,535219	0,482513	0,745044	0,314715
0,115202	-1,17997	-0,0491123	0,636904	0,320852	-1,81438	0,865711	0,141781	NaN	-0,464881
3,18199	2,94267	2,55184	3,12891	2,81946	3,41317	2,69852	2,914	2,40259	2,24434
0,823046	1,26503	1,27577	1,63507	0,875753	0,715744	1,30127	0,953463	0,640649	0,643338
1,32505	1,43178	0,351988	0,99239	0,4615	-2,56575	0,322777	0,63361	0,621448	0,670093
-2,37249	-0,374561	-2,50889	-1,06988	-0,435263	-2,76679	-0,628923	-1,69851	-1,91574	-0,386861
NaN	0,726286	-3,17444	1,99197	2,44991	2,36651	2,50943	NaN	NaN	2,46804
NaN	NaN	NaN	-1,26963	-0,341963	-1,04187	NaN	NaN	-1,81301	-3,00985
NaN	NaN	NaN	0,480612	-3,08154	-0,00179863	0,180737	NaN	NaN	NaN
0,141624	0,396709	0,602875	0,106129	-0,306509	NaN	0,764238	-0,556242	0,676122	0,110463
-0,132919	1,09416	-0,300009	0,293747	-0,166542	-0,344528	0,491922	-0,820202	-0,0562735	-0,671182
-2,92879	NaN	-2,2373	-2,21886	-2,01627	-1,65724	-1,00627	-3,74433	NaN	-2,03196
NaN	-1,05417	NaN	0,0130844	0,50304	NaN	-0,276514	-0,281858	NaN	-0,766303
-0,844782	-1,71807	-0,118681	-0,198952	0,45118	-1,44051	-0,0143509	-0,663248	0,130111	NaN
NaN	NaN	NaN	NaN	NaN	NaN	NaN	0,257406	-0,421992	-0,622109
NaN	NaN	NaN	NaN	NaN	NaN	NaN	NaN	NaN	NaN
-0,658651	-0,285761	-0,844368	NaN	-0,562843	NaN	-1,50828	-1,01572	-1,1736	-0,954139
2,83948	2,88393	0,867607	3,70414	3,17878	-0,638317	3,92317	1,61081	1,14114	NaN
NaN	NaN	NaN	0,0644588	NaN	-0,57863	-0,108112	NaN	NaN	NaN
-0,659286	0,645733	1,17151	1,53008	1,80236	-1,45967	2,31008	-0,424505	-1,39211	0,884656
0,172491	-0,452274	0,0912304	0,292637	1,12725	-0,0994587	NaN	0,341524	0,257829	0,00902462
NaN	NaN	NaN	NaN	NaN	NaN	NaN	NaN	NaN	NaN
NaN	NaN	NaN	NaN	NaN	NaN	NaN	NaN	NaN	NaN
0,917652	1,84356	0,774521	1,21902	2,20615	0,823477	2,21312	0,41983	-0,027936	0,779222
NaN	NaN	NaN	-0,959488	-4,26996	-0,684004	-1,40011	NaN	NaN	NaN
NaN	NaN	NaN	-0,913404	-3,41448	-1,14981	-1,23295	NaN	NaN	NaN
-1,20903	0,0798473	-0,533995	-1,66024	-0,426714	-1,92539	-1,71901	0,231447	NaN	0,283078

-3,45617	-2,86201	-2,0125	-2,56334	-2,1865	NaN	NaN	NaN	-2,91814	-2,32493
0,328413	NaN	-0,517668	2,22167	-0,967752	0,818933	1,35467	0,429363	0,470658	-0,122296
NaN	NaN	NaN	NaN	NaN	NaN	NaN	NaN	NaN	NaN
NaN	NaN	-0,474058	-0,678391	NaN	-1,43383	-1,14809	-2,40776	NaN	-1,3367
NaN	NaN	-1,51149	NaN	NaN	NaN	NaN	-1,43867	-1,62801	-1,33745
NaN	NaN	NaN	NaN	-1,24016	-1,18763	-1,14705	-3,96233	-1,39044	NaN
6,56297	7,33468	5,93097	6,95756	6,8206	4,61473	7,24687	5,9057	5,17117	5,93506
-1,17612	-0,589722	0,24194	-0,851463	0,65016	NaN	-0,309811	-1,02427	-0,5381	-1,15187
-0,0872002	-0,975609	-1,31101	-0,623722	0,611975	NaN	-1,03864	-1,10234	-1,55502	-1,55169
NaN	NaN	NaN	NaN	NaN	NaN	NaN	-1,16567	-1,46141	-0,548804
-0,300653	-0,692467	-0,401794	-0,632244	0,861708	-0,857069	-0,261274	0,0474987	0,0641794	-0,162982
-1,99608	-0,670258	-2,70006	NaN	NaN	NaN	NaN	-1,35475	-0,19988	-1,82299
NaN	NaN	NaN	-3,15771	-3,4133	-2,86936	-2,6282	NaN	NaN	NaN
1,42419	0,0305195	-0,37392	0,354446	0,950085	0,708502	0,257221	0,17174	-0,331393	-0,0409784
-0,918976	-0,643681	-2,79933	-1,98681	0,277128	NaN	-2,58666	NaN	NaN	NaN
1,09958	NaN	0,781813	-1,59476	2,44506	NaN	0,923761	NaN	NaN	NaN
NaN	NaN	NaN	NaN	NaN	NaN	NaN	-3,13894	-3,31618	-2,91341
NaN	NaN	-1,18297	-0,691809	NaN	-0,600559	-1,51753	NaN	NaN	NaN
-0,93412	-1,16079	-1,32454	1,2983	NaN	1,55669	-0,144751	NaN	-0,989394	NaN
NaN	NaN	NaN	-1,73941	NaN	-1,24988	-4,94207	NaN	NaN	NaN
-2,70147	-2,0906	-1,53593	NaN	NaN	NaN	NaN	-1,2185	-1,52067	-1,60529
NaN	-2,07411	NaN	-0,563423	-2,74292	-1,29733	-0,90538	-1,55933	-1,469	-1,22874
NaN	NaN	NaN	NaN	NaN	NaN	NaN	NaN	NaN	NaN
NaN	0,567898	NaN	2,49803	0,942383	2,90859	2,19097	2,07156	1,3251	1,53066
NaN	NaN	NaN	1,43681	-1,92869	-0,452927	0,171837	NaN	NaN	NaN
NaN	NaN	NaN	0,269415	NaN	-1,40517	-1,38556	NaN	NaN	NaN
NaN	NaN	NaN	NaN	NaN	NaN	NaN	NaN	NaN	NaN
-1,07119	-1,91273	-0,732937	-0,722387	-0,241367	-1,3285	NaN	NaN	NaN	NaN
NaN	-3,98504	-0,0989323	-0,488266	-2,16339	-0,91835	0,0573502	-0,641256	NaN	-0,889665
1,72821	1,55809	1,64367	1,37706	2,14002	NaN	1,72205	1,11603	0,814326	1,05371
1,63103	NaN	1,30386	1,33184	2,38041	1,69257	2,12718	0,672455	2,23213	0,783093
NaN	-1,53534	-0,13735	-0,903847	-0,13102	0,205084	0,853271	-1,26019	-1,1933	-1,32769
NaN	NaN	NaN	-0,154715	-1,51997	1,43312	NaN	NaN	NaN	NaN
-1,10445	0,267302	-1,4852	-0,162306	1,04944	0,0788174	-0,223969	-1,77379	-1,59338	-1,54706

Polito et al. Universal ready-to-use immunotherapeutic approach for the treatment of cancer: expanded and activated polyclonal γδ memory T cells.
Phosphorylation sites

NaN	NaN	-2,44206	NaN	-3,01533	-1,50398	-2,72918	NaN	NaN	NaN
0,920839	0,33753	-1,08565	1,55275	-1,01581	1,67954	0,213408	-0,130926	-0,597125	-1,38978
-1,32833	-0,366064	-0,538944	-0,622335	-1,04808	-1,02156	-0,681499	-0,887268	-1,49379	-0,827662
0,557608	1,23126	0,927788	1,83904	0,60808	-1,15767	0,971952	2,01276	1,04254	1,05548
-0,209801	-0,642561	-1,88145	-0,607555	-1,26824	NaN	-1,06776	-0,616533	-0,741382	-1,48055
-2,03296	-0,491312	-1,22229	NaN	NaN	NaN	NaN	NaN	NaN	NaN
0,617973	0,193615	0,11207	1,12447	0,557106	0,168234	0,5725	0,103964	0,146119	0,672544
NaN	NaN	-1,96455	NaN	NaN	NaN	NaN	-1,96688	-2,07841	-1,48851
-1,85641	-1,96207	-2,0349	-1,87174	-1,27228	-1,84299	NaN	NaN	NaN	NaN
NaN	NaN	NaN	-2,17015	-1,39859	-2,82199	-1,9946	NaN	NaN	NaN
NaN	-1,0857	-0,880281	-0,26795	-1,21321	NaN	-1,1969	-0,973579	-1,27318	-1,15667
4,35555	3,62237	3,32006	3,62153	3,97551	4,1044	3,26092	3,25322	3,33009	3,60894
0,485899	0,540739	-0,137957	-0,541559	-1,00122	0,0273018	0,223166	-0,803171	0,460917	-0,104571
NaN	NaN	NaN	-0,496983	NaN	0,278254	NaN	-0,400602	NaN	NaN
NaN	NaN	NaN	NaN	NaN	NaN	NaN	NaN	NaN	NaN
NaN	0,0634518	NaN	-1,77439	0,104294	NaN	-1,15146	-1,79536	-2,19067	-1,09763
0,12014	1,18528	0,275301	1,48193	0,873674	2,72513	0,879503	0,753412	0,889318	1,02884
0,815203	1,72511	1,72678	2,21198	1,66306	2,64505	2,56875	2,21664	1,77897	2,45701
NaN	NaN	NaN	-2,09314	NaN	-3,12362	-1,13346	NaN	NaN	NaN
-0,97583	-1,12118	-0,776714	-0,214781	NaN	1,5315	-0,979145	-1,79596	-1,60331	-2,39659
-0,938692	-1,63511	-0,666061	-0,335789	NaN	1,44919	-1,09633	-1,28696	-1,54613	-1,55176
0,650642	0,84387	1,52045	0,862837	0,804529	0,133806	0,807144	1,0357	1,55662	1,6318
-0,60162	NaN	-0,0572262	3,28123	0,876125	3,57386	2,90273	0,919184	1,51862	1,46002
NaN	NaN	-0,366196	0,121384	NaN	0,334932	-0,170952	-2,35462	-2,23228	-2,55547
4,18258	2,90192	5,35591	5,42905	2,3265	7,07507	4,96569	5,12776	4,58694	4,10502
NaN	-1,60694	NaN	-0,140017	-0,951715	-0,655975	-0,335636	NaN	NaN	NaN
-1,03456	0,732113	-1,31391	0,218552	0,507547	-2,49304	0,375374	-0,573139	-1,55087	0,263558
-1,86006	NaN	-1,84787	-1,95389	-1,68405	-2,9746	-1,83988	-1,32939	-1,74784	-2,02373
0,671392	0,572828	-0,101795	0,378824	-0,108223	NaN	0,371181	-0,0922565	-0,774222	-0,0937071
NaN	-0,745935	NaN	0,0574799	-0,499208	-0,459436	-0,0957603	NaN	NaN	NaN
-1,13476	-0,589371	NaN	-1,19623	-0,972151	NaN	-0,508635	NaN	NaN	NaN
0,489527	0,783607	0,0831299	0,758152	-0,696898	1,23304	0,746632	-1,56692	0,199693	-1,52781
0,895987	2,01381	-0,106146	0,907425	-0,259632	1,56602	1,92264	0,365923	1,233	1,18275
NaN	NaN	-1,47309	NaN	-1,24635	-1,35809	-1,61904	-1,76317	-1,59084	-1,41991

-1,58532	-1,35162	-1,22145	-1,37045	-0,758045	-1,48695	-0,633631	-0,73052	-0,830369	-0,596482
-0,401825	-0,122961	0,164927	1,33067	1,08043	0,867361	1,30516	0,253855	-0,45094	-0,0281897
-1,78599	-0,719326	-1,63818	-1,83082	NaN	-1,47062	-1,62282	-1,66309	-1,66285	-3,01452
NaN	NaN	NaN	0,489149	-0,361374	NaN	-0,482546	-1,08187	-1,1527	0,453628
0,0813198	-0,0787735	0,365871	-0,555405	0,228354	0,439102	0,478224	1,14958	0,746266	0,598723
-3,41984	-2,03209	-1,81685	-2,55489	-0,988079	NaN	NaN	-0,405119	-2,06102	-0,285932
NaN	NaN	NaN	-1,65005	-2,15868	-1,43746	-2,01577	NaN	NaN	NaN
NaN	NaN	NaN	-0,659946	NaN	-0,519667	-1,40017	NaN	NaN	NaN
NaN	NaN	NaN	-0,595255	NaN	0,018219	-2,49122	NaN	NaN	NaN
NaN	NaN	NaN	-2,38549	-2,78518	-1,63623	-1,98192	NaN	NaN	NaN
NaN	0,491505	NaN	NaN	NaN	NaN	NaN	NaN	NaN	NaN
-0,214117	-0,20525	0,129478	1,90958	0,00405884	1,91472	1,37756	0,0855827	-0,0749235	-0,110208
NaN	NaN	NaN	0,342882	-2,55848	0,172918	NaN	NaN	NaN	NaN
NaN	NaN	NaN	-1,02752	NaN	-0,370831	-1,19746	NaN	NaN	NaN
2,48119	4,75515	0,275459	0,946007	-0,165426	1,02182	NaN	1,41108	2,78692	1,86771
-0,0527821	0,881815	1,11978	0,485872	1,55302	0,456673	1,11614	1,77278	1,70736	2,23248
-0,537107	-0,268433	-0,242155	NaN	-0,22579	-1,69407	-1,11156	0,636709	0,445865	0,476405
NaN	NaN	NaN	-1,82735	NaN	NaN	-0,902035	NaN	NaN	NaN
NaN	NaN	NaN	-2,98698	-1,88058	0,658512	-1,60225	-2,41267	-2,97181	-0,000952721
NaN	-0,599791	NaN	NaN	-1,6846	-0,51656	NaN	-0,363291	-0,880536	0,259788
1,14732	2,19051	0,574974	0,611019	1,6793	1,55069	0,335426	1,93675	NaN	1,96008
-0,972542	1,09394	-0,880163	-0,933908	-0,921997	-0,894741	-0,270517	-0,386545	-0,170222	0,390815
2,15129	2,71097	NaN	NaN	3,3768	NaN	NaN	2,33975	2,01859	3,33237
NaN	NaN	NaN	NaN	NaN	NaN	NaN	NaN	NaN	NaN
NaN	NaN	NaN	NaN	NaN	NaN	NaN	NaN	NaN	NaN
0,5023	-0,187311	-0,287407	0,102982	0,478456	NaN	-0,289047	-0,266087	-0,251754	NaN
NaN	-3,98494	-0,783411	-0,102192	NaN	-0,165661	0,325954	NaN	NaN	NaN
NaN	-0,515026	-0,0627289	0,384468	-1,39605	0,520302	0,250193	-0,566401	-0,855308	-0,485648
-0,98946	-1,33828	-1,52472	NaN	NaN	NaN	NaN	-1,20607	-1,76002	-2,44001
NaN	NaN	NaN	NaN	NaN	NaN	NaN	NaN	NaN	NaN
-1,25799	0,0692539	1,04244	-0,879934	1,49247	-1,69031	0,695803	NaN	NaN	NaN
-1,22032	-2,46432	0,0764713	NaN	0,703356	-2,37651	NaN	0,209547	0,394475	0,532378
NaN	NaN	NaN	NaN	NaN	NaN	NaN	NaN	NaN	NaN
NaN	NaN	NaN	-0,00543213	-1,4669	0,294954	0,253176	-0,767279	0,246964	-0,279735

Polito et al. Universal ready-to-use immunotherapeutic approach for the treatment of cancer: expanded and activated polyclonal γδ memory T cells.
Phosphorylation sites

NaN	NaN	NaN	-2,13137	NaN	-2,04634	-1,85645	NaN	NaN	NaN
-1,02005	-1,49377	-0,605917	-1,16696	-0,513708	NaN	-0,90848	-0,714674	-0,722781	-1,16983
NaN	NaN	NaN	-2,39852	-3,08646	NaN	-1,8288	NaN	NaN	NaN
-1,77678	-2,14221	NaN	NaN	-1,9262	-0,477661	-0,112551	NaN	NaN	NaN
NaN	NaN	NaN	0,100996	NaN	0,587477	NaN	NaN	NaN	NaN
NaN	NaN	NaN	-0,524933	-0,994976	-0,948421	-0,369474	-1,37855	-1,34435	-1,6232
NaN	NaN	NaN	-1,77905	-1,88761	-2,46929	-1,2093	NaN	NaN	NaN
2,05612	1,93709	1,93551	2,91498	1,69765	-1,06814	2,36642	1,53598	1,52448	1,25539
NaN	NaN	NaN	NaN	NaN	NaN	NaN	NaN	NaN	NaN
-2,24217	-2,23875	-2,11326	0,995436	-1,22075	0,908672	0,253796	NaN	NaN	NaN
NaN	NaN	NaN	NaN	NaN	NaN	NaN	NaN	NaN	NaN
0,888893	2,15342	NaN	2,94575	-0,770359	4,08667	1,26834	NaN	0,105788	-1,2496
-0,432312	0,911106	-1,04817	-0,380413	-0,27043	-0,0879498	0,410568	NaN	-0,500882	-0,965074
NaN	NaN	NaN	NaN	NaN	NaN	NaN	-4,7456	-3,457	-2,50132
-2,22673	-2,28956	-1,6809	-1,39127	-1,92631	-1,88974	-0,93622	-1,59935	-2,08318	-1,7572
NaN	NaN	NaN	-0,941725	NaN	-0,0771637	-0,914408	NaN	NaN	NaN
NaN	-0,772779	0,552893	1,75902	NaN	-0,879229	-0,478991	-0,690683	-0,742274	0,0429468
NaN	NaN	NaN	NaN	NaN	NaN	NaN	NaN	NaN	NaN
NaN	NaN	NaN	NaN	NaN	NaN	NaN	-0,629749	-1,47896	-1,39279
0,655327	1,11013	0,155422	1,29043	0,638956	0,436272	0,774727	0,19046	0,305324	-0,380065
NaN	NaN	NaN	0,0592632	NaN	-0,499992	-0,980333	NaN	-0,513972	NaN
-1,70117	-1,76352	-1,41084	-1,16235	-2,44494	NaN	-1,38095	-1,74157	-2,2371	-2,45063
2,10504	2,25021	2,27573	2,65152	2,35106	2,79083	2,91499	2,33066	2,23605	1,90594
-2,02822	-3,43795	-1,3694	-1,27456	-1,51403	NaN	-1,22373	-1,577	-1,9965	-2,16818
-0,947832	-2,13206	NaN	-0,793856	-1,14478	-0,247374	0,0170403	-0,326607	-0,627757	-0,851192
-0,118626	0,424137	-0,435766	1,45362	NaN	0,123943	1,09276	-0,255327	0,207351	0,344844
NaN	NaN	NaN	-1,87275	-2,22719	NaN	-1,54812	NaN	NaN	NaN
NaN	NaN	NaN	NaN	NaN	NaN	NaN	NaN	NaN	NaN
2,11311	1,76048	1,32813	2,15406	3,17645	-0,717564	2,19029	2,60414	2,20548	2,39475
-2,0368	-1,60007	-1,62165	NaN	-0,698511	-0,00564194	-0,0883045	-0,38472	-0,520253	-0,333922
NaN	NaN	NaN	-0,265142	-0,815681	-1,29195	-1,09887	-1,29925	-2,02076	-1,40903
-1,21368	-0,891939	1,32703	2,14859	1,37767	2,85342	2,29089	2,41305	2,23897	-0,943154
-1,66632	-1,01672	-1,30503	NaN	NaN	NaN	NaN	NaN	NaN	NaN
NaN	NaN	NaN	NaN	NaN	NaN	NaN	NaN	NaN	NaN

Polito et al. Universal ready-to-use immunotherapeutic approach for the treatment of cancer: expanded and activated polyclonal γδ memory T cells.
Phosphorylation sites

1,19879	2,38534	NaN	1,62324	2,67984	0,0865116	2,14578	1,56459	1,48472	NaN
0,139681	2,06233	0,408287	1,12801	1,42567	0,998022	1,51041	0,147511	0,91042	0,736886
NaN	NaN	-1,06807	NaN	0,265846	-0,484032	-1,24805	-1,14165	-1,08993	-0,00486088
4,08587	3,72041	3,79126	3,91417	4,65233	3,95362	4,05532	4,03328	3,72936	3,56949
2,65448	-0,123537	0,464317	-0,887873	2,5367	0,450197	0,523375	0,0467262	0,765216	1,5097
NaN	NaN	NaN	-1,05495	NaN	NaN	-0,64393	NaN	NaN	NaN
1,84216	2,38787	1,83001	3,06494	2,50379	3,53303	2,95764	1,8722	1,9201	1,61609
NaN	NaN	NaN	NaN	NaN	NaN	NaN	NaN	NaN	NaN
-0,847511	NaN	-0,179384	0,425972	1,03133	-0,894341	1,04914	0,391569	0,423314	0,441478
-0,818485	1,08237	-1,30634	-2,34197	1,91333	NaN	0,708029	-1,33914	-1,3442	0,226045
NaN	NaN	NaN	0,564793	NaN	1,63652	-0,458456	NaN	NaN	NaN
-0,145382	-0,0493298	NaN	0,87291	-0,651886	0,993876	0,58724	NaN	NaN	-1,47856
-0,71858	-0,810123	-1,15084	0,620615	-1,25623	0,278154	-0,516956	NaN	NaN	NaN
NaN	NaN	NaN	-1,42241	-1,46541	NaN	-1,55427	NaN	NaN	NaN
NaN	NaN	NaN	NaN	NaN	NaN	NaN	NaN	NaN	NaN
-0,330402	0,181715	-0,378681	1,00004	NaN	0,409275	0,6548	-0,73616	0,0602312	-0,323173
NaN	NaN	NaN	-2,46081	-2,49	-0,809038	-2,59191	-2,63758	-1,79688	-2,46349
-1,79239	-2,02035	-1,69751	NaN	-0,95001	-3,02922	-1,59218	NaN	NaN	NaN
NaN	NaN	NaN	NaN	NaN	NaN	NaN	NaN	NaN	NaN
NaN	0,15929	-0,661594	-1,63878	0,502478	-1,42392	-0,152554	-1,17207	0,0307417	-0,197171
1,99986	1,06839	2,64194	1,35394	3,09647	2,20957	1,66407	1,84912	2,46034	1,5235
0,316219	0,845257	0,845837	0,190685	1,43253	-3,4067	0,517876	0,112202	0,354434	-0,0799112
-0,777544	-1,46235	1,13636	1,74099	-0,786209	1,81451	1,89605	-0,0886192	-0,387458	0,270881
-0,565168	NaN	-1,04979	-1,63638	0,836597	NaN	-0,610962	-0,863838	-1,25515	-1,18186
NaN	NaN	NaN	NaN	-0,0609798	-0,0765305	0,270824	NaN	NaN	NaN
-0,296152	-0,609219	NaN	-0,202227	NaN	0,0707378	-1,11557	-1,27237	-1,52008	-1,97365
NaN	NaN	NaN	-2,22409	NaN	-0,959867	-1,50207	NaN	NaN	NaN
-0,362095	NaN	-1,79205	NaN	NaN	NaN	NaN	-1,1925	-1,58498	-1,84425
NaN	NaN	NaN	-1,88227	NaN	-1,24226	-2,24022	-0,883121	NaN	-0,928317
-1,83763	-2,56794	NaN	-2,53611	-1,14271	-1,4841	-2,01333	-2,23837	-1,84124	-2,79095
NaN	NaN	NaN	-1,11711	-3,60912	-0,979959	-2,35591	NaN	NaN	NaN
NaN	NaN	NaN	-1,57173	NaN	0,0686836	-3,14831	NaN	NaN	NaN
NaN	NaN	NaN	NaN	NaN	NaN	NaN	NaN	NaN	NaN
-0,887211	-0,335018	-0,375542	-0,795195	-0,140547	NaN	-0,172438	-1,14782	-1,02122	-0,586455

Polito et al. Universal ready-to-use immunotherapeutic approach for the treatment of cancer: expanded and activated polyclonal γδ memory T cells.
Phosphorylation sites

-1,19118	-0,424305	-1,02039	-1,57737	-0,921728	-0,899069	-0,646585	-1,18692	-0,925675	-1,15504
NaN	NaN	NaN	-0,641993	NaN	NaN	NaN	-2,47593	-1,27715	-1,89567
NaN	NaN	NaN	0,649261	NaN	NaN	0,15373	NaN	NaN	NaN
NaN	NaN	NaN	0,840631	NaN	NaN	NaN	NaN	NaN	NaN
0,942604	0,895029	-0,162823	0,983585	1,52376	0,782055	0,316206	0,727827	1,22074	1,06231
NaN	NaN	NaN	NaN	NaN	NaN	NaN	NaN	NaN	NaN
-0,509741	0,174002	0,155346	-0,0273705	-0,218626	NaN	-0,491692	0,62215	0,199264	0,270425
5,41446	4,38883	5,27698	5,23522	5,61888	5,24087	4,70917	5,46948	5,77935	5,40285
-1,25043	-1,15696	-0,656574	-1,38247	-1,16632	NaN	-1,5741	-0,889992	-1,28708	-1,5525
NaN	NaN	1,50209	1,98818	NaN	1,82243	1,70577	1,20635	1,22373	0,81944
-1,4159	1,15699	2,19197	2,49402	1,73767	2,75055	2,05813	2,22173	1,73904	1,79802
-0,0585117	0,0180359	0,394711	1,39946	NaN	NaN	1,02968	0,975321	0,388925	-0,181315
NaN	NaN	NaN	-2,86523	-2,28643	-2,38694	NaN	NaN	NaN	NaN
-1,53652	NaN	NaN	-0,741869	-2,05533	NaN	-1,05702	-0,925413	-0,706157	-2,24204
-1,8616	-0,109032	-1,79499	-1,17093	-2,07568	-0,663359	-0,516829	NaN	NaN	-1,62186
NaN	-1,13155	-0,699438	NaN	-0,553423	0,241119	-0,423748	-1,21242	-0,462508	-1,5579
-1,53226	-0,0589523	0,241922	-0,380293	0,80065	-3,08335	1,15429	-0,450924	-0,437907	1,3434
-1,85061	NaN	-1,8874	-1,10251	-2,08024	-0,332874	-0,598286	NaN	NaN	NaN
NaN	NaN	NaN	-1,30254	NaN	-0,746866	NaN	NaN	NaN	NaN
-0,740677	-1,44859	-1,08961	-0,936123	-0,218758	-0,425484	-0,894039	-1,17764	-1,20976	-1,26524
-2,35743	NaN	-1,99422	-0,484764	-1,51144	-0,157219	-0,72542	NaN	NaN	NaN
-0,307487	0,0227966	0,489462	0,982561	0,910656	1,00072	1,17275	0,0137768	-0,0276213	0,308799
-2,25001	-3,49199	-0,877357	1,0686	-2,05367	2,59717	0,14539	-1,41094	-1,3083	-1,98406
-0,494473	0,387569	-0,886538	0,837858	NaN	-0,477043	0,524229	-0,0190067	-0,226939	0,159408
-1,92121	NaN	-3,31154	-1,87578	NaN	-0,893337	-2,05192	NaN	NaN	NaN
NaN	NaN	NaN	1,218	-2,92824	1,19784	0,0837383	-0,369915	-1,23184	-0,185485
-1,4281	-1,59395	-0,817148	NaN	NaN	NaN	NaN	-1,73641	-1,61746	-1,87053
0,358442	-0,185274	-0,793259	NaN	-0,273588	-1,18256	NaN	NaN	-0,235557	-0,461274
0,165602	-1,32835	-0,25189	-0,375921	0,179581	-0,365063	0,35717	-0,000448227	-0,347064	-0,696212
0,0287056	-0,483253	-0,34663	-0,934223	NaN	-0,122885	0,113356	-1,08134	-1,08679	-1,60138
-0,71858	-0,103783	-0,40822	-0,813105	-0,489515	-0,382984	0,111494	-0,376537	-0,714311	-0,504506
0,427931	-0,027586	-0,348087	-0,522976	0,186743	-0,0345631	0,655085	0,0913296	-0,567281	-0,504733
NaN	NaN	NaN	-2,19805	NaN	-1,6467	-1,74057	NaN	NaN	NaN
1,30703	-0,346916	1,48652	-2,43136	1,57523	NaN	-0,508062	1,33056	0,674581	0,730815

Polito et al. Universal ready-to-use immunotherapeutic approach for the treatment of cancer: expanded and activated polyclonal γδ memory T cells.
Phosphorylation sites

NaN	NaN	NaN	NaN	NaN	NaN	NaN	NaN	NaN	NaN
-0,552052	NaN	NaN	-0,0319862	NaN	1,06702	-1,05647	-0,594372	-2,31947	NaN
-0,182491	-0,953478	-0,609762	0,103786	-0,144564	-1,59253	-0,00701141	-0,105936	-0,0690393	-0,438548
-0,645288	-0,350071	-1,43396	NaN	NaN	NaN	NaN	NaN	NaN	NaN
NaN	NaN	NaN	-3,36666	-2,93034	-2,33269	-2,74707	-2,3801	-2,37818	-2,14719
NaN	-0,792072	NaN	0,552061	NaN	1,12872	0,833969	-1,81181	-1,41889	-1,19058
0,31604	-0,0505714	0,0491142	0,201069	0,814829	0,335312	0,374857	-0,593845	-0,351087	0,774802
0,858809	0,615854	0,486393	0,664186	0,700438	0,672188	0,910864	NaN	NaN	NaN
0,00510216	1,6384	1,40993	0,779406	2,07921	-0,383875	1,04428	0,898058	0,505864	0,645408
-1,00541	-1,59597	-0,750444	-1,86503	-0,9575	NaN	-2,13145	-0,967226	-1,74282	-1,59596
NaN	NaN	0,482485	0,708508	-2,55288	-1,33381	-1,75551	NaN	NaN	NaN
1,3639	1,99087	1,3875	1,28606	2,24499	1,06651	1,3496	1,63751	0,985883	1,13955
-1,86289	-0,221651	-1,36257	-1,41848	0,456184	-2,33801	0,00877953	-2,35718	-1,9263	-1,46628
-1,93779	-1,37232	NaN	-1,0572	-2,47189	-1,73848	-1,24566	NaN	NaN	NaN
2,90394	2,38912	2,29132	2,93845	2,95906	0,901953	2,90234	1,57208	1,34243	1,75773
-3,2709	NaN	NaN	-1,60205	NaN	-1,31637	-1,94712	NaN	NaN	NaN
NaN	NaN	NaN	-1,74273	-2,98246	-2,83587	-2,04609	NaN	NaN	NaN
NaN	NaN	NaN	NaN	NaN	NaN	NaN	NaN	NaN	NaN
NaN	NaN	NaN	-0,428339	NaN	-0,924591	0,0973587	-1,21572	-2,06144	-1,43845
NaN	NaN	NaN	0,134304	NaN	1,70575	-2,4926	NaN	-1,77587	NaN
NaN	NaN	NaN	0,645624	NaN	1,23569	NaN	NaN	NaN	NaN
NaN	NaN	NaN	-0,280912	NaN	-1,3113	NaN	NaN	NaN	NaN
NaN	NaN	NaN	-0,147142	NaN	-0,680273	-2,69333	NaN	NaN	NaN
-0,269756	1,15859	2,07857	0,915438	1,66323	1,13822	0,844337	NaN	NaN	NaN
NaN	NaN	NaN	NaN	NaN	NaN	NaN	NaN	NaN	NaN
-2,49309	-1,76919	-1,61358	0,0375404	-1,60322	-0,150021	-0,22496	NaN	NaN	NaN
-0,526619	-0,388565	-0,420254	0,0581684	0,122431	1,29671	1,02793	-0,521456	-0,555718	-1,33693
NaN	NaN	NaN	-0,336115	NaN	-0,0767841	NaN	NaN	NaN	NaN
-0,190874	NaN	-0,632847	-1,50578	-0,894833	-2,00473	-2,52195	-0,55727	-1,25095	-0,131469
2,88482	2,44131	1,89784	1,67067	2,38847	1,05138	2,41278	1,58369	1,19131	1,3023
1,6566	1,43016	1,16831	0,758913	1,5432	0,936739	2,39491	1,34487	0,923995	1,07522
NaN	NaN	NaN	NaN	NaN	NaN	NaN	NaN	NaN	NaN
-2,69805	-2,07457	-2,9533	-1,67936	-1,75906	-1,81898	-1,84701	-1,90066	-1,95217	-2,04034
0,791412	0,67647	0,709641	-1,08302	1,54125	NaN	-1,41481	0,797318	0,518546	0,512599

Polito et al. Universal ready-to-use immunotherapeutic approach for the treatment of cancer: expanded and activated polyclonal γδ memory T cells.
Phosphorylation sites

0,16411	-0,0856171	-1,29651	-0,685173	0,326078	-0,230122	-1,44729	-0,597916	-0,939965	-0,946113
-1,14459	-0,416821	-0,408108	-0,927252	0,863443	0,574177	1,50829	0,409746	-0,115584	0,131042
NaN	NaN	NaN	NaN	NaN	NaN	NaN	-1,52871	-2,00635	-2,07299
NaN	NaN	NaN	-1,5982	-1,02059	NaN	-1,23753	-1,14594	NaN	NaN
-2,48707	-1,14938	NaN	-1,02061	-1,23493	-2,06302	-0,436516	NaN	NaN	-0,557897
NaN	-0,0376091	NaN	-0,390232	NaN	NaN	NaN	NaN	NaN	NaN
NaN	NaN	NaN	NaN	NaN	NaN	NaN	-1,47558	-1,43949	-1,64522
0,612026	0,754448	0,475382	0,274986	1,38335	-1,39159	-0,306187	0,340263	-0,0224047	0,541078
2,20272	1,83241	1,98299	2,05796	2,32695	1,9643	2,06328	1,44519	1,00082	1,19111
NaN	NaN	NaN	NaN	NaN	-1,52749	NaN	NaN	NaN	NaN
0,13418	0,147102	-0,189793	0,326338	0,594946	0,38167	-0,0142384	NaN	NaN	NaN
NaN	NaN	NaN	-1,52269	NaN	-0,952635	0,0890217	NaN	NaN	NaN
NaN	-1,19134	NaN	NaN	NaN	NaN	NaN	-1,35609	-1,58369	-0,185536
NaN	0,586483	NaN	NaN	-0,0784664	0,335028	NaN	NaN	NaN	NaN
-1,12416	NaN	-0,165245	-0,559788	-0,14447	-1,03527	-0,415796	0,626955	0,576221	0,236934
NaN	NaN	NaN	NaN	NaN	NaN	NaN	NaN	NaN	NaN
NaN	0,689377	0,913229	1,92733	1,69827	2,13623	1,73654	1,3239	0,53251	0,972184
NaN	NaN	NaN	-1,84957	NaN	-0,0695972	-1,86595	NaN	NaN	NaN
NaN	NaN	NaN	-2,20914	-3,29765	-1,41933	NaN	NaN	NaN	NaN
0,295437	1,18165	0,819534	1,42316	0,850304	2,08439	1,51652	0,918526	0,698916	0,655845
NaN	NaN	NaN	-0,491726	-2,83225	-0,724119	-0,16045	NaN	-1,71455	-2,21995
-1,34173	-1,27114	-0,977516	-1,16194	-0,845413	-2,07327	-0,433222	NaN	NaN	NaN
-0,86322	-1,91967	-0,450695	2,43195	-1,89362	0,786022	1,89569	-0,203228	-0,680062	-1,26778
0,972599	3,80331	1,39977	2,39146	1,6848	1,82283	2,37536	1,21862	2,18402	2,11967
-1,04038	NaN	1,7219	-0,796951	-0,464977	0,306822	-0,394344	0,935318	0,813743	NaN
1,44114	1,58721	1,29248	-0,799156	1,55166	NaN	-0,922474	1,29329	1,63979	1,78149
3,28203	4,05451	3,21011	3,47835	3,30724	4,50359	3,8043	3,92716	3,34118	4,11605
NaN	NaN	-1,29277	NaN	NaN	NaN	NaN	-0,631912	-1,10644	-1,18212
-0,321589	-0,326769	0,0159168	-0,103771	0,47578	-0,89118	0,23546	1,06066	0,482039	0,710202
0,612553	-0,307787	1,1452	-1,48623	1,96391	NaN	1,36677	2,05185	1,81129	1,45851
0,900776	NaN	0,72036	-0,704575	0,511482	0,0856075	-0,0918484	0,493414	0,295314	0,451734
NaN	NaN	NaN	NaN	NaN	NaN	NaN	NaN	NaN	NaN
NaN	NaN	NaN	NaN	NaN	NaN	NaN	0,680243	NaN	0,709922
1,45968	NaN	NaN	0,520243	-0,119637	1,5495	NaN	NaN	NaN	NaN

Polito et al. Universal ready-to-use immunotherapeutic approach for the treatment of cancer: expanded and activated polyclonal γδ memory T cells.
Phosphorylation sites

2,45126	1,7884	2,81361	3,78664	2,43321	2,01529	2,77848	-0,341099	2,74526	2,51645
-1,12246	-1,17838	-0,91671	-1,0732	-0,162973	-3,05668	-0,36331	0,749664	0,649568	NaN
-1,53284	NaN	-2,8967	NaN	NaN	NaN	NaN	-1,34597	-2,15708	-1,97393
-1,89367	-2,30642	-0,757057	NaN	NaN	NaN	NaN	NaN	NaN	NaN
-0,47666	-0,170015	0,22365	0,425835	-0,829409	NaN	-1,49853	0,111879	-0,169185	0,04074
0,15892	0,118258	0,997286	1,33985	0,334377	-1,92198	0,719881	1,03363	0,932616	1,52461
-0,0124874	-0,548969	2,177	2,63891	0,481697	2,48223	3,04925	0,586683	1,36092	0,301909
-0,884077	-1,27242	-1,85683	-1,53979	-0,647203	-2,07959	-2,0472	-0,476412	-0,602937	-0,467511
NaN	NaN	NaN	0,139254	-0,56827	-0,0822296	0,70076	NaN	NaN	NaN
NaN	-0,778299	-1,48722	-0,680157	-1,40852	-1,70469	-0,957762	NaN	NaN	NaN
NaN	NaN	NaN	NaN	NaN	NaN	NaN	NaN	NaN	NaN
1,2528	1,20893	1,17743	-1,52147	1,59551	-0,0836258	-0,650826	1,18753	0,306832	0,839585
NaN	-0,878559	NaN	-1,25679	NaN	-0,848478	-1,77068	NaN	NaN	NaN
NaN	NaN	NaN	-1,6177	-2,804	-1,66592	-1,19357	NaN	NaN	NaN
NaN	NaN	NaN	-1,87956	NaN	-1,21703	-1,92472	NaN	NaN	NaN
1,26027	1,26231	2,24324	0,932009	NaN	NaN	1,56785	2,1919	1,7262	1,87405
2,63063	2,21357	2,93288	2,76349	2,07051	3,12295	2,93239	2,17527	2,39567	3,76156
1,40139	2,60813	3,14358	2,62354	1,77944	2,63713	2,65707	3,27054	3,25912	2,87189
3,14313	3,9763	1,98046	3,61701	2,05691	3,55787	3,74588	1,12886	3,20608	3,85191
1,74316	1,1471	2,06749	NaN	2,4066	NaN	1,17315	2,25304	2,04433	2,15457
0,0561867	0,508907	1,13173	-0,620695	0,899481	-1,75789	0,869631	1,62503	1,63775	0,765653
1,33791	2,85481	1,9042	1,18013	2,69291	0,93417	1,97525	1,43683	0,733495	1,5227
-2,88614	NaN	-1,07927	NaN	NaN	NaN	NaN	-2,16204	-2,53041	-1,9164
1,5049	2,16072	1,52333	1,7613	2,34989	0,805763	2,31283	1,48119	1,88449	2,65034
NaN	NaN	NaN	0,212011	-1,45524	-0,128246	-1,42949	NaN	NaN	NaN
NaN	NaN	NaN	NaN	NaN	NaN	NaN	NaN	NaN	NaN
NaN	NaN	NaN	0,489281	NaN	-0,261669	0,637524	NaN	NaN	NaN
5,06642	5,25425	6,09812	6,10211	6,58403	3,36804	5,53201	7,22635	7,00887	2,32785
-1,17779	NaN	-0,498226	-0,352873	-0,0969276	NaN	0,0910797	-0,290131	-0,291915	-0,00331974
NaN	NaN	NaN	-2,36452	NaN	-1,32193	-1,92142	NaN	NaN	NaN
NaN	NaN	1,2379	1,77119	NaN	2,5211	2,52337	-1,44987	NaN	-1,46033
NaN	-4,09484	0,558228	-0,0250874	-3,41551	0,766571	1,41793	NaN	NaN	NaN
NaN	-1,0371	-0,757666	2,0749	-1,28373	2,85423	1,66336	NaN	NaN	NaN
NaN	NaN	NaN	-1,15955	NaN	-1,40848	-1,36371	NaN	NaN	NaN

Polito et al. Universal ready-to-use immunotherapeutic approach for the treatment of cancer: expanded and activated polyclonal γδ memory T cells.
Phosphorylation sites

0,0234222	0,274275	0,505526	NaN	1,25175	-0,26095	0,219139	1,08943	0,122388	0,435569
NaN	NaN	NaN	NaN	NaN	NaN	NaN	-1,32764	-1,49419	0,0758924
-0,378193	-0,213552	0,309021	NaN	NaN	NaN	NaN	-0,0490894	-0,951699	0,479436
0,808592	NaN	3,34993	2,21353	3,34433	1,01158	2,01803	2,22028	1,69632	2,45805
NaN	NaN	NaN	NaN	NaN	NaN	NaN	NaN	NaN	NaN
NaN	NaN	NaN	-2,45788	NaN	-2,30833	-2,65455	NaN	NaN	NaN
NaN	NaN	NaN	-0,0989799	-2,23774	-1,30489	-0,37254	NaN	NaN	NaN
NaN	NaN	NaN	1,8126	1,00522	2,05663	1,22645	NaN	NaN	NaN
-1,07551	1,54651	-0,317678	1,27858	-0,853199	1,93018	1,3	-0,89337	-0,92821	-1,14506
NaN	NaN	NaN	-1,55792	NaN	-1,76818	-3,52772	NaN	NaN	NaN
0,509327	0,404381	0,339228	2,41863	0,017128	1,80902	1,52485	0,66494	0,695754	0,331464
NaN	NaN	NaN	-1,07232	-2,37345	NaN	-1,50759	NaN	NaN	NaN
NaN	-1,49447	-2,57518	0,21542	NaN	0,300804	-0,800032	-3,16999	NaN	-1,86567
NaN	0,159988	-0,737736	2,01207	0,689196	0,478769	0,2136	0,258972	0,19045	-0,000218391
NaN	NaN	NaN	-1,86161	-1,84869	NaN	NaN	NaN	NaN	NaN
-2,01418	-0,921022	-2,09114	-3,24797	NaN	-0,184059	-1,60803	-2,77401	-2,88354	-1,73694
NaN	NaN	NaN	NaN	NaN	NaN	NaN	NaN	NaN	NaN
NaN	NaN	NaN	NaN	NaN	NaN	NaN	-0,514349	0,17872	0,129176
NaN	NaN	NaN	-0,571321	-1,65305	-0,74588	NaN	NaN	NaN	NaN
-0,601484	NaN	-1,38094	1,96299	NaN	0,878027	0,706659	-0,0866833	-0,435824	-0,598392
0,10293	-0,703688	-0,724855	-0,88295	-0,109386	NaN	-1,42234	1,03155	0,885215	0,571525
-1,50736	-1,21256	-1,14427	NaN	NaN	NaN	NaN	NaN	NaN	NaN
-0,94693	-0,553101	-1,38664	-1,44536	-1,86537	-0,564402	-0,31189	NaN	NaN	NaN
3,68127	3,09555	3,52481	2,98137	3,45479	3,26522	3,03296	3,04944	2,88932	3,02326
-3,88792	-1,56522	NaN	-2,44001	-0,333233	NaN	-0,654831	NaN	NaN	-0,820188
-1,87676	-1,5901	-1,93605	NaN	NaN	NaN	NaN	NaN	NaN	NaN
NaN	NaN	NaN	0,386721	NaN	-0,585213	-1,18537	NaN	NaN	NaN
-1,51908	-0,854671	-2,11666	-1,06614	-0,990902	-0,481537	NaN	NaN	NaN	NaN
-0,108843	-0,81201	NaN	-0,141516	-0,332409	-0,200214	NaN	NaN	-0,797988	-0,901019
NaN	NaN	NaN	0,509178	-2,46748	-0,193895	0,575441	NaN	NaN	NaN
0,769451	1,32571	0,556787	2,55093	-0,634392	3,07399	3,26555	-0,0638218	0,114984	-0,275895
1,81235	2,62818	2,79485	2,76214	0,64233	3,31026	3,81676	1,38161	1,93958	1,44896
2,0666	3,03861	2,67082	3,62556	3,01141	3,04969	4,26883	2,90487	3,00297	3,38636
NaN	-1,28345	-3,17924	-1,16519	-1,54622	NaN	0,542158	NaN	NaN	-1,41715

Polito et al. Universal ready-to-use immunotherapeutic approach for the treatment of cancer: expanded and activated polyclonal γδ memory T cells.
Phosphorylation sites

0,883337	0,311531	0,0907135	2,56234	1,03397	3,00171	0,985334	0,495928	0,803439	0,317904
-1,61913	-2,16501	-1,50745	-0,82618	-0,802828	NaN	NaN	-1,15419	-1,33724	-0,185927
NaN	NaN	NaN	NaN	NaN	NaN	NaN	NaN	NaN	NaN
NaN	NaN	NaN	NaN	NaN	NaN	NaN	NaN	NaN	NaN
NaN	NaN	NaN	NaN	NaN	NaN	NaN	NaN	NaN	NaN
NaN	NaN	NaN	-0,461012	NaN	-2,3767	-0,056675	NaN	NaN	NaN
-2,37376	-2,16683	NaN	-1,53483	NaN	-1,10935	-0,0828514	NaN	NaN	NaN
NaN	NaN	NaN	-2,45596	-2,15551	NaN	-1,8354	NaN	NaN	NaN
NaN	NaN	NaN	-2,46294	NaN	-1,4884	-1,67282	NaN	NaN	NaN
NaN	NaN	NaN	-1,99265	NaN	-2,36443	-2,03882	NaN	NaN	NaN
NaN	NaN	NaN	NaN	NaN	NaN	NaN	NaN	NaN	NaN
NaN	NaN	NaN	NaN	NaN	NaN	NaN	NaN	NaN	NaN
NaN	NaN	NaN	NaN	NaN	NaN	NaN	NaN	NaN	NaN
NaN	NaN	NaN	NaN	NaN	NaN	NaN	NaN	NaN	NaN
NaN	NaN	NaN	NaN	NaN	NaN	NaN	NaN	NaN	NaN
1,3626	0,925777	1,53399	2,32782	1,44188	2,04373	2,68295	1,57166	1,46591	2,06198
-1,59352	-1,69781	-1,30001	-0,0583115	NaN	-0,321299	-0,510893	NaN	NaN	NaN
-0,497244	-0,81513	-0,82753	-0,315655	-0,626995	-0,620464	0,268284	-2,47315	-2,47087	-1,54941
1,05644	1,83302	0,624485	0,793579	-0,219221	0,762762	1,82201	0,69099	1,75292	1,47998
3,15182	2,93373	3,6978	2,63092	3,10307	3,45683	2,01472	2,71989	2,51765	2,5812
0,916708	0,647625	NaN	NaN	1,42873	1,73016	NaN	1,78745	1,08511	1,775
1,97362	0,397066	1,2325	1,73823	NaN	1,73016	1,81097	NaN	0,469045	0,466491
-0,482075	NaN	NaN	0,11644	-0,736237	-0,878567	0,616148	-0,334196	-0,632583	-1,27709
-0,153751	0,870443	-0,325493	0,0556068	0,96365	NaN	1,48461	-0,321268	-0,226386	0,545032
-1,17114	-1,24675	-1,1887	-1,24865	NaN	NaN	NaN	-1,18426	-1,39184	NaN
-1,75056	-0,0475578	-1,69367	NaN	NaN	NaN	-0,758043	NaN	NaN	NaN
4,60228	4,721	4,47963	4,41141	4,31834	4,51118	4,46037	4,65328	4,45879	4,09539
1,76768	1,92459	1,84365	1,75826	1,91747	1,91501	2,10049	2,28788	2,19955	1,63247
NaN	NaN	NaN	-0,998951	NaN	-0,834499	-1,20249	-2,04337	-2,04045	-2,09145
-1,72016	-2,09432	-2,17816	-0,828724	-1,70568	-0,369375	-0,45726	-1,37621	-1,86003	-1,45114
0,293053	-0,395147	-0,407349	0,567276	0,674288	0,972843	1,40541	NaN	NaN	NaN
0,287851	0,322784	0,46138	0,64292	0,312611	1,20975	NaN	NaN	-0,0414648	-0,619779
-2,17209	-0,886976	-2,45374	-2,07467	-0,598475	-2,40302	-1,01645	-0,784748	-1,77847	-0,708009
1,29107	1,30076	0,854538	1,34391	1,19873	0,948336	1,39236	0,63308	0,522407	1,17138

Polito et al. Universal ready-to-use immunotherapeutic approach for the treatment of cancer: expanded and activated polyclonal γδ memory T cells.
Phosphorylation sites

NaN	NaN	NaN	-1,88485	-2,37396	NaN	-2,36015	NaN	NaN	NaN
NaN	NaN	NaN	2,13078	1,2222	2,66987	1,4358	NaN	NaN	NaN
NaN	NaN	NaN	NaN	NaN	NaN	NaN	NaN	NaN	NaN
NaN	NaN	NaN	NaN	NaN	NaN	NaN	NaN	NaN	NaN
-0,710047	NaN	0,0176754	1,30158	-0,0716972	1,03071	1,34327	1,29542	1,03405	1,10976
NaN	NaN	NaN	NaN	NaN	NaN	NaN	-1,61043	-1,95702	-0,944921
-0,532354	NaN	0,862953	0,603901	-0,131361	0,958054	-1,3947	0,781385	0,211574	-0,476041
NaN	NaN	NaN	NaN	NaN	NaN	NaN	NaN	NaN	NaN
NaN	NaN	NaN	-1,49771	NaN	-1,45021	-1,62028	NaN	NaN	NaN
-1,78868	-2,21585	-1,54351	NaN	-2,05663	NaN	NaN	NaN	NaN	NaN
NaN	NaN	NaN	NaN	-2,5325	NaN	-1,5874	NaN	NaN	NaN
-0,334196	-0,491508	0,152145	0,512146	0,0498238	0,771423	0,343479	-0,278133	-0,807252	-0,663201
NaN	-3,81548	NaN	NaN	NaN	NaN	NaN	NaN	NaN	NaN
NaN	NaN	NaN	-2,29801	-2,32908	NaN	-1,66146	NaN	NaN	NaN
NaN	NaN	-1,38398	-1,2593	-1,10945	-2,57243	0,1411	NaN	NaN	NaN
NaN	-2,16124	-1,80022	-0,227701	NaN	-1,05897	0,0923996	-1,81717	-0,173804	-0,820107
2,56844	3,2847	-1,02607	-1,49968	3,49718	NaN	NaN	2,59215	-3,58964	3,79713
NaN	NaN	NaN	NaN	NaN	NaN	NaN	NaN	NaN	NaN
NaN	NaN	NaN	NaN	NaN	NaN	NaN	NaN	NaN	NaN
NaN	NaN	NaN	NaN	NaN	NaN	NaN	NaN	NaN	NaN
NaN	NaN	NaN	NaN	NaN	NaN	NaN	NaN	NaN	NaN
NaN	NaN	NaN	-1,28922	NaN	-0,734112	-1,66287	NaN	NaN	NaN
NaN	NaN	NaN	NaN	NaN	NaN	NaN	NaN	NaN	NaN
-0,696854	-2,15381	-0,740105	-1,70221	-1,24993	NaN	-0,961586	-2,05048	NaN	-1,63734
NaN	NaN	-1,53065	-1,06926	-1,48852	NaN	0,151075	-1,68891	NaN	-1,91579
2,54866	NaN	2,00955	2,68114	NaN	1,79603	3,24974	1,30867	1,03335	1,27553
0,783136	1,23643	0,732784	2,02093	1,83986	1,74475	3,75424	1,24969	0,834823	1,37639
3,40063	2,96939	2,86493	3,66622	4,30919	3,63552	4,68991	3,20066	2,90265	3,01213
1,52303	NaN	-2,16403	NaN	2,13187	-2,44873	-0,237368	1,226	NaN	1,25202
NaN	NaN	NaN	-1,35207	-4,03235	-3,30525	-1,27349	NaN	NaN	NaN
NaN	NaN	NaN	-0,306305	-1,55756	-0,203384	0,600685	NaN	NaN	NaN
-2,0315	-2,4446	-1,88075	NaN	-1,46659	-3,70114	NaN	-1,41041	-1,04946	-1,21525
-0,144075	-1,34436	-0,978436	-1,76536	1,01257	-2,27744	-0,839355	-0,626627	-0,457572	-0,906171
2,86231	2,16885	3,31756	3,05241	NaN	2,96413	3,02963	3,04449	2,84924	1,94312

Polito et al. Universal ready-to-use immunotherapeutic approach for the treatment of cancer: expanded and activated polyclonal γδ memory T cells.
Phosphorylation sites

-0,294344	0,423904	-1,12754	-0,592564	-0,0156384	-0,818497	-0,454103	-0,266325	0,0855207	-0,618052
0,856098	0,386141	-0,202223	NaN	NaN	NaN	NaN	NaN	NaN	NaN
NaN	NaN	NaN	NaN	NaN	NaN	NaN	NaN	NaN	NaN
NaN	NaN	NaN	-2,72227	-1,55313	NaN	-1,97954	NaN	NaN	NaN
-1,13564	-1,86303	-1,79308	-1,32746	-1,15285	-2,91817	-1,23022	-1,30115	-1,23368	-1,76763
1,45008	1,94775	1,09766	1,74834	2,19215	0,535513	2,04931	1,77992	1,56939	1,27335
NaN	NaN	NaN	NaN	NaN	NaN	NaN	NaN	NaN	NaN
NaN	NaN	NaN	NaN	NaN	NaN	NaN	-1,54099	-1,69921	-1,70068
NaN	NaN	NaN	NaN	NaN	NaN	NaN	-1,6857	-2,2198	-1,25693
-1,0004	-0,450911	-1,26801	0,215626	-1,07448	0,25082	0,583115	-0,468452	-0,833289	-0,868854
-1,80833	-2,07253	NaN	-1,22499	-2,77026	NaN	-0,704512	NaN	NaN	NaN
-0,0879593	-0,403097	-0,49655	0,363762	NaN	0,162678	0,561518	-0,153608	-0,2552	0,0817366
-0,155714	0,240257	-0,455513	0,633945	0,788668	-0,239145	1,24985	0,267513	0,0321054	0,429378
NaN	NaN	NaN	-1,6212	NaN	0,46451	-1,64096	NaN	NaN	NaN
-1,75695	NaN	NaN	-1,15226	-0,499249	NaN	-0,535753	NaN	NaN	NaN
1,37073	-0,00973892	1,26875	0,37117	2,1565	-0,16217	-0,121931	0,743942	0,344213	0,233502
NaN	NaN	NaN	NaN	NaN	NaN	NaN	-1,4071	-1,95456	-1,65566
NaN	NaN	NaN	-0,865294	NaN	-0,511803	-0,782743	NaN	NaN	NaN
NaN	NaN	NaN	1,84642	0,197966	1,17063	2,4876	0,829956	0,405526	0,988931
NaN	NaN	NaN	-0,573263	-1,25101	0,854494	-2,09901	NaN	NaN	NaN
NaN	NaN	NaN	-1,23738	-3,0495	-0,246235	-0,605953	-0,656981	-0,935723	-1,12941
NaN	NaN	NaN	-1,71422	NaN	-1,9174	-2,58304	NaN	NaN	NaN
NaN	NaN	NaN	NaN	NaN	NaN	NaN	-1,52396	-1,93263	-1,61714
0,622257	-1,22963	0,631891	NaN	NaN	NaN	NaN	0,362415	0,638412	0,404004
NaN	-0,70582	NaN	NaN	-0,303137	-0,371622	-0,158228	-1,40235	-0,404052	-4,02923
NaN	NaN	NaN	-2,05399	-2,77773	-1,74008	-1,8418	NaN	NaN	NaN
NaN	NaN	NaN	-1,46096	NaN	-0,59273	-0,813047	NaN	NaN	NaN
NaN	NaN	NaN	-2,69845	-3,20633	-2,39417	-2,93699	NaN	NaN	NaN
NaN	NaN	NaN	NaN	NaN	NaN	NaN	NaN	NaN	NaN
-0,35323	-1,21498	0,29084	-0,567286	-0,241636	NaN	0,256529	0,726713	0,360757	0,0321684
-2,14341	NaN	0,95647	1,90178	1,07409	2,19277	1,37006	1,39827	NaN	1,2076
0,471144	0,422859	0,905329	0,253189	0,745056	NaN	0,761473	0,654312	0,436271	0,232388
NaN	NaN	NaN	NaN	NaN	NaN	NaN	NaN	NaN	NaN
1,0588	1,96856	2,1509	1,76287	1,89035	0,797169	1,34582	1,38639	1,16576	1,47965

Polito et al. Universal ready-to-use immunotherapeutic approach for the treatment of cancer: expanded and activated polyclonal γδ memory T cells.
Phosphorylation sites

NaN	NaN	NaN	NaN	NaN	NaN	NaN	NaN	NaN	NaN
-0,40333	NaN	NaN	0,545275	NaN	NaN	1,10787	NaN	NaN	NaN
1,94892	1,6809	2,12766	1,71698	2,58796	0,463554	2,33381	2,16679	1,76552	1,78496
NaN	NaN	NaN	0,125433	0,428797	-0,0936928	1,43423	-1,1568	-0,0597105	-0,384133
-1,37706	-0,841267	-1,19558	-1,60641	-2,26195	-1,59666	-0,807678	-0,881834	-0,52798	-0,288199
0,668306	0,512625	0,568413	NaN	0,273371	NaN	NaN	-0,199699	-0,428603	-0,669648
-2,68687	1,21618	-0,722897	NaN	NaN	-0,355494	NaN	NaN	-0,999652	NaN
NaN	NaN	NaN	NaN	-1,50513	-3,09784	NaN	-1,52619	-1,7132	-2,16221
NaN	NaN	NaN	0,57889	-1,51769	-0,257221	NaN	NaN	NaN	NaN
NaN	NaN	NaN	-0,744514	-2,28504	-0,413065	-0,567162	NaN	NaN	NaN
1,37607	1,80886	1,614	2,48176	2,10779	1,98927	2,4559	1,54455	1,31137	1,64294
NaN	NaN	NaN	-1,72889	-3,16829	-1,95575	-1,30616	-1,94559	-2,04383	-1,96109
1,43091	1,00724	1,22	2,70265	1,74601	2,9199	1,68361	1,10331	0,872052	0,641736
NaN	-1,1362	NaN	NaN	NaN	NaN	NaN	NaN	NaN	NaN
-0,0320034	NaN	-1,89921	0,241171	0,906824	NaN	-0,366302	NaN	NaN	-0,870278
0,0298004	0,430393	NaN	-0,207758	-1,78459	-0,642725	-0,285202	NaN	NaN	NaN
NaN	NaN	NaN	NaN	NaN	NaN	NaN	-1,24155	-1,63106	-1,44369
0,317904	0,924135	0,543993	0,0181046	0,767529	-2,17199	0,581429	0,973417	0,661803	0,652024
7,57737	6,08645	6,32998	5,96402	6,26133	2,8952	1,39941	6,50114	6,52398	NaN
2,76562	1,65699	1,93878	1,9772	2,38966	2,35437	1,99954	2,22231	2,04084	1,76612
NaN	NaN	NaN	NaN	NaN	NaN	NaN	NaN	NaN	NaN
NaN	NaN	NaN	NaN	NaN	NaN	NaN	NaN	NaN	NaN
NaN	NaN	NaN	0,56988	NaN	0,620356	-0,496382	NaN	NaN	NaN
NaN	NaN	NaN	NaN	NaN	NaN	NaN	NaN	NaN	NaN
NaN	NaN	NaN	NaN	NaN	NaN	NaN	NaN	NaN	NaN
NaN	NaN	NaN	-2,03503	-1,33524	-0,459766	NaN	-1,17815	-1,4027	-1,07763
NaN	NaN	NaN	-0,417374	-0,882671	-0,567614	-1,06294	0,208965	-0,00874233	-0,689496
NaN	NaN	NaN	-0,243065	-1,96264	-0,126543	-0,591805	NaN	NaN	NaN
NaN	NaN	NaN	NaN	NaN	NaN	NaN	NaN	NaN	NaN
NaN	NaN	NaN	0,247858	NaN	-1,43538	-1,33688	NaN	NaN	NaN
2,30581	2,25123	1,45748	2,4021	2,51478	2,50402	2,91768	1,70539	0,860578	1,32189
-1,32922	-0,672316	-0,594959	-0,00194931	-0,291607	-2,19777	-0,0416012	-0,890316	-0,857425	-1,22188
NaN	NaN	NaN	-1,45909	-3,06286	-1,76957	NaN	NaN	NaN	NaN
1,62829	1,45838	1,56856	1,97683	1,82372	2,5426	1,78546	0,851128	0,929713	0,568496

Polito et al. Universal ready-to-use immunotherapeutic approach for the treatment of cancer: expanded and activated polyclonal γδ memory T cells.
Phosphorylation sites

NaN	NaN	NaN	NaN	NaN	NaN	NaN	NaN	NaN	NaN
NaN	-2,72892	NaN	0,742416	-1,16078	-1,10734	-0,764709	NaN	NaN	NaN
NaN	NaN	NaN	NaN	NaN	NaN	NaN	NaN	NaN	NaN
1,56791	1,89268	1,57959	2,28964	2,26435	2,29395	3,05335	1,49409	1,67535	1,52732
NaN	NaN	NaN	NaN	NaN	NaN	NaN	NaN	NaN	NaN
NaN	NaN	NaN	NaN	NaN	NaN	NaN	NaN	NaN	NaN
-2,58412	NaN	-0,595182	0,97051	-1,06612	0,353809	0,267189	NaN	NaN	NaN
NaN	NaN	NaN	NaN	NaN	NaN	NaN	NaN	NaN	NaN
NaN	NaN	NaN	0,482323	NaN	-0,20919	-1,32855	NaN	NaN	NaN
NaN	NaN	-2,14544	-2,25044	-2,01788	-2,83236	NaN	-2,82974	-2,93754	NaN
0,0522099	0,97788	-0,707411	0,973885	0,533709	0,00131798	1,27998	-1,06247	-0,395934	-0,0698805
NaN	NaN	NaN	NaN	NaN	NaN	NaN	NaN	NaN	NaN
NaN	NaN	NaN	-0,156395	-1,66301	0,388643	-0,966293	NaN	NaN	NaN
NaN	0,18487	1,04121	0,667316	0,249603	NaN	1,01029	0,15066	-0,609118	-0,291961
NaN	NaN	NaN	NaN	NaN	NaN	NaN	NaN	NaN	NaN
-0,415693	-0,290783	0,326233	-0,146248	1,14832	-0,245098	-0,577095	0,431585	0,0625505	0,178008
NaN	NaN	NaN	-0,350151	-0,333483	NaN	NaN	NaN	NaN	NaN
NaN	NaN	NaN	NaN	NaN	NaN	NaN	NaN	NaN	NaN
NaN	NaN	NaN	-1,97563	-1,57702	0,852703	-0,180849	NaN	NaN	NaN
-1,57837	-1,87076	NaN	-1,68491	NaN	NaN	-0,486736	-1,08796	-1,65185	-1,13097
NaN	NaN	-1,42887	-2,46324	NaN	-1,41093	-2,0497	NaN	NaN	NaN
NaN	NaN	NaN	NaN	NaN	NaN	NaN	NaN	NaN	NaN
NaN	NaN	NaN	0,263762	NaN	0,208303	0,0792618	-0,00787926	-0,239135	-0,213416
3,69406	3,14485	3,75322	2,26851	4,39993	1,78063	2,06622	4,24322	3,76236	4,51716
NaN	NaN	NaN	0,362045	NaN	-2,28035	-0,217146	NaN	NaN	NaN
NaN	NaN	NaN	NaN	NaN	NaN	NaN	NaN	NaN	NaN
NaN	NaN	NaN	NaN	NaN	-1,03734	-1,12398	NaN	NaN	NaN
NaN	NaN	-2,07578	-2,40095	NaN	-1,13124	-2,55414	NaN	NaN	NaN
-0,6448	NaN	2,27966	3,62545	-3,61817	4,08179	2,70926	-1,00002	-0,907481	-1,31045
-0,370815	-0,842999	-2,01575	-0,0101261	NaN	NaN	1,50515	-0,111637	-0,287177	-0,644355
-0,479427	NaN	0,936119	1,89734	NaN	1,66846	1,64526	NaN	NaN	NaN
-0,343313	0,226475	0,836489	1,61756	1,19327	3,1908	1,98081	-0,53726	-0,656531	0,121749
0,457359	1,29328	0,544531	0,184649	1,51184	1,89146	1,00194	0,362162	0,106513	0,53721
0,144716	-1,91837	0,732401	-1,26777	0,366095	-0,273497	-0,172235	-0,738321	-0,918874	-0,771504

Polito et al. Universal ready-to-use immunotherapeutic approach for the treatment of cancer: expanded and activated polyclonal γδ memory T cells.
Phosphorylation sites

NaN	NaN	NaN	NaN	NaN	NaN	NaN	-1,43293	-1,44155	-1,46007
0,0650043	-0,562387	-0,79212	0,661633	NaN	0,383234	-0,0207977	NaN	NaN	NaN
0,0650043	-0,562387	-0,79212	0,661633	0,687996	0,383234	NaN	NaN	NaN	NaN
NaN	NaN	NaN	NaN	-2,44999	-2,43111	-0,709253	-2,01658	-1,42235	NaN
4,27679	4,49641	4,63572	4,05774	5,21795	3,84599	4,45121	4,92052	4,69501	4,53736
2,31889	1,4403	2,71294	4,22582	2,09793	NaN	2,989	2,88977	2,48571	2,29493
1,59602	2,01847	2,57327	2,99269	2,00359	-0,121841	2,66502	2,06603	2,28615	2,01659
-1,61934	-1,09093	-1,2382	NaN	-2,71362	1,54166	0,713354	NaN	NaN	NaN
NaN	NaN	NaN	-1,74698	NaN	-1,82687	0,887619	NaN	NaN	NaN
NaN	NaN	NaN	0,929695	NaN	0,701591	-1,08065	NaN	NaN	NaN
NaN	NaN	NaN	0,496334	-2,64041	-0,0408363	NaN	NaN	NaN	NaN
0,348133	-0,249474	1,32008	4,47288	0,041975	4,69108	2,53151	NaN	NaN	-1,1218
0,614273	0,964531	0,659529	-0,138012	NaN	-1,87458	0,238098	-0,106983	-0,426974	-0,17555
NaN	NaN	NaN	-0,549797	-1,68597	0,372999	-2,0293	NaN	NaN	NaN
0,295637	0,179928	0,114697	0,736912	0,763346	-0,0741329	0,830582	-0,203371	-0,608304	-0,273894
0,203779	-1,07329	0,153423	1,19434	0,721386	NaN	1,25314	0,823206	0,654395	0,950736
1,01309	-0,284792	0,271513	-0,648203	1,7219	-0,287003	-0,813637	0,852095	0,534776	0,849172
-0,892464	-0,494097	NaN	-2,09699	0,170364	NaN	-1,15275	-0,881603	-1,14302	-1,39393
NaN	NaN	NaN	-3,36937	-1,92049	-2,66273	NaN	NaN	NaN	NaN
NaN	NaN	NaN	NaN	NaN	NaN	NaN	NaN	NaN	NaN
-0,996033	-0,632696	-1,60363	1,58215	0,0554981	NaN	1,00328	0,226019	NaN	-0,510377
NaN	NaN	NaN	1,34311	-0,765083	-0,393303	0,603497	-0,835564	NaN	-0,589282
NaN	NaN	NaN	-2,01142	-1,159	NaN	NaN	NaN	NaN	NaN
-0,649448	-0,0317535	NaN	-0,43317	-1,21394	-0,937937	-2,63193	NaN	NaN	NaN
-0,260422	-0,313389	NaN	-1,10444	NaN	-1,69508	-1,28607	-0,0243225	-0,617055	-0,633206
NaN	NaN	NaN	-0,48181	NaN	-0,572807	-2,03186	NaN	NaN	NaN
2,82755	2,03738	2,32479	2,66451	1,35451	NaN	2,65413	0,939745	2,23457	1,46667
NaN	NaN	NaN	-2,91607	-2,77121	-2,19058	-2,46643	NaN	NaN	NaN
NaN	NaN	NaN	0,769304	NaN	1,1337	-0,885633	NaN	NaN	NaN
-1,33811	NaN	-0,913074	-0,365866	-1,89256	NaN	-1,00492	-1,37615	-2,72644	-1,73243
-1,59446	-0,475891	-1,59861	NaN	NaN	0,341661	NaN	NaN	NaN	NaN
0,812283	-0,833641	0,138269	-0,370945	0,325375	-1,64873	0,507223	0,506115	0,310079	-0,0684843
NaN	NaN	NaN	-1,25899	NaN	0,0225906	-0,924255	NaN	NaN	NaN
-2,00045	-2,40983	-1,78261	NaN	NaN	0,286922	NaN	NaN	NaN	NaN

-1,54152	-2,13453	-1,96034	-0,1292	-0,990732	2,04318	-1,17842	NaN	NaN	NaN
1,1113	-0,789619	-0,649607	1,61204	0,555578	NaN	1,07515	0,938444	0,900786	0,881045
3,80009	3,89674	3,29545	3,75336	1,49035	5,39135	3,10187	3,03937	3,52037	1,58193
NaN	NaN	2,05688	0,607405	NaN	-0,325047	0,288132	NaN	NaN	-0,907418
2,91854	2,83464	3,05116	3,33758	3,66088	NaN	3,47791	3,62685	3,45402	2,91756
NaN	NaN	NaN	2,89191	NaN	-0,422012	-1,46649	NaN	NaN	NaN
-0,486916	2,15953	-0,0243893	0,386721	0,673058	1,55624	0,75989	-0,125093	-0,31533	0,162364
NaN	0,144569	NaN	0,266836	NaN	0,389376	0,296671	NaN	NaN	NaN
NaN	NaN	-2,57708	-2,20413	-1,82906	-1,18088	NaN	-1,1755	-2,40378	-1,02088
NaN	NaN	NaN	-2,20422	NaN	NaN	-1,81544	NaN	NaN	NaN
NaN	-4,14851	-1,3677	-2,70514	-3,08708	-0,300961	-1,66613	-3,51326	NaN	-3,16264
NaN	-3,66343	-0,521862	-1,25956	-2,21896	1,08154	-0,8512	NaN	NaN	NaN
-1,5797	0,237749	-2,262	NaN	-0,850531	NaN	NaN	NaN	-2,05146	-0,104177
NaN	NaN	NaN	NaN	NaN	0,000598907	NaN	NaN	NaN	NaN
-1,01066	0,452078	NaN	-2,43872	-0,313559	NaN	-2,57543	-1,07776	-0,843741	-0,883554
-1,66327	-1,91884	-0,335514	-1,1769	-1,22373	NaN	-0,886087	-0,743782	-0,506116	-1,03309
-2,90842	-2,07404	-1,58031	-2,89979	-1,21321	NaN	-1,59164	-2,11201	NaN	-1,79087
0,678663	0,144709	0	-1,37416	NaN	-1,24655	NaN	-1,77875	-2,02493	NaN
4,13941	3,81232	4,23133	4,31838	3,04273	4,67241	4,13634	3,14933	3,69749	2,8859
0,27182	0,0501518	0,640619	-0,36762	1,45697	0,351839	0,339636	1,69826	1,01631	1,1121
NaN	NaN	NaN	-1,09782	NaN	-0,405855	-1,42636	NaN	NaN	NaN
-0,61175	-1,02554	NaN	0,24905	-0,133606	-0,208498	0,498613	-0,83112	-0,966451	-0,629403
-0,965202	0,0478783	NaN	-1,18219	-1,2368	NaN	-1,04268	-0,98484	-0,117808	-0,235564
2,20805	-1,0673	2,53735	NaN	3,10155	0,227365	NaN	-5,72639	NaN	2,36579
NaN	NaN	NaN	-1,03259	-1,93901	0,0203476	-0,699713	NaN	NaN	NaN
NaN	NaN	NaN	0,531605	NaN	0,545317	-0,20388	NaN	NaN	NaN
NaN	NaN	NaN	-2,58977	NaN	-1,59275	-1,67553	NaN	NaN	NaN
NaN	NaN	NaN	NaN	NaN	NaN	NaN	NaN	NaN	NaN
-1,28439	-2,978	-1,7164	NaN	-1,23526	NaN	NaN	-0,925365	-1,42114	-1,92945
-1,88482	-2,05288	-1,42813	-1,01224	-1,56293	-2,03845	-0,555882	-1,77635	-1,99838	-2,28099
1,58847	2,67401	2,6297	-0,511847	1,34749	NaN	1,83854	1,75286	2,58868	2,09302
1,80834	2,35393	1,74656	-0,645842	1,97067	NaN	0,165594	1,40967	2,15903	1,39178
NaN	NaN	NaN	-0,703196	NaN	-0,776159	-0,510702	NaN	NaN	NaN
NaN	NaN	NaN	-0,911432	-3,85131	-0,454929	-1,65195	NaN	NaN	NaN

-0,156633	-0,665382	NaN	-0,961885	-0,223166	NaN	-0,310419	-3,55147	-0,420674	-0,72441
-1,63423	NaN	-1,59157	NaN	NaN	NaN	NaN	-1,53332	-1,49096	-2,57052
NaN	NaN	NaN	NaN	NaN	NaN	NaN	NaN	NaN	NaN
-0,367586	-0,863571	0,0662632	0,576551	-0,424652	1,55652	0,234663	-0,156136	-0,477908	-0,862454
NaN	NaN	NaN	-1,26839	NaN	NaN	-0,792349	NaN	NaN	NaN
NaN	NaN	NaN	NaN	NaN	NaN	NaN	NaN	NaN	NaN
2,54836	2,55999	2,85057	3,44182	2,43042	4,40697	3,29622	2,63225	2,40259	2,36757
-1,02868	-1,69101	-0,897985	-0,798229	-1,22619	-0,253359	-0,50362	-1,43968	-1,3405	-1,10635
NaN	-1,47355	NaN	NaN	NaN	-0,744776	NaN	-0,667362	-0,982734	-0,890004
NaN	NaN	-0,793884	-1,30958	NaN	-1,54158	-0,811909	-1,94732	-1,39534	-1,66611
0,372492	0,741833	0,249556	-1,52543	-0,373404	-0,140213	-0,63978	-0,449425	-0,0102911	-0,236672
NaN	NaN	NaN	NaN	NaN	NaN	NaN	NaN	NaN	NaN
NaN	NaN	NaN	NaN	NaN	NaN	-2,23106	NaN	NaN	NaN
NaN	NaN	NaN	-0,71558	NaN	-0,0889683	-2,50257	NaN	NaN	NaN
NaN	NaN	NaN	1,27502	-2,75352	1,46264	-0,873505	NaN	NaN	NaN
NaN	NaN	NaN	0,909285	NaN	0,895267	-0,356113	NaN	NaN	NaN
NaN	NaN	NaN	-0,512737	NaN	-2,92726	-1,27966	NaN	NaN	NaN
2,29912	0,57889	1,89682	2,42124	1,3037	NaN	2,34129	2,54301	2,31009	2,83364
-1,34354	-1,61809	-1,5312	-0,4795	-0,376869	-2,60007	-1,01654	-1,47135	-1,44038	-0,802909
1,12825	NaN	1,01701	1,8454	1,4448	-1,6778	2,09835	0,825741	1,10965	0,346686
-0,41254	-0,687239	-1,17385	-0,721449	-0,706377	NaN	-0,847214	-1,00648	-1,20465	-1,48974
1,46697	0,612087	0,475092	1,66911	1,59353	2,25645	1,98556	1,35971	0,570292	2,4426
NaN	NaN	NaN	-0,701313	NaN	-1,31392	-0,869701	NaN	NaN	NaN
0,791285	NaN	2,9159	2,67755	0,717016	1,95435	2,42435	1,52692	1,56607	1,12804
NaN	NaN	-1,61352	-0,732889	-2,3507	-1,79201	-0,555782	NaN	NaN	NaN
-2,72966	-1,89831	-1,23186	-1,28832	-3,95685	-1,95561	-1,93033	-4,57324	-1,92692	-1,83866
NaN	NaN	NaN	-1,85236	-0,880943	-1,24118	-0,127657	NaN	NaN	NaN
2,79122	4,26927	3,46493	3,54636	3,32712	4,428	3,79757	2,18808	3,04676	3,24391
NaN	NaN	NaN	-1,56611	NaN	-1,0067	-3,73501	NaN	NaN	NaN
NaN	NaN	NaN	-1,43762	NaN	-1,29275	-1,02634	NaN	NaN	NaN
NaN	NaN	NaN	1,53554	NaN	-0,0742588	1,05098	NaN	NaN	NaN
NaN	NaN	NaN	-0,350527	NaN	NaN	-0,993311	NaN	NaN	NaN
NaN	NaN	NaN	NaN	NaN	NaN	NaN	NaN	NaN	NaN
-0,605045	-1,72027	-0,0966415	-0,417545	0,109259	-0,142725	-1,29257	-0,450548	NaN	-1,10201

Polito et al. Universal ready-to-use immunotherapeutic approach for the treatment of cancer: expanded and activated polyclonal γδ memory T cells.
Phosphorylation sites

NaN	NaN	NaN	NaN	NaN	NaN	NaN	NaN	NaN	NaN
NaN	NaN	NaN	NaN	NaN	NaN	NaN	NaN	NaN	NaN
-0,608139	-0,470634	-0,812674	NaN	NaN	NaN	NaN	NaN	NaN	NaN
0,719223	0,425993	1,03976	0,795486	1,43443	NaN	1,4055	1,28829	0,914887	0,252505
NaN	NaN	NaN	NaN	NaN	NaN	NaN	NaN	NaN	NaN
3,14739	3,76647	3,68676	3,9699	4,47881	3,25509	3,72317	3,79012	3,41587	3,98952
-1,25868	-1,24875	-0,550602	-0,654757	-0,383865	-1,03245	-0,355312	-0,462872	-0,657048	-0,829986
NaN	NaN	NaN	NaN	NaN	NaN	NaN	NaN	NaN	NaN
1,31342	1,76035	0,94187	1,43021	1,27943	1,89279	1,59429	1,07908	1,13919	0,759971
-1,91396	-3,53543	-1,30393	-0,202501	-2,03514	-0,866478	-0,670288	-1,72136	-2,49807	-1,96029
-2,00045	-1,51783	-2,67385	-2,04251	-1,38806	NaN	NaN	NaN	NaN	-1,31439
-1,58807	-1,03371	-1,18417	NaN	NaN	-0,52178	-0,703421	NaN	NaN	-0,732318
NaN	NaN	NaN	NaN	-2,04743	-3,5068	-0,920485	NaN	NaN	NaN
NaN	NaN	NaN	NaN	-0,89769	0,275087	-0,525455	NaN	NaN	NaN
-1,98217	NaN	NaN	-0,61787	-0,812283	-1,31461	-1,25796	-1,22717	-1,92163	-1,80547
-2,90507	NaN	NaN	NaN	-1,23406	-1,44172	NaN	-2,35105	-1,46135	-1,68296
-1,19731	-2,67003	0,679255	1,38468	NaN	3,13719	0,199366	-0,387003	0,17578	-1,3961
-1,70356	-2,47572	0,020771	-0,817245	-5,13251	NaN	-0,47871	-1,92897	-1,57695	-2,15249
-0,475883	NaN	NaN	2,13723	1,42884	2,51024	2,27727	1,54669	1,24188	0,962678
-1,99288	-2,50522	-1,3654	-0,726154	-1,34626	-1,64974	-1,27305	-0,945065	-1,25105	-1,44275
NaN	NaN	NaN	-1,24625	-1,75186	-0,275961	-0,67499	-1,07077	-1,41407	-1,235
-0,429365	-1,30027	NaN	NaN	NaN	0,805626	0,27001	-0,371754	-0,900764	NaN
NaN	NaN	NaN	NaN	NaN	NaN	NaN	NaN	NaN	NaN
NaN	NaN	NaN	-2,43136	NaN	-1,85846	-2,64515	NaN	NaN	NaN
NaN	NaN	NaN	-1,12494	NaN	-0,811245	-0,717646	-2,4449	-2,35062	-2,52648
NaN	-2,74085	-0,789516	-0,10977	NaN	2,32049	-0,412552	NaN	-1,02547	NaN
NaN	NaN	NaN	-3,30034	NaN	-1,55529	-2,33908	NaN	NaN	NaN
-0,735872	-2,27554	0,00763321	-1,88607	NaN	NaN	-0,822067	0,0783234	0,0459986	-0,0643988
0,237282	NaN	0,840637	NaN	NaN	NaN	NaN	1,12234	0,446315	0,551747
NaN	NaN	NaN	-1,16804	-3,62454	-1,10445	-1,29685	-2,69502	-2,84526	-2,92194
-1,8655	-3,31046	-1,6044	NaN	NaN	NaN	-2,16884	NaN	-0,878272	-1,61201
NaN	NaN	NaN	NaN	NaN	NaN	NaN	NaN	NaN	NaN
NaN	NaN	NaN	-1,32862	NaN	-1,85694	-0,877111	NaN	NaN	NaN
-2,17552	-1,67684	-2,214	-0,784864	-1,37665	NaN	-0,848743	-0,980799	-1,82654	-1,84409

NaN	NaN	NaN	-1,54692	-1,5194	-2,05483	-1,6395	NaN	NaN	NaN
NaN	NaN	NaN	1,68617	NaN	1,73441	0,249027	NaN	NaN	NaN
NaN	NaN	NaN	-2,02572	NaN	-0,88632	-1,81105	NaN	NaN	NaN
NaN	NaN	NaN	NaN	-0,886293	NaN	-1,26535	-2,24096	NaN	-1,26276
-2,01102	-0,681631	-1,96928	NaN	-0,137197	-1,14315	NaN	-1,81339	-1,61853	-1,32584
NaN	NaN	NaN	0,0680218	NaN	0,728167	1,08361	NaN	NaN	NaN
NaN	NaN	NaN	NaN	NaN	NaN	NaN	NaN	NaN	NaN
NaN	NaN	NaN	-0,560003	NaN	-0,528368	-0,757023	NaN	NaN	NaN
NaN	NaN	NaN	NaN	NaN	NaN	NaN	NaN	NaN	NaN
1,52675	2,0348	-0,00969696	1,51652	1,35762	1,14517	1,20655	0,488428	1,05374	0,299954
1,78359	2,21421	1,3114	2,11449	0,558516	0,988691	1,86816	1,03424	1,28813	0,661225
NaN	NaN	NaN	NaN	NaN	NaN	NaN	NaN	NaN	NaN
NaN	NaN	NaN	-2,65149	NaN	-1,4123	-1,59656	-3,39758	NaN	-2,9637
0,683674	0,5996	0,414869	-0,00430679	0,785521	-1,40387	0,0299015	0,782907	0,166436	0,641646
-1,32251	NaN	-0,43276	NaN	-0,675941	NaN	NaN	0,317867	-0,139083	0,0176649
1,53486	1,53452	2,06136	2,02532	1,89005	-1,00988	1,89292	1,48512	1,50568	2,18648
NaN	NaN	NaN	1,49085	NaN	1,88343	0,579336	NaN	NaN	NaN
0,420628	0,818035	0,682163	0,552813	0,85207	0,895527	1,37928	1,46601	1,22715	1,20571
NaN	NaN	NaN	NaN	NaN	NaN	NaN	NaN	NaN	NaN
NaN	NaN	NaN	NaN	NaN	NaN	NaN	NaN	NaN	NaN
0,438839	0,741554	0,990686	1,18029	0,235037	0,405209	1,14138	-1,57983	1,34065	-0,882497
-1,77288	-1,68679	-1,69353	-0,991623	-1,41647	-3,06648	-1,44571	-1,87537	-1,4376	-1,61257
0,215475	0,650406	0,48353	1,55651	0,903955	0,994358	1,26537	0,251259	0,404489	0,273162
-1,63111	-1,75345	NaN	-1,06414	-2,04367	1,02377	-1,11955	NaN	NaN	NaN
-0,561901	-1,02795	-0,551907	-1,81838	0,305199	NaN	-0,399374	-0,0726604	-1,21323	-0,151084
-0,389112	1,07639	1,18416	0,493595	1,68028	-1,04106	1,51839	0,62398	0,756986	1,67427
NaN	NaN	NaN	NaN	NaN	NaN	NaN	NaN	NaN	NaN
-0,58144	-2,14173	-0,403923	0,233858	NaN	2,3814	NaN	-0,445784	-0,506087	-0,866633
-2,18746	-3,44147	-3,39988	-2,06303	-2,5904	NaN	NaN	NaN	NaN	NaN
NaN	2,65632	NaN	NaN	3,38387	2,46882	2,42144	2,26196	2,49742	2,99159
NaN	NaN	NaN	NaN	NaN	NaN	NaN	NaN	NaN	NaN
NaN	NaN	NaN	-2,36234	NaN	NaN	-1,98183	NaN	NaN	NaN
-0,272423	-0,43347	-0,627401	-0,167025	0,0703602	NaN	-0,496918	-0,865108	-1,00866	-0,834521
NaN	NaN	NaN	NaN	NaN	NaN	NaN	NaN	NaN	NaN

Polito et al. Universal ready-to-use immunotherapeutic approach for the treatment of cancer: expanded and activated polyclonal γδ memory T cells.
Phosphorylation sites

NaN	NaN	NaN	NaN	NaN	NaN	NaN	-1,61753	-0,963449	-1,7332
-1,00914	-1,54954	-1,74923	-0,39192	-1,10877	-0,596132	0,124796	-1,03354	-1,415	-1,34333
NaN	NaN	NaN	NaN	NaN	NaN	NaN	NaN	NaN	NaN
NaN	NaN	NaN	0,760975	-0,799459	0,365112	0,501093	0,186192	-0,24248	-0,307467
NaN	NaN	NaN	NaN	NaN	NaN	NaN	-1,91941	-2,24924	-2,23478
-1,93685	-3,04688	-3,44603	-1,6497	-2,47798	-1,99075	-1,58137	NaN	NaN	NaN
-1,49718	-0,4568	-2,96511	-0,998327	-1,42271	NaN	-0,893209	-0,153276	NaN	-0,177182
NaN	NaN	NaN	NaN	NaN	NaN	NaN	-2,05276	-1,87522	-2,1248
-2,11809	-1,64961	NaN	-0,886751	-1,66978	NaN	-0,94265	-1,92215	-1,96789	-1,89542
1,08039	1,18439	0,221087	0,833532	1,79411	-1,26639	1,63313	0,92103	0,911838	0,956069
-0,801697	-0,888504	-0,675678	-0,295593	-0,246487	NaN	-0,997677	-0,454302	-1,2401	-0,691386
NaN	-0,04846	-0,522257	-0,109076	-0,287401	-0,113779	0,0748978	-0,710669	-0,547721	1,05459
NaN	NaN	NaN	NaN	NaN	NaN	NaN	NaN	NaN	NaN
NaN	NaN	NaN	NaN	NaN	NaN	NaN	NaN	NaN	NaN
-0,387302	0,376692	-0,753122	0,00689697	-0,66851	-0,524225	-0,334959	-0,0418568	-0,328712	0,0898676
NaN	NaN	NaN	NaN	NaN	NaN	NaN	NaN	NaN	NaN
-0,898609	-1,73058	-0,796679	-2,34992	-0,00938606	-3,00718	-0,214369	NaN	NaN	0,918681
1,93702	1,43668	2,14827	NaN	1,93021	0,527803	1,87262	-2,52303	2,8816	2,1893
3,3387	2,16486	4,56199	3,30648	4,27792	0,479029	3,75883	4,8756	4,73075	4,01605
1,66803	0,0673523	1,21863	2,38799	1,6591	-1,69201	1,93765	0,713631	0,584449	0,217683
-0,187668	-1,42887	-0,318075	NaN	1,02548	0,623159	0,674257	-0,749432	-0,384778	-0,159057
NaN	NaN	NaN	-1,20922	-1,83442	NaN	-1,98794	NaN	NaN	NaN
4,26442	4,51404	4,25893	3,46679	5,14557	0,670683	3,70306	4,10841	3,91293	4,05357
-0,338083	NaN	1,64279	0,597719	0,106144	NaN	0,979338	NaN	-0,229918	-0,802108
0,42939	-1,94188	-0,982157	0,373444	0,63134	-0,495018	0,884476	-0,626434	-0,525411	-0,559079
2,25477	2,63218	1,85131	2,63628	2,61095	2,62745	2,46098	1,85637	1,72719	1,76268
-0,175819	1,46144	0,346331	0,574823	2,37148	1,97049	1,076	0,42979	0,326543	1,07162
NaN	NaN	NaN	NaN	0,600281	0,814714	-0,277786	NaN	-1,1082	-0,571134
NaN	NaN	NaN	-2,22839	NaN	-1,55977	-2,41011	NaN	NaN	NaN
NaN	-0,740017	NaN	-1,74132	NaN	NaN	-1,59096	NaN	NaN	NaN
NaN	NaN	NaN	-0,305418	NaN	0,690052	-1,01095	NaN	NaN	NaN
NaN	NaN	-0,870094	-1,36433	NaN	-0,414885	-0,23505	-1,44151	-1,94537	-1,72346
NaN	NaN	0,407217	-0,170599	NaN	-0,305248	-0,144453	NaN	NaN	NaN
-0,191893	-1,08138	-0,328339	-2,38281	-0,197737	-2,06127	-2,91372	-3,02938	-2,82918	-2,53857

Polito et al. Universal ready-to-use immunotherapeutic approach for the treatment of cancer: expanded and activated polyclonal γδ memory T cells.
Phosphorylation sites

-0,135223	-0,25218	NaN	NaN	0,0812855	-1,2592	-1,09423	-0,76156	-0,891908	NaN
-1,16662	-1,02983	-1,82209	-0,651087	NaN	-0,195267	-0,539743	NaN	NaN	NaN
NaN	NaN	NaN	-0,824747	NaN	NaN	-1,04854	NaN	NaN	NaN
-2,0997	-0,493088	-0,943325	-1,94983	NaN	NaN	-0,309811	NaN	NaN	NaN
-1,47281	-1,80707	-1,73908	-0,821104	NaN	0,46373	-1,7568	-1,15719	-1,66233	NaN
NaN	NaN	NaN	-1,89343	NaN	-0,0357914	-2,30843	NaN	NaN	NaN
-1,02699	-1,09652	-0,6154	1,27517	0,105246	1,6679	1,32036	-0,960464	-1,01184	-0,52012
NaN	NaN	NaN	-0,88183	-2,2561	-2,13752	-1,29701	NaN	NaN	NaN
NaN	NaN	NaN	NaN	NaN	1,39143	NaN	NaN	NaN	NaN
NaN	NaN	NaN	-1,26163	NaN	-1,52614	-0,860826	NaN	NaN	NaN
NaN	NaN	NaN	NaN	NaN	NaN	NaN	NaN	NaN	NaN
-2,05385	0,401672	-2,17395	NaN	NaN	NaN	NaN	NaN	NaN	NaN
1,72085	0,935829	NaN	1,7053	0,942293	2,43868	0,86006	NaN	NaN	NaN
-0,360374	0,129694	NaN	2,31926	1,31951	2,70025	2,41994	-2,74271	0,596028	-0,137061
NaN	NaN	-1,12676	0,657677	NaN	1,77121	0,616848	NaN	NaN	NaN
NaN	NaN	NaN	-1,60082	-1,77746	-0,722635	-1,47243	NaN	NaN	NaN
NaN	NaN	NaN	-1,0808	NaN	-0,250078	-0,949667	NaN	NaN	NaN
NaN	NaN	NaN	NaN	NaN	NaN	NaN	NaN	NaN	NaN
0,0219955	-1,09879	-0,866302	-0,290682	0,810167	-0,400404	0,261141	NaN	NaN	-0,585422
-2,11017	-3,38208	-2,63304	-2,7893	-1,90497	-2,62558	-2,4468	-2,00883	-2,42896	-3,03437
-1,48663	-2,66528	-1,78224	NaN	-0,725666	-2,39404	-1,96979	-1,46397	-1,73577	-2,67331
-1,8979	-1,15443	-2,46939	-1,40172	-1,45656	-1,03196	-1,28351	NaN	NaN	NaN
-0,65675	0,712566	-0,16287	-0,454424	0,568213	NaN	0,177973	0,0119724	-0,590747	-0,101787
NaN	NaN	0,26108	0,713112	-1,78596	1,14018	0,39323	NaN	NaN	NaN
5,97828	4,91385	4,8499	6,43646	5,17286	7,1546	6,30438	5,64627	5,52549	5,07081
-1,44733	NaN	3,90993	5,1436	NaN	-0,394106	4,27957	0,179886	NaN	-0,263583
NaN	NaN	NaN	-2,47209	NaN	-2,01734	-1,68082	NaN	NaN	NaN
1,47823	0,597748	-0,254011	2,08533	2,43074	-0,343006	1,67188	1,64191	1,72657	NaN
-2,46066	-3,29152	-2,69882	NaN	-1,79117	NaN	-1,15295	NaN	NaN	NaN
NaN	NaN	NaN	NaN	NaN	NaN	NaN	NaN	NaN	NaN
0,00645828	-0,354685	-0,308777	-0,312136	-1,15014	NaN	-0,626057	-0,920164	-0,578752	-0,69465
-0,404541	NaN	-1,38232	NaN	-0,534891	NaN	NaN	-0,346479	-0,730657	-0,996047
0,452442	0,266783	0,123558	0,322462	0,954641	0,123943	0,677475	-0,124958	-0,474284	-0,644248
NaN	NaN	NaN	-2,21655	-2,61627	NaN	-1,52202	-0,674988	NaN	NaN

Polito et al. Universal ready-to-use immunotherapeutic approach for the treatment of cancer: expanded and activated polyclonal γδ memory T cells.
Phosphorylation sites

0,291048	-0,203329	-0,202444	0,562056	0,652494	1,16983	0,674677	NaN	-0,914615	-0,874858
NaN	NaN	NaN	-2,40883	NaN	-1,97347	-1,72247	NaN	NaN	NaN
NaN	-2,14896	NaN	-1,87066	-1,85022	-1,66775	-1,5034	NaN	NaN	NaN
NaN	NaN	0,584192	NaN	1,52227	NaN	NaN	0,794876	0,377809	0,91211
-0,641802	-0,564253	-1,08749	0,548923	-0,51639	-0,141138	-0,477808	-1,4295	-2,25569	-1,02597
4,8392	4,56021	6,54545	7,79357	3,70465	8,62804	7,92605	4,93462	6,28264	5,49173
2,09462	2,6799	1,24731	2,90811	2,72156	2,85451	2,16848	2,08908	1,89468	1,63232
2,36996	1,30782	1,25292	4,01937	1,99954	4,02646	4,35555	2,09522	2,76147	2,26292
-2,65742	-2,98181	-1,50534	-1,60663	-1,62174	-1,77706	NaN	-0,871849	-0,976949	-0,631713
0,238001	0,959253	0,943302	0,150675	1,34458	NaN	0,384132	0,386065	0,298686	-0,0845766
2,30038	2,58591	1,92366	NaN	3,2111	-2,87252	NaN	1,37384	1,06526	0,888131
3,62074	3,99048	4,14506	4,20255	4,66236	3,96525	4,5245	3,98099	3,76438	3,69409
NaN	NaN	NaN	NaN	-0,183039	NaN	-1,98643	-1,29398	-1,27596	-1,49341
NaN	NaN	NaN	-2,22083	-1,70031	-1,77169	-3,05084	NaN	NaN	NaN
NaN	NaN	NaN	-0,411444	NaN	-0,880241	0,357868	NaN	NaN	NaN
NaN	NaN	NaN	-2,0002	NaN	-0,47506	-2,51811	NaN	NaN	NaN
0,724501	0,332466	1,3947	0,416775	1,77629	-0,356108	0,226894	1,81103	1,56642	1,94491
NaN	NaN	-2,74624	NaN	NaN	NaN	NaN	-1,88023	-2,31232	-2,04573
NaN	NaN	NaN	NaN	NaN	NaN	NaN	NaN	NaN	NaN
-0,093235	0,590834	0,438068	-0,297218	1,09501	0,427567	0,988829	0,548462	1,01022	0,245805
NaN	-0,770891	-0,801289	-2,41176	1,01045	-2,41402	-0,331778	-0,785177	-0,829604	-0,421819
NaN	NaN	NaN	-0,744915	-1,09274	-0,490852	-1,8627	NaN	NaN	NaN
2,46446	1,20204	1,7632	1,9492	1,53036	2,4927	NaN	1,63382	1,17496	1,18688
3,1648	1,63604	3,26636	1,40099	3,14668	-0,40765	1,69677	3,00458	2,36387	2,59262
1,61895	0,328379	4,36951	4,22592	1,30531	5,02628	4,37021	1,6335	1,55481	1,2982
NaN	-2,66104	-1,71801	-0,559977	-3,24306	NaN	0,477228	-0,976816	NaN	-1,10092
NaN	NaN	0,0352821	-0,853355	-2,57629	-1,96507	-0,0716763	NaN	NaN	NaN
NaN	NaN	NaN	NaN	NaN	NaN	NaN	-1,65686	-1,47822	-1,62979
NaN	NaN	NaN	-1,33042	-3,80575	-1,09052	-0,704548	-2,52576	-1,72123	-2,49639
NaN	NaN	NaN	-1,9561	NaN	-0,661482	-1,7646	NaN	NaN	NaN
-0,655062	0,00056076	0,258053	1,33147	NaN	2,15527	0,610773	-0,956345	-0,296241	-1,13016
0,912327	0,716555	0,534147	1,32055	0,438543	2,27902	1,22138	-0,133797	0,0495901	-0,135169
NaN	NaN	NaN	-0,176842	0,423231	NaN	0,0560837	0,181538	-0,654464	-0,598426
2,67269	3,43846	2,96971	2,38886	3,62293	2,67941	2,84701	3,25818	2,97196	2,88254

-0,744598	1,38053	0,148785	0,754234	0,263929	0,741632	1,35773	-1,00457	1,75589	1,71055
NaN	1,59991	0,293678	-0,71706	2,09574	NaN	1,143	NaN	NaN	NaN
NaN	-0,633326	-3,99591	NaN	0,19285	-1,17925	NaN	NaN	NaN	-0,382247
1,13863	1,25325	1,37276	0,765308	2,02464	0,506029	-0,539028	0,123556	0,0568628	0,140962
1,23376	1,17718	1,09588	NaN	0,229446	1,44285	1,8361	0,304499	0,977366	-3,70307
0,292252	0,223942	-0,26342	1,18555	0,650503	-0,219061	0,721102	-0,307497	-0,439269	-0,056859
0,786116	NaN	1,69191	0,571611	1,20121	NaN	1,4692	NaN	NaN	NaN
NaN	NaN	-0,0105724	1,32393	NaN	0,0689106	1,1577	NaN	NaN	NaN
NaN	NaN	NaN	NaN	NaN	NaN	NaN	-1,23437	0,453357	-0,346269
-0,694107	-1,34733	-0,557447	0,573589	-0,794666	-0,372459	0,289083	-0,971916	-1,31763	-0,622708
-0,422018	-1,25461	-0,273376	NaN	-1,3709	NaN	NaN	-0,791683	NaN	-0,597037
-0,694107	-1,34733	-0,557447	0,573589	-0,794666	-0,372459	0,289083	-0,971916	-1,31763	-0,622708
-0,422018	-1,25461	-0,273376	NaN	-1,3709	NaN	NaN	-0,791683	NaN	-0,597037
NaN	NaN	NaN	-2,05598	NaN	-0,82972	-2,49361	NaN	NaN	NaN
-0,868303	-2,0556	-2,13432	0,362761	-0,0790672	-0,621164	0,447727	-1,43826	-1,29219	-0,721721
-1,29777	-1,21654	-3,70039	-0,688013	NaN	0,658665	0,489967	NaN	-1,00911	-2,08852
-0,471544	0,017004	-1,65624	0,179609	0,62252	NaN	0,801643	-0,843868	-0,791158	-0,8072
-0,657558	-1,87436	-1,20508	-1,97939	-0,954798	NaN	NaN	-1,74065	-1,75828	-1,87642
0,766306	0,627396	0,469378	0,352863	1,34983	0,436184	0,232401	NaN	0,575395	NaN
2,73828	2,46937	2,24735	2,32209	2,46322	3,08547	2,8166	2,01109	1,80262	1,97411
3,02226	3,0199	2,98521	2,32117	3,72029	1,89791	3,02299	3,19762	3,55762	3,33576
NaN	NaN	NaN	-2,13057	-0,854637	-1,80234	-2,44778	-0,928259	-1,53468	-1,14186
1,28485	0,81724	1,08123	1,23598	1,7387	1,75234	1,75559	1,91171	1,30665	1,52477
-0,0526657	0,488621	0,715204	1,07594	1,44752	0,817369	1,58717	-0,675863	-0,322749	0,856542
2,56837	2,0271	2,43539	2,27114	-1,19989	2,6848	2,68197	2,73657	2,66239	2,85664
-1,91337	-1,77414	NaN	NaN	-1,18159	NaN	NaN	-2,00252	-1,81315	-1,42675
NaN	1,69656	NaN	-0,380581	2,38111	1,36097	1,07069	2,61358	2,21787	2,13835
NaN	NaN	NaN	0,443514	-1,93988	-0,222691	0,11746	-1,47877	-1,59709	-1,67378
0,102409	-0,89962	-1,06874	-0,309103	NaN	-1,26115	0,810717	0,552601	0,105674	0,481919
NaN	0,578264	0,961813	1,06632	0,64526	0,476274	-0,974613	0,552601	0,105674	0,481919
-1,52062	1,01774	-1,0119	-0,782555	-1,55271	-1,03348	-0,105728	-2,01164	-0,662103	-1,10679
2,12769	2,21856	1,72582	2,32598	1,99591	2,09377	2,25843	2,36964	2,13167	1,98679
-0,194548	-0,701685	-0,0850163	-0,270807	0,112024	-0,637869	-0,112719	0,141825	-0,320621	-0,475818
-0,530676	-0,527927	0,0521851	-1,18557	-0,907471	NaN	-0,414515	NaN	NaN	NaN

Polito et al. Universal ready-to-use immunotherapeutic approach for the treatment of cancer: expanded and activated polyclonal γδ memory T cells.
Phosphorylation sites

-2,35297	-0,919491	-1,53734	-0,349684	-1,42035	0,706301	-0,175917	-0,86166	-0,841645	-0,283555
NaN	NaN	NaN	NaN	NaN	NaN	NaN	-2,30781	-3,89573	-0,925654
NaN	NaN	NaN	-0,365393	NaN	-2,07848	-2,33727	NaN	NaN	NaN
-2,03635	-1,39468	NaN	-0,192133	NaN	0,799854	-0,943939	-2,46624	-2,16912	-2,54231
NaN	NaN	0,0890007	-0,000900269	-1,6658	0,320705	-0,127462	NaN	NaN	NaN
-0,553328	-1,36753	-2,2721	0,381084	1,19091	-0,419943	0,239271	-2,01891	-1,95067	-0,939672
NaN	NaN	NaN	-1,87302	-3,21088	-1,58964	-2,35912	NaN	NaN	NaN
NaN	NaN	NaN	NaN	NaN	NaN	NaN	NaN	NaN	NaN
-0,75971	0,144428	-1,28609	0,590168	-0,424957	NaN	-0,581602	NaN	-1,12296	-1,14055
NaN	NaN	NaN	-0,813396	0,241762	NaN	-0,714819	NaN	NaN	NaN
0,398024	0,51186	1,50485	1,51729	2,18448	-0,631468	0,988716	1,38477	0,703961	2,61946
4,63634	6,0283	4,24737	5,29352	4,52906	NaN	5,99751	4,32949	4,66299	4,93435
2,39856	NaN	4,15295	4,96467	4,60194	5,43846	4,20689	4,42582	4,37627	3,97965
-1,08874	-1,01848	0,55253	1,13965	0,972689	2,39237	0,949448	-0,435326	-1,03923	0,553311
0,906742	0,96509	0,354792	1,09913	1,0642	1,54552	1,13427	0,692627	0,459292	0,334033
NaN	NaN	NaN	NaN	NaN	NaN	-0,986652	NaN	NaN	NaN
NaN	NaN	NaN	NaN	NaN	NaN	NaN	-0,0589809	NaN	-0,870614
-2,4415	-3,36202	-0,263622	-0,0912628	-3,10483	2,92697	-0,935707	NaN	NaN	NaN
NaN	NaN	NaN	-2,312	-2,74797	NaN	-1,55729	NaN	NaN	NaN
-1,03891	-0,594112	1,14588	-0,0610332	-1,70605	-2,1084	-1,06547	-0,37644	-0,306527	-0,436465
-0,575974	-1,14248	-0,240425	-0,443808	NaN	NaN	-0,534069	-0,204433	-0,206513	-0,690385
NaN	NaN	NaN	NaN	NaN	NaN	NaN	0,59864	0,906816	0,421775
1,35363	2,05608	-1,50005	NaN	0,686247	NaN	NaN	NaN	NaN	0,513982
NaN	NaN	NaN	-2,12952	-2,56769	NaN	-1,14186	NaN	NaN	NaN
NaN	NaN	NaN	-1,57135	NaN	-1,1488	-0,555027	NaN	NaN	NaN
NaN	NaN	NaN	NaN	NaN	NaN	NaN	-3,76131	-3,89679	-2,73182
NaN	NaN	NaN	NaN	NaN	NaN	NaN	NaN	NaN	NaN
NaN	NaN	NaN	-2,08236	NaN	-2,10979	-2,27419	NaN	NaN	NaN
NaN	NaN	NaN	-1,60926	NaN	-1,53573	-1,00654	NaN	NaN	NaN
NaN	NaN	NaN	0,570004	-1,34207	0,146961	-0,602522	NaN	NaN	NaN
-0,569864	-0,297258	NaN	-0,0842113	NaN	-1,65286	-1,125	-2,07634	-3,24583	NaN
NaN	NaN	NaN	NaN	-1,92372	NaN	-0,977516	NaN	NaN	NaN
NaN	NaN	-0,703333	0,123461	NaN	NaN	0,302355	NaN	NaN	NaN
-0,00324059	-0,222231	0,225319	0,204948	0,425667	0,799166	0,192888	0,579573	-0,213655	0,412131

1,10041	NaN	1,31762	1,39534	1,08069	1,73686	1,32323	0,47212	0,411845	0,219811
NaN	NaN	-1,99473	-1,21933	NaN	-0,28306	-1,1705	NaN	NaN	NaN
NaN	NaN	NaN	-1,96626	NaN	-2,28012	-2,25028	NaN	NaN	NaN
-2,06697	-2,4052	NaN	-2,13565	NaN	-1,57994	-1,65223	NaN	-1,98826	-2,13353
NaN	NaN	NaN	NaN	-2,19142	-1,52555	-1,92957	-1,77242	-1,98055	-2,04924
-0,735167	NaN	NaN	-1,24625	-0,451189	-0,152681	-1,3138	NaN	NaN	NaN
1,78366	1,53414	1,77316	1,52395	2,48573	0,23278	2,11542	1,7612	1,27191	1,5055
NaN	NaN	NaN	0,736912	0,223467	2,16911	0,607859	NaN	NaN	NaN
-0,823454	-1,14944	-0,584337	-0,361372	NaN	-1,29292	-0,0777245	NaN	NaN	NaN
NaN	-1,10692	-2,16927	-1,01796	-0,540504	-3,29263	-1,39417	NaN	NaN	NaN
-1,14375	-0,915899	NaN	-0,99155	-1,09098	-0,718489	-0,478462	-1,12937	-1,48992	-1,86283
NaN	NaN	NaN	NaN	NaN	NaN	NaN	NaN	NaN	NaN
NaN	NaN	NaN	-0,145292	0,681185	NaN	-0,407652	0,200047	0,208045	0,346072
NaN	NaN	NaN	-1,81378	NaN	-0,609161	NaN	-1,54383	-1,41889	-1,11179
NaN	NaN	NaN	-0,346407	NaN	NaN	-0,764215	-2,6027	NaN	-1,26766
-0,162643	-0,133619	-1,0808	0,159012	-1,45337	0,825642	-0,797466	-1,09988	-1,44428	-0,93677
1,45505	1,45884	1,72582	NaN	1,81292	NaN	NaN	1,26793	1,06545	1,33013
-0,978775	-1,23681	-0,711363	0,627296	-1,03561	-0,207253	-0,466988	-1,10878	-1,35848	-1,63456
-1,9753	-2,15214	-1,96397	-2,43653	-0,861906	NaN	-3,08689	-2,48218	-2,77826	-2,24334
0,648081	1,27247	-0,251738	1,26648	0,055006	1,30406	-0,728638	1,44748	1,39727	0,989278
NaN	-2,11827	NaN	-1,37468	NaN	-0,193758	NaN	-1,76038	-0,562215	-1,18165
NaN	0,469622	NaN	1,01948	NaN	1,42221	0,466158	-1,50955	-0,680522	-1,85235
NaN	NaN	NaN	NaN	NaN	NaN	NaN	NaN	NaN	NaN
NaN	NaN	NaN	0,496855	NaN	0,851442	0,0598392	NaN	NaN	NaN
1,64694	1,52464	1,18023	2,51024	1,37953	2,2951	3,07337	0,903402	0,284713	0,751273
NaN	NaN	NaN	NaN	NaN	NaN	NaN	NaN	NaN	NaN
NaN	NaN	NaN	-1,04172	NaN	NaN	-1,55486	NaN	NaN	NaN
NaN	NaN	NaN	NaN	NaN	NaN	NaN	NaN	NaN	NaN
-1,7652	-1,12705	-0,496281	NaN	-0,392647	-3,07272	NaN	-0,545536	NaN	0,129596
NaN	-1,42904	NaN	-1,54875	NaN	-0,816301	-1,30318	NaN	NaN	NaN
NaN	NaN	NaN	NaN	NaN	0,205605	NaN	NaN	NaN	NaN
NaN	NaN	NaN	NaN	NaN	NaN	NaN	NaN	NaN	NaN
0,202421	-0,763468	-0,807953	1,10427	NaN	0,338917	0,843592	NaN	-0,541499	NaN
0,255384	0,446373	0,076149	1,72639	0,0170155	2,11473	0,772505	-1,47384	NaN	-0,209537

1,93346	1,64474	2,36381	2,35423	2,54354	3,37429	2,75228	2,05383	1,88488	1,60323
-0,674665	-1,42247	-1,79565	1,77651	-0,396872	2,17459	0,264978	-1,13396	-1,35927	-1,39749
NaN	NaN	-0,155603	0,0320148	NaN	0,546057	-4,17907	-1,05126	-0,908326	NaN
0,26734	0,287992	-0,0546932	-2,49771	1,80725	-2,28724	NaN	-0,19059	0,52469	-0,0138693
0,148346	1,2184	1,09707	2,14913	0,56662	1,40688	2,34824	1,17333	1,01388	0,710061
-1,54067	NaN	-0,832203	-1,6941	-0,597399	-2,6641	-0,32835	-1,48483	-1,10958	-0,768491
NaN	NaN	NaN	NaN	NaN	NaN	NaN	NaN	NaN	NaN
-2,3449	-1,84356	-0,677608	-0,330902	NaN	-0,501078	-0,412224	-1,39079	-1,58044	-1,79979
NaN	NaN	NaN	-1,74125	NaN	NaN	NaN	NaN	NaN	NaN
NaN	NaN	NaN	-1,13783	NaN	-1,3395	-1,69739	NaN	NaN	NaN
NaN	NaN	NaN	NaN	NaN	NaN	NaN	NaN	NaN	NaN
-0,943062	3,51063	-0,290462	NaN	0,408653	4,24666	-3,01653	-0,560724	-2,19527	0,161586
NaN	NaN	NaN	NaN	NaN	NaN	NaN	NaN	NaN	NaN
NaN	NaN	NaN	-2,12848	-3,46273	0,593763	NaN	NaN	NaN	NaN
NaN	-0,847336	NaN	NaN	NaN	NaN	NaN	NaN	NaN	NaN
NaN	0,198238	NaN	1,42015	1,18842	1,50898	-0,205246	1,02581	0,687882	0,744302
1,08303	1,7973	1,7657	1,12633	2,24691	2,37826	1,34151	0,866259	1,4697	1,58744
3,76562	3,03824	2,06804	3,53446	3,36958	NaN	3,46195	2,51514	2,22025	2,14256
-1,06978	-2,13015	-3,0447	-3,78089	0,913601	NaN	-1,07829	-2,17979	-1,7278	-1,1116
-1,18462	NaN	NaN	-0,835827	-0,575073	-0,862419	NaN	NaN	NaN	NaN
-2,37087	NaN	-2,12931	-1,73573	-2,22732	-0,931112	-2,08463	-2,54795	NaN	NaN
-2,46966	-2,56039	-2,97662	-1,09428	-1,92826	-1,35122	-0,807209	-2,35564	-2,77981	NaN
1,28256	NaN	0,752672	1,80672	1,45635	-0,22493	2,16634	1,90494	1,53506	1,15426
0,857208	0,575026	-0,0569611	1,76363	0,95326	2,08688	0,893887	NaN	NaN	NaN
-0,995098	-1,70755	-1,17615	1,11534	-0,760983	0,731419	0,908842	NaN	NaN	NaN
NaN	NaN	NaN	0,196695	-1,04743	-0,593887	-1,59224	NaN	-1,8315	-2,07299
0,0548763	-1,35891	0,2911	1,11398	0,562737	1,64451	1,15349	0,671986	0,056016	0,642558
0,164349	-0,973925	0,539333	-0,494339	0,607521	-1,9832	-0,551912	0,773046	0,530097	-0,0904036
NaN	NaN	-0,44919	-0,916698	-2,41781	0,117771	-0,644558	NaN	NaN	NaN
NaN	2,16656	2,37051	2,11949	2,60732	NaN	2,36182	NaN	1,31632	2,0839
0,413925	1,47417	NaN	1,77608	NaN	0,709234	-0,190788	NaN	0,440721	NaN
0,0343189	0,647127	-0,110008	0,422134	NaN	0,420332	0,779551	0,892561	0,0307207	0,519195
0,374645	0,794697	0,576679	1,33359	1,01932	0,13184	1,31427	1,30181	1,07064	1,03401
3,45962	3,80833	3,88834	3,92752	4,52338	2,57794	3,56506	3,99484	3,37861	5,10315

Polito et al. Universal ready-to-use immunotherapeutic approach for the treatment of cancer: expanded and activated polyclonal γδ memory T cells.
Phosphorylation sites

0,9219	0,591764	0,209274	0,818184	2,117	0,830839	1,68201	0,174194	1,15041	0,639749
2,73124	3,06713	2,6903	2,25255	3,72111	1,56213	2,87537	3,01283	2,74978	2,88971
NaN	NaN	NaN	-0,894932	NaN	-1,23381	-1,65314	NaN	NaN	NaN
0,494242	1,37369	1,27892	2,24737	1,73349	3,35205	1,55147	0,964113	0,511514	1,35403
NaN	NaN	NaN	-2,32217	NaN	-1,41642	-2,0782	NaN	NaN	NaN
NaN	NaN	NaN	0,473745	0,465479	-0,572897	0,730829	NaN	NaN	NaN
-1,61941	-1,11658	-1,15631	-0,214674	-1,91447	-0,373421	0,0903454	-0,82638	0,451919	-1,24415
-0,648085	-1,90524	-1,43878	NaN	-0,289381	NaN	-0,568983	-0,745161	-1,12909	-1,08535
NaN	NaN	NaN	NaN	NaN	NaN	-1,88473	NaN	NaN	NaN
0,562784	0,902798	0,512959	1,08447	0,26362	1,69009	1,43382	0,703281	0,421632	0,717618
-0,509583	-0,192982	-0,147669	-0,572525	-0,0360451	0,0222378	0,770279	0,0129375	0,262982	-0,310111
0,90221	2,68519	0,531319	2,34007	0,0977192	2,50445	2,17245	1,0307	2,42327	1,98997
-0,837107	-0,843418	-0,341276	-0,50873	-0,349405	NaN	-0,396725	-1,32383	-1,1476	-1,02583
NaN	NaN	NaN	-3,09979	-2,46764	-3,55289	-1,4017	NaN	NaN	NaN
0,559305	-0,526289	-0,227463	NaN	0,767262	0,254944	-0,201643	1,10575	1,04751	0,327722
0,196396	1,23053	0,399187	0,183126	0,940266	0,653189	1,58932	0,941046	1,3586	0,952041
1,19001	1,4782	0,971142	2,01071	1,09541	2,23528	2,17418	1,49595	1,56711	1,72929
-0,654219	-0,715048	-0,704782	0,0599155	-0,714798	-1,01632	0,0977573	-1,0119	-0,92027	-0,181783
-0,847832	-0,401388	-0,594543	-0,80946	NaN	NaN	-0,617632	NaN	NaN	NaN
NaN	NaN	NaN	NaN	NaN	NaN	NaN	-3,01519	-3,21114	-3,05234
1,14136	1,70652	1,99468	1,11864	1,26802	1,39786	1,00873	1,73903	1,90665	1,75161
NaN	NaN	NaN	NaN	NaN	NaN	0,0444927	NaN	NaN	NaN
NaN	NaN	NaN	-1,15238	NaN	-0,730051	-0,472403	-0,493868	-0,702449	-1,11447
1,26836	0,948799	1,44904	1,0842	NaN	0,280031	NaN	1,54258	1,49989	1,24294
NaN	0,271179	NaN	0,536049	NaN	NaN	-0,148212	-0,0424728	0,994658	NaN
NaN	NaN	NaN	-1,61889	-4,10809	0,0848179	-2,46248	NaN	NaN	NaN
-0,705637	-0,44043	-0,259354	0,521269	-1,6509	1,66948	-0,507935	-0,0236893	0,239099	-1,89252
-1,54191	-1,45366	-0,820026	NaN	NaN	NaN	NaN	-0,954361	-1,33926	-1,71703
1,41844	2,23993	0,313122	1,71865	1,15535	1,72896	1,88883	1,27103	1,69923	1,39064
-1,43938	0,100597	-0,886969	-0,289402	0,31773	-0,849342	1,06205	-1,23831	-0,943196	0,117694
-0,632257	-1,87733	0,93623	1,68446	0,642694	3,11689	1,99383	0,481441	0,939477	-0,00223827
-3,86641	-0,745962	-2,1346	-3,03099	-0,286396	NaN	-0,493265	-2,13659	-2,26868	-1,19037
0,375214	2,1914	0,864351	1,92249	2,1635	-3,69541	1,52842	-0,383614	0,124948	1,49845
0,866175	1,98887	0,849606	1,7315	2,07037	1,84892	1,93257	NaN	0,621715	1,32776

Polito et al. Universal ready-to-use immunotherapeutic approach for the treatment of cancer: expanded and activated polyclonal γδ memory T cells.
Phosphorylation sites

-1,59136	0,259254	-1,24055	-0,577974	0,507887	-1,68792	0,455685	NaN	NaN	NaN
NaN	NaN	NaN	NaN	NaN	NaN	NaN	NaN	NaN	NaN
NaN	-1,41588	-1,19704	NaN	NaN	-1,25225	-0,37014	-0,389385	-0,518285	-0,235618
NaN	NaN	NaN	0,885303	-1,32673	1,62887	1,77962	-0,115955	0,548442	NaN
3,801	4,15692	3,96648	4,12154	4,10657	4,21726	4,29183	4,44736	4,45702	4,05661
-1,17265	NaN	-1,15475	NaN	-0,418711	-1,75708	NaN	NaN	NaN	-0,756423
NaN	NaN	NaN	-1,21523	NaN	NaN	0,888102	-0,531948	0,393397	-0,037652
NaN	NaN	NaN	NaN	-2,69311	NaN	-1,3582	-0,732674	-1,00211	-1,28753
NaN	NaN	-1,48942	NaN	-1,57932	-0,912338	-1,83461	-0,864746	NaN	NaN
3,13797	3,51323	3,52164	3,73305	3,38708	3,83427	4,37237	3,34705	3,38186	3,43632
0,218586	0,342085	1,2474	1,035	0,912394	1,05121	1,29891	0,901535	0,543668	0,416884
NaN	NaN	NaN	-2,24633	NaN	-2,10783	-1,02329	-2,36396	-2,88396	-2,5149
0,116911	0,280188	0,910187	0,424603	-0,195292	0,842514	0,758438	0,468575	-0,0530348	NaN
NaN	4,14895	NaN	NaN	2,9952	-3,96284	3,66718	NaN	NaN	NaN
1,22719	3,33505	1,30171	1,752	0,726583	1,46451	2,3267	2,3721	1,67214	2,03311
-0,534616	1,006	1,0289	0,274637	1,81809	-0,248796	1,16659	1,14485	0,904954	1,25875
0,587564	2,48002	1,51379	1,2602	0,706984	-1,55652	1,96201	1,14564	1,90561	1,76599
0,673494	2,5471	1,57647	1,09268	0,988983	1,42029	1,90944	1,54797	1,67465	1,34439
NaN	-0,162323	-0,574108	-1,05075	NaN	-0,556168	0,0853405	-0,339424	NaN	-0,801509
NaN	NaN	NaN	NaN	NaN	NaN	NaN	NaN	NaN	NaN
NaN	-2,132	-1,53968	-2,38137	NaN	-1,74703	-1,04545	-0,932346	-1,14406	-1,0377
-0,396469	-0,720274	-0,234552	1,55569	0,210159	2,19873	1,53899	0,163546	0,24624	0,0553885
-0,530128	-1,50063	NaN	NaN	NaN	1,12554	-0,404749	-0,442188	0,0598974	NaN
NaN	NaN	NaN	NaN	NaN	NaN	NaN	NaN	NaN	NaN
2,7596	4,45589	3,81466	4,0081	4,30503	2,64985	4,58012	3,41457	4,059	3,76636
5,99605	5,70042	5,60321	6,61735	5,78324	6,94349	6,63498	6,00151	5,69877	6,25795
NaN	NaN	NaN	-0,487675	-2,76353	0,155056	-0,762472	NaN	NaN	NaN
NaN	-1,43286	0,0313663	-0,539366	0,772896	-2,92134	-0,508951	0,625	0,124816	0,38075
NaN	NaN	NaN	-2,00684	-0,428473	NaN	-2,51376	-1,15297	-1,32892	-0,890046
1,18354	1,174	1,4041	0,942472	1,47017	1,55648	1,72185	1,35952	1,24257	0,69823
-1,92375	NaN	-1,63904	NaN	-1,39067	-2,49622	NaN	NaN	-2,17078	-2,55398
NaN	-0,614002	NaN	-0,294962	-0,161383	-0,488834	-0,625988	NaN	NaN	NaN
NaN	-2,23201	NaN	NaN	NaN	NaN	-1,3741	NaN	NaN	NaN
NaN	-2,05748	-0,207035	-0,872141	-1,32894	-0,546509	-1,82238	-0,924183	-0,508857	-0,951739

Polito et al. Universal ready-to-use immunotherapeutic approach for the treatment of cancer: expanded and activated polyclonal γδ memory T cells.
Phosphorylation sites

NaN	-3,1032	-1,21282	0,0981998	NaN	0,299343	0,805866	NaN	NaN	-1,26894
NaN	NaN	NaN	-1,67237	-2,42773	-0,860003	0,0275364	-1,80526	-2,37775	-2,05925
NaN	NaN	NaN	-2,31364	NaN	-2,40365	-3,25905	NaN	NaN	NaN
1,25776	1,25352	NaN	1,11279	0,911064	NaN	1,53468	0,989712	1,29767	NaN
-0,289726	-0,657202	0,0428524	1,09431	0,0587788	1,00914	0,627605	-0,43948	-0,664927	-0,525098
NaN	NaN	NaN	-2,93184	NaN	-1,12189	-2,05108	NaN	NaN	NaN
NaN	-1,45597	-0,500982	0,866976	-2,54352	NaN	0,375217	-0,837614	-0,584952	-1,41458
NaN	NaN	NaN	-0,975159	NaN	-0,34474	NaN	NaN	NaN	NaN
NaN	NaN	NaN	-1,31419	NaN	-1,28023	-1,18582	NaN	NaN	NaN
-0,00413513	NaN	NaN	-0,667606	-1,5971	NaN	-0,754494	-0,922291	NaN	NaN
0,830093	-0,0852699	-0,332014	NaN	0,871159	NaN	NaN	1,27516	0,879207	0,997348
3,42051	-0,649216	2,95177	3,23227	3,91815	0,217609	3,96447	3,11009	3,01474	2,90106
-0,134684	NaN	NaN	0,16432	0,361698	NaN	0,43125	NaN	NaN	NaN
-0,771816	-1,20543	-0,19352	1,35775	-1,13701	1,66925	1,49781	NaN	NaN	NaN
NaN	NaN	NaN	NaN	NaN	NaN	NaN	NaN	NaN	NaN
NaN	NaN	-1,30875	-0,825497	NaN	0,605862	0,160805	NaN	NaN	NaN
NaN	NaN	NaN	NaN	NaN	-0,897081	-0,757475	NaN	NaN	NaN
-0,2635	0,160267	-0,157848	-0,610218	0,236172	-0,307011	-0,51523	0,33349	0,147805	0,342673
0,00510216	0,826845	0,420645	-0,14028	NaN	NaN	-0,116566	NaN	1,25164	NaN
NaN	NaN	NaN	-2,66437	-2,59431	-0,340607	-2,09662	NaN	NaN	-1,71274
-1,23385	-2,29582	-1,81111	-1,09154	-0,96126	-1,30993	-0,647146	NaN	NaN	NaN
NaN	NaN	NaN	NaN	NaN	NaN	NaN	NaN	NaN	NaN
-2,9551	-2,98674	-2,92208	-1,77319	-1,66463	NaN	-1,74491	-2,23508	-3,06532	-2,33149
-0,93681	-1,41331	-1,40056	-1,04092	-0,710512	-2,14572	-0,929396	-1,10433	-1,22766	-1,65111
NaN	NaN	NaN	NaN	-1,97824	-2,04017	NaN	-2,33247	-3,02819	-2,81326
0,992929	0,59816	0,516104	1,10802	0,878431	2,00881	0,514328	0,942343	0,899138	0,639718
NaN	NaN	NaN	-0,0686855	-0,336639	NaN	-0,289812	-0,52479	-0,722308	-0,808806
NaN	NaN	NaN	0,83312	-1,26502	0,329506	NaN	NaN	NaN	NaN
NaN	-1,46424	1,58906	1,6621	NaN	1,01069	1,71581	NaN	NaN	NaN
NaN	NaN	NaN	-1,1205	NaN	-0,509838	-0,961021	NaN	NaN	NaN
-0,899441	NaN	0,908381	NaN	-0,651932	NaN	-0,95174	-0,712547	-0,714816	-0,731441
0,778198	-0,473553	3,14209	1,26151	2,27338	0,728384	2,46353	2,01425	1,93723	1,67592
NaN	-2,16117	NaN	-1,58281	-0,908857	-1,93136	-1,33096	-1,14904	-0,67439	-0,0396795
0,642658	0,396948	0,758947	0,683887	0,395594	1,4436	0,589188	NaN	NaN	NaN

-2,4892	NaN	-1,64267	-2,46689	-2,27799	NaN	-2,74722	-2,39853	-1,20398	-2,8417
NaN	-1,38053	-1,7548	-1,58905	-0,945076	-0,0218544	-0,963852	NaN	NaN	NaN
NaN	NaN	NaN	NaN	NaN	NaN	NaN	NaN	NaN	NaN
NaN	NaN	NaN	NaN	NaN	NaN	NaN	NaN	NaN	NaN
NaN	NaN	NaN	NaN	NaN	-3,60085	0,406189	-0,244236	NaN	0,0060358
NaN	-2,17454	-0,124584	NaN	NaN	NaN	NaN	0,152233	0,15141	0,259423
NaN	NaN	NaN	-1,77615	NaN	-1,92053	-2,44656	NaN	NaN	NaN
NaN	NaN	NaN	-0,828039	NaN	NaN	-0,927397	-2,96628	-0,317449	-3,46167
NaN	NaN	NaN	0,480743	NaN	-1,11544	0,233961	-0,42494	NaN	NaN
1,38815	NaN	NaN	3,07564	0,877348	NaN	2,84356	2,28563	-2,34738	2,48318
0,440502	-0,680357	1,02265	1,79898	-1,52938	3,23627	1,92976	-0,430677	1,82687	0,460765
1,20044	NaN	NaN	NaN	1,51801	-1,16604	NaN	0,987324	NaN	0,243171
1,20548	0,623659	-0,185495	1,48147	0,56085	2,32023	0,892323	0,508221	0,112823	0,266614
-0,733051	-0,474525	-0,658579	-0,939997	-1,24164	-0,587265	-0,293833	-1,32146	0,162686	-1,15939
NaN	NaN	NaN	-0,878853	NaN	0,174513	0,025013	NaN	NaN	NaN
-1,42229	-2,24133	NaN	-0,128717	-0,878693	-0,266705	-1,67125	-0,663643	-1,0099	-1,33491
-5,53441	1,02549	2,67617	3,35236	1,0144	3,52066	2,93482	NaN	NaN	NaN
-0,391659	1,58954	1,37244	3,42143	2,55443	2,00362	3,67557	0,485561	0,370103	2,44298
NaN	NaN	NaN	NaN	NaN	NaN	NaN	-0,608297	-0,435964	-0,743493
3,40084	2,43328	3,80912	5,78338	3,58763	5,56952	5,04979	4,18449	4,01337	4,07756
5,41885	5,66521	5,63938	5,59036	6,47233	5,61915	5,63237	6,14675	5,59706	5,74678
3,33058	5,21608	4,13834	4,44311	4,93845	2,38919	5,16234	4,22626	3,90419	4,44555
NaN	NaN	-0,793884	-1,30958	NaN	-1,54158	-0,811909	-1,94732	-1,39534	-1,66611
-1,46374	-2,70161	-0,682159	NaN	-1,31409	NaN	-2,65596	-0,33769	-0,387191	-0,757276
1,48263	1,32677	1,79023	1,50957	2,00735	1,54917	1,49479	2,05191	2,12304	1,57355
-0,894953	0,279161	0,0501804	-0,954102	NaN	0,460337	-1,18653	-1,62474	-0,239474	NaN
1,24886	0,413065	1,16812	-0,257013	1,53538	NaN	0,840475	1,25868	1,63999	0,743754
1,26947	1,02097	1,32998	1,15946	1,40241	-2,02083	1,8172	1,72793	1,45548	1,26955
-0,679949	-1,10246	-0,403307	-0,842669	-0,496159	NaN	NaN	-0,376635	-0,515615	-1,40848
-1,63888	-1,30392	-0,120731	-1,46588	0,0595417	-3,66111	-0,339703	-0,763166	-0,317193	-0,265733
4,83865	5,80041	5,16605	5,32215	4,98067	5,46529	5,72363	5,59084	4,87681	5,58981
3,91873	5,9441	6,17339	5,67396	5,8102	NaN	5,99384	5,52612	5,52887	5,47216
NaN	NaN	NaN	NaN	NaN	NaN	NaN	NaN	NaN	NaN
-1,04839	-1,63091	-0,0643234	-0,756731	-1,34582	0,750849	0,117336	-0,9942	-3,29167	-1,22107

Polito et al. Universal ready-to-use immunotherapeutic approach for the treatment of cancer: expanded and activated polyclonal γδ memory T cells.
Phosphorylation sites

0,407597	1,81861	0,820974	1,66245	1,62604	1,04524	1,62975	0,181625	NaN	NaN
0,544735	2,44794	0,186581	0,848412	0,0854721	-0,565821	1,23027	1,91204	1,19311	-5,1063
4,25568	NaN	2,27275	2,12738	-1,42981	3,59384	-0,601809	2,79653	4,20892	-1,52913
-0,909552	-2,47148	-1,2143	-1,09801	-2,83724	-0,845869	-1,26471	-1,12583	-1,11997	-1,30081
-0,498568	-1,25688	-1,28077	-0,740702	-1,46376	-0,371792	-1,0262	-1,29074	-0,118766	-0,718696
5,05619	4,97625	4,98929	3,31404	5,79584	0,484606	4,22803	5,40006	5,10906	4,87294
NaN	NaN	NaN	-0,935665	-2,66503	NaN	-0,77829	NaN	NaN	NaN
-0,688557	NaN	-1,38703	-1,18821	0,640179	-1,61097	-1,03795	NaN	-1,30769	0,192239
NaN	NaN	NaN	0,29637	NaN	0,372257	-0,103254	NaN	NaN	NaN
1,67195	-0,121433	3,10372	4,0213	0,57655	1,98927	2,83788	-0,0632744	0,622113	-0,455819
NaN	0,131687	0,195402	1,01285	1,44062	1,01354	-0,0160885	0,298365	0,000287056	-0,242633
-1,61866	-1,20547	-0,946507	-0,138903	-0,704939	NaN	-0,86306	-0,541283	-0,616143	-1,05048
NaN	NaN	NaN	-0,400242	-1,7606	-0,268841	-0,318619	-0,943672	-0,800771	NaN
1,75087	1,51573	2,585	2,96744	0,967043	2,57508	2,8396	1,92103	2,05231	2,37413
1,39096	1,11546	1,55643	2,08922	2,11916	1,27306	1,8587	2,63124	2,25809	1,81162
NaN	NaN	NaN	-1,45243	NaN	-0,0664577	-1,1146	NaN	NaN	NaN
NaN	NaN	NaN	NaN	NaN	NaN	NaN	NaN	NaN	NaN
NaN	NaN	NaN	NaN	NaN	NaN	NaN	NaN	NaN	NaN
NaN	-1,31833	-1,21656	-1,96411	-1,68707	NaN	NaN	-1,12257	-1,47132	-1,62003
NaN	NaN	NaN	-3,10542	NaN	-1,70033	-2,69481	NaN	NaN	NaN
NaN	NaN	NaN	NaN	NaN	NaN	-1,62737	NaN	NaN	NaN
1,46203	NaN	0,338924	0,981352	NaN	0,792471	0,405279	NaN	NaN	NaN
NaN	NaN	NaN	NaN	NaN	NaN	NaN	NaN	NaN	NaN
0,236336	2,51202	2,12732	2,87153	-0,000654221	1,74535	2,65388	1,9645	3,08318	2,52421
0,877031	1,34583	0,786613	0,968445	2,05438	0,830637	1,90479	1,38582	1,68265	1,72881
NaN	NaN	NaN	1,08004	NaN	-0,61879	0,514093	NaN	NaN	NaN
0,237206	0,242233	0,838625	1,38376	0,849846	1,5597	0,454659	0,565248	0,606439	0,422118
1,41082	0,536556	1,24302	1,93735	2,33023	1,78637	1,07526	0,568985	0,427987	0,19057
0,417992	0,362585	1,02255	0,26045	2,45852	-1,71021	-0,0145302	-0,265905	-0,0752687	0,36148
NaN	NaN	NaN	NaN	NaN	NaN	NaN	-1,60738	-1,00252	-1,81642
2,65565	3,47268	2,21261	3,22407	3,56758	3,25509	2,60035	1,87138	1,42258	1,80355
NaN	NaN	NaN	NaN	NaN	NaN	NaN	-2,24616	-2,39459	-2,43653
-0,752247	NaN	-2,53568	NaN	NaN	NaN	NaN	0,242704	0,510219	-0,150371
NaN	-1,51253	-2,55835	-1,87855	-1,98994	-2,58496	NaN	-1,88169	-2,01205	-2,00251

Polito et al. Universal ready-to-use immunotherapeutic approach for the treatment of cancer: expanded and activated polyclonal γδ memory T cells.
Phosphorylation sites

NaN	NaN	NaN	0,222418	0,358019	0,917707	0,62616	NaN	NaN	NaN
2,16862	0,755371	NaN	NaN	1,31174	NaN	NaN	1,85954	1,30009	1,70162
0,237225	-1,68634	-2,1874	-0,834942	-0,150391	NaN	-0,284794	0,204535	-0,123181	-0,298041
0,385759	-0,124794	-0,682533	0,630625	-0,248308	-0,44084	0,571733	-0,186327	-0,851121	0,637361
-0,238241	0,0356655	-1,80118	-0,497009	-0,462076	-0,507963	0,463598	-1,14153	-1,82662	-0,831864
NaN	1,10622	NaN	0,0886364	-1,49035	NaN	0,53377	NaN	-1,07715	NaN
NaN	NaN	NaN	NaN	NaN	NaN	NaN	NaN	NaN	NaN
NaN	NaN	NaN	NaN	NaN	NaN	NaN	NaN	NaN	NaN
NaN	NaN	NaN	-1,17379	-3,03412	-1,53473	-1,21567	NaN	NaN	NaN
NaN	NaN	NaN	0,62789	NaN	0,255947	1,92923	NaN	0,321021	-0,260747
1,20838	0,156218	1,03997	2,04795	0,968191	2,08891	1,98302	1,3795	1,04234	2,03188
1,73721	2,9145	1,43155	2,72361	2,48847	2,98015	2,82226	1,51011	1,25268	2,17199
3,44926	3,21049	3,11028	5,37489	3,18533	5,67475	3,94334	2,38434	2,1229	2,44905
-0,688629	-0,817545	-0,228283	0,446754	0,746058	-1,64216	1,67636	NaN	NaN	NaN
NaN	NaN	NaN	NaN	NaN	NaN	NaN	NaN	NaN	NaN
NaN	NaN	NaN	NaN	NaN	NaN	NaN	NaN	NaN	NaN
-0,652849	NaN	-2,31101	0,281414	-1,4378	0,57176	-1,04098	NaN	-1,37089	NaN
NaN	0,794518	NaN	0,871202	0,0251694	1,32722	1,60255	NaN	NaN	NaN
2,4855	2,84699	1,92511	2,21745	2,54807	0,750776	1,17552	1,97994	1,75535	1,24671
NaN	-0,305397	NaN	NaN	NaN	-0,148426	NaN	NaN	NaN	NaN
NaN	NaN	NaN	-1,49124	NaN	-1,99667	NaN	NaN	NaN	NaN
-0,617422	-0,723719	0,498131	0,81024	-0,0789471	-0,0252647	-0,441271	1,42629	1,10965	1,16597
NaN	NaN	-1,48058	NaN	-1,34272	NaN	NaN	-1,9704	-2,1506	-1,35396
NaN	NaN	NaN	NaN	NaN	NaN	NaN	-1,31399	-1,39151	-1,33856
-0,920748	-1,52606	-0,369286	-0,608843	-0,607847	1,15301	0,0593033	-1,20188	-1,08374	0,447986
-2,1444	-1,1529	-1,49198	-1,01822	NaN	-0,224651	-1,91037	-2,30583	NaN	-2,53046
NaN	NaN	NaN	-0,805599	-1,3064	-0,48147	-0,688253	NaN	NaN	NaN
-1,74351	-0,749048	-1,48123	-0,961277	NaN	-0,825191	0,10078	-2,56519	-0,96173	-1,70838
NaN	NaN	NaN	-2,47801	-2,81318	-1,72952	-1,33327	-2,79278	NaN	NaN
-3,39944	NaN	-1,02892	-0,220316	-1,9413	-0,307114	-0,545212	NaN	NaN	NaN
NaN	NaN	NaN	-3,07818	-1,32545	NaN	-2,78745	NaN	NaN	NaN
NaN	-4,18021	-2,66589	-3,65447	-2,14844	-2,4372	-4,11571	NaN	NaN	NaN
0,519903	-0,322264	0,395823	0,543762	1,38062	0,521805	0,819607	0,588707	0,73965	0,876927
NaN	NaN	NaN	NaN	NaN	NaN	NaN	NaN	NaN	NaN

Polito et al. Universal ready-to-use immunotherapeutic approach for the treatment of cancer: expanded and activated polyclonal γδ memory T cells.
Phosphorylation sites

NaN	NaN	NaN	NaN	NaN	NaN	NaN	NaN	NaN	NaN
0,219717	-0,744995	-0,301184	-0,532305	0,150879	-1,21194	0,70433	0,439184	0,451123	0,366616
NaN	NaN	NaN	NaN	NaN	NaN	NaN	NaN	NaN	NaN
NaN	-0,239416	NaN	NaN	-0,378273	NaN	-1,7361	-1,54288	-2,60931	-0,637485
NaN	NaN	NaN	-1,31428	-1,43934	NaN	-1,7312	NaN	NaN	NaN
-1,00228	-1,52638	-0,00959015	0,381649	-0,273314	0,184774	0,00163078	-1,3605	-0,84562	-1,749
NaN	NaN	-1,91639	-2,81767	-0,598217	NaN	-0,952736	NaN	NaN	NaN
NaN	NaN	NaN	-0,904636	-1,52087	NaN	0,21818	NaN	NaN	NaN
NaN	NaN	NaN	NaN	-3,5973	0,165993	NaN	-2,12872	-2,43582	-2,6106
-1,55097	-0,101776	-0,519854	-2,44011	0,559557	NaN	-0,302818	-0,852308	-1,2109	0,219871
-0,490204	-0,54476	NaN	-0,183422	-1,39597	NaN	0,115667	-0,208233	-1,35213	-1,51608
NaN	NaN	NaN	0,157597	0,631487	-0,822392	0,07617	NaN	NaN	NaN
-0,438349	-0,60438	-0,250652	-1,53111	0,301373	NaN	-1,0013	-0,207281	-0,350303	-2,50457
0,27494	-0,87059	-0,64798	-0,825464	0,247688	NaN	-1,75287	-0,393934	NaN	NaN
NaN	NaN	NaN	NaN	NaN	NaN	NaN	NaN	NaN	NaN
NaN	NaN	NaN	NaN	NaN	NaN	NaN	NaN	NaN	NaN
NaN	NaN	NaN	NaN	NaN	NaN	-1,40005	NaN	NaN	NaN
NaN	NaN	NaN	-2,60904	-1,56755	NaN	-1,24069	NaN	NaN	NaN
0,814821	1,01782	1,09796	2,21039	1,34939	2,13028	1,1116	1,11626	0,924968	0,896415
NaN	NaN	-1,7164	-1,96734	NaN	1,7571	-1,78094	-2,82259	NaN	NaN
-2,84874	-2,34579	-0,746279	-0,449295	-2,20657	-1,12461	-0,931269	-1,43516	-1,21799	-0,984876
-2,9654	-2,17708	-2,44045	NaN	NaN	NaN	NaN	NaN	-2,07141	-1,00721
0,245193	0,33642	0,472712	0,513306	0,830231	NaN	0,441637	0,331766	0,447625	0,148829
1,92178	NaN	0,631207	2,78694	1,60648	3,44697	0,145288	1,38772	1,44098	2,37789
0,931879	1,39677	1,0146	1,02347	0,720108	0,991407	0,7449	0,946102	0,752732	1,11793
1,67713	1,7403	1,87502	1,06685	2,30928	0,75412	1,02046	1,99147	1,95374	1,90257
-0,0610237	0,684732	0,440695	0,331766	NaN	-0,751074	0,532009	0,898861	0,743697	0,860845
-0,739334	-1,21058	NaN	-0,468115	NaN	-0,874979	NaN	NaN	NaN	NaN
0,18236	NaN	-0,420565	0,136095	1,42217	-0,505205	-0,404217	-0,807007	NaN	0,471498
NaN	NaN	NaN	-1,87936	NaN	-1,59923	0,0614471	NaN	NaN	NaN
0,68853	-0,680508	-1,00525	NaN	1,07639	NaN	-0,828402	1,52657	0,944495	1,84317
NaN	NaN	NaN	-0,664076	NaN	0,618874	-0,477621	NaN	NaN	NaN
NaN	NaN	NaN	-2,23729	NaN	-2,09676	NaN	NaN	NaN	NaN
1,90835	1,85778	1,12746	1,47877	0,982378	NaN	1,36365	1,55061	1,32903	1,81775

Polito et al. Universal ready-to-use immunotherapeutic approach for the treatment of cancer: expanded and activated polyclonal γδ memory T cells.
Phosphorylation sites

-2,13947	-1,53317	NaN	NaN	NaN	NaN	NaN	NaN	NaN	NaN
1,90835	1,85778	1,12746	1,47877	0,982378	NaN	1,36365	1,55061	1,32903	1,81775
-2,42426	-1,19862	-1,41971	0,533258	-1,20966	-1,10051	0,204592	0,0855827	-0,214202	-0,492026
-2,88278	NaN	-1,62544	-1,04217	-1,56125	-1,69741	-0,811359	-1,1205	-1,66897	-1,1505
NaN	NaN	NaN	-1,22723	NaN	-0,908216	-1,603	-0,790001	NaN	-1,93488
NaN	NaN	0,615936	1,51838	-2,83771	0,0783634	1,08561	-0,0770226	-1,08453	-0,743033
NaN	NaN	NaN	NaN	NaN	NaN	NaN	NaN	NaN	NaN
-0,559565	-0,575422	-0,93771	0,625271	NaN	0,853765	0,0600967	-1,90589	-1,66968	-2,50002
-2,20547	-2,53552	-2,84472	-0,811911	NaN	NaN	-1,03177	NaN	NaN	NaN
NaN	NaN	NaN	NaN	NaN	-0,856159	-2,61911	NaN	NaN	NaN
NaN	NaN	NaN	-0,998732	NaN	-1,41575	-1,89866	NaN	NaN	NaN
NaN	NaN	NaN	-2,39407	-2,56595	-2,09554	-2,64431	NaN	NaN	NaN
-0,423843	1,11948	-0,833033	-0,314899	NaN	0,0592823	0,313223	NaN	-0,19276	NaN
0,810629	-1,02916	0,686384	-0,420052	1,12034	NaN	-0,245558	NaN	0,0896502	NaN
-1,38952	-1,49241	NaN	-0,550766	-0,0380573	-2,14101	-0,486015	NaN	NaN	-1,22917
-3,02418	-0,955379	-2,22347	-2,5714	NaN	NaN	-2,02876	-2,06962	-1,51327	-2,47595
-0,11647	NaN	0,772537	-1,30922	1,43484	NaN	0,12537	0,192165	-0,04531	0,119596
NaN	NaN	NaN	NaN	-0,63859	-2,32025	0,203796	-1,40565	-1,1995	-0,406863
NaN	NaN	NaN	0,453348	-1,28678	0,846519	0,377972	NaN	NaN	NaN
-0,469997	-0,632214	0,351107	2,71457	-0,377054	2,54995	1,56785	0,392939	0,440629	0,289947
-0,492964	-0,295019	-0,669058	1,36426	0,68577	1,61684	1,17146	-0,156525	0,014245	-0,0906725
3,54234	2,91737	3,95245	3,38704	4,63103	3,41002	2,63616	3,54615	3,86827	3,34604
NaN	NaN	NaN	1,05813	NaN	2,2765	1,95994	NaN	NaN	NaN
NaN	NaN	NaN	NaN	NaN	NaN	NaN	NaN	NaN	NaN
-0,189371	-1,41095	0,151859	0,120014	-0,00998688	-0,200352	0,0706692	-0,288912	-0,272412	-0,255315
NaN	NaN	0,917612	0,639502	NaN	0,71764	0,804972	-0,035017	-0,557528	-0,184806
NaN	2,05825	NaN	2,2852	1,30692	2,17334	0,425188	1,14823	0,713248	0,350308
3,34265	2,82842	1,47703	1,66703	2,61523	NaN	-1,23038	1,55791	1,36044	1,25856
NaN	NaN	NaN	NaN	-1,71433	0,617157	-0,615679	NaN	NaN	NaN
0,205347	-0,314203	-0,53194	1,47176	-0,156841	-1,93879	0,336151	0,952045	-0,0561018	0,0231638
-1,77785	-0,530647	-0,849476	-2,16973	-1,36159	NaN	-2,26772	-1,42782	-0,997684	-1,63242
NaN	NaN	NaN	-1,28954	NaN	-1,7681	-1,32787	NaN	NaN	NaN
NaN	NaN	NaN	-0,975231	-0,754684	-0,577862	-0,467388	NaN	NaN	NaN
NaN	NaN	-1,71195	-2,21305	NaN	-3,31965	0,122416	NaN	NaN	NaN

Polito et al. Universal ready-to-use immunotherapeutic approach for the treatment of cancer: expanded and activated polyclonal γδ memory T cells.
Phosphorylation sites

1,56505	2,45915	1,22626	2,63027	1,9458	1,72088	2,03231	1,67783	1,09686	1,28026
0,0509186	-0,362095	-0,114866	1,12346	0,389072	0,9674	-0,174681	-0,0089817	-0,328223	-0,185197
-2,04323	-2,82092	-1,52796	-0,112152	-1,72689	-3,27269	-0,812065	-1,39275	-1,75046	NaN
1,2182	NaN	-0,0215893	3,47025	1,31871	4,19257	2,19205	0,075037	0,170051	1,26392
NaN	NaN	NaN	-1,34641	NaN	-2,23926	-1,56872	-1,92432	-2,90324	-1,97772
NaN	NaN	NaN	0,580244	NaN	1,21689	-0,209709	NaN	NaN	NaN
NaN	0,341839	NaN	1,35279	0,422594	1,9016	-0,7908	NaN	NaN	NaN
-1,16082	NaN	-1,25267	NaN	NaN	NaN	NaN	-0,176287	-0,408864	-0,334327
-3,07562	NaN	0,954721	1,85269	0,0734711	2,19148	1,73144	0,430567	-0,172555	0,320903
NaN	NaN	NaN	-1,97079	NaN	-0,910851	NaN	NaN	NaN	NaN
1,48343	1,04548	0,958652	1,44331	0,625822	0,0740433	0,948753	1,6144	1,65758	0,677287
NaN	NaN	NaN	NaN	NaN	NaN	NaN	NaN	NaN	-2,03892
-1,13913	-0,671547	-1,19514	NaN	-0,864544	NaN	NaN	NaN	NaN	-1,39333
-0,464258	-0,923355	-1,85506	NaN	NaN	NaN	NaN	NaN	NaN	NaN
2,38913	1,4062	1,81092	NaN	2,15892	NaN	-0,168289	2,207	2,00777	1,65239
1,81748	1,41826	1,54202	NaN	NaN	NaN	NaN	1,96347	1,40867	NaN
NaN	NaN	NaN	-2,65786	NaN	-1,30105	-2,78924	NaN	NaN	NaN
-0,482979	-0,592443	0,117865	0,452812	0,673361	1,1108	0,673557	-0,170885	-0,155208	0,148187
-3,43492	-0,0446949	-1,75052	NaN	1,11129	NaN	-0,616123	-3,14643	NaN	-1,25173
NaN	-0,942478	0,195215	3,14563	0,71298	2,92179	2,14583	-0,490358	0,215058	-0,116065
NaN	NaN	NaN	NaN	-1,44429	-2,01225	-0,950705	-1,48805	-1,44122	-1,36935
-1,6927	-1,54054	-1,67846	-1,62596	-0,7605	-2,76117	NaN	-1,40096	-1,52078	-2,17916
1,17941	1,10456	0,54755	0,231213	1,52356	-0,380003	0,691103	0,820242	0,30622	0,494826
-0,12796	0,201359	0,108772	-0,221087	0,714748	-3,02229	-0,110594	0,42853	0,297252	0,236836
2,42099	3,02463	NaN	2,44868	2,96435	3,06413	2,13377	2,664	2,86128	2,30949
0,657026	0,169437	0,544823	0,357893	0,86474	1,20545	1,1431	0,24416	0,385194	0,445226
NaN	NaN	-1,06807	NaN	0,265846	-0,484032	-1,24805	-1,14165	-1,08993	-0,00486088
0,379869	0,52166	0,444765	0,418427	0,393236	0,407106	0,790445	0,549059	0,433175	0,382247
1,21041	1,53827	1,56304	0,568392	NaN	NaN	0,365068	NaN	NaN	1,24942
-0,532936	0,257431	-0,474442	-1,32599	0,164921	0,328457	-1,00004	-1,00342	-1,30698	-0,386352
NaN	NaN	NaN	NaN	NaN	NaN	NaN	NaN	NaN	NaN
NaN	NaN	NaN	NaN	NaN	NaN	NaN	NaN	NaN	NaN
NaN	NaN	NaN	NaN	NaN	NaN	NaN	NaN	NaN	NaN
NaN	NaN	NaN	NaN	NaN	NaN	NaN	NaN	NaN	NaN

Polito et al. Universal ready-to-use immunotherapeutic approach for the treatment of cancer: expanded and activated polyclonal γδ memory T cells.
Phosphorylation sites

NaN	NaN	NaN	-0,6623	-2,54765	-2,6353	-0,806271	-1,91602	-1,89721	-2,11582
0,299904	NaN	-1,04336	-1,71863	1,46482	NaN	NaN	1,54327	NaN	0,922681
3,88058	4,42634	3,15228	3,99939	3,60704	4,42108	4,21714	3,4993	3,37869	3,69254
-1,17673	-1,0629	-2,42796	-2,12615	-0,031126	NaN	-1,99087	-1,1587	-1,4468	-0,70861
0,960377	1,22694	0,878601	2,68051	1,76462	0,920622	2,47145	0,704966	0,42336	1,32648
0,351881	0,199867	1,15685	1,10913	0,261749	0,041811	2,04682	1,65227	1,76672	1,44501
0,953247	1,65516	1,21845	2,53637	0,535631	2,33877	1,79238	2,08996	2,94178	1,72242
-0,988972	1,24657	0,559811	0,653242	1,76704	-0,507162	1,46248	0,460423	-0,186677	0,939052
-1,69588	NaN	-1,35696	1,29628	-1,73595	-1,38356	-0,244947	-1,87601	-1,79553	-0,761626
NaN	NaN	NaN	-1,20312	NaN	-3,05419	NaN	NaN	NaN	NaN
NaN	NaN	NaN	1,29822	0,620876	-1,49578	-1,90091	-0,267376	-0,470727	-0,474956
-4,1379	-3,56425	-0,909765	NaN	-1,35525	NaN	-2,0283	-0,939457	-1,00203	-1,08999
NaN	NaN	NaN	-2,13856	NaN	-0,270256	-2,58103	NaN	NaN	NaN
NaN	NaN	-0,061821	0,594679	NaN	1,33645	1,76825	NaN	NaN	NaN
NaN	NaN	NaN	NaN	-1,81478	NaN	-1,30776	NaN	NaN	NaN
-0,777775	2,72067	2,89428	1,63104	-2,3211	3,6331	NaN	2,85223	2,63207	2,89992
3,42673	3,8695	4,01055	4,04376	4,39196	0,914278	4,47038	3,82574	3,6439	4,17822
-0,673313	-0,410595	0,326927	0,0872707	0,610653	1,31518	0,973547	-0,290466	0,153644	0,610225
0,554464	0,446144	0,698713	1,34724	0,127436	1,96304	0,298998	0,71217	0,595893	-0,599261
1,44361	1,23861	0,497772	2,15356	2,19066	2,18298	1,63061	1,24601	1,21536	1,22597
3,51939	3,9206	3,68373	2,87261	4,22321	2,08555	4,04452	3,94974	4,24657	3,69437
-1,45925	-1,15457	-1,34749	-0,778639	-0,911903	NaN	-1,61226	-0,636789	-0,199784	-0,59513
NaN	NaN	NaN	0,500368	-1,50949	-0,294714	-0,0907784	NaN	NaN	NaN
NaN	NaN	NaN	NaN	NaN	NaN	NaN	NaN	NaN	NaN
NaN	NaN	NaN	-2,24607	NaN	-2,13405	-2,03241	NaN	NaN	NaN
NaN	-2,60036	-0,277687	0,00440598	1,18991	-1,45759	0,683887	NaN	NaN	NaN
NaN	NaN	NaN	0,0486393	-0,365726	1,11495	-0,169695	NaN	NaN	NaN
0,300249	0,0235634	1,11939	NaN	1,46822	NaN	NaN	0,700108	0,567527	0,747176
NaN	NaN	NaN	NaN	NaN	NaN	NaN	-0,78875	-0,474683	-1,17319
NaN	NaN	NaN	NaN	NaN	NaN	NaN	NaN	NaN	NaN
NaN	NaN	-0,561634	0,0755424	NaN	0,140018	0,0684109	NaN	NaN	NaN
-0,246506	-0,708109	NaN	NaN	-0,442276	-2,50476	NaN	NaN	NaN	-0,277396
NaN	NaN	NaN	0,546911	NaN	0,639099	-1,86733	NaN	NaN	NaN
NaN	NaN	NaN	NaN	NaN	NaN	NaN	NaN	NaN	NaN

Polito et al. Universal ready-to-use immunotherapeutic approach for the treatment of cancer: expanded and activated polyclonal γδ memory T cells.
Phosphorylation sites

NaN	NaN	1,47634	1,18353	1,2772	2,45013	0,665266	0,850159	1,56919	1,07424
NaN	NaN	NaN	0,0646172	-1,9622	-3,15519	NaN	NaN	NaN	NaN
NaN	NaN	NaN	NaN	NaN	NaN	NaN	-0,0942783	0,365987	0,270995
NaN	NaN	NaN	NaN	NaN	NaN	NaN	-3,22667	NaN	-2,40653
NaN	NaN	NaN	-1,17582	-1,1357	-0,682405	-1,11011	NaN	NaN	NaN
-2,20805	-1,74633	NaN	-1,17479	-0,968809	NaN	-1,73573	-0,821346	-0,679011	-0,719224
3,54584	5,45401	4,2401	4,14511	4,3219	4,65771	4,30127	3,82296	3,61829	5,00601
-0,190414	0,922165	0,4855	0,151932	0,189564	0,692871	0,975252	0,41209	-0,418262	0,264342
-1,19394	0,239597	-0,861557	NaN	NaN	NaN	NaN	NaN	NaN	NaN
NaN	NaN	-0,355417	-1,2274	-0,522585	NaN	NaN	-0,427118	-0,327527	-1,37702
NaN	NaN	NaN	NaN	NaN	NaN	NaN	NaN	NaN	NaN
NaN	NaN	NaN	-1,78227	NaN	-0,795601	-2,02912	NaN	NaN	NaN
NaN	NaN	-0,059103	NaN	NaN	NaN	NaN	-1,1104	-1,42197	-1,26193
NaN	NaN	NaN	NaN	NaN	NaN	NaN	NaN	NaN	NaN
-2,82042	-2,31074	0,72793	NaN	NaN	NaN	NaN	0,604591	0,299569	0,552421
NaN	NaN	NaN	-2,52295	NaN	-1,42171	-2,13331	-0,969374	NaN	-0,987512
0,502268	-0,0742893	3,11492	0,14628	1,63064	1,15457	NaN	3,16754	2,94489	2,94535
-1,96081	NaN	-1,51567	NaN	-0,551296	-1,44266	-0,783012	-2,44029	-2,4173	-2,25666
0,703272	1,82095	1,6555	1,39051	2,82183	-2,6638	2,71141	0,729513	0,699422	3,38856
1,17822	1,98793	0,0412464	1,0004	NaN	-1,73449	1,37463	0,0425663	1,07357	0,286262
NaN	-1,92534	-1,32677	-0,648491	NaN	NaN	-1,00506	-0,512213	-0,564033	-0,971404
NaN	-2,85051	-1,34998	NaN	-1,49467	NaN	-3,07733	-2,40565	-2,38406	-1,5898
NaN	NaN	NaN	NaN	NaN	NaN	NaN	NaN	NaN	NaN
-0,380457	NaN	-0,115232	-0,30485	-0,0463142	0,016325	0,018652	-0,756874	-0,323184	-0,936683
1,72403	2,45966	1,9686	2,66662	2,44041	2,36134	2,75351	1,35894	1,20205	2,42065
NaN	NaN	NaN	NaN	NaN	NaN	NaN	NaN	NaN	NaN
1,1855	0,249458	1,02526	2,7693	2,61467	2,31202	3,60178	1,6339	1,30615	1,88739
NaN	NaN	NaN	0,547541	-0,12007	NaN	-1,80432	-2,64203	-2,41643	-2,56657
-0,949902	-1,36047	-1,07186	-0,831242	-1,75339	-2,26575	-1,84911	-1,25078	-1,25163	-2,09331
-0,388353	0,377293	-0,712542	-0,558975	0,736191	-1,22507	0,0594959	0,257156	0,306418	-2,27576
NaN	NaN	NaN	-0,5275	NaN	NaN	-0,290548	-0,33536	0,00832272	-0,378364
NaN	NaN	NaN	0,0671635	NaN	0,107019	0,0718384	-3,21176	-2,96139	NaN
-2,84516	0,103312	-0,566586	NaN	NaN	0,429613	NaN	-0,779131	-1,20106	-0,265595
NaN	NaN	NaN	0,453751	-1,86092	0,629757	1,29454	-0,617146	-1,3913	-1,62908

Polito et al. Universal ready-to-use immunotherapeutic approach for the treatment of cancer: expanded and activated polyclonal γδ memory T cells.
Phosphorylation sites

NaN	NaN	NaN	-0,654612	-1,58724	-0,616362	-1,19531	NaN	NaN	NaN
2,20809	2,19776	1,89892	1,90266	1,79836	2,23013	1,91045	1,69796	1,66608	1,60851
-2,13593	-0,807177	-1,2974	-1,89561	-0,892086	NaN	NaN	-0,634388	-1,42874	-0,942536
-0,00263596	1,30006	0,961485	0,923796	0,708359	-3,31261	1,32421	0,648914	0,646295	0,798745
NaN	NaN	NaN	-0,617363	NaN	-0,506617	0,368137	NaN	NaN	NaN
NaN	NaN	NaN	NaN	NaN	NaN	NaN	-2,2752	-2,40922	-2,65169
0,855482	0,166109	2,15323	2,02523	NaN	3,22067	2,5498	2,15099	2,10265	2,45864
NaN	NaN	NaN	0,208979	-1,06588	NaN	-0,132401	NaN	NaN	NaN
NaN	NaN	NaN	NaN	NaN	NaN	NaN	-0,704193	-1,2357	-1,14166
1,50419	1,59914	1,4547	2,29193	1,61754	2,42875	2,05733	1,18006	0,983499	1,50526
NaN	NaN	NaN	1,76715	-1,59546	2,21666	1,63032	NaN	NaN	NaN
NaN	NaN	NaN	-0,684404	NaN	-0,304804	-0,785244	NaN	NaN	NaN
NaN	NaN	NaN	-1,04912	NaN	-0,346554	-1,63103	NaN	NaN	NaN
NaN	NaN	NaN	NaN	NaN	NaN	NaN	NaN	NaN	NaN
-1,47641	-1,29082	-1,14535	-1,35428	-1,33023	-0,923046	-1,16919	-1,15936	-1,35344	-1,36977
NaN	NaN	NaN	-1,65432	NaN	-1,11978	-1,05034	NaN	NaN	NaN
0,0408859	0,661676	0,972654	1,11135	-0,254807	NaN	0,781242	0,129179	1,54163	0,137774
-4,02684	NaN	-0,886106	NaN	NaN	NaN	NaN	0,353891	0,59752	NaN
NaN	NaN	NaN	2,3706	-0,526062	2,47032	2,11691	NaN	NaN	NaN
-0,817385	-1,71372	-1,62924	-0,361656	NaN	NaN	-1,19005	-0,945641	-0,917014	-1,75093
NaN	NaN	NaN	NaN	NaN	NaN	NaN	NaN	NaN	NaN
NaN	NaN	-2,80053	-2,31346	NaN	NaN	-0,484077	-1,58544	-1,96501	-1,60243
NaN	NaN	NaN	NaN	NaN	NaN	NaN	NaN	NaN	NaN
-1,42091	NaN	-0,344395	-1,53015	-1,57186	1,74546	-0,596495	0,00156975	NaN	NaN
-1,6002	-1,6682	-1,52308	NaN	-1,85999	NaN	NaN	-1,213	-1,17596	-1,67393
NaN	NaN	NaN	NaN	NaN	NaN	NaN	NaN	NaN	NaN
NaN	NaN	NaN	NaN	NaN	NaN	NaN	-1,39873	NaN	-1,88157
NaN	NaN	NaN	1,54697	NaN	1,62906	0,639963	NaN	NaN	NaN
NaN	NaN	NaN	-0,4701	-4,9634	NaN	-0,804825	NaN	NaN	NaN
NaN	1,1643	NaN	0,241838	2,32446	-0,515772	0,99725	1,96015	1,87144	1,70838
2,74964	1,88881	2,76968	3,74077	3,16525	NaN	2,14785	1,3138	0,549424	1,74252
NaN	NaN	NaN	-0,635468	NaN	-0,44136	-2,07123	NaN	NaN	NaN
-2,46398	NaN	-1,96323	-0,706043	-1,94776	-1,42017	-0,397696	NaN	NaN	-1,07116
NaN	NaN	NaN	NaN	NaN	NaN	NaN	NaN	NaN	NaN

Polito et al. Universal ready-to-use immunotherapeutic approach for the treatment of cancer: expanded and activated polyclonal γδ memory T cells.
Phosphorylation sites

NaN	NaN	NaN	0,129372	-0,472452	-1,62635	-0,0774879	-2,33397	-1,25682	-1,20411
NaN	NaN	NaN	NaN	NaN	NaN	NaN	NaN	NaN	NaN
1,55506	0,869335	2,15823	1,52255	3,16401	3,05565	2,39538	1,40195	1,75127	0,956069
3,02641	2,74991	2,10088	2,5029	2,34419	-1,24595	2,76621	2,62398	1,94103	3,94356
NaN	NaN	NaN	NaN	NaN	NaN	NaN	NaN	NaN	NaN
2,29129	0,604115	1,86319	0,82909	3,39664	1,72723	3,28552	1,88765	1,60644	1,64029
-5,49805	-2,45763	NaN	-3,2137	-1,94098	-2,59978	-2,69896	NaN	NaN	NaN
NaN	NaN	NaN	-1,0102	NaN	NaN	-2,15803	NaN	NaN	NaN
NaN	NaN	NaN	NaN	NaN	NaN	NaN	-1,04935	-1,34915	-1,48722
-0,887459	-1,63763	-0,636663	-0,952038	NaN	-2,18949	-0,0783844	0,719156	0,55446	0,64869
-2,31305	-2,70029	-2,15372	NaN	-1,85948	NaN	-1,37052	NaN	NaN	NaN
NaN	NaN	NaN	-2,14545	-2,17464	NaN	-0,0228844	NaN	NaN	NaN
-1,4582	NaN	-0,415045	1,2612	-1,5044	1,36176	-0,249802	NaN	-1,15119	NaN
-1,95794	NaN	0,643606	-1,30158	-1,57067	NaN	1,88332	-1,23608	-0,900535	NaN
-1,90608	-3,74025	NaN	0,118914	NaN	1,14328	-0,563854	-1,77892	-1,20758	-1,87861
2,89067	3,0766	3,08508	-1,23011	0,879034	-0,350428	0,2351	3,06841	3,25905	3,26359
-2,67813	-1,50085	-2,35111	NaN	-2,29324	NaN	-2,18278	-1,69819	NaN	-2,04422
-2,13006	-1,85924	1,46579	0,127354	-1,63563	0,7605	-0,074379	1,42694	1,81753	1,54521
-0,157007	0,548643	-0,00524521	-0,984295	0,66938	-0,955793	0,101593	1,01548	NaN	0,805064
0,38434	NaN	-0,599447	-0,996492	-0,265158	NaN	0,259743	-0,040266	-0,0874414	-0,0462046
4,34855	4,13713	4,86954	5,31422	5,56055	4,74168	5,93314	5,67872	5,325	5,86067
NaN	NaN	NaN	-1,80316	-1,49379	-1,49832	-1,51185	NaN	NaN	NaN
-2,04655	-3,02098	-1,0703	1,71563	-1,08042	0,779823	0,0206127	-0,252325	-0,0910425	-0,357398
-3,08398	-2,27471	-0,550104	1,46977	-1,5478	0,135225	-0,504444	0,155102	-0,16311	-0,421451
-0,986759	NaN	NaN	NaN	-1,18352	-0,115597	-1,52285	NaN	NaN	NaN
NaN	NaN	NaN	NaN	NaN	NaN	NaN	NaN	NaN	NaN
-1,63437	-0,193232	-1,14727	0,799084	NaN	1,11113	-0,0981255	-1,99843	-1,01408	-1,72809
NaN	NaN	NaN	NaN	NaN	NaN	NaN	NaN	NaN	NaN
-3,58077	-1,53286	-1,01027	-0,0213318	-1,8661	-0,690351	-0,157431	NaN	NaN	NaN
NaN	-0,786177	-2,08124	-1,66606	NaN	0,124273	-1,89791	NaN	NaN	NaN
-2,14964	-1,12023	-1,04034	-1,01346	NaN	-1,25728	-1,32782	-1,70647	NaN	-0,636662
-0,0784054	0,326143	NaN	-0,335001	-0,204338	NaN	NaN	NaN	NaN	NaN
1,92572	1,41213	1,81128	1,96459	2,06059	2,3312	2,08134	NaN	1,63412	1,42992
2,69071	2,50995	1,45779	1,47877	1,60133	3,16391	2,24581	2,70933	3,18819	2,3801

Polito et al. Universal ready-to-use immunotherapeutic approach for the treatment of cancer: expanded and activated polyclonal γδ memory T cells.
Phosphorylation sites

NaN	NaN	NaN	-1,17329	-3,36598	NaN	-2,25561	NaN	NaN	NaN
3,71552	4,58209	4,87583	4,08269	2,39378	-1,5289	5,04113	3,92383	4,52567	4,90117
NaN	NaN	NaN	NaN	NaN	NaN	NaN	NaN	NaN	NaN
NaN	NaN	NaN	-1,84726	-0,83782	-1,53251	-2,70003	NaN	NaN	NaN
-1,55956	-1,55269	-1,12657	NaN	NaN	NaN	-1,58519	-1,66919	-1,50551	-1,73656
NaN	-2,30611	NaN	-1,83258	NaN	-1,01743	-2,04471	NaN	NaN	NaN
NaN	NaN	NaN	-0,453768	0,541544	NaN	NaN	0,547916	0,789386	1,17748
NaN	NaN	NaN	NaN	NaN	NaN	NaN	-1,0555	-1,82436	-1,43684
NaN	NaN	NaN	-0,238071	-0,651217	NaN	-0,268713	NaN	NaN	NaN
NaN	NaN	NaN	NaN	NaN	NaN	NaN	NaN	NaN	NaN
NaN	NaN	NaN	0,168825	-1,39343	0,495781	-0,901367	NaN	NaN	NaN
-0,413492	-0,797422	-0,92104	-1,34594	1,29098	-1,51346	NaN	0,0909557	-0,0562944	NaN
-2,12119	-2,09892	-2,69662	0,35387	-1,45461	NaN	-1,30688	-1,64753	-2,01354	-1,84979
-0,900608	-0,99107	-1,94524	-0,142834	-0,0825539	NaN	0,701998	-1,82016	-1,69058	0,240239
-1,44928	-0,634583	-0,0204067	-0,876118	0,351126	NaN	NaN	-0,168388	-0,149169	-0,287835
-0,50971	-0,295212	NaN	NaN	0,271063	0,212963	NaN	NaN	NaN	NaN
NaN	NaN	NaN	NaN	-1,02146	NaN	NaN	NaN	NaN	NaN
0,181948	-0,58182	-0,493027	0,0774956	-0,658064	-1,24663	0,511948	-0,925934	0,10308	-0,802909
-0,927992	-1,67033	-1,31143	0,233217	NaN	NaN	-0,231531	NaN	NaN	NaN
NaN	NaN	NaN	-1,33287	-0,393618	-1,52083	-0,631517	-1,63594	-1,26833	-1,19168
NaN	NaN	NaN	NaN	-1,73255	-2,21741	-1,68232	NaN	NaN	NaN
-0,402534	-1,84317	-0,977766	-0,605904	0,125769	-1,66099	0,334452	-1,16982	-1,17999	-1,02462
1,70074	1,25221	0,98288	1,49725	2,27831	1,07666	1,99125	1,16924	NaN	0,761191
0,721798	0,223543	-0,126898	0,770382	NaN	1,53087	0,4312	NaN	NaN	-0,72969
-0,00201035	0,326391	0,319948	0,540859	1,06719	0,954966	1,24938	0,419888	0,257161	-0,232594
-0,317661	NaN	NaN	NaN	NaN	NaN	NaN	0,107742	-0,660319	-1,57325
5,39422	5,84329	5,46622	4,30811	5,37699	6,18356	4,37042	5,17223	6,11042	6,08224
NaN	NaN	NaN	NaN	NaN	NaN	NaN	NaN	NaN	NaN
4,51682	3,48762	4,8996	5,18153	4,91875	5,24065	4,35325	5,39906	5,09196	5,05046
6,19662	6,81343	6,81789	6,90532	6,86769	6,80203	6,98957	7,07629	6,84589	6,9887
0,0623989	-1,55434	0,848547	2,30661	0,487934	2,37815	2,9829	1,30837	1,00369	1,22724
-0,256176	-0,464024	0,0286484	-0,0724888	0,479231	NaN	0,0671101	-0,0613728	-0,263301	-0,24614
-1,2987	NaN	-2,00346	NaN	NaN	NaN	NaN	-1,10015	-1,63653	-2,29477
-0,167147	NaN	0,616074	NaN	-0,403135	NaN	NaN	-0,675426	-0,129292	-0,59101

Polito et al. Universal ready-to-use immunotherapeutic approach for the treatment of cancer: expanded and activated polyclonal γδ memory T cells.
Phosphorylation sites

-0,936981	-0,350388	-0,77704	0,830021	0,467537	0,174194	-0,0948296	-0,749809	-1,13972	-0,588283
-1,53458	NaN	1,00713	NaN	-1,5922	-0,391699	-0,540653	-0,578642	-0,798808	-1,1617
NaN	NaN	NaN	-1,68686	NaN	-1,08739	-0,546778	NaN	NaN	NaN
NaN	NaN	NaN	NaN	NaN	NaN	NaN	NaN	NaN	NaN
NaN	NaN	NaN	NaN	NaN	NaN	NaN	NaN	NaN	NaN
NaN	NaN	NaN	NaN	NaN	NaN	NaN	NaN	NaN	NaN
NaN	NaN	NaN	NaN	NaN	NaN	NaN	NaN	NaN	NaN
-1,80684	NaN	-0,328657	NaN	NaN	NaN	NaN	-1,81629	-2,3934	-2,46539
NaN	NaN	NaN	NaN	NaN	NaN	NaN	NaN	NaN	NaN
NaN	NaN	NaN	NaN	NaN	NaN	NaN	NaN	NaN	NaN
NaN	NaN	NaN	1,29388	-1,67005	0,869986	NaN	NaN	NaN	NaN
-0,402828	-0,0251427	NaN	NaN	0,643785	NaN	NaN	0,136707	0,0909986	0,680297
3,74109	5,93842	4,49889	4,25278	6,45707	1,5188	5,40153	4,12926	3,53806	6,12712
NaN	NaN	NaN	NaN	NaN	NaN	NaN	-1,65615	-2,02902	-1,90638
3,74109	5,93842	4,49889	4,25278	6,45707	1,5188	5,40153	4,12926	3,53806	6,12712
4,12695	4,21377	4,85452	3,39972	4,39899	4,106	4,07852	4,42478	4,86735	4,54937
NaN	NaN	NaN	1,21832	-2,74679	0,885246	0,663715	NaN	NaN	NaN
-1,87431	-1,52414	-3,41696	-1,60859	-1,10223	-1,7753	NaN	NaN	NaN	NaN
NaN	NaN	NaN	1,12262	NaN	NaN	-0,550209	NaN	NaN	NaN
NaN	NaN	NaN	2,45365	NaN	-0,816111	1,18056	NaN	NaN	NaN
NaN	NaN	NaN	NaN	NaN	NaN	NaN	NaN	NaN	NaN
NaN	NaN	NaN	NaN	NaN	NaN	NaN	NaN	NaN	NaN
NaN	NaN	NaN	1,54697	NaN	1,62906	0,639963	NaN	NaN	NaN
NaN	NaN	NaN	-1,00854	NaN	NaN	-1,38136	NaN	NaN	NaN
6,49018	8,2039	11,2091	-1,68863	5,40128	-1,6943	NaN	12,2981	12,2001	12,0621
NaN	NaN	NaN	1,08568	NaN	-0,112873	-0,180038	-1,00342	-1,71906	-1,30835
NaN	NaN	NaN	1,25174	NaN	-0,506771	-0,669399	NaN	NaN	NaN
-1,74965	-0,162289	NaN	-1,95012	-1,5493	0,775	NaN	NaN	-0,193837	-1,33641
NaN	NaN	NaN	NaN	-2,46139	0,367157	NaN	0,0200386	-0,998094	0,108399
-2,24831	NaN	-1,78941	-0,750008	NaN	NaN	-0,876619	-1,42307	-1,29259	-2,96872
NaN	NaN	NaN	NaN	NaN	NaN	NaN	NaN	NaN	NaN
-2,58733	NaN	NaN	NaN	NaN	NaN	NaN	NaN	NaN	NaN
0,202868	-0,530918	-1,76709	0,762167	NaN	NaN	NaN	NaN	-1,78009	NaN
NaN	NaN	NaN	NaN	NaN	NaN	NaN	NaN	NaN	NaN

Polito et al. Universal ready-to-use immunotherapeutic approach for the treatment of cancer: expanded and activated polyclonal γδ memory T cells.
Phosphorylation sites

NaN	NaN	-0,293264	NaN	NaN	-1,34963	-0,865623	NaN	NaN	NaN
-1,26543	-1,94176	-2,21597	-1,24961	-2,18352	-1,69547	-1,69283	-2,27677	-2,28118	-2,04658
-1,98208	-1,15773	-2,16774	-1,04608	NaN	-0,546474	-1,2409	-1,16221	NaN	-1,72036
NaN	-1,48803	-1,75487	NaN	-0,284212	-2,01748	0,366976	-0,749767	-0,681806	-1,6293
-0,779114	-2,023	-1,20332	5,38006	-0,616539	6,15021	3,92386	-0,550423	-0,00158024	0,029563
-0,829987	-1,00379	-1,16175	NaN	-1,14541	0,494165	-0,366302	-0,165644	-0,292794	-0,249627
NaN	NaN	NaN	-1,28442	NaN	NaN	-1,16965	NaN	NaN	NaN
NaN	NaN	NaN	-0,0891685	NaN	0,185724	0,268822	NaN	NaN	NaN
NaN	-2,34041	2,78486	NaN	NaN	NaN	NaN	-0,195524	-0,295234	0,423488
3,78796	3,9392	2,504	5,46762	5,01044	5,91112	4,85815	2,93737	2,52775	2,62501
NaN	-0,767387	NaN	-0,352262	-1,07556	-0,167276	-0,832176	NaN	NaN	NaN
NaN	NaN	NaN	-2,67995	NaN	NaN	-1,69544	NaN	NaN	NaN
NaN	NaN	1,49074	1,25236	NaN	1,60704	2,26001	NaN	NaN	NaN
-1,27518	NaN	-0,557917	1,7417	-1,58596	NaN	2,35039	-0,66855	-1,23585	-0,702172
NaN	NaN	NaN	NaN	NaN	NaN	NaN	-0,790907	NaN	-1,52701
NaN	NaN	NaN	-0,582592	0,0356636	-2,23705	0,2488	NaN	NaN	NaN
-2,41245	-2,19873	-1,49598	-3,14938	-2,65292	-2,87577	-2,23284	NaN	NaN	NaN
NaN	NaN	NaN	NaN	NaN	NaN	NaN	NaN	NaN	NaN
0,799387	0,982225	0,168854	NaN	-0,644135	NaN	NaN	NaN	NaN	NaN
NaN	NaN	-0,362076	-1,29964	-1,7446	-1,47814	NaN	0,642904	-0,00166225	-0,291707
NaN	NaN	NaN	NaN	NaN	NaN	NaN	-2,46432	-2,27036	-2,06577
1,14722	0,73885	0,66221	1,17696	NaN	0,844919	2,18656	1,70792	1,76907	1,82664
NaN	NaN	NaN	NaN	NaN	NaN	NaN	NaN	NaN	NaN
-1,9493	-1,47741	-0,541348	-1,23512	-0,625811	NaN	NaN	-0,96299	0,889649	1,07413
1,04913	0,0456467	0,232264	7,60835	-0,362066	5,96866	5,3171	0,360338	0,79979	0,493541
-0,715132	NaN	-1,66798	NaN	2,62715	NaN	NaN	-0,267826	-0,593314	-1,96225
3,74109	5,93842	4,49889	4,25278	6,45707	1,5188	5,40153	4,12926	3,53806	6,12712
NaN	NaN	NaN	-1,51752	NaN	-0,725031	NaN	NaN	NaN	NaN
NaN	NaN	NaN	-1,02232	NaN	-0,504559	NaN	-2,40856	-2,33274	-2,71994
6,13392	6,66361	6,35516	6,74329	7,81874	6,30578	7,08142	5,88884	-1,03548	7,52689
NaN	NaN	NaN	NaN	NaN	NaN	NaN	NaN	NaN	NaN
NaN	NaN	NaN	1,98028	-1,77327	1,47537	0,245337	NaN	NaN	NaN
NaN	NaN	NaN	-0,0914001	-2,27125	-2,20333	NaN	NaN	NaN	NaN
NaN	NaN	NaN	-0,587004	NaN	0,0593967	-2,32871	NaN	NaN	NaN

Polito et al. Universal ready-to-use immunotherapeutic approach for the treatment of cancer: expanded and activated polyclonal γδ memory T cells.
Phosphorylation sites

NaN	-0,833473	-1,30539	2,11381	0,522667	1,6496	-0,522146	0,488108	0,173327	0,357205
0,464949	0,808727	1,95089	0,401546	1,25307	0,947466	NaN	1,51309	1,71206	1,10016
3,67166	2,14157	2,70883	5,18875	4,17512	4,96901	3,01411	3,3963	2,89655	2,56341
NaN	0,338886	0,35903	NaN	NaN	NaN	NaN	-0,207916	1,39882	0,164351
NaN	NaN	NaN	NaN	NaN	NaN	NaN	NaN	NaN	NaN
NaN	NaN	NaN	-2,24207	-3,51459	-0,728502	-1,03717	NaN	NaN	NaN
2,59402	1,91858	2,97365	5,04777	3,40676	4,07569	4,28691	3,39632	2,73362	2,82239
NaN	NaN	NaN	NaN	NaN	NaN	NaN	-0,560282	-0,669847	0,0813684
3,83041	2,32222	3,28757	3,36442	3,47068	-0,381079	3,04463	3,50045	3,2749	3,4794
NaN	NaN	NaN	3,32712	0,125092	3,79	1,9267	NaN	NaN	NaN
NaN	NaN	NaN	NaN	NaN	NaN	NaN	NaN	NaN	NaN
-0,499607	-1,55018	NaN	-1,56383	-0,540546	-1,78728	-1,21095	-1,18499	-1,00808	-1,00675
NaN	NaN	NaN	-1,19792	NaN	NaN	-0,552895	NaN	NaN	NaN
NaN	NaN	NaN	-1,90515	NaN	-1,8	-1,69298	NaN	NaN	NaN
NaN	NaN	-1,5201	NaN	NaN	NaN	NaN	NaN	NaN	-1,78461
NaN	NaN	NaN	-1,3743	NaN	-1,01544	-0,593592	NaN	NaN	NaN
-1,13618	NaN	-2,02047	-1,56949	-1,57524	-1,53653	-1,18618	NaN	NaN	NaN
NaN	NaN	NaN	-1,47761	NaN	-0,601559	-0,870396	NaN	NaN	NaN
NaN	NaN	NaN	-0,225185	-0,869781	NaN	0,0109978	NaN	NaN	NaN
NaN	NaN	NaN	NaN	NaN	NaN	NaN	1,65195	NaN	NaN
-2,25309	-2,04656	0,840755	-0,450148	NaN	6,71459	-0,934469	-0,65954	NaN	-0,944877
NaN	NaN	NaN	NaN	NaN	NaN	NaN	NaN	NaN	NaN
NaN	NaN	NaN	NaN	NaN	NaN	NaN	NaN	NaN	NaN
0,741022	0,527615	1,041	0,179058	NaN	-2,22827	0,0238037	1,11476	1,32617	-0,106324
0,162556	0,626387	-0,44627	NaN	NaN	0,234921	-0,225327	NaN	0,623313	NaN
NaN	-1,57137	-1,09209	-0,64613	NaN	NaN	-0,480829	NaN	-0,539769	-0,522061
1,70861	NaN	2,30455	NaN	2,59781	2,94796	2,21201	2,60709	2,42743	NaN
NaN	NaN	NaN	NaN	NaN	NaN	NaN	NaN	NaN	NaN
NaN	NaN	NaN	NaN	NaN	NaN	NaN	NaN	NaN	NaN
-1,06398	0,116678	-0,649408	0,582211	0,837616	0,840508	0,647972	-1,03537	-1,04336	-0,356751
NaN	NaN	NaN	NaN	NaN	NaN	NaN	NaN	NaN	NaN
-0,781261	-0,315113	NaN	-1,11134	0,345005	-2,58863	-0,890265	1,16056	-0,253171	0,217643
-0,881319	-3,28515	-0,391636	0	0,302179	NaN	-0,0215454	-0,982965	-0,68533	-0,352612
NaN	NaN	NaN	NaN	NaN	NaN	NaN	NaN	NaN	NaN

Polito et al. Universal ready-to-use immunotherapeutic approach for the treatment of cancer: expanded and activated polyclonal γδ memory T cells.
Phosphorylation sites

0,0675411	0,211485	NaN	-3,13838	0,445597	0,481091	NaN	NaN	NaN	NaN
NaN	1,44649	0,656977	1,74604	1,90864	0,709015	1,86147	-0,208866	-0,613065	1,22665
NaN	NaN	NaN	1,11678	NaN	1,45768	-1,37851	NaN	NaN	NaN
6,28371	6,44343	6,34494	5,93685	7,21343	6,06195	6,38379	6,79454	6,45164	6,87445
4,66618	5,39518	4,41533	5,02333	4,28032	4,73517	5,17111	4,65941	4,63379	4,9995
3,68645	2,12713	3,78578	4,71328	4,20167	3,94928	3,05685	3,32743	2,76377	2,98838
6,28371	6,44343	6,34494	5,93685	7,21343	6,06195	6,38379	6,79454	6,45164	6,87445
NaN	1,77029	NaN	1,87226	2,81781	NaN	NaN	1,41763	0,931865	0,603564
NaN	NaN	NaN	-0,898663	-1,33338	NaN	NaN	-0,845839	-2,17179	-0,82754
NaN	NaN	NaN	0,249947	NaN	1,85038	-2,9917	NaN	NaN	NaN
NaN	NaN	NaN	NaN	NaN	NaN	NaN	NaN	NaN	NaN
NaN	NaN	NaN	-1,52777	NaN	NaN	NaN	NaN	NaN	NaN
NaN	NaN	NaN	NaN	NaN	NaN	NaN	NaN	NaN	NaN
-1,16557	-2,11442	-0,643536	0,260098	-1,22068	-0,788567	0,363941	-1,2149	-0,77805	-0,126275
NaN	NaN	NaN	-0,125404	NaN	NaN	0,0582314	NaN	NaN	NaN
NaN	NaN	-1,29267	1,81207	NaN	1,16246	0,636374	NaN	NaN	NaN
2,92663	3,55093	3,21861	2,51208	4,58107	2,15304	2,66026	3,01168	3,11162	3,15761
NaN	NaN	NaN	0,121199	NaN	-0,542	NaN	NaN	NaN	NaN
-2,25277	-1,62484	NaN	-0,786133	-1,16836	-2,50531	-0,607008	-1,78234	-1,67858	-1,3559
0,914223	0,472656	NaN	1,17964	0,628695	1,2029	0,462027	NaN	NaN	NaN
NaN	NaN	NaN	NaN	NaN	NaN	NaN	NaN	NaN	NaN
NaN	NaN	NaN	NaN	NaN	NaN	NaN	-1,58903	-0,80894	-2,13373
-0,782757	-0,366003	NaN	0,0780354	1,18792	NaN	1,27823	-0,00412178	0,159053	0,219083
NaN	-0,128025	NaN	-1,31232	0,237499	-2,4034	-0,161949	-1,23408	-1,17512	-0,357162
0,172056	0,293468	-0,187593	NaN	NaN	NaN	NaN	-0,411442	NaN	NaN
2,55046	2,03598	1,70854	3,78824	3,75345	3,39847	3,09874	2,11499	1,87709	1,86715
3,26016	2,98301	3,65847	4,145	3,37461	1,33317	3,02225	2,45031	3,2244	2,34796
NaN	NaN	NaN	-2,87977	NaN	-2,2713	0,206877	NaN	NaN	NaN
NaN	NaN	NaN	-0,220787	NaN	-1,852	-1,21525	NaN	NaN	NaN
NaN	NaN	NaN	NaN	NaN	NaN	NaN	NaN	NaN	NaN
NaN	NaN	NaN	-1,21574	-1,45228	NaN	-2,35694	NaN	NaN	NaN
NaN	NaN	NaN	NaN	NaN	NaN	NaN	-1,92054	-2,00133	-0,636842
-1,73435	-1,38867	-0,890972	0,816202	NaN	-1,78741	NaN	-1,37304	-1,57462	-1,71809
NaN	NaN	NaN	-2,76899	NaN	-1,75935	-3,17442	NaN	NaN	NaN

Polito et al. Universal ready-to-use immunotherapeutic approach for the treatment of cancer: expanded and activated polyclonal γδ memory T cells.
Phosphorylation sites

4,8517	5,54784	4,43917	4,85925	5,12909	5,56333	5,02722	4,5692	3,86052	5,06753
-1,59973	0,476244	0,878832	0,122684	1,15969	-0,791323	0,53887	1,14789	1,51151	0,705424
-1,73851	-0,141296	0,117981	-1,57699	-0,145067	NaN	-0,300781	-0,352009	-0,565427	0,555499
NaN	-1,51085	NaN	-2,90502	-0,699249	NaN	-1,43988	NaN	NaN	-0,0882063
NaN	NaN	NaN	NaN	NaN	NaN	NaN	NaN	NaN	NaN
NaN	NaN	NaN	NaN	NaN	NaN	NaN	NaN	NaN	NaN
NaN	NaN	NaN	-0,410711	NaN	-1,17532	-1,24397	0,583389	NaN	NaN
-0,180769	0,345032	0,131996	-1,26937	-0,253315	-1,23395	-0,186687	0,311428	-0,361695	0,310744
NaN	NaN	NaN	-3,32548	NaN	-1,27765	-2,52826	NaN	NaN	NaN
NaN	NaN	NaN	NaN	NaN	NaN	NaN	NaN	NaN	NaN
-0,322565	0,36101	0,0708714	-0,681129	0,628769	NaN	0,372425	-0,318092	0,0847654	0,0552797
NaN	NaN	NaN	-1,42953	NaN	NaN	-1,52478	NaN	NaN	NaN
-1,40753	-1,26091	1,38936	2,86092	-0,614361	-1,30643	1,49218	1,3226	1,07454	1,0205
NaN	NaN	NaN	0,94677	-0,368073	1,0545	-0,082283	-0,486475	-0,500941	0,344121
NaN	NaN	NaN	NaN	NaN	NaN	NaN	NaN	NaN	NaN
NaN	NaN	NaN	NaN	NaN	NaN	NaN	NaN	NaN	NaN
NaN	1,85821	-0,318129	NaN	NaN	NaN	NaN	NaN	NaN	NaN
NaN	NaN	NaN	-1,8894	-1,43419	NaN	-1,84501	-2,21653	-1,83691	-2,52042
NaN	NaN	NaN	NaN	NaN	NaN	NaN	NaN	NaN	NaN
NaN	NaN	NaN	-1,07293	-2,49661	-1,68749	0,331488	NaN	NaN	NaN
NaN	NaN	NaN	NaN	NaN	NaN	NaN	NaN	NaN	NaN
NaN	NaN	NaN	NaN	NaN	NaN	NaN	-1,72779	-2,01445	-1,94351
NaN	-1,80734	0,723444	NaN	-0,880367	NaN	NaN	1,72409	1,57539	1,5183
NaN	NaN	NaN	-1,62914	-2,24919	-2,51644	-1,29795	NaN	NaN	NaN
-1,3224	-1,92954	-1,79764	-2,51736	-1,20244	NaN	-2,3192	NaN	NaN	NaN
NaN	NaN	NaN	-1,43017	NaN	NaN	-2,23958	NaN	NaN	NaN
-2,92662	NaN	1,58765	-0,974472	NaN	-0,460409	-0,684692	NaN	NaN	NaN
-1,1922	0,657238	0,0654526	-1,2469	-0,691515	-2,45996	-0,141813	-1,43293	0,279889	0,243829
-0,387711	-1,66015	-0,96418	-0,719358	0,0602779	-0,96697	-1,02116	-1,24002	-1,2177	-1,21179
-0,554802	NaN	0,636261	NaN	2,72147	NaN	4,22552	1,15553	1,10746	0,138442
-2,48257	5,62698	3,03654	3,61228	4,91235	NaN	NaN	2,32629	3,11404	NaN
-0,545683	-1,07627	-0,969774	-1,73579	-0,314724	-2,09109	-0,39452	-0,940653	-1,1437	0,672111
NaN	NaN	NaN	NaN	NaN	NaN	NaN	NaN	NaN	NaN
1,15074	NaN	1,90125	1,90845	0,98554	2,05557	NaN	NaN	NaN	NaN

Polito et al. Universal ready-to-use immunotherapeutic approach for the treatment of cancer: expanded and activated polyclonal γδ memory T cells.
Phosphorylation sites

-0,0442638	-0,064497	0,736097	-1,7468	0,480944	NaN	-1,25801	0,697773	0,88288	0,724439
-1,22527	4,19032	-1,34442	-0,934961	4,25698	-2,62891	-0,602488	NaN	NaN	5,3172
3,58617	4,64103	3,83144	4,90616	5,3162	3,85562	4,95506	3,87713	3,45853	5,4858
NaN	NaN	NaN	NaN	NaN	NaN	NaN	-1,1692	-0,800164	-0,622392
0,912563	4,1096	2,93349	2,8575	4,46046	0,478254	3,3734	1,98713	1,77544	5,25786
NaN	NaN	NaN	NaN	NaN	NaN	NaN	NaN	NaN	NaN
NaN	NaN	NaN	-1,20634	NaN	-1,35809	-2,02402	NaN	NaN	NaN
0,368158	-0,378952	1,19299	-1,38228	0,403805	-0,600721	-0,330624	1,19332	0,873509	0,524693
NaN	-1,78978	-0,814463	-0,447763	-2,31303	-1,41994	-0,422461	-1,05426	-1,07128	-0,660441
NaN	NaN	NaN	-3,33457	NaN	-3,25645	-1,67374	NaN	NaN	NaN
NaN	NaN	NaN	-1,66361	NaN	0,161179	-1,34259	NaN	NaN	NaN
-2,22715	-1,03263	0,567841	0,461248	-2,44525	-0,495508	0,829199	0,0411606	0,327197	0,186925
NaN	-1,40995	0,740671	0,381084	-2,04192	-0,146832	0,0991344	NaN	NaN	NaN
NaN	-2,57702	-2,19952	-0,115692	NaN	-0,964373	NaN	-1,31263	-1,85319	-2,16591
-1,21331	-0,0688381	-1,64413	-0,900103	-1,90688	NaN	-1,95681	-1,34857	0,145247	-0,541705
-1,20588	-1,68303	-0,204983	0,697329	-1,28331	0,914532	0,101803	-0,799171	-0,718726	-1,09375
1,68347	1,51502	1,96361	0,58049	-0,117075	0,601446	1,1182	2,41436	2,8271	2,0372
NaN	NaN	NaN	-0,776812	-1,90348	-0,593416	NaN	NaN	-2,24165	-1,85351
NaN	NaN	NaN	-0,779364	NaN	-1,02283	-2,45034	NaN	NaN	NaN
NaN	NaN	NaN	NaN	NaN	NaN	NaN	NaN	NaN	NaN
-2,25383	-0,860712	-1,59413	NaN	NaN	NaN	NaN	NaN	NaN	NaN
-1,48457	-0,609623	-0,172655	NaN	NaN	-1,72463	NaN	-0,521814	-0,286403	-0,292804
NaN	-1,59541	NaN	-0,487854	-0,423126	NaN	NaN	-1,04281	-0,781007	-1,2923
1,76571	2,04435	1,65483	1,66053	2,57342	1,72737	1,0346	2,08215	2,13406	2,41494
NaN	NaN	0,1593	-1,70454	NaN	-2,12675	0,323528	NaN	NaN	NaN
-1,87431	NaN	-0,841288	NaN	0,293251	-0,685738	NaN	-0,890038	-0,535392	-0,951385
NaN	NaN	NaN	-1,58232	0,725483	-1,61075	NaN	-1,29857	-1,84573	-0,544446
-0,759598	-2,36813	0,997391	-1,5628	0,675375	-1,40835	-1,36786	0,821512	0,513526	1,71349
0,620821	0,664726	-0,585831	0,325399	0,249651	1,08363	-0,220449	-0,204002	-0,014905	-0,115096
1,78883	0,73698	0,482819	1,78933	0,511621	1,70192	0,278435	0,0487766	0,627965	0,58398
NaN	NaN	-1,22387	1,49098	NaN	-1,95686	-0,974878	0,0509682	-0,0847425	NaN
NaN	NaN	NaN	NaN	NaN	NaN	NaN	4,1375	4,85649	2,36057
NaN	NaN	NaN	-0,308762	NaN	-1,26624	0,0847511	NaN	NaN	NaN
1,09269	1,78912	1,32153	-0,0689945	1,70944	0,33721	NaN	0,966797	1,56946	0,846749

Polito et al. Universal ready-to-use immunotherapeutic approach for the treatment of cancer: expanded and activated polyclonal γδ memory T cells.
Phosphorylation sites

NaN	NaN	NaN	NaN	NaN	NaN	NaN	NaN	NaN	NaN
1,03045	1,41739	1,17358	1,04617	0,704229	1,43627	1,37549	1,20592	2,59752	2,13307
1,18639	1,0704	-0,244362	1,15649	0,835035	NaN	1,3828	0,670654	0,566281	0,416884
NaN	NaN	0,396742	1,65289	-1,38457	1,91523	NaN	NaN	NaN	NaN
-1,79587	NaN	-0,988115	-0,350573	-0,00667	NaN	-0,0583973	-1,6857	-1,90856	-1,67063
NaN	NaN	NaN	0,201277	NaN	-0,0279503	1,63083	NaN	NaN	NaN
-0,802748	-0,159433	NaN	-0,119761	-1,55622	NaN	-0,249855	-0,945976	NaN	-1,51594
NaN	0,541918	-1,67954	-1,24037	0,899054	-1,35505	1,08002	-2,86499	-2,758	0,0446615
NaN	-1,33738	-0,926386	-1,43926	NaN	-2,39021	0,657637	-1,00938	-1,08092	-0,0618811
NaN	1,45122	1,69341	1,72467	NaN	1,71851	0,71703	1,15071	NaN	NaN
NaN	NaN	NaN	NaN	NaN	NaN	NaN	NaN	NaN	NaN
NaN	NaN	NaN	-2,15533	NaN	NaN	NaN	NaN	NaN	NaN
NaN	-0,206921	-1,21562	NaN	-2,23587	0,559454	0,824411	-0,467485	-0,556804	-0,463298
NaN	-0,8162	-2,57051	-0,0349979	-1,66924	-0,178894	0,183985	NaN	NaN	NaN
NaN	NaN	0,645369	2,88379	NaN	2,10203	0,491016	NaN	NaN	NaN
-0,190109	-0,0694256	-0,271404	NaN	NaN	NaN	-0,85961	NaN	-0,879365	-0,545283
NaN	-1,15076	NaN	NaN	NaN	NaN	NaN	0,111578	0,464875	-0,0587225
-1,96246	-1,77313	-1,63114	NaN	NaN	NaN	NaN	NaN	NaN	NaN
0,617987	0,453899	0,721516	-0,198256	NaN	-0,123016	0,409424	0,342113	0,786536	-0,0143557
NaN	NaN	NaN	NaN	NaN	NaN	NaN	-1,0607	-1,11694	-1,4844
NaN	NaN	NaN	-1,11027	NaN	0,188671	-1,52922	NaN	NaN	NaN
NaN	NaN	NaN	-2,11329	NaN	-1,13826	-0,144527	NaN	NaN	NaN
3,71922	2,3539	2,96429	2,80436	4,21891	4,05565	3,50582	2,86191	NaN	2,64566
1,22108	0,358459	0,226677	NaN	-0,0540257	NaN	-0,54681	NaN	NaN	NaN
NaN	NaN	2,25532	1,00609	NaN	2,12927	1,51464	NaN	NaN	NaN
NaN	NaN	NaN	NaN	-0,919844	NaN	NaN	NaN	NaN	NaN
-0,969042	-0,610216	-0,976223	NaN	-1,34676	NaN	NaN	-1,45553	-0,637902	NaN
-0,695009	-1,13033	-0,898184	-1,88424	-1,3751	NaN	-1,04057	-0,272669	NaN	-0,666003
1,84558	1,7718	1,30189	1,57698	1,93528	1,89069	1,71015	1,40393	1,85077	1,79121
-1,16497	-0,918194	-0,976557	0,912512	-0,262495	-0,0471382	1,20539	-0,288576	-0,428933	0,291747
NaN	NaN	NaN	-1,87889	NaN	-1,43973	NaN	NaN	NaN	NaN
0,266874	0,52729	0,449446	-0,022789	-0,166512	0,150637	1,18744	-0,270863	-0,472804	-0,803309
-1,66774	-1,51792	0,738768	0,0745831	-0,494434	0,570229	-0,151312	0,728806	0,567527	0,1457
-3,04167	-3,85496	-0,110741	NaN	NaN	NaN	NaN	0,275654	NaN	NaN

Polito et al. Universal ready-to-use immunotherapeutic approach for the treatment of cancer: expanded and activated polyclonal γδ memory T cells.
Phosphorylation sites

4,19348	4,50138	4,39088	5,12639	5,1153	4,24458	4,63539	4,70421	4,06379	5,57991
0,318764	1,99036	0,724342	2,29523	1,03203	0,0762043	1,22186	0,292431	0,025773	0,616735
NaN	NaN	NaN	NaN	NaN	NaN	NaN	NaN	NaN	NaN
-1,06109	-1,30215	-0,91567	-0,674807	NaN	-1,0326	NaN	NaN	-1,62864	-1,51956
NaN	NaN	NaN	NaN	-0,766485	-1,0326	-0,478182	NaN	NaN	NaN
0,223164	-0,0723553	0,643335	0,345821	1,38324	-1,9597	0,401781	0,915222	0,724835	0,548049
-0,782373	-2,50823	-1,95449	-0,866802	NaN	NaN	-2,08472	-1,97765	-1,22973	NaN
-1,54204	-0,00730896	-0,528572	-2,04501	-1,02828	-1,15367	-1,80917	-0,770424	-0,719266	NaN
-0,0551224	-0,530806	-0,627041	-0,479013	-0,206699	-0,755457	-0,639641	-0,714714	-1,29813	-0,0770769
-1,69798	-2,44511	NaN	-0,116032	-1,88372	NaN	0,811098	-0,763634	-1,83808	-1,32108
-2,34275	NaN	-1,18249	-2,00839	-1,8494	NaN	0,177559	NaN	-1,26291	-0,987239
-1,637	NaN	-1,9299	1,37713	-0,622568	-0,33988	1,0895	-0,85006	-0,930749	-0,361489
-0,574944	-0,481165	-0,0504723	-1,28532	-1,01788	-1,65045	0,749559	-0,631989	-0,48932	-0,436994
-0,273232	-0,463121	-0,761147	NaN	-0,0614834	1,16118	0,732714	-0,285076	0,0438509	-0,0579348
1,21715	1,16465	1,33797	2,31402	1,37931	1,79813	2,39729	1,78731	1,33879	1,23506
NaN	NaN	NaN	NaN	NaN	NaN	NaN	-0,581364	-1,23865	-0,854466
0,827204	1,07417	0,529234	0,464716	1,98014	-0,331636	1,0135	1,02715	0,0855017	1,28931
0,391283	1,71603	0,392113	-0,204725	2,14767	-3,0069	1,12877	1,02691	0,860126	1,25846
NaN	0,0530529	-0,124723	0,296759	-0,166925	0,283875	1,08993	NaN	-0,0399656	NaN
-1,72687	-1,13022	-1,46236	NaN	NaN	NaN	NaN	-0,789785	-0,243451	-1,01795
0,17063	0,414936	-0,306625	0,256947	2,35401	0,0658226	-0,484171	0,199635	-0,391461	1,89777
-0,214092	-2,35102	0,0957394	NaN	-0,52136	NaN	1,1582	0,743198	NaN	0,233346
1,24783	1,2448	0,719845	1,99197	0,16012	2,34468	1,27869	0,477434	-0,13552	0,247817
-1,97847	-1,95324	NaN	NaN	-2,50599	-0,103186	NaN	NaN	NaN	NaN
-0,574078	-0,530064	NaN	-0,670061	-0,32712	-0,657373	-0,771065	NaN	NaN	NaN
NaN	NaN	NaN	NaN	NaN	NaN	NaN	NaN	NaN	NaN
-2,56167	-0,636543	-2,71571	NaN	-5,45669	-1,68025	NaN	NaN	NaN	NaN
-2,61728	-2,72171	-1,7955	NaN	NaN	NaN	NaN	-2,12621	-2,0752	-1,77452
-0,944006	-0,620239	-0,59993	-0,636438	-0,544729	-1,39628	0,790188	-0,816727	-1,20125	-0,0425367
NaN	NaN	NaN	-0,313894	NaN	-0,93285	-0,268093	NaN	NaN	NaN
0,918007	1,94864	2,73112	3,8515	0,450806	4,94205	1,33558	1,69965	2,21129	2,45826
3,28906	2,86684	2,19332	2,20287	2,47662	3,15452	1,67754	2,74336	2,14091	2,93776
-1,56292	-1,3427	NaN	0,214661	-0,752718	-2,50368	0,247183	-1,70614	-1,60213	-1,83751
NaN	-1,39533	NaN	0,79718	-2,23587	1,23985	-0,520254	-2,09433	NaN	NaN

Polito et al. Universal ready-to-use immunotherapeutic approach for the treatment of cancer: expanded and activated polyclonal γδ memory T cells.
Phosphorylation sites

NaN	NaN	NaN	NaN	NaN	NaN	NaN	NaN	NaN	NaN
NaN	NaN	NaN	-2,88356	NaN	-0,953703	-1,56059	NaN	NaN	NaN
1,92819	1,02281	2,56866	2,09904	2,78613	1,17886	1,9338	3,06472	2,81765	2,87936
NaN	-0,555367	1,11646	2,46518	NaN	3,88609	1,99316	NaN	0,980695	NaN
1,10104	2,5526	0,137825	NaN	NaN	NaN	-0,327368	2,44944	2,85236	1,8329
3,29873	2,12802	3,74197	3,65121	3,33706	3,02058	1,89713	3,68477	3,50175	4,77483
-0,923456	-0,846077	-0,492727	1,2569	0,234892	NaN	0,699522	0,0932007	-0,44406	-0,327313
-1,77977	-1,43442	-1,56273	NaN	-1,04449	NaN	NaN	-1,77345	-1,51931	-1,66211
-0,990789	-1,25446	-1,61612	-1,44546	-0,37698	-2,08752	-0,484295	-1,44891	-1,56537	-1,10768
NaN	NaN	NaN	NaN	NaN	NaN	NaN	NaN	NaN	NaN
NaN	NaN	NaN	NaN	NaN	NaN	NaN	NaN	NaN	NaN
NaN	NaN	NaN	-2,22314	NaN	-1,8815	-2,36084	NaN	NaN	NaN
-0,613455	-1,92747	-1,58062	-0,624912	-1,0087	-1,08736	0,042738	NaN	-1,39631	NaN
0,145765	NaN	-0,352194	0,445271	-3,38754	-3,07464	-1,58881	-1,92612	-2,13385	-1,75526
-2,76689	NaN	-1,45113	-0,105846	NaN	0,0238895	-1,28477	NaN	NaN	NaN
-0,268867	NaN	-0,388441	0,561308	-0,470718	1,27662	NaN	NaN	NaN	NaN
NaN	NaN	NaN	-1,89739	NaN	NaN	-2,32278	NaN	NaN	NaN
NaN	NaN	NaN	-1,56671	NaN	-1,16722	-1,43897	NaN	NaN	NaN
NaN	NaN	NaN	NaN	NaN	NaN	NaN	NaN	NaN	NaN
-0,716379	NaN	NaN	-0,813137	-0,00984383	NaN	-1,33603	NaN	NaN	NaN
-1,74239	-1,42887	-1,2722	NaN	-0,689312	NaN	NaN	-0,652504	-1,04789	-1,34764
-0,818092	-1,13343	-1,63081	-0,280556	NaN	0,262156	-0,463531	-1,17572	-0,831025	-1,21669
1,17862	1,46985	0,675543	2,04037	1,44856	2,34664	0,984205	-0,741863	0,792586	1,07336
NaN	NaN	NaN	-2,36888	NaN	-2,66973	-1,5785	NaN	NaN	NaN
NaN	NaN	NaN	-2,93788	-1,71966	NaN	-2,15027	NaN	NaN	NaN
NaN	NaN	NaN	-1,47454	NaN	-1,22706	-1,21453	NaN	NaN	NaN
NaN	-1,16417	0,417887	-1,27296	-2,63618	0,477135	0,113913	0,262011	1,27106	0,672687
-2,76836	-2,31857	-1,59945	NaN	NaN	NaN	-1,37619	-2,29184	NaN	NaN
NaN	-1,82351	-2,1053	-0,539206	-1,92372	NaN	-0,807365	NaN	-2,80783	-1,49445
NaN	NaN	NaN	NaN	NaN	NaN	NaN	NaN	NaN	NaN
NaN	NaN	NaN	0,302553	1,56566	NaN	0,985897	1,26658	1,30872	NaN
0,625675	-0,668425	-0,725662	0,261646	-0,180555	-0,358194	0,544964	-0,0958233	-0,71226	0,325524
NaN	NaN	NaN	NaN	NaN	NaN	NaN	NaN	NaN	NaN
NaN	1,99947	NaN	0,69982	NaN	-0,0229492	1,33638	NaN	NaN	1,31877

Polito et al. Universal ready-to-use immunotherapeutic approach for the treatment of cancer: expanded and activated polyclonal γδ memory T cells.
Phosphorylation sites

-0,991943	0,163887	-0,987736	0,119152	-0,114088	0,928074	0,985447	-0,69528	-0,620267	-0,549846
-0,882347	0,0376244	0,0995827	-0,915173	-0,758671	NaN	-0,100687	NaN	NaN	1,13517
0,354712	1,01574	1,14041	1,67009	0,830055	-1,15308	1,25567	0,594666	1,03945	NaN
NaN	NaN	NaN	NaN	NaN	NaN	NaN	NaN	NaN	NaN
-0,721964	-0,663555	-1,04205	-0,0174389	0,311008	0,465466	0,429657	-0,533356	-0,749564	-0,913802
-0,901566	-0,897036	-0,4037	-0,228821	-1,79936	1,3042	-1,00479	-1,37751	-1,04104	-1,04431
-1,50647	-0,988472	NaN	-0,146025	-0,412113	-1,19826	-0,327648	-1,01532	-1,01935	-0,50907
NaN	NaN	NaN	-1,79925	-2,32723	NaN	-0,874815	-0,83989	-1,01151	-0,669356
0,460072	1,89888	-0,431644	1,66905	NaN	1,37679	2,33266	-0,62705	1,13602	0,266652
2,20621	1,49245	0,962791	0,7679	-0,129122	0,709455	0,403675	1,00855	1,17378	1,07837
NaN	NaN	NaN	NaN	NaN	NaN	NaN	NaN	NaN	NaN
NaN	NaN	NaN	NaN	NaN	NaN	NaN	-2,94594	NaN	-1,88792
NaN	NaN	0,528397	NaN	1,0471	NaN	NaN	NaN	NaN	NaN
0,235596	0,0932846	1,31524	1,23902	1,21707	0,591858	1,34714	0,479885	0,559617	1,2954
-0,692772	-3,25358	-0,995831	NaN	0,270237	NaN	-1,08942	NaN	NaN	NaN
NaN	-0,48563	-1,56242	-1,27221	-1,14522	-3,4469	-1,50759	-2,92988	-2,80811	-0,974376
-2,21445	-1,22897	-1,19161	0,111158	-0,695194	-0,22451	-0,799759	-1,96679	-1,66194	-1,25627
NaN	NaN	NaN	-1,18148	-0,96032	-1,99643	NaN	NaN	NaN	NaN
NaN	NaN	NaN	-1,15685	NaN	NaN	NaN	NaN	NaN	NaN
1,8396	1,5094	-0,680222	2,40527	2,28597	0,585243	0,50141	NaN	NaN	NaN
NaN	NaN	NaN	0,227812	-2,62032	-0,360994	-1,36032	NaN	NaN	NaN
-1,88194	NaN	-0,689524	0,924377	-0,118618	1,76657	1,39228	NaN	NaN	NaN
-0,314548	NaN	NaN	0,768225	NaN	NaN	0,32868	-0,637062	-2,63074	-0,249137
NaN	NaN	NaN	-0,596201	NaN	-1,7813	-1,58277	-0,646519	-0,330283	-0,626672
-1,15853	-2,26001	NaN	-1,3879	-1,7796	-1,98107	-0,590727	NaN	NaN	NaN
0,407967	2,47622	1,97718	3,13311	3,43658	5,40912	3,78191	1,34221	1,11648	1,72798
0,516178	-0,842691	-0,387859	0,298479	NaN	-0,880287	-0,329275	-1,81717	2,17264	-0,257672
-0,647943	-0,933256	0,248486	0,0802631	0,587982	-0,228016	-0,2117	0,780239	0,485192	1,38124
-3,08084	NaN	-2,30482	0,882515	-2,2751	0,476791	-0,15238	NaN	NaN	NaN
NaN	NaN	-1,10758	-3,23117	0,924908	NaN	NaN	NaN	-1,46152	0,337071
NaN	NaN	NaN	-0,246202	-1,57465	-2,34604	-0,106379	NaN	NaN	NaN
-0,331018	0,0728436	-1,10111	0,184536	-1,41435	-0,207945	-0,0963326	-0,751909	-1,74743	-0,237618
-0,818722	-1,29315	-0,559914	-0,579266	0,451824	-0,73439	0,117811	-0,676661	-1,17128	-1,06582
-0,814751	-0,595877	-0,328285	-0,707302	0,91958	-2,39511	0,236126	-0,9883	-0,578078	1,28545

NaN	-1,63101	-0,378212	NaN	NaN	-0,89706	NaN	0,0887756	1,28712	1,04049
NaN	NaN	NaN	-2,98167	-3,69206	-2,01603	-2,5817	NaN	NaN	NaN
NaN	NaN	NaN	NaN	NaN	NaN	NaN	-2,40869	-2,57517	-2,68027
1,01275	-0,23984	0,768684	-1,153	-0,143087	NaN	-0,454256	0,0312843	0,714374	-0,761315
NaN	NaN	NaN	NaN	NaN	NaN	NaN	NaN	NaN	NaN
-0,315937	NaN	-1,41502	-1,00931	-1,43434	NaN	-0,737144	-0,171026	-0,350354	-1,58973
1,65142	2,80186	0,706657	2,8286	2,48776	2,73425	3,55027	0,856924	0,701945	2,4588
-1,10272	-0,787977	-1,02797	NaN	-0,620556	-0,77157	-1,23726	-0,891796	-1,17332	-0,509267
-0,984726	-1,56439	-1,08884	-1,03756	-1,67249	NaN	-0,870314	-2,1076	-1,70409	-1,65863
1,72882	1,55475	1,89573	1,38123	2,05616	1,22496	1,3019	1,96277	2,06153	1,69121
NaN	NaN	NaN	NaN	NaN	NaN	NaN	NaN	NaN	NaN
NaN	NaN	NaN	-0,629198	-1,46918	-1,08337	NaN	NaN	NaN	NaN
NaN	NaN	NaN	-0,964144	NaN	NaN	NaN	NaN	NaN	NaN
-1,9563	-1,44536	-0,964926	-0,763456	-0,433073	-0,548891	-1,26804	-1,06612	-1,24428	-0,903979
-2,1945	-1,29544	NaN	-0,352755	-1,44801	NaN	-1,37973	-1,65059	-2,09741	-1,52701
1,99836	2,00254	1,61587	1,64956	2,66507	-2,28811	2,4398	1,83514	1,85134	1,98459
NaN	NaN	NaN	-2,72021	NaN	NaN	-3,05235	NaN	NaN	NaN
-1,60379	-0,741163	-1,88262	NaN	-1,84971	-1,67072	-1,31642	-1,88179	-0,864203	-1,49419
NaN	NaN	NaN	NaN	NaN	NaN	NaN	NaN	NaN	NaN
-1,14672	0,418554	-0,863407	0,807196	0,278818	1,46616	1,48285	-0,440866	-0,212752	-0,734111
-0,961372	-0,794827	-1,13418	-0,390739	-1,4808	-0,169025	-0,331244	NaN	NaN	NaN
NaN	NaN	NaN	NaN	NaN	NaN	NaN	NaN	NaN	NaN
0,792831	0,450825	1,31532	1,2377	0,836071	1,88187	1,30172	1,34388	1,2057	1,1462
-1,20485	-0,451059	-1,91335	0,23065	NaN	0,203316	0,396715	NaN	NaN	NaN
NaN	NaN	NaN	NaN	NaN	NaN	NaN	NaN	NaN	NaN
NaN	NaN	NaN	NaN	-2,19663	-1,41278	NaN	NaN	NaN	NaN
NaN	NaN	NaN	NaN	NaN	NaN	NaN	NaN	NaN	NaN
NaN	NaN	0,0625534	0,11849	0,00844765	-1,20775	-0,48683	NaN	-0,192618	0,696248
NaN	NaN	NaN	-1,88207	NaN	-0,514555	-1,63152	NaN	NaN	NaN
3,1858	2,91082	3,26814	3,51118	3,08147	3,48568	3,11113	2,72194	2,56946	3,0401
-3,2643	0,260296	NaN	NaN	-0,91807	NaN	NaN	NaN	NaN	-1,25348
-2,24206	-2,23428	NaN	NaN	-1,62506	-2,03319	-1,50714	NaN	NaN	NaN
NaN	NaN	NaN	NaN	0,356911	-0,926321	-1,06738	NaN	NaN	NaN
-2,63617	-1,4264	-1,2415	-1,69142	-1,79877	-1,18387	-0,837078	NaN	NaN	NaN

Polito et al. Universal ready-to-use immunotherapeutic approach for the treatment of cancer: expanded and activated polyclonal γδ memory T cells.
Phosphorylation sites

2,06062	2,22598	1,72748	1,56355	1,93714	1,50278	2,25319	0,822218	0,912274	1,40032
1,95929	0,740341	1,46824	1,17948	1,10522	1,22982	0,935026	-0,00342178	0,43818	-0,314101
NaN	NaN	NaN	-0,994843	NaN	-0,844835	-2,13311	NaN	NaN	NaN
NaN	NaN	NaN	-0,595533	NaN	-2,32727	-1,56237	NaN	NaN	NaN
0,783136	-4,26912	0,538605	1,71165	1,225	0,970032	NaN	1,29491	1,40573	0,633401
NaN	NaN	NaN	-1,75987	-2,11738	-1,07823	-1,25113	NaN	NaN	NaN
-0,991411	-0,403591	-0,578058	-0,813105	-1,11615	-1,73417	-0,995893	-0,45752	-0,829057	-1,15407
3,51053	1,55968	3,57184	2,69284	4,43939	2,98	2,77707	3,15424	2,89128	2,91513
NaN	NaN	NaN	-0,370897	NaN	NaN	NaN	-1,76911	NaN	-1,59791
NaN	NaN	NaN	NaN	NaN	NaN	NaN	NaN	NaN	NaN
-1,33136	-0,562365	-1,25935	-0,080883	-0,794371	-0,358212	-0,216393	-1,18573	-1,39464	-0,0905027
-1,16437	-1,8276	-0,976349	0,62801	NaN	0,677368	0,113087	NaN	0,519547	NaN
NaN	NaN	NaN	-2,01908	-3,31807	NaN	-2,18684	-1,79761	NaN	-2,45164
NaN	NaN	NaN	NaN	NaN	NaN	NaN	NaN	NaN	NaN
-2,25959	-1,6211	-1,34039	-0,422365	-1,64823	-3,77903	-1,65708	-1,61898	-1,54565	-1,32648
NaN	NaN	NaN	NaN	NaN	NaN	NaN	NaN	NaN	NaN
-0,961287	-1,75756	-0,223795	-0,672754	-0,579443	NaN	-0,572401	NaN	NaN	NaN
NaN	NaN	NaN	-1,15181	NaN	-1,52573	-1,12144	NaN	NaN	NaN
NaN	NaN	NaN	NaN	NaN	NaN	NaN	-1,89573	-2,5888	-2,84575
-2,34026	-1,22063	NaN	NaN	-0,669731	NaN	-2,96682	-1,91498	-1,84492	-0,729157
-1,08093	NaN	-2,50011	-1,31168	-1,23533	NaN	-2,48683	NaN	NaN	NaN
NaN	NaN	NaN	-0,605539	NaN	NaN	NaN	NaN	NaN	NaN
NaN	NaN	NaN	NaN	NaN	NaN	-1,42636	NaN	NaN	NaN
NaN	NaN	NaN	-1,37726	-3,2555	-0,0747643	NaN	NaN	NaN	NaN
NaN	NaN	NaN	NaN	NaN	NaN	NaN	NaN	NaN	NaN
-0,677912	NaN	-0,991945	NaN	NaN	NaN	NaN	-0,101086	0,329061	-0,866384
NaN	NaN	NaN	-1,54386	NaN	NaN	-0,290222	NaN	NaN	NaN
-2,21094	0,19552	-1,32194	-1,47546	-0,644312	-3,27176	-0,462299	-1,38974	-2,14008	-1,38401
NaN	NaN	NaN	-2,07646	-1,49467	NaN	-0,916262	NaN	NaN	NaN
NaN	NaN	NaN	NaN	NaN	NaN	NaN	-0,631525	-1,11272	-0,768725
-1,52717	-0,832642	-2,08403	-2,61297	NaN	-1,89793	-1,3542	-2,15125	-1,37201	-1,04072
NaN	NaN	NaN	-1,89077	-2,92729	-1,26112	NaN	NaN	NaN	NaN
NaN	NaN	NaN	NaN	NaN	NaN	NaN	NaN	NaN	NaN
-1,14825	-1,31938	NaN	-1,62386	-0,676579	-2,10953	-1,93494	-1,33423	-2,28268	NaN

Polito et al. Universal ready-to-use immunotherapeutic approach for the treatment of cancer: expanded and activated polyclonal γδ memory T cells.
Phosphorylation sites

NaN	NaN	NaN	-1,42968	-1,84276	-1,4996	-1,50327	-0,563118	-1,77405	-1,05366
-1,62959	NaN	-0,509346	0,95476	0,238945	-0,205456	0,602716	NaN	-1,37568	-0,742496
NaN	NaN	NaN	-1,4672	-2,82498	-3,22261	-1,05517	NaN	NaN	NaN
-0,574045	-1,40561	-0,894041	-0,696001	0,218246	-1,32232	-0,304888	-1,81699	-2,06625	-1,6041
1,44155	2,73352	2,13559	1,81249	2,95356	1,3286	1,98939	2,21787	2,04129	2,31882
NaN	NaN	1,36801	1,77049	NaN	0,875744	2,8624	-3,40137	-3,2879	-0,271428
NaN	NaN	NaN	-0,991512	-1,04831	-0,783916	-0,398108	NaN	NaN	NaN
-0,692808	NaN	-3,00249	-1,13379	-1,64023	-0,781237	-0,496288	-1,96008	-1,86585	-2,23716
NaN	-0,964714	NaN	-0,857613	NaN	-0,173479	-1,99567	NaN	NaN	NaN
-0,158451	-0,111	0,444454	2,27726	0,949085	1,45855	0,493765	0,178961	-0,762832	-0,489221
0,517241	0,21538	-0,931795	2,55309	1,45043	1,6945	1,59931	NaN	NaN	NaN
NaN	NaN	NaN	NaN	NaN	1,59666	NaN	0,57263	0,204364	0,773595
NaN	NaN	NaN	-1,11515	NaN	-0,929672	-1,49405	NaN	NaN	NaN
-2,24238	NaN	-1,66495	-1,52464	-1,35336	-0,833494	-0,969398	-2,31313	-2,68774	-2,23187
-1,45931	NaN	-1,09703	-0,739012	-1,08777	-0,424036	-0,456032	-0,415062	NaN	-1,88131
-1,71597	NaN	-1,43769	-2,54939	NaN	-1,44322	-1,58003	NaN	NaN	NaN
0,221289	0,232321	-0,289503	1,07952	0,479923	0,865126	0,800873	0,0852051	-0,0625982	-0,154298
0,221289	0,232321	-0,289503	1,07952	0,479923	0,865126	0,800873	0,0852051	-0,0625982	-0,154298
-1,86014	-2,05651	-1,36835	NaN	NaN	NaN	NaN	NaN	NaN	NaN
-0,0443802	0,550558	1,40362	1,26396	1,43884	0,771423	2,17324	1,33313	1,30723	NaN
3,55181	3,36697	3,79377	3,38517	3,91953	1,22106	4,06403	4,05974	3,74146	3,59775
0,855728	1,43276	1,19095	1,81552	-1,08006	-0,202419	1,78954	-0,138594	0,929391	0,336125
NaN	0,472095	NaN	-0,43924	-0,635054	-0,595715	NaN	0,428679	-0,33501	-0,404128
5,22617	4,54567	4,32345	4,81343	4,62943	5,34729	4,56566	4,32439	4,07039	3,99821
-0,50185	1,02266	-0,626451	0,222212	0,0279598	-1,52973	0,650963	0,0696754	0,374555	0,356828
NaN	NaN	NaN	-0,571896	0,0578499	-2,3579	0,184969	-0,247608	-0,584552	0,36123
NaN	NaN	NaN	-0,921564	NaN	NaN	-2,15126	NaN	NaN	NaN
0,821278	0,304737	NaN	1,44378	0,951248	0,125261	0,677195	0,728941	0,419087	0,4185
NaN	NaN	NaN	-1,54386	NaN	NaN	-0,290222	NaN	NaN	NaN
NaN	-2,29674	-1,37791	NaN	-1,7606	NaN	NaN	NaN	NaN	-1,15857
-0,884983	-2,49834	-1,11882	-0,465853	-0,0613651	-0,10087	1,24769	0,0352821	-0,206988	0,243384
NaN	NaN	NaN	0,442297	-1,33739	NaN	-0,869823	NaN	NaN	NaN
-1,40286	-0,642244	-1,05917	-0,508757	-1,38235	-2,74197	-0,374622	-1,1602	-0,711051	NaN
-1,36856	-2,37361	-2,92859	-1,0487	-1,68963	-0,69669	-1,14166	0,130386	-0,663173	-0,0170946

Polito et al. Universal ready-to-use immunotherapeutic approach for the treatment of cancer: expanded and activated polyclonal γδ memory T cells.
Phosphorylation sites

2,99794	2,19793	2,45067	2,67511	2,35679	2,62637	2,92273	2,72556	3,17587	3,18666
2,40224	2,68265	1,46041	2,89087	2,99442	2,30629	2,98984	2,55935	1,14207	3,24265
NaN	NaN	-0,414677	4,56266	NaN	-1,68495	4,1428	NaN	NaN	NaN
NaN	NaN	NaN	-1,24211	-1,48406	NaN	-1,23932	-0,462048	-0,710614	NaN
NaN	NaN	NaN	0,144501	NaN	0,419704	-0,952217	NaN	NaN	NaN
-1,33411	NaN	NaN	-2,58524	-2,05379	-1,83313	-2,36256	NaN	NaN	NaN
-1,41209	-1,87259	-1,90048	NaN	-1,17098	NaN	NaN	-1,63671	-1,11356	-1,91009
-1,21119	NaN	-0,678017	NaN	NaN	NaN	NaN	NaN	-1,05218	-0,602916
-1,68744	-1,92723	-2,2548	NaN	NaN	NaN	NaN	NaN	NaN	NaN
1,97652	0,249065	0,79958	-0,869553	1,99349	-0,475693	0,583145	NaN	NaN	NaN
-0,00666809	-1,04257	-0,98044	-1,73886	0,337105	-1,95027	-1,09648	NaN	NaN	NaN
NaN	NaN	NaN	2,8028	NaN	2,93133	1,85327	NaN	NaN	NaN
NaN	NaN	NaN	-1,57261	NaN	NaN	-2,77816	NaN	NaN	NaN
2,86139	3,47996	0,991753	0,184616	3,61694	1,01781	1,80196	3,56767	3,00038	3,09055
NaN	NaN	NaN	1,40724	NaN	1,91109	2,91804	NaN	NaN	NaN
-0,557756	-3,30423	-1,65217	-2,07475	-0,375025	-0,366762	-2,52504	-0,989092	-1,15352	-1,12986
NaN	NaN	-1,54332	NaN	NaN	NaN	NaN	-1,55522	-1,52031	-0,579339
-0,326225	-0,474634	-0,803286	-0,589905	-0,0804787	-0,0196667	0,714308	-1,35481	-1,30871	-1,33885
2,66834	2,00332	2,50832	3,4102	3,06148	3,2697	4,04072	3,70794	3,31539	3,31169
NaN	NaN	NaN	2,8028	NaN	2,93133	1,85327	NaN	NaN	NaN
NaN	NaN	NaN	-1,57261	NaN	NaN	-2,77816	NaN	NaN	NaN
-0,454897	-0,0161839	-0,281572	1,40509	0,458172	0,0942783	1,70707	-0,811769	-1,15489	NaN
4,13014	2,19194	2,19753	1,57223	4,00178	1,68641	1,64168	3,10085	3,51642	2,85926
1,41326	-0,533396	1,31225	0,686632	1,48548	NaN	2,01411	0,445242	0,0582819	1,24681
-0,730419	NaN	-2,01301	-2,90392	-0,796591	-3,00209	NaN	NaN	NaN	NaN
-0,916193	-1,30622	-2,03769	NaN	NaN	NaN	NaN	NaN	NaN	NaN
1,68687	1,88545	0,636261	0,507111	2,14137	NaN	1,80376	-0,195295	-0,603717	0,0605707
-0,126911	-0,517321	-2,32836	-2,62075	0,651264	NaN	-0,569347	NaN	NaN	NaN
-0,420076	-0,688368	-1,15759	-1,99213	-0,271185	-2,9505	-0,991444	NaN	NaN	NaN
-1,46398	-1,59979	NaN	-2,02057	-0,706516	-2,10736	-0,995003	NaN	NaN	NaN
NaN	NaN	NaN	NaN	NaN	NaN	NaN	NaN	NaN	NaN
-1,65292	NaN	NaN	NaN	NaN	NaN	NaN	NaN	NaN	NaN
1,03776	-0,927734	-0,162632	-0,844746	1,17538	-1,42789	-0,0562572	0,272125	-0,665156	-0,392423
2,12692	2,12991	2,42019	2,77006	2,65707	3,02474	2,6531	2,43304	2,27991	2,27444

Polito et al. Universal ready-to-use immunotherapeutic approach for the treatment of cancer: expanded and activated polyclonal γδ memory T cells.
Phosphorylation sites

NaN	NaN	NaN	NaN	NaN	NaN	NaN	-1,42809	-2,05027	-2,16067
-1,57684	NaN	-0,808399	-2,58369	-1,1865	-2,21881	-1,37103	-1,94118	NaN	-2,30903
NaN	NaN	NaN	NaN	NaN	-2,01511	-2,52942	NaN	NaN	NaN
3,13558	3,36381	3,2143	2,30761	2,86212	3,05853	2,95055	2,74034	3,14003	2,98543
3,12493	3,57962	4,05369	3,74406	4,1116	3,78453	3,43216	4,31051	3,90213	3,6625
NaN	NaN	NaN	NaN	NaN	NaN	NaN	NaN	NaN	NaN
-1,39403	-0,944006	-1,82426	NaN	-0,664	-0,190334	-2,84144	-1,26582	-0,813764	-0,902091
NaN	NaN	NaN	NaN	NaN	NaN	NaN	NaN	NaN	NaN
0,972826	0,309898	-1,63291	0,256701	0,17314	0,38727	-1,04268	1,15899	1,07969	0,761327
-1,01553	-1,06232	NaN	-1,75502	2,10251	NaN	-1,25908	0,944548	-0,00742245	0,700631
-2,97113	-3,46953	-1,8129	NaN	-2,2707	NaN	NaN	-1,27538	-1,86682	-1,84203
-1,77938	-2,18889	-1,75523	-0,722328	NaN	-0,591249	-0,71805	NaN	NaN	NaN
-1,77938	-2,18889	-1,75523	-0,722328	NaN	-0,591249	-0,71805	NaN	NaN	NaN
-0,411501	0,45458	NaN	0,271214	1,31597	NaN	0,6548	-0,0474644	0,712497	0,104264
-0,411501	0,45458	NaN	0,271214	1,31597	NaN	0,6548	-0,0474644	0,712497	0,104264
NaN	NaN	NaN	-0,12952	-0,0273552	0,368736	-0,167711	NaN	NaN	NaN
0,337221	0,00312805	-0,151196	-0,12952	-0,0273552	0,368736	-0,167711	-0,0559177	-0,0957899	-0,817515
NaN	NaN	NaN	NaN	NaN	NaN	NaN	-1,31152	-2,30907	-1,7724
NaN	NaN	NaN	NaN	NaN	NaN	NaN	-1,31152	-2,30907	-1,7724
NaN	0,00810814	NaN	NaN	NaN	NaN	NaN	-0,73616	0,195006	-0,779912
NaN	0,00810814	NaN	NaN	NaN	NaN	NaN	-0,73616	0,195006	-0,779912
NaN	NaN	NaN	-2,9104	NaN	NaN	-3,13881	NaN	NaN	NaN
1,38208	0,910858	1,63599	1,70592	1,91167	1,13266	1,89719	0,472713	0,626769	0,544339
1,38208	0,910858	1,63599	1,70592	1,91167	1,13266	1,89719	0,472713	0,626769	0,544339
3,42837	2,96507	2,58122	3,81552	2,83377	4,72573	3,46414	2,29252	2,17415	1,97598
3,42837	2,96507	2,58122	3,81552	2,83377	4,72573	3,46414	2,29252	2,17415	1,97598
2,90876	2,186	1,77161	1,90605	2,3947	1,31643	2,13117	2,03168	2,06682	1,91558
2,90876	2,186	1,77161	1,90605	2,3947	1,31643	2,13117	2,03168	2,06682	1,91558
-0,476723	-0,963408	-1,6316	-0,909014	-2,31119	NaN	-1,66507	0,407511	-1,55786	-0,881017
-0,476723	-0,963408	-1,6316	-0,909014	-2,31119	NaN	-1,66507	NaN	-1,55786	-0,881017
1,55877	1,81596	1,31054	NaN	2,01524	0,501295	1,90108	NaN	1,00133	NaN
0,870941	2,92809	1,61649	2,13414	1,91167	3,37001	1,85723	1,21486	1,29383	1,39963
0,870941	2,92809	1,61649	2,13414	1,91167	3,37001	1,85723	1,21486	1,29383	1,39963
NaN	-0,144667	NaN	-1,09664	-1,53316	0,300026	-0,486736	NaN	NaN	NaN

NaN	-0,144667	NaN	-1,09664	-1,53316	0,300026	-0,486736	NaN	NaN	NaN
-2,96227	1,66453	NaN	-0,842865	NaN	-1,67535	0,519949	NaN	NaN	NaN
-2,96227	1,66453	NaN	-0,842865	NaN	-1,67535	0,519949	NaN	NaN	NaN
NaN	NaN	NaN	-0,0297527	-0,148403	-0,428577	1,28787	NaN	NaN	NaN
NaN	NaN	NaN	-0,801525	-0,531311	NaN	-0,594131	NaN	NaN	NaN
-0,574677	NaN	1,51089	0,887789	NaN	NaN	1,12662	NaN	NaN	NaN
-0,574677	NaN	1,51089	0,887789	NaN	NaN	1,12662	NaN	NaN	NaN
NaN	NaN	NaN	-1,15104	NaN	-1,98434	0,341188	-0,444426	-0,454337	NaN
NaN	NaN	NaN	0,767685	NaN	0,714941	-0,323532	NaN	NaN	NaN
1,6403	2,6412	1,48818	0,68343	0,228718	0,904388	0,731503	1,13125	2,83313	1,35806
NaN	NaN	NaN	NaN	NaN	NaN	NaN	-2,2572	-0,680753	-1,27781
1,6403	2,6412	-0,781807	0,479425	0,432404	0,904388	2,07096	1,13125	2,83313	1,35806
NaN	NaN	-1,08911	NaN	NaN	NaN	NaN	-2,93525	-2,53908	-2,07154
NaN	NaN	-1,08911	NaN	NaN	NaN	NaN	-2,93525	-2,53908	-2,07154
-0,496141	-1,91884	1,72049	NaN	NaN	NaN	NaN	NaN	NaN	NaN
1,71181	NaN	NaN	2,64947	1,19452	2,90391	1,43555	2,12326	2,0042	2,18633
1,71181	-1,91884	1,72049	2,64947	1,19452	2,90391	1,43555	0,245926	-1,02372	2,18633
1,84116	-0,482578	1,69479	1,7275	1,00224	0,976137	1,42702	1,62487	1,26834	0,918317
1,84116	-0,482578	1,69479	1,7275	1,00224	NaN	1,42702	1,62487	1,26834	0,918317
-2,5164	NaN	-1,85967	-0,25585	-1,4388	-2,0804	-1,98944	NaN	NaN	NaN
1,41861	0,509892	-0,655104	1,07149	0,474184	1,08781	0,506248	-0,400009	-0,618974	-0,167924
1,41861	0,509892	-0,655104	1,07149	0,474184	1,08781	0,506248	-0,400009	-0,618974	-0,167924
1,35398	0,389832	0,587723	1,58858	NaN	2,35826	1,77218	-0,0773106	0,626239	-1,06212
1,35398	0,389832	0,587723	1,58858	NaN	2,35826	1,77218	-0,0773106	0,626239	-1,06212
-1,99368	-1,10467	-1,13065	-1,75254	-1,76195	-1,38438	-0,35454	NaN	NaN	NaN
-1,00465	NaN	NaN	-0,0555019	NaN	-0,0871849	NaN	NaN	NaN	NaN
0,637646	0,535805	0,274479	1,10794	0,921234	-0,609947	1,27639	0,509291	0,096591	0,112014
0,637646	0,535805	0,274479	1,10794	0,921234	-0,609947	1,27639	0,509291	0,096591	0,112014
NaN	NaN	NaN	NaN	NaN	NaN	NaN	NaN	NaN	NaN
NaN	NaN	NaN	NaN	NaN	NaN	NaN	NaN	NaN	NaN
NaN	-2,71168	-1,25647	-0,776779	-1,16409	-1,42254	-1,64935	-1,25482	-2,13044	-1,31079
NaN	-2,71168	-1,25647	-0,776779	-1,16409	-1,42254	-1,64935	-1,25482	-2,13044	-1,31079
-1,45771	-2,61986	-1,36628	-2,077	NaN	-0,894741	-1,07721	NaN	NaN	NaN
-1,45771	-2,61986	-1,36628	-2,077	-1,48191	-0,894741	-1,07721	NaN	NaN	NaN

1,44098	0,909946	0,186228	NaN	0,421745	-0,365742	NaN	NaN	NaN	NaN
1,44098	0,909946	0,186228	NaN	0,421745	-0,365742	NaN	NaN	NaN	NaN
-0,633432	-0,671001	NaN	-1,23664	-0,697496	-1,70524	-1,21478	-1,03486	-1,31875	NaN
-0,633432	-0,671001	NaN	-1,23664	-0,697496	-1,70524	-1,21478	-1,03486	-1,31875	NaN
NaN	NaN	NaN	-0,513762	-1,61972	-1,41543	-0,785089	-2,02366	-1,66877	-0,320907
0,21682	2,69348	-2,36469	1,93085	0,782978	2,37771	2,16345	0,561045	1,5642	1,37736
0,21682	2,69348	-2,36469	1,93085	0,782978	2,37771	2,16345	0,561045	1,5642	1,37736
NaN	-2,34959	-1,0237	0,318325	NaN	1,2225	0,967274	NaN	NaN	NaN
NaN	-2,34959	-1,0237	0,318325	NaN	1,2225	0,967274	NaN	NaN	NaN
NaN	NaN	NaN	NaN	NaN	NaN	NaN	NaN	NaN	NaN
NaN	NaN	NaN	NaN	NaN	NaN	NaN	NaN	NaN	NaN
NaN	NaN	NaN	NaN	NaN	NaN	NaN	NaN	NaN	NaN
1,24839	0,628908	NaN	1,49848	1,44626	2,72996	1,51878	NaN	0,52583	NaN
1,24839	0,628908	NaN	1,49848	1,44626	2,72996	1,51878	NaN	0,52583	NaN
NaN	NaN	NaN	NaN	NaN	NaN	NaN	NaN	NaN	NaN
NaN	NaN	NaN	NaN	NaN	NaN	NaN	NaN	NaN	NaN
NaN	NaN	NaN	NaN	NaN	NaN	NaN	NaN	NaN	NaN
NaN	NaN	NaN	NaN	NaN	NaN	NaN	NaN	NaN	NaN
0,0491276	-0,312828	0,739275	0,75238	1,00664	0,538155	-0,305716	0,494425	-0,679339	-1,14465
0,0491276	-0,312828	0,739275	0,75238	1,00664	0,538155	-0,305716	0,494425	-0,679339	-1,14465
0,734329	-0,0116539	-0,63232	-2,21715	0,380777	-1,66232	-1,56474	-0,259682	-0,365916	-1,00934
0,734329	-0,0116539	-0,63232	-2,21715	0,380777	-1,66232	-1,56474	-0,259682	-0,365916	-1,00934
NaN	NaN	NaN	NaN	NaN	NaN	NaN	NaN	NaN	NaN
NaN	NaN	NaN	-0,150364	-0,518589	0,216784	0,501646	NaN	NaN	NaN
NaN	NaN	NaN	NaN	NaN	NaN	NaN	NaN	NaN	NaN
NaN	NaN	NaN	NaN	NaN	NaN	NaN	NaN	NaN	NaN
-1,5797	NaN	0,107002	0,96666	-1,44723	1,54655	-0,292273	-0,854292	NaN	NaN
1,97663	0,860439	-1,22318	1,61703	1,66924	1,9632	1,19038	1,68924	1,54718	1,47825
-0,113955	NaN	-0,975014	-0,998291	0,480963	-0,429514	-0,120764	-0,992958	-1,1181	-0,172469
-0,113955	NaN	-0,975014	-0,998291	0,480963	-0,429514	-0,120764	-0,992958	-1,1181	-0,172469
-1,76278	NaN	NaN	-1,74513	-0,255758	-0,922363	-1,99612	-1,84145	-1,9799	NaN
NaN	NaN	NaN	NaN	NaN	NaN	NaN	NaN	NaN	NaN
NaN	NaN	NaN	NaN	NaN	NaN	NaN	NaN	NaN	NaN
NaN	NaN	NaN	-0,708263	NaN	-0,662258	-1,39628	NaN	NaN	NaN

Polito et al. Universal ready-to-use immunotherapeutic approach for the treatment of cancer: expanded and activated polyclonal γδ memory T cells.
Phosphorylation sites

NaN	NaN	NaN	-0,708263	NaN	-0,662258	-1,39628	NaN	NaN	NaN
2,74685	2,7507	1,92634	2,14091	2,8771	0,246584	2,71271	2,03873	1,87815	1,77869
2,74685	2,7507	1,92634	2,14091	2,8771	0,246584	2,71271	2,03873	1,87815	1,77869
0,976234	1,09875	1,13105	2,47919	1,03716	NaN	2,36017	NaN	NaN	NaN
0,976234	1,09875	1,13105	2,47919	1,03716	-0,701845	2,36017	-0,320614	NaN	-1,27553
1,5758	1,91953	0,710678	1,59833	1,40413	-1,32793	1,8319	0,496901	1,03845	1,10326
1,5758	1,91953	0,710678	1,59833	1,40413	-1,32793	1,8319	0,496901	1,03845	1,10326
2,06879	0,837433	2,50186	3,532	1,60872	3,25509	2,66724	1,92419	1,92189	1,26325
0,183893	1,44174	0,735079	0,407934	1,02058	NaN	1,30823	0,684532	1,01571	0,405702
0,183893	1,44174	0,735079	0,407934	1,02058	NaN	1,30823	0,684532	1,01571	0,405702
-0,872385	0,13538	-1,23775	-2,09864	-0,966917	-1,47811	-0,771179	-1,15225	-1,00207	-1,4507
-0,872385	0,13538	NaN	-2,09864	-0,966917	-1,47811	-0,771179	-1,15225	-1,00207	-1,4507
NaN	NaN	NaN	NaN	NaN	NaN	NaN	NaN	NaN	NaN
NaN	NaN	NaN	NaN	NaN	NaN	NaN	NaN	NaN	NaN
NaN	NaN	NaN	0,296249	-1,80213	0,090456	1,09715	NaN	NaN	NaN
NaN	NaN	NaN	0,296249	-1,80213	0,090456	1,09715	NaN	NaN	NaN
0,817484	-0,215034	NaN	-1,74427	-0,221043	-0,358519	NaN	-0,950163	NaN	-1,21968
0,817484	-0,215034	NaN	-1,74427	-0,221043	-0,358519	NaN	-0,950163	NaN	-1,21968
1,57452	1,09628	0,540674	-0,00294304	0,402794	NaN	-0,00449753	0,799326	1,2808	NaN
0,884546	1,52264	0,728186	2,3595	1,83771	-2,35257	2,11098	0,860226	0,304559	0,678864
0,884546	1,52264	0,728186	2,3595	1,83771	-2,35257	2,11098	0,860226	0,304559	0,678864
0,48967	-0,281172	-0,471647	0,502707	0,644405	-0,0327225	0,890514	NaN	NaN	NaN
0,48967	-0,281172	-0,471647	0,502707	0,644405	-0,0327225	0,890514	NaN	NaN	NaN
NaN	NaN	NaN	NaN	NaN	NaN	NaN	NaN	NaN	NaN
NaN	NaN	NaN	NaN	NaN	NaN	NaN	NaN	NaN	NaN
NaN	NaN	NaN	-1,25816	-2,57245	-0,981497	-0,179457	NaN	NaN	NaN
-0,728531	-0,996468	-1,515	-0,837534	-1,04614	-2,0241	-1,07645	-1,25233	-1,00895	-1,93972
-0,728531	-0,996468	NaN	NaN	-1,04614	-2,0241	-1,07645	NaN	-1,00895	NaN
NaN	NaN	NaN	2,56526	NaN	NaN	NaN	0,719444	-0,733142	0,266042
-0,9135	NaN	NaN	2,56526	NaN	NaN	NaN	0,719444	0,209126	0,266042
NaN	NaN	NaN	0,299046	-1,06547	-0,300354	-0,220709	-1,74558	NaN	-0,421788
NaN	NaN	NaN	0,299046	-1,06547	-0,300354	-0,220709	-1,74558	NaN	-0,421788
NaN	NaN	NaN	0,96666	-0,831635	-0,243254	NaN	NaN	NaN	NaN
NaN	NaN	NaN	0,96666	-0,831635	-0,243254	NaN	NaN	NaN	NaN

Polito et al. Universal ready-to-use immunotherapeutic approach for the treatment of cancer: expanded and activated polyclonal γδ memory T cells.
Phosphorylation sites

1,67859	2,01855	1,91306	1,127	1,82653	-1,2237	1,00016	1,7868	1,8506	1,58652
1,67859	2,01855	1,91306	1,127	1,82653	-1,2237	1,00016	1,7868	1,8506	1,58652
NaN	-2,98304	-1,71008	-1,98775	-1,49556	NaN	-1,95351	-1,51296	-1,91938	-2,01786
NaN	NaN	NaN	NaN	NaN	NaN	NaN	NaN	-0,798059	NaN
NaN	NaN	NaN	-0,216316	NaN	0,769104	0,298363	NaN	NaN	NaN
NaN	NaN	NaN	-0,216316	NaN	0,769104	0,298363	NaN	NaN	NaN
2,80502	2,84604	3,21256	3,95018	4,26341	1,6922	3,03945	2,55397	3,04328	3,41941
2,93299	1,50858	3,22206	3,97634	2,91894	3,97412	3,50766	2,87247	3,06863	2,37458
NaN	NaN	NaN	NaN	NaN	NaN	NaN	NaN	NaN	NaN
-2,43835	NaN	-0,516363	-0,10565	NaN	-2,07398	-1,71262	NaN	NaN	NaN
-0,380951	0,241838	-0,504648	0,769842	0,667448	2,20714	1,32679	-2,07813	-1,28588	-0,786151
-0,380951	0,241838	-0,504648	0,769842	0,667448	2,20714	1,32679	-2,07813	-1,28588	-0,786151
NaN	NaN	NaN	NaN	NaN	NaN	NaN	NaN	NaN	NaN
NaN	NaN	NaN	NaN	NaN	NaN	NaN	NaN	NaN	NaN
NaN	NaN	NaN	0,39583	-0,188959	NaN	0,356577	NaN	NaN	NaN
NaN	NaN	NaN	0,39583	-0,188959	NaN	0,356577	NaN	NaN	NaN
-1,58238	-1,21509	NaN	-0,388378	NaN	0,244865	-0,313583	NaN	NaN	NaN
-1,58238	-1,21509	NaN	-0,388378	NaN	0,244865	-0,313583	NaN	NaN	NaN
NaN	NaN	NaN	-1,1433	-1,93781	-1,03749	-0,873341	NaN	NaN	NaN
NaN	NaN	NaN	-1,1433	-1,93781	-1,03749	-0,873341	NaN	NaN	NaN
NaN	NaN	NaN	0,627415	-1,39261	-0,651678	-1,74919	NaN	NaN	NaN
NaN	NaN	NaN	0,632523	-0,236904	-0,802044	-1,25753	NaN	NaN	NaN
NaN	NaN	NaN	-2,4272	-2,45394	-1,31115	-2,48696	NaN	NaN	NaN
NaN	NaN	NaN	-2,4272	-2,45394	-1,31115	-2,48696	NaN	NaN	NaN
NaN	NaN	NaN	NaN	NaN	NaN	NaN	2,86875	1,87015	3,02775
NaN	NaN	NaN	NaN	NaN	NaN	NaN	2,86875	1,87015	3,02775
NaN	NaN	NaN	-0,192322	NaN	NaN	-0,505648	-0,794968	-0,943393	-0,810174
0,020874	-0,00519753	-1,15693	-0,474945	-1,10046	-1,15834	0,133379	NaN	NaN	NaN
0,020874	-0,00519753	-1,15693	-0,474945	-1,10046	-1,15834	0,133379	NaN	NaN	NaN
-1,23711	-1,60301	-2,63581	-1,00661	NaN	NaN	-1,35483	-2,31263	NaN	-1,93076
-1,23711	NaN	-2,63581	NaN	-1,94491	NaN	-1,35483	-2,31263	-1,8808	-1,93076
2,02996	3,73207	2,00723	1,96548	1,39799	2,52924	2,69849	1,34005	2,47081	1,63852
-1,54744	NaN	-1,97119	NaN	NaN	NaN	NaN	NaN	NaN	NaN
-1,54744	NaN	-1,97119	NaN	NaN	NaN	NaN	NaN	NaN	NaN

Polito et al. Universal ready-to-use immunotherapeutic approach for the treatment of cancer: expanded and activated polyclonal γδ memory T cells.
Phosphorylation sites

2,87308	2,91198	3,24042	3,74571	2,42036	4,20653	4,30183	2,81053	2,64983	2,29671
1,51251	2,9874	0,883091	NaN	0,351372	1,28378	1,9773	NaN	NaN	NaN
1,51251	2,9874	0,883091	NaN	NaN	1,28378	1,9773	NaN	NaN	NaN
4,25041	4,68772	3,66954	4,11816	4,34824	3,24868	4,45243	3,57346	3,75159	3,52643
4,25041	4,68772	3,66954	4,11816	4,34824	3,24868	4,45243	3,57346	3,75159	3,52643
3,91919	3,89256	4,15889	3,74008	-1,34402	4,02101	3,80316	4,33209	4,35608	4,06404
3,91919	3,89256	4,15889	3,74008	-1,34402	4,02101	3,80316	4,33209	4,35608	4,06404
4,20272	4,94088	4,04909	3,91381	4,08615	4,22804	4,12524	3,92134	4,45664	3,90693
-0,871037	-0,700823	-1,37208	NaN	NaN	NaN	NaN	-1,59535	-1,66181	-1,60194
4,20272	4,94088	4,04909	3,91381	4,08615	4,22804	4,12524	3,92134	4,45664	3,90693
NaN	0,970268	-2,03543	-0,341135	0,207699	-1,01838	0,293119	-2,79714	-2,03431	-0,416419
NaN	0,970268	-2,03543	-0,341135	0,207699	-1,01838	0,293119	-2,79714	-2,03431	-0,416419
-1,01969	0,474787	-1,89164	-1,44411	-1,45875	NaN	NaN	NaN	NaN	NaN
-1,01969	0,474787	-1,89164	-1,44411	-1,45875	NaN	NaN	NaN	NaN	NaN
0,668869	1,43022	-0,156218	0,717144	NaN	NaN	1,67026	NaN	0,818868	-0,45522
0,668869	1,43022	-0,156218	0,717144	NaN	NaN	1,67026	NaN	0,818868	-0,45522
NaN	-1,17859	NaN	-1,11882	-1,40701	-0,460955	-0,236025	-1,54586	-1,00491	-2,11433
NaN	-1,17859	NaN	-1,11882	-1,40701	-0,460955	-0,236025	-1,54586	-1,00491	-2,11433
NaN	1,0886	-1,51064	-0,281559	0,167049	-1,08051	0,0143204	-0,758686	-1,20971	-0,998841
NaN	1,0886	-1,51064	-0,281559	0,167049	-1,08051	0,0143204	-0,758686	-1,20971	-0,998841
NaN	NaN	NaN	3,25994	3,6557	3,55165	3,99296	NaN	NaN	NaN
3,89741	4,52694	3,26276	3,69267	3,68365	2,41187	4,0198	3,57553	3,99522	3,32689
3,89741	4,52694	3,26276	3,69267	3,68365	2,41187	4,0198	3,57553	3,99522	3,32689
NaN	NaN	NaN	NaN	1,63028	1,36423	1,32679	NaN	1,14384	0,868279
NaN	NaN	NaN	NaN	1,63028	1,36423	1,32679	NaN	1,14384	0,868279
NaN	NaN	NaN	-1,81243	NaN	-2,38763	-2,59879	NaN	NaN	NaN
NaN	NaN	NaN	-1,81243	NaN	-2,38763	-2,59879	NaN	NaN	NaN
2,55828	2,89478	1,6396	1,24104	1,70744	-1,1321	2,30272	0,850574	1,7841	2,26555
0,405996	-0,510069	0,195309	1,46178	0,340319	2,37156	-0,23329	-0,923803	-0,208882	-0,534274
NaN	-0,510069	0,195309	0,144186	0,340319	1,04733	NaN	NaN	-0,208882	NaN
-0,73513	-0,356499	0,530909	1,46178	-0,150232	2,37156	0,977184	-0,634813	-0,314845	0,0778933
NaN	NaN	NaN	-0,487261	NaN	NaN	-1,53744	NaN	NaN	NaN
NaN	NaN	NaN	-0,487261	NaN	NaN	-1,53744	NaN	NaN	NaN
NaN	-2,1718	NaN	NaN	NaN	-1,50215	NaN	-1,74207	-2,32449	-2,33925

Polito et al. Universal ready-to-use immunotherapeutic approach for the treatment of cancer: expanded and activated polyclonal γδ memory T cells.
Phosphorylation sites

NaN	-2,1718	NaN	NaN	NaN	NaN	NaN	-1,74207	-2,32449	-2,33925
NaN	-0,69418	0,341537	1,35703	0,301489	2,04826	2,63626	0,901535	0,668271	1,06746
NaN	-0,69418	0,341537	1,35703	0,301489	2,04826	2,63626	0,901535	0,668271	1,06746
3,32581	3,28355	3,19751	3,93307	3,02624	3,34039	3,79053	3,26578	3,33117	4,81607
1,95082	1,71622	1,00723	1,13982	1,08365	-1,35824	1,6391	0,542601	1,32428	1,18688
NaN	NaN	NaN	NaN	NaN	NaN	NaN	NaN	NaN	NaN
NaN	NaN	NaN	0,0483723	-1,23266	NaN	-2,39006	NaN	NaN	NaN
NaN	NaN	NaN	0,0483723	-1,23266	NaN	-2,39006	NaN	NaN	NaN
0,511066	-0,582495	-0,860903	0,614506	NaN	-1,36449	0,219044	-1,86189	0,437999	-0,36728
NaN	NaN	NaN	NaN	NaN	NaN	NaN	-1,86189	0,437999	-0,36728
NaN	NaN	NaN	0,614506	NaN	NaN	0,219044	NaN	NaN	NaN
2,0501	1,24657	0,888481	1,02048	0,826227	1,30007	0,556253	0,381935	0,00503635	0,482791
2,0501	1,24657	0,888481	1,02048	0,826227	1,30007	0,556253	0,381935	0,00503635	0,482791
-1,03988	-0,58147	NaN	-0,154982	NaN	-0,20435	-0,644489	NaN	NaN	NaN
NaN	NaN	NaN	NaN	NaN	NaN	NaN	NaN	NaN	NaN
NaN	NaN	NaN	-2,07995	NaN	-0,497486	-1,12072	NaN	NaN	NaN
NaN	NaN	NaN	-2,07995	NaN	-0,497486	-1,12072	NaN	NaN	NaN
NaN	NaN	NaN	NaN	NaN	NaN	NaN	-1,88972	-2,33833	-2,6445
NaN	NaN	NaN	NaN	NaN	NaN	NaN	-1,88972	-2,33833	-2,6445
-1,68615	NaN	-0,267803	NaN	NaN	NaN	NaN	-0,753632	-0,736215	-1,43386
1,57655	1,46116	2,38689	3,65714	2,66731	NaN	3,41378	2,23328	2,5047	2,31296
1,57655	1,46116	2,38689	3,65714	2,66731	NaN	3,41378	2,23328	2,5047	2,31296
0,446836	0,160267	-0,63706	-0,397842	0,731489	-0,766689	1,19156	NaN	NaN	NaN
-0,126863	0,204201	-0,237398	-0,0517426	0,44912	NaN	-0,117414	0,221041	0,113391	-0,160466
-0,126863	0,204201	-0,237398	-0,0517426	0,44912	NaN	-0,117414	0,221041	0,113391	-0,160466
-0,405428	-0,954172	NaN	NaN	0,394512	NaN	NaN	-0,96752	-0,186934	-0,259181
-0,405428	-0,954172	NaN	NaN	0,394512	NaN	NaN	-0,96752	-0,186934	-0,259181
-1,68349	-0,78344	-2,05124	NaN	NaN	NaN	NaN	NaN	NaN	NaN
1,0715	0,239466	0,62421	1,36932	0,638828	1,40507	0,290728	NaN	NaN	NaN
1,0715	0,239466	0,62421	1,36932	0,638828	1,40507	0,290728	NaN	NaN	NaN
-0,0321178	NaN	-2,32581	0,594923	-0,670954	0,921698	0,925175	NaN	NaN	NaN
-1,02445	-2,28136	-1,27051	-0,649759	-1,22965	NaN	-0,921839	-0,844271	-0,705991	NaN
-1,02445	-2,28136	-1,27051	-0,649759	-1,22965	NaN	-0,921839	-0,844271	-0,705991	NaN
1,22557	1,37074	0,905781	0,614025	1,2376	1,49684	1,34292	0,577602	0,269412	0,801776

Polito et al. Universal ready-to-use immunotherapeutic approach for the treatment of cancer: expanded and activated polyclonal γδ memory T cells.
Phosphorylation sites

3,63427	0,954599	2,31885	2,71955	2,3734	-0,210299	1,42194	1,63535	1,56261	0,737987
3,63427	0,954599	2,31885	2,71955	2,3734	-0,210299	1,42194	1,63535	1,56261	0,737987
0,521786	0,856228	NaN	-0,138113	0,899405	-1,28134	0,00225639	NaN	NaN	NaN
1,36935	NaN	NaN	0,61894	0,204	2,1952	0,597734	0,29241	1,25035	0,0323496
3,20388	3,28198	2,05178	2,42409	1,50514	4,18231	2,64972	1,63855	3,03872	1,88702
3,20388	3,28198	2,05178	2,42409	1,50514	4,18231	2,64972	1,63855	3,03872	1,88702
0,521786	0,856228	NaN	-0,138113	0,899405	-1,28134	0,00225639	NaN	NaN	NaN
1,01253	-0,81801	-0,688704	0,434837	-0,0790367	-2,27588	0,584055	0,504305	0,186328	-0,057004
1,01253	-0,81801	-0,688704	0,434837	-0,0790367	-2,27588	0,584055	0,504305	0,186328	-0,057004
1,56021	0,939569	0,559668	NaN	1,033	NaN	-0,372887	1,06508	1,05473	0,929204
-1,32335	-1,01037	-0,476889	NaN	-1,77133	NaN	-3,75063	-0,841854	-1,38037	-1,90286
-1,32335	-1,01037	-0,476889	NaN	-1,77133	NaN	-3,75063	-0,841854	-1,38037	-1,90286
-0,988352	-1,19934	-1,13228	1,25775	NaN	1,266	-0,882004	NaN	NaN	NaN
2,3073	NaN	0,832329	0,987391	0,855656	2,18319	1,25987	1,04769	0,234782	-0,822703
-0,279449	-1,98526	-0,122622	2,14315	-0,290876	1,48246	2,62076	-1,77524	-0,436437	-0,630149
-0,279449	-1,98526	-0,122622	2,14315	-0,290876	1,48246	2,62076	-1,77524	-0,436437	-0,630149
NaN	NaN	NaN	1,01939	-2,27758	1,3539	-0,388042	NaN	NaN	NaN
NaN	NaN	NaN	1,94953	-1,84001	NaN	0,295052	NaN	NaN	NaN
NaN	NaN	NaN	1,94953	-1,84001	NaN	0,295052	NaN	NaN	NaN
NaN	NaN	-2,45909	1,28244	-0,884666	1,16091	-0,782396	NaN	-1,96309	NaN
NaN	NaN	-2,45909	1,28244	-0,884666	1,16091	-0,782396	NaN	-1,96309	NaN
NaN	NaN	NaN	1,1076	-1,19201	NaN	0,195507	NaN	NaN	NaN
NaN	NaN	NaN	0,0932274	-2,43391	0,685066	-0,503492	NaN	NaN	NaN
NaN	NaN	NaN	0,0932274	-2,43391	0,685066	-0,503492	NaN	NaN	NaN
NaN	NaN	NaN	0,58968	NaN	2,92706	-3,0116	NaN	NaN	NaN
NaN	NaN	NaN	0,58968	NaN	2,92706	-3,0116	NaN	NaN	NaN
NaN	NaN	NaN	-1,34594	NaN	-2,36301	-2,71337	NaN	NaN	NaN
NaN	NaN	NaN	-1,34594	NaN	-2,36301	-2,71337	NaN	NaN	NaN
-3,85164	1,43345	-0,088686	0,785923	1,58966	-0,450911	1,83504	-0,0790501	-0,907134	0,67283
-3,85164	1,43345	-0,088686	0,785923	1,58966	-0,450911	1,83504	-0,0790501	-0,907134	0,67283
-1,76013	0,360039	-2,64745	NaN	-2,42318	NaN	2,57859	0,866394	NaN	0,80046
-1,76013	0,360039	-2,64745	NaN	-2,42318	NaN	2,57859	0,866394	NaN	0,80046
NaN	NaN	NaN	NaN	NaN	NaN	NaN	NaN	NaN	NaN
NaN	NaN	NaN	NaN	NaN	NaN	NaN	NaN	NaN	NaN

NaN	-0,0589695	-0,315752	-0,101068	NaN	-0,692402	0,277403	NaN	NaN	NaN
-0,254393	-0,0589695	-0,315752	-0,101068	NaN	-0,692402	0,277403	NaN	NaN	NaN
0,725042	1,64618	0,384338	-0,16099	0,47868	-0,00973892	0,59466	0,71179	0,586499	-0,186477
1,05816	1,64618	0,799458	1,46558	1,10958	-0,00973892	1,52896	0,795019	0,586499	-0,186477
NaN	NaN	1,26629	1,41705	2,92936	NaN	2,57289	0,52882	0,823741	2,63158
0,0908451	2,6973	1,26629	1,41705	2,92936	NaN	2,57289	0,52882	0,823741	2,63158
-1,38993	-0,0931416	-1,42102	NaN	NaN	NaN	NaN	NaN	NaN	NaN
-1,38993	-0,0931416	-1,42102	NaN	NaN	NaN	NaN	NaN	NaN	NaN
-0,162542	-0,711803	-0,88595	0,401964	NaN	NaN	NaN	NaN	NaN	NaN
-0,162542	-0,711803	-0,88595	0,401964	NaN	0,904516	NaN	NaN	NaN	NaN
NaN	NaN	NaN	-0,660236	-2,21962	-0,837944	-0,834885	NaN	NaN	NaN
-0,404808	-0,254314	-1,01404	-0,858446	-0,556974	-1,07214	NaN	-1,02108	-0,0608854	-0,167794
-0,404808	-0,254314	-1,01404	-0,858446	-0,556974	-1,07214	NaN	-1,02108	-0,0608854	-0,167794
NaN	NaN	NaN	-1,13775	-1,099	-1,86255	-3,30308	NaN	NaN	NaN
1,03569	2,11639	0,567125	1,7341	1,45832	1,81961	2,0873	0,0141468	0,59847	0,507102
1,03569	2,11639	0,567125	1,7341	1,45832	1,81961	2,0873	0,0141468	0,59847	0,507102
0,374422	0,77944	0,210171	NaN	NaN	NaN	NaN	NaN	NaN	NaN
0,374422	0,77944	0,210171	1,10981	0,661619	NaN	NaN	0,280283	-0,00983715	-0,335689
NaN	0,77944	NaN	1,10981	0,661619	NaN	NaN	0,280283	-0,00983715	-0,335689
-0,574211	NaN	-0,263165	0,573713	NaN	-0,0824833	-0,931908	NaN	NaN	0,985921
-0,574211	NaN	-0,263165	0,573713	NaN	-0,0824833	-0,931908	NaN	NaN	0,985921
NaN	NaN	NaN	-2,70179	-1,83432	NaN	-1,49273	NaN	NaN	NaN
NaN	NaN	NaN	-2,70179	-1,83432	NaN	-1,49273	NaN	NaN	NaN
NaN	NaN	NaN	-1,24172	NaN	NaN	-1,53459	-1,96864	-0,738025	-0,895801
NaN	NaN	NaN	-1,16173	NaN	-1,20482	-1,18944	NaN	NaN	NaN
NaN	NaN	NaN	-1,16173	NaN	-1,20482	-1,18944	NaN	NaN	NaN
NaN	NaN	NaN	NaN	NaN	NaN	NaN	NaN	NaN	NaN
NaN	NaN	NaN	NaN	NaN	NaN	NaN	NaN	NaN	NaN
NaN	NaN	NaN	NaN	NaN	NaN	NaN	NaN	NaN	NaN
1,60059	3,10037	0,630249	1,1005	1,37691	NaN	NaN	0,983671	NaN	1,55633
NaN	NaN	NaN	NaN	NaN	NaN	NaN	2,02446	-1,7278	-1,5294
NaN	NaN	NaN	-2,1852	-1,49524	NaN	-1,61917	NaN	NaN	NaN
NaN	NaN	NaN	-2,1852	-1,49524	NaN	-1,61917	NaN	NaN	NaN
NaN	NaN	NaN	-1,62789	-0,79847	-2,35643	-1,96997	NaN	NaN	NaN

Polito et al. Universal ready-to-use immunotherapeutic approach for the treatment of cancer: expanded and activated polyclonal γδ memory T cells.
Phosphorylation sites

NaN	NaN	NaN	-1,62789	-0,79847	-2,35643	-1,96997	NaN	NaN	NaN
NaN	-1,14224	NaN	-1,3525	-1,3695	-1,26023	-0,741583	NaN	NaN	NaN
NaN	-1,14224	NaN	-1,3525	-1,3695	-1,26023	-0,741583	NaN	NaN	NaN
-1,21907	NaN	-1,68077	-0,238569	0,286413	1,86193	0,594156	-0,782043	-0,0438719	-0,343354
3,77071	3,01487	2,43174	2,84373	3,12426	NaN	2,79112	3,34482	2,9816	2,27525
NaN	NaN	NaN	-0,238569	0,286413	1,86193	0,594156	NaN	NaN	NaN
-0,417362	-0,0840321	-0,569603	0,558565	-0,829157	NaN	-0,326694	NaN	-0,981193	NaN
-0,417362	-0,0840321	-0,569603	0,558565	-0,829157	NaN	-0,326694	NaN	-0,981193	NaN
-0,663769	-0,920227	NaN	1,05052	-1,1485	NaN	1,00351	-0,161957	-0,394397	-0,269324
-0,663769	-0,920227	NaN	1,05052	NaN	NaN	1,00351	-0,161957	NaN	-0,269324
NaN	-1,29774	NaN	NaN	NaN	NaN	NaN	NaN	NaN	NaN
NaN	-1,29774	NaN	NaN	NaN	NaN	NaN	NaN	NaN	NaN
NaN	-1,38936	NaN	NaN	NaN	NaN	NaN	-1,47669	-2,17438	-2,44238
NaN	-1,38936	NaN	NaN	NaN	NaN	NaN	-1,47669	-2,17438	-2,44238
NaN	NaN	NaN	NaN	NaN	NaN	NaN	NaN	NaN	NaN
0,418743	-0,948208	-0,871723	0,282049	-1,28442	NaN	-0,969879	0,0920792	-0,264729	-0,943065
0,870453	0,912018	0,192734	1,20642	NaN	NaN	NaN	1,1076	0,569049	NaN
NaN	NaN	NaN	NaN	NaN	NaN	NaN	NaN	NaN	NaN
NaN	NaN	NaN	NaN	NaN	NaN	NaN	NaN	NaN	NaN
-1,25644	-2,09905	-1,98379	NaN	-1,37178	NaN	NaN	NaN	NaN	NaN
-1,25644	-2,09905	-1,98379	NaN	-1,37178	NaN	NaN	NaN	NaN	NaN
NaN	NaN	NaN	0,0807495	0,808138	-1,34777	NaN	NaN	NaN	NaN
-0,531548	-1,05101	NaN	-1,25767	-0,852787	NaN	-0,715479	NaN	NaN	NaN
-0,531548	-1,05101	NaN	-1,25767	-0,852787	NaN	-0,715479	NaN	NaN	NaN
NaN	1,23086	NaN	0,765091	0,383308	0,90765	1,39576	NaN	NaN	NaN
NaN	1,23086	NaN	0,765091	0,383308	0,90765	1,39576	NaN	NaN	NaN
NaN	NaN	NaN	-1,93887	NaN	-0,827742	-0,969355	NaN	NaN	NaN
NaN	NaN	NaN	-1,93887	NaN	-0,827742	-0,969355	NaN	NaN	NaN
-0,678911	-0,548014	-1,23206	-0,106125	NaN	1,06645	-0,50803	NaN	NaN	NaN
-0,678911	-0,548014	-1,23206	-0,106125	NaN	1,06645	-0,50803	NaN	NaN	NaN
-1,07966	-2,76617	-1,00993	NaN	NaN	NaN	NaN	-0,952188	-1,14008	-1,54512
-1,07966	-2,76617	-1,00993	NaN	NaN	NaN	NaN	-0,952188	-1,14008	-1,54512
NaN	NaN	NaN	0,757717	NaN	0,393761	0,733789	NaN	NaN	NaN
NaN	NaN	NaN	0,757717	NaN	0,393761	0,733789	NaN	NaN	NaN

Polito et al. Universal ready-to-use immunotherapeutic approach for the treatment of cancer: expanded and activated polyclonal γδ memory T cells.
Phosphorylation sites

NaN	NaN	NaN	-2,12366	NaN	0,0821037	-2,61911	NaN	NaN	NaN
NaN	NaN	NaN	-2,12366	NaN	0,0821037	-2,61911	NaN	NaN	NaN
NaN	0,204201	-0,319239	-0,193918	0,0857143	NaN	-0,628923	-0,747295	NaN	-0,479716
NaN	NaN	NaN	NaN	NaN	NaN	NaN	NaN	NaN	NaN
NaN	NaN	NaN	NaN	NaN	NaN	NaN	NaN	NaN	NaN
1,03754	NaN	-0,186026	-0,361774	0,679411	NaN	NaN	0,796743	0,090518	0,589557
1,03754	NaN	-0,186026	-0,361774	0,679411	NaN	NaN	0,796743	0,090518	0,589557
0,403112	-0,103916	0,749397	-0,796886	1,95169	-2,00742	0,264662	-0,243233	-0,542096	0,0802832
0,403112	-0,103916	0,749397	-0,796886	1,95169	-2,00742	0,264662	-0,243233	-0,542096	0,0802832
-0,539701	-0,930197	0,34688	0,240143	-0,871704	-1,23689	0,182293	0,164257	0,0550871	0,0801535
-0,539701	-0,930197	0,34688	0,240143	-0,871704	-1,23689	0,182293	0,164257	0,0550871	0,0801535
1,21647	1,51775	1,74207	-0,108757	1,32144	NaN	0,484737	1,57652	2,26962	1,53439
1,21647	1,51775	1,74207	-0,108757	1,32144	NaN	0,484737	1,57652	2,26962	1,53439
NaN	NaN	NaN	-1,65455	-2,69664	-2,32703	NaN	NaN	NaN	NaN
NaN	NaN	NaN	NaN	NaN	NaN	NaN	-3,45737	-2,16304	-2,0993
NaN	NaN	NaN	-1,85136	-2,9468	-1,08973	-2,0534	NaN	NaN	NaN
0,0504646	-0,0182648	-0,55776	-0,340181	0,162535	-1,31858	-0,892628	-1,20308	-0,49894	-0,803149
2,274	2,14749	2,76073	2,37957	4,40652	2,03088	2,90461	1,79502	2,36179	2,35717
2,274	2,14749	2,76073	2,37957	4,40652	2,03088	2,90461	1,79502	2,36179	2,35717
0,231001	-0,201895	-1,43511	0,614145	NaN	1,10022	-0,0241337	NaN	0,138127	-0,729575
0,231001	-0,201895	-1,43511	0,614145	NaN	1,10022	-0,0241337	NaN	0,138127	-0,729575
0,928482	-1,95874	0,345045	1,303	1,18417	2,15976	1,33735	-0,563265	-0,409108	-0,757974
0,928482	-1,95874	0,345045	1,303	1,18417	2,15976	1,33735	-0,563265	-0,409108	-0,757974
0,631031	NaN	0,140387	3,75268	1,35618	-0,805161	2,84484	NaN	NaN	NaN
0,631031	NaN	0,140387	3,75268	1,35618	1,1992	2,84484	NaN	NaN	NaN
1,97805	1,47898	-0,218878	1,39191	0,636032	1,79251	1,00773	0,127306	0,890424	-0,0361929
1,97805	1,47898	-0,218878	1,39191	0,636032	1,79251	1,00773	0,127306	0,890424	-0,0361929
3,13085	2,7052	2,84545	3,69324	3,7381	0,388735	3,10413	2,82602	3,42699	NaN
0,271135	-0,737652	-0,411938	-0,653946	NaN	NaN	NaN	-0,308146	NaN	NaN
-0,170233	-1,21058	0,407232	-1,35287	0,269295	NaN	-1,02525	0,719172	0,370593	0,316817
-0,170233	-1,21058	0,407232	-1,35287	0,269295	NaN	-1,02525	0,719172	0,370593	0,316817
NaN	NaN	NaN	-1,54247	NaN	-0,179573	-1,86546	NaN	NaN	NaN
NaN	NaN	0,394293	NaN	NaN	-0,215578	-0,0826149	NaN	-0,471665	NaN
-1,255	-1,00936	-1,13525	-1,20295	NaN	NaN	-0,490248	-1,98129	-1,62055	-1,10526

Polito et al. Universal ready-to-use immunotherapeutic approach for the treatment of cancer: expanded and activated polyclonal γδ memory T cells.
Phosphorylation sites

-1,255	-1,00936	-1,13525	-1,20295	NaN	NaN	-0,490248	-1,98129	-1,62055	-1,10526
0,851652	-1,18701	-1,65804	-1,81747	0,247593	0,51268	0,520666	0,0497646	-0,201837	0,497674
0,851652	-0,48539	-1,48182	-1,81747	0,247593	0,51268	0,520666	0,0497646	-0,201837	0,497674
-1,1897	-0,48539	-1,48182	NaN	NaN	NaN	NaN	NaN	NaN	NaN
2,71369	2,93508	2,83865	2,4246	2,4805	1,77937	1,91928	2,74511	2,81709	2,85219
-0,978731	-1,53565	-1,53925	-0,349848	2,4805	0,128992	0,297289	2,74511	NaN	2,85219
2,71369	2,93508	2,83865	2,4246	NaN	1,77937	1,91928	NaN	2,81709	NaN
NaN	-0,527142	NaN	-0,907875	NaN	0,357742	-0,9536	NaN	NaN	NaN
NaN	-0,527142	NaN	-0,907875	NaN	0,357742	-0,9536	NaN	NaN	NaN
-1,25954	-2,60045	-0,0605392	0,166811	NaN	0,96261	1,28201	-1,39662	-0,842784	-0,535737
NaN	2,23517	NaN	NaN	NaN	2,71755	2,41647	1,91885	NaN	2,48585
3,14679	NaN	2,46514	2,9996	NaN	NaN	2,41647	1,91885	2,42972	2,48585
-0,787836	NaN	-1,99009	-1,24289	1,09407	NaN	0,354183	0,585669	0,079381	0,823466
1,97385	1,30958	1,88562	0,471228	2,42445	0,681559	0,663857	0,707581	0,71087	1,51364
NaN	NaN	NaN	-0,980154	NaN	-0,584549	-0,325098	NaN	NaN	NaN
NaN	NaN	NaN	-1,11926	-1,84327	-3,29934	-1,91407	NaN	NaN	NaN
NaN	NaN	NaN	-1,11926	-1,84327	-3,29934	-1,91407	NaN	NaN	NaN
NaN	NaN	NaN	-2,24529	NaN	-1,14739	-3,62447	NaN	NaN	NaN
NaN	NaN	NaN	-2,24529	NaN	-1,14739	-3,62447	NaN	NaN	NaN
NaN	NaN	NaN	-0,490564	NaN	-0,498367	-1,83174	NaN	NaN	NaN
-0,445049	-0,718683	NaN	NaN	NaN	NaN	NaN	-0,980896	-0,516757	-1,27364
-0,445049	-0,718683	NaN	NaN	NaN	NaN	NaN	-0,980896	-0,516757	-1,27364
-1,27275	-1,42694	-1,83594	-2,38453	-1,62629	NaN	-1,66323	-1,32858	-1,90825	-1,57086
3,24837	4,0324	3,27409	3,3304	3,26705	1,95234	3,3992	3,41098	2,94271	3,05382
3,24837	4,0324	3,27409	3,3304	3,26705	1,95234	3,3992	3,41098	2,94271	3,05382
3,62755	3,91906	3,65985	3,51732	3,69901	1,17769	4,31312	4,29662	4,11688	3,94159
3,62755	3,91906	3,65985	3,51732	3,69901	1,17769	4,31312	4,29662	4,11688	3,94159
1,56791	4,32007	2,75446	2,19943	1,78382	-1,28446	3,01436	1,21594	2,7248	2,56876
NaN	NaN	NaN	NaN	NaN	NaN	NaN	-0,3566	0,627831	NaN
NaN	1,23636	0,675278	0,228548	NaN	-0,441799	1,82372	1,54788	1,3015	0,79769
NaN	1,23636	0,675278	0,228548	NaN	-0,441799	1,82372	1,54788	1,3015	0,79769
NaN	NaN	NaN	NaN	NaN	NaN	NaN	NaN	NaN	NaN
-1,76528	-2,24546	-1,47993	-0,399368	-1,7725	-0,858656	NaN	NaN	NaN	NaN
-0,0630054	0,381483	-2,21449	-1,6357	0,0747948	-1,18902	-0,369967	-0,474539	-1,07167	-1,29714

Polito et al. Universal ready-to-use immunotherapeutic approach for the treatment of cancer: expanded and activated polyclonal γδ memory T cells.
Phosphorylation sites

NaN	NaN	-0,492071	-0,429453	NaN	-1,36597	NaN	NaN	NaN	NaN
NaN	NaN	NaN	0,170933	NaN	0,582045	-1,60042	NaN	NaN	NaN
NaN	NaN	NaN	0,170933	NaN	0,582045	-1,60042	NaN	NaN	NaN
NaN	NaN	NaN	NaN	NaN	-0,138102	NaN	NaN	NaN	NaN
NaN	NaN	NaN	NaN	NaN	NaN	NaN	NaN	NaN	NaN
NaN	NaN	NaN	-0,139307	-1,74479	0,0371456	NaN	NaN	NaN	NaN
NaN	NaN	NaN	-0,139307	-1,74479	0,0371456	NaN	NaN	NaN	NaN
-0,532969	-1,80103	-0,12027	-0,426361	-2,10583	-0,0936928	-1,32004	NaN	-0,369966	-1,1942
-0,532969	-1,80103	-0,12027	-0,426361	-2,10583	-0,0936928	-1,32004	-0,512854	-0,369966	-1,1942
NaN	NaN	NaN	0,456432	-1,04667	0,253035	NaN	NaN	NaN	NaN
NaN	NaN	NaN	0,456432	-1,04667	0,253035	NaN	NaN	NaN	NaN
0,222149	-0,482513	2,93606	2,71633	2,12001	3,2666	2,4483	2,34962	2,09705	2,28362
0,222149	-0,482513	2,93606	2,71633	2,12001	3,2666	2,4483	2,34962	2,09705	2,28362
4,25041	4,68772	3,66954	4,11816	4,34824	3,24868	4,45243	3,57346	3,75159	3,52643
4,25041	4,68772	3,66954	4,11816	4,34824	3,24868	4,45243	3,57346	3,75159	3,52643
NaN	1,23636	0,675278	0,228548	NaN	-0,441799	1,82372	1,54788	1,3015	0,79769
NaN	1,23636	0,675278	0,228548	NaN	-0,441799	1,82372	1,54788	1,3015	0,79769
NaN	-0,569931	NaN	0,508404	-1,16179	NaN	0,122561	NaN	NaN	NaN
NaN	NaN	NaN	0,508404	-1,16179	NaN	0,122561	NaN	NaN	NaN
NaN	NaN	NaN	0,552437	NaN	-1,52349	-0,654198	NaN	NaN	NaN
NaN	NaN	NaN	0,552437	NaN	-1,52349	-0,654198	NaN	NaN	NaN
NaN	-1,77078	-0,453449	2,09168	-0,669958	NaN	0,52062	0,61536	0,412152	-0,22646
3,60802	2,73579	2,10527	2,53475	3,69964	1,75685	2,6918	3,33585	2,41518	2,96088
1,40097	NaN	-0,451738	1,75576	1,01777	1,80422	1,63889	1,0114	1,0236	1,77768
NaN	NaN	NaN	NaN	NaN	1,80422	NaN	1,0114	1,0236	NaN
1,40097	NaN	-0,451738	1,75576	1,01777	NaN	1,63889	NaN	NaN	1,77768
NaN	NaN	NaN	-1,09617	-1,79019	-0,37035	-1,39129	NaN	NaN	NaN
NaN	NaN	NaN	-1,09617	-1,79019	-0,37035	-1,39129	NaN	NaN	NaN
-1,57917	-1,30368	-2,21696	-1,01584	-0,285875	NaN	NaN	NaN	NaN	NaN
-1,57917	-1,30368	-2,21696	-1,01584	-0,285875	NaN	NaN	NaN	NaN	NaN
2,5047	4,02553	2,93054	1,69011	3,79505	0,993996	2,51847	2,52826	2,90361	2,92785
2,36646	3,81576	3,01005	1,61373	3,84572	0,47196	2,23117	2,5613	2,88876	2,57778
-1,63777	-1,31868	-2,17874	0,0273132	-0,671679	-0,574841	0,0643559	NaN	NaN	NaN
1,29943	1,6773	NaN	1,60939	NaN	0,0125294	1,5674	NaN	1,57629	1,43867

Polito et al. Universal ready-to-use immunotherapeutic approach for the treatment of cancer: expanded and activated polyclonal γδ memory T cells.
Phosphorylation sites

0,611925	2,61209	-2,15683	-0,283707	-0,994297	-1,48028	-0,630167	NaN	NaN	NaN
0,559668	0,154261	-0,27702	-0,731943	-0,0714874	NaN	0,0870457	-0,827353	-0,940793	-1,09532
0,559668	0,154261	-0,27702	-0,731943	-0,0714874	NaN	0,0870457	-0,827353	-0,940793	-1,09532
0,238323	-0,680033	0,541986	-0,632301	0,975887	-2,83219	0,0940342	1,08061	0,468719	0,267835
0,238323	-0,680033	0,541986	-0,632301	0,975887	-2,83219	0,0940342	1,08061	0,468719	0,267835
3,32532	2,27823	3,05482	NaN	3,84833	NaN	NaN	3,63691	3,43655	3,50352
0,118351	0,0233021	0,244608	0,726061	NaN	-0,101128	1,12672	-0,132593	-0,562758	-0,0811491
0,118351	0,0233021	0,244608	0,726061	NaN	-0,101128	1,12672	-0,132593	-0,562758	-0,0811491
-1,21197	-1,64453	NaN	-1,62188	-1,51924	-0,981709	NaN	NaN	NaN	NaN
NaN	-1,50982	-1,5187	0,670092	-1,77882	NaN	-0,401056	NaN	-0,343625	-2,106
NaN	0,255865	-1,5187	NaN	0,381781	NaN	-1,4733	1,70026	1,0044	-2,106
0,940046	0,255865	-1,23121	NaN	0,381781	NaN	-1,4733	1,70026	1,0044	0,771176
-1,05626	-1,26281	-1,36235	NaN	NaN	NaN	NaN	-0,230446	NaN	NaN
NaN	NaN	NaN	0,670092	-1,77882	NaN	-0,401056	NaN	-0,343625	-0,697104
-1,99982	-2,09299	-2,69772	-0,32465	-2,63242	-0,992231	-0,914701	NaN	NaN	NaN
-1,99982	-2,09299	-2,69772	-0,32465	-2,63242	-0,992231	-0,914701	NaN	NaN	NaN
-1,47374	-1,93579	-2,48556	-1,81101	-0,492393	NaN	-0,958412	NaN	NaN	NaN
2,78236	2,22054	1,06915	2,44962	1,53135	2,47017	1,46118	1,62252	1,69607	1,23233
2,78236	2,22054	1,06915	2,44962	1,53135	2,47017	1,46118	1,62252	1,69607	1,23233
-0,660061	-2,11388	0,636398	0,488625	-0,497923	1,3355	-0,271677	-0,909958	-1,01623	-0,97001
-0,660061	-2,11388	0,636398	0,488625	-0,497923	1,3355	-0,271677	-0,909958	-1,01623	-0,97001
0,82052	0,0249424	0,632164	0,746586	0,475616	0,648457	0,506357	0,23806	0,0753374	0,0554552
0,82052	0,0249424	0,632164	0,746586	0,475616	0,648457	0,506357	0,23806	0,0753374	0,0554552
3,0334	1,30095	NaN	1,88764	2,31888	0,868805	NaN	1,21519	0,710618	2,07074
3,0334	1,30095	NaN	1,88764	2,31888	0,868805	NaN	1,21519	0,710618	2,07074
NaN	NaN	NaN	NaN	-1,14246	1,85876	-0,463039	NaN	NaN	NaN
NaN	NaN	NaN	NaN	-1,14246	1,85876	-0,463039	NaN	NaN	NaN
NaN	1,4297	-0,0825005	1,44891	2,46214	0,15753	2,48133	NaN	-0,423615	1,44619
NaN	1,4297	-0,0825005	1,44891	2,46214	0,15753	2,48133	NaN	-0,423615	1,44619
NaN	NaN	NaN	0,223818	NaN	0,630144	0,332695	NaN	NaN	NaN
NaN	NaN	NaN	0,223818	NaN	0,630144	0,332695	NaN	NaN	NaN
NaN	NaN	NaN	0,640327	-2,36389	1,75855	1,89334	NaN	NaN	NaN
NaN	NaN	NaN	0,640327	-2,36389	1,75855	1,89334	NaN	NaN	NaN
NaN	NaN	NaN	1,22139	NaN	-0,823641	0,84409	NaN	NaN	NaN

Polito et al. Universal ready-to-use immunotherapeutic approach for the treatment of cancer: expanded and activated polyclonal γδ memory T cells.
Phosphorylation sites

NaN	NaN	NaN	1,22139	NaN	-0,823641	0,84409	NaN	NaN	NaN
NaN	NaN	NaN	-0,26523	NaN	NaN	-1,298	NaN	NaN	NaN
NaN	NaN	NaN	-0,26523	NaN	NaN	-1,298	NaN	NaN	NaN
0,0236874	0,0240231	-0,187883	0,593706	-0,507107	0,0154953	0,329712	-0,803694	0,0690355	-0,632602
NaN	NaN	NaN	-2,46934	NaN	-1,51922	NaN	NaN	NaN	NaN
NaN	NaN	NaN	-2,46934	NaN	-1,51922	NaN	NaN	NaN	NaN
2,43855	0,98443	1,18946	2,67721	2,26961	3,16203	1,92729	1,73507	1,66018	1,08185
2,43855	0,98443	1,18946	2,67721	2,26961	3,16203	1,92729	1,73507	1,66018	1,08185
-0,807232	-1,00295	-0,819691	NaN	-0,838226	-0,959867	-0,442425	NaN	NaN	NaN
-0,807232	-1,00295	-0,819691	NaN	-0,838226	-0,959867	-0,442425	NaN	NaN	NaN
NaN	NaN	NaN	-2,32143	-1,26913	-2,27124	NaN	NaN	NaN	NaN
NaN	NaN	NaN	-2,32143	-1,26913	-2,27124	NaN	NaN	NaN	NaN
1,0101	-0,502943	-2,32199	NaN	0,731918	-0,424021	NaN	NaN	-0,0432596	-0,885377
1,0101	-0,502943	-2,32199	-0,806532	0,731918	-0,424021	-1,84284	-1,23825	-0,0432596	-0,885377
NaN	NaN	NaN	-0,99675	-1,43749	-0,420296	-1,277	-1,72112	-1,33853	NaN
NaN	NaN	NaN	-0,99675	-1,43749	-0,420296	-1,277	-1,72112	-1,33853	NaN
NaN	NaN	-0,708761	-0,319136	NaN	-0,501383	-2,15017	NaN	NaN	NaN
-0,682526	-1,6499	-0,208897	-1,33723	-0,0555325	-2,59644	-0,365784	0,272621	0,763646	0,173579
-0,682526	-1,6499	-0,208897	-1,33723	-0,0555325	-2,59644	-0,365784	NaN	0,763646	0,173579
0,50482	3,15739	0,786367	2,21515	1,88328	3,03707	2,72405	-0,214558	1,86706	1,61459
0,50482	3,15739	0,786367	2,21515	1,88328	3,03707	2,72405	-0,214558	1,86706	1,61459
-1,31555	-1,0243	-1,36218	-2,07475	-1,4898	NaN	-1,48633	-1,07834	NaN	-1,74884
0,903522	-1,33769	-1,41253	-0,353718	NaN	0,315226	-1,00861	NaN	NaN	NaN
NaN	NaN	NaN	-0,353718	NaN	0,315226	-3,37879	NaN	NaN	NaN
-1,16802	NaN	-2,21922	NaN	-1,28525	NaN	NaN	-1,88694	-2,22583	-1,95449
-1,16802	NaN	-2,21922	NaN	-1,28525	NaN	NaN	-1,88694	-2,22583	-1,95449
2,85483	1,50694	2,37779	2,12409	1,57026	2,02224	2,1844	1,36969	1,67702	1,15972
2,85483	1,50694	2,37779	2,12409	1,57026	2,02224	-2,05972	1,36969	1,67702	1,15972
NaN	NaN	NaN	-1,08684	-1,81318	-0,655277	-1,29252	NaN	NaN	NaN
2,30387	2,59259	1,59539	-0,212179	2,28521	-0,0913925	0,0868359	1,71119	1,69575	1,31803
2,30387	2,59259	1,59539	-0,212179	2,28521	-0,0913925	NaN	1,71119	1,69575	1,31803
-0,485727	-1,46979	NaN	-0,651634	NaN	-0,130735	-1,60701	NaN	NaN	NaN
-0,485727	-1,46979	NaN	-0,651634	NaN	-0,130735	-1,60701	NaN	NaN	NaN
NaN	NaN	NaN	-0,0289268	-2,43313	NaN	-1,78949	NaN	NaN	NaN

Polito et al. Universal ready-to-use immunotherapeutic approach for the treatment of cancer: expanded and activated polyclonal γδ memory T cells.
Phosphorylation sites

NaN	NaN	NaN	-0,0289268	-2,43313	NaN	-1,78949	NaN	NaN	NaN
1,47478	1,15852	NaN	NaN	NaN	0,698051	NaN	NaN	0,494161	NaN
1,47478	1,15852	NaN	NaN	NaN	0,698051	NaN	NaN	0,494161	NaN
NaN	NaN	-0,973183	-1,90502	-1,38866	-2,18698	-0,368608	NaN	NaN	NaN
-0,185736	NaN	-0,135464	-0,528587	NaN	NaN	0,245468	-0,502569	-0,397149	-0,782634
-0,185736	NaN	-0,135464	-0,528587	NaN	NaN	0,245468	-0,502569	-0,397149	-0,782634
NaN	NaN	NaN	NaN	-2,47807	0,408463	-0,561378	NaN	NaN	NaN
NaN	NaN	NaN	0,173574	0,245268	NaN	-0,0817623	NaN	NaN	NaN
NaN	NaN	NaN	0,173574	0,245268	NaN	-0,0817623	NaN	NaN	NaN
NaN	1,88814	NaN	1,75728	1,75993	2,32325	2,44104	-0,712791	-0,467093	1,67161
NaN	1,88814	NaN	1,75728	1,75993	2,32325	2,44104	-0,712791	-0,467093	1,67161
1,28979	1,40049	1,27849	0,050663	1,7982	-0,854034	0,879381	NaN	NaN	NaN
-0,478867	0,624973	-1,60331	NaN	NaN	NaN	NaN	NaN	NaN	NaN
NaN	4,77617	NaN	3,80241	3,493	NaN	3,79659	3,27163	NaN	NaN
NaN	4,77617	NaN	3,80241	3,493	NaN	3,79659	3,27163	NaN	NaN
2,33707	5,36552	4,69739	3,88573	5,68032	1,18814	4,88002	3,74546	4,00023	5,14934
NaN	-2,40264	NaN	NaN	-2,29324	-3,73685	NaN	-2,67507	-2,28518	-2,23176
NaN	-2,40264	NaN	NaN	-2,29324	-3,73685	NaN	-2,67507	-2,28518	-2,23176
2,33707	5,36552	4,69739	3,88573	5,68032	1,18814	4,88002	3,74546	4,00023	5,14934
NaN	NaN	NaN	NaN	-2,07149	-1,55437	-0,737778	NaN	NaN	NaN
-0,165144	NaN	0,523598	-0,604925	0,340633	NaN	1,36495	0,80319	0,635245	0,300756
-0,165144	NaN	0,523598	-0,604925	0,340633	NaN	1,36495	0,80319	0,635245	0,300756
0,748083	1,30032	1,1475	0,978462	0,649942	0,419167	1,13193	0,836676	0,570846	0,659787
0,429605	0,489841	0,41787	-0,141415	0,0648251	0,421227	0,502893	0,794445	0,401137	0,431457
0,429605	0,489841	0,41787	-0,141415	0,0648251	0,421227	0,502893	0,794445	0,401137	0,431457
2,10571	0,437313	1,51994	1,80882	2,52854	1,84512	2,04156	0,669714	0,561564	0,53991
-2,07203	NaN	0,395807	1,72367	-2,8626	0,786787	2,41709	NaN	NaN	NaN
-2,07203	NaN	0,395807	1,72367	-2,8626	0,786787	2,41709	NaN	NaN	NaN
0,385725	-1,06965	0,00484657	-1,73947	0,279356	-1,21959	NaN	NaN	NaN	NaN
0,385725	-1,06965	0,00484657	-1,73947	0,279356	-1,21959	NaN	NaN	NaN	NaN
NaN	-0,296015	NaN	-1,30154	-1,5794	-2,01515	-0,58341	NaN	NaN	NaN
5,68998	5,30974	5,10195	4,96667	4,85475	5,12216	4,93351	5,00233	4,87967	5,2989
3,42754	1,7456	5,67918	5,54867	3,79025	5,5964	4,00094	3,83941	4,00458	4,33959
3,42754	1,7456	5,67918	5,54867	3,79025	5,5964	4,00094	3,83941	4,00458	4,33959

Polito et al. Universal ready-to-use immunotherapeutic approach for the treatment of cancer: expanded and activated polyclonal γδ memory T cells.
Phosphorylation sites

NaN	NaN	NaN	-2,23512	NaN	1,50788	1,26704	NaN	NaN	NaN
1,47357	-0,13658	NaN	2,87877	NaN	NaN	3,37947	-1,52504	-1,58973	1,46808
1,47357	-0,13658	2,79433	2,87877	NaN	3,32169	3,37947	1,52015	1,43788	1,46808
NaN	NaN	NaN	1,04162	NaN	0,386171	0,874149	NaN	NaN	NaN
2,69871	1,00871	1,53113	3,63869	2,05205	4,4185	3,75928	2,13165	2,0352	1,65137
2,69871	1,00871	1,53113	3,63869	2,05205	4,4185	3,75928	2,13165	2,0352	1,65137
NaN	NaN	-0,912674	-1,59681	-4,9267	-1,57055	-1,12758	NaN	NaN	NaN
-1,49504	NaN	-2,98185	-1,62313	-0,556221	-1,62247	-1,9305	-1,26312	-0,684276	-1,39973
NaN	-2,07096	NaN	0,38306	NaN	0,71348	-0,431503	NaN	NaN	-0,814406
-0,921932	NaN	-1,26668	-0,515337	NaN	-1,13926	NaN	NaN	NaN	NaN
-0,921932	-1,43918	-1,26668	-0,515337	NaN	-1,13926	-0,245901	NaN	NaN	NaN
1,56006	-0,733191	0,189781	1,28108	0,941895	2,08374	1,45043	1,26139	0,615302	1,70247
1,56006	-0,733191	0,189781	1,28108	0,941895	2,08374	1,45043	1,26139	0,615302	1,70247
NaN	NaN	NaN	-2,01588	NaN	-1,34826	-2,63498	NaN	NaN	NaN
NaN	NaN	NaN	-2,01588	NaN	-1,34826	-2,63498	NaN	NaN	NaN
0,915405	0,329618	0,785137	0,460314	0,849421	0,0366783	0,390539	0,69113	0,549283	0,743342
0,915405	0,329618	0,785137	0,460314	0,849421	0,0366783	0,390539	0,69113	0,549283	0,743342
0,301771	-0,211153	-0,0734615	NaN	NaN	NaN	NaN	-0,599464	-0,34287	NaN
1,15255	1,31335	NaN	0,572105	0,242748	NaN	0,805738	NaN	NaN	NaN
-0,120928	-0,459347	-0,797674	-2,1187	0,318438	-3,04427	-0,959759	-0,992313	-0,990251	-0,944699
-1,49208	-2,88807	0,678328	NaN	NaN	NaN	NaN	NaN	NaN	NaN
-1,49208	-2,88807	0,678328	NaN	NaN	NaN	NaN	NaN	NaN	NaN
-0,540091	-0,0998878	-0,409178	0,881716	-0,847866	1,67204	0,231089	0,243422	-0,256228	-0,542706
-0,540091	-0,0998878	-0,409178	0,881716	-0,847866	1,67204	0,231089	0,243422	-0,256228	-0,542706
NaN	NaN	NaN	-1,29769	-1,73937	-0,798954	-1,20023	NaN	NaN	NaN
NaN	NaN	NaN	-1,29769	-1,73937	-0,798954	-1,20023	NaN	NaN	NaN
1,44893	1,11402	1,08722	0,848515	2,12537	1,359	1,19527	0,643894	1,13499	0,622067
1,44893	1,11402	1,08722	0,848515	2,12537	1,359	1,19527	0,643894	1,13499	0,622067
NaN	NaN	NaN	NaN	NaN	NaN	NaN	2,1158	2,38381	1,40249
NaN	-0,566673	NaN	NaN	NaN	NaN	NaN	2,1158	2,38381	1,40249
NaN	-0,566673	NaN	NaN	NaN	NaN	NaN	0,222689	-0,0473661	-0,190425
NaN	NaN	NaN	-1,31529	-2,99263	-1,1644	-0,359377	-2,2998	-2,63188	-3,01361
NaN	NaN	NaN	-1,10705	-2,65988	-1,54343	-1,70247	NaN	NaN	NaN
NaN	NaN	NaN	-0,295729	NaN	-0,50001	-0,808304	NaN	NaN	NaN

NaN	NaN	NaN	-0,295729	NaN	-0,50001	-0,808304	NaN	NaN	NaN
NaN	NaN	NaN	NaN	NaN	NaN	NaN	NaN	NaN	NaN
NaN	NaN	NaN	NaN	NaN	NaN	NaN	NaN	NaN	NaN
0,796047	1,49847	1,8942	1,02274	0,835196	1,89945	1,70789	-0,271133	0,164077	0,0361147
0,796047	1,49847	1,8942	1,02274	0,835196	1,89945	1,70789	-0,271133	0,164077	0,0361147
NaN	NaN	NaN	-0,240414	NaN	-0,271688	-0,728897	NaN	NaN	NaN
NaN	NaN	NaN	-2,9104	NaN	NaN	-3,13881	NaN	NaN	NaN
5,23319	5,71271	5,05388	NaN	5,09946	-2,07479	1,31938	4,10867	2,41948	4,13341
5,23319	5,71271	5,05388	-0,206505	5,09946	-2,07479	1,31938	4,10867	5,054	4,13341
0,91848	0,906958	1,02432	1,20442	1,27191	1,39362	0,336489	1,29603	0,623845	2,30132
0,91848	0,906958	1,14511	2,10888	1,70831	1,39362	1,03132	1,29603	1,70421	2,30132
-0,768314	-1,22897	-0,534822	1,01285	0,593269	0,634398	0,310684	-0,657335	-0,407014	-0,293029
NaN	NaN	NaN	0,684116	NaN	2,34916	-2,54969	NaN	NaN	NaN
NaN	NaN	NaN	-1,27243	-0,197247	-1,42821	-0,928501	NaN	NaN	NaN
NaN	NaN	NaN	-1,27243	-0,197247	-1,42821	-0,928501	NaN	NaN	NaN
NaN	NaN	NaN	-0,508913	NaN	1,15236	-0,967255	NaN	NaN	NaN
NaN	NaN	NaN	NaN	NaN	NaN	NaN	NaN	NaN	NaN
NaN	NaN	NaN	-0,151873	NaN	-2,47168	-1,66096	NaN	NaN	NaN
NaN	NaN	NaN	-0,151873	NaN	-2,47168	-1,66096	NaN	NaN	NaN
NaN	NaN	NaN	NaN	NaN	NaN	NaN	NaN	NaN	NaN
NaN	NaN	NaN	-1,1911	NaN	-1,94362	-2,27084	NaN	NaN	NaN
NaN	NaN	NaN	-2,78512	NaN	-1,99467	0,113024	NaN	NaN	NaN
NaN	NaN	NaN	-2,78512	NaN	-1,99467	0,113024	NaN	NaN	NaN
NaN	NaN	NaN	NaN	NaN	NaN	NaN	-0,962261	-1,2254	-1,95405
NaN	NaN	NaN	NaN	NaN	NaN	NaN	NaN	NaN	NaN
NaN	NaN	NaN	NaN	NaN	NaN	NaN	-1,96581	-1,85601	-1,28116
NaN	NaN	NaN	NaN	NaN	NaN	NaN	-1,96581	-1,85601	-1,28116
NaN	NaN	NaN	NaN	NaN	NaN	NaN	NaN	NaN	NaN
NaN	NaN	NaN	NaN	NaN	NaN	NaN	NaN	NaN	NaN
-1,8655	-0,949713	NaN	-0,471603	-0,0117054	-1,70955	-0,786978	NaN	NaN	NaN
-1,8655	-0,949713	NaN	-0,471603	-0,0117054	-1,70955	-0,786978	NaN	NaN	NaN
4,56373	4,22281	4,29134	3,88261	4,39643	3,56355	3,96361	4,5099	5,0625	5,27409
1,65462	0,727228	0,954065	-0,556648	2,57738	-0,735048	0,613317	2,32305	1,69271	2,10155
NaN	NaN	NaN	-1,00872	NaN	-1,97385	-1,39846	NaN	NaN	NaN

Polito et al. Universal ready-to-use immunotherapeutic approach for the treatment of cancer: expanded and activated polyclonal γδ memory T cells.
Phosphorylation sites

NaN	NaN	NaN	-1,00872	NaN	-1,97385	-1,39846	NaN	NaN	NaN
NaN	NaN	NaN	-0,430443	-2,20591	0,706301	0,224161	NaN	NaN	NaN
NaN	NaN	NaN	NaN	-2,20591	0,706301	0,224161	NaN	NaN	NaN
NaN	0,415287	NaN	-0,0806694	0,461664	1,03504	NaN	-0,75835	NaN	0,526479
1,43009	2,7568	1,62008	1,80567	2,60904	3,23296	2,56547	1,23493	1,07171	1,82987
NaN	2,7568	NaN	1,80567	2,60904	3,23296	NaN	1,23493	NaN	NaN
-0,817896	-2,48354	-2,38415	NaN	NaN	NaN	NaN	-0,137468	-0,0693178	-0,866048
-0,817896	-2,48354	-2,38415	NaN	NaN	NaN	NaN	-0,137468	-0,0693178	-0,866048
NaN	NaN	NaN	1,80704	NaN	0,164602	0,537622	-0,975639	0,672782	-0,375295
0,21122	0,934116	1,03563	-0,0530968	1,6418	-0,424261	0,572153	0,597092	-0,213323	0,471282
0,21122	0,934116	1,03563	-0,0530968	1,6418	-0,424261	0,572153	0,597092	NaN	NaN
-2,07288	-1,99877	1,71074	0,89255	NaN	0,914978	1,99361	-1,17195	-1,35397	-1,83497
-2,07288	-1,99877	1,71074	0,89255	NaN	0,914978	1,99361	-1,17195	-1,35397	-1,83497
2,85184	2,27273	1,69728	NaN	3,37929	NaN	1,32599	1,83346	1,811	-0,919131
NaN	NaN	NaN	NaN	3,37929	NaN	1,32599	1,83346	1,811	-0,919131
2,85184	2,27273	1,69728	NaN	NaN	NaN	1,32599	NaN	NaN	NaN
NaN	4,77617	NaN	3,80241	3,493	NaN	3,79659	3,27163	NaN	NaN
NaN	4,77617	NaN	3,80241	3,493	NaN	3,79659	3,27163	NaN	NaN
NaN	NaN	NaN	-0,24435	NaN	-2,42724	-2,36532	NaN	NaN	NaN
NaN	NaN	NaN	-0,24435	NaN	-2,42724	-2,36532	NaN	NaN	NaN
0,191048	NaN	-0,401569	1,92849	0,00836182	2,45949	-0,657154	0,00627136	-0,445931	-0,667205
0,191048	NaN	-0,401569	1,92849	0,00836182	2,45949	-0,657154	0,00627136	-0,445931	-0,667205
-1,46922	NaN	-0,589941	1,24058	-1,83118	1,96633	0,327166	-1,13336	-1,26438	-0,670014
-1,46922	NaN	-0,589941	1,24058	-1,83118	1,96633	0,327166	-1,13336	-1,26438	-0,670014
-1,12601	-0,568382	-0,982325	NaN	NaN	NaN	NaN	-1,41075	-1,59375	-0,87469
-1,12601	-0,568382	-0,982325	NaN	NaN	NaN	NaN	-1,41075	-1,59375	-0,87469
NaN	-2,40133	NaN	NaN	NaN	NaN	NaN	-2,37427	-2,20858	-1,8362
-1,13035	-0,475285	-1,11308	0,522036	0,332121	-0,943827	0,703506	-0,664789	-0,398526	-0,80071
-1,13035	-0,475285	-1,11308	0,522036	0,332121	-0,943827	0,703506	-0,664789	-0,398526	-0,80071
4,56297	3,14687	3,69286	2,13259	4,56253	-0,244104	3,15143	3,74295	3,77917	3,25887
4,56297	3,14687	3,69286	2,13259	4,56253	-0,244104	3,15143	3,74295	3,77917	3,25887
NaN	NaN	-0,449884	NaN	-2,01731	NaN	NaN	1,05575	0,522836	1,4738
NaN	NaN	-0,449884	NaN	-2,01731	NaN	NaN	1,05575	0,522836	1,4738
NaN	NaN	NaN	NaN	NaN	NaN	NaN	-2,07392	-2,50364	-2,10521

Polito et al. Universal ready-to-use immunotherapeutic approach for the treatment of cancer: expanded and activated polyclonal γδ memory T cells.
Phosphorylation sites

NaN	NaN	NaN	NaN	NaN	NaN	NaN	-2,07392	-2,50364	-2,10521
NaN	NaN	NaN	NaN	NaN	NaN	NaN	NaN	NaN	NaN
1,01673	1,38459	-0,222855	NaN	0,463543	2,23418	NaN	0,24975	NaN	NaN
-0,494631	-3,52635	-0,782026	-1,8429	-0,215948	-0,661272	-1,8858	-0,287693	0,260795	0,155746
1,44672	0,784151	2,34149	1,78944	2,06325	1,31643	2,25229	1,56459	1,23065	1,25462
1,44672	0,784151	2,34149	1,78944	2,06325	1,31643	2,25229	1,56459	1,23065	1,25462
0,926252	1,52778	0,779716	1,53122	0,17938	NaN	1,26751	0,878792	0,9894	1,21553
0,284979	-0,814089	0,589132	NaN	-0,732689	3,81958	3,86691	1,2889	2,01854	1,38318
0,862741	-0,708746	0,822893	4,56996	-0,482864	4,8357	3,46885	1,32529	0,819797	0,62177
NaN	NaN	NaN	1,91188	NaN	0,646391	-0,1639	-1,4863	-1,09513	NaN
-1,35834	NaN	1,34207	3,28638	-1,53753	3,45223	1,25576	NaN	NaN	NaN
-1,35834	NaN	1,34207	3,28638	-1,53753	3,45223	1,25576	NaN	NaN	NaN
-0,191103	NaN	-1,41242	-2,55414	-2,3516	-1,20546	-2,19143	-1,10959	-1,03628	-2,78557
NaN	NaN	NaN	-0,604198	-0,387728	NaN	-0,711374	NaN	NaN	NaN
0,530064	-0,0986519	0,263147	1,80861	0,186993	-3,10545	0,97047	1,11649	0,651788	0,367131
0,530064	-0,0986519	0,263147	1,80861	0,186993	-3,10545	0,97047	1,11649	0,651788	0,367131
-1,5785	-1,61336	-1,812	-0,677626	-2,15425	-1,18739	-1,53239	-1,52526	-1,71166	-0,163085
NaN	NaN	NaN	NaN	NaN	NaN	NaN	NaN	NaN	NaN
NaN	NaN	NaN	-0,256575	NaN	0,712675	-1,05688	NaN	NaN	NaN
NaN	NaN	NaN	0,914757	NaN	NaN	NaN	NaN	NaN	NaN
-3,95273	NaN	-3,02247	NaN	-3,8426	-2,24385	-2,52285	-3,22651	-2,85345	-3,22887
-3,95273	NaN	-3,02247	NaN	-3,8426	-2,24385	-2,52285	-3,22651	-2,85345	-3,22887
1,70012	1,70881	0,943413	1,74335	NaN	NaN	1,10413	0,906866	0,609265	0,753316
1,70012	1,70881	0,943413	1,74335	NaN	NaN	1,10413	0,906866	0,609265	0,753316
NaN	NaN	NaN	NaN	NaN	NaN	NaN	NaN	NaN	NaN
NaN	NaN	NaN	NaN	NaN	NaN	NaN	NaN	NaN	NaN
-1,94053	-3,55771	-1,72598	2,2632	-2,5165	0,407017	1,21302	NaN	NaN	NaN
-1,94053	-3,55771	-1,72598	2,2632	-2,5165	0,407017	1,21302	NaN	NaN	NaN
NaN	NaN	NaN	-1,51368	-1,81668	-2,02551	-2,15594	NaN	NaN	NaN
-1,81829	-2,54813	-1,32486	0,0238113	-0,331514	-0,278982	-1,03726	NaN	NaN	NaN
-1,81829	-2,54813	-1,32486	0,0238113	-0,331514	-0,278982	-1,03726	-1,49274	NaN	-2,68289
NaN	NaN	NaN	1,09578	NaN	-0,00456047	-1,68326	NaN	NaN	NaN
NaN	NaN	NaN	0,642096	-1,70225	2,8316	0,0462685	-2,02448	-2,63379	-0,995452
NaN	NaN	NaN	0,642096	-1,70225	2,8316	NaN	NaN	NaN	-0,995452

Polito et al. Universal ready-to-use immunotherapeutic approach for the treatment of cancer: expanded and activated polyclonal γδ memory T cells.
Phosphorylation sites

NaN	NaN	NaN	0,916607	NaN	0,715452	NaN	NaN	NaN	NaN
NaN	NaN	NaN	3,4322	NaN	3,91815	0,7876	NaN	NaN	NaN
NaN	NaN	NaN	3,4322	NaN	3,91815	0,7876	NaN	NaN	NaN
NaN	NaN	NaN	NaN	NaN	NaN	NaN	-0,505823	-0,531533	-1,23873
NaN	NaN	NaN	NaN	NaN	NaN	NaN	-0,505823	-0,531533	-1,23873
-2,32072	0,109577	NaN	NaN	NaN	NaN	NaN	0,0153332	0,301318	0,705424
-2,32072	0,109577	NaN	NaN	NaN	NaN	NaN	0,0153332	0,301318	0,705424
NaN	-0,829205	-0,60947	-1,6233	0,485764	NaN	-0,547398	-0,951706	-1,25363	-0,218587
2,4982	0,472095	1,24963	0,0545616	1,09221	NaN	0,980358	1,52856	1,21191	0,875552
-0,0265827	0,312033	-1,28919	-0,684198	NaN	NaN	-0,204834	NaN	NaN	NaN
-0,870668	0,312033	-1,28919	-0,684198	NaN	NaN	-0,204834	NaN	NaN	NaN
NaN	NaN	NaN	-0,167212	NaN	-1,63738	1,6107	NaN	NaN	NaN
NaN	NaN	NaN	-0,167212	NaN	-1,63738	1,6107	NaN	NaN	NaN
-0,912365	NaN	-0,997353	-1,38881	-0,717834	-0,98358	-1,44073	-1,08409	-1,66577	-1,68976
-0,912365	NaN	-0,997353	-1,38881	-0,717834	NaN	-1,44073	-1,08409	-1,66577	-1,68976
NaN	NaN	NaN	-2,50218	-2,20434	-0,680542	NaN	NaN	NaN	NaN
-0,186701	-2,07812	-1,47586	-1,73451	-1,26817	-1,829	-1,6839	-1,42213	-1,49177	-1,4962
-0,186701	-0,980089	-1,47586	NaN	-0,445641	-1,829	NaN	-1,09817	NaN	-1,4962
0,433792	0,797747	-0,382019	0,0129204	0,689936	NaN	1,71988	-0,185307	-0,426093	-0,244481
2,17526	0,526642	2,14415	1,57439	2,435	1,45558	1,46952	1,57258	1,6998	1,31444
0,721931	0,797747	-0,146236	0,0129204	0,789358	-0,979652	1,71988	-0,185307	-0,426093	-0,244481
2,17526	NaN	2,14415	1,57439	NaN	1,45558	NaN	1,57258	1,6998	NaN
1,19519	-0,244095	0,527472	-0,0135174	0,663055	-0,0912647	NaN	0,0764084	-0,0667601	NaN
NaN	NaN	NaN	NaN	0,663055	-0,0912647	0,0819035	NaN	NaN	NaN
NaN	-0,0751247	-0,91543	NaN	0,71376	NaN	NaN	0,57387	0,0804281	-0,375564
NaN	NaN	NaN	NaN	NaN	NaN	NaN	NaN	NaN	NaN
-0,317577	-0,152819	-0,45171	0,933838	0,892742	-0,848543	-0,612804	-0,53462	-0,806678	-1,31884
-0,317577	-0,152819	-0,45171	0,933838	0,892742	-0,848543	-0,612804	-0,53462	-0,806678	-1,31884
-0,151543	0,326143	0,626272	-0,135426	2,76591	NaN	1,54521	NaN	NaN	NaN
-0,151543	0,326143	0,626272	-0,135426	2,76591	NaN	1,54521	NaN	NaN	NaN
0,77442	1,26931	-0,245869	NaN	NaN	NaN	NaN	-0,0920715	0,906596	0,471629
0,77442	1,26931	-0,245869	NaN	NaN	NaN	NaN	-0,0920715	0,906596	0,471629
NaN	NaN	NaN	-0,270697	NaN	0,810211	-0,562334	NaN	NaN	NaN
NaN	NaN	NaN	-0,270697	NaN	0,810211	-0,562334	NaN	NaN	NaN

Polito et al. Universal ready-to-use immunotherapeutic approach for the treatment of cancer: expanded and activated polyclonal γδ memory T cells.
Phosphorylation sites

NaN	NaN	NaN	NaN	NaN	NaN	NaN	-1,27201	-1,09693	0,633903
NaN	NaN	NaN	NaN	NaN	NaN	NaN	-1,27201	-1,09693	0,633903
NaN	NaN	NaN	NaN	NaN	NaN	NaN	-1,42347	-1,35565	-1,61996
0,616808	1,29639	0,602596	1,05699	0,893967	1,14377	1,79212	0,914164	0,836087	1,35052
0,616808	1,29639	0,602596	1,05699	0,893967	1,14377	1,79212	0,914164	0,836087	1,35052
NaN	-0,0180283	-1,87226	-2,45425	NaN	-1,25699	-1,47946	NaN	NaN	-1,26988
NaN	-0,0180283	-1,87226	-2,45425	NaN	-1,25699	-1,47946	NaN	NaN	-1,26988
0,616808	1,29639	0,602596	1,05699	0,893967	1,14377	1,79212	0,914164	0,836087	1,35052
0,616808	1,29639	0,602596	1,05699	0,893967	1,14377	1,79212	0,914164	0,836087	1,35052
0,0445919	NaN	NaN	0,976315	-0,546057	-2,49507	0,440567	-0,481312	-0,220473	-0,853429
0,0445919	-2,80767	NaN	0,976315	-0,546057	-2,49507	0,440567	-0,481312	-0,220473	-0,853429
NaN	NaN	1,28398	2,29927	-1,58946	2,23899	2,83816	NaN	NaN	NaN
NaN	NaN	1,28398	2,29927	-1,58946	2,23899	2,83816	NaN	NaN	NaN
NaN	NaN	NaN	NaN	-2,82825	1,50544	-2,38772	NaN	NaN	NaN
NaN	NaN	NaN	1,19731	-2,82825	1,50544	-2,38772	-1,56367	-1,72285	-1,85958
-0,723143	-0,971266	0,111757	-2,90007	-0,101496	NaN	-1,03008	-2,33209	-1,76238	NaN
-1,63215	-0,971266	-1,50017	NaN	NaN	NaN	NaN	NaN	NaN	NaN
5,12924	4,96173	4,64303	4,17584	3,63589	3,97763	4,65858	6,00396	5,63272	6,83868
5,02328	4,96173	4,91521	5,27441	4,56566	6,20004	4,85738	6,00396	5,63272	6,83868
5,02328	4,40422	4,91521	5,27441	4,56566	6,20004	4,85738	4,99068	5,01749	5,24679
-0,899525	-2,77116	1,06064	0,681025	-1,46682	0,924606	0,403912	0,447348	0,193177	0,0377913
-0,899525	-2,77116	1,06064	0,681025	-1,46682	0,924606	0,403912	0,447348	0,193177	0,0377913
-0,289698	NaN	-1,26979	-0,998695	1,36349	0,274197	-0,106594	-1,9407	-1,9728	-0,87242
-0,289698	NaN	-1,26979	-0,998695	1,36349	0,274197	-0,106594	-1,9407	-1,9728	-0,87242
1,21945	NaN	-1,04545	0,739777	0,270096	-0,301254	-0,227339	NaN	NaN	NaN
NaN	NaN	NaN	NaN	NaN	NaN	NaN	-2,23133	-1,22795	-1,22418
NaN	NaN	NaN	NaN	NaN	NaN	NaN	-2,23133	-1,22795	-1,22418
-0,156858	-0,226929	-0,338032	0,724504	0,0397358	1,74271	0,0156708	-1,22437	-1,14229	-1,422
-0,156858	-0,226929	-0,338032	0,724504	0,0397358	1,74271	0,0156708	-1,22437	-1,14229	-1,422
NaN	NaN	NaN	-0,772974	-0,905766	NaN	-0,752913	-1,1751	-0,4325	-3,8382
-2,04959	NaN	-1,57033	-1,3854	-1,41958	NaN	-1,96403	-1,95494	-1,51175	-1,50145
-2,04959	NaN	-1,57033	-1,3854	-1,41958	NaN	-1,96403	-1,95494	-1,51175	-1,50145
NaN	NaN	NaN	NaN	NaN	NaN	NaN	-2,51617	-2,23962	-3,13009
NaN	NaN	0,0854492	NaN	NaN	-3,28665	1,60784	NaN	NaN	NaN

Polito et al. Universal ready-to-use immunotherapeutic approach for the treatment of cancer: expanded and activated polyclonal γδ memory T cells.
Phosphorylation sites

NaN	NaN	0,0854492	NaN	NaN	-3,28665	1,60784	NaN	NaN	NaN
-0,0499134	-0,629103	0,809652	0,915926	0,979353	0,244158	0,494431	0,619539	1,44475	1,26019
NaN	NaN	NaN	NaN	NaN	NaN	NaN	NaN	NaN	NaN
2,74459	1,83796	2,33356	3,05608	2,27937	3,31376	1,8376	1,47196	1,60516	1,87518
2,5118	1,1874	1,48712	0,886696	-0,150705	1,24669	-0,706224	0,810888	1,50878	-0,676528
2,5118	1,1874	1,48712	0,886696	-0,150705	1,24669	-0,706224	0,810888	1,50878	-0,676528
-1,25043	0,122414	-0,997141	NaN	-0,0500469	-3,51527	0,414495	NaN	NaN	NaN
-1,25043	0,122414	-0,997141	-0,969425	-0,0500469	-3,51527	0,414495	-0,194324	-0,383359	-0,470084
-0,713009	-0,403633	-0,665119	NaN	NaN	NaN	NaN	-0,135386	-3,7201	-1,44288
1,57587	2,39795	1,91109	3,01898	1,34436	3,21268	3,21372	NaN	0,603337	1,57786
1,57587	2,39795	1,91109	3,01898	1,34436	3,21268	3,21372	0,65379	1,16886	NaN
-1,25043	0,122414	-0,997141	NaN	-0,0500469	-3,51527	0,414495	NaN	NaN	NaN
-1,25043	0,122414	-0,997141	-0,969425	-0,0500469	-3,51527	0,414495	-0,194324	-0,383359	-0,470084
-0,713009	-0,403633	-0,665119	NaN	NaN	NaN	NaN	-0,135386	-3,7201	-1,44288
-1,12358	NaN	-1,52101	NaN	-1,06344	NaN	NaN	-1,42273	-1,63972	-1,1275
-1,12358	NaN	-1,52101	NaN	-1,06344	NaN	NaN	-1,42273	-1,63972	-1,1275
0,0146999	1,72214	2,54354	2,93374	4,15064	3,14018	2,75513	2,69056	3,22313	3,47926
0,0146999	1,72214	2,54354	2,93374	4,15064	3,14018	2,75513	-0,549437	3,22313	3,47926
0,381378	-0,384573	-0,0184307	0,25456	0,24534	-1,21923	-0,208729	0,182878	-0,0313272	0,710202
NaN	NaN	0,0905743	NaN	NaN	NaN	NaN	0,594055	0,686608	1,07609
NaN	NaN	NaN	NaN	NaN	NaN	NaN	NaN	NaN	NaN
NaN	NaN	NaN	NaN	NaN	NaN	NaN	NaN	NaN	NaN
NaN	NaN	NaN	-1,16961	-0,848431	-1,63062	-1,51217	NaN	NaN	NaN
1,21561	1,39837	0,706398	1,1643	2,16737	NaN	1,63616	1,30978	0,176942	1,28978
1,51477	1,71176	1,43704	1,25505	2,5061	NaN	NaN	NaN	1,58404	1,76767
1,51477	1,71176	1,43704	1,25505	2,5061	NaN	1,7111	1,70562	1,58404	1,76767
0,58737	1,05825	0,74864	0,488625	1,33987	-0,791199	0,649824	1,00346	0,91162	0,80375
0,58737	1,05825	0,74864	0,488625	1,33987	-0,791199	0,649824	1,00346	0,91162	0,80375
-0,0191212	-0,0059166	-0,846806	-0,284512	-1,38866	-1,54549	-1,38866	-0,334101	NaN	-1,34065
-0,0191212	-0,0059166	-0,846806	-0,284512	-1,38866	-1,54549	-1,38866	-0,334101	NaN	-1,34065
-0,91186	-0,494469	-1,98833	-1,33746	-0,371857	-1,68977	-1,40502	NaN	NaN	NaN
-0,91186	-0,494469	-1,98833	-1,33746	-0,371857	-1,68977	-1,40502	NaN	NaN	NaN
1,61567	1,21229	1,31191	0,544392	1,43179	1,1968	0,40646	0,901936	0,0398664	NaN
-2,32441	1,21229	1,31191	NaN	1,43179	1,1968	NaN	NaN	NaN	0,310151

NaN	NaN	NaN	-1,29242	NaN	-0,998083	-1,60578	NaN	NaN	NaN
NaN	NaN	NaN	-1,29242	NaN	-0,998083	-1,60578	NaN	NaN	NaN
NaN	NaN	NaN	1,26557	NaN	0,581564	-2,17488	NaN	0,934657	NaN
-0,19276	-0,882029	1,10063	1,26557	NaN	0,581564	1,15108	NaN	NaN	NaN
NaN	NaN	NaN	NaN	NaN	NaN	-0,576029	0,61484	NaN	1,05271
NaN	NaN	NaN	NaN	NaN	NaN	NaN	NaN	NaN	NaN
NaN	NaN	NaN	NaN	NaN	NaN	NaN	NaN	NaN	NaN
NaN	NaN	NaN	-0,526308	NaN	-0,469688	-0,380953	NaN	NaN	NaN
3,85938	3,12267	3,73369	3,70075	3,99286	2,75965	3,97801	3,98614	3,89484	3,68623
3,85938	3,12267	3,73369	3,70075	3,99286	2,75965	3,97801	3,98614	3,89484	3,68623
NaN	-1,39312	NaN	-1,2779	-1,08783	-0,584244	-0,111584	-1,65686	-1,0521	-2,13928
NaN	-1,39312	NaN	-1,2779	-1,08783	-0,584244	-0,111584	-1,65686	-1,0521	-2,13928
NaN	NaN	NaN	NaN	NaN	NaN	NaN	0,442472	-0,195928	0,647754
NaN	NaN	NaN	0,740326	0,236849	-1,89784	NaN	0,442472	-0,195928	0,647754
NaN	NaN	NaN	-1,36532	NaN	-0,160429	-0,419678	NaN	NaN	NaN
NaN	NaN	NaN	NaN	NaN	NaN	NaN	NaN	NaN	NaN
NaN	NaN	NaN	NaN	NaN	NaN	NaN	NaN	NaN	NaN
NaN	NaN	NaN	NaN	NaN	NaN	NaN	NaN	NaN	NaN
NaN	NaN	NaN	NaN	NaN	NaN	NaN	NaN	NaN	NaN
-0,477188	-0,975149	-1,21626	-1,44271	-0,652914	-1,03054	-1,14532	0,264484	0,37642	0,761462
-0,477188	-0,975149	-1,21626	-1,44271	-0,652914	-1,03054	-1,14532	0,264484	0,37642	0,761462
0,771284	-0,20005	0,565838	NaN	0,422997	NaN	-1,85023	NaN	1,43439	0,400165
0,771284	-0,20005	0,565838	NaN	0,422997	NaN	-1,85023	1,55138	1,43439	0,400165
0,771284	-0,20005	0,565838	NaN	0,422997	NaN	-1,85023	1,55138	1,43439	0,400165
1,99399	1,42379	1,22554	2,0055	-0,187889	3,11606	1,47234	1,20072	1,56697	0,42323
1,15024	0,834641	-0,0847683	2,28497	0,429325	1,43689	1,15469	0,0784187	0,732385	0,105481
1,15024	0,834641	-0,0847683	2,28497	0,429325	1,43689	1,15469	0,0784187	0,732385	0,105481
0,856592	-0,806795	0,474497	0,0401173	0,274475	-0,810467	0,0416737	1,14857	1,42831	NaN
0,856592	-0,806795	0,474497	0,0401173	0,274475	-0,810467	0,0416737	1,14857	1,42831	NaN
-1,19567	-1,47476	-1,3148	-1,58353	-1,15285	-2,35104	-1,41505	-1,20412	-0,780337	-1,47582
-1,19567	-1,47476	-1,3148	-1,58353	-1,15285	-2,35104	-1,41505	-1,20412	-0,780337	-1,47582
-0,79096	-0,834585	-1,69902	NaN	NaN	NaN	NaN	-0,221897	-1,8995	-2,15944
-0,776779	-1,73591	-1,48984	NaN	NaN	NaN	NaN	-1,94674	-0,457994	-1,66059
-1,41691	-1,32648	-1,03938	NaN	-1,20493	NaN	NaN	-0,478426	-0,614415	-1,21803

Polito et al. Universal ready-to-use immunotherapeutic approach for the treatment of cancer: expanded and activated polyclonal γδ memory T cells.
Phosphorylation sites

-1,41691	-1,32648	-1,03938	NaN	-1,20493	NaN	NaN	-0,478426	-0,614415	-1,21803
NaN	NaN	NaN	-0,174681	-1,97132	0,73243	-1,33874	NaN	NaN	NaN
NaN	NaN	NaN	-0,174681	-1,97132	0,73243	-1,33874	NaN	NaN	NaN
2,68006	4,71526	3,30597	3,15973	1,47723	-1,28551	2,46758	2,17703	2,78674	3,17588
2,68006	4,71526	3,30597	3,15973	1,47723	-1,28551	2,46758	2,17703	2,78674	3,17588
NaN	NaN	NaN	NaN	NaN	NaN	NaN	NaN	NaN	NaN
NaN	NaN	NaN	NaN	NaN	NaN	NaN	NaN	NaN	NaN
NaN	NaN	NaN	0,912317	-2,07532	0,68096	-0,584883	NaN	NaN	NaN
NaN	NaN	NaN	0,912317	-2,07532	0,68096	-0,584883	NaN	NaN	NaN
-0,872181	-2,12593	-1,42142	NaN	NaN	NaN	NaN	-1,54514	-1,87492	-1,82323
NaN	-2,12593	-1,42142	NaN	-1,40107	NaN	NaN	-1,54514	-1,87492	-1,82323
-0,872181	-2,12593	-1,42142	NaN	-1,40107	NaN	NaN	-1,54514	-1,87492	-1,82323
NaN	NaN	NaN	-1,24851	-1,22094	-0,187193	-0,296328	NaN	NaN	NaN
NaN	NaN	NaN	-1,14716	NaN	-2,25811	-2,66387	NaN	NaN	NaN
NaN	NaN	NaN	-1,14716	NaN	-2,25811	-2,66387	NaN	NaN	NaN
2,79061	1,99009	1,505	2,16885	1,67085	1,40652	0,747564	1,93532	1,28443	0,860085
2,79061	1,99009	1,505	2,16885	1,67085	1,40652	0,747564	1,93532	1,28443	0,860085
-0,587263	0,485174	NaN	-0,650249	NaN	3,15398	-0,0259762	NaN	NaN	NaN
-0,587263	0,485174	NaN	-0,650249	NaN	3,15398	-0,0259762	NaN	NaN	NaN
NaN	NaN	NaN	NaN	NaN	NaN	NaN	-1,53188	-1,23368	-2,09106
NaN	NaN	NaN	-0,268061	NaN	-0,857309	0,466934	NaN	NaN	NaN
NaN	NaN	NaN	-0,268061	-0,995317	-0,857309	0,466934	NaN	NaN	NaN
NaN	NaN	NaN	-0,773697	NaN	1,45256	-1,85233	NaN	NaN	NaN
NaN	NaN	NaN	-0,773697	NaN	1,45256	-1,85233	NaN	NaN	NaN
NaN	NaN	NaN	0,0551109	NaN	-1,35548	0,332483	NaN	NaN	NaN
NaN	NaN	NaN	1,45576	NaN	-0,437801	-0,237419	NaN	NaN	NaN
NaN	NaN	NaN	1,45576	NaN	-0,437801	-0,237419	NaN	NaN	NaN
NaN	NaN	NaN	NaN	NaN	NaN	NaN	NaN	NaN	NaN
-0,554802	NaN	-1,80769	NaN	-0,385534	0,767204	NaN	NaN	NaN	NaN
NaN	1,57618	NaN	NaN	NaN	NaN	NaN	NaN	NaN	NaN
3,35899	3,22157	2,66799	3,28977	0,855326	2,41036	3,20514	1,93519	3,04865	1,75515
3,35899	3,22157	2,66799	3,28977	0,855326	2,41036	3,20514	1,93519	3,04865	1,75515
-0,574677	NaN	1,51089	0,887789	NaN	NaN	1,12662	NaN	NaN	NaN
-0,574677	NaN	1,51089	0,887789	NaN	NaN	1,12662	NaN	NaN	NaN

Polito et al. Universal ready-to-use immunotherapeutic approach for the treatment of cancer: expanded and activated polyclonal γδ memory T cells.
Phosphorylation sites

NaN	-1,57619	0,168797	-0,171366	0,754847	-1,24291	0,528929	NaN	-0,706759	NaN
NaN	-1,57619	0,168797	-0,171366	0,754847	-1,24291	0,528929	NaN	-0,706759	NaN
NaN	NaN	NaN	NaN	NaN	NaN	NaN	NaN	NaN	NaN
5,00699	7,6459	NaN	6,02641	5,32899	6,23613	6,292	5,36478	6,43433	5,59765
5,00699	7,6459	NaN	6,02641	5,32899	6,23613	6,292	5,36478	6,43433	5,59765
-1,42528	-1,25484	-1,37268	1,49418	-0,919359	-1,50235	1,48804	NaN	NaN	NaN
-1,42528	-1,25484	-1,37268	1,49418	-0,919359	-1,50235	1,48804	NaN	NaN	NaN
1,17615	1,81046	-0,338379	0,631454	0,272852	2,29368	-0,0414848	NaN	NaN	NaN
3,06276	2,18908	1,8008	0,639029	2,68103	1,42583	1,55102	1,80155	2,10766	NaN
3,06276	2,18908	1,8008	0,639029	2,68103	1,42583	1,55102	1,80155	2,10766	0,275458
0,795662	-0,390974	0,971249	0,537317	0,179331	0,4685	0,0985298	0,449614	0,723096	0,275458
1,49027	-0,769138	0,593775	NaN	0,502296	NaN	NaN	1,36331	0,79069	0,493964
NaN	0,319168	NaN	-1,0215	NaN	NaN	NaN	NaN	NaN	NaN
2,26581	1,01543	0,966919	0,690054	1,87276	-0,7005	1,17245	1,48654	1,83729	0,99401
2,26581	1,01543	0,966919	0,690054	1,87276	-0,7005	1,17245	1,48654	1,83729	0,99401
0,513649	1,64409	-0,977224	-1,22053	0,451263	-0,590853	-0,0908012	-0,400471	-1,53397	-1,32786
1,21724	-2,26554	NaN	NaN	0,832691	2,15263	NaN	0,352427	-0,248168	0,159101
NaN	NaN	NaN	NaN	NaN	NaN	0,330832	NaN	NaN	NaN
NaN	NaN	NaN	NaN	NaN	NaN	0,330832	NaN	NaN	NaN
NaN	-1,17159	NaN	NaN	NaN	NaN	NaN	NaN	NaN	NaN
NaN	-1,17159	NaN	NaN	NaN	NaN	NaN	NaN	NaN	NaN
1,20461	0,753984	NaN	0,109251	0,447765	NaN	1,11336	0,319046	0,307197	0,234458
2,28214	1,96844	NaN	1,01421	2,37959	1,15619	1,78274	-0,462906	NaN	2,07113
2,28214	1,96844	0,345413	1,01421	2,37959	1,15619	1,78274	-0,462906	NaN	2,07113
NaN	NaN	NaN	0,447834	0,202198	NaN	0,916084	-2,22466	NaN	0,118434
NaN	-0,0216923	NaN	0,447834	0,202198	NaN	0,916084	-2,22466	NaN	0,118434
-0,663628	-1,16365	-0,914951	-0,529886	-0,626995	-1,1633	-0,7932	-1,13205	-1,41506	-0,922128
-0,663628	-1,16365	-0,914951	-0,529886	-0,626995	-1,1633	-0,7932	-1,13205	-1,41506	-0,922128
1,04156	0,00651169	0,83078	0,151436	1,45987	NaN	0,147327	1,3203	1,34945	0,671823
1,04156	0,00651169	0,83078	0,151436	1,45987	NaN	0,147327	1,3203	1,34945	0,671823
4,33524	4,13146	3,51818	4,03336	4,02349	NaN	3,01383	3,10102	0,862271	3,68378
2,04789	4,13146	1,17151	1,42823	4,02349	NaN	3,01383	3,10102	0,862271	3,68378
1,11533	-0,782608	-0,721357	-0,0350342	1,42747	NaN	0,238817	0,644852	1,05907	0,991242
1,11533	NaN	-1,01087	-0,595812	1,42747	NaN	0,238817	0,115406	0,673038	0,748954

Polito et al. Universal ready-to-use immunotherapeutic approach for the treatment of cancer: expanded and activated polyclonal γδ memory T cells.
Phosphorylation sites

-0,142841	-0,782608	-0,721357	-0,0350342	0,318163	NaN	-0,112961	0,644852	1,05907	0,991242
-1,46762	2,15076	2,63408	0,918652	3,0089	1,95422	0,243034	NaN	2,45642	3,75355
1,53602	1,71636	0,90793	0,416086	1,54846	0,868542	0,756588	0,872129	0,63247	0,524325
1,53602	1,71636	0,90793	0,416086	1,54846	0,868542	0,756588	0,872129	0,63247	0,524325
NaN	NaN	NaN	-0,576111	-0,393095	-0,604689	NaN	NaN	NaN	NaN
NaN	0,11014	NaN	-0,576111	-0,393095	-0,604689	NaN	NaN	NaN	NaN
NaN	NaN	NaN	NaN	NaN	NaN	NaN	-0,718897	-0,291991	-1,00036
NaN	NaN	NaN	NaN	NaN	NaN	NaN	-0,718897	-0,291991	-1,00036
0,572693	0,569788	0,527325	NaN	NaN	0,23604	NaN	0,678717	NaN	0,208417
1,72531	1,05353	1,20949	0,469236	1,58938	0,85277	0,211016	0,619165	0,446496	0,273807
-1,54392	-0,509626	-1,30581	-1,43404	-0,896103	NaN	-0,193571	-0,298296	-1,15781	-0,524964
-1,54392	-0,509626	NaN	-1,43404	-0,896103	NaN	-0,193571	-0,298296	NaN	-0,524964
NaN	NaN	NaN	-1,24459	-0,762814	-0,486984	-0,726456	NaN	NaN	NaN
NaN	NaN	NaN	-1,24459	-0,762814	-0,486984	-0,726456	NaN	NaN	NaN
-1,83699	NaN	-2,46049	-0,762957	-0,600716	NaN	-0,641035	NaN	NaN	NaN
-1,83699	NaN	-2,46049	-0,762957	-0,600716	NaN	-0,641035	NaN	NaN	NaN
0,356699	0,315668	NaN	0,0407085	-0,0608311	-0,588923	-1,01998	0,449961	0,568219	0,238685
NaN	NaN	NaN	NaN	NaN	NaN	NaN	NaN	NaN	NaN
NaN	NaN	NaN	NaN	NaN	NaN	NaN	NaN	NaN	NaN
NaN	NaN	NaN	-0,090519	NaN	1,92442	-1,62647	NaN	NaN	NaN
NaN	NaN	NaN	-0,33021	-0,895468	0,386904	NaN	NaN	NaN	NaN
-1,15813	NaN	-0,940638	-0,33021	-0,895468	0,386904	NaN	NaN	-0,412818	NaN
-2,82672	-2,30349	0,624348	NaN	NaN	NaN	NaN	NaN	-0,337392	NaN
-0,63954	-2,27426	-0,407602	-3,27004	-0,14469	NaN	-1,44802	-0,139692	-0,475795	0,111377
-0,63954	-2,27426	-0,407602	-3,27004	-0,14469	NaN	-1,44802	-0,139692	-0,475795	0,111377
NaN	NaN	NaN	0,411947	-0,878014	NaN	-0,403124	NaN	NaN	NaN
NaN	NaN	NaN	0,411947	-0,878014	NaN	-0,403124	NaN	NaN	NaN
-1,56411	-1,81494	NaN	-1,88044	-1,5086	-1,19291	NaN	0,632677	0,313434	0,148229
-1,56411	-1,81494	-0,404232	-1,88044	-1,5086	-1,19291	NaN	0,632677	0,313434	0,148229
NaN	NaN	NaN	-0,780279	NaN	0,15914	-1,35608	NaN	NaN	NaN
-1,03722	-1,08544	-2,09558	-2,61533	-1,46211	-1,69982	-2,31308	-1,85592	NaN	NaN
NaN	NaN	NaN	-1,58138	-1,04667	-1,10909	-1,35209	-1,85592	-2,09759	-1,88292
NaN	NaN	NaN	-0,335117	NaN	0,342134	-1,06785	NaN	NaN	NaN
NaN	NaN	NaN	2,49164	2,18785	3,24413	0,892563	0,974434	2,00021	0,578012

Polito et al. Universal ready-to-use immunotherapeutic approach for the treatment of cancer: expanded and activated polyclonal γδ memory T cells.
Phosphorylation sites

NaN	NaN	NaN	2,49164	2,18785	3,24413	0,892563	0,974434	2,00021	0,578012
4,79711	4,4854	3,21902	2,76928	3,81421	-0,16943	3,07449	3,83418	3,71615	3,79031
4,79711	4,4854	3,21902	2,76928	3,81421	-0,16943	3,07449	3,83418	3,71615	3,79031
NaN	NaN	NaN	NaN	-0,449751	-2,11124	-0,757324	-0,593355	-3,99923	NaN
NaN	NaN	NaN	-1,6497	-2,282	-2,66008	-0,143738	-1,92423	NaN	-1,14734
NaN	NaN	NaN	-1,6497	-2,282	-2,66008	-0,143738	NaN	NaN	NaN
0,09762	-0,918076	1,61206	0,593096	0,277035	0,42721	1,79683	-0,254019	-0,328815	-0,33702
0,09762	-0,918076	1,61206	0,593096	0,277035	0,42721	1,79683	-0,254019	-0,328815	-0,33702
NaN	NaN	NaN	0,923214	NaN	0,55636	0,241957	NaN	NaN	NaN
1,26623	1,6016	1,78741	2,55516	1,35206	1,52251	NaN	2,28946	1,67297	2,16464
-0,07551	-0,400566	NaN	-1,60099	-1,78156	0,607201	NaN	NaN	NaN	NaN
1,26623	1,6016	1,78741	2,55516	1,35206	1,52251	2,03023	2,28946	1,67297	2,16464
NaN	NaN	NaN	NaN	NaN	NaN	NaN	NaN	NaN	NaN
-0,801191	NaN	-0,72868	NaN	NaN	NaN	NaN	-1,69649	-0,765689	-1,10566
-1,68142	-1,66479	-1,38011	-1,10618	-1,69561	NaN	-1,31547	NaN	NaN	-1,29393
-1,68142	-1,66479	-1,38011	-1,10618	-1,69561	NaN	-1,31547	NaN	NaN	-1,29393
-1,63923	-1,74957	-0,10339	0,312136	NaN	-0,12145	1,49098	0,206091	NaN	-0,0732088
-1,63923	-1,74957	-0,10339	0,312136	-1,35525	-0,12145	1,49098	0,206091	0,116555	-0,0732088
-1,2129	-2,24975	-1,10184	NaN	NaN	NaN	NaN	-0,7437	NaN	-1,36326
-1,2129	-2,24975	-1,10184	NaN	NaN	NaN	NaN	NaN	NaN	NaN
-0,572584	NaN	-0,68693	-0,102764	-0,6206	-1,45667	0,423872	-0,510221	0,308986	0,32002
-0,572584	NaN	-0,68693	-0,102764	-0,6206	-1,45667	0,423872	-0,510221	0,308986	0,32002
2,86139	1,86498	1,92411	1,87592	2,44161	1,37235	2,50892	2,14367	1,5946	1,52461
2,86139	1,86498	1,92411	1,87592	2,44161	1,37235	2,50892	2,14367	1,5946	1,52461
-1,05677	NaN	-1,11818	NaN	-0,67263	-3,84159	-1,59393	NaN	-1,30932	NaN
-1,05677	NaN	-1,11818	NaN	-0,67263	-3,84159	-1,59393	NaN	NaN	NaN
-1,3151	0,974398	-2,21233	-0,466616	-0,987404	-0,0629482	-0,0316067	NaN	-1,53414	-0,85675
-1,3151	0,974398	-2,21233	-0,466616	-0,987404	-0,0629482	-0,0316067	NaN	-1,53414	-0,85675
-2,45673	NaN	-2,67764	-2,08524	-1,6158	-2,30058	-1,91567	NaN	NaN	NaN
-2,45673	NaN	-2,67764	-2,08524	-1,6158	-2,30058	-1,91567	NaN	NaN	NaN
-1,73933	-1,28622	-1,65791	-0,955704	NaN	-1,7739	-1,20141	-1,87409	NaN	-2,26645
NaN	-1,84552	-2,26159	-1,26282	NaN	-2,1721	-1,62757	NaN	NaN	NaN
0,998413	NaN	NaN	0,274834	NaN	-1,44877	-0,439543	NaN	NaN	NaN
0,998413	NaN	NaN	0,274834	NaN	-1,44877	-0,439543	NaN	NaN	NaN

Polito et al. Universal ready-to-use immunotherapeutic approach for the treatment of cancer: expanded and activated polyclonal γδ memory T cells.
Phosphorylation sites

NaN	NaN	NaN	NaN	NaN	NaN	NaN	NaN	NaN	NaN
NaN	NaN	NaN	NaN	NaN	NaN	NaN	NaN	NaN	NaN
NaN	NaN	NaN	0,0904331	-1,41056	-0,00528145	-1,09676	NaN	NaN	NaN
NaN	NaN	NaN	0,0294056	NaN	-0,332813	-0,421951	NaN	NaN	NaN
NaN	NaN	NaN	0,0294056	NaN	-0,332813	-0,421951	NaN	NaN	NaN
NaN	NaN	NaN	-0,265297	-1,6784	0,266048	-1,1924	NaN	NaN	NaN
NaN	NaN	NaN	-0,265297	-1,6784	0,266048	-1,1924	NaN	NaN	NaN
0,104322	1,35329	-0,121284	-0,375301	NaN	-0,313593	-1,03044	-1,08626	0,738421	-0,494254
0,104322	1,35329	-0,121284	-0,375301	NaN	-0,313593	-1,03044	-1,08626	0,738421	-0,494254
2,07521	2,86652	1,54746	NaN	NaN	NaN	NaN	2,09212	NaN	2,30128
NaN	NaN	NaN	-1,44216	NaN	NaN	-2,25145	NaN	NaN	NaN
-1,37979	-1,42084	NaN	-1,69326	-0,40856	-1,14585	-1,15335	-2,43664	NaN	-1,15739
NaN	NaN	NaN	-1,69326	-0,40856	-1,14585	-1,15335	-2,43664	NaN	-1,15739
1,44451	1,70852	0,214178	2,02685	0,934029	1,71238	2,03901	0,310926	0,355718	0,434159
1,44451	1,70852	0,214178	2,02685	0,934029	1,71238	2,03901	0,310926	0,355718	0,434159
2,94629	2,90839	1,72486	2,21198	1,90887	-0,413321	1,85938	1,5948	2,13111	1,50316
2,94629	2,90839	1,72486	2,21198	1,90887	-0,413321	1,85938	1,5948	2,13111	1,50316
-1,07368	NaN	NaN	-2,11177	0,801432	NaN	-1,55191	NaN	NaN	NaN
-1,07368	NaN	NaN	-2,11177	0,801432	NaN	-1,55191	NaN	NaN	NaN
NaN	NaN	NaN	0,482059	NaN	-1,9506	-0,95105	NaN	NaN	NaN
1,67356	2,23861	0,920414	1,76957	1,29631	1,97214	0,937712	0,823347	1,09004	0,992396
1,67356	2,23861	0,920414	1,76957	1,29631	1,97214	0,937712	0,823347	1,09004	0,992396
NaN	NaN	NaN	-0,569029	-0,937487	-0,806355	-0,576895	-1,49316	-1,40075	-0,78457
-1,09602	NaN	NaN	-1,88526	-0,963421	NaN	-1,64662	-1,1669	-2,0166	-1,75751
-1,09602	NaN	NaN	-1,88526	-0,963421	NaN	-1,64662	-1,1669	-2,0166	-1,75751
0,998749	1,17373	0,299374	0,819643	NaN	1,30643	0,438292	1,25189	1,78546	NaN
0,998749	1,17373	0,299374	0,819643	NaN	1,30643	0,438292	1,25189	1,78546	0,960084
1,39801	-0,144823	1,43343	1,28463	0,97648	1,68208	1,25679	1,28676	1,42097	0,289496
1,39801	-0,144823	1,43343	1,28463	0,97648	1,68208	1,25679	1,28676	1,42097	0,289496
1,43653	-0,25746	0,322763	1,27616	1,37636	1,06009	1,25342	0,702961	0,892966	-0,0106058
1,43653	-0,25746	0,322763	1,27616	1,37636	1,06009	1,25342	0,702961	0,892966	-0,0106058
0,279968	NaN	-0,432446	NaN	-0,183783	1,05986	0,905863	1,24549	-0,0759401	0,821908
0,279968	NaN	-0,432446	NaN	NaN	1,05986	0,905863	1,24549	-0,0759401	0,821908
NaN	NaN	NaN	-2,37726	NaN	-3,14529	-1,22687	NaN	NaN	NaN

Polito et al. Universal ready-to-use immunotherapeutic approach for the treatment of cancer: expanded and activated polyclonal γδ memory T cells.
Phosphorylation sites

3,53613	3,18466	3,42822	2,58095	4,06221	0,0862865	3,33773	3,4959	3,59037	3,314
3,53613	3,18466	3,42822	2,58095	4,06221	0,0862865	3,33773	3,4959	3,59037	3,314
NaN	3,20341	0,912216	NaN	1,75858	2,84255	1,63342	0,473989	0,143317	0,779222
NaN	3,20341	0,912216	NaN	1,75858	2,84255	1,63342	0,473989	0,143317	0,779222
NaN	NaN	NaN	-1,17524	-1,01886	NaN	-1,12042	NaN	NaN	NaN
4,24724	5,10558	4,31762	4,80458	4,69379	NaN	4,9392	3,30683	4,68165	4,22903
4,24724	5,10558	4,31762	4,80458	4,69379	NaN	4,9392	3,30683	4,68165	4,22903
-1,45056	1,04306	-0,832014	-0,771372	0,43594	-0,749523	-0,0936356	-1,3277	-1,53302	-0,46729
-1,45056	1,04306	-0,832014	-0,771372	0,43594	-0,749523	-0,0936356	-1,3277	-1,53302	-0,46729
-0,283491	1,15999	-0,789186	-1,22534	-1,82906	-1,17305	-0,212477	-1,93955	-0,36925	-1,88216
-0,283491	1,15999	-0,789186	-1,22534	-1,82906	-1,17305	-0,212477	-1,93955	-0,36925	-1,88216
0,444345	0,27582	0,453568	0,202427	0,183743	0,620045	0,740358	1,31229	0,935193	0,518025
0,444345	0,27582	0,453568	0,202427	0,183743	0,620045	0,740358	1,31229	0,935193	0,518025
1,24623	0,932241	0,715849	NaN	2,08827	0,522556	-0,139891	0,86981	0,259578	1,61901
1,24623	0,932241	-1,02763	NaN	2,08827	0,522556	-0,139891	0,86981	0,259578	1,61901
NaN	NaN	NaN	NaN	NaN	NaN	NaN	NaN	NaN	NaN
NaN	NaN	-1,82809	-2,19324	NaN	-1,94201	-1,21618	NaN	NaN	NaN
NaN	NaN	-1,82809	-2,19324	NaN	-1,94201	-1,21618	NaN	NaN	NaN
-1,75801	-0,0917301	-1,77092	-1,44081	-1,15634	0,408913	-0,980995	-1,42467	-1,9235	-1,62781
-1,75801	-0,0917301	-1,77092	-1,44081	-1,15634	0,408913	-0,980995	-1,42467	-1,9235	-1,62781
NaN	0,619711	-0,838518	1,35005	0,549238	1,55489	-0,694929	0,915354	0,641702	0,438636
1,12344	0,619711	-0,838518	1,35005	0,549238	1,66499	0,76437	0,915354	0,641702	0,438636
1,12344	NaN	NaN	0,303612	0,90099	-0,568056	0,76437	-1,0569	NaN	-1,17428
NaN	NaN	-0,661829	NaN	-0,936289	-0,664478	NaN	0,532446	-0,190675	-0,220378
-1,36718	-1,97825	-0,661829	NaN	-0,936289	-0,664478	NaN	NaN	-0,190675	NaN
NaN	0,204336	0,587582	-0,0916538	0,872681	-0,206976	0,403725	0,713465	0,494466	0,515956
NaN	0,204336	0,587582	-0,0916538	0,872681	-0,206976	0,403725	0,713465	0,494466	0,515956
NaN	NaN	NaN	NaN	NaN	NaN	NaN	NaN	NaN	NaN
-2,2186	-1,17058	-0,344099	-2,04479	-1,42714	-2,01438	0,710352	-0,512106	-0,622506	-0,451669
3,73346	3,43789	2,9654	NaN	3,39607	NaN	NaN	3,54214	2,91888	2,36084
3,73346	3,43789	2,9654	NaN	3,39607	0,343458	NaN	3,54214	2,91888	2,36084
NaN	NaN	NaN	-0,106045	NaN	0,242842	-0,229616	NaN	NaN	NaN
1,29189	1,48267	1,11107	1,27076	1,23143	1,19004	1,00518	1,51815	1,11969	0,692277
1,29189	1,48267	1,11107	1,27076	1,23143	1,19004	1,00518	1,51815	1,11969	0,692277

NaN	NaN	NaN	NaN	NaN	NaN	NaN	0,501699	0,0417204	-0,179807
NaN	NaN	NaN	NaN	NaN	NaN	NaN	0,501699	0,0417204	-0,179807
NaN	NaN	NaN	NaN	0,657425	NaN	NaN	-0,217455	-0,160356	0,554782
1,76263	1,33364	1,00755	0,738455	NaN	-0,102028	NaN	1,65599	NaN	1,88204
NaN	NaN	NaN	-0,733011	-2,10229	NaN	-1,53582	NaN	NaN	NaN
NaN	NaN	NaN	2,20506	1,92775	2,05862	1,04428	NaN	NaN	NaN
NaN	NaN	NaN	2,20506	1,92775	2,05862	1,04428	NaN	NaN	NaN
-2,28406	-0,320862	NaN	NaN	NaN	NaN	NaN	1,53382	2,14142	0,856795
1,67027	0,974634	0,721903	0,417049	1,73332	1,9042	0,186756	0,898727	0,683164	1,8945
1,67027	0,974634	0,721903	0,417049	1,73332	1,9042	0,186756	0,898727	0,683164	1,8945
NaN	0,381124	NaN	-0,789465	1,37647	NaN	-0,214758	NaN	NaN	NaN
NaN	0,381124	NaN	-0,789465	1,37647	NaN	-0,214758	NaN	NaN	NaN
NaN	NaN	NaN	NaN	NaN	NaN	NaN	NaN	NaN	NaN
NaN	NaN	NaN	-1,26155	-0,808294	-2,14291	0,107828	NaN	NaN	NaN
NaN	NaN	NaN	-1,26155	-0,808294	-2,14291	0,107828	NaN	NaN	NaN
NaN	1,25103	-0,338915	-0,821495	-0,824768	NaN	NaN	0,248259	-0,0877466	-0,436465
NaN	1,25103	-0,338915	-0,821495	-0,824768	NaN	NaN	0,248259	-0,0877466	-0,436465
-0,411531	-0,289221	0,0946884	0,246527	NaN	1,14235	0,0355606	NaN	NaN	NaN
-1,01933	0,281855	-1,36017	NaN	NaN	NaN	NaN	NaN	NaN	NaN
0,348045	0,320789	-0,544374	-0,0947723	-0,0483112	0,445629	NaN	-0,878069	-0,725289	0,151435
NaN	1,84773	-0,662231	0,337626	NaN	0,681484	0,494003	NaN	0,953525	NaN
1,68998	0,586794	1,43021	0,486265	1,25319	1,06284	0,744633	0,478455	1,22038	NaN
NaN	-2,49166	-2,26464	NaN	NaN	NaN	NaN	-0,854832	-0,909135	-0,983195
NaN	-2,49166	-2,26464	NaN	NaN	NaN	NaN	-0,854832	-0,909135	-0,983195
NaN	NaN	NaN	0,860106	0,978256	0,980333	0,744499	NaN	NaN	NaN
NaN	NaN	NaN	0,860106	0,978256	0,980333	0,744499	NaN	NaN	NaN
NaN	NaN	NaN	NaN	NaN	NaN	NaN	NaN	NaN	NaN
1,14166	NaN	NaN	1,84816	NaN	2,68386	NaN	NaN	NaN	NaN
NaN	NaN	NaN	-0,7435	NaN	-1,14344	NaN	NaN	NaN	NaN
NaN	NaN	NaN	-0,7435	NaN	-1,14344	NaN	NaN	NaN	NaN
0,310598	NaN	-0,250401	2,38366	-0,126263	2,86588	-0,280064	0,864752	0,422734	1,4312
NaN	NaN	0,506453	2,38366	-0,126263	2,86588	1,34292	NaN	NaN	NaN
NaN	NaN	NaN	NaN	NaN	NaN	NaN	NaN	NaN	NaN
NaN	NaN	NaN	NaN	NaN	NaN	NaN	NaN	NaN	NaN

Polito et al. Universal ready-to-use immunotherapeutic approach for the treatment of cancer: expanded and activated polyclonal γδ memory T cells.
Phosphorylation sites

NaN	NaN	NaN	0,804251	-1,41094	NaN	-2,35248	NaN	NaN	NaN
NaN	NaN	NaN	0,804251	-1,41094	NaN	-2,35248	NaN	NaN	NaN
-2,12382	NaN	-1,85875	1,20171	NaN	1,21519	0,215969	NaN	NaN	NaN
-2,12382	NaN	-1,85875	1,20171	NaN	1,21519	0,215969	NaN	NaN	NaN
0,201139	-1,32558	-1,20904	-1,19005	0,211609	-0,969738	0,000648499	-0,598822	-1,24432	-1,66458
2,7241	-0,0645142	2,46958	2,26549	3,08059	-0,969738	1,75672	0,206783	-1,81229	NaN
2,7241	-0,0645142	-1,26627	2,26549	0,211609	NaN	-0,785398	0,206783	0,00374889	-0,399459
2,74386	1,46602	2,78581	0,795382	3,31492	2,16311	2,01809	0,972273	0,853898	0,738261
2,74386	1,46602	2,78581	0,795382	3,31492	2,16311	2,01809	0,972273	0,853898	0,738261
0,297945	-0,325109	0,0364189	-0,774986	0,869432	-0,856071	0,126127	-0,0313663	-0,702748	-0,983876
0,156719	-1,0556	-0,784323	0,72016	0,0312195	0,743567	0,646545	NaN	0,297	0,166522
0,156719	-1,0556	-0,784323	0,72016	0,0312195	0,743567	0,646545	0,133982	0,297	0,166522
-0,0190086	0,507263	-1,05027	NaN	0,0186996	0,823681	NaN	-0,729692	NaN	-0,727941
-0,0190086	0,507263	-1,05027	NaN	0,0186996	0,823681	NaN	-0,729692	NaN	-0,727941
0,576799	-0,690931	-0,945812	-0,911501	0,563084	NaN	0,646545	-0,361719	-0,569835	-0,525428
0,576799	-0,690931	-0,945812	-0,911501	0,563084	NaN	0,646545	-0,361719	-0,569835	-0,525428
0,925196	1,03366	1,20021	0,943047	2,08967	NaN	1,47081	1,18972	0,705472	0,908324
0,925196	1,03366	1,20021	0,943047	2,08967	NaN	1,47081	1,18972	0,705472	0,908324
5,31514	5,17643	4,7317	4,98658	4,50635	4,10552	4,73408	4,95848	4,94291	4,5523
5,31514	5,17643	4,7317	4,98658	4,50635	4,10552	4,73408	4,95848	4,94291	4,5523
NaN	NaN	NaN	-0,133307	NaN	NaN	-0,496824	NaN	NaN	NaN
-0,220215	0,312786	-0,658579	0,157959	-0,673082	0,0964088	-0,312361	-0,940891	-0,885036	-1,1191
NaN	NaN	NaN	0,157959	NaN	0,0964088	-0,312361	-0,940891	-0,885036	-1,1191
NaN	NaN	-0,319475	NaN	NaN	NaN	NaN	-0,393246	-0,989762	-1,55378
0,521444	NaN	0,188183	NaN	0,243444	-2,71504	-1,29509	-1,11812	-1,4968	-1,80139
0,521444	NaN	0,188183	NaN	0,243444	-2,71504	-1,29509	-1,11812	-1,4968	-1,80139
NaN	NaN	NaN	NaN	NaN	NaN	NaN	NaN	NaN	NaN
NaN	NaN	NaN	NaN	NaN	NaN	NaN	NaN	NaN	NaN
NaN	NaN	NaN	-1,65647	NaN	-1,33889	-1,41624	NaN	NaN	NaN
NaN	NaN	NaN	-2,20617	NaN	-1,43801	-0,971981	NaN	NaN	NaN
-0,481575	-1,31651	-0,573698	-0,254862	NaN	-0,0547104	-0,511467	0,031992	-1,062	-1,38078
-0,481575	-1,31651	-0,573698	-0,254862	NaN	-0,0547104	-0,511467	0,031992	-1,062	-1,38078
2,33322	1,61575	2,39445	2,14822	2,49746	NaN	2,9713	1,45313	0,96313	0,803092
2,33322	1,61575	2,39445	2,14822	2,49746	NaN	2,9713	1,45313	0,96313	0,803092

NaN	NaN	NaN	1,43796	1,65558	0,219566	1,96979	NaN	NaN	NaN
NaN	NaN	NaN	1,43796	1,65558	0,219566	1,96979	NaN	NaN	NaN
0,029953	1,27762	-0,895027	NaN	NaN	NaN	NaN	NaN	NaN	NaN
NaN	NaN	NaN	NaN	NaN	NaN	NaN	NaN	NaN	NaN
0,029953	1,27762	-0,895027	NaN	NaN	NaN	NaN	NaN	NaN	NaN
NaN	NaN	NaN	NaN	NaN	NaN	NaN	-1,61852	-0,892821	-0,274394
NaN	NaN	NaN	NaN	NaN	NaN	NaN	-1,61852	-0,892821	-0,274394
-1,60176	-2,62619	-2,40219	-2,34655	-2,46097	NaN	-1,27246	-1,34243	-2,44127	-2,31404
-1,60176	-2,62619	-2,40219	-2,34655	-2,46097	NaN	-1,27246	-1,34243	-2,44127	-2,31404
-2,34139	-0,0764561	-1,2368	NaN	NaN	NaN	NaN	-0,331997	0,491275	-0,6407
NaN	NaN	3,38728	2,19638	2,77537	NaN	2,42523	2,97906	2,39235	3,71266
-1,42552	-2,64331	-1,92901	-2,39629	-1,03911	NaN	-1,85031	-1,92442	-2,06016	-2,38072
1,44065	1,36947	1,12503	-0,565979	1,74304	-0,358717	0,871098	1,10656	0,765698	0,791604
2,85471	2,23871	1,6019	1,69863	2,38259	1,64681	1,75168	2,43212	1,95141	1,47619
-1,35566	-0,214493	-2,5347	-0,851629	-1,87869	NaN	-0,145493	-1,5455	NaN	-2,11841
-1,96516	-1,79637	-2,69607	-0,851629	-1,87869	NaN	-0,562368	NaN	NaN	NaN
-1,35566	-0,214493	-2,5347	-1,37984	-2,52999	NaN	-0,145493	NaN	NaN	-2,11841
1,96507	1,75648	1,11323	1,35581	1,56489	0,0275383	1,98776	0,475569	0,797551	0,504807
1,96507	1,75648	1,11323	1,35581	1,56489	0,0275383	1,98776	0,475569	0,797551	0,504807
-0,475355	-1,30099	-1,20078	-0,187351	0,187017	NaN	-0,552763	-0,68594	-0,823058	-0,867303
-0,475355	-1,30099	-1,20078	-0,187351	0,187017	NaN	-0,552763	-0,68594	-0,823058	-0,867303
0,821783	3,39057	-0,548925	2,19566	2,6018	-0,0690956	2,53938	NaN	NaN	1,67592
NaN	0,910028	NaN	1,85061	0,145336	NaN	1,5068	-0,334415	-0,561185	0,0845957
NaN	0,910028	NaN	1,85061	0,145336	NaN	1,5068	-0,334415	-0,561185	0,0845957
0,570366	0,716839	1,29171	0,926023	0,901186	-1,40654	1,8246	0,66387	0,698916	1,18981
1,80489	0,474339	1,45732	1,1389	1,30243	NaN	1,25969	1,36214	1,373	1,45843
NaN	0,474339	-1,36229	NaN	1,30243	NaN	NaN	-1,16495	1,373	-2,2762
0,893465	2,06724	1,72017	NaN	2,27079	0,69997	2,3583	1,48244	1,34849	NaN
0,893465	2,06724	1,72017	1,71837	2,27079	0,69997	2,3583	1,48244	1,34849	1,89524
1,92426	0,82086	NaN	NaN	2,62014	-2,58281	3,0814	NaN	1,18879	2,25366
1,92426	0,82086	0,401051	3,25711	2,62014	3,61248	3,0814	0,79818	1,18879	2,25366
1,41861	0,718922	0,0777168	-0,152426	0,518505	0,303234	-0,253075	0,627005	0,052598	-0,699636
NaN	-0,317207	0,124977	1,96266	NaN	1,83108	1,72124	NaN	NaN	NaN
-0,858679	-1,89501	-0,704679	-1,25009	-0,515739	NaN	NaN	NaN	NaN	NaN

Polito et al. Universal ready-to-use immunotherapeutic approach for the treatment of cancer: expanded and activated polyclonal γδ memory T cells.
Phosphorylation sites

-0,991234	-0,86091	NaN	-0,701521	-1,83128	-0,824978	NaN	-1,97883	-2,14569	-2,42885
NaN	NaN	NaN	-1,34856	NaN	0,511839	-1,68887	NaN	NaN	NaN
NaN	NaN	NaN	-1,74119	-0,417799	-1,78435	NaN	NaN	NaN	NaN
NaN	NaN	NaN	-1,74119	-0,417799	-1,78435	NaN	NaN	NaN	NaN
0,45723	-0,460268	-0,487488	-1,61674	-0,498446	-1,32802	-0,424828	-0,608145	-0,828837	-1,30302
0,45723	-0,460268	-0,487488	-1,61674	-0,498446	-1,32802	-0,424828	-0,608145	-0,828837	-1,30302
2,86115	2,56514	2,06384	2,62929	2,23222	2,94739	2,39924	1,60484	1,1621	1,03491
-2,52024	-3,11219	-1,48978	NaN	-1,07574	NaN	NaN	NaN	NaN	NaN
NaN	NaN	NaN	NaN	NaN	NaN	NaN	-1,7802	NaN	-3,13353
NaN	NaN	NaN	NaN	NaN	NaN	NaN	-1,7802	NaN	-3,13353
1,94243	2,16197	1,37726	1,40391	0,11297	-3,24368	1,44887	0,0994549	0,93036	-0,200332
1,94243	2,16197	1,37726	1,40391	0,11297	-3,24368	1,44887	0,0994549	0,93036	-0,200332
NaN	NaN	NaN	1,11083	-0,675579	0,930151	NaN	NaN	NaN	NaN
1,81025	1,8846	1,39526	2,00522	2,12765	0,171217	1,39253	0,994476	1,0155	0,724162
1,81025	1,8846	1,39526	2,00522	2,12765	0,171217	1,39253	0,994476	1,0155	0,724162
0,151403	NaN	0,739401	-0,256882	-0,199955	-0,380033	0,199793	NaN	NaN	NaN
0,151403	NaN	0,739401	-0,256882	-0,199955	-0,380033	0,199793	NaN	NaN	NaN
NaN	NaN	NaN	NaN	NaN	NaN	NaN	NaN	NaN	NaN
-0,589075	-1,21364	-1,42716	-0,692848	-1,2653	-0,82114	-1,74229	NaN	NaN	NaN
-0,589075	-1,21364	-1,42716	-0,692848	-1,2653	-0,82114	-1,74229	-1,56301	-1,68167	-2,28613
2,83758	4,56916	1,54507	2,45244	-0,0158672	-0,615976	2,73661	0,369286	2,58479	1,69844
-0,311636	-1,90215	-2,0963	-2,38521	-0,8365	NaN	-1,92175	-1,23525	-1,26789	-2,26391
-0,311636	-1,90215	-2,0963	-2,38521	-0,8365	NaN	-1,92175	-1,23525	-1,26789	-2,26391
0,569883	0,620827	0,4487	0,840221	-1,67521	NaN	0,179888	1,59422	1,03635	1,52382
0,569883	0,620827	0,4487	0,840221	-1,67521	NaN	0,179888	1,59422	1,03635	1,52382
NaN	NaN	NaN	NaN	NaN	NaN	NaN	-2,83437	-2,69423	-3,07276
NaN	NaN	NaN	NaN	NaN	NaN	NaN	-2,83437	-2,69423	-3,07276
-1,28238	-1,22583	NaN	-0,310265	NaN	3,05493	-1,52613	-2,04615	-1,84448	-2,30823
-1,28238	-1,22583	NaN	-0,310265	NaN	3,05493	-1,52613	-2,04615	-1,84448	-2,30823
NaN	1,10637	NaN	1,18426	NaN	NaN	0,879503	1,12131	1,05216	0,848023
NaN	-1,18011	-1,11459	-1,55797	NaN	-1,26247	-0,860826	-1,49668	-1,70395	-0,377618
NaN	-0,173788	-1,11459	-1,55797	-2,88927	-1,26247	-0,860826	-1,49668	-1,70395	-0,377618
NaN	NaN	NaN	NaN	-2,88927	-1,38175	-1,83333	NaN	NaN	NaN
0,559895	1,41751	0,646048	0,535795	0,623814	0,240511	0,826429	0,191488	0,415385	-0,105706

Polito et al. Universal ready-to-use immunotherapeutic approach for the treatment of cancer: expanded and activated polyclonal γδ memory T cells.
Phosphorylation sites

0,559895	1,41751	0,646048	0,535795	0,623814	0,240511	0,826429	0,191488	0,415385	-0,105706
NaN	NaN	NaN	0,574207	-1,59066	0,533112	0,0342293	NaN	NaN	NaN
NaN	NaN	NaN	0,574207	-1,59066	0,533112	0,0342293	NaN	NaN	NaN
NaN	NaN	NaN	-0,46081	NaN	-1,01661	NaN	NaN	NaN	NaN
-0,437321	-2,16648	NaN	-0,349379	-0,332911	-0,699215	NaN	0,360861	-0,346126	0,12674
-0,437321	-2,16648	NaN	-0,349379	-0,332911	-0,699215	NaN	0,360861	-0,346126	0,12674
NaN	NaN	NaN	-1,39436	NaN	-0,640089	-1,61657	NaN	NaN	NaN
NaN	NaN	NaN	-1,39436	NaN	-0,640089	-1,61657	NaN	NaN	NaN
-0,907206	-0,681581	-2,75466	NaN	-1,18236	NaN	-0,994246	-1,59234	-2,28088	-2,01851
-0,907206	-0,681581	-2,75466	-1,21698	-1,18236	NaN	-0,994246	-1,59234	-2,28088	-2,01851
2,75508	4,24861	1,68908	2,16537	3,18	1,96141	3,04298	1,28174	3,07498	2,16919
2,75508	4,24861	1,68908	2,16537	3,18	1,96141	3,04298	1,28174	3,07498	2,16919
2,84902	NaN	NaN	NaN	3,63154	0,563919	3,09553	NaN	NaN	NaN
2,84902	NaN	NaN	NaN	3,63154	0,563919	3,09553	NaN	NaN	NaN
NaN	NaN	NaN	-2,23348	-1,65189	-1,19239	-1,38468	NaN	NaN	NaN
-0,0376282	1,41855	-0,430586	-0,443808	0,517294	0,409094	0,433815	-1,03568	0,250831	-0,0800085
-0,0376282	1,41855	-0,430586	-0,443808	0,517294	0,409094	0,433815	-1,03568	0,250831	-0,0800085
-0,856415	-0,297487	-0,795942	-1,46969	-1,34286	NaN	-1,92353	-1,3353	-0,769216	-1,72695
-0,856415	-0,297487	-0,795942	-1,46969	-1,34286	NaN	-1,92353	-1,3353	-0,769216	-1,72695
-1,55176	-2,17553	-2,68853	NaN	NaN	NaN	NaN	NaN	NaN	NaN
-1,55176	-2,17553	-2,68853	NaN	NaN	NaN	NaN	NaN	NaN	NaN
-1,77517	NaN	-0,667339	-0,600904	NaN	-1,69951	-1,12636	0,199417	-0,193814	-0,646544
-1,77517	NaN	-0,667339	-0,600904	NaN	-1,69951	-1,12636	0,199417	-0,193814	-0,646544
2,4596	1,18645	2,37211	2,00618	2,37499	NaN	1,89455	1,47214	1,2456	2,69086
2,4596	1,18645	2,37211	2,00618	2,37499	NaN	1,89455	1,47214	1,2456	2,69086
-1,97145	NaN	-0,948347	-1,59298	-1,97455	-0,184332	-0,874897	NaN	NaN	NaN
2,62504	2,49469	2,73492	1,89002	2,12204	2,41378	1,65196	2,18364	2,59752	1,68787
2,62504	2,49469	2,73492	1,89002	2,12204	2,41378	1,65196	2,18364	2,59752	1,68787
NaN	NaN	NaN	-2,50312	-1,40084	-2,61894	NaN	NaN	NaN	NaN
0,66943	-1,10625	-0,565111	NaN	0,299896	-0,0252647	NaN	NaN	NaN	NaN
0,66943	-1,10625	-0,565111	NaN	0,299896	-0,0252647	NaN	NaN	NaN	NaN
1,34399	2,87132	0,43412	1,07376	0,0254478	1,24183	1,87969	0,0399971	1,11155	1,11666
0,26169	-2,13275	-1,05948	NaN	NaN	-0,0206375	NaN	NaN	0,204881	-1,09336
NaN	-1,33734	-0,745356	-1,7407	0,397558	NaN	-2,19572	0,00907516	-0,248363	0,112227

Polito et al. Universal ready-to-use immunotherapeutic approach for the treatment of cancer: expanded and activated polyclonal γδ memory T cells.
Phosphorylation sites

NaN	NaN	NaN	NaN	NaN	NaN	NaN	-3,56569	NaN	-1,67899
NaN	NaN	NaN	NaN	NaN	NaN	NaN	-3,56569	NaN	-1,67899
NaN	NaN	NaN	0,276884	NaN	-0,670647	-0,560982	NaN	NaN	NaN
NaN	NaN	NaN	0,276884	NaN	-0,670647	-0,560982	NaN	NaN	NaN
NaN	NaN	NaN	0,861523	1,80887	-0,00708771	0,461393	NaN	NaN	0,742795
1,16723	-0,561905	0,858751	2,92704	0,109522	2,90024	2,56623	1,52371	1,15971	0,950736
1,16723	-0,561905	0,858751	2,92704	0,109522	2,90024	2,56623	1,52371	1,15971	0,950736
NaN	NaN	NaN	NaN	NaN	NaN	NaN	NaN	NaN	NaN
-2,05135	-2,14358	-1,57234	-1,48191	-2,2088	-0,517776	-1,688	-1,18868	-1,02213	-1,76062
-2,05135	-2,14358	-1,57234	NaN	-2,2088	-0,517776	-1,688	-1,18868	-1,02213	-1,76062
1,19363	2,87093	-0,836739	1,53706	-1,12897	0,952986	1,34898	-0,991371	2,19602	0,807818
2,22567	2,45691	1,83239	1,93148	2,01981	2,12858	0,833845	0,999226	1,80361	2,10817
2,22567	2,45691	1,83239	1,93148	2,01981	2,12858	0,833845	0,999226	1,80361	2,10817
NaN	NaN	NaN	-0,613758	NaN	0,00227547	-1,50074	NaN	NaN	NaN
NaN	NaN	NaN	-0,613758	NaN	0,00227547	-1,50074	NaN	NaN	NaN
-0,691221	-3,09545	0,676737	2,67021	-2,56705	3,54334	0,941092	-0,145613	0,581712	0,91941
-0,691221	-3,09545	0,676737	2,67021	-2,56705	3,54334	0,941092	-0,145613	0,581712	0,91941
-0,835791	-2,15637	-0,663305	NaN	NaN	NaN	NaN	-1,83085	NaN	-2,60738
-0,835791	-2,15637	-0,663305	NaN	NaN	NaN	NaN	-1,83085	NaN	-2,60738
NaN	NaN	NaN	-1,09601	NaN	-1,87678	-1,16949	NaN	NaN	NaN
NaN	NaN	NaN	-1,09601	NaN	-1,87678	-1,16949	NaN	NaN	NaN
NaN	NaN	NaN	0,420488	0,981802	0,810349	1,59946	NaN	NaN	NaN
NaN	NaN	NaN	0,420488	0,981802	0,810349	1,59946	NaN	NaN	NaN
0,20277	NaN	0,938444	0,766497	NaN	1,06043	0,365139	NaN	0,304061	1,50736
NaN	NaN	NaN	NaN	NaN	NaN	NaN	NaN	NaN	NaN
NaN	NaN	NaN	NaN	NaN	NaN	NaN	NaN	NaN	NaN
NaN	NaN	NaN	NaN	NaN	NaN	NaN	1,79854	1,44105	1,96285
NaN	NaN	NaN	NaN	NaN	NaN	NaN	1,79854	1,44105	1,96285
NaN	0,438692	-2,86855	-1,48711	NaN	-0,853861	-2,06645	NaN	-2,07676	NaN
NaN	0,438692	-2,86855	-1,48711	NaN	-0,853861	-2,06645	NaN	-2,07676	NaN
8,77E-05	-0,835085	NaN	0,681828	-0,502991	1,95243	0,251976	-1,19764	NaN	-1,87566
8,77E-05	-0,835085	NaN	0,681828	-0,502991	1,95243	0,251976	-1,19764	NaN	-1,87566
0,894547	1,4754	0,398607	1,30114	NaN	-0,666571	1,57379	0,297453	0,751758	NaN
0,894547	1,4754	0,398607	1,30114	NaN	-0,666571	1,57379	0,297453	0,751758	NaN

Polito et al. Universal ready-to-use immunotherapeutic approach for the treatment of cancer: expanded and activated polyclonal γδ memory T cells.
Phosphorylation sites

NaN	NaN	NaN	NaN	-0,895256	0,823545	-0,255367	NaN	NaN	NaN
-1,17229	-0,548901	NaN	NaN	-2,62865	-0,894674	2,2266	NaN	NaN	NaN
-1,17229	-0,548901	NaN	1,44324	-2,62865	-0,894674	2,2266	NaN	NaN	NaN
0,935268	0,808283	-0,672161	2,17668	0,0114403	3,29116	1,32349	NaN	NaN	NaN
0,935268	0,808283	-0,672161	2,17668	0,0114403	3,29116	1,32349	NaN	NaN	NaN
NaN	NaN	NaN	-0,698803	NaN	2,42089	0,668505	NaN	NaN	NaN
NaN	NaN	NaN	-0,698803	NaN	2,42089	0,668505	NaN	NaN	NaN
-1,71465	NaN	-2,56135	1,05654	-1,68314	1,29045	0,801001	NaN	NaN	NaN
-1,71465	NaN	-2,56135	1,05654	-1,68314	1,29045	0,801001	NaN	NaN	NaN
NaN	NaN	NaN	-0,388498	NaN	-0,267817	-0,873341	NaN	NaN	NaN
1,35433	0,492723	0,859919	0,841043	0,810944	1,16091	NaN	1,15194	0,748954	0,421586
NaN	NaN	NaN	NaN	NaN	NaN	NaN	-1,97167	-2,27194	-2,47914
2,92666	-0,749571	NaN	-2,3904	2,99933	0,823883	-1,95291	2,15598	1,8421	0,93077
2,92666	-0,749571	NaN	-2,3904	2,99933	0,823883	-1,95291	2,15598	1,8421	0,93077
0,835108	0,714277	0,483061	0,00790215	0,425497	-1,30762	0,398869	0,693151	0,260024	0,0347471
1,37573	0,311657	-0,103275	1,16758	0,631653	0,549417	1,6987	1,32788	0,920207	1,14868
0,931293	1,68855	1,8196	0,804251	0,147186	-0,261812	0,442886	0,724571	0,952889	0,58525
-0,348534	-0,724232	-0,117439	-1,50411	-0,661839	NaN	-2,43286	-0,697989	-0,685891	-1,49678
-1,5373	-0,963104	-0,642376	-1,04308	NaN	-0,664005	NaN	-1,04795	0,482949	-0,851151
NaN	NaN	0,345898	1,38503	NaN	1,77934	1,98528	1,2111	NaN	1,21879
-1,66497	-0,980827	-3,33976	NaN	NaN	NaN	NaN	NaN	NaN	NaN
NaN	NaN	NaN	-1,19295	NaN	-0,997581	-0,448572	NaN	NaN	NaN
NaN	NaN	NaN	-1,19295	NaN	-0,997581	-0,448572	NaN	NaN	NaN
NaN	NaN	NaN	1,73039	NaN	1,9343	0,925409	NaN	NaN	NaN
NaN	NaN	NaN	1,73039	NaN	1,9343	0,925409	NaN	NaN	NaN
-1,65292	-2,76225	0,187475	0,576674	-1,10534	0,832994	-0,0950203	-0,911694	-0,850234	-0,836938
-1,65292	-2,76225	0,187475	0,576674	-1,10534	0,832994	-0,0950203	-0,911694	-0,850234	-0,836938
0,282761	0,182539	0,186916	0,68549	0,336271	1,09006	0,753012	-0,0068512	0,15705	0,732472
NaN	NaN	NaN	NaN	NaN	NaN	NaN	NaN	NaN	NaN
1,63053	1,93579	NaN	1,94457	2,04483	2,02389	1,20248	2,33832	1,7798	1,48859
1,63053	1,93579	3,1145	1,94457	2,04483	2,02389	1,20248	2,33832	1,7798	1,48859
NaN	NaN	NaN	-0,980989	NaN	-1,09892	NaN	NaN	NaN	NaN
-0,569864	-1,43821	-1,38869	-1,37903	-0,00276184	-1,70951	-0,784128	-1,49584	-3,14597	-0,720622
-0,33057	-1,33702	-0,879969	-0,203941	-0,404001	-2,34963	-0,212969	-0,486336	-1,57517	-0,711387

Polito et al. Universal ready-to-use immunotherapeutic approach for the treatment of cancer: expanded and activated polyclonal γδ memory T cells.
Phosphorylation sites

-1,39245	-1,34765	-1,41926	NaN	NaN	NaN	NaN	NaN	NaN	NaN
-1,39245	-1,34765	-1,41926	NaN	NaN	NaN	NaN	NaN	NaN	NaN
1,02663	0,753061	-0,241302	0,604748	1,03355	0,624949	0,0911846	-0,49777	-0,299846	-0,418502
NaN	-0,0532036	NaN	-0,787687	NaN	0,219976	-1,26299	-0,929779	-1,54757	-1,37286
0,0368652	0,648022	NaN	-0,13377	NaN	0,576429	-0,221073	NaN	-1,21527	NaN
-0,0339222	-0,559309	-0,623053	-0,24618	-0,634083	-2,52097	-0,732681	-0,0135326	-0,231627	-0,548602
-0,0339222	-0,559309	-0,623053	-0,24618	-0,634083	-2,52097	-0,732681	-0,0135326	-0,231627	-0,548602
4,34328	NaN	NaN	NaN	NaN	NaN	NaN	NaN	3,35536	3,15186
4,34328	NaN	3,45396	NaN	NaN	1,36479	0,0779705	NaN	3,35536	NaN
-0,367643	-2,75245	-0,268772	NaN	-1,38829	NaN	NaN	NaN	NaN	NaN
-0,367643	-2,75245	-0,268772	NaN	-1,38829	NaN	NaN	NaN	NaN	NaN
0,059082	-0,280321	-0,184195	1,20634	0,438816	1,43299	1,92099	0,228384	-0,179994	-0,733806
1,54813	0,895363	0,658857	2,12923	NaN	1,40489	1,33682	0,715668	0,953313	0,74348
1,54813	0,895363	0,658857	2,12923	NaN	1,40489	1,33682	0,715668	0,953313	0,74348
-1,16687	-1,95462	NaN	-1,60926	-1,77502	-1,24297	NaN	NaN	NaN	NaN
-1,16687	-1,95462	NaN	-1,60926	-1,77502	-1,24297	NaN	NaN	NaN	NaN
-0,30448	-0,0572662	-1,15863	-0,636209	0,123787	-1,33535	-0,578362	-0,504019	NaN	-0,47585
0,865438	0,202307	-0,138399	-0,0313663	-0,453135	NaN	-0,519035	0,401556	-2,69915	-1,30613
0,865438	0,202307	-0,138399	-0,0313663	-0,453135	NaN	-0,519035	0,401556	-2,69915	-1,30613
NaN	NaN	NaN	-1,00079	-1,68835	-1,14222	-1,09672	NaN	NaN	NaN
NaN	NaN	NaN	NaN	-1,68835	-1,14222	NaN	NaN	NaN	NaN
NaN	NaN	NaN	-1,33681	NaN	-2,5586	NaN	NaN	NaN	NaN
NaN	NaN	NaN	-1,33681	NaN	-2,5586	NaN	NaN	NaN	NaN
NaN	NaN	NaN	-1,72585	-2,08216	-2,03869	-1,3313	NaN	NaN	NaN
NaN	NaN	NaN	-1,72585	-2,08216	-2,03869	-1,3313	NaN	NaN	NaN
-1,07966	-0,255709	-0,151196	NaN	-0,827793	NaN	1,12508	-0,673117	-0,457656	-0,747611
-1,07966	-0,255709	-0,151196	NaN	-0,827793	NaN	1,12508	-0,673117	-0,457656	-0,747611
NaN	NaN	NaN	NaN	NaN	NaN	NaN	-1,42782	-1,03569	-1,01318
NaN	NaN	NaN	NaN	NaN	NaN	NaN	-1,42782	-1,03569	-1,01318
0,461386	0,995491	0,312065	0,348957	1,88266	1,23904	0,604452	0,416471	1,46435	0,836633
NaN	0,40391	NaN	-0,0827885	1,27884	NaN	NaN	-0,303396	0,539298	-0,453397
1,40679	1,36228	1,39357	2,74519	1,13944	3,35919	2,3562	NaN	NaN	NaN
1,40679	1,36228	1,39357	2,74519	1,13944	3,35919	2,3562	NaN	NaN	NaN
NaN	NaN	NaN	NaN	NaN	NaN	NaN	-1,80657	-1,83603	-2,55911

Polito et al. Universal ready-to-use immunotherapeutic approach for the treatment of cancer: expanded and activated polyclonal γδ memory T cells.
Phosphorylation sites

NaN	NaN	NaN	NaN	NaN	NaN	NaN	-1,80657	-1,83603	-2,55911
-0,960764	-1,77611	-0,491594	1,75935	-2,09693	2,27306	1,27297	-0,46652	-0,732972	-1,45938
-0,960764	-1,77611	-0,491594	1,75935	-2,09693	2,27306	1,27297	-0,46652	-0,732972	-1,45938
-1,32872	-0,367088	-2,72549	0,972761	NaN	2,21799	0,86006	-3,10891	-1,02568	-2,21461
-1,32872	-0,367088	-2,72549	0,972761	NaN	2,21799	0,86006	-3,10891	-1,02568	-2,21461
-1,16943	NaN	NaN	0,253759	-0,729763	0,414768	0,941441	-1,42628	-1,54219	-1,83809
-2,69234	-0,196869	NaN	-1,88037	-0,648762	NaN	-1,09295	-2,33171	NaN	-1,77343
-2,69234	-0,196869	NaN	-1,88037	-0,648762	NaN	-1,09295	-2,33171	NaN	-1,77343
-0,991056	-1,35851	NaN	-0,817116	-2,32651	-0,977432	NaN	NaN	-1,12564	NaN
-0,991056	-1,35851	NaN	-0,817116	-2,32651	-0,977432	NaN	-0,812428	-1,12564	NaN
-1,50571	-0,732258	-1,04423	-0,530258	0,0304394	-1,27477	-0,506601	-1,08949	-1,30187	-0,932822
-1,50571	-0,732258	-1,04423	-0,530258	0,0304394	-1,27477	-0,506601	-1,08949	-1,30187	-0,932822
NaN	NaN	NaN	-1,95538	-2,30795	-2,34847	NaN	NaN	NaN	NaN
-0,416319	0,777349	NaN	0,407518	-0,298059	-1,1659	NaN	-1,4959	0,234138	-0,0493364
-0,416319	0,777349	NaN	0,407518	-0,298059	-1,1659	NaN	-1,4959	0,234138	-0,0493364
1,00143	NaN	NaN	2,42597	1,08526	1,9488	1,12754	NaN	NaN	-0,46564
1,00143	NaN	-0,0308285	2,42597	1,08526	-0,412952	1,12754	NaN	-0,398284	-0,46564
0,265688	0,401554	0,157269	0,850351	0,779686	0,780312	0,741964	-0,162235	-0,361905	0,425948
0,265688	0,401554	0,157269	0,850351	0,779686	0,780312	0,741964	-0,162235	-0,361905	0,425948
0,178045	-0,643169	-1,08466	0,442026	-0,212122	1,07518	0,340342	NaN	NaN	NaN
0,178045	-0,643169	-1,08466	0,442026	-0,212122	1,07518	0,340342	NaN	NaN	NaN
NaN	-0,329605	NaN	1,5792	0,617414	NaN	0,0818176	NaN	NaN	NaN
-0,134022	-0,846804	-1,09065	NaN	-0,294254	-2,43428	NaN	-1,06648	-0,648953	-1,59548
-0,692303	-0,232134	-1,15598	0,151718	-2,55051	NaN	-1,29679	0,122641	-0,709239	-0,300273
-0,692303	-0,232134	-1,15598	0,151718	-2,55051	NaN	-1,29679	0,122641	-0,709239	-0,300273
-0,502546	-2,22762	-1,09133	-1,17362	-1,01903	-0,728481	-1,355	-0,439276	-1,04874	-1,05447
-0,502546	-2,22762	-1,09133	-1,17362	-1,01903	-0,728481	-1,355	-0,439276	-1,04874	-1,05447
1,1365	-0,0611935	0,542786	0,652306	0,248718	1,72683	0,219543	0,695141	0,960919	0,567443
1,1365	-0,0611935	0,542786	0,652306	NaN	1,72683	0,219543	NaN	0,960919	0,567443
0,397041	1,00142	0,726778	0,235201	0,926121	0,616453	1,12242	0,316986	-0,264655	-0,0294313
0,397041	1,00142	0,726778	0,235201	0,926121	0,616453	1,12242	0,316986	-0,264655	-0,0294313
0,536556	-0,315113	1,31379	-0,0482788	1,08967	0,579802	2,162	0,726849	0,764371	0,850062
0,536556	-0,315113	1,31379	-0,0482788	1,08967	0,579802	2,162	0,726849	0,764371	0,850062
NaN	NaN	NaN	-0,211668	NaN	1,00538	2,37075	NaN	NaN	NaN

Polito et al. Universal ready-to-use immunotherapeutic approach for the treatment of cancer: expanded and activated polyclonal γδ memory T cells.
Phosphorylation sites

NaN	NaN	NaN	-0,211668	NaN	1,00538	2,37075	NaN	NaN	NaN
0,872528	1,60483	-1,09521	0,740986	-0,0570107	1,15538	-0,212038	-0,381079	0,742595	-0,339923
NaN	NaN	-0,171963	NaN	1,23252	-1,14227	0,41823	-1,44776	-0,714345	-0,68274
1,4535	0,533762	-0,649839	-0,444258	1,12738	0,968563	0,117727	0,0780869	-0,00451946	-0,429557
1,4535	0,533762	-0,649839	-0,444258	1,12738	0,968563	0,117727	0,0780869	-0,00451946	-0,429557
-0,683924	NaN	-1,02512	-0,925051	0,0179424	0,992611	-0,160675	-2,7842	-2,50143	0,236056
-0,683924	NaN	-1,02512	-0,925051	0,0179424	0,992611	-0,160675	-2,7842	-2,50143	0,236056
0,185112	-0,232353	-0,650173	1,00966	0,353264	1,49136	-0,0207291	0,317766	0,31462	0,970077
0,185112	-0,232353	-0,650173	1,00966	-0,823107	1,49136	-0,171658	-0,515953	-0,176837	-0,68031
-0,18462	-0,421757	-0,760033	0,572971	NaN	-0,269751	NaN	0,317766	0,31462	0,970077
0,694885	NaN	1,32998	1,63997	0,960117	-0,655146	1,59695	0,575008	0,474914	0,316413
NaN	-1,95886	-0,648876	-0,365202	-1,11043	-1,90195	-0,517948	-1,26067	-1,61344	-1,38809
NaN	0,0503941	1,32998	1,63997	NaN	-0,655146	1,59695	0,575008	NaN	0,316413
NaN	NaN	NaN	-0,918503	-2,68808	-0,428352	-1,12417	NaN	NaN	NaN
NaN	NaN	NaN	-0,918503	-2,68808	-0,428352	-1,12417	NaN	NaN	NaN
0,329176	-0,947308	-0,674664	0,63773	NaN	2,1056	0,944695	-0,18284	-2,62124	0,788419
3,86553	3,13156	3,23153	4,38473	3,08291	4,53065	3,92894	3,87366	3,40086	4,42027
3,86553	3,13156	3,23153	4,38473	3,08291	4,53065	3,92894	3,87366	3,40086	4,42027
-0,790728	-1,73011	NaN	-0,35767	-1,12922	0,769667	1,11851	-0,613211	-0,316401	NaN
-0,790728	-1,73011	NaN	-0,35767	-1,12922	0,769667	1,11851	-0,613211	-0,316401	NaN
3,25899	1,26575	2,8801	2,81314	3,50662	2,77081	3,7293	3,34886	3,23466	2,92682
-0,178291	-0,620646	-0,0238285	-0,268969	1,00678	-1,09648	0,770016	-0,7442	-1,22675	-0,376636
-0,178291	-0,620646	-0,0238285	-0,268969	1,00678	-1,09648	0,770016	-0,7442	-1,22675	-0,376636
NaN	NaN	NaN	NaN	NaN	NaN	NaN	NaN	NaN	NaN
NaN	NaN	NaN	NaN	NaN	NaN	NaN	NaN	NaN	NaN
NaN	-1,82153	NaN	NaN	NaN	NaN	-1,11674	NaN	NaN	NaN
NaN	-1,82153	NaN	NaN	NaN	NaN	-1,11674	NaN	NaN	NaN
1,35912	2,73192	1,873	1,74746	2,47805	0,442547	2,47821	2,0052	2,0118	2,40795
1,35912	2,73192	1,873	1,74746	2,47805	0,442547	2,47821	2,0052	2,0118	2,40795
-0,782988	0,509127	-0,756126	-1,93549	0,352196	-2,10633	0,776293	-0,253662	0,0148535	0,465627
-0,782988	0,509127	-0,756126	-1,93549	0,352196	-2,10633	0,776293	-0,253662	0,0148535	0,465627
NaN	NaN	NaN	-1,5854	NaN	-1,06937	NaN	NaN	NaN	NaN
NaN	NaN	NaN	-1,5854	NaN	-1,06937	NaN	NaN	NaN	NaN
-1,62566	-0,688293	-1,75896	-1,846	-0,159666	NaN	-1,4615	-2,01587	NaN	-0,250228

Polito et al. Universal ready-to-use immunotherapeutic approach for the treatment of cancer: expanded and activated polyclonal γδ memory T cells.
Phosphorylation sites

-1,62566	-0,688293	-1,75896	NaN	NaN	NaN	NaN	-2,01587	-1,20054	-0,250228
1,01309	0,33605	-0,199884	0,87702	-0,0752277	-0,449207	0,570965	-0,14027	-0,490645	-1,02154
1,01309	0,33605	-0,199884	0,87702	-0,0752277	-0,449207	0,570965	-0,14027	-0,490645	-1,02154
NaN	NaN	NaN	-1,08723	-2,78647	-0,139421	NaN	NaN	NaN	NaN
NaN	NaN	NaN	-1,08723	-2,78647	-0,139421	NaN	NaN	NaN	NaN
NaN	NaN	NaN	0,273466	-0,416887	-1,06927	0,219639	-1,43982	NaN	-1,53997
NaN	NaN	NaN	0,273466	-0,416887	-1,06927	0,219639	-1,43982	NaN	-1,53997
3,02512	2,94296	2,54067	3,03791	2,33773	2,83146	2,48253	1,38084	1,18528	1,63269
3,02512	2,94296	2,54067	3,03791	2,33773	2,83146	2,48253	1,38084	1,18528	1,63269
-2,15003	-2,16236	-1,7848	-2,19089	-0,830774	-3,36474	-1,5911	NaN	NaN	NaN
NaN	NaN	NaN	0,0225811	-2,92845	1,04623	-1,61239	-1,94598	-0,111768	NaN
NaN	NaN	NaN	0,0225811	-2,92845	1,04623	-1,61239	-1,94598	-0,111768	NaN
-2,02959	-2,98724	-3,22811	-2,48212	-2,03549	-1,46046	-2,55598	-1,48393	-0,567857	-1,77995
-2,02959	-2,98724	-3,22811	-2,48212	-2,03549	-1,46046	-2,55598	-1,48393	-0,567857	-1,77995
-1,18259	-1,67674	NaN	-1,04744	NaN	-0,632898	-1,54082	NaN	NaN	NaN
0,739552	-0,49493	-1,0356	-0,423498	0,205456	0,551218	0,0609112	-1,02641	-0,781114	-0,0792818
0,739552	NaN	-1,0356	-0,423498	0,205456	0,551218	0,0609112	NaN	NaN	-0,0792818
-1,30349	-1,13104	-0,885557	1,69976	NaN	0,418987	-0,0864773	-1,84853	-1,09434	-2,1066
-1,30349	-1,13104	-0,885557	1,69976	NaN	0,418987	-0,0864773	-1,84853	-1,09434	-2,1066
NaN	NaN	-0,57313	-0,712986	NaN	0,384977	-0,676168	NaN	NaN	NaN
NaN	NaN	-0,57313	-0,712986	NaN	0,384977	-0,676168	NaN	NaN	NaN
-0,95686	-1,17402	-1,13828	-2,39455	-0,575499	-2,98083	-0,982849	-1,73276	-1,08292	-1,4534
-0,95686	-1,17402	-1,13828	-2,39455	-0,575499	-2,98083	-0,982849	-1,73276	-1,08292	-1,4534
-1,84851	-0,0452404	-1,22372	-2,76162	-1,01707	NaN	-2,03405	-1,73907	-1,66623	-0,607524
-1,84851	-0,0452404	-1,22372	-2,76162	-1,01707	NaN	-2,03405	-1,73907	-1,66623	-0,607524
NaN	NaN	NaN	NaN	NaN	NaN	NaN	NaN	NaN	NaN
-0,909092	-2,03841	-0,187763	0,720606	-0,920328	1,2734	-0,0889225	-0,289124	-0,0343065	-0,744224
-0,909092	-2,03841	-0,187763	0,720606	-0,920328	1,2734	-0,0889225	-0,289124	-0,0343065	-0,744224
-2,17552	1,21531	-1,39922	NaN	NaN	NaN	NaN	NaN	-0,700015	-0,761004
-2,17552	1,21531	-1,39922	NaN	NaN	NaN	NaN	NaN	-0,700015	-0,761004
0,806679	0,309898	0,318588	-0,686769	1,70796	-0,839745	-1,19608	NaN	0,65244	0,225202
0,806679	0,309898	0,318588	-0,686769	1,70796	-0,839745	-1,19608	0,427084	0,65244	0,225202
NaN	NaN	NaN	-2,93296	NaN	-2,08538	-2,42864	NaN	NaN	NaN
-1,13554	-2,23435	-1,76824	NaN	NaN	NaN	NaN	NaN	NaN	NaN

Polito et al. Universal ready-to-use immunotherapeutic approach for the treatment of cancer: expanded and activated polyclonal γδ memory T cells.
Phosphorylation sites

NaN	NaN	NaN	-2,08127	NaN	NaN	-2,72102	NaN	NaN	NaN
NaN	NaN	NaN	-2,08127	NaN	NaN	-2,72102	NaN	NaN	NaN
NaN	-2,35963	-0,344637	-0,152548	-2,42028	-0,909927	-0,383167	NaN	NaN	NaN
-3,28711	-3,91851	NaN	-2,48984	NaN	-2,58352	-2,90946	NaN	NaN	NaN
-3,28711	-3,91851	NaN	-2,48984	NaN	-2,58352	-2,90946	NaN	NaN	NaN
-1,98049	-0,0916138	-1,37214	-1,10034	-0,43457	NaN	-1,2125	-1,70598	-2,69609	-1,90845
NaN	NaN	NaN	-2,10417	NaN	-1,93507	-2,59852	NaN	NaN	NaN
NaN	NaN	NaN	1,92597	-0,760115	0,553015	-1,21774	NaN	NaN	NaN
NaN	NaN	NaN	1,92597	-0,760115	0,553015	-1,21774	NaN	NaN	NaN
-1,13065	-1,43353	-3,25954	1,28259	-0,346403	1,36292	-0,356285	-0,3267	-1,91218	-1,76513
-1,13065	-1,43353	-3,25954	1,28259	-0,346403	1,36292	-0,356285	-0,3267	-1,91218	-1,76513
NaN	NaN	NaN	NaN	NaN	NaN	NaN	-1,89815	NaN	-2,39044
-0,668125	-0,548697	-0,128241	2,17794	0,515266	1,69753	1,40178	NaN	NaN	NaN
-0,668125	-0,548697	-0,128241	2,17794	0,515266	1,69753	1,40178	NaN	NaN	NaN
2,06351	2,48066	1,57838	0,378967	1,55911	0,375217	0,863625	1,44665	1,57904	1,53566
-0,0570011	0,512733	-1,22987	NaN	-0,847252	NaN	-0,0526142	NaN	NaN	NaN
0,952902	0,512733	-0,136116	1,51684	1,06243	1,85588	0,646259	0,585901	1,06172	0,948359
-0,531385	-0,680656	-0,543293	NaN	NaN	NaN	NaN	-0,63114	-0,772855	-0,962388
0,111818	-0,578722	-0,0809517	-0,427029	-0,383865	-0,0670853	0,263266	0,645699	-0,11732	-0,603091
0,111818	-0,578722	-0,0809517	-0,427029	-0,383865	-0,0670853	0,263266	0,645699	-0,11732	-0,603091
-0,889318	-0,341223	-1,02845	NaN	NaN	NaN	NaN	-1,1715	-1,5543	NaN
NaN	NaN	NaN	NaN	-0,712467	NaN	-1,10237	NaN	NaN	NaN
NaN	NaN	NaN	NaN	-0,712467	NaN	-1,10237	NaN	NaN	NaN
-0,776779	-0,537912	-1,3605	-0,914791	-1,18069	-0,217945	NaN	NaN	-0,697286	-0,150905
NaN	NaN	NaN	-1,01826	-0,699389	-0,239429	-0,964418	0,451019	-0,117564	-0,290051
NaN	NaN	NaN	-1,01826	-0,699389	-0,239429	-0,964418	0,451019	-0,117564	-0,290051
-0,139101	0,435816	0,048254	-0,311268	0,182289	-0,233641	0,441587	0,143858	0,967856	0,476981
1,14903	1,71959	1,26735	0,973978	0,547781	0,988691	0,830456	0,436771	2,08878	0,678433
-0,998171	-2,4038	-2,01309	-1,54022	-2,63227	-1,42647	-1,82452	-1,94521	-2,33636	-2,63249
-0,998171	-2,4038	-2,01309	-1,54022	-2,63227	-1,42647	-1,82452	-1,94521	-2,33636	-2,63249
NaN	NaN	NaN	-2,60378	NaN	NaN	0,157742	NaN	NaN	NaN
NaN	NaN	NaN	-2,60378	NaN	NaN	0,157742	NaN	NaN	NaN
-0,471018	2,78914	0,0622902	0,332964	2,61118	4,45464	1,90198	-0,203054	-0,365653	-2,97402
-0,471018	2,78914	0,0622902	0,332964	2,61118	4,45464	1,90198	-0,203054	-0,365653	-2,97402

Polito et al. Universal ready-to-use immunotherapeutic approach for the treatment of cancer: expanded and activated polyclonal γδ memory T cells.
Phosphorylation sites

NaN	NaN	NaN	NaN	NaN	NaN	NaN	-0,134508	-0,566125	-0,540603
NaN	NaN	NaN	NaN	NaN	NaN	NaN	-1,56146	-1,55774	-1,6034
NaN	NaN	NaN	NaN	NaN	NaN	NaN	-1,56146	-1,55774	-1,6034
1,32718	1,26814	1,32525	0,991465	2,13769	0,365019	1,29791	0,24618	0,99805	NaN
1,32718	1,26814	1,32525	0,991465	2,13769	0,365019	1,29791	0,24618	0,99805	NaN
NaN	NaN	NaN	-1,65652	-2,37049	NaN	-1,30301	NaN	NaN	NaN
NaN	NaN	NaN	-1,65652	-2,37049	NaN	-1,30301	NaN	NaN	NaN
NaN	NaN	NaN	-1,92177	NaN	-1,1243	-1,92965	NaN	NaN	NaN
NaN	NaN	NaN	NaN	NaN	NaN	NaN	NaN	NaN	NaN
NaN	NaN	NaN	NaN	NaN	NaN	NaN	NaN	NaN	NaN
-0,779919	NaN	-2,22407	-1,70269	-1,32103	-1,01627	-1,84935	NaN	NaN	NaN
-0,574677	NaN	1,51089	0,887789	NaN	NaN	1,12662	NaN	NaN	NaN
-0,574677	NaN	1,51089	0,887789	NaN	NaN	1,12662	NaN	NaN	NaN
NaN	NaN	NaN	NaN	NaN	NaN	NaN	NaN	NaN	NaN
NaN	NaN	NaN	NaN	NaN	NaN	NaN	NaN	NaN	NaN
NaN	-0,0667801	0,406706	NaN	-0,96903	NaN	-0,269682	0,147242	1,28249	-0,227803
NaN	-0,0667801	0,406706	-0,330048	-0,96903	NaN	-0,269682	0,147242	1,28249	-0,227803
NaN	NaN	NaN	NaN	NaN	NaN	NaN	NaN	NaN	NaN
NaN	NaN	NaN	NaN	NaN	NaN	NaN	NaN	NaN	NaN
NaN	1,50342	NaN	1,11881	2,20738	0,778915	NaN	1,54583	1,65257	NaN
NaN	NaN	NaN	0,507885	NaN	0,623859	0,471485	NaN	NaN	NaN
NaN	NaN	NaN	0,507885	NaN	0,623859	0,471485	NaN	NaN	NaN
0,81723	2,43746	1,26875	1,80573	1,24277	1,90054	2,42789	1,23387	1,53661	1,65378
0,81723	2,43746	1,26875	1,80573	1,24277	1,90054	2,42789	1,23387	1,53661	1,65378
NaN	NaN	NaN	-0,751987	-1,32039	-0,53968	-1,38229	NaN	NaN	NaN
NaN	NaN	NaN	-1,36291	NaN	-0,683542	-1,24736	NaN	NaN	NaN
0,222935	0,452534	-0,380198	NaN	NaN	NaN	NaN	-0,0924702	-0,270507	-0,167743
NaN	NaN	NaN	NaN	NaN	NaN	NaN	NaN	NaN	NaN
NaN	NaN	NaN	NaN	NaN	NaN	NaN	NaN	NaN	NaN
1,31647	1,37177	0,943302	2,21448	1,39583	NaN	2,1328	NaN	0,948113	0,181836
1,31647	1,37177	0,943302	2,21448	1,39583	NaN	2,1328	NaN	0,948113	0,181836
-2,21892	-0,891333	-0,220951	-0,432377	0,705973	-0,467783	0,250267	-0,498863	0,0232725	-0,239188
-2,21892	-0,891333	-0,220951	-0,432377	0,705973	-0,467783	0,250267	-0,498863	0,0232725	-0,239188
0,489464	NaN	0,509583	-0,666029	-0,0930405	-0,208359	-0,509493	-0,445953	-1,09855	-0,687573

0,489464	NaN	0,509583	-0,666029	-0,0930405	-0,208359	-0,509493	-0,445953	-1,09855	-0,687573
NaN	NaN	NaN	NaN	-0,0930405	-0,208359	-0,509493	-0,445953	-1,09855	-0,687573
-0,98102	NaN	0,058691	1,22729	0,318415	2,02085	0,00265694	NaN	NaN	NaN
NaN	NaN	NaN	-1,56546	NaN	-1,79027	-1,44079	NaN	NaN	NaN
NaN	NaN	NaN	NaN	NaN	NaN	NaN	-2,42588	-2,18199	-2,73555
NaN	NaN	NaN	NaN	NaN	NaN	NaN	-2,42588	-2,18199	-2,73555
-1,91186	-1,23688	-2,27722	0,404327	NaN	NaN	NaN	-2,11115	-2,06221	-2,45101
-1,91186	-1,23688	-2,27722	0,404327	-1,34988	NaN	-1,49336	-2,11115	-2,06221	-2,45101
0,222954	NaN	-0,979313	0,439997	1,16172	1,40995	1,41642	0,0262489	-0,967374	-0,257974
0,222954	NaN	-0,979313	0,439997	1,16172	1,40995	1,41642	0,0262489	-0,967374	-0,257974
0,322481	NaN	1,36365	NaN	1,15497	NaN	0,314571	0,423889	0,437831	1,04517
NaN	NaN	NaN	-1,66664	NaN	0,0692539	-0,991043	NaN	NaN	NaN
NaN	NaN	NaN	-1,66664	NaN	0,0692539	-0,991043	NaN	NaN	NaN
0,234514	-0,24951	0,71236	1,97042	0,787647	NaN	0,983753	0,569592	0,234172	0,183839
0,234514	-0,24951	0,71236	1,97042	0,787647	NaN	0,983753	0,569592	0,234172	0,183839
NaN	NaN	NaN	1,53561	-1,58946	2,61538	-0,723095	-1,19238	0,804027	1,76504
NaN	NaN	NaN	1,53561	-1,58946	2,61538	NaN	NaN	0,804027	1,76504
-0,67934	-1,31833	-1,80873	NaN	-0,623751	NaN	NaN	NaN	NaN	NaN
-0,67934	-1,31833	-1,80873	NaN	-0,623751	NaN	NaN	NaN	NaN	NaN
3,61556	3,33657	3,67422	3,46738	3,32102	2,43186	3,31658	3,94262	3,62939	3,77252
3,61556	3,33657	3,67422	3,46738	3,32102	2,43186	3,31658	3,94262	3,62939	3,77252
NaN	NaN	NaN	-0,579128	NaN	-0,540117	-0,660789	NaN	NaN	NaN
0,248318	-0,551594	0,843948	0,413052	NaN	0,480059	-0,00156403	-0,39991	0,237029	-0,234132
-0,610521	-1,00974	-1,20069	0,159374	-1,04367	NaN	-0,307541	-0,636286	-0,880914	-0,740767
-1,27113	-2,20267	-0,437115	0,221125	-1,28747	-2,18392	-0,117949	-0,434786	0,183152	-0,534705
NaN	NaN	NaN	-1,09687	-2,62214	NaN	-1,52317	NaN	NaN	NaN
NaN	NaN	NaN	-1,09687	-2,62214	NaN	-1,52317	NaN	NaN	NaN
NaN	NaN	NaN	-0,702509	-2,63844	-1,40886	-1,06715	NaN	NaN	NaN
NaN	NaN	NaN	-0,702509	-2,63844	NaN	-1,06715	NaN	NaN	NaN
1,51727	1,58498	1,05993	2,7824	1,59174	-3,52835	2,22062	1,17532	1,16814	1,48843
1,51727	1,58498	1,05993	2,7824	1,59174	-3,52835	2,22062	1,17532	1,16814	1,48843
-0,540966	0,32999	NaN	0,181135	NaN	0,911797	0,477999	NaN	0,301717	-0,646113
1,16952	NaN	NaN	0,877119	1,97704	0,911797	NaN	NaN	NaN	NaN
0,159401	0,527508	-0,329243	-0,139874	0,957045	NaN	NaN	-0,560577	-0,264384	-0,0466318

Polito et al. Universal ready-to-use immunotherapeutic approach for the treatment of cancer: expanded and activated polyclonal γδ memory T cells.
Phosphorylation sites

NaN	NaN	NaN	NaN	NaN	NaN	NaN	NaN	NaN	NaN
NaN	NaN	NaN	NaN	NaN	NaN	NaN	NaN	NaN	NaN
NaN	NaN	NaN	-1,69	-0,351109	NaN	-1,08842	NaN	NaN	NaN
NaN	NaN	NaN	-1,69	-0,351109	NaN	-1,08842	NaN	NaN	NaN
0,190128	-0,523779	-0,358946	NaN	-0,402422	NaN	-1,5969	-1,35929	-1,15516	-0,785914
-0,668941	NaN	-1,97894	0,303522	-0,316282	0,766079	-0,72986	-0,927689	-0,856755	-1,57701
-0,668941	NaN	-1,97894	0,303522	-0,316282	0,766079	-0,72986	-0,927689	-0,856755	-1,57701
NaN	NaN	NaN	-1,29738	NaN	-0,0961246	-1,18526	NaN	NaN	NaN
NaN	NaN	NaN	-1,29738	NaN	-0,0961246	-1,18526	NaN	NaN	NaN
-1,58632	-0,187489	NaN	0,936914	-1,18553	0,898037	0,299633	NaN	NaN	NaN
NaN	NaN	NaN	0,936914	-1,18553	0,898037	0,299633	NaN	NaN	NaN
1,63621	0,947023	0,546986	1,4839	0,653976	NaN	NaN	0,246243	0,543948	0,0729933
1,63621	0,947023	0,546986	1,4839	0,653976	NaN	0,587866	0,246243	0,543948	0,0729933
NaN	NaN	NaN	-1,96031	NaN	-1,46889	-2,43298	NaN	NaN	NaN
NaN	-2,00645	NaN	-0,444508	-1,09152	NaN	-0,429426	NaN	NaN	NaN
NaN	-2,00645	NaN	-0,444508	-1,09152	NaN	-0,429426	NaN	NaN	NaN
NaN	NaN	NaN	-0,718904	-2,31586	-0,493095	-0,881716	NaN	NaN	NaN
NaN	NaN	NaN	NaN	NaN	NaN	NaN	NaN	NaN	NaN
-1,65911	-1,39459	-1,94638	-1,40717	-1,32174	NaN	-0,396431	-1,42668	-2,05845	-1,94421
-1,65911	-1,39459	-1,94638	-1,40717	-1,32174	NaN	-0,396431	-1,42668	-2,05845	-1,94421
1,16405	1,33765	1,97249	0,321772	1,63028	-1,26662	0,395033	1,56273	1,53845	1,39195
1,16405	1,33765	1,97249	0,321772	1,63028	-1,26662	0,395033	1,56273	1,53845	1,39195
NaN	NaN	NaN	NaN	NaN	NaN	NaN	NaN	NaN	NaN
0,308184	1,17986	NaN	-0,832615	1,53498	-1,10172	0,768442	0,797892	0,99332	0,650476
-1,19322	-1,54558	NaN	-1,68066	-1,8069	-0,778214	-1,62185	-2,16685	-2,06461	-2,31324
-1,19322	-1,54558	NaN	-1,68066	-1,8069	-0,778214	-1,62185	-2,16685	-2,06461	-2,31324
-0,17252	-0,882862	-0,936129	-1,66076	-1,01173	-0,00215721	-1,01957	-1,84154	-1,52786	-0,188174
-0,17252	-0,882862	-0,936129	-1,66076	-1,01173	-0,00215721	-1,01957	-1,84154	-1,52786	-0,188174
1,3703	NaN	0,0943108	0,145498	NaN	-0,953262	-0,723795	0,101873	0,485734	-1,26243
1,17417	-0,524899	1,48273	2,47434	1,012	2,44283	2,86378	1,90214	1,84776	1,32995
1,17417	-0,524899	1,48273	2,47434	1,012	2,44283	2,86378	1,90214	1,84776	1,32995
0,956013	0,491062	1,80074	0,65102	1,25283	0,951933	-1,74701	2,19474	2,3686	1,60149
0,956013	0,491062	1,80074	0,65102	1,25283	0,951933	-1,74701	2,19474	2,3686	1,60149
NaN	NaN	NaN	-0,587252	-1,90827	NaN	-0,52035	NaN	NaN	NaN

Polito et al. Universal ready-to-use immunotherapeutic approach for the treatment of cancer: expanded and activated polyclonal γδ memory T cells.
Phosphorylation sites

NaN	NaN	NaN	-0,587252	-1,90827	NaN	-0,52035	NaN	NaN	NaN
NaN	NaN	NaN	-0,413671	NaN	-0,342035	NaN	NaN	NaN	NaN
NaN	NaN	NaN	-0,413671	NaN	-0,342035	NaN	NaN	NaN	NaN
NaN	NaN	NaN	1,29845	NaN	1,11473	-0,255819	NaN	NaN	NaN
NaN	NaN	NaN	1,29845	NaN	1,11473	-0,255819	NaN	NaN	NaN
-0,0465908	1,82654	1,0582	1,82463	0,842699	2,79886	2,5827	1,02311	1,09561	1,8154
-1,49441	-2,04637	-1,90032	-1,71115	-1,07023	NaN	NaN	NaN	NaN	NaN
-1,49441	-2,04637	-1,90032	-1,71115	-1,07023	NaN	NaN	NaN	NaN	NaN
0,0929394	-0,263281	0,134296	0,531479	0,83424	0,0831223	0,971611	0,777607	0,645638	0,871293
1,01794	-0,359854	0,942751	1,13681	1,36083	0,763609	1,79219	1,46167	1,41383	1,75304
1,01794	-0,359854	0,942751	1,13681	1,36083	0,763609	1,79219	1,46167	1,41383	1,75304
-1,55693	-1,28724	-1,08709	-0,78363	-1,20022	NaN	-1,42366	NaN	-0,595977	-1,46292
0,402269	0,0920734	-1,04756	-0,54488	-0,297047	NaN	-0,45294	-1,47836	NaN	-0,95935
0,402269	0,0920734	-1,04756	-0,54488	-0,297047	NaN	-0,45294	-1,47836	NaN	-0,95935
0,163712	1,59192	NaN	0,597113	NaN	1,73052	0,758307	NaN	NaN	0,223277
0,163712	1,59192	NaN	0,597113	NaN	1,73052	0,758307	NaN	NaN	NaN
1,2865	0,904381	1,35812	2,12228	0,591194	3,35557	1,35651	0,998102	0,736453	0,0639334
1,2865	0,904381	1,35812	2,12228	0,591194	3,35557	1,35651	0,998102	0,736453	0,0639334
NaN	NaN	NaN	-1,09444	-2,3981	-0,896658	-1,36965	NaN	NaN	NaN
-1,58291	-1,97194	-0,894909	-0,77153	-1,73094	0,00943756	NaN	NaN	NaN	NaN
NaN	-1,45571	-0,608437	NaN	NaN	NaN	NaN	-0,911272	-0,827054	-1,1507
NaN	-1,45571	-0,608437	NaN	NaN	NaN	NaN	-0,911272	-0,827054	-1,1507
-1,02395	NaN	-1,32991	NaN	NaN	-0,247087	NaN	NaN	NaN	NaN
-1,02395	NaN	-1,32991	NaN	NaN	-0,247087	NaN	NaN	NaN	NaN
0,394751	-0,531076	-1,01695	1,20554	-0,21242	2,35463	1,21206	1,25168	1,301	0,770772
NaN	NaN	NaN	-2,43354	-2,93799	NaN	-2,16453	NaN	NaN	-1,98215
0,394751	-0,531076	-1,01695	1,20554	-0,21242	2,35463	1,21206	1,25168	1,301	0,770772
NaN	NaN	NaN	NaN	NaN	NaN	NaN	NaN	NaN	NaN
-1,17355	-1,46231	NaN	0,552563	-1,09778	NaN	-0,618729	-0,698555	-1,10203	-1,22075
-1,17355	-1,46231	NaN	0,552563	-1,09778	NaN	-0,618729	-0,698555	-1,10203	-1,22075
-0,522799	-1,38451	-0,393696	-1,75205	-0,561792	-0,562365	-1,15305	-0,761856	-1,07516	-0,582395
-0,522799	-1,38451	-0,393696	-1,75205	-0,561792	-0,562365	-1,15305	-0,761856	-1,07516	-0,582395
NaN	NaN	NaN	NaN	NaN	NaN	NaN	NaN	NaN	NaN
NaN	NaN	NaN	NaN	NaN	NaN	NaN	NaN	NaN	NaN

Polito et al. Universal ready-to-use immunotherapeutic approach for the treatment of cancer: expanded and activated polyclonal γδ memory T cells.
Phosphorylation sites

NaN	NaN	NaN	NaN	NaN	NaN	NaN	NaN	NaN	NaN
NaN	NaN	NaN	NaN	NaN	NaN	NaN	NaN	NaN	NaN
-0,681166	-0,673906	-2,21587	-1,0977	-1,07143	-0,538584	-0,589315	NaN	NaN	NaN
-0,681166	-0,673906	-2,21587	-1,0977	-1,07143	-0,538584	-0,589315	NaN	NaN	NaN
0,272615	-0,451357	-0,133718	-0,359083	NaN	-1,49916	NaN	0,0355511	0,300685	-0,675172
NaN	NaN	-0,133718	NaN	-0,330942	NaN	NaN	0,0355511	0,300685	-0,675172
NaN	NaN	NaN	-3,05279	-2,42394	-1,17754	NaN	NaN	NaN	NaN
NaN	NaN	NaN	NaN	NaN	NaN	NaN	NaN	NaN	NaN
NaN	NaN	NaN	-0,702148	NaN	-0,720917	-1,55093	NaN	NaN	NaN
NaN	NaN	NaN	-0,702148	NaN	-0,720917	-1,55093	NaN	NaN	NaN
NaN	NaN	NaN	-0,656437	0,897863	-0,398949	-0,512072	0,174503	0,189406	0,021945
NaN	NaN	NaN	-0,656437	0,897863	-0,398949	-0,512072	0,174503	0,189406	0,021945
-0,165144	NaN	0,523598	-0,604925	0,340633	NaN	1,36495	0,80319	0,635245	0,300756
-0,165144	NaN	0,523598	-0,604925	0,340633	NaN	1,36495	0,80319	0,635245	0,300756
-0,54207	-2,82401	-1,31417	-1,33783	-0,971985	NaN	-1,30158	-0,811377	-0,992902	-0,575709
-0,54207	-2,82401	-1,31417	-1,33783	-0,971985	NaN	-1,30158	-0,811377	-0,992902	-0,575709
NaN	NaN	NaN	-0,0795422	-0,758238	-0,193073	-0,2974	NaN	NaN	NaN
-0,86639	-6,1739	-1,1524	-0,494858	0,407305	-1,88504	-0,208031	0,614643	-0,29803	-0,311534
-0,86639	-6,1739	-1,1524	-0,494858	0,407305	-1,88504	-0,208031	0,614643	-0,29803	-0,311534
1,09958	1,5505	0,901476	0,728394	1,57418	-0,50902	1,60924	0,851404	1,46599	1,61122
1,09958	1,5505	0,901476	0,728394	1,57418	-0,50902	1,60924	0,851404	1,46599	1,61122
-0,186981	0,399075	NaN	NaN	-0,447849	-3,08701	NaN	NaN	NaN	NaN
-0,186981	0,399075	NaN	NaN	-0,447849	-3,08701	NaN	NaN	NaN	NaN
0,505936	-0,0981522	-0,205153	-0,288795	1,15344	-1,91175	0,922346	0,312876	0,0180368	0,884656
0,505936	-0,0981522	-0,205153	-0,288795	1,15344	-1,91175	0,922346	0,312876	0,0180368	0,884656
2,72602	3,08811	3,0169	3,00098	3,00696	0,159033	3,31438	2,62451	2,65312	2,70641
2,72602	3,08811	3,0169	3,00098	3,00696	0,159033	3,31438	2,62451	2,65312	2,70641
1,64223	1,57612	1,35688	0,744173	0,729897	0,782961	1,03722	1,37162	1,56462	0,852098
1,64223	1,57612	1,35688	0,744173	0,729897	0,782961	1,03722	1,37162	1,56462	0,852098
3,5538	2,51219	2,56179	2,85841	2,58683	-0,0174809	2,80267	2,4675	2,81228	2,40488
3,5538	2,51219	2,56179	2,85841	2,58683	-0,0174809	2,80267	2,4675	2,81228	2,40488
-0,316521	-1,12047	-1,10189	0,222464	-1,10235	NaN	-0,885839	NaN	-1,15001	NaN
-1,00228	-0,165308	-0,666397	1,01758	NaN	0,853168	-0,433916	NaN	NaN	NaN
0,754448	0,171791	0,386156	-0,00471306	0,27471	1,17849	-0,204086	0,209978	0,000144005	-0,220485

Polito et al. Universal ready-to-use immunotherapeutic approach for the treatment of cancer: expanded and activated polyclonal γδ memory T cells.
Phosphorylation sites

0,754448	0,171791	0,386156	-0,00471306	0,27471	1,17849	-0,204086	0,209978	0,000144005	-0,220485
-2,00842	NaN	-1,80303	-0,480885	-2,47546	NaN	-0,996384	NaN	NaN	NaN
-2,00842	NaN	-1,80303	-0,480885	-2,47546	NaN	-0,996384	NaN	NaN	NaN
-0,191103	-0,103884	NaN	NaN	NaN	NaN	NaN	-0,814356	-0,340453	-0,708684
-0,191103	-0,103884	NaN	NaN	NaN	NaN	NaN	-0,814356	-0,340453	-0,708684
NaN	NaN	NaN	NaN	NaN	NaN	NaN	NaN	NaN	NaN
NaN	NaN	NaN	NaN	NaN	NaN	NaN	NaN	NaN	NaN
-1,19439	-1,14837	0,638578	-1,15914	0,261845	NaN	0,384886	1,0374	0,658299	1,39492
0,203487	-0,0439548	-0,310589	2,95459	0,607727	3,57828	1,95925	-0,505045	NaN	-0,690719
0,203487	-0,0439548	-0,310589	2,95459	0,607727	3,57828	1,95925	-0,505045	NaN	-0,690719
NaN	NaN	NaN	4,07114	-0,0545292	4,07775	1,32661	NaN	NaN	NaN
NaN	NaN	NaN	4,07114	-0,0545292	4,07775	1,32661	NaN	NaN	NaN
0,413406	-0,799997	-1,02107	NaN	NaN	1,02336	NaN	NaN	NaN	NaN
0,194075	-2,90128	-0,680426	-1,0933	-0,946501	-3,02756	-0,799915	0,0316505	-0,622254	-0,871076
0,194075	-2,90128	-0,680426	-1,0933	-0,946501	-3,02756	-0,799915	0,0316505	-0,622254	-0,871076
NaN	NaN	NaN	-1,19152	NaN	NaN	-1,02161	NaN	NaN	NaN
NaN	NaN	NaN	-1,19152	NaN	NaN	-1,02161	NaN	NaN	NaN
-1,4398	NaN	-1,25885	NaN	NaN	NaN	NaN	-1,36963	-1,26734	-1,60522
-1,4398	NaN	-1,25885	-1,7073	-1,82502	-2,6962	-1,59393	-1,36963	-1,26734	-1,60522
-1,60298	-2,17201	-2,17251	-2,20701	-0,993221	-1,56274	-1,96037	NaN	NaN	NaN
-1,60298	-2,17201	-2,17251	-2,20701	-0,993221	-1,56274	-1,96037	-2,46666	-1,38384	NaN
NaN	-0,206993	0,112169	NaN	0,902449	NaN	1,00829	NaN	-1,713	0,875052
NaN	-0,206993	0,112169	0,0727482	0,902449	-2,90178	1,00829	NaN	-1,713	0,875052
NaN	NaN	NaN	0,469769	-1,32203	1,28136	NaN	NaN	NaN	NaN
NaN	NaN	NaN	0,469769	-1,32203	1,28136	NaN	NaN	NaN	NaN
2,98917	2,4594	1,0743	-0,703976	3,27476	-1,1687	-0,265137	1,96245	1,42097	1,0839
2,98917	2,4594	1,0743	-0,703976	3,27476	-1,1687	-0,265137	1,96245	1,42097	1,0839
-0,415396	-2,01077	-1,80347	-0,617167	NaN	1,02017	-0,327705	NaN	NaN	NaN
-0,415396	-2,01077	-1,80347	-0,617167	NaN	1,02017	-0,327705	NaN	NaN	NaN
NaN	NaN	NaN	-1,25412	-1,40988	-0,900612	-1,5128	NaN	NaN	NaN
NaN	NaN	NaN	-1,25412	-1,40988	-0,900612	-1,5128	NaN	NaN	NaN
NaN	NaN	NaN	-0,0944576	NaN	-0,908329	-1,42612	NaN	NaN	NaN
NaN	NaN	NaN	-0,0944576	NaN	-0,908329	-1,42612	NaN	NaN	NaN
NaN	0,213634	-1,00376	-0,439415	NaN	0,356432	-0,775724	NaN	NaN	NaN

Polito et al. Universal ready-to-use immunotherapeutic approach for the treatment of cancer: expanded and activated polyclonal γδ memory T cells.
Phosphorylation sites

NaN	0,213634	-1,00376	-0,439415	NaN	0,356432	-0,775724	NaN	NaN	NaN
2,18128	1,96425	1,14348	1,37408	1,6809	1,62631	1,57739	0,981903	1,58855	0,889123
2,18128	1,96425	1,14348	1,37408	1,6809	1,62631	1,57739	0,981903	1,58855	0,889123
2,11089	1,96025	1,30103	2,8325	1,50795	1,78167	2,56328	1,62931	1,07434	1,41709
-0,199638	NaN	-1,94549	-0,12158	NaN	NaN	-0,879988	-0,572731	-1,10463	-2,09832
-1,98031	-0,92984	-3,04164	NaN	NaN	NaN	NaN	NaN	NaN	NaN
-0,456244	-0,755529	-1,58736	-0,46423	-0,153675	-1,20775	-0,678099	-0,696451	-1,82968	-1,19063
-0,456244	-0,755529	-1,58736	-0,46423	-0,153675	-1,20775	-0,678099	-0,696451	-1,82968	-1,19063
-0,747892	NaN	NaN	0,164797	-1,769	-1,03756	-0,699024	NaN	NaN	NaN
-0,747892	NaN	NaN	NaN	-1,769	-1,03756	NaN	NaN	NaN	NaN
NaN	NaN	NaN	-1,26207	-1,75329	-0,688921	NaN	NaN	NaN	NaN
-0,59264	-0,199389	NaN	0,271458	-1,26735	-0,509872	-0,589079	-0,915264	NaN	-1,23343
-2,41055	-1,2027	-3,30181	NaN	NaN	NaN	NaN	NaN	NaN	NaN
1,28951	1,91462	0,89431	0,911827	0,97047	0,241627	1,21022	-0,104084	NaN	0,649699
1,28951	1,91462	0,89431	0,911827	0,97047	0,241627	1,21022	-0,104084	NaN	0,649699
NaN	0,0184498	NaN	-0,229769	2,7253	NaN	0,404417	NaN	NaN	NaN
NaN	0,0184498	NaN	-0,229769	2,7253	NaN	0,404417	NaN	NaN	NaN
NaN	NaN	NaN	-1,61618	-2,44532	-1,219	-1,25619	NaN	NaN	NaN
NaN	NaN	NaN	-1,61618	-2,44532	-1,219	-1,25619	NaN	NaN	NaN
0,335629	0,895699	0,229176	0,95039	1,41398	1,26908	0,934792	-0,301859	-0,552676	1,17067
NaN	0,895699	NaN	0,95039	1,41398	NaN	0,934792	-0,301859	NaN	NaN
-0,997637	-1,44409	-1,91471	-0,728466	-1,88551	0,446772	-0,145863	-1,38043	-0,695956	-1,3508
-0,997637	-1,44409	-1,91471	-0,728466	-1,88551	0,446772	-0,145863	-1,38043	-0,695956	-1,3508
NaN	1,94973	NaN	NaN	NaN	NaN	NaN	NaN	NaN	NaN
0,12829	1,94973	0,747505	NaN	NaN	NaN	NaN	-1,55992	0,850377	0,34327
NaN	NaN	NaN	-0,603527	NaN	-1,00345	-1,6282	NaN	NaN	NaN
NaN	NaN	NaN	-0,603527	NaN	-1,00345	-1,6282	NaN	NaN	NaN
NaN	2,25932	NaN	0,583805	1,19116	1,45138	0,428146	1,81805	1,92642	1,79868
0,942255	2,25932	NaN	0,583805	1,19116	1,45138	NaN	1,81805	1,92642	NaN
2,37004	NaN	2,10817	2,64624	NaN	1,10278	2,54057	1,34614	1,48017	1,02952
2,37004	NaN	2,10817	2,64624	NaN	1,10278	2,54057	1,34614	1,48017	1,02952
NaN	NaN	NaN	-1,13065	-1,55055	-1,96793	-1,00434	NaN	NaN	NaN
NaN	NaN	NaN	-1,13065	-1,55055	-1,96793	-1,00434	NaN	NaN	NaN
-0,126936	-0,30138	-0,340794	1,36419	0,4196	0,990925	1,07037	-1,21757	-0,975495	-0,604381

-0,126936	-0,30138	-0,340794	1,36419	0,4196	0,990925	1,07037	-1,21757	-0,975495	-0,604381
0,168108	-1,26667	-0,300034	0,973602	-1,04978	-1,32232	-1,04808	-1,43428	-0,865884	-2,7586
0,168108	-1,26667	-0,300034	0,973602	-1,04978	-1,32232	-1,04808	-1,43428	-0,865884	-2,7586
-1,20167	-2,66291	-1,16693	-0,21871	-1,4958	-0,338303	-0,857826	-1,44131	-1,52296	-1,93199
-1,20167	-2,66291	-1,16693	-0,21871	-1,4958	-0,338303	-0,857826	-1,44131	-1,52296	-1,93199
1,78476	1,64439	1,58546	2,87218	1,43137	1,87581	1,88023	1,05743	0,93337	0,896663
1,78476	1,64439	1,58546	2,87218	1,43137	1,87581	1,88023	1,05743	0,93337	0,896663
NaN	NaN	NaN	NaN	NaN	0,826183	NaN	NaN	NaN	NaN
1,57107	1,69632	0,48934	2,02879	0,162001	2,74	2,01317	-0,984346	1,2808	0,715383
1,57107	1,69632	0,48934	2,02879	0,162001	2,74	2,01317	-0,984346	1,2808	0,715383
NaN	NaN	NaN	-1,53313	-2,75216	-1,34851	-0,498716	NaN	NaN	NaN
-0,356598	NaN	-0,462709	0,579628	1,13284	NaN	0,912052	-0,0124493	0,763766	0,363069
2,82739	3,28731	1,55484	2,70533	0,915863	-1,57593	3,42467	1,6887	3,37154	2,20213
2,82739	3,28731	1,55484	2,70533	0,915863	-1,57593	3,42467	1,6887	3,37154	2,20213
-1,18006	-1,09017	-1,29256	-0,913025	-1,76465	-1,27718	-1,48358	-1,13402	-1,57316	NaN
-1,18006	-1,09017	-1,29256	-0,913025	-1,76465	-1,27718	-1,48358	-1,13402	-1,57316	NaN
-1,30442	-0,746981	-1,41039	-0,385181	-0,697821	0,0974178	-0,415319	NaN	NaN	NaN
-1,30442	-0,746981	-1,41039	-0,385181	-0,697821	0,0974178	-0,415319	NaN	NaN	NaN
NaN	NaN	NaN	-1,81385	NaN	NaN	-1,41338	NaN	NaN	NaN
NaN	NaN	NaN	-1,81385	NaN	NaN	-1,41338	NaN	NaN	NaN
NaN	0,0216007	-1,05617	-1,24646	-0,63629	-0,34436	-0,144157	NaN	NaN	NaN
NaN	0,0216007	-1,05617	-1,24646	-0,63629	-0,34436	-0,144157	NaN	NaN	NaN
NaN	0,939001	NaN	1,40911	1,30001	1,13855	1,53105	0,949852	-0,532542	1,17515
NaN	NaN	NaN	NaN	NaN	1,13855	NaN	NaN	-0,532542	NaN
NaN	0,939001	NaN	1,40911	1,30001	NaN	1,53105	0,949852	NaN	1,17515
1,55011	NaN	1,64069	1,55469	1,69572	1,52581	1,72834	1,10899	1,00818	1,30861
0,866541	0,647923	-0,0038414	1,17379	-1,00979	0,122183	1,81097	-0,764057	1,31599	0,144059
-1,13643	-2,30673	-2,71154	-1,32788	-1,76185	-1,66779	-1,82586	-1,64449	-2,6007	-1,72695
-1,13643	-2,30673	-2,91152	-1,32788	-1,76185	-1,66779	-1,82586	-1,64449	-2,6007	-1,72695
-1,62394	NaN	-2,71154	NaN	NaN	NaN	NaN	NaN	NaN	NaN
NaN	NaN	NaN	-0,700834	-0,670773	-0,401669	-0,968699	NaN	NaN	NaN
-1,83866	-1,79177	-1,53084	-1,89397	-1,17387	NaN	-1,08591	NaN	NaN	NaN
-1,83866	-1,79177	-1,53084	-1,89397	-1,17387	NaN	-1,08591	NaN	NaN	NaN
NaN	NaN	NaN	NaN	NaN	NaN	NaN	NaN	NaN	-0,819863

Polito et al. Universal ready-to-use immunotherapeutic approach for the treatment of cancer: expanded and activated polyclonal γδ memory T cells.
Phosphorylation sites

NaN	NaN	NaN	NaN	NaN	NaN	NaN	NaN	NaN	-0,819863
0,31509	-0,174314	-0,57061	-0,987207	-0,314407	-0,0430565	-0,584112	NaN	NaN	NaN
0,31509	-0,174314	-0,57061	-0,987207	-0,314407	-0,0430565	-0,584112	NaN	NaN	NaN
NaN	NaN	NaN	1,45891	NaN	2,53069	-0,577429	NaN	NaN	NaN
NaN	NaN	NaN	1,45891	NaN	2,53069	-0,577429	NaN	NaN	NaN
NaN	NaN	NaN	-0,012516	-1,62139	-0,298306	0,998257	NaN	NaN	NaN
NaN	NaN	NaN	-0,012516	-1,62139	-0,298306	0,998257	NaN	NaN	NaN
-0,565598	-0,44791	-1,37692	NaN	NaN	NaN	NaN	-1,95959	-1,94758	-2,41899
-0,565598	-0,44791	-1,37692	NaN	NaN	NaN	NaN	-1,95959	-1,94758	-2,41899
2,62656	3,13337	3,06827	3,00721	5,04856	3,78008	3,96261	2,50963	2,69071	3,40985
2,62656	3,13337	3,06827	3,00721	5,04856	3,78008	3,96261	2,50963	2,69071	3,40985
NaN	NaN	NaN	-2,52962	-2,14428	-1,70994	-1,21639	NaN	NaN	NaN
-0,692015	-1,60045	-1,94198	-1,44761	-0,770651	-0,925798	-1,33755	NaN	NaN	-2,21557
-0,692015	-1,60045	-1,94198	-1,44761	-0,770651	-0,925798	-1,21639	NaN	NaN	-2,21557
NaN	NaN	NaN	NaN	NaN	NaN	NaN	NaN	NaN	NaN
NaN	NaN	NaN	NaN	NaN	NaN	NaN	NaN	NaN	NaN
-0,754656	0,320541	-1,67914	-0,189091	NaN	-0,0649529	-0,574863	NaN	-0,496102	NaN
-0,754656	0,320541	NaN	-0,189091	NaN	-0,0649529	-0,574863	NaN	-0,496102	-1,33635
NaN	NaN	NaN	0,31403	NaN	-0,798851	-1,3944	NaN	NaN	NaN
1,80169	2,90273	1,138	0,375004	1,77596	NaN	0,321276	0,791853	1,94827	1,39335
4,52886	5,15303	3,83112	2,00938	5,0736	-1,16965	3,40203	4,28451	4,85692	4,94571
NaN	NaN	NaN	-1,56808	-2,51379	-1,79716	-2,134	NaN	NaN	NaN
NaN	NaN	NaN	-1,56808	-2,51379	-1,79716	-2,134	NaN	NaN	NaN
-3,21408	NaN	-2,75996	NaN	NaN	NaN	NaN	-1,68273	-1,7372	-1,85476
-3,21408	NaN	-2,75996	NaN	NaN	NaN	NaN	-1,68273	-1,7372	-1,85476
-0,159323	-0,263262	-0,131393	0,4053	-0,215651	NaN	0,0466805	0,10943	0,274304	-0,136933
-0,159323	-0,263262	-0,131393	0,4053	-0,215651	NaN	0,0466805	0,10943	0,274304	-0,136933
-0,0588379	-0,520512	-0,655739	0,181702	-0,0620785	-1,17297	0,0863934	-0,741987	-1,1129	-1,05029
-0,0588379	-0,520512	-0,655739	0,181702	-0,0620785	-1,17297	0,0863934	-0,741987	-1,1129	-1,05029
1,47743	0,245785	0,528765	0,404188	0,996983	-0,966784	-0,296602	0,709671	0,486993	1,3424
NaN	0,661972	NaN	-0,00966454	NaN	-1,77682	1,08824	0,979881	NaN	NaN
1,71427	0,661972	1,16906	0,404188	1,78316	-0,966784	1,08824	0,979881	0,968379	1,37789
NaN	NaN	NaN	-1,11344	NaN	-0,852606	-0,598118	NaN	NaN	NaN
NaN	NaN	NaN	-1,11344	NaN	-0,852606	-0,598118	NaN	NaN	NaN

Polito et al. Universal ready-to-use immunotherapeutic approach for the treatment of cancer: expanded and activated polyclonal γδ memory T cells.
Phosphorylation sites

NaN	NaN	NaN	-0,868814	NaN	-0,80077	-1,57137	NaN	NaN	NaN
NaN	NaN	NaN	-0,868814	NaN	-0,80077	-1,57137	NaN	NaN	NaN
0,402168	0,930117	NaN	0,117458	0,22337	0,963226	1,22435	-0,0235367	0,839644	0,51472
NaN	NaN	NaN	0,593826	0,313595	1,05011	0,33548	NaN	0,134994	NaN
-0,667559	-0,29657	-0,349518	0,449585	0,152721	-2,61592	-0,527838	-0,804434	-0,32812	0,651746
-0,667559	-0,29657	-0,349518	0,449585	0,152721	-2,61592	-0,527838	-0,804434	-0,32812	0,651746
0,292105	-1,27845	0,631754	NaN	NaN	-0,416979	NaN	NaN	NaN	NaN
0,292105	-1,27845	NaN	NaN	-0,134043	0,275087	0,107517	0,997978	NaN	1,63321
NaN	NaN	NaN	-0,816532	-1,29666	-0,74337	0,159485	NaN	NaN	-1,5486
NaN	NaN	NaN	-0,816532	-1,29666	-0,74337	0,159485	NaN	NaN	NaN
NaN	NaN	NaN	-0,759218	NaN	-1,22524	-0,234158	NaN	NaN	NaN
NaN	NaN	NaN	-0,759218	NaN	-1,22524	-0,234158	NaN	NaN	NaN
NaN	4,06096	NaN	2,91639	2,14568	2,9276	3,85921	1,54899	3,47075	2,88422
NaN	4,06096	NaN	2,91639	2,14568	NaN	3,85921	1,54899	NaN	2,88422
-0,715755	NaN	0,793356	NaN	-0,846178	-0,0572014	NaN	0,479851	0,320479	0,0743227
-0,715755	NaN	0,793356	NaN	-0,846178	-0,0572014	NaN	0,479851	0,320479	0,0743227
3,33495	2,9783	2,88039	2,85058	2,51955	0,0614662	3,63088	3,44864	2,9387	2,78918
3,33495	2,9783	2,88039	2,85058	2,51955	0,0614662	3,63088	3,44864	2,9387	2,78918
-1,84972	1,53392	NaN	-0,678656	-0,800251	0,134462	0,893164	NaN	NaN	NaN
-1,84972	1,53392	NaN	-0,678656	-0,800251	0,134462	0,893164	NaN	NaN	NaN
2,51423	2,56126	NaN	1,56087	2,20337	1,09231	2,02985	0,810036	2,04607	NaN
2,51423	2,56126	NaN	NaN	2,20337	1,09231	2,02985	0,810036	2,04607	NaN
0,157701	-0,970045	-1,54958	NaN	NaN	NaN	NaN	NaN	NaN	NaN
0,157701	-0,970045	-1,54958	NaN	-0,0337734	-2,70524	-1,69146	NaN	NaN	NaN
1,00132	-0,2192	1,47726	NaN	3,17699	0,424534	1,09349	0,640587	-1,59654	2,46252
1,00132	-0,2192	1,47726	NaN	3,17699	0,424534	1,09349	0,640587	-1,59654	2,46252
NaN	NaN	NaN	0,272598	0,218199	-0,499857	2,06071	-0,888098	0,0540018	-0,522622
NaN	NaN	NaN	0,272598	0,218199	-0,499857	2,06071	-0,888098	0,0540018	-0,522622
1,3055	3,4648	0,434402	1,35013	1,12152	1,5564	2,12979	0,279379	1,49851	0,698371
1,3055	3,4648	0,434402	1,35013	1,12152	1,5564	2,12979	0,279379	1,49851	0,698371
-1,0733	-0,628765	NaN	NaN	NaN	NaN	NaN	-2,43462	-1,62352	-2,23564
-1,0733	-0,628765	NaN	NaN	-1,60253	-1,1422	-2,09233	-2,43462	-1,62352	-2,23564
0,0104294	1,00577	-0,386917	0,0349236	0,900684	-0,649044	NaN	NaN	NaN	NaN
0,0104294	1,00577	-0,386917	0,0349236	0,900684	-0,649044	NaN	NaN	NaN	NaN

Polito et al. Universal ready-to-use immunotherapeutic approach for the treatment of cancer: expanded and activated polyclonal γδ memory T cells.
Phosphorylation sites

NaN	NaN	NaN	NaN	NaN	NaN	NaN	-1,94713	-2,00084	-2,44213
NaN	NaN	NaN	NaN	NaN	NaN	NaN	-1,94713	-2,00084	-2,44213
2,23608	1,5261	1,61704	2,92273	3,09091	2,26248	1,35668	1,71264	1,09235	1,59033
2,23608	1,5261	1,61704	2,92273	3,09091	2,26248	1,35668	1,71264	1,09235	1,59033
-2,24725	-1,85219	-2,05449	-1,60747	-2,15159	-1,55783	-1,21572	NaN	NaN	NaN
-2,24725	-1,85219	-2,05449	-1,60747	-2,15159	-1,55783	-1,21572	NaN	NaN	NaN
-0,529581	-0,259863	-0,986391	NaN	NaN	NaN	NaN	-0,659342	-0,780337	-0,68403
-0,529581	-0,259863	-0,986391	NaN	NaN	NaN	NaN	-0,659342	-0,780337	-0,68403
-1,43823	-0,745283	-1,20679	-0,71839	-1,16881	0,664497	-2,74778	-0,943815	-1,10498	-0,458528
-1,43823	-0,745283	-1,20679	-0,71839	-1,16881	0,664497	-0,0760975	-0,943815	-1,10498	-0,458528
-1,08358	-0,627224	-0,619432	0,0379715	-0,138073	0,918848	0,634359	-0,754011	-0,640426	-1,08555
3,88554	2,59625	3,63645	3,47396	2,81918	4,16002	3,0385	3,40471	3,30626	4,23221
3,88554	2,59625	3,63645	3,47396	2,81918	4,16002	3,0385	3,40471	3,30626	4,23221
NaN	NaN	NaN	-1,1675	NaN	-0,307781	NaN	NaN	NaN	NaN
-0,83934	-0,161592	-0,399107	0,185472	-0,144659	0,242233	-0,207644	-0,916348	-1,29089	-0,140294
NaN	-0,853657	-0,743011	-0,230696	-0,702808	-0,0638256	NaN	-1,57589	-2,47189	-0,695357
NaN	-0,853657	-0,743011	-0,230696	-0,702808	-0,0638256	NaN	-1,57589	-2,47189	-0,695357
-1,51283	-1,25837	-1,50263	0,310862	-0,82185	-1,16236	-0,123999	-1,13802	-2,06213	-2,16283
-1,51283	-1,25837	-1,50263	0,310862	-0,82185	-1,16236	-0,123999	-1,13802	-2,06213	-2,16283
NaN	NaN	NaN	-0,0576801	-0,418558	-0,967791	-0,881964	NaN	NaN	NaN
-1,20367	-2,32043	-1,73795	-1,81987	-1,92146	-1,6467	-1,83237	NaN	NaN	NaN
-1,20367	-2,32043	-1,73795	-1,81987	-1,92146	-1,6467	-1,83237	NaN	NaN	NaN
-0,931223	-0,753847	-1,58507	-1,27283	-0,852634	0,427744	-0,908564	NaN	NaN	NaN
-0,931223	-0,753847	-1,58507	-1,27283	-0,852634	0,427744	-0,908564	NaN	NaN	NaN
-0,351833	0,0871105	-0,960711	-0,292257	-0,426638	0,418629	NaN	NaN	NaN	NaN
-0,351833	0,0871105	-0,960711	-0,292257	-0,426638	0,418629	NaN	NaN	NaN	NaN
NaN	NaN	NaN	NaN	NaN	NaN	NaN	NaN	NaN	NaN
NaN	NaN	NaN	NaN	NaN	NaN	NaN	NaN	NaN	NaN
NaN	NaN	NaN	-1,26155	-1,63589	-1,15404	NaN	NaN	NaN	NaN
NaN	NaN	NaN	-1,26155	-1,63589	-1,15404	NaN	NaN	NaN	NaN
NaN	NaN	NaN	NaN	NaN	NaN	NaN	NaN	NaN	NaN
NaN	NaN	NaN	1,32833	NaN	2,24719	0,893164	NaN	NaN	NaN
NaN	NaN	NaN	1,32833	NaN	2,24719	0,893164	NaN	NaN	NaN
NaN	NaN	NaN	0,122076	NaN	1,04198	-1,18141	NaN	NaN	NaN

Polito et al. Universal ready-to-use immunotherapeutic approach for the treatment of cancer: expanded and activated polyclonal γδ memory T cells.
Phosphorylation sites

-1,64934	-0,0871887	-2,06936	-1,56323	-1,16932	-2,5289	-1,21281	-2,06106	-1,31426	-2,1189
-1,64934	-0,0871887	-2,06936	-1,56323	-1,16932	-2,5289	-1,21281	-2,06106	-1,31426	-2,1189
NaN	0,86908	-0,862753	0,824738	NaN	1,0003	-0,241272	NaN	0,290811	NaN
1,44098	NaN	-0,862753	NaN	NaN	NaN	-0,241272	NaN	0,290811	NaN
1,44098	0,86908	NaN	0,824738	NaN	1,0003	-0,241272	NaN	NaN	NaN
NaN	NaN	NaN	NaN	NaN	NaN	NaN	NaN	NaN	NaN
NaN	NaN	NaN	NaN	NaN	NaN	NaN	NaN	NaN	NaN
NaN	NaN	NaN	-2,16527	-0,931997	-3,20792	-2,45083	-1,35488	-1,18055	-1,65032
NaN	NaN	NaN	-2,16527	-0,931997	-3,20792	-2,45083	-1,35488	-1,18055	-1,65032
NaN	NaN	NaN	-2,06364	-3,01103	NaN	-1,74874	NaN	NaN	NaN
NaN	NaN	NaN	-2,06364	-3,01103	NaN	-1,74874	NaN	NaN	NaN
0,0660934	1,40273	-0,143049	1,08793	-1,23225	2,52118	1,19293	0,964241	0,953101	0,572282
0,0660934	1,40273	-0,143049	1,08793	-1,23225	2,52118	1,19293	0,964241	0,953101	0,572282
0,0686283	-0,640715	-0,655205	0,732603	0,22315	-2,69869	0,7876	-0,459679	-0,745055	-0,657363
0,0686283	-0,640715	-0,655205	0,732603	0,22315	-2,69869	0,7876	-0,459679	-0,745055	-0,657363
3,80704	5,04416	3,82192	2,64759	4,74399	4,87817	3,47516	3,00309	3,81766	4,07074
3,80704	5,04416	3,82192	2,64759	4,74399	4,87817	3,47516	3,00309	3,81766	4,07074
NaN	NaN	NaN	NaN	NaN	NaN	-0,874979	-2,23531	-1,02877	NaN
NaN	NaN	NaN	NaN	NaN	NaN	-0,874979	-2,23531	-1,02877	NaN
2,44007	2,14777	2,60643	2,19759	3,39896	2,99096	2,50097	1,20603	2,86261	2,39614
2,44007	2,14777	2,60643	2,19759	3,39896	2,99096	2,50097	1,20603	2,86261	2,39614
1,25533	0,827372	0,852425	1,05601	1,21045	1,33251	1,03667	0,677237	0,320725	0,190932
1,25533	0,827372	0,852425	1,05601	1,21045	1,33251	1,03667	0,677237	0,320725	0,190932
-0,191103	0,456171	-2,00993	0,284512	0,765656	-0,0375118	0,760946	NaN	NaN	NaN
-0,191103	0,456171	-2,00993	0,284512	0,765656	NaN	0,760946	NaN	NaN	NaN
NaN	NaN	NaN	NaN	NaN	NaN	NaN	-1,85015	-0,793572	-1,64787
NaN	NaN	NaN	1,0728	0,742714	0,481176	0,701998	0,241461	0,506269	0,260383
0,708063	NaN	-0,393696	1,0728	0,742714	0,481176	0,701998	0,241461	0,506269	0,260383
NaN	NaN	NaN	NaN	NaN	NaN	NaN	NaN	NaN	NaN
NaN	NaN	NaN	NaN	NaN	NaN	NaN	NaN	NaN	NaN
NaN	NaN	NaN	NaN	NaN	NaN	NaN	NaN	NaN	NaN
NaN	NaN	NaN	NaN	NaN	NaN	NaN	NaN	NaN	NaN
NaN	NaN	NaN	-1,7967	-2,21684	-0,510828	-1,58037	NaN	NaN	NaN
NaN	NaN	NaN	-1,7967	-2,21684	-0,510828	-1,58037	NaN	NaN	NaN

Polito et al. Universal ready-to-use immunotherapeutic approach for the treatment of cancer: expanded and activated polyclonal γδ memory T cells.
Phosphorylation sites

-0,429455	-0,23303	-0,0901279	1,42946	0,0985432	0,576992	0,569792	0,371159	0,699927	NaN
-0,429455	-0,23303	-0,0901279	1,42946	0,0985432	0,576992	0,569792	0,371159	0,699927	NaN
NaN	NaN	NaN	-1,74772	-2,5754	-1,38166	NaN	NaN	NaN	NaN
NaN	NaN	NaN	-1,74772	-2,5754	-1,38166	NaN	NaN	NaN	NaN
NaN	NaN	NaN	-2,56052	-1,88445	NaN	-2,20847	-2,29147	-1,95424	-2,47288
NaN	NaN	NaN	-2,56052	-1,88445	NaN	-2,20847	-2,29147	-1,95424	-2,47288
NaN	NaN	NaN	NaN	-1,10058	-0,384626	-0,381128	NaN	NaN	NaN
NaN	NaN	NaN	NaN	-1,10058	-0,384626	-0,381128	NaN	NaN	NaN
-0,356054	-1,31767	-1,56568	0,0619774	-0,473164	-0,561586	0,239725	-1,29135	-1,59796	-1,46691
-0,356054	-1,31767	-1,56568	0,0619774	-0,473164	-0,561586	0,239725	-1,29135	-1,59796	-1,46691
-2,11451	-1,86196	-1,90214	-1,98972	-1,06517	-1,33481	-1,54316	NaN	NaN	NaN
-2,11451	-1,86196	-1,90214	-1,98972	-1,06517	-1,33481	-1,54316	NaN	NaN	NaN
NaN	NaN	NaN	NaN	-2,31586	-2,19546	-1,17463	NaN	NaN	NaN
NaN	NaN	NaN	NaN	-2,31586	-2,19546	-1,17463	NaN	NaN	NaN
NaN	NaN	NaN	0,198795	NaN	1,12185	NaN	NaN	NaN	NaN
NaN	NaN	NaN	0,198795	NaN	1,12185	NaN	NaN	NaN	NaN
-0,948006	0,830528	-1,47179	-0,494444	-0,112396	-2,34299	-1,72431	-0,787931	-0,447637	-1,25622
NaN	NaN	NaN	-0,494444	-0,112396	-2,34299	-1,72431	-0,787931	-0,447637	-1,25622
-0,948006	0,830528	-1,47179	NaN	NaN	NaN	NaN	NaN	NaN	NaN
NaN	NaN	NaN	-0,992792	NaN	-0,715416	-1,94463	NaN	NaN	NaN
NaN	NaN	NaN	-0,992792	NaN	-0,715416	-1,94463	NaN	NaN	NaN
-2,12772	-0,887695	-2,75137	NaN	NaN	NaN	NaN	NaN	NaN	NaN
-2,12772	-0,887695	-2,75137	NaN	NaN	NaN	NaN	NaN	NaN	NaN
NaN	NaN	NaN	-1,36485	-1,53596	-0,567755	-0,73127	NaN	NaN	NaN
NaN	NaN	NaN	-1,5161	NaN	0,628674	-1,21276	NaN	NaN	NaN
-1,92172	-0,264328	-3,28565	NaN	-1,26072	-2,98852	-2,60844	NaN	NaN	NaN
-1,92172	-0,264328	-3,28565	NaN	-1,26072	-2,98852	-2,60844	NaN	NaN	NaN
0,48098	0,433973	0,0369568	-0,0498009	0,360634	-0,141666	0,362812	0,337719	-0,55343	0,439414
0,48098	0,433973	0,0369568	-0,0498009	0,360634	-0,141666	0,362812	0,337719	-0,55343	0,439414
NaN	NaN	NaN	-1,47924	NaN	1,52427	-1,62351	NaN	NaN	NaN
NaN	NaN	NaN	-1,47924	NaN	1,52427	-1,62351	NaN	NaN	NaN
NaN	NaN	NaN	NaN	NaN	NaN	NaN	NaN	NaN	NaN
NaN	NaN	NaN	NaN	NaN	NaN	NaN	NaN	NaN	NaN
NaN	NaN	NaN	-0,238787	-0,866253	0,885313	-1,84044	NaN	NaN	NaN

NaN	NaN	NaN	-0,238787	-0,866253	0,885313	-1,84044	NaN	NaN	NaN
NaN	-2,99417	2,73829	3,80806	NaN	4,13916	3,45033	0,448353	0,385318	0,237283
NaN	NaN	0,0993462	1,48987	-2,53395	1,92533	0,509846	NaN	NaN	NaN
NaN	-2,99417	2,73829	3,80806	0,32099	4,13916	3,45033	0,448353	0,385318	0,237283
NaN	NaN	NaN	1,80346	NaN	1,06038	0,0106869	NaN	NaN	NaN
NaN	NaN	NaN	1,80346	NaN	1,06038	0,0106869	NaN	NaN	NaN
NaN	NaN	NaN	NaN	NaN	0,848051	NaN	NaN	NaN	NaN
NaN	NaN	NaN	0,51742	0,45224	0,707695	NaN	NaN	-0,66392	-0,26728
NaN	NaN	NaN	-1,0559	-1,40302	-0,619968	-1,75937	NaN	NaN	NaN
NaN	-0,774004	-0,971062	-1,83494	-0,273794	-1,86871	-0,767561	-0,57626	-0,759149	NaN
NaN	-0,774004	-0,971062	-1,83494	-0,273794	-1,86871	-0,767561	-0,57626	-0,759149	NaN
NaN	2,30076	NaN	0,955233	1,27344	1,29393	1,41257	0,0295753	1,55481	-0,00115871
NaN	2,30076	NaN	0,955233	1,27344	1,29393	1,41257	0,0295753	1,55481	-0,00115871
2,13467	2,47714	1,23051	2,26124	1,63312	1,43543	1,18302	0,843225	0,53704	0,367629
-1,33422	-1,63468	-2,0906	-1,99191	-0,654749	NaN	NaN	-2,12577	NaN	-1,26584
NaN	NaN	NaN	-0,897429	-1,69266	0,0132408	-0,342108	NaN	NaN	NaN
NaN	NaN	NaN	-0,897429	-1,69266	0,0132408	-0,342108	NaN	NaN	NaN
-0,281454	-0,0468006	-0,395952	0,112722	0,960381	-0,328053	0,625492	-1,48281	-0,75686	0,418757
-0,281454	-0,0468006	-0,395952	0,112722	0,960381	-0,328053	0,625492	-1,48281	-0,75686	0,418757
2,67891	3,07277	1,80511	2,63737	4,39521	2,29564	2,36208	-0,290619	0,708362	2,53558
2,67891	3,07277	1,80511	2,63737	4,39521	2,29564	2,36208	-0,290619	0,708362	2,53558
0,0935059	NaN	-0,253733	0,91378	0,437031	0,526804	0,824789	-0,720375	-0,248534	0,143624
0,0935059	NaN	-0,253733	0,91378	0,437031	0,526804	0,824789	-0,720375	-0,248534	0,143624
1,04004	0,741833	1,63715	0,940941	1,13828	NaN	1,767	1,37538	1,39485	1,24739
1,04004	0,741833	1,63715	0,940941	1,13828	NaN	1,767	1,37538	1,39485	1,24739
0,108778	NaN	NaN	1,21444	1,56921	-0,572737	0,882658	NaN	NaN	NaN
NaN	NaN	NaN	NaN	NaN	NaN	-1,56059	-0,810457	-1,2045	-0,734225
NaN	NaN	NaN	NaN	NaN	NaN	-1,56059	-0,810457	-1,2045	-0,734225
NaN	NaN	NaN	NaN	NaN	NaN	NaN	-1,81339	-0,892707	-0,694167
NaN	NaN	NaN	NaN	NaN	NaN	NaN	-1,81339	-0,892707	-0,694167
NaN	NaN	NaN	-0,688044	-0,313522	NaN	-1,02525	NaN	NaN	NaN
NaN	NaN	NaN	-0,688044	-0,313522	NaN	-1,02525	NaN	NaN	NaN
-0,405811	-0,14558	NaN	-0,15984	-1,15254	0,0307083	-1,37567	NaN	NaN	NaN
-0,405811	-0,14558	NaN	-0,15984	-1,15254	0,0307083	-1,37567	NaN	NaN	NaN

-1,97495	-2,13685	NaN	-2,82117	-2,35243	-2,41326	-1,89907	-2,58273	NaN	NaN
-1,97495	-2,13685	NaN	-2,82117	-2,35243	-2,41326	-1,89907	-2,58273	NaN	NaN
-0,723364	-2,1828	-1,01596	-0,472572	-0,886871	-0,985809	-0,544559	-2,12664	-1,76155	-0,609694
NaN	1,56184	0,593214	1,09199	1,31094	1,21905	1,31058	0,606754	0,699042	0,296857
NaN	1,56184	0,593214	1,09199	1,31094	1,21905	1,31058	0,606754	0,699042	0,296857
NaN	NaN	NaN	-2,19678	NaN	-2,13578	-3,19439	NaN	NaN	NaN
NaN	NaN	NaN	-2,19678	NaN	-2,13578	-3,19439	NaN	NaN	NaN
-0,0749226	-0,430822	-0,750801	-0,384317	0,948053	0,206747	-0,864403	-0,246454	0,216258	-0,374938
-0,0749226	-0,430822	-0,750801	-0,384317	0,948053	0,206747	-0,864403	-0,246454	0,216258	-0,374938
NaN	NaN	NaN	-0,435406	-0,51306	NaN	-0,094614	-1,29637	-1,23445	-1,75666
0,470919	NaN	-0,508955	1,00902	0,773062	NaN	1,12887	0,142979	NaN	-0,392453
0,470919	-1,12985	-0,508955	1,00902	0,773062	NaN	1,12887	0,142979	-0,115052	-0,392453
0,629589	0,0247269	-0,424202	0,852388	1,16794	0,564243	0,30056	NaN	NaN	NaN
NaN	NaN	1,21215	-0,999025	NaN	-0,782557	-0,286373	NaN	NaN	NaN
-1,21881	-1,43825	-0,802029	-0,279081	-0,105925	-1,67933	-0,855398	NaN	NaN	NaN
-1,21881	-1,43825	-0,802029	-0,279081	-0,105925	-1,67933	-0,855398	NaN	NaN	NaN
NaN	NaN	NaN	NaN	NaN	-0,432922	-2,41595	NaN	NaN	NaN
NaN	NaN	NaN	-0,368807	NaN	-0,432922	-2,41595	NaN	NaN	NaN
NaN	1,26633	NaN	0,283121	0,68577	-0,849125	-0,540848	NaN	NaN	-0,441661
NaN	1,26633	NaN	0,283121	0,68577	-0,849125	-0,540848	NaN	NaN	-0,441661
NaN	1,26633	NaN	0,283121	0,68577	-0,849125	-0,540848	NaN	NaN	-0,441661
-1,36207	-1,70328	-2,18875	-0,962172	-1,97054	-1,83471	-1,51817	NaN	NaN	NaN
-1,36207	-1,70328	-2,18875	-0,962172	-1,97054	-1,83471	-1,51817	NaN	NaN	NaN
3,03212	0,28595	0,823372	1,63513	NaN	3,0298	1,26936	NaN	1,17369	NaN
3,03212	0,28595	0,823372	NaN	NaN	3,0298	1,26936	NaN	1,17369	NaN
NaN	NaN	NaN	-1,78075	-2,66299	-1,28697	-0,663795	NaN	NaN	NaN
1,13345	1,37062	0,500141	0,375427	0,65168	-0,862659	0,806633	1,18105	0,561564	0,19991
1,13345	1,37062	0,500141	0,375427	0,65168	-0,862659	0,806633	1,18105	0,561564	0,19991
-1,89632	-2,38936	-1,68478	NaN	NaN	NaN	NaN	-1,95166	-1,86869	NaN
0,936901	1,22401	0,967134	1,97683	0,497372	0,667669	1,32857	NaN	NaN	NaN
1,9279	3,57139	1,79513	2,45804	3,16718	2,73071	3,77273	1,64295	2,2192	2,70929
1,43892	1,76122	2,07787	1,915	1,5738	1,83023	1,64619	1,60566	1,46814	1,22095
1,43892	1,76122	2,07787	1,915	1,5738	1,83023	1,64619	1,60566	1,46814	1,22095
2,87466	1,86245	1,99633	1,3392	1,97777	0,518881	0,903116	1,55444	1,66206	1,89246

Polito et al. Universal ready-to-use immunotherapeutic approach for the treatment of cancer: expanded and activated polyclonal γδ memory T cells.
Phosphorylation sites

2,87466	1,90953	1,99633	2,98931	1,97777	2,41166	2,52022	1,81018	1,66206	2,0163
0,112314	NaN	-1,99922	-0,575891	-0,319471	-1,14614	-0,353085	-0,325136	-0,242455	-0,349248
2,09248	1,90953	1,72434	2,98931	1,57342	2,41166	2,52022	1,81018	1,51123	2,0163
NaN	NaN	NaN	-1,89097	-0,353832	-0,581587	-0,820606	NaN	NaN	NaN
NaN	-0,869423	NaN	-1,89097	-0,353832	-0,581587	-0,820606	NaN	NaN	NaN
NaN	-0,869423	NaN	NaN	NaN	NaN	NaN	NaN	NaN	NaN
-0,671179	1,85088	0,733805	0,465115	2,10171	-2,01603	1,79728	0,233166	1,05868	1,63779
-0,671179	1,85088	0,733805	0,465115	2,10171	-2,01603	1,79728	0,233166	1,05868	1,63779
-1,90624	-1,97157	-0,163582	NaN	NaN	NaN	NaN	NaN	NaN	NaN
-1,90624	-1,97157	-0,163582	NaN	NaN	NaN	NaN	NaN	NaN	NaN
NaN	-1,96189	NaN	NaN	NaN	NaN	NaN	NaN	NaN	NaN
NaN	-1,96189	NaN	NaN	NaN	NaN	NaN	NaN	NaN	NaN
4,23875	3,70509	3,91893	3,53564	3,72698	2,34017	4,27842	4,18914	3,9544	4,12778
4,23875	3,70509	3,91893	3,53564	3,72698	2,34017	4,27842	4,18914	3,9544	4,12778
NaN	1,96074	NaN	2,29073	NaN	NaN	2,21937	1,83793	2,18226	NaN
0,0647488	-1,4078	-1,0616	0,208469	-1,61223	0,777519	-0,602182	-1,84297	-1,40835	-2,55844
0,0647488	-1,4078	-1,0616	0,208469	-1,61223	0,777519	-0,602182	-1,84297	-1,40835	-2,55844
0,228926	1,85429	NaN	0,444731	-2,56264	0,785883	1,65025	-0,74696	0,52896	-0,293113
0,228926	-0,614265	NaN	0,444731	NaN	NaN	0,0827465	-0,74696	0,52896	-0,293113
-0,6369	2,6191	-0,811743	1,18118	0,825569	0,271915	1,9996	NaN	NaN	0,922803
NaN	NaN	NaN	2,07389	NaN	2,81149	NaN	NaN	3,4293	1,21345
NaN	NaN	NaN	NaN	NaN	NaN	NaN	NaN	NaN	NaN
NaN	NaN	NaN	-2,10504	-1,35409	-2,07701	-1,61363	NaN	NaN	NaN
-0,32729	-0,0454006	-0,628284	-0,803513	0,329702	-0,700462	0,113665	-0,00935936	0,368133	-0,161365
-0,32729	-0,0454006	-0,628284	-0,803513	0,329702	-0,700462	0,113665	-0,00935936	0,368133	-0,161365
NaN	NaN	2,46033	-1,27616	-1,8327	-1,29545	-0,564581	NaN	NaN	NaN
2,70632	3,02699	NaN	2,67807	2,95323	2,43157	2,85157	NaN	2,35191	2,38981
-0,281862	-0,0753555	-0,24474	-0,181023	0,0714169	-0,666702	-0,110884	NaN	NaN	NaN
2,70632	3,02699	2,46033	2,67807	2,95323	2,43157	2,85157	2,57576	2,35191	2,38981
3,66581	4,21712	3,32443	3,6368	3,65599	2,85921	3,88269	3,25335	3,73539	3,53255
3,66581	4,21712	3,32443	3,6368	3,65599	2,85921	3,88269	3,25335	3,73539	3,53255
-2,27755	-1,98563	-1,99102	-1,51868	-0,333805	-2,02147	-1,79142	-1,16388	-1,5093	-1,59104
-2,27755	-1,98563	-1,99102	NaN	NaN	NaN	NaN	NaN	NaN	NaN
NaN	NaN	NaN	-1,51868	-0,333805	-2,02147	-1,79142	-1,16388	-1,5093	-1,59104

Polito et al. Universal ready-to-use immunotherapeutic approach for the treatment of cancer: expanded and activated polyclonal γδ memory T cells.
Phosphorylation sites

NaN	-0,387016	NaN	-1,0717	-3,0541	-0,0233154	0,0883484	NaN	NaN	NaN
NaN	-0,387016	NaN	-1,0717	-3,0541	-0,0233154	0,0883484	NaN	NaN	NaN
NaN	NaN	NaN	-1,85821	NaN	-1,36653	-1,45763	NaN	NaN	NaN
NaN	NaN	NaN	-1,85821	NaN	-1,36653	-1,45763	NaN	NaN	NaN
NaN	-2,25428	-0,506544	NaN	-1,77007	-0,391132	0,865467	0,0522442	0,0574541	NaN
NaN	-2,25428	-0,506544	NaN	-1,77007	-1,29433	-0,285419	-0,426548	0,0574541	NaN
NaN	NaN	0,426571	-0,0402622	0,526028	-0,267801	1,02321	NaN	NaN	NaN
NaN	NaN	NaN	NaN	NaN	NaN	NaN	NaN	NaN	NaN
NaN	NaN	NaN	NaN	NaN	NaN	NaN	NaN	NaN	NaN
0,965645	2,51003	2,00913	1,63312	NaN	NaN	2,41512	NaN	2,56243	2,19833
0,965645	2,51003	2,00913	1,63312	NaN	NaN	2,41512	NaN	2,56243	2,19833
2,1715	4,99796	3,50953	3,80977	4,16811	2,7915	4,31458	2,5286	3,71894	3,52066
2,1715	4,99796	3,50953	3,80977	4,16811	2,7915	4,31458	2,5286	3,71894	3,52066
-0,76338	-0,393898	-0,597715	NaN	NaN	NaN	NaN	NaN	NaN	NaN
-0,76338	-0,393898	-0,597715	NaN	NaN	NaN	NaN	NaN	NaN	NaN
-0,929522	0,497034	NaN	-0,746487	-0,334236	-0,554688	1,01328	NaN	NaN	NaN
NaN	3,34724	1,36891	1,37224	2,72039	-0,025631	2,76887	NaN	1,31665	2,50841
0,709019	1,28742	1,10438	0,567648	1,15574	0,470318	1,60439	1,13068	0,946837	1,85114
0,709019	1,28742	1,10438	0,567648	1,15574	0,470318	1,60439	1,13068	0,946837	1,85114
NaN	2,65496	1,11009	NaN	3,10509	NaN	NaN	0,518009	0,76835	1,81729
NaN	1,18233	NaN	NaN	NaN	NaN	NaN	NaN	NaN	NaN
0,570982	3,46537	1,92069	1,69318	3,86009	-0,0149364	2,97027	1,6928	1,5082	2,69504
1,25111	3,12068	1,2825	2,21895	3,59334	-0,686335	2,64551	1,05203	1,41668	2,80326
1,25111	3,12068	1,2825	2,21895	3,59334	-0,686335	2,64551	1,05203	1,41668	2,80326
-0,375498	1,39671	0,613998	0,698008	2,08573	-2,11778	1,42194	-0,187634	-0,0651064	0,948001
-0,375498	1,39671	0,613998	0,698008	2,08573	-2,11778	1,42194	-0,187634	-0,0651064	0,948001
NaN	0,37405	NaN	NaN	0,197048	NaN	-2,26224	-2,35271	-2,55298	-1,20453
NaN	0,37405	NaN	NaN	0,197048	NaN	-2,26224	-2,35271	-2,55298	-1,20453
-1,97206	NaN	NaN	0,0680923	NaN	0,0997677	-0,242567	-1,13117	-1,23425	NaN
-1,97206	NaN	-2,05555	0,0680923	NaN	0,0997677	-0,242567	-1,13117	-1,23425	NaN
1,02849	1,27826	-1,07409	0,296595	0,554804	-0,53195	0,657921	0,368437	0,205396	-0,173503
4,75235	5,99388	3,63435	5,07671	4,51321	5,22164	5,37872	4,09567	4,55911	4,06705
4,75235	5,99388	3,63435	5,07671	4,51321	5,22164	5,37872	4,09567	4,55911	4,06705
NaN	NaN	NaN	NaN	NaN	NaN	NaN	-2,249	-2,65056	-2,18969

Polito et al. Universal ready-to-use immunotherapeutic approach for the treatment of cancer: expanded and activated polyclonal γδ memory T cells.
Phosphorylation sites

NaN	NaN	NaN	NaN	NaN	NaN	NaN	-2,249	-2,65056	-2,18969
NaN	NaN	NaN	-1,99961	0,0944214	-3,00122	-1,31703	-0,237486	-0,856942	-1,39985
NaN	NaN	NaN	-1,99961	0,0944214	-3,00122	-1,31703	-0,237486	-0,856942	-1,39985
NaN	NaN	-1,15589	-0,932117	-2,79543	NaN	-0,420694	NaN	NaN	NaN
2,63114	NaN	0,812672	0,416637	2,42297	NaN	1,89593	NaN	1,50047	2,46489
2,63114	0,252338	0,812672	0,416637	2,42297	NaN	1,89593	NaN	1,50047	2,46489
NaN	NaN	NaN	-0,563993	NaN	0,546549	-1,39858	NaN	NaN	NaN
NaN	NaN	NaN	-0,563993	NaN	0,546549	-1,39858	NaN	NaN	NaN
NaN	NaN	NaN	-1,05973	-1,80442	-0,476059	-1,08899	NaN	NaN	NaN
NaN	NaN	NaN	-2,17927	-1,02475	NaN	NaN	NaN	NaN	NaN
NaN	NaN	NaN	-2,17927	-1,02475	NaN	NaN	NaN	NaN	NaN
-2,30476	-2,85388	-1,42466	NaN	NaN	NaN	NaN	-0,512177	-1,05529	-0,838414
-2,30476	-2,85388	-1,42466	NaN	NaN	NaN	NaN	NaN	-1,05529	-0,838414
1,86911	2,84753	2,10256	3,82956	2,91721	2,15841	4,0305	1,90893	1,48992	2,53546
1,86911	2,84753	2,10256	3,82956	2,91721	2,15841	4,0305	1,90893	1,48992	2,53546
1,80086	3,29954	1,26488	1,95077	-0,887819	1,91275	2,35576	0,247503	2,64963	0,932214
1,80086	3,29954	1,26488	1,95077	-0,887819	1,91275	2,35576	0,247503	2,64963	0,932214
-0,0425167	1,96732	-0,330894	0,157661	-0,486525	NaN	0,898689	0,0515957	1,04612	0,503594
-0,0425167	1,96732	-0,330894	0,157661	-0,486525	NaN	0,898689	0,0515957	1,04612	0,503594
NaN	0,277233	NaN	NaN	NaN	NaN	NaN	-0,297529	NaN	0,24055
NaN	0,277233	NaN	NaN	NaN	NaN	NaN	-0,297529	0,545074	0,24055
3,12137	1,79689	2,81318	3,52715	2,17324	1,46103	3,24755	2,38591	2,68032	2,08947
3,12137	1,79689	2,81318	3,52715	2,17324	1,46103	3,24755	2,38591	2,68032	2,08947
0,347029	0,222208	0,214289	1,35488	0,663271	-1,90554	1,58262	0,158436	-0,0029974	-0,0787725
0,347029	0,222208	0,214289	1,35488	0,663271	-1,90554	1,58262	0,158436	-0,0029974	-0,0787725
-0,482855	NaN	-0,858902	1,25959	-0,661343	0,310402	0,572304	-0,64188	-0,880498	-1,18572
-0,482855	NaN	-0,858902	1,25959	-0,661343	0,310402	0,572304	-0,64188	-0,880498	-1,18572
-0,173401	-0,944817	-0,579576	NaN	-0,229052	0,986637	NaN	NaN	-0,146625	0,359639
NaN	NaN	NaN	1,3624	NaN	NaN	1,44165	0,5137	NaN	NaN
4,84045	NaN	4,38754	3,90696	5,18909	3,57667	2,81644	3,49278	2,79655	2,8321
1,60331	NaN	-0,431101	1,37854	1,11089	NaN	1,77303	1,68506	1,26066	NaN
NaN	NaN	NaN	0,451872	NaN	0,508472	-0,452511	NaN	NaN	NaN
NaN	NaN	NaN	0,451872	NaN	0,508472	-0,452511	NaN	NaN	NaN
NaN	NaN	NaN	-1,62698	-1,5828	-1,7923	-1,91281	-1,67586	-2,123	-2,70422

Polito et al. Universal ready-to-use immunotherapeutic approach for the treatment of cancer: expanded and activated polyclonal γδ memory T cells.
Phosphorylation sites

NaN	NaN	NaN	-1,62698	-1,5828	-1,7923	NaN	NaN	NaN	NaN
0,792315	1,09555	0,613306	1,81845	0,582733	3,43261	0,918924	-0,314304	-0,332812	0,68744
-0,0977898	-0,163912	-1,50119	NaN	-0,587667	NaN	NaN	NaN	NaN	NaN
-0,736952	-0,371635	NaN	1,81845	NaN	3,43261	NaN	NaN	NaN	NaN
NaN	NaN	NaN	NaN	NaN	NaN	NaN	-1,93592	-2,10348	NaN
0,808081	0,972731	0,613167	0,349693	0,723091	NaN	-0,0732269	-0,585735	-0,3789	-0,181106
0,808081	0,972731	0,613167	0,349693	0,723091	NaN	-0,0732269	-0,585735	-0,3789	-0,181106
-0,62756	0,256779	0,0576744	-0,483221	0,357531	-3,17713	-0,554436	NaN	0,00495434	0,0220804
NaN	0,256779	0,0576744	-0,483221	0,357531	-3,17713	-0,554436	0,382778	NaN	0,0220804
NaN	NaN	NaN	-2,97692	-4,12272	NaN	-3,09322	NaN	NaN	NaN
NaN	NaN	NaN	-2,97692	-4,12272	NaN	-3,09322	NaN	NaN	NaN
0,50115	-0,681482	-1,72794	-0,143787	0,660486	0,782961	0,60928	-3,35314	-2,59908	-1,26287
NaN	NaN	NaN	NaN	NaN	NaN	NaN	-1,47225	-1,62586	-0,716054
NaN	NaN	NaN	NaN	NaN	NaN	NaN	-1,47225	-1,62586	-0,716054
-3,72198	NaN	-0,855141	-1,73243	NaN	-1,67389	0,699522	NaN	NaN	NaN
-3,72198	NaN	-0,855141	-1,73243	NaN	-1,67389	0,699522	NaN	NaN	NaN
NaN	NaN	NaN	-0,465321	NaN	-1,90545	-0,50873	-2,32833	-2,63252	-1,98524
NaN	NaN	NaN	-0,465321	NaN	-1,90545	-0,50873	-2,32833	-2,63252	-1,98524
1,85832	2,10177	1,38109	1,73316	1,19016	2,12072	1,84707	0,688995	1,77598	1,29643
1,85832	2,10177	1,38109	1,73316	1,19016	2,12072	1,84707	0,688995	1,77598	1,29643
NaN	NaN	NaN	-3,06343	-1,68095	NaN	-2,3007	NaN	NaN	NaN
NaN	NaN	NaN	-3,06343	-1,68095	NaN	-2,3007	NaN	NaN	NaN
NaN	NaN	NaN	-0,748678	NaN	0,0708523	-1,41601	NaN	NaN	NaN
NaN	NaN	NaN	-0,415754	-0,930099	NaN	-1,704	NaN	NaN	NaN
-1,53445	-2,1586	NaN	0,0811672	-0,930099	0,392027	-1,704	NaN	-0,730077	NaN
-0,696384	-2,35309	-1,65764	-3,01173	-1,60227	-2,46996	-1,9182	-1,40044	-1,40639	-0,451606
-0,696384	-2,35309	-1,65764	-3,01173	-1,60227	-2,46996	-1,9182	-1,40044	-1,40639	-0,451606
NaN	NaN	NaN	-0,0191326	-0,339262	-1,96948	0,37496	NaN	NaN	NaN
NaN	NaN	NaN	-0,0191326	-0,339262	-1,96948	0,37496	NaN	NaN	NaN
0,696815	NaN	-0,157234	0,764334	1,84778	1,01247	-0,753025	-0,916111	0,565866	0,554265
-1,61079	-1,96724	-2,44795	-0,457802	-1,201	-1,01852	-1,14285	NaN	NaN	NaN
-1,61079	-1,96724	-2,44795	-0,457802	-1,201	-1,01852	-1,14285	NaN	NaN	NaN
0,662537	0,188429	-0,728575	NaN	-0,544605	-3,31607	NaN	-0,665501	-0,0423307	-1,23992
0,662537	0,188429	-0,728575	NaN	-0,544605	-3,31607	NaN	-0,665501	-0,0423307	-1,23992

0,756563	0,0787258	-0,186966	NaN	-0,166637	NaN	NaN	NaN	NaN	NaN
1,35581	0,777349	0,375772	NaN	0,830278	NaN	NaN	NaN	0,120011	NaN
1,35581	0,777349	0,375772	NaN	0,830278	NaN	NaN	0,199308	0,120011	0,788949
NaN	NaN	-1,45293	NaN	NaN	NaN	NaN	-2,76846	-2,53587	-0,354196
-2,32732	0,189795	-1,45293	-0,323269	0,583075	0,555546	0,360743	-2,76846	-2,53587	-0,354196
-2,32732	0,189795	-1,45293	-0,323269	0,583075	0,555546	0,360743	-2,76846	-2,53587	-0,354196
NaN	NaN	NaN	-1,80329	-0,976116	NaN	-1,9134	NaN	NaN	NaN
NaN	NaN	NaN	-1,80329	-0,976116	NaN	-1,9134	NaN	NaN	NaN
1,27667	0,844044	-0,361668	-0,564919	0,872744	0,453089	0,55636	-0,0338936	-0,567645	-0,146911
1,27667	0,844044	-0,361668	-0,564919	0,872744	0,453089	0,55636	-0,0338936	-0,567645	-0,146911
-1,50457	-1,98761	-1,42636	NaN	-1,62358	NaN	NaN	NaN	NaN	NaN
-1,50457	-1,98761	-1,42636	NaN	-1,62358	NaN	NaN	NaN	NaN	NaN
-0,879511	-1,71055	-1,75673	-2,79274	-0,399607	-2,24885	-1,45303	-1,5992	-1,69151	-1,80195
-0,879511	-1,71055	-1,75673	-2,79274	-0,399607	-2,24885	-1,45303	-1,5992	-1,69151	-1,80195
2,57875	2,01366	1,38182	2,59751	0,89765	3,04251	2,40478	NaN	1,7275	NaN
2,57875	2,01366	1,38182	2,59751	0,89765	3,04251	2,40478	NaN	1,7275	NaN
-0,540966	0,32999	NaN	0,181135	NaN	0,911797	0,477999	NaN	0,301717	-0,646113
1,16952	NaN	NaN	0,877119	1,97704	0,911797	NaN	NaN	NaN	NaN
0,159401	0,527508	-0,329243	-0,139874	0,957045	NaN	NaN	-0,560577	-0,264384	-0,0466318
0,337221	0,00312805	-0,151196	NaN	NaN	NaN	NaN	NaN	-0,0957899	NaN
NaN	NaN	NaN	-2,9104	NaN	NaN	-3,13881	NaN	NaN	NaN
1,55877	1,81596	1,31054	NaN	2,01524	0,501295	1,90108	NaN	1,00133	NaN
NaN	NaN	NaN	-0,444208	1,83596	4,26073	4,77701	NaN	NaN	NaN
2,61476	3,10564	2,05785	3,58604	1,83596	4,26073	4,77701	NaN	3,1466	2,57108
NaN	NaN	NaN	0,767685	NaN	0,714941	0,341188	-0,444426	-0,454337	NaN
-2,5164	NaN	-1,85967	-0,25585	-1,4388	-2,0804	-1,98944	NaN	NaN	NaN
-1,99368	-1,10467	-1,13065	-1,75254	-1,76195	-1,38438	-0,35454	NaN	NaN	NaN
NaN	NaN	NaN	-0,355904	-0,430426	0,480488	NaN	NaN	NaN	NaN
NaN	NaN	NaN	NaN	NaN	NaN	NaN	NaN	NaN	NaN
-1,5797	NaN	0,107002	0,96666	-1,44723	1,54655	-0,292273	-0,854292	NaN	NaN
1,97663	0,860439	-1,22318	1,61703	1,66924	1,9632	1,19038	1,68924	1,54718	1,47825
NaN	NaN	-0,975014	NaN	NaN	NaN	-0,120764	-0,992958	-1,1181	-0,172469
NaN	NaN	NaN	-0,927147	-1,43542	-1,19206	-0,461838	-1,29361	-1,97505	-1,56291
NaN	NaN	NaN	-0,773571	-1,43043	-1,19206	-0,461838	-1,29361	-1,97505	-1,56291

Polito et al. Universal ready-to-use immunotherapeutic approach for the treatment of cancer: expanded and activated polyclonal γδ memory T cells.
Phosphorylation sites

NaN	NaN	NaN	-1,25816	-2,57245	-0,981497	-0,179457	NaN	NaN	NaN
2,80502	2,84604	3,21256	3,95018	4,26341	1,6922	3,03945	2,55397	3,04328	3,41941
2,93299	1,50858	3,22206	3,97634	2,91894	3,97412	3,50766	2,87247	3,06863	2,37458
1,37134	-0,0992203	NaN	NaN	NaN	-1,43428	-1,86343	-0,785736	-0,860923	-0,376874
-0,265886	-0,874384	-0,875917	-1,07088	1,48436	NaN	-1,31114	-0,48295	0,707858	-0,334529
NaN	NaN	NaN	NaN	NaN	-1,29601	NaN	-2,07813	-1,28588	-0,786151
-1,4118	-1,66568	-0,208235	0,482454	0,487732	NaN	1,02068	0,00520325	-0,588679	0,0775232
NaN	NaN	NaN	0,627415	-1,39261	-0,651678	-1,74919	NaN	NaN	NaN
NaN	NaN	NaN	-0,192322	NaN	NaN	-0,505648	-0,794968	-0,943393	-0,810174
2,02996	3,73207	2,00723	1,96548	1,39799	2,52924	2,69849	1,34005	2,47081	1,63852
2,87308	2,91198	3,24042	3,74571	2,42036	4,20653	4,30183	2,81053	2,64983	2,29671
NaN	NaN	NaN	3,25994	3,6557	3,55165	3,99296	NaN	NaN	NaN
3,32581	3,28355	3,19751	3,93307	3,02624	3,34039	3,79053	3,26578	3,33117	4,81607
1,95082	1,71622	1,00723	1,13982	1,08365	-1,35824	1,6391	0,542601	1,32428	1,18688
-1,53187	-2,23494	-0,799295	0,435925	-2,12047	NaN	-0,936092	NaN	-0,749082	-1,1229
-1,53187	-2,23494	-0,799295	0,435925	-2,12047	NaN	-0,936092	NaN	-0,749082	-1,1229
NaN	NaN	NaN	NaN	NaN	NaN	NaN	NaN	NaN	NaN
NaN	NaN	NaN	NaN	NaN	NaN	NaN	NaN	NaN	NaN
-1,68615	NaN	-0,267803	NaN	NaN	NaN	NaN	-0,753632	-0,736215	-1,43386
NaN	1,37074	0,905781	0,614025	1,2376	1,49684	1,34292	0,577602	0,269412	NaN
1,56021	0,939569	0,559668	NaN	1,033	NaN	-0,372887	1,06508	1,05473	0,929204
-0,988352	-1,19934	-1,13228	1,25775	NaN	1,266	-0,882004	NaN	NaN	NaN
2,3073	-0,021534	0,832329	0,987391	0,855656	2,18319	1,25987	1,04769	0,234782	-0,822703
NaN	NaN	NaN	1,01939	-2,27758	1,3539	-0,388042	NaN	NaN	NaN
NaN	NaN	NaN	1,14124	-1,18069	1,87861	1,33647	NaN	NaN	NaN
1,05816	1,61748	0,799458	1,46558	1,10958	-2,20526	1,52896	0,795019	0,405665	-4,75356
NaN	NaN	NaN	4,43888	-2,02701	3,33017	NaN	NaN	NaN	NaN
NaN	NaN	NaN	NaN	NaN	NaN	NaN	NaN	NaN	NaN
1,60059	3,10037	0,630249	1,1005	1,37691	0,218124	2,10382	0,983671	2,66342	1,55633
NaN	NaN	NaN	3,16196	-3,02835	3,01658	1,52477	NaN	NaN	NaN
3,13085	2,7052	2,84545	3,69324	3,7381	0,388735	3,10413	2,82602	3,42699	NaN
-0,978731	-1,53565	-1,53925	-0,650452	-0,576092	-1,16332	NaN	NaN	NaN	NaN
3,14679	2,23517	2,46514	2,9996	NaN	2,71755	2,41647	1,91885	2,42972	2,48585
NaN	2,03465	1,27402	1,75777	2,12908	NaN	2,42614	NaN	NaN	1,44332

Polito et al. Universal ready-to-use immunotherapeutic approach for the treatment of cancer: expanded and activated polyclonal γδ memory T cells.
Phosphorylation sites

1,97385	1,30958	1,88562	0,471228	2,42445	0,681559	0,663857	0,707581	0,71087	1,51364
NaN	NaN	NaN	-0,490564	NaN	-0,498367	-1,83174	NaN	NaN	NaN
-1,27275	-1,42694	-1,83594	-2,38453	-1,62629	NaN	-1,66323	-1,32858	-1,90825	-1,57086
-0,0630054	0,381483	-2,21449	-1,6357	0,0747948	-1,18902	-0,369967	-0,474539	-1,07167	-1,29714
NaN	NaN	-0,492071	-0,429453	NaN	-1,36597	NaN	NaN	NaN	NaN
NaN	-1,77078	-0,453449	2,09168	-0,669958	NaN	0,52062	0,61536	0,412152	-0,22646
3,60802	2,73579	2,10527	2,53475	3,69964	1,75685	2,6918	3,33585	2,41518	2,96088
0,0236874	0,0240231	-0,187883	0,593706	-0,507107	0,0154953	0,329712	-0,803694	0,0690355	-0,632602
NaN	NaN	NaN	0,822453	-0,675669	1,23604	0,54501	-1,19838	-0,368403	-1,03591
1,28979	1,40049	1,27849	0,050663	1,7982	-0,854034	0,879381	NaN	NaN	NaN
0,408319	-0,506922	-0,456036	0,463249	0,0887642	-3,0067	0,276814	NaN	NaN	NaN
0,408319	-0,506922	-0,456036	0,463249	0,0887642	-3,0067	0,276814	NaN	NaN	NaN
NaN	NaN	NaN	-2,60848	-1,84215	NaN	-1,83868	NaN	NaN	NaN
2,60617	1,71076	1,69381	2,08164	2,36812	0,175362	2,85172	2,3227	1,42686	1,89752
2,60617	1,71076	1,69381	2,08164	2,36812	0,175362	2,85172	2,3227	1,42686	1,89752
2,10571	0,437313	1,51994	1,80882	2,52854	1,84512	2,04156	0,669714	0,561564	0,53991
-0,136276	-1,11226	-1,81052	-0,679567	0,0523224	NaN	0,198275	-0,703989	-0,573371	-1,04365
-0,136276	-1,11226	-1,81052	-0,679567	0,0523224	NaN	0,198275	-0,703989	-0,573371	-1,04365
NaN	-0,296015	NaN	-1,30154	-1,5794	-2,01515	-0,58341	NaN	NaN	NaN
5,68998	5,30974	5,10195	4,96667	4,85475	5,12216	4,93351	5,00233	4,87967	5,2989
NaN	NaN	NaN	1,04162	NaN	0,386171	0,874149	NaN	NaN	NaN
NaN	NaN	-0,912674	-1,59681	-4,9267	-1,57055	-1,12758	NaN	NaN	NaN
1,15255	1,31335	NaN	0,572105	0,242748	NaN	0,805738	0,0398273	NaN	NaN
NaN	NaN	NaN	-1,31529	-2,99263	-1,1644	-0,359377	-2,2998	-2,63188	-3,01361
NaN	NaN	NaN	-2,9104	NaN	NaN	-3,13881	NaN	NaN	NaN
NaN	NaN	NaN	0,684116	NaN	2,34916	-2,54969	NaN	NaN	NaN
NaN	NaN	NaN	-1,1911	NaN	-1,94362	-2,27084	NaN	NaN	NaN
NaN	NaN	NaN	3,16196	-3,02835	3,01658	1,52477	NaN	NaN	NaN
NaN	NaN	NaN	NaN	NaN	NaN	NaN	NaN	NaN	NaN
NaN	NaN	NaN	1,80704	NaN	0,164602	0,537622	-0,975639	0,672782	-0,375295
NaN	NaN	NaN	NaN	NaN	NaN	NaN	0,597092	-0,213323	0,471282
NaN	NaN	NaN	1,26595	-0,0414772	0,880047	-0,0583496	NaN	NaN	NaN
NaN	NaN	NaN	1,26595	-0,0414772	0,880047	-0,0583496	NaN	NaN	NaN
NaN	NaN	NaN	NaN	NaN	NaN	NaN	NaN	NaN	NaN

Polito et al. Universal ready-to-use immunotherapeutic approach for the treatment of cancer: expanded and activated polyclonal γδ memory T cells. Phosphorylation sites

0,926252	1,52778	0,779716	1,53122	0,17938	NaN	1,26751	0,878792	0,9894	1,21553
NaN	NaN	NaN	3,76463	-2,33811	2,79762	1,79077	NaN	NaN	NaN
NaN	NaN	NaN	0,889576	NaN	-0,882252	-0,667091	NaN	NaN	NaN
NaN	NaN	NaN	1,91188	NaN	0,646391	-0,1639	-1,4863	-1,09513	-1,33989
-0,191103	NaN	-1,41242	-2,55414	-2,3516	-1,20546	-2,19143	-1,10959	-1,03628	-2,78557
-1,5785	-1,61336	-1,812	-0,677626	-2,15425	-1,18739	-1,53239	-1,52526	-1,71166	-0,163085
NaN	NaN	NaN	-0,121399	NaN	-0,338469	NaN	NaN	-1,99257	NaN
NaN	NaN	NaN	-0,121399	NaN	-0,338469	NaN	NaN	NaN	NaN
NaN	NaN	NaN	NaN	NaN	NaN	NaN	NaN	NaN	NaN
NaN	NaN	NaN	1,09578	NaN	-0,00456047	-1,68326	NaN	NaN	NaN
NaN	NaN	NaN	0,916607	NaN	0,715452	NaN	NaN	NaN	NaN
NaN	NaN	NaN	-2,50218	-2,20434	-0,680542	NaN	NaN	NaN	NaN
NaN	NaN	NaN	NaN	NaN	NaN	NaN	NaN	NaN	NaN
NaN	NaN	NaN	NaN	NaN	NaN	NaN	NaN	NaN	NaN
6,84054	6,71014	6,53112	3,84857	5,02387	2,5515	5,44931	7,37517	6,74087	6,38851
1,21945	NaN	-1,04545	0,739777	0,270096	-0,301254	-0,227339	NaN	NaN	NaN
NaN	NaN	NaN	-0,772974	-0,905766	NaN	-0,752913	-1,1751	-0,4325	-3,8382
-1,21777	-1,99231	-2,02797	-0,976498	-1,15026	NaN	-1,6979	-1,33574	-1,86862	-2,09802
-0,0499134	NaN	0,809652	0,915926	0,979353	0,244158	0,494431	0,619539	1,44475	1,26019
2,74459	1,83796	2,33356	3,05608	2,27937	3,31376	1,8376	1,47196	1,60516	1,87518
-0,713009	0,122414	-0,665119	-0,969425	-0,0500469	-3,51527	0,414495	-0,194324	-0,383359	-0,470084
-0,350666	0,376572	0,375069	0,135811	0,61747	0,1423	0,906698	0,65379	1,16886	1,57786
-0,713009	0,122414	-0,665119	-0,969425	-0,0500469	-3,51527	0,414495	-0,194324	-0,383359	-0,470084
0,381378	-0,384573	-0,0184307	0,25456	0,24534	-1,21923	-0,208729	0,182878	-0,0313272	0,710202
NaN	NaN	0,0905743	-0,270187	1,56892	0,383692	0,322474	0,594055	1,15585	1,07609
1,21561	1,39837	0,706398	1,1643	2,16737	NaN	1,63616	1,30978	0,176942	1,28978
NaN	NaN	NaN	-0,721661	NaN	-0,469688	-0,66185	NaN	NaN	NaN
NaN	NaN	NaN	-0,806435	NaN	-0,937571	-1,05493	NaN	NaN	NaN
NaN	NaN	NaN	-0,669767	NaN	-0,22311	-0,833809	NaN	NaN	NaN
NaN	NaN	NaN	-1,36532	NaN	-0,160429	-0,419678	NaN	NaN	NaN
1,99399	1,42379	1,22554	2,0055	-0,187889	3,11606	1,47234	1,20072	1,56697	0,42323
NaN	NaN	NaN	-2,12182	NaN	-2,08055	-1,74559	NaN	NaN	NaN
NaN	NaN	NaN	0,0551109	NaN	-1,35548	0,332483	NaN	NaN	NaN
NaN	1,57618	NaN	NaN	NaN	NaN	NaN	NaN	NaN	NaN

Polito et al. Universal ready-to-use immunotherapeutic approach for the treatment of cancer: expanded and activated polyclonal γδ memory T cells.
Phosphorylation sites

1,49027	-0,769138	0,593775	NaN	0,502296	NaN	NaN	1,36331	0,79069	0,493964
NaN	NaN	NaN	NaN	NaN	NaN	NaN	-1,04935	-0,916703	-0,644212
1,20461	0,753984	NaN	0,109251	0,447765	-2,05041	1,11336	0,319046	0,307197	0,234458
NaN	NaN	NaN	1,56112	NaN	NaN	NaN	NaN	NaN	NaN
1,72531	1,05353	1,20949	0,469236	1,58938	0,85277	0,211016	0,619165	0,446496	0,273807
0,356699	0,315668	NaN	NaN	-0,0608311	-0,588923	-1,01998	0,449961	0,568219	0,238685
NaN	NaN	NaN	-0,780279	NaN	0,15914	-1,35608	NaN	NaN	NaN
-1,03722	-1,08544	-2,09558	-1,58138	-1,04667	-1,10909	-1,35209	-1,85592	-2,09759	-1,88292
NaN	NaN	NaN	-0,335117	NaN	0,342134	-1,06785	NaN	NaN	NaN
NaN	NaN	NaN	0,0904331	-1,41056	-0,00528145	-1,09676	NaN	NaN	NaN
NaN	NaN	NaN	-1,44216	NaN	NaN	-2,25145	NaN	NaN	NaN
NaN	-3,41019	0,986948	NaN	NaN	NaN	NaN	0,096262	-0,00108814	-0,675941
NaN	-3,41019	0,986948	NaN	NaN	NaN	NaN	0,096262	-0,00108814	-0,675941
NaN	NaN	NaN	-0,569029	-0,937487	-0,806355	-0,576895	-1,49316	-1,40075	-0,78457
NaN	NaN	NaN	-2,37726	NaN	-3,14529	-1,22687	NaN	NaN	NaN
NaN	NaN	NaN	2,70118	-0,16011	1,94924	2,75006	NaN	NaN	NaN
NaN	NaN	NaN	2,70118	-0,16011	1,94924	2,75006	NaN	NaN	NaN
NaN	NaN	NaN	NaN	NaN	NaN	NaN	NaN	NaN	NaN
1,85789	NaN	3,08947	3,89428	1,59955	3,05565	3,26959	NaN	NaN	NaN
1,85789	NaN	3,08947	3,89428	NaN	3,05565	NaN	NaN	NaN	NaN
-2,2186	-1,17058	-0,344099	-2,04479	-1,42714	-2,01438	0,710352	-0,512106	-0,622506	-0,451669
3,73346	3,43789	2,9654	NaN	3,39607	0,343458	NaN	3,54214	2,91888	2,36084
NaN	NaN	NaN	NaN	NaN	NaN	NaN	NaN	NaN	NaN
-2,28406	-0,320862	NaN	NaN	NaN	NaN	NaN	1,53382	2,14142	0,856795
-0,411531	-0,289221	0,0946884	0,246527	NaN	1,14235	0,0355606	NaN	NaN	NaN
0,348045	0,320789	-0,544374	-0,0947723	-0,0483112	0,445629	NaN	-0,878069	-0,725289	0,151435
NaN	1,84773	-0,662231	0,337626	NaN	0,681484	0,494003	NaN	0,953525	NaN
1,68998	0,586794	1,43021	0,486265	1,25319	1,06284	0,744633	0,478455	1,22038	NaN
2,40029	0,918625	1,62908	2,49487	NaN	NaN	NaN	1,98499	NaN	1,36155
-0,728642	-0,0645142	2,46958	NaN	3,08059	1,77633	1,75672	NaN	0,00374889	-0,399459
NaN	NaN	NaN	-2,20617	NaN	-1,43801	-0,971981	NaN	NaN	NaN
0,675032	0,521551	0,295839	0,538076	1,82789	0,951624	0,93046	-0,372011	-0,799201	-0,774602
-1,42552	-2,64331	-1,92901	-2,39629	-1,03911	NaN	-1,85031	-1,92442	-2,06016	-2,38072
NaN	1,36947	1,12503	-0,565979	1,74304	-0,358717	0,871098	1,10656	0,765698	0,791604

Polito et al. Universal ready-to-use immunotherapeutic approach for the treatment of cancer: expanded and activated polyclonal γδ memory T cells.
Phosphorylation sites

NaN	NaN	NaN	0,0509834	NaN	0,952616	-1,32984	NaN	NaN	NaN
2,85471	2,23871	1,6019	1,69863	2,38259	1,64681	1,75168	2,43212	1,95141	1,47619
0,821783	3,39057	-0,548925	2,19566	2,6018	-0,0690956	2,53938	NaN	NaN	1,67592
0,570366	0,716839	1,29171	0,926023	0,901186	-1,40654	1,8246	0,66387	0,698916	1,18981
NaN	NaN	NaN	-0,273272	-1,91845	NaN	-0,808069	NaN	NaN	NaN
1,41861	0,718922	0,0777168	-0,152426	0,518505	0,303234	-0,253075	0,627005	0,052598	-0,699636
NaN	NaN	NaN	-1,34856	NaN	0,511839	-1,68887	NaN	NaN	NaN
2,86115	2,56514	2,06384	2,62929	2,23222	2,94739	2,39924	1,60484	1,1621	1,03491
NaN	NaN	NaN	NaN	NaN	NaN	NaN	NaN	NaN	NaN
NaN	NaN	NaN	NaN	NaN	NaN	NaN	-1,56301	-1,68167	-2,28613
2,83758	4,56916	1,54507	2,45244	-0,0158672	-0,615976	2,73661	0,369286	2,58479	1,69844
1,32184	1,10637	0,412657	1,18426	NaN	NaN	0,879503	1,12131	1,05216	0,848023
NaN	NaN	NaN	-0,46081	NaN	-1,01661	NaN	NaN	NaN	NaN
1,34399	2,87132	0,43412	1,07376	0,0254478	1,24183	1,87969	0,0399971	1,11155	1,11666
NaN	NaN	NaN	-1,32806	NaN	0,118214	-0,585688	NaN	NaN	NaN
NaN	NaN	NaN	NaN	0,668253	NaN	0,365139	NaN	NaN	NaN
1,35433	0,492723	0,859919	0,841043	0,810944	1,16091	NaN	1,15194	0,748954	0,421586
-1,79123	-3,27518	NaN	-2,14277	-2,68778	-2,2517	-0,737106	-2,2705	-2,46724	-2,40629
-0,348534	-0,724232	-0,117439	-1,50411	-0,661839	NaN	-2,43286	-0,697989	-0,685891	-1,49678
0,282761	0,182539	0,186916	0,68549	0,336271	1,09006	0,753012	-0,0068512	0,15705	0,732472
NaN	NaN	NaN	NaN	NaN	NaN	NaN	NaN	NaN	NaN
-0,569864	-1,43821	-1,38869	-1,37903	-0,00276184	-1,70951	-0,784128	-1,49584	-3,14597	-0,720622
-0,33057	-1,33702	-0,879969	-0,203941	-0,404001	-2,34963	-0,212969	-0,486336	-1,57517	-0,711387
-0,718508	-1,77414	NaN	-0,894897	-1,07693	NaN	-1,65568	NaN	NaN	NaN
NaN	NaN	NaN	-0,539419	-1,19435	0,231762	-0,454868	NaN	NaN	NaN
1,02663	0,753061	-0,241302	0,604748	1,03355	0,624949	0,0911846	-0,49777	-0,299846	-0,418502
0,0368652	0,648022	-0,697374	-0,13377	0,00292587	0,576429	-0,221073	-0,769445	-1,21527	-1,15335
0,059082	-0,280321	-0,184195	1,20634	0,438816	1,43299	1,92099	0,228384	-0,179994	-0,733806
0,818876	1,20542	NaN	0,637848	1,07558	0,210894	0,51981	NaN	NaN	NaN
NaN	NaN	NaN	-0,632643	0,202099	-0,415749	-0,994556	-0,551226	NaN	-0,507831
-0,440044	-2,88439	-1,98774	-1,44711	NaN	-0,545284	-0,410233	-0,619366	-0,842565	NaN
-0,659462	-0,807503	-2,0879	NaN	NaN	NaN	NaN	-2,46253	-0,412026	-0,637485
NaN	NaN	NaN	-1,846	-0,159666	NaN	-1,4615	-2,01587	-1,20054	-0,250228
NaN	NaN	NaN	NaN	NaN	NaN	NaN	NaN	NaN	NaN

Polito et al. Universal ready-to-use immunotherapeutic approach for the treatment of cancer: expanded and activated polyclonal γδ memory T cells.
Phosphorylation sites

-1,13554	-2,23435	-1,76824	NaN	NaN	NaN	NaN	NaN	NaN	NaN
2,06351	2,48066	1,57838	0,378967	1,55911	0,375217	0,863625	1,44665	1,57904	1,53566
0,952902	-0,484322	-0,136116	1,51684	1,06243	1,85588	0,646259	0,585901	1,06172	0,948359
-0,531385	-0,680656	-0,543293	NaN	NaN	NaN	NaN	-0,63114	-0,772855	-0,962388
-0,889318	-0,341223	-1,02845	-1,81294	-0,866407	NaN	-2,16413	-1,1715	-1,5543	NaN
NaN	-0,642586	NaN	-0,588329	-0,22403	NaN	-1,48046	-1,86443	-1,48017	-1,27553
NaN	-0,642586	NaN	-0,588329	-0,22403	NaN	-1,48046	-1,86443	-1,48017	-1,27553
NaN	-0,368877	-0,312826	NaN	NaN	NaN	NaN	0,444216	0,507394	0,678004
0,222935	0,452534	-0,380198	-1,36291	NaN	-0,683542	-1,24736	-0,0924702	-0,270507	-0,167743
-0,98102	NaN	0,058691	1,22729	0,318415	2,02085	0,00265694	NaN	NaN	NaN
NaN	NaN	NaN	-2,14554	NaN	-0,38361	-3,3178	NaN	NaN	NaN
NaN	NaN	NaN	-0,579128	NaN	-0,540117	-0,660789	NaN	NaN	NaN
0,248318	-0,551594	0,843948	0,413052	NaN	0,480059	-0,00156403	-0,39991	0,237029	-0,234132
-1,27113	-2,20267	-0,437115	0,221125	-1,28747	-2,18392	-0,117949	-0,434786	0,183152	-0,534705
NaN	NaN	NaN	NaN	NaN	NaN	NaN	-2,01971	-1,94916	-1,55966
NaN	NaN	NaN	NaN	NaN	NaN	NaN	NaN	NaN	NaN
-1,63568	-1,69751	-1,22749	-1,39591	NaN	-0,949369	-1,26342	NaN	NaN	NaN
NaN	NaN	NaN	NaN	NaN	NaN	NaN	NaN	NaN	NaN
7,80122	NaN	8,00528	NaN	8,78271	NaN	NaN	8,36538	7,93333	8,1371
-0,316521	-1,12047	-1,10189	0,222464	-1,10235	NaN	-0,885839	NaN	-1,15001	NaN
-1,19439	-1,14837	0,638578	-1,15914	0,261845	NaN	0,384886	1,0374	0,658299	1,39492
NaN	NaN	NaN	NaN	NaN	NaN	NaN	NaN	NaN	NaN
-0,453522	-1,68624	-1,63594	-1,9208	-1,09056	NaN	-1,29115	-0,513281	-1,82524	-1,56019
-0,453522	-1,68624	-1,63594	-1,9208	-1,09056	NaN	-1,29115	-0,513281	-1,82524	-1,56019
2,11089	1,96025	1,30103	2,8325	1,50795	1,78167	2,56328	1,62931	1,07434	1,41709
-0,199638	NaN	-1,94549	-0,12158	NaN	NaN	-0,879988	-0,572731	-1,10463	-2,09832
NaN	-1,27554	NaN	-0,92956	NaN	0,12075	NaN	NaN	NaN	NaN
-0,59264	-0,199389	NaN	0,271458	-1,26735	-0,509872	-0,589079	-0,915264	NaN	-1,23343
NaN	NaN	NaN	NaN	NaN	NaN	NaN	NaN	NaN	NaN
NaN	NaN	NaN	NaN	NaN	0,826183	NaN	NaN	NaN	NaN
NaN	NaN	NaN	NaN	NaN	NaN	NaN	0,0804253	-0,560489	-1,66691
NaN	NaN	NaN	NaN	NaN	NaN	NaN	-1,71791	-1,35754	-0,967225
NaN	NaN	NaN	0,222418	-4,26618	NaN	-0,218237	NaN	NaN	NaN
NaN	NaN	NaN	0,222418	-4,26618	NaN	-0,218237	NaN	NaN	NaN

Polito et al. Universal ready-to-use immunotherapeutic approach for the treatment of cancer: expanded and activated polyclonal γδ memory T cells.
Phosphorylation sites

NaN	-0,454662	-0,58094	0,74527	NaN	1,41378	1,62714	NaN	-0,264237	0,489181
1,19538	-0,454662	-0,58094	0,74527	0,615227	1,41378	1,62714	0,272766	-0,264237	0,489181
NaN	NaN	NaN	0,31403	NaN	-0,798851	-1,3944	NaN	NaN	NaN
4,52886	5,15303	3,83112	2,00938	5,0736	-1,16965	3,40203	4,28451	4,85692	4,94571
0,402168	0,930117	NaN	0,117458	0,22337	0,770792	1,22435	-0,0235367	0,839644	0,51472
NaN	-0,178205	0,903631	NaN	-0,134043	0,275087	0,107517	0,997978	0,638543	1,63321
2,59698	2,1712	2,2531	2,04746	2,33897	1,76735	2,24905	NaN	1,69182	2,08542
NaN	NaN	NaN	NaN	NaN	NaN	NaN	0,640587	-1,59654	-0,0143557
2,03912	1,86532	1,13144	1,04599	2,45448	1,21079	1,87811	0,901268	0,714498	0,685016
2,03912	1,86532	1,13144	1,04599	2,45448	1,21079	1,87811	0,901268	0,714498	0,685016
NaN	NaN	NaN	0,631218	NaN	1,59801	-0,193239	NaN	NaN	NaN
NaN	NaN	NaN	0,51742	0,45224	0,707695	NaN	NaN	-0,66392	-0,26728
NaN	NaN	NaN	-1,0559	-1,40302	-0,619968	-1,75937	NaN	NaN	NaN
0,108778	NaN	NaN	1,21444	1,56921	-0,572737	0,882658	NaN	NaN	NaN
-0,226809	NaN	-0,865183	-2,01699	-0,270842	-1,25467	-1,18653	-0,653877	-1,073	-1,07623
-0,723364	-2,1828	-1,01596	-0,472572	-0,886871	-0,985809	-0,544559	-2,12664	-1,76155	-0,609694
NaN	NaN	1,21215	-0,999025	NaN	-0,782557	-0,286373	NaN	NaN	NaN
0,936901	1,22401	0,967134	1,97683	0,497372	0,667669	1,32857	NaN	NaN	NaN
1,9279	3,57139	1,79513	2,45804	3,16718	2,73071	3,77273	1,64295	2,2192	2,70929
NaN	1,96074	NaN	2,29073	NaN	NaN	2,21937	1,83793	2,18226	NaN
0,228926	1,85429	NaN	-0,682074	-2,56264	0,785883	1,65025	NaN	NaN	NaN
NaN	2,6191	-0,811743	1,18118	NaN	NaN	1,9996	NaN	NaN	NaN
NaN	NaN	NaN	NaN	NaN	NaN	NaN	NaN	NaN	NaN
NaN	3,34724	1,36891	1,37224	2,72039	-0,025631	2,76887	NaN	1,31665	2,50841
NaN	2,65496	1,11009	NaN	3,10509	NaN	NaN	0,518009	0,76835	1,81729
NaN	1,18233	NaN	NaN	NaN	NaN	NaN	NaN	NaN	NaN
0,570982	3,46537	1,92069	1,69318	3,86009	-0,0149364	2,97027	1,6928	1,5082	2,69504
NaN	NaN	NaN	2,21895	NaN	NaN	NaN	1,05203	1,41668	2,80326
1,02849	1,27826	-1,07409	0,296595	0,554804	-0,53195	0,657921	NaN	0,205396	-0,173503
3,20304	3,46198	1,74915	2,72909	1,04848	3,21526	2,33377	2,15609	3,67712	1,24158
NaN	NaN	NaN	-1,05973	-1,80442	-0,476059	-1,08899	NaN	NaN	NaN
4,84045	NaN	4,38754	3,90696	5,18909	3,57667	2,81644	3,49278	2,79655	2,8321
1,42303	2,1106	1,31907	1,06606	1,70604	3,94127	1,62272	NaN	NaN	1,55867
NaN	NaN	NaN	NaN	NaN	NaN	NaN	-1,93592	-2,10348	NaN

Polito et al. Universal ready-to-use immunotherapeutic approach for the treatment of cancer: expanded and activated polyclonal γδ memory T cells.
Phosphorylation sites

0,50115	-0,681482	-1,72794	-0,143787	0,660486	0,782961	0,60928	-3,35314	-2,59908	-1,26287
-1,37168	NaN	-1,09331	0,0811672	NaN	0,392027	-1,8212	NaN	-0,730077	NaN
-0,905785	-1,08838	-0,701298	-0,561306	-0,218428	-0,729794	-1,00049	-0,329048	-0,162993	0,249747
-0,905785	-1,08838	-0,701298	-0,561306	-0,218428	-0,729794	-1,00049	-0,329048	-0,162993	0,249747
0,696815	NaN	-0,157234	0,764334	1,84778	1,01247	-0,753025	-0,916111	0,565866	0,554265
NaN	NaN	NaN	-1,31515	0,0126247	NaN	-0,411718	-2,35194	-1,72359	-0,987784
NaN	NaN	NaN	3,16196	-3,02835	3,01658	1,52477	NaN	NaN	NaN
NaN	NaN	NaN	3,16196	-3,02835	3,01658	1,52477	NaN	NaN	NaN
1,01673	1,38459	-0,222855	NaN	0,463543	NaN	NaN	0,24975	NaN	NaN
NaN	NaN	NaN	NaN	NaN	NaN	NaN	NaN	NaN	NaN
NaN	2,07964	NaN	NaN	NaN	NaN	NaN	1,06341	1,76618	1,16852
0,675032	0,521551	0,295839	0,538076	1,82789	0,951624	0,93046	-0,372011	-0,799201	-0,774602
NaN	NaN	NaN	NaN	NaN	NaN	NaN	NaN	NaN	NaN
NaN	-0,683706	NaN	-2,20498	-0,331478	NaN	-1,45181	NaN	NaN	NaN
NaN	-0,683706	NaN	-2,20498	-0,331478	NaN	-1,45181	NaN	NaN	NaN
NaN	-0,683706	NaN	-2,20498	-0,331478	NaN	-1,45181	NaN	NaN	NaN
NaN	-0,683706	NaN	-2,20498	-0,331478	NaN	-1,45181	NaN	NaN	NaN
NaN	NaN	NaN	0,492811	NaN	1,36734	0,76923	-0,921722	NaN	NaN
NaN	NaN	NaN	-0,609068	NaN	1,59654	0,76923	-0,921722	NaN	NaN
2,10628	1,2541	-1,86437	2,41736	0,908051	2,9084	2,35995	1,04092	2,50495	1,3838
2,164	1,34147	-0,95355	2,32584	0,908051	2,66799	2,77355	1,37037	2,36395	1,99453
1,49416	4,23337	NaN	0,649261	NaN	0,496971	1,90682	-1,75346	1,98531	-0,102475
1,49416	4,23337	NaN	0,649261	NaN	0,496971	1,90682	-1,75346	1,98531	-0,102475
1,49416	4,23337	NaN	0,649261	NaN	0,496971	1,90682	-1,75346	1,98531	-0,102475
1,14883	NaN	NaN	1,03311	0,232475	NaN	-0,298965	0,108343	-0,462113	-2,28837
1,14883	NaN	NaN	1,03311	0,232475	NaN	-0,298965	0,108343	-0,462113	-2,28837
1,6011	1,31373	-0,812786	0,981352	0,567427	1,55412	0,448334	-0,0675545	-0,19527	-0,0364046
1,6011	1,31373	-0,812786	0,981352	0,567427	1,55412	0,448334	-0,0675545	-0,19527	-0,0364046
1,07606	0,150335	0,496044	-2,76224	0,405653	NaN	-1,62495	0,800043	-0,0596895	-0,149453
1,07606	0,150335	0,496044	-2,76224	0,405653	NaN	-1,62495	0,800043	-0,0596895	-0,149453
NaN	NaN	NaN	-2,76224	0,405653	NaN	-1,62495	0,800043	NaN	-0,149453
1,79495	0,051445	-0,0531082	0,0173225	0,834335	NaN	-0,167007	1,07577	1,40766	0,362409
1,79495	0,051445	-0,0531082	0,0173225	0,834335	NaN	-0,167007	1,07577	1,40766	0,362409
1,79495	0,051445	-0,0531082	0,0173225	0,834335	NaN	-0,167007	1,07577	1,40766	0,362409

Polito et al. Universal ready-to-use immunotherapeutic approach for the treatment of cancer: expanded and activated polyclonal γδ memory T cells.
Phosphorylation sites

NaN	NaN	NaN	-1,85037	NaN	-0,824045	-1,36337	NaN	NaN	NaN
NaN	NaN	NaN	-1,85037	NaN	-0,824045	-1,36337	NaN	NaN	NaN
1,28732	2,93208	NaN	0,900547	NaN	0,359798	1,73076	-0,581139	0,973924	0,0655336
1,28732	2,93208	NaN	0,900547	NaN	0,359798	NaN	-0,581139	0,973924	0,0655336
1,28732	2,93208	NaN	0,900547	NaN	0,359798	1,73076	-0,581139	0,973924	0,0655336
3,54007	3,9004	2,62421	3,38119	3,64603	3,8994	4,06883	3,44129	3,33316	2,99196
3,54007	3,9004	2,62421	3,38119	3,64603	3,8994	4,06883	3,44129	3,33316	2,99196
3,54007	3,9004	2,62421	3,38119	3,64603	3,8994	4,06883	3,44129	3,33316	2,99196
-1,47108	0,141747	-3,00324	NaN	NaN	NaN	NaN	NaN	NaN	NaN
-1,47108	0,141747	-3,00324	NaN	NaN	NaN	NaN	NaN	NaN	NaN
1,16564	1,1544	-0,174856	1,07166	0,509924	-0,324326	0,465317	-0,369818	0,305274	NaN
1,16564	1,1544	-0,174856	1,07166	0,509924	-0,324326	0,465317	-0,369818	0,305274	NaN
1,16564	1,1544	-0,174856	1,07166	0,509924	-0,324326	0,465317	-0,369818	0,305274	NaN
NaN	NaN	NaN	-1,85868	NaN	-0,810404	-2,80245	NaN	NaN	NaN
NaN	NaN	NaN	-1,85868	NaN	-0,810404	-2,80245	NaN	NaN	NaN
NaN	NaN	NaN	-1,85868	NaN	-0,810404	-2,80245	NaN	NaN	NaN
-0,489513	-1,21581	-1,59106	-1,1431	-0,629457	-0,879824	0,308161	-1,11234	-0,110307	-1,45881
-0,489513	-1,21581	-1,59106	-1,1431	-0,629457	-0,879824	0,308161	-1,11234	-0,110307	-1,45881
-0,489513	-1,21581	NaN	-1,1431	-0,629457	-0,879824	0,308161	NaN	-0,110307	-1,45881
NaN	NaN	NaN	-0,235991	NaN	0,249813	-1,04448	NaN	NaN	NaN
NaN	NaN	NaN	-0,235991	NaN	0,249813	-1,04448	NaN	NaN	NaN
NaN	0,441105	-1,37752	-1,24555	NaN	-1,84798	-0,470947	NaN	NaN	-0,717412
NaN	NaN	NaN	-0,735182	NaN	-0,0804539	-0,687101	NaN	NaN	NaN
NaN	NaN	NaN	-0,735182	NaN	-0,0804539	-0,687101	NaN	NaN	NaN
2,06287	0,904213	1,91407	2,14457	2,41845	1,01009	2,39776	2,32345	2,6062	2,41778
2,06287	0,904213	1,91407	2,14457	2,41845	1,01009	2,39776	2,32345	2,6062	2,41778
2,06287	0,904213	1,91407	2,14457	2,41845	1,01009	2,39776	2,32345	2,6062	2,41778
1,77155	1,53774	1,16576	NaN	NaN	NaN	NaN	0,508869	1,38585	0,606658
1,77155	1,53774	1,16576	NaN	NaN	NaN	NaN	0,508869	1,38585	0,606658
1,77155	1,53774	1,16576	NaN	NaN	NaN	NaN	0,508869	1,38585	0,606658
0,896826	1,30688	1,17761	1,78859	0,854555	2,14912	1,14614	NaN	NaN	NaN
0,896826	1,30688	1,17761	1,78859	0,854555	2,14912	1,14614	NaN	NaN	NaN
NaN	NaN	NaN	NaN	NaN	NaN	NaN	1,85348	1,58547	4,5754
NaN	NaN	1,69328	2,18264	2,71681	0,507967	2,60027	NaN	2,44279	2,32254

Polito et al. Universal ready-to-use immunotherapeutic approach for the treatment of cancer: expanded and activated polyclonal γδ memory T cells.
Phosphorylation sites

NaN	NaN	NaN	0,749649	1,33987	0,507967	1,0433	NaN	NaN	1,02411
NaN	NaN	1,69328	2,18264	2,71681	0,507967	2,60027	NaN	2,44279	2,81468
NaN	-1,31291	NaN	NaN	NaN	0,815941	NaN	NaN	NaN	NaN
NaN	-1,31291	-1,61735	0,469236	-0,228619	0,815941	NaN	NaN	NaN	NaN
NaN	1,0271	NaN	NaN	NaN	NaN	NaN	NaN	NaN	NaN
NaN	1,0271	NaN	NaN	NaN	NaN	NaN	NaN	NaN	NaN
2,56573	4,7703	3,02119	3,00778	4,30761	-0,131262	4,60724	2,52462	3,54662	NaN
2,56573	4,7703	3,02119	3,00778	4,30761	-0,131262	4,60724	2,52462	3,54662	NaN
2,56573	4,7703	3,02119	3,00778	4,30761	-0,131262	4,60724	2,52462	3,54662	NaN
NaN	1,0271	NaN	NaN	NaN	NaN	NaN	NaN	NaN	NaN
NaN	NaN	NaN	-0,686769	-1,08036	-0,40457	-0,475849	NaN	NaN	NaN
NaN	NaN	NaN	-0,686769	-1,08036	-0,40457	-0,475849	NaN	NaN	NaN
NaN	NaN	NaN	0,725506	NaN	1,51045	1,05281	NaN	NaN	NaN
NaN	NaN	NaN	0,725506	NaN	1,51045	1,05281	NaN	NaN	NaN
NaN	NaN	NaN	0,725506	NaN	1,51045	1,05281	NaN	NaN	NaN
NaN	NaN	NaN	NaN	NaN	NaN	NaN	NaN	NaN	NaN
NaN	NaN	NaN	NaN	NaN	NaN	NaN	NaN	NaN	NaN
NaN	NaN	NaN	NaN	NaN	NaN	NaN	NaN	NaN	NaN
-1,38078	-0,974049	-1,66126	-0,329931	-1,08856	-1,26783	0,339794	-2,47343	-1,84802	NaN
-1,38078	-0,974049	-1,66126	-0,329931	-1,08856	-1,26783	0,339794	-2,47343	-1,84802	NaN
-1,38078	-0,974049	-1,66126	-0,329931	-1,08856	-1,26783	0,339794	-2,47343	-1,84802	NaN
3,59654	3,2701	-2,42842	2,9169	2,77666	1,6267	2,81329	1,61065	2,43863	1,51645
3,59654	3,2701	-2,42842	2,9169	2,77666	1,6267	2,81329	1,61065	2,43863	1,51645
3,59654	3,2701	-2,42842	2,9169	2,77666	1,6267	2,81329	1,61065	2,43863	1,51645
2,12201	0,325026	1,26258	-1,20337	2,27632	NaN	NaN	1,5244	2,03074	1,3395
2,12201	0,325026	1,26258	-1,20337	2,27632	NaN	NaN	1,5244	2,03074	1,3395
2,12201	0,325026	1,26258	-1,20337	2,27632	NaN	NaN	1,5244	2,03074	1,3395
2,48199	1,77594	1,45532	0,785711	1,87229	NaN	1,054	1,2733	1,77214	0,891847
2,48199	1,77594	1,45532	0,785711	1,87229	NaN	1,054	1,2733	1,77214	0,891847
2,48199	1,77594	1,45532	0,785711	1,87229	NaN	1,054	1,2733	1,77214	0,891847
1,52822	NaN	0,441195	0,891064	0,68722	1,26046	-0,371353	-0,0214596	-0,734405	0,0428133
1,52822	NaN	0,441195	0,891064	0,68722	1,26046	-0,371353	-0,0214596	-0,734405	0,0428133
1,52822	NaN	NaN	0,891064	NaN	NaN	-0,371353	-0,0214596	-0,734405	NaN
0,0371704	1,85295	0,227329	0,982002	0,971457	-0,898958	2,08761	-0,783718	0,458411	1,07631

Polito et al. Universal ready-to-use immunotherapeutic approach for the treatment of cancer: expanded and activated polyclonal γδ memory T cells.
Phosphorylation sites

-0,570528	-2,45464	-0,90873	-1,40007	0,808788	-1,3009	1,20383	-0,972258	0,126151	0,689006
-0,570528	-2,45464	-0,90873	-1,40007	0,808788	-1,3009	1,20383	-0,972258	0,126151	0,689006
0,0371704	1,85295	0,227329	0,982002	0,971457	-0,898958	2,08761	-0,783718	0,458411	1,07631
-0,570528	-2,45464	-0,90873	-1,40007	0,808788	-1,3009	1,20383	-0,972258	0,126151	0,689006
0,0371704	1,85295	0,227329	0,982002	0,971457	-0,898958	2,08761	-0,783718	0,458411	1,07631
2,5743	-3,4582	2,10497	-4,08348	3,22037	2,71489	NaN	2,46967	3,5714	3,15052
2,5743	-3,4582	2,10497	-4,08348	3,22037	2,71489	NaN	2,46967	3,5714	3,15052
2,5743	-3,4582	2,10497	-4,08348	3,22037	2,71489	NaN	2,46967	3,5714	3,15052
-0,86249	-0,66432	-1,12866	-0,855249	-0,877544	-1,26809	-0,375233	-0,792461	-0,964688	-1,54498
-0,86249	-0,66432	-1,12866	-0,855249	-0,877544	-1,26809	NaN	-0,792461	-0,964688	-1,54498
-0,86249	-0,66432	-1,12866	-0,855249	-0,877544	-1,26809	-0,375233	-0,792461	-0,964688	-1,54498
NaN	NaN	NaN	0,295382	-0,860975	-0,0594482	NaN	NaN	NaN	NaN
1,01982	0,250507	0,172413	1,06518	-2,02898	2,08778	0,417812	0,101456	0,521121	-0,619674
1,01982	0,250507	0,172413	1,06518	-2,02898	2,08778	0,417812	0,101456	0,521121	-0,619674
-0,529613	-0,657301	-2,31712	-0,996088	NaN	NaN	-0,682861	NaN	NaN	NaN
NaN	NaN	NaN	-2,41558	-1,2232	-1,58788	NaN	NaN	NaN	NaN
NaN	NaN	NaN	-2,41558	-1,2232	-1,58788	NaN	NaN	NaN	NaN
NaN	NaN	NaN	0,949438	-1,91223	1,98724	1,04904	NaN	NaN	NaN
NaN	NaN	NaN	0,949438	-1,91223	1,98724	1,04904	NaN	NaN	NaN
NaN	NaN	NaN	0,949438	-1,91223	1,98724	1,04904	NaN	NaN	NaN
NaN	1,13382	1,43233	3,29066	2,61011	3,78052	1,8928	1,49656	0,590728	NaN
NaN	1,13382	1,43233	3,29066	2,61011	3,78052	1,8928	1,49656	0,590728	NaN
NaN	1,13382	1,43233	3,29066	2,61011	3,78052	1,8928	1,49656	0,590728	NaN
NaN	NaN	NaN	0,181557	NaN	0,195385	-0,755211	NaN	NaN	NaN
NaN	NaN	NaN	0,181557	NaN	0,195385	-0,755211	NaN	NaN	NaN
NaN	NaN	NaN	0,181557	NaN	0,195385	-0,755211	NaN	NaN	NaN
-2,10143	NaN	-1,90357	-1,84785	-0,499249	NaN	-0,500107	NaN	-1,46656	-0,562566
-2,10143	NaN	-1,90357	-1,84785	-0,499249	NaN	-0,500107	NaN	-1,46656	-0,562566
NaN	NaN	NaN	-1,84785	-0,499249	NaN	-0,500107	NaN	-1,46656	NaN
0,526831	0,0687046	0,342474	-1,74439	0,932585	-1,18943	-1,28744	NaN	NaN	NaN
0,526831	0,0687046	0,342474	-1,74439	0,932585	-1,18943	-1,28744	NaN	NaN	NaN
-1,87202	-1,03886	-2,46692	NaN	NaN	NaN	NaN	NaN	NaN	NaN
-1,87202	-1,03886	-2,46692	NaN	NaN	NaN	NaN	NaN	NaN	NaN
-1,87202	-1,03886	-2,46692	NaN	NaN	NaN	NaN	NaN	NaN	NaN

Polito et al. Universal ready-to-use immunotherapeutic approach for the treatment of cancer: expanded and activated polyclonal γδ memory T cells.
Phosphorylation sites

1,07849	-0,814417	0,486485	0,359468	0,0377426	NaN	0,517469	0,890545	0,657519	0,875552
1,07849	-0,814417	0,486485	0,359468	0,0377426	NaN	0,517469	0,890545	0,657519	0,875552
1,07849	-0,814417	0,486485	0,359468	0,0377426	NaN	0,517469	0,890545	0,657519	0,875552
6,2303	4,61775	6,28575	5,85477	6,06535	4,58543	5,97967	6,40622	6,01971	2,98061
NaN	NaN	NaN	0,679304	2,01081	1,72629	0,25894	2,15216	2,10689	2,4164
NaN	NaN	-0,404148	NaN	NaN	NaN	NaN	NaN	NaN	NaN
NaN	NaN	NaN	NaN	NaN	NaN	NaN	NaN	NaN	NaN
0,228754	NaN	-0,404148	NaN	NaN	-0,85972	NaN	NaN	NaN	NaN
4,54109	4,83695	3,62787	4,0881	4,45295	4,434	3,89501	2,74049	3,36852	3,2953
4,54109	4,83695	3,62787	4,0881	4,45295	4,434	3,89501	2,74049	3,36852	3,2953
4,54109	4,83695	3,62787	4,0881	4,45295	4,434	3,89501	2,74049	3,36852	3,2953
1,31549	0,587521	-0,99106	NaN	-0,17602	NaN	0,0188713	NaN	NaN	NaN
1,31549	0,587521	-0,99106	NaN	-0,17602	NaN	0,0188713	NaN	NaN	NaN
1,31549	0,587521	-0,99106	NaN	-0,17602	NaN	0,0188713	NaN	NaN	NaN
-0,162144	-1,41809	NaN	1,40043	-0,715033	NaN	0,94655	-0,768681	-0,480224	-1,46501
-0,162144	-1,41809	NaN	1,40043	-0,715033	NaN	0,94655	-0,768681	-0,480224	-1,46501
-0,162144	-1,41809	NaN	1,40043	-0,715033	NaN	0,94655	-0,768681	-0,480224	-1,46501
1,0587	-0,0648232	0,0739956	NaN	1,70543	NaN	-1,01279	NaN	-0,9581	-1,61812
1,0587	-0,0648232	0,0739956	NaN	1,70543	NaN	-1,01279	NaN	-0,9581	-1,61812
1,0587	-0,0648232	0,0739956	NaN	1,70543	NaN	-1,01279	NaN	-0,9581	-1,61812
7,79911	7,70868	7,08666	5,69651	5,66718	4,7018	5,78793	7,45176	8,03777	7,68334
7,37617	8,05739	5,85774	6,03339	5,92597	6,01259	5,9583	6,28332	6,18332	6,72336
2,63826	0,0619602	NaN	NaN	3,54527	NaN	1,84987	3,56482	2,91606	4,82816
-1,66937	NaN	NaN	-2,68054	-3,05821	-3,04852	-1,21728	NaN	-0,448083	-1,41385
-1,66937	NaN	NaN	-2,68054	-3,05821	-3,04852	-1,21728	NaN	-0,448083	-1,41385
-1,66937	-0,100254	NaN	-2,68054	-3,05821	-3,04852	-1,21728	NaN	-0,448083	-1,41385
-1,66937	NaN	NaN	-2,68054	-3,05821	-3,04852	-1,21728	NaN	-0,448083	-1,41385
-1,66937	-0,100254	NaN	-2,68054	-3,05821	-3,04852	-1,21728	NaN	-0,448083	-1,41385
3,06038	1,70222	2,09717	2,62456	2,21958	2,53469	2,18896	2,33837	3,40115	1,86828
3,06038	1,70222	2,09717	2,62456	2,21958	2,53469	2,18896	2,33837	3,40115	1,86828
NaN	NaN	2,09717	NaN	NaN	2,53469	2,18896	2,33837	3,40115	1,86828
-0,537237	-0,625589	-0,519703	-1,25337	-0,105314	NaN	NaN	NaN	NaN	NaN
-0,537237	-0,625589	-0,519703	-1,25337	-0,105314	NaN	NaN	NaN	NaN	NaN
-0,537237	-0,625589	-0,519703	-1,25337	-0,105314	NaN	NaN	NaN	NaN	NaN

Polito et al. Universal ready-to-use immunotherapeutic approach for the treatment of cancer: expanded and activated polyclonal γδ memory T cells.
Phosphorylation sites

NaN	NaN	NaN	0,492159	NaN	-1,8111	1,04904	NaN	NaN	NaN
NaN	NaN	NaN	0,492159	NaN	-1,8111	1,04904	NaN	NaN	NaN
NaN	NaN	NaN	0,492159	NaN	-1,8111	1,04904	NaN	NaN	NaN
NaN	NaN	NaN	NaN	NaN	NaN	NaN	NaN	NaN	NaN
NaN	NaN	NaN	NaN	NaN	NaN	NaN	NaN	NaN	NaN
NaN	NaN	NaN	NaN	NaN	NaN	NaN	NaN	NaN	NaN
NaN	NaN	NaN	NaN	NaN	NaN	NaN	NaN	NaN	NaN
NaN	NaN	NaN	NaN	NaN	NaN	NaN	NaN	NaN	NaN
1,54293	2,51388	0,477636	1,10913	0,482307	1,65475	1,67901	0,549877	1,43036	1,47965
1,54293	2,51388	0,477636	1,10913	0,482307	1,65475	1,67901	0,549877	1,43036	1,47965
-0,806881	2,50803	-0,00352287	NaN	0,482307	NaN	NaN	0,549877	1,43036	-1,70554
1,22786	-5,43905	-1,34039	1,10913	NaN	1,65475	1,67901	NaN	NaN	1,31167
0,597717	1,10897	1,07721	0,217398	0,756765	0,294075	1,12846	0,328453	2,17442	1,54016
0,597717	1,10897	1,07721	0,217398	0,756765	0,294075	1,12846	0,328453	2,17442	1,54016
0,597717	1,10897	1,07721	0,217398	0,756765	0,294075	1,12846	0,328453	2,17442	1,54016
2,34245	4,30226	3,22163	-2,28702	4,25795	1,52697	4,86476	2,42075	3,65151	4,05881
1,70389	1,41488	1,50134	NaN	1,751	1,54991	NaN	NaN	-0,114942	1,05978
1,4639	-0,386425	0,890541	-1,31593	0,998833	NaN	-0,518845	0,460295	0,154767	0,0297441
1,4639	-0,386425	0,890541	-1,31593	0,998833	NaN	-0,518845	0,460295	0,154767	0,0297441
1,4639	-0,386425	0,890541	-1,31593	0,998833	NaN	-0,518845	0,460295	0,154767	0,0297441
1,70389	1,41488	1,50134	NaN	1,751	1,54991	NaN	NaN	-0,114942	1,05978
3,03974	2,2128	1,45028	2,14718	2,77334	2,30515	1,52788	1,61342	1,14977	1,1315
3,03974	2,2128	1,45028	2,14718	2,77334	2,30515	1,52788	1,61342	1,14977	1,1315
2,96762	2,2128	1,45028	2,14718	2,77334	NaN	1,52788	1,61342	1,14977	1,1315
1,73908	1,10369	-0,0123463	NaN	0,00680733	NaN	NaN	-0,455328	-0,406306	-0,073884
1,73908	1,10369	-0,0123463	NaN	0,00680733	NaN	NaN	-0,455328	-0,406306	-0,073884
1,73908	NaN	-0,0123463	NaN	0,00680733	NaN	NaN	-0,455328	-0,406306	-0,073884
1,73908	1,10369	NaN	NaN	0,00680733	NaN	NaN	-0,455328	-0,406306	-0,073884
NaN	NaN	NaN	NaN	-0,432152	2,22049	NaN	NaN	NaN	NaN
NaN	NaN	NaN	NaN	-0,432152	2,22049	NaN	NaN	NaN	NaN
NaN	NaN	NaN	NaN	-0,432152	2,22049	NaN	NaN	NaN	NaN
NaN	0,75786	NaN	0,99967	NaN	NaN	NaN	0,413834	-0,307313	1,09898
0,528595	0,890497	-0,652603	NaN	0,628822	0,935236	0,931398	0,413834	-0,307313	1,09898
-0,7241	-0,75774	-2,06501	0,10763	-0,542906	NaN	-0,422371	-0,707773	-1,45122	-1,5149

Polito et al. Universal ready-to-use immunotherapeutic approach for the treatment of cancer: expanded and activated polyclonal γδ memory T cells.
Phosphorylation sites

-0,7241	-0,75774	-2,06501	0,10763	-0,542906	NaN	-0,422371	-0,707773	-1,45122	-1,5149
NaN	NaN	NaN	-1,61793	-2,34272	NaN	-1,76444	NaN	NaN	NaN
NaN	NaN	NaN	0,10763	-0,542906	NaN	-0,422371	-0,707773	-1,45122	-1,5149
-0,297962	-1,73846	-1,34168	0,306067	-0,0699043	-0,567381	0,594215	-0,318466	0,852536	-0,176639
-0,297962	-1,73846	-1,34168	0,306067	-0,0699043	-0,567381	0,594215	-0,318466	0,852536	-0,176639
-0,297962	-1,73846	-1,34168	0,306067	-0,0699043	-0,567381	0,594215	-0,318466	0,852536	-0,176639
-1,2589	0,865492	-1,66005	-0,588219	-0,445719	-1,52949	0,318071	-1,74316	0,0370913	-0,655988
-1,2589	0,865492	-1,66005	-0,588219	-0,445719	-1,52949	0,318071	-1,74316	0,0370913	-0,655988
-1,2589	0,865492	-1,66005	-0,588219	-0,445719	-1,52949	0,318071	-1,74316	0,0370913	-0,655988
1,98331	2,36703	0,160534	1,11381	2,13025	1,27107	2,1164	1,28992	1,51589	1,35017
1,98331	2,36703	0,160534	1,11381	2,13025	1,27107	2,1164	1,28992	1,51589	1,35017
1,98331	2,36703	0,160534	1,11381	2,13025	1,27107	2,1164	1,28992	1,51589	1,35017
0,361433	1,95697	2,28819	0,790182	3,3602	-0,284592	2,70255	1,32847	1,05256	1,87905
0,361433	1,95697	2,28819	0,790182	3,3602	-0,284592	2,70255	1,32847	1,05256	1,87905
NaN	NaN	NaN	1,15096	NaN	NaN	1,39168	NaN	NaN	NaN
NaN	NaN	NaN	1,15096	NaN	NaN	1,39168	NaN	NaN	NaN
NaN	NaN	NaN	1,15096	NaN	NaN	1,39168	NaN	NaN	NaN
-1,16642	-1,05194	NaN	NaN	-0,567764	NaN	-0,233685	NaN	-1,42869	NaN
-1,16642	-1,05194	NaN	NaN	-0,567764	NaN	-0,233685	NaN	-1,42869	NaN
-1,16642	-1,05194	NaN	NaN	-0,567764	NaN	-0,233685	NaN	-1,42869	NaN
-1,04167	0,0321503	-3,31856	-1,0258	-0,800894	-0,202831	-0,5732	NaN	NaN	NaN
-1,04167	0,0321503	-3,31856	-1,0258	-0,800894	-0,202831	-0,5732	NaN	NaN	NaN
-1,04167	0,0321503	-3,31856	-1,0258	-0,800894	-0,202831	-0,5732	NaN	NaN	NaN
NaN	NaN	NaN	NaN	NaN	NaN	NaN	1,68359	1,42403	1,0691
NaN	NaN	NaN	2,09048	-2,28283	NaN	NaN	NaN	NaN	NaN
NaN	NaN	NaN	2,09048	-2,28283	1,55252	NaN	NaN	NaN	NaN
NaN	NaN	NaN	2,09048	-2,28283	1,55252	NaN	-0,331211	-0,186818	-1,33404
NaN	NaN	NaN	0,367756	NaN	-0,284124	-0,297096	NaN	NaN	NaN
NaN	NaN	NaN	0,367756	NaN	-0,284124	-0,297096	NaN	NaN	NaN
NaN	NaN	NaN	0,367756	NaN	-0,284124	-0,297096	NaN	NaN	NaN
NaN	NaN	NaN	-0,149261	NaN	1,93062	-0,639467	NaN	NaN	NaN
NaN	NaN	NaN	-0,149261	NaN	1,93062	-0,639467	NaN	NaN	NaN
NaN	NaN	NaN	-0,149261	NaN	1,93062	-0,639467	NaN	NaN	NaN
NaN	3,4147	NaN	0,559813	NaN	NaN	2,33164	0,372738	1,65849	1,62728

Polito et al. Universal ready-to-use immunotherapeutic approach for the treatment of cancer: expanded and activated polyclonal γδ memory T cells.
Phosphorylation sites

NaN	3,4147	NaN	0,559813	NaN	NaN	2,33164	0,372738	1,65849	1,62728
NaN	3,4147	NaN	0,559813	NaN	NaN	2,33164	0,372738	1,65849	1,62728
0,719088	0,641245	0,478884	0,670784	0,633577	0,128115	1,98562	0,569525	0,770635	0,850318
0,719088	0,641245	0,478884	0,670784	0,633577	0,128115	1,98562	0,569525	0,770635	0,850318
0,719088	0,641245	0,478884	0,670784	0,633577	0,128115	1,98562	0,569525	0,770635	0,850318
NaN	NaN	NaN	-0,239393	-2,37909	0,505606	-1,06593	NaN	NaN	NaN
NaN	NaN	NaN	-0,239393	-2,37909	0,505606	-1,06593	NaN	NaN	NaN
NaN	NaN	NaN	-0,597784	NaN	-0,01651	-0,801277	NaN	NaN	NaN
NaN	NaN	NaN	-0,597784	NaN	-0,01651	-0,801277	NaN	NaN	NaN
NaN	NaN	NaN	-0,268459	NaN	NaN	NaN	NaN	NaN	NaN
NaN	NaN	NaN	-0,268459	NaN	NaN	NaN	NaN	NaN	NaN
NaN	NaN	NaN	-0,268459	NaN	NaN	NaN	NaN	NaN	NaN
1,04026	0,304737	0,999407	NaN	0,792234	-0,57983	NaN	1,20916	0,510364	0,364978
1,04026	0,304737	0,999407	0,0860233	0,792234	-0,57983	0,556177	1,20916	0,510364	0,364978
1,04026	0,304737	0,999407	0,0860233	0,792234	-0,57983	0,556177	1,20916	0,510364	0,364978
1,0111	2,01115	0,701843	1,16225	1,79812	2,45447	2,05006	0,390961	0,449098	1,11845
1,0111	2,01115	0,701843	1,16225	1,79812	2,45447	2,05006	0,390961	0,449098	1,11845
1,0111	2,01115	0,701843	1,16225	1,79812	2,45447	2,05006	0,390961	0,449098	1,11845
NaN	-0,185007	NaN	NaN	NaN	NaN	NaN	NaN	NaN	NaN
NaN	-0,185007	NaN	NaN	NaN	NaN	NaN	NaN	NaN	NaN
NaN	-0,185007	NaN	NaN	NaN	NaN	NaN	NaN	NaN	NaN
NaN	NaN	-0,854948	1,54389	-1,73861	1,08323	-0,0284119	NaN	NaN	NaN
NaN	NaN	-0,854948	1,03266	-0,722801	1,95768	0,598442	NaN	NaN	NaN
NaN	NaN	NaN	1,54389	-0,722801	1,09932	-0,603914	NaN	NaN	NaN
NaN	NaN	NaN	NaN	NaN	NaN	NaN	NaN	NaN	NaN
NaN	NaN	NaN	-0,738644	-2,40366	-0,838072	-1,24932	NaN	NaN	NaN
NaN	NaN	NaN	-0,738644	-2,40366	-0,838072	NaN	NaN	NaN	NaN
NaN	NaN	NaN	-0,738644	-2,40366	-0,838072	-1,24932	NaN	NaN	NaN
NaN	NaN	NaN	-0,977909	NaN	0,104012	-0,558018	NaN	NaN	NaN
NaN	NaN	NaN	-0,977909	NaN	0,104012	-0,558018	NaN	NaN	NaN
NaN	-0,909916	NaN	-1,16243	0,147161	0,708136	-1,56283	-1,15675	0,313449	NaN
NaN	NaN	NaN	-1,16243	0,147161	0,708136	-1,56283	NaN	NaN	NaN
NaN	NaN	NaN	NaN	NaN	NaN	NaN	NaN	NaN	NaN
NaN	NaN	NaN	NaN	NaN	NaN	NaN	NaN	NaN	NaN

Polito et al. Universal ready-to-use immunotherapeutic approach for the treatment of cancer: expanded and activated polyclonal γδ memory T cells.
Phosphorylation sites

NaN	NaN	NaN	-0,0384865	-1,98138	-0,551453	0,142578	NaN	NaN	NaN
NaN	NaN	NaN	-0,668978	NaN	-1,31124	0,0874443	NaN	NaN	NaN
NaN	NaN	NaN	-0,668978	NaN	-1,31124	0,0874443	NaN	NaN	NaN
1,30658	1,91693	1,09737	1,54999	1,09886	1,20285	1,73413	0,789389	1,22811	1,38186
1,30658	1,91693	1,09737	1,54999	1,09886	1,20285	1,73413	0,789389	1,22811	1,38186
1,30658	1,91693	1,09737	1,54999	1,09886	1,20285	1,73413	0,789389	1,22811	1,38186
0,544844	-0,0136013	0,150772	2,50235	-0,368513	3,28282	1,77388	0,204859	1,20952	-0,402429
1,13039	0,528912	0,150772	2,50235	1,01805	3,63407	2,24943	0,204859	1,92432	-0,402429
1,13039	0,528912	0,150772	2,50235	1,01805	3,63407	2,24943	0,204859	1,92432	-0,402429
NaN	NaN	NaN	-0,88529	NaN	NaN	0,358391	NaN	NaN	NaN
NaN	NaN	NaN	-0,88529	NaN	NaN	0,358391	NaN	NaN	NaN
NaN	NaN	NaN	-0,88529	NaN	NaN	0,358391	NaN	NaN	NaN
NaN	NaN	NaN	-2,84858	-1,26059	NaN	-1,18496	NaN	NaN	NaN
NaN	NaN	NaN	-2,84858	-1,26059	NaN	-1,18496	NaN	NaN	NaN
-1,76444	0,0586739	-2,95596	NaN	NaN	NaN	-0,676668	NaN	NaN	NaN
1,92119	NaN	0,476191	-0,677685	0,948877	NaN	0,0548363	NaN	NaN	-0,00550556
NaN	-1,96195	NaN	-0,688339	NaN	-1,6586	-1,69124	NaN	NaN	NaN
NaN	-1,96195	NaN	-0,688339	NaN	-1,6586	-1,69124	NaN	NaN	NaN
NaN	-1,96195	NaN	NaN	NaN	-1,6586	NaN	NaN	NaN	NaN
NaN	3,31601	NaN	1,86793	-0,00938606	2,47774	2,68743	NaN	NaN	NaN
NaN	3,31601	NaN	1,86793	-0,00938606	2,47774	2,68743	NaN	NaN	NaN
NaN	3,31601	NaN	1,86793	-0,00938606	2,47774	2,68743	NaN	NaN	NaN
0,862619	0,575548	NaN	0,943428	-0,913939	1,81093	1,022	NaN	0,549563	NaN
0,862619	0,575548	NaN	0,943428	-0,913939	1,81093	1,022	NaN	0,549563	NaN
0,0975571	0,0197105	-0,207966	-0,44136	-0,0417404	-3,34793	-0,175564	-1,26558	0,881101	0,30351
0,0975571	0,0197105	-0,207966	-0,44136	-0,0417404	-3,34793	-0,175564	-1,26558	0,881101	0,30351
0,0975571	0,0197105	-3,11872	NaN	NaN	-3,34793	-0,175564	NaN	NaN	NaN
NaN	2,70317	NaN	-1,40318	1,23071	-0,817736	-1,36907	0,231659	0,274693	-0,155499
-0,237452	2,70317	0,299116	-1,40318	1,23071	-0,817736	-1,36907	0,231659	0,274693	-0,155499
NaN	2,70317	NaN	-1,40318	1,23071	-0,817736	-1,36907	0,231659	NaN	NaN
0,597717	1,10897	1,07721	0,217398	0,756765	0,294075	1,12846	0,328453	2,17442	1,54016
0,597717	1,10897	1,07721	0,217398	0,756765	0,294075	1,12846	0,328453	2,17442	1,54016
2,56588	2,10351	0,299219	1,94996	1,03369	2,81922	1,79509	0,842808	0,781413	1,9943
1,54591	0,984825	0,202694	1,44958	-0,516756	2,42373	0,950951	1,30988	0,465751	0,261626

Polito et al. Universal ready-to-use immunotherapeutic approach for the treatment of cancer: expanded and activated polyclonal γδ memory T cells.
Phosphorylation sites

0,118824	NaN	-1,08623	0,830744	0,325739	NaN	0,809315	1,24748	0,963971	1,65756
2,46369	2,11847	1,25176	1,62985	0,431181	2,44012	1,56059	1,55893	1,00236	1,82132
NaN	NaN	NaN	-1,81909	-0,74003	-1,81228	NaN	NaN	NaN	NaN
0,284428	1,00118	-0,809067	NaN	0,554319	-0,807003	0,940159	-0,52565	NaN	0,352736
0,284428	1,00118	-0,809067	NaN	0,554319	-0,807003	0,940159	-0,52565	NaN	0,352736
-0,0280151	2,11768	-0,0165424	0,533003	-1,34236	0,970278	0,619381	NaN	0,639333	0,0891562
-0,0280151	2,11768	-0,0165424	0,533003	-1,34236	0,970278	0,619381	NaN	0,639333	0,0891562
-0,0280151	2,11768	-0,0165424	0,533003	-1,34236	0,970278	0,619381	NaN	0,639333	0,0891562
NaN	NaN	NaN	-0,950758	NaN	-0,465134	NaN	NaN	NaN	NaN
NaN	NaN	NaN	-0,950758	NaN	-0,465134	NaN	NaN	NaN	NaN
NaN	NaN	NaN	-0,950758	NaN	-0,465134	NaN	NaN	NaN	NaN
NaN	0,549814	-1,92627	-0,5791	-1,71545	0,639252	NaN	NaN	NaN	NaN
NaN	0,549814	-1,92627	-0,5791	-1,71545	0,639252	NaN	NaN	NaN	NaN
-1,05617	NaN	-2,33348	-1,13945	-0,942997	-1,02283	-1,49103	-2,11288	-1,37142	-1,7954
-1,05617	NaN	-2,33348	-1,13945	-0,942997	-1,02283	-1,49103	-2,11288	-1,37142	-1,7954
-1,05617	NaN	-2,33348	-1,13945	-0,942997	-1,02283	-1,49103	-2,11288	-1,37142	-1,7954
NaN	NaN	NaN	1,63838	NaN	0,659498	1,33461	NaN	NaN	NaN
NaN	NaN	NaN	1,63838	NaN	0,659498	1,33461	NaN	NaN	NaN
-0,745346	-1,37559	NaN	-1,05434	-1,48493	-0,399076	-1,0993	NaN	NaN	NaN
-0,745346	-1,37559	NaN	-1,05434	-1,48493	-0,399076	-1,0993	NaN	NaN	NaN
-0,745346	NaN	NaN	-1,05434	-1,48493	-0,399076	-1,0993	NaN	NaN	NaN
NaN	NaN	NaN	1,46491	0,438921	0,821106	0,863871	NaN	NaN	NaN
NaN	NaN	NaN	1,46491	0,438921	0,821106	0,863871	NaN	NaN	NaN
NaN	NaN	NaN	1,46491	0,438921	0,821106	0,863871	NaN	NaN	NaN
NaN	NaN	NaN	0,829193	-1,03078	0,800957	0,413809	NaN	NaN	NaN
NaN	NaN	NaN	NaN	-0,420727	NaN	1,76154	NaN	NaN	NaN
NaN	NaN	NaN	0,102846	NaN	-0,74321	-2,11635	NaN	NaN	NaN
NaN	NaN	NaN	0,102846	NaN	-0,74321	-2,11635	NaN	NaN	NaN
NaN	NaN	NaN	0,102846	NaN	-0,74321	-2,11635	NaN	NaN	NaN
-0,504698	0,025156	-0,634556	-0,0883293	-0,778286	-0,003479	-0,759556	-1,87107	-0,12732	NaN
-0,504698	0,025156	-0,634556	-0,0883293	-0,778286	-0,003479	-0,759556	-1,87107	-0,12732	-1,97095
-0,504698	0,025156	-0,634556	-0,0883293	-0,778286	-0,003479	-0,759556	-1,87107	-0,12732	-1,97095
NaN	NaN	NaN	1,26013	0,0905552	0,534189	NaN	NaN	NaN	NaN
NaN	NaN	NaN	1,26013	0,0905552	0,534189	NaN	NaN	NaN	NaN

Polito et al. Universal ready-to-use immunotherapeutic approach for the treatment of cancer: expanded and activated polyclonal γδ memory T cells.
Phosphorylation sites

NaN	NaN	NaN	NaN	NaN	NaN	NaN	NaN	NaN	NaN
NaN	NaN	NaN	NaN	NaN	NaN	NaN	NaN	NaN	NaN
NaN	NaN	NaN	-1,23772	NaN	-2,24868	-0,805334	NaN	NaN	NaN
NaN	NaN	NaN	-1,23772	NaN	-2,24868	-0,805334	NaN	NaN	NaN
NaN	NaN	NaN	NaN	NaN	-0,0903721	NaN	NaN	NaN	NaN
1,05698	0,272211	0,342791	1,05221	1,16032	1,08978	1,32644	NaN	NaN	NaN
1,05698	0,272211	0,342791	1,05221	1,16032	1,08978	1,32644	0,873764	0,927989	0,23276
1,05698	0,272211	0,342791	1,05221	1,16032	1,08978	1,32644	0,873764	0,927989	0,23276
NaN	NaN	NaN	-1,66828	NaN	-1,12717	-0,51013	NaN	NaN	NaN
NaN	NaN	NaN	-1,66828	NaN	-1,12717	-0,51013	NaN	NaN	NaN
NaN	NaN	NaN	-1,66828	NaN	-1,12717	-0,51013	NaN	NaN	NaN
NaN	NaN	NaN	NaN	NaN	NaN	NaN	NaN	NaN	NaN
NaN	NaN	NaN	NaN	NaN	NaN	NaN	NaN	NaN	NaN
NaN	NaN	NaN	NaN	NaN	NaN	NaN	NaN	NaN	NaN
-1,16692	NaN	-1,22982	1,01558	-1,24919	-1,56472	-0,602419	NaN	NaN	NaN
-1,16692	NaN	-1,22982	1,01558	-1,24919	-1,56472	-0,602419	NaN	NaN	NaN
-1,16692	NaN	-1,22982	1,01558	-1,24919	-1,56472	-0,602419	NaN	NaN	NaN
0,196472	0,695642	0,293507	-0,678949	1,35584	0,179705	-0,745663	0,178059	1,02913	0,467469
0,196472	0,695642	0,293507	-0,678949	1,35584	0,179705	-0,745663	0,178059	1,02913	0,467469
0,196472	0,695642	0,293507	-0,678949	1,35584	0,179705	-0,745663	0,178059	1,02913	0,467469
NaN	NaN	NaN	-0,68825	-1,80412	1,00967	-1,01948	NaN	NaN	NaN
NaN	NaN	NaN	-0,68825	-1,80412	1,00967	-1,01948	NaN	NaN	NaN
NaN	NaN	NaN	-0,68825	-1,80412	1,00967	-1,01948	NaN	NaN	NaN
-1,31527	-2,24273	-1,79992	-1,36641	-1,43695	-1,51124	-1,84164	NaN	NaN	NaN
-1,31527	-2,24273	-1,79992	-1,36641	-1,43695	-1,51124	-1,84164	NaN	NaN	NaN
-1,31527	-2,24273	-1,79992	-1,36641	-1,43695	-1,51124	-1,84164	NaN	NaN	NaN
NaN	NaN	NaN	0,500498	NaN	1,17154	0,117975	NaN	NaN	NaN
NaN	NaN	NaN	0,500498	NaN	1,17154	0,117975	NaN	NaN	NaN
NaN	NaN	NaN	0,500498	NaN	1,17154	0,117975	NaN	NaN	NaN
1,50183	0,801863	0,549044	3,81E-05	0,76462	-0,551155	-0,340324	0,0857468	NaN	0,626817
1,50183	0,801863	0,549044	3,81E-05	0,76462	-0,551155	-0,340324	0,0857468	-0,290685	0,626817
NaN	NaN	NaN	NaN	NaN	NaN	NaN	0,0857468	-0,290685	0,626817
NaN	NaN	-0,393026	-1,25245	NaN	-1,60611	-1,29066	NaN	NaN	NaN
NaN	NaN	NaN	-0,705713	NaN	0,496292	-1,3066	NaN	NaN	NaN

NaN	NaN	NaN	-0,705713	NaN	0,496292	-1,3066	NaN	NaN	NaN
NaN	NaN	NaN	-0,705713	NaN	0,496292	-1,3066	NaN	NaN	NaN
1,20102	0,666592	0,428036	NaN	1,22937	NaN	1,69608	NaN	0,735591	0,87305
NaN	NaN	NaN	NaN	NaN	NaN	NaN	NaN	NaN	NaN
NaN	1,06735	NaN	0,722612	1,47201	4,27275	1,81364	NaN	NaN	NaN
NaN	1,06735	NaN	0,722612	1,47201	4,27275	1,81364	NaN	NaN	NaN
-1,37093	-1,28053	-0,744219	-0,846397	-1,61301	0,572245	-0,448023	NaN	NaN	NaN
-1,37093	-1,28053	-0,744219	-0,846397	-1,61301	0,572245	-0,448023	NaN	NaN	NaN
-1,37093	-1,28053	-0,744219	-0,846397	-1,61301	0,572245	-0,448023	NaN	NaN	NaN
0,538494	0,379929	-0,306704	-1,36243	0,236631	-1,99887	NaN	-1,15792	-0,506845	0,143664
0,538494	0,379929	-0,306704	-1,36243	0,236631	-1,99887	NaN	-1,15792	-0,506845	0,143664
0,420612	NaN	NaN	NaN	0,174173	-1,87099	-0,225222	NaN	-0,865212	0,0293608
0,538494	0,379929	-0,306704	-1,36243	0,236631	-1,99887	NaN	-1,15792	-0,506845	0,143664
1,0573	2,14337	-0,710217	0,925636	1,05232	-0,935528	1,12498	1,10471	0,834823	0,468366
1,0573	2,14337	-0,710217	0,925636	1,05232	-0,935528	1,12498	1,10471	0,834823	0,468366
1,0573	2,14337	-0,710217	0,925636	1,05232	-0,935528	1,12498	1,10471	0,834823	0,468366
NaN	1,28806	NaN	NaN	-2,57521	1,13391	NaN	NaN	NaN	NaN
NaN	1,28806	NaN	NaN	-2,57521	1,13391	NaN	NaN	NaN	NaN
NaN	1,28806	NaN	NaN	-2,57521	1,13391	NaN	NaN	NaN	NaN
-1,37985	-1,01275	NaN	-1,10717	-2,78577	-1,73206	0,730156	NaN	NaN	NaN
-1,37985	-1,01275	NaN	-0,411543	-2,78577	-1,73206	1,29709	NaN	NaN	NaN
-1,37985	-1,01275	NaN	-0,411543	-2,78577	-1,73206	1,29709	NaN	NaN	NaN
2,23129	2,01943	2,29158	2,23286	2,30675	-0,181747	1,42652	2,56295	2,3688	2,66915
NaN	-0,92812	0,307207	-0,566523	NaN	NaN	-0,454287	0,252558	-0,249606	-0,334472
0,790638	0,923397	0,12307	-0,179691	-0,111904	-0,181747	NaN	1,33085	0,287421	-0,445781
2,23129	1,81914	1,21808	2,00911	2,30675	NaN	0,969444	2,2381	2,11236	2,47702
0,406839	0,485064	-0,703815	1,48941	0,435497	1,92621	2,08788	-2,08076	0,431153	0,243829
0,406839	0,485064	-0,703815	1,48941	0,435497	1,92621	2,08788	-2,08076	0,431153	0,243829
NaN	-0,768951	-1,61898	NaN	NaN	NaN	NaN	-1,91188	-0,479594	-0,49322
-0,741982	1,26127	NaN	NaN	-0,773661	0,292902	NaN	-0,972454	0,503579	-0,435069
0,887928	2,25495	0,282709	1,08264	-0,746361	NaN	2,0276	0,0366936	1,78141	0,460832
0,887928	2,25495	0,282709	1,08264	-0,746361	NaN	2,0276	0,0366936	1,78141	0,460832
NaN	NaN	NaN	-1,42528	0,36446	-1,7368	0,884596	NaN	NaN	NaN
NaN	NaN	NaN	-1,42528	0,36446	-1,7368	0,884596	NaN	NaN	NaN

Polito et al. Universal ready-to-use immunotherapeutic approach for the treatment of cancer: expanded and activated polyclonal γδ memory T cells.
Phosphorylation sites

NaN	NaN	NaN	-1,42528	0,36446	-1,7368	0,884596	NaN	NaN	NaN
-0,52482	1,00554	NaN	0,16519	-1,85402	1,01544	0,834597	-1,95601	0,0190687	NaN
-0,52482	1,00554	NaN	0,16519	-1,85402	1,01544	0,834597	-1,95601	0,0190687	NaN
NaN	NaN	NaN	-1,62863	-2,73094	-1,12038	-1,42456	NaN	NaN	NaN
NaN	NaN	NaN	-1,62863	-2,73094	-1,12038	-1,42456	NaN	NaN	NaN
NaN	NaN	NaN	-1,61736	-1,66987	-1,7853	NaN	-0,541828	-1,18209	NaN
NaN	NaN	NaN	-1,61736	-1,66987	-1,7853	NaN	-0,541828	-1,18209	NaN
1,67643	3,60558	2,21972	2,65672	NaN	NaN	3,86181	2,04733	3,34403	3,0886
1,67643	3,60558	2,21972	2,65672	NaN	NaN	3,86181	2,04733	3,34403	3,0886
-0,506853	-0,16147	-1,35355	-0,767862	-0,320288	-1,59811	-0,405874	-1,33033	-1,56052	-0,956229
-0,506853	-0,16147	-1,35355	-0,767862	-0,320288	-1,59811	-0,405874	-1,33033	-1,56052	-0,956229
NaN	NaN	NaN	NaN	NaN	-1,59811	-0,405874	-1,33033	-1,56052	-0,956229
-0,506853	-0,16147	-1,35355	-0,767862	-0,320288	NaN	NaN	NaN	NaN	NaN
1,67643	3,60558	2,21972	2,65672	NaN	NaN	3,86181	2,04733	3,34403	3,0886
0,471193	1,85924	-1,12657	NaN	NaN	NaN	NaN	NaN	NaN	NaN
0,471193	1,85924	-1,12657	NaN	NaN	NaN	NaN	NaN	NaN	NaN
NaN	NaN	NaN	NaN	NaN	NaN	NaN	NaN	NaN	NaN
NaN	NaN	NaN	-1,7923	-2,00459	-1,88676	-0,742182	NaN	NaN	NaN
NaN	NaN	NaN	-1,7923	-2,00459	-1,88676	-0,742182	NaN	NaN	NaN
NaN	NaN	NaN	-1,7923	-2,00459	-1,88676	-0,742182	NaN	NaN	NaN
NaN	NaN	NaN	NaN	NaN	NaN	NaN	-1,57114	-1,09259	-0,934663
NaN	NaN	NaN	NaN	NaN	NaN	NaN	-1,57114	-1,09259	-0,934663
1,38405	2,49109	1,0484	3,45499	0,383722	2,76002	4,29593	0,497431	2,47217	2,16448
1,38405	2,49109	1,0484	3,45499	0,383722	2,76002	4,29593	0,497431	2,47217	2,16448
1,38405	2,49109	1,0484	3,45499	0,383722	2,76002	4,29593	0,497431	2,47217	2,16448
1,14883	NaN	NaN	1,03311	0,232475	NaN	-0,298965	NaN	-0,462113	-2,28837
1,07606	0,150335	0,496044	-2,76224	0,405653	NaN	-1,62495	0,800043	-0,0596895	-0,149453
NaN	NaN	NaN	-1,85037	NaN	-0,824045	-1,36337	NaN	NaN	NaN
NaN	NaN	NaN	-0,235991	NaN	0,249813	-1,04448	NaN	NaN	NaN
NaN	NaN	NaN	-0,735182	NaN	-0,0804539	-0,687101	NaN	NaN	NaN
NaN	NaN	NaN	NaN	NaN	NaN	NaN	1,85348	1,58547	4,5754
NaN	NaN	NaN	NaN	NaN	NaN	NaN	1,85348	1,58547	4,5754
NaN	NaN	1,69328	1,51524	2,01538	NaN	2,00111	NaN	2,44279	2,32254
NaN	-1,31291	-1,61735	0,469236	-0,228619	0,815941	NaN	NaN	NaN	NaN

Polito et al. Universal ready-to-use immunotherapeutic approach for the treatment of cancer: expanded and activated polyclonal γδ memory T cells.
Phosphorylation sites

NaN	NaN	NaN	-0,686769	-1,08036	-0,40457	-0,475849	NaN	NaN	NaN
NaN	-3,40525	NaN	-1,62897	NaN	-0,458761	-1,71511	NaN	NaN	NaN
NaN	NaN	NaN	0,295382	-0,860975	-0,0594482	NaN	NaN	NaN	NaN
1,01982	NaN	0,172413	1,06518	-2,02898	2,08778	NaN	0,101456	NaN	-0,619674
0,526831	0,0687046	0,342474	-1,74439	0,932585	-1,18943	-1,28744	NaN	NaN	NaN
7,79911	7,70868	7,08666	5,69651	5,66718	4,7018	5,78793	7,45176	8,03777	7,68334
-1,66937	NaN	NaN	-2,68054	-3,05821	-3,04852	-1,21728	NaN	-0,448083	-1,41385
1,26242	0,00357819	0,0749226	NaN	NaN	0,185724	2,18184	NaN	NaN	1,12352
1,26242	0,00357819	0,0749226	NaN	NaN	0,185724	2,18184	NaN	NaN	1,12352
1,70389	1,41488	1,50134	NaN	1,751	1,54991	NaN	NaN	-0,114942	1,05978
0,528595	-2,61813	-0,652603	0,99967	0,628822	0,935236	0,931398	0,413834	-0,307313	1,09898
0,361433	1,95697	2,28819	0,790182	3,3602	-0,284592	2,70255	1,32847	1,05256	1,87905
NaN	NaN	NaN	NaN	NaN	NaN	NaN	1,68359	1,42403	1,0691
-1,53174	NaN	NaN	-0,0650272	-2,24312	1,28633	-0,762661	NaN	NaN	NaN
NaN	NaN	NaN	NaN	NaN	NaN	NaN	NaN	NaN	NaN
NaN	NaN	NaN	-0,977909	NaN	0,104012	-0,558018	NaN	NaN	NaN
NaN	NaN	NaN	-0,0384865	-1,98138	-0,551453	0,142578	NaN	NaN	NaN
NaN	NaN	NaN	-0,0384865	-1,98138	-0,551453	0,142578	NaN	NaN	NaN
-0,453308	-1,14493	NaN	NaN	0,322649	1,42364	0,417126	NaN	0,568496	NaN
NaN	NaN	NaN	-2,84858	-1,26059	NaN	-1,18496	NaN	NaN	NaN
0,862619	0,575548	NaN	0,943428	-0,913939	1,81093	1,022	NaN	0,549563	NaN
-0,237452	2,70317	0,299116	-1,40318	1,23071	-0,817736	-1,36907	0,231659	0,274693	-0,155499
2,84371	2,40858	1,25176	2,27646	1,03369	3,15788	2,06237	1,85182	1,35138	2,24274
NaN	NaN	NaN	-1,81909	-0,74003	-1,81228	NaN	NaN	NaN	NaN
NaN	NaN	NaN	1,63838	NaN	0,659498	1,33461	NaN	NaN	NaN
NaN	NaN	NaN	0,829193	-1,03078	0,800957	0,413809	NaN	NaN	NaN
NaN	NaN	NaN	1,26013	0,0905552	0,534189	NaN	NaN	NaN	NaN
-1,42373	1,04329	-1,24889	-0,447138	0,778791	0,0397129	0,708029	-1,52117	-0,459207	NaN
NaN	NaN	-0,393026	-1,25245	NaN	-1,60611	-1,29066	NaN	NaN	NaN
1,20102	0,666592	0,428036	NaN	1,22937	NaN	1,69608	0,715	0,735591	0,87305
NaN	1,06735	NaN	0,722612	1,47201	4,27275	1,81364	NaN	NaN	NaN
1,56844	1,10948	1,36151	1,6519	NaN	NaN	NaN	NaN	NaN	2,27297
0,406839	0,485064	-0,703815	1,48941	0,435497	1,92621	2,08788	-2,08076	0,431153	0,243829
0,887928	2,25495	0,282709	1,08264	-0,746361	NaN	2,0276	0,0366936	1,78141	0,460832

Polito et al. Universal ready-to-use immunotherapeutic approach for the treatment of cancer: expanded and activated polyclonal γδ memory T cells.
Phosphorylation sites

NaN	NaN	NaN	NaN	NaN	NaN	NaN	NaN	NaN	NaN
0,684647	1,26127	0,423286	0,0159245	-0,773661	0,292902	NaN	0,289188	1,5925	0,727214
NaN	NaN	NaN	NaN	NaN	NaN	NaN	-1,57114	-1,09259	-0,934663
NaN	NaN	NaN	0,725506	NaN	1,51045	1,05281	NaN	NaN	NaN
NaN	NaN	NaN	0,295382	-0,860975	-0,0594482	NaN	NaN	NaN	NaN
NaN	NaN	NaN	NaN	NaN	NaN	NaN	-1,54958	-1,68985	-1,81586

Polito et al. Universal ready-to-use immunotherapeutic approach for the treatment of cancer: expanded and activated polyclonal γδ memory T cells.
Phosphorylation sites

GD1_Dp18	GD2_Dp18	GD3_Dp18	GD4_Dp18	C: Amino acid	C: Charge	C: Reverse	C: Potential contaminant	C: Diagnostic peak	C: Multiplicity
-0,883081	0,0398064	0,997625	2,0513	S	2				___1
-2,11741	NaN	-1,78806	-0,190226	S	2				___1
0,152548	-0,155127	NaN	0,685595	S	2				___1
NaN	-3,32273	0,351311	2,06486	S	3				___1
-1,74005	-1,6626	NaN	-0,830212	S	2				___1
1,13894	0,561749	2,1708	0,434645	S	2				___1
NaN	1,66098	-0,899002	2,39976	S	3				___1
1,69374	2,90032	-0,17445	2,98032	S	3				___1
1,97046	1,87725	NaN	NaN	S	2				___1
NaN	0,696232	-0,24408	-0,953442	S	3				___1
NaN	NaN	-0,7714	NaN	S	2				___1
NaN	-2,42456	0,302382	-0,422993	S	2				___1
NaN	NaN	NaN	NaN	S	2				___1
NaN	-0,7444	NaN	-1,60801	S	2				___1
NaN	NaN	NaN	NaN	S	2				___1
-0,576069	-0,765505	NaN	0,219284	S	2				___1
NaN	-0,520123	0,114412	0,17915	S	2				___1
NaN	NaN	NaN	NaN	S	2				___1
NaN	NaN	NaN	NaN	S	2				___1
NaN	-0,7444	1,21903	-1,4524	S	3				___1
NaN	NaN	-1,85023	NaN	S	3				___1
1,38102	-0,713125	NaN	-1,16837	S	2				___1
-1,48156	NaN	-2,87819	0,138317	S	2				___1
-1,65358	-2,64571	-2,13627	NaN	S	2				___1
0,967979	0,235462	1,5792	-0,46703	S	2				___1
-0,238554	-1,66169	NaN	-1,99831	S	2				___1
-0,706646	0,935797	1,39395	0,540655	S	3				___1
NaN	NaN	NaN	NaN	S	2				___1
NaN	-3,18518	NaN	-0,812412	S	3				___1
NaN	NaN	NaN	NaN	S	3				___1
NaN	NaN	NaN	-0,175388	S	2				___1
NaN	NaN	NaN	NaN	S	2				___1
-0,250004	0,729618	NaN	1,73088	S	3				___1

-0,893148	NaN	NaN	NaN	S	2	___1
NaN	NaN	NaN	NaN	S	2	___1
NaN	-0,384033	NaN	-1,38185	S	3	___1
-0,241253	NaN	-0,33777	2,07823	S	3	___1
NaN	NaN	NaN	NaN	S	3	___1
3,05355	2,84728	3,539	4,03127	S	3	___1
1,55052	2,96076	-0,436838	3,64648	S	2	___1
3,4509	4,95879	4,73007	4,39171	S	2	___1
NaN	-1,0819	-0,708702	-0,412853	S	2	___1
-2,11534	-2,45694	NaN	-2,58365	S	2	___1
NaN	NaN	NaN	NaN	S	3	___1
NaN	-2,34043	-1,55206	NaN	S	2	___1
-1,25017	-0,309908	1,47631	-0,415257	S	3	___1
0,907738	2,12183	2,90747	2,0004	S	3	___1
NaN	NaN	NaN	NaN	S	2	___1
-1,44356	-1,05397	NaN	-1,98332	S	2	___1
-0,659367	-0,968208	-2,20844	NaN	S	2	___1
-1,0701	-0,0898666	NaN	-1,44142	S	3	___1
-0,534975	1,5427	NaN	0,38052	S	4	___1
NaN	NaN	NaN	NaN	S	2	___1
0,185398	0,317549	0,690107	-0,580601	S	2	___1
-2,73345	-0,73933	-0,49338	NaN	S	3	___1
-2,39318	-1,84676	-1,2688	-1,66377	S	2	___1
-0,538696	-1,05454	0,232904	-2,0865	S	2	___1
1,21989	0,846138	-0,297855	0,909292	S	3	___1
1,40307	-0,233335	1,8889	0,432251	S	2	___1
1,03304	2,80582	2,45609	2,22374	S	3	___1
1,89868	1,31961	2,26991	0,765953	S	2	___1
-0,673002	1,59103	-0,946159	0,139763	S	3	___1
2,70728	-4,89267	2,55077	NaN	S	2	___1
3,81369	4,2264	5,08008	4,11868	S	2	___1
0,307617	1,24895	-1,1018	1,4977	S	2	___1
NaN	NaN	NaN	NaN	S	2	___1
-0,250664	-1,05064	1,00518	-0,458185	S	2	___1

NaN	-0,273998	-1,17348	0,627281	S	4	___1
NaN	NaN	NaN	NaN	S	3	___1
NaN	NaN	NaN	NaN	S	3	___1
NaN	NaN	NaN	NaN	S	3	___1
NaN	NaN	NaN	NaN	S	2	___1
NaN	-1,1211	NaN	NaN	S	3	___1
-0,898952	0,349724	1,2288	0,367687	S	3	___1
NaN	1,0619	1,60934	2,95888	S	2	___1
NaN	NaN	NaN	NaN	S	3	___1
-0,761625	NaN	-3,02562	-0,685343	S	2	___1
0,45376	NaN	0,525352	-0,0292473	S	2	___1
1,60313	3,08743	3,92385	2,35961	S	2	___1
2,93483	2,66677	-1,78101	2,41925	S	2	___1
1,31502	1,47176	0,361637	0,91502	S	2	___1
-0,0282001	-0,993475	-0,315414	NaN	S	2	___1
-4,80262	2,41195	1,31617	2,39861	S	3	___1
1,9574	1,56754	1,78589	-0,328074	S	2	___1
-0,150467	0,556707	NaN	NaN	S	2	___1
NaN	NaN	NaN	NaN	S	2	___1
-1,51741	-0,433126	-0,196177	NaN	S	3	___1
NaN	NaN	NaN	NaN	S	2	___1
0,550022	0,974499	1,82215	-0,645113	S	2	___1
-0,543636	-0,3055	-1,65216	-1,33766	S	2	___1
NaN	NaN	NaN	NaN	S	3	___1
-0,694765	-1,26081	-2,88622	NaN	S	2	___1
-1,3258	-1,28663	-1,20179	NaN	S	2	___1
NaN	NaN	NaN	NaN	S	2	___1
NaN	NaN	NaN	NaN	S	2	___1
NaN	0,00351906	NaN	-0,461246	S	3	___1
-0,0588379	-1,07212	-1,35881	NaN	S	3	___1
NaN	-0,422421	-1,47615	0,0899906	S	3	___1
-1,32172	0,523926	NaN	-0,421329	S	2	___1
NaN	NaN	NaN	NaN	S	2	___1
NaN	NaN	NaN	NaN	S	2	___1

2,62722	4,31284	4,42245	2,73462	S	3	___1
4,68447	4,46195	4,40542	2,67513	S	2	___1
1,98375	3,03282	0,830349	1,42192	S	3	___1
0,0413265	0,986101	-0,707865	NaN	S	3	___1
NaN	NaN	NaN	NaN	S	3	___1
1,17891	-0,18239	NaN	NaN	S	2	___1
2,75246	2,75159	NaN	2,12237	S	2	___1
2,46274	3,21673	0,514967	0,246708	S	2	___1
-0,66194	-0,191969	-0,223036	-1,90115	S	2	___1
1,14622	1,22593	NaN	NaN	S	2	___1
NaN	-0,636189	2,86616	1,54439	S	2	___1
NaN	3,66432	NaN	NaN	S	2	___1
-1,32094	0,143284	-1,21545	-0,491159	S	3	___1
-2,22403	-0,519579	-1,15688	-0,941425	S	2	___1
NaN	-2,20986	-1,92488	NaN	S	2	___1
-2,36604	-0,865805	-0,336977	-1,49918	S	3	___1
0,834805	0,188745	0,191071	-0,81126	S	3	___1
NaN	NaN	NaN	NaN	S	2	___1
NaN	NaN	NaN	NaN	S	2	___1
-0,612049	-1,65187	-0,416815	-0,91853	S	3	___1
NaN	NaN	NaN	NaN	S	2	___1
0,817118	0,160881	0,770893	0,268244	S	2	___1
NaN	NaN	NaN	0,72374	S	4	___1
NaN	NaN	-1,43014	NaN	S	2	___1
-0,994432	-1,64363	-0,0587368	-1,05253	S	2	___1
NaN	-2,29732	-0,320084	NaN	S	2	___1
NaN	-1,59146	-0,70973	-0,325811	S	3	___1
1,7251	1,15589	1,0112	-0,314085	S	2	___1
NaN	NaN	NaN	NaN	S	2	___1
NaN	NaN	1,83505	NaN	S	3	___1
-0,984116	-1,69493	NaN	-1,60694	S	2	___1
0,799946	1,64022	1,74001	NaN	S	2	___1
1,42891	1,1929	2,14656	0,685406	S	2	___1
-1,2952	-0,952841	NaN	-2,32364	S	2	___1

0,605158	0,464251	-0,431242	NaN	S	2	___1
NaN	NaN	NaN	NaN	S	2	___1
NaN	NaN	NaN	NaN	S	4	___1
NaN	NaN	NaN	NaN	S	2	___1
1,4645	-0,747068	NaN	-1,37782	S	2	___1
NaN	-0,131189	0,124098	-1,09398	S	2	___1
NaN	NaN	NaN	NaN	S	2	___1
-1,45928	-2,32288	NaN	-2,50004	S	2	___1
0,713802	0,697214	NaN	0,289637	S	2	___1
-0,997749	0,637613	-0,13755	-0,275608	S	3	___1
-0,764683	0,360922	-0,351038	-0,776217	S	2	___1
-0,74905	-1,59297	-1,14073	-0,509182	S	2	___1
NaN	NaN	NaN	NaN	S	3	___1
1,29638	2,23406	0,683252	2,15214	S	3	___1
-0,144711	-0,525785	NaN	1,46243	S	2	___1
-1,74946	-0,199869	NaN	-0,78257	S	2	___1
0,649281	0,213926	-1,25434	0,619099	S	2	___1
2,28675	1,86078	1,6117	1,45271	S	3	___1
2,47725	2,04987	1,71272	1,40158	S	2	___1
NaN	NaN	NaN	NaN	S	2	___1
2,00747	1,20396	1,43042	1,12839	S	4	___1
NaN	NaN	NaN	NaN	S	2	___1
NaN	NaN	NaN	NaN	S	2	___1
NaN	NaN	NaN	NaN	S	2	___1
-2,32427	-1,37679	NaN	NaN	S	2	___1
-0,844557	NaN	NaN	1,09796	S	2	___1
NaN	NaN	NaN	NaN	S	3	___1
-0,448511	-1,14809	-1,21313	1,12613	S	2	___1
-0,60013	NaN	0,573412	NaN	S	2	___1
NaN	NaN	NaN	NaN	S	2	___1
NaN	-1,00805	1,05083	-2,71226	S	2	___1
NaN	NaN	NaN	NaN	S	3	___1
NaN	NaN	NaN	NaN	S	3	___1
-0,187275	0,258581	NaN	0,577635	S	2	___1

NaN	NaN	NaN	NaN	S	2	___1
0,811481	0,593891	1,47706	NaN	S	3	___1
-3,16028	-2,47297	0,539658	NaN	S	2	___1
-1,34354	-1,6304	1,02476	NaN	S	3	___1
-1,5195	-2,34643	1,46758	NaN	S	2	___1
-1,46741	-2,53391	0,492678	NaN	S	2	___1
0,513134	-0,434458	2,71034	-1,58309	S	2	___1
NaN	NaN	NaN	NaN	S	4	___1
NaN	NaN	NaN	NaN	S	2	___1
NaN	NaN	NaN	NaN	S	2	___1
0,489786	-0,659225	NaN	-1,67862	S	2	___1
-1,48951	-1,76249	0,340195	-1,57093	S	2	___1
-1,45595	-1,07395	0,336163	-0,513758	S	2	___1
0,824884	NaN	NaN	NaN	S	2	___1
NaN	0,207706	NaN	NaN	S	3	___1
NaN	NaN	NaN	NaN	S	3	___1
3,82361	3,6283	NaN	2,05463	S	2	___1
NaN	NaN	NaN	NaN	S	2	___1
NaN	NaN	NaN	NaN	S	2	___1
-0,722738	-1,92801	-0,0280609	NaN	S	2	___1
NaN	-0,513832	0,677473	-0,993362	S	2	___1
-1,269	1,0323	NaN	0,210686	S	2	___1
-1,07149	-2,32664	NaN	-1,53444	S	2	___1
NaN	-0,223303	NaN	0,197672	S	3	___1
-0,14847	-0,697554	-1,34284	-0,818432	S	2	___1
NaN	NaN	NaN	NaN	S	2	___1
-1,83389	-2,47242	NaN	-0,924608	S	2	___1
NaN	NaN	NaN	NaN	S	2	___1
NaN	NaN	-0,491383	NaN	S	3	___1
NaN	NaN	NaN	2,14108	S	2	___1
NaN	NaN	NaN	NaN	S	2	___1
-2,68777	-1,39667	NaN	-1,53532	S	2	___1
NaN	-0,689096	1,48971	1,27149	S	3	___1
NaN	-1,06911	0,117247	-0,587765	S	3	___1

-0,789022	1,42276	1,90384	1,27265	S	2	___1
-0,780128	-0,932285	NaN	-1,12593	S	2	___1
-0,898048	-0,246223	NaN	-0,803349	S	2	___1
NaN	NaN	NaN	NaN	S	3	___1
2,87691	1,31237	3,5636	-0,577276	S	3	___1
0,0962582	0,937025	NaN	0,0879421	S	3	___1
4,65809	6,7959	7,89502	6,34653	S	2	___1
NaN	-1,23503	-1,47667	-1,66778	S	2	___1
0,280231	0,0282574	NaN	-0,859663	S	2	___1
0,158581	2,31857	NaN	-0,0984097	S	3	___1
NaN	NaN	NaN	NaN	S	2	___1
1,07194	0,812742	1,70746	0,827703	S	3	___1
0,251823	-2,18401	NaN	NaN	S	2	___1
1,84761	1,87855	1,95654	0,548204	S	2	___1
NaN	-0,844418	-0,377401	-1,82921	S	2	___1
-1,2809	-2,24849	-1,4623	-0,998922	S	2	___1
-0,388681	-0,99692	-1,52562	-0,199583	S	2	___1
-1,43229	NaN	NaN	-1,97299	S	2	___1
-2,54651	-2,34239	NaN	-1,61008	S	2	___1
NaN	NaN	NaN	NaN	S	2	___1
0,00487709	-0,432104	NaN	0,331657	S	3	___1
NaN	-1,97963	-1,33801	0,917006	S	3	___1
-0,531216	-0,826832	0,00627136	-0,723976	S	2	___1
NaN	-1,13588	-0,549875	-1,44301	S	2	___1
-1,27042	-0,628082	-1,12841	-0,964279	S	2	___1
NaN	NaN	0,218834	-1,89486	S	2	___1
1,90295	1,29159	NaN	3,45892	S	2	___1
1,28362	1,2514	1,79718	2,84935	S	2	___1
-0,811405	-0,390179	-0,164869	0,132645	S	2	___1
NaN	NaN	NaN	NaN	S	2	___1
NaN	-1,64015	-1,30548	NaN	S	2	___1
NaN	NaN	NaN	NaN	S	2	___1
NaN	NaN	NaN	NaN	S	2	___1
0,169498	-0,688242	NaN	-1,13105	S	2	___1

-0,25087	NaN	1,92324	0,567554	S	3	___1
-0,25087	NaN	1,92324	0,567554	S	3	___1
3,97696	2,69279	3,08074	2,55016	S	2	___1
0,759748	NaN	2,69288	1,1963	S	3	___1
-0,954741	-0,460295	NaN	NaN	S	2	___1
-0,180584	2,00479	2,41466	2,09494	S	2	___1
-2,0734	-2,04634	NaN	-1,84133	S	2	___1
-1,01964	-0,513832	-2,15046	-2,01167	S	2	___1
NaN	NaN	-2,11212	NaN	S	2	___1
0,499622	-0,19813	1,7421	-0,282942	S	2	___1
-0,0312424	-0,429344	0,00731277	-0,18082	S	2	___1
NaN	-3,10872	-0,672752	-2,76676	S	2	___1
-0,678148	-1,24809	-0,311192	NaN	S	2	___1
-0,64234	-1,71566	NaN	0,598051	S	2	___1
NaN	NaN	NaN	NaN	S	3	___1
0,131067	-0,518602	-2,21264	1,3703	S	2	___1
NaN	NaN	NaN	NaN	S	2	___1
0,381077	1,04789	3,51491	1,06844	S	3	___1
-0,641582	-1,97357	NaN	-2,9943	S	2	___1
0,100918	-0,0449963	3,1742	NaN	S	2	___1
-0,444403	-0,794527	0,416376	-1,27402	S	2	___1
-1,7228	-2,82729	NaN	-1,99644	S	2	___1
-0,574364	-1,42348	-1,73938	-0,185474	S	2	___1
-1,41122	-2,13721	-1,71251	-0,435167	S	2	___1
1,31	1,89985	3,98508	-0,681694	S	3	___1
NaN	NaN	NaN	NaN	S	3	___1
-2,29745	-1,95145	-1,00273	NaN	S	3	___1
NaN	NaN	NaN	NaN	S	3	___1
-1,50349	0,897493	1,77444	-0,43618	S	2	___1
0,722795	0,313833	0,38722	NaN	S	3	___1
NaN	NaN	NaN	NaN	S	2	___1
NaN	0,198092	-2,98994	-1,10059	S	2	___1
-1,42121	-1,01237	0,322771	NaN	S	3	___1
NaN	NaN	NaN	NaN	S	2	___1

NaN	NaN	0,274483	NaN	S	2	___1
-0,213028	-0,281073	0,873926	0,535801	S	2	___1
NaN	-1,30526	-1,52568	-1,03016	S	3	___1
NaN	NaN	NaN	NaN	S	2	___1
1,0809	0,551338	0,158661	0,186148	S	2	___1
-1,46033	-1,24808	-2,62004	-0,978516	S	2	___1
-2,5515	-3,48577	-3,10722	-0,12826	S	2	___1
2,41546	3,3936	-0,615969	4,26816	S	3	___1
-2,59978	NaN	-0,63518	NaN	S	2	___1
0,734921	0,463921	-0,689619	1,53875	S	2	___1
NaN	0,899202	1,05622	-0,675507	S	2	___1
NaN	NaN	NaN	NaN	S	2	___1
NaN	NaN	NaN	NaN	S	2	___1
2,59621	2,96416	3,9136	6,11909	S	2	___1
NaN	NaN	NaN	NaN	S	2	___1
0,0461998	-0,713249	NaN	0,663094	S	2	___1
NaN	NaN	NaN	NaN	S	2	___1
-0,296864	-0,499081	-1,69312	-0,490171	S	2	___1
-1,90491	-0,581858	2,56869	0,646399	S	2	___1
NaN	-0,732765	-0,621687	0,925047	S	3	___1
NaN	NaN	NaN	NaN	S	2	___1
NaN	NaN	NaN	NaN	S	3	___1
NaN	NaN	0,765701	-0,749702	S	2	___1
-1,25802	-2,02626	-3,90859	-1,65733	S	2	___1
0,107462	-0,0739822	NaN	-0,0460072	S	2	___1
1,60541	1,80682	1,11939	-0,447252	S	2	___1
-1,52467	-0,420593	-1,64428	-0,0275764	S	2	___1
-1,06357	0,32634	1,42418	-0,653086	S	3	___1
0,967306	5,1826	4,53585	2,14569	S	4	___1
NaN	NaN	1,25048	NaN	S	3	___1
-0,770699	-1,17774	NaN	NaN	S	2	___1
-0,337482	-0,0808601	0,433538	-0,736139	S	2	___1
-1,49146	NaN	0,115164	NaN	S	2	___1
-0,618259	-0,448248	-0,967028	0,0281372	S	2	___1

Polito et al. Universal ready-to-use immunotherapeutic approach for the treatment of cancer: expanded and activated polyclonal γδ memory T cells.
Phosphorylation sites

NaN	NaN	NaN	NaN	S	2	___1
NaN	NaN	NaN	NaN	S	2	___1
-0,452772	-0,508324	-2,07458	NaN	S	2	___1
-1,16457	-0,841833	-1,15943	-1,58907	S	3	___1
NaN	NaN	NaN	NaN	S	3	___1
4,58083	3,84891	4,74923	3,01261	S	2	___1
NaN	0,898876	NaN	-0,149668	S	2	___1
NaN	-0,551548	1,42036	-1,31761	S	3	___1
2,58673	2,13563	1,01846	1,79446	S	2	___1
0,538021	1,60038	0,33452	0,532646	S	3	___1
NaN	0,477375	0,217693	0,684153	S	3	___1
4,16092	4,27975	3,31336	3,14506	S	3	___1
NaN	0,505892	0,40394	0,109098	S	3	___1
NaN	NaN	NaN	NaN	S	2	___1
NaN	NaN	NaN	NaN	S	2	___1
NaN	NaN	NaN	-0,036581	S	2	___1
-2,41596	-2,55175	NaN	-1,60658	S	2	___1
1,29609	0,693377	NaN	0,29067	S	2	___1
0,737789	0,718513	NaN	0,294399	S	2	___1
1,84346	2,33799	1,87342	2,22665	S	4	___1
NaN	NaN	NaN	NaN	S	2	___1
0,395582	1,79044	2,6077	-1,37321	S	3	___1
-0,352001	-0,465099	-0,582161	-2,10401	S	2	___1
NaN	NaN	NaN	NaN	S	3	___1
NaN	NaN	NaN	NaN	S	2	___1
0,894733	0,320707	0,9548	-0,691307	S	2	___1
0,745251	1,80076	0,0684299	NaN	S	3	___1
NaN	NaN	NaN	NaN	S	2	___1
-0,507921	-0,988878	0,107214	NaN	S	2	___1
3,04229	2,68527	2,83779	0,0990944	S	3	___1
-0,489513	-0,41361	NaN	-0,722624	S	2	___1
0,61648	-0,146124	-0,515234	-1,25634	S	2	___1
-0,48209	1,17491	NaN	0,240396	S	3	___1
1,30804	1,02013	0,805529	-0,291203	S	2	___1

NaN	0,346748	-2,14974	-2,88862	S	3	___1
NaN	NaN	NaN	NaN	S	3	___1
NaN	NaN	NaN	NaN	S	2	___1
0,493137	0,527452	4,08503	-0,460279	S	3	___1
0,493137	NaN	4,08503	NaN	S	3	___1
NaN	NaN	NaN	NaN	S	2	___1
-0,591909	-0,891445	-0,822716	-0,21459	S	2	___1
1,78263	NaN	NaN	NaN	S	3	___1
0,887766	-0,578459	NaN	0,556065	S	3	___1
2,55842	3,10736	0,922869	2,37676	S	2	___1
-1,11924	-0,961891	NaN	-1,87505	S	3	___1
-0,134825	-0,534431	-0,955563	-2,44008	S	2	___1
-0,391178	0,587399	0,123478	NaN	S	3	___1
2,12102	2,79199	3,77583	2,68478	S	3	___1
NaN	-0,0591412	-0,673569	-0,457575	S	2	___1
0,294201	-1,39044	-0,271967	-0,283028	S	2	___1
-1,10695	-1,81476	-2,04633	-0,451187	S	2	___1
-0,795502	-1,31875	0,522472	-0,92581	S	2	___1
1,91365	1,31718	1,2804	0,660147	S	3	___1
0,113272	0,428371	0,531824	-0,405977	S	3	___1
NaN	NaN	NaN	NaN	S	2	___1
-1,44648	NaN	-1,2893	-0,138666	S	2	___1
NaN	NaN	-1,78365	NaN	S	2	___1
NaN	NaN	NaN	NaN	S	2	___1
NaN	NaN	NaN	NaN	S	2	___1
NaN	NaN	NaN	NaN	S	2	___1
NaN	NaN	NaN	NaN	S	2	___1
-1,86536	-2,36923	NaN	-2,00808	S	2	___1
-1,66562	-2,0386	-3,26878	NaN	S	2	___1
-1,26708	-1,07591	-2,30738	0,200529	S	2	___1
4,77229	4,05943	2,15912	3,89382	S	2	___1
-0,332457	0,963657	NaN	0,577869	S	3	___1
-0,506985	-1,72676	-4,48688	-0,970415	S	2	___1
0,289135	-0,675959	-2,02079	-1,18404	S	2	___1

0,502584	-1,00084	-2,53085	1,03053	S	2	___1
-1,30939	NaN	-2,78126	-1,59691	S	2	___1
NaN	-3,07569	NaN	-2,1195	S	2	___1
2,41319	-0,246853	2,19285	1,58961	S	2	___1
0,904114	-1,1647	-2,4025	0,379333	S	3	___1
1,25612	0,681185	NaN	0,235609	S	2	___1
-0,110643	-1,13058	1,47111	2,61744	S	2	___1
-1,13505	-2,02666	-3,41829	-1,63402	S	2	___1
-1,67057	-4,15888	-1,9424	0,402166	S	2	___1
2,84428	1,45034	0,857964	1,19231	S	2	___1
NaN	NaN	NaN	NaN	S	2	___1
2,26745	1,25398	0,532869	-1,1511	S	2	___1
-0,705967	0,113956	-0,297569	-1,53024	S	3	___1
-1,38755	-1,70063	-1,63363	-0,475603	S	3	___1
-2,89189	-1,57669	NaN	-1,07456	S	2	___1
-2,74722	-0,657669	-0,150719	-0,114552	S	3	___1
-1,77544	-2,59112	NaN	NaN	S	2	___1
1,08351	2,01275	-1,76265	1,05226	S	3	___1
0,774975	1,54189	-0,257454	1,26974	S	3	___1
NaN	-0,818388	0,442404	-0,746006	S	3	___1
1,78454	2,20864	NaN	0,250238	S	4	___1
-0,630758	-2,71593	NaN	NaN	S	2	___1
-0,534725	-1,16905	0,310925	-0,636822	S	2	___1
NaN	1,35708	0,208679	0,456984	S	3	___1
-0,325146	-1,88276	-0,152218	-1,40579	S	2	___1
2,22287	1,04279	NaN	1,35582	S	2	___1
3,5856	2,35837	1,71158	4,57673	S	2	___1
-0,734392	-0,664501	0,240845	-0,641527	S	2	___1
NaN	NaN	NaN	NaN	S	3	___1
7,04204	6,23388	5,44981	5,29168	S	4	___1
NaN	0,56498	-0,976797	0,801466	S	2	___1
-0,561115	-1,44486	NaN	-1,46826	S	2	___1
-0,74806	NaN	NaN	NaN	S	2	___1
0,692028	-0,502621	-1,27481	-0,229095	S	2	___1

Polito et al. Universal ready-to-use immunotherapeutic approach for the treatment of cancer: expanded and activated polyclonal γδ memory T cells.
Phosphorylation sites

NaN	NaN	NaN	NaN	S	2	___1
-0,932417	-0,928185	1,55904	-1,83163	S	2	___1
0,487316	-0,563709	NaN	NaN	S	2	___1
2,77849	3,23866	4,24443	1,15787	S	2	___1
NaN	NaN	NaN	NaN	S	3	___1
0,00911903	0,750942	-0,552229	NaN	S	2	___1
3,45483	3,08476	4,24529	1,81425	S	3	___1
1,24274	0,676546	0,755949	0,0253315	S	2	___1
0,109005	-0,598976	-0,874216	-0,458218	S	2	___1
NaN	NaN	NaN	NaN	S	2	___1
0,601053	1,18818	0,232014	0,551437	S	3	___1
NaN	NaN	NaN	NaN	S	2	___1
-0,789381	-1,41393	0,0381279	-0,473814	S	2	___1
NaN	-0,673903	-0,373869	-0,32872	S	3	___1
NaN	NaN	NaN	NaN	S	2	___1
-0,0519238	0,724535	-1,08901	0,0236053	S	3	___1
1,8329	1,79402	2,9085	0,993708	S	2	___1
-2,3379	-2,25434	-2,50565	-1,80798	S	2	___1
1,54945	2,20812	1,72122	0,692873	S	2	___1
NaN	-2,72789	NaN	NaN	S	2	___1
-0,284149	-0,162828	NaN	1,54021	S	2	___1
NaN	NaN	NaN	NaN	S	2	___1
-1,0804	-0,860565	-0,644062	-1,11351	S	2	___1
0,507931	1,38611	1,12996	-0,303667	S	3	___1
-1,58826	-0,504877	-0,917566	NaN	S	2	___1
NaN	NaN	NaN	NaN	S	2	___1
NaN	NaN	NaN	NaN	S	2	___1
0,898325	0,590069	NaN	NaN	S	2	___1
-0,897274	-2,88681	-0,325558	NaN	S	2	___1
NaN	NaN	NaN	NaN	S	3	___1
-0,897854	-0,524368	1,93083	-1,77049	S	2	___1
NaN	NaN	NaN	NaN	S	2	___1
-1,94944	0,706488	1,75726	0,0319462	S	3	___1
-0,721367	-1,10666	-1,0081	NaN	S	2	___1

Polito et al. Universal ready-to-use immunotherapeutic approach for the treatment of cancer: expanded and activated polyclonal γδ memory T cells.
Phosphorylation sites

0,58712	2,57664	1,67057	0,390974	S	3	___1
0,872992	NaN	NaN	NaN	S	2	___1
1,24508	0,718191	3,42119	-0,494555	S	3	___1
-1,75799	-1,66602	-2,47208	-3,24024	S	3	___1
NaN	NaN	NaN	NaN	S	2	___1
NaN	NaN	NaN	NaN	S	3	___1
0,969149	1,02536	NaN	0,141357	S	2	___1
NaN	NaN	NaN	NaN	S	2	___1
NaN	NaN	NaN	NaN	S	3	___1
4,49748	0,301771	0,271612	4,15359	S	3	___1
-0,868711	-2,44666	-1,77693	NaN	S	2	___1
NaN	NaN	NaN	NaN	S	2	___1
5,01235	4,28454	1,34376	1,94891	S	2	___1
NaN	NaN	NaN	NaN	S	2	___1
0,727468	1,90702	0,322474	0,639572	S	3	___1
NaN	NaN	NaN	NaN	S	2	___1
-0,549355	0,0445175	NaN	0,0335712	S	2	___1
NaN	NaN	NaN	NaN	S	2	___1
-0,27042	0,819801	0,550442	-0,852751	S	3	___1
NaN	0,195976	-1,64924	NaN	S	3	___1
0,711245	0,724947	0,025629	-0,2941	S	2	___1
-1,03234	0,2334	NaN	-3,39701	S	2	___1
1,24817	0,902409	0,838312	1,67725	S	2	___1
0,132015	-0,863459	NaN	0,0129185	S	2	___1
-0,0942764	NaN	NaN	-0,211983	S	2	___1
-2,09474	-2,6559	NaN	-0,65596	S	2	___1
NaN	NaN	NaN	NaN	S	2	___1
-2,00733	-1,48361	-1,24565	NaN	S	2	___1
-1,7996	-2,56627	NaN	1,00215	S	2	___1
NaN	NaN	NaN	NaN	S	2	___1
-0,634626	-2,195	-0,629263	NaN	S	2	___1
-3,98097	NaN	-2,19016	-1,03389	S	2	___1
NaN	NaN	NaN	NaN	S	2	___1
-2,16812	0,0589218	0,700171	NaN	S	2	___1

NaN	NaN	-0,849514	NaN	S	2	___1
NaN	-1,8323	-0,715145	NaN	S	2	___1
NaN	NaN	NaN	NaN	S	2	___1
-0,888138	-0,979031	-1,16453	NaN	S	2	___1
NaN	-0,699768	-1,15196	-0,347345	S	2	___1
NaN	NaN	NaN	NaN	S	3	___1
NaN	NaN	NaN	NaN	S	2	___1
-0,340067	-1,02802	-1,84078	-0,428595	S	2	___1
NaN	NaN	NaN	NaN	S	2	___1
-1,06952	-1,14338	-1,43159	-1,64453	S	2	___1
NaN	-1,95794	NaN	-1,66169	S	3	___1
-0,594261	-2,66078	NaN	-1,47502	S	3	___1
1,06783	1,79686	NaN	-0,742083	S	2	___1
NaN	NaN	NaN	NaN	S	2	___1
1,2822	1,74315	NaN	NaN	S	2	___1
2,27334	1,97513	NaN	0,550829	S	3	___1
-0,161121	0,520864	NaN	0,0341206	S	2	___1
NaN	1,47255	1,19738	1,17447	S	2	___1
-1,07762	1,09492	-1,6556	-0,740986	S	2	___1
3,63164	3,22316	3,55448	2,9324	S	2	___1
1,86102	1,19515	-2,31547	-2,73801	S	2	___1
1,69866	2,26344	2,85982	2,15593	S	2	___1
NaN	-0,398645	NaN	-0,084137	S	3	___1
NaN	NaN	NaN	NaN	S	3	___1
-0,346926	-0,406963	0,99272	-0,600033	S	2	___1
2,33108	1,52535	-0,494658	NaN	S	2	___1
1,46161	NaN	2,16904	0,0141487	S	2	___1
0,746098	-0,767181	1,04532	-0,267408	S	2	___1
NaN	NaN	NaN	NaN	S	3	___1
-0,0843601	1,17766	NaN	-1,19724	S	2	___1
-0,469467	0,34227	-2,89366	-1,53573	S	2	___1
NaN	NaN	NaN	NaN	S	4	___1
-2,06877	-0,591078	NaN	0,765402	S	2	___1
-2,00733	-2,68695	-1,80846	0,534233	S	2	___1

Polito et al. Universal ready-to-use immunotherapeutic approach for the treatment of cancer: expanded and activated polyclonal γδ memory T cells.
Phosphorylation sites

NaN	NaN	NaN	NaN	S	2	___1
-0,706816	-2,34982	-2,92292	-0,72727	S	2	___1
-0,0984612	-1,46458	NaN	0,8701	S	3	___1
NaN	NaN	NaN	NaN	S	3	___1
0,391731	-0,791376	-0,292685	-0,620703	S	2	___1
-0,753191	-0,511993	NaN	-0,482821	S	2	___1
NaN	NaN	NaN	NaN	S	2	___1
-3,37231	NaN	-0,840734	NaN	S	2	___1
0,0932407	-0,78418	-1,069	0,24576	S	2	___1
NaN	NaN	NaN	NaN	S	3	___1
NaN	NaN	NaN	NaN	S	2	___1
0,198774	NaN	-2,05114	NaN	S	2	___1
2,11887	0,51207	0,347145	-1,3532	S	2	___1
NaN	NaN	NaN	NaN	S	3	___1
NaN	NaN	NaN	NaN	S	3	___1
1,28092	0,0792961	-1,13366	1,18171	S	2	___1
1,40255	1,1781	2,32994	0,900911	S	2	___1
2,45852	1,08813	0,810314	0,131405	S	2	___1
3,13788	1,64633	2,3334	1,66157	S	2	___1
NaN	NaN	NaN	NaN	S	2	___1
-1,91467	-1,26297	NaN	-1,4883	S	3	___1
4,27935	4,78483	5,80612	4,85114	S	3	___1
2,57259	0,936312	1,81766	1,43489	S	2	___1
3,239	1,99769	3,78717	2,11725	S	2	___1
NaN	NaN	NaN	NaN	S	2	___1
0,767729	-0,4123	NaN	0,576756	S	2	___1
1,12988	4,00056	2,1901	4,27846	S	2	___1
NaN	NaN	NaN	NaN	S	2	___1
-1,44705	-1,70759	NaN	-1,36642	S	2	___1
NaN	0,867191	-0,269663	0,602671	S	2	___1
-1,08216	-3,44947	-4,98901	-1,92772	S	2	___1
-0,664354	-2,17006	NaN	-1,35158	S	2	___1
2,53446	1,8669	3,30458	0,979403	S	2	___1
2,8492	3,40151	2,73338	2,93962	S	2	___1

Polito et al. Universal ready-to-use immunotherapeutic approach for the treatment of cancer: expanded and activated polyclonal γδ memory T cells.
Phosphorylation sites

1,22152	0,554026	1,33336	1,40769	S	2	___1
-0,767982	-2,74572	NaN	0,621431	S	2	___1
NaN	0,296103	-1,34438	0,630068	S	2	___1
0,212145	-1,10884	NaN	NaN	S	2	___1
-4,74119	1,7851	-3,17808	-1,22698	S	2	___1
NaN	-0,589252	1,06703	2,27561	S	2	___1
-1,01578	-0,0594559	1,15891	2,72884	S	3	___1
-1,56583	-0,430672	-1,22168	-1,90079	S	2	___1
-2,60578	-1,94452	-0,469078	NaN	S	3	___1
-1,40919	-1,02817	-0,579931	NaN	S	3	___1
0,126884	-4,38148	NaN	1,08851	S	2	___1
NaN	1,94076	-0,992039	2,82016	S	3	___1
NaN	NaN	NaN	NaN	S	2	___1
4,04079	3,7161	1,40665	3,6193	S	2	___1
-0,38063	-0,502943	0,4716	-1,87823	S	2	___1
1,31279	1,95692	2,879	2,11525	S	2	___1
0,995211	1,00835	NaN	1,77454	S	3	___1
-1,39655	-2,47133	NaN	-0,910406	S	4	___1
-0,48621	-1,25934	0,235043	NaN	S	2	___1
-0,617142	-0,364874	0,0959339	-0,424627	S	2	___1
0,185337	-0,352434	0,999954	-0,0442657	S	2	___1
NaN	NaN	NaN	NaN	S	2	___1
-2,1165	-1,52655	NaN	-1,56233	S	2	___1
-1,39363	-3,16852	-2,57997	-2,60858	S	2	___1
NaN	NaN	NaN	NaN	S	2	___1
NaN	NaN	NaN	NaN	S	2	___1
-0,0959053	-0,534431	-0,403978	0,108578	S	2	___1
3,27316	1,92819	0,831003	3,0065	S	2	___1
NaN	NaN	NaN	NaN	S	2	___1
NaN	NaN	NaN	NaN	S	2	___1
1,04079	NaN	NaN	NaN	S	2	___1
NaN	-3,63193	-2,67594	-0,350956	S	2	___1
-2,7257	NaN	-1,12328	-0,350088	S	3	___1
0,372028	-0,611429	NaN	-0,171715	S	2	___1

-0,176035	-0,290403	-0,337124	-0,310711	S	2	___1
0,733795	NaN	-2,06647	-2,08014	S	3	___1
-0,295713	0,360744	1,72587	0,546104	S	4	___1
NaN	-1,47486	NaN	NaN	S	2	___1
-1,19797	-1,26929	-1,87913	-1,53342	S	2	___1
-1,1145	NaN	NaN	NaN	S	2	___1
-0,653414	-3,56788	NaN	-1,20684	S	3	___1
-3,32627	-2,71469	NaN	-1,86905	S	3	___1
1,38739	0,94681	1,46943	0,937943	S	3	___1
NaN	NaN	NaN	NaN	S	2	___1
-1,815	-1,27989	-2,33319	-2,53954	S	2	___1
-0,860258	-0,482784	NaN	0,813778	S	4	___1
NaN	NaN	NaN	NaN	S	2	___1
0,372028	-0,611429	NaN	-0,171715	S	2	___1
-1,43126	-2,48841	NaN	0,347376	S	3	___1
0,0715446	-0,934935	-0,899044	-0,667374	S	2	___1
0,210409	-0,00550652	0	-1,79321	S	2	___1
NaN	NaN	NaN	NaN	S	2	___1
NaN	NaN	NaN	NaN	S	3	___1
NaN	NaN	NaN	NaN	S	2	___1
NaN	2,67284	-0,142628	1,68434	S	2	___1
NaN	NaN	NaN	NaN	S	2	___1
2,27069	0,562159	NaN	1,54551	S	2	___1
0,335619	0,235397	-0,807972	-1,06497	S	2	___1
-2,48567	NaN	-3,00771	NaN	S	2	___1
0,736065	0,382305	-0,589531	-0,474594	S	2	___1
1,40595	-0,677898	-0,897873	-0,399721	S	2	___1
-1,25413	-0,348665	NaN	-0,00544548	S	2	___1
NaN	NaN	NaN	-0,00544548	S	2	___1
0,703627	-0,655041	-0,0227776	0,33363	S	3	___1
NaN	NaN	NaN	NaN	S	2	___1
0,641062	1,87217	-1,75304	-0,530983	S	2	___1
-0,677483	0,513397	-0,569187	0,215212	S	2	___1
NaN	0,985182	0,527777	-0,000469208	S	2	___1

NaN	-1,95488	-2,35089	-1,00968	S	3	___1
-3,16776	-2,46387	-1,09955	NaN	S	2	___1
0,370234	0,065094	0,899353	-0,635328	S	3	___1
NaN	-2,0278	-2,30969	NaN	S	2	___1
-0,295118	0,109961	1,49616	-0,11483	S	3	___1
-1,49156	-2,60803	-1,48962	NaN	S	2	___1
1,17906	2,07784	0,389372	-0,348806	S	2	___1
NaN	NaN	NaN	NaN	S	2	___1
NaN	NaN	NaN	NaN	S	2	___1
NaN	-1,69421	-1,08585	-2,0518	S	3	___1
NaN	NaN	NaN	NaN	S	3	___1
NaN	NaN	NaN	NaN	S	2	___1
0,0438824	-1,11706	NaN	-0,511217	S	2	___1
-1,89045	-1,22488	-0,720926	NaN	S	2	___1
NaN	NaN	-1,93963	NaN	S	2	___1
-2,72332	-1,81857	-0,68602	NaN	S	2	___1
-1,03539	-0,201263	-1,13965	NaN	S	2	___1
NaN	-1,07871	NaN	NaN	S	2	___1
1,25316	2,45488	3,77523	0,395575	S	2	___1
0,485758	0,586843	1,49781	-0,695057	S	2	___1
0,666801	0,992439	-2,82563	-1,57232	S	2	___1
-1,2524	0,194983	-2,44483	0,159592	S	5	___1
NaN	2,42301	NaN	-1,21617	S	3	___1
2,92122	1,79048	2,5755	1,35435	S	2	___1
-0,431776	-0,706802	NaN	-0,31074	S	2	___1
3,54818	1,93475	1,69349	1,35252	S	2	___1
2,82207	1,83422	-1,13121	3,5298	S	2	___1
NaN	1,19568	NaN	0,305058	S	3	___1
NaN	NaN	NaN	NaN	S	3	___1
-0,247656	-0,399845	0,289719	-0,828629	S	3	___1
2,0291	1,58336	NaN	1,3644	S	4	___1
-1,35643	-1,6676	-3,22979	-1,69658	S	2	___1
0,815996	1,71358	-3,07188	0,28476	S	2	___1
-1,35723	-2,52952	NaN	-0,144144	S	2	___1

Polito et al. Universal ready-to-use immunotherapeutic approach for the treatment of cancer: expanded and activated polyclonal γδ memory T cells.
Phosphorylation sites

4,71527	4,11359	1,31299	4,40304	S	2	___1
2,42633	-0,705442	2,27478	0,291264	S	2	___1
0,627377	NaN	0,679916	NaN	S	2	___1
-4,12008	NaN	-0,982212	-1,11793	S	2	___1
-1,05304	0,74968	NaN	0,0959206	S	3	___1
-0,737566	NaN	NaN	NaN	S	2	___1
0,400446	0,273457	NaN	1,49936	S	2	___1
NaN	NaN	NaN	NaN	S	3	___1
-1,49312	-2,19455	-0,575937	-2,00066	S	2	___1
-1,85982	NaN	-3,63931	-1,94765	S	2	___1
-2,0356	-2,30367	-2,10199	0,469542	S	3	___1
-0,32958	-1,08463	-1,06034	-0,699919	S	2	___1
NaN	-0,957733	0,0922699	-0,491028	S	2	___1
-2,14928	-2,60946	-1,19997	NaN	S	2	___1
0,347431	NaN	NaN	2,4663	S	2	___1
-0,0521755	1,71533	0,356192	2,25549	S	3	___1
NaN	-0,56831	-0,541552	0,837044	S	2	___1
-0,63151	0,856682	1,85559	0,838747	S	2	___1
-0,63151	0,856682	1,85559	0,838747	S	2	___1
NaN	NaN	NaN	NaN	S	2	___1
-0,293419	-1,87245	NaN	-0,670427	S	2	___1
NaN	2,01063	2,87005	1,5648	S	2	___1
NaN	1,20086	2,57807	2,08664	S	3	___1
-1,38909	-1,01238	NaN	-0,248779	S	3	___1
NaN	NaN	NaN	NaN	S	2	___1
-0,192926	-0,504662	-0,476215	-3,00418	S	3	___1
NaN	-0,418262	-0,693792	NaN	S	3	___1
NaN	NaN	NaN	NaN	S	2	___1
0,554346	0,0472336	NaN	NaN	S	3	___1
-1,98919	-2,17098	NaN	-0,353348	S	2	___1
-1,8458	NaN	NaN	-1,92995	S	2	___1
NaN	-2,59836	-0,874557	0,818834	S	2	___1
-1,7765	0,2248	NaN	NaN	S	2	___1
NaN	NaN	NaN	NaN	S	2	___1

NaN	-0,167162	-1,13642	-0,434406	S	3	___1
NaN	NaN	NaN	NaN	S	2	___1
-0,698189	-1,02322	0,881905	-0,663473	S	2	___1
NaN	NaN	NaN	NaN	S	3	___1
-1,54465	-1,53241	-1,54661	-0,343475	S	4	___1
2,53476	3,68756	NaN	4,17229	S	3	___1
NaN	NaN	NaN	NaN	S	3	___1
NaN	NaN	NaN	NaN	S	2	___1
NaN	-0,842512	NaN	0,370825	S	2	___1
0,796101	-0,321037	NaN	NaN	S	3	___1
-0,455147	NaN	NaN	-1,52733	S	2	___1
1,82529	1,54909	0,0184326	2,9239	S	2	___1
NaN	NaN	0,116409	NaN	S	3	___1
-0,408964	-0,845234	0,826548	-0,945841	S	2	___1
-0,526117	1,26051	NaN	-1,22239	S	2	___1
0,32346	NaN	NaN	-0,216141	S	2	___1
2,71548	5,57991	NaN	4,90647	S	2	___1
-0,682146	-1,38461	NaN	0,142376	S	2	___1
-1,24967	-0,858778	-1,80748	-0,31426	S	2	___1
1,28973	1,36455	1,66052	3,18563	S	2	___1
-1,20834	NaN	NaN	-1,08406	S	3	___1
NaN	NaN	NaN	NaN	S	3	___1
NaN	0,716486	1,0259	-0,0530853	S	2	___1
-0,521187	0,378462	-0,565807	-0,445784	S	2	___1
-0,473791	-0,381859	NaN	-0,646322	S	2	___1
0,63246	0,197233	NaN	NaN	S	2	___1
NaN	NaN	NaN	NaN	S	2	___1
0,573891	-0,399046	1,82307	NaN	S	3	___1
-1,03823	-1,76963	NaN	NaN	S	2	___1
NaN	NaN	NaN	NaN	S	2	___1
2,21289	0,960194	-0,15992	1,39028	S	2	___1
NaN	NaN	NaN	NaN	S	3	___1
NaN	NaN	NaN	NaN	S	3	___1
2,2807	1,50271	-3,10367	1,33108	S	2	___1

1,71785	1,57257	1,45354	1,07056	S	2	___1
-1,52567	NaN	NaN	NaN	S	3	___1
NaN	NaN	NaN	NaN	S	2	___1
NaN	NaN	NaN	NaN	S	2	___1
1,44745	1,86401	-0,658171	1,28751	S	2	___1
NaN	0,84124	-1,56736	-0,173405	S	3	___1
NaN	0,450006	-0,145044	-0,168447	S	3	___1
0,685127	0,382771	NaN	0,260658	S	2	___1
0,469835	-0,0981998	NaN	-0,195177	S	2	___1
-1,63012	-1,91078	NaN	0,14176	S	2	___1
NaN	-1,60339	-2,55144	NaN	S	3	___1
NaN	-0,189892	-1,53537	0,369213	S	2	___1
0,303888	-0,304657	0,00675774	0,0442657	S	2	___1
NaN	-2,19646	-1,91906	-0,375694	S	2	___1
NaN	NaN	NaN	NaN	S	3	___1
NaN	NaN	NaN	NaN	S	2	___1
NaN	NaN	NaN	NaN	S	2	___1
-1,58784	NaN	-2,2049	-0,973633	S	2	___1
NaN	NaN	NaN	NaN	S	2	___1
-0,245026	0,354771	-0,762251	-0,212933	S	3	___1
-1,24007	-0,395552	NaN	NaN	S	3	___1
-2,73797	-1,43458	-2,1046	-1,70518	S	3	___1
-1,5387	-2,62271	NaN	NaN	S	2	___1
NaN	NaN	NaN	NaN	S	3	___1
NaN	NaN	NaN	NaN	S	2	___1
NaN	0,202972	0,554598	-1,36088	S	2	___1
2,53512	2,63613	3,84104	2,7064	S	2	___1
0,493507	1,83736	NaN	1,52523	S	3	___1
-0,708174	0,334719	0,788382	0,850489	S	2	___1
-0,70908	-0,557783	0,625151	0,155033	S	2	___1
2,27169	1,52266	-1,00571	1,48529	S	2	___1
-0,0302505	0,402687	-1,15103	-0,336004	S	2	___1
-3,19403	NaN	-0,775099	-0,390081	S	2	___1
2,33204	1,7178	NaN	0,582617	S	2	___1

-2,75401	NaN	NaN	-3,12181	S	2	___1
NaN	NaN	-1,88798	-0,236059	S	2	___1
NaN	-0,0347118	NaN	-0,167343	S	2	___1
-1,00232	-1,46103	NaN	NaN	S	3	___1
0,833582	-0,870369	0,0621567	-0,833464	S	2	___1
5,67806	4,72585	3,21941	4,26158	S	4	___1
-3,48588	NaN	-1,75446	-1,94855	S	2	___1
-1,42837	-2,22247	-2,3752	-1,89095	S	2	___1
-1,75975	-2,04897	NaN	-1,64497	S	2	___1
NaN	NaN	NaN	NaN	S	2	___1
-1,23892	NaN	NaN	NaN	S	2	___1
-0,653631	0,826057	NaN	-0,615812	S	3	___1
-2,00844	0,501829	-0,662474	-0,424187	S	2	___1
-0,903677	NaN	NaN	0,0695534	S	2	___1
NaN	NaN	NaN	NaN	S	3	___1
NaN	1,16061	NaN	0,804018	S	3	___1
-0,219912	-0,229866	NaN	0,178591	S	2	___1
-1,82001	-1,88611	NaN	-3,4529	S	2	___1
-3,22706	1,48232	NaN	-1,5926	S	4	___1
-0,741615	-1,40447	-2,01307	NaN	S	4	___1
2,41819	2,69138	-0,700357	NaN	S	2	___1
0,282	-0,472757	0,0516415	1,40371	S	3	___1
-0,262625	-0,664021	0,669769	2,47555	S	2	___1
3,74937	3,52299	4,13952	5,40293	S	2	___1
-1,34169	-1,09572	-2,08669	-2,09228	S	2	___1
0,585575	0,533749	NaN	0,0492325	S	3	___1
0,193087	NaN	0,222071	0,116615	S	3	___1
4,11053	4,23601	3,25092	3,5097	S	3	___1
-0,79484	1,7586	-1,61732	NaN	S	2	___1
0,458181	-0,590506	NaN	NaN	S	2	___1
2,60256	2,68444	3,2089	1,4534	S	2	___1
NaN	NaN	0,619102	0,597448	S	3	___1
NaN	NaN	NaN	NaN	S	3	___1
4,11683	2,16241	6,11908	3,49478	S	2	___1

Polito et al. Universal ready-to-use immunotherapeutic approach for the treatment of cancer: expanded and activated polyclonal γδ memory T cells.
Phosphorylation sites

NaN	NaN	NaN	NaN	S	2	___1
0,192905	-0,144447	NaN	-0,5376	S	3	___1
0,072403	NaN	NaN	NaN	S	2	___1
0,99328	-0,564381	-0,949976	NaN	S	2	___1
NaN	2,62928	4,26878	2,49671	S	2	___1
5,31179	4,70377	5,24572	3,99949	S	2	___1
NaN	NaN	NaN	NaN	S	2	___1
2,0035	2,0328	0,943468	1,10886	S	2	___1
-0,727491	-1,06468	-0,890219	-0,318081	S	2	___1
3,82759	NaN	1,571	4,08733	S	2	___1
-2,16915	-0,630075	NaN	-0,69574	S	3	___1
1,15169	0,544182	0,398088	0,301413	S	2	___1
0,0256519	1,78433	-1,5289	-0,887733	S	2	___1
0,27503	-0,635246	-0,130041	-0,293785	S	3	___1
NaN	NaN	NaN	NaN	S	2	___1
-0,901669	NaN	-1,59107	NaN	S	3	___1
4,32948	3,73365	5,20402	4,03556	S	2	___1
4,51224	3,88634	4,76258	3,3569	S	2	___1
NaN	-0,182047	-0,0530987	NaN	S	3	___1
-0,019289	0,980272	-3,60097	-0,303436	S	2	___1
-1,07178	-0,526876	-2,3729	-1,39288	S	2	___1
0,0659294	-0,611893	NaN	NaN	S	2	___1
1,02769	2,09423	1,01143	0,645784	S	2	___1
-1,31498	-2,04034	-1,60439	NaN	S	2	___1
-2,45352	-1,50168	-1,4816	-2,29381	S	3	___1
-0,00114441	-1,54773	NaN	-0,622723	S	3	___1
NaN	1,52102	-0,908188	-0,104443	S	4	___1
NaN	NaN	NaN	NaN	S	3	___1
NaN	0,413198	NaN	NaN	S	2	___1
-0,0637894	-0,942476	NaN	-0,107971	S	2	___1
-0,14728	1,19859	-1,39489	-0,455067	S	2	___1
NaN	NaN	NaN	NaN	S	2	___1
NaN	NaN	NaN	NaN	S	3	___1
NaN	NaN	NaN	NaN	S	2	___1

1,59053	-0,171082	2,90514	-1,03918	S	3	___1
0,948542	NaN	2,44331	-0,62999	S	2	___1
-0,261379	0,789339	NaN	1,1242	S	2	___1
1,64782	NaN	-1,1685	0,0839024	S	2	___1
2,43257	0,84293	4,75751	1,50383	S	2	___1
NaN	-0,309252	-1,04498	-0,130873	S	3	___1
NaN	2,62941	NaN	2,47496	S	2	___1
-0,324104	-1,1569	-2,29625	NaN	S	2	___1
2,97804	2,34623	0,481369	1,56899	S	2	___1
-1,55377	1,04712	0,289604	0,917501	S	3	___1
2,85313	2,39591	2,11918	1,33043	S	2	___1
NaN	1,92098	NaN	NaN	S	2	___1
-1,01641	-1,12037	-1,4904	NaN	S	2	___1
-0,396185	-1,06984	NaN	-2,88425	S	2	___1
0,119154	2,03975	0,707886	0,812845	S	2	___1
-0,812378	-0,523932	NaN	-0,634237	S	2	___1
1,75672	-0,872866	NaN	-0,716568	S	3	___1
0,108685	-1,70347	-2,74595	-0,157484	S	2	___1
NaN	-0,81345	0,760214	-0,739733	S	2	___1
-0,196695	0,276463	NaN	1,14267	S	2	___1
2,11055	NaN	-3,16266	1,62467	S	2	___1
NaN	NaN	-1,67249	NaN	S	2	___1
1,01333	2,26059	NaN	-1,35763	S	3	___1
NaN	NaN	NaN	NaN	S	3	___1
1,48296	2,11546	2,71393	NaN	S	2	___1
-0,127008	-0,569883	NaN	-1,70175	S	1	___1
-1,96221	-1,3638	NaN	-2,57959	S	2	___1
-0,485481	0,896355	1,18658	0,404522	S	2	___1
-1,35865	-0,562588	-1,50513	-2,74654	S	2	___1
NaN	NaN	NaN	NaN	S	3	___1
-0,756407	-2,71231	-0,835325	0,872406	S	2	___1
0,0710182	-0,261951	-2,60906	-0,676968	S	2	___1
NaN	NaN	NaN	NaN	S	2	___1
-1,38465	NaN	NaN	NaN	S	2	___1

Polito et al. Universal ready-to-use immunotherapeutic approach for the treatment of cancer: expanded and activated polyclonal γδ memory T cells.
Phosphorylation sites

1,21892	-0,333496	NaN	-2,5276	S	2	___1
1,36618	0,315357	-1,05438	-1,06037	S	2	___1
NaN	NaN	NaN	NaN	S	2	___1
-0,902121	-1,3565	NaN	-1,75744	S	2	___1
-0,902121	NaN	NaN	NaN	S	2	___1
-1,60144	-3,25047	NaN	NaN	S	2	___1
NaN	NaN	NaN	NaN	S	3	___1
-2,09486	-1,85428	-0,518198	-2,52113	S	2	___1
0,0959682	2,2582	NaN	0,61447	S	3	___1
3,7522	4,00297	4,32607	3,30356	S	2	___1
2,588	2,51393	NaN	-0,61725	S	2	___1
0,736002	-1,88923	-1,09164	NaN	S	2	___1
NaN	NaN	NaN	NaN	S	3	___1
-0,470715	-0,932316	-1,83769	-1,6736	S	2	___1
3,83868	1,9223	2,99155	1,70784	S	2	___1
4,26783	-0,609577	-1,50229	2,24188	S	3	___1
NaN	NaN	NaN	NaN	S	2	___1
NaN	NaN	NaN	NaN	S	2	___1
-0,988989	-0,631601	NaN	-2,55611	S	2	___1
1,27749	2,15722	-0,930416	0,673985	S	2	___1
-1,39865	-2,38032	-2,18233	-1,9116	S	2	___1
0,284449	-0,453844	-0,843573	NaN	S	2	___1
-2,22616	-2,14977	-4,24385	-2,87117	S	2	___1
0,465477	-0,931751	NaN	-1,59578	S	2	___1
3,67564	3,06841	4,03834	3,18957	S	2	___1
NaN	NaN	NaN	NaN	S	2	___1
NaN	NaN	NaN	NaN	S	2	___1
-1,24351	-0,838034	NaN	-1,49093	S	3	___1
-0,970743	-1,26492	NaN	-1,79337	S	2	___1
NaN	-0,912691	0,338152	0,254271	S	3	___1
NaN	-1,63409	NaN	-0,356644	S	3	___1
NaN	NaN	NaN	NaN	S	2	___1
NaN	NaN	NaN	NaN	S	3	___1
-1,9027	NaN	NaN	NaN	S	2	___1

NaN	NaN	NaN	NaN	S	3	___1
NaN	NaN	NaN	NaN	S	4	___1
NaN	NaN	NaN	NaN	S	3	___1
NaN	NaN	NaN	NaN	S	3	___1
-1,61581	NaN	NaN	NaN	S	2	___1
-1,53357	-3,04303	0,796177	-1,76101	S	2	___1
3,89956	3,00127	3,82147	3,24253	S	2	___1
2,21035	2,50595	1,61596	1,44551	S	2	___1
4,17544	2,377	-0,617575	1,56883	S	3	___1
-0,273432	-1,36825	-1,48707	0,319189	S	2	___1
2,54406	-0,112141	2,6337	2,25003	S	2	___1
-3,95914	-0,123407	0,804462	NaN	S	2	___1
0,091877	-0,238335	-0,441666	NaN	S	2	___1
NaN	NaN	NaN	NaN	S	2	___1
0,165981	0,387934	0,601984	-0,643539	S	2	___1
NaN	1,85455	1,40805	1,86059	S	4	___1
NaN	NaN	NaN	NaN	S	1	___1
-1,25215	-2,34323	NaN	NaN	S	2	___1
-1,36846	-1,72621	NaN	-0,742474	S	2	___1
0,00636101	-4,06126	0,846489	-2,06027	S	2	___1
0,987787	-0,0338573	1,32947	NaN	S	2	___1
-0,281916	-0,110502	NaN	-1,7603	S	2	___1
-0,498053	-0,0589828	2,31125	0,454594	S	2	___1
-2,80659	-1,73282	-2,77395	-3,59004	S	3	___1
-0,741905	-1,86511	-0,730896	NaN	S	2	___1
NaN	NaN	NaN	NaN	S	2	___1
-0,398102	-1,218	NaN	NaN	S	2	___1
2,03317	2,17865	1,64304	1,16624	S	2	___1
NaN	0,411716	NaN	2,35669	S	4	___1
2,9816	3,22697	2,18325	2,03453	S	2	___1
3,92387	1,95838	1,78818	1,75274	S	3	___1
3,96363	5,18924	1,70468	4,35337	S	3	___1
NaN	NaN	NaN	NaN	S	3	___1
3,97342	3,91943	4,27673	3,94529	S	2	___1

Polito et al. Universal ready-to-use immunotherapeutic approach for the treatment of cancer: expanded and activated polyclonal γδ memory T cells.
Phosphorylation sites

2,23742	0,523451	0,23838	0,72714	S	2	___1
5,16761	6,34144	6,01538	4,47822	S	2	___1
-0,300783	-0,207726	0,469046	-1,00305	S	2	___1
1,22781	2,68244	2,59851	2,09368	S	2	___1
-0,213348	1,25163	-1,69779	0,783888	S	4	___1
NaN	0,976425	NaN	0,747515	S	4	___1
1,41273	1,37271	1,52944	0,53441	S	2	___1
NaN	NaN	NaN	NaN	S	2	___1
NaN	NaN	NaN	NaN	S	2	___1
4,19849	3,14786	3,51288	3,69532	S	2	___1
1,92227	0,816147	NaN	1,20069	S	2	___1
1,86901	1,62801	2,91604	3,7134	S	2	___1
NaN	NaN	NaN	NaN	S	2	___1
0,347567	1,33877	2,32813	-1,23231	S	3	___1
-0,943634	-1,09838	-0,714724	-2,11503	S	2	___1
2,1	1,45485	2,76152	0,628084	S	3	___1
NaN	-1,13185	0,332581	NaN	S	2	___1
-1,30519	-1,67678	0,275232	NaN	S	2	___1
NaN	NaN	NaN	NaN	S	2	___1
NaN	NaN	NaN	NaN	S	3	___1
NaN	NaN	NaN	NaN	S	2	___1
NaN	NaN	NaN	NaN	S	2	___1
-0,717201	1,83902	NaN	1,04694	S	3	___1
2,71896	2,36849	1,31664	2,20115	S	2	___1
-3,24499	-3,62247	-3,65442	-3,8797	S	2	___1
-0,050703	NaN	NaN	NaN	S	2	___1
NaN	NaN	NaN	NaN	S	2	___1
NaN	NaN	NaN	NaN	S	3	___1
1,20702	0,63698	1,81964	1,72884	S	2	___1
NaN	NaN	NaN	NaN	S	2	___1
-1,63635	-2,88044	NaN	-2,46768	S	2	___1
1,00495	0,774439	1,83048	0,61038	S	2	___1
0,135616	0,224411	1,23909	-0,143549	S	2	___1
NaN	-0,576424	0,259628	-0,825222	S	2	___1

6,1636	6,82018	4,21628	6,40465	S	3	___1
NaN	NaN	NaN	NaN	S	2	___1
NaN	NaN	NaN	NaN	S	3	___1
NaN	1,51835	0,424774	0,961351	S	2	___1
1,64073	0,327427	1,41461	0,815777	S	2	___1
0,0137939	2,57511	1,69378	2,40592	S	4	___1
0,07691	-0,245684	NaN	-0,195553	S	2	___1
-0,894501	-1,26979	-1,7958	-2,39073	S	2	___1
0,72332	1,97371	-0,353951	0,227409	S	3	___1
-1,55581	1,47263	2,89486	4,13744	S	3	___1
-0,472395	-0,649324	NaN	0,373957	S	3	___1
-0,774132	-0,655161	-1,56964	-0,0812588	S	3	___1
NaN	-1,31012	-2,95872	NaN	S	3	___1
NaN	-1,81313	NaN	NaN	S	2	___1
NaN	NaN	NaN	NaN	S	3	___1
1,15409	1,54916	-3,82201	1,08289	S	2	___1
2,14252	1,43245	0,0835762	1,07323	S	3	___1
NaN	3,13842	1,70824	2,97375	S	4	___1
NaN	NaN	NaN	NaN	S	2	___1
-1,1726	NaN	-0,205164	-1,76945	S	2	___1
2,40438	2,71293	3,638	2,2163	S	2	___1
1,00401	0,254139	-1,57143	-0,470304	S	3	___1
-1,66002	-1,83879	NaN	-2,10714	S	2	___1
NaN	NaN	NaN	NaN	S	2	___1
NaN	NaN	NaN	NaN	S	2	___1
NaN	NaN	NaN	NaN	S	3	___1
0,239784	0,419163	NaN	-0,207108	S	2	___1
NaN	NaN	NaN	NaN	S	3	___1
NaN	NaN	NaN	NaN	S	2	___1
NaN	NaN	NaN	NaN	S	2	___1
-1,57014	-0,940365	-0,458101	NaN	S	2	___1
-0,951588	-0,0663433	NaN	-0,72921	S	2	___1
-1,93745	0,507599	-0,195749	2,07307	S	2	___1
NaN	NaN	NaN	NaN	S	3	___1

-3,61369	-2,86556	-2,62592	-0,478212	S	2	___1
-0,232271	-0,335407	NaN	0,549599	S	2	___1
NaN	-1,39893	NaN	NaN	S	2	___1
0,810593	NaN	1,23395	-0,511084	S	2	___1
0,514833	0,782833	0,242733	0,69681	S	3	___1
1,33617	0,860779	0,322435	0,61739	S	2	___1
2,33308	0,0112114	NaN	2,55925	S	2	___1
3,05543	1,66727	1,92084	4,27713	S	2	___1
-0,588518	-1,82664	-0,669935	0,16831	S	2	___1
1,09248	1,03959	2,5708	3,58227	S	2	___1
-0,956757	-1,50742	NaN	-0,740868	S	2	___1
-0,852606	-0,685314	-1,96603	-1,19972	S	2	___1
-0,891413	-2,03232	NaN	0,0600128	S	2	___1
0,301304	1,02051	-1,05467	0,122608	S	3	___1
0,667871	-0,676201	-2,25434	-1,41089	S	2	___1
-1,45415	-1,42649	NaN	-2,09458	S	3	___1
-0,652487	0,0861664	-0,791603	-1,2116	S	2	___1
NaN	NaN	NaN	NaN	S	2	___1
NaN	NaN	0,491154	-1,00813	S	4	___1
-0,53779	-0,200218	0,117119	NaN	S	3	___1
0,584974	0,839716	0,385441	2,82688	S	2	___1
-0,298651	-1,30421	NaN	-0,753252	S	2	___1
-0,537136	-2,46428	NaN	-1,38956	S	2	___1
1,83387	1,57636	0,328657	NaN	S	3	___1
-1,10389	-1,26711	-2,55131	-0,500805	S	2	___1
0,924793	0,205801	2,13415	-0,389282	S	2	___1
2,68854	2,48159	2,42232	3,01956	S	2	___1
1,19055	1,96189	1,21553	2,2826	S	2	___1
-1,14882	-1,15995	NaN	-1,2713	S	2	___1
NaN	-1,49463	NaN	-1,76818	S	3	___1
0,835894	0,896151	1,11349	1,07412	S	2	___1
-1,31214	-1,83687	NaN	NaN	S	2	___1
-0,448984	-0,215977	NaN	-0,756809	S	2	___1
-1,0728	NaN	-0,601418	-0,371962	S	2	___1

Polito et al. Universal ready-to-use immunotherapeutic approach for the treatment of cancer: expanded and activated polyclonal γδ memory T cells.
Phosphorylation sites

-2,22266	-2,91592	NaN	-1,57274	S	2	___1
-0,336212	-0,895529	-1,48674	1,0449	S	2	___1
NaN	NaN	NaN	NaN	S	2	___1
-1,85054	-2,2822	-1,44426	-1,8457	S	2	___1
NaN	-0,0951195	0,18758	2,35417	S	2	___1
NaN	NaN	NaN	NaN	S	2	___1
NaN	NaN	NaN	NaN	S	3	___1
-1,01249	-2,02083	-0,00557327	-1,41376	S	2	___1
1,57701	1,6274	-0,269775	0,926527	S	2	___1
0,694427	1,01684	-0,556707	0,330225	S	2	___1
0,170483	-0,368587	NaN	0,105227	S	2	___1
2,74957	1,4717	0,745159	1,12988	S	2	___1
NaN	0,0272903	NaN	1,05936	S	2	___1
2,98777	3,14922	3,70428	2,49417	S	2	___1
0,646694	1,07725	-1,74023	-0,257797	S	2	___1
NaN	NaN	NaN	NaN	S	3	___1
NaN	NaN	NaN	NaN	S	2	___1
NaN	0,341015	NaN	-1,24137	S	2	___1
0,0618191	-0,322649	0,799658	-0,628866	S	2	___1
3,15261	2,38958	0,469517	3,90833	S	2	___1
0,066227	-0,451769	-0,451054	-1,7244	S	3	___1
-2,46262	NaN	-2,33366	-0,798983	S	2	___1
0,158768	NaN	-0,444704	1,88521	S	2	___1
2,28576	NaN	NaN	2,20268	S	2	___1
0,521244	-0,914976	NaN	0,935127	S	2	___1
-0,0607147	-1,34402	NaN	1,30145	S	2	___1
NaN	-1,91084	-1,09676	1,31498	S	2	___1
5,7667	5,28762	1,05397	4,77447	S	2	___1
-0,331453	-0,991287	-2,22783	NaN	S	2	___1
-0,661448	-1,56526	-0,0794907	-2,49991	S	2	___1
0,695263	2,47955	4,51941	0,869204	S	3	___1
2,12341	1,0168	-1,55301	NaN	S	2	___1
0,264223	0,16	NaN	NaN	S	2	___1
NaN	NaN	NaN	NaN	S	2	___1

Polito et al. Universal ready-to-use immunotherapeutic approach for the treatment of cancer: expanded and activated polyclonal γδ memory T cells.
Phosphorylation sites

NaN	NaN	NaN	NaN	S	3	___1
NaN	NaN	NaN	NaN	S	3	___1
NaN	NaN	NaN	NaN	S	2	___1
NaN	NaN	NaN	NaN	S	3	___1
NaN	NaN	NaN	NaN	S	3	___1
0,094799	-0,550438	-0,772791	1,97273	S	2	___1
-1,47663	-2,28245	-1,23132	0,308504	S	2	___1
-1,34283	-1,51379	NaN	0,37533	S	2	___1
NaN	NaN	NaN	NaN	S	3	___1
2,13228	1,16176	2,33169	0,75988	S	2	___1
NaN	NaN	NaN	NaN	S	3	___1
-0,557848	-2,27826	-1,6214	-1,67495	S	2	___1
NaN	NaN	NaN	NaN	S	2	___1
0,840717	0,00601196	1,34513	-0,368298	S	2	___1
0,643148	0,479986	NaN	NaN	S	2	___1
-2,39227	NaN	-0,422606	NaN	S	2	___1
NaN	NaN	1,35208	NaN	S	2	___1
NaN	NaN	NaN	NaN	S	3	___1
NaN	NaN	NaN	NaN	S	2	___1
NaN	NaN	NaN	NaN	S	2	___1
-1,92069	NaN	NaN	NaN	S	2	___1
-0,478224	1,2749	2,81525	1,23712	S	2	___1
0,107527	1,79728	1,00761	-0,292406	S	2	___1
3,63614	3,94056	2,92302	3,15516	S	2	___1
0,409292	0,455765	-0,352493	0,0344868	S	2	___1
NaN	-0,248653	1,61102	0,609581	S	3	___1
-1,51029	NaN	NaN	NaN	S	3	___1
2,45991	2,25303	2,01374	1,10679	S	2	___1
2,29008	1,32722	NaN	0,450468	S	2	___1
NaN	-0,638901	0,00230217	-1,58478	S	3	___1
-0,667048	NaN	-2,60609	-0,935276	S	3	___1
-0,667048	NaN	-2,60609	-0,935276	S	3	___1
NaN	1,16869	0,954199	-0,429068	S	2	___1
1,47014	NaN	NaN	NaN	S	2	___1

2,57107	0,974691	NaN	1,91328	S	2	___1
-1,80419	-2,98517	NaN	-1,97474	S	2	___1
2,03182	2,35013	0,911283	-0,0371342	S	2	___1
NaN	NaN	NaN	NaN	S	2	___1
-0,588205	-1,95992	NaN	-2,50562	S	2	___1
-0,0307102	0,467707	-1,33589	0,214245	S	2	___1
NaN	NaN	NaN	NaN	S	2	___1
1,87743	1,0631	2,12618	1,15008	S	3	___1
NaN	NaN	NaN	NaN	S	2	___1
NaN	NaN	NaN	NaN	S	3	___1
0,155661	-0,427305	0,284071	-0,26091	S	3	___1
-0,996367	-0,974287	-2,57413	NaN	S	2	___1
-0,541971	0,525295	-0,468306	0,618702	S	3	___1
3,57122	2,6508	1,00356	2,46477	S	2	___1
1,70739	0,608976	-0,255398	-0,530882	S	2	___1
NaN	NaN	NaN	NaN	S	3	___1
NaN	NaN	NaN	NaN	S	2	___1
-0,390179	-0,719725	NaN	-2,25226	S	2	___1
0,649855	1,08037	0,743492	-1,09398	S	4	___1
0,683273	-0,405758	1,03422	-0,377703	S	2	___1
-0,53322	-0,990414	NaN	NaN	S	2	___1
0,713802	0,376654	0,329863	-0,217611	S	2	___1
1,3543	1,77255	0,864368	1,15986	S	3	___1
1,16521	1,01151	1,44494	3,3091	S	2	___1
1,78283	1,01654	0,575693	1,85055	S	2	___1
NaN	-1,82423	-2,04124	0,58844	S	2	___1
3,74937	3,52299	4,13952	5,40293	S	2	___1
-1,85405	-2,90248	-1,65158	-0,419855	S	2	___1
NaN	NaN	NaN	NaN	S	2	___1
NaN	NaN	NaN	NaN	S	2	___1
NaN	NaN	0,455719	NaN	S	2	___1
-0,53603	-0,547777	-0,580906	-1,51275	S	2	___1
0,922474	0,0918674	0,474115	-0,225697	S	3	___1
-1,62465	-1,65339	-2,53868	-1,07856	S	2	___1

NaN	-0,424759	-0,22385	-0,744671	S	3	___1
NaN	NaN	NaN	NaN	S	2	___1
-1,33529	-0,740089	NaN	NaN	S	3	___1
-1,41991	0,655046	NaN	NaN	S	2	___1
-1,65915	-0,702354	-1,21502	0,366144	S	2	___1
-0,683035	0,354059	0,0601273	-0,159943	S	2	___1
NaN	-1,84665	NaN	NaN	S	3	___1
NaN	-1,30355	NaN	-0,657179	S	3	___1
-2,19943	-2,09567	-0,163279	NaN	S	3	___1
-1,17267	-1,6939	0,445723	-0,98115	S	2	___1
2,42246	1,11885	2,07434	0,596611	S	3	___1
2,25596	0,32634	-0,271517	-0,397251	S	2	___1
NaN	NaN	1,08678	NaN	S	3	___1
NaN	-0,311506	NaN	-1,26144	S	4	___1
-0,161005	-0,371231	-0,282486	-1,67742	S	3	___1
1,05886	0,108063	NaN	NaN	S	2	___1
3,56333	3,30228	0,215672	1,31977	S	2	___1
1,08647	1,00412	NaN	NaN	S	3	___1
0,623581	NaN	0,556499	2,06637	S	2	___1
NaN	NaN	NaN	NaN	S	2	___1
0,571001	-0,304188	0,395737	NaN	S	2	___1
-0,612686	0,677588	NaN	-0,144066	S	2	___1
NaN	NaN	NaN	NaN	S	3	___1
-1,18349	NaN	-0,881487	NaN	S	2	___1
-0,395866	-0,887094	NaN	-1,23011	S	2	___1
NaN	NaN	NaN	NaN	S	2	___1
NaN	NaN	NaN	NaN	S	2	___1
NaN	NaN	NaN	NaN	S	2	___1
0,138672	0,902815	1,6227	0,346087	S	2	___1
2,26738	-1,16101	1,61421	NaN	S	3	___1
NaN	NaN	NaN	NaN	S	3	___1
0,0655327	-1,10624	-2,02212	-1,23441	S	2	___1
1,79096	1,15102	0,672283	1,09752	S	2	___1
-1,50743	NaN	-1,36251	NaN	S	2	___1

Polito et al. Universal ready-to-use immunotherapeutic approach for the treatment of cancer: expanded and activated polyclonal γδ memory T cells. Phosphorylation sites

-2,10414	-1,42262	-2,49486	NaN	S	2	___1
NaN	-1,44862	NaN	NaN	S	2	___1
-1,52707	-0,350403	NaN	NaN	S	2	___1
NaN	NaN	NaN	NaN	S	4	___1
0,836029	3,75794	2,4635	2,00863	S	2	___1
NaN	NaN	NaN	NaN	S	3	___1
NaN	NaN	NaN	NaN	S	2	___1
-0,349306	-0,708782	-0,92444	-0,654264	S	2	___1
NaN	NaN	NaN	NaN	S	2	___1
-0,533571	NaN	NaN	-0,0974579	S	3	___1
NaN	NaN	NaN	NaN	S	2	___1
NaN	NaN	NaN	NaN	S	2	___1
NaN	NaN	NaN	NaN	S	3	___1
NaN	NaN	NaN	NaN	S	2	___1
NaN	NaN	NaN	NaN	S	2	___1
3,13477	2,72249	0,580183	3,09525	S	2	___1
-0,874226	-1,62329	4,17229	-0,708927	S	2	___1
0,165548	-0,8899	1,0417	0,765953	S	2	___1
-2,13109	-1,81924	-1,30118	-1,91645	S	3	___1
0,75766	NaN	0,750978	-0,523455	S	2	___1
-1,76174	-2,33767	-1,75973	NaN	S	2	___1
NaN	NaN	NaN	NaN	S	2	___1
0,797077	-0,510481	0,225498	1,78007	S	2	___1
-0,111841	-4,48006	NaN	1,69313	S	2	___1
-1,76174	-1,93977	-2,31977	-2,33956	S	2	___1
-2,58324	-0,519796	NaN	-0,122892	S	2	___1
-1,47432	-2,23555	NaN	-2,38741	S	2	___1
2,56605	3,02167	2,21111	3,14678	S	2	___1
-0,850542	-0,486071	-1,37514	-1,30813	S	3	___1
-0,865742	0,134886	0,118555	-0,927368	S	2	___1
NaN	-1,64188	1,26343	-0,960171	S	2	___1
2,79985	1,79599	0,158264	1,2712	S	2	___1
-1,22541	NaN	-0,676802	-1,76181	S	2	___1
-0,99851	-1,61442	NaN	0,00339127	S	2	___1

NaN	-2,37264	NaN	NaN	S	3	___1
NaN	-0,647541	0,704033	-1,73583	S	3	___1
-0,173025	0,841747	-0,237534	NaN	S	3	___1
-2,14105	-2,2981	NaN	-0,758593	S	2	___1
NaN	-0,375944	-0,811153	0,203888	S	3	___1
1,73513	0,542727	-0,77494	1,28463	S	2	___1
NaN	NaN	NaN	NaN	S	2	___1
-0,319298	-1,10877	NaN	0,492001	S	2	___1
2,27356	1,86055	2,06074	1,49205	S	2	___1
1,35688	NaN	NaN	0,315899	S	2	___1
-0,956083	-2,23973	NaN	NaN	S	2	___1
-0,966202	-1,23237	NaN	-1,02072	S	2	___1
-1,3689	1,52366	0,456465	NaN	S	3	___1
NaN	NaN	NaN	NaN	S	2	___1
NaN	NaN	NaN	NaN	S	2	___1
4,06011	2,06402	2,47015	1,55694	S	2	___1
-2,33185	NaN	-2,53287	-3,1576	S	2	___1
NaN	-2,69056	-2,19208	NaN	S	2	___1
-1,68677	-2,11603	NaN	NaN	S	2	___1
NaN	-1,49594	NaN	-1,18324	S	3	___1
1,4655	0,308886	-2,40669	-1,61345	S	2	___1
-1,12535	-1,46361	NaN	-0,821077	S	2	___1
NaN	-0,599207	-0,145998	-0,779551	S	3	___1
NaN	NaN	NaN	NaN	S	3	___1
-1,43575	1,08102	0,123756	NaN	S	2	___1
NaN	-1,18003	-1,58897	-2,49912	S	2	___1
NaN	NaN	NaN	NaN	S	3	___1
-1,74562	-0,0480499	-1,79023	NaN	S	2	___1
0,533218	0,0375919	0,446644	-0,538109	S	2	___1
0,426151	-0,125969	0,853205	0,0936604	S	2	___1
-0,576689	NaN	NaN	NaN	S	2	___1
1,15412	0,536261	NaN	-0,276033	S	2	___1
NaN	NaN	NaN	NaN	S	4	___1
0,753351	0,607784	-2,87402	NaN	S	2	___1

0,441439	NaN	NaN	-0,469395	S	2	___1
-0,255867	-0,82737	-0,0417709	NaN	S	2	___1
NaN	0,0125637	NaN	NaN	S	3	___1
NaN	NaN	NaN	NaN	S	2	___1
-2,73142	-2,89192	-1,61483	NaN	S	2	___1
NaN	NaN	NaN	NaN	S	2	___1
-0,52482	-0,395054	NaN	0,22727	S	2	___1
0,205099	1,23069	1,89811	1,10516	S	3	___1
2,07891	0,763676	-1,10735	1,6866	S	2	___1
NaN	NaN	NaN	NaN	S	2	___1
2,70494	2,62327	-0,842319	1,69769	S	2	___1
NaN	NaN	-1,2155	NaN	S	2	___1
NaN	NaN	NaN	NaN	S	2	___1
-0,187077	0,247961	1,27819	0,295832	S	2	___1
0,517693	0,248472	1,9111	0,724165	S	3	___1
-0,275108	-1,57421	NaN	-0,143963	S	2	___1
-1,47086	-0,447422	-0,336918	NaN	S	3	___1
NaN	0,0924358	NaN	-0,234129	S	2	___1
NaN	-1,57516	-1,01053	-1,88246	S	3	___1
0,486847	0,529922	0,583744	-0,0315685	S	2	___1
-1,54778	-1,54984	NaN	-0,939987	S	2	___1
NaN	NaN	-0,55032	NaN	S	2	___1
NaN	NaN	NaN	NaN	S	2	___1
2,2777	3,35019	1,35781	2,37428	S	3	___1
0,303862	0,203302	-0,127039	0,80039	S	2	___1
NaN	NaN	NaN	NaN	S	2	___1
NaN	NaN	NaN	0,203827	S	2	___1
0,670334	0,213274	1,54651	NaN	S	2	___1
1,09867	1,81774	1,37711	NaN	S	2	___1
NaN	NaN	0,72629	NaN	S	3	___1
0,0135536	-0,441542	-0,793255	NaN	S	2	___1
-0,650419	-1,19832	NaN	-2,49119	S	2	___1
0,793783	0,465952	NaN	-0,0741882	S	2	___1
-0,800503	-0,345772	-2,9633	-1,3906	S	2	___1

0,682085	-0,116159	-2,34757	0,0615635	S	2	___1
0,131636	-1,50041	-0,220213	NaN	S	3	___1
-0,0856476	NaN	1,18125	-1,10406	S	2	___1
NaN	NaN	NaN	NaN	S	3	___1
NaN	-0,0155067	0,540937	-0,294762	S	2	___1
0,0343056	-1,49583	NaN	-0,0132256	S	3	___1
NaN	NaN	NaN	NaN	S	2	___1
NaN	NaN	NaN	NaN	S	2	___1
-2,32047	-0,688242	NaN	-1,62114	S	2	___1
NaN	-1,73309	-0,395748	-2,49067	S	3	___1
NaN	1,96523	NaN	NaN	S	3	___1
-2,36271	-0,883453	NaN	-1,32135	S	2	___1
-0,502216	1,22363	NaN	0,158501	S	2	___1
-0,769754	1,06335	1,87887	0,723598	S	2	___1
-1,64055	-0,448765	-0,760988	-0,0665874	S	2	___1
0,222784	1,63276	0,946491	0,967234	S	2	___1
-0,919907	-1,56775	NaN	-0,126572	S	2	___1
NaN	NaN	NaN	NaN	S	2	___1
4,28663	2,66581	0,139647	1,74947	S	2	___1
NaN	NaN	NaN	NaN	S	2	___1
-0,123684	1,11739	0,243952	2,35582	S	2	___1
NaN	NaN	NaN	NaN	S	2	___1
NaN	1,19836	NaN	NaN	S	3	___1
1,63973	1,30174	-2,77653	NaN	S	2	___1
-0,453152	-1,83476	NaN	-1,08322	S	2	___1
2,55442	1,75321	1,66801	0,0106182	S	2	___1
-1,32111	-1,15189	NaN	-1,44168	S	2	___1
NaN	NaN	NaN	NaN	S	3	___1
-0,718113	0,721367	NaN	NaN	S	3	___1
NaN	NaN	NaN	NaN	S	3	___1
-0,255701	NaN	NaN	-2,23462	S	2	___1
-2,17898	-0,438047	-0,729467	-1,79361	S	2	___1
1,37676	1,39964	1,16006	NaN	S	2	___1
-0,942369	-2,0731	-1,89194	0,283817	S	2	___1

Polito et al. Universal ready-to-use immunotherapeutic approach for the treatment of cancer: expanded and activated polyclonal γδ memory T cells.
Phosphorylation sites

0,0862083	0,158642	1,19138	0,490316	S	2	___1
-0,832661	-1,87531	0,109264	-0,461826	S	2	___1
-0,626099	-0,16164	0,364008	-0,592737	S	3	___1
-0,583111	-1,28389	NaN	-2,70992	S	2	___1
-2,12122	-2,57785	-1,92497	-0,204865	S	2	___1
-1,37096	-1,08481	-1,81352	-0,534712	S	2	___1
0,894323	0,423124	0,7113	1,75336	S	2	___1
0,293608	0,497057	0,845583	-0,828878	S	2	___1
NaN	-2,54125	-1,54091	-0,318518	S	3	___1
NaN	NaN	NaN	NaN	S	2	___1
NaN	NaN	NaN	NaN	S	3	___1
3,45644	1,93604	NaN	-0,156597	S	3	___1
-0,550318	-0,0401554	-0,560854	-1,30708	S	2	___1
NaN	NaN	NaN	NaN	S	2	___1
2,51441	2,15243	1,26198	0,330914	S	2	___1
0,647291	-1,86205	-0,777172	-1,2642	S	2	___1
NaN	NaN	NaN	NaN	S	2	___1
NaN	NaN	NaN	NaN	S	2	___1
0,439651	0,642429	-2,43397	-0,310595	S	2	___1
NaN	NaN	NaN	NaN	S	2	___1
-1,27284	-1,74951	NaN	NaN	S	2	___1
NaN	-0,927464	NaN	-0,743925	S	3	___1
-2,20093	-2,28959	NaN	-0,761766	S	2	___1
-2,94969	-2,98031	NaN	-2,28271	S	2	___1
-1,71652	-2,55776	-1,98203	-0,668081	S	3	___1
4,81686	5,0975	2,72393	6,3296	S	2	___1
-0,993328	-0,522842	2,50435	1,75399	S	2	___1
-0,320421	-3,80567	-1,23361	NaN	S	2	___1
0,899609	0,337481	NaN	2,95257	S	2	___1
-1,182	-1,46543	-0,806911	-1,96812	S	2	___1
1,69941	0,667984	NaN	NaN	S	2	___1
-1,43613	-0,827908	NaN	-0,893379	S	2	___1
0,90254	0,715424	1,34064	1,21428	S	2	___1
0,134668	0,950344	1,86469	2,52119	S	4	___1

NaN	NaN	NaN	NaN	S	2	___1
3,09084	2,57435	2,19448	2,18352	S	2	___1
-0,837976	3,76264	2,52653	4,24747	S	4	___1
NaN	NaN	NaN	NaN	S	2	___1
NaN	-0,66799	-1,67012	-0,775976	S	3	___1
0,603882	0,865486	0,91017	0,0582142	S	2	___1
8,01406	6,7375	0,913137	0,511505	S	2	___1
-0,80558	-1,55921	-2,91832	-1,95926	S	3	___1
NaN	NaN	NaN	NaN	S	2	___1
NaN	NaN	NaN	NaN	S	2	___1
NaN	NaN	NaN	NaN	S	2	___1
NaN	NaN	NaN	NaN	S	2	___1
NaN	NaN	NaN	NaN	S	2	___1
NaN	NaN	NaN	NaN	S	2	___1
NaN	NaN	NaN	NaN	S	2	___1
-0,947306	-1,21503	NaN	0,922825	S	2	___1
-1,90556	-2,79416	NaN	-1,94783	S	2	___1
NaN	NaN	0,460835	NaN	S	2	___1
-0,597507	-0,921564	-0,9259	0,775564	S	2	___1
0,46961	-1,39987	NaN	1,38086	S	2	___1
NaN	-2,22155	0,0625134	1,95611	S	2	___1
-0,230848	-0,775459	-0,0939484	NaN	S	2	___1
0,24295	0,427979	-0,918314	NaN	S	2	___1
-0,549456	0,00993156	NaN	NaN	S	2	___1
NaN	NaN	NaN	NaN	S	2	___1
-0,628721	-0,980419	NaN	-0,890034	S	2	___1
-0,555706	-2,67489	NaN	-2,15871	S	2	___1
-0,825705	NaN	-3,02369	-1,69901	S	2	___1
0,426538	-0,79282	-2,00091	NaN	S	2	___1
NaN	-0,20388	-0,116934	-1,46231	S	2	___1
NaN	-1,08655	-1,04081	NaN	S	2	___1
NaN	NaN	-0,384737	NaN	S	2	___1
0,464371	1,04709	0,410067	0,474371	S	2	___1
-1,01936	-1,52718	NaN	-1,00964	S	2	___1

Polito et al. Universal ready-to-use immunotherapeutic approach for the treatment of cancer: expanded and activated polyclonal γδ memory T cells.
Phosphorylation sites

6,39393	6,25874	5,3782	5,83719	S	3	___1
-0,498541	0,328333	0,0157642	-2,29508	S	3	___1
NaN	1,14267	1,21623	-0,513491	S	3	___1
NaN	-0,0405464	1,26605	-0,797434	S	3	___1
-1,7681	NaN	NaN	NaN	S	2	___1
-1,70258	-0,776628	-2,71315	-0,571522	S	2	___1
NaN	NaN	NaN	NaN	S	3	___1
NaN	NaN	NaN	NaN	S	2	___1
-1,30253	-0,76306	NaN	-2,05812	S	2	___1
-0,420279	-0,365459	-0,156361	1,2599	S	2	___1
0,866848	-0,10096	1,83179	2,72335	S	2	___1
-1,78114	-1,29559	1,40093	-0,00577545	S	2	___1
NaN	NaN	NaN	NaN	S	3	___1
NaN	NaN	NaN	0,0320606	S	3	___1
-1,09394	-1,71808	-0,483683	-0,0233326	S	2	___1
NaN	NaN	NaN	NaN	S	2	___1
-0,1917	-1,68412	NaN	0,508083	S	2	___1
1,61358	1,7692	-0,355349	1,95393	S	3	___1
NaN	NaN	NaN	NaN	S	3	___1
-1,98165	-2,82171	NaN	-0,791462	S	2	___1
-0,453817	-0,973602	3,80753	NaN	S	2	___1
-3,25144	-3,29553	-1,38568	NaN	S	2	___1
2,98982	4,63652	3,94416	2,34152	S	2	___1
-1,2204	-1,96965	NaN	0,486393	S	2	___1
-1,45919	-2,53514	NaN	-1,62554	S	2	___1
-1,93984	0,943266	1,76049	3,08139	S	3	___1
2,57259	0,936312	1,81766	1,43489	S	2	___1
-0,993328	-0,522842	2,50435	1,75399	S	2	___1
-0,689558	-1,68653	-2,37218	0,08461	S	2	___1
0,77161	0,608032	0,953117	0,901915	S	3	___1
2,69374	0,6562	1,05139	1,78075	S	2	___1
NaN	0,977928	-0,634565	2,71174	S	3	___1
NaN	NaN	NaN	NaN	S	2	___1
1,70516	0,772533	-0,226871	-1,28061	S	2	___1

3,62388	3,18542	3,17359	1,75357	S	2	___1
-0,50802	-1,10251	-1,87289	-2,51523	S	2	___1
NaN	-1,62271	0,51425	NaN	S	2	___1
NaN	NaN	NaN	NaN	S	3	___1
2,40077	2,98757	3,30674	2,91846	S	3	___1
-0,551384	-1,7744	NaN	-1,99757	S	2	___1
-0,565979	-1,33656	0,56601	0,317968	S	2	___1
NaN	NaN	NaN	NaN	S	2	___1
-0,0149059	-0,753826	NaN	0,112764	S	2	___1
4,52465	3,70255	NaN	4,22146	S	3	___1
-1,32901	-2,04303	NaN	-0,8652	S	2	___1
-0,879953	-1,62317	NaN	0,396109	S	2	___1
-0,838163	-1,68697	-1,33789	-0,475018	S	3	___1
NaN	-0,0579567	-0,985756	0,238651	S	3	___1
-1,1883	-1,2457	-0,777929	0,0884037	S	2	___1
-0,255453	-0,901747	-3,9295	-1,65135	S	2	___1
NaN	NaN	NaN	NaN	S	2	___1
-1,47596	NaN	-2,4375	0,156673	S	2	___1
-1,8857	NaN	NaN	-2,76865	S	2	___1
-0,723597	3,02976	1,93692	NaN	S	4	___1
1,14423	1,77597	-1,12602	0,265753	S	2	___1
-1,42232	-2,50763	-0,413773	-1,87754	S	2	___1
-0,492144	0,0156422	-1,26364	0,406219	S	2	___1
-1,19876	NaN	-0,846163	0,0848083	S	2	___1
-1,52218	-2,21144	NaN	-0,109739	S	2	___1
-0,0503445	-0,554329	0,419685	-0,612335	S	2	___1
-0,818598	-0,554329	0,365616	-0,612335	S	2	___1
NaN	NaN	NaN	NaN	S	2	___1
-2,46919	-1,44362	NaN	-1,47021	S	2	___1
2,76958	2,83224	-3,96577	2,45984	S	2	___1
-0,757753	-1,38328	NaN	-1,02471	S	2	___1
-1,70258	-2,09749	-1,88496	NaN	S	3	___1
-0,317915	-0,44113	-0,592054	-0,545338	S	2	___1
6,17218	5,10672	5,08256	6,21646	S	2	___1

4,48395	3,04811	-0,460758	5,48817	S	2	___1
1,86398	0,0589218	0,0576687	0,839796	S	2	___1
NaN	-1,33866	-1,68434	-2,4359	S	3	___1
-0,795982	-0,0684891	NaN	-0,55195	S	2	___1
NaN	NaN	NaN	NaN	S	2	___1
1,34846	-0,476128	-0,936506	1,80268	S	2	___1
-1,87613	-3,07588	-1,80252	0,527277	S	2	___1
1,35701	0,908115	-2,6169	2,09451	S	2	___1
-0,0698509	NaN	-1,21033	NaN	S	2	___1
-1,28478	NaN	-2,23877	-0,221348	S	2	___1
NaN	NaN	NaN	NaN	S	2	___1
0,377399	2,50087	-3,01457	2,50713	S	2	___1
NaN	NaN	NaN	NaN	S	2	___1
NaN	-2,94761	-2,11565	-2,34728	S	2	___1
NaN	NaN	NaN	NaN	S	3	___1
NaN	NaN	NaN	NaN	S	2	___1
-1,73278	-1,79666	NaN	-3,37723	S	3	___1
0,511265	1,19223	0,503717	NaN	S	2	___1
1,06157	0,332674	1,38319	-1,30055	S	2	___1
-1,21283	NaN	-1,21049	NaN	S	2	___1
-0,712425	1,2168	3,00643	0,359404	S	3	___1
NaN	-1,75746	NaN	NaN	S	2	___1
2,69004	2,6032	1,97489	2,0815	S	2	___1
-1,43613	-1,80112	-1,91721	-1,29945	S	2	___1
NaN	NaN	NaN	NaN	S	2	___1
NaN	-1,00247	NaN	NaN	S	3	___1
NaN	NaN	NaN	NaN	S	2	___1
NaN	NaN	NaN	NaN	S	2	___1
4,9433	4,48731	2,10177	4,17501	S	2	___1
-0,852104	-1,6517	NaN	-2,00469	S	2	___1
2,97852	2,3645	1,53202	1,1414	S	2	___1
0,919765	NaN	NaN	0,496922	S	2	___1
0,50576	NaN	NaN	NaN	S	3	___1
NaN	-2,5647	-2,18921	0,00903702	S	2	___1

-1,25802	0,275085	-0,942532	-0,708775	S	3	___1
-0,863218	-0,245235	-2,84355	-0,582388	S	2	___1
NaN	NaN	NaN	NaN	S	2	___1
2,53303	2,63795	2,79155	1,59582	S	2	___1
0,717663	0,966724	1,99173	1,06631	S	2	___1
1,62991	2,46246	2,9107	1,40539	S	3	___1
1,10579	0,568459	-1,92038	NaN	S	3	___1
NaN	-1,19097	-0,968075	-1,23022	S	2	___1
NaN	-0,78157	0,0719986	-0,535492	S	2	___1
NaN	2,38292	1,24499	1,20842	S	3	___1
2,14581	0,834667	0,148535	0,698816	S	2	___1
NaN	1,07772	NaN	0,450451	S	3	___1
-0,458761	NaN	3,70269	3,05869	S	2	___1
3,2762	-0,0688057	0,0352917	0,115404	S	2	___1
NaN	NaN	NaN	NaN	S	3	___1
4,35261	3,39871	5,0027	3,66499	S	2	___1
NaN	0,900948	-2,55773	NaN	S	2	___1
0,432327	0,332914	NaN	-0,535595	S	2	___1
NaN	-1,72117	-1,81467	-1,78899	S	2	___1
-1,01887	0,368868	NaN	2,21625	S	2	___1
-0,540358	-0,0604038	-0,326286	-0,639738	S	3	___1
1,52004	2,32065	NaN	1,61001	S	3	___1
NaN	NaN	NaN	NaN	S	2	___1
NaN	NaN	NaN	NaN	S	2	___1
NaN	-0,951462	0,762548	-0,413166	S	3	___1
NaN	NaN	0,50919	NaN	S	4	___1
NaN	0,287186	NaN	NaN	S	3	___1
NaN	NaN	NaN	NaN	S	3	___1
NaN	NaN	NaN	NaN	S	3	___1
3,53099	1,71025	2,33599	0,925663	S	2	___1
0,133343	0,607536	0,124268	-0,721426	S	2	___1
NaN	NaN	NaN	NaN	S	2	___1
1,06373	1,10211	NaN	-1,11757	S	2	___1
-0,1527	0,507492	NaN	1,10723	S	2	___1

-1,99692	-1,10598	-1,97707	-0,611012	S	2	___1
NaN	NaN	NaN	NaN	S	3	___1
-1,15389	NaN	-3,34878	NaN	S	2	___1
NaN	-1,44294	0,785549	NaN	S	3	___1
0,657278	-0,909271	NaN	-3,76427	S	2	___1
1,96034	-0,331682	1,84182	-0,374662	S	2	___1
3,77804	5,25606	5,35553	3,90187	S	2	___1
NaN	1,94681	0,269337	NaN	S	3	___1
NaN	NaN	NaN	NaN	S	2	___1
5,18714	4,44944	1,77953	3,98474	S	2	___1
-0,219025	-0,929424	NaN	0,318964	S	2	___1
-0,335775	-0,0356426	-1,77255	-0,722275	S	2	___1
-1,75682	-1,78004	NaN	-1,39812	S	2	___1
NaN	NaN	NaN	NaN	S	2	___1
1,17929	1,02681	NaN	NaN	S	4	___1
1,62542	NaN	1,36451	NaN	S	2	___1
-0,228167	-0,740849	0,762274	NaN	S	2	___1
-1,37061	-1,85023	NaN	-1,16637	S	2	___1
2,53781	2,03382	2,15855	4,12259	S	2	___1
NaN	-1,17937	NaN	0,711775	S	2	___1
3,91151	3,27137	0,230089	0,370644	S	2	___1
0,147551	0,116056	NaN	NaN	S	2	___1
NaN	NaN	NaN	NaN	S	2	___1
2,46324	2,68727	2,32218	-0,125267	S	2	___1
NaN	-1,67797	-2,37013	NaN	S	3	___1
NaN	NaN	NaN	NaN	S	3	___1
0,112343	0,212555	NaN	NaN	S	2	___1
-2,17846	-1,50716	-0,737787	-1,4094	S	3	___1
-0,999065	-0,141853	0,626675	-1,04923	S	2	___1
NaN	NaN	NaN	NaN	S	2	___1
-1,38122	-2,11203	NaN	-2,24137	S	2	___1
NaN	-0,134014	0,4841	NaN	S	2	___1
-0,816523	-1,1157	-0,789549	NaN	S	2	___1
NaN	0,155582	0,44842	NaN	S	3	___1

-2,37887	-0,195436	NaN	-2,28021	S	3	___1
NaN	1,37031	2,00998	1,76588	S	3	___1
-1,92266	-2,07924	1,06914	NaN	S	2	___1
-1,02823	0,241373	NaN	-1,22381	S	3	___1
NaN	NaN	NaN	NaN	S	2	___1
NaN	-4,15617	-2,08857	NaN	S	2	___1
-2,09597	-2,28604	-1,95128	NaN	S	2	___1
NaN	NaN	NaN	NaN	S	2	___1
NaN	NaN	NaN	NaN	S	3	___1
NaN	NaN	NaN	NaN	S	2	___1
NaN	NaN	NaN	NaN	S	2	___1
0,737083	0,160814	0,564672	NaN	S	2	___1
NaN	NaN	NaN	NaN	S	3	___1
-1,00212	0,00510597	NaN	-1,07155	S	2	___1
-1,84356	NaN	-1,9467	NaN	S	3	___1
-1,38393	NaN	NaN	NaN	S	2	___1
NaN	-0,878708	-1,30359	-1,51095	S	2	___1
NaN	NaN	NaN	NaN	S	3	___1
0,969149	1,02536	NaN	0,141357	S	2	___1
-2,27459	-3,41689	NaN	-0,424345	S	2	___1
-0,225855	1,7722	-0,224422	NaN	S	4	___1
NaN	-0,00223732	NaN	0,629084	S	2	___1
-0,771053	-1,4162	-1,45993	1,30913	S	3	___1
NaN	NaN	NaN	NaN	S	2	___1
NaN	NaN	NaN	NaN	S	2	___1
-0,0503807	1,25086	NaN	-0,227478	S	2	___1
0,660656	0,380209	1,22572	0,488783	S	3	___1
0,387632	0,109257	1,64453	-0,0642605	S	3	___1
-0,2033	-1,18668	-2,54179	2,14881	S	2	___1
-1,6812	-0,529938	NaN	0,950361	S	2	___1
-1,13658	-1,71631	NaN	-0,517105	S	2	___1
-0,0240364	-1,34955	-1,17406	-0,0339165	S	2	___1
NaN	NaN	NaN	NaN	S	3	___1
NaN	NaN	NaN	NaN	S	2	___1

-1,3416	0,956333	-1,28918	NaN	S	3	___1
-2,40546	-2,80991	NaN	0,297205	S	2	___1
-1,30416	-0,365263	3,40144	NaN	S	2	___1
3,92198	1,88878	2,93293	2,00636	S	2	___1
NaN	NaN	NaN	NaN	S	2	___1
-0,352179	0,59173	NaN	0,149046	S	3	___1
NaN	0,548698	0,741266	0,160744	S	2	___1
NaN	NaN	NaN	NaN	S	2	___1
NaN	-2,75035	-3,2411	-0,732748	S	2	___1
-1,17784	-1,51801	NaN	-1,47424	S	2	___1
NaN	NaN	NaN	NaN	S	2	___1
NaN	NaN	NaN	NaN	S	2	___1
-1,82294	NaN	NaN	NaN	S	3	___1
0,785929	0,128244	NaN	NaN	S	2	___1
-1,49712	-1,35199	0,089735	-2,46768	S	2	___1
1,03799	0,666265	1,39235	0,46007	S	2	___1
-0,892698	-1,72809	-2,43813	-1,98518	S	2	___1
0,76157	-0,10495	1,08974	0,0910683	S	2	___1
-2,92353	NaN	-2,89653	-0,800287	S	2	___1
-2,45392	NaN	-1,96657	0,120886	S	2	___1
2,11815	4,21735	3,30085	3,81199	S	3	___1
-1,63162	-0,897928	-0,34219	-0,356613	S	2	___1
NaN	NaN	NaN	NaN	S	2	___1
NaN	0,705278	0,341995	0,298388	S	2	___1
2,52275	1,5117	0,329361	-0,087841	S	2	___1
NaN	-1,9451	-1,1352	NaN	S	2	___1
-0,666388	-0,110258	NaN	0,43041	S	3	___1
-0,172869	-0,385517	-0,830931	0,566891	S	2	___1
-1,02041	2,19865	0,75939	1,26078	S	2	___1
-1,84929	-1,29693	-3,59139	NaN	S	2	___1
NaN	-1,39147	-1,11585	-1,01204	S	3	___1
-0,699427	-1,74544	-1,09233	1,4241	S	2	___1
1,34096	-0,497904	0,634821	1,91979	S	2	___1
NaN	NaN	NaN	NaN	S	2	___1

Polito et al. Universal ready-to-use immunotherapeutic approach for the treatment of cancer: expanded and activated polyclonal γδ memory T cells.
Phosphorylation sites

-0,210018	0,313467	0,499493	-0,856478	S	2	___1
-1,44054	NaN	4,47436	0,944895	S	2	___1
-0,878361	-1,57498	NaN	-2,87225	S	2	___1
NaN	NaN	NaN	NaN	S	2	___1
0,0635452	0,112135	-0,285519	-0,266872	S	2	___1
NaN	-2,37029	NaN	-2,09348	S	2	___1
NaN	NaN	NaN	NaN	S	4	___1
-0,81098	-1,65612	-1,66369	NaN	S	2	___1
2,53368	2,01358	3,24945	0,940996	S	2	___1
-0,832722	-0,457273	NaN	-1,27623	S	2	___1
NaN	-1,37086	-1,16991	-1,47215	S	2	___1
-0,0363483	-0,380476	0,221811	-1,70396	S	2	___1
NaN	NaN	NaN	NaN	S	2	___1
1,74032	2,91567	2,58402	1,95623	S	2	___1
NaN	NaN	NaN	NaN	S	2	___1
NaN	NaN	-0,074482	-0,895906	S	2	___1
4,30863	3,74979	-1,08264	1,98905	S	2	___1
0,392735	0,602701	-0,432245	NaN	S	2	___1
NaN	-1,34348	-0,564911	NaN	S	2	___1
-1,86045	-2,48221	NaN	-1,27374	S	2	___1
-0,278801	0,0697231	-0,0801544	1,32604	S	3	___1
NaN	0,903828	NaN	0,153349	S	2	___1
NaN	1,62327	NaN	NaN	S	3	___1
0,341866	0,571165	0,958038	-0,704912	S	3	___1
0,29126	-0,370056	-1,07644	0,354446	S	2	___1
NaN	0,891256	0,772257	0,23332	S	2	___1
-0,0401554	-0,815451	0,0887737	-0,166237	S	2	___1
NaN	NaN	NaN	NaN	S	2	___1
NaN	-2,60692	NaN	-1,33435	S	2	___1
-1,01873	-1,33497	-1,79297	-0,280094	S	2	___1
1,72405	0,783274	0,400276	-1,36341	S	2	___1
1,26158	0,994722	NaN	NaN	S	2	___1
2,62413	4,76183	-2,54016	1,6293	S	3	___1
2,68326	2,81199	NaN	1,17758	S	2	___1

0,552742	0,852156	-1,1532	-0,566725	S	2	___1
3,09546	2,66891	NaN	-0,107265	S	2	___1
NaN	1,67846	NaN	-1,32511	S	2	___1
1,23424	1,03108	NaN	-1,08565	S	2	___1
-0,309774	-0,217211	NaN	-1,36854	S	2	___1
0,897657	0,415586	NaN	-1,36854	S	2	___1
1,83572	0,607435	-1,41362	0,707766	S	2	___1
4,24228	5,29059	1,70096	4,21605	S	3	___1
3,79883	1,31185	0,791479	-0,757998	S	2	___1
0,189598	-1,66016	2,01322	0,221655	S	3	___1
0,492695	-0,0729465	NaN	0,853601	S	2	___1
-0,541668	-0,152765	-1,04081	0,759741	S	3	___1
NaN	-4,05331	-3,42672	-2,27748	S	3	___1
1,0148	-0,760361	NaN	NaN	S	3	___1
-0,392315	-2,28327	-0,846373	-1,40102	S	2	___1
NaN	-1,13888	-1,94293	-0,746988	S	3	___1
NaN	0,307661	NaN	NaN	S	2	___1
-1,37078	-1,51962	2,20472	0,206835	S	2	___1
2,88749	3,00392	1,5203	2,41675	S	2	___1
NaN	NaN	NaN	NaN	S	2	___1
NaN	-1,99438	-1,28844	NaN	S	2	___1
-0,198442	-0,996813	NaN	0,300785	S	2	___1
-0,176779	-0,476759	0,301079	0,392527	S	2	___1
-0,986172	-2,95706	NaN	0,95121	S	2	___1
2,29489	2,3674	1,96836	2,89661	S	3	___1
-2,01936	-2,77023	NaN	-0,766182	S	2	___1
NaN	NaN	NaN	NaN	S	2	___1
1,96612	1,21976	0,223227	1,92035	S	2	___1
NaN	NaN	NaN	NaN	S	2	___1
NaN	NaN	NaN	NaN	S	2	___1
-1,81512	0,0846672	-0,425354	-0,505619	S	2	___1
0,221298	-0,540592	NaN	0,852695	S	3	___1
NaN	NaN	-0,541079	NaN	S	2	___1
NaN	NaN	NaN	NaN	S	2	___1

Polito et al. Universal ready-to-use immunotherapeutic approach for the treatment of cancer: expanded and activated polyclonal γδ memory T cells.
Phosphorylation sites

NaN	NaN	0,0976524	NaN	S	3	___1
NaN	NaN	NaN	NaN	S	3	___1
0,782286	0,898796	-1,46037	NaN	S	2	___1
NaN	NaN	1,61475	NaN	S	2	___1
NaN	NaN	NaN	NaN	S	3	___1
NaN	NaN	NaN	NaN	S	2	___1
NaN	NaN	NaN	NaN	S	4	___1
1,77748	0,748011	1,74113	0,741783	S	2	___1
NaN	-2,03698	-1,96367	-1,59904	S	2	___1
-1,96749	-1,23678	1,3199	-1,20516	S	2	___1
-2,0008	NaN	NaN	NaN	S	2	___1
NaN	2,61054	NaN	0,883621	S	4	___1
-2,25294	-1,53193	NaN	-2,38509	S	2	___1
NaN	NaN	NaN	NaN	S	2	___1
NaN	-1,31665	NaN	-0,414787	S	2	___1
NaN	NaN	NaN	NaN	S	2	___1
2,78172	2,3361	NaN	2,66172	S	2	___1
-2,09565	-2,55228	-0,143476	NaN	S	2	___1
1,89533	1,39602	0,0239582	NaN	S	2	___1
2,22094	1,33451	NaN	NaN	S	2	___1
-0,709589	-0,656235	NaN	-0,425224	S	2	___1
1,44465	-0,179811	NaN	-1,87926	S	2	___1
0,947268	-0,39147	NaN	NaN	S	2	___1
NaN	NaN	NaN	NaN	S	2	___1
-1,98919	-1,78514	NaN	-1,71468	S	2	___1
-0,651726	-0,688608	NaN	-0,341427	S	3	___1
NaN	NaN	NaN	NaN	S	3	___1
-1,27821	-1,91809	-1,39581	NaN	S	2	___1
-0,47562	0,577173	-0,230663	0,215313	S	2	___1
0,407412	-1,28783	NaN	-1,2894	S	2	___1
-1,28133	-1,70641	-3,21523	-1,198	S	2	___1
1,86131	2,25983	3,05571	2,65794	S	3	___1
NaN	NaN	NaN	NaN	S	3	___1
-2,02598	-1,56185	1,22003	-1,38938	S	2	___1

Polito et al. Universal ready-to-use immunotherapeutic approach for the treatment of cancer: expanded and activated polyclonal γδ memory T cells.
Phosphorylation sites

-2,01319	-1,68744	NaN	-1,94008	S	2	___1
0,608868	1,26903	1,10594	2,07968	S	2	___1
5,00135	4,40934	4,49711	3,97857	S	2	___1
NaN	NaN	3,18967	NaN	S	3	___1
NaN	0,234625	-0,258121	0,303616	S	3	___1
NaN	NaN	0,291716	-0,44537	S	2	___1
-1,264	-1,91141	NaN	-1,86077	S	2	___1
2,56207	1,56334	2,13368	-0,0701504	S	2	___1
-0,309818	NaN	4,54715	-0,461924	S	1	___1
-1,53057	-0,477499	NaN	-0,916008	S	2	___1
-1,82282	-1,06268	NaN	-1,72991	S	2	___1
NaN	NaN	NaN	NaN	S	2	___1
2,7711	1,4397	-0,647085	1,65265	S	3	___1
NaN	NaN	NaN	NaN	S	2	___1
1,18733	0,867149	1,83009	1,32586	S	2	___1
NaN	NaN	NaN	NaN	S	2	___1
1,15723	0,846308	2,37877	0,797697	S	2	___1
NaN	1,05101	NaN	1,71328	S	4	___1
0,31588	-0,0855179	NaN	-1,61732	S	2	___1
NaN	-0,500368	-1,77917	-0,205811	S	3	___1
NaN	2,37568	0,593372	0,555204	S	3	___1
-2,32042	-0,860703	0,137871	NaN	S	2	___1
NaN	NaN	0,154381	-1,59034	S	2	___1
0,520569	0,0795116	0,86245	NaN	S	2	___1
-0,236715	0,400793	NaN	-1,86887	S	2	___1
-0,119875	-0,262133	0,496428	-0,980457	S	2	___1
NaN	0,911222	2,29882	NaN	S	3	___1
2,82827	2,48809	2,96318	1,62132	S	2	___1
-0,628828	-1,44868	-1,12501	-0,0724869	S	2	___1
NaN	-0,870785	-1,32395	-1,28283	S	2	___1
NaN	NaN	NaN	NaN	S	2	___1
-0,215921	-1,50716	NaN	-1,67345	S	2	___1
NaN	NaN	NaN	NaN	S	3	___1
3,86984	2,89585	2,24916	1,6252	S	2	___1

Polito et al. Universal ready-to-use immunotherapeutic approach for the treatment of cancer: expanded and activated polyclonal γδ memory T cells.
Phosphorylation sites

-1,13292	NaN	NaN	NaN	S	2		___1
0,7334	1,10992	1,50242	0,189644	S	3		___1
-0,219265	-0,524914	NaN	-1,4302	S	2		___1
-0,273306	-1,57597	NaN	-0,531288	S	2		___1
NaN	-1,16218	-2,2435	-1,70411	S	3		___1
0,287006	0,184416	NaN	-0,637842	S	2		___1
-0,0729809	-0,310566	0,213083	0,547628	S	2		___1
0,80517	2,78497	1,7646	2,07651	S	3		___1
1,53386	0,978966	0,873291	2,98958	S	2		___1
NaN	NaN	-1,67331	-0,300375	S	2		___1
-1,70202	NaN	-2,69002	-1,93945	S	2		___1
NaN	0,490383	-0,937754	-0,645809	S	2		___1
-0,651453	1,97968	1,87399	1,36604	S	3		___1
NaN	0,509729	-0,716713	-0,39966	S	3		___1
2,64843	2,01377	1,30905	2,22033	S	3		___1
-2,55115	-0,929844	NaN	NaN	S	2		___1
NaN	NaN	NaN	NaN	S	2		___1
NaN	-0,359125	NaN	NaN	S	2		___1
NaN	NaN	NaN	NaN	S	2		___1
1,37516	1,31688	1,95792	0,933779	S	2		___1
NaN	-1,35905	0,982981	NaN	S	2		___1
-1,27368	0,0189362	NaN	NaN	S	2		___1
-0,530464	-0,0559845	1,5155	NaN	S	3		___1
-0,490536	-0,555777	1,4717	-0,358564	S	2		___1
1,71838	1,20139	-1,40768	0,846455	S	2		___1
0,163013	0,466829	NaN	0,732367	S	4		___1
0,45171	1,77599	2,16094	1,2397	S	3		___1
NaN	NaN	NaN	NaN	S	3		___1
0,646915	-0,0349445	0,53451	0,419731	S	2		___1
NaN	NaN	NaN	NaN	S	2		___1
4,55347	5,05684	2,85905	2,0815	S	3		___1
-1,97455	-1,96461	-0,395014	-1,62243	S	2		___1
NaN	NaN	NaN	NaN	S	2		___1
-1,99099	-0,929354	NaN	-1,02657	S	3		___1

NaN	NaN	NaN	NaN	S	2	___1
NaN	-1,07465	-1,55993	-1,42579	S	2	___1
1,72918	2,75092	0,75001	1,4873	S	2	___1
0,204046	NaN	-0,507935	-0,403311	S	2	___1
0,301641	-0,518276	-1,60128	-0,690325	S	2	___1
-1,2705	-3,23926	NaN	0,534637	S	3	___1
1,18799	0,677588	NaN	0,443026	S	2	___1
NaN	NaN	NaN	NaN	S	2	___1
NaN	-1,27383	NaN	-1,10059	S	2	___1
0,238756	-0,271984	0,0954113	-0,957848	S	2	___1
NaN	NaN	NaN	NaN	S	2	___1
1,01089	2,35903	2,75322	2,01392	S	2	___1
-1,93666	-0,48926	NaN	NaN	S	3	___1
-0,200592	0,420862	NaN	-1,3271	S	3	___1
NaN	NaN	NaN	NaN	S	2	___1
1,65971	1,1297	0,100586	2,34055	S	3	___1
-0,0426884	NaN	NaN	-0,779551	S	3	___1
NaN	NaN	NaN	NaN	S	2	___1
-1,36176	-2,1079	NaN	NaN	S	2	___1
1,75158	0,639805	NaN	1,28049	S	3	___1
-1,3238	-1,51109	-0,687593	-0,550339	S	3	___1
0,0926247	-0,607727	-0,276724	-0,205648	S	2	___1
NaN	NaN	NaN	NaN	S	2	___1
1,13372	1,32183	-0,981661	0,669764	S	2	___1
-2,59928	-4,25526	NaN	-3,55493	S	3	___1
0,71171	NaN	-1,60715	-0,996584	S	2	___1
0,936392	-0,0252705	1,09574	0,0599689	S	2	___1
NaN	NaN	NaN	NaN	S	2	___1
NaN	0,756784	-0,461464	-0,142565	S	3	___1
-0,436926	0,156128	-1,93571	-0,679028	S	2	___1
NaN	NaN	NaN	NaN	S	2	___1
1,28433	-0,00383377	NaN	NaN	S	2	___1
NaN	NaN	NaN	NaN	S	2	___1
0,3193	-0,149231	NaN	0,0909138	S	2	___1

-0,534674	0,60853	1,47606	0,545431	S	2	___1
0,423544	-1,13581	-1,87939	NaN	S	2	___1
NaN	NaN	NaN	NaN	S	2	___1
0,0975876	0,454714	NaN	-1,25701	S	2	___1
-0,782808	0,418539	NaN	NaN	S	2	___1
-0,325884	-0,112305	1,23682	-0,764828	S	3	___1
-0,0278473	-0,671366	NaN	-2,55212	S	3	___1
NaN	-1,00269	-0,27354	-0,0366783	S	2	___1
4,40592	2,16807	1,87183	2,96609	S	2	___1
NaN	-1,44399	-2,22097	1,33777	S	2	___1
NaN	NaN	NaN	NaN	S	2	___1
-0,567417	-0,792164	-0,201279	-0,731543	S	2	___1
0,463417	-0,00056839	-0,342869	-0,375633	S	3	___1
-1,53167	-3,02219	NaN	-2,22693	S	2	___1
-1,56326	-1,79474	NaN	NaN	S	2	___1
NaN	0,904152	NaN	0,905046	S	4	___1
-0,382481	0,0473061	-0,303617	-1,12205	S	2	___1
NaN	NaN	NaN	NaN	S	3	___1
NaN	NaN	NaN	NaN	S	2	___1
-1,55326	-0,347893	-0,751945	-2,79136	S	2	___1
NaN	NaN	NaN	NaN	S	4	___1
NaN	NaN	NaN	NaN	S	5	___1
6,83591	4,98583	3,8169	5,7816	S	3	___1
2,37476	1,31971	1,30688	0,133392	S	2	___1
NaN	NaN	NaN	0,133392	S	2	___1
-0,936323	-1,60727	NaN	-1,25987	S	2	___1
2,00548	0,966879	1,48432	0,750164	S	3	___1
-2,17972	0,309803	-0,109257	0,224384	S	2	___1
0,62079	-0,790588	NaN	-0,234516	S	2	___1
NaN	NaN	NaN	NaN	S	2	___1
NaN	NaN	NaN	NaN	S	3	___1
NaN	-0,502298	-1,35048	NaN	S	4	___1
5,48192	4,33216	5,70944	3,41552	S	2	___1
1,4905	0,498184	0,43297	-0,949591	S	3	___1

Polito et al. Universal ready-to-use immunotherapeutic approach for the treatment of cancer: expanded and activated polyclonal γδ memory T cells.
Phosphorylation sites

0,550236	0,283142	NaN	-0,918087	S	2	___1
1,13606	-2,08534	NaN	-2,04773	S	2	___1
5,74245	5,24709	4,63173	4,88839	S	2	___1
0,0135536	-1,61416	-2,46448	-0,907021	S	2	___1
-3,11536	NaN	-1,1001	-1,44288	S	2	___1
-1,97796	-1,26929	-2,78829	-1,40331	S	2	___1
-0,782331	-0,510157	-1,54967	-0,863068	S	3	___1
-0,387005	0,0363369	NaN	-1,65084	S	3	___1
-0,590656	0,597603	NaN	-0,798166	S	2	___1
-0,550165	NaN	NaN	NaN	S	2	___1
-1,60271	-2,59405	NaN	-2,08431	S	2	___1
NaN	2,2366	1,83616	1,07568	S	3	___1
-0,508858	-0,388193	-2,26225	-0,827755	S	2	___1
-0,526266	1,33982	1,34467	NaN	S	3	___1
0,133627	2,63417	3,4413	-1,05827	S	2	___1
1,83543	1,56562	-0,934147	0,392956	S	2	___1
2,21207	3,67373	1,64341	NaN	S	2	___1
-1,8468	0,583307	-1,11761	-1,95471	S	2	___1
NaN	NaN	NaN	NaN	S	2	___1
5,36413	4,49345	4,48625	3,04629	S	2	___1
-1,06054	0,507118	-0,866388	-2,75418	S	3	___1
2,32328	1,47113	-1,46769	0,0225449	S	3	___1
3,84254	3,91722	2,39992	0,244318	S	2	___1
NaN	NaN	NaN	NaN	S	2	___1
-2,06237	-1,865	NaN	-1,69695	S	2	___1
NaN	NaN	NaN	NaN	S	2	___1
NaN	NaN	NaN	NaN	S	2	___1
NaN	NaN	NaN	NaN	S	2	___1
NaN	NaN	NaN	NaN	S	3	___1
-0,739416	1,68539	1,33705	1,48596	S	3	___1
2,81179	1,03597	-2,2498	1,91756	S	2	___1
NaN	NaN	NaN	NaN	S	3	___1
2,14111	0,385324	1,78082	-0,126469	S	2	___1
-2,70665	-1,41535	-2,3516	-2,62434	S	2	___1

0,820908	2,25379	2,01299	1,49795	S	2	___1
-0,324972	-0,851511	-0,959133	-0,345648	S	2	___1
-0,881868	0,889742	0,0645847	-0,295164	S	3	___1
-1,97973	-2,72854	-4,28441	0,0217609	S	2	___1
-0,386415	-0,285315	NaN	1,40282	S	2	___1
2,10379	2,08957	2,32307	2,24988	S	2	___1
NaN	NaN	NaN	NaN	S	2	___1
NaN	2,11836	-0,248219	NaN	S	3	___1
0,47056	0,201128	NaN	-0,063942	S	3	___1
NaN	NaN	NaN	NaN	S	3	___1
NaN	NaN	NaN	NaN	S	2	___1
-1,0398	-1,4763	-1,1516	NaN	S	2	___1
-2,42383	-0,875923	-2,98737	NaN	S	2	___1
4,19069	3,23133	3,14858	3,51774	S	2	___1
2,06829	2,49092	1,63407	2,44473	S	3	___1
NaN	NaN	NaN	NaN	S	2	___1
-0,434114	-1,37679	NaN	0,32131	S	2	___1
2,77156	1,63747	-0,281439	2,01618	S	2	___1
-1,3278	NaN	NaN	-1,83121	S	2	___1
NaN	NaN	NaN	NaN	S	2	___1
0,500847	1,15419	-1,02576	0,185448	S	2	___1
0,423647	-0,282454	NaN	NaN	S	2	___1
NaN	NaN	NaN	NaN	S	2	___1
NaN	-2,80027	-0,505125	NaN	S	3	___1
0,16626	1,16997	0,628603	-0,0426979	S	2	___1
NaN	-0,562141	3,34087	0,421185	S	2	___1
0,550022	1,98734	3,27324	1,21388	S	3	___1
NaN	NaN	1,37864	0,849188	S	2	___1
-3,17244	1,02383	NaN	NaN	S	2	___1
-0,19729	NaN	-2,24031	-1,1883	S	2	___1
-1,28782	-0,326635	-0,471334	0,628569	S	2	___1
-1,00218	-1,10054	NaN	-1,74459	S	3	___1
NaN	NaN	NaN	NaN	S	2	___1
NaN	NaN	NaN	NaN	S	2	___1

Polito et al. Universal ready-to-use immunotherapeutic approach for the treatment of cancer: expanded and activated polyclonal γδ memory T cells.
Phosphorylation sites

NaN	NaN	NaN	NaN	S	2	___1
NaN	NaN	NaN	NaN	S	3	___1
NaN	NaN	NaN	NaN	S	2	___1
NaN	NaN	NaN	1,64294	S	2	___1
NaN	NaN	NaN	NaN	S	2	___1
-1,40121	-1,7444	NaN	-1,77722	S	2	___1
-0,211662	-0,849733	NaN	0,503382	S	2	___1
0,718401	1,48148	1,973	2,07462	S	3	___1
NaN	NaN	NaN	NaN	S	2	___1
4,38499	5,80338	3,33447	3,85388	S	2	___1
5,11209	5,36633	4,90331	4,32092	S	2	___1
1,08569	0,900137	1,0478	-0,0852547	S	2	___1
NaN	NaN	NaN	NaN	S	2	___1
NaN	NaN	NaN	NaN	S	2	___1
NaN	-0,747959	NaN	1,13842	S	3	___1
-1,92595	-2,69556	NaN	-1,70526	S	2	___1
NaN	-2,45548	-2,46731	-1,75207	S	2	___1
NaN	NaN	NaN	NaN	S	2	___1
NaN	NaN	NaN	NaN	S	2	___1
-1,30356	-1,94881	-1,722	NaN	S	2	___1
NaN	NaN	NaN	NaN	S	2	___1
-1,78353	-1,95438	-1,81984	0,73152	S	3	___1
NaN	NaN	NaN	NaN	S	2	___1
-0,931822	-0,848095	NaN	0,0153313	S	2	___1
0,678431	0,486492	-0,877634	-0,0718231	S	2	___1
NaN	0,905043	-0,315964	-0,626839	S	3	___1
NaN	NaN	NaN	NaN	S	3	___1
-1,74643	-1,89063	NaN	-1,90088	S	2	___1
NaN	NaN	NaN	NaN	S	2	___1
NaN	NaN	NaN	NaN	S	3	___1
NaN	NaN	-1,1561	NaN	S	2	___1
NaN	NaN	NaN	NaN	S	2	___1
0,210499	-0,912834	-0,529173	NaN	S	3	___1
2,82255	2,34681	0,844286	2,81903	S	3	___1

-0,581398	-1,49814	-0,0557327	-0,598011	S	2	___1
1,51495	2,04065	NaN	1,68237	S	3	___1
NaN	-0,606112	NaN	NaN	S	3	___1
NaN	-0,487665	0,555645	1,32043	S	3	___1
NaN	0,243614	0,143423	1,85542	S	3	___1
0,227171	0,795902	NaN	-0,866182	S	2	___1
NaN	-1,10137	NaN	0,226109	S	3	___1
-0,910629	-1,07574	-0,235861	-0,718801	S	2	___1
NaN	NaN	NaN	NaN	S	2	___1
NaN	NaN	NaN	NaN	S	4	___1
NaN	NaN	3,77578	NaN	S	4	___1
NaN	NaN	NaN	NaN	S	2	___1
NaN	NaN	NaN	NaN	S	3	___1
-4,58526	-2,67277	NaN	0,364544	S	2	___1
1,37476	0,380152	0,802464	3,65375	S	2	___1
0,804398	-1,13956	0,482836	1,06318	S	2	___1
NaN	NaN	NaN	NaN	S	3	___1
-1,58109	-2,76594	NaN	-2,77817	S	2	___1
NaN	NaN	NaN	NaN	S	2	___1
-3,85866	NaN	-1,45737	-2,16227	S	2	___1
-0,00319099	-0,838713	-0,306808	0,315918	S	2	___1
NaN	NaN	NaN	NaN	S	2	___1
-1,3639	NaN	0,200439	NaN	S	2	___1
-1,17119	-3,43358	NaN	-1,80208	S	2	___1
0,0490494	0,0134659	-1,76083	-0,869303	S	2	___1
-1,08524	-0,9391	-1,12481	-2,22989	S	2	___1
NaN	NaN	NaN	-1,16327	S	2	___1
NaN	0,953125	2,17327	0,857744	S	2	___1
-0,03578	0,847193	-0,14806	-0,639519	S	2	___1
1,92838	1,19389	1,67743	NaN	S	2	___1
NaN	NaN	NaN	NaN	S	3	___1
-0,974279	-1,83603	NaN	0,0791874	S	2	___1
1,43161	0,366283	-0,285915	-0,883968	S	2	___1
-0,297501	-2,35742	-2,16887	-3,14966	S	2	___1

Polito et al. Universal ready-to-use immunotherapeutic approach for the treatment of cancer: expanded and activated polyclonal γδ memory T cells.
Phosphorylation sites

NaN	NaN	NaN	NaN	S	2	___1
NaN	0,789295	-2,03236	-0,323698	S	3	___1
3,21483	2,31482	-0,35083	3,1097	S	2	___1
-1,51612	-1,52219	NaN	-2,59805	S	2	___1
0,89188	-0,261406	0,0261326	0,393312	S	2	___1
-2,46174	-3,65056	NaN	-1,89599	S	2	___1
NaN	NaN	NaN	NaN	S	4	___1
NaN	-0,704824	0,912519	NaN	S	3	___1
2,10677	2,20072	1,95804	3,57959	S	2	___1
NaN	NaN	NaN	NaN	S	2	___1
-3,78007	NaN	-2,83029	-1,04333	S	2	___1
-1,49654	-2,67112	NaN	-1,02533	S	2	___1
-1,47461	-1,83357	-0,617609	0,170456	S	3	___1
NaN	NaN	NaN	NaN	S	3	___1
NaN	-2,75209	-4,01252	-0,7251	S	2	___1
-0,790039	-0,135679	-0,869404	1,06631	S	2	___1
NaN	-2,1868	NaN	0,422602	S	2	___1
NaN	NaN	NaN	NaN	S	2	___1
1,86854	0,0142174	0,46438	3,04266	S	2	___1
-0,74556	-1,1226	-0,432056	1,34101	S	2	___1
1,01861	0,208164	1,4748	1,94081	S	2	___1
NaN	NaN	NaN	NaN	S	2	___1
NaN	NaN	NaN	NaN	S	2	___1
NaN	NaN	NaN	NaN	S	2	___1
NaN	NaN	6,56304	0,576065	S	3	___1
3,54162	3,32424	4,2149	3,52811	S	2	___1
0,892645	2,23193	2,94845	1,27246	S	3	___1
-0,208857	0,759251	-0,611588	0,16856	S	2	___1
0,709465	1,06459	-0,122953	0,710917	S	2	___1
-1,08774	-0,974064	0,207691	-0,302195	S	2	___1
NaN	NaN	NaN	NaN	S	3	___1
0,0625153	-0,87829	NaN	-0,423496	S	2	___1
-1,91637	-2,55055	NaN	-1,6748	S	2	___1
0,0464687	4,19617	1,85649	4,16734	S	2	___1

Polito et al. Universal ready-to-use immunotherapeutic approach for the treatment of cancer: expanded and activated polyclonal γδ memory T cells.
Phosphorylation sites

-1,03376	2,60569	NaN	4,61126	S	2	___1
NaN	0,853331	0,508356	0,834288	S	2	___1
-0,521732	1,06473	NaN	0,222799	S	3	___1
NaN	NaN	NaN	NaN	S	3	___1
NaN	-1,65087	-2,66088	2,08233	S	2	___1
0,211517	0,268053	1,85121	3,7619	S	2	___1
0,891264	1,70957	-1,59283	1,8012	S	2	___1
-1,96492	-0,870924	0,932901	-0,709158	S	2	___1
1,09543	-0,584471	-1,40829	-0,966063	S	3	___1
-0,545404	-2,15682	-3,32942	2,51731	S	2	___1
2,63617	1,756	2,77546	5,93489	S	2	___1
NaN	NaN	NaN	0,192638	S	3	___1
1,05536	0,46661	1,91153	0,821753	S	2	___1
1,1063	NaN	NaN	0,535379	S	3	___1
-1,92687	-0,873421	NaN	NaN	S	2	___1
NaN	NaN	NaN	NaN	S	3	___1
-0,656359	NaN	NaN	NaN	S	2	___1
0,166414	-1,69188	-2,4735	-1,30488	S	2	___1
0,699921	-1,34911	-2,44242	-0,453012	S	2	___1
4,79918	2,87564	4,5957	4,55696	S	2	___1
7,64431	6,34797	5,77543	6,7058	S	2	___1
4,79918	2,87564	4,5957	4,55696	S	2	___1
7,64431	6,34797	5,77543	6,7058	S	2	___1
NaN	-0,171679	-1,874	-0,190413	S	2	___1
1,09692	1,57672	0,854362	NaN	S	2	___1
1,57259	0,200996	-0,204762	NaN	S	2	___1
1,26529	-2,798	0,659491	-0,127134	S	2	___1
NaN	NaN	NaN	NaN	S	2	___1
-0,721539	-0,648849	NaN	1,29459	S	2	___1
0,685946	0,676926	1,23009	1,74325	S	2	___1
5,02389	5,46049	1,59601	4,92979	S	3	___1
4,27467	4,26299	NaN	6,15309	S	2	___1
0,372885	-1,47779	0,299246	-0,152191	S	2	___1
NaN	NaN	NaN	NaN	S	3	___1

5,05466	4,40398	6,04219	4,82079	S	3	___1
-1,05174	NaN	NaN	NaN	S	3	___1
NaN	NaN	NaN	NaN	S	3	___1
NaN	0,45499	NaN	NaN	S	2	___1
-0,992226	NaN	-0,29491	-1,43311	S	2	___1
2,03774	1,54621	-0,842403	1,30449	S	4	___1
-2,25261	NaN	0,198372	-3,20683	S	2	___1
-2,35514	-2,13668	0,734568	-2,82758	S	2	___1
NaN	NaN	NaN	NaN	S	3	___1
-1,74783	-1,84905	-0,314285	NaN	S	2	___1
-1,8612	-1,45657	-0,940475	NaN	S	2	___1
NaN	-1,13263	NaN	-0,0431805	S	3	___1
-0,224802	1,62364	0,341333	1,83218	S	2	___1
3,62067	3,77847	0,814821	2,78229	S	3	___1
2,13094	2,50073	0,938498	2,92249	S	2	___1
-0,405197	-1,28194	NaN	1,61407	S	2	___1
-1,55489	-3,32987	-1,34479	0,715347	S	2	___1
-0,593529	-1,89434	NaN	NaN	S	2	___1
NaN	-0,70804	NaN	0,391973	S	2	___1
NaN	NaN	-2,53206	NaN	S	2	___1
NaN	NaN	NaN	NaN	S	2	___1
3,75905	1,7708	-0,711939	2,93047	S	3	___1
0,546587	2,48944	-0,296711	2,59629	S	3	___1
0,345768	-0,0258102	NaN	-1,93085	S	3	___1
0,27503	-1,16706	-2,69953	-0,735397	S	2	___1
1,52257	NaN	NaN	NaN	S	2	___1
-1,23745	-3,74425	NaN	-1,57302	S	2	___1
1,95213	2,11452	3,31427	1,34018	S	2	___1
NaN	NaN	NaN	NaN	S	2	___1
-0,52054	-1,6042	-0,769573	-1,50868	S	2	___1
NaN	NaN	NaN	NaN	S	3	___1
NaN	NaN	3,09009	0,0179253	S	2	___1
-2,39138	-0,163591	0,292686	NaN	S	2	___1
-0,0370235	-0,00276947	0,585251	2,92997	S	2	___1

-0,258724	-2,32523	-0,575693	0,73645	S	2	___1
NaN	-1,65087	-2,66088	2,08233	S	3	___1
NaN	-2,06462	-2,70692	-0,179228	S	2	___1
-2,78567	-2,71419	-3,35887	-1,48462	S	2	___1
1,60882	-0,201437	NaN	NaN	S	2	___1
NaN	4,39601	NaN	NaN	S	2	___1
4,46406	4,17316	-0,921703	3,84571	S	2	___1
7,93316	7,71239	0,087635	8,21608	S	2	___1
-0,748003	-2,79043	NaN	0,314997	S	2	___1
0,684891	-1,05117	2,15907	0,205292	S	2	___1
-2,5802	-1,76026	-1,03804	-2,12012	S	2	___1
-0,446432	-0,0804596	1,81237	-0,678579	S	2	___1
0,458635	-0,347893	0,0411835	NaN	S	3	___1
-2,05856	-3,75608	NaN	-1,23028	S	2	___1
8,53769	8,29859	3,08365	8,61025	S	3	___1
8,84406	8,00731	5,4324	9,08695	S	3	___1
0,708427	-1,0855	NaN	1,26009	S	3	___1
0,378706	-0,444221	-0,604633	1,13778	S	2	___1
1,89085	3,05406	2,87072	5,26554	S	2	___1
0,140528	-0,560799	0,353098	1,17052	S	2	___1
-1,70495	3,02558	3,30279	2,80244	S	3	___1
NaN	-1,73572	-1,87307	-1,41782	S	3	___1
3,53986	4,75797	3,58987	3,9797	S	3	___1
1,76561	1,2569	-1,0181	0,778572	S	2	___1
1,71838	1,36522	-2,21113	1,01287	S	2	___1
NaN	NaN	NaN	NaN	S	3	___1
NaN	NaN	NaN	NaN	S	3	___1
1,96514	2,66187	2,72009	3,07231	S	2	___1
5,73186	7,12932	5,67182	6,0066	S	2	___1
-1,24926	-0,598173	-0,62966	NaN	S	4	___1
-2,7743	-3,43202	-4,48224	NaN	S	2	___1
-2,41232	0,179874	-0,862827	-1,29554	S	2	___1
1,62498	1,82544	1,27242	1,89456	S	2	___1
1,43917	3,01127	1,6891	0,435944	S	2	___1

Polito et al. Universal ready-to-use immunotherapeutic approach for the treatment of cancer: expanded and activated polyclonal γδ memory T cells.
Phosphorylation sites

NaN	0,840012	NaN	NaN	S	2	___1
1,67683	3,23045	2,07191	1,55288	S	2	___1
NaN	NaN	NaN	NaN	S	2	___1
NaN	NaN	NaN	NaN	S	2	___1
-0,665453	NaN	NaN	-1,32077	S	2	___1
0,88973	1,34571	NaN	0,362961	S	2	___1
NaN	NaN	NaN	NaN	S	2	___1
0,72208	2,21152	1,13166	NaN	S	2	___1
6,60142	7,14199	6,67819	5,37233	S	2	___1
-2,8077	NaN	1,08239	-2,67188	S	2	___1
NaN	-1,15886	1,38346	NaN	S	2	___1
NaN	NaN	NaN	NaN	S	2	___1
-0,637049	NaN	-1,05066	NaN	S	2	___1
0,131384	3,30711	1,6414	0,927759	S	3	___1
-2,26368	0,236685	NaN	NaN	S	3	___1
NaN	-0,550661	-1,55815	-1,97815	S	2	___1
NaN	NaN	NaN	NaN	S	2	___1
-0,537186	-2,01081	-1,37985	0,919485	S	2	___1
NaN	NaN	NaN	NaN	S	3	___1
NaN	NaN	NaN	NaN	S	2	___1
NaN	-0,795317	0,433331	NaN	S	3	___1
-0,347498	-1,72558	-1,70308	2,5723	S	2	___1
3,42772	2,72685	2,36469	6,15582	S	2	___1
0,907331	-2,39959	-0,871958	1,74248	S	2	___1
NaN	NaN	NaN	NaN	S	2	___1
1,90387	-1,62679	0,723757	-0,100117	S	2	___1
-2,35716	NaN	1,87596	-0,496735	S	2	___1
-1,43895	NaN	1,40849	-0,835258	S	2	___1
6,68252	5,67393	6,17684	6,15717	S	3	___1
NaN	NaN	NaN	-0,0371094	S	3	___1
-0,997957	-1,38573	-1,44502	-0,786242	S	2	___1
NaN	NaN	NaN	NaN	S	2	___1
NaN	-0,57744	-0,960175	-0,258217	S	3	___1
-0,916698	-0,451769	NaN	NaN	S	2	___1

1,67086	1,57336	1,24509	1,12517	S	2	___1
-2,26473	-4,36841	NaN	-2,23198	S	2	___1
0,981396	-0,0816631	1,92311	4,39014	S	2	___1
-2,3827	-2,37013	NaN	-1,20927	S	2	___1
-1,90763	-1,89139	NaN	-1,68991	S	2	___1
-1,86979	-1,34421	NaN	-0,295048	S	3	___1
NaN	-0,765247	NaN	NaN	S	2	___1
-0,425714	1,01163	-1,47479	-0,471863	S	2	___1
5,5723	5,92849	6,34563	6,83775	S	2	___1
NaN	0,0661793	-1,9725	-1,035	S	2	___1
-1,16418	-0,978252	-2,45475	-0,956711	S	2	___1
NaN	NaN	NaN	NaN	S	2	___1
NaN	NaN	1,03149	-4,40848	S	2	___1
-2,97316	-3,02768	7,66816	NaN	S	2	___1
3,6291	3,71962	3,45675	2,84678	S	2	___1
-1,88801	-2,35098	-0,813442	-1,35889	S	2	___1
-1,31154	-1,58955	-0,690184	NaN	S	2	___1
-1,37007	NaN	NaN	NaN	S	2	___1
NaN	0,209673	1,02201	NaN	S	4	___1
NaN	-0,398846	NaN	NaN	S	3	___1
-0,325493	NaN	-1,5168	NaN	S	3	___1
NaN	NaN	NaN	NaN	S	3	___1
0,785105	NaN	2,63486	1,50184	S	2	___1
NaN	0,058485	NaN	-0,227587	S	4	___1
NaN	NaN	NaN	NaN	S	2	___1
NaN	0,797298	1,54595	1,03396	S	3	___1
NaN	NaN	NaN	NaN	S	2	___1
NaN	NaN	NaN	NaN	S	2	___1
-0,316576	-1,79574	NaN	NaN	S	2	___1
NaN	NaN	NaN	NaN	S	3	___1
-1,14452	-0,873838	-2,90082	0,316803	S	3	___1
-0,502365	0,893419	-2,84946	NaN	S	2	___1
-0,954874	1,0168	2,29513	2,46617	S	2	___1
NaN	NaN	NaN	NaN	S	2	___1

Polito et al. Universal ready-to-use immunotherapeutic approach for the treatment of cancer: expanded and activated polyclonal γδ memory T cells.
Phosphorylation sites

1,948	2,31826	-0,845118	0,746538	S	2	___1
2,7579	2,62536	4,63735	1,08554	S	3	___1
3,00479	3,26192	-0,834827	2,22449	S	2	___1
1,16459	0,0937157	NaN	-1,75935	S	2	___1
1,06624	-0,479715	-1,23943	-0,582108	S	2	___1
1,32874	0,00699425	NaN	-0,202734	S	2	___1
-0,522181	-1,5279	NaN	-1,11854	S	2	___1
-0,649658	-0,251898	NaN	-0,70908	S	2	___1
-0,649658	-2,73908	NaN	-0,70908	S	2	___1
-1,07564	-0,990519	-1,12197	-1,23281	S	2	___1
3,57072	3,6399	2,81055	2,54948	S	2	___1
3,85368	2,71038	3,63905	2,75919	S	2	___1
-3,40849	-2,92516	NaN	-2,6573	S	2	___1
0,860909	1,36024	2,46826	1,63474	S	2	___1
-3,65068	-3,83111	NaN	-1,35296	S	2	___1
NaN	-0,151419	-2,66891	-0,771851	S	2	___1
-0,88238	-1,5776	NaN	0,0444698	S	2	___1
NaN	NaN	NaN	NaN	S	2	___1
NaN	-1,79109	NaN	NaN	S	3	___1
-0,174509	-0,701614	NaN	-0,677605	S	2	___1
0,87594	0,172159	-0,455292	0,182518	S	2	___1
1,46826	2,57791	-2,28464	NaN	S	3	___1
NaN	-0,0623817	NaN	NaN	S	3	___1
-1,18877	NaN	-3,14055	0,346676	S	2	___1
-0,675987	-0,926455	-3,42098	0,462757	S	2	___1
NaN	-1,42015	0,457481	1,38068	S	3	___1
-4,61263	NaN	-4,23914	-1,92177	S	2	___1
-0,860888	-1,34876	-2,23701	0,665827	S	2	___1
1,04571	1,072	1,09236	0,605661	S	4	___1
NaN	-1,69645	-0,0772285	1,40007	S	3	___1
0,616163	NaN	0,531389	-0,660692	S	2	___1
NaN	NaN	NaN	NaN	S	3	___1
2,82637	4,15991	2,44686	3,08192	S	2	___1
-1,12504	NaN	-0,50906	1,13363	S	2	___1

NaN	NaN	NaN	NaN	S	2	___1
NaN	NaN	NaN	NaN	S	2	___1
-1,06111	-1,29639	0,503914	-1,90237	S	2	___1
1,553	2,02833	4,72109	1,81817	S	2	___1
NaN	NaN	NaN	5,00481	S	2	___1
4,98252	2,48957	-1,36911	2,71827	S	3	___1
-0,780128	-0,932285	NaN	-1,12593	S	2	___1
NaN	NaN	NaN	NaN	S	2	___1
NaN	NaN	NaN	NaN	S	2	___1
NaN	NaN	NaN	NaN	S	3	___1
NaN	NaN	NaN	NaN	S	2	___1
-2,07621	NaN	-2,67564	-2,29554	S	2	___1
-2,33049	NaN	NaN	-0,490204	S	2	___1
-0,0586567	-1,30073	NaN	0,501017	S	2	___1
2,48494	3,11726	3,73375	3,69103	S	2	___1
NaN	NaN	NaN	NaN	S	2	___1
-2,22505	-2,31951	NaN	-2,7048	S	2	___1
-0,987064	0,473886	0,647268	-0,333052	S	3	___1
NaN	NaN	NaN	NaN	S	3	___1
NaN	NaN	NaN	NaN	S	2	___1
3,7787	4,25582	2,0295	4,07501	S	2	___1
NaN	NaN	-0,31666	NaN	S	3	___1
NaN	-2,18621	-1,57275	0,39472	S	2	___1
NaN	NaN	NaN	NaN	S	2	___1
NaN	NaN	NaN	NaN	S	2	___1
NaN	NaN	NaN	NaN	S	2	___1
1,88861	1,63683	2,63972	3,37365	S	2	___1
-1,10791	-1,62937	NaN	0,654249	S	3	___1
NaN	NaN	NaN	NaN	S	2	___1
-0,561831	-0,753698	-1,23597	-0,338284	S	2	___1
-0,158606	-1,38186	-1,99051	0,287123	S	2	___1
-0,531616	-1,34785	-2,00567	0,813513	S	2	___1
-1,74364	-1,37323	1,73973	NaN	S	2	___1
-2,68759	-2,42509	NaN	-0,8873	S	2	___1

-2,47735	NaN	0,54565	-0,72599	S	2	___1
1,44592	1,96261	0,600773	3,59677	S	2	___1
0,927273	3,19943	2,26977	1,45801	S	3	___1
1,67162	1,0418	2,28432	1,6569	S	3	___1
NaN	-2,42439	-0,46015	-1,31086	S	2	___1
-0,6147	0,378345	NaN	NaN	S	2	___1
3,22165	3,14212	-1,06087	1,76119	S	2	___1
0,968723	3,14212	NaN	1,76119	S	2	___1
3,74102	2,27768	0,427355	1,28539	S	2	___1
-0,9848	-0,164356	1,67816	0,802542	S	2	___1
-1,44497	-1,93312	3,09083	1,22434	S	2	___1
-1,50703	-2,36325	-0,32617	-0,359015	S	2	___1
-0,406895	-1,03414	-1,24394	NaN	S	2	___1
NaN	0,930439	-0,508432	NaN	S	2	___1
NaN	NaN	NaN	NaN	S	2	___1
NaN	NaN	NaN	NaN	S	2	___1
NaN	0,725956	NaN	NaN	S	3	___1
-0,610779	3,78128	0,284645	-1,23518	S	3	___1
NaN	NaN	NaN	NaN	S	3	___1
NaN	NaN	NaN	NaN	S	3	___1
NaN	0,405378	0,0779877	0,771589	S	2	___1
-0,72646	-1,74908	1,0814	-0,879436	S	2	___1
7,27368	7,17708	6,12889	5,7813	S	2	___1
5,53288	3,83075	5,68346	4,79436	S	2	___1
4,54806	1,97002	2,40989	NaN	S	2	___1
0,208282	0,441435	0,395029	1,05779	S	2	___1
2,14291	2,60108	1,51404	0,303064	S	3	___1
5,72487	4,31498	5,17526	1,97029	S	3	___1
-0,0804348	-0,0574837	-0,233614	NaN	S	2	___1
6,1889	5,98223	-1,53382	4,00694	S	3	___1
5,59053	5,5705	NaN	3,13834	S	2	___1
0,864168	0,301771	-0,0407162	NaN	S	2	___1
1,36605	1,30638	1,85153	0,853214	S	2	___1
NaN	NaN	NaN	NaN	S	2	___1

NaN	NaN	NaN	NaN	S	4	___1
0,0414944	-1,18558	NaN	-0,336508	S	2	___1
3,52637	0,249556	NaN	NaN	S	2	___1
NaN	NaN	NaN	1,28078	S	2	___1
NaN	NaN	NaN	NaN	S	2	___1
0,913471	-0,786007	0,994474	1,77351	S	2	___1
NaN	NaN	NaN	NaN	S	2	___1
0,199015	-0,0748615	-2,06734	-0,167368	S	2	___1
1,15742	NaN	NaN	-0,679741	S	3	___1
NaN	NaN	NaN	NaN	S	3	___1
NaN	-0,970896	-0,833292	-1,24858	S	4	___1
0,309912	0,945866	NaN	1,67901	S	2	___1
0,871838	1,07822	1,99091	1,36367	S	2	___1
0,954418	1,6686	2,18238	2,98648	S	2	___1
-1,55856	-2,15709	NaN	-0,646946	S	2	___1
NaN	-2,80254	1,56676	0,224827	S	3	___1
2,7983	1,16616	1,67401	1,102	S	2	___1
-1,66848	-2,70586	0,369602	-2,56233	S	2	___1
0,291855	-0,427406	0,0559464	0,465576	S	2	___1
0,82275	1,19954	-1,23635	2,53225	S	2	___1
-1,09631	0,133297	NaN	-0,336094	S	2	___1
NaN	NaN	-0,0221157	1,23762	S	2	___1
NaN	-0,470341	-1,83362	0,434195	S	2	___1
-1,3615	-1,70344	0,175781	-0,12394	S	2	___1
NaN	NaN	NaN	NaN	S	3	___1
NaN	NaN	1,3051	-2,46548	S	2	___1
-1,6974	0,307232	0,608196	-0,264675	S	3	___1
NaN	3,35271	NaN	NaN	S	4	___1
0,533697	0,269312	2,11526	0,49478	S	3	___1
NaN	-1,66294	NaN	-2,70986	S	2	___1
0,760527	0,238483	0,838181	1,47209	S	3	___1
0,778009	0,666983	1,46387	0,432163	S	2	___1
-0,331104	-1,57398	-0,99028	0,280022	S	2	___1
-0,562241	-0,753443	-2,15481	-0,767378	S	2	___1

Polito et al. Universal ready-to-use immunotherapeutic approach for the treatment of cancer: expanded and activated polyclonal γδ memory T cells.
Phosphorylation sites

-3,45413	-2,93093	-1,52234	0,401724	S	2	___1
-2,30148	-1,7646	-0,812664	1,29926	S	2	___1
-0,686264	-3,26228	-1,84119	-0,55092	S	3	___1
-1,77437	-1,41014	-0,198124	-2,57916	S	2	___1
1,74643	2,5401	1,28413	0,829023	S	2	___1
0,154043	NaN	-0,98019	-2,20916	S	2	___1
NaN	-1,31705	NaN	NaN	S	2	___1
3,43584	2,63308	2,94661	1,92369	S	2	___1
-0,80855	-1,23049	NaN	NaN	S	2	___1
0,39057	-0,264225	NaN	0,335915	S	2	___1
NaN	2,69443	2,38315	5,76205	S	3	___1
-1,72188	-1,40501	NaN	0,873556	S	3	___1
0,621353	1,93304	2,89178	2,46791	S	2	___1
1,81055	2,82231	0,79336	1,93874	S	3	___1
-1,04301	-0,534103	-1,81222	-1,26696	S	2	___1
1,55677	2,40952	NaN	-0,0875168	S	2	___1
2,14988	3,46205	3,47342	1,58047	S	3	___1
2,30964	2,17552	1,19015	1,33907	S	2	___1
3,23278	2,8222	0,0705376	1,58602	S	2	___1
1,29877	1,07228	2,92428	-0,320683	S	2	___1
-0,797548	0,471975	0,552568	-2,13475	S	3	___1
-2,42457	NaN	0,230288	NaN	S	5	___1
0,763184	-0,0363426	-1,89022	0,661598	S	2	___1
-0,919973	-1,37416	NaN	-0,227148	S	2	___1
1,98024	1,70284	2,7903	2,74504	S	2	___1
0,0311222	3,42548	7,5347	3,80029	S	3	___1
NaN	NaN	NaN	NaN	S	2	___1
0,831421	0,504183	NaN	2,41719	S	2	___1
-1,57839	NaN	-0,683214	1,01252	S	2	___1
0,0068779	0,291651	-0,863337	-1,1081	S	2	___1
-0,0367756	-0,504017	-0,319677	-1,00291	S	2	___1
0,905262	-0,512426	-0,289097	0,426685	S	2	___1
1,81253	1,93121	-0,167219	0,712347	S	2	___1
-1,54535	-2,14762	-2,25601	-1,07249	S	2	___1

Polito et al. Universal ready-to-use immunotherapeutic approach for the treatment of cancer: expanded and activated polyclonal γδ memory T cells.
Phosphorylation sites

NaN	NaN	NaN	NaN	S	3	___1
0,0535641	-0,564045	NaN	0,322172	S	2	___1
NaN	NaN	-0,54542	NaN	S	2	___1
1,48939	1,64587	1,68527	0,586273	S	2	___1
NaN	NaN	NaN	NaN	S	3	___1
-0,00138664	-0,26988	NaN	-0,177744	S	3	___1
-0,289984	-2,28279	NaN	-1,66667	S	2	___1
0,501925	0,242783	0,961269	0,721895	S	3	___1
0,70171	-2,25354	-0,575937	-1,49238	S	2	___1
NaN	NaN	NaN	NaN	S	2	___1
-1,66925	-2,9706	NaN	0,493916	S	2	___1
-0,743122	-0,365459	-0,735228	NaN	S	2	___1
NaN	NaN	NaN	NaN	S	2	___1
NaN	0,249939	0,831526	-0,0281963	S	3	___1
-2,28035	-4,84233	NaN	-1,58801	S	2	___1
1,92447	0,475523	NaN	1,52783	S	2	___1
1,01123	-0,41725	-0,39958	1,17747	S	2	___1
1,08846	2,38396	-0,119131	NaN	S	4	___1
-2,16386	-3,35044	-3,47515	-1,37941	S	2	___1
-1,38465	NaN	NaN	NaN	S	2	___1
-1,35297	-2,49109	NaN	NaN	S	2	___1
1,95034	NaN	NaN	NaN	S	2	___1
-1,90115	NaN	1,3213	NaN	S	2	___1
NaN	NaN	NaN	NaN	S	2	___1
-0,102846	NaN	0,799391	NaN	S	2	___1
-0,0972385	1,27104	-0,253315	0,571342	S	2	___1
0,673269	3,13212	-1,52669	1,12323	S	3	___1
-0,644775	0,322647	-0,824486	-0,819464	S	2	___1
NaN	NaN	NaN	NaN	S	2	___1
NaN	NaN	NaN	NaN	S	2	___1
NaN	NaN	NaN	NaN	S	2	___1
NaN	NaN	NaN	NaN	S	2	___1
NaN	NaN	NaN	NaN	S	2	___1
NaN	NaN	NaN	NaN	S	2	___1

NaN	NaN	NaN	NaN	S	2	___1
2,51101	1,8339	0,173967	3,71131	S	2	___1
-2,40892	-2,16339	-0,32836	0,251301	S	2	___1
4,40637	3,69425	0,929361	3,1217	S	3	___1
NaN	NaN	NaN	NaN	S	3	___1
0,390806	-0,0623817	NaN	1,35829	S	2	___1
NaN	-1,48561	-0,882988	0,296518	S	3	___1
-0,834513	-2,1448	-2,58596	-1,38631	S	2	___1
0,775887	-0,742496	NaN	NaN	S	2	___1
-0,834513	-2,1448	-2,58596	-1,38631	S	2	___1
NaN	NaN	NaN	NaN	S	2	___1
1,32832	-4,51776	0,624502	4,03899	S	2	___1
3,44293	3,78985	4,05103	3,38578	S	2	___1
1,09212	0,537252	-1,56991	1,1675	S	2	___1
2,2099	1,58931	0,288366	0,140995	S	2	___1
0,472586	-3,11029	-1,77685	1,01032	S	2	___1
NaN	-0,214745	-0,42351	-1,12246	S	2	___1
0,600573	0,794767	1,73576	1,51059	S	2	___1
-0,65325	-3,56959	NaN	-0,331577	S	2	___1
NaN	0,137438	NaN	NaN	S	2	___1
NaN	NaN	NaN	NaN	S	2	___1
-1,63172	-0,987526	0,14538	-1,32575	S	3	___1
-1,92371	-0,181101	-0,852915	NaN	S	3	___1
-2,75972	NaN	-2,87315	-2,25416	S	2	___1
-0,743587	-1,62063	NaN	NaN	S	2	___1
-1,8227	-2,17974	NaN	-1,29755	S	3	___1
-2,23663	NaN	-0,651026	0,090519	S	2	___1
-1,29589	0,569633	NaN	1,0962	S	3	___1
-1,78902	-1,45432	NaN	NaN	S	2	___1
3,93456	3,04021	1,63227	3,32749	S	2	___1
1,23163	1,78025	NaN	-0,204676	S	5	___1
NaN	NaN	NaN	NaN	S	2	___1
1,26265	3,22503	NaN	1,3223	S	2	___1
-0,336432	-1,33611	NaN	-1,07861	S	2	___1

NaN	-0,66378	NaN	NaN	S	3	___1
-0,624708	-0,475494	NaN	-0,0151653	S	2	___1
NaN	0,988701	0,64155	0,731943	S	2	___1
NaN	NaN	NaN	NaN	S	2	___1
5,65782	5,04306	1,0165	1,17436	S	2	___1
-1,05512	NaN	-1,67338	NaN	S	2	___1
NaN	NaN	2,01685	NaN	S	3	___1
NaN	NaN	NaN	NaN	S	2	___1
-1,27201	-1,83701	NaN	-2,20754	S	2	___1
NaN	NaN	NaN	NaN	S	2	___1
1,01468	-2,14879	-0,428263	-0,702356	S	2	___1
NaN	NaN	NaN	NaN	S	2	___1
NaN	-2,70048	-1,46435	-2,1128	S	3	___1
0,858236	-0,856443	0,832703	NaN	S	2	___1
0,332567	-0,381069	0,146727	-1,29801	S	2	___1
-0,341909	-1,05015	NaN	NaN	S	2	___1
NaN	NaN	NaN	NaN	S	2	___1
2,21528	1,54753	2,16831	0,811646	S	2	___1
-1,63883	-2,22134	NaN	-1,71138	S	2	___1
-1,43145	NaN	NaN	-2,42177	S	2	___1
2,34288	1,91607	2,20482	1,40442	S	2	___1
0,593794	-0,6633	0,613302	0,274843	S	2	___1
NaN	0,939833	NaN	NaN	S	2	___1
NaN	NaN	NaN	NaN	S	2	___1
NaN	1,0415	NaN	NaN	S	2	___1
NaN	NaN	NaN	NaN	S	3	___1
NaN	NaN	NaN	NaN	S	2	___1
-0,582125	-1,50936	-0,840027	1,67498	S	2	___1
-1,6928	-2,83945	-1,40459	-0,295652	S	2	___1
0,897936	-0,762802	NaN	0,52231	S	3	___1
4,94533	3,52641	-1,92745	5,36831	S	2	___1
-0,707722	-1,56335	-1,98212	-2,11209	S	1	___1
NaN	NaN	NaN	NaN	S	3	___1
0,151768	-0,54512	NaN	0,00287819	S	3	___1

Polito et al. Universal ready-to-use immunotherapeutic approach for the treatment of cancer: expanded and activated polyclonal γδ memory T cells.
Phosphorylation sites

1,75754	1,35207	1,28699	1,2011	S	3	___1
0,96303	0,883429	-0,588585	1,85684	S	2	___1
-0,101618	-0,751656	NaN	-1,80724	S	2	___1
1,74643	1,57976	2,83097	5,96768	S	2	___1
-2,91006	NaN	-2,87639	-0,748678	S	2	___1
NaN	NaN	NaN	NaN	S	2	___1
NaN	NaN	NaN	NaN	S	3	___1
2,66541	1,81653	1,56708	0,882097	S	1	___1
1,51253	0,703047	-3,54347	-1,78632	S	2	___1
-0,647865	1,18231	0,654339	0,733353	S	2	___1
-2,04515	-1,6196	-3,17733	-2,16542	S	2	___1
2,50467	2,44169	2,20264	3,02418	S	2	___1
-3,03606	NaN	-2,85912	-0,235838	S	2	___1
NaN	-1,5908	-2,33319	-0,45122	S	2	___1
-1,01221	0,521341	-0,250128	0,0671196	S	2	___1
NaN	NaN	NaN	NaN	S	3	___1
7,3859	7,80199	5,19503	6,96869	S	2	___1
4,6508	3,55475	5,24431	3,67412	S	2	___1
NaN	NaN	NaN	NaN	S	2	___1
-1,10478	-0,966089	-0,663284	0,516214	S	2	___1
-1,65609	NaN	-1,55308	-0,632603	S	2	___1
NaN	NaN	NaN	NaN	S	3	___1
NaN	-0,598976	NaN	NaN	S	2	___1
-0,846861	-0,263315	NaN	-0,861322	S	3	___1
NaN	NaN	NaN	NaN	S	2	___1
-0,660734	-1,44103	-0,983822	NaN	S	2	___1
-0,19606	NaN	-1,44122	NaN	S	2	___1
2,49536	3,22015	-0,904917	2,31752	S	3	___1
-0,991123	-0,698046	-0,780407	1,06542	S	2	___1
NaN	-3,65399	-2,15761	-0,330515	S	2	___1
-2,59983	-2,57568	-1,74475	0,566795	S	2	___1
-3,43737	-3,72829	-0,943918	NaN	S	2	___1
7,7101	7,65604	7,63798	6,86262	S	3	___1
2,39301	4,6342	-3,53922	1,70977	S	3	___1

4,49358	5,02425	2,41435	3,64822	S	4	___1
-0,482527	-1,10397	-0,110991	-1,21655	S	2	___1
-1,1154	-2,25372	NaN	NaN	S	2	___1
-1,38845	-1,4615	NaN	-1,99626	S	2	___1
0,668003	-0,694117	-0,0882969	2,23955	S	2	___1
0,409004	-1,07236	0,417543	2,11293	S	2	___1
-1,96208	-1,21993	NaN	-1,49839	S	2	___1
NaN	-1,3294	NaN	-0,772409	S	2	___1
-1,5892	0,986637	-1,26269	-0,703806	S	3	___1
NaN	NaN	NaN	NaN	S	2	___1
NaN	NaN	NaN	NaN	S	2	___1
NaN	NaN	4,02181	NaN	S	3	___1
-1,09261	-1,70475	NaN	-1,77321	S	3	___1
NaN	NaN	NaN	NaN	S	3	___1
-0,842878	NaN	NaN	NaN	S	2	___1
1,05	0,278776	1,37514	-0,650766	S	2	___1
NaN	NaN	NaN	NaN	S	2	___1
NaN	4,25404	NaN	4,594	S	2	___1
0,418732	NaN	NaN	-0,274899	S	2	___1
-0,785673	-1,15885	NaN	NaN	S	2	___1
NaN	NaN	-1,92356	NaN	S	2	___1
NaN	NaN	NaN	NaN	S	2	___1
-1,98878	-2,57925	-1,96657	0,352118	S	2	___1
0,401548	-2,29851	NaN	0,212688	S	3	___1
-2,14753	-2,06063	-1,99774	-1,74939	S	4	___1
-0,657234	-0,134346	1,06436	-0,048233	S	3	___1
NaN	NaN	-0,688719	-3,49309	S	2	___1
0,391098	-0,177153	0,173988	2,64851	S	2	___1
1,509	0,587297	1,97637	0,917625	S	3	___1
0,401548	-2,29851	NaN	0,212688	S	2	___1
0,401548	-2,29851	NaN	0,212688	S	2	___1
-0,259636	-0,437122	-0,0309582	-0,715567	S	2	___1
-2,26358	-2,73131	-1,56964	-0,825678	S	2	___1
-2,14753	-2,06063	-1,99774	-1,74939	S	3	___1

-0,657234	-0,134346	1,06436	-0,048233	S	3	___1
4,85615	6,53024	0,930216	5,13391	S	4	___1
-3,39743	-2,04537	NaN	-0,82464	S	3	___1
NaN	NaN	NaN	NaN	S	2	___1
NaN	NaN	NaN	NaN	S	2	___1
NaN	NaN	NaN	NaN	S	2	___1
-0,70993	-3,26804	-0,996119	1,07912	S	2	___1
-1,37905	NaN	-0,110739	1,49563	S	2	___1
3,60803	2,50033	3,91638	3,85026	S	2	___1
-3,93498	1,50653	0,0312424	-2,53872	S	2	___1
NaN	NaN	NaN	NaN	S	2	___1
0,286978	-0,528189	-1,33942	-0,439131	S	2	___1
-1,1936	-0,454674	-1,51321	-1,536	S	2	___1
0,811876	-0,0067234	-3,1957	-0,307257	S	2	___1
NaN	NaN	NaN	NaN	S	2	___1
0,622906	0,472467	-1,9054	NaN	S	2	___1
NaN	NaN	NaN	NaN	S	2	___1
1,4905	0,901152	NaN	NaN	S	2	___1
NaN	NaN	NaN	NaN	S	2	___1
NaN	NaN	NaN	NaN	S	2	___1
NaN	-2,41628	-0,724958	NaN	S	2	___1
NaN	NaN	NaN	NaN	S	2	___1
NaN	NaN	NaN	NaN	S	2	___1
0,403803	0,746656	0,826942	2,03568	S	2	___1
5,92551	7,00383	2,08557	4,96209	S	2	___1
-0,147163	0,903057	0,533722	-0,289713	S	2	___1
NaN	NaN	NaN	NaN	S	3	___1
NaN	NaN	-0,111618	-2,53125	S	2	___1
-1,97197	-1,40608	NaN	-1,7915	S	2	___1
NaN	-2,20297	-1,22369	NaN	S	3	___1
-0,642664	-2,01847	-0,473015	-0,501335	S	3	___1
NaN	NaN	NaN	NaN	S	2	___1
-0,597403	-0,56539	NaN	0,529339	S	2	___1
-2,06265	-1,34337	-1,66413	-0,626659	S	2	___1

-0,753016	NaN	-0,535332	0,284721	S	2	___1
-1,12739	-3,11718	-3,3648	-0,768055	S	3	___1
NaN	0,939674	-0,235229	0,283587	S	2	___1
NaN	NaN	NaN	NaN	S	2	___1
NaN	NaN	NaN	NaN	S	3	___1
-0,174431	0,0897331	NaN	NaN	S	2	___1
NaN	NaN	NaN	NaN	S	2	___1
1,32432	0,471975	1,78744	0,122847	S	2	___1
NaN	NaN	1,14856	NaN	S	4	___1
0,615892	-0,178011	2,95047	1,08719	S	2	___1
2,80184	2,09624	3,90687	3,78998	S	2	___1
2,60615	2,78837	3,49121	1,84548	S	2	___1
-0,771645	-0,923576	NaN	-2,33896	S	2	___1
1,18398	0,995672	4,9302	1,19323	S	2	___1
1,32127	1,32098	1,83994	1,48671	S	2	___1
5,03218	3,56336	4,4688	3,22156	S	2	___1
NaN	NaN	NaN	NaN	S	2	___1
NaN	NaN	NaN	NaN	S	2	___1
-1,46348	-2,35102	NaN	-0,745142	S	2	___1
2,97311	2,93274	-1,60545	3,24438	S	2	___1
-2,27313	-1,58097	NaN	-1,74577	S	2	___1
0,297222	-0,78105	-1,02339	-0,682859	S	2	___1
-0,0973129	-0,619907	-0,774183	0,105902	S	2	___1
NaN	-1,35125	-0,697865	1,4038	S	3	___1
-0,61401	-1,46491	NaN	0,0147057	S	2	___1
-3,21517	NaN	-3,58792	-0,946112	S	2	___1
0,2682	-0,687754	NaN	2,05356	S	2	___1
-2,52013	-1,88147	NaN	-0,526928	S	2	___1
1,29919	1,38718	1,19341	0,272421	S	2	___1
0,677176	0,383293	-0,622368	2,27241	S	2	___1
-0,7736	-1,33981	-2,77486	0,587038	S	2	___1
NaN	-0,0629349	-1,81582	1,16018	S	2	___1
3,13887	2,69584	-1,39924	-0,422083	S	2	___1
1,4384	1,23646	1,34174	1,01809	S	2	___1

Polito et al. Universal ready-to-use immunotherapeutic approach for the treatment of cancer: expanded and activated polyclonal γδ memory T cells.
Phosphorylation sites

NaN	NaN	NaN	NaN	S	2	___1
NaN	NaN	0,138821	-1,41288	S	3	___1
NaN	NaN	NaN	NaN	S	3	___1
-0,0364552	-2,75926	NaN	-0,320711	S	3	___1
NaN	NaN	0,522455	NaN	S	2	___1
2,36685	2,28386	-0,421669	2,43077	S	2	___1
-1,82343	-2,22477	-1,9919	-1,05964	S	2	___1
NaN	NaN	-0,167664	NaN	S	2	___1
NaN	NaN	NaN	NaN	S	2	___1
NaN	-2,1974	NaN	-1,37904	S	2	___1
NaN	NaN	-1,78557	NaN	S	2	___1
-0,852606	-0,242184	2,94552	-1,13336	S	2	___1
-0,0775452	-1,2043	NaN	1,08189	S	3	___1
0,949835	-1,16476	NaN	0,579124	S	2	___1
1,62711	4,1241	0,976845	2,95759	S	3	___1
2,0654	1,83026	0,709879	1,50292	S	2	___1
2,38036	2,62609	-0,320898	0,603504	S	2	___1
NaN	NaN	NaN	NaN	S	3	___1
-2,56845	-2,96597	3,15724	NaN	S	3	___1
NaN	NaN	NaN	NaN	S	3	___1
0,680033	1,14651	NaN	1,90454	S	2	___1
NaN	NaN	NaN	NaN	S	2	___1
-2,11437	-1,30488	NaN	-2,04395	S	3	___1
-0,1024	0,630777	NaN	0,448256	S	2	___1
NaN	NaN	NaN	NaN	S	2	___1
-1,0938	NaN	-0,579967	-0,522446	S	2	___1
-1,39902	-3,66924	-2,97782	-1,84343	S	2	___1
NaN	0,968391	0,117462	-0,0777187	S	3	___1
-0,000139236	-0,925734	-0,490793	2,58856	S	2	___1
-2,88683	-0,975016	-1,27413	0,429853	S	2	___1
-0,527964	-0,56842	NaN	0,529453	S	4	___1
0,322599	0,46842	NaN	-1,41613	S	4	___1
0,98152	1,39562	NaN	NaN	S	3	___1
3,19439	2,3355	2,05784	3,37365	S	3	___1

2,88495	1,98837	0,428566	1,48068	S	2	___1
2,85605	1,08263	2,31017	NaN	S	3	___1
-0,160694	0,0372219	1,87494	0,811779	S	4	___1
NaN	NaN	NaN	NaN	S	2	___1
NaN	NaN	NaN	NaN	S	2	___1
2,42104	2,18402	NaN	5,54442	S	2	___1
1,57072	1,49894	4,41669	0,886032	S	2	___1
NaN	NaN	NaN	NaN	S	2	___1
2,26882	0,801653	NaN	0,540462	S	2	___1
1,10794	0,865362	0,418711	0,778708	S	2	___1
NaN	NaN	0,560228	-2,90248	S	2	___1
0,919474	0,395329	1,45031	0,096096	S	2	___1
NaN	NaN	1,2645	NaN	S	3	___1
-0,744457	-2,32941	NaN	-1,46826	S	3	___1
-2,45201	-3,31916	-3,63694	NaN	S	2	___1
-0,760744	-0,547998	-2,57026	NaN	S	2	___1
5,48363	5,10562	4,72638	4,93485	S	2	___1
1,38342	-0,0156612	1,42661	0,488998	S	2	___1
2,05726	2,0099	3,15076	1,80005	S	2	___1
-0,531666	-1,04774	-1,45494	-1,39614	S	2	___1
NaN	0,0618305	NaN	-1,62337	S	3	___1
-0,219992	-0,279324	-1,91388	-1,31138	S	2	___1
0,285416	NaN	0,113874	0,1516	S	2	___1
-2,74553	NaN	-1,26225	-0,0484028	S	2	___1
-1,42046	NaN	-1,17574	NaN	S	2	___1
1,00204	3,46686	0,741405	2,81996	S	2	___1
1,19431	-2,08357	1,13346	0,163486	S	2	___1
-1,97851	NaN	-2,71512	-0,222385	S	2	___1
NaN	NaN	NaN	NaN	S	2	___1
NaN	-3,31147	-1,13463	0,556559	S	2	___1
NaN	NaN	NaN	NaN	S	2	___1
-1,65467	-2,14671	-2,63217	NaN	S	2	___1
0,551914	2,501	-0,733448	1,96069	S	3	___1
2,47264	3,34453	1,07091	3,32457	S	2	___1

-0,862776	-2,4235	-1,94446	-0,149252	S	2	___1
0,681135	0,299002	2,24656	1,65466	S	2	___1
-0,644016	-1,62184	NaN	1,19467	S	2	___1
-2,20116	NaN	1,52629	-0,0179844	S	2	___1
NaN	-2,10696	-0,436554	1,24149	S	3	___1
2,08554	1,99305	2,29209	2,06838	S	2	___1
NaN	NaN	NaN	NaN	S	2	___1
-0,598818	-0,513615	NaN	-0,531118	S	2	___1
1,43161	3,05177	NaN	3,31552	S	3	___1
0,624727	0,602753	2,46935	1,90035	S	2	___1
-0,287317	-1,72666	-1,35887	0,738136	S	2	___1
0,355389	-0,407969	-0,3368	0,338102	S	2	___1
0,355389	-1,56766	1,06392	1,12431	S	2	___1
1,69289	0,348118	-1,9188	2,88476	S	3	___1
-0,660898	0,299555	0,284607	-1,63861	S	2	___1
-1,27628	-1,96775	NaN	NaN	S	2	___1
3,79579	2,66975	5,15816	2,46888	S	2	___1
NaN	NaN	NaN	NaN	S	2	___1
NaN	2,47001	2,39421	1,45246	S	3	___1
1,57852	2,62781	1,89153	1,57498	S	3	___1
1,34315	0,991905	-0,132439	0,898024	S	2	___1
-3,37357	NaN	1,06425	-1,67697	S	2	___1
NaN	NaN	NaN	NaN	S	2	___1
NaN	NaN	NaN	NaN	S	2	___1
NaN	-0,139681	-1,37314	-2,46134	S	3	___1
NaN	NaN	NaN	NaN	S	2	___1
2,10516	2,09247	0,892977	0,414135	S	2	___1
1,8674	-0,163761	-0,750927	-1,61438	S	2	___1
NaN	NaN	NaN	NaN	S	2	___1
NaN	NaN	NaN	NaN	S	3	___1
-0,598923	1,00895	NaN	-2,03605	S	2	___1
NaN	-1,5389	-2,82341	1,30164	S	2	___1
2,89603	2,78607	3,73977	4,16094	S	2	___1
2,68687	3,24483	0,543766	1,89689	S	3	___1

-1,20762	-1,40469	NaN	-0,00124168	S	2	___1
NaN	NaN	NaN	NaN	S	2	___1
0,24131	0,505251	2,20971	-0,866182	S	2	___1
-1,47933	0,0250587	0,383871	-1,53051	S	2	___1
0,120428	-1,14222	4,94373	-1,43634	S	2	___1
NaN	0,241436	2,19	-0,195044	S	3	___1
-2,03319	-2,89237	NaN	NaN	S	2	___1
NaN	NaN	NaN	NaN	S	2	___1
NaN	-0,122169	1,11864	-1,07569	S	2	___1
NaN	NaN	NaN	NaN	S	3	___1
0,468409	1,43772	NaN	-0,252457	S	3	___1
0,379932	-0,781179	NaN	0,310677	S	2	___1
-0,205856	0,669844	-2,66945	NaN	S	2	___1
-2,75506	-2,39987	-2,99691	NaN	S	2	___1
-2,10425	0,054987	-2,90442	NaN	S	3	___1
1,67975	1,60163	-0,5042	2,87145	S	3	___1
-1,60039	-1,62724	NaN	-0,942415	S	2	___1
1,70739	0,668985	-2,17002	0,935005	S	2	___1
0,726715	0,566515	NaN	-0,690172	S	2	___1
-0,711744	-0,963945	-1,21788	-1,65844	S	2	___1
-0,231499	NaN	0,160015	0,637617	S	2	___1
NaN	NaN	NaN	NaN	S	2	___1
1,64239	2,31228	1,05263	1,30316	S	3	___1
NaN	NaN	NaN	NaN	S	2	___1
3,13887	2,69584	-1,39924	-0,422083	S	2	___1
-0,0280933	-0,257233	NaN	-1,36969	S	2	___1
-0,112814	-1,46491	NaN	-0,798574	S	2	___1
NaN	NaN	NaN	NaN	S	2	___1
1,74446	1,27396	-1,28646	3,30423	S	2	___1
-2,02273	-3,52747	NaN	-1,4017	S	2	___1
1,50583	NaN	0,391344	0,550158	S	2	___1
NaN	-0,340292	1,03502	0,551277	S	2	___1
NaN	NaN	NaN	NaN	S	3	___1
2,22673	1,12425	-0,474825	0,493099	S	3	___1

-0,167839	-0,0666618	NaN	-1,24992	S	2	___1
4,40747	3,96906	4,18194	4,60212	S	2	___1
-0,395411	0,149584	-0,579792	NaN	S	2	___1
-2,02217	-0,180586	0,241573	-2,67517	S	3	___1
0,329069	0,748056	1,37604	-1,85313	S	3	___1
NaN	0,0541859	0,36227	-0,855759	S	2	___1
0,124788	0,562262	-1,24919	-0,855673	S	3	___1
-0,337044	-0,542578	-1,7407	-0,859919	S	2	___1
0,883772	0,341373	-1,86185	-0,097332	S	2	___1
-0,438616	-0,48724	NaN	-1,37594	S	2	___1
1,16843	1,13337	NaN	-0,300232	S	2	___1
-0,822208	0,11017	NaN	-1,77714	S	2	___1
-1,76975	-0,239855	NaN	-0,585794	S	4	___1
-1,06989	-0,213427	0,497911	NaN	S	3	___1
1,5101	1,37502	1,97732	0,724874	S	2	___1
1,98103	2,04089	NaN	NaN	S	2	___1
1,20584	1,92887	-0,446289	0,699999	S	3	___1
2,67802	2,45695	3,06088	1,67835	S	2	___1
1,14288	-0,0591412	-1,69244	-0,0777931	S	2	___1
2,46305	1,3334	-1,6567	0,171724	S	2	___1
-0,620495	-0,491495	NaN	NaN	S	2	___1
NaN	NaN	NaN	NaN	S	2	___1
2,29263	1,88847	NaN	1,85859	S	2	___1
-0,64234	-1,06063	NaN	-0,907942	S	2	___1
-0,33976	-0,122747	0,500793	0,106859	S	3	___1
-0,95837	NaN	-0,240774	-0,384995	S	3	___1
0,768541	0,9067	-3,07121	-0,0971813	S	2	___1
0,0429077	1,39022	NaN	-0,326105	S	2	___1
NaN	NaN	NaN	NaN	S	2	___1
NaN	-0,920704	NaN	-1,73668	S	3	___1
0,455708	NaN	1,95396	1,20608	S	2	___1
1,55772	0,881126	1,87361	2,74049	S	2	___1
NaN	-2,29148	-1,37447	NaN	S	2	___1
-0,405746	NaN	1,53395	-1,21508	S	2	___1

-0,135662	-1,88853	-0,67498	-1,6915	S	2	___1
0,876261	-0,170654	-0,788544	2,34632	S	2	___1
NaN	NaN	NaN	NaN	S	2	___1
2,41175	1,72364	0,293293	3,97442	S	2	___1
-1,68554	NaN	-2,06978	0,603365	S	2	___1
4,23731	3,74929	1,58542	3,17143	S	2	___1
1,0574	1,13599	NaN	0,853344	S	2	___1
-1,61444	-0,935225	-0,970808	-0,0690441	S	2	___1
-2,04444	-2,5731	-2,23767	NaN	S	3	___1
NaN	-1,87045	-0,430017	-0,320332	S	2	___1
0,271561	-0,0716705	0,483234	-1,44607	S	2	___1
0,888103	-0,487984	-0,391254	0,880062	S	2	___1
-2,32248	-1,42737	-1,53415	NaN	S	2	___1
NaN	NaN	0,374226	NaN	S	4	___1
-0,469898	-0,173729	-0,0330048	0,868818	S	2	___1
-0,752489	-1,49026	NaN	NaN	S	2	___1
NaN	NaN	NaN	NaN	S	3	___1
NaN	NaN	NaN	NaN	S	2	___1
0,806974	-0,211758	-1,78999	-0,874361	S	2	___1
0,421631	-0,0727863	-0,398691	-0,278561	S	2	___1
0,118036	-0,375059	0,559217	0,266026	S	2	___1
NaN	NaN	NaN	NaN	S	2	___1
NaN	NaN	NaN	NaN	S	2	___1
NaN	NaN	NaN	NaN	S	2	___1
NaN	NaN	NaN	NaN	S	2	___1
1,65785	2,17192	1,99972	0,924923	S	2	___1
0,363247	0,690937	-2,25501	0,343929	S	2	___1
NaN	NaN	NaN	NaN	S	3	___1
-1,49918	-1,48852	-1,30669	-2,88926	S	2	___1
NaN	NaN	NaN	NaN	S	2	___1
-1,93666	1,32649	NaN	0,665251	S	3	___1
4,22352	3,78931	4,8912	2,36239	S	2	___1
-2,3389	NaN	1,60223	-1,4127	S	3	___1
NaN	NaN	NaN	NaN	S	3	___1

Polito et al. Universal ready-to-use immunotherapeutic approach for the treatment of cancer: expanded and activated polyclonal γδ memory T cells.
Phosphorylation sites

-0,717545	1,21781	0,0145206	0,633289	S	2	___1
-0,704556	-1,11891	NaN	NaN	S	2	___1
NaN	NaN	NaN	NaN	S	2	___1
3,65823	3,23255	1,11069	2,90552	S	2	___1
-1,29342	1,53689	NaN	0,127829	S	3	___1
-0,381758	0,264965	NaN	0,110813	S	2	___1
1,43122	0,713438	-0,41728	2,92754	S	3	___1
-0,999273	-0,0880127	-0,389484	0,837175	S	2	___1
-1,66518	-1,35761	NaN	0,0840359	S	2	___1
-1,20338	0,104965	-0,166931	-0,445147	S	3	___1
-0,412649	-0,90984	-1,78718	1,98207	S	2	___1
2,40189	-0,470865	-1,2498	1,33888	S	3	___1
-1,91716	-1,52696	NaN	NaN	S	2	___1
-1,12724	-1,69047	-2,45054	NaN	S	2	___1
NaN	0,882936	-0,139292	0,383055	S	2	___1
0,481695	0,0574665	1,59701	0,93721	S	2	___1
2,36699	2,07827	1,61695	1,96951	S	2	___1
-0,923582	-0,779488	-0,466921	0,0695095	S	2	___1
-0,358828	0,897249	0,393524	1,86965	S	2	___1
-0,118521	-3,01543	NaN	NaN	S	2	___1
2,2927	0,171015	1,90452	0,121727	S	2	___1
NaN	-1,13283	2,31477	NaN	S	3	___1
-0,0966816	-0,658506	NaN	0,412518	S	2	___1
4,93811	6,38236	6,53596	5,4149	S	2	___1
NaN	NaN	0,0476837	NaN	S	2	___1
5,23521	4,39666	4,97462	3,01008	S	2	___1
4,91568	4,59381	2,70017	3,57853	S	2	___1
0,607525	-0,361559	-2,59761	-0,783657	S	2	___1
3,18173	2,05707	2,45444	2,1044	S	2	___1
NaN	NaN	1,39669	NaN	S	3	___1
NaN	NaN	-1,1072	NaN	S	2	___1
1,46838	0,438866	0,249386	0,946476	S	2	___1
-0,813231	-0,329395	NaN	0,397604	S	3	___1
0,929222	2,65442	3,63251	2,80029	S	2	___1

0,271475	0,500973	0,00545883	-1,07116	S	2	___1
0,209869	1,82552	-0,128897	-0,151047	S	3	___1
-0,498247	-2,80381	-1,98719	NaN	S	2	___1
-0,752024	-0,429039	NaN	NaN	S	2	___1
-2,0423	-2,54488	-2,83939	-3,03628	S	2	___1
-0,137146	0,289358	-0,0555401	-0,247583	S	2	___1
NaN	NaN	NaN	NaN	S	3	___1
-1,75214	0,750401	-0,0080986	-0,852327	S	2	___1
0,207142	0,161898	-1,72869	-1,08059	S	2	___1
-0,94824	-1,83517	NaN	-0,591326	S	2	___1
-0,745213	-1,81132	-1,59339	0,888691	S	2	___1
NaN	-1,30676	-2,09571	-2,20819	S	2	___1
NaN	1,78532	NaN	1,11569	S	4	___1
-1,70134	-0,957233	-0,851109	-0,872559	S	2	___1
NaN	NaN	NaN	NaN	S	2	___1
NaN	1,48156	2,70974	-0,36742	S	3	___1
3,3616	2,55836	4,68382	3,5893	S	3	___1
-0,360962	-1,03074	0,560986	0,86252	S	2	___1
1,9231	NaN	NaN	NaN	S	2	___1
-0,223709	0,0205822	NaN	NaN	S	2	___1
-0,227558	NaN	0,172709	-0,744787	S	3	___1
1,28689	2,78211	4,25471	1,85988	S	2	___1
NaN	NaN	NaN	NaN	S	2	___1
NaN	0,368868	-1,85974	-0,935768	S	3	___1
NaN	NaN	NaN	NaN	S	2	___1
NaN	-2,69763	-1,93321	-0,224382	S	3	___1
0,464371	-0,313013	0,441238	1,11514	S	2	___1
NaN	0,861654	-2,67756	0,588612	S	2	___1
1,79995	0,942242	-2,7739	1,17696	S	2	___1
1,33397	0,315357	-0,660486	-0,538654	S	2	___1
1,25297	1,17887	1,44417	2,20532	S	2	___1
NaN	1,13847	0,763235	1,46863	S	3	___1
0	1,01841	-1,64501	-0,795401	S	2	___1
-0,403225	-0,394655	NaN	-1,82904	S	2	___1

Polito et al. Universal ready-to-use immunotherapeutic approach for the treatment of cancer: expanded and activated polyclonal γδ memory T cells.
Phosphorylation sites

NaN	NaN	NaN	NaN	S	4	___1
2,10056	1,5235	0,82221	3,11623	S	2	___1
1,79066	1,6708	NaN	-1,74483	S	2	___1
NaN	NaN	NaN	NaN	S	2	___1
1,28959	0,77767	NaN	2,34632	S	2	___1
NaN	NaN	NaN	NaN	S	3	___1
-0,513351	-0,125721	NaN	-0,768814	S	3	___1
0,490797	2,20207	-1,1003	2,21822	S	2	___1
-0,339233	1,7412	-0,744986	NaN	S	3	___1
-1,40598	-1,38514	0,106825	-0,569433	S	2	___1
-0,921612	2,61302	NaN	0,24576	S	3	___1
NaN	NaN	NaN	NaN	S	2	___1
1,6225	0,398209	-0,8916	0,988287	S	3	___1
2,27262	0,769602	-0,275145	-0,406008	S	2	___1
0,0972958	-0,954205	-2,15813	NaN	S	2	___1
NaN	-0,998674	NaN	NaN	S	2	___1
2,22865	1,90044	NaN	1,76126	S	2	___1
-1,14559	-2,53044	NaN	NaN	S	2	___1
NaN	NaN	NaN	NaN	S	2	___1
NaN	NaN	1,0374	NaN	S	2	___1
3,08133	2,03971	1,0374	2,36039	S	2	___1
NaN	NaN	NaN	NaN	S	2	___1
NaN	NaN	NaN	NaN	S	3	___1
NaN	NaN	NaN	NaN	S	2	___1
NaN	NaN	NaN	NaN	S	2	___1
0,417616	0,201654	-0,879858	-0,209896	S	2	___1
NaN	NaN	NaN	NaN	S	2	___1
-1,64337	-3,3802	-2,91349	-2,38778	S	3	___1
NaN	NaN	NaN	NaN	S	3	___1
0,844679	0,448563	NaN	-0,412729	S	3	___1
-1,961	-2,71111	-2,33003	-0,142876	S	2	___1
-1,24605	-0,58777	-1,64931	1,08973	S	2	___1
0,180302	-0,621771	NaN	1,07601	S	2	___1
0,29092	NaN	0,957558	-0,253798	S	3	___1

3,29613	1,29564	2,39364	2,03859	S	2	___1
0,82725	2,19298	NaN	2,55889	S	3	___1
0,664116	0,0765667	1,92704	0,726999	S	2	___1
-0,887241	NaN	NaN	0,328957	S	2	___1
3,77511	3,24371	1,31074	2,53779	S	2	___1
NaN	-1,92134	NaN	-0,354664	S	3	___1
NaN	NaN	NaN	NaN	S	3	___1
NaN	NaN	NaN	NaN	S	3	___1
NaN	NaN	NaN	NaN	S	3	___1
NaN	-1,55317	-0,563087	-1,5742	S	3	___1
0,26203	-1,30118	NaN	1,18779	S	2	___1
6,34929	5,26984	6,88356	5,28538	S	2	___1
1,09937	0,579254	1,43894	NaN	S	2	___1
NaN	NaN	NaN	NaN	S	2	___1
0,446583	-0,539932	-0,245872	2,59791	S	2	___1
-4,30687	0,0703011	-0,666237	0,175898	S	3	___1
NaN	-1,68788	NaN	-0,508516	S	3	___1
NaN	NaN	NaN	NaN	S	3	___1
1,29905	1,6392	-0,0427284	1,62998	S	2	___1
NaN	NaN	NaN	NaN	S	2	___1
-3,66837	-2,86534	-1,19938	NaN	S	2	___1
-1,26516	-1,13094	2,06958	-0,416256	S	2	___1
NaN	0,98012	1,49203	NaN	S	3	___1
0,636076	1,44227	-0,974371	0,212465	S	3	___1
0,681545	-0,849188	NaN	-0,428185	S	2	___1
NaN	NaN	0,659977	NaN	S	3	___1
-1,44026	-2,51819	-2,40583	-0,45944	S	2	___1
1,08412	-0,666304	NaN	0,525291	S	2	___1
2,59942	1,598	NaN	5,16159	S	2	___1
NaN	NaN	-2,33202	-2,44187	S	2	___1
3,26515	2,35502	-0,527092	2,83888	S	2	___1
-2,31695	-0,554329	-1,61184	0,391367	S	2	___1
NaN	NaN	NaN	NaN	S	4	___1
2,60427	1,91326	1,55366	1,75614	S	2	___1

Polito et al. Universal ready-to-use immunotherapeutic approach for the treatment of cancer: expanded and activated polyclonal γδ memory T cells.
Phosphorylation sites

3,06362	2,44043	3,78589	2,11514	S	2	___1
NaN	NaN	NaN	NaN	S	3	___1
NaN	-2,30786	-1,50235	0,43854	S	3	___1
NaN	-1,78771	-0,193274	-0,912565	S	3	___1
-1,54556	-2,63904	NaN	-1,60608	S	2	___1
-1,56726	-1,68917	NaN	-1,92257	S	2	___1
3,54845	2,75047	3,44931	2,79162	S	2	___1
-1,49615	-1,5817	0,00988197	-1,58162	S	2	___1
1,07862	1,14438	0,462372	1,0517	S	2	___1
2,59381	2,62265	0,340635	2,66064	S	2	___1
NaN	-2,80413	-1,13663	-0,899397	S	2	___1
-1,41574	-2,48124	-1,50578	0,486677	S	2	___1
-0,662434	-1,48858	-2,04758	-1,22036	S	2	___1
-1,33477	1,10669	NaN	NaN	S	3	___1
NaN	-0,747068	NaN	-0,932634	S	2	___1
NaN	1,82836	3,03947	1,85276	S	3	___1
2,47638	1,69152	1,14813	1,1879	S	2	___1
-0,546316	-0,0795784	-0,401577	0,727848	S	3	___1
NaN	NaN	NaN	NaN	S	3	___1
0,31181	0,0958443	-0,379368	1,81165	S	2	___1
NaN	0,373907	NaN	0,665428	S	2	___1
NaN	0,133366	NaN	0,439955	S	3	___1
-1,82368	-0,465622	NaN	-1,98583	S	2	___1
-0,154125	-0,429243	-1,74226	-0,539301	S	2	___1
4,91809	5,35978	5,1666	6,44599	S	3	___1
-1,25711	-1,33736	1,28011	NaN	S	2	___1
NaN	0,367517	0,21513	-0,037159	S	3	___1
0,54808	2,1971	2,09813	1,02524	S	2	___1
0,0939884	-0,132767	NaN	-0,483475	S	2	___1
NaN	NaN	NaN	NaN	S	2	___1
NaN	-1,93968	-0,296425	-1,28728	S	2	___1
-0,543636	-2,44195	-0,324625	NaN	S	2	___1
-1,45691	-1,61725	NaN	-2,09849	S	2	___1
0,804953	-0,10218	NaN	0,259054	S	2	___1

-2,2463	-2,47305	NaN	-0,363287	S	2	___1
NaN	-0,8899	-1,61483	0,584024	S	3	___1
NaN	3,32809	0,635151	2,52445	S	3	___1
NaN	NaN	NaN	NaN	S	2	___1
NaN	0,598854	NaN	-1,00009	S	3	___1
-2,32001	-1,88673	0,531534	-1,6887	S	2	___1
NaN	NaN	NaN	NaN	S	2	___1
0,506712	-0,301851	NaN	NaN	S	2	___1
-1,70619	-2,07387	NaN	NaN	S	2	___1
NaN	NaN	NaN	NaN	S	2	___1
0,937025	1,54051	-0,509989	-0,561289	S	2	___1
3,4934	4,32068	3,67021	2,69487	S	2	___1
NaN	NaN	NaN	NaN	S	3	___1
NaN	-0,473915	1,76926	0,504686	S	3	___1
2,5248	3,06497	0,219074	3,94537	S	2	___1
NaN	-0,694483	NaN	1,23672	S	3	___1
NaN	NaN	NaN	NaN	S	2	___1
NaN	NaN	NaN	NaN	S	2	___1
NaN	NaN	NaN	NaN	S	2	___1
NaN	NaN	NaN	NaN	S	3	___1
NaN	0,275085	NaN	1,99512	S	3	___1
NaN	NaN	1,46176	1,47917	S	2	___1
NaN	NaN	NaN	NaN	S	3	___1
3,23742	NaN	NaN	NaN	S	3	___1
NaN	NaN	NaN	NaN	S	3	___1
NaN	-3,37799	0,258015	-0,700642	S	2	___1
-0,54677	-0,352047	0,61537	1,01924	S	2	___1
0,127487	0,0381832	NaN	NaN	S	2	___1
NaN	0,0536747	-0,680117	-0,300924	S	3	___1
-1,06046	-0,829927	-1,2072	-1,98027	S	1	___1
1,85873	1,46441	-0,357405	2,98775	S	2	___1
0,341919	-0,509617	-4,53436	-1,35434	S	2	___1
-0,617781	0,0261002	1,66324	NaN	S	2	___1
-1,61922	-0,239407	NaN	-1,47854	S	3	___1

Polito et al. Universal ready-to-use immunotherapeutic approach for the treatment of cancer: expanded and activated polyclonal γδ memory T cells.
Phosphorylation sites

NaN	NaN	NaN	NaN	S	2	___1
-0,241253	-0,0479698	-2,07713	-0,771051	S	2	___1
NaN	NaN	1,74551	1,33489	S	2	___1
-2,28321	NaN	-2,61747	-0,371778	S	2	___1
1,12704	-0,334259	0,917458	2,77611	S	3	___1
-0,368725	-0,18325	-1,08397	2,56171	S	3	___1
1,78172	3,22624	2,56547	3,85508	S	2	___1
NaN	NaN	NaN	NaN	S	2	___1
NaN	NaN	NaN	NaN	S	3	___1
-1,12519	-2,39221	-2,57289	-1,74939	S	2	___1
-0,569574	-0,809858	0,0756912	-0,358232	S	2	___1
-1,67698	0,0314503	0,308916	1,3926	S	2	___1
1,38964	-0,69866	NaN	-1,57044	S	2	___1
NaN	NaN	-2,0861	-1,00169	S	2	___1
NaN	NaN	NaN	NaN	S	2	___1
NaN	NaN	-0,00922203	-0,317467	S	2	___1
NaN	NaN	NaN	NaN	S	2	___1
NaN	NaN	NaN	NaN	S	2	___1
-0,527664	-1,7234	3,36366	-2,24825	S	3	___1
0,507906	-1,70517	-2,32151	-1,75649	S	2	___1
-1,08399	-0,173046	-1,55959	0,173969	S	2	___1
-1,88404	NaN	-2,35315	-1,52295	S	2	___1
-2,37963	-4,17919	NaN	-2,61173	S	2	___1
-0,227152	-1,49352	0,584412	2,56219	S	2	___1
-0,508463	-0,769634	-2,76345	1,79952	S	2	___1
4,00304	2,34061	3,5991	3,28039	S	2	___1
4,21424	3,68956	1,72115	3,30757	S	2	___1
3,82869	2,08025	-1,04191	4,82675	S	3	___1
6,40489	6,93303	1,14803	6,65858	S	4	___1
3,914	3,43357	1,12025	3,00098	S	2	___1
NaN	NaN	0,650192	NaN	S	2	___1
-0,530014	-1,33046	-0,20401	0,657846	S	2	___1
2,43974	1,79238	NaN	2,64451	S	2	___1
NaN	NaN	0,585823	1,57506	S	2	___1

1,27735	0,516369	NaN	4,4699	S	2	___1
-0,742079	NaN	-0,650953	-0,548796	S	2	___1
NaN	NaN	NaN	NaN	S	2	___1
NaN	-1,91852	NaN	NaN	S	3	___1
1,51617	-0,226048	-0,155636	-0,917158	S	3	___1
1,75631	0,0872383	NaN	1,95756	S	2	___1
-1,48001	-2,37362	-1,92012	NaN	S	2	___1
NaN	NaN	NaN	NaN	S	2	___1
-1,45519	-3,14192	NaN	-1,63941	S	2	___1
1,05911	1,64621	2,74925	1,68003	S	2	___1
NaN	NaN	NaN	NaN	S	2	___1
1,43558	2,76682	2,70607	3,11065	S	2	___1
-1,45329	-1,49733	-2,40706	0,183283	S	2	___1
-0,358561	-2,93712	NaN	-0,600922	S	2	___1
NaN	NaN	NaN	NaN	S	2	___1
NaN	NaN	NaN	NaN	S	2	___1
-1,7426	-2,92533	NaN	-0,338076	S	2	___1
-0,543434	NaN	-2,44597	0,233419	S	2	___1
-2,04758	-3,93382	NaN	-0,72019	S	3	___1
-0,441061	-1,61071	NaN	0,214325	S	2	___1
NaN	NaN	NaN	NaN	S	3	___1
3,26168	-0,397448	2,7555	4,64676	S	2	___1
1,41494	-0,023035	0,543129	-0,0324783	S	2	___1
-3,43842	-2,18974	NaN	NaN	S	2	___1
3,20337	2,12646	1,24244	1,69971	S	2	___1
-3,43842	-2,18974	NaN	NaN	S	2	___1
4,11633	2,95598	-2,51068	2,40486	S	2	___1
0,966562	-0,125887	NaN	-0,562429	S	2	___1
1,24853	-0,878986	NaN	-0,55171	S	2	___1
3,40588	2,26653	-1,98802	2,88644	S	2	___1
0,282484	-0,426592	0,901848	0,385818	S	2	___1
-0,748642	-1,64233	NaN	-1,3532	S	2	___1
1,50827	0,846476	0,727415	2,24436	S	2	___1
NaN	NaN	NaN	NaN	S	2	___1

-1,74794	-2,97972	NaN	-1,01787	S	2	___1
NaN	NaN	NaN	NaN	S	2	___1
NaN	NaN	NaN	NaN	S	2	___1
-2,41493	-1,69768	-2,53597	-2,45478	S	2	___1
NaN	1,32528	NaN	1,50894	S	3	___1
2,03883	1,16967	0,464718	0,100451	S	2	___1
NaN	NaN	NaN	NaN	S	2	___1
NaN	NaN	NaN	NaN	S	2	___1
-1,77615	-0,603115	-3,42395	-0,975519	S	3	___1
-0,677872	-2,38063	-1,01049	NaN	S	2	___1
-1,85379	-0,0168877	-2,27514	1,32099	S	2	___1
0,515003	0,310047	NaN	1,8306	S	2	___1
-0,310247	-1,07091	NaN	0,101105	S	2	___1
NaN	0,428541	0,297447	0,447758	S	3	___1
NaN	-1,91967	-2,08857	0,338417	S	2	___1
-0,405472	NaN	-0,192581	-0,15827	S	2	___1
-1,81293	-2,71913	-2,49932	-2,42403	S	2	___1
-2,12166	-3,17556	-2,65586	0,675598	S	2	___1
-0,8125	NaN	NaN	-3,41706	S	2	___1
-1,03121	-2,13159	-0,365747	1,24159	S	3	___1
0,082058	NaN	-1,94213	0,0483036	S	3	___1
NaN	0,398149	0,000535965	2,18846	S	2	___1
0,812902	1,0404	1,62904	2,90648	S	2	___1
0,794144	-0,622705	-0,432812	3,65064	S	2	___1
NaN	-0,0610371	NaN	NaN	S	2	___1
-1,10084	-0,21167	-1,28215	-1,93058	S	2	___1
1,23004	1,36337	0,635345	1,01473	S	2	___1
2,48389	1,64469	NaN	NaN	S	3	___1
NaN	-2,36939	NaN	-0,54657	S	3	___1
NaN	NaN	NaN	NaN	S	3	___1
NaN	NaN	NaN	NaN	S	2	___1
1,78031	0,310415	NaN	NaN	S	3	___1
-0,635218	-0,607843	0,302778	0,143883	S	3	___1
NaN	-0,0658684	NaN	-0,448563	S	2	___1

1,99821	2,26648	2,71609	2,30922	S	2	___1
0,312201	-0,816116	NaN	-0,221647	S	2	___1
NaN	NaN	NaN	NaN	S	2	___1
0,586174	1,16359	1,99208	1,1228	S	2	___1
-2,76125	-2,29467	-2,68652	-1,32622	S	2	___1
1,11326	0,58275	-1,81181	0,522213	S	2	___1
-0,622093	-0,686411	-0,318691	1,26877	S	2	___1
NaN	NaN	NaN	NaN	S	2	___1
-1,80395	-2,00639	-0,440687	NaN	S	2	___1
-0,864668	1,97249	NaN	2,5216	S	2	___1
-1,68766	NaN	-1,20866	-0,519253	S	2	___1
1,32915	1,88725	2,12629	0,467154	S	3	___1
-1,65271	0,315174	-2,69909	NaN	S	3	___1
NaN	-0,111977	NaN	2,20725	S	3	___1
NaN	0,216082	-0,34763	1,36549	S	3	___1
-0,830502	-1,42755	NaN	-3,61643	S	2	___1
-3,78986	-0,770151	-4,69381	NaN	S	3	___1
-1,07886	1,14613	NaN	0,538399	S	2	___1
0,261538	0,25013	-1,43637	-0,493895	S	3	___1
NaN	NaN	NaN	NaN	S	2	___1
-1,17408	-2,38986	NaN	-0,599464	S	2	___1
NaN	NaN	NaN	NaN	S	3	___1
4,74632	2,13587	0,401758	1,4185	S	4	___1
1,65807	1,06001	0,136515	0,692743	S	3	___1
-0,894501	0,151699	NaN	0,387163	S	3	___1
1,60359	NaN	-0,253286	NaN	S	2	___1
-0,464294	-1,06458	3,0472	NaN	S	2	___1
NaN	NaN	NaN	NaN	S	2	___1
NaN	NaN	NaN	NaN	S	2	___1
NaN	NaN	NaN	NaN	S	2	___1
NaN	NaN	NaN	NaN	S	2	___1
2,61308	2,23038	-1,35714	2,16154	S	2	___1
NaN	NaN	NaN	NaN	S	2	___1
1,85778	0,846392	2,46947	0,483078	S	2	___1

Polito et al. Universal ready-to-use immunotherapeutic approach for the treatment of cancer: expanded and activated polyclonal γδ memory T cells.
Phosphorylation sites

NaN	NaN	NaN	NaN	S	2	___1
NaN	2,06618	NaN	NaN	S	2	___1
1,55666	1,29872	NaN	1,86548	S	2	___1
NaN	NaN	NaN	NaN	S	3	___1
0,0661621	-0,722223	NaN	0,0564594	S	3	___1
4,29872	2,40323	4,16518	4,35943	S	2	___1
-0,565107	-1,30122	-1,1518	NaN	S	2	___1
-1,05491	NaN	-0,891125	0,78837	S	2	___1
3,93384	3,68167	3,62138	4,16538	S	2	___1
NaN	-2,55193	2,68678	0,419153	S	2	___1
2,25625	1,53244	2,56515	2,07401	S	2	___1
-0,346882	0,126162	-0,726885	-0,568287	S	2	___1
NaN	0,512281	-0,140497	0,0227985	S	3	___1
0,561125	0,552526	-2,18719	NaN	S	2	___1
0,748615	1,74064	1,66185	2,10554	S	3	___1
1,1235	1,52816	0,947094	1,4883	S	3	___1
0,134321	NaN	-1,56591	NaN	S	3	___1
NaN	NaN	NaN	NaN	S	2	___1
0,944839	1,63141	-3,48247	0,978096	S	2	___1
NaN	NaN	NaN	NaN	S	2	___1
4,32622	4,8142	-1,41722	3,20481	S	2	___1
0,359579	0,482267	1,29593	-0,173246	S	2	___1
NaN	NaN	NaN	NaN	S	3	___1
0,817884	0,251404	NaN	-0,986477	S	2	___1
-0,0977936	2,66438	NaN	-1,03591	S	4	___1
NaN	-3,14641	-2,2064	-1,00314	S	2	___1
NaN	NaN	0,500759	NaN	S	2	___1
NaN	NaN	NaN	NaN	S	2	___1
-0,859253	-0,780529	-0,0659542	NaN	S	2	___1
0,998562	NaN	NaN	0,940752	S	2	___1
-1,11713	NaN	-1,92267	-2,51684	S	2	___1
NaN	NaN	NaN	NaN	S	2	___1
NaN	NaN	NaN	NaN	S	2	___1
-0,656305	-0,44515	NaN	-1,35721	S	2	___1

NaN	NaN	-1,81746	NaN	S	2	___1
1,06017	0,454659	1,97139	1,42428	S	2	___1
NaN	-1,31345	NaN	NaN	S	3	___1
0,815228	1,27283	-2,0471	-0,442087	S	2	___1
1,35566	0,732357	-1,86118	1,01924	S	2	___1
-5,19058	-3,22651	-0,465057	-2,95466	S	2	___1
1,32376	-0,0463257	2,55746	-0,393124	S	2	___1
NaN	NaN	NaN	NaN	S	3	___1
NaN	NaN	NaN	NaN	S	2	___1
-0,697403	0,8258	NaN	-1,47476	S	3	___1
4,43204	3,82472	2,77505	2,47749	S	2	___1
NaN	-2,15536	-1,64953	-0,0282917	S	2	___1
2,42704	2,47656	3,61524	1,08266	S	2	___1
-0,263	NaN	0,749596	NaN	S	2	___1
2,51271	1,81449	2,0911	3,72156	S	2	___1
1,21676	-0,137011	NaN	1,72565	S	3	___1
-0,918139	-1,07397	NaN	0,46133	S	2	___1
-3,06163	-3,20601	NaN	-0,964643	S	2	___1
0,404379	-1,54095	NaN	-1,39997	S	2	___1
0,487835	-2,51329	NaN	NaN	S	3	___1
1,38408	0,58275	-0,0691242	0,892103	S	3	___1
4,50798	3,95128	0,0644073	3,38638	S	2	___1
NaN	NaN	NaN	NaN	S	2	___1
4,38262	2,02148	6,69606	3,60166	S	2	___1
NaN	NaN	-0,338978	NaN	S	2	___1
-0,343668	-1,39093	NaN	-1,26769	S	3	___1
0,44521	-0,510914	NaN	-1,4314	S	2	___1
1,80174	NaN	NaN	NaN	S	3	___1
2,34445	1,24397	-0,491611	-1,31347	S	2	___1
NaN	NaN	NaN	NaN	S	3	___1
NaN	NaN	-1,07399	NaN	S	2	___1
NaN	NaN	NaN	NaN	S	2	___1
0,405739	-1,33671	-0,836823	NaN	S	2	___1
0,196205	0,0859528	0,444183	0,0121059	S	3	___1

Polito et al. Universal ready-to-use immunotherapeutic approach for the treatment of cancer: expanded and activated polyclonal γδ memory T cells.
Phosphorylation sites

-0,260134	-0,735918	0,585932	-0,936754	S	2	___1
0,851166	0,3361	-4,06926	NaN	S	2	___1
0,160566	0,162237	0,587915	0,767056	S	2	___1
0,805923	0,436291	0,668013	-0,150969	S	3	___1
-2,08206	-1,71835	NaN	NaN	S	2	___1
NaN	NaN	NaN	NaN	S	3	___1
0,978586	-0,121922	-2,63168	-1,18675	S	2	___1
NaN	-0,562252	1,47798	0,203625	S	3	___1
NaN	-1,71581	NaN	NaN	S	2	___1
-0,387049	-0,817585	NaN	NaN	S	2	___1
NaN	NaN	0,0738583	-1,45484	S	2	___1
0,454847	-0,0389881	-0,667826	-1,10835	S	2	___1
1,44961	0,249493	0,416584	1,96448	S	2	___1
NaN	NaN	NaN	NaN	S	2	___1
-1,23917	-0,994471	NaN	-1,46347	S	2	___1
NaN	0,740953	NaN	NaN	S	4	___1
-1,51098	-2,24156	-2,70052	-2,43412	S	2	___1
NaN	-0,908844	-1,21356	-0,522142	S	3	___1
0,423519	-0,439896	-1,77813	1,32922	S	2	___1
0,446482	-1,44564	0,664095	NaN	S	2	___1
0,0497856	-0,410484	1,44912	-1,19773	S	2	___1
NaN	-1,92974	-2,30106	-2,25438	S	2	___1
NaN	1,80896	0,770893	0,642202	S	2	___1
-1,93281	-2,02641	NaN	-0,598259	S	3	___1
0,882231	1,17371	2,01137	-0,317642	S	2	___1
-0,12822	0,0677719	1,76008	-1,30321	S	2	___1
NaN	NaN	NaN	NaN	S	3	___1
NaN	NaN	NaN	NaN	S	2	___1
NaN	0,873907	1,3529	-0,518179	S	2	___1
5,32292	4,39936	2,38462	4,19087	S	2	___1
NaN	NaN	-0,137012	-1,3746	S	2	___1
3,49213	2,52687	2,00327	NaN	S	2	___1
NaN	-0,718353	0,0767956	0,196651	S	2	___1
2,878	2,44489	NaN	1,84058	S	2	___1

NaN	NaN	0,567677	NaN	S	2	___1
0,567545	-1,82775	-2,37447	-1,86614	S	2	___1
NaN	NaN	-1,67978	NaN	S	2	___1
1,16441	2,55926	NaN	0,26729	S	2	___1
NaN	-0,428223	NaN	NaN	S	2	___1
NaN	NaN	-0,605024	NaN	S	3	___1
3,78826	4,05287	3,28826	3,87971	S	2	___1
NaN	-2,82315	-0,792126	-0,973816	S	3	___1
-2,0305	-1,71858	0,0719986	0,530231	S	3	___1
-0,72291	-1,09227	0,227051	-0,512554	S	2	___1
0,135174	-0,493416	NaN	1,78606	S	3	___1
NaN	1,09293	-0,659603	NaN	S	3	___1
-0,835564	-2,13865	-2,60396	-1,78527	S	2	___1
4,96842	3,45826	3,46783	3,61463	S	3	___1
1,83785	2,83128	-3,79388	NaN	S	2	___1
-0,69982	-0,599436	0,275425	-1,29049	S	2	___1
1,73377	1,43065	2,33072	NaN	S	2	___1
0,660393	-1,45003	0,689314	-0,177029	S	2	___1
NaN	NaN	NaN	NaN	S	2	___1
NaN	-1,29579	NaN	NaN	S	2	___1
2,25051	2,43582	2,51998	2,54752	S	2	___1
-1,74585	-2,45382	NaN	0,159655	S	2	___1
-1,40699	-2,85132	-3,16075	-2,45221	S	3	___1
NaN	0,0642242	0,78636	NaN	S	2	___1
-0,00142097	NaN	NaN	-0,557146	S	2	___1
-2,50751	NaN	-1,59395	-0,731503	S	2	___1
0,227615	1,33592	-4,84266	NaN	S	2	___1
NaN	-0,648373	2,76577	NaN	S	3	___1
-0,920956	-1,81457	-1,2982	-0,527433	S	2	___1
3,71073	3,03883	3,00946	NaN	S	3	___1
NaN	NaN	NaN	NaN	S	2	___1
-1,33346	-1,88052	NaN	NaN	S	2	___1
NaN	NaN	NaN	NaN	S	2	___1
-1,25918	0,531916	NaN	NaN	S	3	___1

Polito et al. Universal ready-to-use immunotherapeutic approach for the treatment of cancer: expanded and activated polyclonal γδ memory T cells.
Phosphorylation sites

NaN	NaN	NaN	NaN	S	2	___1
NaN	NaN	NaN	NaN	S	2	___1
-2,32668	NaN	-2,29078	-2,73439	S	2	___1
NaN	NaN	NaN	NaN	S	2	___1
NaN	NaN	NaN	NaN	S	3	___1
NaN	-1,41478	NaN	NaN	S	3	___1
NaN	NaN	NaN	NaN	S	2	___1
1,39057	0,779305	NaN	-0,426579	S	2	___1
-0,458189	-0,983488	0,348389	NaN	S	2	___1
NaN	NaN	NaN	NaN	S	2	___1
-0,0967197	0,442495	1,0551	NaN	S	3	___1
NaN	-2,44315	-1,83271	-3,23399	S	2	___1
-0,873402	-1,04	-1,96694	NaN	S	2	___1
0,0759907	0,95805	NaN	-1,03937	S	2	___1
0,200825	0,463591	NaN	NaN	S	2	___1
2,45309	2,61004	1,69198	0,504637	S	2	___1
NaN	NaN	-1,35696	NaN	S	3	___1
-0,593058	-2,02549	-2,88643	NaN	S	2	___1
NaN	NaN	NaN	NaN	S	2	___1
2,1823	3,07885	2,02384	2,13245	S	3	___1
0,254526	0,197298	0,0874367	0,521984	S	3	___1
1,91457	1,85239	-0,284639	2,68813	S	3	___1
-1,32085	-0,768084	NaN	0,208275	S	3	___1
-1,57581	-1,54099	-1,23833	1,02674	S	2	___1
-0,127388	0,361336	NaN	1,33536	S	3	___1
-1,03688	-0,107559	NaN	NaN	S	4	___1
-1,02541	-1,71945	-0,783688	-0,591185	S	2	___1
0,982134	NaN	0,395082	0,255703	S	3	___1
NaN	1,8066	1,95798	1,05846	S	3	___1
2,44713	NaN	0,249895	0,426407	S	3	___1
NaN	NaN	NaN	NaN	S	2	___1
-1,41796	0,332191	-0,0876522	-0,140215	S	2	___1
1,3907	1,40458	2,97145	NaN	S	2	___1
-1,05045	2,15296	-3,01343	NaN	S	2	___1

Polito et al. Universal ready-to-use immunotherapeutic approach for the treatment of cancer: expanded and activated polyclonal γδ memory T cells.
Phosphorylation sites

-0,717031	1,04778	2,81406	-2,77265	S	3	___1
-0,175644	-0,471916	-0,385983	0,306574	S	2	___1
3,12325	3,32022	1,75567	2,29225	S	2	___1
0,148052	-2,09992	-0,196524	NaN	S	2	___1
NaN	NaN	NaN	NaN	S	2	___1
-3,68811	-2,22548	1,89172	-2,21611	S	2	___1
NaN	NaN	NaN	NaN	S	2	___1
NaN	NaN	NaN	NaN	S	3	___1
2,57148	4,235	1,10172	3,23881	S	3	___1
NaN	NaN	NaN	NaN	S	3	___1
NaN	NaN	0,84247	NaN	S	3	___1
NaN	NaN	NaN	NaN	S	3	___1
3,29937	2,94726	1,09759	1,14945	S	2	___1
0,252405	-0,397148	0,0475044	-2,035	S	3	___1
5,45627	4,99579	0,199993	3,32689	S	3	___1
NaN	NaN	NaN	NaN	S	3	___1
-0,271673	0,819973	0,2556	0,518211	S	2	___1
1,49702	1,32728	0,79631	NaN	S	2	___1
NaN	NaN	NaN	NaN	S	3	___1
NaN	-1,2551	NaN	NaN	S	2	___1
0,413929	-0,785093	-0,686844	-2,00893	S	2	___1
-0,696112	0,469898	0,0815334	NaN	S	2	___1
NaN	NaN	NaN	NaN	S	3	___1
NaN	-1,98493	NaN	NaN	S	2	___1
-1,2105	2,60345	3,20772	1,54736	S	3	___1
0,443758	1,08284	0,434364	-0,336538	S	2	___1
-0,00128174	-0,0377426	-1,50057	-1,39954	S	2	___1
-1,08231	NaN	0,627911	-2,64013	S	2	___1
-2,09683	-0,689585	-0,268515	NaN	S	3	___1
3,14421	4,48795	5,41368	3,51247	S	3	___1
NaN	NaN	NaN	NaN	S	2	___1
2,30053	-0,651228	-2,28294	2,05835	S	2	___1
NaN	NaN	NaN	NaN	S	2	___1
4,2817	4,36229	4,67737	3,94596	S	2	___1

Polito et al. Universal ready-to-use immunotherapeutic approach for the treatment of cancer: expanded and activated polyclonal γδ memory T cells.
Phosphorylation sites

NaN	NaN	NaN	NaN	S	2	___1
-2,19485	-0,270428	0,820631	-2,61158	S	2	___1
0,604794	1,04532	-1,49184	-1,16437	S	2	___1
-1,13239	-0,185921	NaN	NaN	S	3	___1
0,386042	-0,156816	0,261805	-0,814512	S	3	___1
NaN	NaN	-1,6477	NaN	S	2	___1
-1,12315	-1,34289	0,0651855	NaN	S	2	___1
-0,69701	-0,379293	0,936188	NaN	S	2	___1
2,27463	1,7319	1,17909	0,257351	S	2	___1
NaN	-0,549217	-0,203045	NaN	S	2	___1
NaN	NaN	NaN	NaN	S	3	___1
-1,00872	0,179674	0,521824	-0,804617	S	2	___1
NaN	NaN	4,06472	-0,890945	S	3	___1
NaN	NaN	NaN	NaN	S	3	___1
0,0336285	0,276525	-0,317413	-1,6468	S	2	___1
NaN	NaN	NaN	NaN	S	2	___1
NaN	NaN	NaN	NaN	S	3	___1
-0,0741119	1,56529	1,26072	-0,238903	S	3	___1
NaN	NaN	NaN	NaN	S	3	___1
-3,98151	-3,42541	NaN	-1,10406	S	2	___1
NaN	-0,483419	NaN	3,36237	S	3	___1
-1,14069	-1,95726	-1,13188	-0,50695	S	2	___1
NaN	-1,59108	-1,42264	-1,40691	S	2	___1
NaN	-1,17993	NaN	-0,434788	S	2	___1
-1,72463	-1,81007	-1,2539	-0,0919304	S	2	___1
-1,91011	-3,0581	-1,21507	0,635689	S	2	___1
NaN	-0,951155	NaN	-0,530409	S	2	___1
-1,11555	-0,428427	-0,0387783	-0,779188	S	2	___1
-2,22135	-1,3251	-1,36689	-1,23567	S	3	___1
NaN	NaN	NaN	NaN	S	2	___1
3,27388	1,77142	NaN	-0,133492	S	2	___1
-0,433554	-1,05906	1,7026	-1,30975	S	2	___1
NaN	NaN	NaN	NaN	S	3	___1
0,493679	0,298754	1,3328	-0,42705	S	2	___1

NaN	1,53545	1,74516	0,326399	S	2	___1
NaN	-2,77515	-0,586767	-2,36926	S	2	___1
-1,18593	-1,38093	-0,500969	-2,99603	S	2	___1
-0,817133	-2,40011	NaN	NaN	S	4	___1
-2,66461	NaN	-1,0379	NaN	S	2	___1
1,50082	1,03999	2,30316	0,0736771	S	3	___1
1,1877	1,07707	2,21728	0,440611	S	2	___1
-0,123079	-1,44713	-1,9217	NaN	S	2	___1
NaN	1,80654	3,25877	1,40194	S	3	___1
NaN	-1,3355	-1,12719	-0,669907	S	2	___1
0,173435	-1,54382	NaN	NaN	S	2	___1
-2,38951	-3,00444	NaN	NaN	S	2	___1
-0,270796	0,119829	NaN	-1,29054	S	2	___1
-0,537035	-0,111895	NaN	-1,30396	S	3	___1
-0,611998	-1,29745	-0,614578	-1,37381	S	3	___1
-2,14021	-2,46675	NaN	NaN	S	2	___1
NaN	NaN	NaN	NaN	S	2	___1
NaN	-0,972486	NaN	NaN	S	3	___1
-0,790878	-0,916836	-0,416845	-0,691912	S	2	___1
-0,567621	NaN	1,39678	1,26117	S	2	___1
-1,06039	-1,25644	NaN	-1,22693	S	2	___1
-1,86372	-1,84597	-1,21189	-1,5322	S	2	___1
0,190933	-0,0876102	0,551363	-0,568148	S	2	___1
0,932493	-1,73625	2,56382	1,76036	S	3	___1
-0,0540447	NaN	NaN	-2,5513	S	2	___1
NaN	NaN	NaN	NaN	S	2	___1
NaN	0,230431	1,18064	1,30657	S	2	___1
-0,572405	-1,99248	-0,830103	-2,38362	S	2	___1
NaN	NaN	NaN	NaN	S	2	___1
-2,51942	NaN	NaN	NaN	S	2	___1
1,63002	1,2259	NaN	1,32099	S	2	___1
NaN	1,47558	NaN	1,64241	S	3	___1
NaN	NaN	NaN	NaN	S	2	___1
0,646694	-0,410788	1,17137	0,807501	S	2	___1

Polito et al. Universal ready-to-use immunotherapeutic approach for the treatment of cancer: expanded and activated polyclonal γδ memory T cells.
Phosphorylation sites

0,130117	-0,137928	-0,38249	0,140825	S	2	___1
2,05901	1,4991	-1,47298	1,80697	S	2	___1
NaN	NaN	NaN	NaN	S	2	___1
2,10773	2,11681	NaN	1,92424	S	2	___1
1,3698	0,135853	-0,822962	0,935495	S	2	___1
-0,265705	-0,876759	0,7869	-0,367966	S	2	___1
NaN	NaN	NaN	NaN	S	2	___1
2,95047	2,50781	2,39983	2,53908	S	2	___1
NaN	NaN	0,59873	NaN	S	2	___1
NaN	NaN	NaN	NaN	S	2	___1
NaN	NaN	NaN	NaN	S	2	___1
NaN	5,48201	2,2869	4,50447	S	2	___1
NaN	-1,07323	NaN	-1,11483	S	3	___1
2,78333	4,09104	1,85931	3,83583	S	3	___1
NaN	-1,65631	NaN	NaN	S	2	___1
NaN	NaN	NaN	NaN	S	3	___1
NaN	NaN	NaN	NaN	S	2	___1
NaN	NaN	NaN	NaN	S	2	___1
-0,929247	-0,832624	NaN	-0,921144	S	2	___1
0,587143	-0,432001	-1,12151	-0,717184	S	2	___1
0,310581	-0,974018	0,173574	-0,372295	S	3	___1
NaN	NaN	NaN	NaN	S	3	___1
NaN	NaN	NaN	NaN	S	3	___1
0,468935	0,205736	NaN	0,139528	S	4	___1
-0,024601	NaN	1,75918	0,144432	S	2	___1
NaN	NaN	NaN	NaN	S	2	___1
NaN	NaN	-1,26806	NaN	S	2	___1
NaN	NaN	NaN	NaN	S	2	___1
NaN	NaN	NaN	NaN	S	2	___1
-1,48398	-1,24847	NaN	-3,11167	S	2	___1
-1,08907	-1,57618	-0,234463	-1,01011	S	2	___1
NaN	-0,974464	-0,589075	-0,473455	S	3	___1
NaN	NaN	NaN	NaN	S	3	___1
NaN	NaN	-0,870808	NaN	S	3	___1

Polito et al. Universal ready-to-use immunotherapeutic approach for the treatment of cancer: expanded and activated polyclonal γδ memory T cells.
Phosphorylation sites

NaN	NaN	NaN	NaN	S	2	___1
NaN	NaN	NaN	NaN	S	2	___1
2,10982	NaN	NaN	NaN	S	2	___1
NaN	2,68161	2,20633	3,01119	S	3	___1
NaN	NaN	NaN	NaN	S	3	___1
NaN	NaN	NaN	NaN	S	2	___1
0,594734	-0,250816	1,27175	0,0902538	S	2	___1
-0,761154	NaN	2,00871	0,62722	S	2	___1
-1,46348	-0,999039	-1,86465	-0,74577	S	3	___1
1,99326	1,96579	3,36797	1,12474	S	3	___1
NaN	0,346687	NaN	-1,19526	S	2	___1
NaN	NaN	NaN	NaN	S	2	___1
NaN	NaN	NaN	0,320089	S	2	___1
0,948936	2,44656	2,45422	1,7981	S	3	___1
1,0615	-0,644814	-0,0169735	-0,20949	S	2	___1
0,254498	-0,296629	0,801262	-1,13393	S	2	___1
NaN	NaN	NaN	NaN	S	2	___1
0,966261	1,37303	-2,18984	-1,15814	S	3	___1
-0,87747	-0,911407	0,667997	NaN	S	3	___1
1,07148	0,711033	-0,690821	2,12011	S	3	___1
NaN	NaN	NaN	NaN	S	2	___1
NaN	NaN	NaN	NaN	S	2	___1
NaN	NaN	NaN	NaN	S	3	___1
-0,970812	-2,19816	-0,476345	NaN	S	2	___1
-0,317266	-0,913834	-0,842653	NaN	S	2	___1
-0,987133	0,0196095	NaN	-1,66303	S	2	___1
1,92948	1,60515	1,69773	1,68471	S	2	___1
4,19352	3,90179	1,40586	2,72657	S	2	___1
0,413538	-5,55071	1,33345	1,19579	S	2	___1
-3,57577	NaN	0,2062	NaN	S	2	___1
3,97962	3,19488	3,91351	5,22666	S	2	___1
NaN	NaN	NaN	NaN	S	2	___1
NaN	-1,59286	-0,790113	NaN	S	2	___1
-0,487278	1,36775	-1,46976	2,18548	S	3	___1

Polito et al. Universal ready-to-use immunotherapeutic approach for the treatment of cancer: expanded and activated polyclonal γδ memory T cells.
Phosphorylation sites

NaN	NaN	NaN	NaN	S	2	___1
-1,74422	NaN	-2,3971	-3,69878	S	2	___1
-0,22084	-1,30179	-0,296539	NaN	S	2	___1
NaN	-2,03462	-2,00277	-1,19983	S	3	___1
NaN	-0,0337791	1,51575	0,178261	S	2	___1
-0,116566	NaN	NaN	NaN	S	2	___1
2,35789	2,88593	-0,784328	2,38706	S	2	___1
2,93972	5,24735	3,91195	3,72836	S	2	___1
-1,85455	-0,880659	-2,45897	-1,11716	S	2	___1
1,65113	0,671606	0,312481	-0,199959	S	2	___1
0,555101	-1,05312	-1,90688	-0,304159	S	2	___1
-0,0984249	-0,890461	0,320387	1,06351	S	2	___1
1,05795	NaN	NaN	-3,39168	S	3	___1
1,82705	1,26216	1,1273	2,30695	S	2	___1
2,8173	2,29815	2,96103	3,07396	S	2	___1
0,736523	0,274208	-1,86329	2,0889	S	2	___1
-0,538343	NaN	NaN	-1,04197	S	2	___1
-0,29291	NaN	NaN	NaN	S	2	___1
1,53816	1,30941	-0,438414	1,10178	S	2	___1
-2,58476	-1,6479	NaN	0,55147	S	2	___1
-3,68044	NaN	0,31897	1,75988	S	2	___1
0,374945	-0,398746	0,635511	-0,0599556	S	2	___1
1,65323	NaN	2,80886	NaN	S	3	___1
0,91185	-0,556667	1,18125	0,916136	S	3	___1
7,18529	6,86046	6,63667	6,45542	S	2	___1
NaN	NaN	NaN	NaN	S	2	___1
4,07575	2,08236	2,51616	2,77372	S	3	___1
NaN	-1,44261	NaN	-2,18117	S	2	___1
1,28419	1,83018	2,09116	1,22314	S	2	___1
NaN	NaN	1,65639	-0,440275	S	2	___1
-1,27527	NaN	-2,09005	-1,1219	S	2	___1
0,773739	0,464689	-0,162027	1,90948	S	2	___1
NaN	NaN	NaN	NaN	S	2	___1
-1,52228	-1,8212	-0,775578	NaN	S	2	___1

Polito et al. Universal ready-to-use immunotherapeutic approach for the treatment of cancer: expanded and activated polyclonal γδ memory T cells.
Phosphorylation sites

NaN	NaN	NaN	NaN	S	2	___1
-1,04959	-1,89911	-1,66266	-2,56912	S	2	___1
NaN	NaN	NaN	NaN	S	2	___1
NaN	-2,05721	NaN	NaN	S	3	___1
NaN	-1,60346	-3,606	-1,59698	S	2	___1
-0,211662	-1,42285	-0,420174	0,218599	S	2	___1
4,01217	4,62413	4,27577	3,62594	S	2	___1
-2,24052	-0,321606	-0,774223	-0,136398	S	2	___1
NaN	NaN	NaN	NaN	S	2	___1
-0,929113	-1,77256	-1,46461	NaN	S	2	___1
-0,170176	NaN	-0,396973	NaN	S	2	___1
NaN	NaN	NaN	NaN	S	3	___1
1,97742	2,04773	1,06903	1,9987	S	3	___1
0,851166	0,169195	-0,56319	0,219463	S	2	___1
NaN	NaN	NaN	NaN	S	2	___1
0,800385	NaN	NaN	-2,09558	S	2	___1
NaN	NaN	NaN	NaN	S	2	___1
5,47079	4,57699	3,19184	4,23906	S	3	___1
0,944677	1,61163	0,0618668	0,855286	S	3	___1
-0,114576	0,776831	0,955639	NaN	S	2	___1
0,0119038	-1,77476	0,313211	0,477571	S	2	___1
NaN	-3,72137	2,30118	NaN	S	2	___1
NaN	NaN	NaN	NaN	S	2	___1
NaN	-0,935776	1,87424	NaN	S	2	___1
-0,11484	-0,661022	-1,27813	-0,402969	S	2	___1
-0,273684	NaN	1,95029	-0,791746	S	2	___1
0,838802	-0,216858	-2,42845	-0,567106	S	2	___1
-0,0578632	0,8092	0,411642	-0,537666	S	2	___1
-2,87715	-2,9105	-0,534254	1,66231	S	3	___1
NaN	NaN	NaN	NaN	S	3	___1
-1,57282	-1,1823	NaN	-2,53275	S	2	___1
0,520014	0,218233	NaN	-0,239042	S	2	___1
-0,777275	-1,14604	-4,40644	NaN	S	2	___1
2,71274	2,80853	NaN	-1,41195	S	2	___1

NaN	NaN	NaN	NaN	S	2	___1
0,0804863	2,30519	1,65676	1,78239	S	2	___1
2,76408	2,82038	2,59951	2,42511	S	2	___1
1,51423	0,781115	2,1324	0,806431	S	2	___1
2,15867	1,24118	NaN	0,334448	S	3	___1
NaN	-0,0475788	2,82976	0,485123	S	2	___1
-1,14812	-0,787838	-2,00408	NaN	S	2	___1
3,95881	3,07948	2,84338	3,15443	S	3	___1
0,999325	0,300724	NaN	NaN	S	3	___1
0,661949	NaN	NaN	-0,810644	S	2	___1
NaN	0,235525	NaN	NaN	S	2	___1
NaN	NaN	NaN	NaN	S	2	___1
NaN	NaN	NaN	NaN	S	3	___1
1,3589	1,22337	0,360332	NaN	S	3	___1
1,25293	0,156876	-0,583555	0,104204	S	2	___1
NaN	-1,65087	-2,66088	2,08233	S	2	___1
NaN	-0,145538	1,27473	0,0554218	S	3	___1
-1,52367	-0,539822	-1,35261	0,911287	S	2	___1
NaN	NaN	NaN	NaN	S	3	___1
4,66821	4,50327	-2,03341	4,19536	S	2	___1
-1,13886	NaN	NaN	-1,25349	S	2	___1
NaN	NaN	NaN	NaN	S	2	___1
2,29765	1,73741	2,27661	2,01739	S	2	___1
NaN	1,29326	0,0374012	NaN	S	2	___1
1,18841	0,363222	0,800058	0,902918	S	2	___1
NaN	2,33607	3,67118	2,25205	S	2	___1
NaN	-0,411493	-0,954525	-0,240454	S	2	___1
-1,02203	-0,587315	-2,2942	-1,09468	S	2	___1
NaN	NaN	NaN	NaN	S	3	___1
NaN	NaN	NaN	NaN	S	3	___1
0,0527611	0,798346	NaN	-2,27487	S	2	___1
-0,473984	0,895418	1,06202	1,17633	S	3	___1
-0,924109	-0,10944	NaN	-1,61151	S	2	___1
-0,396231	-0,99707	0,259455	-0,778706	S	2	___1

2,42123	1,1532	2,12873	1,4903	S	4	___1
NaN	NaN	NaN	NaN	S	2	___1
0,711012	1,03364	2,49492	2,61066	S	3	___1
NaN	-1,83063	NaN	NaN	S	2	___1
NaN	NaN	NaN	NaN	S	2	___1
NaN	NaN	0,457363	NaN	S	2	___1
2,98934	2,50033	3,04718	2,30335	S	2	___1
1,96114	2,43666	5,55835	2,75687	S	3	___1
NaN	-0,773386	-1,44819	NaN	S	3	___1
-1,51068	NaN	-2,15305	NaN	S	3	___1
0,703733	2,21429	3,63533	1,47015	S	2	___1
1,99839	3,41337	3,08085	0,8335	S	2	___1
0,283253	-0,404253	NaN	-0,543324	S	2	___1
1,06462	2,15267	NaN	-2,87043	S	2	___1
2,6082	2,51419	5,20541	2,62277	S	2	___1
1,49678	1,48982	-0,106974	0,0677223	S	3	___1
0,264858	-1,99166	-1,04994	-0,408058	S	2	___1
-0,263872	-1,26124	NaN	-0,279469	S	2	___1
-0,815058	-2,00083	NaN	NaN	S	2	___1
1,94881	1,94506	2,20148	1,0606	S	2	___1
NaN	NaN	NaN	NaN	S	2	___1
1,34955	0,93322	NaN	0,113586	S	3	___1
-1,64759	1,23279	-1,17532	-0,584776	S	2	___1
-2,0058	-3,52093	-1,74608	0,47368	S	2	___1
NaN	NaN	NaN	NaN	S	2	___1
-0,243631	0,250257	-2,00296	-0,134161	S	2	___1
NaN	NaN	NaN	NaN	S	2	___1
NaN	NaN	NaN	NaN	S	2	___1
NaN	-0,107641	-1,38544	1,12033	S	3	___1
NaN	NaN	NaN	NaN	S	3	___1
0,782951	0,227198	0,151302	-1,66362	S	2	___1
0,326557	-2,10125	NaN	NaN	S	4	___1
2,50967	2,56874	0,987917	0,616228	S	2	___1
NaN	NaN	NaN	NaN	S	3	___1

-1,36363	-0,655041	-0,532368	2,62899	S	2	___1
1,76948	0,444614	1,0196	0,969748	S	2	___1
0,136089	0,541426	1,3806	NaN	S	2	___1
-1,02746	NaN	-0,0268269	NaN	S	2	___1
0,59602	-0,221535	NaN	-1,55894	S	2	___1
-1,58337	-0,63607	NaN	NaN	S	3	___1
NaN	NaN	NaN	NaN	S	2	___1
NaN	NaN	2,19941	NaN	S	3	___1
0,833561	1,77187	-0,188704	1,74891	S	2	___1
NaN	1,39688	-0,977026	0,48955	S	3	___1
NaN	NaN	NaN	NaN	S	2	___1
1,69246	2,01787	1,86692	1,82599	S	2	___1
1,35173	1,62904	1,18852	0,488464	S	2	___1
NaN	NaN	NaN	NaN	S	2	___1
-0,344416	-0,70433	NaN	-0,870972	S	3	___1
NaN	NaN	NaN	NaN	S	2	___1
-0,653851	0,811447	1,67247	-0,17515	S	2	___1
-1,68744	-0,74987	NaN	NaN	S	3	___1
1,17002	0,950304	-0,603113	0,140295	S	2	___1
3,35295	2,32918	NaN	-1,94188	S	2	___1
NaN	0,994381	-0,90666	0,0719852	S	2	___1
NaN	NaN	NaN	NaN	S	2	___1
NaN	NaN	NaN	NaN	S	3	___1
NaN	NaN	NaN	NaN	S	2	___1
2,60011	2,1268	5,23111	2,21469	S	3	___1
3,6068	2,57956	NaN	3,05181	S	2	___1
-2,56529	-3,78954	NaN	-0,773771	S	2	___1
NaN	0,277151	NaN	NaN	S	2	___1
0,503294	-1,40984	NaN	-0,291948	S	2	___1
-2,75623	-2,37315	NaN	-2,94049	S	2	___1
2,66998	1,5705	1,72355	2,27139	S	2	___1
NaN	0,082449	0,644754	NaN	S	4	___1
NaN	1,25073	NaN	-0,0051403	S	3	___1
0,827776	0,465843	-1,32588	NaN	S	2	___1

Polito et al. Universal ready-to-use immunotherapeutic approach for the treatment of cancer: expanded and activated polyclonal γδ memory T cells.
Phosphorylation sites

-0,123419	0,834879	NaN	NaN	S	2		___1
6,08596	4,04288	4,11797	3,99372	S	3		___1
0,847145	0,62607	0,359915	NaN	S	2		___1
NaN	-3,50845	-0,349318	NaN	S	2		___1
NaN	NaN	NaN	NaN	S	3		___1
NaN	-1,00401	-2,01825	-1,65977	S	2		___1
NaN	NaN	NaN	NaN	S	3		___1
-0,325752	-3,4972	-3,06526	NaN	S	2		___1
-0,658764	-1,14457	-1,3164	-2,7483	S	2		___1
NaN	-1,36886	1,25497	-1,32212	S	3		___1
-1,63151	-1,17894	-0,230717	NaN	S	2		___1
-0,985968	-0,191795	-1,64625	-1,74491	S	2		___1
0,605341	1,70086	2,0542	-0,183878	S	2		___1
-1,14789	-0,898069	0,614473	-1,72859	S	2		___1
-0,68738	0,149719	NaN	-1,60729	S	2		___1
NaN	0,247131	1,05521	0,926649	S	2		___1
-0,520838	-1,41163	1,13463	NaN	S	2		___1
NaN	NaN	NaN	NaN	S	2		___1
NaN	NaN	0,298035	NaN	S	2		___1
NaN	NaN	NaN	NaN	S	4		___1
1,66759	0,144724	NaN	0,510288	S	2		___1
NaN	-3,34112	-1,05264	0,963514	S	2		___1
-1,32467	0,106022	NaN	-0,210302	S	2		___1
0,812153	0,938015	NaN	0,338381	S	2		___1
NaN	NaN	NaN	-0,62999	S	2		___1
2,03917	2,58399	3,11392	2,55579	S	3		___1
-0,956957	-0,81039	-2,9183	-1,03783	S	2		___1
0,427156	-0,439589	-0,434414	-1,2638	S	2		___1
NaN	-4,61625	NaN	-1,44008	S	2		___1
0,090868	0,38184	0,629942	1,1653	S	3		___1
0,5051	1,84462	0,612434	0,771315	S	3		___1
-0,714186	-0,947231	-0,649309	0,988523	S	2		___1
NaN	NaN	NaN	NaN	S	2		___1
NaN	1,67065	-2,42095	1,37473	S	2		___1

5,17425	7,69563	7,14623	8,223	S	3	___1
-2,37037	NaN	1,45184	-1,22457	S	2	___1
NaN	NaN	NaN	NaN	S	2	___1
-0,185383	NaN	-0,453856	0,384026	S	2	___1
NaN	-2,6054	-0,292315	-1,66935	S	2	___1
-1,1124	-1,63675	-0,79859	-0,278986	S	2	___1
-1,92964	NaN	-1,78919	-0,824558	S	2	___1
4,8144	5,02341	5,04399	3,86271	S	3	___1
2,10524	1,77699	2,7873	0,920847	S	2	___1
-1,11248	-1,83012	NaN	-1,20738	S	2	___1
2,29715	1,37884	1,25741	1,54125	S	2	___1
-0,378016	3,2079	1,15338	2,96327	S	3	___1
-2,28428	-0,0861607	NaN	0,327091	S	3	___1
1,66497	0,873451	2,8666	1,63255	S	2	___1
1,76775	1,54434	-0,10705	2,95127	S	2	___1
0,743576	-0,887936	-0,129532	1,97184	S	2	___1
1,47051	2,02959	-0,321276	1,40309	S	2	___1
NaN	NaN	NaN	NaN	S	2	___1
NaN	NaN	-1,05052	NaN	S	3	___1
NaN	NaN	NaN	NaN	S	2	___1
-1,78974	-1,46914	-0,898567	-1,66838	S	2	___1
-0,356741	-0,0322285	0,30884	-2,37788	S	2	___1
NaN	NaN	NaN	NaN	S	2	___1
2,94035	2,69209	-0,0413151	2,69099	S	2	___1
NaN	NaN	NaN	NaN	S	2	___1
NaN	NaN	NaN	NaN	S	2	___1
NaN	NaN	NaN	NaN	S	2	___1
1,25443	-0,309345	NaN	-0,859919	S	2	___1
-1,36998	-2,1623	-2,10419	-1,12277	S	4	___1
-1,31205	-1,50617	NaN	-1,63598	S	2	___1
-1,25396	-2,97864	-0,767511	-0,292263	S	2	___1
2,00721	1,47961	2,58375	1,1607	S	2	___1
0,0729294	-1,28127	1,20254	0,412111	S	3	___1
NaN	NaN	NaN	NaN	S	2	___1

2,70574	2,63478	-0,708435	1,313	S	2	___1
0,73344	1,24601	0,951313	-0,00135994	S	3	___1
NaN	-0,434561	NaN	NaN	S	3	___1
-1,10292	0,0946388	NaN	NaN	S	2	___1
NaN	NaN	-0,251873	NaN	S	3	___1
-1,457	-0,247753	1,37631	0,672764	S	3	___1
NaN	-0,391569	-1,26225	-0,893684	S	3	___1
NaN	0,574579	1,59007	2,40309	S	4	___1
3,63806	0,52656	1,10853	0,737013	S	2	___1
0,301811	-2,79054	NaN	-0,906845	S	2	___1
NaN	NaN	NaN	NaN	S	2	___1
NaN	-1,07409	-1,3329	-1,36395	S	2	___1
-1,37518	-1,15614	NaN	-1,64775	S	2	___1
0,4461	0,112206	-0,94809	0,459372	S	2	___1
NaN	0,0642242	NaN	0,948906	S	2	___1
-0,832106	-0,951256	-2,70419	-1,15396	S	2	___1
NaN	NaN	NaN	NaN	S	2	___1
-0,814022	NaN	NaN	NaN	S	2	___1
NaN	-0,125555	0,771576	-1,43951	S	2	___1
-4,88182	-0,363214	2,1853	2,84938	S	2	___1
1,67964	3,53886	-2,41064	0,570902	S	2	___1
0,0996914	0,0117378	-0,786978	-0,393923	S	3	___1
-1,53538	-2,3928	NaN	0,625628	S	2	___1
-1,57014	NaN	-1,23181	0,123749	S	2	___1
NaN	-0,0919666	-0,976614	-0,70373	S	3	___1
NaN	NaN	1,55462	-1,10004	S	2	___1
1,21843	1,07883	-1,92807	1,85645	S	2	___1
-2,32124	-2,042	NaN	-0,266535	S	3	___1
NaN	NaN	NaN	NaN	S	3	___1
NaN	-0,123489	1,38239	0,54641	S	3	___1
1,11043	-0,444118	0,394234	-0,600176	S	2	___1
-0,255205	3,32658	2,5089	2,41908	S	3	___1
NaN	NaN	NaN	NaN	S	3	___1
1,20705	-0,0872879	-0,172718	-0,526928	S	2	___1

Polito et al. Universal ready-to-use immunotherapeutic approach for the treatment of cancer: expanded and activated polyclonal γδ memory T cells.
Phosphorylation sites

4,84571	4,95361	4,99659	4,25092	S	2	___1
2,94044	4,09787	4,04597	3,45143	S	3	___1
-1,86347	NaN	0,860144	2,37207	S	2	___1
NaN	0,193193	1,1323	0,620319	S	3	___1
NaN	0,541946	1,83688	2,4847	S	3	___1
1,30943	-0,350403	-0,701645	-2,36588	S	2	___1
NaN	2,06268	NaN	NaN	S	2	___1
1,33397	NaN	NaN	-0,772051	S	3	___1
NaN	NaN	NaN	NaN	S	3	___1
NaN	NaN	NaN	NaN	S	2	___1
NaN	NaN	NaN	NaN	S	2	___1
-0,68593	-1,33974	-0,297197	-1,77049	S	2	___1
-0,365374	0,0542583	-0,379307	-0,907415	S	2	___1
NaN	0,00071907	-0,519264	-1,3101	S	2	___1
1,53111	1,69199	2,32293	0,434124	S	2	___1
NaN	NaN	NaN	NaN	S	2	___1
NaN	NaN	-1,95218	NaN	S	2	___1
-0,978575	-1,7898	NaN	-1,23556	S	2	___1
-0,19368	NaN	NaN	NaN	S	2	___1
NaN	-0,378799	0,00543594	-0,292234	S	2	___1
-1,26283	-0,123489	0,102798	NaN	S	2	___1
NaN	NaN	NaN	NaN	S	2	___1
2,77313	3,54355	-2,90132	2,27023	S	2	___1
NaN	NaN	NaN	NaN	S	2	___1
1,7042	0,898146	NaN	1,50639	S	2	___1
-1,14062	1,23691	1,00402	0,126242	S	3	___1
0,277262	2,09158	NaN	-2,55515	S	3	___1
-0,725084	NaN	-0,27416	NaN	S	2	___1
NaN	NaN	NaN	NaN	S	2	___1
1,6584	2,19938	NaN	1,89859	S	2	___1
2,14699	1,36807	-1,56819	1,28289	S	2	___1
-1,04329	1,81535	NaN	NaN	S	2	___1
NaN	NaN	NaN	NaN	S	3	___1
NaN	NaN	NaN	NaN	S	2	___1

Polito et al. Universal ready-to-use immunotherapeutic approach for the treatment of cancer: expanded and activated polyclonal γδ memory T cells.
Phosphorylation sites

NaN	NaN	NaN	NaN	S	2	___1
NaN	NaN	NaN	NaN	S	2	___1
-1,20354	-1,73236	-0,755085	-1,27867	S	2	___1
4,76963	4,272	4,26346	3,81088	S	2	___1
-1,54364	-1,20594	-0,450607	-2,05481	S	2	___1
-1,6842	-1,00263	NaN	-0,43672	S	2	___1
-1,60165	-0,771315	-0,911905	-1,07436	S	3	___1
-0,937119	0,28849	-1,25384	-0,552122	S	2	___1
0,896019	1,91681	1,41269	0,0606422	S	2	___1
NaN	NaN	NaN	NaN	S	3	___1
NaN	0,491407	-2,1517	-1,43311	S	2	___1
NaN	NaN	NaN	NaN	S	3	___1
-0,655596	0,239769	-3,72335	-2,4048	S	2	___1
2,07431	1,11238	1,12719	0,997467	S	2	___1
-0,826012	0,992552	-1,19671	-0,128414	S	3	___1
1,06981	NaN	1,35089	-1,34568	S	2	___1
NaN	NaN	NaN	NaN	S	3	___1
NaN	NaN	NaN	NaN	S	2	___1
-1,35484	-1,37247	-0,693867	NaN	S	2	___1
0,608231	0,630972	NaN	-0,645041	S	2	___1
-0,429768	-0,417149	-0,978401	NaN	S	3	___1
-2,84553	NaN	-0,507835	-0,924564	S	2	___1
NaN	NaN	NaN	NaN	S	2	___1
1,23156	1,07318	-0,194019	-0,608866	S	3	___1
4,46965	3,85279	4,73593	3,188	S	2	___1
NaN	NaN	NaN	NaN	S	2	___1
2,89165	NaN	0,970207	1,93556	S	3	___1
3,27316	1,92819	0,831003	3,0065	S	2	___1
NaN	NaN	NaN	NaN	S	2	___1
0,648308	0,096838	2,28675	0,0439701	S	2	___1
NaN	NaN	NaN	NaN	S	3	___1
NaN	NaN	NaN	NaN	S	2	___1
NaN	NaN	NaN	NaN	S	2	___1
NaN	NaN	NaN	NaN	S	2	___1

Polito et al. Universal ready-to-use immunotherapeutic approach for the treatment of cancer: expanded and activated polyclonal γδ memory T cells. Phosphorylation sites

2,10266	1,84337	NaN	1,5567	S	2	___1
1,66715	0,620806	2,25068	-3,40773	S	2	___1
NaN	NaN	NaN	NaN	S	2	___1
6,69136	6,4789	4,33705	7,11397	S	3	___1
NaN	NaN	NaN	NaN	S	2	___1
-0,0298958	0,46447	0,973173	1,00927	S	2	___1
NaN	NaN	NaN	NaN	S	2	___1
-2,65458	-3,3465	2,27016	-2,42504	S	2	___1
-1,92556	-1,56979	NaN	-2,35027	S	2	___1
NaN	NaN	NaN	NaN	S	2	___1
NaN	NaN	NaN	NaN	S	2	___1
-0,695101	-1,41159	0,635466	-1,67248	S	2	___1
NaN	-1,25304	NaN	NaN	S	2	___1
1,42401	1,22975	1,77797	-0,439163	S	2	___1
NaN	NaN	-2,09045	NaN	S	2	___1
NaN	NaN	NaN	NaN	S	2	___1
0,697786	-0,455818	0,0958462	-0,707397	S	2	___1
1,84684	2,27893	1,5947	-0,110954	S	2	___1
0,567383	0,491947	NaN	NaN	S	3	___1
1,54542	NaN	1,99745	2,90301	S	2	___1
2,31083	3,11997	-2,46576	NaN	S	2	___1
-1,2289	-2,03242	-0,513206	1,81638	S	2	___1
-0,404921	-1,40263	-3,51313	NaN	S	2	___1
NaN	NaN	NaN	NaN	S	2	___1
-0,945501	-2,79159	-0,751749	NaN	S	2	___1
NaN	-1,0873	NaN	NaN	S	2	___1
-3,6857	NaN	-0,626099	-2,19215	S	2	___1
-0,27113	1,97764	2,30566	0,247301	S	3	___1
-1,10471	-2,78755	NaN	-1,26775	S	2	___1
NaN	NaN	NaN	NaN	S	2	___1
-0,689894	-1,78345	NaN	NaN	S	2	___1
0,759729	0,0139904	0,564861	0,155706	S	2	___1
2,77966	3,1258	3,48654	3,33475	S	2	___1
2,56476	NaN	-2,1005	1,84169	S	2	___1

-3,36994	NaN	-1,49886	-3,21587	S	2	___1
3,29613	1,29564	2,39364	2,03859	S	2	___1
1,06117	2,18826	NaN	2,24228	S	3	___1
-0,770699	NaN	-0,537594	NaN	S	2	___1
0,505003	0,966919	0,985569	NaN	S	2	___1
NaN	NaN	NaN	NaN	S	2	___1
NaN	-0,257414	1,98674	NaN	S	2	___1
NaN	0,628965	NaN	NaN	S	3	___1
-0,115477	NaN	-1,78702	1,58665	S	3	___1
-1,8866	-1,32833	-0,776773	-1,2814	S	3	___1
-1,14024	-2,3815	NaN	-2,08889	S	3	___1
-0,87042	-3,0109	NaN	-2,34633	S	3	___1
-0,433319	NaN	NaN	NaN	S	2	___1
-1,51612	-0,0466385	1,24372	-1,30095	S	3	___1
2,62632	2,59783	2,32832	2,34609	S	2	___1
NaN	-0,305687	-1,94123	-1,2781	S	3	___1
3,34209	3,09295	2,48223	2,9171	S	2	___1
-1,24523	-2,62523	-2,16416	NaN	S	2	___1
-0,75869	NaN	0,20141	-1,33642	S	2	___1
NaN	-1,98226	-1,04821	NaN	S	2	___1
NaN	NaN	NaN	-1,13429	S	3	___1
-0,618099	0,7495	1,01397	0,529987	S	3	___1
-2,35068	0,317608	-0,877804	1,37302	S	3	___1
-2,51541	NaN	-2,79199	-3,56083	S	2	___1
-1,51246	NaN	-1,77733	NaN	S	2	___1
NaN	-0,990519	-1,74983	NaN	S	2	___1
1,06142	0,557789	-3,36028	0,379423	S	2	___1
-0,645697	-0,740089	-2,33413	-0,616531	S	3	___1
0,151798	1,14352	-0,0360565	2,8613	S	2	___1
NaN	NaN	NaN	NaN	S	2	___1
NaN	NaN	NaN	NaN	S	2	___1
-0,927927	-2,17518	-0,371939	-1,00827	S	2	___1
-1,11893	NaN	-0,627176	-1,185	S	2	___1
-0,201387	-2,93951	-0,723883	-1,55556	S	2	___1

Polito et al. Universal ready-to-use immunotherapeutic approach for the treatment of cancer: expanded and activated polyclonal γδ memory T cells.
Phosphorylation sites

0,889637	-1,36335	NaN	1,31017	S	2	___1
NaN	NaN	NaN	NaN	S	2	___1
NaN	-0,856991	3,02284	NaN	S	3	___1
-0,79472	-0,984718	-2,54695	-1,09638	S	2	___1
1,73429	0,956879	1,38462	2,54065	S	2	___1
NaN	NaN	NaN	-0,725681	S	2	___1
-1,01047	NaN	-0,935125	-0,407095	S	2	___1
NaN	NaN	NaN	NaN	S	3	___1
-0,980009	6,73052	3,13402	2,13347	S	2	___1
0,808695	1,05918	NaN	0,461584	S	2	___1
1,13864	1,28734	-0,953081	-0,31531	S	2	___1
0,00432396	NaN	NaN	NaN	S	3	___1
-0,900892	-0,755745	NaN	NaN	S	2	___1
2,50943	2,65213	NaN	2,41011	S	2	___1
NaN	-1,12746	NaN	-0,922033	S	3	___1
NaN	NaN	NaN	NaN	S	2	___1
-0,223669	-0,661381	-0,100422	0,987223	S	2	___1
-1,53558	-1,85337	NaN	0,304678	S	2	___1
0,443962	0,285196	NaN	-0,300577	S	2	___1
-2,08258	-1,90444	NaN	-0,922388	S	2	___1
NaN	-0,444427	NaN	-1,54179	S	2	___1
NaN	1,20659	0,213062	0,0380745	S	2	___1
-0,0593433	-1,02516	NaN	-3,6223	S	2	___1
5,21241	3,85782	1,81944	4,39215	S	2	___1
-0,185383	NaN	0,744881	-0,560459	S	2	___1
-1,46645	-0,569321	0,612206	-0,236197	S	2	___1
1,70197	1,69293	NaN	3,73282	S	2	___1
4,37926	3,90326	3,66128	2,56635	S	3	___1
NaN	NaN	-0,459063	-2,19794	S	2	___1
-2,51533	NaN	0,353991	0,15337	S	2	___1
-2,2064	-2,55219	NaN	-2,40554	S	2	___1
-0,333023	1,17451	0,68787	1,27507	S	2	___1
2,34363	0,465622	NaN	3,2912	S	2	___1
NaN	NaN	NaN	NaN	S	2	___1

2,46481	0,442942	NaN	3,70927	S	2	___1
-2,19913	-0,0264282	NaN	2,06452	S	2	___1
3,80964	2,76012	3,3399	3,65767	S	2	___1
0,980448	-0,21501	0,79336	1,12667	S	2	___1
NaN	NaN	NaN	NaN	S	2	___1
3,67786	2,6382	5,11877	2,11558	S	2	___1
0,618715	NaN	NaN	-1,11285	S	2	___1
-1,05794	-0,683487	-0,529408	-0,302107	S	3	___1
0,115097	-0,817585	1,27723	-1,26938	S	2	___1
3,19397	1,30601	3,43793	1,38741	S	2	___1
1,0001	1,35897	-2,51693	0,93071	S	2	___1
1,55501	0,730028	-1,60382	2,46452	S	2	___1
NaN	NaN	2,68034	NaN	S	2	___1
-3,28674	-5,07272	NaN	-0,146656	S	2	___1
NaN	NaN	NaN	NaN	S	2	___1
NaN	NaN	NaN	NaN	S	2	___1
NaN	NaN	NaN	NaN	S	2	___1
-1,42009	-2,21703	-2,31872	-0,0201454	S	2	___1
0,246752	-0,525131	-2,61504	-0,313416	S	2	___1
0,830078	-1,46577	-0,685009	-0,634346	S	3	___1
2,34383	1,83131	4,4063	0,908045	S	2	___1
NaN	NaN	NaN	NaN	S	2	___1
2,1382	NaN	5,22943	NaN	S	4	___1
5,09252	5,24561	NaN	4,28604	S	2	___1
NaN	-0,0762177	NaN	NaN	S	3	___1
NaN	NaN	NaN	NaN	S	3	___1
NaN	NaN	-0,371218	NaN	S	4	___1
2,08391	1,43209	1,83473	0,647341	S	3	___1
NaN	NaN	NaN	NaN	S	2	___1
0,123644	-1,36031	-0,925943	-1,38325	S	3	___1
NaN	NaN	NaN	NaN	S	3	___1
-0,312698	-0,696939	-2,88638	NaN	S	2	___1
-1,05577	-1,12134	NaN	-1,99981	S	2	___1
-0,934998	-0,199173	NaN	-1,69407	S	2	___1

Polito et al. Universal ready-to-use immunotherapeutic approach for the treatment of cancer: expanded and activated polyclonal γδ memory T cells.
Phosphorylation sites

NaN	NaN	NaN	NaN	S	2	___1
NaN	NaN	NaN	NaN	S	2	___1
NaN	NaN	NaN	NaN	S	2	___1
-1,24499	-2,01537	NaN	-1,51892	S	2	___1
1,39808	1,08184	0,393665	NaN	S	3	___1
NaN	-0,621189	NaN	NaN	S	3	___1
-1,0033	-1,57984	-2,64873	-1,81736	S	2	___1
NaN	1,23408	NaN	0,0490742	S	3	___1
0,53281	-0,0387535	-0,335274	-0,0713558	S	3	___1
-0,343317	-0,831411	-2,01796	NaN	S	2	___1
0,583241	1,36039	1,45948	0,51149	S	2	___1
0,0211868	0,271761	NaN	0,475737	S	3	___1
0,920572	1,71925	NaN	1,22815	S	2	___1
NaN	NaN	NaN	NaN	S	2	___1
1,23191	1,3955	0,709024	NaN	S	2	___1
2,15487	1,87358	NaN	0,825327	S	2	___1
-0,624334	NaN	NaN	NaN	S	2	___1
1,06177	1,10764	NaN	NaN	S	2	___1
NaN	1,09921	NaN	-0,146009	S	3	___1
NaN	NaN	NaN	NaN	S	2	___1
1,12942	1,79937	-0,921131	0,421394	S	2	___1
0,178343	NaN	-1,16656	NaN	S	2	___1
-0,865868	-1,78006	NaN	-0,179096	S	2	___1
NaN	NaN	NaN	NaN	S	2	___1
NaN	NaN	NaN	NaN	S	3	___1
-1,60376	-1,31021	-1,49722	-2,66662	S	2	___1
-0,668589	NaN	0,0935574	-0,787251	S	2	___1
NaN	NaN	NaN	NaN	S	2	___1
-2,41476	-2,65299	NaN	-0,563156	S	2	___1
-2,78185	-1,5055	-2,50328	-1,62814	S	3	___1
-0,999203	-2,64377	1,97448	NaN	S	2	___1
-2,27208	-2,17262	-2,28623	NaN	S	2	___1
-2,09846	NaN	0,108208	NaN	S	2	___1
-1,5397	-0,44381	1,95065	NaN	S	2	___1

-0,538746	-0,381069	NaN	NaN	S	2	___1
2,30741	2,02109	NaN	1,42924	S	2	___1
0,825626	-0,263952	1,12175	-1,00117	S	2	___1
1,93438	1,46051	1,90812	0,622648	S	2	___1
2,32701	1,54374	2,09645	NaN	S	2	___1
-1,13376	-1,94431	-4,29328	NaN	S	2	___1
-1,42186	0,447062	-0,0115204	NaN	S	4	___1
0,235523	-0,170485	-0,675909	-0,714375	S	2	___1
1,44884	1,17753	0,505194	-0,252262	S	2	___1
0,276632	NaN	0,00455475	NaN	S	2	___1
-2,10161	-0,478342	0,0108986	-1,4448	S	2	___1
NaN	-0,0967388	NaN	NaN	S	2	___1
NaN	NaN	-1,78165	NaN	S	2	___1
NaN	-1,18402	-1,68868	-1,80691	S	3	___1
NaN	1,5174	NaN	1,50902	S	4	___1
-0,284277	0,590723	-1,40638	0,846716	S	2	___1
2,99244	1,88133	3,56877	3,17231	S	3	___1
NaN	-1,75401	-1,3006	-2,57819	S	2	___1
NaN	NaN	NaN	NaN	S	3	___1
0,853065	-0,43149	1,01846	0,232443	S	2	___1
NaN	-0,231554	-0,103495	-0,852667	S	3	___1
-1,40277	NaN	-1,65019	-1,38209	S	2	___1
2,10347	1,44497	0,851786	0,0371151	S	2	___1
NaN	NaN	-1,06705	NaN	S	3	___1
-0,969116	-0,479715	NaN	-1,06934	S	2	___1
NaN	NaN	NaN	NaN	S	2	___1
2,51368	1,7247	3,39472	1,31639	S	2	___1
NaN	NaN	NaN	NaN	S	2	___1
NaN	NaN	NaN	NaN	S	3	___1
NaN	-2,60881	4,41756	-0,705141	S	3	___1
-1,13065	0,243935	2,81618	0,0655975	S	2	___1
NaN	-0,650633	-1,46435	-1,11712	S	3	___1
2,54192	2,62167	0,424549	2,54896	S	2	___1
2,1695	1,55848	-0,00107002	1,89878	S	2	___1

Polito et al. Universal ready-to-use immunotherapeutic approach for the treatment of cancer: expanded and activated polyclonal γδ memory T cells.
Phosphorylation sites

-1,35457	-1,47995	-0,762489	NaN	S	2		___1
NaN	0,195843	-0,449718	NaN	S	2		___1
NaN	NaN	NaN	NaN	S	2		___1
-2,10335	-3,71658	-2,01373	NaN	S	2		___1
NaN	NaN	NaN	NaN	S	2		___1
0,137569	0,216539	0,426403	-0,371475	S	2		___1
2,75738	1,57549	1,86731	0,994413	S	3		___1
-1,65423	-2,41721	NaN	0,0488701	S	2		___1
1,8504	2,10572	-0,276413	0,700289	S	2		___1
2,53983	4,06293	0,733307	2,40145	S	2		___1
1,2409	3,01643	1,23237	-0,168737	S	3		___1
0,209061	1,10841	NaN	-2,88353	S	2		___1
0,769152	0,399185	-1,41921	-1,55157	S	2		___1
2,2532	2,43076	-0,295881	0,869204	S	2		___1
-0,253262	NaN	NaN	NaN	S	2		___1
NaN	-1,59334	-0,219482	-1,91928	S	2		___1
-0,0496998	-1,1293	NaN	-1,24137	S	2		___1
0,261799	0,822033	-0,47761	1,13331	S	2		___1
3,91163	3,69402	NaN	3,29285	S	3		___1
3,30601	3,03273	NaN	3,76937	S	2		___1
-0,261877	-1,46807	-1,45967	NaN	S	2		___1
-1,34512	-2,63989	-2,82973	NaN	S	2		___1
0,0240173	2,08386	2,25834	2,71367	S	2		___1
NaN	NaN	NaN	NaN	S	2		___1
NaN	NaN	-0,170856	NaN	S	2		___1
0,049551	NaN	NaN	-0,618723	S	3		___1
1,77748	1,6177	0,110731	1,35646	S	2		___1
1,57084	0,239897	NaN	0,165661	S	2		___1
NaN	NaN	NaN	NaN	S	2		___1
NaN	3,28605	NaN	NaN	S	2		___1
1,02851	1,63849	NaN	-0,133801	S	3		___1
0,6562	-1,74465	2,1054	-0,633875	S	3		___1
NaN	NaN	NaN	NaN	S	2		___1
0,613811	0,864862	0,259706	-0,0155449	S	3		___1

NaN	NaN	NaN	NaN	S	3	___1
NaN	NaN	NaN	NaN	S	2	___1
NaN	NaN	NaN	NaN	S	2	___1
2,95352	2,41052	1,99757	1,83448	S	2	___1
-0,0864201	-0,0570889	-1,18085	-0,8155	S	2	___1
0,128311	0,0634995	-2,04182	-0,940975	S	2	___1
0,0502548	-0,334354	NaN	-1,00005	S	2	___1
-0,452868	0,0741215	-0,953804	NaN	S	3	___1
1,3051	0,00495529	1,8014	-0,713837	S	2	___1
-0,991055	-1,58424	NaN	-0,566135	S	2	___1
1,9209	1,74894	-0,482641	2,14807	S	2	___1
1,1608	2,28923	0,821684	1,92683	S	3	___1
-1,10575	-0,931129	NaN	-1,04758	S	2	___1
-2,39158	-2,40175	-1,53807	-1,88912	S	2	___1
-0,41551	-0,356794	NaN	-1,25461	S	2	___1
-2,61206	-2,82337	NaN	-0,0666866	S	2	___1
-0,246464	NaN	NaN	NaN	S	3	___1
-1,36658	-0,841425	0,54356	NaN	S	3	___1
-0,0163059	-1,13591	-1,31628	-0,231129	S	3	___1
NaN	NaN	0,893227	0,139721	S	3	___1
2,82207	1,83422	-1,13121	3,5298	S	2	___1
2,0291	1,58336	NaN	1,3644	S	2	___1
1,48098	0,256107	NaN	0,234415	S	2	___1
1,73419	0,958714	1,00007	1,8067	S	2	___1
-2,59128	-2,0287	NaN	0,337751	S	3	___1
-0,867701	-1,46768	0,0543327	-1,15407	S	2	___1
-0,114576	-0,290312	-0,680117	-0,0473366	S	2	___1
0,327938	-0,193874	-1,17705	NaN	S	2	___1
NaN	NaN	NaN	NaN	S	4	___1
-0,760216	-2,11685	NaN	NaN	S	2	___1
NaN	NaN	NaN	NaN	S	3	___1
0,910082	0,612551	NaN	-0,406754	S	3	___1
0,643303	-0,753826	0,260231	0,653296	S	4	___1
NaN	NaN	NaN	NaN	S	4	___1

-1,96478	-2,05236	-2,9826	NaN	S	2	___1
3,1771	2,21479	3,13979	1,6531	S	2	___1
0,584373	0,252104	NaN	NaN	S	2	___1
1,65158	1,31673	2,03115	1,62193	S	3	___1
-0,985144	NaN	NaN	NaN	S	3	___1
-2,19192	-1,53145	-1,14504	-1,35883	S	2	___1
-0,423342	1,36169	-0,51178	1,52164	S	2	___1
-0,696392	NaN	NaN	NaN	S	2	___1
0,379452	-0,524914	0,999954	-0,348776	S	2	___1
NaN	NaN	NaN	NaN	S	2	___1
-0,43965	-1,08009	-1,4536	-1,26532	S	3	___1
NaN	0,0142174	NaN	-3,50394	S	3	___1
-0,337132	-1,22424	1,48407	NaN	S	2	___1
1,33218	0,516846	2,76908	0,732367	S	2	___1
-0,697739	NaN	0,853075	-1,03899	S	2	___1
1,40176	0,723204	1,26276	0,604475	S	2	___1
0,418394	-0,373487	-1,31385	0,0746784	S	2	___1
2,38521	2,08697	2,12292	1,53875	S	2	___1
0,618107	0,576258	NaN	-0,14702	S	3	___1
-1,70258	-1,61544	-0,592091	-2,29496	S	3	___1
1,3374	0,181545	NaN	NaN	S	2	___1
NaN	-2,88894	-0,421515	-2,91806	S	3	___1
0,71302	2,52701	-1,01283	2,05824	S	2	___1
NaN	NaN	NaN	NaN	S	2	___1
0,514881	1,3057	2,05369	-0,327602	S	2	___1
NaN	NaN	NaN	NaN	S	2	___1
-1,2117	-2,27327	NaN	0,39716	S	2	___1
NaN	-1,74379	0,965446	-0,292206	S	3	___1
-2,02372	3,1107	-3,22883	NaN	S	2	___1
3,35118	3,1107	3,95552	4,35314	S	2	___1
-1,72944	-1,44829	NaN	NaN	S	2	___1
-0,467117	1,46054	-1,25862	0,490818	S	3	___1
-1,56265	-0,542688	NaN	NaN	S	2	___1
NaN	NaN	NaN	NaN	S	2	___1

-1,17111	NaN	0,841303	-0,244831	S	2	___1
NaN	1,17931	1,5535	-0,0229988	S	3	___1
NaN	-0,263134	-0,602795	-0,517643	S	3	___1
NaN	NaN	NaN	NaN	S	2	___1
-0,299376	-1,25682	-1,05496	NaN	S	2	___1
NaN	-3,5245	-2,4792	-1,09793	S	2	___1
NaN	NaN	NaN	NaN	S	2	___1
-0,086235	NaN	-0,36359	NaN	S	2	___1
0,285757	-1,56842	-3,24778	-1,21796	S	2	___1
-2,44079	-2,153	-2,33636	NaN	S	2	___1
NaN	NaN	NaN	NaN	S	3	___1
0,0607567	0,363163	NaN	1,44309	S	2	___1
NaN	NaN	NaN	NaN	S	3	___1
NaN	-1,43821	-0,529879	NaN	S	2	___1
NaN	NaN	NaN	NaN	S	3	___1
-2,28357	0,153881	NaN	-0,701023	S	3	___1
-1,96262	-1,47737	-1,55924	-1,70022	S	2	___1
-2,45691	-3,14507	-2,13883	NaN	S	2	___1
NaN	NaN	0,766386	NaN	S	2	___1
2,79241	2,6868	2,72534	2,96783	S	2	___1
-0,287657	-0,596567	NaN	-0,782892	S	2	___1
NaN	NaN	NaN	NaN	S	3	___1
NaN	-1,1895	-1,54084	NaN	S	3	___1
-0,345472	-0,233158	-0,455866	-1,057	S	2	___1
1,61404	1,11203	-1,81893	0,377369	S	2	___1
NaN	NaN	NaN	NaN	S	2	___1
-0,0414391	0,175854	1,31589	-0,34997	S	2	___1
NaN	0,876305	NaN	-0,164898	S	3	___1
0,834728	0,868729	1,98839	0,168539	S	2	___1
NaN	NaN	NaN	NaN	S	2	___1
NaN	NaN	NaN	NaN	S	2	___1
NaN	NaN	NaN	NaN	S	3	___1
NaN	2,06413	NaN	NaN	S	3	___1
-2,26908	NaN	1,90091	-2,3079	S	3	___1

Polito et al. Universal ready-to-use immunotherapeutic approach for the treatment of cancer: expanded and activated polyclonal γδ memory T cells. Phosphorylation sites

NaN	NaN	NaN	NaN	S	2	___1
2,92934	2,88778	3,08722	2,09599	S	2	___1
1,08727	NaN	NaN	NaN	S	2	___1
NaN	-2,50638	-1,15237	0,78511	S	2	___1
0,427671	-0,68507	NaN	NaN	S	2	___1
0,490452	-0,0631733	0,438992	0,242714	S	3	___1
-1,53618	-3,0073	NaN	-0,800287	S	2	___1
NaN	NaN	NaN	NaN	S	2	___1
NaN	NaN	NaN	NaN	S	2	___1
-0,544142	NaN	-2,33026	NaN	S	2	___1
-2,12823	-2,40966	NaN	-0,170002	S	2	___1
NaN	NaN	-0,065321	NaN	S	3	___1
0,927839	0,0690727	NaN	0,102306	S	2	___1
NaN	-0,461441	-0,395596	0,726292	S	3	___1
-0,726345	-0,519144	-0,594864	NaN	S	2	___1
NaN	NaN	NaN	NaN	S	2	___1
-2,31112	-0,126135	0,0624466	-0,940256	S	2	___1
NaN	NaN	NaN	NaN	S	2	___1
0,00225067	NaN	-0,584881	-0,157721	S	2	___1
-2,67104	-1,9142	-1,4543	NaN	S	2	___1
NaN	NaN	NaN	NaN	S	2	___1
-1,41935	-1,63005	NaN	-1,6847	S	2	___1
-0,350189	-0,592447	NaN	-1,97889	S	2	___1
0,424345	-1,58838	NaN	1,63963	S	2	___1
NaN	-0,787708	-0,594547	-1,09818	S	2	___1
NaN	-0,318106	0,917582	NaN	S	3	___1
-1,8483	1,10627	1,63737	0,827703	S	3	___1
NaN	NaN	NaN	NaN	S	2	___1
NaN	NaN	NaN	NaN	S	3	___1
-1,75506	-1,14205	NaN	-1,23066	S	2	___1
NaN	NaN	0,466034	-0,99313	S	2	___1
-0,646671	2,26509	2,57464	1,31026	S	3	___1
0,148552	0,516951	NaN	-0,230223	S	2	___1
-0,425016	0,150881	0,287832	-0,966658	S	2	___1

NaN	1,04837	0,863985	-1,20684	S	3		___1
-0,274311	-0,363117	-0,269327	-1,47164	S	2		___1
NaN	NaN	NaN	NaN	S	2		___1
NaN	NaN	NaN	NaN	S	2		___1
-0,0896606	-0,87134	NaN	-0,341278	S	2		___1
-0,627275	-1,40588	-0,422449	-1,20998	S	2		___1
NaN	NaN	NaN	NaN	S	2		___1
-0,321938	-0,340868	-4,4537	-0,62265	S	2		___1
-0,493851	-1,74044	0,558681	-0,810192	S	2		___1
2,49327	1,57527	2,75022	1,17125	S	2		___1
-1,2952	-0,952841	NaN	-2,32364	S	2		___1
0,791103	-1,13042	-1,10279	0,334986	S	2		___1
-1,62861	-1,43026	-2,47014	-0,800613	S	2		___1
NaN	-0,20257	0,559534	-0,465477	S	3		___1
NaN	NaN	NaN	NaN	S	2		___1
NaN	NaN	NaN	NaN	S	3		___1
NaN	NaN	-1,25634	NaN	S	2		___1
0,0192738	-1,24383	-0,836863	-0,983603	S	3		___1
1,94414	2,163	0,388342	0,933413	S	2		___1
NaN	-0,734529	-0,827209	NaN	S	3		___1
0,236965	1,75602	NaN	-0,366575	S	3		___1
NaN	NaN	NaN	NaN	S	2		___1
2,906	2,63929	3,02299	2,35234	S	2		___1
-0,847298	-1,24468	-1,74569	NaN	S	2		___1
NaN	NaN	NaN	NaN	S	2		___1
NaN	NaN	NaN	NaN	S	3		___1
NaN	NaN	NaN	NaN	S	4		___1
-1,97851	-1,23564	-0,610983	NaN	S	2		___1
NaN	NaN	NaN	NaN	S	2		___1
NaN	NaN	NaN	NaN	S	2		___1
NaN	NaN	NaN	NaN	S	3		___1
NaN	-1,2171	NaN	-0,125574	S	2		___1
NaN	NaN	NaN	NaN	S	2		___1
NaN	-1,48886	NaN	NaN	S	3		___1

-0,398056	0,256107	0,201046	1,14289	S	3	___1
-0,0610409	1,85251	1,79899	2,0794	S	3	___1
NaN	0,855886	0,587374	0,930466	S	4	___1
NaN	NaN	2,74217	NaN	S	2	___1
3,12987	3,38789	NaN	3,61967	S	3	___1
NaN	-0,166225	NaN	-0,0353813	S	3	___1
-2,03276	-2,28874	NaN	-1,34556	S	2	___1
NaN	NaN	NaN	NaN	S	2	___1
0,581875	0,0886631	NaN	NaN	S	3	___1
NaN	NaN	NaN	NaN	S	3	___1
NaN	NaN	NaN	NaN	S	2	___1
-0,5061	-0,59062	-0,370886	-1,00643	S	2	___1
NaN	-0,810923	-0,85174	-1,9901	S	3	___1
2,11335	1,60604	2,87859	0,852566	S	2	___1
-1,8002	NaN	-0,345823	NaN	S	2	___1
-0,584721	-0,90984	NaN	-0,632494	S	2	___1
1,52655	1,80651	1,36867	0,298615	S	2	___1
-0,781796	-1,63989	NaN	NaN	S	2	___1
NaN	-1,38385	-0,850185	NaN	S	3	___1
NaN	-2,42867	-1,12628	NaN	S	2	___1
NaN	NaN	NaN	NaN	S	3	___1
NaN	NaN	NaN	NaN	S	4	___1
-2,1191	-2,35327	NaN	-1,56454	S	2	___1
NaN	NaN	NaN	NaN	S	2	___1
0,276575	0,360804	0,151491	-1,95145	S	2	___1
1,18615	0,622038	NaN	0,658678	S	2	___1
-1,66826	-1,41652	-0,0867634	NaN	S	2	___1
NaN	NaN	1,42791	NaN	S	2	___1
0,619211	0,782833	1,35135	0,456507	S	2	___1
-0,964375	0,247131	-0,054863	-0,602308	S	2	___1
NaN	NaN	1,16826	-3,10472	S	2	___1
NaN	NaN	NaN	NaN	S	2	___1
NaN	NaN	NaN	NaN	S	3	___1
-0,325188	-0,569096	-1,81467	-2,1017	S	2	___1

Polito et al. Universal ready-to-use immunotherapeutic approach for the treatment of cancer: expanded and activated polyclonal γδ memory T cells.
Phosphorylation sites

NaN	NaN	NaN	NaN	S	2	___1
0,111572	NaN	-2,78599	-0,617393	S	2	___1
NaN	NaN	NaN	NaN	S	2	___1
-0,688776	0,195446	1,69558	NaN	S	3	___1
5,75077	5,17341	4,01449	3,65751	S	2	___1
2,32459	3,96751	2,60526	1,50151	S	4	___1
2,6993	4,89348	3,12041	2,10696	S	4	___1
0,717474	1,26667	NaN	NaN	S	4	___1
-1,23859	-1,5517	1,72397	NaN	S	3	___1
3,0344	1,82152	0,844028	0,41482	S	2	___1
3,10202	-0,302038	1,24539	NaN	S	3	___1
4,74968	4,43984	4,39672	2,54165	S	3	___1
-2,08059	-1,08176	NaN	-0,50243	S	3	___1
2,21267	1,47996	-2,30336	1,81431	S	2	___1
2,52883	2,38014	-0,182192	1,73631	S	3	___1
NaN	NaN	NaN	NaN	S	2	___1
-0,706476	-0,747324	-0,753593	NaN	S	3	___1
-0,14632	0,5037	NaN	-0,499945	S	2	___1
2,51368	2,73803	4,00514	2,68033	S	2	___1
-0,234594	0,104401	0,191336	-0,591995	S	2	___1
NaN	NaN	NaN	NaN	S	3	___1
NaN	NaN	NaN	NaN	S	3	___1
-1,28926	-2,18649	NaN	-2,08332	S	2	___1
NaN	NaN	NaN	NaN	S	3	___1
-1,47461	-1,91715	0,273733	NaN	S	3	___1
NaN	NaN	NaN	NaN	S	3	___1
-1,53437	-1,28458	-0,383764	-0,499149	S	2	___1
-2,34108	-0,70433	-1,1396	-0,205622	S	2	___1
-1,99997	NaN	-3,83381	-0,768494	S	2	___1
1,07726	0,502792	1,39076	0,240932	S	2	___1
NaN	NaN	NaN	NaN	S	2	___1
NaN	NaN	NaN	NaN	S	2	___1
NaN	0,569788	NaN	-0,679667	S	2	___1
NaN	-2,11324	-0,885567	-2,11017	S	2	___1

Polito et al. Universal ready-to-use immunotherapeutic approach for the treatment of cancer: expanded and activated polyclonal γδ memory T cells.
Phosphorylation sites

NaN	NaN	-2,80324	NaN	S	2	___1
-1,27117	-1,69859	NaN	NaN	S	2	___1
NaN	NaN	NaN	NaN	S	2	___1
0,211697	-1,07067	NaN	-2,35386	S	2	___1
NaN	NaN	NaN	NaN	S	2	___1
-1,44017	-2,85631	-1,04768	NaN	S	2	___1
NaN	NaN	NaN	NaN	S	2	___1
-0,261711	-0,703342	-0,413555	-0,507816	S	2	___1
NaN	NaN	NaN	NaN	S	3	___1
3,41981	2,68397	2,63546	2,19318	S	3	___1
-0,763681	-1,5374	-0,214666	NaN	S	3	___1
2,48723	1,18555	1,98033	NaN	S	3	___1
0,644457	NaN	2,13633	0,0221519	S	3	___1
NaN	NaN	NaN	NaN	S	2	___1
-0,3524	0,242397	-1,36989	-0,822029	S	2	___1
1,33053	0,0731125	NaN	-1,39343	S	2	___1
-1,34143	-1,51096	-4,20586	-3,0981	S	2	___1
NaN	1,20712	-1,90862	NaN	S	2	___1
NaN	NaN	NaN	NaN	S	2	___1
-0,124212	0,217192	-3,82188	NaN	S	2	___1
-0,296097	-1,17496	0,640341	-0,924608	S	2	___1
1,64672	0,597052	NaN	-0,249475	S	2	___1
NaN	NaN	NaN	NaN	S	2	___1
1,53183	NaN	-3,58929	NaN	S	3	___1
-0,419027	-0,616766	-0,137243	NaN	S	2	___1
NaN	2,18891	NaN	NaN	S	2	___1
NaN	NaN	NaN	NaN	S	2	___1
0,225424	-1,00508	-2,27323	NaN	S	2	___1
0,893969	1,64636	NaN	1,54117	S	3	___1
0,0122471	0,696701	2,31706	-0,163612	S	3	___1
-3,33257	-0,819725	-0,861855	-2,39873	S	2	___1
1,61653	-0,140934	0,93631	-0,0981102	S	2	___1
-1,35608	-1,13278	NaN	-1,85771	S	3	___1
0,50317	0,134541	1,1929	1,36794	S	3	___1

Polito et al. Universal ready-to-use immunotherapeutic approach for the treatment of cancer: expanded and activated polyclonal γδ memory T cells.
Phosphorylation sites

NaN	NaN	NaN	NaN	S	3	___1
NaN	0,886303	NaN	NaN	S	3	___1
-1,44572	-1,95879	-0,350683	-2,1537	S	2	___1
-0,475235	-0,358349	-0,962757	1,58579	S	2	___1
NaN	NaN	NaN	NaN	S	3	___1
3,12341	2,11404	3,76375	2,2295	S	2	___1
0,338772	-0,375847	NaN	0,295374	S	2	___1
NaN	NaN	NaN	NaN	S	3	___1
-1,74934	-1,69556	-2,07105	-1,81381	S	3	___1
NaN	NaN	NaN	NaN	S	2	___1
1,44834	3,27967	1,49104	3,0127	S	3	___1
5,25467	4,1135	4,99279	3,97254	S	2	___1
NaN	NaN	-0,603998	0,0769901	S	3	___1
NaN	0,294497	1,009	NaN	S	3	___1
0,637524	1,18908	-1,75359	1,43783	S	2	___1
2,8644	3,23978	3,50084	2,77152	S	2	___1
0,742727	2,2323	2,49356	2,44029	S	3	___1
-0,460238	-0,314331	NaN	-1,78487	S	2	___1
-0,588413	-0,32559	1,36334	NaN	S	3	___1
0,437759	1,42114	-0,469336	1,18933	S	2	___1
-2,21361	-1,97589	NaN	-1,17348	S	2	___1
NaN	NaN	NaN	NaN	S	3	___1
-1,43388	-2,10761	0,207108	-1,20624	S	2	___1
NaN	NaN	NaN	NaN	S	3	___1
NaN	-1,33556	-2,51002	-1,83438	S	2	___1
-0,357319	0,726963	-1,42939	0,599041	S	2	___1
-0,139626	-0,10993	NaN	-2,12696	S	2	___1
-0,276072	-1,41446	-1,522	1,23145	S	3	___1
-0,0111656	-0,492668	NaN	NaN	S	2	___1
1,12715	0,42222	2,36659	0,553896	S	2	___1
NaN	-0,82885	NaN	NaN	S	3	___1
-1,49556	-1,28674	-1,16458	NaN	S	2	___1
-1,9679	-2,41232	-2,53732	-1,83054	S	2	___1
0,0241203	-1,04125	-0,753475	NaN	S	2	___1

-0,935461	-1,62558	-0,620863	-2,53641	S	2	___1
-1,0228	-0,963604	-1,27391	-1,16401	S	2	___1
1,54851	0,46216	0,957918	0,22827	S	2	___1
2,27255	2,56963	1,36839	1,81464	S	2	___1
NaN	-0,934515	1,65499	-3,91375	S	2	___1
0,160753	1,59708	1,82326	-0,0454998	S	3	___1
NaN	NaN	NaN	NaN	S	2	___1
1,20756	0,329479	NaN	-1,33046	S	2	___1
1,98085	1,88517	2,33958	NaN	S	3	___1
-1,70371	-2,56609	-0,0706387	NaN	S	2	___1
0,833055	1,01991	-1,07644	-1,71714	S	2	___1
NaN	0,95598	-0,677061	0,0243645	S	2	___1
NaN	NaN	NaN	NaN	S	2	___1
NaN	NaN	NaN	NaN	S	2	___1
1,10434	-0,0743027	NaN	-1,14104	S	2	___1
NaN	-0,0693626	NaN	-0,0574913	S	4	___1
NaN	NaN	NaN	NaN	S	2	___1
-0,818354	-1,02992	NaN	-1,15021	S	3	___1
0,296911	0,505999	NaN	-0,377975	S	3	___1
NaN	1,28118	NaN	NaN	S	2	___1
-0,439226	-0,850826	-0,87789	-1,41914	S	2	___1
NaN	NaN	NaN	NaN	S	2	___1
NaN	-3,25445	1,27704	NaN	S	3	___1
NaN	-0,576197	NaN	-1,14616	S	3	___1
-0,147932	-0,680325	0,502897	-1,26487	S	2	___1
NaN	NaN	NaN	NaN	S	3	___1
2,81356	2,58727	NaN	2,22289	S	4	___1
-0,498346	-0,5352	-1,30411	-0,176897	S	2	___1
-0,529814	0,109961	-2,27301	-1,26071	S	2	___1
2,81499	1,27515	1,0219	-0,386402	S	2	___1
NaN	1,47006	NaN	-1,51168	S	2	___1
0,441923	0,519756	0,0950623	-1,0023	S	3	___1
0,899645	NaN	NaN	1,04513	S	2	___1
NaN	1,29342	-0,147518	-1,25528	S	2	___1

NaN	NaN	NaN	NaN	S	2	___1
2,1083	1,87161	2,43115	2,26862	S	2	___1
0,749956	1,18332	-0,237507	-0,103939	S	2	___1
2,36954	2,34221	1,54484	1,87873	S	2	___1
1,6518	3,57244	3,0033	3,03959	S	2	___1
NaN	0,313406	-0,252954	-0,386065	S	2	___1
NaN	NaN	-2,2877	NaN	S	2	___1
1,31822	3,63655	3,70847	1,00461	S	2	___1
-0,268248	NaN	NaN	NaN	S	2	___1
NaN	NaN	NaN	NaN	S	2	___1
-0,415049	0,00056839	NaN	-0,715914	S	3	___1
-1,2293	NaN	NaN	NaN	S	3	___1
NaN	NaN	NaN	NaN	S	3	___1
NaN	NaN	NaN	NaN	S	2	___1
-1,6385	-1,56925	0,710163	-0,913845	S	2	___1
NaN	NaN	NaN	NaN	S	2	___1
-0,841139	-0,366339	NaN	-0,989359	S	2	___1
-1,61136	-0,934168	1,82701	-1,81505	S	2	___1
-0,678646	-1,28279	NaN	-1,77433	S	2	___1
-0,255577	-0,371624	-0,789549	-0,380474	S	2	___1
-1,05218	-2,21732	-1,39049	-2,95859	S	2	___1
NaN	NaN	NaN	NaN	S	2	___1
-0,683813	NaN	NaN	NaN	S	2	___1
-1,7739	NaN	-3,46628	-1,02229	S	2	___1
NaN	NaN	NaN	NaN	S	3	___1
-0,804792	0,171284	NaN	1,20771	S	3	___1
NaN	NaN	NaN	NaN	S	3	___1
NaN	NaN	NaN	NaN	S	3	___1
-0,323454	-1,32823	NaN	-0,633619	S	2	___1
-0,930038	-0,938999	-1,74772	-0,460892	S	2	___1
-0,915392	-2,44997	NaN	-2,20354	S	2	___1
NaN	NaN	NaN	NaN	S	3	___1
-0,747421	-1,45767	-2,1004	-0,995977	S	2	___1
1,00967	-1,0235	NaN	0,140295	S	2	___1

0,738226	-0,465309	0,746269	0,00145149	S	2	___1
2,41351	2,11721	0,949144	3,61551	S	3	___1
-2,28225	-2,16281	-1,10214	-0,803513	S	2	___1
-2,28225	-2,16281	-1,10214	-0,803513	S	2	___1
NaN	-0,685556	NaN	-0,492443	S	3	___1
-0,846113	0,869184	-2,54967	0,0134296	S	3	___1
NaN	NaN	NaN	NaN	S	2	___1
-2,87499	-5,8991	-4,71909	-1,34353	S	2	___1
-1,54758	-1,37504	NaN	NaN	S	2	___1
0,906279	1,18315	-1,36635	0,190588	S	3	___1
-0,832476	-0,259682	-0,346741	0,52063	S	2	___1
2,63778	1,40998	0,0361309	1,76925	S	2	___1
3,48995	2,48836	1,80406	2,58196	S	2	___1
-1,2113	-2,09911	-1,5403	NaN	S	2	___1
NaN	NaN	NaN	NaN	S	3	___1
-1,97387	-2,3261	0,310738	-0,705484	S	2	___1
NaN	NaN	NaN	NaN	S	2	___1
-1,11871	NaN	1,5296	-0,447573	S	2	___1
0,96287	1,48663	0,40181	NaN	S	2	___1
-2,35218	-2,10546	NaN	NaN	S	2	___1
NaN	0,600454	NaN	NaN	S	3	___1
NaN	NaN	NaN	NaN	S	3	___1
NaN	NaN	NaN	NaN	S	3	___1
-0,366356	0,918497	NaN	1,8512	S	2	___1
1,55005	-0,0835075	NaN	NaN	S	3	___1
2,41182	2,02978	-0,11701	1,59256	S	2	___1
-1,44497	NaN	-1,8039	NaN	S	2	___1
2,47264	1,04128	3,16567	0,971779	S	3	___1
NaN	NaN	NaN	NaN	S	2	___1
-1,40754	-2,11827	NaN	1,07334	S	2	___1
-0,302492	-0,550106	1,09236	-0,213911	S	3	___1
NaN	0,187283	-0,232	0,142822	S	3	___1
3,24193	2,99776	2,59693	2,27802	S	2	___1
NaN	-2,35975	-1,47453	-3,2088	S	2	___1

Polito et al. Universal ready-to-use immunotherapeutic approach for the treatment of cancer: expanded and activated polyclonal γδ memory T cells.
Phosphorylation sites

-1,77058	-2,17878	NaN	-1,18761	S	2	___1
-0,939575	-1,68441	NaN	-1,13244	S	2	___1
NaN	-0,795317	NaN	-1,7898	S	2	___1
NaN	-0,2686	-1,63927	-1,39128	S	2	___1
NaN	NaN	NaN	NaN	S	2	___1
NaN	NaN	NaN	NaN	S	2	___1
0,606024	0,502096	NaN	NaN	S	2	___1
-1,02506	-0,815317	NaN	0,232164	S	2	___1
NaN	0,126995	NaN	0,464949	S	3	___1
NaN	NaN	NaN	NaN	S	2	___1
-0,534674	NaN	-0,478418	-0,33633	S	2	___1
-1,33337	-2,73	-2,38878	-1,02209	S	2	___1
NaN	NaN	NaN	NaN	S	2	___1
-1,01943	-0,538389	NaN	-0,869602	S	2	___1
-0,015852	-1,00007	0,332932	-0,293325	S	3	___1
0,602743	0,865362	0,701315	3,06525	S	2	___1
1,4577	1,56582	2,10615	3,69529	S	3	___1
NaN	-0,221535	-0,135197	1,39081	S	3	___1
-1,66639	-1,84325	-1,03794	NaN	S	2	___1
-0,0909519	1,26405	NaN	1,93715	S	4	___1
NaN	NaN	NaN	NaN	S	2	___1
-0,361273	0,799479	NaN	-0,216631	S	2	___1
1,06114	0,704674	NaN	1,55495	S	2	___1
-1,0696	NaN	-0,0849342	-1,8555	S	2	___1
-0,0213623	-0,954866	2,03757	1,24801	S	2	___1
NaN	0,494854	1,55311	0,341064	S	3	___1
-1,95844	-1,15005	-0,694319	-0,908163	S	2	___1
-1,5095	-1,38417	0,460733	NaN	S	2	___1
0,933056	2,0055	NaN	0,325932	S	2	___1
3,57075	3,23133	1,85301	3,29755	S	2	___1
-1,44318	NaN	-1,72315	-2,64106	S	2	___1
0,959951	1,03752	0,432539	1,16248	S	2	___1
0,214148	-1,09629	-0,874685	-0,266985	S	2	___1
NaN	0,569174	NaN	0,393793	S	2	___1

NaN	1,52213	0,283918	1,777	S	3	___1
-0,617727	3,01333	NaN	0,66148	S	3	___1
NaN	-0,0994167	NaN	NaN	S	3	___1
NaN	0,399876	0,610241	-0,262623	S	3	___1
NaN	1,01305	0,718107	1,34027	S	2	___1
NaN	0,409664	-1,52958	-0,707052	S	2	___1
NaN	0,736366	-0,280054	-0,692972	S	2	___1
-1,2117	-1,65044	-1,37055	-1,86896	S	3	___1
2,34554	1,98952	2,48971	NaN	S	2	___1
-0,926807	-0,412199	-1,28594	-1,34478	S	3	___1
4,57547	2,43147	-0,639885	0,966515	S	2	___1
0,849993	-0,602884	NaN	1,20984	S	3	___1
1,79706	0,962646	1,51867	0,63739	S	2	___1
-1,25943	-2,71923	-1,94034	NaN	S	2	___1
0,203144	0,513557	-0,831179	0,451067	S	3	___1
2,7411	0,860027	-1,50446	2,24154	S	4	___1
2,12904	2,54374	NaN	1,04251	S	3	___1
NaN	NaN	NaN	NaN	S	2	___1
NaN	NaN	NaN	NaN	S	2	___1
-0,961201	-1,98901	-2,32349	-2,01991	S	2	___1
NaN	NaN	NaN	NaN	S	2	___1
-0,288755	1,45499	1,28814	NaN	S	3	___1
-1,59212	-2,47103	-1,20844	NaN	S	2	___1
0,393368	-0,332541	-0,33448	-1,37661	S	2	___1
5,11679	3,43343	3,39927	1,97238	S	2	___1
0,531685	0,529976	0,638773	NaN	S	2	___1
1,07448	1,14239	0,696728	NaN	S	2	___1
1,08235	1,15946	-1,9801	-0,714067	S	2	___1
1,34942	-0,312164	0,268505	-1,11068	S	2	___1
-0,467836	2,17585	2,64345	0,655914	S	2	___1
-0,31567	-4,77945	-0,351366	-1,90237	S	3	___1
NaN	NaN	NaN	NaN	S	2	___1
-1,24515	-1,21325	0,400717	NaN	S	2	___1
-0,839649	-0,0634899	-2,65914	-1,39824	S	2	___1

Polito et al. Universal ready-to-use immunotherapeutic approach for the treatment of cancer: expanded and activated polyclonal γδ memory T cells.
Phosphorylation sites

0,497242	-1,24703	NaN	NaN	S	2	___1
-0,508562	-0,990278	-1,62154	-2,15454	S	2	___1
NaN	-2,63602	2,12724	NaN	S	3	___1
NaN	NaN	NaN	NaN	S	2	___1
-2,91875	-3,13272	-2,19986	NaN	S	2	___1
NaN	NaN	NaN	NaN	S	2	___1
0,515245	0,975	0,568022	1,07446	S	2	___1
1,55276	1,65449	1,77021	-0,167343	S	2	___1
-1,06872	-0,607382	-2,45667	NaN	S	2	___1
-1,48262	-0,808929	NaN	NaN	S	2	___1
-0,40947	-0,562813	-1,70923	-1,51362	S	2	___1
2,53739	1,99354	2,23237	2,36162	S	2	___1
NaN	NaN	NaN	NaN	S	2	___1
-0,0213623	0,309803	NaN	-0,588751	S	2	___1
3,93637	3,2294	4,519	3,58397	S	2	___1
NaN	NaN	NaN	NaN	S	3	___1
NaN	-0,355534	NaN	0,498581	S	2	___1
1,62509	2,37985	2,96354	3,06738	S	3	___1
3,60356	3,20881	3,70933	1,79081	S	2	___1
NaN	NaN	NaN	NaN	S	2	___1
0,746469	1,44026	1,42522	1,81425	S	3	___1
NaN	0,0205078	0,407612	-0,167185	S	2	___1
0,053997	1,46886	1,74856	1,30211	S	2	___1
0,749853	1,48652	NaN	1,3741	S	3	___1
-0,413433	-0,62504	-0,760437	-0,0501003	S	2	___1
NaN	NaN	0,865009	NaN	S	3	___1
NaN	NaN	NaN	NaN	S	2	___1
NaN	NaN	NaN	NaN	S	2	___1
NaN	NaN	NaN	NaN	S	3	___1
2,50528	2,27781	0,890091	0,765265	S	2	___1
-0,103888	-1,0668	-2,47298	-1,81917	S	2	___1
1,19657	0,532806	-0,100447	-1,74797	S	2	___1
2,06358	1,00552	NaN	NaN	S	2	___1
1,47326	1,11721	-2,32395	-1,33861	S	2	___1

Polito et al. Universal ready-to-use immunotherapeutic approach for the treatment of cancer: expanded and activated polyclonal γδ memory T cells.
Phosphorylation sites

NaN	NaN	NaN	NaN	S	2	___1
0,671574	-0,493416	0,625996	-0,211765	S	3	___1
0,708532	-0,120274	1,20184	NaN	S	2	___1
-0,413248	NaN	NaN	NaN	S	2	___1
3,72756	3,9611	NaN	2,75065	S	2	___1
-2,965	-1,93049	-1,00969	-1,54575	S	2	___1
3,48349	3,12197	4,48718	3,59289	S	2	___1
NaN	NaN	NaN	NaN	S	2	___1
0,942011	1,46461	NaN	0,914646	S	3	___1
-0,86511	-0,0747814	-0,905046	0,317554	S	3	___1
-0,86511	-0,0747814	-0,905046	0,317554	S	2	___1
NaN	NaN	NaN	NaN	S	2	___1
NaN	NaN	NaN	NaN	S	3	___1
NaN	NaN	NaN	NaN	S	2	___1
-1,18925	NaN	-0,738991	NaN	S	2	___1
NaN	NaN	NaN	NaN	S	2	___1
-0,401257	0,119549	-0,787458	-1,69711	S	2	___1
-0,264622	0,641846	-1,30416	NaN	S	2	___1
1,56757	NaN	1,85443	1,30581	S	2	___1
NaN	NaN	NaN	NaN	S	3	___1
1,39334	NaN	1,84759	-0,7386	S	2	___1
NaN	NaN	NaN	NaN	S	3	___1
NaN	NaN	-0,81881	NaN	S	3	___1
NaN	NaN	NaN	NaN	S	4	___1
0,334766	0,7089	NaN	NaN	S	2	___1
-0,869471	-0,54944	-1,40638	0,11272	S	3	___1
-0,185303	2,59945	2,76433	3,01447	S	3	___1
NaN	-2,21901	-2,34402	-2,8335	S	3	___1
2,68186	NaN	NaN	3,31886	S	3	___1
-1,80467	-1,46163	NaN	-1,0854	S	2	___1
-1,27729	-0,71748	-2,1958	-0,712454	S	2	___1
NaN	0,632343	-0,796768	NaN	S	3	___1
-0,792559	-1,23252	-3,6538	-0,400711	S	2	___1
NaN	0,103342	0,000720978	-0,411264	S	3	___1

-2,71256	-2,63602	NaN	-2,84207	S	2	___1
NaN	NaN	NaN	NaN	S	2	___1
-1,31214	1,93651	0,644413	2,29864	S	3	___1
NaN	NaN	NaN	NaN	S	2	___1
1,76683	0,575647	NaN	NaN	S	2	___1
-1,25479	-0,404955	NaN	-1,12557	S	2	___1
1,50364	0,723204	NaN	0,502308	S	3	___1
3,93517	3,09126	0,228361	3,05302	S	3	___1
-3,061	-2,89119	-1,722	NaN	S	2	___1
NaN	NaN	NaN	NaN	S	2	___1
NaN	NaN	NaN	NaN	S	2	___1
NaN	NaN	NaN	NaN	S	2	___1
-0,422319	-0,148811	1,32576	-1,28437	S	3	___1
NaN	NaN	NaN	NaN	S	3	___1
-1,81927	-1,49784	NaN	NaN	S	2	___1
NaN	NaN	NaN	NaN	S	3	___1
-0,119085	-0,912691	0,476358	NaN	S	2	___1
-1,17941	-1,24274	-1,58862	-1,4972	S	2	___1
-1,34662	-2,3149	NaN	NaN	S	2	___1
-0,501432	-0,502514	NaN	NaN	S	2	___1
1,88101	0,392792	NaN	-1,60174	S	2	___1
0,859076	-1,01633	0,32102	-0,959213	S	2	___1
NaN	NaN	NaN	NaN	S	2	___1
NaN	2,09624	2,0723	1,58626	S	2	___1
NaN	-1,08843	-4,40196	-1,86128	S	2	___1
NaN	NaN	NaN	NaN	S	2	___1
-2,06179	-0,639372	NaN	-2,0634	S	2	___1
NaN	-2,15304	-2,70266	NaN	S	2	___1
NaN	-0,322269	-0,282883	-1,20376	S	3	___1
-1,26067	-2,06636	-0,988432	NaN	S	2	___1
0,368303	-0,160114	1,17827	NaN	S	2	___1
2,11423	2,35838	1,69522	-0,445179	S	2	___1
1,07858	0,858273	-0,866472	NaN	S	2	___1
-2,42251	NaN	NaN	NaN	S	2	___1

0,268948	0,00759697	0,383211	-2,51055	S	2	___1
NaN	0,226873	-2,63691	-1,78237	S	2	___1
-0,462193	-0,871618	-1,3164	-0,77943	S	2	___1
NaN	1,45576	0,895981	0,827969	S	3	___1
0,438705	0,760906	1,87722	-0,339647	S	2	___1
NaN	-0,409781	-1,59149	-1,02381	S	2	___1
-1,96046	NaN	-1,44141	NaN	S	3	___1
NaN	NaN	NaN	NaN	S	3	___1
NaN	NaN	NaN	NaN	S	2	___1
NaN	NaN	NaN	NaN	S	3	___1
NaN	NaN	-2,19282	-0,754122	S	3	___1
NaN	NaN	NaN	NaN	S	2	___1
NaN	NaN	NaN	NaN	S	2	___1
NaN	NaN	NaN	-1,28135	S	3	___1
3,42582	2,78607	3,86635	2,23881	S	2	___1
-1,55662	-2,72197	-2,11031	0,0793419	S	2	___1
NaN	-0,235655	0,106436	0,0420837	S	3	___1
-1,22744	-1,0216	-2,50764	1,43636	S	2	___1
-0,607502	-1,23512	NaN	-0,472416	S	2	___1
NaN	NaN	NaN	NaN	S	3	___1
NaN	-0,4123	2,96652	0,282007	S	3	___1
-0,948574	-0,147802	NaN	NaN	S	2	___1
-2,80763	-1,63838	NaN	-3,24974	S	2	___1
0,39086	1,09655	0,256008	0,157156	S	4	___1
-0,347454	-0,799795	NaN	-1,07713	S	2	___1
2,68918	2,02655	-0,474016	0,545607	S	3	___1
NaN	NaN	NaN	0,511868	S	2	___1
1,4398	1,0436	-0,734377	0,523924	S	2	___1
NaN	NaN	-0,435987	NaN	S	2	___1
-0,811283	-0,382648	-0,887589	-0,982029	S	2	___1
-0,347454	-0,799795	NaN	-1,07713	S	2	___1
NaN	0,765327	1,89548	0,359678	S	3	___1
-0,395683	1,6154	2,43382	-0,145231	S	3	___1
1,54626	0,623367	1,46607	-0,34309	S	2	___1

4,82067	4,56602	5,24068	3,32857	S	2	___1
0,6527	0,24662	-1,01237	-0,478571	S	3	___1
NaN	NaN	NaN	NaN	S	3	___1
0,751911	-0,591078	-0,409624	-0,804617	S	2	___1
0,301641	0,166903	0,948542	-0,643867	S	2	___1
-2,11158	-0,497368	NaN	-2,37181	S	2	___1
1,49886	NaN	0,782846	NaN	S	2	___1
4,11817	3,16029	4,5619	2,98089	S	2	___1
4,83044	3,6754	1,77055	2,97008	S	2	___1
-0,244246	-0,407969	-2,30577	-1,98388	S	2	___1
3,08888	2,19945	1,12164	1,37256	S	2	___1
NaN	NaN	NaN	NaN	S	2	___1
NaN	NaN	NaN	NaN	S	2	___1
NaN	NaN	NaN	NaN	S	3	___1
NaN	NaN	NaN	NaN	S	3	___1
NaN	NaN	NaN	NaN	S	2	___1
NaN	NaN	NaN	NaN	S	2	___1
NaN	NaN	NaN	NaN	S	2	___1
-1,58057	-0,480455	-0,466311	-2,22353	S	2	___1
1,37489	0,887655	-0,13604	0,441509	S	2	___1
-0,530966	-0,360973	-0,310123	NaN	S	2	___1
-2,29684	NaN	-3,29468	-0,679066	S	2	___1
NaN	-1,84717	NaN	NaN	S	3	___1
-1,62113	-3,57961	-0,188093	NaN	S	3	___1
NaN	-0,126797	1,47614	-0,000234604	S	3	___1
NaN	NaN	NaN	NaN	S	3	___1
NaN	NaN	NaN	NaN	S	3	___1
-0,63554	NaN	NaN	NaN	S	2	___1
-1,389	-2,5877	NaN	-1,68666	S	2	___1
-1,80503	NaN	NaN	NaN	S	2	___1
1,16187	1,27906	1,11693	-0,956757	S	2	___1
NaN	NaN	NaN	NaN	S	2	___1
NaN	NaN	NaN	NaN	S	2	___1
-0,116077	-0,610041	-0,174213	-1,31214	S	3	___1

NaN	-0,555443	0,597038	-1,84899	S	2	___1
-1,99388	-2,35017	NaN	-2,85381	S	2	___1
-0,437302	-1,54572	NaN	-0,8929	S	2	___1
-1,73566	NaN	NaN	1,89696	S	2	___1
1,87309	0,284948	1,31102	0,742342	S	2	___1
NaN	-1,81473	-3,747	-1,86222	S	3	___1
0,132553	0,714962	1,13145	1,20537	S	2	___1
-0,131102	-0,655041	NaN	1,04377	S	2	___1
NaN	-0,582312	-1,50598	-0,963091	S	3	___1
-0,187511	-0,212635	-0,366919	-1,11017	S	2	___1
-1,10463	-2,2182	NaN	-1,39775	S	2	___1
-0,675545	-0,0676937	NaN	-1,99598	S	2	___1
2,04178	2,737	1,64661	3,35039	S	3	___1
NaN	NaN	NaN	NaN	S	2	___1
-1,87321	-3,65963	-2,20318	0,160137	S	2	___1
-0,0170784	-0,215273	-0,647303	0,12727	S	2	___1
NaN	NaN	NaN	NaN	S	2	___1
1,0036	1,34737	0,895981	-0,263411	S	4	___1
NaN	-2,58311	-1,70233	NaN	S	2	___1
-1,55215	NaN	-1,2665	-3,49803	S	2	___1
-0,2978	NaN	-1,75046	-0,412573	S	2	___1
NaN	-1,33149	NaN	-0,413883	S	3	___1
NaN	NaN	NaN	NaN	S	2	___1
NaN	-3,46571	-2,00044	-0,469753	S	3	___1
-1,22825	-0,265865	-1,77701	-3,12354	S	2	___1
-0,0921326	0,245022	NaN	-1,2541	S	2	___1
-1,60239	-1,85806	-1,63312	0,0665817	S	2	___1
-2,76525	-3,03723	-0,423416	1,03785	S	3	___1
-1,07879	-0,865528	NaN	-2,09678	S	2	___1
NaN	-1,37274	-2,5969	NaN	S	2	___1
NaN	NaN	NaN	NaN	S	2	___1
-1,14981	-0,308125	-2,13504	-1,09688	S	3	___1
NaN	-0,0592194	-0,935839	NaN	S	3	___1
NaN	-2,1916	-0,204439	0,727423	S	3	___1

Polito et al. Universal ready-to-use immunotherapeutic approach for the treatment of cancer: expanded and activated polyclonal γδ memory T cells.
Phosphorylation sites

-0,957092	0,989655	-2,59325	0,157072	S	3	___1
-0,591074	NaN	-0,119562	0,483364	S	3	___1
-1,28032	0,486492	NaN	-0,0249271	S	4	___1
-0,501579	-0,896376	-3,55459	-0,204	S	2	___1
1,01611	-0,186697	NaN	2,79979	S	2	___1
1,77587	1,14763	-0,768026	NaN	S	2	___1
-1,81098	NaN	-3,39651	-0,32197	S	3	___1
-1,4747	-3,79405	-2,69146	1,35719	S	3	___1
-0,0329418	-0,254879	NaN	-1,45786	S	2	___1
-1,57148	-2,02351	NaN	-1,29525	S	2	___1
-3,2632	-2,6795	NaN	-1,86068	S	2	___1
-2,59941	-2,11942	NaN	-1,34943	S	2	___1
1,40752	0,332853	NaN	0,161016	S	2	___1
-0,418009	-1,25901	1,53724	-1,34812	S	3	___1
-0,760098	-0,799269	NaN	-0,495974	S	2	___1
-1,80395	-3,55874	NaN	-1,02734	S	2	___1
0,0168438	-1,53318	-0,023037	NaN	S	2	___1
-1,41574	-1,4558	-2,62463	-2,49502	S	2	___1
NaN	0,369513	-2,53678	-2,19344	S	3	___1
-2,08635	-1,54583	-2,35994	NaN	S	2	___1
-2,3671	-2,34558	-2,62664	-1,32646	S	2	___1
NaN	-0,863596	NaN	-0,052309	S	3	___1
NaN	-0,574842	-2,28827	0,578686	S	2	___1
-1,45187	-2,27488	-3,13396	NaN	S	2	___1
NaN	-2,30767	0,0998268	NaN	S	2	___1
NaN	NaN	NaN	NaN	S	3	___1
-0,47456	-0,817986	NaN	0,364107	S	2	___1
NaN	1,04099	-0,310123	-0,101826	S	3	___1
-1,99471	NaN	NaN	NaN	S	2	___1
-0,985144	0,620461	NaN	-0,582844	S	2	___1
NaN	-0,289755	NaN	-1,42976	S	3	___1
-0,790819	-0,175184	-2,08047	-0,818142	S	3	___1
-1,45919	-1,10702	-2,61761	NaN	S	2	___1
1,72887	1,26607	2,86315	0,895004	S	2	___1

-0,315714	0,204224	-0,2663	0,695999	S	2	___1
NaN	NaN	NaN	NaN	S	2	___1
-2,35106	-1,59053	1,97974	NaN	S	2	___1
-1,18121	NaN	-0,0450325	NaN	S	2	___1
-2,20183	-2,50625	-1,13832	NaN	S	2	___1
NaN	0,367929	-2,05086	0,112938	S	3	___1
0,502216	0,497004	-0,743076	-1,06492	S	2	___1
NaN	-0,672935	0,513468	NaN	S	2	___1
1,35836	1,17807	0,991434	0,836782	S	2	___1
NaN	NaN	NaN	NaN	S	2	___1
-3,2144	NaN	-2,44826	-2,98308	S	2	___1
NaN	0,215494	NaN	-0,649958	S	2	___1
NaN	NaN	NaN	NaN	S	3	___1
0,0956764	0,223242	NaN	0,776384	S	2	___1
-2,10389	-1,05838	NaN	-0,926344	S	2	___1
-2,96931	-2,61526	NaN	-1,23485	S	2	___1
-0,0396919	-0,254248	0,72629	NaN	S	2	___1
-2,53668	-2,88721	-2,99403	NaN	S	2	___1
0,427311	0,341373	0,0443249	-1,13439	S	2	___1
-0,715437	0,0681324	NaN	NaN	S	2	___1
NaN	NaN	NaN	NaN	S	3	___1
NaN	NaN	NaN	NaN	S	2	___1
NaN	NaN	NaN	NaN	S	3	___1
NaN	-0,789148	NaN	-0,0655346	S	3	___1
0,805824	0,673935	1,22123	0,154318	S	2	___1
0,947895	0,750536	0,384905	1,11168	S	2	___1
0,547155	0,411375	1,11445	1,05497	S	3	___1
NaN	NaN	NaN	NaN	S	2	___1
-0,407997	-1,7678	NaN	-1,06208	S	2	___1
-1,64228	-1,01418	1,67903	1,88057	S	2	___1
0,390516	-0,0471878	NaN	0,803616	S	2	___1
-1,84157	NaN	-2,9516	-0,211956	S	2	___1
-0,823189	-2,14561	-1,4138	-0,320038	S	3	___1
-0,939575	-1,54313	NaN	0,623713	S	2	___1

Polito et al. Universal ready-to-use immunotherapeutic approach for the treatment of cancer: expanded and activated polyclonal γδ memory T cells.
Phosphorylation sites

-1,03582	-2,17481	NaN	0,213779	S	3	___1
-0,369978	-1,76	-2,33518	0,696476	S	2	___1
-1,53678	-1,78753	0,603424	-1,21747	S	2	___1
1,80858	2,82167	0,976254	2,10522	S	2	___1
0,440596	-0,689463	-1,62901	1,97643	S	2	___1
1,48234	1,58437	-0,553288	2,79122	S	2	___1
-1,56081	-1,6393	-1,61426	-1,70419	S	2	___1
-0,600708	-0,0872078	NaN	-0,170424	S	3	___1
-1,225	-0,617462	-2,45794	-1,4526	S	2	___1
-1,69965	-2,23808	NaN	-1,45093	S	2	___1
-2,68113	-0,693134	0,746269	NaN	S	3	___1
2,91742	1,29026	NaN	NaN	S	2	___1
-1,5096	NaN	-2,14736	-1,02338	S	2	___1
2,42084	2,42061	0,967234	2,08183	S	2	___1
NaN	NaN	NaN	NaN	S	3	___1
2,42084	2,42061	0,967234	2,08183	S	2	___1
0,700325	-0,785093	NaN	0,789183	S	2	___1
-0,868649	-0,843056	-0,223932	NaN	S	2	___1
-2,14874	-2,97369	-0,635612	NaN	S	2	___1
-1,48563	-1,2274	NaN	NaN	S	2	___1
2,33184	1,85312	NaN	0,914894	S	2	___1
NaN	NaN	NaN	NaN	S	3	___1
NaN	NaN	NaN	NaN	S	2	___1
0,130655	1,61148	0,167225	0,422041	S	2	___1
-1,58805	-1,2203	-0,355499	-2,20106	S	2	___1
NaN	-0,0336246	1,79752	2,11439	S	4	___1
0,0860138	1,36231	NaN	-0,456867	S	3	___1
-1,33346	-1,4732	0,0684528	-1,057	S	3	___1
NaN	-0,952166	-1,82123	-1,403	S	3	___1
NaN	NaN	NaN	-0,142851	S	2	___1
0,355713	-0,00695229	1,53972	NaN	S	2	___1
-0,9848	-1,52384	-1,76028	-1,87136	S	2	___1
0,302794	NaN	1,20295	NaN	S	2	___1
0,936989	-0,242453	1,49525	NaN	S	2	___1

-0,202185	-1,22942	-0,669601	NaN	S	2	___1
-0,792919	0,114168	-1,5751	0,0181789	S	3	___1
0,795403	0,343048	NaN	1,03075	S	2	___1
-1,28377	-1,0119	-0,125237	-1,80675	S	2	___1
0,955759	-0,0151253	NaN	-1,0798	S	2	___1
-4,42158	NaN	0,386332	NaN	S	2	___1
NaN	0,641895	2,03405	-0,398857	S	3	___1
-0,504576	-0,848505	-3,07953	NaN	S	2	___1
0,319326	0,363989	NaN	NaN	S	2	___1
NaN	NaN	NaN	NaN	S	2	___1
NaN	NaN	NaN	NaN	S	2	___1
-1,34345	-0,80138	NaN	-0,719149	S	2	___1
2,95976	1,96853	NaN	2,40994	S	2	___1
-1,99195	-0,997871	0,0317669	1,07245	S	2	___1
-1,23394	-1,41928	NaN	-0,279413	S	2	___1
1,15315	0,191204	NaN	-0,991777	S	3	___1
NaN	NaN	NaN	NaN	S	2	___1
-1,68554	-1,59197	NaN	-0,755583	S	2	___1
NaN	NaN	NaN	NaN	S	2	___1
-2,46318	-0,683851	-0,559689	NaN	S	2	___1
NaN	NaN	NaN	NaN	S	2	___1
-2,77134	-3,46152	NaN	-2,42327	S	2	___1
-2,1293	-1,87453	NaN	-1,07411	S	2	___1
NaN	-2,47651	-1,4755	NaN	S	3	___1
4,40745	3,93036	2,41832	3,79013	S	2	___1
-1,55571	NaN	-1,55862	NaN	S	3	___1
-0,323107	1,61851	2,00483	0,460035	S	2	___1
0,136971	0,800739	NaN	-2,47339	S	2	___1
-1,11398	0,150677	NaN	-1,65393	S	3	___1
2,10749	3,69922	3,58953	1,31648	S	3	___1
NaN	NaN	NaN	NaN	S	2	___1
NaN	NaN	NaN	NaN	S	2	___1
NaN	NaN	NaN	NaN	S	2	___1
NaN	-0,983339	-1,33301	-1,29456	S	2	___1

NaN	-4,31679	-0,177158	NaN	S	2	___1
1,83814	1,73859	NaN	-2,08521	S	3	___1
NaN	NaN	NaN	NaN	S	2	___1
0,294428	2,6855	3,08302	1,49961	S	3	___1
NaN	NaN	NaN	NaN	S	2	___1
-1,9796	-1,48754	-1,26325	-1,46192	S	3	___1
NaN	NaN	NaN	NaN	S	2	___1
-0,706928	-0,240749	1,29004	0,0937481	S	3	___1
NaN	NaN	0,337803	-1,42736	S	2	___1
NaN	-1,16112	-0,787178	-1,48259	S	2	___1
-0,660078	-2,38716	-1,99914	-1,47137	S	2	___1
-2,92748	-2,78865	NaN	-2,39208	S	2	___1
NaN	NaN	1,16016	NaN	S	3	___1
3,61655	3,16317	4,29107	2,93767	S	2	___1
-2,07367	-3,24701	-2,16062	0,286873	S	2	___1
0,966543	0,983267	-1,82502	0,526903	S	3	___1
0,945326	-0,836004	-1,90496	0,506468	S	2	___1
NaN	NaN	NaN	NaN	S	2	___1
NaN	NaN	NaN	NaN	S	2	___1
NaN	NaN	NaN	NaN	S	4	___1
NaN	NaN	-0,680004	-1,74475	S	2	___1
-1,01515	-0,974064	NaN	-0,3657	S	2	___1
3,28753	1,8853	2,22437	2,19661	S	2	___1
-2,05304	-1,8874	NaN	-2,4691	S	2	___1
3,29062	2,98269	0,457363	1,41201	S	2	___1
NaN	0,353527	1,6423	0,622511	S	4	___1
NaN	-0,906284	-0,242067	-3,32066	S	4	___1
NaN	-0,902313	-0,605944	-1,77738	S	3	___1
1,84317	1,05513	-0,580976	0,740101	S	2	___1
0,179445	-0,852194	-1,02699	0,337528	S	2	___1
-1,34988	0,829601	NaN	1,17737	S	2	___1
2,13323	2,50968	0,198473	2,48382	S	2	___1
0,604156	0,889742	NaN	-1,8723	S	3	___1
-0,971968	1,74521	1,52597	1,61009	S	4	___1

-2,27137	-1,40722	-0,931303	-1,80486	S	2	___1
NaN	NaN	-0,235697	NaN	S	2	___1
0,755323	2,05272	3,29498	1,69726	S	2	___1
-2,62827	-1,94568	-5,27483	NaN	S	2	___1
-0,11529	-0,428936	-1,07345	0,0579453	S	2	___1
-1,48708	-0,872171	-2,15595	-0,411669	S	2	___1
NaN	NaN	NaN	NaN	S	2	___1
-0,843872	-0,767181	-0,994633	0,676493	S	2	___1
-2,33042	-1,54136	NaN	-1,15892	S	3	___1
0,544237	2,16935	-0,125896	-0,843685	S	2	___1
0,360741	1,05079	-0,809277	1,38607	S	3	___1
-1,78938	-1,8018	-1,25762	-0,678242	S	2	___1
3,69773	5,10487	0,784199	2,37915	S	2	___1
NaN	NaN	NaN	NaN	S	2	___1
-1,54546	-1,61236	NaN	NaN	S	2	___1
-0,398832	-0,782875	0,102322	-1,01545	S	2	___1
-1,53036	NaN	NaN	NaN	S	2	___1
NaN	NaN	NaN	NaN	S	2	___1
NaN	NaN	NaN	NaN	S	2	___1
NaN	NaN	NaN	NaN	S	2	___1
NaN	NaN	NaN	NaN	S	2	___1
NaN	NaN	NaN	NaN	S	2	___1
0,172667	0,739594	NaN	-1,02305	S	3	___1
0,949278	2,03909	-0,303617	1,45997	S	2	___1
2,62498	1,88854	-1,95525	NaN	S	3	___1
-0,63253	-0,551437	-0,258373	-0,579901	S	3	___1
1,97531	1,74354	2,99398	NaN	S	2	___1
NaN	-0,1591	0,729944	NaN	S	2	___1
3,05368	3,42237	2,94803	2,57529	S	2	___1
-1,37905	-1,56768	NaN	-1,64541	S	2	___1
1,65532	0,972027	0,336752	0,547611	S	3	___1
-0,648897	-1,79334	NaN	-1,47371	S	2	___1
-0,419584	-1,13197	NaN	-1,16453	S	2	___1
1,18987	1,18315	0,455719	NaN	S	2	___1

-0,930567	0,937857	1,34256	0,48291	S	2	___1
0,149582	0,243038	-0,155479	-0,33506	S	2	___1
1,22102	-0,141769	1,30613	-0,600956	S	2	___1
-1,05534	-0,937895	NaN	-1,80748	S	2	___1
NaN	1,10119	-1,04936	-0,95553	S	3	___1
1,98305	2,92777	NaN	1,576	S	2	___1
4,59067	4,38709	5,35837	3,04765	S	2	___1
1,62991	0,268242	2,84575	-0,638643	S	2	___1
0,563847	-0,366827	-1,85814	-0,526625	S	2	___1
-2,04844	-2,63621	-1,11469	-1,15829	S	2	___1
-1,35324	-0,859327	-2,12222	0,152359	S	3	___1
1,26115	0,252357	2,09867	0,663389	S	3	___1
NaN	0,263008	-0,306204	-0,314638	S	2	___1
3,10958	4,16579	5,82611	3,63141	S	2	___1
1,94459	1,69527	-1,64443	NaN	S	2	___1
-0,496927	0,205406	NaN	0,342266	S	3	___1
-1,67865	-3,20895	NaN	-1,81027	S	2	___1
-1,89611	-1,66054	-0,683926	-0,861917	S	2	___1
-0,812319	-2,10116	-3,1223	NaN	S	2	___1
NaN	NaN	NaN	NaN	S	2	___1
1,52835	1,25896	1,70153	0,205881	S	2	___1
1,50388	0,731947	-2,32909	0,350245	S	3	___1
-1,1062	-0,210968	NaN	-2,13753	S	2	___1
NaN	-0,948547	NaN	-1,12343	S	3	___1
2,21132	3,53749	NaN	1,20476	S	3	___1
2,1473	1,51207	-2,45769	NaN	S	2	___1
NaN	NaN	NaN	NaN	S	2	___1
NaN	NaN	NaN	NaN	S	2	___1
-2,07635	-2,39026	-0,841234	NaN	S	2	___1
NaN	NaN	NaN	NaN	S	2	___1
-0,916895	-1,7124	-1,2181	-2,38435	S	2	___1
NaN	-1,04645	0,383924	-1,27142	S	3	___1
-0,551384	-2,02552	-0,647923	-1,40529	S	2	___1
NaN	-0,417452	-0,131086	NaN	S	3	___1

-0,262043	NaN	NaN	NaN	S	2	___1
-2,00177	-2,19254	NaN	-1,25232	S	3	___1
-2,19546	-2,34315	-2,5042	-1,35434	S	2	___1
NaN	NaN	NaN	NaN	S	4	___1
-1,32345	-1,26473	-0,25815	NaN	S	2	___1
-1,36221	-0,440411	NaN	1,48018	S	2	___1
-2,91499	-2,00332	-1,20914	0,7614	S	2	___1
NaN	NaN	NaN	NaN	S	3	___1
NaN	NaN	NaN	NaN	S	2	___1
NaN	NaN	NaN	NaN	S	2	___1
-0,633713	-1,48516	-1,65891	NaN	S	2	___1
-0,604603	-0,570108	1,20497	-1,06752	S	3	___1
0,084837	0,139366	-1,16667	-1,25847	S	2	___1
0,00473976	-0,445045	0,689674	-1,2386	S	2	___1
NaN	-0,347218	NaN	0,655306	S	2	___1
NaN	NaN	NaN	NaN	S	1	___1
NaN	0,0160923	1,01362	-0,529562	S	3	___1
NaN	NaN	NaN	NaN	S	3	___1
NaN	NaN	NaN	NaN	S	2	___1
-0,186485	NaN	NaN	NaN	S	2	___1
NaN	NaN	NaN	NaN	S	3	___1
2,77506	2,86101	NaN	3,19671	S	3	___1
-1,00274	-0,94335	-1,4247	-1,30118	S	2	___1
NaN	NaN	NaN	NaN	S	2	___1
2,48488	3,11613	2,75729	2,27826	S	3	___1
0,692842	1,40088	1,25381	0,941973	S	2	___1
1,36012	1,16575	1,64572	1,15651	S	3	___1
NaN	NaN	NaN	NaN	S	3	___1
NaN	NaN	NaN	NaN	S	2	___1
-0,0497704	-0,196478	-0,0832081	1,39376	S	2	___1
-3,72474	0,236748	NaN	-1,24253	S	2	___1
-0,451731	0,24387	NaN	-0,00358963	S	3	___1
0,322351	-1,3003	0,319605	2,15545	S	2	___1
-1,74736	NaN	-0,286739	-0,452244	S	2	___1

Polito et al. Universal ready-to-use immunotherapeutic approach for the treatment of cancer: expanded and activated polyclonal γδ memory T cells.
Phosphorylation sites

NaN	-0,667627	-1,73155	-0,743532	S	3	___1
NaN	-0,622938	-0,161585	-0,94075	S	3	___1
2,3079	2,28824	3,06428	1,68003	S	3	___1
-1,44638	NaN	-1,77446	-2,07925	S	2	___1
-1,2454	-1,1811	-0,182219	-0,955439	S	2	___1
NaN	NaN	NaN	NaN	S	2	___1
NaN	-0,886253	-1,57469	-2,35673	S	3	___1
NaN	NaN	NaN	NaN	S	3	___1
-0,436129	1,43402	0,248251	0,571075	S	2	___1
1,05893	0,483297	2,51656	0,277395	S	2	___1
-1,09357	-0,711262	-0,692062	1,70194	S	2	___1
3,49892	3,23037	3,81113	3,61816	S	2	___1
-2,04087	NaN	1,82056	-1,255	S	3	___1
0,0332222	-1,01537	NaN	1,34857	S	3	___1
NaN	NaN	NaN	NaN	S	2	___1
-0,498247	-0,264133	-1,74148	-1,30946	S	2	___1
-0,221527	-1,1879	NaN	NaN	S	1	___1
NaN	NaN	NaN	NaN	S	3	___1
0,968033	1,11452	-0,595709	-0,458603	S	2	___1
-2,39935	-1,24181	-0,551104	-2,19558	S	2	___1
-3,28901	0,278776	0,133421	-1,34217	S	2	___1
1,20683	2,92508	4,15933	2,44046	S	3	___1
0,346205	-0,0607204	0,767206	-0,116199	S	2	___1
1,54091	1,74077	0,405188	2,30055	S	2	___1
0,725626	1,51034	NaN	1,64952	S	2	___1
3,22169	1,51555	2,36234	2,27537	S	3	___1
NaN	1,46348	NaN	-1,67547	S	3	___1
-2,61854	-2,76378	NaN	-1,12108	S	2	___1
-1,19241	-0,876898	NaN	-1,0102	S	2	___1
NaN	NaN	NaN	NaN	S	2	___1
NaN	-1,72117	-1,81467	-1,78899	S	2	___1
NaN	NaN	NaN	NaN	S	2	___1
NaN	NaN	NaN	NaN	S	2	___1
-1,20362	-1,70021	NaN	NaN	S	2	___1

Polito et al. Universal ready-to-use immunotherapeutic approach for the treatment of cancer: expanded and activated polyclonal γδ memory T cells.
Phosphorylation sites

NaN	NaN	NaN	NaN	S	2	___1
0,424835	-0,317255	1,24843	-0,220583	S	2	___1
-2,2741	NaN	-2,32582	-0,512821	S	2	___1
NaN	-2,04891	-0,619469	NaN	S	2	___1
NaN	NaN	NaN	NaN	S	2	___1
-1,99983	NaN	-1,77901	-3,66466	S	2	___1
NaN	NaN	NaN	NaN	S	2	___1
NaN	-0,947187	-0,597784	NaN	S	2	___1
-1,56388	-1,95089	NaN	-2,20343	S	2	___1
-3,25885	-1,25248	NaN	-0,433426	S	3	___1
NaN	-0,724226	-1,45469	-2,1017	S	3	___1
-1,37195	-0,421101	NaN	-0,862812	S	3	___1
-1,22444	-0,762802	-1,63963	NaN	S	2	___1
-0,427902	-0,353498	NaN	-0,607224	S	2	___1
NaN	-1,21519	-0,853588	0,598919	S	3	___1
-1,07996	-0,451769	NaN	NaN	S	2	___1
NaN	NaN	NaN	NaN	S	3	___1
-1,34635	-3,41761	NaN	NaN	S	2	___1
-2,06281	-1,56476	NaN	-0,948732	S	2	___1
NaN	NaN	NaN	NaN	S	2	___1
NaN	NaN	NaN	NaN	S	2	___1
NaN	NaN	NaN	NaN	S	2	___1
NaN	NaN	NaN	NaN	S	2	___1
NaN	-2,34292	-1,51334	-1,61977	S	2	___1
NaN	-1,82831	-1,0306	-2,2184	S	2	___1
1,04605	1,76965	0,307882	2,17145	S	2	___1
-1,05837	-1,29231	NaN	-1,62157	S	2	___1
NaN	NaN	NaN	NaN	S	2	___1
-1,38918	NaN	0,150402	0,151136	S	2	___1
-0,63953	0,360687	-1,33384	0,514345	S	3	___1
NaN	NaN	NaN	NaN	S	2	___1
NaN	NaN	NaN	NaN	S	2	___1
0,215939	-0,0847931	-0,344198	0,668541	S	3	___1
NaN	NaN	NaN	NaN	S	2	___1

Polito et al. Universal ready-to-use immunotherapeutic approach for the treatment of cancer: expanded and activated polyclonal γδ memory T cells.
Phosphorylation sites

NaN	0,112766	-0,37858	-0,849201	S	2	___1
-0,719938	-1,99429	-1,32891	-1,0022	S	2	___1
2,15393	1,95871	0,361401	2,99171	S	3	___1
NaN	1,7111	NaN	1,80382	S	2	___1
1,21583	-0,298958	-3,05101	-0,779268	S	2	___1
2,88021	2,63686	1,25653	2,76057	S	3	___1
1,97566	1,01849	-0,469788	2,1988	S	2	___1
NaN	NaN	2,00553	NaN	S	3	___1
1,19524	1,36978	-2,09204	-0,0505848	S	3	___1
-0,83742	-1,71885	-1,93028	-3,01367	S	2	___1
NaN	NaN	NaN	NaN	S	2	___1
2,57608	1,47058	1,54548	1,25696	S	3	___1
3,00203	1,58662	2,29683	2,30634	S	2	___1
-0,659695	-0,640673	NaN	-0,913977	S	3	___1
NaN	NaN	NaN	NaN	S	2	___1
-2,21793	-1,59108	NaN	-0,608795	S	3	___1
-0,00325966	-0,137011	1,28785	NaN	S	2	___1
NaN	NaN	NaN	NaN	S	2	___1
NaN	NaN	NaN	NaN	S	2	___1
NaN	NaN	-2,35362	NaN	S	2	___1
NaN	NaN	NaN	NaN	S	2	___1
0,172791	-0,97146	NaN	-0,844736	S	2	___1
2,69567	1,37566	3,06792	1,27469	S	2	___1
NaN	NaN	NaN	NaN	S	3	___1
-0,678314	-1,41968	-1,37616	NaN	S	2	___1
0,68573	0,555006	-0,226789	0,800257	S	2	___1
-3,70635	-2,97757	1,21383	NaN	S	2	___1
NaN	0,983419	-1,71656	2,44684	S	2	___1
1,09702	1,02789	1,80919	1,67403	S	2	___1
2,35451	2,47547	3,26806	2,13667	S	2	___1
NaN	NaN	NaN	NaN	S	2	___1
1,5051	2,35936	0,699453	1,58509	S	2	___1
1,08618	1,52456	1,19596	NaN	S	2	___1
-0,999273	-0,231466	NaN	-1,29066	S	3	___1

-1,30245	NaN	0,7157	-0,15678	S	2	___1
3,36433	2,4564	1,93497	3,0859	S	2	___1
NaN	0,746838	-0,394892	-0,336035	S	2	___1
-2,65543	-2,43858	-4,20354	-1,42749	S	2	___1
1,58361	0,923542	1,42401	-0,373175	S	2	___1
1,12503	NaN	NaN	NaN	S	3	___1
NaN	NaN	NaN	NaN	S	2	___1
-1,34582	-1,07847	NaN	NaN	S	2	___1
NaN	NaN	NaN	NaN	S	2	___1
-1,14429	-1,46196	-1,17526	NaN	S	3	___1
NaN	-0,908987	0,695578	NaN	S	2	___1
0,824728	0,84462	1,0792	1,59853	S	2	___1
0,402256	0,667364	0,279074	NaN	S	2	___1
-1,30947	-0,708286	NaN	-1,23088	S	2	___1
1,04153	1,28572	-0,81144	0,00112343	S	3	___1
NaN	NaN	NaN	NaN	S	2	___1
-0,843811	-0,945566	-0,82527	NaN	S	3	___1
0,216982	-0,562141	-3,24476	-0,700148	S	2	___1
1,94989	3,50774	NaN	0,7798	S	3	___1
4,77685	4,85644	1,29299	2,57577	S	2	___1
2,78921	2,83745	2,96321	2,86168	S	4	___1
-1,16372	-1,21274	-3,35472	-2,8383	S	2	___1
0,699621	-1,42955	NaN	-0,5016	S	3	___1
0,546516	0,0470867	1,49228	-0,146864	S	2	___1
-1,43613	NaN	-2,75107	-0,480597	S	2	___1
-1,80129	-0,649443	-1,54579	-1,20608	S	2	___1
-2,85192	-2,75746	NaN	-2,13198	S	2	___1
NaN	-1,41763	-0,081852	NaN	S	3	___1
1,08866	0,829302	0,704319	-0,755939	S	2	___1
-0,402309	0,365107	-0,369081	-0,592491	S	2	___1
-2,33348	-1,85941	-0,37574	NaN	S	2	___1
NaN	NaN	NaN	NaN	S	3	___1
-3,24023	-3,31644	NaN	-1,71791	S	2	___1
NaN	2,53409	-0,148678	1,29	S	2	___1

Polito et al. Universal ready-to-use immunotherapeutic approach for the treatment of cancer: expanded and activated polyclonal γδ memory T cells.
Phosphorylation sites

-1,88494	-2,33506	NaN	-0,960945	S	2	___1
4,65851	NaN	6,10369	3,37229	S	2	___1
4,65851	5,08723	6,10369	3,37229	S	2	___1
0,241016	0,663876	1,2803	3,67762	S	3	___1
-0,894823	-2,07413	NaN	NaN	S	2	___1
NaN	0,968275	1,19555	1,65042	S	2	___1
2,80352	1,7553	1,41356	0,256565	S	2	___1
0,970139	-1,9904	-0,534893	-1,65489	S	2	___1
2,8176	3,46226	3,64149	2,96585	S	3	___1
NaN	NaN	NaN	NaN	S	2	___1
1,38885	3,04316	3,18663	2,59776	S	3	___1
-0,553366	0,582548	NaN	0,232103	S	4	___1
0,00729179	0,579254	NaN	NaN	S	2	___1
NaN	-2,10614	NaN	NaN	S	2	___1
NaN	NaN	NaN	NaN	S	2	___1
-1,06938	-0,304937	-1,40669	-1,98434	S	2	___1
NaN	NaN	NaN	NaN	S	3	___1
-0,126705	-0,778708	0,77212	NaN	S	2	___1
-0,604128	-0,136929	-2,74656	-0,458635	S	2	___1
-0,811283	-0,486071	-0,428232	-1,27527	S	2	___1
-0,0762291	0,0304852	0,751671	0,225107	S	3	___1
-0,702074	1,38935	-0,471464	NaN	S	2	___1
1,14218	1,6555	NaN	0,616243	S	2	___1
-1,2705	0,0232697	-0,626169	-1,6918	S	3	___1
2,32756	2,07924	1,30585	0,673838	S	2	___1
NaN	NaN	NaN	NaN	S	2	___1
NaN	NaN	NaN	NaN	S	2	___1
3,13706	3,01924	5,30579	3,6481	S	2	___1
2,51137	5,02663	NaN	2,65046	S	3	___1
0,0172901	0,79048	-0,186928	0,686119	S	2	___1
4,86688	4,65099	2,81413	3,34094	S	2	___1
NaN	2,65839	-0,101095	1,2488	S	4	___1
-2,80826	-0,860979	NaN	-1,07421	S	2	___1
5,26894	4,96516	5,45364	5,16284	S	2	___1

Polito et al. Universal ready-to-use immunotherapeutic approach for the treatment of cancer: expanded and activated polyclonal γδ memory T cells.
Phosphorylation sites

1,37983	0,318703	-0,288273	NaN	S	2	___1
-0,850479	NaN	2,16769	NaN	S	2	___1
-1,02435	0,316271	0,941288	-1,09728	S	2	___1
-1,52687	-1,8883	-1,9643	-1,60837	S	2	___1
0,689022	0,665119	-0,0704441	2,21917	S	2	___1
NaN	NaN	1,23405	NaN	S	4	___1
-0,113415	-0,661022	NaN	-1,47482	S	2	___1
-1,06858	-0,180758	0,759115	0,775564	S	3	___1
-0,855862	-1,51022	NaN	2,43176	S	2	___1
1,8479	1,77873	1,07191	1,76271	S	3	___1
-0,479866	-0,787838	NaN	-1,76324	S	2	___1
NaN	-1,23898	-1,52642	-1,78148	S	2	___1
NaN	NaN	NaN	NaN	S	3	___1
4,88809	4,7442	4,62089	2,96622	S	2	___1
-0,0661831	NaN	-0,390949	NaN	S	2	___1
1,06197	1,17649	2,2033	-0,303841	S	4	___1
-3,13257	NaN	-0,859783	NaN	S	2	___1
3,44726	2,33625	NaN	0,329441	S	3	___1
1,61483	0,785475	0,776747	-1,18382	S	2	___1
3,87934	6,10249	NaN	3,03419	S	3	___1
-0,676929	0,336941	NaN	-0,381237	S	3	___1
2,33822	1,73889	-2,25323	-0,187101	S	2	___1
0,497219	1,14507	1,36551	NaN	S	2	___1
4,17632	6,18513	3,75148	5,99838	S	3	___1
NaN	NaN	NaN	NaN	S	2	___1
NaN	NaN	NaN	NaN	S	2	___1
-1,62915	NaN	-0,789106	-2,07204	S	2	___1
-0,764093	-2,45137	-0,110235	NaN	S	2	___1
NaN	NaN	NaN	NaN	S	2	___1
-0,893986	-1,30309	NaN	-0,974691	S	2	___1
0,844351	-1,10852	NaN	-1,06194	S	3	___1
NaN	NaN	NaN	NaN	S	3	___1
0,539238	2,79035	1,76803	NaN	S	2	___1
-0,742832	-0,915691	NaN	NaN	S	2	___1

Polito et al. Universal ready-to-use immunotherapeutic approach for the treatment of cancer: expanded and activated polyclonal γδ memory T cells.
Phosphorylation sites

-0,631456	NaN	0,844675	NaN	S	2	___1
-2,66648	-0,612587	-0,0773525	-2,55984	S	2	___1
0,666998	1,84291	2,3724	1,4918	S	4	___1
-0,5604	0,838614	0,968781	-0,813358	S	2	___1
NaN	-1,03487	-0,227253	-0,285395	S	3	___1
-0,314592	1,70616	NaN	-0,105499	S	2	___1
NaN	0,380384	-1,30055	NaN	S	3	___1
NaN	NaN	NaN	NaN	S	2	___1
-3,50605	-0,82428	-3,04107	-0,707014	S	3	___1
NaN	NaN	NaN	NaN	S	2	___1
-0,17874	1,31277	-0,891382	-0,0765076	S	2	___1
0,578863	NaN	NaN	0,242378	S	3	___1
-0,604918	-1,41458	NaN	-3,3335	S	4	___1
NaN	NaN	NaN	NaN	S	2	___1
-1,01641	NaN	NaN	-0,23391	S	4	___1
0,678713	2,49899	3,9327	2,48917	S	3	___1
-0,206856	-0,53652	-2,28419	NaN	S	2	___1
1,32183	1,81317	0,475822	2,09522	S	2	___1
NaN	-1,00599	-2,82076	-2,15725	S	3	___1
2,22658	2,39165	-0,299973	3,05615	S	2	___1
NaN	NaN	0,216112	NaN	S	4	___1
2,11767	2,26057	NaN	2,25033	S	2	___1
-0,475765	0,204683	NaN	NaN	S	2	___1
NaN	NaN	NaN	NaN	S	2	___1
NaN	NaN	NaN	NaN	S	2	___1
-0,478561	0,275963	NaN	-1,93281	S	2	___1
-1,67477	-1,23131	-0,604387	-1,85356	S	2	___1
-1,1982	-1,24444	-1,08585	-1,21312	S	2	___1
NaN	-0,293652	0,560276	-0,84129	S	3	___1
1,46136	-0,581972	NaN	1,91297	S	2	___1
NaN	NaN	NaN	NaN	S	2	___1
0,669201	-0,143192	1,36243	0,076458	S	2	___1
-2,9983	-2,79463	2,26314	-1,50782	S	2	___1
0,282997	0,698851	-1,10891	-1,38545	S	2	___1

0,0488491	NaN	NaN	NaN	S	2	___1
-0,86347	-1,39743	-2,54274	NaN	S	2	___1
NaN	NaN	NaN	NaN	S	2	___1
2,78514	2,66007	2,0357	2,09313	S	3	___1
-0,623693	1,69789	0,800861	0,179853	S	2	___1
-2,31025	-1,59323	-1,06905	-3,14852	S	2	___1
NaN	-0,514698	-1,08748	-3,36017	S	2	___1
NaN	NaN	0,365923	NaN	S	3	___1
NaN	NaN	NaN	-0,401484	S	2	___1
1,10326	1,15776	1,10594	-1,62785	S	2	___1
NaN	NaN	NaN	NaN	S	2	___1
3,39703	2,58992	1,59516	NaN	S	2	___1
NaN	0,075058	-0,26141	1,05113	S	2	___1
0,680248	0,578594	-0,763239	0,557673	S	2	___1
1,52438	2,41593	-1,54003	0,439592	S	2	___1
NaN	NaN	0,758289	-1,02557	S	3	___1
NaN	NaN	NaN	NaN	S	2	___1
-1,60365	NaN	2,37554	-0,417913	S	2	___1
-1,48543	NaN	-0,681908	-0,289885	S	2	___1
4,028	3,45073	NaN	-0,905132	S	2	___1
-0,0953865	0,331951	0,443413	0,031992	S	4	___1
2,0659	0,217386	NaN	1,59977	S	2	___1
1,8573	2,00648	2,706	2,68689	S	2	___1
-2,03987	-2,26818	NaN	-1,77137	S	2	___1
-1,29827	-1,22766	NaN	-1,86281	S	2	___1
-1,29827	-1,22766	NaN	-1,86281	S	2	___1
NaN	NaN	NaN	NaN	S	3	___1
NaN	NaN	NaN	NaN	S	3	___1
-0,827301	-1,23469	NaN	-1,31196	S	2	___1
-2,04844	-1,95494	NaN	-1,28934	S	3	___1
0,423725	0,38335	0,609951	-0,135471	S	2	___1
NaN	NaN	NaN	NaN	S	3	___1
NaN	NaN	NaN	NaN	S	3	___1
0,562346	0,0864525	1,48581	NaN	S	2	___1

Polito et al. Universal ready-to-use immunotherapeutic approach for the treatment of cancer: expanded and activated polyclonal γδ memory T cells.
Phosphorylation sites

NaN	-1,62019	NaN	-1,67368	S	2	___1
1,00508	0,818771	0,942499	1,22083	S	2	___1
NaN	NaN	NaN	NaN	S	3	___1
0,380064	NaN	1,75347	-1,12962	S	3	___1
-0,572353	-1,03226	NaN	-0,500175	S	2	___1
NaN	NaN	NaN	NaN	S	2	___1
1,71659	2,82851	2,66948	1,45818	S	4	___1
NaN	NaN	-0,276497	NaN	S	2	___1
-2,5918	1,85562	1,73813	2,00542	S	3	___1
NaN	-3,59767	-0,11401	NaN	S	3	___1
NaN	NaN	NaN	NaN	S	2	___1
0,378519	-0,223923	-0,272556	0,0258389	S	2	___1
-2,63243	-2,17988	NaN	-2,60231	S	2	___1
2,64871	2,96232	2,98204	1,255	S	2	___1
-0,147163	2,46562	-0,062109	NaN	S	3	___1
NaN	NaN	NaN	NaN	S	2	___1
3,70911	2,95383	2,69533	2,2263	S	2	___1
NaN	-2,29944	-0,89242	-1,08059	S	2	___1
NaN	NaN	NaN	NaN	S	2	___1
NaN	NaN	1,60169	NaN	S	3	___1
NaN	NaN	NaN	NaN	S	3	___1
4,78504	5,90806	3,54414	3,84753	S	2	___1
NaN	NaN	NaN	NaN	S	5	___1
NaN	1,22308	NaN	NaN	S	5	___1
NaN	NaN	NaN	NaN	S	2	___1
0,673334	0,485464	NaN	NaN	S	2	___1
-1,55917	-1,62128	-2,20436	-1,32382	S	2	___1
NaN	NaN	NaN	NaN	S	2	___1
NaN	NaN	NaN	NaN	S	2	___1
NaN	NaN	NaN	NaN	S	1	___1
NaN	NaN	NaN	NaN	S	2	___1
NaN	0,534378	-1,38483	-1,11498	S	2	___1
NaN	0,632488	NaN	NaN	S	2	___1
0,107204	-0,774035	NaN	-1,95181	S	2	___1

0,432173	-0,446594	NaN	1,06441	S	2	___1
NaN	NaN	NaN	NaN	S	3	___1
2,1651	2,75833	-0,179739	1,35179	S	2	___1
0,716568	NaN	NaN	NaN	S	2	___1
-1,70179	-3,3699	-1,55411	-0,398949	S	2	___1
-4,70948	-4,40361	1,83381	NaN	S	2	___1
4,21424	3,50874	3,2244	3,20895	S	2	___1
-0,912193	-1,99767	NaN	-2,35901	S	2	___1
1,28035	0,860487	2,73879	-0,511118	S	2	___1
1,01348	-0,689585	0,333857	2,92381	S	2	___1
-0,704161	NaN	-0,663174	-0,801634	S	2	___1
NaN	NaN	NaN	NaN	S	3	___1
-0,823189	-1,51943	-0,752966	-1,3683	S	2	___1
-1,68788	3,81877	2,35495	3,12806	S	3	___1
1,58072	-2,50746	0,59856	NaN	S	2	___1
-1,82429	0,157148	NaN	-0,542469	S	2	___1
NaN	2,15364	-1,74156	0,858778	S	4	___1
-0,351162	-0,545452	0,863985	-0,8606	S	2	___1
NaN	-2,36864	NaN	-0,667894	S	3	___1
0,778736	0,0528698	-0,983271	-0,179892	S	2	___1
-0,115627	-3,81844	-2,1837	NaN	S	2	___1
-1,55306	-1,37	NaN	-0,312513	S	2	___1
-1,05786	0,720907	1,2288	0,783751	S	2	___1
NaN	0,0873795	0,848946	0,0241814	S	2	___1
1,64837	1,71381	0,905455	NaN	S	3	___1
2,27599	NaN	1,39102	0,0695992	S	2	___1
0,919474	0,395329	1,45031	0,096096	S	2	___1
-2,16355	-2,43825	-0,725998	NaN	S	2	___1
-0,300869	-0,64588	NaN	0,458418	S	2	___1
1,86188	0,722193	-0,428984	-0,0762367	S	2	___1
0,00966835	-0,580271	NaN	-1,07718	S	2	___1
-1,52846	-0,158253	NaN	-1,29749	S	2	___1
NaN	NaN	NaN	NaN	S	3	___1
NaN	NaN	NaN	NaN	S	2	___1

Polito et al. Universal ready-to-use immunotherapeutic approach for the treatment of cancer: expanded and activated polyclonal γδ memory T cells.
Phosphorylation sites

NaN	NaN	NaN	NaN	S	2	___1
1,5671	1,66211	1,69888	1,60085	S	3	___1
NaN	-0,515781	3,2321	1,53059	S	3	___1
4,34675	2,6508	0,695004	1,96441	S	2	___1
NaN	NaN	NaN	NaN	S	2	___1
-2,38051	-1,91415	0,34104	NaN	S	2	___1
-0,87245	-1,23022	NaN	-0,416256	S	2	___1
NaN	NaN	NaN	NaN	S	2	___1
1,46236	0,484543	-1,20667	0,427921	S	2	___1
-0,915979	-2,68949	-2,71872	NaN	S	2	___1
-0,847609	-2,01604	-2,45386	-1,65585	S	2	___1
-1,13718	1,6333	0,59334	0,733494	S	4	___1
1,60427	1,46699	NaN	0,646652	S	3	___1
0,288994	0,688869	1,88405	-0,274559	S	3	___1
0,859955	-0,272442	0,977907	-0,546227	S	2	___1
NaN	NaN	-0,0366287	NaN	S	2	___1
-0,342569	2,00511	2,76898	1,25941	S	2	___1
-2,58795	-1,84172	-0,455069	NaN	S	3	___1
-1,67123	-2,2729	NaN	NaN	S	3	___1
-0,464485	-1,46656	NaN	0,653013	S	2	___1
-0,967352	-0,679111	-0,456409	NaN	S	3	___1
NaN	NaN	NaN	NaN	S	2	___1
0,140213	-0,968149	0,492165	-0,170319	S	2	___1
2,05876	1,16768	NaN	0,878662	S	2	___1
NaN	NaN	0,209063	NaN	S	2	___1
NaN	NaN	NaN	NaN	S	2	___1
-1,43304	-1,18449	NaN	0,235352	S	2	___1
-2,07595	-1,55075	-2,00184	-0,306677	S	2	___1
-2,52645	-1,73032	-0,87426	-0,781803	S	2	___1
-0,806549	-2,52267	-2,22641	-1,75428	S	2	___1
NaN	0,236555	0,342674	0,596209	S	3	___1
-1,36408	-1,1624	-3,79624	-1,19945	S	2	___1
-0,0870457	0,314625	NaN	0,808037	S	3	___1
0,901928	-0,356989	0,493752	2,77382	S	2	___1

2,86093	2,90822	2,66852	2,4072	S	3	___1
NaN	NaN	NaN	NaN	S	2	___1
-1,91154	-1,05474	-1,80228	-0,380566	S	3	___1
0,00632668	1,21944	NaN	-1,46794	S	2	___1
NaN	NaN	NaN	NaN	S	2	___1
0,364082	-0,914976	-0,157919	-0,825636	S	2	___1
2,25973	2,32936	-0,717897	1,78334	S	2	___1
-2,38702	-3,50526	-2,30301	0,247044	S	2	___1
-1,20754	-1,94498	NaN	-0,128542	S	2	___1
NaN	NaN	NaN	NaN	S	2	___1
1,35417	0,386717	NaN	0,701296	S	2	___1
1,07469	0,655046	NaN	0,0545654	S	2	___1
-0,485579	NaN	0,874434	-0,215923	S	2	___1
NaN	NaN	NaN	NaN	S	2	___1
NaN	-0,832085	0,231676	NaN	S	2	___1
-0,0893288	-0,895952	0,580526	-0,28894	S	2	___1
NaN	NaN	NaN	NaN	S	2	___1
-0,294056	NaN	-0,182932	-0,599501	S	2	___1
NaN	NaN	NaN	NaN	S	2	___1
-0,804127	-1,1211	-0,822346	-1,14161	S	2	___1
1,54352	0,447229	-1,76415	2,28995	S	3	___1
1,90156	1,16383	2,83261	1,10756	S	2	___1
-0,494925	0,207312	0,19154	1,42985	S	3	___1
-0,106049	0,868355	1,6334	NaN	S	3	___1
-0,900309	-1,33182	-1,28566	-1,64856	S	3	___1
-1,43136	-2,03984	NaN	-1,8841	S	2	___1
2,76978	2,55655	2,43868	1,79851	S	2	___1
NaN	NaN	0,888081	1,12302	S	3	___1
NaN	0,729389	0,387985	0,418839	S	3	___1
1,15784	0,713438	NaN	0,79108	S	2	___1
1,26558	2,18084	3,21096	1,23244	S	2	___1
-0,92786	-0,935587	0,667397	-1,32452	S	2	___1
-0,935528	-2,36302	-1,0446	-2,21698	S	2	___1
NaN	NaN	NaN	NaN	S	2	___1

Polito et al. Universal ready-to-use immunotherapeutic approach for the treatment of cancer: expanded and activated polyclonal γδ memory T cells.
Phosphorylation sites

-0,631134	-1,4198	NaN	NaN	S	2	___1
2,35613	2,32966	2,3715	-0,229261	S	2	___1
1,84867	0,978466	1,3888	-1,28118	S	2	___1
-0,439133	-0,0890598	-1,64217	NaN	S	2	___1
1,13023	0,784023	-2,1046	NaN	S	2	___1
-1,53548	-1,02415	-0,179132	NaN	S	2	___1
1,41052	0,653843	0,235199	-1,9836	S	2	___1
-1,07572	-1,76687	-1,09164	NaN	S	2	___1
2,38089	1,74299	1,81892	-1,48705	S	2	___1
NaN	NaN	NaN	NaN	S	2	___1
0,586567	1,10943	-1,19149	-0,702051	S	2	___1
2,27356	1,86055	2,06074	1,49205	S	2	___1
NaN	NaN	NaN	NaN	S	2	___1
NaN	-0,116159	-0,0415554	-0,0535965	S	3	___1
0,507809	0,347343	-1,43876	0,898401	S	2	___1
0,0463333	NaN	-1,18619	-0,276941	S	2	___1
-0,797188	-1,1577	-1,24769	-1,53777	S	2	___1
-2,46888	-1,44445	-1,58918	0,647236	S	2	___1
NaN	NaN	NaN	NaN	S	3	___1
NaN	-2,50028	0,804462	-1,83589	S	2	___1
NaN	NaN	NaN	NaN	S	2	___1
1,38726	1,11616	-0,946697	0,127163	S	2	___1
NaN	-2,76687	1,20759	-2,57707	S	3	___1
0,0848694	-0,0389099	0,821947	-1,44168	S	2	___1
NaN	0,656971	NaN	0,326437	S	3	___1
NaN	NaN	NaN	NaN	S	2	___1
-0,255617	-0,588339	NaN	NaN	S	3	___1
0,173067	1,53435	1,13251	1,1544	S	2	___1
-1,87983	-1,49143	-0,257454	-1,75191	S	2	___1
0,81016	0,481941	-0,412348	-0,54924	S	2	___1
0,463316	-0,804819	-0,372814	2,4034	S	2	___1
2,28817	1,52669	2,77522	1,66792	S	3	___1
0,175615	-0,620605	NaN	1,08675	S	2	___1
NaN	NaN	NaN	NaN	S	3	___1

Polito et al. Universal ready-to-use immunotherapeutic approach for the treatment of cancer: expanded and activated polyclonal γδ memory T cells.
Phosphorylation sites

3,06639	2,65201	3,41501	2,30491	S	2	___1
-0,586643	-0,919701	-2,87307	0,95351	S	2	___1
NaN	NaN	NaN	NaN	S	2	___1
0,721703	1,23308	-3,80512	-1,05126	S	2	___1
0,192419	-0,431286	-3,0235	-0,709923	S	2	___1
-0,30262	-0,134098	NaN	NaN	S	2	___1
0,029562	3,00618	0,340342	1,73483	S	2	___1
-0,459522	-1,28083	-2,89894	0,130741	S	2	___1
-0,85718	-0,73933	1,32335	-2,33046	S	2	___1
0,999498	0,313345	NaN	0,0157948	S	3	___1
0,0234375	-0,553104	NaN	-0,52919	S	2	___1
4,13832	4,51959	4,05201	NaN	S	2	___1
-1,47596	-1,83049	-3,32759	-0,223124	S	2	___1
-0,973598	NaN	NaN	NaN	S	2	___1
-2,09479	-2,10353	-2,93904	0,0252399	S	2	___1
-0,757284	-0,859877	-0,342218	-0,454264	S	2	___1
-1,14889	-2,04025	-4,07442	-1,4904	S	2	___1
NaN	-2,70329	-1,35048	0,392973	S	2	___1
-0,611202	-1,25278	0,0236149	-1,27964	S	2	___1
-0,980623	0,0616112	-0,273174	-1,68124	S	2	___1
-0,850918	-0,410988	NaN	0,321758	S	2	___1
NaN	NaN	NaN	NaN	S	2	___1
NaN	-0,00954437	-2,36185	-1,51905	S	3	___1
-0,363234	1,24198	2,19321	1,14278	S	2	___1
NaN	NaN	NaN	NaN	S	2	___1
-1,6654	-1,57417	-2,86738	NaN	S	2	___1
0,0588627	1,59261	NaN	-0,888168	S	2	___1
-2,50057	-1,53896	NaN	-3,21329	S	2	___1
NaN	NaN	NaN	NaN	S	2	___1
NaN	NaN	NaN	NaN	S	2	___1
-1,79562	NaN	-1,0819	NaN	S	2	___1
-1,71515	NaN	NaN	NaN	S	2	___1
NaN	NaN	1,83212	NaN	S	2	___1
4,11839	2,57537	3,37004	4,23521	S	3	___1

3,16668	1,92116	3,27903	2,50097	S	2	___1
NaN	NaN	0,120655	NaN	S	2	___1
-2,09223	-0,765762	-1,47298	0,463165	S	2	___1
NaN	NaN	NaN	NaN	S	2	___1
NaN	-1,83779	NaN	0,22274	S	3	___1
-0,658655	-0,291517	-2,1205	-1,74341	S	2	___1
NaN	NaN	NaN	NaN	S	2	___1
NaN	NaN	NaN	NaN	S	2	___1
-0,730707	-0,980091	-1,00184	-1,47684	S	3	___1
-0,0183411	0,0645866	-0,0708828	-0,11179	S	2	___1
-0,270754	NaN	0,490095	NaN	S	2	___1
-0,253262	0,233271	NaN	-1,16154	S	2	___1
-1,36168	-0,0954437	NaN	0,38788	S	3	___1
NaN	NaN	NaN	NaN	S	3	___1
NaN	NaN	NaN	NaN	S	2	___1
2,53099	1,41774	-1,69635	0,35944	S	2	___1
-1,23166	-1,07	-0,364908	0,4925	S	2	___1
0,589449	-1,31539	0,705603	-0,88986	S	2	___1
0,296432	0,147532	0,00562096	-0,282259	S	2	___1
2,06316	2,02666	2,69169	1,13341	S	2	___1
1,2676	1,89287	2,32009	0,922701	S	2	___1
0,0419331	-0,669073	-0,0349579	NaN	S	2	___1
0,71171	NaN	-1,60715	-0,996584	S	2	___1
3,73552	3,06079	NaN	1,99776	S	3	___1
-1,12202	-1,6659	-1,35309	-1,59154	S	2	___1
-0,160694	-0,579819	NaN	0,982845	S	2	___1
-0,985487	-2,0066	NaN	-1,66637	S	2	___1
NaN	NaN	NaN	NaN	S	3	___1
-0,753307	-0,615602	NaN	0,0651493	S	3	___1
-0,753307	NaN	NaN	NaN	S	3	___1
NaN	NaN	NaN	NaN	S	3	___1
1,94953	0,809589	0,490459	0,677795	S	2	___1
-1,92135	NaN	1,00599	-2,04173	S	2	___1
0,944641	-1,31535	-0,00470924	-1,80159	S	3	___1

-1,38982	NaN	-4,21898	-1,95999	S	2	___1
-1,14912	-1,30728	-2,04729	NaN	S	2	___1
0,722586	-0,665943	-0,369291	-0,0377827	S	2	___1
0,961163	2,50314	-1,6041	NaN	S	2	___1
1,17036	-0,246313	-0,92882	NaN	S	2	___1
2,51495	2,09531	2,57037	1,5169	S	2	___1
NaN	-0,356407	-2,13014	-1,37503	S	3	___1
NaN	NaN	NaN	NaN	S	3	___1
NaN	0,550407	-0,315819	NaN	S	3	___1
0,498886	-0,399246	0,00492668	-0,67738	S	2	___1
NaN	NaN	NaN	NaN	S	2	___1
NaN	NaN	NaN	NaN	S	2	___1
NaN	-1,55406	NaN	-0,967756	S	2	___1
NaN	NaN	NaN	NaN	S	2	___1
-0,850792	-2,71419	NaN	-2,14601	S	2	___1
-0,240763	-0,940176	NaN	NaN	S	2	___1
NaN	-1,64349	-0,585196	-1,54657	S	3	___1
-1,69605	1,05892	1,85719	-0,168421	S	3	___1
-1,18854	-2,65523	0,320965	-2,51992	S	2	___1
NaN	NaN	0,514397	NaN	S	3	___1
NaN	NaN	NaN	NaN	S	2	___1
-0,923517	NaN	-1,41337	NaN	S	2	___1
0,986109	0,394581	0,659138	0,629993	S	2	___1
NaN	-1,07558	-0,618431	NaN	S	4	___1
NaN	0,83186	-0,431492	0,570696	S	3	___1
1,1885	0,697495	1,26789	0,0695095	S	2	___1
NaN	1,25496	0,991083	0,352833	S	4	___1
-1,15297	-1,6335	-0,863209	0,513525	S	1	___1
-0,613905	1,2754	-0,503803	0,911163	S	3	___1
0,0852947	-0,566288	NaN	0,79365	S	3	___1
NaN	3,2709	1,30321	0,446692	S	2	___1
1,8293	3,66521	2,54448	0,645006	S	3	___1
3,58115	3,44475	3,3147	3,87783	S	2	___1
NaN	-0,999992	NaN	-0,554358	S	2	___1

-1,22112	-2,80927	NaN	-1,42014	S	2	___1
0,352352	NaN	0,624352	-0,225147	S	3	___1
2,16286	2,23006	NaN	NaN	S	2	___1
NaN	0,753012	0,969376	-0,201548	S	2	___1
0,854925	-0,108213	NaN	NaN	S	2	___1
0,118324	0,537619	-2,18931	-0,501204	S	2	___1
-0,0454731	-1,42342	NaN	-0,779993	S	2	___1
0,99004	NaN	NaN	1,30183	S	2	___1
0,736002	-0,0960102	-1,34976	1,29411	S	2	___1
2,3239	1,91233	3,00515	1,59946	S	2	___1
0,815308	1,29712	0,835184	1,01739	S	2	___1
0,774387	1,28311	NaN	0,935005	S	3	___1
-0,121231	-1,15411	-2,43574	-1,41226	S	2	___1
NaN	1,35005	3,0862	1,18274	S	2	___1
NaN	NaN	NaN	NaN	S	2	___1
1,59639	1,38292	-0,198257	3,13792	S	2	___1
0,310358	0,907711	0,28747	3,41104	S	3	___1
-0,446527	0,821646	-0,275822	NaN	S	3	___1
NaN	NaN	NaN	NaN	S	2	___1
0,323957	0,072752	-1,67889	1,11871	S	3	___1
NaN	-0,521753	NaN	0,571106	S	3	___1
NaN	NaN	NaN	NaN	S	2	___1
-0,763918	-2,25625	NaN	-0,590691	S	2	___1
-3,07857	-2,67848	NaN	NaN	S	2	___1
2,54364	3,25249	NaN	3,14188	S	2	___1
NaN	0,977196	-0,898481	-0,564264	S	3	___1
1,25783	1,09453	NaN	1,76561	S	3	___1
0,103243	-0,70026	1,34797	-1,27306	S	2	___1
NaN	-0,913548	-1,30433	-1,81769	S	3	___1
-1,73013	-2,11445	NaN	-0,562603	S	2	___1
-0,710497	-0,600815	0,878365	0,860327	S	3	___1
-1,80358	-0,283285	0,47212	-0,656773	S	3	___1
-0,304974	-0,807468	NaN	-0,406567	S	2	___1
-0,643854	-1,41901	-0,454079	-0,312834	S	2	___1

Polito et al. Universal ready-to-use immunotherapeutic approach for the treatment of cancer: expanded and activated polyclonal γδ memory T cells.
Phosphorylation sites

-1,29096	-2,33583	NaN	-1,71399	S	2	___1
NaN	NaN	-0,107901	NaN	S	4	___1
0,822672	0,9552	-2,23954	-0,0146446	S	2	___1
NaN	NaN	NaN	NaN	S	2	___1
NaN	NaN	NaN	NaN	S	2	___1
NaN	NaN	-0,806383	NaN	S	3	___1
6,1636	6,82018	4,21628	6,40465	S	3	___1
-0,707607	NaN	NaN	NaN	S	2	___1
NaN	-0,195869	NaN	-1,64937	S	2	___1
0,309408	-0,425369	NaN	NaN	S	3	___1
NaN	NaN	NaN	NaN	S	2	___1
NaN	NaN	NaN	NaN	S	3	___1
-2,64004	NaN	-1,68966	NaN	S	2	___1
1,11979	NaN	0,617571	NaN	S	2	___1
NaN	NaN	NaN	NaN	S	3	___1
NaN	NaN	NaN	NaN	S	2	___1
NaN	NaN	NaN	NaN	S	2	___1
NaN	-0,956425	NaN	-0,755188	S	3	___1
NaN	1,97062	1,82897	1,97512	S	3	___1
NaN	NaN	NaN	NaN	S	2	___1
0,967484	-0,206238	NaN	0,697126	S	2	___1
0,514711	0,443275	-1,38325	0,360279	S	2	___1
-1,22088	NaN	-1,24075	0,922701	S	2	___1
1,88082	NaN	NaN	NaN	S	2	___1
2,27284	2,61525	-0,436996	2,75642	S	2	___1
1,01736	0,271511	-1,38744	-0,210085	S	2	___1
-0,572405	-1,11822	NaN	-2,5272	S	2	___1
-0,933144	-2,07694	-2,64272	-2,44288	S	2	___1
NaN	NaN	NaN	NaN	S	2	___1
0,903725	0,19591	NaN	-1,66481	S	2	___1
3,14808	1,83156	1,50258	3,78351	S	3	___1
-0,195147	0,72916	0,424862	2,39959	S	2	___1
2,8292	1,80109	1,43102	1,83442	S	2	___1
0,371359	0,725084	0,054512	0,820162	S	3	___1

Polito et al. Universal ready-to-use immunotherapeutic approach for the treatment of cancer: expanded and activated polyclonal γδ memory T cells.
Phosphorylation sites

-1,66254	-2,01069	NaN	-1,61416	S	2	___1
0,689194	1,57058	1,2386	-0,631804	S	2	___1
-1,89973	-1,53193	0,402779	-0,795765	S	2	___1
1,57387	1,51464	-0,990974	0,548653	S	2	___1
NaN	NaN	NaN	NaN	S	3	___1
NaN	NaN	NaN	NaN	S	2	___1
1,3239	0,994799	-0,110964	0,414873	S	2	___1
NaN	NaN	NaN	NaN	S	2	___1
NaN	NaN	NaN	NaN	S	2	___1
NaN	NaN	NaN	NaN	S	2	___1
-1,66661	-1,64524	NaN	NaN	S	2	___1
3,35958	3,08931	4,37881	1,05644	S	3	___1
-1,42176	1,01437	NaN	NaN	S	3	___1
0,833969	0,912632	1,72987	-0,47831	S	3	___1
-0,28356	-2,99245	NaN	-0,25679	S	2	___1
-0,0717793	-1,45191	NaN	-0,327515	S	2	___1
0,0456295	1,33631	2,18637	1,95508	S	3	___1
3,13564	2,29052	3,81466	2,98453	S	2	___1
NaN	NaN	NaN	NaN	S	2	___1
NaN	NaN	NaN	NaN	S	2	___1
NaN	NaN	1,13907	NaN	S	2	___1
2,47057	1,85986	2,00854	4,00755	S	2	___1
2,92469	4,06998	2,18253	1,795	S	2	___1
NaN	-2,06573	-0,489193	NaN	S	2	___1
NaN	-2,5862	3,1672	-0,116428	S	2	___1
-1,24425	-3,99801	-0,24938	-0,769133	S	2	___1
1,49763	-0,17544	NaN	0,378576	S	2	___1
-2,04515	-2,59657	-2,54111	NaN	S	2	___1
-1,06205	0,393023	NaN	NaN	S	4	___1
NaN	NaN	NaN	NaN	S	2	___1
NaN	NaN	NaN	NaN	S	2	___1
-1,99775	1,30493	0,964375	-0,521235	S	3	___1
NaN	1,91245	1,04554	0,960629	S	3	___1
-1,8563	-1,47712	NaN	NaN	S	2	___1

-0,446762	-0,230755	-1,00861	0,79311	S	3	___1
1,80025	1,74713	1,07202	1,35316	S	2	___1
NaN	NaN	-0,921219	-1,65128	S	3	___1
-0,216244	-1,17296	0,546749	-0,45796	S	3	___1
1,21761	0,158777	1,54675	0,82955	S	2	___1
NaN	NaN	NaN	NaN	S	2	___1
-2,08636	-1,82794	-1,99208	NaN	S	2	___1
0,381449	0,76086	-1,30537	NaN	S	2	___1
NaN	-0,20825	0,454733	0,22863	S	2	___1
NaN	NaN	NaN	NaN	S	2	___1
-1,40048	0,162779	NaN	-0,496799	S	3	___1
1,90859	2,08465	2,018	1,59698	S	2	___1
NaN	-0,438766	-0,11109	-0,936396	S	2	___1
-0,994986	-0,916264	0,0123062	-0,782932	S	2	___1
NaN	1,66187	0,277	0,162483	S	3	___1
2,6365	2,038	0,652874	1,05328	S	2	___1
-1,00524	-1,50604	-0,808052	-0,944126	S	2	___1
-1,17299	-1,60183	0,261223	0,205271	S	3	___1
0,14304	NaN	2,48602	-0,278048	S	3	___1
0,579142	-0,836679	NaN	-0,270966	S	2	___1
1,82275	1,39103	1,26353	0,766642	S	2	___1
0,561008	1,01211	NaN	0,981659	S	3	___1
3,72001	NaN	NaN	NaN	S	2	___1
-1,06068	-1,70547	NaN	-0,272379	S	2	___1
-0,930368	-2,04078	NaN	-0,549927	S	2	___1
-0,880465	-1,74359	NaN	-2,08213	S	2	___1
-1,43398	-2,47655	-0,426823	NaN	S	2	___1
-0,194433	-0,2288	1,0275	-0,241089	S	2	___1
-2,50173	-1,25343	-2,20318	-0,858517	S	3	___1
NaN	-0,875923	-2,69605	-1,19354	S	2	___1
2,11903	-0,0211105	-1,33589	0,606739	S	3	___1
NaN	NaN	NaN	NaN	S	3	___1
-2,14144	-3,04078	NaN	-0,690626	S	2	___1
-0,10054	-0,631014	0,575256	-0,21277	S	2	___1

Polito et al. Universal ready-to-use immunotherapeutic approach for the treatment of cancer: expanded and activated polyclonal γδ memory T cells.
Phosphorylation sites

NaN	NaN	NaN	NaN	S	2	___1
NaN	NaN	NaN	NaN	S	2	___1
-1,48922	NaN	NaN	-2,12062	S	2	___1
-0,357939	-0,591991	-0,544807	-0,45109	S	2	___1
0,466003	1,21324	0,263474	-0,822983	S	2	___1
-1,0539	-1,1153	NaN	-1,81093	S	2	___1
NaN	NaN	NaN	NaN	S	2	___1
2,69572	2,51618	-1,80561	1,80643	S	3	___1
-0,964308	-1,78165	NaN	-2,80598	S	2	___1
2,34629	0,978235	0,718391	1,07257	S	3	___1
-0,948977	-1,25166	NaN	-0,349581	S	2	___1
-0,369665	4,28103	3,04411	3,5262	S	4	___1
NaN	NaN	NaN	NaN	S	3	___1
NaN	NaN	NaN	NaN	S	3	___1
-0,545658	-0,860428	-0,866388	0,944166	S	2	___1
NaN	-0,375454	-0,0318623	NaN	S	2	___1
NaN	-0,68507	NaN	0,635372	S	2	___1
NaN	-1,75549	-2,1206	-0,694981	S	2	___1
-2,07382	NaN	-0,761501	NaN	S	2	___1
NaN	-1,52712	0,388697	-0,1106	S	3	___1
NaN	-0,526222	-2,11444	NaN	S	2	___1
NaN	NaN	NaN	NaN	S	2	___1
2,7624	3,25852	3,55473	2,99056	S	2	___1
-1,66441	-1,10884	NaN	NaN	S	2	___1
-0,504477	0,0601578	0,169256	-1,05029	S	2	___1
0,885836	0,644224	1,38524	0,678719	S	2	___1
-1,65129	NaN	NaN	-1,51142	S	2	___1
1,44338	0,0295944	-0,38726	2,20613	S	2	___1
2,44802	1,93487	0,0708923	1,16739	S	2	___1
-0,438757	0,136955	-0,0666599	-0,818886	S	2	___1
0,649899	NaN	NaN	NaN	S	2	___1
NaN	0,962492	2,91804	0,376394	S	2	___1
NaN	NaN	NaN	NaN	S	3	___1
NaN	-1,26063	-0,834993	0,621202	S	3	___1

Polito et al. Universal ready-to-use immunotherapeutic approach for the treatment of cancer: expanded and activated polyclonal γδ memory T cells.
Phosphorylation sites

3,13729	3,30811	1,34073	2,59799	S	3	___1
0,368624	0,627396	0,745436	1,29717	S	2	___1
-0,0614738	1,35596	-6,08443	0,274803	S	2	___1
4,45745	3,25852	4,8883	2,68493	S	2	___1
-0,727549	-1,42761	-2,26751	NaN	S	2	___1
0,473333	-0,53553	-1,38234	1,6987	S	2	___1
2,15557	2,28819	3,32425	2,65366	S	2	___1
0,0024929	-1,60091	-0,269888	0,29627	S	2	___1
1,39057	-0,0801392	-0,28229	1,25872	S	3	___1
2,09506	2,18001	NaN	NaN	S	3	___1
NaN	NaN	NaN	NaN	S	3	___1
NaN	NaN	NaN	NaN	S	2	___1
NaN	NaN	NaN	NaN	S	3	___1
NaN	NaN	NaN	NaN	S	2	___1
-2,6398	-2,35257	-2,85214	NaN	S	2	___1
NaN	-0,102018	-0,02882	NaN	S	3	___1
-1,13848	-1,42509	-0,466793	-2,88234	S	2	___1
NaN	NaN	NaN	NaN	S	2	___1
NaN	0,464909	-0,943336	1,51911	S	3	___1
-1,32467	-0,35932	NaN	-1,37296	S	2	___1
1,42813	-2,60318	1,42713	NaN	S	2	___1
NaN	-2,16081	NaN	-0,861492	S	2	___1
-0,737682	-1,92384	-1,94715	-3,37368	S	2	___1
-1,28512	-1,12923	NaN	NaN	S	2	___1
NaN	NaN	NaN	NaN	S	2	___1
NaN	NaN	NaN	NaN	S	3	___1
NaN	NaN	NaN	NaN	S	2	___1
NaN	NaN	NaN	NaN	S	3	___1
NaN	NaN	NaN	NaN	S	3	___1
NaN	NaN	-1,34148	NaN	S	2	___1
-1,94024	-4,7139	-2,20264	NaN	S	2	___1
NaN	NaN	NaN	NaN	S	2	___1
-1,8094	-2,44802	-1,49998	0,763058	S	2	___1
-0,650202	NaN	-0,619469	NaN	S	2	___1

-2,11492	NaN	-0,903696	NaN	S	2	___1
NaN	-1,18484	NaN	-1,83346	S	3	___1
NaN	-0,611893	-0,963573	-1,32299	S	3	___1
-0,331453	-0,991287	-2,22783	NaN	S	2	___1
0,00601578	-0,515781	1,2496	-1,32171	S	2	___1
NaN	0,122616	-1,39287	0,255566	S	3	___1
0,298237	-0,0908356	NaN	-0,424187	S	2	___1
5,08407	4,91311	5,31137	3,35176	S	4	___1
NaN	NaN	NaN	NaN	S	2	___1
NaN	0,847656	2,49307	-0,797028	S	2	___1
1,73284	1,38715	2,54412	0,82876	S	2	___1
1,066	-0,653849	-4,23335	-0,0526962	S	3	___1
NaN	NaN	NaN	NaN	S	2	___1
-1,63409	-1,49292	NaN	-1,41513	S	2	___1
-2,30603	-0,249733	-1,1819	-1,67345	S	3	___1
-2,11071	-1,56225	-0,464287	-0,717607	S	2	___1
2,16363	1,8755	NaN	1,45511	S	2	___1
-1,59997	-1,39334	-1,38294	NaN	S	2	___1
-1,89482	0,484001	NaN	0,103811	S	3	___1
-1,03525	NaN	NaN	NaN	S	2	___1
-0,433788	-0,176981	0,612177	-0,948551	S	2	___1
1,16086	1,27051	0,660021	0,999578	S	2	___1
1,97936	1,74858	1,8312	1,19385	S	2	___1
-1,06771	0,459738	0,386278	-0,174807	S	3	___1
-1,05621	0,473124	-1,05835	NaN	S	3	___1
NaN	2,17159	0,421198	NaN	S	2	___1
NaN	NaN	NaN	NaN	S	2	___1
NaN	NaN	NaN	NaN	S	2	___1
NaN	NaN	NaN	NaN	S	2	___1
NaN	NaN	NaN	NaN	S	2	___1
NaN	NaN	-0,918621	NaN	S	3	___1
NaN	NaN	-1,36923	-1,53085	S	2	___1
-0,298056	-0,976551	-0,910725	1,12581	S	2	___1
1,03383	0,438921	NaN	-1,07347	S	2	___1

Polito et al. Universal ready-to-use immunotherapeutic approach for the treatment of cancer: expanded and activated polyclonal γδ memory T cells.
Phosphorylation sites

-0,166515	-2,94201	NaN	-0,224573	S	2	___1
NaN	0,313528	NaN	-1,0795	S	3	___1
-0,607555	-1,76793	NaN	-1,67442	S	2	___1
NaN	NaN	NaN	NaN	S	2	___1
NaN	NaN	NaN	NaN	S	2	___1
NaN	-0,738697	0,610744	-1,78374	S	3	___1
-0,656141	-1,21292	-1,95065	-2,39405	S	2	___1
-0,268707	0,127203	0,459414	0,100035	S	4	___1
1,27234	0,830582	-0,760832	0,448393	S	3	___1
NaN	-1,03995	-1,24444	NaN	S	2	___1
NaN	NaN	NaN	NaN	S	2	___1
0,526859	0,356016	0,579124	-1,36546	S	2	___1
-0,650257	-1,1689	-1,9663	-0,367754	S	2	___1
-2,56175	-1,30174	NaN	NaN	S	3	___1
2,75656	2,52055	-1,08852	1,68303	S	2	___1
-2,22418	-1,01022	NaN	-2,99833	S	2	___1
-1,23069	-0,803495	NaN	NaN	S	2	___1
-0,134178	-1,8106	NaN	0,622755	S	2	___1
NaN	NaN	NaN	NaN	S	2	___1
0,604931	1,43217	1,30066	NaN	S	3	___1
0,645077	0,208231	1,23227	-0,998409	S	2	___1
-2,44229	-2,17094	-0,191996	NaN	S	2	___1
2,52631	1,32453	-1,05878	-2,30061	S	2	___1
-1,49419	-1,15405	0,969255	-1,89617	S	3	___1
-1,10583	-3,85691	-2,81446	-3,21405	S	3	___1
-1,06894	NaN	-0,679184	NaN	S	2	___1
-0,360428	-0,12118	0,000837326	NaN	S	2	___1
-0,987616	0,843016	0,481686	0,239624	S	2	___1
-1,70303	NaN	-1,34095	-1,67151	S	2	___1
0,319994	0,643253	-0,321653	-0,070076	S	2	___1
0,713781	0,520443	0,927526	-0,0876675	S	2	___1
-2,62011	-3,34751	-2,05733	-0,905746	S	2	___1
-0,925753	-1,94143	-1,68973	-1,99766	S	2	___1
-0,320724	-0,53817	NaN	-0,389559	S	3	___1

-0,226179	-1,15959	-0,292828	-1,64116	S	3	___1
0,453836	-0,508215	1,45295	1,02903	S	2	___1
-0,632046	-0,683365	-1,32664	0,970703	S	2	___1
-0,435005	-2,56407	-2,46294	-1,61115	S	2	___1
-0,822577	-0,163082	NaN	-0,443233	S	3	___1
0,542145	-0,807337	NaN	-0,00937653	S	2	___1
-1,03937	-0,472548	-2,38878	-1,00573	S	2	___1
NaN	NaN	-0,830973	0,319603	S	3	___1
1,78715	1,2448	1,33797	1,75898	S	2	___1
0,768278	NaN	-1,45564	0,0260448	S	2	___1
-0,614117	-0,987076	1,45601	-0,397499	S	2	___1
NaN	NaN	NaN	NaN	S	3	___1
NaN	NaN	NaN	NaN	S	3	___1
0,792444	0,914282	0,333984	-0,17008	S	3	___1
0,147175	0,519703	1,71591	-2,14218	S	3	___1
-2,23111	-1,69473	-2,50222	-0,048645	S	3	___1
1,78765	1,41664	2,03831	2,95284	S	2	___1
NaN	NaN	NaN	NaN	S	2	___1
NaN	NaN	NaN	NaN	S	2	___1
0,742664	0,163794	1,54284	-0,424219	S	2	___1
NaN	-1,61793	NaN	NaN	S	2	___1
-0,573385	-1,17679	-2,03579	NaN	S	2	___1
1,19595	2,11557	0,482967	NaN	S	3	___1
-0,35293	0,0153427	1,99529	0,71549	S	3	___1
1,19773	-0,587315	0,95504	-0,282686	S	3	___1
-1,57757	NaN	NaN	NaN	S	2	___1
3,21856	2,24727	4,82023	3,6728	S	2	___1
NaN	NaN	NaN	NaN	S	2	___1
0,839363	-1,57085	-0,231974	-1,11752	S	2	___1
1,02847	1,85249	NaN	1,35252	S	3	___1
NaN	NaN	NaN	NaN	S	2	___1
-1,37177	-1,56629	-1,24119	0,500288	S	3	___1
-0,564238	-0,783787	0,948181	NaN	S	2	___1
NaN	NaN	NaN	NaN	S	3	___1

Polito et al. Universal ready-to-use immunotherapeutic approach for the treatment of cancer: expanded and activated polyclonal γδ memory T cells.
Phosphorylation sites

1,85911	2,93819	1,42972	2,27812	S	2	___1
0,419016	NaN	NaN	NaN	S	2	___1
NaN	NaN	NaN	NaN	S	2	___1
0,589172	0,452168	NaN	-0,63126	S	2	___1
-0,0123882	-0,240749	NaN	NaN	S	3	___1
-1,19574	NaN	NaN	-0,437639	S	3	___1
0,461782	0,634005	1,08733	0,605722	S	3	___1
NaN	-1,52334	-0,871275	-1,13666	S	3	___1
NaN	NaN	NaN	NaN	S	2	___1
NaN	NaN	NaN	NaN	S	3	___1
-0,485968	-1,60226	NaN	-1,05073	S	2	___1
NaN	NaN	NaN	NaN	S	3	___1
NaN	-1,32068	-1,23707	NaN	S	4	___1
-1,09246	NaN	-2,31189	-0,465929	S	2	___1
-1,8453	NaN	-0,691122	NaN	S	2	___1
0,182592	3,5826	NaN	1,41306	S	3	___1
2,36463	NaN	2,91063	1,81158	S	2	___1
2,3155	2,31403	2,46244	1,63542	S	2	___1
0,635363	3,4474	2,31472	3,57979	S	3	___1
0,842863	-0,555107	NaN	-0,366695	S	2	___1
0,581621	1,14115	-2,22968	0,599722	S	3	___1
1,58257	1,17044	0,144138	1,56773	S	2	___1
NaN	NaN	NaN	NaN	S	2	___1
1,97848	0,426174	1,20598	1,56131	S	2	___1
-2,87362	-0,146795	NaN	-0,986988	S	2	___1
-2,03333	-2,94353	NaN	-1,40902	S	2	___1
NaN	0,872707	-0,928022	NaN	S	3	___1
5,48973	4,15311	3,18691	6,09455	S	2	___1
NaN	NaN	NaN	-0,651096	S	2	___1
NaN	NaN	NaN	NaN	S	2	___1
NaN	NaN	NaN	NaN	S	3	___1
NaN	NaN	NaN	NaN	S	2	___1
2,69085	2,75283	2,45961	2,14564	S	2	___1
-1,50546	0,09272	-0,984604	-0,752148	S	2	___1

Polito et al. Universal ready-to-use immunotherapeutic approach for the treatment of cancer: expanded and activated polyclonal γδ memory T cells.
Phosphorylation sites

0,845509	-0,835869	1,31869	0,599815	S	2	___1
NaN	NaN	NaN	NaN	S	2	___1
NaN	NaN	NaN	NaN	S	3	___1
1,18303	1,59657	1,02224	0,575546	S	2	___1
-0,501186	-2,09917	NaN	0,406414	S	2	___1
NaN	NaN	-0,864014	-1,11103	S	3	___1
-2,52079	-3,18539	0,476677	1,22855	S	3	___1
NaN	0,965096	0,344418	1,62338	S	3	___1
-0,215117	-0,190325	0,75939	0,155769	S	2	___1
NaN	-0,979553	-0,834538	-1,03337	S	2	___1
0,932438	2,13053	2,1323	1,20222	S	2	___1
NaN	-0,256962	NaN	0,180494	S	2	___1
-0,375048	-0,379095	NaN	-0,58004	S	2	___1
1,00208	1,2905	-0,245872	0,24943	S	2	___1
-0,420418	-1,59297	NaN	-1,47547	S	2	___1
NaN	NaN	NaN	NaN	S	2	___1
-0,153008	-1,18841	NaN	0,604536	S	2	___1
-4,1472	-1,87353	NaN	1,02926	S	2	___1
NaN	NaN	NaN	NaN	S	3	___1
2,54311	1,68416	0,707315	1,46854	S	2	___1
-0,521833	-0,768988	NaN	-0,375269	S	2	___1
NaN	NaN	NaN	NaN	S	4	___1
NaN	0,0297432	-0,217316	NaN	S	3	___1
2,78147	2,75159	3,63176	1,80435	S	2	___1
-0,807823	NaN	NaN	-1,93308	S	2	___1
-0,769695	-1,79993	-1,08126	0,714348	S	2	___1
NaN	-0,415127	-0,0915165	0,645515	S	2	___1
NaN	NaN	-0,361855	NaN	S	3	___1
-0,770227	NaN	-0,257956	NaN	S	2	___1
1,77859	1,73446	-1,40885	1,43246	S	3	___1
1,8843	3,1732	2,61551	2,76157	S	3	___1
1,16077	3,73268	2,65964	2,69719	S	3	___1
4,12325	3,30481	2,27886	3,64078	S	2	___1
0,145266	-0,962791	NaN	0,15476	S	4	___1

0,510948	1,99715	1,60865	0,801868	S	2	___1
-0,165934	NaN	NaN	NaN	S	2	___1
-1,17181	NaN	-2,32465	-0,399506	S	2	___1
-1,28293	NaN	-1,42226	0,276585	S	2	___1
-2,79082	-2,72222	NaN	-0,427807	S	3	___1
NaN	-1,91552	NaN	-0,674198	S	2	___1
NaN	NaN	NaN	NaN	S	3	___1
-4,04753	NaN	-2,35422	NaN	S	2	___1
NaN	NaN	NaN	NaN	S	4	___1
-0,276407	-1,92458	NaN	-0,768534	S	2	___1
-1,63065	-2,22757	NaN	-1,83296	S	2	___1
NaN	-0,883034	1,3017	0,226088	S	3	___1
-0,110008	-0,79479	NaN	-0,294216	S	2	___1
-0,931688	-0,984913	-2,33824	-0,456417	S	2	___1
-1,88008	-2,74944	1,69349	NaN	S	2	___1
2,94378	2,58866	2,74207	3,12967	S	2	___1
-2,37166	-0,315462	-1,12867	0,43885	S	2	___1
-1,6751	-2,35847	-3,25319	NaN	S	2	___1
-0,404966	1,30852	1,6558	1,91166	S	3	___1
1,9747	1,79155	2,56963	-0,200121	S	2	___1
0,98378	-0,525131	1,63594	NaN	S	3	___1
1,50937	0,169802	2,32302	0,0549488	S	3	___1
0,55345	0,775414	-0,345379	-0,538891	S	2	___1
2,32425	1,88899	NaN	0,0835266	S	2	___1
NaN	-1,98232	NaN	-1,59147	S	2	___1
NaN	NaN	NaN	NaN	S	2	___1
4,64334	3,98972	4,54695	3,11612	S	3	___1
1,87988	1,05346	2,71818	0,890715	S	2	___1
-1,86032	-2,47651	-0,73159	NaN	S	2	___1
-1,57633	-2,1672	-1,04287	-2,98568	S	2	___1
1,42478	0,25363	0,8374	NaN	S	3	___1
0,495106	0,140879	NaN	-0,230635	S	2	___1
0,185642	-1,18335	-2,05849	-1,96428	S	2	___1
1,04442	2,39691	0,289738	0,723173	S	3	___1

NaN	NaN	NaN	NaN	S	2	___1
NaN	NaN	NaN	NaN	S	2	___1
-1,14306	-1,6484	NaN	-0,875263	S	2	___1
NaN	-0,803759	0,0802116	0,291666	S	3	___1
1,12035	-0,227201	2,5018	-1,01706	S	2	___1
NaN	NaN	NaN	NaN	S	2	___1
NaN	NaN	NaN	NaN	S	2	___1
-2,30724	-1,80463	-0,766481	0,329422	S	2	___1
-2,59785	-0,817318	-0,467051	-0,124578	S	3	___1
NaN	NaN	NaN	NaN	S	2	___1
-0,110418	-0,669676	NaN	-1,04637	S	2	___1
0,556351	0,053236	-0,521736	-0,273087	S	2	___1
-2,98939	-3,03449	-2,88445	NaN	S	3	___1
-1,58867	-1,80125	NaN	NaN	S	2	___1
NaN	NaN	NaN	NaN	S	2	___1
NaN	-0,205889	-1,61697	-1,18622	S	2	___1
NaN	NaN	NaN	NaN	S	3	___1
NaN	-1,30026	-2,65764	-0,863195	S	3	___1
-0,232759	NaN	-1,54579	1,23891	S	2	___1
0,458483	1,42665	-2,58359	NaN	S	2	___1
-1,70157	0,978159	NaN	-0,15712	S	3	___1
0,411039	0,564672	-0,577881	-0,296976	S	2	___1
-0,111092	0,463205	NaN	-0,127134	S	2	___1
NaN	NaN	NaN	NaN	S	2	___1
-1,14085	NaN	NaN	NaN	S	2	___1
4,06364	2,68939	0,791613	1,52074	S	2	___1
2,17158	1,29657	3,37617	2,13106	S	2	___1
3,34581	2,83946	4,8278	2,91471	S	3	___1
NaN	NaN	NaN	NaN	S	2	___1
NaN	NaN	NaN	NaN	S	3	___1
0,0905113	0,306376	0,726994	0,11257	S	2	___1
NaN	NaN	NaN	NaN	S	2	___1
-0,812075	NaN	-1,33759	-2,0061	S	3	___1
2,76877	1,44603	3,05638	1,69451	S	3	___1

Polito et al. Universal ready-to-use immunotherapeutic approach for the treatment of cancer: expanded and activated polyclonal γδ memory T cells.
Phosphorylation sites

NaN	-1,13953	-0,791845	-1,40374	S	2	___1
1,10219	0,00691795	-1,00885	-1,50117	S	2	___1
NaN	-1,0988	-3,0064	1,69241	S	3	___1
-1,7462	-2,27991	NaN	NaN	S	2	___1
-0,902769	-2,15928	NaN	-1,11859	S	2	___1
2,03909	0,917496	0,657677	1,03293	S	2	___1
-0,0673065	-0,863459	-1,04787	NaN	S	2	___1
-1,09077	-1,18732	0,182482	-1,1649	S	2	___1
-2,50696	NaN	-0,755596	-1,92461	S	2	___1
0,0638428	0,487034	0,325819	0,246412	S	2	___1
-2,12457	-2,87187	NaN	-0,319717	S	2	___1
NaN	1,26149	1,03411	0,94063	S	3	___1
1,30299	0,628622	-0,391102	1,42498	S	2	___1
NaN	-3,13644	0	-2,24447	S	3	___1
-0,809462	-1,0393	NaN	NaN	S	2	___1
0,0140324	0,375835	-1,39287	0,308731	S	3	___1
-1,65096	-2,69655	-1,87922	-0,717299	S	2	___1
NaN	NaN	NaN	NaN	S	2	___1
2,21551	2,81134	0,919428	3,90869	S	3	___1
NaN	NaN	NaN	NaN	S	3	___1
0,458357	0,473669	-0,377522	1,22454	S	2	___1
NaN	NaN	NaN	NaN	S	2	___1
NaN	NaN	NaN	NaN	S	3	___1
-0,38212	NaN	-1,58757	-0,0676689	S	2	___1
0,0648041	0,369865	NaN	0,422461	S	3	___1
NaN	NaN	NaN	NaN	S	2	___1
NaN	NaN	NaN	NaN	S	2	___1
NaN	NaN	NaN	NaN	S	2	___1
-1,70529	-1,99272	NaN	-1,06335	S	2	___1
1,15835	0,531654	NaN	0,896137	S	2	___1
NaN	0,969398	NaN	NaN	S	3	___1
NaN	NaN	NaN	NaN	S	3	___1
-1,89999	NaN	-2,25901	-2,0454	S	2	___1
1,23034	1,74186	0,527987	0,920969	S	3	___1

-1,37536	-0,553883	NaN	-1,19601	S	3	___1
0,335535	0,471975	-2,13402	-0,234846	S	2	___1
2,17472	1,97693	0,89723	1,85645	S	2	___1
NaN	-1,56071	1,65248	1,25047	S	4	___1
-2,22497	0,172361	-0,794344	1,0862	S	3	___1
NaN	NaN	NaN	NaN	S	3	___1
NaN	NaN	0,713005	0,791756	S	3	___1
NaN	NaN	NaN	NaN	S	2	___1
NaN	NaN	-0,816021	NaN	S	2	___1
NaN	NaN	NaN	NaN	S	2	___1
2,27499	0,940584	1,79812	NaN	S	2	___1
-0,971287	NaN	NaN	-1,82647	S	2	___1
3,37285	2,99719	2,63418	2,46698	S	2	___1
1,00343	0,0280342	NaN	-0,117012	S	2	___1
-0,896435	NaN	NaN	NaN	S	2	___1
-2,37943	-0,501654	-0,637495	NaN	S	2	___1
NaN	NaN	NaN	NaN	S	3	___1
0,00397873	-0,105438	-1,07115	0,614838	S	2	___1
3,29306	2,13418	2,6891	3,82135	S	3	___1
3,01678	0,941767	0,638025	0,417454	S	2	___1
-0,333548	-0,450214	NaN	-1,75919	S	2	___1
-1,66518	-0,141436	NaN	-0,11808	S	2	___1
1,41598	1,10992	0,825104	-0,376455	S	2	___1
NaN	2,0032	-1,37284	-0,33713	S	3	___1
-1,40442	-0,873283	NaN	-0,976027	S	3	___1
0,35812	0,0164661	-2,16354	NaN	S	2	___1
0,515778	-0,220209	-1,42126	NaN	S	2	___1
NaN	-0,865528	NaN	NaN	S	3	___1
-1,84133	-1,54541	NaN	1,91203	S	2	___1
0,671204	1,14729	-1,81181	-0,214319	S	2	___1
1,65014	1,27308	1,18156	0,972137	S	3	___1
-0,853481	-0,122417	NaN	NaN	S	2	___1
NaN	-2,23615	0,217754	0,916882	S	2	___1
2,07801	2,57524	2,62168	2,81727	S	2	___1

Polito et al. Universal ready-to-use immunotherapeutic approach for the treatment of cancer: expanded and activated polyclonal γδ memory T cells.
Phosphorylation sites

-1,35687	-1,33828	NaN	-0,739655	S	3	___1
1,30594	0,557995	-2,10981	0,951452	S	3	___1
0,621939	-1,89336	-0,0219498	2,4003	S	2	___1
2,39697	2,53304	2,08749	2,3801	S	2	___1
-2,72124	-4,14554	NaN	-2,07441	S	2	___1
-1,17275	0,545221	-2,08244	0,686628	S	3	___1
1,69962	1,59915	0,0275249	0,150312	S	2	___1
-1,69045	-3,38907	-1,97469	-1,35859	S	2	___1
-2,52242	0,465019	NaN	-0,840954	S	3	___1
NaN	NaN	NaN	NaN	S	2	___1
0,368008	2,09618	-0,542196	2,97043	S	2	___1
NaN	-1,52677	-1,65333	-1,56136	S	3	___1
0,25127	0,754045	0,106976	-1,01157	S	2	___1
-0,0278473	-1,38795	NaN	-0,490072	S	3	___1
-1,20642	NaN	-0,993011	-0,794302	S	2	___1
-1,36132	-1,63127	-0,323341	-0,658953	S	2	___1
0,716736	0,0749855	-0,324945	-0,459795	S	2	___1
-1,46827	-0,27519	NaN	-1,75452	S	2	___1
-0,052248	-0,409376	1,7361	-0,0758419	S	3	___1
-1,47634	1,90626	NaN	-0,276232	S	3	___1
NaN	NaN	NaN	NaN	S	2	___1
-0,0593071	-0,66222	NaN	1,40866	S	2	___1
NaN	-0,134264	NaN	-3,46783	S	2	___1
2,69224	2,27567	1,7006	4,06749	S	2	___1
NaN	0,665693	NaN	1,51338	S	2	___1
-2,09094	-2,85203	NaN	-0,759861	S	2	___1
-1,5394	-1,78635	NaN	-1,82896	S	2	___1
NaN	NaN	NaN	NaN	S	2	___1
0,494492	1,0712	-0,993984	1,28789	S	3	___1
NaN	0,780849	NaN	0,820692	S	3	___1
NaN	-2,04879	-0,663984	-1,22271	S	3	___1
2,19809	1,64585	-0,0435677	-0,849287	S	2	___1
0,643524	0,212423	2,36537	-0,798983	S	2	___1
NaN	NaN	NaN	NaN	S	2	___1

Polito et al. Universal ready-to-use immunotherapeutic approach for the treatment of cancer: expanded and activated polyclonal γδ memory T cells.
Phosphorylation sites

NaN	NaN	NaN	NaN	S	2	___1
0,586452	NaN	-0,31724	-0,646544	S	2	___1
NaN	-0,00885773	-0,31724	-0,646544	S	2	___1
-1,53759	-0,450733	NaN	0,179977	S	2	___1
3,9257	2,89697	4,42484	3,08064	S	2	___1
3,23768	3,37473	-2,35255	3,26185	S	3	___1
1,78705	3,81307	0,0975437	NaN	S	2	___1
NaN	NaN	NaN	NaN	S	3	___1
NaN	-1,95409	-1,01768	-1,37691	S	2	___1
0,898884	0,473886	0,449631	0,528868	S	2	___1
1,87233	0,92462	NaN	NaN	S	3	___1
3,92353	3,67456	4,53836	3,22128	S	2	___1
-1,25993	-1,74354	-0,17771	-1,95571	S	2	___1
NaN	NaN	NaN	NaN	S	3	___1
0,391546	-0,281717	-0,413464	1,33999	S	2	___1
1,63248	0,270945	NaN	1,0606	S	2	___1
NaN	NaN	NaN	NaN	S	2	___1
NaN	NaN	NaN	NaN	S	2	___1
NaN	NaN	NaN	NaN	S	2	___1
-0,570654	-1,27512	-2,25768	-2,25595	S	2	___1
0,731689	0,86607	-2,30946	0,424557	S	2	___1
-0,386505	-0,276016	NaN	-1,40015	S	2	___1
-1,21138	-1,47229	-2,21534	-1,21949	S	2	___1
NaN	-1,06861	-0,62047	-2,55501	S	2	___1
1,14548	-0,202484	-0,328039	0,668718	S	2	___1
1,13919	0,328093	0,550251	2,18589	S	2	___1
0,178741	2,75238	NaN	1,78355	S	3	___1
-2,37867	NaN	-1,33783	-0,315746	S	2	___1
1,80491	3,16975	0,603685	1,08023	S	2	___1
NaN	1,26679	NaN	0,688549	S	3	___1
NaN	NaN	NaN	NaN	S	4	___1
0,645807	1,42984	-1,27233	0,712776	S	2	___1
NaN	-0,221975	NaN	NaN	S	3	___1
NaN	NaN	NaN	NaN	S	3	___1

NaN	NaN	NaN	NaN	S	2	___1
1,10722	1,54119	NaN	-0,218756	S	2	___1
-0,261255	2,31112	6,64407	1,37347	S	3	___1
NaN	NaN	NaN	NaN	S	2	___1
3,02502	2,27038	0,458227	2,14198	S	2	___1
NaN	NaN	NaN	NaN	S	3	___1
-0,328056	0,509144	1,84655	-0,300577	S	2	___1
NaN	0,371799	-0,192289	NaN	S	3	___1
0,491562	NaN	NaN	-1,99523	S	2	___1
-0,0816441	-2,70715	NaN	-2,65838	S	2	___1
-0,322586	-1,0599	0,330084	-2,12849	S	2	___1
0,0145817	-0,96133	0,892977	-1,67427	S	2	___1
NaN	0,796383	NaN	-0,292292	S	3	___1
NaN	0,725956	-1,11237	-0,730453	S	4	___1
0,0801907	-0,0508728	NaN	NaN	S	2	___1
-0,134481	0,604099	NaN	2,05706	S	2	___1
-1,14238	-2,34346	NaN	-1,71176	S	2	___1
0,385008	-0,915834	NaN	-1,56863	S	2	___1
1,15656	4,1912	4,77692	3,14601	S	3	___1
1,00448	-1,11765	0,776882	-0,527906	S	2	___1
NaN	NaN	NaN	-0,38316	S	3	___1
1,07732	-1,23569	-0,525415	0,599892	S	2	___1
NaN	NaN	NaN	NaN	S	2	___1
0,966898	1,3417	-1,23351	NaN	S	3	___1
NaN	NaN	NaN	NaN	S	2	___1
NaN	-0,0317631	-0,0921364	1,25715	S	2	___1
-2,25765	-2,33246	-1,02695	-2,34086	S	2	___1
-1,5985	-1,64093	NaN	0,534201	S	2	___1
NaN	NaN	NaN	NaN	S	2	___1
NaN	-1,44373	NaN	-1,45221	S	2	___1
NaN	NaN	NaN	0,271042	S	3	___1
NaN	-1,05125	NaN	-0,115387	S	3	___1
NaN	2,12005	0,290461	1,48219	S	2	___1
-0,602339	-0,66222	NaN	-0,99649	S	2	___1

Polito et al. Universal ready-to-use immunotherapeutic approach for the treatment of cancer: expanded and activated polyclonal γδ memory T cells.
Phosphorylation sites

0,978252	0,7495	NaN	NaN	S	2	___1
NaN	NaN	NaN	NaN	S	2	___1
-2,74862	-2,46843	-2,99732	NaN	S	2	___1
-0,182274	0,0864525	0,914621	0,650776	S	2	___1
0,00141907	0,793411	NaN	0,923195	S	2	___1
-2,94501	-1,12072	-1,52964	0,621111	S	2	___1
3,86868	4,17312	3,79222	3,36547	S	2	___1
-0,990572	-1,3055	-0,173033	-1,00512	S	2	___1
-0,460142	-1,24213	-0,330788	-1,22069	S	2	___1
NaN	NaN	NaN	NaN	S	2	___1
-2,35519	-0,831545	0,0326996	-1,02386	S	3	___1
-1,61242	-2,55745	NaN	0,403654	S	2	___1
NaN	-0,761259	-0,165991	1,24564	S	2	___1
NaN	NaN	NaN	NaN	S	2	___1
-0,107206	-0,025425	1,56385	0,0278625	S	3	___1
1,57677	0,875271	1,47932	0,877769	S	2	___1
-0,899405	-0,0706367	NaN	-1,56662	S	2	___1
3,49287	2,67284	NaN	1,3983	S	2	___1
NaN	-1,32726	NaN	NaN	S	3	___1
1,25474	2,50394	-1,72331	0,572918	S	2	___1
-0,566185	-2,14701	NaN	-1,34353	S	2	___1
2,56488	1,1926	1,65632	0,798506	S	3	___1
NaN	0,26206	-1,66258	-1,16338	S	2	___1
0,75185	0,0629177	NaN	0,866379	S	2	___1
NaN	NaN	NaN	NaN	S	2	___1
NaN	-1,82061	-1,7068	-1,79988	S	3	___1
NaN	NaN	NaN	NaN	S	3	___1
3,2849	4,67109	4,09473	3,04927	S	2	___1
NaN	NaN	NaN	NaN	S	3	___1
NaN	NaN	NaN	NaN	S	3	___1
1,8504	0,62612	NaN	1,70741	S	3	___1
0,699366	0,979273	-2,02032	1,79554	S	2	___1
NaN	-1,51578	-1,43611	1,98368	S	3	___1
-1,13741	NaN	NaN	NaN	S	2	___1

-2,57012	NaN	-2,14335	-0,521975	S	2	___1
-1,04272	-0,755234	NaN	-0,565615	S	2	___1
-0,0416527	0,931511	-0,502682	0,400322	S	4	___1
0,90626	1,10091	-2,53193	1,43506	S	2	___1
-1,2417	-0,762802	NaN	-1,08491	S	2	___1
4,44143	4,52401	3,6534	3,62421	S	3	___1
NaN	NaN	-0,0344334	-0,699425	S	3	___1
-0,0685062	-0,583107	-3,44501	-1,34961	S	2	___1
2,24935	1,51098	1,53435	2,30918	S	2	___1
-0,00496292	-0,186352	-0,394127	0,52355	S	2	___1
-1,68376	-2,37284	NaN	-1,64467	S	2	___1
NaN	NaN	NaN	NaN	S	3	___1
-0,404783	-0,553104	NaN	NaN	S	2	___1
NaN	NaN	NaN	NaN	S	3	___1
0,0841522	-1,38016	-0,956736	-0,758949	S	2	___1
1,00667	-0,123325	-1,80187	-1,29893	S	2	___1
NaN	-0,799927	-1,01928	NaN	S	2	___1
NaN	0,494207	NaN	0,272421	S	2	___1
2,30615	1,47906	2,8228	1,59155	S	2	___1
-0,857681	NaN	NaN	-1,434	S	2	___1
-0,0961266	NaN	-0,664391	NaN	S	2	___1
0,430811	0,0138416	0,685049	-1,43761	S	2	___1
-2,0423	-0,968758	NaN	-0,586111	S	2	___1
NaN	NaN	NaN	NaN	S	2	___1
NaN	-2,80392	-2,48766	NaN	S	2	___1
NaN	NaN	NaN	NaN	S	3	___1
NaN	NaN	NaN	NaN	S	2	___1
-1,04637	0,270506	NaN	NaN	S	2	___1
1,0976	0,163321	NaN	NaN	S	2	___1
-0,819759	NaN	-1,34331	NaN	S	2	___1
NaN	NaN	NaN	NaN	S	2	___1
-0,627008	-1,70046	NaN	1,19692	S	2	___1
NaN	-0,643629	-2,8015	-0,855631	S	3	___1
0,16193	NaN	-1,2329	2,32127	S	2	___1

Polito et al. Universal ready-to-use immunotherapeutic approach for the treatment of cancer: expanded and activated polyclonal γδ memory T cells.
Phosphorylation sites

-0,521784	-1,69223	-1,55904	0,136377	S	2	___1
-0,55179	NaN	NaN	0,295717	S	3	___1
NaN	NaN	NaN	NaN	S	2	___1
-1,02703	-2,00225	NaN	-1,42844	S	3	___1
NaN	NaN	NaN	NaN	S	2	___1
NaN	NaN	NaN	NaN	S	3	___1
-1,38728	-1,62736	-1,7751	-2,04077	S	2	___1
-2,10798	-1,37473	NaN	-0,514694	S	2	___1
-1,66782	NaN	-0,974829	0,567286	S	2	___1
0,69029	1,93463	0,590515	1,43515	S	3	___1
-0,59285	0,669415	1,15557	0,738417	S	3	___1
-1,16955	-2,30471	NaN	0,485607	S	2	___1
NaN	NaN	NaN	NaN	S	2	___1
1,0031	-0,568758	-1,86482	NaN	S	2	___1
NaN	NaN	NaN	NaN	S	2	___1
2,93664	3,63576	NaN	1,70432	S	2	___1
0,869753	-0,102425	0,861553	NaN	S	3	___1
1,05336	1,06078	1,56818	0,472094	S	2	___1
1,81342	-0,572248	NaN	0,0659103	S	2	___1
1,37729	-0,70903	NaN	4,48974	S	2	___1
-0,203859	2,09053	0,202524	0,63069	S	3	___1
NaN	NaN	NaN	NaN	S	2	___1
-0,206177	1,83252	1,05476	NaN	S	2	___1
NaN	-0,315369	-0,601877	-1,48633	S	3	___1
0,155163	NaN	NaN	NaN	S	2	___1
2,29962	1,15626	-0,541382	2,71606	S	1	___1
-2,00121	-0,880241	0,407156	NaN	S	2	___1
NaN	NaN	NaN	NaN	S	2	___1
NaN	NaN	NaN	NaN	S	2	___1
2,44153	1,1272	2,90558	1,54254	S	2	___1
-1,4589	-1,04242	NaN	-1,26927	S	2	___1
-0,68939	-1,3125	-2,78386	0,479504	S	2	___1
-1,35812	-0,0850353	NaN	-0,623226	S	2	___1
-2,26566	NaN	-2,77265	-0,986849	S	2	___1

NaN	NaN	NaN	NaN	S	3	___1
-0,517263	-0,855482	-0,0128345	1,90242	S	2	___1
NaN	-3,97208	-3,64734	-1,1366	S	3	___1
-0,156286	-0,0185757	-0,0156555	1,37012	S	2	___1
-1,27343	-0,387995	NaN	-0,103888	S	2	___1
NaN	NaN	NaN	NaN	S	2	___1
-1,64738	NaN	-0,859108	-0,0854034	S	2	___1
-2,40367	-2,55718	NaN	-0,311001	S	2	___1
0,588964	-0,740597	NaN	2,07612	S	2	___1
0,585991	1,41684	-1,80683	0,00437355	S	2	___1
0,297504	-0,388491	NaN	-0,335413	S	2	___1
1,41182	1,48332	0,316822	-0,680042	S	2	___1
-0,519497	-2,54368	-1,92887	NaN	S	2	___1
-0,519497	-2,54368	-1,92887	NaN	S	2	___1
-0,888844	-0,0930977	-0,0625229	-0,975887	S	2	___1
-1,17439	-0,760874	NaN	-1,2615	S	2	___1
0,525608	NaN	-0,439045	-1,79491	S	3	___1
NaN	-4,60092	1,55097	NaN	S	3	___1
2,38036	2,62609	-0,320898	0,603504	S	2	___1
-0,381758	NaN	2,9385	0,357759	S	2	___1
2,51301	2,82092	-0,0827389	1,92363	S	2	___1
-0,695381	-0,855619	NaN	-0,0865479	S	2	___1
2,00962	2,02928	0,32988	1,33619	S	2	___1
NaN	NaN	NaN	NaN	S	2	___1
-1,30561	-0,913261	-1,60085	-1,87556	S	3	___1
1,72594	1,08138	2,4751	0,436792	S	2	___1
1,72101	0,684065	2,79832	1,84417	S	2	___1
-0,190275	-0,877592	1,20092	0,135866	S	2	___1
NaN	-1,74372	-0,274302	-0,342882	S	2	___1
-2,09273	0,329781	-2,14788	1,5301	S	2	___1
NaN	0,120735	1,4019	1,28616	S	2	___1
NaN	NaN	NaN	NaN	S	2	___1
NaN	NaN	NaN	NaN	S	2	___1
NaN	NaN	-2,15387	NaN	S	2	___1

Polito et al. Universal ready-to-use immunotherapeutic approach for the treatment of cancer: expanded and activated polyclonal γδ memory T cells.
Phosphorylation sites

-0,463961	-0,869955	NaN	NaN	S	2	___1
-0,432571	0,314564	-0,220972	-1,38845	S	3	___1
0,655804	-0,307938	NaN	-0,491488	S	2	___1
NaN	-1,37726	NaN	0,129028	S	2	___1
NaN	NaN	0,419754	NaN	S	2	___1
NaN	NaN	1,13304	-2,13352	S	2	___1
0,722712	2,22287	-0,221245	-0,200552	S	2	___1
-1,18475	-1,10267	NaN	NaN	S	2	___1
NaN	0,78054	0,909798	0,775974	S	4	___1
NaN	-1,92194	-1,09144	-0,266872	S	2	___1
-0,15066	-0,981855	NaN	-3,25385	S	2	___1
NaN	NaN	NaN	NaN	S	2	___1
NaN	1,27214	2,59774	NaN	S	2	___1
0,758583	0,599905	2,92636	0,74514	S	4	___1
NaN	NaN	0,411991	NaN	S	4	___1
-1,72405	NaN	-0,378611	NaN	S	2	___1
NaN	NaN	NaN	NaN	S	3	___1
-2,65818	-3,04378	NaN	-3,50442	S	2	___1
-0,815485	-0,782484	-1,21475	0,352852	S	2	___1
-2,41718	-1,62168	NaN	-2,44441	S	2	___1
NaN	NaN	NaN	NaN	S	2	___1
NaN	-1,24858	NaN	NaN	S	3	___1
-0,159031	-0,528406	NaN	NaN	S	2	___1
NaN	NaN	NaN	NaN	S	2	___1
3,83127	4,56532	7,30895	3,55273	S	3	___1
1,00814	2,84664	NaN	0,0862656	S	2	___1
NaN	NaN	NaN	NaN	S	2	___1
0,799547	2,36033	-0,432467	0,599134	S	2	___1
-1,26691	-1,91918	NaN	NaN	S	3	___1
-1,48466	-1,10807	NaN	-0,827299	S	2	___1
-1,39975	0,290907	NaN	-2,18926	S	2	___1
-2,16993	-2,17522	NaN	NaN	S	2	___1
1,61957	1,02584	0,618587	0,0918808	S	2	___1
NaN	NaN	NaN	NaN	S	2	___1

0,444344	0,174713	1,58658	-0,131027	S	2	___1
NaN	NaN	NaN	NaN	S	2	___1
NaN	NaN	NaN	NaN	S	2	___1
0,743969	NaN	0,732607	NaN	S	2	___1
0,288681	-1,28454	-0,619862	-1,47066	S	3	___1
3,89153	6,98439	7,61869	6,28069	S	3	___1
4,41494	3,0305	3,28857	2,77269	S	2	___1
1,90739	2,82766	3,76442	2,6854	S	3	___1
-2,83959	NaN	-1,19778	-1,52073	S	2	___1
1,07553	0,444391	-1,97423	0,226088	S	2	___1
-0,561575	NaN	NaN	-1,72231	S	2	___1
4,9451	4,13545	4,63782	4,11882	S	2	___1
NaN	NaN	NaN	NaN	S	2	___1
NaN	NaN	NaN	NaN	S	2	___1
1,05826	0,621593	-0,739613	0,85438	S	2	___1
NaN	NaN	-0,54974	NaN	S	3	___1
3,32822	2,63747	1,12964	4,53904	S	4	___1
0,729685	-0,842512	1,28842	-0,308126	S	2	___1
NaN	-0,135345	0,543943	-0,610331	S	2	___1
NaN	-0,749489	1,39554	NaN	S	2	___1
NaN	-1,34921	NaN	-0,959898	S	2	___1
NaN	NaN	NaN	NaN	S	2	___1
NaN	NaN	1,90769	NaN	S	3	___1
1,39861	0,523188	1,1638	0,167498	S	2	___1
0,766914	0,852911	-0,193434	-1,69658	S	2	___1
-0,360294	NaN	NaN	-0,995884	S	2	___1
NaN	NaN	NaN	NaN	S	2	___1
NaN	NaN	NaN	NaN	S	2	___1
-1,92635	-0,340292	NaN	-0,982306	S	2	___1
NaN	NaN	NaN	NaN	S	2	___1
NaN	-0,188423	1,31224	-1,00535	S	2	___1
0,713549	0,727558	1,6174	0,433344	S	2	___1
0,547228	0,00185585	NaN	-0,463472	S	2	___1
2,53237	2,28359	2,8451	2,12753	S	2	___1

1,2334	1,65372	0,861683	1,87853	S	2	___1
0,624392	0,838995	NaN	1,3884	S	2	___1
-0,159494	-1,01753	-0,638544	-1,00676	S	2	___1
0,670094	0,800087	-0,733835	-0,0372066	S	3	___1
-1,46339	0,963385	1,372	0,855675	S	3	___1
1,34219	0,413597	-1,69236	-1,08699	S	2	___1
0,466955	-2,67533	NaN	NaN	S	2	___1
1,57689	2,27341	NaN	1,39634	S	4	___1
NaN	NaN	NaN	NaN	S	2	___1
1,19353	1,22058	-2,0991	-0,0797729	S	2	___1
NaN	NaN	NaN	NaN	S	2	___1
1,19353	1,22058	-2,0991	-0,0797729	S	2	___1
NaN	NaN	NaN	NaN	S	2	___1
NaN	NaN	NaN	NaN	S	2	___1
-0,262875	-0,154789	-0,650076	-1,334	S	2	___1
NaN	-0,489046	NaN	-1,46833	S	3	___1
-0,142452	-1,08637	NaN	-0,699728	S	2	___1
-2,21225	-1,96334	NaN	NaN	S	2	___1
NaN	-3,28028	-0,397097	NaN	S	2	___1
1,96559	1,70925	2,74387	2,53488	S	2	___1
1,56605	1,27934	1,60606	0,198408	S	2	___1
NaN	NaN	NaN	NaN	S	2	___1
0,0221767	1,34341	0,955639	-0,70129	S	3	___1
-0,907181	-0,891867	-1,6097	-1,02319	S	2	___1
1,58014	1,20198	2,31289	0,324568	S	2	___1
-0,555248	-1,2545	NaN	NaN	S	2	___1
1,44044	0,575699	1,63669	NaN	S	2	___1
-1,59588	NaN	-2,42645	NaN	S	2	___1
NaN	NaN	NaN	0,0766354	S	2	___1
0,675636	0,0536003	-0,32378	NaN	S	2	___1
NaN	0,594494	-2,00026	-0,386616	S	3	___1
2,11135	1,98637	2,87491	1,41165	S	2	___1
-0,19912	-0,327492	-0,622833	-0,907021	S	2	___1
NaN	NaN	NaN	NaN	S	2	___1

0,516409	0,14719	1,78542	-0,913094	S	2	___1
NaN	NaN	NaN	NaN	S	2	___1
NaN	NaN	NaN	NaN	S	2	___1
NaN	-2,95847	0,946249	0,430044	S	2	___1
NaN	NaN	NaN	NaN	S	3	___1
1,61698	0,113398	-0,64279	NaN	S	2	___1
-2,14144	NaN	-0,507637	-0,457285	S	2	___1
-0,587841	-2,31079	NaN	0,563232	S	2	___1
NaN	0,608181	NaN	-0,590372	S	3	___1
-0,131329	0,522924	NaN	-0,631332	S	3	___1
3,59684	0,23501	-0,784891	0,378017	S	2	___1
4,08933	5,98941	NaN	5,66829	S	2	___1
4,08266	4,16532	5,45995	3,17488	S	3	___1
0,694086	-0,259953	1,02842	-0,4391	S	2	___1
NaN	0,943069	1,93223	0,752111	S	2	___1
-0,169241	-0,412601	-2,14943	-1,29198	S	3	___1
0,192905	-0,144447	NaN	-0,5376	S	3	___1
NaN	NaN	NaN	NaN	S	2	___1
NaN	NaN	NaN	-0,872986	S	2	___1
NaN	0,359446	NaN	-0,648415	S	2	___1
-1,64478	-1,00805	NaN	-1,14135	S	2	___1
-1,03951	0,468584	NaN	-0,553909	S	2	___1
NaN	NaN	NaN	NaN	S	4	___1
NaN	-0,341637	NaN	-0,875692	S	2	___1
NaN	NaN	NaN	NaN	S	4	___1
NaN	NaN	NaN	NaN	S	2	___1
-2,57399	-2,85345	NaN	-0,704798	S	3	___1
NaN	NaN	NaN	NaN	S	2	___1
NaN	NaN	NaN	NaN	S	2	___1
0,815779	0,894196	-0,727539	0,688868	S	3	___1
NaN	NaN	NaN	NaN	S	3	___1
-1,57076	-0,931404	NaN	-1,41813	S	2	___1
0,129198	-0,569433	NaN	-0,315659	S	2	___1
-0,163408	-0,357668	0,232765	-0,456642	S	2	___1

0,618807	1,22327	0,493603	-0,708124	S	2	___1
NaN	-3,05482	-0,791361	NaN	S	3	___1
NaN	NaN	NaN	NaN	S	2	___1
NaN	NaN	NaN	NaN	S	2	___1
-1,22517	-1,89948	-1,32087	-3,362	S	2	___1
NaN	NaN	NaN	NaN	S	3	___1
1,59535	2,58412	-0,163122	NaN	S	3	___1
NaN	NaN	NaN	NaN	S	2	___1
NaN	NaN	NaN	NaN	S	2	___1
NaN	-1,57324	NaN	NaN	S	2	___1
-2,44518	-2,28478	-0,676207	-1,22874	S	2	___1
NaN	-0,702108	-0,157581	0,823341	S	2	___1
-0,86158	NaN	NaN	NaN	S	2	___1
NaN	NaN	NaN	NaN	S	2	___1
-0,0698147	-1,52884	NaN	-1,28368	S	2	___1
-0,428509	0,743673	-0,276075	-0,504158	S	2	___1
1,13615	-1,80784	NaN	0,351421	S	2	___1
1,03679	1,04899	-1,1005	0,57435	S	2	___1
-2,00747	-2,92637	NaN	NaN	S	2	___1
0,0870895	1,24831	1,54076	-2,50018	S	3	___1
-1,70766	0,476885	-0,514303	-0,761488	S	2	___1
-1,26608	0,81002	0,481253	-0,533863	S	3	___1
-0,309475	0,376595	-1,48095	NaN	S	2	___1
1,77415	1,96733	0,96497	1,86709	S	2	___1
1,98076	2,38089	1,92041	4,34705	S	2	___1
-1,86196	-0,408672	NaN	1,48102	S	3	___1
-0,99395	-1,8714	NaN	-3,26003	S	2	___1
-1,84967	-0,828043	-0,558865	NaN	S	3	___1
NaN	NaN	NaN	NaN	S	2	___1
NaN	-0,264317	-0,565462	-1,08665	S	3	___1
-0,0648403	-0,224985	-0,496265	-0,757166	S	2	___1
-1,23223	-1,31947	-0,337595	-1,71115	S	2	___1
NaN	0,169668	0,669579	0,229729	S	2	___1
NaN	1,82567	-0,0480137	0,478413	S	2	___1

0,794922	1,24134	3,41518	1,62323	S	2	___1
1,4343	1,05696	0,533545	-0,552191	S	2	___1
NaN	NaN	NaN	NaN	S	2	___1
NaN	NaN	NaN	NaN	S	2	___1
3,04841	2,65032	2,23672	4,00837	S	2	___1
-0,710724	-1,13863	-1,70847	0,065239	S	2	___1
-2,89132	-1,09749	-2,60906	0,944286	S	2	___1
NaN	-0,313013	0,176708	0,190279	S	2	___1
-0,839092	-0,485222	NaN	-0,795807	S	2	___1
0,575636	0,368868	NaN	-0,498421	S	2	___1
-2,07398	-2,75127	-0,854429	0,0944061	S	2	___1
-2,56345	-2,19126	2,7317	-0,276913	S	2	___1
-0,734566	-1,9494	-1,50956	0,023859	S	2	___1
NaN	NaN	NaN	NaN	S	3	___1
NaN	-1,99928	-0,666864	1,83054	S	3	___1
0,752157	1,02606	0,703604	NaN	S	3	___1
0,75643	2,63978	2,86766	1,09708	S	2	___1
2,87677	2,27943	2,34476	1,84971	S	2	___1
0,691235	0,259785	-2,04671	-1,75444	S	2	___1
NaN	-1,3688	-1,0423	NaN	S	2	___1
-1,03596	-1,20126	-1,65377	-1,51108	S	2	___1
-0,322716	-1,28192	-0,175396	-0,0279331	S	2	___1
1,4806	NaN	-0,386074	0,532419	S	2	___1
1,07161	-0,244965	1,40831	-0,60124	S	3	___1
0,941036	0,6038	0,810183	-0,53607	S	2	___1
-2,41002	-1,74796	-0,116556	-1,26769	S	2	___1
1,11125	-0,0567741	0,635092	1,41218	S	2	___1
-0,0744762	-0,473072	-1,48975	-0,706211	S	2	___1
NaN	NaN	NaN	NaN	S	2	___1
2,79371	2,58462	NaN	NaN	S	2	___1
NaN	1,82306	0,68054	1,76354	S	3	___1
0,456364	-0,122087	1,07025	NaN	S	2	___1
2,32058	2,15264	0,697159	1,58321	S	2	___1
4,81009	4,11289	2,27454	2,88407	S	2	___1

NaN	-0,383043	0,577753	-0,24012	S	2	___1
2,96714	2,12923	2,04216	2,94575	S	2	___1
NaN	NaN	NaN	NaN	S	2	___1
1,77029	-1,78643	4,21365	1,1257	S	2	___1
-1,26225	-0,864977	NaN	NaN	S	2	___1
1,04821	0,676023	0,322027	1,56638	S	2	___1
0,372324	NaN	0,821947	0,776794	S	2	___1
NaN	-1,02438	NaN	-1,64636	S	2	___1
-2,11991	-2,19282	NaN	-2,63912	S	2	___1
-1,1127	0,864113	1,51892	0,137592	S	2	___1
-0,85605	-0,451355	-0,393057	-0,881809	S	3	___1
-0,212626	3,20256	1,86072	2,82318	S	3	___1
-0,0100479	0,134335	NaN	-1,77137	S	3	___1
NaN	NaN	NaN	NaN	S	2	___1
-0,0576115	-0,477604	-0,893932	NaN	S	2	___1
0,529598	-0,0990105	1,13368	0,179708	S	2	___1
1,3013	2,6283	1,8532	0,523012	S	3	___1
0,587444	0,408237	-1,22728	0,19528	S	2	___1
NaN	NaN	NaN	NaN	S	2	___1
NaN	NaN	NaN	NaN	S	2	___1
1,23137	0,516155	1,48971	-0,174541	S	2	___1
-0,667873	0,184284	-0,79257	1,11817	S	3	___1
-0,190474	0,072176	-1,16646	NaN	S	3	___1
2,06084	2,17822	0,371008	2,17182	S	3	___1
-0,373835	-0,0286674	-1,37055	0,916882	S	2	___1
NaN	NaN	0,562201	NaN	S	3	___1
NaN	-1,48306	1,66067	-2,08252	S	2	___1
NaN	NaN	NaN	NaN	S	2	___1
0,531492	0,702579	1,75229	0,579424	S	2	___1
1,61732	1,1697	-0,88789	1,15924	S	2	___1
NaN	-1,97429	-1,27808	-0,439068	S	2	___1
-1,50379	-0,229954	NaN	-1,12624	S	3	___1
1,87913	1,44831	NaN	1,38167	S	2	___1
1,30734	0,457752	0,393223	NaN	S	2	___1

0,849628	0,457697	NaN	0,196405	S	2	___1
0,760752	-0,477816	NaN	-0,772331	S	2	___1
-3,91823	-1,66253	NaN	0,395931	S	2	___1
-0,837296	-0,491177	2,39315	0,5721	S	2	___1
3,95753	3,78145	5,13972	4,3528	S	2	___1
-1,12527	-1,44616	-0,502188	NaN	S	2	___1
1,42194	0,505678	0,369873	0,881208	S	2	___1
NaN	NaN	NaN	NaN	S	2	___1
NaN	NaN	NaN	NaN	S	2	___1
3,94515	3,68019	3,92489	3,36951	S	2	___1
0,864586	0,157555	1,42297	NaN	S	2	___1
NaN	NaN	NaN	NaN	S	2	___1
NaN	1,13178	1,68273	2,59876	S	2	___1
NaN	NaN	NaN	NaN	S	2	___1
2,05776	2,18238	3,34621	4,4005	S	2	___1
2,35836	1,32513	0,23678	0,871895	S	2	___1
1,70005	2,30326	NaN	NaN	S	2	___1
1,94567	1,99295	2,82937	3,48832	S	2	___1
0,298349	-0,246943	0,988035	1,64061	S	2	___1
0,260612	-2,2219	1,04227	1,63933	S	2	___1
-1,86625	-2,21063	-1,58485	-1,97796	S	2	___1
1,53602	0,943226	1,91295	1,13149	S	2	___1
NaN	0,3361	0,906946	0,734901	S	2	___1
-2,96259	0,570656	-1,92515	-1,2083	S	2	___1
5,47384	4,60214	3,6015	5,39516	S	2	___1
7,26663	5,06653	6,73746	5,38578	S	2	___1
-1,91141	NaN	NaN	-1,47678	S	2	___1
0,600618	-0,870785	-1,86533	NaN	S	2	___1
-0,662653	NaN	NaN	NaN	S	3	___1
1,07727	0,45499	2,14298	0,748909	S	2	___1
-0,80764	-1,38423	-2,22684	-1,11376	S	2	___1
NaN	NaN	NaN	NaN	S	2	___1
-0,601076	-0,946487	NaN	-2,29473	S	2	___1
-1,48311	-1,49651	-1,34698	NaN	S	2	___1

NaN	0,531235	0,66342	0,477621	S	3	___1
-1,06626	-1,10743	-0,0351486	NaN	S	2	___1
-2,3681	-1,27014	NaN	NaN	S	2	___1
-0,649441	0,0529442	1,40155	-0,106106	S	2	___1
1,02305	1,40995	1,09334	1,23792	S	2	___1
NaN	NaN	NaN	NaN	S	2	___1
1,59363	1,10168	NaN	0,77529	S	2	___1
-0,211863	-1,25965	-2,30347	-0,915081	S	2	___1
NaN	NaN	NaN	NaN	S	2	___1
-0,255081	2,287	NaN	-0,748129	S	3	___1
NaN	NaN	NaN	NaN	S	2	___1
2,04942	3,1335	NaN	2,01785	S	2	___1
0,374784	NaN	NaN	-0,200497	S	2	___1
1,42607	1,48711	1,75091	1,22815	S	2	___1
-1,06292	-1,00803	-3,82332	NaN	S	2	___1
NaN	-1,44259	NaN	NaN	S	2	___1
NaN	0,916452	-1,73078	-1,10039	S	3	___1
-0,471724	-1,44988	NaN	NaN	S	2	___1
NaN	NaN	NaN	NaN	S	2	___1
-1,8313	-2,82176	0,847782	NaN	S	2	___1
-0,529215	-1,27732	-0,862194	-1,85983	S	2	___1
1,169	-0,193701	-0,799967	NaN	S	2	___1
-0,163797	-1,56561	NaN	-0,218918	S	2	___1
-1,16504	-1,96071	-1,89212	NaN	S	2	___1
NaN	NaN	NaN	NaN	S	2	___1
0,0149937	0,206787	1,57272	0,0993576	S	2	___1
NaN	-1,26552	NaN	NaN	S	2	___1
NaN	NaN	NaN	NaN	S	3	___1
NaN	-1,40421	NaN	-1,29106	S	2	___1
NaN	NaN	NaN	NaN	S	3	___1
NaN	NaN	NaN	NaN	S	2	___1
-0,610304	-1,19401	-0,76486	NaN	S	2	___1
0,314098	NaN	-1,65209	-0,606047	S	2	___1
NaN	0,973457	2,09056	1,08222	S	3	___1

Polito et al. Universal ready-to-use immunotherapeutic approach for the treatment of cancer: expanded and activated polyclonal γδ memory T cells.
Phosphorylation sites

-1,53709	-0,325686	NaN	NaN	S	2	___1
-2,08926	-0,883175	-0,440119	-1,1289	S	3	___1
-1,46291	-2,18036	NaN	1,11287	S	2	___1
NaN	-2,31977	-2,36833	-0,290686	S	2	___1
1,24994	1,92054	NaN	1,04218	S	2	___1
NaN	NaN	NaN	NaN	S	2	___1
NaN	NaN	NaN	NaN	S	2	___1
NaN	-1,77075	NaN	NaN	S	3	___1
NaN	0,995863	-0,607113	0,56926	S	2	___1
0,611816	2,58727	5,0675	NaN	S	3	___1
0,0648365	2,15117	2,414	1,36803	S	2	___1
0,687647	0,504076	NaN	-0,443329	S	2	___1
-0,0817547	1,29761	1,9049	0,194195	S	3	___1
-2,53349	0,326944	NaN	-1,24247	S	2	___1
NaN	-0,322365	-0,319649	-1,01308	S	2	___1
1,13462	0,0189362	-1,98507	NaN	S	2	___1
2,77536	NaN	3,35401	2,39554	S	3	___1
5,64514	4,60604	1,11166	4,10105	S	4	___1
NaN	NaN	NaN	NaN	S	2	___1
6,41905	5,61815	5,98127	4,81872	S	2	___1
5,67671	5,693	6,16284	5,24135	S	2	___1
5,54354	4,56016	3,21686	4,23943	S	3	___1
NaN	NaN	NaN	NaN	S	2	___1
-2,48505	-2,73357	NaN	-0,698208	S	2	___1
1,54031	0,82126	2,45095	1,66394	S	2	___1
NaN	0,318155	-0,296568	-0,15214	S	3	___1
2,09879	1,97168	NaN	1,52246	S	3	___1
2,59518	1,80931	-2,65058	1,44042	S	2	___1
-1,08297	-1,73196	NaN	-1,63351	S	2	___1
1,32183	0,66268	-2,55076	0,478748	S	2	___1
5,64625	5,62988	6,12469	5,57737	S	2	___1
6,51987	5,63359	NaN	5,91375	S	3	___1
-2,25093	-1,56362	NaN	-2,19977	S	3	___1
-1,03624	-1,47381	-0,818645	-0,860769	S	2	___1

Polito et al. Universal ready-to-use immunotherapeutic approach for the treatment of cancer: expanded and activated polyclonal γδ memory T cells.
Phosphorylation sites

2,01717	0,781115	NaN	NaN	S	2	___1
1,8763	0,356371	1,20133	1,38095	S	2	___1
NaN	NaN	3,43731	-2,04927	S	2	___1
-1,27059	-1,2641	-0,76775	-1,80839	S	2	___1
-2,63725	-0,663179	1,07832	-0,920612	S	3	___1
4,87493	4,04628	0,933388	2,74553	S	2	___1
0,204107	0,349844	NaN	0,71063	S	3	___1
0,423338	-0,0669003	-1,45182	-1,10437	S	3	___1
NaN	NaN	NaN	NaN	S	2	___1
-0,225451	1,28012	0,0469627	0,379044	S	3	___1
0,305405	NaN	NaN	NaN	S	2	___1
-0,555962	-0,651585	NaN	0,153244	S	2	___1
NaN	NaN	NaN	NaN	S	3	___1
-2,44984	4,31942	2,44528	3,31071	S	2	___1
1,87667	1,17495	2,742	NaN	S	2	___1
-0,297459	0,102636	-1,44439	-1,25668	S	2	___1
0,321852	-0,65361	-2,0582	0,287584	S	2	___1
0,390251	-0,364386	NaN	2,25936	S	3	___1
-0,444357	-0,639608	NaN	-1,63948	S	2	___1
NaN	NaN	NaN	NaN	S	2	___1
NaN	-1,72631	0,801662	1,67351	S	2	___1
NaN	NaN	NaN	NaN	S	3	___1
-0,126404	-1,74676	-0,941324	1,98195	S	2	___1
1,04138	3,57892	1,24017	3,98703	S	4	___1
1,6021	0,46183	1,35563	0,525356	S	2	___1
0,3134	1,08427	-1,23526	0,708769	S	3	___1
2,27169	2,27907	1,25497	0,895885	S	2	___1
-0,0169029	0,384804	NaN	-1,82008	S	2	___1
-0,287994	-0,534761	NaN	-2,0398	S	2	___1
NaN	NaN	NaN	NaN	S	4	___1
0,745312	NaN	0,504734	-1,50702	S	3	___1
NaN	NaN	NaN	NaN	S	2	___1
NaN	NaN	NaN	NaN	S	2	___1
-1,37644	NaN	-2,85903	NaN	S	2	___1

NaN	NaN	NaN	NaN	S	4	___1
1,4937	1,77045	NaN	NaN	S	2	___1
1,36564	1,24163	NaN	-0,018364	S	2	___1
1,93011	-0,0280495	-0,460278	0,540157	S	2	___1
0,98115	-1,03384	-1,19373	-0,308243	S	2	___1
-0,543636	0,51074	NaN	0,817106	S	3	___1
-0,288376	-2,23034	-2,43083	NaN	S	3	___1
NaN	-2,6763	-2,67171	0,349253	S	2	___1
NaN	NaN	NaN	NaN	S	3	___1
0,780453	-0,159523	-0,276836	NaN	S	2	___1
2,33802	1,64391	1,642	0,356699	S	2	___1
NaN	NaN	1,06225	NaN	S	3	___1
4,328	5,05677	4,4849	3,73328	S	2	___1
1,71859	NaN	-2,49972	1,29239	S	2	___1
-2,1536	-3,37406	NaN	-3,14249	S	2	___1
-1,64402	NaN	-2,20715	-0,356403	S	2	___1
-1,39309	-0,967157	0,344398	-0,520731	S	2	___1
NaN	2,72776	1,19971	1,68769	S	4	___1
2,44248	3,12119	0,118919	0,661316	S	3	___1
NaN	-0,207989	-0,116455	0,408445	S	3	___1
0,100401	-2,71638	NaN	-3,2361	S	3	___1
-1,05045	-1,99767	0,0991306	-1,62193	S	2	___1
NaN	NaN	NaN	NaN	S	4	___1
NaN	NaN	NaN	NaN	S	2	___1
-0,11027	2,20119	1,41129	1,89128	S	2	___1
NaN	NaN	NaN	NaN	S	2	___1
NaN	NaN	NaN	NaN	S	3	___1
NaN	-0,573376	-0,879173	-0,109411	S	3	___1
NaN	-1,81321	-1,98414	NaN	S	2	___1
-1,12293	-1,51825	-2,93772	NaN	S	2	___1
NaN	NaN	NaN	NaN	S	2	___1
NaN	NaN	NaN	NaN	S	2	___1
1,56464	0,70672	2,01033	NaN	S	2	___1
-0,506542	-1,26364	NaN	0,990175	S	1	___1

0,67466	-0,35195	NaN	0,520155	S	3	___1
1,56499	2,3812	0,0321541	2,69957	S	2	___1
NaN	-0,740849	-0,724537	2,23339	S	2	___1
-0,539904	-0,94903	NaN	NaN	S	2	___1
-0,705402	NaN	NaN	NaN	S	2	___1
NaN	-0,454155	0,301645	-1,57406	S	2	___1
NaN	0,231205	NaN	NaN	S	3	___1
1,15044	0,0753441	NaN	-0,279327	S	2	___1
NaN	-1,02106	1,34422	-1,80306	S	2	___1
-0,524023	-1,54395	NaN	-1,15323	S	2	___1
NaN	-0,211231	NaN	-0,26206	S	2	___1
0,936789	0,306559	-1,55582	0,370409	S	3	___1
-1,8313	-0,966076	NaN	-2,10411	S	2	___1
-0,0390511	NaN	NaN	NaN	S	2	___1
1,33768	-0,377224	NaN	-0,707474	S	3	___1
-1,3291	-2,63386	NaN	-0,815914	S	2	___1
-0,880081	-1,8373	-1,32384	0,138678	S	2	___1
NaN	NaN	NaN	NaN	S	2	___1
0,670834	0,660187	0,685629	0,243387	S	3	___1
NaN	NaN	NaN	NaN	S	3	___1
-1,33958	-2,0166	NaN	-1,87333	S	2	___1
NaN	NaN	NaN	NaN	S	2	___1
0,751335	0,69375	NaN	-0,0418777	S	2	___1
3,64259	1,22982	2,37577	NaN	S	2	___1
NaN	1,07804	0,825891	0,0698891	S	3	___1
1,26932	0,534534	1,77532	0,252089	S	2	___1
NaN	0,589869	NaN	0,540302	S	3	___1
-0,741442	-1,38429	-1,22826	-2,36178	S	2	___1
1,78946	0,201193	-1,25029	NaN	S	2	___1
NaN	NaN	NaN	NaN	S	3	___1
NaN	NaN	NaN	NaN	S	2	___1
NaN	NaN	NaN	NaN	S	3	___1
-0,941837	-1,83584	NaN	1,21469	S	2	___1
1,64461	2,94824	0,704889	0,775427	S	2	___1

-0,897209	-1,22295	NaN	-1,3876	S	2	___1
1,64461	2,94824	0,704889	0,775427	S	2	___1
0,415203	0,544495	-0,995375	-0,154745	S	2	___1
0,705902	-0,150913	-1,79394	-1,1988	S	2	___1
-0,910889	-0,376436	-1,27926	-0,992523	S	2	___1
-3,69958	NaN	0,226255	-2,08939	S	2	___1
-1,94985	-3,13491	NaN	-1,05447	S	2	___1
NaN	-0,127459	-0,652672	-1,23198	S	2	___1
NaN	0,371508	NaN	-1,15016	S	3	___1
NaN	-0,246674	-4,75452	-1,85796	S	3	___1
-1,28461	-1,17619	-1,52649	NaN	S	2	___1
-1,85167	NaN	-2,85831	NaN	S	2	___1
NaN	-0,166225	-1,15315	0,312357	S	3	___1
0,370663	NaN	NaN	-0,358353	S	2	___1
-0,129242	0,268934	NaN	-0,867933	S	2	___1
NaN	NaN	NaN	NaN	S	3	___1
NaN	NaN	NaN	NaN	S	2	___1
-0,321157	-0,477604	NaN	NaN	S	2	___1
1,19203	0,524401	0,38114	0,782253	S	2	___1
2,15292	3,36578	2,52002	2,88872	S	3	___1
1,71764	1,04481	1,5402	1,12162	S	2	___1
2,83586	2,61166	3,47262	3,22008	S	2	___1
NaN	NaN	NaN	NaN	S	2	___1
-0,744108	-0,930264	-2,51122	-0,986755	S	2	___1
0,540834	0,0243874	1,19596	-0,0960808	S	2	___1
NaN	NaN	NaN	NaN	S	2	___1
-0,790878	0,172159	2,94586	NaN	S	2	___1
NaN	NaN	NaN	NaN	S	2	___1
-1,84642	NaN	0,606623	NaN	S	3	___1
0,565416	-1,81548	-2,07018	-0,856775	S	2	___1
NaN	NaN	NaN	NaN	S	2	___1
NaN	NaN	NaN	NaN	S	2	___1
0,443121	0,0902309	0,599806	-0,211412	S	3	___1
-0,754009	-2,37583	NaN	-0,401546	S	3	___1

Polito et al. Universal ready-to-use immunotherapeutic approach for the treatment of cancer: expanded and activated polyclonal γδ memory T cells.
Phosphorylation sites

1,07957	2,57804	2,02533	2,3417	S	4	___1
1,65069	-0,301384	0,990028	0,666758	S	2	___1
-0,44026	-1,97116	NaN	-0,29525	S	2	___1
3,24127	2,16672	4,13635	1,99618	S	3	___1
-0,451067	-1,59403	-2,57788	-0,402878	S	4	___1
-1,22792	0,65822	0,286173	NaN	S	3	___1
0,772968	1,00974	1,23415	-1,58183	S	2	___1
0,529453	-0,791769	0,694141	-1,07367	S	2	___1
1,32473	1,32165	1,44502	0,59292	S	2	___1
NaN	-1,38889	-1,1055	0,570412	S	2	___1
-2,30836	0,09478	0,51692	-1,27176	S	2	___1
0,550283	1,83037	NaN	-0,237164	S	2	___1
NaN	NaN	NaN	NaN	S	2	___1
NaN	NaN	NaN	NaN	S	3	___1
NaN	NaN	NaN	NaN	S	2	___1
NaN	NaN	NaN	NaN	S	2	___1
NaN	NaN	NaN	NaN	S	3	___1
0,860146	0,0987492	1,42149	0,0932884	S	2	___1
2,52004	1,44848	NaN	-1,08496	S	2	___1
0,617701	1,38547	2,6524	2,13085	S	3	___1
NaN	-2,8343	-0,97753	-1,8106	S	2	___1
-1,5034	-2,22939	NaN	NaN	S	2	___1
-0,288248	-0,904581	-0,173662	-1,71576	S	2	___1
-0,720909	-1,10114	NaN	-1,01148	S	3	___1
2,2245	1,36687	2,93789	1,29669	S	2	___1
1,1877	1,07707	2,21728	0,440611	S	2	___1
-0,0614738	1,35596	-6,08443	0,274803	S	2	___1
0,0286446	-0,375355	1,20234	-0,0629864	S	2	___1
NaN	NaN	0,758289	-1,02557	S	3	___1
-1,51454	-1,69468	0,293882	NaN	S	2	___1
-1,44516	-1,88119	NaN	1,30951	S	2	___1
-1,33748	-2,90799	-2,39857	-0,817894	T	2	___1
-2,40442	-3,02533	NaN	-0,953215	T	2	___1
-1,81646	-2,72142	NaN	-2,97612	T	2	___1

Polito et al. Universal ready-to-use immunotherapeutic approach for the treatment of cancer: expanded and activated polyclonal γδ memory T cells.
Phosphorylation sites

NaN	NaN	NaN	NaN	T	2	___1
-0,293674	1,31557	NaN	3,61179	T	3	___1
3,61915	4,10705	4,79178	4,15345	T	2	___1
-1,11803	NaN	NaN	NaN	T	2	___1
3,38567	3,69431	1,64661	2,77283	T	2	___1
1,90932	2,85641	NaN	1,0399	T	3	___1
0,340662	2,63552	2,34646	0,716345	T	3	___1
1,54031	1,52432	-1,39918	1,20862	T	2	___1
1,43878	1,10827	NaN	0,984976	T	3	___1
-0,894115	-0,424963	NaN	-0,0878906	T	2	___1
NaN	-0,591991	0,683964	-0,566343	T	2	___1
-1,66441	-1,48503	NaN	-0,456512	T	2	___1
NaN	NaN	NaN	NaN	T	2	___1
NaN	NaN	NaN	NaN	T	3	___1
-0,836058	-0,993685	-1,81164	NaN	T	2	___1
NaN	-0,321228	3,53597	2,11758	T	3	___1
4,23317	3,96877	0,185822	3,30221	T	2	___1
-0,136614	0,714409	-1,92541	NaN	T	2	___1
NaN	-0,526768	2,25078	0,617802	T	2	___1
1,38315	0,358738	2,14124	-0,414194	T	2	___1
2,67916	2,59356	2,4218	0,845411	T	2	___1
-2,00302	-1,87787	NaN	-0,723242	T	3	___1
0,867191	1,01677	-1,29523	-0,357092	T	2	___1
-1,54859	-2,23826	-1,78726	0,559439	T	2	___1
-1,46396	-2,29594	NaN	NaN	T	2	___1
0,319715	0,402458	NaN	NaN	T	3	___1
0,0632458	0,485519	NaN	NaN	T	3	___1
NaN	NaN	NaN	NaN	T	2	___1
NaN	NaN	-1,59458	NaN	T	2	___1
NaN	-1,57989	-2,68458	-0,124628	T	3	___1
NaN	NaN	NaN	NaN	T	2	___1
-0,227112	-1,43282	0,473595	NaN	T	2	___1
0,597576	0,179539	NaN	0,236305	T	2	___1
-0,700327	-1,93968	NaN	-1,20543	T	3	___1

Polito et al. Universal ready-to-use immunotherapeutic approach for the treatment of cancer: expanded and activated polyclonal γδ memory T cells.
Phosphorylation sites

0,615982	-0,0517368	-1,25412	0,333649	T	3	___1
NaN	NaN	NaN	NaN	T	2	___1
NaN	NaN	NaN	NaN	T	2	___1
-0,0506325	-0,317728	NaN	-1,66987	T	2	___1
-1,45026	-2,91466	-0,553457	-1,45459	T	2	___1
NaN	NaN	NaN	NaN	T	2	___1
4,56054	3,94687	5,43773	3,92962	T	3	___1
-1,62819	-0,245325	0,7174	-0,0755463	T	3	___1
-1,2302	NaN	-0,251652	NaN	T	2	___1
NaN	1,54436	NaN	NaN	T	2	___1
-3,09272	-3,63184	NaN	-0,0810108	T	2	___1
NaN	-1,27321	-0,218561	NaN	T	3	___1
NaN	-0,537289	NaN	NaN	T	2	___1
-1,49059	-3,12074	-3,09101	1,01519	T	2	___1
-2,31419	0,301216	NaN	NaN	T	3	___1
NaN	0,0370007	NaN	NaN	T	3	___1
NaN	1,33658	-2,13157	-0,663956	T	2	___1
-0,46377	-1,22536	NaN	-0,474594	T	2	___1
4,78076	2,23986	-1,64029	3,28239	T	2	___1
NaN	2,7241	NaN	1,92979	T	3	___1
-0,452583	NaN	-0,780046	-1,33879	T	2	___1
-3,15221	NaN	NaN	-0,774412	T	2	___1
-0,686321	-1,65192	-1,77981	0,345791	T	2	___1
-0,0130157	-0,515673	0,339256	NaN	T	2	___1
2,68062	1,9092	NaN	NaN	T	2	___1
0,588804	-0,92631	NaN	0,0536861	T	2	___1
3,62455	3,2138	1,30858	3,3209	T	2	___1
0,831305	NaN	NaN	NaN	T	2	___1
NaN	NaN	NaN	NaN	T	2	___1
-0,575861	-2,09402	-0,515102	0,0757904	T	2	___1
NaN	NaN	NaN	NaN	T	3	___1
-0,908155	0,114658	-0,550285	-0,723783	T	3	___1
NaN	NaN	-0,604563	NaN	T	4	___1
0,537449	0,142323	0,227468	-0,489054	T	2	___1

-0,272385	-1,26253	NaN	NaN	T	2	___1
NaN	0,680618	1,83812	-0,040575	T	2	___1
-0,872196	-2,0233	NaN	NaN	T	2	___1
0,381794	-0,182734	NaN	-0,0221176	T	3	___1
-0,140274	1,83015	-1,37755	0,396395	T	3	___1
NaN	NaN	NaN	NaN	T	2	___1
2,47338	2,42103	NaN	0,0337315	T	2	___1
NaN	-1,02439	-1,91845	-2,3158	T	2	___1
0,352867	NaN	NaN	NaN	T	2	___1
0,875374	0,505091	2,54412	-0,760416	T	2	___1
1,5888	1,29038	NaN	0,549406	T	3	___1
-0,798512	-0,915548	-1,55035	NaN	T	3	___1
NaN	-0,154367	-1,026	-1,20495	T	2	___1
-2,01207	-0,728867	-2,71207	NaN	T	3	___1
NaN	-0,560686	-0,28413	NaN	T	2	___1
-0,172049	NaN	-2,0081	NaN	T	2	___1
0,102339	0,840521	NaN	NaN	T	3	___1
-0,945969	-1,14267	NaN	-1,21204	T	2	___1
2,65494	1,32966	1,3497	0,631414	T	2	___1
NaN	NaN	NaN	NaN	T	2	___1
2,63617	0,819544	0,603762	2,20166	T	2	___1
1,35633	0,336159	0,844545	3,95353	T	2	___1
0,924829	-1,07255	NaN	2,26146	T	3	___1
NaN	-2,72824	-0,967392	-0,335886	T	3	___1
-1,80722	-2,30355	NaN	NaN	T	2	___1
-1,07309	-0,913548	NaN	-0,929554	T	2	___1
-1,82478	-1,26301	NaN	-1,73481	T	2	___1
1,79146	1,83162	2,38885	2,59097	T	2	___1
1,1585	-0,973169	NaN	-3,50978	T	3	___1
0,465	0,637077	-0,655821	-1,55956	T	3	___1
0,906593	3,64154	-4,75408	1,36613	T	2	___1
NaN	NaN	NaN	NaN	T	3	___1
NaN	NaN	NaN	NaN	T	2	___1
-1,34274	-1,19799	NaN	-1,55963	T	2	___1

-1,81195	-0,506599	-2,45858	0,754057	T	3	___1
-1,45074	0,0282574	0,0885315	1,25274	T	3	___1
3,64262	3,82338	0,648664	-0,0342293	T	2	___1
3,32127	1,68218	-0,868937	NaN	T	2	___1
-0,0488033	-0,421507	NaN	-0,637222	T	2	___1
2,11934	2,69829	3,44065	1,99359	T	2	___1
-3,73804	-3,03599	-2,36281	NaN	T	2	___1
0,981659	1,83844	NaN	-0,169554	T	4	___1
NaN	NaN	NaN	NaN	T	2	___1
0,314154	-0,53784	NaN	NaN	T	2	___1
1,12496	NaN	-1,21913	0,476309	T	2	___1
NaN	NaN	NaN	NaN	T	2	___1
1,00382	0,358679	1,49352	0,73996	T	3	___1
NaN	-0,833841	NaN	NaN	T	2	___1
NaN	1,06078	0,696297	0,0927391	T	3	___1
NaN	-0,291239	1,12036	0,883114	T	2	___1
NaN	NaN	NaN	NaN	T	2	___1
0,48358	0,0385513	-0,37719	1,23871	T	2	___1
-0,125647	NaN	NaN	NaN	T	2	___1
1,86607	-0,225428	NaN	-1,54842	T	2	___1
5,68488	4,60048	6,07495	3,69009	T	3	___1
1,4611	-1,40726	-0,968121	0,171682	T	2	___1
0,401129	-0,0382099	-0,201332	0,363998	T	2	___1
0,408195	-0,148811	-0,115118	-0,291317	T	2	___1
-1,15051	-0,0766983	0,729944	-1,70962	T	4	___1
-0,411312	-0,36673	-1,55233	0,43067	T	2	___1
NaN	NaN	NaN	NaN	T	2	___1
-2,1222	-1,74098	NaN	0,718908	T	2	___1
NaN	NaN	NaN	NaN	T	3	___1
NaN	NaN	NaN	NaN	T	3	___1
NaN	-0,352142	-0,523876	-2,7683	T	3	___1
1,11736	-2,53478	-1,61568	-0,432098	T	4	___1
NaN	NaN	2,49488	3,32968	T	3	___1
0,815956	2,35226	1,01696	-0,673975	T	2	___1

-0,647051	NaN	NaN	NaN	T	2	___1
1,51556	4,91054	-0,697071	4,5781	T	3	___1
-0,298864	-0,172192	-0,647995	0,469049	T	3	___1
NaN	NaN	NaN	NaN	T	2	___1
NaN	NaN	NaN	NaN	T	3	___1
NaN	NaN	NaN	NaN	T	2	___1
NaN	NaN	NaN	NaN	T	2	___1
NaN	NaN	NaN	NaN	T	2	___1
0,546967	0,268934	NaN	-2,96055	T	2	___1
-1,58264	-2,23006	0,812304	0,0798302	T	3	___1
NaN	-1,59352	-0,375408	NaN	T	3	___1
NaN	-1,20003	-1,77223	-1,67076	T	2	___1
1,09537	1,90075	-3,30189	-0,382366	T	2	___1
0,538618	-0,255604	NaN	-0,452629	T	2	___1
NaN	NaN	NaN	NaN	T	3	___1
NaN	-0,653967	0,230904	-1,11483	T	3	___1
-1,20778	-2,23127	NaN	-2,28545	T	3	___1
NaN	0,461941	-2,71324	-1,35278	T	2	___1
NaN	NaN	NaN	NaN	T	2	___1
-0,143946	0,542831	-2,28294	-1,4359	T	3	___1
NaN	NaN	NaN	NaN	T	2	___1
-0,365286	-0,202396	NaN	-1,3244	T	2	___1
2,02127	1,05827	1,03001	-0,343773	T	2	___1
NaN	-0,243889	NaN	NaN	T	3	___1
0,389299	0,0192356	0,384335	-1,92523	T	2	___1
NaN	NaN	NaN	NaN	T	4	___1
2,03672	4,32666	4,43294	3,14188	T	3	___1
-1,7949	-1,33128	NaN	-1,18974	T	2	___1
3,94742	3,24579	4,81131	2,874	T	3	___1
6,21185	5,76863	6,39713	4,49536	T	2	___1
3,5251	2,76448	2,84003	0,936352	T	2	___1
1,13861	0,581991	-0,0122471	-0,807894	T	2	___1
-1,44752	-1,46297	NaN	NaN	T	3	___1
NaN	NaN	NaN	NaN	T	2	___1

-1,068	-0,22286	-1,21805	-1,2243	T	2	___1
NaN	NaN	NaN	NaN	T	2	___1
NaN	NaN	NaN	NaN	T	2	___1
-0,397692	0,0023098	-1,73178	NaN	T	2	___1
-1,51424	NaN	-2,08629	1,01635	T	2	___1
3,04996	1,14688	1,87608	5,3564	T	2	___1
-3,63976	-4,11777	NaN	-0,388578	T	2	___1
NaN	NaN	NaN	NaN	T	2	___1
NaN	-2,87181	-1,36221	-0,412603	T	2	___1
-1,78878	NaN	-1,32017	-0,246777	T	2	___1
1,03917	-0,888636	0,750841	NaN	T	2	___1
NaN	NaN	NaN	NaN	T	3	___1
6,92978	5,14647	-0,340834	4,65905	T	2	___1
NaN	NaN	NaN	NaN	T	2	___1
6,92978	5,14647	-0,340834	4,65905	T	2	___1
2,80253	3,81598	4,52245	3,99623	T	3	___1
NaN	NaN	NaN	NaN	T	2	___1
-2,46969	NaN	-0,453123	NaN	T	2	___1
2,89104	3,05679	-1,11847	1,80415	T	3	___1
2,49346	3,22495	-1,37834	2,1008	T	2	___1
-0,719027	-0,182304	-3,0814	NaN	T	2	___1
NaN	-2,83392	-1,6699	2,34447	T	3	___1
1,79146	1,83162	2,38885	2,59097	T	2	___1
1,68148	-0,966045	-2,0447	1,67791	T	3	___1
-1,41593	-1,98118	-1,71579	1,40998	T	1	___1
NaN	NaN	NaN	NaN	T	2	___1
4,31853	1,94396	NaN	2,84782	T	2	___1
NaN	NaN	1,42869	NaN	T	3	___1
NaN	NaN	NaN	0,95496	T	2	___1
NaN	NaN	NaN	NaN	T	3	___1
0,31329	-0,299797	0,623384	2,68	T	2	___1
-2,78988	-2,44984	-1,3153	NaN	T	3	___1
-1,22306	0,461445	NaN	NaN	T	2	___1
-1,78031	-1,85195	-1,66428	1,49579	T	3	___1

-1,78627	-2,00764	-0,0328388	NaN	T	3	___1
-1,68365	-1,77507	-3,27654	-1,58022	T	2	___1
NaN	NaN	NaN	NaN	T	3	___1
NaN	-3,02768	-0,904873	NaN	T	3	___1
7,42128	7,1166	5,66072	5,21781	T	4	___1
-0,109222	0,248024	NaN	NaN	T	3	___1
-0,695326	-0,882895	-0,302067	1,52197	T	2	___1
NaN	-1,0914	-0,226816	-1,75824	T	3	___1
NaN	NaN	NaN	NaN	T	2	___1
3,14236	3,99567	4,18553	2,53452	T	3	___1
NaN	NaN	NaN	NaN	T	3	___1
-0,888268	-1,25937	NaN	1,81064	T	2	___1
NaN	NaN	NaN	NaN	T	2	___1
2,47051	2,42668	NaN	0,959183	T	2	___1
-0,834019	-1,68013	NaN	-0,474529	T	3	___1
2,43257	3,07355	NaN	2,23921	T	2	___1
NaN	NaN	NaN	NaN	T	2	___1
-1,31912	-2,15223	-1,4921	0,634165	T	2	___1
1,23066	4,817	NaN	-0,670092	T	2	___1
-1,32285	NaN	NaN	NaN	T	2	___1
NaN	-1,01819	NaN	-0,270061	T	2	___1
1,29242	NaN	1,92942	NaN	T	2	___1
0,886755	0,677588	-0,89324	NaN	T	2	___1
NaN	NaN	NaN	NaN	T	2	___1
5,59391	8,46867	4,99834	5,8838	T	2	___1
NaN	NaN	-1,27633	NaN	T	2	___1
6,92978	5,14647	-0,340834	4,65905	T	2	___1
NaN	-1,76424	0,146139	0,0171146	T	2	___1
-0,869724	-0,425472	-1,41387	-1,2172	T	2	___1
8,13658	8,44166	5,92197	6,64282	T	2	___1
-0,496731	-2,49899	NaN	-1,5685	T	3	___1
2,06871	2,1167	1,04791	1,13224	T	2	___1
1,35674	1,66206	-2,14963	0,671824	T	2	___1
1,22961	0,605394	-0,167271	0,426231	T	2	___1

Polito et al. Universal ready-to-use immunotherapeutic approach for the treatment of cancer: expanded and activated polyclonal γδ memory T cells.
Phosphorylation sites

1,45391	1,93782	-0,795355	NaN	T	3	___1
0,370903	-3,83999	0,949507	NaN	T	2	___1
3,99798	4,37312	4,4075	4,00307	T	3	___1
NaN	NaN	NaN	NaN	T	2	___1
-1,73335	-2,66299	NaN	0,523256	T	2	___1
-0,344063	0,819372	0,785549	3,51473	T	2	___1
6,0829	5,32509	4,57661	6,50129	T	2	___1
NaN	NaN	NaN	NaN	T	2	___1
2,7603	4,28857	0,0695171	2,04762	T	3	___1
NaN	NaN	NaN	NaN	T	1	___1
-2,68411	-2,48242	NaN	-1,13506	T	2	___1
NaN	NaN	NaN	NaN	T	2	___1
0,303186	0,384165	-2,44091	NaN	T	2	___1
0,265924	0,133436	NaN	-1,07678	T	2	___1
NaN	-1,07788	-0,585091	-1,09919	T	3	___1
NaN	-0,508646	NaN	NaN	T	2	___1
-2,11158	-1,45788	-2,48962	NaN	T	2	___1
NaN	0,137712	-3,2007	-0,81806	T	3	___1
1,27248	-0,370251	-1,56619	-0,314814	T	3	___1
1,30846	0,290102	0,69845	NaN	T	3	___1
NaN	NaN	NaN	NaN	T	3	___1
-0,0536137	-1,37661	NaN	-0,985874	T	2	___1
-1,90167	-0,22171	NaN	-1,38582	T	3	___1
NaN	NaN	NaN	NaN	T	2	___1
NaN	NaN	NaN	0,14057	T	4	___1
0,523609	0,317974	-0,675091	0,723598	T	2	___1
3,6862	2,67759	2,90393	NaN	T	2	___1
-0,417406	-0,502514	NaN	1,32202	T	3	___1
-2,67863	-3,172	NaN	-2,2983	T	2	___1
1,59398	1,29783	0,328009	0,457445	T	2	___1
-0,519299	-0,174414	-3,19659	1,81604	T	2	___1
-1,26675	-2,1941	-2,0277	NaN	T	2	___1
NaN	NaN	NaN	NaN	T	2	___1
-1,46425	-2,99637	NaN	0,0597439	T	2	___1

Polito et al. Universal ready-to-use immunotherapeutic approach for the treatment of cancer: expanded and activated polyclonal γδ memory T cells.
Phosphorylation sites

NaN	NaN	NaN	NaN	T	3	___1
1,81263	1,32999	NaN	2,06441	T	3	___1
NaN	NaN	NaN	NaN	T	2	___1
7,2752	6,16403	6,77002	4,62062	T	2	___1
3,62789	4,19488	6,3971	3,7702	T	3	___1
5,1314	4,74287	4,07194	2,83698	T	2	___1
7,2752	6,16403	6,77002	4,62062	T	2	___1
NaN	NaN	NaN	NaN	T	2	___1
-0,900373	-2,03879	-2,07458	1,22434	T	2	___1
NaN	NaN	NaN	NaN	T	3	___1
-0,255163	0,140604	-2,33014	NaN	T	2	___1
-0,142147	0,230108	NaN	-1,74593	T	2	___1
-0,998926	-0,53883	NaN	-0,827131	T	2	___1
1,01958	NaN	-0,584007	0,322752	T	2	___1
-0,00284386	-0,500689	NaN	-0,475994	T	2	___1
NaN	NaN	1,11467	NaN	T	2	___1
1,05418	0,770668	2,03683	1,0851	T	3	___1
NaN	-0,266594	-0,674126	0,403955	T	2	___1
0,944244	NaN	NaN	-1,32007	T	2	___1
NaN	-1,57873	0,704319	NaN	T	3	___1
-1,29622	-1,24795	NaN	-1,7395	T	2	___1
NaN	NaN	NaN	NaN	T	3	___1
1,49616	1,70642	-0,540909	1,97845	T	2	___1
-0,547682	-2,00645	-2,26269	0,377604	T	2	___1
NaN	NaN	NaN	NaN	T	3	___1
3,77189	2,92837	1,9683	2,05317	T	2	___1
3,36069	3,22178	1,83159	2,74988	T	2	___1
0,97143	-1,08415	0,398811	2,38271	T	2	___1
0,255833	0,118433	-0,446924	1,63081	T	2	___1
-1,84692	-1,56772	NaN	-2,25327	T	2	___1
NaN	NaN	NaN	NaN	T	3	___1
NaN	NaN	NaN	NaN	T	2	___1
NaN	NaN	NaN	NaN	T	2	___1
NaN	NaN	NaN	NaN	T	3	___1

5,22014	4,43785	4,91084	4,43491	T	3	___1
0,414787	0,914242	-0,143707	0,210423	T	2	___1
-0,802736	-1,03907	NaN	0,376141	T	3	___1
-1,26683	-2,48255	NaN	-0,39534	T	2	___1
-1,00524	-1,36884	-0,231619	2,68091	T	3	___1
-1,70382	-1,47234	NaN	-0,236805	T	3	___1
NaN	NaN	NaN	NaN	T	2	___1
NaN	-2,31482	NaN	-0,622686	T	3	___1
NaN	-0,635246	NaN	-0,297148	T	3	___1
-3,48798	-0,243708	NaN	0,90204	T	3	___1
-0,151545	-0,507677	NaN	1,83363	T	2	___1
-1,16838	-1,63355	NaN	-0,844189	T	2	___1
1,46976	1,32374	NaN	0,677385	T	3	___1
-0,372355	-1,05416	0,0436707	-0,88345	T	2	___1
0,370932	-0,904297	-3,76822	0,834944	T	2	___1
-1,53257	-2,20598	-0,416225	0,573406	T	2	___1
-0,40144	2,42992	NaN	1,98012	T	3	___1
-1,57529	-2,67538	NaN	-0,210709	T	3	___1
0,610706	-1,15518	0,29261	2,28501	T	2	___1
1,3426	0,0335979	0,0204067	2,74017	T	3	___1
-0,45064	-0,68434	-1,24913	-0,563641	T	2	___1
NaN	NaN	NaN	NaN	T	2	___1
NaN	NaN	NaN	NaN	T	3	___1
0,176659	-1,47984	-1,17863	-2,03884	T	2	___1
-1,59484	-2,25192	-2,3353	-1,28854	T	2	___1
-2,57148	-2,17566	-1,57969	0,11832	T	2	___1
NaN	NaN	NaN	NaN	T	2	___1
NaN	-2,56339	NaN	-1,13419	T	3	___1
-1,41546	-2,3509	-2,34036	NaN	T	2	___1
NaN	NaN	NaN	NaN	T	3	___1
-0,360872	0,442385	NaN	3,13117	T	3	___1
-0,740458	-1,20736	NaN	NaN	T	2	___1
0,335453	-0,111485	-0,566048	NaN	T	2	___1
NaN	NaN	NaN	NaN	T	2	___1

-1,57705	-2,47343	-1,11731	NaN	T	2	___1
0,662977	2,84094	NaN	-1,26527	T	2	___1
5,66392	4,53527	2,57116	3,13181	T	2	___1
NaN	NaN	NaN	NaN	T	4	___1
4,55842	2,02034	NaN	2,00507	T	2	___1
-1,30236	-4,25494	-2,44103	-1,31569	T	2	___1
0,3654	0,421484	-2,66896	-2,28774	T	2	___1
0,404772	2,59004	-2,6288	NaN	T	2	___1
1,40516	2,07005	NaN	-0,570894	T	2	___1
NaN	NaN	NaN	NaN	T	2	___1
NaN	NaN	NaN	NaN	T	2	___1
1,24567	2,98164	NaN	-0,401453	T	3	___1
-4,14175	2,85955	NaN	-0,125294	T	2	___1
-0,556572	1,7016	NaN	-0,587765	T	4	___1
NaN	0,0857391	NaN	NaN	T	3	___1
-0,309216	0,413652	-0,0552254	-0,474855	T	2	___1
NaN	0,202709	2,12943	0,232922	T	2	___1
NaN	NaN	NaN	NaN	T	2	___1
-0,456476	-0,60923	NaN	-0,884703	T	2	___1
-0,304373	0,43214	NaN	-0,0680599	T	2	___1
NaN	NaN	NaN	NaN	T	3	___1
NaN	NaN	NaN	-0,437735	T	2	___1
NaN	-0,207376	NaN	-0,915567	T	2	___1
1,90637	-1,81294	1,46337	1,01519	T	2	___1
NaN	NaN	NaN	NaN	T	2	___1
0,0975552	0,477049	NaN	NaN	T	2	___1
-0,555553	-0,235832	NaN	NaN	T	3	___1
NaN	NaN	NaN	NaN	T	2	___1
-0,825397	0,414791	1,47463	1,28347	T	4	___1
-0,437536	0,83853	1,69766	1,79284	T	4	___1
-0,890257	-0,155802	NaN	1,68521	T	2	___1
NaN	NaN	NaN	NaN	T	2	___1
NaN	NaN	NaN	NaN	T	3	___1
NaN	-2,0349	-0,142139	NaN	T	2	___1

Polito et al. Universal ready-to-use immunotherapeutic approach for the treatment of cancer: expanded and activated polyclonal γδ memory T cells.
Phosphorylation sites

-1,15158	-0,818388	-3,17327	NaN	T	3	___1
0,722185	1,60739	2,09203	1,09445	T	2	___1
2,51544	2,28226	NaN	NaN	T	2	___1
0,751932	-0,44216	NaN	0,200304	T	4	___1
-0,221729	-1,98029	NaN	-0,538925	T	2	___1
-3,21209	NaN	-0,871744	NaN	T	2	___1
NaN	0,630581	-2,98289	0,371857	T	4	___1
2,73033	2,03571	-1,00468	1,7519	T	2	___1
1,49751	0,023344	2,12692	1,95912	T	2	___1
NaN	NaN	NaN	NaN	T	3	___1
-2,57047	NaN	-1,40176	-0,250172	T	3	___1
-2,42608	-1,43051	NaN	-0,538246	T	2	___1
NaN	0,455875	0,737082	-0,711725	T	3	___1
NaN	0,99118	-2,28022	-0,0785351	T	3	___1
NaN	NaN	NaN	NaN	T	3	___1
-1,19574	NaN	NaN	NaN	T	2	___1
NaN	NaN	NaN	NaN	T	2	___1
NaN	NaN	NaN	NaN	T	2	___1
NaN	NaN	NaN	NaN	T	3	___1
1,42311	1,6309	NaN	-1,5379	T	2	___1
1,17038	1,40835	1,0196	-0,360247	T	2	___1
NaN	0,0519924	-0,490435	-0,445753	T	3	___1
NaN	2,29852	NaN	NaN	T	3	___1
NaN	NaN	NaN	NaN	T	3	___1
NaN	NaN	NaN	NaN	T	2	___1
NaN	-0,0583515	0,676439	-1,29986	T	2	___1
NaN	NaN	NaN	NaN	T	3	___1
NaN	NaN	NaN	NaN	T	3	___1
1,83523	NaN	2,38698	NaN	T	2	___1
1,51895	0,0508957	-0,725536	-0,189369	T	2	___1
1,88711	1,33123	NaN	2,52722	T	2	___1
-0,99526	-0,55522	-0,719238	-0,894381	T	3	___1
1,28661	0,622923	-0,902174	-1,02743	T	2	___1
NaN	NaN	0,296272	NaN	T	2	___1

6,04415	4,41369	3,11502	3,76076	T	2	___1
-1,23077	0,0735455	-0,0118484	0,182602	T	2	___1
-0,55047	-0,247843	NaN	-1,10165	T	2	___1
NaN	-1,79251	NaN	NaN	T	3	___1
NaN	NaN	-1,25857	-1,71077	T	3	___1
-0,109484	-1,44453	NaN	-1,07057	T	2	___1
NaN	-2,23694	-1,7941	NaN	T	2	___1
NaN	-2,41916	-0,439928	NaN	T	2	___1
-0,257357	-2,43682	-0,982489	NaN	T	2	___1
0,958597	0,390306	-0,940296	1,67381	T	2	___1
NaN	-0,821867	NaN	-1,41757	T	2	___1
1,58222	1,60905	-0,559139	0,978096	T	3	___1
-0,970743	-0,343557	-0,82391	0,383701	T	2	___1
-1,08245	NaN	NaN	NaN	T	2	___1
-0,0185165	-0,367022	1,09987	-0,349552	T	2	___1
NaN	NaN	NaN	NaN	T	2	___1
0,900833	2,33138	-1,12811	2,22103	T	2	___1
1,60336	0,288179	-1,75941	-0,877499	T	2	___1
0,148865	-0,503695	0,85565	-0,497263	T	2	___1
NaN	NaN	NaN	NaN	T	3	___1
NaN	NaN	NaN	NaN	T	3	___1
-0,438381	0,633467	NaN	NaN	T	3	___1
NaN	1,09464	1,642	0,070425	T	3	___1
-1,70631	-0,208689	-0,818523	-0,156441	T	2	___1
-1,83229	-1,05534	-0,590372	-2,52477	T	2	___1
-6,99918	-2,25213	-3,91283	-4,57307	T	3	___1
NaN	NaN	NaN	NaN	T	3	___1
NaN	NaN	NaN	NaN	T	2	___1
0,402651	0,331228	-1,2028	-0,739538	T	2	___1
NaN	-1,61565	-0,796768	0,081049	T	4	___1
2,5242	3,85746	4,36724	3,19664	T	3	___1
2,61805	3,07318	2,13812	1,84672	T	2	___1
-0,347939	-1,94621	NaN	-1,27765	T	2	___1
-2,48119	0,172428	-0,505983	-0,826965	T	3	___1

Polito et al. Universal ready-to-use immunotherapeutic approach for the treatment of cancer: expanded and activated polyclonal γδ memory T cells.
Phosphorylation sites

NaN	-0,956572	-0,10227	-1,61811	T	2	___1
NaN	NaN	NaN	NaN	T	2	___1
2,45972	2,29297	1,37801	0,600973	T	2	___1
NaN	3,29078	3,94148	NaN	T	2	___1
NaN	NaN	-2,64405	0,475147	T	4	___1
4,38355	3,787	3,25307	2,68678	T	2	___1
0,938471	0,435452	NaN	NaN	T	3	___1
NaN	-1,05103	0,898729	-0,376455	T	3	___1
NaN	-0,565615	NaN	NaN	T	3	___1
0,905519	-0,360292	0,383604	2,78082	T	2	___1
0,241133	-0,671606	-1,97799	-1,09854	T	2	___1
NaN	-0,774164	-1,16281	-1,20074	T	3	___1
0,290863	0,859024	-1,06457	-0,380901	T	3	___1
-3,70409	NaN	-3,71961	-1,13841	T	2	___1
NaN	NaN	NaN	NaN	T	2	___1
0,186403	0,140329	0,273329	-0,719997	T	2	___1
-1,81427	-1,18473	NaN	-0,688473	T	3	___1
NaN	-1,2803	-0,995932	NaN	T	3	___1
-0,945435	-1,70759	-1,7591	NaN	T	2	___1
NaN	NaN	NaN	NaN	T	2	___1
NaN	NaN	NaN	NaN	T	2	___1
NaN	0,77231	0,34656	-0,16044	T	3	___1
0,389484	-0,0823841	2,0672	-0,422617	T	3	___1
NaN	NaN	NaN	NaN	T	2	___1
NaN	NaN	NaN	NaN	T	3	___1
NaN	NaN	NaN	NaN	T	2	___1
-0,835997	4,11818	NaN	-3,13085	T	3	___1
-0,257729	0,351923	NaN	NaN	T	2	___1
-0,967962	0,0661793	NaN	-0,706402	T	2	___1
-2,00705	-2,8171	NaN	-0,508749	T	2	___1
NaN	NaN	NaN	NaN	T	2	___1
1,9805	-0,229332	0,268234	1,86477	T	2	___1
NaN	-2,09111	-2,21167	-1,12706	T	3	___1
1,85673	0,082592	-1,38903	NaN	T	3	___1

-0,093647	-0,319998	1,12826	-0,543631	T	2	___1
-0,767038	-1,1878	NaN	NaN	T	3	___1
1,58949	1,18238	-2,65969	0,932552	T	2	___1
-3,10751	-3,92102	NaN	-2,04222	T	2	___1
0,629843	0,163794	-1,07301	NaN	T	3	___1
-0,984116	0,541113	1,64706	0,91502	T	2	___1
0,702372	-0,070158	-0,839067	-0,914154	T	2	___1
-1,2033	-0,861668	NaN	-1,99869	T	2	___1
NaN	1,5607	1,13495	0,86355	T	4	___1
-1,01915	0,167038	2,6204	0,519159	T	3	___1
-0,797308	-0,686777	NaN	-0,13452	T	2	___1
-1,52188	-0,933502	NaN	-0,166948	T	2	___1
NaN	-1,32882	1,17219	-0,214046	T	2	___1
1,36025	1,23864	-0,0437851	0,858261	T	3	___1
-0,778168	-1,50471	NaN	-1,25864	T	2	___1
NaN	-2,10239	NaN	NaN	T	3	___1
1,24279	0,918579	-0,442299	1,80536	T	2	___1
-1,82209	NaN	-1,94939	NaN	T	2	___1
NaN	-0,56595	-1,13423	-1,61223	T	2	___1
1,53542	1,26114	NaN	NaN	T	2	___1
NaN	NaN	NaN	NaN	T	3	___1
-1,1687	-0,808662	-0,0498676	NaN	T	2	___1
0,147707	NaN	0,0687408	NaN	T	3	___1
NaN	NaN	NaN	NaN	T	3	___1
NaN	NaN	NaN	NaN	T	2	___1
3,32013	2,64221	4,24737	1,30932	T	2	___1
3,4165	-1,33962	-0,15377	NaN	T	2	___1
0,633242	0,581484	-1,80813	0,62851	T	2	___1
1,81479	2,58715	1,50996	0,985924	T	2	___1
0,2264	1,20044	NaN	0,427155	T	2	___1
NaN	NaN	NaN	NaN	T	2	___1
-0,184555	2,1013	NaN	0,979877	T	2	___1
0,238756	-1,22454	-0,922895	-1,81752	T	2	___1
-0,0762291	0,0412064	NaN	-1,25987	T	2	___1

Polito et al. Universal ready-to-use immunotherapeutic approach for the treatment of cancer: expanded and activated polyclonal γδ memory T cells.
Phosphorylation sites

NaN	NaN	NaN	NaN	T	3	___1
-1,95172	-1,89722	-2,74638	NaN	T	2	___1
NaN	NaN	NaN	NaN	T	2	___1
0,462486	1,00045	NaN	-1,15809	T	3	___1
-1,04744	-1,95509	NaN	-3,01522	T	2	___1
-0,134672	1,04584	NaN	-0,916142	T	3	___1
3,17787	3,64433	2,61216	3,4769	T	2	___1
-1,77472	-2,53817	NaN	-2,28614	T	2	___1
NaN	-1,65268	NaN	NaN	T	2	___1
0,713232	0,0757771	2,46489	0,35527	T	2	___1
-2,77864	NaN	-2,06686	-0,389957	T	2	___1
0,74843	-0,128204	-0,520199	NaN	T	2	___1
-1,27737	NaN	-1,20694	-1,12864	T	3	___1
0,344105	-0,500153	-1,00581	-1,51322	T	3	___1
NaN	NaN	NaN	NaN	T	2	___1
2,84254	2,45197	-0,934771	2,09225	T	2	___1
-1,54697	-2,69291	-0,529711	-0,370264	T	2	___1
NaN	NaN	NaN	NaN	T	3	___1
0,417669	-2,05299	NaN	0,668438	T	2	___1
-0,554281	0,474215	1,53948	0,812311	T	2	___1
-0,375902	0,194983	-0,210163	-2,21949	T	2	___1
-1,20474	-1,54393	-1,36623	NaN	T	2	___1
0,897005	0,105951	1,58325	-0,844063	T	2	___1
NaN	-0,194136	-0,220837	NaN	T	3	___1
-1,14506	-3,0819	0,271053	-0,339321	T	2	___1
-0,38628	-1,18699	NaN	-2,58632	T	2	___1
-1,2804	-0,733269	NaN	-1,8926	T	2	___1
NaN	0,532755	-1,85065	-1,16369	T	2	___1
NaN	NaN	NaN	NaN	T	2	___1
4,39803	4,23025	4,06222	4,68678	T	2	___1
-0,854296	1,30555	NaN	-1,06125	T	3	___1
NaN	NaN	NaN	NaN	T	2	___1
-1,53427	-0,0568523	-0,480822	-0,786444	T	3	___1
-0,248892	NaN	-1,75101	-2,06184	T	2	___1

0,961802	1,93716	-1,60474	-0,819382	T	2	___1
-0,325752	-0,070158	1,66295	-0,657475	T	2	___1
-0,796886	0,177799	-0,764463	-0,378981	T	2	___1
NaN	0,854002	NaN	-0,637259	T	3	___1
-0,09939	0,273832	1,48664	0,106075	T	2	___1
NaN	NaN	NaN	NaN	T	2	___1
-1,62103	-1,53971	-0,953848	NaN	T	3	___1
3,764	3,69642	2,71102	0,135418	T	2	___1
-1,9182	-1,66028	NaN	0,422968	T	2	___1
1,00318	-0,659704	0,859118	0,137188	T	3	___1
0,876411	0,318277	-0,7225	-1,37072	T	2	___1
NaN	-0,0488319	0,1418	-0,12376	T	3	___1
-0,947239	NaN	-3,23062	-1,70243	T	2	___1
0,417669	-2,05299	NaN	0,668438	T	2	___1
-0,0589447	-0,294767	NaN	0,625383	T	2	___1
-2,45483	-2,10268	-4,31823	-1,26088	T	2	___1
-0,232679	0,667841	NaN	NaN	T	2	___1
-0,704725	-2,41591	-1,85334	-0,98959	T	3	___1
NaN	NaN	NaN	NaN	T	2	___1
NaN	NaN	NaN	NaN	T	2	___1
NaN	NaN	NaN	NaN	T	2	___1
1,17856	0,439201	NaN	1,52262	T	2	___1
-1,08517	-0,205364	-0,0604095	NaN	T	3	___1
NaN	NaN	-0,626818	NaN	T	2	___1
NaN	-2,43817	-2,2549	-0,177612	T	3	___1
NaN	-1,17491	-1,71022	-0,895208	T	2	___1
0,259716	0,234882	-3,56268	-1,07559	T	2	___1
-1,32441	-0,905575	NaN	-1,15214	T	2	___1
-0,394819	0,13916	NaN	NaN	T	2	___1
NaN	NaN	NaN	NaN	T	3	___1
NaN	NaN	NaN	NaN	T	3	___1
-1,69931	-1,63322	-2,09224	NaN	T	2	___1
-1,2121	-0,903305	-1,73333	1,84925	T	2	___1
-1,71652	NaN	-1,81737	-0,894598	T	2	___1

Polito et al. Universal ready-to-use immunotherapeutic approach for the treatment of cancer: expanded and activated polyclonal γδ memory T cells.
Phosphorylation sites

NaN	NaN	-0,483421	-2,59394	T	3	___1
NaN	NaN	NaN	-0,163218	T	3	___1
-0,868078	-2,33151	NaN	-1,37369	T	2	___1
-0,23447	-0,574503	-0,325294	NaN	T	2	___1
2,61952	0,753866	2,08343	2,60944	T	2	___1
NaN	NaN	NaN	NaN	T	2	___1
0,694792	-2,19348	0,948421	2,47812	T	2	___1
-1,01746	-1,93654	-1,63673	-2,06428	T	2	___1
NaN	NaN	NaN	NaN	T	2	___1
-0,603128	-0,52644	1,93655	0,43821	T	2	___1
2,51538	2,6052	1,06825	1,36112	T	2	___1
0,397951	1,14005	1,10063	NaN	T	4	___1
NaN	-3,32644	-1,67784	NaN	T	2	___1
-0,323063	-1,80146	-0,604139	-2,43298	T	2	___1
-0,872894	-0,591648	-0,119385	NaN	T	2	___1
NaN	-1,51344	-2,74104	NaN	T	3	___1
1,1302	0,606541	-0,528635	1,34166	T	2	___1
1,1302	0,606541	-0,528635	1,34166	T	3	___1
NaN	NaN	NaN	NaN	T	3	___1
2,27942	1,62752	1,56015	2,20405	T	2	___1
3,88858	3,88522	1,55255	3,2598	T	2	___1
-0,820923	1,60744	-1,5922	1,42139	T	3	___1
-0,320639	-0,665222	0,439318	NaN	T	2	___1
4,62436	3,91552	4,9347	3,74091	T	2	___1
0,826349	1,03652	NaN	NaN	T	2	___1
1,62823	0,153881	-1,73666	0,0546112	T	2	___1
-1,73635	-1,13112	NaN	-0,087492	T	2	___1
1,23429	1,14486	0,110558	0,233339	T	2	___1
0,259716	0,234882	-3,56268	-1,07559	T	2	___1
-0,287233	0,609276	-0,631929	-0,630098	T	2	___1
0,352461	0,0690002	-0,789268	-0,461988	T	2	___1
NaN	NaN	NaN	NaN	T	2	___1
-0,809462	-1,2436	-1,4944	-0,419512	T	2	___1
-0,180859	-1,45609	-2,29625	-2,59961	T	2	___1

Polito et al. Universal ready-to-use immunotherapeutic approach for the treatment of cancer: expanded and activated polyclonal γδ memory T cells.
Phosphorylation sites

1,59753	1,55503	3,36051	1,75572	T	2		___1
1,72929	1,45527	NaN	-0,722586	T	4		___1
4,21108	4,78123	-4,43763	2,70479	T	2		___1
1,51822	1,32716	-4,14403	-1,19263	T	3		___1
-1,12655	-0,443293	-0,499191	-0,861748	T	2		___1
NaN	NaN	NaN	NaN	T	3		___1
NaN	NaN	NaN	NaN	T	2		___1
0,469034	-0,29291	-0,518599	-1,19805	T	3		___1
NaN	NaN	NaN	NaN	Y	3	+	___1
0,692413	0,560926	NaN	NaN	Y	2		___1
-0,360384	-0,577894	NaN	-2,16847	Y	2	+	___1
3,63686	3,66199	1,97927	2,60216	Y	2	+	___1
-1,47943	-1,34119	NaN	-1,85423	Y	2		___1
2,53518	1,80096	-0,761147	NaN	Y	2		___1
3,29352	3,09579	2,69227	2,14188	Y	3	+	___1
NaN	NaN	-1,27481	NaN	Y	2		___1
NaN	NaN	NaN	NaN	Y	3		___1
0,0552654	-0,415834	0,879629	-1,77818	Y	2		___1
6,57047	5,31999	4,0765	2,45852	Y	2	+	___1
3,63686	3,66199	1,97927	2,60216	Y	2	+	___1
-1,47943	-1,34119	NaN	-1,85423	Y	2		___1
-0,0457249	NaN	NaN	NaN	Y	2		___1
2,76025	2,07439	2,08283	0,670897	Y	2	+	___1
1,4421	NaN	NaN	-1,25137	Y	2	+	___1
NaN	NaN	NaN	NaN	Y	2		___1
NaN	-0,746943	NaN	NaN	Y	3	+	___1
0,793804	0,715101	NaN	NaN	Y	2		___1
-0,304417	0,361454	NaN	NaN	Y	2	+	___1
-2,10815	-0,891727	NaN	NaN	Y	2	+	___1
NaN	-0,391869	NaN	NaN	Y	2		___1
-0,44313	1,08888	NaN	-1,10669	Y	3		___1
NaN	-1,84553	0,365309	0,882732	Y	3		___1
-0,0083046	-0,339142	-0,717056	-0,796295	Y	2		___1
2,80402	2,49389	3,3463	2,37084	Y	2		___1

NaN	NaN	NaN	NaN	Y	2		___1
NaN	NaN	NaN	NaN	Y	2		___1
NaN	-1,79748	-2,89915	-0,417633	Y	2	+	___1
2,50364	2,98882	2,97142	2,96627	Y	2		___1
3,54406	3,32083	4,315	4,24178	Y	2	+	___1
-1,09653	-1,36565	NaN	0,31477	Y	2	+	___1
NaN	NaN	NaN	NaN	Y	2		___1
-1,80286	-2,38581	NaN	-0,762362	Y	2		___1
NaN	NaN	-1,20581	NaN	Y	3	+	___1
NaN	NaN	NaN	NaN	Y	3		___1
-0,896887	-1,49598	NaN	NaN	Y	2	+	___1
-0,939243	-1,04759	0,361855	-0,0274334	S	2		___2
-0,939243	-1,04759	0,361855	-0,0274334	S	2		___2
0,539906	1,07106	-1,04048	1,35188	S	3		___2
0,539906	1,07106	-1,04048	1,35188	S	2		___2
NaN	NaN	NaN	NaN	S	2		___2
-0,757519	-0,211231	0,583216	-1,43526	S	2		___2
NaN	NaN	NaN	NaN	S	3		___2
NaN	NaN	NaN	NaN	S	3		___2
NaN	NaN	NaN	NaN	S	3		___2
NaN	NaN	NaN	NaN	S	3		___2
-0,337788	-2,55923	NaN	0,416893	S	2		___2
-0,272343	0,231012	0,108553	0,49448	S	3		___2
-0,272343	0,231012	0,108553	0,49448	S	3		___2
2,17626	3,46336	3,89428	3,10905	S	2		___2
2,17626	3,46336	3,89428	3,10905	S	2		___2
0,444294	0,102495	2,45108	-0,256704	S	3		___2
0,444294	0,102495	2,45108	-0,256704	S	3		___2
NaN	NaN	NaN	NaN	S	3		___2
NaN	NaN	NaN	NaN	S	4		___2
NaN	1,25229	NaN	2,15309	S	2		___2
2,32134	1,83473	2,4481	1,05158	S	2		___2
2,32134	1,83473	2,4481	1,05158	S	2		___2
-1,97428	-0,225693	0,16567	-0,945299	S	3		___2

-1,97428	-0,225693	0,16567	-0,945299	S	3	___2
NaN	2,31854	NaN	1,14161	S	4	___2
NaN	2,31854	NaN	1,14161	S	4	___2
NaN	NaN	NaN	NaN	S	2	___2
NaN	-0,0161972	NaN	-0,145309	S	2	___2
1,89347	-0,200914	-1,99561	NaN	S	2	___2
1,89347	-0,200914	-1,99561	NaN	S	2	___2
-0,397053	1,40065	-0,987001	-1,60167	S	3	___2
-0,132696	0,723112	-0,0739422	-1,20786	S	3	___2
NaN	-0,844826	-0,051506	NaN	S	3	___2
NaN	NaN	NaN	NaN	S	3	___2
-1,99817	-1,0356	0,65123	-0,942955	S	2	___2
-1,10166	-1,01985	NaN	NaN	S	2	___2
-1,10166	-1,01985	NaN	NaN	S	2	___2
2,22732	-0,239855	0,0594349	-1,15386	S	2	___2
0,693399	3,37925	2,59451	1,61965	S	2	___2
2,22732	3,37925	2,59451	1,61965	S	2	___2
1,70229	2,00626	-0,723883	0,828892	S	2	___2
-1,42949	2,00626	-0,723883	0,828892	S	2	___2
-0,624548	0,545118	NaN	1,00133	S	3	___2
-1,01753	-2,37382	0,148598	-1,02081	S	2	___2
-1,01753	-2,37382	0,148598	-1,02081	S	2	___2
-0,343317	1,49749	1,13155	NaN	S	3	___2
-0,343317	1,49749	1,13155	NaN	S	3	___2
-0,719597	-1,40726	-1,58324	-0,117496	S	2	___2
0,538618	-0,0211887	-0,409006	-0,359825	S	2	___2
0,911629	0,622284	-0,319475	0,210707	S	2	___2
0,911629	0,622284	-0,319475	0,210707	S	2	___2
NaN	-3,954	-2,94772	-1,33323	S	2	___2
NaN	-3,954	-2,94772	-1,33323	S	2	___2
-1,54232	-1,63544	-1,5646	NaN	S	2	___2
-1,54232	-1,63544	-1,5646	NaN	S	2	___2
NaN	NaN	NaN	NaN	S	2	___2
NaN	NaN	NaN	NaN	S	2	___2

Polito et al. Universal ready-to-use immunotherapeutic approach for the treatment of cancer: expanded and activated polyclonal γδ memory T cells.
Phosphorylation sites

NaN	NaN	NaN	NaN	S	2	___2
NaN	NaN	NaN	NaN	S	2	___2
-1,95239	NaN	-1,65289	NaN	S	2	___2
-1,95239	NaN	-1,65289	NaN	S	2	___2
-1,56963	-1,37341	NaN	NaN	S	3	___2
-1,56963	1,25109	1,3693	1,13405	S	3	___2
-1,56963	1,25109	1,3693	1,13405	S	3	___2
NaN	NaN	NaN	NaN	S	3	___2
NaN	NaN	NaN	NaN	S	2	___2
0,419327	NaN	0,527342	0,0209293	S	3	___2
0,419327	NaN	0,527342	0,0209293	S	3	___2
-0,708626	-1,02583	-3,22073	NaN	S	2	___2
NaN	2,75441	-1,13474	0,132408	S	3	___2
NaN	2,75441	-1,13474	0,132408	S	3	___2
0,402964	0,615473	NaN	0,326847	S	2	___2
0,402964	0,615473	NaN	0,326847	S	3	___2
NaN	-1,94023	-3,05787	-0,494555	S	2	___2
NaN	-1,94023	-3,05787	-0,494555	S	2	___2
0,346123	0,180878	0,324741	-0,0946789	S	2	___2
0,346123	0,180878	0,324741	-0,0946789	S	2	___2
-2,77734	-2,61094	-1,5164	NaN	S	2	___2
-2,77734	-2,61094	-1,5164	NaN	S	2	___2
-1,772	-1,59797	-2,70987	NaN	S	2	___2
-1,93004	-0,754211	NaN	NaN	S	3	___2
-1,60197	-1,39022	NaN	-1,05998	S	2	___2
-1,60197	-1,39022	NaN	-1,05998	S	2	___2
-0,217976	-0,765892	1,95378	0,128664	S	2	___2
2,2319	0,0883064	2,037	-0,686737	S	2	___2
-0,996504	-0,219503	-1,71388	NaN	S	3	___2
-0,996504	-0,219503	-1,71388	NaN	S	3	___2
-0,882826	-0,777927	NaN	NaN	S	2	___2
-2,37873	NaN	-2,02183	0,320747	S	2	___2
-2,37873	NaN	-2,02183	0,320747	S	2	___2
-0,310204	NaN	-0,613224	-0,866224	S	2	___2

-0,310204	NaN	-0,613224	-0,866224	S	2	___2
2,20013	2,33035	0,464937	-0,41807	S	2	___2
2,20013	2,33035	0,464937	-0,41807	S	2	___2
NaN	NaN	NaN	NaN	S	2	___2
NaN	NaN	NaN	-0,247444	S	3	___2
1,12535	1,58308	-2,18423	0,523827	S	2	___2
1,12535	1,58308	-2,18423	0,523827	S	2	___2
NaN	2,33106	2,17188	-0,347643	S	3	___2
1,00375	0,934807	NaN	0,603104	S	3	___2
1,00375	0,934807	NaN	0,603104	S	3	___2
-1,71152	NaN	-0,99979	-2,0751	S	2	___2
NaN	NaN	NaN	NaN	S	2	___2
-1,18972	-1,60236	NaN	-2,27249	S	2	___2
-1,18972	-1,60236	NaN	-2,27249	S	2	___2
NaN	NaN	NaN	NaN	S	3	___2
NaN	NaN	NaN	NaN	S	3	___2
NaN	1,31081	-0,73872	0,0495949	S	2	___2
NaN	1,31081	-0,73872	0,0495949	S	3	___2
-0,854734	-0,891727	NaN	-0,0704441	S	2	___2
-0,0987587	1,19915	-1,14674	1,97041	S	2	___2
-0,0987587	1,19915	-1,14674	1,97041	S	3	___2
-0,282589	1,66297	-1,96803	-0,439386	S	2	___2
-0,282589	1,66297	-1,96803	-0,439386	S	2	___2
-1,55357	-1,16044	NaN	-1,45754	S	3	___2
-1,55357	-1,16044	NaN	-1,45754	S	3	___2
-2,31381	NaN	-1,18566	NaN	S	2	___2
-0,844433	-2,34212	-1,57517	NaN	S	2	___2
-0,844433	-2,34212	NaN	NaN	S	2	___2
NaN	0,552113	NaN	0,448238	S	2	___2
NaN	0,552113	-1,84361	0,448238	S	2	___2
NaN	-1,00155	NaN	NaN	S	3	___2
NaN	NaN	NaN	NaN	S	2	___2
NaN	NaN	NaN	NaN	S	3	___2
NaN	NaN	NaN	NaN	S	3	___2

0,227407	0,883881	-1,25484	0,876368	S	3	___2
0,227407	0,883881	-1,25484	0,876368	S	3	___2
NaN	NaN	NaN	NaN	S	3	___2
NaN	0,459904	-1,97396	-2,32787	S	3	___2
NaN	NaN	NaN	NaN	S	3	___2
-1,09187	-0,00908661	NaN	-2,09929	S	2	___2
4,87599	4,89383	0,435812	2,59031	S	2	___2
1,91485	1,7597	3,42957	1,76554	S	2	___2
-0,755587	-0,955145	-2,65144	NaN	S	3	___2
NaN	-0,995014	NaN	NaN	S	3	___2
1,05087	2,83925	0,244425	2,25121	S	2	___2
1,05087	2,83925	0,244425	2,25121	S	2	___2
-0,54981	0,403259	NaN	1,28645	S	4	___2
-0,54981	0,403259	NaN	1,28645	S	4	___2
NaN	NaN	NaN	NaN	S	3	___2
NaN	NaN	NaN	NaN	S	3	___2
NaN	NaN	NaN	NaN	S	3	___2
NaN	0,0378876	0,562216	-1,57372	S	3	___2
NaN	NaN	NaN	NaN	S	3	___2
NaN	NaN	NaN	NaN	S	3	___2
NaN	NaN	NaN	NaN	S	2	___2
NaN	NaN	NaN	NaN	S	2	___2
-2,7724	0,153812	NaN	-1,31557	S	2	___2
-2,7724	0,153812	NaN	-1,31557	S	2	___2
NaN	NaN	NaN	NaN	S	2	___2
NaN	NaN	NaN	NaN	S	2	___2
NaN	-0,319807	-3,41325	NaN	S	2	___2
-0,706024	-2,77541	-1,39122	-1,45703	S	2	___2
-0,706024	-2,77541	-1,39122	-1,45703	S	2	___2
-1,15397	NaN	NaN	-1,96419	S	2	___2
-1,15397	NaN	NaN	-1,96419	S	2	___2
-2,4667	2,9751	-1,78141	NaN	S	2	___2
-5,832	1,39392	NaN	-1,18697	S	3	___2
-5,832	1,39392	NaN	-1,18697	S	3	___2

3,98151	5,13891	4,02439	3,83476	S	3	___2
0,912348	2,82467	0,0186157	2,17747	S	3	___2
NaN	2,82467	NaN	2,17747	S	3	___2
4,22413	4,10573	2,78697	3,58124	S	2	___2
4,22413	4,10573	2,78697	3,58124	S	2	___2
3,757	NaN	4,34325	3,35296	S	2	___2
3,757	NaN	4,34325	3,35296	S	2	___2
3,28124	3,3366	4,39262	2,71056	S	2	___2
NaN	NaN	NaN	NaN	S	2	___2
3,28124	3,3366	4,39262	2,71056	S	2	___2
0,182959	-0,628904	NaN	-0,0807629	S	2	___2
0,182959	-0,628904	NaN	-0,0807629	S	2	___2
NaN	NaN	NaN	NaN	S	2	___2
NaN	NaN	NaN	NaN	S	2	___2
-0,478996	1,28784	NaN	1,07524	S	2	___2
-0,478996	1,28784	NaN	1,07524	S	2	___2
NaN	NaN	NaN	NaN	S	2	___2
NaN	NaN	NaN	NaN	S	2	___2
-0,0209045	NaN	-1,57934	-1,40641	S	2	___2
-0,0209045	NaN	-1,57934	-1,40641	S	2	___2
NaN	NaN	NaN	NaN	S	2	___2
3,11123	3,57753	2,12762	3,70816	S	2	___2
3,11123	3,57753	2,12762	3,70816	S	2	___2
NaN	NaN	NaN	NaN	S	2	___2
NaN	NaN	NaN	NaN	S	2	___2
NaN	NaN	NaN	NaN	S	2	___2
NaN	NaN	NaN	NaN	S	2	___2
-0,182194	1,59873	NaN	0,198244	S	3	___2
NaN	0,532545	1,40972	NaN	S	2	___2
NaN	0,532545	1,40972	NaN	S	2	___2
NaN	NaN	NaN	NaN	S	3	___2
NaN	-0,366924	-1,97588	-1,91309	S	3	___2
NaN	-0,366924	-1,97588	-1,91309	S	3	___2
NaN	NaN	NaN	NaN	S	2	___2

NaN	NaN	NaN	NaN	S	2	___2
1,02091	0,247194	2,05543	NaN	S	2	___2
1,02091	0,247194	2,05543	NaN	S	3	___2
-3,18093	-0,0354881	2,63501	3,89146	S	2	___2
NaN	2,54789	-0,880116	1,46175	S	3	___2
-2,95212	-1,37669	NaN	0,484068	S	2	___2
-0,205736	0,0259514	NaN	NaN	S	2	___2
-0,205736	0,0259514	NaN	NaN	S	2	___2
NaN	0,179472	NaN	1,07723	S	3	___2
NaN	NaN	NaN	NaN	S	3	___2
NaN	0,179472	0,968781	1,07723	S	3	___2
0,698832	-0,0181141	0,127085	-0,166817	S	3	___2
0,698832	-0,0181141	0,127085	-0,166817	S	3	___2
NaN	0,0032177	NaN	NaN	S	3	___2
-0,0631027	0,86989	NaN	-1,66407	S	3	___2
-0,177994	0,00887871	-1,27498	NaN	S	2	___2
-0,177994	0,00887871	-1,27498	NaN	S	2	___2
NaN	NaN	NaN	NaN	S	2	___2
NaN	NaN	NaN	NaN	S	2	___2
NaN	NaN	NaN	NaN	S	2	___2
3,5697	3,80354	-3,48617	3,22605	S	3	___2
3,5697	3,80354	-3,48617	3,22605	S	3	___2
NaN	NaN	NaN	NaN	S	3	___2
-0,583784	-0,705564	NaN	0,26544	S	3	___2
-0,583784	-0,705564	NaN	0,26544	S	3	___2
NaN	NaN	NaN	NaN	S	3	___2
NaN	NaN	NaN	NaN	S	3	___2
NaN	NaN	NaN	NaN	S	3	___2
NaN	1,19604	NaN	-0,831669	S	3	___2
NaN	1,19604	NaN	-0,831669	S	3	___2
NaN	NaN	NaN	-0,143963	S	3	___2
0,443121	-0,257687	NaN	NaN	S	3	___2
0,443121	-0,257687	NaN	NaN	S	3	___2
0,897823	1,76213	1,13325	-0,689756	S	2	___2

NaN	NaN	NaN	NaN	S	3	___2
NaN	NaN	NaN	NaN	S	3	___2
NaN	NaN	NaN	NaN	S	2	___2
-2,80957	-1,67804	2,19799	NaN	S	2	___2
-1,94157	0,33556	3,92554	-0,220173	S	3	___2
-1,94157	0,33556	3,92554	-0,220173	S	3	___2
NaN	NaN	NaN	NaN	S	3	___2
-1,01536	-2,41591	-1,5496	-0,310305	S	2	___2
-1,01536	-2,41591	-1,5496	-0,310305	S	2	___2
NaN	-0,814249	NaN	NaN	S	2	___2
NaN	NaN	NaN	NaN	S	2	___2
NaN	NaN	NaN	NaN	S	2	___2
NaN	0,400967	-0,0977287	0,202892	S	2	___2
NaN	-1,84815	1,58867	-2,43033	S	3	___2
1,25872	2,99234	0,455345	3,00539	S	2	___2
1,25872	2,99234	0,455345	3,00539	S	4	___2
-0,756289	-1,78781	NaN	-1,24364	S	2	___2
NaN	NaN	NaN	NaN	S	2	___2
NaN	NaN	NaN	NaN	S	2	___2
-1,37923	-0,489473	NaN	-1,00752	S	2	___2
-1,37923	-0,489473	NaN	-1,00752	S	2	___2
-1,64077	-0,553995	NaN	NaN	S	2	___2
0,22995	-1,85395	-0,806587	-0,14435	S	2	___2
0,22995	-1,85395	-0,806587	-0,14435	S	3	___2
NaN	NaN	1,26004	NaN	S	2	___2
NaN	NaN	1,26004	NaN	S	2	___2
NaN	NaN	NaN	NaN	S	2	___2
NaN	NaN	NaN	NaN	S	2	___2
1,79156	0,344421	NaN	0,238689	S	2	___2
1,79156	0,344421	NaN	0,238689	S	2	___2
-0,468939	-4,57538	3,32309	2,0681	S	2	___2
-0,468939	-4,57538	3,32309	2,0681	S	2	___2
-0,701172	-2,18739	NaN	-1,16196	S	2	___2
-0,701172	-2,18739	NaN	-1,16196	S	4	___2

0,423571	-0,995195	NaN	NaN	S	2	___2
0,423571	-0,995195	NaN	NaN	S	3	___2
NaN	NaN	0,23978	NaN	S	3	___2
1,17465	0,864071	0,23978	0,162775	S	2	___2
3,44541	4,18687	-1,78887	2,88124	S	4	___2
3,44541	4,18687	-1,78887	2,88124	S	4	___2
NaN	NaN	NaN	NaN	S	2	___2
NaN	NaN	NaN	NaN	S	2	___2
-0,25252	1,66879	NaN	NaN	S	2	___2
-0,25252	1,66879	NaN	NaN	S	2	___2
-0,225815	-0,15243	-0,644135	-0,218565	S	3	___2
-0,362431	-0,35137	-0,491611	-1,42945	S	2	___2
-0,362431	-0,35137	-0,491611	-1,42945	S	2	___2
NaN	NaN	NaN	NaN	S	3	___2
0,756533	1,82349	1,19178	0,314224	S	3	___2
0,756533	1,82349	1,19178	0,314224	S	2	___2
-0,0467625	NaN	NaN	NaN	S	3	___2
-0,0467625	0,0666866	NaN	NaN	S	3	___2
-0,0467625	0,0666866	NaN	NaN	S	2	___2
0,0487137	0,0993156	-0,799156	NaN	S	3	___2
0,0487137	0,0993156	-0,799156	NaN	S	3	___2
NaN	NaN	NaN	NaN	S	3	___2
NaN	NaN	NaN	NaN	S	4	___2
NaN	1,0648	NaN	-0,37093	S	2	___2
NaN	NaN	NaN	NaN	S	2	___2
NaN	NaN	NaN	NaN	S	2	___2
-0,244616	-2,40263	-1,6559	1,00682	S	2	___2
-0,244616	-1,28225	-1,6559	1,00682	S	2	___2
-1,98686	-0,443705	NaN	-1,79906	S	3	___2
0,633108	NaN	NaN	2,07496	S	3	___2
-0,345823	2,76135	0,64868	-0,303869	S	4	___2
NaN	NaN	NaN	NaN	S	2	___2
NaN	NaN	NaN	NaN	S	2	___2
0,102533	-1,1963	NaN	-0,254971	S	2	___2

0,102533	-1,1963	NaN	-0,254971	S	2	___2
NaN	-0,358446	NaN	NaN	S	2	___2
NaN	-0,358446	NaN	NaN	S	2	___2
-1,00198	-0,808001	2,13654	0,0611591	S	2	___2
2,83552	1,40269	NaN	1,23244	S	2	___2
NaN	-0,808001	2,13654	NaN	S	2	___2
NaN	NaN	-0,439993	NaN	S	2	___2
NaN	NaN	-0,439993	NaN	S	2	___2
1,09645	1,37435	NaN	0,156862	S	2	___2
1,09645	1,37435	NaN	0,156862	S	2	___2
-0,203739	-1,59403	-1,01311	NaN	S	3	___2
-0,203739	-1,59403	-1,01311	NaN	S	2	___2
NaN	NaN	NaN	NaN	S	2	___2
NaN	NaN	NaN	NaN	S	2	___2
-0,203739	-1,59403	-1,01311	NaN	S	2	___2
NaN	NaN	NaN	NaN	S	2	___2
NaN	0,412516	NaN	-0,0476513	S	2	___2
1,02831	0,221357	2,37406	0,606077	S	3	___2
1,02831	0,221357	2,37406	0,606077	S	3	___2
NaN	NaN	NaN	NaN	S	2	___2
NaN	NaN	NaN	NaN	S	2	___2
NaN	NaN	NaN	NaN	S	3	___2
NaN	-1,62453	-0,215044	NaN	S	3	___2
NaN	-1,62453	-0,215044	NaN	S	3	___2
NaN	0,128868	NaN	NaN	S	3	___2
NaN	0,128868	NaN	NaN	S	3	___2
NaN	NaN	NaN	NaN	S	2	___2
NaN	NaN	NaN	NaN	S	2	___2
NaN	NaN	NaN	NaN	S	4	___2
NaN	-0,280888	1,51957	-2,26718	S	2	___2
-1,03503	-0,188078	-2,7573	-1,97372	S	3	___2
-1,03503	-0,188078	-2,7573	-1,97372	S	3	___2
0,918024	-0,431387	-1,47803	-2,01195	S	2	___2
0,918024	-0,431387	-1,47803	-2,01195	S	2	___2

NaN	NaN	NaN	NaN	S	2	___2
NaN	NaN	NaN	NaN	S	2	___2
NaN	NaN	NaN	NaN	S	3	___2
0,643215	2,68974	-0,61013	0,601175	S	3	___2
0,643215	2,68974	-0,61013	0,601175	S	3	___2
0,945145	0,731079	NaN	0,2684	S	3	___2
0,945145	0,731079	NaN	0,2684	S	3	___2
0,291006	-0,611776	NaN	-2,49568	S	2	___2
0,291006	-0,611776	NaN	-2,49568	S	2	___2
0,399107	-1,05054	NaN	-1,06924	S	2	___2
0,399107	-1,05054	NaN	-1,06924	S	2	___2
NaN	NaN	NaN	NaN	S	4	___2
NaN	NaN	NaN	NaN	S	2	___2
-1,26159	-2,35555	NaN	NaN	S	2	___2
NaN	NaN	NaN	NaN	S	2	___2
NaN	NaN	NaN	NaN	S	2	___2
0,933237	NaN	0,0320396	0,696896	S	2	___2
NaN	2,69232	NaN	NaN	S	2	___2
0,933237	2,69232	0,0320396	0,696896	S	2	___2
NaN	-0,0947151	NaN	NaN	S	3	___2
NaN	-0,0947151	NaN	NaN	S	3	___2
0,949404	0,0797272	1,73478	0,294706	S	2	___2
0,949404	0,0797272	1,73478	0,294706	S	2	___2
NaN	1,0827	NaN	NaN	S	2	___2
1,26066	1,0827	NaN	0,657253	S	4	___2
NaN	0,735638	0,772665	-0,301819	S	2	___2
NaN	0,735638	0,772665	-0,301819	S	2	___2
4,08468	3,85939	0,232824	3,25723	S	2	___2
NaN	-0,692156	NaN	-1,13064	S	3	___2
NaN	NaN	NaN	NaN	S	2	___2
NaN	NaN	NaN	NaN	S	2	___2
NaN	NaN	NaN	NaN	S	3	___2
NaN	0,739183	0,187908	-0,486589	S	2	___2
NaN	NaN	NaN	NaN	S	3	___2

NaN	NaN	NaN	NaN	S	3	___2
NaN	NaN	NaN	NaN	S	3	___2
NaN	NaN	NaN	NaN	S	3	___2
NaN	NaN	NaN	NaN	S	3	___2
3,17373	2,79276	2,17297	2,24332	S	3	___2
NaN	0,377295	NaN	NaN	S	3	___2
3,17373	2,79276	2,17297	2,24332	S	3	___2
NaN	-1,26534	0,411098	NaN	S	4	___2
NaN	-1,26534	0,411098	NaN	S	4	___2
1,69663	2,51645	2,65151	1,28405	S	3	___2
2,74322	2,99425	2,74526	NaN	S	3	___2
2,74322	2,99425	NaN	NaN	S	3	___2
0,634604	-3,23241	-2,64136	-1,30078	S	2	___2
0,340416	0,158981	1,78163	0,315674	S	3	___2
NaN	NaN	-0,68572	-1,83455	S	3	___2
-1,95306	-1,21148	NaN	-2,12839	S	2	___2
-1,95306	-1,21148	NaN	-2,12839	S	2	___2
NaN	NaN	NaN	NaN	S	2	___2
NaN	NaN	NaN	NaN	S	2	___2
NaN	NaN	NaN	NaN	S	3	___2
-1,10024	-1,39937	0,0112438	NaN	S	2	___2
-1,10024	-1,39937	0,0112438	NaN	S	2	___2
NaN	NaN	NaN	NaN	S	2	___2
3,33672	2,42344	2,42934	2,60012	S	2	___2
3,33672	2,42344	2,42934	2,60012	S	2	___2
4,98405	4,55704	1,89617	4,38679	S	2	___2
4,98405	4,55704	1,89617	4,38679	S	2	___2
0,273998	2,5006	-1,23624	3,56846	S	3	___2
-1,99997	-0,010231	-0,558317	0,294189	S	2	___2
3,2766	1,55268	1,79175	3,19341	S	2	___2
3,2766	1,55268	1,79175	3,19341	S	2	___2
3,17077	3,50327	NaN	3,56759	S	2	___2
-1,34785	-2,29926	-1,51613	NaN	S	2	___2
NaN	NaN	NaN	NaN	S	2	___2

2,88627	5,31452	-0,983086	1,0851	S	2	___2
NaN	-0,093502	-0,416286	-1,55137	S	2	___2
NaN	-0,093502	-0,416286	-1,55137	S	2	___2
1,04197	-0,0719891	-0,541958	NaN	S	2	___2
1,04197	-0,0719891	-0,541958	NaN	S	2	___2
-1,41509	-0,779228	-0,703878	NaN	S	3	___2
-1,41509	-0,779228	-0,703878	NaN	S	3	___2
NaN	NaN	0,648962	-1,15511	S	3	___2
NaN	NaN	0,648962	-1,15511	S	3	___2
NaN	NaN	NaN	NaN	S	3	___2
-0,830009	NaN	NaN	NaN	S	3	___2
NaN	NaN	NaN	NaN	S	2	___2
NaN	NaN	NaN	NaN	S	2	___2
4,22413	4,10573	2,78697	3,58124	S	2	___2
4,22413	4,10573	2,78697	3,58124	S	2	___2
3,2766	1,55268	1,79175	3,19341	S	2	___2
3,2766	1,55268	1,79175	3,19341	S	2	___2
NaN	NaN	NaN	NaN	S	2	___2
NaN	NaN	NaN	NaN	S	3	___2
-0,0569973	NaN	NaN	1,22174	S	3	___2
-0,0569973	0,794811	NaN	1,22174	S	2	___2
0,568293	0,0729694	NaN	1,16384	S	2	___2
NaN	NaN	NaN	NaN	S	2	___2
1,30972	0,95199	0,885563	-0,160257	S	2	___2
1,30972	NaN	0,885563	-0,160257	S	2	___2
NaN	0,95199	NaN	NaN	S	2	___2
-0,420696	-2,34185	-0,503704	-1,36576	S	2	___2
-0,420696	-2,34185	-0,503704	-1,36576	S	2	___2
0,154011	-1,07024	NaN	NaN	S	3	___2
0,154011	-1,07024	NaN	NaN	S	2	___2
0,790321	-0,144951	-0,401178	NaN	S	2	___2
0,994133	-0,153357	0,052248	NaN	S	2	___2
NaN	-1,24093	NaN	NaN	S	2	___2
NaN	NaN	NaN	NaN	S	3	___2

Polito et al. Universal ready-to-use immunotherapeutic approach for the treatment of cancer: expanded and activated polyclonal γδ memory T cells.
Phosphorylation sites

NaN	-1,96083	NaN	-0,941065	S	2	___2
-0,920038	-1,2701	NaN	NaN	S	3	___2
-0,920038	-1,2701	NaN	NaN	S	3	___2
0,757681	-0,191191	-1,53159	-1,46703	S	2	___2
0,757681	-0,191191	-1,53159	-1,46703	S	2	___2
0,0961285	NaN	NaN	NaN	S	2	___2
NaN	0,984224	-0,250212	1,66238	S	2	___2
NaN	0,984224	-0,250212	1,66238	S	2	___2
NaN	-1,93301	-0,4963	NaN	S	3	___2
1,54578	0,534063	NaN	0,726574	S	2	___2
-0,7946	NaN	NaN	NaN	S	2	___2
-0,7946	NaN	NaN	NaN	S	2	___2
-1,70936	NaN	NaN	NaN	S	2	___2
1,54578	0,534063	NaN	0,726574	S	2	___2
-0,267162	-1,3009	-0,872383	-0,682859	S	2	___2
-0,267162	-1,3009	-0,872383	-0,682859	S	2	___2
NaN	NaN	NaN	NaN	S	2	___2
0,135521	2,26485	1,62686	0,472599	S	2	___2
0,135521	2,26485	1,62686	0,472599	S	2	___2
NaN	0,0870953	0,00483322	-0,34642	S	2	___2
NaN	0,0870953	0,00483322	-0,34642	S	2	___2
-0,28474	NaN	-0,279518	NaN	S	3	___2
-0,28474	NaN	-0,279518	NaN	S	3	___2
NaN	0,268871	NaN	NaN	S	3	___2
NaN	0,268871	NaN	NaN	S	3	___2
0,0305462	NaN	1,11101	-0,361179	S	3	___2
0,0305462	NaN	1,11101	-0,361179	S	3	___2
NaN	NaN	-0,516565	0,256546	S	2	___2
2,22169	1,60487	-0,516565	0,256546	S	2	___2
NaN	-0,264133	-1,14278	-1,75744	S	3	___2
NaN	-0,264133	-1,14278	-1,75744	S	3	___2
NaN	NaN	NaN	NaN	S	2	___2
NaN	NaN	NaN	NaN	S	2	___2
NaN	-0,554329	NaN	NaN	S	2	___2

NaN	NaN	NaN	NaN	S	2	___2
-0,0315247	-0,347603	NaN	-1,78859	S	3	___2
-0,0315247	-0,347603	NaN	-1,78859	S	2	___2
-0,725828	0,345734	0,0704041	1,4842	S	3	___2
-3,35913	-2,89722	0,0336094	NaN	S	2	___2
-3,35913	-2,89722	0,0336094	NaN	S	2	___2
2,36752	2,09627	2,6157	1,32464	S	2	___2
2,36752	2,09627	2,6157	1,32464	S	2	___2
NaN	NaN	NaN	NaN	S	2	___2
NaN	NaN	NaN	NaN	S	2	___2
NaN	-1,17332	NaN	NaN	S	2	___2
NaN	-1,17332	NaN	NaN	S	2	___2
-1,46587	NaN	0,420834	-1,71776	S	2	___2
-1,46587	1,12963	0,420834	-1,71776	S	2	___2
NaN	NaN	NaN	NaN	S	2	___2
NaN	NaN	NaN	NaN	S	2	___2
NaN	NaN	NaN	NaN	S	2	___2
-1,04194	-2,08534	NaN	-1,67802	S	2	___2
-1,04194	-2,08534	NaN	-1,67802	S	2	___2
0,30966	2,40709	2,59858	2,25936	S	2	___2
0,30966	2,40709	2,59858	2,25936	S	2	___2
NaN	NaN	NaN	NaN	S	2	___2
NaN	NaN	-0,257509	NaN	S	4	___2
NaN	NaN	NaN	NaN	S	4	___2
NaN	NaN	NaN	NaN	S	2	___2
NaN	NaN	NaN	NaN	S	2	___2
0,882006	2,56861	2,73436	2,87605	S	3	___2
0,882006	2,56861	2,73436	2,87605	S	3	___2
-1,13194	-0,744019	-0,796324	-0,958076	S	2	___2
-1,15667	-0,931837	-0,285774	-0,673414	S	2	___2
-1,15667	-0,931837	-0,285774	-0,673414	S	2	___2
NaN	NaN	-0,337948	NaN	S	2	___2
NaN	NaN	-0,337948	NaN	S	2	___2
-0,373924	-1,05906	NaN	-0,77746	S	3	___2

-0,373924	-1,05906	NaN	-0,77746	S	2	___2
1,37703	0,858606	0,0605736	NaN	S	2	___2
1,37703	0,858606	0,0605736	NaN	S	2	___2
NaN	NaN	NaN	NaN	S	2	___2
NaN	NaN	NaN	NaN	S	2	___2
NaN	NaN	NaN	NaN	S	2	___2
NaN	-0,273266	NaN	NaN	S	2	___2
0,927637	1,49555	NaN	NaN	S	3	___2
0,927637	1,49555	NaN	NaN	S	3	___2
2,73002	1,92168	1,7277	2,03944	S	3	___2
2,73002	1,92168	1,7277	2,03944	S	3	___2
NaN	NaN	NaN	NaN	S	3	___2
NaN	NaN	NaN	NaN	S	3	___2
3,75004	2,82317	3,00967	2,92236	S	2	___2
3,75004	2,82317	3,00967	2,92236	S	2	___2
6,08141	5,65924	0,665401	4,70171	S	2	___2
-1,7455	-3,08017	NaN	NaN	S	3	___2
-1,7455	-3,08017	NaN	NaN	S	3	___2
6,08141	5,65924	0,665401	4,70171	S	2	___2
NaN	NaN	NaN	NaN	S	3	___2
1,65895	-0,912121	-0,889614	-0,339024	S	2	___2
1,65895	-0,912121	-0,889614	-0,339024	S	2	___2
0,847223	NaN	0,825891	-0,117952	S	2	___2
-0,563622	NaN	1,12911	NaN	S	2	___2
-0,563622	NaN	1,12911	NaN	S	2	___2
1,32763	0,957542	1,01004	-1,27663	S	2	___2
2,24307	3,71328	1,36298	2,47408	S	2	___2
2,24307	3,71328	1,36298	2,47408	S	2	___2
-1,73797	-1,71766	-0,961987	NaN	S	2	___2
-1,73797	-1,71766	-0,961987	NaN	S	2	___2
NaN	0,977312	NaN	-0,389927	S	3	___2
4,45296	4,70301	4,67063	2,64155	S	3	___2
2,58315	NaN	2,79976	NaN	S	3	___2
2,58315	2,13335	2,79976	2,8631	S	2	___2

Polito et al. Universal ready-to-use immunotherapeutic approach for the treatment of cancer: expanded and activated polyclonal γδ memory T cells.
Phosphorylation sites

NaN	0,966143	NaN	NaN	S	4	___2
-1,54354	2,86153	2,01206	2,20059	S	2	___2
2,34874	2,86153	2,01206	2,20059	S	3	___2
NaN	0,976194	-0,780846	NaN	S	3	___2
3,80759	3,87497	3,9014	1,65935	S	3	___2
3,80759	3,87497	3,9014	1,65935	S	2	___2
NaN	NaN	NaN	NaN	S	2	___2
-0,823372	0,342869	-2,85218	-1,47593	S	2	___2
NaN	NaN	0,99272	-0,32036	S	3	___2
-0,845926	-1,6297	-0,968758	NaN	S	3	___2
-0,845926	-1,6297	-0,968758	NaN	S	3	___2
2,48976	0,945236	0,810314	0,278362	S	2	___2
2,48976	0,945236	0,810314	0,278362	S	2	___2
NaN	NaN	NaN	NaN	S	2	___2
NaN	NaN	NaN	NaN	S	2	___2
-0,450687	0,412628	0,328917	-0,236473	S	2	___2
-0,450687	0,412628	0,328917	-0,236473	S	2	___2
NaN	NaN	NaN	NaN	S	2	___2
NaN	0,434778	1,27598	-0,298069	S	4	___2
-1,48602	NaN	NaN	NaN	S	2	___2
NaN	NaN	NaN	NaN	S	3	___2
NaN	NaN	NaN	NaN	S	2	___2
0,214537	1,27452	1,5648	1,40459	S	3	___2
0,214537	1,27452	1,5648	1,40459	S	3	___2
-0,806791	-0,853563	-1,01862	-0,895731	S	2	___2
-0,806791	-0,853563	-1,01862	-0,895731	S	2	___2
0,519264	0,0503845	1,12303	-0,460602	S	2	___2
0,519264	0,0503845	1,12303	-0,460602	S	2	___2
NaN	NaN	NaN	NaN	S	3	___2
NaN	NaN	NaN	NaN	S	3	___2
NaN	NaN	NaN	NaN	S	3	___2
NaN	NaN	NaN	NaN	S	2	___2
NaN	NaN	NaN	NaN	S	2	___2
NaN	NaN	0,00369644	-1,48416	S	3	___2

NaN	NaN	0,00369644	-1,48416	S	3	___2
NaN	0,935915	-0,784128	2,39861	S	2	___2
NaN	0,935915	-0,784128	2,39861	S	2	___2
1,77384	1,09396	2,00848	3,91499	S	2	___2
1,77384	1,09396	2,00848	3,91499	S	2	___2
-0,78627	-0,228088	-0,732828	0,0416508	S	3	___2
-0,337788	-2,55923	NaN	0,416893	S	2	___2
NaN	NaN	NaN	NaN	S	2	___2
NaN	NaN	NaN	NaN	S	2	___2
1,13962	1,59728	0,55398	-0,366938	S	3	___2
1,63416	2,23153	1,12911	1,82836	S	3	___2
-0,0118294	0,726688	0,106478	0,383846	S	3	___2
NaN	NaN	NaN	NaN	S	3	___2
NaN	NaN	NaN	NaN	S	2	___2
NaN	NaN	NaN	NaN	S	3	___2
-0,949177	0,0684948	NaN	NaN	S	5	___2
2,51204	2,30363	NaN	-4,92303	S	2	___2
2,51137	2,87905	NaN	1,13672	S	2	___2
2,51137	2,87905	NaN	1,13672	S	2	___2
2,51204	2,30363	NaN	-0,128004	S	2	___2
-0,0355682	0,418142	NaN	0,967833	S	2	___2
NaN	NaN	NaN	NaN	S	2	___2
NaN	NaN	NaN	NaN	S	3	___2
NaN	NaN	NaN	NaN	S	2	___2
NaN	-1,82149	-3,03408	-0,872044	S	2	___2
NaN	NaN	NaN	NaN	S	2	___2
NaN	NaN	NaN	NaN	S	2	___2
0,586658	-0,364775	NaN	1,39509	S	2	___2
0,586658	-0,364775	NaN	1,39509	S	2	___2
0,622051	-1,15989	-1,35404	-0,0648956	S	3	___2
0,622051	-1,15989	-1,35404	-0,0648956	S	3	___2
1,5251	2,18637	1,8889	1,84091	S	3	___2
0,853104	-1,02117	-0,419365	-1,57777	S	2	___2
NaN	-2,11619	-1,03508	-1,34496	S	2	___2

NaN	-2,11619	-1,03508	-1,34496	S	2	___2
NaN	NaN	NaN	NaN	S	2	___2
NaN	NaN	NaN	NaN	S	2	___2
0,739098	NaN	2,16318	-0,158739	S	2	___2
2,73794	1,81997	3,92639	2,20197	S	3	___2
2,73794	1,81997	3,92639	NaN	S	2	___2
NaN	NaN	NaN	NaN	S	2	___2
NaN	NaN	NaN	NaN	S	2	___2
1,8291	2,32944	-0,0288429	2,97232	S	2	___2
0,138136	0,171352	0,0729504	1,03556	S	2	___2
0,138136	0,171352	0,0729504	1,03556	S	2	___2
NaN	NaN	NaN	NaN	S	2	___2
NaN	NaN	NaN	NaN	S	2	___2
NaN	NaN	NaN	NaN	S	3	___2
NaN	NaN	NaN	NaN	S	3	___2
NaN	NaN	NaN	NaN	S	3	___2
3,75004	2,82317	3,00967	2,92236	S	2	___2
3,75004	2,82317	3,00967	2,92236	S	2	___2
NaN	0,753866	NaN	-1,29709	S	2	___2
NaN	0,753866	NaN	-1,29709	S	2	___2
NaN	-0,63666	0,95612	NaN	S	2	___2
NaN	-0,63666	0,95612	NaN	S	2	___2
2,45619	1,4882	1,77613	1,03156	S	2	___2
2,45619	1,4882	1,77613	1,03156	S	2	___2
NaN	NaN	NaN	NaN	S	3	___2
NaN	NaN	NaN	NaN	S	3	___2
NaN	NaN	NaN	NaN	S	3	___2
-0,492046	-0,742243	-1,72384	-0,515699	S	2	___2
-0,492046	-0,742243	-1,72384	-0,515699	S	2	___2
2,1292	2,44057	-0,84654	1,94191	S	2	___2
2,1292	2,44057	-0,84654	1,94191	S	2	___2
NaN	NaN	NaN	NaN	S	2	___2
NaN	NaN	NaN	NaN	S	2	___2
NaN	NaN	NaN	NaN	S	2	___2

NaN	NaN	NaN	NaN	S	2	___2
-1,37932	-0,273357	-2,50249	NaN	S	2	___2
0,266674	1,22376	1,37406	0,517672	S	3	___2
0,686958	0,971951	-1,09258	-2,21416	S	2	___2
2,0762	2,29535	0,898979	0,341694	S	2	___2
2,0762	2,29535	0,898979	0,341694	S	2	___2
NaN	NaN	NaN	NaN	S	3	___2
3,64818	3,43917	3,89301	3,70931	S	2	___2
5,10918	4,19844	3,56985	3,86497	S	2	___2
1,48507	2,14825	-1,04053	1,05947	S	2	___2
3,18238	3,38005	2,33949	1,36858	S	3	___2
3,18238	3,38005	2,33949	1,36858	S	3	___2
-2,26621	-2,12609	0,0629826	-0,33639	S	2	___2
NaN	NaN	NaN	NaN	S	2	___2
1,26243	1,68333	NaN	0,484034	S	3	___2
1,26243	1,68333	NaN	0,484034	S	3	___2
NaN	NaN	NaN	NaN	S	2	___2
-0,496878	0,020134	1,38426	1,07112	S	3	___2
NaN	NaN	NaN	NaN	S	3	___2
-0,340111	-0,353014	NaN	-0,409489	S	2	___2
-1,01123	-1,06498	NaN	-2,83126	S	3	___2
-1,01123	-1,06498	NaN	-2,83126	S	3	___2
1,54126	NaN	NaN	-0,292006	S	2	___2
1,54126	NaN	NaN	-0,292006	S	2	___2
-1,82896	-2,81956	NaN	-2,08292	S	2	___2
1,56066	0,00284004	NaN	1,03053	S	2	___2
3,36957	3,1574	-0,182932	1,6812	S	2	___2
3,36957	3,1574	-0,182932	1,6812	S	2	___2
NaN	NaN	NaN	NaN	S	3	___2
-1,02965	-1,44051	-0,437437	NaN	S	2	___2
-1,02965	-1,44051	-0,437437	NaN	S	2	___2
NaN	NaN	NaN	NaN	S	3	___2
NaN	NaN	-0,972406	NaN	S	2	___2
NaN	NaN	NaN	NaN	S	2	___2

0,785265	0,871838	-0,640865	NaN	S	2	___2
2,06017	1,23376	3,95603	2,40795	S	2	___2
2,06017	1,23376	3,95603	2,40795	S	2	___2
-0,541668	-3,04097	1,63505	-1,40846	S	2	___2
-0,541668	-3,04097	1,63505	-1,40846	S	2	___2
NaN	NaN	NaN	NaN	S	3	___2
NaN	NaN	NaN	NaN	S	3	___2
-1,36123	NaN	NaN	NaN	S	2	___2
NaN	NaN	NaN	NaN	S	2	___2
NaN	NaN	NaN	NaN	S	3	___2
NaN	NaN	NaN	NaN	S	3	___2
NaN	NaN	NaN	NaN	S	3	___2
NaN	NaN	NaN	NaN	S	3	___2
-1,54515	-2,63664	-0,719431	NaN	S	2	___2
-1,54515	NaN	NaN	NaN	S	2	___2
NaN	NaN	NaN	NaN	S	2	___2
-1,14475	NaN	NaN	NaN	S	2	___2
0,0471058	-0,0166569	NaN	NaN	S	2	___2
0,513645	0,00789833	NaN	-1,18011	S	2	___2
2,16927	1,82567	0,14378	0,871765	S	2	___2
0,513645	0,00789833	-0,384312	-1,18011	S	2	___2
2,16927	1,82567	0,14378	0,871765	S	2	___2
0,755959	-0,193874	-0,682953	-0,863409	S	2	___2
NaN	NaN	NaN	NaN	S	2	___2
NaN	NaN	-0,393972	NaN	S	2	___2
-0,598137	-1,08887	NaN	0,267466	S	2	___2
1,95123	-0,0984421	NaN	0,351017	S	3	___2
1,95123	-0,0984421	NaN	0,351017	S	3	___2
2,01905	4,24994	NaN	0,613796	S	3	___2
2,01905	4,24994	NaN	0,613796	S	4	___2
-1,42865	-1,91389	-0,451849	-2,11767	S	2	___2
-1,42865	-1,91389	-0,451849	-2,11767	S	2	___2
NaN	0,930597	NaN	-0,262707	S	3	___2
NaN	0,930597	NaN	-0,262707	S	3	___2

NaN	NaN	NaN	NaN	S	3	___2
NaN	NaN	NaN	NaN	S	3	___2
NaN	NaN	NaN	NaN	S	3	___2
0,799608	1,46812	2,19066	0,642471	S	4	___2
0,799608	1,46812	2,19066	0,642471	S	3	___2
NaN	NaN	NaN	NaN	S	3	___2
NaN	NaN	NaN	NaN	S	3	___2
0,799608	1,46812	2,19066	0,642471	S	3	___2
0,799608	1,46812	2,19066	0,642471	S	3	___2
0,0660954	0,378229	-1,49164	-0,703424	S	2	___2
0,0660954	0,378229	-1,49164	-0,703424	S	2	___2
-1,35236	-0,496834	2,21568	-0,946472	S	2	___2
-1,35236	-0,496834	2,21568	-0,946472	S	2	___2
NaN	NaN	NaN	NaN	S	2	___2
NaN	NaN	NaN	NaN	S	2	___2
NaN	NaN	NaN	NaN	S	2	___2
NaN	NaN	NaN	NaN	S	2	___2
3,26752	1,49275	3,14314	1,24416	S	2	___2
6,00864	5,31654	5,56565	4,2367	S	2	___2
6,00864	5,31654	5,56565	4,2367	S	2	___2
NaN	-1,71648	0,173864	NaN	S	2	___2
NaN	-1,71648	0,173864	NaN	S	2	___2
NaN	1,44909	-0,948988	NaN	S	3	___2
0,88018	1,44909	-0,948988	NaN	S	2	___2
-0,758867	-0,234138	-0,797255	NaN	S	2	___2
NaN	NaN	NaN	NaN	S	2	___2
NaN	NaN	NaN	NaN	S	2	___2
0,479013	NaN	0,917458	-0,228601	S	2	___2
0,479013	NaN	0,917458	-0,228601	S	2	___2
-0,243959	0,947477	NaN	NaN	S	3	___2
-2,17959	-2,52149	NaN	-0,717377	S	2	___2
-2,17959	-2,52149	NaN	-0,717377	S	2	___2
NaN	NaN	NaN	-1,51155	S	2	___2
-1,60597	0,465458	0,361219	2,1235	S	2	___2

Polito et al. Universal ready-to-use immunotherapeutic approach for the treatment of cancer: expanded and activated polyclonal γδ memory T cells.
Phosphorylation sites

-1,60597	0,465458	0,361219	2,1235	S	2	___2
0,744837	1,51295	-0,234272	0,10725	S	4	___2
-0,955345	-1,88443	-1,30152	-1,17148	S	3	___2
1,47189	2,14608	2,81849	2,66438	S	3	___2
NaN	NaN	1,22183	NaN	S	2	___2
NaN	NaN	1,22183	NaN	S	2	___2
-0,315756	-1,78087	-1,18106	NaN	S	3	___2
-0,315756	-1,78087	-1,18106	NaN	S	3	___2
NaN	NaN	NaN	NaN	S	2	___2
2,03292	3,26893	3,81341	3,01635	S	3	___2
2,03292	3,26893	3,81341	3,01635	S	3	___2
-0,315756	-1,78087	-1,18106	NaN	S	3	___2
-0,315756	-1,78087	-1,18106	NaN	S	3	___2
NaN	NaN	NaN	NaN	S	2	___2
NaN	NaN	NaN	NaN	S	2	___2
NaN	NaN	NaN	NaN	S	2	___2
3,71303	3,39403	1,98663	0,490501	S	2	___2
3,71303	3,39403	-0,276836	0,490501	S	2	___2
-0,774073	-1,06892	NaN	-2,86394	S	2	___2
NaN	NaN	NaN	NaN	S	3	___2
NaN	-1,92036	-1,70081	-1,75215	S	2	___2
NaN	-1,92036	-1,70081	-1,75215	S	2	___2
NaN	NaN	NaN	NaN	S	3	___2
2,14189	-0,121098	-1,52113	-0,0629368	S	3	___2
NaN	NaN	NaN	0,110792	S	2	___2
1,00876	-0,35224	NaN	0,110792	S	2	___2
0,0504551	-2,05155	-0,591949	-1,15276	S	2	___2
0,0504551	-2,05155	-0,591949	-1,15276	S	2	___2
NaN	NaN	NaN	NaN	S	2	___2
NaN	NaN	NaN	NaN	S	3	___2
NaN	-0,722599	NaN	NaN	S	2	___2
NaN	-0,722599	NaN	NaN	S	2	___2
NaN	NaN	NaN	NaN	S	3	___2
NaN	NaN	NaN	NaN	S	3	___2

0,74946	0,441547	-3,0859	NaN	S	2	___2
NaN	0,441547	NaN	NaN	S	2	___2
0,576403	0,285881	-0,0633259	0,948299	S	3	___2
0,576403	0,285881	0,0470314	0,948299	S	2	___2
-1,8313	-1,95303	0,0470314	-0,802002	S	2	___2
-1,53036	-1,74768	-1,62305	NaN	S	2	___2
-1,53036	-1,74768	-1,62305	NaN	S	2	___2
NaN	NaN	NaN	NaN	S	3	___2
3,88873	3,70021	3,24688	3,1544	S	2	___2
3,88873	3,70021	3,24688	3,1544	S	2	___2
NaN	NaN	0,287851	NaN	S	2	___2
NaN	NaN	0,287851	NaN	S	2	___2
1,51992	0,308641	NaN	-0,0651169	S	2	___2
1,51992	0,308641	NaN	-0,0651169	S	2	___2
NaN	NaN	-0,303101	NaN	S	2	___2
0,660305	0,716024	-2,28192	NaN	S	3	___2
0,660305	0,716024	-2,28192	NaN	S	2	___2
-2,8669	0,835346	NaN	-0,359735	S	3	___2
-2,8669	0,835346	NaN	-0,359735	S	3	___2
0,883772	0,500168	0,00415993	-0,447157	S	2	___2
0,883772	0,500168	0,00415993	-0,447157	S	2	___2
-1,02788	NaN	NaN	-0,298328	S	2	___2
-1,02788	NaN	NaN	-0,298328	S	2	___2
-1,02788	NaN	NaN	-0,298328	S	2	___2
NaN	-1,9371	3,29889	-0,530037	S	2	___2
1,19624	0,692579	-0,508101	-1,95981	S	3	___2
1,19624	0,692579	-0,508101	-1,95981	S	3	___2
0,579536	1,68352	-0,261187	-0,0787582	S	2	___2
0,579536	1,68352	-0,261187	-0,0787582	S	2	___2
-1,23598	-1,01993	-0,9543	-1,64849	S	2	___2
-1,23598	-1,01993	-0,9543	-1,64849	S	2	___2
NaN	NaN	NaN	NaN	S	2	___2
NaN	NaN	NaN	NaN	S	3	___2
NaN	NaN	NaN	NaN	S	2	___2

Polito et al. Universal ready-to-use immunotherapeutic approach for the treatment of cancer: expanded and activated polyclonal γδ memory T cells.
Phosphorylation sites

NaN	NaN	NaN	NaN	S	2	___2
NaN	NaN	-0,720427	NaN	S	3	___2
NaN	NaN	-0,720427	NaN	S	3	___2
NaN	NaN	-0,738914	1,25843	S	3	___2
NaN	NaN	-0,738914	1,25843	S	3	___2
0,0863075	-2,54682	-1,24042	0,109251	S	3	___2
0,0863075	-2,54682	-1,24042	0,109251	S	3	___2
1,14689	1,36972	0,939833	0,102633	S	3	___2
1,14689	1,36972	0,939833	0,102633	S	2	___2
NaN	NaN	NaN	NaN	S	2	___2
NaN	NaN	NaN	NaN	S	2	___2
NaN	NaN	NaN	NaN	S	2	___2
NaN	-0,595766	0,557369	NaN	S	3	___2
NaN	NaN	NaN	NaN	S	3	___2
NaN	NaN	NaN	NaN	S	3	___2
1,61506	3,49456	NaN	NaN	S	2	___2
1,61506	3,49456	NaN	NaN	S	2	___2
NaN	NaN	2,18663	NaN	S	3	___2
NaN	NaN	2,18663	NaN	S	3	___2
NaN	NaN	NaN	NaN	S	2	___2
NaN	NaN	NaN	NaN	S	3	___2
NaN	NaN	NaN	NaN	S	2	___2
NaN	NaN	NaN	NaN	S	2	___2
NaN	NaN	NaN	NaN	S	2	___2
NaN	NaN	NaN	NaN	S	2	___2
3,29669	2,70086	NaN	2,32586	S	2	___2
NaN	2,70086	NaN	2,32586	S	2	___2
0,341728	-0,394257	-2,90238	0,986042	S	2	___2
2,57445	2,53514	2,3128	3,35834	S	2	___2
-0,344503	2,0943	-1,55035	-0,219027	S	2	___2
2,61636	2,95891	1,88329	2,1881	S	2	___2
2,61636	2,95891	1,88329	2,1881	S	2	___2
1,89347	-0,200914	-1,99561	NaN	S	2	___2
1,89347	-0,200914	-1,99561	NaN	S	2	___2

Polito et al. Universal ready-to-use immunotherapeutic approach for the treatment of cancer: expanded and activated polyclonal γδ memory T cells.
Phosphorylation sites

NaN	NaN	NaN	NaN	S	5	___2
NaN	NaN	NaN	NaN	S	5	___2
-1,01585	-0,451769	-1,85342	0,494629	S	2	___2
NaN	NaN	5,51234	5,78113	S	3	___2
NaN	NaN	5,51234	5,78113	S	3	___2
2,42117	2,09561	-1,52287	3,38797	S	2	___2
2,42117	2,09561	-1,52287	3,38797	S	2	___2
NaN	NaN	NaN	NaN	S	2	___2
0,383415	0,074913	-0,106422	-0,571278	S	2	___2
0,153015	0,468637	0,46347	1,39936	S	2	___2
0,383415	0,468637	0,46347	1,39936	S	2	___2
-0,129318	NaN	-3,09186	-1,29129	S	2	___2
-0,203819	-0,31584	-0,0506382	0,523403	S	2	___2
-0,494581	-0,0988483	-0,82325	-0,764509	S	2	___2
-0,494581	-0,0988483	-0,82325	-0,764509	S	2	___2
NaN	NaN	NaN	NaN	S	3	___2
NaN	NaN	NaN	NaN	S	2	___2
2,02008	0,31694	NaN	1,67623	S	2	___2
2,02008	0,31694	NaN	1,67623	S	2	___2
0,769335	0,0367794	NaN	-2,38118	S	2	___2
0,769335	0,0367794	NaN	-2,38118	S	2	___2
0,267193	1,44762	0,376038	0,158417	S	2	___2
2,3541	1,94338	NaN	1,58969	S	3	___2
2,3541	1,94338	NaN	1,58969	S	3	___2
1,46161	2,05934	NaN	1,91483	S	2	___2
1,46161	2,05934	NaN	1,91483	S	2	___2
-1,44818	-0,571121	NaN	-1,22605	S	2	___2
-1,44818	-0,571121	NaN	-1,22605	S	2	___2
-0,118671	-0,506491	NaN	-0,177797	S	2	___2
-0,118671	-0,506491	NaN	-0,177797	S	3	___2
2,68321	1,46971	NaN	NaN	S	3	___2
2,68321	-0,146795	NaN	NaN	S	2	___2
1,10892	1,29447	NaN	NaN	S	2	___2
-1,24154	-0,416239	NaN	NaN	S	2	___2

1,10892	1,29447	NaN	NaN	S	2	___2
0,342712	-0,145203	2,12655	NaN	S	2	___2
0,268803	0,849886	0,735405	-0,369295	S	2	___2
0,268803	0,849886	0,735405	-0,369295	S	2	___2
-1,15397	NaN	0,206018	NaN	S	3	___2
-1,15397	NaN	0,206018	NaN	S	3	___2
-0,173532	1,40812	-1,3618	2,55825	S	3	___2
-0,173532	1,40812	-1,3618	2,55825	S	3	___2
NaN	NaN	0,653791	-1,01072	S	2	___2
NaN	0,0865231	1,05979	-0,952761	S	3	___2
NaN	NaN	NaN	NaN	S	3	___2
NaN	NaN	NaN	NaN	S	3	___2
NaN	NaN	NaN	NaN	S	3	___2
NaN	NaN	NaN	NaN	S	3	___2
-2,65958	0,355246	NaN	0,544275	S	3	___2
-2,65958	0,355246	NaN	0,544275	S	3	___2
NaN	NaN	NaN	NaN	S	3	___2
-0,1087	NaN	-1,17605	0,738276	S	2	___2
-0,1087	NaN	-1,17605	0,738276	S	2	___2
NaN	NaN	1,26943	NaN	S	2	___2
NaN	NaN	-0,555441	NaN	S	2	___2
NaN	NaN	-0,555441	NaN	S	2	___2
NaN	NaN	NaN	NaN	S	3	___2
NaN	NaN	NaN	NaN	S	2	___2
NaN	NaN	NaN	NaN	S	2	___2
-0,581865	NaN	NaN	-0,34021	S	2	___2
-0,581865	NaN	NaN	-0,34021	S	2	___2
-1,19028	-2,13262	NaN	NaN	S	2	___2
-1,19028	-2,13262	NaN	NaN	S	2	___2
-0,406115	-0,0201893	-1,94132	-4,58494	S	2	___2
NaN	-1,687	NaN	NaN	S	2	___2
-0,84462	NaN	NaN	NaN	S	2	___2
NaN	-0,52524	NaN	NaN	S	3	___2
1,89487	1,96317	1,87824	1,47555	S	3	___2

1,89487	1,96317	1,87824	1,47555	S	3	___2
2,17779	2,36082	0,868965	-0,400463	S	2	___2
2,17779	2,36082	0,868965	-0,400463	S	2	___2
NaN	0,359446	-1,59416	NaN	S	2	___2
-0,86202	-1,85439	NaN	NaN	S	2	___2
NaN	NaN	NaN	NaN	S	3	___2
2,62531	2,52319	1,05767	0,638384	S	2	___2
NaN	NaN	1,05767	0,638384	S	2	___2
NaN	NaN	1,27992	NaN	S	2	___2
1,95749	1,64919	1,32474	1,14257	S	2	___2
NaN	NaN	1,00866	NaN	S	2	___2
1,95749	1,64919	1,32474	1,14257	S	2	___2
-0,193918	-0,887234	NaN	0,178137	S	2	___2
NaN	NaN	NaN	NaN	S	3	___2
NaN	-1,30801	-2,20447	NaN	S	2	___2
NaN	-1,30801	-2,20447	NaN	S	2	___2
1,77495	1,76209	0,136473	-0,142565	S	2	___2
1,77495	1,76209	0,136473	-0,142565	S	3	___2
0,142004	0,430904	0,809519	0,124115	S	3	___2
0,142004	0,430904	NaN	0,124115	S	2	___2
2,16988	1,62641	-1,1846	-0,704569	S	2	___2
2,16988	1,62641	-1,1846	-0,704569	S	3	___2
0,84857	-0,022419	1,73687	-0,350746	S	2	___2
0,84857	-0,022419	1,73687	-0,350746	S	2	___2
NaN	NaN	NaN	NaN	S	2	___2
NaN	NaN	NaN	NaN	S	2	___2
NaN	0,215103	-1,41003	0,787285	S	3	___2
NaN	0,215103	-1,41003	0,787285	S	3	___2
NaN	NaN	NaN	NaN	S	2	___2
NaN	NaN	NaN	NaN	S	2	___2
NaN	-1,0994	-1,71381	-1,4837	S	3	___2
NaN	-2,12162	NaN	NaN	S	3	___2
NaN	NaN	NaN	NaN	S	3	___2
NaN	NaN	NaN	NaN	S	3	___2

0,975523	NaN	0,732327	2,81691	S	4	___2
0,975523	NaN	0,732327	2,81691	S	4	___2
-1,13178	NaN	-0,311134	-1,52767	S	2	___2
-0,485336	-0,814383	-0,475763	-1,21003	S	3	___2
NaN	-0,814383	-0,475763	-1,21003	S	2	___2
NaN	NaN	NaN	NaN	S	2	___2
NaN	NaN	NaN	NaN	S	2	___2
-1,22549	-0,176039	-1,41238	-0,415443	S	2	___2
-1,22549	-0,176039	-1,41238	-0,415443	S	2	___2
NaN	NaN	NaN	NaN	S	2	___2
-0,797367	NaN	-1,3952	0,897646	S	2	___2
NaN	NaN	-1,57601	NaN	S	2	___2
-1,64542	NaN	NaN	NaN	S	2	___2
2,22339	2,01033	0,703747	1,32193	S	2	___2
2,22339	2,01033	0,703747	1,32193	S	2	___2
1,78786	1,18855	-0,659824	0,876368	S	2	___2
1,78786	1,18855	-0,659824	0,876368	S	2	___2
-1,06553	-1,86484	NaN	-1,16663	S	2	___2
-1,06553	-1,86484	NaN	-1,16663	S	2	___2
0,681784	1,26178	NaN	-0,148811	S	3	___2
NaN	NaN	1,45931	0,517868	S	2	___2
NaN	NaN	1,45931	0,517868	S	2	___2
0,425198	0,781071	0,100021	-0,738834	S	2	___2
-1,51979	-3,11981	NaN	-2,68528	S	2	___2
-1,51979	-3,11981	NaN	-2,68528	S	2	___2
NaN	0,620264	0,258598	-0,676556	S	2	___2
NaN	0,620264	0,258598	-0,676556	S	3	___2
0,352135	0,435787	1,25955	0,42597	S	3	___2
0,352135	0,435787	1,25955	0,42597	S	3	___2
0,542976	-0,327396	-0,155039	0,150904	S	2	___2
0,542976	-0,327396	-0,155039	0,150904	S	2	___2
2,16216	2,86892	-0,769096	0,986397	S	3	___2
2,16216	2,86892	-0,769096	0,986397	S	3	___2
-1,18027	-1,23619	-1,25523	NaN	S	2	___2

2,62728	3,09437	0,493008	2,45485	S	3	___2
2,62728	3,09437	0,493008	2,45485	S	2	___2
-2,76365	0,264399	2,32032	NaN	S	3	___2
-2,76365	0,264399	2,32032	NaN	S	3	___2
NaN	NaN	NaN	NaN	S	3	___2
2,91025	4,47366	NaN	3,7893	S	2	___2
2,91025	4,47366	NaN	3,7893	S	2	___2
1,16188	-0,652895	-1,00571	-0,382763	S	2	___2
1,16188	-0,652895	-1,00571	-0,382763	S	2	___2
-2,14454	0,369806	-1,36851	-0,19132	S	3	___2
-2,14454	0,369806	-1,36851	-0,19132	S	3	___2
1,59993	1,73265	1,22582	1,97184	S	2	___2
1,59993	1,73265	1,22582	1,97184	S	2	___2
0,127869	NaN	0,771711	NaN	S	3	___2
0,127869	NaN	0,771711	NaN	S	3	___2
-1,4698	-0,238512	NaN	-2,94128	S	4	___2
NaN	NaN	NaN	NaN	S	2	___2
NaN	NaN	NaN	NaN	S	3	___2
0,487984	-0,827236	1,3278	-1,41601	S	3	___2
0,487984	-0,827236	1,3278	-1,41601	S	2	___2
NaN	0,507225	0,0784721	-2,26854	S	2	___2
-0,497122	0,507225	3,31355	-0,767258	S	2	___2
-1,7668	-0,870092	1,26373	-2,12982	S	2	___2
NaN	NaN	NaN	NaN	S	2	___2
NaN	NaN	NaN	NaN	S	2	___2
1,94504	0,701183	0,0299435	0	S	2	___2
1,94504	0,701183	0,0299435	0	S	2	___2
0,161497	-0,385319	NaN	-1,03798	S	2	___2
1,51107	0,0792961	1,01719	1,72353	S	2	___2
3,41747	NaN	-1,20575	3,33243	S	3	___2
3,41747	NaN	-1,20575	3,33243	S	3	___2
NaN	NaN	NaN	NaN	S	2	___2
0,426615	1,7795	0,696728	0,701586	S	2	___2
0,426615	1,7795	0,696728	0,701586	S	2	___2

Polito et al. Universal ready-to-use immunotherapeutic approach for the treatment of cancer: expanded and activated polyclonal γδ memory T cells.
Phosphorylation sites

NaN	NaN	NaN	NaN	S	4	___2
NaN	NaN	NaN	NaN	S	3	___2
0,074543	-1,10581	NaN	-0,823107	S	3	___2
NaN	-0,712255	NaN	NaN	S	3	___2
NaN	NaN	NaN	NaN	S	2	___2
-0,554688	2,17949	1,91869	0,335228	S	2	___2
-0,554688	2,17949	1,91869	0,335228	S	2	___2
NaN	NaN	NaN	NaN	S	3	___2
0,625784	-0,840746	1,62836	-2,69887	S	2	___2
0,625784	-0,840746	1,62836	-2,69887	S	2	___2
2,82422	1,22632	-2,75383	-0,45796	S	2	___2
2,82422	1,22632	-2,75383	-0,45796	S	2	___2
-1,84605	-2,23551	-1,11893	NaN	S	2	___2
-1,0488	-1,16522	-2,3029	-1,49192	S	3	___2
-1,0488	-1,16522	-2,3029	-1,49192	S	2	___2
NaN	NaN	NaN	NaN	S	3	___2
NaN	NaN	NaN	NaN	S	3	___2
NaN	2,28163	-0,133587	NaN	S	3	___2
NaN	NaN	NaN	NaN	S	3	___2
-1,3278	NaN	-0,61861	-1,09368	S	3	___2
NaN	0,697262	-2,21913	NaN	S	4	___2
NaN	0,210197	0,472723	NaN	S	3	___2
-0,713732	-0,824146	-0,144171	-0,841667	S	2	___2
-0,713732	-0,824146	-0,144171	-0,841667	S	2	___2
0,896149	0,985641	-0,140139	-0,114677	S	2	___2
0,896149	0,985641	-0,140139	-0,114677	S	2	___2
0,135994	1,23958	2,40295	NaN	S	3	___2
0,135994	1,23958	2,40295	NaN	S	3	___2
0,104757	-0,235119	-2,18868	-1,75159	S	2	___2
0,104757	-0,235119	-2,18868	-1,75159	S	2	___2
NaN	2,76816	0,777969	0,628069	S	2	___2
2,86303	2,76816	0,777969	0,628069	S	3	___2
-1,37824	-0,623638	-0,457878	-0,670727	S	2	___2
-1,37824	-0,623638	-0,457878	-0,670727	S	2	___2

0,387712	0,695719	NaN	-2,75286	S	2	___2
0,387712	0,695719	NaN	-2,75286	S	2	___2
-0,876387	0,508398	0,661846	-0,12537	S	3	___2
-0,876387	0,508398	0,661846	-0,12537	S	3	___2
NaN	NaN	NaN	NaN	S	2	___2
0,695349	-0,560799	0,820631	-1,45915	S	2	___2
NaN	NaN	NaN	NaN	S	2	___2
NaN	-0,703342	NaN	-1,46405	S	2	___2
-0,605446	-0,703342	0,0788021	-1,46405	S	2	___2
-2,44777	-2,6559	-1,84704	NaN	S	2	___2
-0,917484	-1,46497	-0,0737953	-1,99822	S	2	___2
-0,917484	-1,46497	-0,0737953	-1,99822	S	2	___2
NaN	0,328213	NaN	-0,896254	S	5	___2
NaN	0,328213	NaN	-0,896254	S	4	___2
0,352406	0,196968	NaN	0,305531	S	3	___2
0,352406	0,196968	NaN	0,305531	S	3	___2
1,38342	-0,453117	NaN	-0,408182	S	3	___2
1,38342	-0,453117	NaN	-0,408182	S	3	___2
4,52257	4,51426	4,18719	4,05599	S	3	___2
4,52257	4,51426	4,18719	4,05599	S	3	___2
0,150301	0,289669	0,313923	0,731237	S	3	___2
-0,387094	0,101507	0,649511	-0,795401	S	3	___2
NaN	0,101507	NaN	NaN	S	3	___2
NaN	NaN	NaN	NaN	S	2	___2
0,622591	-1,59556	0,668188	0,80991	S	2	___2
0,622591	-1,59556	0,668188	0,80991	S	2	___2
-2,74228	-1,9855	-0,629406	-4,0185	S	3	___2
-2,74228	-1,9855	-0,629406	-4,0185	S	3	___2
NaN	NaN	NaN	NaN	S	3	___2
NaN	NaN	NaN	NaN	S	2	___2
0,55821	-0,0116825	0,861298	1,22745	S	2	___2
0,55821	-0,0116825	0,861298	1,22745	S	2	___2
0,324677	-1,72696	NaN	-0,742826	S	2	___2
0,324677	-1,72696	NaN	-0,742826	S	2	___2

Polito et al. Universal ready-to-use immunotherapeutic approach for the treatment of cancer: expanded and activated polyclonal γδ memory T cells.
Phosphorylation sites

NaN	NaN	NaN	NaN	S	2	___2
NaN	NaN	NaN	NaN	S	2	___2
-1,67787	-3,32842	-3,1208	NaN	S	2	___2
-1,67787	-3,32842	-3,1208	NaN	S	2	___2
NaN	NaN	NaN	NaN	S	2	___2
NaN	NaN	NaN	NaN	S	2	___2
NaN	NaN	NaN	NaN	S	2	___2
-0,212305	-0,702478	-1,96694	-1,11235	S	2	___2
-0,212305	-0,702478	-1,96694	-1,11235	S	2	___2
1,71162	1,01523	2,11058	2,50358	S	2	___2
NaN	NaN	NaN	NaN	S	2	___2
NaN	NaN	NaN	NaN	S	2	___2
NaN	-1,758	-0,307587	NaN	S	3	___2
1,12449	0,370745	0,516823	0,0338001	S	2	___2
-0,917877	-1,57669	NaN	-1,5701	S	2	___2
NaN	NaN	NaN	NaN	S	2	___2
-0,997957	-1,57669	NaN	-1,5701	S	2	___2
0,783072	1,3791	0,493999	0,225607	S	2	___2
0,783072	1,3791	0,493999	0,225607	S	2	___2
0,227289	0,118502	NaN	0,359859	S	2	___2
0,227289	0,118502	NaN	0,359859	S	2	___2
1,11108	2,23456	-0,319271	1,67285	S	3	___2
1,20278	1,65367	NaN	0,966394	S	3	___2
1,20278	1,65367	NaN	0,966394	S	3	___2
1,93737	2,09712	-1,58429	1,65444	S	2	___2
0,928602	1,41755	NaN	0,927143	S	2	___2
NaN	NaN	NaN	0,927143	S	2	___2
NaN	2,28445	1,18586	1,65949	S	2	___2
2,44688	2,28445	1,18586	1,65949	S	2	___2
2,52751	4,65851	NaN	NaN	S	2	___2
2,52751	4,65851	2,11246	1,70159	S	2	___2
-0,417824	-0,577555	0,101345	-1,4732	S	2	___2
3,05589	2,43441	0,66411	2,3294	S	3	___2
NaN	-0,860565	NaN	NaN	S	3	___2

-1,9249	-1,8928	-0,527361	NaN	S	2	___2
NaN	-0,720224	-1,5087	NaN	S	2	___2
-0,950047	0,015192	NaN	-1,59507	S	3	___2
-0,950047	0,015192	NaN	-1,59507	S	3	___2
NaN	NaN	NaN	NaN	S	3	___2
NaN	NaN	NaN	NaN	S	2	___2
-0,185658	-1,81236	2,68639	2,08222	S	2	___2
NaN	NaN	NaN	NaN	S	2	___2
-0,181841	-1,28602	-0,582613	-0,253519	S	2	___2
-0,181841	-1,28602	-0,582613	-0,253519	S	2	___2
NaN	NaN	NaN	NaN	S	2	___2
NaN	NaN	NaN	NaN	S	3	___2
NaN	-0,560463	0,270012	NaN	S	2	___2
1,19063	1,1619	NaN	0,957375	S	2	___2
1,19063	1,1619	NaN	0,957375	S	2	___2
-0,367966	-0,979464	-0,238247	-1,27493	S	3	___2
-0,367966	-0,979464	-0,238247	-1,27493	S	2	___2
-1,25132	-1,58918	NaN	-2,09418	S	2	___2
NaN	NaN	0,279438	NaN	S	2	___2
NaN	NaN	0,279438	NaN	S	2	___2
-0,342876	3,26689	-0,887417	2,26073	S	2	___2
NaN	NaN	NaN	NaN	S	2	___2
NaN	NaN	NaN	NaN	S	2	___2
NaN	0,451225	NaN	-0,815872	S	3	___2
NaN	0,451225	NaN	-0,815872	S	3	___2
-2,31484	NaN	-2,5441	-3,18933	S	3	___2
-2,31484	NaN	-2,5441	-3,18933	S	3	___2
-2,47912	NaN	2,94335	-1,73825	S	2	___2
-2,47912	NaN	2,94335	-1,73825	S	2	___2
0,843039	-0,244427	NaN	NaN	S	2	___2
NaN	NaN	-0,167585	NaN	S	2	___2
NaN	NaN	-0,167585	NaN	S	2	___2
NaN	NaN	NaN	NaN	S	2	___2
0,484348	0,992895	0,201006	0,479136	S	2	___2

0,484348	0,992895	0,201006	0,479136	S	2	___2
NaN	NaN	NaN	NaN	S	2	___2
NaN	NaN	NaN	NaN	S	2	___2
-1,24244	-1,44544	-1,36365	NaN	S	2	___2
NaN	NaN	NaN	NaN	S	2	___2
NaN	NaN	NaN	NaN	S	2	___2
NaN	NaN	NaN	NaN	S	3	___2
NaN	NaN	NaN	NaN	S	3	___2
-0,974892	-2,96473	NaN	-3,40017	S	2	___2
-0,974892	-2,96473	NaN	-3,40017	S	2	___2
0,278862	1,85232	1,55049	2,03853	S	3	___2
0,278862	1,85232	1,55049	2,03853	S	3	___2
NaN	NaN	NaN	NaN	S	2	___2
NaN	NaN	NaN	NaN	S	2	___2
NaN	NaN	NaN	NaN	S	3	___2
NaN	NaN	NaN	NaN	S	3	___2
NaN	NaN	NaN	NaN	S	3	___2
NaN	NaN	NaN	NaN	S	2	___2
NaN	NaN	NaN	NaN	S	2	___2
NaN	NaN	NaN	NaN	S	2	___2
NaN	NaN	NaN	NaN	S	2	___2
0,547819	0,143419	-2,43272	NaN	S	2	___2
0,547819	0,143419	-2,43272	NaN	S	2	___2
1,34479	1,44923	1,65573	NaN	S	3	___2
1,34479	1,44923	1,65573	0,536657	S	3	___2
NaN	NaN	NaN	NaN	S	2	___2
-2,13068	1,99947	2,07396	0,96867	S	3	___2
-2,13068	1,99947	2,07396	0,96867	S	3	___2
0,0356922	-0,336651	NaN	NaN	S	2	___2
NaN	NaN	NaN	NaN	S	3	___2
NaN	NaN	NaN	NaN	S	3	___2
NaN	-0,744654	0,173574	2,43705	S	2	___2
NaN	NaN	NaN	NaN	S	3	___2
NaN	NaN	NaN	NaN	S	2	___2

-0,404783	0,963541	NaN	-1,99094	S	3	___2
-0,404783	0,963541	NaN	-1,99094	S	3	___2
NaN	0,0403957	NaN	NaN	S	3	___2
NaN	0,0403957	NaN	NaN	S	3	___2
1,26197	1,80246	NaN	0,572493	S	2	___2
1,29849	1,35415	2,70085	-1,35775	S	2	___2
1,29849	1,35415	2,70085	-1,15929	S	3	___2
NaN	0,960117	0,972343	-1,15929	S	3	___2
NaN	NaN	NaN	NaN	S	2	___2
NaN	NaN	NaN	NaN	S	2	___2
NaN	NaN	NaN	-0,452852	S	4	___2
1,29793	2,34851	-0,0445995	0,807367	S	4	___2
1,29793	2,34851	-0,0445995	0,807367	S	4	___2
NaN	NaN	NaN	NaN	S	3	___2
NaN	NaN	NaN	NaN	S	3	___2
1,0977	2,51804	0,69644	3,54922	S	3	___2
1,0977	2,51804	0,69644	3,54922	S	3	___2
NaN	NaN	NaN	NaN	S	3	___2
NaN	NaN	NaN	NaN	S	2	___2
-0,729271	-0,206501	NaN	-0,15378	S	2	___2
-0,729271	-0,206501	NaN	-0,15378	S	2	___2
NaN	NaN	2,16976	NaN	S	3	___2
NaN	NaN	2,16976	NaN	S	3	___2
NaN	0,89505	0,806726	-0,43729	S	3	___2
-0,884232	-1,17834	NaN	-1,54527	S	2	___2
-0,884232	-1,17834	NaN	-1,54527	S	2	___2
2,27742	1,69326	-1,9188	1,91303	S	3	___2
2,27742	1,69326	-1,9188	1,91303	S	3	___2
-2,5779	-1,47562	-1,01002	NaN	S	3	___2
-2,5779	-1,47562	-1,01002	NaN	S	3	___2
-0,304544	0,131292	-1,41641	NaN	S	3	___2
-0,304544	0,131292	-1,41641	NaN	S	3	___2
NaN	-1,75787	NaN	NaN	S	2	___2
NaN	-1,75787	NaN	NaN	S	2	___2

NaN	NaN	NaN	NaN	S	3	___2
NaN	0,398323	NaN	0,945261	S	3	___2
NaN	0,398323	NaN	0,945261	S	3	___2
NaN	NaN	1,45999	-1,44748	S	3	___2
NaN	NaN	1,45999	-1,44748	S	2	___2
NaN	NaN	0,974833	NaN	S	3	___2
NaN	NaN	0,974833	NaN	S	3	___2
1,88204	1,65273	-1,85679	1,91216	S	2	___2
1,88204	1,65273	-1,85679	1,91216	S	2	___2
NaN	NaN	NaN	NaN	S	2	___2
0,182806	0,722055	1,03989	-0,239042	S	2	___2
NaN	NaN	NaN	NaN	S	2	___2
NaN	0,912552	0,679829	0,545271	S	2	___2
NaN	0,912552	0,679829	0,545271	S	2	___2
0,190994	0,065094	-1,12354	0,59796	S	2	___2
0,32548	0,0943546	-0,0647354	NaN	S	2	___2
0,876129	1,04569	0,782846	1,04944	S	3	___2
NaN	NaN	NaN	NaN	S	2	___2
NaN	NaN	NaN	NaN	S	3	___2
NaN	NaN	2,65396	NaN	S	3	___2
NaN	NaN	NaN	NaN	S	2	___2
0,42215	-0,245056	-1,57885	-0,349939	S	2	___2
-0,0810223	-0,245056	-1,57885	-0,349939	S	2	___2
1,75579	1,60437	1,5271	1,45151	S	3	___2
1,75579	1,60437	1,5271	1,45151	S	3	___2
0,173283	0,575596	0,253668	-0,928839	S	2	___2
0,173283	0,575596	0,253668	-0,928839	S	2	___2
0,418316	0,794985	1,9919	0,361246	S	3	___2
-1,01047	-1,01917	-1,29403	-2,14083	S	3	___2
2,52655	2,24855	1,12826	0,189295	S	2	___2
2,52655	2,24855	1,12826	0,189295	S	2	___2
-1,25744	-1,1079	-1,05898	-0,860174	S	2	___2
-0,505951	-1,45158	NaN	-0,427176	S	3	___2
-0,915522	-1,10857	NaN	-1,11113	S	2	___2

NaN	NaN	NaN	NaN	S	2	___2
NaN	NaN	NaN	NaN	S	2	___2
-0,902639	0,0582676	-0,572817	NaN	S	2	___2
-1,3199	NaN	0,933998	-1,97796	S	2	___2
NaN	NaN	NaN	NaN	S	3	___2
-0,874416	-2,75803	-2,50433	-1,83555	S	2	___2
-0,874416	-2,75803	-2,50433	-1,83555	S	2	___2
-2,20263	3,04371	3,81171	NaN	S	3	___2
-2,20263	3,04371	3,81171	NaN	S	3	___2
NaN	NaN	NaN	NaN	S	3	___2
NaN	NaN	NaN	NaN	S	3	___2
0,735128	0,674362	1,59045	0,726292	S	3	___2
NaN	1,09279	0,278307	NaN	S	2	___2
NaN	1,09279	0,278307	NaN	S	2	___2
NaN	-2,18508	NaN	NaN	S	2	___2
NaN	-2,18508	NaN	NaN	S	2	___2
-0,0180244	-1,88088	0,138865	-0,960125	S	3	___2
NaN	NaN	NaN	NaN	S	3	___2
NaN	-0,965513	NaN	-0,291346	S	3	___2
0,162363	-0,790459	-1,66775	-1,93774	S	2	___2
0,162363	-0,790459	-1,66775	-1,93774	S	2	___2
-1,44535	-0,216682	NaN	-2,12542	S	2	___2
-1,44535	-0,216682	NaN	-2,12542	S	2	___2
NaN	NaN	NaN	NaN	S	2	___2
NaN	NaN	NaN	NaN	S	2	___2
-0,0990562	-0,459982	-1,6107	-0,26462	S	3	___2
-0,0990562	-0,459982	-1,6107	-0,26462	S	3	___2
NaN	NaN	NaN	NaN	S	3	___2
NaN	NaN	NaN	NaN	S	3	___2
1,498	2,96037	0,913633	0,924429	S	2	___2
0,947123	1,85566	NaN	NaN	S	2	___2
0,904373	3,11656	3,61837	1,63489	S	2	___2
0,904373	3,11656	3,61837	1,63489	S	2	___2
-1,54182	-0,712379	NaN	NaN	S	2	___2

-1,54182	-0,712379	NaN	NaN	S	2	___2
-0,7444	-0,312635	0,326488	NaN	S	2	___2
-0,7444	-0,312635	0,326488	NaN	S	2	___2
-2,83096	-0,696201	0,586924	1,01785	S	2	___2
-2,83096	-0,696201	0,586924	1,01785	S	2	___2
-0,606026	-2,01286	-1,42808	NaN	S	2	___2
NaN	0,253885	NaN	-1,16311	S	2	___2
NaN	0,253885	NaN	-1,16311	S	2	___2
NaN	NaN	NaN	NaN	S	2	___2
NaN	NaN	NaN	NaN	S	2	___2
0,108297	-0,84034	NaN	-1,65688	S	2	___2
0,108297	-0,84034	NaN	-1,65688	S	2	___2
NaN	NaN	NaN	NaN	S	2	___2
-0,926279	-1,46178	NaN	-1,72479	S	2	___2
NaN	NaN	NaN	NaN	S	2	___2
0,824512	0,0935726	0,964731	-0,678129	S	3	___2
0,824512	0,0935726	0,964731	-0,678129	S	3	___2
0,739016	1,35216	NaN	NaN	S	3	___2
0,739016	1,35216	NaN	NaN	S	3	___2
-0,0531101	1,07808	1,09138	-0,939312	S	2	___2
-0,0531101	1,07808	1,09138	-0,939312	S	2	___2
NaN	NaN	NaN	NaN	S	2	___2
NaN	NaN	NaN	NaN	S	2	___2
NaN	NaN	NaN	NaN	S	3	___2
NaN	NaN	NaN	NaN	S	3	___2
-0,699539	-2,43468	-1,73271	NaN	S	2	___2
-0,699539	-2,43468	-1,73271	NaN	S	2	___2
0,71701	1,10158	1,09889	NaN	S	2	___2
NaN	1,10158	1,09889	NaN	S	2	___2
0,340797	-0,822269	1,29346	NaN	S	3	___2
0,340797	-1,22304	1,29346	NaN	S	3	___2
NaN	NaN	NaN	NaN	S	2	___2
NaN	NaN	NaN	NaN	S	2	___2
NaN	NaN	NaN	NaN	S	4	___2

NaN	NaN	NaN	NaN	S	4	___2
-2,36413	-1,32384	1,06936	-1,13599	S	2	___2
NaN	NaN	NaN	NaN	S	3	___2
-2,15289	NaN	NaN	-2,60359	S	2	___2
-2,15289	NaN	NaN	-2,60359	S	2	___2
-0,702242	0,633808	-1,07399	-0,00103188	S	3	___2
-0,702242	0,633808	-1,07399	-0,00103188	S	3	___2
1,05983	1,44007	0,722204	-0,582037	S	4	___2
0,480902	1,44007	0,722204	-0,582037	S	3	___2
1,05983	1,32047	NaN	-0,738989	S	2	___2
0,992846	0,349546	NaN	-0,208866	S	2	___2
NaN	NaN	NaN	NaN	S	3	___2
0,992846	0,349546	NaN	-0,208866	S	2	___2
NaN	NaN	NaN	NaN	S	2	___2
NaN	NaN	NaN	NaN	S	2	___2
1,6445	-2,6812	0,723192	NaN	S	3	___2
4,26316	3,01305	4,87607	2,93712	S	3	___2
4,26316	3,01305	4,87607	2,93712	S	3	___2
NaN	NaN	1,4349	-0,672705	S	3	___2
-1,22492	NaN	1,4349	-0,672705	S	3	___2
3,14229	2,06636	3,0463	1,49488	S	2	___2
1,19463	-0,239586	-1,09422	0,0839252	S	2	___2
1,19463	-0,239586	-1,09422	0,0839252	S	2	___2
NaN	-0,681175	-0,786255	0,0576973	S	3	___2
0,163322	-0,681175	-0,786255	0,0576973	S	3	___2
-1,85492	-0,222506	-0,670231	NaN	S	3	___2
-1,85492	-0,222506	-0,670231	NaN	S	3	___2
3,11367	3,14186	1,07733	4,23022	S	3	___2
3,11367	3,14186	1,07733	4,23022	S	3	___2
0,872215	0,726826	0,110279	2,43562	S	3	___2
0,872215	0,726826	0,110279	2,43562	S	3	___2
NaN	0,457697	0,906696	0,516476	S	2	___2
NaN	0,457697	0,906696	0,516476	S	2	___2
-0,460953	-0,457689	NaN	-0,709042	S	2	___2

-0,460953	-0,457689	NaN	NaN	S	2	___2
0,0441856	-0,47961	-0,974142	-0,745848	S	3	___2
0,0441856	-0,47961	-0,974142	-0,745848	S	3	___2
NaN	NaN	NaN	NaN	S	2	___2
NaN	NaN	NaN	NaN	S	2	___2
0,337265	-0,947363	NaN	-1,45234	S	2	___2
0,337265	-0,947363	NaN	-1,45234	S	2	___2
NaN	NaN	1,37317	1,34701	S	2	___2
NaN	NaN	1,37317	1,34701	S	2	___2
NaN	NaN	NaN	NaN	S	2	___2
NaN	-0,768858	1,04757	NaN	S	3	___2
NaN	-0,768858	1,04757	NaN	S	3	___2
NaN	NaN	-1,0982	NaN	S	2	___2
NaN	NaN	-1,0982	NaN	S	3	___2
-1,07426	0,0973339	-1,51713	NaN	S	2	___2
-1,07426	0,0973339	-0,154003	-3,19833	S	2	___2
-1,52397	-2,06275	-0,154003	-3,19833	S	2	___2
-1,30999	0,384977	-0,750967	0,87151	S	3	___2
-1,30999	0,384977	-0,750967	0,87151	S	3	___2
NaN	NaN	NaN	NaN	S	3	___2
NaN	NaN	NaN	NaN	S	3	___2
NaN	NaN	NaN	NaN	S	2	___2
NaN	NaN	NaN	NaN	S	2	___2
NaN	NaN	NaN	NaN	S	2	___2
NaN	NaN	NaN	NaN	S	2	___2
-0,899082	-1,87506	NaN	-0,670988	S	3	___2
0,538595	0,294497	0,76296	0,00631142	S	3	___2
0,538595	0,294497	0,76296	0,00631142	S	2	___2
NaN	NaN	1,02602	0,485022	S	3	___2
NaN	NaN	1,02602	0,485022	S	3	___2
-1,20858	-1,01733	-0,26857	NaN	S	2	___2
-1,20858	-1,01733	-0,26857	NaN	S	2	___2
-3,25131	-1,71673	-0,478289	-1,20619	S	2	___2
NaN	-0,729872	-1,0995	NaN	S	2	___2

0,131668	0,931114	NaN	-2,68491	S	2	___2
0,131668	0,931114	NaN	-2,68491	S	2	___2
0,991695	0,289978	-1,53908	NaN	S	3	___2
NaN	NaN	NaN	NaN	S	2	___2
NaN	NaN	NaN	NaN	S	2	___2
NaN	-2,13468	-3,58169	-1,53519	S	3	___2
NaN	NaN	NaN	NaN	S	2	___2
0,389776	0,634785	-1,05404	-0,57773	S	3	___2
0,389776	0,634785	-1,05404	-0,57773	S	2	___2
0,0692348	0,157284	0,395826	-1,29686	S	2	___2
0,0692348	0,157284	0,395826	-1,29686	S	3	___2
0,0425377	0,00857735	-0,543823	-1,80913	S	2	___2
2,02817	1,46902	0,241001	1,66268	S	2	___2
2,02817	1,46902	0,241001	1,66268	S	2	___2
0,859076	0,205341	0,913879	-0,249836	S	3	___2
1,67031	NaN	NaN	NaN	S	2	___2
1,67031	2,26608	1,76433	0,771179	S	2	___2
NaN	NaN	NaN	NaN	S	2	___2
-0,550318	-0,371525	0,649704	-1,255	S	2	___2
-0,550318	-0,371525	0,649704	-1,255	S	2	___2
NaN	NaN	NaN	NaN	S	3	___2
-0,460762	-0,986956	NaN	-1,30268	S	3	___2
-0,460762	-0,986956	NaN	-1,30268	S	3	___2
NaN	-0,398447	-0,534355	-1,35931	S	3	___2
NaN	NaN	NaN	NaN	S	2	___2
NaN	NaN	NaN	NaN	S	2	___2
-0,500256	0,118294	0,0379677	0,385426	S	2	___2
-0,569677	1,37019	1,01351	1,62148	S	3	___2
-2,56101	NaN	-2,19399	NaN	S	2	___2
-2,56101	NaN	-2,19399	NaN	S	2	___2
-1,27017	-1,13983	-0,414114	1,91359	S	2	___2
-1,27017	-1,13983	-0,414114	1,91359	S	2	___2
NaN	NaN	4,19788	1,21044	S	3	___2
NaN	NaN	4,19788	1,21044	S	3	___2

NaN	NaN	NaN	NaN	S	2	___2
NaN	NaN	NaN	NaN	S	2	___2
NaN	NaN	NaN	NaN	S	2	___2
NaN	NaN	NaN	NaN	S	3	___2
NaN	NaN	NaN	NaN	S	3	___2
NaN	NaN	NaN	NaN	S	2	___2
NaN	NaN	NaN	NaN	S	2	___2
NaN	NaN	NaN	NaN	S	2	___2
-0,430841	-0,714741	NaN	-1,12032	S	3	___2
-0,430841	-0,714741	NaN	-1,12032	S	3	___2
NaN	NaN	NaN	NaN	S	2	___2
1,89347	-0,200914	-1,99561	NaN	S	2	___2
1,89347	-0,200914	-1,99561	NaN	S	2	___2
-1,5602	0,0830936	NaN	-0,214834	S	2	___2
-1,5602	0,0830936	NaN	-0,214834	S	2	___2
-0,68738	-0,316877	NaN	-0,971289	S	2	___2
-0,68738	-0,316877	NaN	-0,971289	S	2	___2
-2,68105	0,23604	NaN	-0,178221	S	3	___2
-2,68105	0,23604	NaN	-0,178221	S	3	___2
NaN	NaN	NaN	NaN	S	3	___2
NaN	NaN	NaN	NaN	S	2	___2
NaN	NaN	NaN	NaN	S	2	___2
0,0499191	0,579	3,13163	2,29788	S	2	___2
0,0499191	0,579	3,13163	2,29788	S	2	___2
-1,33591	-1,49318	NaN	NaN	S	2	___2
-0,588154	-0,858641	-0,970581	-1,58625	S	3	___2
NaN	NaN	NaN	NaN	S	2	___2
-0,890642	-0,396749	NaN	0,0894833	S	3	___2
-0,890642	-0,396749	NaN	0,0894833	S	3	___2
0,07901	2,58929	NaN	NaN	S	2	___2
0,07901	2,58929	NaN	NaN	S	2	___2
0,937368	0,291342	-0,472239	0,525145	S	2	___2
0,937368	0,291342	-0,472239	0,525145	S	3	___2
NaN	NaN	NaN	NaN	S	3	___2

NaN	0,0464993	NaN	NaN	S	2	___2
NaN	0,0464993	NaN	NaN	S	2	___2
-0,148201	-0,145287	0,336107	-1,64805	S	2	___2
NaN	NaN	NaN	NaN	S	2	___2
-1,72383	-1,35069	NaN	NaN	S	2	___2
-1,72383	-1,35069	NaN	NaN	S	2	___2
-2,10087	-1,91255	-1,13387	NaN	S	2	___2
-2,10087	-1,91255	-1,13387	NaN	S	2	___2
0,515755	-0,035099	0,475622	-1,46483	S	2	___2
0,515755	-0,035099	0,475622	-1,46483	S	2	___2
-0,489902	-0,557783	NaN	NaN	S	3	___2
NaN	NaN	NaN	NaN	S	3	___2
NaN	NaN	NaN	NaN	S	3	___2
2,40451	2,63783	NaN	0,366999	S	3	___2
2,40451	2,63783	NaN	0,366999	S	3	___2
-0,655323	2,35886	NaN	3,17941	S	4	___2
NaN	2,35886	NaN	3,17941	S	3	___2
NaN	NaN	NaN	NaN	S	2	___2
NaN	NaN	NaN	NaN	S	2	___2
2,45555	3,19877	2,62199	2,45481	S	3	___2
2,45555	3,19877	2,62199	2,45481	S	3	___2
NaN	NaN	NaN	NaN	S	2	___2
NaN	0,0260258	0,0753384	NaN	S	4	___2
-0,636349	-0,966164	NaN	NaN	S	2	___2
0,215103	0,0246849	-2,3821	0,0487118	S	2	___2
-2,12587	-1,65963	NaN	-1,23899	S	2	___2
-2,12587	-1,65963	NaN	-1,23899	S	2	___2
-0,106758	-0,393162	NaN	NaN	S	2	___2
-0,106758	-0,393162	NaN	NaN	S	2	___2
2,98633	1,73934	NaN	1,77159	S	2	___2
2,98633	1,73934	NaN	1,77159	S	3	___2
NaN	-1,38383	0,932413	NaN	S	3	___2
NaN	-1,38383	0,932413	0,190977	S	3	___2
-1,58867	-0,66835	-0,76886	NaN	S	3	___2

-2,36701	NaN	-1,38216	0,389437	S	2	___2
-2,36701	NaN	-1,38216	0,389437	S	2	___2
NaN	NaN	NaN	NaN	S	2	___2
NaN	NaN	NaN	NaN	S	2	___2
NaN	NaN	NaN	NaN	S	3	___2
-1,22484	-0,852331	0,504604	NaN	S	3	___2
-1,22484	-0,852331	0,504604	NaN	S	3	___2
NaN	NaN	-0,1668	-0,68395	S	2	___2
NaN	NaN	-0,1668	-0,68395	S	2	___2
0,258268	1,49843	0,116024	0,0993137	S	3	___2
0,258268	1,49843	NaN	0,0993137	S	3	___2
0,601967	0,759607	NaN	0,278921	S	3	___2
0,601967	0,759607	NaN	0,278921	S	3	___2
NaN	NaN	NaN	NaN	S	2	___2
NaN	NaN	NaN	NaN	S	2	___2
NaN	NaN	NaN	NaN	S	2	___2
-1,05412	-0,162659	-0,917654	-0,969912	S	3	___2
1,25419	0,88088	NaN	0,978453	S	2	___2
-1,4938	-2,50802	NaN	NaN	S	3	___2
-1,4938	-2,50802	NaN	NaN	S	3	___2
0,800821	1,17223	-0,6252	1,29812	S	2	___2
0,800821	1,17223	NaN	1,29812	S	2	___2
NaN	-0,695711	-2,98096	-2,21285	S	3	___2
NaN	NaN	-0,711786	NaN	S	3	___2
-1,57891	-0,900614	-1,52958	-1,72139	S	2	___2
-1,57891	-0,900614	-1,52958	-1,72139	S	2	___2
0,253132	1,33171	-1,97955	-1,83898	S	2	___2
0,253132	1,33171	-1,97955	-1,83898	S	2	___2
NaN	NaN	NaN	NaN	S	2	___2
2,00038	1,14702	2,55628	1,04229	S	3	___2
2,00038	1,14702	2,55628	1,04229	S	2	___2
-0,896627	-0,648729	1,601	0,0530529	S	2	___2
-0,896627	-0,648729	1,601	0,0530529	S	2	___2
-0,962687	NaN	NaN	-0,264421	S	2	___2

Polito et al. Universal ready-to-use immunotherapeutic approach for the treatment of cancer: expanded and activated polyclonal γδ memory T cells.
Phosphorylation sites

-0,962687	NaN	NaN	-0,264421	S	2	___2
-0,65325	-0,852058	-0,0132103	NaN	S	3	___2
-0,65325	-0,852058	-0,0132103	NaN	S	3	___2
NaN	0,106443	0,590361	0,295298	S	3	___2
NaN	0,106443	NaN	0,295298	S	3	___2
1,40005	3,04389	3,572	2,2758	S	2	___2
NaN	NaN	NaN	NaN	S	2	___2
NaN	NaN	NaN	NaN	S	2	___2
0,578051	-0,0840702	-0,14525	-0,613482	S	2	___2
0,854351	1,17957	0,399466	NaN	S	3	___2
0,854351	1,17957	0,399466	NaN	S	2	___2
NaN	NaN	NaN	NaN	S	2	___2
0,185978	-1,12809	NaN	NaN	S	2	___2
0,185978	-1,12809	NaN	NaN	S	3	___2
NaN	1,0336	0,404961	0,411303	S	3	___2
NaN	1,0336	NaN	0,411303	S	3	___2
2,78358	2,51645	2,38965	2,51711	S	2	___2
2,78358	2,51645	2,38965	2,51711	S	2	___2
NaN	NaN	NaN	NaN	S	2	___2
NaN	NaN	NaN	NaN	S	2	___2
NaN	NaN	NaN	NaN	S	2	___2
NaN	NaN	NaN	NaN	S	2	___2
0,327883	-0,200827	-0,145174	NaN	S	2	___2
0,327883	-0,200827	-0,145174	NaN	S	2	___2
1,83611	0,91408	1,02018	-1,87428	S	2	___2
NaN	NaN	-1,2317	NaN	S	2	___2
1,83611	0,91408	1,02018	-1,87428	S	2	___2
-0,979393	-0,511129	-3,17535	-0,903336	S	2	___2
-0,0507755	0,589968	NaN	-0,06213	S	2	___2
-0,0507755	0,589968	NaN	-0,06213	S	2	___2
NaN	NaN	-0,375378	NaN	S	3	___2
NaN	NaN	-0,375378	NaN	S	3	___2
-0,904259	-2,61986	NaN	-1,43456	S	2	___2
-0,904259	-2,61986	NaN	-1,43456	S	2	___2

Polito et al. Universal ready-to-use immunotherapeutic approach for the treatment of cancer: expanded and activated polyclonal γδ memory T cells.
Phosphorylation sites

0,973337	0,283953	NaN	0,373541	S	2	___2
0,973337	0,283953	NaN	0,373541	S	2	___2
-2,89451	-1,22256	NaN	NaN	S	2	___2
-2,89451	-1,22256	NaN	NaN	S	2	___2
NaN	NaN	NaN	NaN	S	2	___2
NaN	NaN	NaN	NaN	S	2	___2
-3,66533	-4,03704	-0,717859	NaN	S	2	___2
2,32977	1,83071	NaN	1,63564	S	3	___2
-1,90582	0,017664	-1,81491	-2,16648	S	2	___2
-1,90582	0,017664	-1,81491	-2,16648	S	2	___2
NaN	NaN	-0,0371056	NaN	S	2	___2
NaN	NaN	-0,0371056	NaN	S	2	___2
1,65895	-0,912121	-0,889614	-0,339024	S	2	___2
1,65895	-0,912121	-0,889614	-0,339024	S	2	___2
-1,07433	NaN	NaN	-1,6557	S	2	___2
-1,07433	NaN	NaN	-1,6557	S	2	___2
NaN	NaN	NaN	NaN	S	3	___2
0,197021	-2,27579	NaN	-2,00591	S	3	___2
0,197021	-2,27579	NaN	-2,00591	S	3	___2
2,67867	2,6868	-0,479912	2,08659	S	3	___2
2,67867	2,6868	-0,479912	2,08659	S	3	___2
0,236759	0,215168	NaN	0,129457	S	3	___2
0,236759	0,215168	NaN	0,129457	S	3	___2
1,86236	1,4423	NaN	1,12452	S	2	___2
1,86236	1,4423	NaN	1,12452	S	2	___2
2,51653	2,14337	0,155029	2,35339	S	3	___2
2,51653	2,14337	0,155029	2,35339	S	2	___2
-3,01213	1,47181	1,19951	0,0699558	S	3	___2
-3,01213	1,47181	1,19951	NaN	S	3	___2
2,78197	2,18195	-1,17238	1,13416	S	2	___2
2,78197	2,18195	-1,17238	1,13416	S	2	___2
0,540882	0,491838	NaN	NaN	S	3	___2
0,286383	-0,596338	-0,573545	-0,116047	S	2	___2
NaN	0,579964	0,732607	-0,811426	S	3	___2

NaN	0,579964	0,732607	-0,811426	S	3	___2
NaN	-0,826698	NaN	NaN	S	2	___2
NaN	-0,826698	NaN	NaN	S	2	___2
0,549974	-0,451561	-1,04816	-0,893858	S	2	___2
0,549974	-0,451561	-1,04816	-0,893858	S	2	___2
-0,0305328	-0,366045	NaN	-0,860897	S	2	___2
-0,0305328	-0,366045	NaN	-0,860897	S	2	___2
NaN	NaN	NaN	NaN	S	2	___2
2,25109	1,91068	1,53596	-0,585831	S	2	___2
2,25109	1,91068	1,53596	-0,585831	S	2	___2
1,42685	2,24286	0,693277	-0,239098	S	3	___2
1,42685	2,24286	0,693277	-0,239098	S	3	___2
NaN	0,206327	NaN	NaN	S	4	___2
-1,57705	0,268494	NaN	0,576521	S	2	___2
-1,57705	0,268494	NaN	0,576521	S	2	___2
-0,252106	-0,458523	NaN	-0,145309	S	2	___2
-0,252106	-0,458523	NaN	-0,145309	S	2	___2
-2,38528	0,290598	NaN	NaN	S	2	___2
-2,38528	0,290598	NaN	NaN	S	2	___2
NaN	NaN	NaN	NaN	S	2	___2
NaN	NaN	NaN	NaN	S	2	___2
0,983114	-0,386904	-2,0583	NaN	S	3	___2
0,983114	-0,386904	-2,0583	-0,581581	S	3	___2
-0,666332	2,03099	1,23118	1,40406	S	3	___2
-0,666332	2,03099	1,23118	1,40406	S	2	___2
1,26731	0,826313	-1,33971	NaN	S	3	___2
1,26731	0,826313	-1,33971	NaN	S	3	___2
NaN	NaN	NaN	NaN	S	2	___2
NaN	NaN	NaN	NaN	S	3	___2
-0,921942	-0,493309	0,128107	-1,45548	S	2	___2
-0,921942	-0,493309	0,128107	-1,45548	S	2	___2
-0,872068	-1,46107	-0,495281	0,100887	S	3	___2
-0,872068	-1,46107	-0,495281	0,100887	S	3	___2
NaN	-0,823208	-0,058567	-1,20295	S	2	___2

NaN	-0,823208	-0,058567	-1,20295	S	4	___2
NaN	0,130461	NaN	-0,830835	S	3	___2
NaN	0,130461	NaN	-0,830835	S	3	___2
2,9317	2,22313	1,99085	0,751554	S	2	___2
-0,881996	-1,73637	NaN	-2,17153	S	3	___2
-1,99057	-2,39607	NaN	NaN	S	2	___2
0,397293	-1,13381	NaN	-0,533422	S	2	___2
0,397293	-1,13381	NaN	-0,533422	S	2	___2
-0,23382	-1,27903	NaN	-2,27408	S	2	___2
-0,23382	-1,27903	NaN	-2,27408	S	2	___2
NaN	NaN	NaN	NaN	S	2	___2
0,316408	-1,55587	-1,63536	-1,91734	S	2	___2
NaN	NaN	NaN	NaN	S	2	___2
-0,129206	0,917213	-0,7747	-0,246693	S	3	___2
-0,129206	0,917213	-0,7747	-0,246693	S	3	___2
0,0781574	0,0412788	NaN	NaN	S	2	___2
0,0781574	0,0412788	NaN	NaN	S	3	___2
-1,78245	-2,04321	-0,834703	NaN	S	2	___2
-1,78245	-2,04321	-0,834703	NaN	S	2	___2
1,07265	0,294249	0,0956287	-0,137455	S	2	___2
1,07265	0,294249	0,0956287	NaN	S	2	___2
-1,60819	-0,140015	0,922869	-1,01715	S	2	___2
-1,60819	-0,140015	0,922869	-1,01715	S	2	___2
-0,829517	1,63883	-1,14808	0,535137	S	2	___2
-0,829517	1,63883	-1,14808	0,535137	S	3	___2
-2,15505	-1,69284	NaN	NaN	S	2	___2
-2,15505	-1,69284	NaN	NaN	S	2	___2
1,78122	1,78752	1,19453	0,826118	S	2	___2
1,78122	1,78752	1,19453	0,826118	S	2	___2
1,60199	1,22012	NaN	1,63202	S	4	___2
1,60199	1,22012	NaN	1,63202	S	4	___2
NaN	NaN	NaN	NaN	S	2	___2
NaN	NaN	NaN	NaN	S	2	___2
1,4765	-0,154284	0,958038	-0,516436	S	2	___2

1,4765	-0,154284	0,958038	-0,516436	S	2	___2
NaN	NaN	NaN	NaN	S	2	___2
NaN	NaN	NaN	NaN	S	2	___2
1,02871	-0,580385	-0,280788	-1,25763	S	2	___2
1,02871	-0,580385	-0,280788	-1,25763	S	2	___2
2,87105	3,55068	1,512	2,0961	S	2	___2
2,87105	3,55068	1,512	2,0961	S	3	___2
1,68126	0,725727	1,02956	-0,780476	S	2	___2
-0,7444	2,6192	0,407331	0,925047	S	3	___2
-0,7444	2,6192	0,407331	0,925047	S	3	___2
-1,74574	NaN	NaN	NaN	S	2	___2
-1,03937	-0,375944	NaN	-0,26004	S	2	___2
NaN	3,28426	-0,9382	2,68011	S	3	___2
NaN	3,28426	-0,9382	2,68011	S	2	___2
NaN	NaN	NaN	NaN	S	3	___2
NaN	NaN	NaN	NaN	S	3	___2
-1,21822	-0,703094	0,140533	-0,977732	S	2	___2
-1,21822	-0,703094	0,140533	-0,977732	S	2	___2
-2,57072	NaN	0,387772	-2,09478	S	3	___2
-2,57072	NaN	0,387772	-2,09478	S	3	___2
NaN	NaN	NaN	NaN	S	3	___2
NaN	NaN	NaN	NaN	S	3	___2
1,74022	1,95156	1,11789	1,65526	S	4	___2
0,197113	-1,7138	-1,37779	NaN	S	4	___2
1,74022	1,95156	1,11789	1,65526	S	3	___2
-0,0607147	0,929165	1,50357	1,18037	S	3	___2
NaN	NaN	NaN	NaN	S	4	___2
0,410257	-0,578913	-1,92382	-2,34348	S	2	___2
-1,64467	-0,608074	-1,92382	-3,23582	S	2	___2
0,410257	-0,578913	NaN	-2,34348	S	2	___2
NaN	NaN	NaN	NaN	S	2	___2
NaN	NaN	NaN	NaN	S	2	___2
NaN	NaN	NaN	NaN	S	2	___2
1,02846	0,0584126	-2,09432	2,34179	S	2	___2

1,02846	0,0584126	-2,09432	2,34179	S	2	___2
-2,58082	-2,25213	-1,15036	NaN	S	2	___2
-2,58082	-2,25213	-1,15036	NaN	S	2	___2
-1,49849	-1,26364	2,40221	-1,17618	S	2	___2
-0,614912	-1,11767	2,40221	-1,17618	S	2	___2
NaN	NaN	NaN	NaN	S	3	___2
NaN	NaN	NaN	NaN	S	3	___2
NaN	NaN	-0,941011	NaN	S	2	___2
NaN	NaN	-0,941011	NaN	S	2	___2
3,63825	2,59733	4,47342	3,13413	S	2	___2
3,63825	2,59733	4,47342	3,13413	S	2	___2
NaN	NaN	NaN	NaN	S	2	___2
-1,16854	-1,64612	-1,21572	NaN	S	2	___2
-1,16854	-1,64612	-1,21572	NaN	S	2	___2
-3,02679	-3,07627	-3,19676	-1,57854	S	2	___2
-3,02679	-3,07627	-3,19676	-1,57854	S	2	___2
-1,93176	-0,459044	-1,0999	-1,36715	S	2	___2
-1,93176	-0,459044	-1,0999	NaN	S	2	___2
0,115641	-1,37196	NaN	-1,13702	S	3	___2
-1,36025	-0,406963	NaN	0,26622	S	3	___2
2,54726	2,66689	0,581196	2,49475	S	2	___2
NaN	NaN	NaN	NaN	S	2	___2
NaN	NaN	NaN	NaN	S	2	___2
NaN	NaN	NaN	NaN	S	2	___2
NaN	NaN	NaN	NaN	S	2	___2
-0,626686	-1,40427	NaN	-0,611441	S	3	___2
-0,626686	-1,40427	NaN	-0,611441	S	3	___2
NaN	0,367399	-1,27588	-1,13867	S	3	___2
NaN	0,367399	-1,27588	-1,13867	S	3	___2
NaN	NaN	NaN	NaN	S	2	___2
-1,27151	NaN	NaN	NaN	S	3	___2
-1,27151	-1,58786	-1,31021	-0,11615	S	2	___2
NaN	NaN	NaN	NaN	S	2	___2
NaN	NaN	NaN	NaN	S	3	___2

NaN	NaN	NaN	NaN	S	2	___2
NaN	NaN	NaN	NaN	S	3	___2
-0,462336	-0,133099	0,876591	-0,107845	S	3	___2
NaN	NaN	0,663876	NaN	S	2	___2
-0,259594	1,59457	NaN	0,0163269	S	2	___2
-0,259594	1,59457	NaN	0,0163269	S	2	___2
NaN	NaN	NaN	NaN	S	2	___2
NaN	1,03123	NaN	-0,700642	S	2	___2
NaN	NaN	NaN	NaN	S	3	___2
NaN	NaN	NaN	NaN	S	2	___2
NaN	0,0639343	NaN	0,918493	S	2	___2
NaN	0,0639343	NaN	0,918493	S	2	___2
0,427183	3,05898	2,6258	3,06936	S	3	___2
NaN	NaN	NaN	3,06936	S	3	___2
NaN	NaN	NaN	NaN	S	2	___2
NaN	NaN	NaN	NaN	S	2	___2
2,29729	2,2414	0,199203	2,17716	S	2	___2
2,29729	2,2414	0,199203	2,17716	S	2	___2
-0,353151	0,6297	NaN	0,479706	S	3	___2
-0,353151	0,6297	NaN	0,479706	S	3	___2
NaN	0,489735	0,629099	1,5271	S	3	___2
NaN	0,489735	0,629099	1,5271	S	3	___2
NaN	NaN	NaN	NaN	S	2	___2
NaN	NaN	NaN	NaN	S	2	___2
1,01744	0,294436	-0,614969	-0,602165	S	3	___2
1,01744	0,294436	-0,614969	-0,602165	S	3	___2
0,403515	-0,000947952	0,799791	0,177433	S	2	___2
0,403515	-0,000947952	0,799791	0,177433	S	3	___2
0,0947018	2,57409	0,947336	1,39135	S	3	___2
0,0947018	2,57409	0,947336	1,39135	S	3	___2
NaN	NaN	-0,605518	NaN	S	2	___2
NaN	NaN	-0,605518	NaN	S	2	___2
NaN	-0,610966	NaN	-1,19119	S	3	___2
NaN	-0,610966	NaN	-1,19119	S	2	___2

-1,10501	-1,42051	0,168386	-1,35943	S	2	___2
-1,10501	-1,42051	0,168386	-1,35943	S	2	___2
1,75179	1,50786	NaN	1,82301	S	3	___2
1,75179	1,50786	1,93382	1,82301	S	3	___2
-1,61964	-2,34466	-2,20061	-1,52261	S	2	___2
-1,61964	-2,34466	-2,20061	-1,52261	S	2	___2
-1,0785	-2,02219	NaN	-1,08595	S	2	___2
-1,0785	-2,02219	NaN	-1,08595	S	2	___2
-1,22947	-1,93272	0,0583839	-1,47339	S	2	___2
-1,22947	-1,93272	0,0583839	-1,47339	S	2	___2
-1,29189	-1,68998	0,381193	-0,69589	S	2	___2
4,34339	3,8991	3,30236	3,04354	S	3	___2
4,34339	3,8991	3,30236	3,04354	S	3	___2
-0,224033	-0,361071	NaN	-0,764908	S	2	___2
1,61822	1,16663	-0,664576	NaN	S	2	___2
0,774611	-0,174841	-1,17012	-0,0491543	S	3	___2
0,774611	NaN	-1,17012	-0,0491543	S	2	___2
-0,606976	-0,198738	-2,36377	NaN	S	2	___2
-0,606976	-0,198738	-2,36377	NaN	S	2	___2
NaN	NaN	NaN	NaN	S	3	___2
NaN	NaN	NaN	NaN	S	2	___2
NaN	NaN	NaN	NaN	S	2	___2
NaN	NaN	-0,525215	NaN	S	2	___2
NaN	NaN	-0,525215	NaN	S	2	___2
NaN	NaN	NaN	NaN	S	4	___2
NaN	NaN	NaN	NaN	S	4	___2
-2,05117	-1,3472	NaN	-0,0525265	S	2	___2
-2,05117	-1,3472	NaN	-0,0525265	S	2	___2
-0,0906925	0,321859	-0,827044	-1,05842	S	2	___2
NaN	NaN	NaN	NaN	S	2	___2
-1,58826	-1,40815	NaN	-1,1988	S	3	___2
NaN	NaN	NaN	NaN	S	3	___2
NaN	NaN	NaN	NaN	S	3	___2
-2,61463	-1,08142	1,1095	-0,990753	S	2	___2

Polito et al. Universal ready-to-use immunotherapeutic approach for the treatment of cancer: expanded and activated polyclonal γδ memory T cells.
Phosphorylation sites

NaN	NaN	NaN	NaN	S	2		___2
NaN	NaN	NaN	NaN	S	3		___2
NaN	-1,03307	-0,440245	-0,683498	S	3		___2
NaN	-1,03307	-0,440245	-0,683498	S	4		___2
NaN	-1,03307	-0,440245	-0,683498	S	3		___2
0,613993	-0,401146	-1,84353	-1,56074	S	3		___2
0,613993	NaN	-1,84353	-1,56074	S	3		___2
-0,455051	NaN	NaN	-3,83542	S	3		___2
-0,455051	NaN	NaN	-3,83542	S	3		___2
NaN	NaN	NaN	NaN	S	3		___2
NaN	NaN	NaN	NaN	S	3		___2
-0,0161324	2,42879	2,88723	1,58774	S	3		___2
-0,0161324	2,42879	2,88723	1,58774	S	2		___2
2,18999	1,91623	NaN	0,510668	S	2		___2
2,18999	1,91623	NaN	0,510668	S	2		___2
1,31683	2,56669	4,45635	2,81631	S	3		___2
1,31683	2,56669	4,45635	2,81631	S	3		___2
NaN	-1,26594	-2,26269	-1,82555	S	2		___2
NaN	-1,26594	-2,26269	-1,82555	S	2		___2
1,63036	3,33967	2,80789	2,65094	S	3		___2
1,63036	3,33967	2,80789	2,65094	S	2		___2
0,295528	0,689621	0,915731	-0,00650406	S	3		___2
0,295528	0,689621	0,915731	-0,00650406	S	3		___2
0,090023	0,161016	-0,468531	-0,10346	S	2		___2
0,090023	0,161016	-0,468531	-0,10346	S	2		___2
NaN	NaN	NaN	NaN	S	2		___2
NaN	NaN	NaN	NaN	S	2		___2
0,150707	0,156876	0,921764	0,0488243	S	2		___2
-1,00858	-1,19174	-3,96105	-2,77544	S	2		___2
-1,00858	-1,19174	-3,96105	-2,77544	S	2		___2
1,07806	0,722652	NaN	-0,787979	S	2		___2
1,07806	0,722652	NaN	-0,787979	S	2		___2
-1,10419	NaN	NaN	NaN	S	2		___2
NaN	NaN	NaN	NaN	S	2		___2

Polito et al. Universal ready-to-use immunotherapeutic approach for the treatment of cancer: expanded and activated polyclonal γδ memory T cells.
Phosphorylation sites

0,73267	-0,60692	0,797651	-0,446009	S	2	___2
0,73267	-0,60692	0,797651	-0,446009	S	2	___2
NaN	-1,75204	NaN	NaN	S	2	___2
NaN	-1,75204	NaN	NaN	S	2	___2
-1,05628	-0,620838	NaN	NaN	S	3	___2
-1,05628	-0,620838	NaN	NaN	S	3	___2
NaN	-0,525568	NaN	NaN	S	3	___2
NaN	-0,525568	NaN	NaN	S	3	___2
-0,0441875	-0,827236	-0,255508	1,18512	S	2	___2
-0,0441875	-0,827236	-0,255508	1,18512	S	2	___2
-0,8139	0,297028	NaN	-0,985828	S	2	___2
-0,8139	0,297028	NaN	-0,985828	S	2	___2
NaN	-1,34951	NaN	NaN	S	2	___2
NaN	-1,34951	NaN	NaN	S	3	___2
-1,71379	-2,06744	-0,273485	NaN	S	2	___2
-1,71379	-2,06744	-0,273485	NaN	S	2	___2
-0,874798	-0,834381	NaN	NaN	S	2	___2
NaN	NaN	NaN	NaN	S	2	___2
NaN	NaN	NaN	NaN	S	2	___2
-0,402081	-0,869677	NaN	-0,64724	S	2	___2
-0,402081	-0,869677	-0,416039	-0,64724	S	2	___2
NaN	NaN	NaN	NaN	S	2	___2
NaN	NaN	NaN	NaN	S	2	___2
-0,246628	0,437019	NaN	NaN	S	2	___2
NaN	NaN	NaN	NaN	S	2	___2
NaN	NaN	NaN	NaN	S	2	___2
NaN	NaN	NaN	NaN	S	2	___2
0,842844	0,544235	-0,269495	0,291819	S	2	___2
0,842844	0,544235	-0,269495	0,291819	S	2	___2
NaN	NaN	NaN	NaN	S	3	___2
NaN	NaN	NaN	NaN	S	2	___2
-0,574158	-1,96995	0,410259	NaN	S	2	___2
-0,574158	-1,96995	0,410259	NaN	S	2	___2
0,970934	-1,0219	1,97282	NaN	S	2	___2

0,970934	-1,0219	1,97282	NaN	S	2	___2
NaN	2,11866	NaN	0,722464	S	2	___2
0,0301723	-0,233246	-1,23855	-1,74326	S	3	___2
NaN	2,11866	1,46691	0,722464	S	2	___2
NaN	NaN	0,522001	NaN	S	2	___2
NaN	NaN	0,522001	NaN	S	2	___2
-1,71447	1,14174	NaN	0,272167	S	3	___2
NaN	NaN	NaN	NaN	S	4	___2
NaN	-1,92519	-1,93241	-1,53553	S	2	___2
NaN	NaN	NaN	NaN	S	2	___2
NaN	NaN	NaN	NaN	S	2	___2
NaN	NaN	NaN	NaN	S	2	___2
NaN	NaN	NaN	NaN	S	2	___2
1,59249	1,93187	0,961746	0,979166	S	2	___2
-0,349396	-0,529173	NaN	-2,57833	S	2	___2
NaN	NaN	NaN	NaN	S	3	___2
NaN	NaN	NaN	NaN	S	3	___2
0,926088	-0,944195	-1,64741	-0,934111	S	3	___2
0,926088	-0,944195	-1,64741	-0,934111	S	3	___2
1,81509	1,61824	-1,68366	0,422897	S	2	___2
1,81509	1,61824	-1,68366	0,422897	S	2	___2
-1,23012	0,452999	0,760077	0,449062	S	3	___2
-1,23012	0,452999	0,760077	0,449062	S	2	___2
0,881159	1,86688	NaN	0,883114	S	2	___2
0,881159	1,86688	NaN	0,883114	S	2	___2
1,27663	1,09599	-1,00969	0,576912	S	2	___2
NaN	NaN	NaN	NaN	S	3	___2
NaN	NaN	NaN	NaN	S	3	___2
NaN	NaN	NaN	NaN	S	2	___2
NaN	NaN	NaN	NaN	S	3	___2
-0,7752	NaN	NaN	-0,936262	S	2	___2
-0,7752	NaN	NaN	-0,936262	S	2	___2
-1,94918	-1,23524	-0,883718	NaN	S	2	___2
-1,94918	-1,23524	-0,883718	NaN	S	2	___2

NaN	NaN	NaN	NaN	S	2	___2
NaN	NaN	NaN	NaN	S	2	___2
-0,575655	1,67006	-2,85396	-2,68327	S	2	___2
-0,0681057	-0,329775	0,673685	0,0382786	S	3	___2
-0,0681057	-0,329775	0,673685	NaN	S	3	___2
NaN	NaN	NaN	NaN	S	3	___2
NaN	NaN	NaN	NaN	S	3	___2
-0,912062	0,815802	-0,0669289	-1,96922	S	2	___2
-0,912062	0,815802	-0,0669289	-1,96922	S	2	___2
-1,01592	NaN	NaN	NaN	S	3	___2
0,3916	NaN	NaN	-1,08724	S	2	___2
0,3916	-0,145454	NaN	-1,08724	S	2	___2
-1,37878	-0,987089	-0,63341	NaN	S	3	___2
NaN	NaN	NaN	NaN	S	2	___2
1,77485	1,31255	NaN	2,00326	S	2	___2
1,77485	1,31255	NaN	2,00326	S	2	___2
-0,0295429	0,42465	-2,04518	-1,69566	S	2	___2
-0,0295429	0,42465	-2,04518	-1,69566	S	2	___2
NaN	1,46658	0,52968	2,02438	S	4	___2
NaN	1,46658	0,52968	2,02438	S	3	___2
NaN	NaN	0,52968	2,02438	S	3	___2
NaN	-0,655519	NaN	NaN	S	2	___2
NaN	-0,655519	NaN	NaN	S	2	___2
NaN	NaN	3,12092	-0,865498	S	3	___2
NaN	NaN	3,12092	-0,865498	S	2	___2
NaN	NaN	NaN	NaN	S	3	___2
1,0027	-0,330729	-0,898134	0,0855598	S	2	___2
1,0027	-0,330729	-0,898134	0,0855598	S	2	___2
-1,17674	-1,03896	-1,21631	NaN	S	2	___2
0,645432	1,80959	0,9566	1,10386	S	2	___2
2,54091	2,88306	1,54468	2,81578	S	3	___2
1,1385	0,504877	1,55485	0,85425	S	2	___2
1,1385	0,504877	1,55485	0,85425	S	2	___2
0,997625	0,447229	-0,17692	0,17453	S	3	___2

Polito et al. Universal ready-to-use immunotherapeutic approach for the treatment of cancer: expanded and activated polyclonal γδ memory T cells.
Phosphorylation sites

1,68977	2,27099	2,31612	1,09588	S	3	___2
-0,0718517	-0,965971	-0,862448	-0,90601	S	3	___2
1,68977	2,27099	2,31612	1,09588	S	2	___2
NaN	-1,22709	NaN	NaN	S	2	___2
NaN	-1,22709	NaN	NaN	S	2	___2
1,13244	NaN	-2,40546	1,86843	S	2	___2
2,7295	3,99153	-1,37068	2,50618	S	2	___2
2,7295	3,99153	-1,37068	2,50618	S	2	___2
NaN	NaN	NaN	NaN	S	3	___2
NaN	NaN	NaN	NaN	S	3	___2
-1,92056	-2,04268	NaN	-1,18569	S	3	___2
-1,92056	-2,04268	NaN	-1,18569	S	3	___2
4,25942	3,4822	3,08035	3,92988	S	3	___2
4,25942	3,4822	3,08035	3,92988	S	3	___2
-0,932549	1,22758	-2,10399	NaN	S	2	___2
-3,42596	-0,824146	0,7819	-1,08714	S	3	___2
-3,42596	-0,824146	0,7819	-1,08714	S	2	___2
-0,688272	0,537462	0,320387	0,502836	S	2	___2
-0,688272	0,537462	0,320387	0,502836	S	3	___2
1,04917	2,24841	-1,33577	1,38464	S	3	___2
NaN	1,1395	1,67174	0,765127	S	2	___2
-1,71481	-2,1289	NaN	-2,44352	S	3	___2
NaN	NaN	NaN	NaN	S	2	___2
0,254932	-0,153524	-0,583067	-0,9849	S	2	___2
0,254932	NaN	-0,583067	-0,9849	S	2	___2
NaN	NaN	NaN	NaN	S	2	___2
3,45571	2,06929	2,98389	2,36235	S	2	___2
NaN	NaN	NaN	NaN	S	3	___2
3,45571	2,06929	2,98389	2,36235	S	2	___2
3,24512	3,04012	2,96416	2,87391	S	2	___2
3,24512	3,04012	2,96416	2,87391	S	2	___2
-1,21106	NaN	-2,42933	-2,13167	S	2	___2
-1,21106	NaN	-2,42933	-2,13167	S	2	___2
NaN	NaN	NaN	NaN	S	2	___2

NaN	NaN	0,200054	NaN	S	3	___2
NaN	NaN	0,200054	NaN	S	3	___2
NaN	NaN	NaN	NaN	S	2	___2
NaN	NaN	NaN	NaN	S	2	___2
-1,18341	0,0706615	-1,38252	NaN	S	3	___2
-1,18341	-1,68492	-1,38252	NaN	S	2	___2
0,717369	0,86636	-0,970079	0,0814476	S	2	___2
-0,0249519	-0,640909	NaN	-1,03927	S	2	___2
-0,0249519	-0,640909	NaN	-1,03927	S	2	___2
2,5586	1,99049	-0,00270271	1,90066	S	2	___2
2,5586	1,99049	-0,00270271	1,90066	S	2	___2
4,58526	5,28978	2,40185	4,65208	S	2	___2
4,58526	5,28978	2,40185	4,65208	S	2	___2
-0,839153	-1,65805	-1,69432	-1,40853	S	2	___2
-0,839153	-1,65805	-1,69432	-1,40853	S	2	___2
NaN	NaN	NaN	NaN	S	2	___2
3,11459	4,36718	-0,691948	3,72177	S	2	___2
1,03637	1,29357	0,413425	0,725725	S	2	___2
NaN	1,29357	0,413425	0,725725	S	3	___2
4,00826	3,81134	-0,858307	3,09856	S	2	___2
0,818964	-0,794922	-2,45769	0,253778	S	2	___2
4,86913	4,08543	-0,691799	3,99202	S	2	___2
3,57372	3,16689	NaN	2,52596	S	2	___2
3,57372	3,16689	NaN	2,52596	S	2	___2
2,83091	2,25393	NaN	1,60594	S	2	___2
2,83091	2,25393	NaN	1,60594	S	2	___2
0,855309	-0,686289	-3,53427	-0,261387	S	2	___2
0,855309	-0,686289	-3,53427	-0,261387	S	2	___2
0,577772	-1,06313	0,429033	-1,74726	S	2	___2
0,577772	-1,06313	0,429033	-1,74726	S	2	___2
0,772787	-1,85132	-0,611374	-0,562603	S	3	___2
4,07801	5,25258	5,28379	5,01557	S	3	___2
4,07801	5,25258	5,28379	5,01557	S	2	___2
-0,422831	-0,431387	NaN	-0,0881157	S	3	___2

Polito et al. Universal ready-to-use immunotherapeutic approach for the treatment of cancer: expanded and activated polyclonal γδ memory T cells.
Phosphorylation sites

-0,422831	-0,431387	NaN	-0,0881157	S	2	___2
NaN	NaN	NaN	NaN	S	2	___2
NaN	NaN	NaN	NaN	S	2	___2
NaN	NaN	NaN	NaN	S	2	___2
1,16836	1,39242	-1,68576	0,376844	S	2	___2
1,16836	1,39242	-1,68576	0,376844	S	2	___2
-0,874735	-1,38859	0,656954	NaN	S	3	___2
-0,874735	-1,38859	0,656954	NaN	S	3	___2
NaN	NaN	NaN	NaN	S	3	___2
-1,55866	1,19313	NaN	-0,304564	S	3	___2
-1,55866	1,19313	NaN	-0,304564	S	2	___2
NaN	NaN	NaN	NaN	S	2	___2
NaN	NaN	NaN	NaN	S	2	___2
5,87735	4,5767	NaN	3,73569	S	3	___2
5,87735	4,5767	NaN	3,73569	S	3	___2
-0,314636	4,17978	0,949387	3,01223	S	3	___2
-0,314636	4,17978	0,949387	3,01223	S	3	___2
-0,590343	1,07768	NaN	0,444317	S	3	___2
-0,590343	1,07768	NaN	0,444317	S	3	___2
-1,04465	-2,11784	-1,94544	-0,702394	S	2	___2
-1,04465	-2,11784	-1,94544	-0,702394	S	2	___2
2,43032	3,30826	2,07086	2,89125	S	2	___2
2,43032	3,30826	2,07086	2,89125	S	2	___2
3,06076	2,48457	NaN	2,19824	S	2	___2
3,06076	2,48457	NaN	2,19824	S	3	___2
0,623558	0,172428	0,114391	NaN	S	3	___2
0,623558	0,172428	0,114391	NaN	S	3	___2
NaN	NaN	NaN	0,184273	S	2	___2
1,68827	0,392099	0,557985	NaN	S	2	___2
0,38599	0,865404	0,325745	NaN	S	3	___2
0,967253	1,73555	0,121468	NaN	S	4	___2
1,28576	0,867357	0,103773	1,09467	S	3	___2
1,28576	0,867357	0,103773	1,09467	S	3	___2
-1,66068	NaN	NaN	NaN	S	2	___2

NaN	NaN	NaN	NaN	S	2	___2
NaN	1,16741	0,0101585	-0,60619	S	3	___2
NaN	NaN	NaN	NaN	S	3	___2
0,922199	1,16741	0,0101585	-0,60619	S	2	___2
-2,83224	-1,97804	NaN	-1,91442	S	2	___2
-0,617409	-0,0442924	NaN	-0,943316	S	3	___2
-0,617409	-0,0442924	NaN	-0,943316	S	3	___2
NaN	NaN	NaN	NaN	S	2	___2
NaN	NaN	NaN	NaN	S	2	___2
-1,37761	NaN	NaN	NaN	S	2	___2
NaN	NaN	NaN	NaN	S	1	___2
NaN	-2,41119	-1,13617	NaN	S	3	___2
NaN	NaN	NaN	NaN	S	2	___2
NaN	NaN	NaN	NaN	S	2	___2
NaN	NaN	NaN	NaN	S	2	___2
NaN	NaN	NaN	NaN	S	2	___2
NaN	NaN	NaN	NaN	S	2	___2
NaN	NaN	NaN	NaN	S	2	___2
NaN	1,63173	1,61665	1,33545	S	3	___2
NaN	1,63173	1,61665	1,33545	S	3	___2
NaN	NaN	NaN	NaN	S	2	___2
NaN	NaN	NaN	NaN	S	2	___2
NaN	0,779659	-2,04441	-0,216003	S	4	___2
-1,02266	-1,10272	-1,17337	-0,241423	S	2	___2
-1,02266	-1,10272	-1,17337	-0,241423	S	2	___2
NaN	NaN	-1,32209	NaN	S	2	___2
NaN	NaN	-1,32209	NaN	S	2	___2
0,799866	0,589869	-0,945353	0,0975628	S	3	___2
0,799866	0,589869	-0,945353	0,0975628	S	2	___2
1,06286	2,48944	2,10767	1,33489	S	3	___2
-1,0995	-1,35518	-1,78325	-1,98342	S	2	___2
-1,0995	-1,35518	-1,78325	-1,98342	S	2	___2
NaN	NaN	NaN	NaN	S	2	___2
NaN	NaN	NaN	NaN	S	2	___2

NaN	NaN	NaN	NaN	S	3	___2
NaN	NaN	NaN	NaN	S	2	___2
NaN	NaN	NaN	NaN	S	2	___2
NaN	NaN	NaN	NaN	S	3	___2
NaN	-1,1874	1,17961	-1,75459	S	3	___2
NaN	-1,1874	1,17961	-1,75459	S	3	___2
NaN	-1,57119	NaN	NaN	S	2	___2
NaN	-1,57119	NaN	NaN	S	2	___2
NaN	-0,115257	0,361637	NaN	S	4	___2
NaN	-0,115257	0,361637	NaN	S	4	___2
NaN	NaN	NaN	NaN	S	2	___2
NaN	NaN	NaN	NaN	S	2	___2
NaN	NaN	NaN	NaN	S	2	___2
NaN	NaN	NaN	NaN	S	2	___2
0,245115	2,64427	NaN	NaN	S	3	___2
0,245115	2,64427	NaN	NaN	S	3	___2
NaN	-1,38383	0,932413	NaN	S	3	___2
NaN	-1,38383	0,932413	0,190977	S	3	___2
-1,58867	-0,66835	-0,76886	NaN	S	3	___2
-0,757519	NaN	0,583216	-1,43526	T	2	___2
-0,337788	-2,55923	NaN	0,416893	T	2	___2
NaN	1,25229	NaN	2,15309	T	2	___2
NaN	3,89223	2,9166	2,24978	T	3	___2
0,522621	3,89223	2,9166	2,24978	T	3	___2
-0,132696	1,40065	-0,0739422	-1,20786	T	3	___2
-0,624548	0,545118	NaN	1,00133	T	3	___2
-0,719597	-1,40726	-1,58324	-0,117496	T	2	___2
0,112438	0,580065	NaN	NaN	T	3	___2
-1,772	-1,59797	-2,70987	NaN	T	2	___2
-0,217976	-0,765892	1,95378	0,128664	T	2	___2
2,2319	0,0883064	2,037	-0,686737	T	2	___2
NaN	NaN	NaN	NaN	T	3	___2
NaN	NaN	NaN	NaN	T	2	___2
NaN	-1,7828	NaN	NaN	T	2	___2

-2,31381	NaN	-1,18566	NaN	T	2	___2
4,87599	4,89383	0,435812	2,59031	T	2	___2
1,91485	1,7597	3,42957	1,76554	T	2	___2
0,339539	0,659277	-0,310268	-1,45774	T	2	___2
NaN	NaN	NaN	NaN	T	3	___2
NaN	NaN	NaN	NaN	T	2	___2
1,34969	-0,0812607	NaN	1,2009	T	2	___2
0,237024	0,86532	NaN	-0,391678	T	2	___2
NaN	-0,319807	-3,41325	NaN	T	2	___2
-2,4667	2,9751	-1,78141	NaN	T	2	___2
3,98151	5,13891	4,02439	3,83476	T	3	___2
NaN	NaN	NaN	NaN	T	2	___2
-3,18093	-0,0354881	2,63501	3,89146	T	2	___2
NaN	2,54789	-0,880116	1,46175	T	3	___2
-1,16916	-1,21526	-1,03871	-0,687567	T	2	___2
-1,16916	-1,21526	-1,03871	-0,687567	T	2	___2
-2,95212	-1,37669	NaN	0,484068	T	2	___2
-0,849606	0,86989	NaN	-1,66407	T	3	___2
NaN	NaN	NaN	NaN	T	2	___2
0,897823	1,76213	1,13325	-0,689756	T	2	___2
NaN	-0,814249	NaN	NaN	T	2	___2
NaN	0,400967	-0,0977287	0,202892	T	2	___2
NaN	-1,84815	1,58867	-2,43033	T	3	___2
-0,756289	-1,78781	NaN	-1,24364	T	2	___2
NaN	NaN	0,689962	0,0118275	T	3	___2
1,17465	0,864071	NaN	0,162775	T	4	___2
NaN	4,70809	3,33364	3,4024	T	2	___2
-0,738201	-1,28225	-3,05658	0,172514	T	2	___2
NaN	2,23153	0,481102	2,07496	T	3	___2
3,23337	3,53481	2,12948	2,28818	T	2	___2
4,08468	3,85939	0,232824	3,25723	T	2	___2
NaN	0,731764	NaN	NaN	T	3	___2
2,74322	NaN	2,74526	NaN	T	3	___2
2,34976	1,80178	NaN	1,41965	T	2	___2

0,340416	0,158981	1,78163	0,315674	T	3	___2
NaN	NaN	NaN	NaN	T	3	___2
NaN	NaN	NaN	NaN	T	3	___2
NaN	NaN	NaN	NaN	T	2	___2
2,88627	5,31452	-0,983086	1,0851	T	2	___2
0,568293	0,0729694	NaN	1,16384	T	2	___2
NaN	NaN	NaN	NaN	T	2	___2
-0,725828	0,345734	0,0704041	1,4842	T	3	___2
NaN	0,148489	-0,118727	-0,185581	T	2	___2
NaN	NaN	NaN	NaN	T	3	___2
-2,33388	-0,541586	NaN	-1,12992	T	2	___2
-2,33388	-0,541586	NaN	-1,12992	T	3	___2
NaN	NaN	NaN	NaN	T	3	___2
1,39268	1,516	NaN	1,36221	T	2	___2
1,39268	1,516	NaN	1,36221	T	2	___2
1,32763	0,957542	1,01004	-1,27663	T	3	___2
-0,0929089	-0,230665	NaN	-2,03894	T	2	___2
-0,0929089	-0,230665	NaN	-2,03894	T	2	___2
NaN	0,977312	NaN	-0,389927	T	3	___2
4,45296	4,70301	4,67063	2,64155	T	3	___2
NaN	0,976194	-0,780846	NaN	T	3	___2
NaN	NaN	NaN	NaN	T	2	___2
NaN	0,434778	1,27598	-0,298069	T	4	___2
NaN	NaN	NaN	NaN	T	2	___2
-0,337788	-2,55923	NaN	0,416893	T	2	___2
NaN	NaN	NaN	NaN	T	4	___2
-0,0355682	0,418142	NaN	0,967833	T	2	___2
3,23337	3,53481	2,12948	2,28818	T	2	___2
NaN	-1,82149	-3,03408	-0,872044	T	2	___2
1,8291	2,32944	-0,0288429	2,97232	T	4	___2
NaN	NaN	NaN	NaN	T	3	___2
3,0066	3,23625	NaN	-1,38852	T	3	___2
3,0066	3,23625	NaN	-1,38852	T	3	___2
-1,37932	-0,273357	-2,50249	NaN	T	2	___2

NaN	NaN	NaN	NaN	T	3	___2
3,3	2,97885	2,47484	3,32298	T	2	___2
-0,324711	0,91906	NaN	-0,0717983	T	2	___2
1,48507	2,14825	-1,04053	1,05947	T	2	___2
-2,26621	-2,12609	0,0629826	-0,33639	T	2	___2
NaN	NaN	NaN	NaN	T	2	___2
-1,48874	-1,2659	-1,96058	NaN	T	2	___2
-1,48874	-1,2659	-1,96058	NaN	T	2	___2
-1,82896	-2,81956	NaN	-2,08292	T	2	___2
NaN	NaN	NaN	NaN	T	3	___2
0,785265	0,871838	-0,640865	NaN	T	2	___2
NaN	NaN	NaN	NaN	T	2	___2
-0,598137	-1,08887	NaN	-1,75697	T	2	___2
0,281088	0,325432	-2,47699	-1,93228	T	3	___2
-1,2831	-2,60928	2,06124	NaN	T	2	___2
-0,758867	-0,234138	-0,797255	NaN	T	2	___2
-0,243959	0,947477	NaN	NaN	T	3	___2
NaN	NaN	NaN	NaN	T	2	___2
0,744837	1,51295	-0,234272	0,10725	T	3	___2
1,47189	2,14608	2,81849	2,66438	T	3	___2
-0,315756	-1,78087	-1,18106	NaN	T	2	___2
0,601463	-0,186266	1,34788	-0,463118	T	3	___2
-0,315756	-1,78087	-1,18106	NaN	T	2	___2
-0,774073	-1,06892	NaN	-2,86394	T	2	___2
NaN	NaN	NaN	NaN	T	3	___2
2,14189	-0,121098	-1,52113	-0,0629368	T	3	___2
NaN	NaN	NaN	NaN	T	2	___2
NaN	NaN	NaN	NaN	T	2	___2
NaN	NaN	NaN	NaN	T	2	___2
NaN	NaN	-0,303101	NaN	T	2	___2
NaN	-1,9371	3,29889	-0,530037	T	2	___2
-1,76492	NaN	NaN	NaN	T	3	___2
NaN	NaN	NaN	NaN	T	2	___2
-0,344503	2,0943	-1,55035	-0,219027	T	2	___2

-0,129318	NaN	-3,09186	-1,29129	T	2	___2
NaN	NaN	NaN	NaN	T	2	___2
0,267193	1,44762	0,376038	0,158417	T	2	___2
NaN	0,98131	0,200397	0,816574	T	3	___2
0,295387	0,0865231	1,05979	-0,952761	T	3	___2
NaN	NaN	NaN	NaN	T	3	___2
-0,406115	-0,0201893	-1,94132	-4,58494	T	2	___2
-0,84462	-1,687	NaN	NaN	T	2	___2
NaN	-0,52524	NaN	NaN	T	3	___2
-1,13178	NaN	-0,311134	-1,52767	T	2	___2
-0,797367	NaN	-1,3952	0,897646	T	2	___2
NaN	NaN	NaN	NaN	T	2	___2
NaN	NaN	NaN	NaN	T	2	___2
0,425198	0,781071	0,100021	-0,738834	T	2	___2
-1,18027	-1,23619	-1,25523	NaN	T	2	___2
NaN	2,76102	-1,18027	2,02334	T	3	___2
NaN	2,76102	-1,18027	NaN	T	3	___2
-1,4698	-0,238512	NaN	-2,94128	T	4	___2
4,95564	5,20435	NaN	3,73133	T	3	___2
4,95564	5,20435	1,25429	3,73133	T	4	___2
1,51107	0,0792961	1,01719	1,72353	T	2	___2
3,41747	NaN	-1,20575	3,33243	T	3	___2
-0,0553417	-1,47754	-0,634962	1,58446	T	3	___2
NaN	NaN	NaN	NaN	T	2	___2
NaN	2,28163	-0,133587	NaN	T	3	___2
-1,3278	NaN	-0,61861	-1,09368	T	3	___2
NaN	0,697262	-2,21913	NaN	T	4	___2
NaN	0,210197	0,472723	NaN	T	3	___2
NaN	NaN	NaN	NaN	T	3	___2
0,695349	-0,560799	0,820631	-1,45915	T	2	___2
NaN	NaN	NaN	NaN	T	3	___2
0,601282	0,918377	NaN	NaN	T	2	___2
NaN	NaN	NaN	NaN	T	2	___2
NaN	-1,758	-0,307587	NaN	T	3	___2

NaN	NaN	NaN	NaN	T	3	___2
1,12449	0,370745	0,516823	0,0338001	T	2	___2
1,11108	2,23456	-0,319271	1,67285	T	3	___2
1,93737	2,09712	-1,58429	1,65444	T	2	___2
1,23855	1,03475	NaN	NaN	T	3	___2
-0,417824	-0,577555	0,101345	-1,4732	T	2	___2
NaN	-0,720224	-1,5087	NaN	T	2	___2
-0,185658	-1,81236	2,68639	2,08222	T	2	___2
-1,25132	-1,58918	NaN	-2,09418	T	2	___2
NaN	NaN	NaN	NaN	T	2	___2
-0,342876	3,26689	-0,887417	2,26073	T	2	___2
0,843039	-0,244427	NaN	NaN	T	2	___2
-1,24244	-1,44544	-1,36365	NaN	T	2	___2
NaN	2,1215	0,173574	2,43705	T	3	___2
NaN	NaN	NaN	NaN	T	3	___2
NaN	0,89505	0,806726	-0,43729	T	3	___2
0,182806	0,722055	1,03989	-0,239042	T	2	___2
-2,06085	-1,94694	NaN	NaN	T	2	___2
NaN	NaN	NaN	NaN	T	2	___2
0,418316	0,794985	1,9919	0,361246	T	3	___2
-1,01047	-1,01917	-1,29403	-2,14083	T	3	___2
-0,505951	-1,45158	NaN	-0,427176	T	3	___2
-0,915522	-1,10857	NaN	-1,11113	T	2	___2
NaN	NaN	NaN	NaN	T	3	___2
-0,875689	-0,656593	-0,913921	NaN	T	3	___2
-0,902639	0,0582676	-0,572817	NaN	T	2	___2
-1,26558	NaN	1,25146	-1,19869	T	2	___2
0,735128	0,674362	1,59045	0,726292	T	2	___2
NaN	-0,520123	0,32292	NaN	T	3	___2
NaN	-0,822269	-0,475925	NaN	T	3	___2
-1,37725	NaN	0,524609	NaN	T	3	___2
NaN	NaN	NaN	NaN	T	4	___2
-0,460953	NaN	NaN	-0,709042	T	2	___2
-0,899082	-1,87506	NaN	-0,670988	T	3	___2

NaN	-0,729872	-1,0995	NaN	T	2	___2
0,859076	0,205341	0,913879	-0,249836	T	3	___2
1,67031	2,26608	1,76433	0,771179	T	2	___2
NaN	NaN	NaN	NaN	T	2	___2
NaN	NaN	NaN	NaN	T	3	___2
NaN	NaN	NaN	NaN	T	3	___2
NaN	NaN	NaN	NaN	T	3	___2
NaN	1,40426	NaN	0,0585747	T	3	___2
-0,588154	-0,858641	-0,128641	-1,58625	T	2	___2
-0,148201	-0,145287	0,336107	-1,64805	T	2	___2
NaN	NaN	NaN	NaN	T	3	___2
NaN	NaN	NaN	NaN	T	2	___2
NaN	0,0260258	0,0753384	NaN	T	4	___2
0,215103	0,0246849	-2,3821	0,0487118	T	2	___2
NaN	NaN	NaN	NaN	T	3	___2
NaN	-0,511129	-3,17535	-0,903336	T	2	___2
NaN	-1,43595	0,925446	-0,197218	T	2	___2
2,32977	1,83071	NaN	1,63564	T	3	___2
8,61978	8,66111	NaN	NaN	T	2	___2
0,540882	0,491838	NaN	NaN	T	3	___2
-0,27021	-1,61073	NaN	-0,583231	T	2	___2
-0,27021	-1,61073	NaN	-0,583231	T	2	___2
NaN	0,0307827	NaN	-1,90605	T	3	___2
NaN	0,0307827	NaN	-1,90605	T	3	___2
2,9317	2,22313	1,99085	0,751554	T	2	___2
-0,881996	-1,73637	NaN	-2,17153	T	3	___2
-3,4676	-0,779358	NaN	-3,95435	T	2	___2
0,316408	-1,55587	-1,63536	-1,91734	T	2	___2
-1,1532	0,33201	-1,52991	-0,69627	T	2	___2
1,68126	0,725727	1,02956	-0,780476	T	2	___2
NaN	NaN	NaN	NaN	T	2	___2
NaN	NaN	NaN	NaN	T	2	___2
NaN	NaN	NaN	NaN	T	3	___2
NaN	NaN	NaN	NaN	T	3	___2

NaN	0,892685	0,851141	-0,133698	T	2	___2
NaN	0,892685	0,851141	-0,133698	T	2	___2
0,115641	-1,37196	NaN	-1,13702	T	2	___2
2,54726	2,66689	0,581196	2,49475	T	2	___2
-0,462336	-0,133099	0,876591	-0,107845	T	3	___2
1,42362	1,03123	NaN	-0,700642	T	2	___2
2,2446	1,73693	0,974121	0,33177	T	2	___2
NaN	NaN	-0,614969	NaN	T	3	___2
NaN	-1,40141	1,66896	-0,0229988	T	3	___2
NaN	-1,40141	1,66896	-0,0229988	T	3	___2
NaN	NaN	NaN	NaN	T	2	___2
NaN	NaN	NaN	NaN	T	4	___2
NaN	-1,92519	-1,93241	-1,53553	T	2	___2
1,27663	1,09599	-1,00969	0,576912	T	2	___2
NaN	NaN	-0,870808	NaN	T	2	___2
-0,575655	1,67006	-2,85396	-2,68327	T	2	___2
NaN	NaN	NaN	NaN	T	2	___2
0,645432	1,80959	0,9566	1,10386	T	3	___2
2,54091	2,88306	1,54468	2,81578	T	2	___2
-0,932549	1,22758	-2,10399	NaN	T	2	___2
-1,60925	-0,19145	-0,291744	NaN	T	2	___2
1,04917	2,24841	-1,33577	1,38464	T	2	___2
-1,71481	-2,1289	NaN	-2,44352	T	3	___2
3,11459	4,36718	-0,691948	3,72177	T	2	___2
4,00826	3,81134	-0,858307	3,09856	T	2	___2
0,818964	-0,794922	-2,45769	0,253778	T	2	___2
4,86913	4,08543	-0,691799	3,99202	T	2	___2
3,57372	NaN	NaN	2,52596	T	2	___2
0,772787	-1,85132	-0,611374	-0,562603	T	2	___2
NaN	1,35359	1,63287	0,705755	T	2	___2
NaN	NaN	NaN	NaN	T	3	___2
0,38599	0,865404	0,325745	NaN	T	3	___2
0,263647	1,2195	0,0801029	NaN	T	4	___2
-2,83224	-1,97804	NaN	-1,91442	T	2	___2

NaN	-2,41119	-1,13617	NaN	T	3		___2
NaN	-1,77962	NaN	NaN	T	2		___2
NaN	0,79468	-1,27104	NaN	T	2		___2
-0,15016	0,79468	-1,27104	-1,03976	T	2		___2
1,06286	2,48944	2,10767	1,33489	T	3		___2
NaN	NaN	NaN	0,228989	Y	3		___2
3,23337	3,53481	2,12948	2,28818	Y	2	+	___2
3,23337	3,53481	2,12948	2,28818	Y	2	+	___2
0,266674	1,22376	NaN	0,517672	Y	3		___2
0,281088	0,325432	-2,47699	-1,93228	Y	3		___2
NaN	NaN	NaN	NaN	Y	2		___2
0,601282	0,918377	NaN	NaN	Y	2		___2
1,13244	NaN	-2,40546	1,86843	Y	3		___2
NaN	NaN	NaN	NaN	S	4		___3
NaN	NaN	NaN	NaN	S	4		___3
NaN	NaN	NaN	NaN	S	4		___3
NaN	NaN	NaN	NaN	S	3		___3
NaN	NaN	0,00955772	NaN	S	3		___3
NaN	NaN	0,00955772	NaN	S	3		___3
NaN	1,79381	2,90188	-0,147305	S	3		___3
NaN	1,07549	3,24938	-0,147305	S	2		___3
NaN	2,57511	NaN	3,07604	S	3		___3
NaN	2,57511	NaN	3,07604	S	3		___3
NaN	2,57511	NaN	3,07604	S	3		___3
NaN	1,73748	NaN	NaN	S	3		___3
NaN	1,73748	NaN	NaN	S	3		___3
-0,290407	NaN	1,12751	NaN	S	2		___3
-0,290407	NaN	1,12751	NaN	S	2		___3
NaN	NaN	NaN	NaN	S	3		___3
NaN	NaN	NaN	NaN	S	3		___3
NaN	NaN	NaN	NaN	S	3		___3
-0,523426	-0,369469	NaN	0,00479317	S	2		___3
-0,523426	-0,369469	NaN	0,00479317	S	2		___3
-0,523426	-0,369469	NaN	0,00479317	S	3		___3

NaN	-0,346542	NaN	NaN	S	2	___3
NaN	-0,346542	NaN	NaN	S	2	___3
0,078289	2,81447	NaN	1,07112	S	3	___3
0,078289	2,81447	NaN	1,07112	S	3	___3
0,078289	2,81447	NaN	1,07112	S	3	___3
2,77004	2,61957	3,53429	3,20474	S	2	___3
2,77004	2,61957	3,53429	3,20474	S	2	___3
2,77004	2,61957	3,53429	3,20474	S	2	___3
NaN	NaN	NaN	NaN	S	3	___3
NaN	NaN	NaN	NaN	S	2	___3
-2,29674	-0,583448	NaN	NaN	S	4	___3
-2,29674	-0,583448	NaN	NaN	S	4	___3
-2,29674	-0,583448	NaN	NaN	S	4	___3
NaN	NaN	NaN	NaN	S	2	___3
NaN	NaN	NaN	NaN	S	2	___3
NaN	NaN	NaN	NaN	S	3	___3
NaN	NaN	NaN	NaN	S	2	___3
NaN	NaN	NaN	NaN	S	2	___3
NaN	NaN	NaN	NaN	S	2	___3
NaN	0,0373707	NaN	0,0668507	S	2	___3
NaN	NaN	NaN	NaN	S	2	___3
NaN	NaN	NaN	NaN	S	4	___3
NaN	NaN	NaN	NaN	S	3	___3
NaN	-0,610502	NaN	NaN	S	3	___3
2,70744	3,01886	1,18453	1,69016	S	3	___3
2,70744	3,01886	1,18453	1,69016	S	3	___3
2,70744	3,01886	1,18453	1,69016	S	3	___3
NaN	0,347105	0,573223	-0,172243	S	2	___3
-0,713505	0,347105	0,573223	-0,172243	S	3	___3
-0,713505	0,347105	0,573223	-0,172243	S	2	___3
-1,74364	-0,231377	1,04216	NaN	S	3	___3
-1,74364	-0,231377	1,04216	NaN	S	3	___3
2,75631	NaN	-3,16087	NaN	S	2	___3
-0,166088	NaN	0,667587	NaN	S	3	___3

Polito et al. Universal ready-to-use immunotherapeutic approach for the treatment of cancer: expanded and activated polyclonal γδ memory T cells.
Phosphorylation sites

NaN	NaN	NaN	NaN	S	3	___3
-0,166088	NaN	0,667587	NaN	S	3	___3
1,67889	0,577122	NaN	0,631203	S	3	___3
1,67889	0,577122	0,380156	0,631203	S	3	___3
0,141188	0,223242	-1,4314	-0,122892	S	2	___3
0,141188	0,223242	-1,4314	-0,122892	S	2	___3
3,96306	5,29674	0,329473	3,99707	S	2	___3
3,96306	5,29674	0,329473	3,99707	S	2	___3
3,96306	5,29674	0,329473	3,99707	S	3	___3
0,141188	0,223242	-1,4314	-0,122892	S	2	___3
NaN	0,292456	0,191948	NaN	S	3	___3
NaN	0,292456	0,191948	NaN	S	3	___3
NaN	0,511484	NaN	NaN	S	3	___3
NaN	0,511484	NaN	NaN	S	3	___3
NaN	0,511484	NaN	NaN	S	3	___3
-1,26208	-2,17971	NaN	-0,270203	S	3	___3
-1,26208	-2,17971	NaN	-0,270203	S	3	___3
-1,26208	-2,17971	NaN	-0,270203	S	2	___3
NaN	NaN	NaN	NaN	S	2	___3
NaN	NaN	NaN	NaN	S	2	___3
NaN	NaN	NaN	NaN	S	2	___3
NaN	1,9352	0,390402	1,16823	S	2	___3
NaN	1,9352	0,390402	1,16823	S	3	___3
NaN	1,9352	0,390402	1,16823	S	3	___3
0,453533	-1,06287	NaN	-0,652458	S	2	___3
0,453533	-1,06287	NaN	-0,652458	S	2	___3
0,453533	-1,06287	NaN	-0,652458	S	2	___3
-1,265	-0,203617	NaN	-0,270937	S	2	___3
-1,265	-0,203617	NaN	-0,270937	S	2	___3
-1,265	-0,203617	NaN	-0,270937	S	2	___3
-0,343756	-0,534872	0,519196	0,377188	S	4	___3
-0,343756	-0,534872	0,519196	0,377188	S	4	___3
-0,343756	-0,534872	0,519196	0,377188	S	4	___3
0,49198	2,39418	-1,2707	1,42052	S	2	___3

-0,724283	-0,0896244	-0,575069	0,376593	S	3	___3
-0,724283	-0,0896244	-0,575069	0,376593	S	3	___3
0,49198	2,39418	-1,2707	1,42052	S	2	___3
-0,724283	-0,0896244	-0,575069	0,376593	S	3	___3
0,49198	2,39418	-1,2707	1,42052	S	2	___3
2,21729	-4,47717	2,17503	2,72629	S	2	___3
2,21729	-4,47717	2,17503	2,72629	S	2	___3
2,21729	-4,47717	2,17503	2,72629	S	2	___3
NaN	NaN	-0,271572	NaN	S	2	___3
NaN	NaN	-0,271572	NaN	S	2	___3
NaN	NaN	-0,271572	NaN	S	2	___3
NaN	NaN	NaN	NaN	S	3	___3
0,15852	0,937223	2,03309	0,349051	S	3	___3
0,15852	0,937223	2,03309	NaN	S	3	___3
NaN	NaN	NaN	NaN	S	2	___3
NaN	NaN	NaN	NaN	S	2	___3
NaN	NaN	NaN	NaN	S	3	___3
-2,80673	-1,62103	1,19484	-1,19816	S	2	___3
-2,80673	-1,62103	1,19484	-1,19816	S	2	___3
-2,80673	-1,62103	1,19484	-1,19816	S	2	___3
0,805389	0,764168	2,6783	0,530167	S	4	___3
0,805389	0,764168	2,6783	0,530167	S	4	___3
0,805389	0,764168	2,6783	0,530167	S	4	___3
-0,828102	0,440933	0,0357914	NaN	S	3	___3
-0,828102	0,440933	0,0357914	NaN	S	3	___3
-0,828102	0,440933	0,0357914	NaN	S	3	___3
NaN	NaN	NaN	NaN	S	2	___3
NaN	NaN	NaN	NaN	S	2	___3
NaN	NaN	NaN	NaN	S	2	___3
NaN	-1,51459	-0,781326	0,299492	S	2	___3
NaN	-1,51459	-0,781326	0,299492	S	2	___3
NaN	-0,95089	-1,83828	-1,13583	S	3	___3
NaN	-0,95089	-1,83828	-1,13583	S	3	___3
NaN	-0,95089	-1,83828	-1,13583	S	3	___3

Polito et al. Universal ready-to-use immunotherapeutic approach for the treatment of cancer: expanded and activated polyclonal γδ memory T cells.
Phosphorylation sites

NaN	NaN	NaN	NaN	S	2	___3
NaN	NaN	NaN	NaN	S	2	___3
NaN	NaN	NaN	NaN	S	2	___3
3,15883	3,87657	0,696154	NaN	S	3	___3
NaN	NaN	3,14245	NaN	S	4	___3
NaN	-0,00984955	-1,81442	-1,99402	S	2	___3
NaN	-0,00984955	-1,81442	-1,99402	S	2	___3
NaN	-0,00984955	-1,81442	-1,99402	S	2	___3
1,16151	2,12181	4,02367	2,7102	S	4	___3
1,16151	2,12181	4,02367	2,7102	S	3	___3
1,16151	2,12181	4,02367	2,7102	S	4	___3
NaN	-1,07186	NaN	NaN	S	3	___3
NaN	-1,07186	NaN	NaN	S	3	___3
NaN	-1,07186	NaN	NaN	S	4	___3
0,81576	1,15262	NaN	-0,513021	S	2	___3
0,81576	1,15262	NaN	-0,513021	S	2	___3
0,81576	1,15262	NaN	-0,513021	S	2	___3
NaN	NaN	NaN	NaN	S	2	___3
NaN	NaN	NaN	NaN	S	2	___3
NaN	NaN	NaN	NaN	S	2	___3
1,49075	1,4783	2,88616	1,85606	S	2	___3
NaN	1,34051	3,69437	1,7585	S	2	___3
NaN	0,0538921	3,18302	1,58438	S	2	___3
NaN	NaN	NaN	NaN	S	2	___3
NaN	NaN	NaN	NaN	S	3	___3
NaN	NaN	NaN	NaN	S	3	___3
NaN	NaN	NaN	NaN	S	3	___3
NaN	NaN	NaN	NaN	S	3	___3
0,797037	1,50105	1,84176	1,05091	S	2	___3
0,797037	1,50105	1,84176	1,05091	S	2	___3
0,797037	1,50105	1,84176	1,05091	S	2	___3
NaN	NaN	NaN	NaN	S	2	___3
NaN	NaN	NaN	NaN	S	2	___3
NaN	NaN	NaN	NaN	S	2	___3

Polito et al. Universal ready-to-use immunotherapeutic approach for the treatment of cancer: expanded and activated polyclonal γδ memory T cells.
Phosphorylation sites

NaN	NaN	NaN	NaN	S	2	___3
NaN	NaN	NaN	NaN	S	2	___3
NaN	NaN	NaN	NaN	S	2	___3
1,90211	4,09064	NaN	2,95988	S	2	___3
-1,33328	-0,82522	NaN	-0,902285	S	2	___3
1,90211	4,09064	NaN	2,95988	S	2	___3
-1,33328	-0,82522	NaN	-0,902285	S	2	___3
-1,33328	-0,82522	NaN	-0,902285	S	2	___3
0,298828	2,67296	1,61831	2,31059	S	2	___3
0,298828	2,67296	1,61831	2,31059	S	3	___3
0,298828	2,67296	1,61831	NaN	S	2	___3
-0,101954	2,61809	NaN	2,31059	S	2	___3
0,47266	1,15003	-1,55965	0,873812	S	2	___3
0,47266	1,15003	-1,55965	0,873812	S	2	___3
0,47266	1,15003	-1,55965	0,873812	S	2	___3
3,63675	4,65029	1,13918	3,54465	S	2	___3
NaN	NaN	NaN	NaN	S	2	___3
-0,0710144	-0,816517	-1,08235	NaN	S	2	___3
-0,0710144	-0,816517	-1,08235	NaN	S	2	___3
-0,0710144	-0,816517	-1,08235	NaN	S	2	___3
NaN	NaN	NaN	NaN	S	2	___3
NaN	NaN	1,42271	-0,780556	S	2	___3
0,205881	-0,971638	1,42271	-0,780556	S	2	___3
NaN	-0,971638	NaN	NaN	S	3	___3
-2,4202	-1,72418	NaN	NaN	S	3	___3
-2,4202	-1,72418	NaN	NaN	S	3	___3
-2,4202	-1,72418	NaN	NaN	S	3	___3
-2,4202	-1,72418	NaN	NaN	S	2	___3
-0,476151	-0,92013	1,13006	-0,390358	S	2	___3
-0,476151	-0,92013	1,13006	-0,390358	S	2	___3
-0,476151	-0,92013	1,13006	-0,390358	S	2	___3
1,63683	0,113956	-0,294653	-1,84511	S	2	___3
NaN	0,113956	-0,294653	NaN	S	2	___3
-1,904	-0,7339	NaN	NaN	S	2	___3

-1,904	-0,7339	NaN	NaN	S	2	___3
NaN	NaN	NaN	NaN	S	2	___3
-1,904	-0,7339	NaN	NaN	S	3	___3
-0,797548	-0,520014	-0,664169	NaN	S	2	___3
-0,797548	-0,520014	-0,664169	NaN	S	2	___3
-0,797548	-0,520014	-0,664169	NaN	S	2	___3
-2,10496	0,356607	-2,49748	0,060957	S	3	___3
-2,10496	0,356607	-2,49748	0,060957	S	2	___3
-2,10496	0,356607	-2,49748	0,060957	S	3	___3
1,18975	0,872004	1,14614	1,0652	S	2	___3
1,18975	0,872004	1,14614	1,0652	S	2	___3
1,18975	0,872004	1,14614	1,0652	S	2	___3
3,22728	3,56093	-0,465763	2,30368	S	2	___3
3,22728	3,56093	-0,465763	2,30368	S	2	___3
-2,5975	-2,25098	0,607174	-0,45141	S	4	___3
-2,5975	-2,25098	0,607174	-0,45141	S	4	___3
-2,5975	-2,25098	0,607174	-0,45141	S	4	___3
NaN	0,0976162	-0,631027	-1,22583	S	3	___3
NaN	0,0976162	-0,631027	-1,22583	S	3	___3
NaN	0,0976162	-0,631027	-1,22583	S	3	___3
NaN	0,132261	NaN	NaN	S	2	___3
NaN	0,132261	NaN	NaN	S	2	___3
NaN	0,132261	NaN	NaN	S	2	___3
NaN	NaN	NaN	NaN	S	3	___3
0,729017	2,1631	NaN	-1,67203	S	2	___3
0,729017	2,1631	NaN	-1,67203	S	2	___3
0,729017	2,1631	NaN	-1,67203	S	3	___3
NaN	-0,22401	-2,63997	0,155222	S	3	___3
NaN	-0,22401	-2,63997	0,155222	S	3	___3
NaN	-0,22401	-2,63997	0,155222	S	3	___3
NaN	NaN	NaN	NaN	S	3	___3
NaN	NaN	NaN	NaN	S	3	___3
NaN	NaN	NaN	NaN	S	3	___3
NaN	NaN	NaN	NaN	S	2	___3

Polito et al. Universal ready-to-use immunotherapeutic approach for the treatment of cancer: expanded and activated polyclonal γδ memory T cells.
Phosphorylation sites

NaN	NaN	NaN	NaN	S	2	___3
NaN	NaN	NaN	NaN	S	2	___3
2,14048	1,75672	1,45635	1,61873	S	2	___3
2,14048	1,75672	1,45635	1,61873	S	2	___3
2,14048	1,75672	1,45635	1,61873	S	2	___3
NaN	NaN	NaN	NaN	S	2	___3
NaN	0,0065403	NaN	0,167747	S	3	___3
NaN	NaN	NaN	NaN	S	3	___3
NaN	NaN	NaN	NaN	S	3	___3
-0,754885	-0,220297	NaN	-1,9985	S	2	___3
-0,754885	-0,220297	NaN	-1,9985	S	2	___3
-0,754885	-0,220297	NaN	-1,9985	S	2	___3
-0,466352	0,400047	-1,06073	NaN	S	3	___3
-0,466352	0,400047	-1,06073	-0,138073	S	2	___3
-0,466352	0,400047	-1,06073	-0,138073	S	2	___3
1,81539	0,631462	2,47048	0,310413	S	2	___3
1,81539	0,631462	2,47048	0,310413	S	2	___3
1,81539	0,631462	NaN	0,310413	S	2	___3
-2,2642	-3,0821	-0,103643	NaN	S	2	___3
-2,2642	-3,0821	-0,103643	NaN	S	2	___3
-2,2642	-3,0821	-0,103643	NaN	S	2	___3
-0,429861	1,0509	-0,279631	-0,834757	S	2	___3
0,419043	0,218559	1,16089	-0,276941	S	3	___3
0,419043	0,218559	0,497929	-0,276941	S	2	___3
0,302092	-2,1689	NaN	-0,336035	S	2	___3
NaN	NaN	NaN	NaN	S	2	___3
NaN	NaN	NaN	NaN	S	2	___3
NaN	NaN	NaN	NaN	S	2	___3
-0,695662	-1,21777	-0,523308	NaN	S	3	___3
-0,695662	-1,21777	-0,523308	NaN	S	3	___3
NaN	NaN	NaN	NaN	S	2	___3
NaN	NaN	NaN	NaN	S	2	___3
-1,2519	1,71182	NaN	0,892609	S	2	___3
-1,2519	1,71182	NaN	0,892609	S	2	___3

NaN	NaN	NaN	NaN	S	2	___3
NaN	0,507225	NaN	NaN	S	2	___3
NaN	0,507225	NaN	NaN	S	2	___3
2,49592	3,3923	1,35717	1,33758	S	4	___3
2,49592	3,3923	1,35717	1,33758	S	3	___3
2,49592	3,3923	1,35717	1,33758	S	2	___3
2,30741	2,68043	1,11338	0,734901	S	3	___3
2,30741	2,99738	1,80373	0,734901	S	3	___3
2,30741	2,99738	1,80373	0,734901	S	3	___3
-0,170216	-0,623289	NaN	-2,12205	S	2	___3
-0,170216	-0,623289	NaN	-2,12205	S	2	___3
-0,170216	-0,623289	NaN	-2,12205	S	3	___3
-3,22159	0,486115	NaN	NaN	S	3	___3
-3,22159	0,486115	NaN	NaN	S	3	___3
NaN	NaN	NaN	NaN	S	3	___3
NaN	NaN	NaN	NaN	S	3	___3
-0,938845	-0,876062	-1,82494	NaN	S	3	___3
-0,938845	-0,876062	-1,82494	NaN	S	3	___3
-0,938845	-0,876062	-1,82494	NaN	S	3	___3
NaN	1,36228	NaN	2,52657	S	2	___3
NaN	1,36228	NaN	2,52657	S	2	___3
NaN	1,36228	NaN	2,52657	S	2	___3
NaN	0,0412788	1,04103	-0,0504398	S	2	___3
NaN	0,0412788	1,04103	-0,0504398	S	2	___3
-2,00455	NaN	-0,189737	-1,20868	S	2	___3
-2,00455	NaN	-0,189737	-1,20868	S	3	___3
NaN	NaN	NaN	NaN	S	3	___3
1,07303	0,818384	NaN	NaN	S	3	___3
1,07303	0,818384	NaN	NaN	S	3	___3
NaN	0,818384	NaN	NaN	S	2	___3
0,47266	1,15003	-1,55965	0,873812	S	2	___3
0,47266	1,15003	-1,55965	0,873812	S	2	___3
1,17062	1,51255	2,11117	0,51898	S	2	___3
0,691299	1,38387	1,18289	-0,184677	S	3	___3

1,24504	1,12258	1,6694	-0,423935	S	2	___3
0,423725	0,947399	1,64125	-0,154432	S	2	___3
NaN	-1,35788	NaN	NaN	S	3	___3
-1,27419	-0,686167	NaN	NaN	S	3	___3
-1,27419	-0,686167	NaN	NaN	S	3	___3
NaN	2,25503	NaN	1,51616	S	3	___3
NaN	2,25503	NaN	1,51616	S	3	___3
NaN	2,25503	NaN	1,51616	S	3	___3
-2,42046	-0,807468	NaN	-0,809044	S	3	___3
-2,42046	-0,807468	NaN	-0,809044	S	3	___3
-2,42046	-0,807468	NaN	-0,809044	S	3	___3
NaN	NaN	NaN	NaN	S	2	___3
NaN	NaN	NaN	NaN	S	2	___3
NaN	0,295238	-1,14427	-1,63533	S	2	___3
NaN	0,295238	-1,14427	-1,63533	S	2	___3
NaN	0,295238	-1,14427	-1,63533	S	2	___3
NaN	NaN	NaN	NaN	S	4	___3
NaN	NaN	NaN	NaN	S	4	___3
-1,01634	-1,20363	-1,07821	-2,35505	S	2	___3
-1,01634	-1,20363	-1,07821	-2,35505	S	2	___3
-1,01634	-1,20363	-1,07821	-2,35505	S	2	___3
NaN	1,14979	NaN	0,263432	S	2	___3
NaN	1,14979	NaN	0,263432	S	3	___3
NaN	1,14979	NaN	0,263432	S	3	___3
NaN	0,448839	0,0171223	NaN	S	3	___3
NaN	4,48954	-0,715794	0,447498	S	3	___3
NaN	NaN	NaN	NaN	S	2	___3
NaN	NaN	NaN	NaN	S	2	___3
NaN	NaN	NaN	NaN	S	2	___3
NaN	NaN	NaN	NaN	S	2	___3
NaN	NaN	NaN	NaN	S	2	___3
NaN	NaN	NaN	NaN	S	2	___3
-0,678646	NaN	0,873926	-1,66266	S	3	___3
-0,678646	NaN	0,873926	-1,66266	S	2	___3

Polito et al. Universal ready-to-use immunotherapeutic approach for the treatment of cancer: expanded and activated polyclonal γδ memory T cells.
Phosphorylation sites

-1,25297	-1,22157	NaN	-1,68991	S	3	___3
-1,25297	-1,22157	NaN	-1,68991	S	4	___3
NaN	NaN	NaN	NaN	S	2	___3
NaN	NaN	NaN	NaN	S	2	___3
-0,870548	0,430679	NaN	-1,34663	S	3	___3
NaN	0,198223	1,10204	0,278149	S	2	___3
NaN	0,198223	1,10204	0,278149	S	2	___3
0,778172	0,198223	1,10204	0,278149	S	2	___3
-2,5664	-2,08017	NaN	NaN	S	2	___3
-2,5664	-2,08017	NaN	NaN	S	2	___3
-2,5664	-2,08017	NaN	NaN	S	2	___3
-2,48369	-3,82541	-2,98593	0,0253792	S	3	___3
-2,48369	-3,82541	-2,98593	0,0253792	S	3	___3
-2,48369	-3,82541	-2,98593	0,0253792	S	3	___3
0,566727	0,485519	NaN	-0,170977	S	2	___3
0,566727	0,485519	NaN	-0,170977	S	2	___3
0,566727	0,485519	NaN	-0,170977	S	2	___3
-0,716063	NaN	0,39687	-2,60117	S	3	___3
-0,716063	NaN	0,39687	-2,60117	S	2	___3
-0,716063	NaN	0,39687	-2,60117	S	2	___3
NaN	NaN	NaN	NaN	S	2	___3
NaN	NaN	NaN	NaN	S	2	___3
NaN	NaN	NaN	NaN	S	2	___3
NaN	NaN	NaN	NaN	S	2	___3
NaN	NaN	NaN	NaN	S	2	___3
NaN	NaN	NaN	NaN	S	2	___3
NaN	-0,227734	NaN	-0,23686	S	2	___3
NaN	-0,227734	NaN	-0,23686	S	2	___3
NaN	-0,227734	NaN	-0,23686	S	3	___3
NaN	NaN	NaN	NaN	S	2	___3
NaN	NaN	NaN	NaN	S	2	___3
NaN	NaN	NaN	NaN	S	3	___3
NaN	NaN	NaN	NaN	S	2	___3
NaN	NaN	NaN	NaN	S	3	___3

NaN	NaN	NaN	NaN	S	3	___3
NaN	NaN	NaN	NaN	S	2	___3
1,09092	1,52892	-1,14808	0,524624	S	3	___3
-0,212265	0,294621	NaN	-0,0974331	S	2	___3
NaN	NaN	NaN	NaN	S	2	___3
NaN	NaN	NaN	NaN	S	3	___3
-2,009	NaN	0,432297	-1,71069	S	2	___3
-2,009	NaN	0,432297	-1,71069	S	2	___3
-2,009	NaN	0,432297	-1,71069	S	2	___3
NaN	NaN	NaN	NaN	S	3	___3
NaN	NaN	NaN	NaN	S	3	___3
NaN	NaN	NaN	NaN	S	3	___3
NaN	NaN	NaN	NaN	S	2	___3
1,63326	0,628622	-0,827003	0,271566	S	2	___3
1,63326	0,628622	-0,827003	0,271566	S	2	___3
1,63326	0,628622	-0,827003	0,271566	S	2	___3
-1,31619	0,968584	-0,181585	0,767193	S	3	___3
-1,31619	0,968584	-0,181585	0,767193	S	2	___3
-1,31619	0,968584	-0,181585	0,767193	S	2	___3
NaN	NaN	NaN	NaN	S	3	___3
NaN	NaN	NaN	NaN	S	3	___3
NaN	NaN	NaN	NaN	S	3	___3
2,35965	1,94789	0,140913	0,855286	S	3	___3
NaN	-0,40626	NaN	NaN	S	3	___3
-0,417915	NaN	0,140913	NaN	S	3	___3
1,85049	1,63391	-2,07262	0,855286	S	3	___3
0,956099	0,655142	2,57386	1,6643	S	2	___3
0,956099	0,655142	2,57386	1,6643	S	3	___3
NaN	NaN	NaN	NaN	S	2	___3
NaN	NaN	NaN	NaN	S	2	___3
-3,75422	2,91828	NaN	2,45549	S	3	___3
-3,75422	2,91828	NaN	2,45549	S	3	___3
-1,31446	0,310965	NaN	0,952421	S	2	___3
-1,31446	0,310965	NaN	0,952421	S	2	___3

-1,31446	0,310965	NaN	0,952421	S	2	___3
-2,28338	0,713993	-0,759333	NaN	S	2	___3
-2,28338	0,713993	-0,759333	NaN	S	2	___3
NaN	NaN	NaN	NaN	S	2	___3
NaN	NaN	NaN	NaN	S	2	___3
NaN	NaN	NaN	NaN	S	3	___3
NaN	NaN	NaN	NaN	S	2	___3
1,89449	3,28364	1,7496	4,04907	S	2	___3
1,89449	3,28364	1,7496	4,04907	S	2	___3
-0,417315	-2,52476	NaN	-1,90605	S	2	___3
-0,417315	-2,52476	NaN	-1,90605	S	2	___3
-0,417315	-2,52476	NaN	-1,90605	S	2	___3
NaN	NaN	NaN	NaN	S	2	___3
1,89449	3,28364	1,7496	4,04907	S	2	___3
NaN	NaN	NaN	NaN	S	3	___3
NaN	NaN	NaN	NaN	S	2	___3
-1,2408	0,501776	NaN	1,23224	S	2	___3
NaN	NaN	NaN	NaN	S	3	___3
NaN	NaN	NaN	NaN	S	2	___3
NaN	NaN	NaN	NaN	S	3	___3
NaN	NaN	NaN	NaN	S	2	___3
NaN	NaN	NaN	NaN	S	2	___3
1,75127	4,84862	1,28116	3,16937	S	3	___3
1,75127	4,84862	1,28116	3,16937	S	3	___3
1,75127	4,84862	1,28116	3,16937	S	4	___3
NaN	1,73748	NaN	NaN	T	3	___3
NaN	NaN	NaN	NaN	T	3	___3
NaN	-0,346542	NaN	NaN	T	2	___3
NaN	0,0373707	NaN	0,0668507	T	2	___3
NaN	-0,610502	NaN	NaN	T	3	___3
2,75631	NaN	-3,16087	NaN	T	2	___3
2,75631	NaN	-3,16087	NaN	T	2	___3
-0,166088	NaN	0,667587	NaN	T	3	___3
1,67889	0,577122	0,380156	0,631203	T	3	___3

Polito et al. Universal ready-to-use immunotherapeutic approach for the treatment of cancer: expanded and activated polyclonal γδ memory T cells.
Phosphorylation sites

NaN	0,292456	0,191948	NaN	T	3	___3
-1,99099	-1,33474	NaN	NaN	T	3	___3
NaN	NaN	NaN	NaN	T	3	___3
0,15852	NaN	2,03309	NaN	T	3	___3
NaN	-1,51459	-0,781326	0,299492	T	2	___3
1,49075	1,4783	2,88616	1,85606	T	2	___3
NaN	NaN	NaN	NaN	T	2	___3
NaN	NaN	NaN	NaN	T	2	___3
NaN	NaN	NaN	NaN	T	2	___3
NaN	NaN	NaN	NaN	T	2	___3
1,63683	0,113956	-0,294653	-1,84511	T	2	___3
3,22728	3,56093	-0,465763	2,30368	T	2	___3
NaN	NaN	NaN	NaN	T	2	___3
NaN	-0,461233	-0,409191	-2	T	3	___3
0,302092	-2,1689	NaN	-0,336035	T	2	___3
-0,695662	-1,21777	-0,523308	NaN	T	2	___3
NaN	NaN	NaN	NaN	T	4	___3
NaN	NaN	NaN	NaN	T	4	___3
NaN	0,655577	0,408804	-0,876854	T	3	___3
-3,22159	0,486115	NaN	NaN	T	3	___3
NaN	0,0412788	1,04103	-0,0504398	T	2	___3
1,07303	0,818384	NaN	NaN	T	3	___3
1,56371	1,88581	2,27949	0,830473	T	2	___3
NaN	-1,35788	NaN	NaN	T	3	___3
NaN	NaN	NaN	NaN	T	4	___3
NaN	0,448839	0,0171223	NaN	T	4	___3
-0,678646	NaN	0,873926	-1,66266	T	3	___3
-1,03511	1,60597	-0,0134449	NaN	T	2	___3
NaN	NaN	NaN	NaN	T	2	___3
1,09092	1,52892	-1,14808	0,524624	T	3	___3
NaN	NaN	NaN	NaN	T	3	___3
1,51483	1,63391	-0,209436	NaN	T	3	___3
0,956099	0,655142	2,57386	1,6643	T	2	___3
-3,75422	2,91828	NaN	2,45549	T	3	___3

-1,2408	0,501776	NaN	1,23224	T	2	___3
NaN	NaN	NaN	NaN	T	2	___3
NaN	NaN	NaN	NaN	T	2	___3
NaN	0,511484	NaN	NaN	Y	3	___3
NaN	NaN	NaN	NaN	Y	3	___3
NaN	NaN	NaN	NaN	Y	3	___3

Polito et al. Universal ready-to-use immunotherapeutic approach for the treatment of cancer: expanded and activated polyclonal γδ memory T cells.
Phosphorylation sites

C: KEGG name
Lysosome
Lysosome
Ribosome
Cell adhesion molecules (CAMs);Leukocyte transendothelial migration;Malaria
Axon guidance
RNA transport
RNA transport
Alzheimer's disease
Osteoclast differentiation
Cytosolic DNA-sensing pathway;Purine metabolism;Pyrimidine metabolism;RNA polymerase
Measles;NOD-like receptor signaling pathway
Measles;NOD-like receptor signaling pathway
Measles;NOD-like receptor signaling pathway
Measles;NOD-like receptor signaling pathway
Measles;NOD-like receptor signaling pathway
Spliceosome

RNA transport
RNA transport
RNA transport
RNA transport
RNA transport
RNA transport
Ribosome biogenesis in eukaryotes
Endocytosis
Endocytosis
Endocytosis
Purine metabolism
RNA transport;Spliceosome

RNA transport;Spliceosome
Ribosome
RIG-I-like receptor signaling pathway
RIG-I-like receptor signaling pathway
Hepatitis C;Measles

Antigen processing and presentation;Proteasome

Phosphorylation sites

Lysine degradation
Fat digestion and absorption;Glycerolipid metabolism;Retinol metabolism
Tight junction
Tight junction
Endocytosis;Fc gamma R-mediated phagocytosis
Endocytosis;Fc gamma R-mediated phagocytosis
Endocytosis;Fc gamma R-mediated phagocytosis
Endocytosis;Fc gamma R-mediated phagocytosis

Axon guidance
Axon guidance
Axon guidance
Axon guidance
Axon guidance
pathway;Gastric acid secretion;Glioma;GnRH signaling pathway;ko05152;Long-term potentiation;Melanogenesis;Neurotrophin signaling pathway;Olfactory transduction;Oocyte meiosis;Photo
pathway;Gastric acid secretion;Glioma;GnRH signaling pathway;ko05152;Long-term potentiation;Melanogenesis;Neurotrophin signaling pathway;Olfactory transduction;Oocyte meiosis;Photo
RNA transport;Viral myocarditis
RNA transport;Viral myocarditis
Insulin signaling pathway

Polito et al. Universal ready-to-use immunotherapeutic approach for the treatment of cancer: expanded and activated polyclonal γδ memory T cells.
Phosphorylation sites

Cell adhesion molecules (CAMs);Fc gamma R-mediated phagocytosis;Primary immunodeficiency;T cell receptor signaling pathway
Cell adhesion molecules (CAMs);Fc gamma R-mediated phagocytosis;Primary immunodeficiency;T cell receptor signaling pathway
Cell adhesion molecules (CAMs);Fc gamma R-mediated phagocytosis;Primary immunodeficiency;T cell receptor signaling pathway
Cell adhesion molecules (CAMs);Fc gamma R-mediated phagocytosis;Primary immunodeficiency;T cell receptor signaling pathway
Bacterial invasion of epithelial cells;Endometrial cancer;Focal adhesion;PPAR signaling pathway
Cell cycle;Pathways in cancer;Small cell lung cancer;Ubiquitin mediated proteolysis
Cell cycle;Pathways in cancer;Small cell lung cancer;Ubiquitin mediated proteolysis

Polito et al. Universal ready-to-use immunotherapeutic approach for the treatment of cancer: expanded and activated polyclonal γδ memory T cells.
Phosphorylation sites

Mineral absorption
Cell adhesion molecules (CAMs);Staphylococcus aureus infection
Cell adhesion molecules (CAMs);Staphylococcus aureus infection
Ribosome
Peroxisome
Peroxisome
Spliceosome
Spliceosome

Polito et al. Universal ready-to-use immunotherapeutic approach for the treatment of cancer: expanded and activated polyclonal γδ memory T cells.
Phosphorylation sites

RIG-I-like receptor signaling pathway
RIG-I-like receptor signaling pathway
RIG-I-like receptor signaling pathway

Inositol phosphate metabolism;Phosphatidylinositol signaling system
Inositol phosphate metabolism;Phosphatidylinositol signaling system
ocessing and presentation;Autoimmune thyroid disease;Cell adhesion molecules (CAMs);Endocytosis;Graft-versus-host disease;Natural killer cell mediated cytotoxicity;Phagosome;Type I diabe
ocessing and presentation;Autoimmune thyroid disease;Cell adhesion molecules (CAMs);Endocytosis;Graft-versus-host disease;Natural killer cell mediated cytotoxicity;Phagosome;Type I diabe
ocessing and presentation;Autoimmune thyroid disease;Cell adhesion molecules (CAMs);Endocytosis;Graft-versus-host disease;Natural killer cell mediated cytotoxicity;Phagosome;Type I diabe
Lysine degradation
Collecting duct acid secretion

ocessing and presentation;Autoimmune thyroid disease;Cell adhesion molecules (CAMs);Endocytosis;Graft-versus-host disease;Natural killer cell mediated cytotoxicity;Phagosome;Type I diabe
Alanine, aspartate and glutamate metabolism;Purine metabolism

Endocytosis
Bacterial invasion of epithelial cells;Chemokine signaling pathway;Focal adhesion;Leukocyte transendothelial migration;Regulation of actin cytoskeleton;VEGF signaling pathway
Bacterial invasion of epithelial cells;Chemokine signaling pathway;Focal adhesion;Leukocyte transendothelial migration;Regulation of actin cytoskeleton;VEGF signaling pathway
Bacterial invasion of epithelial cells;Chemokine signaling pathway;Focal adhesion;Leukocyte transendothelial migration;Regulation of actin cytoskeleton;VEGF signaling pathway
Bacterial invasion of epithelial cells;Chemokine signaling pathway;Focal adhesion;Leukocyte transendothelial migration;Regulation of actin cytoskeleton;VEGF signaling pathway
Bacterial invasion of epithelial cells;Chemokine signaling pathway;Focal adhesion;Leukocyte transendothelial migration;Regulation of actin cytoskeleton;VEGF signaling pathway
Bacterial invasion of epithelial cells;Chemokine signaling pathway;Focal adhesion;Leukocyte transendothelial migration;Regulation of actin cytoskeleton;VEGF signaling pathway
Bacterial invasion of epithelial cells;Chemokine signaling pathway;Focal adhesion;Leukocyte transendothelial migration;Regulation of actin cytoskeleton;VEGF signaling pathway
Leishmaniasis;MAPK signaling pathway;Measles;NOD-like receptor signaling pathway;Osteoclast differentiation;Toll-like receptor signaling pathway;Toxoplasmosis
Leishmaniasis;MAPK signaling pathway;Measles;NOD-like receptor signaling pathway;Osteoclast differentiation;Toll-like receptor signaling pathway;Toxoplasmosis
Tight junction
Tight junction
Tight junction
Tight junction

Polito et al. Universal ready-to-use immunotherapeutic approach for the treatment of cancer: expanded and activated polyclonal γδ memory T cells.
Phosphorylation sites

Bacterial invasion of epithelial cells;Fc gamma R-mediated phagocytosis;Pathogenic Escherichia coli infection;Regulation of actin cytoskeleton;Shigellosis
Fructose and mannose metabolism
RNA transport
RNA transport
Inositol phosphate metabolism;ko05152;Phagosome;Phosphatidylinositol signaling system;Regulation of autophagy
Ubiquitin mediated proteolysis
Ubiquitin mediated proteolysis
Ubiquitin mediated proteolysis
Ubiquitin mediated proteolysis

on;Dilated cardiomyopathy;Focal adhesion;Hypertrophic cardiomyopathy (HCM);Leukocyte transendothelial migration;Pathogenic Escherichia coli infection;Phagosome;Phototransduction - fly
al cells;Dilated cardiomyopathy;Focal adhesion;Hypertrophic cardiomyopathy (HCM);Leukocyte transendothelial migration;Pathogenic Escherichia coli infection;Phagosome;Phototransduction
on;Dilated cardiomyopathy;Focal adhesion;Hypertrophic cardiomyopathy (HCM);Leukocyte transendothelial migration;Pathogenic Escherichia coli infection;Phagosome;Phototransduction - fly
RNA degradation

Polito et al. Universal ready-to-use immunotherapeutic approach for the treatment of cancer: expanded and activated polyclonal γδ memory T cells.
Phosphorylation sites

RNA transport
RNA transport
RNA transport
RNA transport
RIG-I-like receptor signaling pathway
RNA transport
mRNA surveillance pathway;RNA transport
mRNA surveillance pathway;RNA transport
mRNA surveillance pathway;RNA transport
mRNA surveillance pathway;RNA transport
mRNA surveillance pathway;RNA transport
mRNA surveillance pathway;RNA transport
mRNA surveillance pathway;RNA transport
mRNA surveillance pathway;RNA transport
mRNA surveillance pathway;RNA transport
mRNA surveillance pathway;RNA transport
mRNA surveillance pathway;RNA transport
mRNA surveillance pathway;RNA transport
mRNA surveillance pathway;RNA transport
mRNA surveillance pathway;RNA transport
mRNA surveillance pathway;RNA transport
mRNA surveillance pathway;RNA transport

mRNA surveillance pathway;RNA transport
mRNA surveillance pathway;RNA transport
mRNA surveillance pathway;RNA transport
mRNA surveillance pathway;RNA transport
mRNA surveillance pathway;RNA transport
Regulation of actin cytoskeleton
Regulation of actin cytoskeleton
Regulation of actin cytoskeleton
Regulation of actin cytoskeleton
Regulation of actin cytoskeleton
mRNA surveillance pathway;RNA degradation;RNA transport
mRNA surveillance pathway;RNA degradation;RNA transport

Polito et al. Universal ready-to-use immunotherapeutic approach for the treatment of cancer: expanded and activated polyclonal γδ memory T cells.
Phosphorylation sites

pylori infection;ErbB signaling pathway;Fc gamma R-mediated phagocytosis;Focal adhesion;MAPK signaling pathway;MAPK signaling pathway - yeast;Natural killer cell mediated cytotoxicity;Re
pylori infection;ErbB signaling pathway;Fc gamma R-mediated phagocytosis;Focal adhesion;MAPK signaling pathway;MAPK signaling pathway - yeast;Natural killer cell mediated cytotoxicity;Re
pylori infection;ErbB signaling pathway;Fc gamma R-mediated phagocytosis;Focal adhesion;MAPK signaling pathway;MAPK signaling pathway - yeast;Natural killer cell mediated cytotoxicity;Re
Measles;RNA transport
Non-homologous end-joining;Primary immunodeficiency
Cell cycle;MAPK signaling pathway;Progesterone-mediated oocyte maturation
Cell cycle;MAPK signaling pathway;Progesterone-mediated oocyte maturation
Parkinson's disease
Parkinson's disease
Spliceosome
Spliceosome
Spliceosome
Spliceosome
Spliceosome
Amyotrophic lateral sclerosis (ALS);Protein processing in endoplasmic reticulum

Axon guidance;Fc gamma R-mediated phagocytosis;Regulation of actin cytoskeleton
Amino sugar and nucleotide sugar metabolism;Galactose metabolism

Polito et al. Universal ready-to-use immunotherapeutic approach for the treatment of cancer: expanded and activated polyclonal γδ memory T cells.
Phosphorylation sites

RNA transport
MAPK signaling pathway
MAPK signaling pathway
Antigen processing and presentation;Cell adhesion molecules (CAMs);Hematopoietic cell lineage;Primary immunodeficiency;T cell receptor signaling pathway
Notch signaling pathway
Notch signaling pathway
Notch signaling pathway
Notch signaling pathway
Notch signaling pathway
Non-homologous end-joining

Phosphorylation sites

Ribosome biogenesis in eukaryotes;RNA transport
Protein processing in endoplasmic reticulum
Ribosome
ko05152;RNA degradation;Type I diabetes mellitus
Apoptosis;Insulin signaling pathway
Apoptosis;Insulin signaling pathway

Polito et al. Universal ready-to-use immunotherapeutic approach for the treatment of cancer: expanded and activated polyclonal γδ memory T cells.
Phosphorylation sites

ocessing and presentation;Autoimmune thyroid disease;Cell adhesion molecules (CAMs);Endocytosis;Graft-versus-host disease;Natural killer cell mediated cytotoxicity;Phagosome;Type I diabe
RNA transport;Viral myocarditis
AMs);ko05152;Leishmaniasis;Leukocyte transendothelial migration;Malaria;Natural killer cell mediated cytotoxicity;Phagosome;Regulation of actin cytoskeleton;Rheumatoid arthritis;Staphyloc
Apoptosis;Chagas disease (American trypanosomiasis);ko05152;Leishmaniasis;Measles;Neurotrophin signaling pathway;Toll-like receptor signaling pathway;Toxoplasmosis
Ribosome
Ubiquitin mediated proteolysis
MAPK signaling pathway
MAPK signaling pathway
Focal adhesion;MAPK signaling pathway
Protein processing in endoplasmic reticulum
Protein processing in endoplasmic reticulum
Protein processing in endoplasmic reticulum
RNA degradation

Polito et al. Universal ready-to-use immunotherapeutic approach for the treatment of cancer: expanded and activated polyclonal γδ memory T cells.
Phosphorylation sites

MAPK signaling pathway
MAPK signaling pathway
RIG-I-like receptor signaling pathway
mRNA surveillance pathway;RNA transport;Spliceosome
mRNA surveillance pathway;RNA transport;Spliceosome
mRNA surveillance pathway;RNA transport;Spliceosome
mRNA surveillance pathway;RNA transport;Spliceosome
mRNA surveillance pathway;RNA transport;Spliceosome
mRNA surveillance pathway;RNA transport;Spliceosome
mRNA surveillance pathway;RNA transport;Spliceosome
mRNA surveillance pathway;RNA transport;Spliceosome
mRNA surveillance pathway;RNA transport;Spliceosome

mRNA surveillance pathway;RNA transport;Spliceosome
mRNA surveillance pathway;RNA transport;Spliceosome
mRNA surveillance pathway;RNA transport;Spliceosome
mRNA surveillance pathway;RNA transport;Spliceosome
mRNA surveillance pathway;RNA transport;Spliceosome
mRNA surveillance pathway;RNA transport;Spliceosome
mRNA surveillance pathway;RNA transport;Spliceosome
mRNA surveillance pathway;RNA transport;Spliceosome
mRNA surveillance pathway;RNA transport;Spliceosome
mRNA surveillance pathway;RNA transport;Spliceosome
mRNA surveillance pathway;RNA transport;Spliceosome
mRNA surveillance pathway;RNA transport;Spliceosome

Polito et al. Universal ready-to-use immunotherapeutic approach for the treatment of cancer: expanded and activated polyclonal γδ memory T cells.
Phosphorylation sites

Ribosome biogenesis in eukaryotes
Ribosome biogenesis in eukaryotes
Ribosome biogenesis in eukaryotes
Ribosome biogenesis in eukaryotes
Ribosome biogenesis in eukaryotes
Ribosome biogenesis in eukaryotes
Ribosome biogenesis in eukaryotes
Ribosome biogenesis in eukaryotes
Ribosome biogenesis in eukaryotes
Ribosome biogenesis in eukaryotes
Glutathione metabolism;Pentose phosphate pathway
Spliceosome
Spliceosome

Polito et al. Universal ready-to-use immunotherapeutic approach for the treatment of cancer: expanded and activated polyclonal γδ memory T cells.
Phosphorylation sites

mRNA surveillance pathway
mTOR signaling pathway;RNA transport
mTOR signaling pathway;RNA transport
mTOR signaling pathway;RNA transport
mTOR signaling pathway;RNA transport
mTOR signaling pathway;RNA transport
mTOR signaling pathway;RNA transport
SNARE interactions in vesicular transport
Inositol phosphate metabolism;Phagosome;Phosphatidylinositol signaling system;Regulation of actin cytoskeleton
osterone-regulated sodium reabsorption;Endometrial cancer;Focal adhesion;Insulin signaling pathway;mTOR signaling pathway;Non-small cell lung cancer;PPAR signaling pathway;Prostate car

RNA transport;Viral myocarditis
RNA transport;Viral myocarditis
RNA transport;Viral myocarditis
RNA transport;Viral myocarditis
RNA transport;Viral myocarditis
RNA transport;Viral myocarditis
RNA transport;Viral myocarditis
Cell cycle - yeast
Tight junction
Tight junction

Polito et al. Universal ready-to-use immunotherapeutic approach for the treatment of cancer: expanded and activated polyclonal γδ memory T cells.
Phosphorylation sites

Homologous recombination
Adherens junction
Adherens junction
Adherens junction
Adherens junction
Adherens junction
Endocytosis
mRNA surveillance pathway
mRNA surveillance pathway
Endocytosis
Endocytosis

Polito et al. Universal ready-to-use immunotherapeutic approach for the treatment of cancer: expanded and activated polyclonal γδ memory T cells.
Phosphorylation sites

Chagas disease (American trypanosomiasis);Hematopoietic cell lineage;Measles;Primary immunodeficiency;T cell receptor signaling pathway
Ribosome biogenesis in eukaryotes;RNA transport
One carbon pool by folate
One carbon pool by folate

Polito et al. Universal ready-to-use immunotherapeutic approach for the treatment of cancer: expanded and activated polyclonal γδ memory T cells.
Phosphorylation sites

Porphyrin and chlorophyll metabolism
Regulation of actin cytoskeleton
Regulation of actin cytoskeleton
GnRH signaling pathway;MAPK signaling pathway
GnRH signaling pathway;MAPK signaling pathway
Gap junction;Pathogenic Escherichia coli infection;Phagosome
Gap junction;Pathogenic Escherichia coli infection;Phagosome
Gap junction;Pathogenic Escherichia coli infection;Phagosome
Valine, leucine and isoleucine degradation
Valine, leucine and isoleucine degradation
Spliceosome
Base excision repair
Base excision repair
NOD-like receptor signaling pathway
NOD-like receptor signaling pathway
Spliceosome

Polito et al. Universal ready-to-use immunotherapeutic approach for the treatment of cancer: expanded and activated polyclonal γδ memory T cells.
Phosphorylation sites

RNA degradation
RNA degradation
MAPK signaling pathway
Endocytosis
RNA transport
Spliceosome

Polito et al. Universal ready-to-use immunotherapeutic approach for the treatment of cancer: expanded and activated polyclonal γδ memory T cells.

Phosphorylation sites

Purine metabolism
is;Focal adhesion;Glioma;Hepatitis C;Inositol phosphate metabolism;Insulin signaling pathway;Jak-STAT signaling pathway;Leukocyte transendothelial migration;Measles;Melanoma;mTOR sig
Fructose and mannose metabolism
Fructose and mannose metabolism
Vascular smooth muscle contraction

Polito et al. Universal ready-to-use immunotherapeutic approach for the treatment of cancer: expanded and activated polyclonal γδ memory T cells.
Phosphorylation sites

Protein processing in endoplasmic reticulum
vay;Focal adhesion;Gap junction;Glioma;GnRH signaling pathway;Hepatitis C;Insulin signaling pathway;Jak-STAT signaling pathway;MAPK signaling pathway;MAPK signaling pathway - fly;Natur
vay;Focal adhesion;Gap junction;Glioma;GnRH signaling pathway;Hepatitis C;Insulin signaling pathway;Jak-STAT signaling pathway;MAPK signaling pathway;MAPK signaling pathway - fly;Natur
vay;Focal adhesion;Gap junction;Glioma;GnRH signaling pathway;Hepatitis C;Insulin signaling pathway;Jak-STAT signaling pathway;MAPK signaling pathway;MAPK signaling pathway - fly;Natur
vay;Focal adhesion;Gap junction;Glioma;GnRH signaling pathway;Hepatitis C;Insulin signaling pathway;Jak-STAT signaling pathway;MAPK signaling pathway;MAPK signaling pathway - fly;Natur
vay;Focal adhesion;Gap junction;Glioma;GnRH signaling pathway;Hepatitis C;Insulin signaling pathway;Jak-STAT signaling pathway;MAPK signaling pathway;MAPK signaling pathway - fly;Natur
vay;Focal adhesion;Gap junction;Glioma;GnRH signaling pathway;Hepatitis C;Insulin signaling pathway;Jak-STAT signaling pathway;MAPK signaling pathway;MAPK signaling pathway - fly;Natur
vay;Focal adhesion;Gap junction;Glioma;GnRH signaling pathway;Hepatitis C;Insulin signaling pathway;Jak-STAT signaling pathway;MAPK signaling pathway;MAPK signaling pathway - fly;Natur
Alzheimer's disease;Calcium signaling pathway;Pancreatic secretion
MAPK signaling pathway

Polito et al. Universal ready-to-use immunotherapeutic approach for the treatment of cancer: expanded and activated polyclonal γδ memory T cells.
Phosphorylation sites

Focal adhesion;MAPK signaling pathway
ignaling pathway;ko05152;Long-term potentiation;Melanogenesis;Neurotrophin signaling pathway;Olfactory transduction;Oocyte meiosis;Phosphatidylinositol signaling system;Phototransduc
Colorectal cancer;Endometrial cancer;Mismatch repair;Pathways in cancer
Spliceosome
Spliceosome
Spliceosome
Spliceosome

ECM-receptor interaction;Hematopoietic cell lineage;Shigellosis
ECM-receptor interaction;Hematopoietic cell lineage;Shigellosis
ECM-receptor interaction;Hematopoietic cell lineage;Shigellosis
ECM-receptor interaction;Hematopoietic cell lineage;Shigellosis
Vascular smooth muscle contraction
Vascular smooth muscle contraction
Ribosome
Ribosome
mRNA surveillance pathway;RNA transport
mRNA surveillance pathway;RNA transport
Insulin signaling pathway;mTOR signaling pathway;p53 signaling pathway
Insulin signaling pathway;mTOR signaling pathway;p53 signaling pathway
Insulin signaling pathway;mTOR signaling pathway;p53 signaling pathway
Insulin signaling pathway;mTOR signaling pathway;p53 signaling pathway
Insulin signaling pathway;mTOR signaling pathway;p53 signaling pathway
Insulin signaling pathway;mTOR signaling pathway;p53 signaling pathway

Insulin signaling pathway;mTOR signaling pathway;p53 signaling pathway
Sulfur relay system
Sulfur relay system
Circadian rhythm - mammal;Gap junction;Hedgehog signaling pathway

N-Glycan biosynthesis;Various types of N-glycan biosynthesis
Long-term depression;Long-term potentiation;MAPK signaling pathway;Melanoma;mTOR signaling pathway;Natural killer cell mediated cytotoxicity;Neurotrophin signaling pathway;Non-small
Long-term depression;Long-term potentiation;MAPK signaling pathway;Melanoma;mTOR signaling pathway;Natural killer cell mediated cytotoxicity;Neurotrophin signaling pathway;Non-small
Long-term depression;Long-term potentiation;MAPK signaling pathway;Melanoma;mTOR signaling pathway;Natural killer cell mediated cytotoxicity;Neurotrophin signaling pathway;Non-small
Endocytosis

Polito et al. Universal ready-to-use immunotherapeutic approach for the treatment of cancer: expanded and activated polyclonal γδ memory T cells.

Phosphorylation sites

ABC transporters;Vitamin digestion and absorption
Adherens junction;Leukocyte transendothelial migration;Tight junction
Adherens junction;Leukocyte transendothelial migration;Tight junction
Adherens junction;Leukocyte transendothelial migration;Tight junction
Adherens junction;Leukocyte transendothelial migration;Tight junction
Adherens junction;Leukocyte transendothelial migration;Tight junction
Adherens junction;Leukocyte transendothelial migration;Tight junction
Adherens junction;Leukocyte transendothelial migration;Tight junction
Adherens junction;Leukocyte transendothelial migration;Tight junction
Amyotrophic lateral sclerosis (ALS)
Axon guidance

Polito et al. Universal ready-to-use immunotherapeutic approach for the treatment of cancer: expanded and activated polyclonal γδ memory T cells.
Phosphorylation sites

Adipocytokine signaling pathway
MAPK signaling pathway
MAPK signaling pathway
MAPK signaling pathway
MAPK signaling pathway
osis;Focal adhesion;Glioma;Hepatitis C;Insulin signaling pathway;Jak-STAT signaling pathway;Leukocyte transendothelial migration;Measles;Melanoma;mTOR signaling pathway;Natural killer ce
osis;Focal adhesion;Glioma;Hepatitis C;Insulin signaling pathway;Jak-STAT signaling pathway;Leukocyte transendothelial migration;Measles;Melanoma;mTOR signaling pathway;Natural killer ce
osis;Focal adhesion;Glioma;Hepatitis C;Insulin signaling pathway;Jak-STAT signaling pathway;Leukocyte transendothelial migration;Measles;Melanoma;mTOR signaling pathway;Natural killer ce
Ribosome
Ribosome
Spliceosome
Spliceosome
Ribosome

Polito et al. Universal ready-to-use immunotherapeutic approach for the treatment of cancer: expanded and activated polyclonal γδ memory T cells.

Phosphorylation sites

Focal adhesion;Leukocyte transendothelial migration;Regulation of actin cytoskeleton;Tight junction;Vascular smooth muscle contraction
SNARE interactions in vesicular transport
Systemic lupus erythematosus
Acute myeloid leukemia;Chronic myeloid leukemia;ErbB signaling pathway;Jak-STAT signaling pathway;ko05152;Measles;Pathways in cancer
Acute myeloid leukemia;Chronic myeloid leukemia;ErbB signaling pathway;Jak-STAT signaling pathway;ko05152;Measles;Pathways in cancer
Acute myeloid leukemia;Chemokine signaling pathway;Chronic myeloid leukemia;ErbB signaling pathway;Jak-STAT signaling pathway;ko05152;Measles;Pathways in cancer
RNA transport
RNA transport
on;Dilated cardiomyopathy;Focal adhesion;Hypertrophic cardiomyopathy (HCM);Leukocyte transendothelial migration;Pathogenic Escherichia coli infection;Phagosome;Phototransduction - fly
on;Dilated cardiomyopathy;Focal adhesion;Hypertrophic cardiomyopathy (HCM);Leukocyte transendothelial migration;Pathogenic Escherichia coli infection;Phagosome;Phototransduction - fly
Adipocytokine signaling pathway;mTOR signaling pathway

Polito et al. Universal ready-to-use immunotherapeutic approach for the treatment of cancer: expanded and activated polyclonal γδ memory T cells.
Phosphorylation sites

MAPK signaling pathway
MAPK signaling pathway
MAPK signaling pathway
Natural killer cell mediated cytotoxicity;Osteoclast differentiation
Spliceosome
Spliceosome
Spliceosome
Spliceosome
Spliceosome
Spliceosome
Spliceosome
Spliceosome
ko05152;Regulation of actin cytoskeleton;Vascular smooth muscle contraction
ko05152;Regulation of actin cytoskeleton;Vascular smooth muscle contraction
ko05152;Regulation of actin cytoskeleton;Vascular smooth muscle contraction
Spliceosome
Spliceosome
Spliceosome
mRNA surveillance pathway;RNA transport;Spliceosome
Ribosome

Polito et al. Universal ready-to-use immunotherapeutic approach for the treatment of cancer: expanded and activated polyclonal γδ memory T cells.
Phosphorylation sites

Ribosome biogenesis in eukaryotes
Ribosome biogenesis in eukaryotes
Calcium signaling pathway;Fc gamma R-mediated phagocytosis;ko05152;Sphingolipid metabolism;VEGF signaling pathway
Calcium signaling pathway;Fc gamma R-mediated phagocytosis;ko05152;Sphingolipid metabolism;VEGF signaling pathway
Ether lipid metabolism;Peroxisome
Ether lipid metabolism;Peroxisome
SNARE interactions in vesicular transport
SNARE interactions in vesicular transport
SNARE interactions in vesicular transport
Lysosome
Proteasome

Polito et al. Universal ready-to-use immunotherapeutic approach for the treatment of cancer: expanded and activated polyclonal γδ memory T cells.
Phosphorylation sites

Basal transcription factors;Huntington's disease
Apoptosis
RNA transport
Bacterial invasion of epithelial cells;Endocrine and other factor-regulated calcium reabsorption;Endocytosis;Fc gamma R-mediated phagocytosis
Bacterial invasion of epithelial cells;Endocrine and other factor-regulated calcium reabsorption;Endocytosis;Fc gamma R-mediated phagocytosis
Bacterial invasion of epithelial cells;Endocrine and other factor-regulated calcium reabsorption;Endocytosis;Fc gamma R-mediated phagocytosis
Inositol phosphate metabolism;Phosphatidylinositol signaling system
Ribosome biogenesis in eukaryotes
Ribosome biogenesis in eukaryotes
Ribosome biogenesis in eukaryotes
Ribosome biogenesis in eukaryotes
Ribosome biogenesis in eukaryotes
Ribosome biogenesis in eukaryotes
Ribosome biogenesis in eukaryotes

Lysosome
Lysosome

Polito et al. Universal ready-to-use immunotherapeutic approach for the treatment of cancer: expanded and activated polyclonal γδ memory T cells.
Phosphorylation sites

Cell cycle;Cell cycle - yeast;Meiosis - yeast;p53 signaling pathway
Cell cycle;Cell cycle - yeast;Meiosis - yeast;p53 signaling pathway
Natural killer cell mediated cytotoxicity
cell signaling in Helicobacter pylori infection;Hepatitis C;Insulin signaling pathway;MAPK signaling pathway;Neurotrophin signaling pathway;NOD-like receptor signaling pathway;Osteoclast diff
Focal adhesion;Long-term potentiation;Regulation of actin cytoskeleton;Vascular smooth muscle contraction
Focal adhesion;Long-term potentiation;Regulation of actin cytoskeleton;Vascular smooth muscle contraction
Focal adhesion;Long-term potentiation;Regulation of actin cytoskeleton;Vascular smooth muscle contraction
Focal adhesion;Long-term potentiation;Regulation of actin cytoskeleton;Vascular smooth muscle contraction
Focal adhesion;Long-term potentiation;Regulation of actin cytoskeleton;Vascular smooth muscle contraction
Focal adhesion;Long-term potentiation;Regulation of actin cytoskeleton;Vascular smooth muscle contraction
Spliceosome

Polito et al. Universal ready-to-use immunotherapeutic approach for the treatment of cancer: expanded and activated polyclonal γδ memory T cells.
Phosphorylation sites

Spliceosome
Lysine degradation
Inositol phosphate metabolism;Phosphatidylinositol signaling system
Inositol phosphate metabolism;Phosphatidylinositol signaling system
ko05152;Vascular smooth muscle contraction
ko05152;Vascular smooth muscle contraction
Bacterial invasion of epithelial cells;Fc gamma R-mediated phagocytosis;Pathogenic Escherichia coli infection;Regulation of actin cytoskeleton;Shigellosis
Bacterial invasion of epithelial cells;Fc gamma R-mediated phagocytosis;Pathogenic Escherichia coli infection;Regulation of actin cytoskeleton;Shigellosis
Cytosolic DNA-sensing pathway;Purine metabolism;Pyrimidine metabolism;RNA polymerase
mRNA surveillance pathway;RNA transport
mRNA surveillance pathway;RNA transport
mRNA surveillance pathway;RNA transport
Phagosome;SNARE interactions in vesicular transport

Circadian rhythm - fly;Circadian rhythm - mammal
Endocytosis;Fc gamma R-mediated phagocytosis
Spliceosome
Proteasome
Spliceosome
naling pathway;Measles;NOD-like receptor signaling pathway;Osteoclast differentiation;RIG-I-like receptor signaling pathway;T cell receptor signaling pathway;Toll-like receptor signaling path
naling pathway;Measles;NOD-like receptor signaling pathway;Osteoclast differentiation;RIG-I-like receptor signaling pathway;T cell receptor signaling pathway;Toll-like receptor signaling path
naling pathway;Measles;NOD-like receptor signaling pathway;Osteoclast differentiation;RIG-I-like receptor signaling pathway;T cell receptor signaling pathway;Toll-like receptor signaling path
ko05152;Neurotrophin signaling pathway;NOD-like receptor signaling pathway;Shigellosis
Spliceosome

Polito et al. Universal ready-to-use immunotherapeutic approach for the treatment of cancer: expanded and activated polyclonal γδ memory T cells.
Phosphorylation sites

Lysine degradation
Chemokine signaling pathway;Endometrial cancer;Neurotrophin signaling pathway;Non-small cell lung cancer
Chemokine signaling pathway;Endometrial cancer;Neurotrophin signaling pathway;Non-small cell lung cancer
Cytokine-cytokine receptor interaction
Fc epsilon RI signaling pathway;Fc gamma R-mediated phagocytosis;Natural killer cell mediated cytotoxicity;T cell receptor signaling pathway
Fc epsilon RI signaling pathway;Fc gamma R-mediated phagocytosis;Natural killer cell mediated cytotoxicity;T cell receptor signaling pathway
Fc epsilon RI signaling pathway;Fc gamma R-mediated phagocytosis;Natural killer cell mediated cytotoxicity;T cell receptor signaling pathway
Fc epsilon RI signaling pathway;Fc gamma R-mediated phagocytosis;Natural killer cell mediated cytotoxicity;T cell receptor signaling pathway
NOD-like receptor signaling pathway
NOD-like receptor signaling pathway
NOD-like receptor signaling pathway
NOD-like receptor signaling pathway
NOD-like receptor signaling pathway
ErbB signaling pathway;Pathogenic Escherichia coli infection;T cell receptor signaling pathway
Cell cycle;Cell cycle - yeast

Polito et al. Universal ready-to-use immunotherapeutic approach for the treatment of cancer: expanded and activated polyclonal γδ memory T cells.
Phosphorylation sites

Adipocytokine signaling pathway;Hypertrophic cardiomyopathy (HCM);Insulin signaling pathway
Aminoacyl-tRNA biosynthesis
Cell cycle;Cell cycle - yeast
Spliceosome
Spliceosome

Polito et al. Universal ready-to-use immunotherapeutic approach for the treatment of cancer: expanded and activated polyclonal γδ memory T cells.
Phosphorylation sites

Cell cycle
Insulin signaling pathway;mTOR signaling pathway;RNA transport
Insulin signaling pathway;mTOR signaling pathway;RNA transport

Polito et al. Universal ready-to-use immunotherapeutic approach for the treatment of cancer: expanded and activated polyclonal γδ memory T cells.
Phosphorylation sites

N-Glycan biosynthesis
Ribosome biogenesis in eukaryotes
Ribosome biogenesis in eukaryotes
Ribosome biogenesis in eukaryotes
Ribosome biogenesis in eukaryotes
Ribosome biogenesis in eukaryotes
Ribosome biogenesis in eukaryotes
Ribosome biogenesis in eukaryotes
Protein processing in endoplasmic reticulum
DNA replication
Homologous recombination
Homologous recombination

Endocytosis
Endocytosis
Systemic lupus erythematosus
Systemic lupus erythematosus
Focal adhesion;MAPK signaling pathway
SNARE interactions in vesicular transport
SNARE interactions in vesicular transport
mTOR signaling pathway;Regulation of autophagy
mTOR signaling pathway;Regulation of autophagy

Polito et al. Universal ready-to-use immunotherapeutic approach for the treatment of cancer: expanded and activated polyclonal γδ memory T cells.
Phosphorylation sites

Phagosome;SNARE interactions in vesicular transport
Phagosome;SNARE interactions in vesicular transport
Phagosome;SNARE interactions in vesicular transport
Spliceosome
Spliceosome
Spliceosome
Spliceosome
Spliceosome
Spliceosome
Spliceosome
Spliceosome
Spliceosome
Spliceosome
Spliceosome
Spliceosome
SNARE interactions in vesicular transport

Polito et al. Universal ready-to-use immunotherapeutic approach for the treatment of cancer: expanded and activated polyclonal γδ memory T cells.

Phosphorylation sites

MAPK signaling pathway;Neurotrophin signaling pathway
Spliceosome
Spliceosome
MAPK signaling pathway;Neurotrophin signaling pathway
T cell receptor signaling pathway
T cell receptor signaling pathway
T cell receptor signaling pathway
T cell receptor signaling pathway
RNA transport
RNA transport
RNA transport
RNA transport
Hepatitis C;Jak-STAT signaling pathway;Pathways in cancer;Small cell lung cancer;Ubiquitin mediated proteolysis
Hepatitis C;Jak-STAT signaling pathway;Pathways in cancer;Small cell lung cancer;Ubiquitin mediated proteolysis

Protein export

Polito et al. Universal ready-to-use immunotherapeutic approach for the treatment of cancer: expanded and activated polyclonal γδ memory T cells.
Phosphorylation sites

Cell cycle;p53 signaling pathway;Progesterone-mediated oocyte maturation
Cell cycle;p53 signaling pathway;Progesterone-mediated oocyte maturation
Ubiquitin mediated proteolysis
Ubiquitin mediated proteolysis
RNA transport
Protein processing in endoplasmic reticulum;Ubiquitin mediated proteolysis
Spliceosome

Polito et al. Universal ready-to-use immunotherapeutic approach for the treatment of cancer: expanded and activated polyclonal γδ memory T cells.
Phosphorylation sites

RNA degradation
RNA degradation
RNA degradation
RNA degradation
Protein processing in endoplasmic reticulum
Nicotinate and nicotinamide metabolism
Nicotinate and nicotinamide metabolism
Nicotinate and nicotinamide metabolism
Adipocytokine signaling pathway;Fatty acid metabolism;Peroxisome;PPAR signaling pathway
RNA degradation
Axon guidance;B cell receptor signaling pathway;Natural killer cell mediated cytotoxicity;T cell receptor signaling pathway;VEGF signaling pathway;Wnt signaling pathway
Axon guidance;B cell receptor signaling pathway;Natural killer cell mediated cytotoxicity;T cell receptor signaling pathway;VEGF signaling pathway;Wnt signaling pathway
Axon guidance;B cell receptor signaling pathway;Natural killer cell mediated cytotoxicity;T cell receptor signaling pathway;VEGF signaling pathway;Wnt signaling pathway
Axon guidance;B cell receptor signaling pathway;Natural killer cell mediated cytotoxicity;T cell receptor signaling pathway;VEGF signaling pathway;Wnt signaling pathway
Axon guidance;B cell receptor signaling pathway;Natural killer cell mediated cytotoxicity;T cell receptor signaling pathway;VEGF signaling pathway;Wnt signaling pathway
Axon guidance;B cell receptor signaling pathway;Natural killer cell mediated cytotoxicity;T cell receptor signaling pathway;VEGF signaling pathway;Wnt signaling pathway
Axon guidance;B cell receptor signaling pathway;Natural killer cell mediated cytotoxicity;T cell receptor signaling pathway;VEGF signaling pathway;Wnt signaling pathway

Glycerophospholipid metabolism;Phosphatidylinositol signaling system
Glycerophospholipid metabolism;Phosphatidylinositol signaling system
Insulin signaling pathway
Ubiquitin mediated proteolysis
Antigen processing and presentation;Protein processing in endoplasmic reticulum
Endocrine and other factor-regulated calcium reabsorption;Endocytosis;Huntington's disease
Apoptosis
Neuroactive ligand-receptor interaction

Polito et al. Universal ready-to-use immunotherapeutic approach for the treatment of cancer: expanded and activated polyclonal γδ memory T cells.
Phosphorylation sites

B cell receptor signaling pathway;ko05152;T cell receptor signaling pathway
Axon guidance;ErbB signaling pathway;Focal adhesion;Regulation of actin cytoskeleton;Renal cell carcinoma;T cell receptor signaling pathway
Axon guidance;ErbB signaling pathway;Focal adhesion;Regulation of actin cytoskeleton;Renal cell carcinoma;T cell receptor signaling pathway
Cell cycle;Cell cycle - yeast;p53 signaling pathway
Alanine, aspartate and glutamate metabolism;Arginine and proline metabolism;D-Glutamine and D-glutamate metabolism;Nitrogen metabolism;Proximal tubule bicarbonate reclamation
Amyotrophic lateral sclerosis (ALS);Huntington's disease;Peroxisome;Prion diseases
xon guidance;Cell cycle;Chronic myeloid leukemia;ErbB signaling pathway;Neurotrophin signaling pathway;Pathogenic Escherichia coli infection;Pathways in cancer;Shigellosis;Viral myocarditi
Carbon fixation in photosynthetic organisms;Glycolysis / Gluconeogenesis
Antigen processing and presentation;Cell adhesion molecules (CAMs);Hematopoietic cell lineage;Primary immunodeficiency;T cell receptor signaling pathway
Arrhythmogenic right ventricular cardiomyopathy (ARVC);Dilated cardiomyopathy;Hypertrophic cardiomyopathy (HCM)
Arrhythmogenic right ventricular cardiomyopathy (ARVC);Dilated cardiomyopathy;Hypertrophic cardiomyopathy (HCM)
Arrhythmogenic right ventricular cardiomyopathy (ARVC);Dilated cardiomyopathy;Hypertrophic cardiomyopathy (HCM)
Arrhythmogenic right ventricular cardiomyopathy (ARVC);Dilated cardiomyopathy;Hypertrophic cardiomyopathy (HCM)
Arrhythmogenic right ventricular cardiomyopathy (ARVC);Dilated cardiomyopathy;Hypertrophic cardiomyopathy (HCM)
Arrhythmogenic right ventricular cardiomyopathy (ARVC);Dilated cardiomyopathy;Hypertrophic cardiomyopathy (HCM)
Arrhythmogenic right ventricular cardiomyopathy (ARVC);Dilated cardiomyopathy;Hypertrophic cardiomyopathy (HCM)
Complement and coagulation cascades
Collecting duct acid secretion
Mineral absorption;Porphyrin and chlorophyll metabolism
g pathway;Hepatitis C;Insulin signaling pathway;ko05152;Long-term depression;Long-term potentiation;MAPK signaling pathway;Melanogenesis;Melanoma;Natural killer cell mediated cytoto
g pathway;Hepatitis C;Insulin signaling pathway;ko05152;Long-term depression;Long-term potentiation;MAPK signaling pathway;Melanogenesis;Melanoma;Natural killer cell mediated cytoto
Carbon fixation in photosynthetic organisms;Fructose and mannose metabolism;Glycolysis / Gluconeogenesis;Pentose phosphate pathway
Carbon fixation in photosynthetic organisms;Fructose and mannose metabolism;Glycolysis / Gluconeogenesis;Pentose phosphate pathway
Carbon fixation in photosynthetic organisms;Fructose and mannose metabolism;Glycolysis / Gluconeogenesis;Pentose phosphate pathway
Drug metabolism - other enzymes;Pyrimidine metabolism
Drug metabolism - other enzymes;Pyrimidine metabolism
Drug metabolism - other enzymes;Pyrimidine metabolism

Polito et al. Universal ready-to-use immunotherapeutic approach for the treatment of cancer: expanded and activated polyclonal γδ memory T cells.
Phosphorylation sites

Drug metabolism - other enzymes;Pyrimidine metabolism
Antigen processing and presentation;ko05152
Antigen processing and presentation;ko05152
Chagas disease (American trypanosomiasis);Hematopoietic cell lineage;Measles;Primary immunodeficiency;T cell receptor signaling pathway
Gap junction;Pathogenic Escherichia coli infection;Phagosome
Gap junction;Pathogenic Escherichia coli infection;Phagosome
Alzheimer's disease;Glycolysis / Gluconeogenesis
Alzheimer's disease;Glycolysis / Gluconeogenesis
Amoebiasis;MAPK signaling pathway;VEGF signaling pathway
Amoebiasis;MAPK signaling pathway;VEGF signaling pathway
Amoebiasis;MAPK signaling pathway;VEGF signaling pathway
omiasis);Chemokine signaling pathway;Gap junction;Gastric acid secretion;Leukocyte transendothelial migration;Long-term depression;Melanogenesis;Phototransduction;Progesterone-mediat
Bile secretion;Gastric acid secretion;Pancreatic secretion;Salivary secretion
Bile secretion;Gastric acid secretion;Pancreatic secretion;Salivary secretion
Bile secretion;Gastric acid secretion;Pancreatic secretion;Salivary secretion
gestion and absorption;Cardiac muscle contraction;Endocrine and other factor-regulated calcium reabsorption;Gastric acid secretion;Mineral absorption;Pancreatic secretion;Protein digestion
Ribosome
Ribosome
Ribosome
Ribosome
Ribosome
Ribosome
Ribosome
Ribosome
Ribosome
ori infection;ErbB signaling pathway;Focal adhesion;GnRH signaling pathway;Leishmaniasis;MAPK signaling pathway;Neurotrophin signaling pathway;Osteoclast differentiation;Pathways in can
ori infection;ErbB signaling pathway;Focal adhesion;GnRH signaling pathway;Leishmaniasis;MAPK signaling pathway;Neurotrophin signaling pathway;Osteoclast differentiation;Pathways in can
Systemic lupus erythematosus
Systemic lupus erythematosus
oma;Leukocyte transendothelial migration;Long-term depression;Long-term potentiation;MAPK signaling pathway;MAPK signaling pathway - yeast;Melanogenesis;Natural killer cell mediated c
oma;Leukocyte transendothelial migration;Long-term depression;Long-term potentiation;MAPK signaling pathway;MAPK signaling pathway - yeast;Melanogenesis;Natural killer cell mediated c

oma;Leukocyte transendothelial migration;Long-term depression;Long-term potentiation;MAPK signaling pathway;MAPK signaling pathway - yeast;Melanogenesis;Natural killer cell mediated c
Hematopoietic cell lineage
Hematopoietic cell lineage
Hematopoietic cell lineage
Natural killer cell mediated cytotoxicity;Osteoclast differentiation;Primary immunodeficiency;T cell receptor signaling pathway
Natural killer cell mediated cytotoxicity;Osteoclast differentiation;Primary immunodeficiency;T cell receptor signaling pathway
Natural killer cell mediated cytotoxicity;Osteoclast differentiation;Primary immunodeficiency;T cell receptor signaling pathway
;Fc epsilon RI signaling pathway;Focal adhesion;Measles;Natural killer cell mediated cytotoxicity;Osteoclast differentiation;Pathogenic Escherichia coli infection;Prion diseases;T cell receptor si
;Fc epsilon RI signaling pathway;Focal adhesion;Measles;Natural killer cell mediated cytotoxicity;Osteoclast differentiation;Pathogenic Escherichia coli infection;Prion diseases;T cell receptor si
;Fc epsilon RI signaling pathway;Focal adhesion;Measles;Natural killer cell mediated cytotoxicity;Osteoclast differentiation;Pathogenic Escherichia coli infection;Prion diseases;T cell receptor si
;Fc epsilon RI signaling pathway;Focal adhesion;Measles;Natural killer cell mediated cytotoxicity;Osteoclast differentiation;Pathogenic Escherichia coli infection;Prion diseases;T cell receptor si
Bladder cancer;Cell cycle;Chronic myeloid leukemia;Glioma;Melanoma;Non-small cell lung cancer;Pancreatic cancer;Pathways in cancer;Prostate cancer;Small cell lung cancer
Bladder cancer;Cell cycle;Chronic myeloid leukemia;Glioma;Melanoma;Non-small cell lung cancer;Pancreatic cancer;Pathways in cancer;Prostate cancer;Small cell lung cancer
Bladder cancer;Cell cycle;Chronic myeloid leukemia;Glioma;Melanoma;Non-small cell lung cancer;Pancreatic cancer;Pathways in cancer;Prostate cancer;Small cell lung cancer
Bladder cancer;Cell cycle;Chronic myeloid leukemia;Glioma;Melanoma;Non-small cell lung cancer;Pancreatic cancer;Pathways in cancer;Prostate cancer;Small cell lung cancer
Cell cycle;Gap junction;Oocyte meiosis;p53 signaling pathway;Progesterone-mediated oocyte maturation
Cell cycle;Gap junction;Oocyte meiosis;p53 signaling pathway;Progesterone-mediated oocyte maturation
Glycolysis / Gluconeogenesis;Methane metabolism;RNA degradation
Glycolysis / Gluconeogenesis;Methane metabolism;RNA degradation
Glycolysis / Gluconeogenesis;Methane metabolism;RNA degradation
Glycolysis / Gluconeogenesis;Methane metabolism;RNA degradation
Gap junction;Pathogenic Escherichia coli infection;Phagosome

Gap junction;Pathogenic Escherichia coli infection;Phagosome
Gap junction;Pathogenic Escherichia coli infection;Phagosome
Purine metabolism
Purine metabolism
Aminoacyl-tRNA biosynthesis;Porphyrin and chlorophyll metabolism
Aminoacyl-tRNA biosynthesis;Porphyrin and chlorophyll metabolism
g and presentation;NOD-like receptor signaling pathway;Pathways in cancer;Plant-pathogen interaction;Progesterone-mediated oocyte maturation;Prostate cancer;Protein processing in endo
g and presentation;NOD-like receptor signaling pathway;Pathways in cancer;Plant-pathogen interaction;Progesterone-mediated oocyte maturation;Prostate cancer;Protein processing in endo
g and presentation;NOD-like receptor signaling pathway;Pathways in cancer;Plant-pathogen interaction;Progesterone-mediated oocyte maturation;Prostate cancer;Protein processing in endo
eptor signaling pathway;Chemokine signaling pathway;Epithelial cell signaling in Helicobacter pylori infection;Fc epsilon RI signaling pathway;Fc gamma R-mediated phagocytosis;Long-term de
Huntington's disease;TGF-beta signaling pathway
Huntington's disease;TGF-beta signaling pathway
g and presentation;NOD-like receptor signaling pathway;Pathways in cancer;Plant-pathogen interaction;Progesterone-mediated oocyte maturation;Prostate cancer;Protein processing in endo
g and presentation;NOD-like receptor signaling pathway;Pathways in cancer;Plant-pathogen interaction;Progesterone-mediated oocyte maturation;Prostate cancer;Protein processing in endo
g and presentation;NOD-like receptor signaling pathway;Pathways in cancer;Plant-pathogen interaction;Progesterone-mediated oocyte maturation;Prostate cancer;Protein processing in endo
g and presentation;NOD-like receptor signaling pathway;Pathways in cancer;Plant-pathogen interaction;Progesterone-mediated oocyte maturation;Prostate cancer;Protein processing in endo
g and presentation;NOD-like receptor signaling pathway;Pathways in cancer;Plant-pathogen interaction;Progesterone-mediated oocyte maturation;Prostate cancer;Protein processing in endo
g and presentation;NOD-like receptor signaling pathway;Pathways in cancer;Plant-pathogen interaction;Progesterone-mediated oocyte maturation;Prostate cancer;Protein processing in endo
Butanoate metabolism;Citrate cycle (TCA cycle);Glycolysis / Gluconeogenesis;Pyruvate metabolism;Valine, leucine and isoleucine biosynthesis
Butanoate metabolism;Citrate cycle (TCA cycle);Glycolysis / Gluconeogenesis;Pyruvate metabolism;Valine, leucine and isoleucine biosynthesis
Spliceosome
Spliceosome
Spliceosome

Polito et al. Universal ready-to-use immunotherapeutic approach for the treatment of cancer: expanded and activated polyclonal γδ memory T cells.
Phosphorylation sites

Glycolysis / Gluconeogenesis;Methane metabolism;RNA degradation
Drug metabolism - cytochrome P450;Glutathione metabolism;Metabolism of xenobiotics by cytochrome P450
Base excision repair
Bacterial invasion of epithelial cells;Endocrine and other factor-regulated calcium reabsorption;Endocytosis;Huntington's disease;Lysosome
Cytokine-cytokine receptor interaction;Hematopoietic cell lineage;Osteoclast differentiation;Rheumatoid arthritis
Spliceosome
Spliceosome
Spliceosome
Spliceosome
Spliceosome
Spliceosome
Spliceosome
Chagas disease (American trypanosomiasis);Hematopoietic cell lineage;Measles;T cell receptor signaling pathway
Base excision repair
Carbon fixation in photosynthetic organisms;Fructose and mannose metabolism;Glycolysis / Gluconeogenesis;Pentose phosphate pathway
Carbon fixation in photosynthetic organisms;Fructose and mannose metabolism;Glycolysis / Gluconeogenesis;Pentose phosphate pathway

Shigellosis;Spliceosome
Shigellosis;Spliceosome
Systemic lupus erythematosus
Insulin signaling pathway;Long-term depression;Long-term potentiation;Melanoma;Natural killer cell mediated cytotoxicity;Non-small cell lung cancer;Pancreatic cancer;Pathways in cancer;Pr
Apoptosis;Insulin signaling pathway
Tight junction
Tight junction
Tight junction
Tight junction
Tight junction
Tight junction
Insulin signaling pathway;Starch and sucrose metabolism
Chronic myeloid leukemia;Pathways in cancer
Chronic myeloid leukemia;Pathways in cancer
Chronic myeloid leukemia;Pathways in cancer
Chronic myeloid leukemia;Pathways in cancer
Chronic myeloid leukemia;Pathways in cancer
Chronic myeloid leukemia;Pathways in cancer

Polito et al. Universal ready-to-use immunotherapeutic approach for the treatment of cancer: expanded and activated polyclonal γδ memory T cells.
Phosphorylation sites

Lysosome
Lysosome
Lysosome
MAPK signaling pathway
mRNA surveillance pathway;RNA degradation;RNA transport
Drug metabolism - other enzymes;Purine metabolism
Drug metabolism - other enzymes;Purine metabolism
MAPK signaling pathway - yeast;Pathways in cancer;RNA transport;Thyroid cancer
MAPK signaling pathway - yeast;Pathways in cancer;RNA transport;Thyroid cancer
MAPK signaling pathway - yeast;Pathways in cancer;RNA transport;Thyroid cancer
MAPK signaling pathway - yeast;Pathways in cancer;RNA transport;Thyroid cancer
MAPK signaling pathway - yeast;Pathways in cancer;RNA transport;Thyroid cancer
Non-homologous end-joining
Non-homologous end-joining
Non-homologous end-joining
Non-homologous end-joining
Non-homologous end-joining
Base excision repair;Primary immunodeficiency
ECM-receptor interaction;Hematopoietic cell lineage
Leishmaniasis;Leukocyte transendothelial migration;Osteoclast differentiation;Phagosome
s (CAMs);Dilated cardiomyopathy;ECM-receptor interaction;Focal adhesion;Hematopoietic cell lineage;Hypertrophic cardiomyopathy (HCM);Intestinal immune network for IgA production;Leis

ocessing and presentation;Autoimmune thyroid disease;Cell adhesion molecules (CAMs);Endocytosis;Graft-versus-host disease;Natural killer cell mediated cytotoxicity;Phagosome;Type I diabe
ocessing and presentation;Autoimmune thyroid disease;Cell adhesion molecules (CAMs);Endocytosis;Graft-versus-host disease;Natural killer cell mediated cytotoxicity;Phagosome;Type I diabe
ocessing and presentation;Autoimmune thyroid disease;Cell adhesion molecules (CAMs);Endocytosis;Graft-versus-host disease;Natural killer cell mediated cytotoxicity;Phagosome;Type I diabe
Insulin signaling pathway;Starch and sucrose metabolism
Insulin signaling pathway;Starch and sucrose metabolism
Apoptosis;Insulin signaling pathway
Apoptosis;Insulin signaling pathway
Basal transcription factors
Cell adhesion molecules (CAMs);Leukocyte transendothelial migration
Bacterial invasion of epithelial cells;Pathogenic Escherichia coli infection;Shigellosis;Tight junction
Bacterial invasion of epithelial cells;Pathogenic Escherichia coli infection;Shigellosis;Tight junction
Caprolactam degradation;Glycerolipid metabolism;Glycolysis / Gluconeogenesis
Chemokine signaling pathway;Fc gamma R-mediated phagocytosis;Leishmaniasis;Leukocyte transendothelial migration;Osteoclast differentiation;Phagosome
Chemokine signaling pathway;Fc gamma R-mediated phagocytosis;Leishmaniasis;Leukocyte transendothelial migration;Osteoclast differentiation;Phagosome
Chemokine signaling pathway;Fc gamma R-mediated phagocytosis;Leishmaniasis;Leukocyte transendothelial migration;Osteoclast differentiation;Phagosome
Carbon fixation in photosynthetic organisms;Glycolysis / Gluconeogenesis;Purine metabolism;Pyruvate metabolism;Type II diabetes mellitus
NOD-like receptor signaling pathway;Pathways in cancer;Plant-pathogen interaction;Prostate cancer;Protein processing in endoplasmic reticulum
Aminoacyl-tRNA biosynthesis

Polito et al. Universal ready-to-use immunotherapeutic approach for the treatment of cancer: expanded and activated polyclonal γδ memory T cells.
Phosphorylation sites

Dorso-ventral axis formation;MAPK signaling pathway - fly
Dorso-ventral axis formation;MAPK signaling pathway - fly
Dorso-ventral axis formation;MAPK signaling pathway - fly
.ong-term depression;Long-term potentiation;MAPK signaling pathway;Melanoma;mTOR signaling pathway;Natural killer cell mediated cytotoxicity;Neurotrophin signaling pathway;Non-small
Ribosome
Cell adhesion molecules (CAMs)
Cell adhesion molecules (CAMs)
Cell adhesion molecules (CAMs)
Cell adhesion molecules (CAMs)
Cell adhesion molecules (CAMs)
Antigen processing and presentation;Huntington's disease;ko05152;Melanogenesis;Osteoclast differentiation;Prostate cancer;Vasopressin-regulated water reabsorption
Antigen processing and presentation;Huntington's disease;ko05152;Melanogenesis;Osteoclast differentiation;Prostate cancer;Vasopressin-regulated water reabsorption
l receptor signaling pathway;Calcium signaling pathway;ko05152;Long-term potentiation;MAPK signaling pathway;Natural killer cell mediated cytotoxicity;Oocyte meiosis;Osteoclast differenti
l receptor signaling pathway;Calcium signaling pathway;ko05152;Long-term potentiation;MAPK signaling pathway;Natural killer cell mediated cytotoxicity;Oocyte meiosis;Osteoclast differenti
ErbB signaling pathway;Pathogenic Escherichia coli infection;T cell receptor signaling pathway
ErbB signaling pathway;Pathogenic Escherichia coli infection;T cell receptor signaling pathway
Alzheimer's disease;Calcium signaling pathway;Pancreatic secretion
MAPK signaling pathway
MAPK signaling pathway
MAPK signaling pathway
MAPK signaling pathway
MAPK signaling pathway

Polito et al. Universal ready-to-use immunotherapeutic approach for the treatment of cancer: expanded and activated polyclonal γδ memory T cells.

Phosphorylation sites

Cytokine-cytokine receptor interaction;Hepatitis C;Jak-STAT signaling pathway;Measles;Natural killer cell mediated cytotoxicity;Osteoclast differentiation;Toll-like receptor signaling pathway
oma;Leukocyte transendothelial migration;Long-term depression;Long-term potentiation;MAPK signaling pathway;MAPK signaling pathway - yeast;Melanogenesis;Natural killer cell mediated c
oma;Leukocyte transendothelial migration;Long-term depression;Long-term potentiation;MAPK signaling pathway;MAPK signaling pathway - yeast;Melanogenesis;Natural killer cell mediated c
MAPK signaling pathway;Osteoclast differentiation
MAPK signaling pathway;Osteoclast differentiation
naling pathway;Hedgehog signaling pathway;Insulin signaling pathway;Long-term potentiation;MAPK signaling pathway;Meiosis - yeast;Melanogenesis;Olfactory transduction;Oocyte meiosis;
Pyrimidine metabolism
Pyrimidine metabolism
Spliceosome
Spliceosome
Spliceosome
Fructose and mannose metabolism;Galactose metabolism;Glycolysis / Gluconeogenesis;Methane metabolism;Pentose phosphate pathway
Proteasome
Adherens junction;Insulin signaling pathway
Adherens junction;Insulin signaling pathway
Adherens junction;Insulin signaling pathway
Adherens junction;Amoebiasis;Bacterial invasion of epithelial cells;Focal adhesion;Leukocyte transendothelial migration;Regulation of actin cytoskeleton;Shigellosis

Polito et al. Universal ready-to-use immunotherapeutic approach for the treatment of cancer: expanded and activated polyclonal γδ memory T cells.
Phosphorylation sites

Glycolysis / Gluconeogenesis;Methane metabolism
Glycolysis / Gluconeogenesis;Methane metabolism
Glycolysis / Gluconeogenesis;Methane metabolism
Base excision repair;DNA replication;Mismatch repair;Nucleotide excision repair
Base excision repair;DNA replication;Mismatch repair;Nucleotide excision repair
Base excision repair;DNA replication;Mismatch repair;Nucleotide excision repair
Base excision repair;DNA replication;Mismatch repair;Nucleotide excision repair
vay;Fc gamma R-mediated phagocytosis;Glioma;Inositol phosphate metabolism;Leukocyte transendothelial migration;Natural killer cell mediated cytotoxicity;Neurotrophin signaling pathway;
vay;Fc gamma R-mediated phagocytosis;Glioma;Inositol phosphate metabolism;Leukocyte transendothelial migration;Natural killer cell mediated cytotoxicity;Neurotrophin signaling pathway;
vay;Fc gamma R-mediated phagocytosis;Glioma;Inositol phosphate metabolism;Leukocyte transendothelial migration;Natural killer cell mediated cytotoxicity;Neurotrophin signaling pathway;
Pathogenic Escherichia coli infection
Pathogenic Escherichia coli infection
Pathogenic Escherichia coli infection
Pathogenic Escherichia coli infection
Pathogenic Escherichia coli infection
Pathogenic Escherichia coli infection
Huntington's disease;Purine metabolism;Pyrimidine metabolism;RNA polymerase
Endometrial cancer;ErbB signaling pathway;Focal adhesion;GnRH signaling pathway;Insulin signaling pathway;Leishmaniasis;MAPK signaling pathway;Prion diseases
Bile secretion;Cardiac muscle contraction;Gastric acid secretion;Pancreatic secretion;Regulation of actin cytoskeleton;Salivary secretion
Bile secretion;Cardiac muscle contraction;Gastric acid secretion;Pancreatic secretion;Regulation of actin cytoskeleton;Salivary secretion
Bile secretion;Cardiac muscle contraction;Gastric acid secretion;Pancreatic secretion;Regulation of actin cytoskeleton;Salivary secretion

Polito et al. Universal ready-to-use immunotherapeutic approach for the treatment of cancer: expanded and activated polyclonal γδ memory T cells.
Phosphorylation sites

Bile secretion;Cardiac muscle contraction;Gastric acid secretion;Pancreatic secretion;Regulation of actin cytoskeleton;Salivary secretion
way;Epithelial cell signaling in Helicobacter pylori infection;Hepatitis C;ko05152;Leishmaniasis;MAPK signaling pathway;Measles;Neurotrophin signaling pathway;NOD-like receptor signaling p
way;Epithelial cell signaling in Helicobacter pylori infection;Hepatitis C;ko05152;Leishmaniasis;MAPK signaling pathway;Measles;Neurotrophin signaling pathway;NOD-like receptor signaling p
way;Epithelial cell signaling in Helicobacter pylori infection;Hepatitis C;ko05152;Leishmaniasis;MAPK signaling pathway;Measles;Neurotrophin signaling pathway;NOD-like receptor signaling p
Leishmaniasis;Leukocyte transendothelial migration;Osteoclast differentiation;Phagosome
Calcium signaling pathway;Pancreatic secretion;Salivary secretion
RNA transport
RNA transport
RNA transport
RNA transport
Endocytosis
Colorectal cancer;Mismatch repair;Pathways in cancer
Lysosome;Phagosome
olecules (CAMs);Leukocyte transendothelial migration;Malaria;Natural killer cell mediated cytotoxicity;Regulation of actin cytoskeleton;Rheumatoid arthritis;Staphylococcus aureus infection;\
Chagas disease (American trypanosomiasis);Natural killer cell mediated cytotoxicity;T cell receptor signaling pathway
Focal adhesion;MAPK signaling pathway
Focal adhesion;MAPK signaling pathway
Focal adhesion;MAPK signaling pathway
Focal adhesion;MAPK signaling pathway
Axon guidance
Complement and coagulation cascades;Neuroactive ligand-receptor interaction;Staphylococcus aureus infection
Complement and coagulation cascades;Neuroactive ligand-receptor interaction;Staphylococcus aureus infection
Complement and coagulation cascades;Neuroactive ligand-receptor interaction;Staphylococcus aureus infection
Complement and coagulation cascades;Neuroactive ligand-receptor interaction;Staphylococcus aureus infection

Polito et al. Universal ready-to-use immunotherapeutic approach for the treatment of cancer: expanded and activated polyclonal γδ memory T cells.
Phosphorylation sites

Calcium signaling pathway;Neuroactive ligand-receptor interaction
Calcium signaling pathway;Huntington's disease;Parkinson's disease
Betalain biosynthesis;Steroid hormone biosynthesis;Tyrosine metabolism
Parkinson's disease;Ubiquitin mediated proteolysis
Parkinson's disease;Ubiquitin mediated proteolysis
Parkinson's disease;Ubiquitin mediated proteolysis
Parkinson's disease;Ubiquitin mediated proteolysis
Purine metabolism;Pyrimidine metabolism
pithelial cells;Chronic myeloid leukemia;Endocytosis;ErbB signaling pathway;Insulin signaling pathway;Jak-STAT signaling pathway;Pathways in cancer;T cell receptor signaling pathway;Ubiquiti
pithelial cells;Chronic myeloid leukemia;Endocytosis;ErbB signaling pathway;Insulin signaling pathway;Jak-STAT signaling pathway;Pathways in cancer;T cell receptor signaling pathway;Ubiquiti
pithelial cells;Chronic myeloid leukemia;Endocytosis;ErbB signaling pathway;Insulin signaling pathway;Jak-STAT signaling pathway;Pathways in cancer;T cell receptor signaling pathway;Ubiquiti
naling pathway;Hedgehog signaling pathway;Insulin signaling pathway;Long-term potentiation;MAPK signaling pathway;Meiosis - yeast;Melanogenesis;Olfactory transduction;Oocyte meiosis;
Aminoacyl-tRNA biosynthesis;Tryptophan metabolism
Ribosome
Acute myeloid leukemia;ErbB signaling pathway;Fc gamma R-mediated phagocytosis;Insulin signaling pathway;mTOR signaling pathway;TGF-beta signaling pathway

Polito et al. Universal ready-to-use immunotherapeutic approach for the treatment of cancer: expanded and activated polyclonal γδ memory T cells.
Phosphorylation sites

Antigen processing and presentation;ko05152
Systemic lupus erythematosus
Systemic lupus erythematosus
Systemic lupus erythematosus
Axon guidance;Fc gamma R-mediated phagocytosis;Regulation of actin cytoskeleton
Axon guidance;Fc gamma R-mediated phagocytosis;Regulation of actin cytoskeleton
Calcium signaling pathway;Pancreatic secretion;Salivary secretion
Calcium signaling pathway;Pancreatic secretion;Salivary secretion
Tight junction;Vascular smooth muscle contraction
Tight junction;Vascular smooth muscle contraction
Tight junction;Vascular smooth muscle contraction
Tight junction;Vascular smooth muscle contraction
Huntington's disease;Purine metabolism;Pyrimidine metabolism;RNA polymerase
Huntington's disease;Purine metabolism;Pyrimidine metabolism;RNA polymerase
Huntington's disease;Purine metabolism;Pyrimidine metabolism;RNA polymerase
Huntington's disease;Purine metabolism;Pyrimidine metabolism;RNA polymerase
Huntington's disease;Purine metabolism;Pyrimidine metabolism;RNA polymerase
Huntington's disease;Purine metabolism;Pyrimidine metabolism;RNA polymerase
Huntington's disease;Purine metabolism;Pyrimidine metabolism;RNA polymerase
Huntington's disease;Purine metabolism;Pyrimidine metabolism;RNA polymerase
Huntington's disease;Purine metabolism;Pyrimidine metabolism;RNA polymerase
Huntington's disease;Purine metabolism;Pyrimidine metabolism;RNA polymerase
Chemokine signaling pathway;Endocytosis;Phototransduction - fly
Chemokine signaling pathway;Endocytosis;Phototransduction - fly
Cell cycle;Cell cycle - yeast;DNA replication;Meiosis - yeast
Cell cycle;Cell cycle - yeast;DNA replication;Meiosis - yeast
Cell cycle;Cell cycle - yeast;DNA replication;Meiosis - yeast
Cell cycle;Cell cycle - yeast;DNA replication;Meiosis - yeast
mune thyroid disease;Chagas disease (American trypanosomiasis);Cytokine-cytokine receptor interaction;Graft-versus-host disease;MAPK signaling pathway;Measles;Natural killer cell mediat
mune thyroid disease;Chagas disease (American trypanosomiasis);Cytokine-cytokine receptor interaction;Graft-versus-host disease;MAPK signaling pathway;Measles;Natural killer cell mediat
mune thyroid disease;Chagas disease (American trypanosomiasis);Cytokine-cytokine receptor interaction;Graft-versus-host disease;MAPK signaling pathway;Measles;Natural killer cell mediat

Proteasome
Proteasome
yopathy (ARVC);Cell adhesion molecules (CAMs);Dilated cardiomyopathy;ECM-receptor interaction;Focal adhesion;Hypertrophic cardiomyopathy (HCM);Intestinal immune network for IgA pr
yopathy (ARVC);Cell adhesion molecules (CAMs);Dilated cardiomyopathy;ECM-receptor interaction;Focal adhesion;Hypertrophic cardiomyopathy (HCM);Intestinal immune network for IgA pr
Leukocyte transendothelial migration;Measles;Regulation of actin cytoskeleton
Leukocyte transendothelial migration;Measles;Regulation of actin cytoskeleton
Leukocyte transendothelial migration;Measles;Regulation of actin cytoskeleton
Cysteine and methionine metabolism
Cysteine and methionine metabolism
Cysteine and methionine metabolism
Cysteine and methionine metabolism
Cysteine and methionine metabolism
Spliceosome
Spliceosome
Spliceosome
Ribosome
Ribosome
Cytokine-cytokine receptor interaction
Pathways in cancer;Renal cell carcinoma
DNA replication;Homologous recombination;Mismatch repair;Nucleotide excision repair
Purine metabolism;Pyrimidine metabolism
Purine metabolism;Pyrimidine metabolism
Alanine, aspartate and glutamate metabolism;Pyrimidine metabolism
Alanine, aspartate and glutamate metabolism;Pyrimidine metabolism
Alanine, aspartate and glutamate metabolism;Pyrimidine metabolism
Alanine, aspartate and glutamate metabolism;Pyrimidine metabolism
Purine metabolism
Antigen processing and presentation;Phagosome;Protein processing in endoplasmic reticulum
Antigen processing and presentation;Phagosome;Protein processing in endoplasmic reticulum

Polito et al. Universal ready-to-use immunotherapeutic approach for the treatment of cancer: expanded and activated polyclonal γδ memory T cells.

Phosphorylation sites

Antigen processing and presentation;Phagosome;Protein processing in endoplasmic reticulum
Calcium signaling pathway;Inositol phosphate metabolism;Phosphatidylinositol signaling system
Calcium signaling pathway;Inositol phosphate metabolism;Phosphatidylinositol signaling system
Calcium signaling pathway;Inositol phosphate metabolism;Phosphatidylinositol signaling system
Proteasome
Proteasome
Proteasome
MAPK signaling pathway
Cell cycle;TGF-beta signaling pathway
Adherens junction;B cell receptor signaling pathway;Jak-STAT signaling pathway;Leishmaniasis;Natural killer cell mediated cytotoxicity;T cell receptor signaling pathway
Adherens junction;B cell receptor signaling pathway;Jak-STAT signaling pathway;Leishmaniasis;Natural killer cell mediated cytotoxicity;T cell receptor signaling pathway
Adherens junction;B cell receptor signaling pathway;Jak-STAT signaling pathway;Leishmaniasis;Natural killer cell mediated cytotoxicity;T cell receptor signaling pathway
Adherens junction;B cell receptor signaling pathway;Jak-STAT signaling pathway;Leishmaniasis;Natural killer cell mediated cytotoxicity;T cell receptor signaling pathway
ithelial cells;Chemokine signaling pathway;Chronic myeloid leukemia;ErbB signaling pathway;Focal adhesion;Glioma;Insulin signaling pathway;Natural killer cell mediated cytotoxicity;Neurotro
Biosynthesis of ansamycins;Carbon fixation in photosynthetic organisms;Pentose phosphate pathway
Biosynthesis of ansamycins;Carbon fixation in photosynthetic organisms;Pentose phosphate pathway
Acute myeloid leukemia;Endocytosis;Pathways in cancer;Ubiquitin mediated proteolysis
Acute myeloid leukemia;Endocytosis;Pathways in cancer;Ubiquitin mediated proteolysis
Acute myeloid leukemia;Endocytosis;Pathways in cancer;Ubiquitin mediated proteolysis
Acute myeloid leukemia;Endocytosis;Pathways in cancer;Ubiquitin mediated proteolysis
Acute myeloid leukemia;Endocytosis;Pathways in cancer;Ubiquitin mediated proteolysis
Acute myeloid leukemia;Endocytosis;Pathways in cancer;Ubiquitin mediated proteolysis
Acute myeloid leukemia;Endocytosis;Pathways in cancer;Ubiquitin mediated proteolysis
Acute myeloid leukemia;Endocytosis;Pathways in cancer;Ubiquitin mediated proteolysis
Acute myeloid leukemia;Endocytosis;Pathways in cancer;Ubiquitin mediated proteolysis
Acute myeloid leukemia;Endocytosis;Pathways in cancer;Ubiquitin mediated proteolysis
Acute myeloid leukemia;Endocytosis;Pathways in cancer;Ubiquitin mediated proteolysis
Acute myeloid leukemia;Endocytosis;Pathways in cancer;Ubiquitin mediated proteolysis

Polito et al. Universal ready-to-use immunotherapeutic approach for the treatment of cancer: expanded and activated polyclonal γδ memory T cells.
Phosphorylation sites

Acute myeloid leukemia;Endocytosis;Pathways in cancer;Ubiquitin mediated proteolysis
Fc gamma R-mediated phagocytosis
Fc gamma R-mediated phagocytosis
Fc gamma R-mediated phagocytosis
Porphyrin and chlorophyll metabolism;Riboflavin metabolism
Porphyrin and chlorophyll metabolism;Riboflavin metabolism
Ribosome
ko05152;Phagosome
Apoptosis;Insulin signaling pathway
Glutathione metabolism;p53 signaling pathway;Purine metabolism;Pyrimidine metabolism
Drug metabolism - cytochrome P450;Methane metabolism
nediated phagocytosis;Focal adhesion;Glioma;Hepatitis C;Insulin signaling pathway;Jak-STAT signaling pathway;ko05152;MAPK signaling pathway;Measles;Melanoma;mTOR signaling pathway
nediated phagocytosis;Focal adhesion;Glioma;Hepatitis C;Insulin signaling pathway;Jak-STAT signaling pathway;ko05152;MAPK signaling pathway;Measles;Melanoma;mTOR signaling pathway
Alzheimer's disease;Cardiac muscle contraction;Huntington's disease;Oxidative phosphorylation;Parkinson's disease

Prion diseases
Chemokine signaling pathway;Cytokine-cytokine receptor interaction
Cell adhesion molecules (CAMs)
Cell adhesion molecules (CAMs)
Pyrimidine metabolism
Pyrimidine metabolism
Cell cycle;Cell cycle - yeast
Cell cycle;Cell cycle - yeast;DNA replication;Meiosis - yeast
Cell cycle;Cell cycle - yeast;DNA replication;Meiosis - yeast
Cell cycle;Cell cycle - yeast;DNA replication;Meiosis - yeast
Cell cycle;Cell cycle - yeast;DNA replication;Meiosis - yeast
Cell cycle;Cell cycle - yeast;DNA replication;Meiosis - yeast
Cell cycle;Cell cycle - yeast;DNA replication;Meiosis - yeast
Cyanoamino acid metabolism;Glycine, serine and threonine metabolism;Methane metabolism;One carbon pool by folate
Antigen processing and presentation
Antigen processing and presentation
Antigen processing and presentation
Chemokine signaling pathway;Endocytosis
ens junction;Arrhythmogenic right ventricular cardiomyopathy (ARVC);Bacterial invasion of epithelial cells;Endometrial cancer;Leukocyte transendothelial migration;Pathways in cancer;Tight ju

Polito et al. Universal ready-to-use immunotherapeutic approach for the treatment of cancer: expanded and activated polyclonal γδ memory T cells.
Phosphorylation sites

DNA replication;Mismatch repair;Nucleotide excision repair
DNA replication;Mismatch repair;Nucleotide excision repair
DNA replication;Mismatch repair;Nucleotide excision repair
DNA replication;Mismatch repair;Nucleotide excision repair
DNA replication;Mismatch repair;Nucleotide excision repair
Basal transcription factors
Tight junction;Viral myocarditis
Tight junction;Viral myocarditis
Tight junction;Viral myocarditis
Tight junction;Viral myocarditis
Tight junction;Viral myocarditis
RNA transport
RNA transport
RNA transport
RNA transport
RNA transport
-term depression;Long-term potentiation;MAPK signaling pathway;Melanogenesis;Melanoma;Natural killer cell mediated cytotoxicity;Neurotrophin signaling pathway;Non-small cell lung canc
-term depression;Long-term potentiation;MAPK signaling pathway;Melanogenesis;Melanoma;Natural killer cell mediated cytotoxicity;Neurotrophin signaling pathway;Non-small cell lung canc
-term depression;Long-term potentiation;MAPK signaling pathway;Melanogenesis;Melanoma;Natural killer cell mediated cytotoxicity;Neurotrophin signaling pathway;Non-small cell lung canc
Ribosome

sugar and nucleotide sugar metabolism;Galactose metabolism;Glycolysis / Gluconeogenesis;Pentose phosphate pathway;Purine metabolism;Starch and sucrose metabolism;Streptomycin biosy
an trypanosomiasis);Chronic myeloid leukemia;Colorectal cancer;Cytokine-cytokine receptor interaction;Endocytosis;MAPK signaling pathway;Osteoclast differentiation;Pancreatic cancer;Path
an trypanosomiasis);Chronic myeloid leukemia;Colorectal cancer;Cytokine-cytokine receptor interaction;Endocytosis;MAPK signaling pathway;Osteoclast differentiation;Pancreatic cancer;Path
RNA transport
Pentose phosphate pathway
Spliceosome
Spliceosome
Spliceosome
Spliceosome
Spliceosome
Spliceosome
Spliceosome
Spliceosome
Spliceosome
Spliceosome
Spliceosome
Ubiquitin mediated proteolysis
Ubiquitin mediated proteolysis
Collecting duct acid secretion;Epithelial cell signaling in Helicobacter pylori infection;Oxidative phosphorylation;Phagosome;Rheumatoid arthritis;Vibrio cholerae infection
mRNA surveillance pathway;RNA transport;Spliceosome
Ribosome
Ribosome
Ribosome
Base excision repair;DNA replication;Non-homologous end-joining

Polito et al. Universal ready-to-use immunotherapeutic approach for the treatment of cancer: expanded and activated polyclonal γδ memory T cells.
Phosphorylation sites

Proteasome
te myeloid leukemia;Adipocytokine signaling pathway;Chemokine signaling pathway;Hepatitis C;Jak-STAT signaling pathway;ko05152;Measles;Pancreatic cancer;Pathways in cancer;Toxoplasm
te myeloid leukemia;Adipocytokine signaling pathway;Chemokine signaling pathway;Hepatitis C;Jak-STAT signaling pathway;ko05152;Measles;Pancreatic cancer;Pathways in cancer;Toxoplasm
Endocytosis
Endocytosis
Peroxisome
Nucleotide excision repair
Dorso-ventral axis formation;MAPK signaling pathway - fly
Aminoacyl-tRNA biosynthesis;Valine, leucine and isoleucine biosynthesis
MAPK signaling pathway;T cell receptor signaling pathway;Toll-like receptor signaling pathway
Chemokine signaling pathway;Cytokine-cytokine receptor interaction
gnaling pathway;Hepatitis C;Jak-STAT signaling pathway;ko05152;Leishmaniasis;Measles;Osteoclast differentiation;Pancreatic cancer;Pathways in cancer;Toll-like receptor signaling pathway;T
gnaling pathway;Hepatitis C;Jak-STAT signaling pathway;ko05152;Leishmaniasis;Measles;Osteoclast differentiation;Pancreatic cancer;Pathways in cancer;Toll-like receptor signaling pathway;T

Polito et al. Universal ready-to-use immunotherapeutic approach for the treatment of cancer: expanded and activated polyclonal γδ memory T cells.
Phosphorylation sites

e myeloid leukemia;Adipocytokine signaling pathway;ErbB signaling pathway;Glioma;Insulin signaling pathway;mTOR signaling pathway;Pathways in cancer;Prostate cancer;Type II diabetes me
Endocytosis
Endocytosis
Endocytosis
Endocytosis
Endocytosis
Endocytosis
Endocytosis
Ribosome
ErbB signaling pathway;Viral myocarditis
Huntington's disease
Huntington's disease
Huntington's disease
Neuroactive ligand-receptor interaction
Chemokine signaling pathway;Endocytosis
Chemokine signaling pathway;Endocytosis
Natural killer cell mediated cytotoxicity;Primary immunodeficiency;T cell receptor signaling pathway
B cell receptor signaling pathway;Fc epsilon RI signaling pathway;Fc gamma R-mediated phagocytosis;ko05152;Natural killer cell mediated cytotoxicity;Osteoclast differentiation
Calcium signaling pathway;Huntington's disease;Parkinson's disease
n trypanosomiasis);Epithelial cell signaling in Helicobacter pylori infection;ErbB signaling pathway;Fc epsilon RI signaling pathway;GnRH signaling pathway;MAPK signaling pathway;Toll-like rec

Calcium signaling pathway;Insulin signaling pathway
RNA transport
c myeloid leukemia;ErbB signaling pathway;Fc gamma R-mediated phagocytosis;Focal adhesion;Insulin signaling pathway;MAPK signaling pathway;Neurotrophin signaling pathway;Pathways i
Cell cycle;Chronic myeloid leukemia;Measles;Pathways in cancer;Prostate cancer
Cell cycle;Chronic myeloid leukemia;Measles;Pathways in cancer;Prostate cancer

myotrophic lateral sclerosis (ALS);Fc epsilon RI signaling pathway;GnRH signaling pathway;MAPK signaling pathway;Osteoclast differentiation;Toll-like receptor signaling pathway;Toxoplasmos
Ribosome
Ribosome
Ribosome
Ribosome
Ribosome
Ribosome
Regulation of actin cytoskeleton
Ribosome
Antigen processing and presentation;ko05152;Primary immunodeficiency
Cytokine-cytokine receptor interaction;Hepatitis C;Jak-STAT signaling pathway;Measles;Natural killer cell mediated cytotoxicity;Osteoclast differentiation;Toll-like receptor signaling pathway
Glycerophospholipid metabolism
Glycerophospholipid metabolism
Fc gamma R-mediated phagocytosis;Leishmaniasis

Fc gamma R-mediated phagocytosis;Leishmaniasis
Ribosome
Fatty acid biosynthesis;Insulin signaling pathway
Fatty acid biosynthesis;Insulin signaling pathway
Fatty acid biosynthesis;Insulin signaling pathway
Chemokine signaling pathway;Endocytosis;MAPK signaling pathway;Phototransduction
Glycerophospholipid metabolism;Phosphonate and phosphinate metabolism
Glycerophospholipid metabolism;Phosphonate and phosphinate metabolism
Glycerophospholipid metabolism;Phosphonate and phosphinate metabolism
Glycerophospholipid metabolism;Phosphonate and phosphinate metabolism
Aminoacyl-tRNA biosynthesis
Aminoacyl-tRNA biosynthesis
Chemokine signaling pathway;Cytokine-cytokine receptor interaction
Cell cycle;Cell cycle - yeast;DNA replication;Meiosis - yeast
Cell cycle;Cell cycle - yeast;DNA replication;Meiosis - yeast
Cell cycle;Cell cycle - yeast;DNA replication;Meiosis - yeast
Cell cycle;Cell cycle - yeast;DNA replication;Meiosis - yeast
Cell cycle;Cell cycle - yeast;DNA replication;Meiosis - yeast
Cell cycle;Cell cycle - yeast;DNA replication;Meiosis - yeast
Cell cycle;Cell cycle - yeast;DNA replication;Meiosis - yeast
Spliceosome
Spliceosome
Notch signaling pathway
Notch signaling pathway

Polito et al. Universal ready-to-use immunotherapeutic approach for the treatment of cancer: expanded and activated polyclonal γδ memory T cells.
Phosphorylation sites

Alzheimer's disease;Neurotrophin signaling pathway;Notch signaling pathway;Wnt signaling pathway
Alzheimer's disease;Neurotrophin signaling pathway;Notch signaling pathway;Wnt signaling pathway
RNA transport
RNA transport
RNA transport
RNA transport
RNA transport
RNA transport
RNA transport
RNA transport
RNA transport
RNA transport
RNA transport
RNA transport
RNA transport
RNA transport
RNA transport
RNA transport
Axon guidance
Axon guidance
Axon guidance
Axon guidance
Chemokine signaling pathway
ay;Circadian rhythm - fly;Colorectal cancer;Endometrial cancer;ErbB signaling pathway;Focal adhesion;Hedgehog signaling pathway;Hepatitis C;Insulin signaling pathway;Measles;Melanogenes
Basal transcription factors
Drug metabolism - other enzymes;Purine metabolism
Drug metabolism - other enzymes;Purine metabolism
Base excision repair
Base excision repair
Homologous recombination;Non-homologous end-joining
Arrhythmogenic right ventricular cardiomyopathy (ARVC);Dilated cardiomyopathy;Hypertrophic cardiomyopathy (HCM)
Arrhythmogenic right ventricular cardiomyopathy (ARVC);Dilated cardiomyopathy;Hypertrophic cardiomyopathy (HCM)
Arrhythmogenic right ventricular cardiomyopathy (ARVC);Dilated cardiomyopathy;Hypertrophic cardiomyopathy (HCM)

Fc gamma R-mediated phagocytosis;Focal adhesion;Leukocyte transendothelial migration
Basal transcription factors;Cell cycle;Nucleotide excision repair
mRNA surveillance pathway
Amoebiasis;Endocytosis;ko05152;Phagosome
Peroxisome;Primary bile acid biosynthesis
Acute myeloid leukemia;Chemokine signaling pathway;Chronic myeloid leukemia;ErbB signaling pathway;Jak-STAT signaling pathway;ko05152;Measles;Pathways in cancer
Acute myeloid leukemia;Chemokine signaling pathway;Chronic myeloid leukemia;ErbB signaling pathway;Jak-STAT signaling pathway;ko05152;Measles;Pathways in cancer

Long-term potentiation;MAPK signaling pathway;mTOR signaling pathway;Neurotrophin signaling pathway;Oocyte meiosis;Progesterone-mediated oocyte maturation
Long-term potentiation;MAPK signaling pathway;mTOR signaling pathway;Neurotrophin signaling pathway;Oocyte meiosis;Progesterone-mediated oocyte maturation
Long-term potentiation;MAPK signaling pathway;mTOR signaling pathway;Neurotrophin signaling pathway;Oocyte meiosis;Progesterone-mediated oocyte maturation
Long-term potentiation;MAPK signaling pathway;mTOR signaling pathway;Neurotrophin signaling pathway;Oocyte meiosis;Progesterone-mediated oocyte maturation
Basal transcription factors;Nucleotide excision repair
Spliceosome
Spliceosome
Spliceosome
Spliceosome
Spliceosome
Spliceosome
Spliceosome
Spliceosome
mRNA surveillance pathway;RNA transport;Spliceosome
Chemokine signaling pathway;Jak-STAT signaling pathway;ko05152;Measles
Chemokine signaling pathway;Jak-STAT signaling pathway;ko05152;Measles
Neurotrophin signaling pathway;Vasopressin-regulated water reabsorption
Neurotrophin signaling pathway;Vasopressin-regulated water reabsorption
Colorectal cancer;Mismatch repair;Pathways in cancer
Colorectal cancer;Mismatch repair;Pathways in cancer
Colorectal cancer;Mismatch repair;Pathways in cancer
Colorectal cancer;Mismatch repair;Pathways in cancer
Colorectal cancer;Mismatch repair;Pathways in cancer

Polito et al. Universal ready-to-use immunotherapeutic approach for the treatment of cancer: expanded and activated polyclonal γδ memory T cells.
Phosphorylation sites

African trypanosomiasis;Renin-angiotensin system
RNA transport
RNA transport
RNA transport
RNA transport
RNA transport
RNA transport
Porphyrin and chlorophyll metabolism
Carbon fixation pathways in prokaryotes;Citrate cycle (TCA cycle)
Carbon fixation pathways in prokaryotes;Citrate cycle (TCA cycle)
Terpenoid backbone biosynthesis
Neuroactive ligand-receptor interaction
Neuroactive ligand-receptor interaction
RNA transport
Mismatch repair

Nucleotide excision repair;Protein processing in endoplasmic reticulum
Nucleotide excision repair;Protein processing in endoplasmic reticulum
RNA transport
Pancreatic secretion;Salivary secretion;Vibrio cholerae infection
Proteasome
Protein processing in endoplasmic reticulum
Regulation of actin cytoskeleton
Alzheimer's disease;Apoptosis
Alzheimer's disease;Apoptosis
Cytosolic DNA-sensing pathway;Measles
Cytosolic DNA-sensing pathway;Measles
Cytosolic DNA-sensing pathway;Measles
RNA transport
RNA transport
Alzheimer's disease;Huntington's disease;Oxidative phosphorylation;Parkinson's disease
Alzheimer's disease;Huntington's disease;Oxidative phosphorylation;Parkinson's disease
Ribosome biogenesis in eukaryotes
Ribosome biogenesis in eukaryotes
RNA transport

Polito et al. Universal ready-to-use immunotherapeutic approach for the treatment of cancer: expanded and activated polyclonal γδ memory T cells.
Phosphorylation sites

RNA transport
RNA transport
Carbon fixation in photosynthetic organisms;Fructose and mannose metabolism;Glycolysis / Gluconeogenesis;Inositol phosphate metabolism
Phagosome;Protein export;Protein processing in endoplasmic reticulum;Vibrio cholerae infection
Vascular smooth muscle contraction
al cells;Dilated cardiomyopathy;Focal adhesion;Hypertrophic cardiomyopathy (HCM);Leukocyte transendothelial migration;Pathogenic Escherichia coli infection;Phagosome;Phototransductior
on;Dilated cardiomyopathy;Focal adhesion;Hypertrophic cardiomyopathy (HCM);Leukocyte transendothelial migration;Pathogenic Escherichia coli infection;Phagosome;Phototransduction - fly
Ribosome
Axon guidance;Chemokine signaling pathway;Cytokine-cytokine receptor interaction;Endocytosis;Intestinal immune network for IgA production;Leukocyte transendothelial migration
Axon guidance;Chemokine signaling pathway;Cytokine-cytokine receptor interaction;Endocytosis;Intestinal immune network for IgA production;Leukocyte transendothelial migration
Axon guidance;Chemokine signaling pathway;Cytokine-cytokine receptor interaction;Endocytosis;Intestinal immune network for IgA production;Leukocyte transendothelial migration
MAPK signaling pathway;Pathways in cancer;Small cell lung cancer
MAPK signaling pathway;Pathways in cancer;Small cell lung cancer
Ribosome
Antigen processing and presentation;Hepatitis C;Proteasome
Ribosome
Ribosome
Ribosome
Spliceosome
Spliceosome
Spliceosome
Spliceosome
MAPK signaling pathway;Regulation of actin cytoskeleton;Tight junction
Focal adhesion;Insulin signaling pathway;Long-term potentiation;Meiosis - yeast;Oocyte meiosis;Regulation of actin cytoskeleton;Vascular smooth muscle contraction
Ribosome
Cell cycle;Neurotrophin signaling pathway;Oocyte meiosis
Ribosome

Polito et al. Universal ready-to-use immunotherapeutic approach for the treatment of cancer: expanded and activated polyclonal γδ memory T cells.
Phosphorylation sites

Spliceosome;Systemic lupus erythematosus
Spliceosome
RNA degradation
Regulation of actin cytoskeleton
Regulation of actin cytoskeleton
Proteasome
Endocrine and other factor-regulated calcium reabsorption;Endocytosis;Pancreatic secretion;Vasopressin-regulated water reabsorption
Ribosome
Ribosome
Insulin signaling pathway;mTOR signaling pathway;Ribosome
Systemic lupus erythematosus
Ribosome
Ribosome
Chemokine signaling pathway;Phototransduction;Taste transduction
Chemokine signaling pathway
Ribosome
Parkinson's disease;PPAR signaling pathway;Ribosome
Spliceosome
Spliceosome
Spliceosome
Spliceosome
Spliceosome
Spliceosome
Cell cycle;Neurotrophin signaling pathway;Oocyte meiosis
Gap junction;Pathogenic Escherichia coli infection;Phagosome
Gap junction;Pathogenic Escherichia coli infection;Phagosome
Ether lipid metabolism
African trypanosomiasis;Malaria

Polito et al. Universal ready-to-use immunotherapeutic approach for the treatment of cancer: expanded and activated polyclonal γδ memory T cells.
Phosphorylation sites

Natural killer cell mediated cytotoxicity
Natural killer cell mediated cytotoxicity
Ribosome biogenesis in eukaryotes;RNA transport
Ribosome biogenesis in eukaryotes;RNA transport
Basal transcription factors
Basal transcription factors
Basal transcription factors
Basal transcription factors
Basal transcription factors
Basal transcription factors
Cell cycle;Non-homologous end-joining
Cell cycle;Non-homologous end-joining
Cell cycle;Non-homologous end-joining
Cell cycle;Non-homologous end-joining
Cell cycle;Non-homologous end-joining
Cell cycle;Non-homologous end-joining
Alzheimer's disease;Epithelial cell signaling in Helicobacter pylori infection;Notch signaling pathway
Vibrio cholerae infection
Spliceosome
Spliceosome

Polito et al. Universal ready-to-use immunotherapeutic approach for the treatment of cancer: expanded and activated polyclonal γδ memory T cells.

Phosphorylation sites

Huntington's disease
Huntington's disease
Bacterial invasion of epithelial cells;Endocrine and other factor-regulated calcium reabsorption;Endocytosis;Huntington's disease;Lysosome
ctor-regulated calcium reabsorption;Gap junction;Gastric acid secretion;GnRH signaling pathway;Huntington's disease;Inositol phosphate metabolism;Long-term depression;Long-term potenti
Spliceosome
Spliceosome
Spliceosome
Spliceosome
Spliceosome

Polito et al. Universal ready-to-use immunotherapeutic approach for the treatment of cancer: expanded and activated polyclonal γδ memory T cells.
Phosphorylation sites

Spliceosome
Spliceosome
Spliceosome
Spliceosome
Spliceosome
Acute myeloid leukemia;Chronic myeloid leukemia;Pathways in cancer
Acute myeloid leukemia;Chronic myeloid leukemia;Pathways in cancer
Acute myeloid leukemia;Chronic myeloid leukemia;Pathways in cancer
Acute myeloid leukemia;Chronic myeloid leukemia;Pathways in cancer
Purine metabolism
Purine metabolism
Purine metabolism
Butanoate metabolism;Synthesis and degradation of ketone bodies;Terpenoid backbone biosynthesis;Valine, leucine and isoleucine degradation
Butanoate metabolism;Synthesis and degradation of ketone bodies;Terpenoid backbone biosynthesis;Valine, leucine and isoleucine degradation
Fructose and mannose metabolism;Galactose metabolism;Glycolysis / Gluconeogenesis;Methane metabolism;Pentose phosphate pathway
Fructose and mannose metabolism;Galactose metabolism;Glycolysis / Gluconeogenesis;Methane metabolism;Pentose phosphate pathway

Polito et al. Universal ready-to-use immunotherapeutic approach for the treatment of cancer: expanded and activated polyclonal γδ memory T cells.

Phosphorylation sites

Nucleotide excision repair
Ribosome
Ribosome
n;Long-term potentiation;MAPK signaling pathway;MAPK signaling pathway - fly;Melanogenesis;Melanoma;Natural killer cell mediated cytotoxicity;Neurotrophin signaling pathway;Non-small
n;Long-term potentiation;MAPK signaling pathway;MAPK signaling pathway - fly;Melanogenesis;Melanoma;Natural killer cell mediated cytotoxicity;Neurotrophin signaling pathway;Non-small

Polito et al. Universal ready-to-use immunotherapeutic approach for the treatment of cancer: expanded and activated polyclonal γδ memory T cells.

Phosphorylation sites

Dorso-ventral axis formation;Notch signaling pathway
Tight junction;Vascular smooth muscle contraction
Tight junction;Vascular smooth muscle contraction
Tight junction;Vascular smooth muscle contraction
Tight junction;Vascular smooth muscle contraction
Insulin signaling pathway
Tight junction;Vascular smooth muscle contraction

Tight junction;Vascular smooth muscle contraction
Tight junction;Vascular smooth muscle contraction
Tight junction;Vascular smooth muscle contraction
mia;Epithelial cell signaling in Helicobacter pylori infection;Jak-STAT signaling pathway;Leukocyte transendothelial migration;MAPK signaling pathway - fly;Natural killer cell mediated cytotoxic
Alanine, aspartate and glutamate metabolism;Amino sugar and nucleotide sugar metabolism
Notch signaling pathway
Notch signaling pathway
Spliceosome
Spliceosome
Spliceosome
Spliceosome
Spliceosome
Spliceosome
Spliceosome
Cytokine-cytokine receptor interaction;Jak-STAT signaling pathway;ko05152;Toxoplasmosis
Cytokine-cytokine receptor interaction;Jak-STAT signaling pathway;ko05152;Toxoplasmosis

ECM-receptor interaction
Calcium signaling pathway;Huntington's disease;Parkinson's disease
Chemokine signaling pathway;Leukocyte transendothelial migration;T cell receptor signaling pathway
Cell cycle;TGF-beta signaling pathway
Cell cycle;TGF-beta signaling pathway
Cell cycle;TGF-beta signaling pathway
Cell cycle;TGF-beta signaling pathway
Cell cycle;TGF-beta signaling pathway
Cell cycle;TGF-beta signaling pathway
Cell cycle;TGF-beta signaling pathway
Cell cycle;TGF-beta signaling pathway
Cell cycle;TGF-beta signaling pathway
Cell cycle;TGF-beta signaling pathway
mRNA surveillance pathway;RNA transport;Spliceosome
mRNA surveillance pathway;RNA transport;Spliceosome
N-Glycan biosynthesis
ngton's disease;Jak-STAT signaling pathway;ko05152;Long-term potentiation;Melanogenesis;Notch signaling pathway;Pathways in cancer;Prostate cancer;Renal cell carcinoma;TGF-beta signali

Polito et al. Universal ready-to-use immunotherapeutic approach for the treatment of cancer: expanded and activated polyclonal γδ memory T cells.

Phosphorylation sites

RNA transport

Insulin signaling pathway;Pathways in cancer;Prostate cancer

Polito et al. Universal ready-to-use immunotherapeutic approach for the treatment of cancer: expanded and activated polyclonal γδ memory T cells.
Phosphorylation sites

SNARE interactions in vesicular transport;Vasopressin-regulated water reabsorption
SNARE interactions in vesicular transport;Vasopressin-regulated water reabsorption
MAPK signaling pathway
MAPK signaling pathway
Spliceosome

Polito et al. Universal ready-to-use immunotherapeutic approach for the treatment of cancer: expanded and activated polyclonal γδ memory T cells.
Phosphorylation sites

Adherens junction
Adherens junction
Basal transcription factors
Axon guidance;B cell receptor signaling pathway;Natural killer cell mediated cytotoxicity;T cell receptor signaling pathway;VEGF signaling pathway;Wnt signaling pathway
Axon guidance;B cell receptor signaling pathway;Natural killer cell mediated cytotoxicity;T cell receptor signaling pathway;VEGF signaling pathway;Wnt signaling pathway
mRNA surveillance pathway
Chemokine signaling pathway;Regulation of actin cytoskeleton
Chemokine signaling pathway;Regulation of actin cytoskeleton
Chemokine signaling pathway;Regulation of actin cytoskeleton
Focal adhesion;Leukocyte transendothelial migration
Cell cycle;Cell cycle - yeast;Meiosis - yeast;Oocyte meiosis;Progesterone-mediated oocyte maturation;Ubiquitin mediated proteolysis

Polito et al. Universal ready-to-use immunotherapeutic approach for the treatment of cancer: expanded and activated polyclonal γδ memory T cells.

Phosphorylation sites

MAPK signaling pathway;Non-small cell lung cancer;Pathways in cancer
MAPK signaling pathway;Non-small cell lung cancer;Pathways in cancer
Pantothenate and CoA biosynthesis
Pathways in cancer;Small cell lung cancer
Fatty acid biosynthesis;Insulin signaling pathway;Propanoate metabolism;Pyruvate metabolism
Fatty acid biosynthesis;Insulin signaling pathway;Propanoate metabolism;Pyruvate metabolism
Fatty acid biosynthesis;Insulin signaling pathway;Propanoate metabolism;Pyruvate metabolism
Fc epsilon RI signaling pathway;Natural killer cell mediated cytotoxicity;Osteoclast differentiation;T cell receptor signaling pathway
Fc epsilon RI signaling pathway;Natural killer cell mediated cytotoxicity;Osteoclast differentiation;T cell receptor signaling pathway
Cell cycle
Adipocytokine signaling pathway;Hypertrophic cardiomyopathy (HCM);Insulin signaling pathway;mTOR signaling pathway;Regulation of autophagy
Adipocytokine signaling pathway;Hypertrophic cardiomyopathy (HCM);Insulin signaling pathway;mTOR signaling pathway;Regulation of autophagy
RNA transport
Alzheimer's disease;Apoptosis;Chagas disease (American trypanosomiasis);ko05152;Pathways in cancer;RIG-I-like receptor signaling pathway;Toll-like receptor signaling pathway
Axon guidance;ErbB signaling pathway;Focal adhesion;MAPK signaling pathway;Regulation of actin cytoskeleton;Renal cell carcinoma;T cell receptor signaling pathway
Axon guidance;ErbB signaling pathway;Focal adhesion;MAPK signaling pathway;Regulation of actin cytoskeleton;Renal cell carcinoma;T cell receptor signaling pathway
Axon guidance;ErbB signaling pathway;Focal adhesion;MAPK signaling pathway;Regulation of actin cytoskeleton;Renal cell carcinoma;T cell receptor signaling pathway
Axon guidance;ErbB signaling pathway;Focal adhesion;MAPK signaling pathway;Regulation of actin cytoskeleton;Renal cell carcinoma;T cell receptor signaling pathway
pithelial cells;Chronic myeloid leukemia;Endocytosis;ErbB signaling pathway;Insulin signaling pathway;Jak-STAT signaling pathway;Pathways in cancer;T cell receptor signaling pathway;Ubiquiti
pithelial cells;Chronic myeloid leukemia;Endocytosis;ErbB signaling pathway;Insulin signaling pathway;Jak-STAT signaling pathway;Pathways in cancer;T cell receptor signaling pathway;Ubiquiti
Proteasome

Polito et al. Universal ready-to-use immunotherapeutic approach for the treatment of cancer: expanded and activated polyclonal γδ memory T cells.
Phosphorylation sites

Proteasome
GnRH signaling pathway;MAPK signaling pathway;Neurotrophin signaling pathway;RIG-I-like receptor signaling pathway;Ubiquitin mediated proteolysis
GnRH signaling pathway;MAPK signaling pathway;Neurotrophin signaling pathway;RIG-I-like receptor signaling pathway;Ubiquitin mediated proteolysis
GnRH signaling pathway;MAPK signaling pathway;Neurotrophin signaling pathway;RIG-I-like receptor signaling pathway;Ubiquitin mediated proteolysis
Spliceosome
Spliceosome
Spliceosome
Spliceosome
Spliceosome
Spliceosome
Spliceosome
Spliceosome
Apoptosis;Cell cycle;p53 signaling pathway
Apoptosis;Cell cycle;p53 signaling pathway

Meiosis - yeast;mRNA surveillance pathway;Oocyte meiosis;Wnt signaling pathway
Meiosis - yeast;mRNA surveillance pathway;Oocyte meiosis;Wnt signaling pathway
Insulin signaling pathway;Progesterone-mediated oocyte maturation;Purine metabolism
Phagosome;Vasopressin-regulated water reabsorption
Cell cycle;Cell cycle - yeast;Meiosis - yeast
Cell cycle;Cell cycle - yeast;Meiosis - yeast
Non-homologous end-joining
Non-homologous end-joining
Ribosome biogenesis in eukaryotes
thway;Focal adhesion;Leukocyte transendothelial migration;Pathogenic Escherichia coli infection;Regulation of actin cytoskeleton;Shigellosis;TGF-beta signaling pathway;Vascular smooth mus
Axon guidance;B cell receptor signaling pathway;Natural killer cell mediated cytotoxicity;T cell receptor signaling pathway;VEGF signaling pathway;Wnt signaling pathway

Polito et al. Universal ready-to-use immunotherapeutic approach for the treatment of cancer: expanded and activated polyclonal γδ memory T cells.
Phosphorylation sites

Axon guidance;B cell receptor signaling pathway;Natural killer cell mediated cytotoxicity;T cell receptor signaling pathway;VEGF signaling pathway;Wnt signaling pathway
Axon guidance;B cell receptor signaling pathway;Natural killer cell mediated cytotoxicity;T cell receptor signaling pathway;VEGF signaling pathway;Wnt signaling pathway
Axon guidance;B cell receptor signaling pathway;Natural killer cell mediated cytotoxicity;T cell receptor signaling pathway;VEGF signaling pathway;Wnt signaling pathway
Axon guidance;B cell receptor signaling pathway;Natural killer cell mediated cytotoxicity;T cell receptor signaling pathway;VEGF signaling pathway;Wnt signaling pathway
Axon guidance;B cell receptor signaling pathway;Natural killer cell mediated cytotoxicity;T cell receptor signaling pathway;VEGF signaling pathway;Wnt signaling pathway
Axon guidance;B cell receptor signaling pathway;Natural killer cell mediated cytotoxicity;T cell receptor signaling pathway;VEGF signaling pathway;Wnt signaling pathway
Axon guidance;B cell receptor signaling pathway;Natural killer cell mediated cytotoxicity;T cell receptor signaling pathway;VEGF signaling pathway;Wnt signaling pathway
Axon guidance;B cell receptor signaling pathway;Natural killer cell mediated cytotoxicity;T cell receptor signaling pathway;VEGF signaling pathway;Wnt signaling pathway
Axon guidance;B cell receptor signaling pathway;Natural killer cell mediated cytotoxicity;T cell receptor signaling pathway;VEGF signaling pathway;Wnt signaling pathway
Collecting duct acid secretion;Epithelial cell signaling in Helicobacter pylori infection;ko05152;Lysosome;Oxidative phosphorylation;Phagosome;Rheumatoid arthritis;Vibrio cholerae infection
Collecting duct acid secretion;Epithelial cell signaling in Helicobacter pylori infection;ko05152;Lysosome;Oxidative phosphorylation;Phagosome;Rheumatoid arthritis;Vibrio cholerae infection
Osteoclast differentiation
Osteoclast differentiation
Gap junction;Pathogenic Escherichia coli infection;Phagosome
Cell cycle;p53 signaling pathway
Acute myeloid leukemia;ErbB signaling pathway;Insulin signaling pathway;mTOR signaling pathway;RNA transport
Apoptosis;Cytosolic DNA-sensing pathway;Hepatitis C;RIG-I-like receptor signaling pathway;Toll-like receptor signaling pathway
Apoptosis;Cytosolic DNA-sensing pathway;Hepatitis C;RIG-I-like receptor signaling pathway;Toll-like receptor signaling pathway
Cell cycle;Chronic myeloid leukemia;Huntington's disease;Notch signaling pathway;Pathways in cancer
Cell cycle;Chronic myeloid leukemia;Huntington's disease;Notch signaling pathway;Pathways in cancer
Cell cycle;Chronic myeloid leukemia;Huntington's disease;Notch signaling pathway;Pathways in cancer

Cell cycle;Chronic myeloid leukemia;Huntington's disease;Notch signaling pathway;Pathways in cancer
Notch signaling pathway;Spliceosome
Notch signaling pathway;Spliceosome
Regulation of actin cytoskeleton
Regulation of actin cytoskeleton
Regulation of actin cytoskeleton
Regulation of actin cytoskeleton
Spliceosome
Spliceosome
Spliceosome
Nucleotide excision repair;Ubiquitin mediated proteolysis
Nucleotide excision repair;Ubiquitin mediated proteolysis
Nucleotide excision repair;Ubiquitin mediated proteolysis
RNA transport
RNA degradation

Focal adhesion;Insulin signaling pathway;Neurotrophin signaling pathway;Renal cell carcinoma
Focal adhesion;Insulin signaling pathway;Neurotrophin signaling pathway;Renal cell carcinoma
Focal adhesion;Insulin signaling pathway;Neurotrophin signaling pathway;Renal cell carcinoma
Focal adhesion;Insulin signaling pathway;Neurotrophin signaling pathway;Renal cell carcinoma
Focal adhesion;Insulin signaling pathway;Neurotrophin signaling pathway;Renal cell carcinoma
Focal adhesion;Insulin signaling pathway;Neurotrophin signaling pathway;Renal cell carcinoma
Purine metabolism
Purine metabolism

Polito et al. Universal ready-to-use immunotherapeutic approach for the treatment of cancer: expanded and activated polyclonal γδ memory T cells.
Phosphorylation sites

RNA transport
RNA transport
RNA transport
RNA transport
RNA transport
Endocytosis
Endocytosis
Endocytosis
Cytosolic DNA-sensing pathway;Hepatitis C;Measles;RIG-I-like receptor signaling pathway;Toll-like receptor signaling pathway
DNA replication;Purine metabolism;Pyrimidine metabolism
Cell cycle;TGF-beta signaling pathway
Phagosome;Vasopressin-regulated water reabsorption
Phagosome;Vasopressin-regulated water reabsorption
Bacterial invasion of epithelial cells;Pathogenic Escherichia coli infection;Shigellosis;Tight junction
RIG-I-like receptor signaling pathway
Calcium signaling pathway;Chemokine signaling pathway;GnRH signaling pathway;Leukocyte transendothelial migration;Natural killer cell mediated cytotoxicity
Focal adhesion;MAPK signaling pathway
Glycerolipid metabolism;Plant-pathogen interaction;PPAR signaling pathway

Cell cycle;Cell cycle - yeast;DNA replication;Meiosis - yeast
Cell cycle;Cell cycle - yeast;DNA replication;Meiosis - yeast
Cell cycle;Cell cycle - yeast;DNA replication;Meiosis - yeast
;Gap junction;Gastric acid secretion;GnRH signaling pathway;Long-term depression;Long-term potentiation;Oocyte meiosis;Pancreatic secretion;Phosphatidylinositol signaling system;Salivary
tion;Gastric acid secretion;GnRH signaling pathway;Long-term depression;Long-term potentiation;Oocyte meiosis;Pancreatic secretion;Phosphatidylinositol signaling system;Salivary secretion
tion;Gastric acid secretion;GnRH signaling pathway;Long-term depression;Long-term potentiation;Oocyte meiosis;Pancreatic secretion;Phosphatidylinositol signaling system;Salivary secretion
tion;Gastric acid secretion;GnRH signaling pathway;Long-term depression;Long-term potentiation;Oocyte meiosis;Pancreatic secretion;Phosphatidylinositol signaling system;Salivary secretion
tion;Gastric acid secretion;GnRH signaling pathway;Long-term depression;Long-term potentiation;Oocyte meiosis;Pancreatic secretion;Phosphatidylinositol signaling system;Salivary secretion
ion;Gastric acid secretion;GnRH signaling pathway;Huntington's disease;Long-term depression;Long-term potentiation;Oocyte meiosis;Pancreatic secretion;Phosphatidylinositol signaling syste
MAPK signaling pathway - fly
MAPK signaling pathway - fly
MAPK signaling pathway - fly
Cytosolic DNA-sensing pathway;Hepatitis C;Measles;RIG-I-like receptor signaling pathway;Toll-like receptor signaling pathway
Ubiquitin mediated proteolysis
Ubiquitin mediated proteolysis
Ubiquitin mediated proteolysis
Ubiquitin mediated proteolysis
Ubiquitin mediated proteolysis
Ubiquitin mediated proteolysis

Polito et al. Universal ready-to-use immunotherapeutic approach for the treatment of cancer: expanded and activated polyclonal γδ memory T cells.

Phosphorylation sites

Cell cycle;Cell cycle - yeast;Meiosis - yeast;Oocyte meiosis
Ribosome biogenesis in eukaryotes
N-Glycan biosynthesis;Protein processing in endoplasmic reticulum
Meiosis - yeast;mRNA surveillance pathway;Oocyte meiosis;Wnt signaling pathway

Polito et al. Universal ready-to-use immunotherapeutic approach for the treatment of cancer: expanded and activated polyclonal γδ memory T cells.
Phosphorylation sites

Jak-STAT signaling pathway
Jak-STAT signaling pathway
Jak-STAT signaling pathway

Polito et al. Universal ready-to-use immunotherapeutic approach for the treatment of cancer: expanded and activated polyclonal γδ memory T cells.
Phosphorylation sites

Proteasome
Cell cycle - yeast
RNA degradation
Aminoacyl-tRNA biosynthesis
Pancreatic cancer;Regulation of actin cytoskeleton
Pancreatic cancer;Regulation of actin cytoskeleton
Pancreatic cancer;Regulation of actin cytoskeleton
Pancreatic cancer;Regulation of actin cytoskeleton
Pancreatic cancer;Regulation of actin cytoskeleton
Base excision repair;DNA replication;Homologous recombination;Mismatch repair;Nucleotide excision repair;Purine metabolism;Pyrimidine metabolism
Base excision repair;DNA replication;Homologous recombination;Mismatch repair;Nucleotide excision repair;Purine metabolism;Pyrimidine metabolism
Base excision repair;DNA replication;Homologous recombination;Mismatch repair;Nucleotide excision repair;Purine metabolism;Pyrimidine metabolism
Endocytosis

Polito et al. Universal ready-to-use immunotherapeutic approach for the treatment of cancer: expanded and activated polyclonal γδ memory T cells.
Phosphorylation sites

Endocytosis
Endocytosis;ko05152;Phagosome
Protein processing in endoplasmic reticulum
Endocytosis
Endocytosis
Pancreatic cancer;Pathways in cancer
Pancreatic cancer;Pathways in cancer
Pancreatic cancer;Pathways in cancer
Spliceosome
Spliceosome

Long-term potentiation;MAPK signaling pathway;mTOR signaling pathway;Neurotrophin signaling pathway;Oocyte meiosis;Progesterone-mediated oocyte maturation
Long-term potentiation;MAPK signaling pathway;mTOR signaling pathway;Neurotrophin signaling pathway;Oocyte meiosis;Progesterone-mediated oocyte maturation
Long-term potentiation;MAPK signaling pathway;mTOR signaling pathway;Neurotrophin signaling pathway;Oocyte meiosis;Progesterone-mediated oocyte maturation
Long-term potentiation;MAPK signaling pathway;mTOR signaling pathway;Neurotrophin signaling pathway;Oocyte meiosis;Progesterone-mediated oocyte maturation
Spliceosome
Spliceosome
RNA degradation
Basal transcription factors
Insulin signaling pathway
Neuroactive ligand-receptor interaction
Neuroactive ligand-receptor interaction

Polito et al. Universal ready-to-use immunotherapeutic approach for the treatment of cancer: expanded and activated polyclonal γδ memory T cells.
Phosphorylation sites

Calcium signaling pathway;Focal adhesion;Gastric acid secretion;Regulation of actin cytoskeleton;Vascular smooth muscle contraction
Protein digestion and absorption
Cell adhesion molecules (CAMs)
Focal adhesion
Focal adhesion
Focal adhesion
Focal adhesion
Focal adhesion
Focal adhesion
Pathways in cancer;Thyroid cancer
Pathways in cancer;Thyroid cancer
Pathways in cancer;Thyroid cancer
Pathways in cancer;Thyroid cancer
Basal transcription factors
ishmaniasis;Leukocyte transendothelial migration;MAPK signaling pathway;MAPK signaling pathway - yeast;Neurotrophin signaling pathway;NOD-like receptor signaling pathway;Osteoclast di
Calcium signaling pathway;Long-term potentiation;Neurotrophin signaling pathway;Osteoclast differentiation
MAPK signaling pathway
MAPK signaling pathway
Spliceosome
Spliceosome

Polito et al. Universal ready-to-use immunotherapeutic approach for the treatment of cancer: expanded and activated polyclonal γδ memory T cells.
Phosphorylation sites

Spliceosome
Spliceosome
Spliceosome
Spliceosome
mRNA surveillance pathway
Ubiquitin mediated proteolysis
Drug metabolism - other enzymes;Pyrimidine metabolism
Amino sugar and nucleotide sugar metabolism;Galactose metabolism;Pentose and glucuronate interconversions;Starch and sucrose metabolism
Fructose and mannose metabolism

Phosphorylation sites

Glycerolipid metabolism;Glycerophospholipid metabolism

Polito et al. Universal ready-to-use immunotherapeutic approach for the treatment of cancer: expanded and activated polyclonal γδ memory T cells.
Phosphorylation sites

Spliceosome
Spliceosome
Lysine degradation
Oocyte meiosis

Polito et al. Universal ready-to-use immunotherapeutic approach for the treatment of cancer: expanded and activated polyclonal γδ memory T cells.
Phosphorylation sites

Regulation of actin cytoskeleton
Regulation of actin cytoskeleton
Regulation of actin cytoskeleton
Regulation of actin cytoskeleton
Tight junction
Tight junction
Tight junction
Proteasome
RNA transport
RNA transport

Spliceosome
Spliceosome
Spliceosome
Spliceosome

Polito et al. Universal ready-to-use immunotherapeutic approach for the treatment of cancer: expanded and activated polyclonal γδ memory T cells.
Phosphorylation sites

Basal transcription factors
Basal transcription factors
Basal transcription factors

RNA transport

Polito et al. Universal ready-to-use immunotherapeutic approach for the treatment of cancer: expanded and activated polyclonal γδ memory T cells.
Phosphorylation sites

Cell cycle - yeast
Cell cycle - yeast
Cell cycle - yeast
Cell cycle - yeast
Cell cycle - yeast
Cell cycle - yeast
Cell cycle - yeast
Cell cycle - yeast

Polito et al. Universal ready-to-use immunotherapeutic approach for the treatment of cancer: expanded and activated polyclonal γδ memory T cells.
Phosphorylation sites

RNA degradation
RNA degradation
RNA degradation
RNA degradation
RNA degradation
RNA degradation
RNA degradation
RNA degradation
Starch and sucrose metabolism

Polito et al. Universal ready-to-use immunotherapeutic approach for the treatment of cancer: expanded and activated polyclonal γδ memory T cells.
Phosphorylation sites

mTOR signaling pathway
mTOR signaling pathway
mTOR signaling pathway

Polito et al. Universal ready-to-use immunotherapeutic approach for the treatment of cancer: expanded and activated polyclonal γδ memory T cells.
Phosphorylation sites

mRNA surveillance pathway
mRNA surveillance pathway
mRNA surveillance pathway
mRNA surveillance pathway
Endocytosis
Endocytosis
Endocytosis
Endocytosis
Endocytosis
Endocytosis
Endocytosis
Endocytosis

Nicotinate and nicotinamide metabolism

B cell receptor signaling pathway

MAPK signaling pathway
Chemokine signaling pathway;MAPK signaling pathway
Chemokine signaling pathway;MAPK signaling pathway
Chemokine signaling pathway;MAPK signaling pathway
Chemokine signaling pathway;MAPK signaling pathway

Endocytosis;TGF-beta signaling pathway

Cytosolic DNA-sensing pathway;Hepatitis C;Measles;RIG-I-like receptor signaling pathway
Cytosolic DNA-sensing pathway;Hepatitis C;Measles;RIG-I-like receptor signaling pathway
Cytosolic DNA-sensing pathway;Hepatitis C;Measles;RIG-I-like receptor signaling pathway

Polito et al. Universal ready-to-use immunotherapeutic approach for the treatment of cancer: expanded and activated polyclonal γδ memory T cells.
Phosphorylation sites

Ubiquitin mediated proteolysis
Ubiquitin mediated proteolysis
Ubiquitin mediated proteolysis
Ubiquitin mediated proteolysis
Ubiquitin mediated proteolysis
Ubiquitin mediated proteolysis
Ubiquitin mediated proteolysis
Ubiquitin mediated proteolysis
Ubiquitin mediated proteolysis
Ubiquitin mediated proteolysis
Ubiquitin mediated proteolysis
RNA degradation
RNA degradation
RNA degradation
mRNA surveillance pathway
mRNA surveillance pathway

Polito et al. Universal ready-to-use immunotherapeutic approach for the treatment of cancer: expanded and activated polyclonal γδ memory T cells.

Phosphorylation sites

mRNA surveillance pathway
mRNA surveillance pathway
mRNA surveillance pathway;RNA transport;Spliceosome
mRNA surveillance pathway;RNA transport;Spliceosome

Phosphorylation sites

Neuroactive ligand-receptor interaction
Notch signaling pathway
Spliceosome
Spliceosome

Spliceosome

Glycerophospholipid metabolism
Glycerophospholipid metabolism
Glycerophospholipid metabolism

RNA degradation
RNA degradation
RNA degradation
RNA degradation
Ribosome biogenesis in eukaryotes;RNA degradation
Insulin signaling pathway;mTOR signaling pathway
Insulin signaling pathway;mTOR signaling pathway
Insulin signaling pathway;mTOR signaling pathway
RNA transport

Polito et al. Universal ready-to-use immunotherapeutic approach for the treatment of cancer: expanded and activated polyclonal γδ memory T cells.
Phosphorylation sites

RNA transport
RNA transport
RNA transport
Ribosome biogenesis in eukaryotes
Cell cycle;Cell cycle - yeast;Meiosis - yeast
Cell cycle;Cell cycle - yeast;Meiosis - yeast

mRNA surveillance pathway

Polito et al. Universal ready-to-use immunotherapeutic approach for the treatment of cancer: expanded and activated polyclonal γδ memory T cells.

Phosphorylation sites

Endocytosis
Endocytosis
Endocytosis
Spliceosome

Polito et al. Universal ready-to-use immunotherapeutic approach for the treatment of cancer: expanded and activated polyclonal γδ memory T cells.
Phosphorylation sites

Endocytosis
Endocytosis
Endocytosis
Endocytosis
Endocytosis
Renal cell carcinoma
Renal cell carcinoma

Polito et al. Universal ready-to-use immunotherapeutic approach for the treatment of cancer: expanded and activated polyclonal γδ memory T cells.
Phosphorylation sites

RNA transport
RNA transport
Cell cycle;Cell cycle - yeast;Meiosis - yeast;Oocyte meiosis;Progesterone-mediated oocyte maturation;Ubiquitin mediated proteolysis
Cell cycle;Cell cycle - yeast;Meiosis - yeast;Oocyte meiosis;Progesterone-mediated oocyte maturation;Ubiquitin mediated proteolysis
RNA transport;Spliceosome
RNA transport;Spliceosome
RNA transport;Spliceosome
Tight junction

N-Glycan biosynthesis;Protein processing in endoplasmic reticulum;Various types of N-glycan biosynthesis
N-Glycan biosynthesis;Protein processing in endoplasmic reticulum;Various types of N-glycan biosynthesis
N-Glycan biosynthesis;Protein processing in endoplasmic reticulum;Various types of N-glycan biosynthesis
Chemokine signaling pathway
Notch signaling pathway
Notch signaling pathway
Notch signaling pathway
Notch signaling pathway
Regulation of actin cytoskeleton
Regulation of actin cytoskeleton

Polito et al. Universal ready-to-use immunotherapeutic approach for the treatment of cancer: expanded and activated polyclonal γδ memory T cells.
Phosphorylation sites

Lysine degradation
Lysine degradation
Lysine degradation
Lysine degradation
Lysine degradation
Lysine degradation
Lysine degradation
RNA transport
RNA transport
Galactose metabolism;Starch and sucrose metabolism

Polito et al. Universal ready-to-use immunotherapeutic approach for the treatment of cancer: expanded and activated polyclonal γδ memory T cells.

Phosphorylation sites

Endocytosis
Endocytosis
Spliceosome
Spliceosome

Bacterial invasion of epithelial cells
Bacterial invasion of epithelial cells
Bacterial invasion of epithelial cells
Regulation of actin cytoskeleton
Regulation of actin cytoskeleton
Regulation of actin cytoskeleton

Endocytosis
Nucleotide excision repair;p53 signaling pathway;Ubiquitin mediated proteolysis
Nucleotide excision repair;p53 signaling pathway;Ubiquitin mediated proteolysis
Bacterial invasion of epithelial cells;Chemokine signaling pathway;Shigellosis
Insulin signaling pathway;mTOR signaling pathway
Insulin signaling pathway;mTOR signaling pathway
Protein processing in endoplasmic reticulum
Chemokine signaling pathway;Fc gamma R-mediated phagocytosis
Chemokine signaling pathway;Fc gamma R-mediated phagocytosis
Chemokine signaling pathway;Fc gamma R-mediated phagocytosis
Chemokine signaling pathway;Fc gamma R-mediated phagocytosis
Chemokine signaling pathway;Fc gamma R-mediated phagocytosis
Chemokine signaling pathway;Fc gamma R-mediated phagocytosis

RNA transport
Glycosylphosphatidylinositol(GPI)-anchor biosynthesis
Pathways in cancer;Thyroid cancer

Polito et al. Universal ready-to-use immunotherapeutic approach for the treatment of cancer: expanded and activated polyclonal γδ memory T cells.
Phosphorylation sites

Cell cycle;Chronic myeloid leukemia;Huntington's disease;Notch signaling pathway;Pathways in cancer
Cell cycle;Chronic myeloid leukemia;Huntington's disease;Notch signaling pathway;Pathways in cancer
Cell cycle;Chronic myeloid leukemia;Huntington's disease;Notch signaling pathway;Pathways in cancer
ngton's disease;Jak-STAT signaling pathway;ko05152;Long-term potentiation;Melanogenesis;Notch signaling pathway;Pathways in cancer;Prostate cancer;Renal cell carcinoma;TGF-beta signali
ngton's disease;Jak-STAT signaling pathway;ko05152;Long-term potentiation;Melanogenesis;Notch signaling pathway;Pathways in cancer;Prostate cancer;Renal cell carcinoma;TGF-beta signali
mRNA surveillance pathway;Tight junction
mRNA surveillance pathway;Tight junction
mRNA surveillance pathway;Tight junction
B cell receptor signaling pathway;Fc epsilon RI signaling pathway;Fc gamma R-mediated phagocytosis;Insulin signaling pathway;Phosphatidylinositol signaling system
B cell receptor signaling pathway;Fc epsilon RI signaling pathway;Fc gamma R-mediated phagocytosis;Insulin signaling pathway;Phosphatidylinositol signaling system
B cell receptor signaling pathway;Fc epsilon RI signaling pathway;Fc gamma R-mediated phagocytosis;Insulin signaling pathway;Phosphatidylinositol signaling system
B cell receptor signaling pathway;Fc epsilon RI signaling pathway;Fc gamma R-mediated phagocytosis;Insulin signaling pathway;Phosphatidylinositol signaling system
B cell receptor signaling pathway;Fc epsilon RI signaling pathway;Fc gamma R-mediated phagocytosis;Insulin signaling pathway;Phosphatidylinositol signaling system
B cell receptor signaling pathway;Fc epsilon RI signaling pathway;Fc gamma R-mediated phagocytosis;Insulin signaling pathway;Phosphatidylinositol signaling system
B cell receptor signaling pathway;Fc epsilon RI signaling pathway;Fc gamma R-mediated phagocytosis;Insulin signaling pathway;Phosphatidylinositol signaling system
B cell receptor signaling pathway;Fc epsilon RI signaling pathway;Fc gamma R-mediated phagocytosis;Insulin signaling pathway;Phosphatidylinositol signaling system
B cell receptor signaling pathway;Fc epsilon RI signaling pathway;Fc gamma R-mediated phagocytosis;Insulin signaling pathway;Phosphatidylinositol signaling system
B cell receptor signaling pathway;Fc epsilon RI signaling pathway;Fc gamma R-mediated phagocytosis;Insulin signaling pathway;Phosphatidylinositol signaling system
B cell receptor signaling pathway;Fc epsilon RI signaling pathway;Fc gamma R-mediated phagocytosis;Insulin signaling pathway;Phosphatidylinositol signaling system
RIG-I-like receptor signaling pathway
RIG-I-like receptor signaling pathway
Axon guidance
Axon guidance
Homologous recombination;Non-homologous end-joining
Protein processing in endoplasmic reticulum
mRNA surveillance pathway;RNA transport

mRNA surveillance pathway;RNA transport
leukemia;Colorectal cancer;Endometrial cancer;ErbB signaling pathway;Focal adhesion;Hepatitis C;Insulin signaling pathway;ko05152;Melanoma;Neurotrophin signaling pathway;Non-small ce
leukemia;Colorectal cancer;Endometrial cancer;ErbB signaling pathway;Focal adhesion;Hepatitis C;Insulin signaling pathway;ko05152;Melanoma;Neurotrophin signaling pathway;Non-small ce
leukemia;Colorectal cancer;Endometrial cancer;ErbB signaling pathway;Focal adhesion;Hepatitis C;Insulin signaling pathway;ko05152;Melanoma;Neurotrophin signaling pathway;Non-small ce
Cytokine-cytokine receptor interaction
Pathogenic Escherichia coli infection
Pathogenic Escherichia coli infection
Pathogenic Escherichia coli infection
Pathogenic Escherichia coli infection
Pathogenic Escherichia coli infection
Pathogenic Escherichia coli infection
Pathogenic Escherichia coli infection
Pathogenic Escherichia coli infection
Pathogenic Escherichia coli infection
Alzheimer's disease;Calcium signaling pathway;Pancreatic secretion
Calcium signaling pathway;Insulin signaling pathway
Calcium signaling pathway;Insulin signaling pathway
Tight junction

Polito et al. Universal ready-to-use immunotherapeutic approach for the treatment of cancer: expanded and activated polyclonal γδ memory T cells.
Phosphorylation sites

Endocytosis
Endocytosis
Endocytosis
Endocytosis
Endocytosis
Endocytosis
Endocytosis
Endocytosis
Endocytosis
Endocytosis
Endocytosis

Polito et al. Universal ready-to-use immunotherapeutic approach for the treatment of cancer: expanded and activated polyclonal γδ memory T cells.

Phosphorylation sites

Regulation of autophagy
Spliceosome
Spliceosome
Spliceosome

Polito et al. Universal ready-to-use immunotherapeutic approach for the treatment of cancer: expanded and activated polyclonal γδ memory T cells.
Phosphorylation sites

Cytokine-cytokine receptor interaction
Cytokine-cytokine receptor interaction
RNA degradation
RNA degradation
RNA degradation
Leukocyte transendothelial migration
Leukocyte transendothelial migration
Leukocyte transendothelial migration
Leukocyte transendothelial migration
Leukocyte transendothelial migration
Leukocyte transendothelial migration
Leukocyte transendothelial migration
sugar and nucleotide sugar metabolism;Galactose metabolism;Glycolysis / Gluconeogenesis;Pentose phosphate pathway;Purine metabolism;Starch and sucrose metabolism;Streptomycin biosy
Cysteine and methionine metabolism

Spliceosome
Spliceosome
Protein processing in endoplasmic reticulum
RNA transport;Spliceosome

Lysine degradation
Glycerophospholipid metabolism
Endocytosis

Endocytosis
Inositol phosphate metabolism;Phosphatidylinositol signaling system
Measles;p53 signaling pathway;Ubiquitin mediated proteolysis
Aldosterone-regulated sodium reabsorption;Endocytosis;Ubiquitin mediated proteolysis

Polito et al. Universal ready-to-use immunotherapeutic approach for the treatment of cancer: expanded and activated polyclonal γδ memory T cells.
Phosphorylation sites

Adipocytokine signaling pathway
Adipocytokine signaling pathway
RNA transport
RNA transport
NOD-like receptor signaling pathway
Huntington's disease
Huntington's disease

Huntington's disease
Huntington's disease
Huntington's disease
Protein export;Protein processing in endoplasmic reticulum

Polito et al. Universal ready-to-use immunotherapeutic approach for the treatment of cancer: expanded and activated polyclonal γδ memory T cells.
Phosphorylation sites

Protein export;Protein processing in endoplasmic reticulum
Endocytosis
Endocytosis
Endocytosis
Endocytosis
Lysosome;Neurotrophin signaling pathway
RNA transport
RNA transport
RNA transport
Regulation of autophagy
Ribosome biogenesis in eukaryotes;RNA transport
Ribosome biogenesis in eukaryotes;RNA transport
RNA transport
Cell cycle;Oocyte meiosis;Progesterone-mediated oocyte maturation
Cell cycle;Oocyte meiosis;Progesterone-mediated oocyte maturation
Amyotrophic lateral sclerosis (ALS);MAPK signaling pathway;Neurotrophin signaling pathway;Protein processing in endoplasmic reticulum

Polito et al. Universal ready-to-use immunotherapeutic approach for the treatment of cancer: expanded and activated polyclonal γδ memory T cells.
Phosphorylation sites

Measles
Measles
GnRH signaling pathway;MAPK signaling pathway;Neurotrophin signaling pathway
GnRH signaling pathway;MAPK signaling pathway;Neurotrophin signaling pathway
GnRH signaling pathway;MAPK signaling pathway;Neurotrophin signaling pathway
GnRH signaling pathway;MAPK signaling pathway;Neurotrophin signaling pathway
GnRH signaling pathway;MAPK signaling pathway;Neurotrophin signaling pathway
GnRH signaling pathway;MAPK signaling pathway;Neurotrophin signaling pathway
GnRH signaling pathway;MAPK signaling pathway;Neurotrophin signaling pathway
Systemic lupus erythematosus
Systemic lupus erythematosus
MAPK signaling pathway
Endocytosis
Cell cycle - yeast
Cell cycle - yeast

mRNA surveillance pathway;RNA transport
mRNA surveillance pathway

Polito et al. Universal ready-to-use immunotherapeutic approach for the treatment of cancer: expanded and activated polyclonal γδ memory T cells.
Phosphorylation sites

Inositol phosphate metabolism;Phosphatidylinositol signaling system
Inositol phosphate metabolism;Phosphatidylinositol signaling system
RNA transport
Insulin signaling pathway;MAPK signaling pathway
Cytosolic DNA-sensing pathway;Purine metabolism;Pyrimidine metabolism;RNA polymerase
Spliceosome
Pathways in cancer;Small cell lung cancer
Cysteine and methionine metabolism
SNARE interactions in vesicular transport
Glycerophospholipid metabolism
Glycerophospholipid metabolism

Ribosome biogenesis in eukaryotes
Ribosome biogenesis in eukaryotes
Ribosome biogenesis in eukaryotes
Ribosome biogenesis in eukaryotes
mRNA surveillance pathway
mRNA surveillance pathway
mRNA surveillance pathway
Sphingolipid metabolism
B cell receptor signaling pathway;T cell receptor signaling pathway
B cell receptor signaling pathway;T cell receptor signaling pathway
B cell receptor signaling pathway;T cell receptor signaling pathway
B cell receptor signaling pathway;T cell receptor signaling pathway
B cell receptor signaling pathway;T cell receptor signaling pathway

Polito et al. Universal ready-to-use immunotherapeutic approach for the treatment of cancer: expanded and activated polyclonal γδ memory T cells.
Phosphorylation sites

Lysine degradation
Lysine degradation
Lysine degradation
Measles;RIG-I-like receptor signaling pathway
Pantothenate and CoA biosynthesis
Pantothenate and CoA biosynthesis
Lysine degradation
Lysine degradation
Ribosome biogenesis in eukaryotes
Ribosome biogenesis in eukaryotes

Polito et al. Universal ready-to-use immunotherapeutic approach for the treatment of cancer: expanded and activated polyclonal γδ memory T cells.

Phosphorylation sites

mRNA surveillance pathway;RNA transport
Ubiquitin mediated proteolysis

Ubiquitin mediated proteolysis
Ubiquitin mediated proteolysis
Purine metabolism;Pyrimidine metabolism;RNA polymerase
Pathways in cancer;Renal cell carcinoma
Ribosome biogenesis in eukaryotes

Polito et al. Universal ready-to-use immunotherapeutic approach for the treatment of cancer: expanded and activated polyclonal γδ memory T cells.
Phosphorylation sites

Ribosome biogenesis in eukaryotes;RNA degradation
Ribosome biogenesis in eukaryotes;RNA degradation
Ribosome biogenesis in eukaryotes;RNA degradation
Cytosolic DNA-sensing pathway
Cell cycle;Cell cycle - yeast;Meiosis - yeast;Oocyte meiosis;Progesterone-mediated oocyte maturation;Ubiquitin mediated proteolysis
Cell cycle;Cell cycle - yeast;Meiosis - yeast;Oocyte meiosis;Progesterone-mediated oocyte maturation;Ubiquitin mediated proteolysis
Cell cycle;Cell cycle - yeast;Meiosis - yeast;Oocyte meiosis;Progesterone-mediated oocyte maturation;Ubiquitin mediated proteolysis
Endocytosis
Endocytosis

Polito et al. Universal ready-to-use immunotherapeutic approach for the treatment of cancer: expanded and activated polyclonal γδ memory T cells.
Phosphorylation sites

Oocyte meiosis
Oocyte meiosis
Oocyte meiosis
Oocyte meiosis
Oocyte meiosis
Oocyte meiosis
MAPK signaling pathway
MAPK signaling pathway
MAPK signaling pathway
Oxidative phosphorylation
mRNA surveillance pathway;RNA transport
mRNA surveillance pathway;RNA transport
mRNA surveillance pathway;RNA transport
mRNA surveillance pathway;RNA transport
mRNA surveillance pathway;RNA transport
mRNA surveillance pathway;RNA transport
Protein processing in endoplasmic reticulum
Protein processing in endoplasmic reticulum

Gap junction;Pathogenic Escherichia coli infection;Phagosome
Endocytosis

Ribosome biogenesis in eukaryotes
Ribosome biogenesis in eukaryotes

Polito et al. Universal ready-to-use immunotherapeutic approach for the treatment of cancer: expanded and activated polyclonal γδ memory T cells.

Phosphorylation sites

Cell cycle - yeast
Lysine degradation
Non-homologous end-joining
Drug metabolism - other enzymes;Pyrimidine metabolism
Nicotinate and nicotinamide metabolism
RNA transport

Polito et al. Universal ready-to-use immunotherapeutic approach for the treatment of cancer: expanded and activated polyclonal γδ memory T cells.
Phosphorylation sites

Wnt signaling pathway
Wnt signaling pathway
Wnt signaling pathway
Wnt signaling pathway
Spliceosome
VEGF signaling pathway
VEGF signaling pathway
VEGF signaling pathway
Endocytosis
Endocytosis;Tight junction

Polito et al. Universal ready-to-use immunotherapeutic approach for the treatment of cancer: expanded and activated polyclonal γδ memory T cells.
Phosphorylation sites

RNA degradation
RNA degradation
RNA degradation
RNA degradation
Osteoclast differentiation;RIG-I-like receptor signaling pathway
Osteoclast differentiation;RIG-I-like receptor signaling pathway
Ubiquitin mediated proteolysis
Carbon fixation pathways in prokaryotes;Glycolysis / Gluconeogenesis;Methane metabolism;Propanoate metabolism;Pyruvate metabolism
Carbon fixation pathways in prokaryotes;Glycolysis / Gluconeogenesis;Methane metabolism;Propanoate metabolism;Pyruvate metabolism

Polito et al. Universal ready-to-use immunotherapeutic approach for the treatment of cancer: expanded and activated polyclonal γδ memory T cells.
Phosphorylation sites

Protein processing in endoplasmic reticulum
Ribosome biogenesis in eukaryotes
MAPK signaling pathway
Focal adhesion;Leukocyte transendothelial migration;Regulation of actin cytoskeleton
Focal adhesion;Leukocyte transendothelial migration;Regulation of actin cytoskeleton
Focal adhesion;Leukocyte transendothelial migration;Regulation of actin cytoskeleton
Focal adhesion;Leukocyte transendothelial migration;Regulation of actin cytoskeleton
Bladder cancer;Non-small cell lung cancer;Pathways in cancer
Jak-STAT signaling pathway

Polito et al. Universal ready-to-use immunotherapeutic approach for the treatment of cancer: expanded and activated polyclonal γδ memory T cells.
Phosphorylation sites

Cell cycle - yeast
Cell cycle - yeast
Ribosome biogenesis in eukaryotes
Pantothenate and CoA biosynthesis
Ribosome biogenesis in eukaryotes
Cytosolic DNA-sensing pathway;Purine metabolism;Pyrimidine metabolism;RNA polymerase

Spliceosome
Drug metabolism - other enzymes;Pyrimidine metabolism
Drug metabolism - other enzymes;Pyrimidine metabolism
Sphingolipid metabolism

Polito et al. Universal ready-to-use immunotherapeutic approach for the treatment of cancer: expanded and activated polyclonal γδ memory T cells.
Phosphorylation sites

Ribosome biogenesis in eukaryotes
Ribosome biogenesis in eukaryotes
MAPK signaling pathway;Tight junction
MAPK signaling pathway;Tight junction
MAPK signaling pathway;Tight junction
MAPK signaling pathway;Tight junction
SNARE interactions in vesicular transport
p53 signaling pathway
Lysosome
Lysosome
Cell cycle;Oocyte meiosis

Axon guidance;ko05152;Regulation of actin cytoskeleton;Vascular smooth muscle contraction

Polito et al. Universal ready-to-use immunotherapeutic approach for the treatment of cancer: expanded and activated polyclonal γδ memory T cells.
Phosphorylation sites

Protein processing in endoplasmic reticulum
mRNA surveillance pathway
Cell cycle;Cell cycle - yeast;Meiosis - yeast
Inositol phosphate metabolism;Phosphatidylinositol signaling system
Inositol phosphate metabolism;Phosphatidylinositol signaling system
Inositol phosphate metabolism;Phosphatidylinositol signaling system
Steroid biosynthesis
Acute myeloid leukemia;ErbB signaling pathway;Fc gamma R-mediated phagocytosis;Insulin signaling pathway;mTOR signaling pathway;TGF-beta signaling pathway
Ubiquitin mediated proteolysis
Cell cycle
Cell cycle

Tight junction;Vibrio cholerae infection
Tight junction;Vibrio cholerae infection
Tight junction;Vibrio cholerae infection
Tight junction;Vibrio cholerae infection
Tight junction;Vibrio cholerae infection
Tight junction;Vibrio cholerae infection
Tight junction;Vibrio cholerae infection
Tight junction;Vibrio cholerae infection
Amyotrophic lateral sclerosis (ALS);MAPK signaling pathway
Amyotrophic lateral sclerosis (ALS);MAPK signaling pathway
Amyotrophic lateral sclerosis (ALS);MAPK signaling pathway
Amyotrophic lateral sclerosis (ALS);MAPK signaling pathway
Amyotrophic lateral sclerosis (ALS);MAPK signaling pathway
Amyotrophic lateral sclerosis (ALS);MAPK signaling pathway

Phosphorylation sites

RNA transport
RNA transport

Polito et al. Universal ready-to-use immunotherapeutic approach for the treatment of cancer: expanded and activated polyclonal γδ memory T cells.
Phosphorylation sites

Bladder cancer;Pathways in cancer
Bladder cancer;Pathways in cancer
Renin-angiotensin system
Amino sugar and nucleotide sugar metabolism
Cell cycle;Cell cycle - yeast;Meiosis - yeast;Oocyte meiosis;Progesterone-mediated oocyte maturation;Ubiquitin mediated proteolysis
Cell cycle;Cell cycle - yeast;Meiosis - yeast;Oocyte meiosis;Progesterone-mediated oocyte maturation;Ubiquitin mediated proteolysis

Colorectal cancer;Pathways in cancer

Polito et al. Universal ready-to-use immunotherapeutic approach for the treatment of cancer: expanded and activated polyclonal γδ memory T cells.
Phosphorylation sites

Base excision repair
Base excision repair
Base excision repair
Base excision repair
Huntington's disease
Huntington's disease
Huntington's disease
mRNA surveillance pathway;RNA transport;Spliceosome
mRNA surveillance pathway;RNA transport;Spliceosome
RNA transport
Hedgehog signaling pathway
Adipocytokine signaling pathway;Fatty acid metabolism;Peroxisome;PPAR signaling pathway
Neurotrophin signaling pathway
Neurotrophin signaling pathway
Neurotrophin signaling pathway

Ribosome biogenesis in eukaryotes
Ribosome biogenesis in eukaryotes
Cytosolic DNA-sensing pathway;NOD-like receptor signaling pathway

Polito et al. Universal ready-to-use immunotherapeutic approach for the treatment of cancer: expanded and activated polyclonal γδ memory T cells.
Phosphorylation sites

B cell receptor signaling pathway
Protein processing in endoplasmic reticulum;Ubiquitin mediated proteolysis
Protein processing in endoplasmic reticulum;Ubiquitin mediated proteolysis
Protein processing in endoplasmic reticulum

Polito et al. Universal ready-to-use immunotherapeutic approach for the treatment of cancer: expanded and activated polyclonal γδ memory T cells.

Phosphorylation sites

Chronic myeloid leukemia;Fc epsilon RI signaling pathway;Fc gamma R-mediated phagocytosis;Osteoclast differentiation
Cell cycle;Cell cycle - yeast;Meiosis - yeast;Oocyte meiosis
Endocytosis

Polito et al. Universal ready-to-use immunotherapeutic approach for the treatment of cancer: expanded and activated polyclonal γδ memory T cells.
Phosphorylation sites

Fructose and mannose metabolism
Fructose and mannose metabolism
Inositol phosphate metabolism;Phagosome;Phosphatidylinositol signaling system;Regulation of actin cytoskeleton

Polito et al. Universal ready-to-use immunotherapeutic approach for the treatment of cancer: expanded and activated polyclonal γδ memory T cells.
Phosphorylation sites

RNA degradation
RNA degradation
Gap junction;GnRH signaling pathway;MAPK signaling pathway
Gap junction;GnRH signaling pathway;MAPK signaling pathway
Gap junction;GnRH signaling pathway;MAPK signaling pathway

Polito et al. Universal ready-to-use immunotherapeutic approach for the treatment of cancer: expanded and activated polyclonal γδ memory T cells.
Phosphorylation sites

Spliceosome
Spliceosome
Ribosome biogenesis in eukaryotes
Ribosome biogenesis in eukaryotes
Endocytosis;Epithelial cell signaling in Helicobacter pylori infection;Regulation of actin cytoskeleton
Endocytosis;Epithelial cell signaling in Helicobacter pylori infection;Regulation of actin cytoskeleton
Endocytosis;Epithelial cell signaling in Helicobacter pylori infection;Regulation of actin cytoskeleton
Endocytosis;Epithelial cell signaling in Helicobacter pylori infection;Regulation of actin cytoskeleton
Parkinson's disease;Protein processing in endoplasmic reticulum;Ubiquitin mediated proteolysis
Ribosome biogenesis in eukaryotes
Spliceosome
Endocytosis

Polito et al. Universal ready-to-use immunotherapeutic approach for the treatment of cancer: expanded and activated polyclonal γδ memory T cells.
Phosphorylation sites

mRNA surveillance pathway
Adipocytokine signaling pathway;Hypertrophic cardiomyopathy (HCM);Insulin signaling pathway
Adipocytokine signaling pathway;Hypertrophic cardiomyopathy (HCM);Insulin signaling pathway
Collecting duct acid secretion;Epithelial cell signaling in Helicobacter pylori infection;ko05152;Lysosome;Oxidative phosphorylation;Phagosome;Rheumatoid arthritis;Vibrio cholerae infection
Focal adhesion
Focal adhesion
Focal adhesion
Focal adhesion
Adipocytokine signaling pathway;Aldosterone-regulated sodium reabsorption;Insulin signaling pathway;Neurotrophin signaling pathway;Type II diabetes mellitus
Adipocytokine signaling pathway;Aldosterone-regulated sodium reabsorption;Insulin signaling pathway;Neurotrophin signaling pathway;Type II diabetes mellitus

Polito et al. Universal ready-to-use immunotherapeutic approach for the treatment of cancer: expanded and activated polyclonal γδ memory T cells.

Phosphorylation sites

Cytosolic DNA-sensing pathway
Cytosolic DNA-sensing pathway
Cytosolic DNA-sensing pathway
Ribosome biogenesis in eukaryotes
Ribosome biogenesis in eukaryotes
Bacterial invasion of epithelial cells
Bacterial invasion of epithelial cells
Bacterial invasion of epithelial cells
Bacterial invasion of epithelial cells
mRNA surveillance pathway;RNA transport;Spliceosome
mRNA surveillance pathway;RNA transport;Spliceosome
Peroxisome

Polito et al. Universal ready-to-use immunotherapeutic approach for the treatment of cancer: expanded and activated polyclonal γδ memory T cells.
Phosphorylation sites

Cell adhesion molecules (CAMs);Epithelial cell signaling in Helicobacter pylori infection;Leukocyte transendothelial migration;Tight junction
RNA transport
RNA transport
RNA transport
RNA transport
RNA transport
Cell cycle;Progesterone-mediated oocyte maturation
Phagosome;Vasopressin-regulated water reabsorption
Phagosome;Vasopressin-regulated water reabsorption
Phagosome;Vasopressin-regulated water reabsorption
Phagosome;Vasopressin-regulated water reabsorption
Phagosome;Vasopressin-regulated water reabsorption
Endocytosis
Endocytosis
Endocytosis
Endocytosis
Endocytosis
Cysteine and methionine metabolism
-sensing pathway;Epithelial cell signaling in Helicobacter pylori infection;Hepatitis C;MAPK signaling pathway;NOD-like receptor signaling pathway;Osteoclast differentiation;Pancreatic cancer;

Polito et al. Universal ready-to-use immunotherapeutic approach for the treatment of cancer: expanded and activated polyclonal γδ memory T cells.
Phosphorylation sites

Adherens junction;Bacterial invasion of epithelial cells;Fc gamma R-mediated phagocytosis;Regulation of actin cytoskeleton;Shigellosis
Adherens junction;Bacterial invasion of epithelial cells;Fc gamma R-mediated phagocytosis;Regulation of actin cytoskeleton;Shigellosis
Nucleotide excision repair
Nucleotide excision repair
Nucleotide excision repair
Nucleotide excision repair
ABC transporters;Antigen processing and presentation;Phagosome;Primary immunodeficiency
Ribosome
Ribosome
RNA transport

Proteasome
Endocrine and other factor-regulated calcium reabsorption;Endocytosis;Huntington's disease
[illegible] pathway;Gastric acid secretion;Glioma;GnRH signaling pathway;ko05152;Long-term potentiation;Melanogenesis;Neurotrophin signaling pathway;Olfactory transduction;Oocyte meiosis;Phot[illegible]
[illegible] pathway;Gastric acid secretion;Glioma;GnRH signaling pathway;ko05152;Long-term potentiation;Melanogenesis;Neurotrophin signaling pathway;Olfactory transduction;Oocyte meiosis;Phot[illegible]
Cell adhesion molecules (CAMs);Staphylococcus aureus infection
Cell adhesion molecules (CAMs);Staphylococcus aureus infection
Collecting duct acid secretion
ABC transporters;Antigen processing and presentation;Phagosome;Primary immunodeficiency
Axon guidance
Ribosome

Polito et al. Universal ready-to-use immunotherapeutic approach for the treatment of cancer: expanded and activated polyclonal γδ memory T cells.
Phosphorylation sites

Ribosome
RNA transport
al cells;Dilated cardiomyopathy;Focal adhesion;Hypertrophic cardiomyopathy (HCM);Leukocyte transendothelial migration;Pathogenic Escherichia coli infection;Phagosome;Phototransductio
mRNA surveillance pathway;RNA transport
mRNA surveillance pathway;RNA transport
mRNA surveillance pathway;RNA transport
mRNA surveillance pathway;RNA degradation;RNA transport
Spliceosome
MAPK signaling pathway
ate metabolism;Caprolactam degradation;Fatty acid metabolism;Gap junction;Lysine degradation;Pathogenic Escherichia coli infection;Peroxisome;Phagosome;PPAR signaling pathway;Propar
Ribosome biogenesis in eukaryotes;RNA transport
RNA transport;Viral myocarditis
AMs);ko05152;Leishmaniasis;Leukocyte transendothelial migration;Malaria;Natural killer cell mediated cytotoxicity;Phagosome;Regulation of actin cytoskeleton;Rheumatoid arthritis;Staphyloc
RNA degradation
cycle;Cell cycle - yeast;Circadian rhythm - mammal;Oocyte meiosis;Protein processing in endoplasmic reticulum;TGF-beta signaling pathway;Ubiquitin mediated proteolysis;Wnt signaling path
MAPK signaling pathway

Polito et al. Universal ready-to-use immunotherapeutic approach for the treatment of cancer: expanded and activated polyclonal γδ memory T cells.
Phosphorylation sites

mRNA surveillance pathway;RNA transport;Spliceosome
mRNA surveillance pathway;RNA transport;Spliceosome
mRNA surveillance pathway;RNA transport;Spliceosome
RNA transport;Viral myocarditis
RNA transport;Viral myocarditis
Endocytosis
Regulation of actin cytoskeleton
Gap junction;Pathogenic Escherichia coli infection;Phagosome
Gap junction;Pathogenic Escherichia coli infection;Phagosome
Gap junction;Pathogenic Escherichia coli infection;Phagosome
Valine, leucine and isoleucine degradation
Endocytosis
Cell cycle;Measles;Oocyte meiosis;p53 signaling pathway;Pathways in cancer;Progesterone-mediated oocyte maturation;Prostate cancer;Small cell lung cancer
Cell cycle;Measles;Oocyte meiosis;p53 signaling pathway;Pathways in cancer;Progesterone-mediated oocyte maturation;Prostate cancer;Small cell lung cancer
ECM-receptor interaction;Hematopoietic cell lineage;Shigellosis

Hepatitis C;Measles;Protein processing in endoplasmic reticulum
Cell cycle;Cell cycle - yeast;Meiosis - yeast
Endocytosis
ABC transporters;Vitamin digestion and absorption
Calcium signaling pathway;Insulin signaling pathway
MAPK signaling pathway
mRNA surveillance pathway
Focal adhesion;Leukocyte transendothelial migration;Regulation of actin cytoskeleton;Tight junction;Vascular smooth muscle contraction
Systemic lupus erythematosus
Systemic lupus erythematosus
Systemic lupus erythematosus
Ribosome
Biosynthesis of unsaturated fatty acids;PPAR signaling pathway
Alzheimer's disease;Epithelial cell signaling in Helicobacter pylori infection
Focal adhesion;Long-term potentiation;Regulation of actin cytoskeleton;Vascular smooth muscle contraction

Polito et al. Universal ready-to-use immunotherapeutic approach for the treatment of cancer: expanded and activated polyclonal γδ memory T cells.

Phosphorylation sites

Axon guidance
Systemic lupus erythematosus
Systemic lupus erythematosus
Spliceosome
Spliceosome
Spliceosome
Spliceosome
Spliceosome
Spliceosome
Spliceosome
Spliceosome
Spliceosome
T cell receptor signaling pathway
T cell receptor signaling pathway
RNA transport
Spliceosome
mRNA surveillance pathway

Polito et al. Universal ready-to-use immunotherapeutic approach for the treatment of cancer: expanded and activated polyclonal γδ memory T cells.
Phosphorylation sites

Lysine degradation
Arrhythmogenic right ventricular cardiomyopathy (ARVC);Dilated cardiomyopathy;Hypertrophic cardiomyopathy (HCM)
Gap junction;Pathogenic Escherichia coli infection;Phagosome
Gap junction;Pathogenic Escherichia coli infection;Phagosome
Bile secretion;Gastric acid secretion;Pancreatic secretion;Salivary secretion
oma;Leukocyte transendothelial migration;Long-term depression;Long-term potentiation;MAPK signaling pathway;MAPK signaling pathway - yeast;Melanogenesis;Natural killer cell mediated c
cules (CAMs);Dilated cardiomyopathy;ECM-receptor interaction;Focal adhesion;Hypertrophic cardiomyopathy (HCM);Leishmaniasis;Leukocyte transendothelial migration;Pathogenic Escherich
oma;Leukocyte transendothelial migration;Long-term depression;Long-term potentiation;MAPK signaling pathway;MAPK signaling pathway - yeast;Melanogenesis;Natural killer cell mediated c
oma;Leukocyte transendothelial migration;Long-term depression;Long-term potentiation;MAPK signaling pathway;MAPK signaling pathway - yeast;Melanogenesis;Natural killer cell mediated c
Hematopoietic cell lineage
Natural killer cell mediated cytotoxicity;Osteoclast differentiation;Primary immunodeficiency;T cell receptor signaling pathway
Bladder cancer;Cell cycle;Chronic myeloid leukemia;Glioma;Melanoma;Non-small cell lung cancer;Pancreatic cancer;Pathways in cancer;Prostate cancer;Small cell lung cancer
Bladder cancer;Cell cycle;Chronic myeloid leukemia;Glioma;Melanoma;Non-small cell lung cancer;Pancreatic cancer;Pathways in cancer;Prostate cancer;Small cell lung cancer
Bladder cancer;Cell cycle;Chronic myeloid leukemia;Glioma;Melanoma;Non-small cell lung cancer;Pancreatic cancer;Pathways in cancer;Prostate cancer;Small cell lung cancer
Cell cycle;Gap junction;Oocyte meiosis;p53 signaling pathway;Progesterone-mediated oocyte maturation
Cell cycle;Gap junction;Oocyte meiosis;p53 signaling pathway;Progesterone-mediated oocyte maturation
Cell cycle;Gap junction;Oocyte meiosis;p53 signaling pathway;Progesterone-mediated oocyte maturation
Amino sugar and nucleotide sugar metabolism;Glycolysis / Gluconeogenesis;Pentose phosphate pathway;Starch and sucrose metabolism
Gap junction;Pathogenic Escherichia coli infection;Phagosome
Gap junction;Pathogenic Escherichia coli infection;Phagosome
g and presentation;NOD-like receptor signaling pathway;Pathways in cancer;Plant-pathogen interaction;Progesterone-mediated oocyte maturation;Prostate cancer;Protein processing in endo
Spliceosome

Polito et al. Universal ready-to-use immunotherapeutic approach for the treatment of cancer: expanded and activated polyclonal γδ memory T cells.
Phosphorylation sites

Base excision repair
Lysosome
Non-homologous end-joining
Base excision repair;Primary immunodeficiency
Leishmaniasis;Leukocyte transendothelial migration;Osteoclast differentiation;Phagosome
Cell adhesion molecules (CAMs);Leukocyte transendothelial migration
Bacterial invasion of epithelial cells;Pathogenic Escherichia coli infection;Shigellosis;Tight junction
Dorso-ventral axis formation;MAPK signaling pathway - fly
Purine metabolism;Pyrimidine metabolism
Ribosome
Cell adhesion molecules (CAMs)
oma;Leukocyte transendothelial migration;Long-term depression;Long-term potentiation;MAPK signaling pathway;MAPK signaling pathway - yeast;Melanogenesis;Natural killer cell mediated c
Osteoclast differentiation
naling pathway;Hedgehog signaling pathway;Insulin signaling pathway;Long-term potentiation;MAPK signaling pathway;Meiosis - yeast;Melanogenesis;Olfactory transduction;Oocyte meiosis;I
Ribosome
Base excision repair;DNA replication;Mismatch repair;Nucleotide excision repair
Base excision repair;DNA replication;Mismatch repair;Nucleotide excision repair
Pathogenic Escherichia coli infection

Polito et al. Universal ready-to-use immunotherapeutic approach for the treatment of cancer: expanded and activated polyclonal γδ memory T cells.
Phosphorylation sites

RNA transport
Focal adhesion;MAPK signaling pathway
Ribosome
Axon guidance;Fc gamma R-mediated phagocytosis;Regulation of actin cytoskeleton
Tight junction;Vascular smooth muscle contraction
nyopathy (ARVC);Cell adhesion molecules (CAMs);Dilated cardiomyopathy;ECM-receptor interaction;Focal adhesion;Hypertrophic cardiomyopathy (HCM);Intestinal immune network for IgA pro
Aldosterone-regulated sodium reabsorption;Cell cycle;Neurotrophin signaling pathway;Oocyte meiosis;p53 signaling pathway
Calcium signaling pathway;Inositol phosphate metabolism;Phosphatidylinositol signaling system
Cell cycle;TGF-beta signaling pathway
Cell cycle;TGF-beta signaling pathway
Acute myeloid leukemia;Endocytosis;Pathways in cancer;Ubiquitin mediated proteolysis
Cell cycle;Focal adhesion;Jak-STAT signaling pathway;Measles;p53 signaling pathway;Wnt signaling pathway
Glutathione metabolism;p53 signaling pathway;Purine metabolism;Pyrimidine metabolism
nediated phagocytosis;Focal adhesion;Glioma;Hepatitis C;Insulin signaling pathway;Jak-STAT signaling pathway;ko05152;MAPK signaling pathway;Measles;Melanoma;mTOR signaling pathway
DNA replication;Mismatch repair;Nucleotide excision repair
Basal transcription factors
Basal transcription factors
-term depression;Long-term potentiation;MAPK signaling pathway;Melanogenesis;Melanoma;Natural killer cell mediated cytotoxicity;Neurotrophin signaling pathway;Non-small cell lung canc
Ribosome
Ribosome
mRNA surveillance pathway;RNA transport;Spliceosome
Endocytosis
DNA replication;Mismatch repair;Nucleotide excision repair

Polito et al. Universal ready-to-use immunotherapeutic approach for the treatment of cancer: expanded and activated polyclonal γδ memory T cells.
Phosphorylation sites

Dorso-ventral axis formation;MAPK signaling pathway - fly
Endocytosis;Insulin signaling pathway;Tight junction
Chemokine signaling pathway;Endocytosis
Fatty acid biosynthesis;Insulin signaling pathway
Chemokine signaling pathway;Endocytosis;MAPK signaling pathway;Phototransduction
Cell cycle;Cell cycle - yeast;DNA replication;Meiosis - yeast
Nicotinate and nicotinamide metabolism;Purine metabolism;Pyrimidine metabolism
Basal transcription factors;Cell cycle;Nucleotide excision repair
naling pathway;Hedgehog signaling pathway;Insulin signaling pathway;Long-term potentiation;MAPK signaling pathway;Meiosis - yeast;Melanogenesis;Olfactory transduction;Oocyte meiosis;[illegible]
Basal transcription factors;Cell cycle;Nucleotide excision repair
Spliceosome
RNA transport
RNA transport

Polito et al. Universal ready-to-use immunotherapeutic approach for the treatment of cancer: expanded and activated polyclonal γδ memory T cells.
Phosphorylation sites

Cytosolic DNA-sensing pathway;Measles
al cells;Dilated cardiomyopathy;Focal adhesion;Hypertrophic cardiomyopathy (HCM);Leukocyte transendothelial migration;Pathogenic Escherichia coli infection;Phagosome;Phototransductio
RNA transport
Ribosome
Axon guidance;Chemokine signaling pathway;Cytokine-cytokine receptor interaction;Endocytosis;Intestinal immune network for IgA production;Leukocyte transendothelial migration
Ribosome
Focal adhesion;Insulin signaling pathway;Long-term potentiation;Meiosis - yeast;Oocyte meiosis;Regulation of actin cytoskeleton;Vascular smooth muscle contraction
Ribosome
Spliceosome
Regulation of actin cytoskeleton
Cardiac muscle contraction;Dilated cardiomyopathy;Hypertrophic cardiomyopathy (HCM);Vascular smooth muscle contraction
Insulin signaling pathway;mTOR signaling pathway;Ribosome
Ribosome
Spliceosome
Gap junction;Pathogenic Escherichia coli infection;Phagosome
Basal transcription factors
dherens junction;Cell cycle;Chagas disease (American trypanosomiasis);Colorectal cancer;Endocytosis;Pancreatic cancer;Pathways in cancer;TGF-beta signaling pathway;Wnt signaling pathwa
Alzheimer's disease;Axon guidance
Bacterial invasion of epithelial cells;Endocrine and other factor-regulated calcium reabsorption;Endocytosis;Huntington's disease;Lysosome
Spliceosome
Tight junction;Vascular smooth muscle contraction
Maturity onset diabetes of the young

Polito et al. Universal ready-to-use immunotherapeutic approach for the treatment of cancer: expanded and activated polyclonal γδ memory T cells.
Phosphorylation sites

Tight junction;Vascular smooth muscle contraction
Tight junction;Vascular smooth muscle contraction
Tight junction;Vascular smooth muscle contraction
Cell cycle;TGF-beta signaling pathway
Cell cycle;Cell cycle - yeast;Meiosis - yeast;Oocyte meiosis;Ubiquitin mediated proteolysis
Spliceosome
ling pathway;Apoptosis;Hepatitis C;MAPK signaling pathway;Osteoclast differentiation;Pathways in cancer;Protein processing in endoplasmic reticulum;RIG-I-like receptor signaling pathway;S
Chemokine signaling pathway;Regulation of actin cytoskeleton
MAPK signaling pathway;Non-small cell lung cancer;Pathways in cancer
MAPK signaling pathway;Non-small cell lung cancer;Pathways in cancer
MAPK signaling pathway;Non-small cell lung cancer;Pathways in cancer
Fc epsilon RI signaling pathway;Natural killer cell mediated cytotoxicity;Osteoclast differentiation;T cell receptor signaling pathway
Axon guidance;ErbB signaling pathway;Focal adhesion;MAPK signaling pathway;Regulation of actin cytoskeleton;Renal cell carcinoma;T cell receptor signaling pathway
Measles
Apoptosis;Cell cycle;p53 signaling pathway

Polito et al. Universal ready-to-use immunotherapeutic approach for the treatment of cancer: expanded and activated polyclonal γδ memory T cells.
Phosphorylation sites

Cell cycle;Cell cycle - yeast;Meiosis - yeast
Spliceosome
pathway;Gastric acid secretion;Glioma;GnRH signaling pathway;ko05152;Long-term potentiation;Melanogenesis;Neurotrophin signaling pathway;Olfactory transduction;Oocyte meiosis;Phot
pathway;Gastric acid secretion;Glioma;GnRH signaling pathway;ko05152;Long-term potentiation;Melanogenesis;Neurotrophin signaling pathway;Olfactory transduction;Oocyte meiosis;Phot
Huntington's disease;Vasopressin-regulated water reabsorption
Spliceosome
Spliceosome
mRNA surveillance pathway;RNA transport;Spliceosome
Bacterial invasion of epithelial cells;Pathogenic Escherichia coli infection;Shigellosis;Tight junction
Endocytosis
Spliceosome
dherens junction;Cell cycle;Chagas disease (American trypanosomiasis);Colorectal cancer;Endocytosis;Pancreatic cancer;Pathways in cancer;TGF-beta signaling pathway;Wnt signaling pathwa

ishmaniasis;Leukocyte transendothelial migration;MAPK signaling pathway;MAPK signaling pathway - yeast;Neurotrophin signaling pathway;NOD-like receptor signaling pathway;Osteoclast di
mRNA surveillance pathway
mRNA surveillance pathway
Regulation of actin cytoskeleton
Regulation of actin cytoskeleton
Cell cycle - yeast
Cell cycle - yeast
RNA degradation

mTOR signaling pathway

Ubiquitin mediated proteolysis

Polito et al. Universal ready-to-use immunotherapeutic approach for the treatment of cancer: expanded and activated polyclonal γδ memory T cells.

Phosphorylation sites

mRNA surveillance pathway
RNA transport
RNA transport
RNA transport
Chemokine signaling pathway;Fc gamma R-mediated phagocytosis
B cell receptor signaling pathway;Fc epsilon RI signaling pathway;Fc gamma R-mediated phagocytosis;Insulin signaling pathway;Phosphatidylinositol signaling system
Homologous recombination;Non-homologous end-joining
Pathogenic Escherichia coli infection

Polito et al. Universal ready-to-use immunotherapeutic approach for the treatment of cancer: expanded and activated polyclonal γδ memory T cells.
Phosphorylation sites

sugar and nucleotide sugar metabolism;Galactose metabolism;Glycolysis / Gluconeogenesis;Pentose phosphate pathway;Purine metabolism;Starch and sucrose metabolism;Streptomycin biosy
Huntington's disease
RNA transport
GnRH signaling pathway;MAPK signaling pathway;Neurotrophin signaling pathway
Parkinson's disease
Lysosome
Lysine degradation
Lysine degradation

Polito et al. Universal ready-to-use immunotherapeutic approach for the treatment of cancer: expanded and activated polyclonal γδ memory T cells.
Phosphorylation sites

Oocyte meiosis
Cell cycle - yeast
RNA degradation
Protein processing in endoplasmic reticulum
Pantothenate and CoA biosynthesis
Drug metabolism - other enzymes;Pyrimidine metabolism

RNA transport
Base excision repair
Base excision repair

Polito et al. Universal ready-to-use immunotherapeutic approach for the treatment of cancer: expanded and activated polyclonal γδ memory T cells.
Phosphorylation sites

Focal adhesion
Chagas disease (American trypanosomiasis);Hematopoietic cell lineage;Measles;Primary immunodeficiency;T cell receptor signaling pathway
Cell cycle;Measles;Oocyte meiosis;p53 signaling pathway;Pathways in cancer;Progesterone-mediated oocyte maturation;Prostate cancer;Small cell lung cancer
Cell cycle;Measles;Oocyte meiosis;p53 signaling pathway;Pathways in cancer;Progesterone-mediated oocyte maturation;Prostate cancer;Small cell lung cancer
thway;Fc epsilon RI signaling pathway;Focal adhesion;Gap junction;GnRH signaling pathway;ko05152;Measles;Natural killer cell mediated cytotoxicity;Osteoclast differentiation;Pathogenic Esc
Acute myeloid leukemia;Chemokine signaling pathway;Chronic myeloid leukemia;ErbB signaling pathway;Jak-STAT signaling pathway;ko05152;Measles;Pathways in cancer
Natural killer cell mediated cytotoxicity;Osteoclast differentiation;Primary immunodeficiency;T cell receptor signaling pathway
Natural killer cell mediated cytotoxicity;Osteoclast differentiation;Primary immunodeficiency;T cell receptor signaling pathway
Cell cycle;Gap junction;Oocyte meiosis;p53 signaling pathway;Progesterone-mediated oocyte maturation
Cell cycle;Gap junction;Oocyte meiosis;p53 signaling pathway;Progesterone-mediated oocyte maturation
Glycolysis / Gluconeogenesis;Methane metabolism;RNA degradation
aling pathway;Fc epsilon RI signaling pathway;Fc gamma R-mediated phagocytosis;Focal adhesion;Leukocyte transendothelial migration;Natural killer cell mediated cytotoxicity;Regulation of a
Glycolysis / Gluconeogenesis;Methane metabolism
Chagas disease (American trypanosomiasis);Natural killer cell mediated cytotoxicity;T cell receptor signaling pathway
Chagas disease (American trypanosomiasis);Natural killer cell mediated cytotoxicity;T cell receptor signaling pathway
Chagas disease (American trypanosomiasis);Natural killer cell mediated cytotoxicity;T cell receptor signaling pathway
Chagas disease (American trypanosomiasis);Natural killer cell mediated cytotoxicity;T cell receptor signaling pathway
Chagas disease (American trypanosomiasis);Natural killer cell mediated cytotoxicity;T cell receptor signaling pathway
cal adhesion;Gap junction;Glioma;GnRH signaling pathway;Hepatitis C;Insulin signaling pathway;ko05152;Leishmaniasis;Long-term depression;Long-term potentiation;MAPK signaling pathway
te myeloid leukemia;Adipocytokine signaling pathway;Chemokine signaling pathway;Hepatitis C;Jak-STAT signaling pathway;ko05152;Measles;Pancreatic cancer;Pathways in cancer;Toxoplasm
rens junction;Bacterial invasion of epithelial cells;Chemokine signaling pathway;Fc gamma R-mediated phagocytosis;Pathogenic Escherichia coli infection;Regulation of actin cytoskeleton;Shige
ay;Circadian rhythm - fly;Colorectal cancer;Endometrial cancer;ErbB signaling pathway;Focal adhesion;Hedgehog signaling pathway;Hepatitis C;Insulin signaling pathway;Measles;Melanogenes

Systemic lupus erythematosus
mia;Epithelial cell signaling in Helicobacter pylori infection;Jak-STAT signaling pathway;Leukocyte transendothelial migration;MAPK signaling pathway - fly;Natural killer cell mediated cytotoxic
Measles
Jak-STAT signaling pathway
ishmaniasis;Leukocyte transendothelial migration;MAPK signaling pathway;MAPK signaling pathway - yeast;Neurotrophin signaling pathway;NOD-like receptor signaling pathway;Osteoclast di
Lysosome
Lysosome
Measles;NOD-like receptor signaling pathway
Measles;NOD-like receptor signaling pathway
Measles;NOD-like receptor signaling pathway
Measles;NOD-like receptor signaling pathway
onic myeloid leukemia;Colorectal cancer;Endometrial cancer;ErbB signaling pathway;Jak-STAT signaling pathway;MAPK signaling pathway;Pathways in cancer;Small cell lung cancer;TGF-beta si
RNA transport
RNA transport
RNA transport
RNA transport
Proteasome

Endocytosis;Fc gamma R-mediated phagocytosis
Endocytosis;Fc gamma R-mediated phagocytosis
Axon guidance
Axon guidance

Polito et al. Universal ready-to-use immunotherapeutic approach for the treatment of cancer: expanded and activated polyclonal γδ memory T cells.
Phosphorylation sites

Axon guidance
Axon guidance
Mineral absorption
Mineral absorption
Cell adhesion molecules (CAMs);Staphylococcus aureus infection
Cell adhesion molecules (CAMs);Staphylococcus aureus infection

Polito et al. Universal ready-to-use immunotherapeutic approach for the treatment of cancer: expanded and activated polyclonal γδ memory T cells.
Phosphorylation sites

ocessing and presentation;Autoimmune thyroid disease;Cell adhesion molecules (CAMs);Endocytosis;Graft-versus-host disease;Natural killer cell mediated cytotoxicity;Phagosome;Type I diabe
ocessing and presentation;Autoimmune thyroid disease;Cell adhesion molecules (CAMs);Endocytosis;Graft-versus-host disease;Natural killer cell mediated cytotoxicity;Phagosome;Type I diabe
ocessing and presentation;Autoimmune thyroid disease;Cell adhesion molecules (CAMs);Endocytosis;Graft-versus-host disease;Natural killer cell mediated cytotoxicity;Phagosome;Type I diabe
ocessing and presentation;Autoimmune thyroid disease;Cell adhesion molecules (CAMs);Endocytosis;Graft-versus-host disease;Natural killer cell mediated cytotoxicity;Phagosome;Type I diabe
Bacterial invasion of epithelial cells;Chemokine signaling pathway;Focal adhesion;Leukocyte transendothelial migration;Regulation of actin cytoskeleton;VEGF signaling pathway
Bacterial invasion of epithelial cells;Chemokine signaling pathway;Focal adhesion;Leukocyte transendothelial migration;Regulation of actin cytoskeleton;VEGF signaling pathway
Bacterial invasion of epithelial cells;Chemokine signaling pathway;Focal adhesion;Leukocyte transendothelial migration;Regulation of actin cytoskeleton;VEGF signaling pathway
Bacterial invasion of epithelial cells;Chemokine signaling pathway;Focal adhesion;Leukocyte transendothelial migration;Regulation of actin cytoskeleton;VEGF signaling pathway

Polito et al. Universal ready-to-use immunotherapeutic approach for the treatment of cancer: expanded and activated polyclonal γδ memory T cells.
Phosphorylation sites

Tight junction
Tight junction
RNA transport
RNA transport
RNA transport
mRNA surveillance pathway;RNA transport
mRNA surveillance pathway;RNA transport
mRNA surveillance pathway;RNA transport
mRNA surveillance pathway;RNA transport
mRNA surveillance pathway;RNA transport
mRNA surveillance pathway;RNA transport
mRNA surveillance pathway;RNA transport

mRNA surveillance pathway;RNA transport
mRNA surveillance pathway;RNA transport
mRNA surveillance pathway;RNA transport
mRNA surveillance pathway;RNA transport
mRNA surveillance pathway;RNA transport
mRNA surveillance pathway;RNA transport
mRNA surveillance pathway;RNA transport
mRNA surveillance pathway;RNA transport
mRNA surveillance pathway;RNA transport
mRNA surveillance pathway;RNA transport
mRNA surveillance pathway;RNA transport
mRNA surveillance pathway;RNA transport
mRNA surveillance pathway;RNA transport
mRNA surveillance pathway;RNA transport
mRNA surveillance pathway;RNA transport
mRNA surveillance pathway;RNA transport
mRNA surveillance pathway;RNA transport
mRNA surveillance pathway;RNA transport
mRNA surveillance pathway;RNA transport
mRNA surveillance pathway;RNA transport
mRNA surveillance pathway;RNA transport
mRNA surveillance pathway;RNA transport
mRNA surveillance pathway;RNA transport
mRNA surveillance pathway;RNA transport
mRNA surveillance pathway;RNA transport

Polito et al. Universal ready-to-use immunotherapeutic approach for the treatment of cancer: expanded and activated polyclonal γδ memory T cells.
Phosphorylation sites

Notch signaling pathway
Notch signaling pathway
Notch signaling pathway
Apoptosis;Insulin signaling pathway
Protein processing in endoplasmic reticulum
Protein processing in endoplasmic reticulum
RNA degradation
RNA degradation
mRNA surveillance pathway;RNA transport;Spliceosome
mRNA surveillance pathway;RNA transport;Spliceosome
mRNA surveillance pathway;RNA transport;Spliceosome

mRNA surveillance pathway;RNA transport;Spliceosome
mRNA surveillance pathway;RNA transport;Spliceosome
mRNA surveillance pathway;RNA transport;Spliceosome
mRNA surveillance pathway;RNA transport;Spliceosome
mRNA surveillance pathway;RNA transport;Spliceosome
mRNA surveillance pathway;RNA transport;Spliceosome
mRNA surveillance pathway;RNA transport;Spliceosome
Ribosome biogenesis in eukaryotes
Ribosome biogenesis in eukaryotes
Ribosome biogenesis in eukaryotes
Spliceosome
mTOR signaling pathway;RNA transport
mTOR signaling pathway;RNA transport
RNA transport;Viral myocarditis
RNA transport;Viral myocarditis
RNA transport;Viral myocarditis
RNA transport;Viral myocarditis
RNA transport;Viral myocarditis

Polito et al. Universal ready-to-use immunotherapeutic approach for the treatment of cancer: expanded and activated polyclonal γδ memory T cells.

Phosphorylation sites

Hematopoietic cell lineage
Hematopoietic cell lineage
mRNA surveillance pathway
mRNA surveillance pathway
MAPK signaling pathway;T cell receptor signaling pathway
GnRH signaling pathway;MAPK signaling pathway
GnRH signaling pathway;MAPK signaling pathway
Gap junction;Pathogenic Escherichia coli infection;Phagosome
Gap junction;Pathogenic Escherichia coli infection;Phagosome
Base excision repair
Base excision repair

vay;Focal adhesion;Gap junction;Glioma;GnRH signaling pathway;Hepatitis C;Insulin signaling pathway;Jak-STAT signaling pathway;MAPK signaling pathway;MAPK signaling pathway - fly;Natur
vay;Focal adhesion;Gap junction;Glioma;GnRH signaling pathway;Hepatitis C;Insulin signaling pathway;Jak-STAT signaling pathway;MAPK signaling pathway;MAPK signaling pathway - fly;Natur

vay;Focal adhesion;Gap junction;Glioma;GnRH signaling pathway;Hepatitis C;Insulin signaling pathway;Jak-STAT signaling pathway;MAPK signaling pathway;MAPK signaling pathway - fly;Natur
vay;Focal adhesion;Gap junction;Glioma;GnRH signaling pathway;Hepatitis C;Insulin signaling pathway;Jak-STAT signaling pathway;MAPK signaling pathway;MAPK signaling pathway - fly;Natur
Spliceosome
Spliceosome
ECM-receptor interaction;Hematopoietic cell lineage;Shigellosis
ECM-receptor interaction;Hematopoietic cell lineage;Shigellosis

Polito et al. Universal ready-to-use immunotherapeutic approach for the treatment of cancer: expanded and activated polyclonal γδ memory T cells.
Phosphorylation sites

Adherens junction;Leukocyte transendothelial migration;Tight junction
Spliceosome
Spliceosome
Spliceosome
Spliceosome
Spliceosome
Spliceosome
Spliceosome
Spliceosome
Spliceosome

Focal adhesion;Leukocyte transendothelial migration;Regulation of actin cytoskeleton;Tight junction;Vascular smooth muscle contraction
MAPK signaling pathway
MAPK signaling pathway
mRNA surveillance pathway;RNA transport;Spliceosome
mRNA surveillance pathway;RNA transport;Spliceosome

Polito et al. Universal ready-to-use immunotherapeutic approach for the treatment of cancer: expanded and activated polyclonal γδ memory T cells.
Phosphorylation sites

Ribosome biogenesis in eukaryotes
Ribosome biogenesis in eukaryotes
Ribosome biogenesis in eukaryotes
Ribosome biogenesis in eukaryotes
Ribosome biogenesis in eukaryotes
Ribosome biogenesis in eukaryotes
Phagosome;SNARE interactions in vesicular transport
Spliceosome
Spliceosome
Chemokine signaling pathway;Endometrial cancer;Neurotrophin signaling pathway;Non-small cell lung cancer
Chemokine signaling pathway;Endometrial cancer;Neurotrophin signaling pathway;Non-small cell lung cancer
Fc epsilon RI signaling pathway;Fc gamma R-mediated phagocytosis;Natural killer cell mediated cytotoxicity;T cell receptor signaling pathway
Fc epsilon RI signaling pathway;Fc gamma R-mediated phagocytosis;Natural killer cell mediated cytotoxicity;T cell receptor signaling pathway
NOD-like receptor signaling pathway
NOD-like receptor signaling pathway

Polito et al. Universal ready-to-use immunotherapeutic approach for the treatment of cancer: expanded and activated polyclonal γδ memory T cells.

Phosphorylation sites

Spliceosome
Spliceosome
Ribosome biogenesis in eukaryotes
Ribosome biogenesis in eukaryotes
Ribosome biogenesis in eukaryotes
Ribosome biogenesis in eukaryotes
Ribosome biogenesis in eukaryotes
Systemic lupus erythematosus
Systemic lupus erythematosus

Polito et al. Universal ready-to-use immunotherapeutic approach for the treatment of cancer: expanded and activated polyclonal γδ memory T cells.

Phosphorylation sites

Spliceosome
Spliceosome
Spliceosome
Spliceosome
Spliceosome
Spliceosome
Spliceosome
Spliceosome
Spliceosome
Spliceosome
Spliceosome
Spliceosome
Spliceosome
Spliceosome
Spliceosome
Spliceosome
MAPK signaling pathway;Neurotrophin signaling pathway
MAPK signaling pathway;Neurotrophin signaling pathway
MAPK signaling pathway;Neurotrophin signaling pathway
MAPK signaling pathway;Neurotrophin signaling pathway
RNA transport

Polito et al. Universal ready-to-use immunotherapeutic approach for the treatment of cancer: expanded and activated polyclonal γδ memory T cells.
Phosphorylation sites

Protein export
Insulin signaling pathway
Insulin signaling pathway
Neuroactive ligand-receptor interaction

Polito et al. Universal ready-to-use immunotherapeutic approach for the treatment of cancer: expanded and activated polyclonal γδ memory T cells.
Phosphorylation sites

Neuroactive ligand-receptor interaction
pathway;Graft-versus-host disease;Hematopoietic cell lineage;Hepatitis C;Hypertrophic cardiomyopathy (HCM);ko05152;Leishmaniasis;Malaria;MAPK signaling pathway;Natural killer cell medi
pathway;Graft-versus-host disease;Hematopoietic cell lineage;Hepatitis C;Hypertrophic cardiomyopathy (HCM);ko05152;Leishmaniasis;Malaria;MAPK signaling pathway;Natural killer cell medi
Arrhythmogenic right ventricular cardiomyopathy (ARVC);Dilated cardiomyopathy;Hypertrophic cardiomyopathy (HCM)
Arrhythmogenic right ventricular cardiomyopathy (ARVC);Dilated cardiomyopathy;Hypertrophic cardiomyopathy (HCM)
g pathway;Hepatitis C;Insulin signaling pathway;ko05152;Long-term depression;Long-term potentiation;MAPK signaling pathway;Melanogenesis;Melanoma;Natural killer cell mediated cytoto
oma;Leukocyte transendothelial migration;Long-term depression;Long-term potentiation;MAPK signaling pathway;MAPK signaling pathway - yeast;Melanogenesis;Natural killer cell mediated c
oma;Leukocyte transendothelial migration;Long-term depression;Long-term potentiation;MAPK signaling pathway;MAPK signaling pathway - yeast;Melanogenesis;Natural killer cell mediated c
oma;Leukocyte transendothelial migration;Long-term depression;Long-term potentiation;MAPK signaling pathway;MAPK signaling pathway - yeast;Melanogenesis;Natural killer cell mediated c
Hematopoietic cell lineage
;Fc epsilon RI signaling pathway;Focal adhesion;Measles;Natural killer cell mediated cytotoxicity;Osteoclast differentiation;Pathogenic Escherichia coli infection;Prion diseases;T cell receptor si
;Fc epsilon RI signaling pathway;Focal adhesion;Measles;Natural killer cell mediated cytotoxicity;Osteoclast differentiation;Pathogenic Escherichia coli infection;Prion diseases;T cell receptor si
Bladder cancer;Cell cycle;Chronic myeloid leukemia;Glioma;Melanoma;Non-small cell lung cancer;Pancreatic cancer;Pathways in cancer;Prostate cancer;Small cell lung cancer
Bladder cancer;Cell cycle;Chronic myeloid leukemia;Glioma;Melanoma;Non-small cell lung cancer;Pancreatic cancer;Pathways in cancer;Prostate cancer;Small cell lung cancer
Bladder cancer;Cell cycle;Chronic myeloid leukemia;Glioma;Melanoma;Non-small cell lung cancer;Pancreatic cancer;Pathways in cancer;Prostate cancer;Small cell lung cancer
Bladder cancer;Cell cycle;Chronic myeloid leukemia;Glioma;Melanoma;Non-small cell lung cancer;Pancreatic cancer;Pathways in cancer;Prostate cancer;Small cell lung cancer
Bladder cancer;Cell cycle;Chronic myeloid leukemia;Glioma;Melanoma;Non-small cell lung cancer;Pancreatic cancer;Pathways in cancer;Prostate cancer;Small cell lung cancer
Bladder cancer;Cell cycle;Chronic myeloid leukemia;Glioma;Melanoma;Non-small cell lung cancer;Pancreatic cancer;Pathways in cancer;Prostate cancer;Small cell lung cancer
Cell adhesion molecules (CAMs);Hematopoietic cell lineage
Cell adhesion molecules (CAMs);Hematopoietic cell lineage
Amino sugar and nucleotide sugar metabolism;Glycolysis / Gluconeogenesis;Pentose phosphate pathway;Starch and sucrose metabolism
Gap junction;Pathogenic Escherichia coli infection;Phagosome
ceptor signaling pathway;Chemokine signaling pathway;Epithelial cell signaling in Helicobacter pylori infection;Fc epsilon RI signaling pathway;Fc gamma R-mediated phagocytosis;Long-term de
ceptor signaling pathway;Chemokine signaling pathway;Epithelial cell signaling in Helicobacter pylori infection;Fc epsilon RI signaling pathway;Fc gamma R-mediated phagocytosis;Long-term de
g and presentation;NOD-like receptor signaling pathway;Pathways in cancer;Plant-pathogen interaction;Progesterone-mediated oocyte maturation;Prostate cancer;Protein processing in endo
g and presentation;NOD-like receptor signaling pathway;Pathways in cancer;Plant-pathogen interaction;Progesterone-mediated oocyte maturation;Prostate cancer;Protein processing in endo
g and presentation;NOD-like receptor signaling pathway;Pathways in cancer;Plant-pathogen interaction;Progesterone-mediated oocyte maturation;Prostate cancer;Protein processing in endo
g and presentation;NOD-like receptor signaling pathway;Pathways in cancer;Plant-pathogen interaction;Progesterone-mediated oocyte maturation;Prostate cancer;Protein processing in endo
Butanoate metabolism;Citrate cycle (TCA cycle);Glycolysis / Gluconeogenesis;Pyruvate metabolism;Valine, leucine and isoleucine biosynthesis
Butanoate metabolism;Citrate cycle (TCA cycle);Glycolysis / Gluconeogenesis;Pyruvate metabolism;Valine, leucine and isoleucine biosynthesis

Polito et al. Universal ready-to-use immunotherapeutic approach for the treatment of cancer: expanded and activated polyclonal γδ memory T cells.
Phosphorylation sites

Spliceosome
Spliceosome
Spliceosome
Apoptosis;Insulin signaling pathway
Apoptosis;Insulin signaling pathway
Tight junction
Tight junction
Tight junction
Tight junction
Tight junction
Chronic myeloid leukemia;Pathways in cancer
Chronic myeloid leukemia;Pathways in cancer
Lysosome
Lysosome
Lysosome
Lysosome
Tight junction;Viral myocarditis
Tight junction;Viral myocarditis
ECM-receptor interaction;Hematopoietic cell lineage
s (CAMs);Dilated cardiomyopathy;ECM-receptor interaction;Focal adhesion;Hematopoietic cell lineage;Hypertrophic cardiomyopathy (HCM);Intestinal immune network for IgA production;Leis
s (CAMs);Dilated cardiomyopathy;ECM-receptor interaction;Focal adhesion;Hematopoietic cell lineage;Hypertrophic cardiomyopathy (HCM);Intestinal immune network for IgA production;Leis
Apoptosis;Insulin signaling pathway
Apoptosis;Insulin signaling pathway
Chemokine signaling pathway;Fc gamma R-mediated phagocytosis;Leishmaniasis;Leukocyte transendothelial migration;Osteoclast differentiation;Phagosome
Chemokine signaling pathway;Fc gamma R-mediated phagocytosis;Leishmaniasis;Leukocyte transendothelial migration;Osteoclast differentiation;Phagosome
Cytokine-cytokine receptor interaction;Endocytosis;Jak-STAT signaling pathway;Measles

Cytokine-cytokine receptor interaction;Endocytosis;Jak-STAT signaling pathway;Measles
Dorso-ventral axis formation;MAPK signaling pathway - fly
Dorso-ventral axis formation;MAPK signaling pathway - fly
Osteoclast differentiation
Cell adhesion molecules (CAMs)
Cell adhesion molecules (CAMs)
MAPK signaling pathway
MAPK signaling pathway
Osteoclast differentiation
Osteoclast differentiation
MAPK signaling pathway;Osteoclast differentiation
MAPK signaling pathway;Osteoclast differentiation
Pyrimidine metabolism
Pyrimidine metabolism
Pyrimidine metabolism
Base excision repair;DNA replication;Mismatch repair;Nucleotide excision repair
Base excision repair;DNA replication;Mismatch repair;Nucleotide excision repair
Base excision repair;DNA replication;Mismatch repair;Nucleotide excision repair
Base excision repair;DNA replication;Mismatch repair;Nucleotide excision repair
Bile secretion;Cardiac muscle contraction;Gastric acid secretion;Pancreatic secretion;Regulation of actin cytoskeleton;Salivary secretion
way;Epithelial cell signaling in Helicobacter pylori infection;Hepatitis C;ko05152;Leishmaniasis;MAPK signaling pathway;Measles;Neurotrophin signaling pathway;NOD-like receptor signaling p
way;Epithelial cell signaling in Helicobacter pylori infection;Hepatitis C;ko05152;Leishmaniasis;MAPK signaling pathway;Measles;Neurotrophin signaling pathway;NOD-like receptor signaling p
RNA transport
RNA transport
RNA transport

Polito et al. Universal ready-to-use immunotherapeutic approach for the treatment of cancer: expanded and activated polyclonal γδ memory T cells.
Phosphorylation sites

Focal adhesion;MAPK signaling pathway
Focal adhesion;MAPK signaling pathway
Calcium signaling pathway;Pancreatic secretion;Salivary secretion
Calcium signaling pathway;Pancreatic secretion;Salivary secretion
Huntington's disease;Purine metabolism;Pyrimidine metabolism;RNA polymerase
Huntington's disease;Purine metabolism;Pyrimidine metabolism;RNA polymerase
Huntington's disease;Purine metabolism;Pyrimidine metabolism;RNA polymerase
Huntington's disease;Purine metabolism;Pyrimidine metabolism;RNA polymerase
Huntington's disease;Purine metabolism;Pyrimidine metabolism;RNA polymerase
Huntington's disease;Purine metabolism;Pyrimidine metabolism;RNA polymerase
Huntington's disease;Purine metabolism;Pyrimidine metabolism;RNA polymerase
Huntington's disease;Purine metabolism;Pyrimidine metabolism;RNA polymerase
Huntington's disease;Purine metabolism;Pyrimidine metabolism;RNA polymerase
Huntington's disease;Purine metabolism;Pyrimidine metabolism;RNA polymerase
Huntington's disease;Purine metabolism;Pyrimidine metabolism;RNA polymerase
Huntington's disease;Purine metabolism;Pyrimidine metabolism;RNA polymerase
Cell cycle;Cell cycle - yeast;DNA replication;Meiosis - yeast
mune thyroid disease;Chagas disease (American trypanosomiasis);Cytokine-cytokine receptor interaction;Graft-versus-host disease;MAPK signaling pathway;Measles;Natural killer cell mediat
mune thyroid disease;Chagas disease (American trypanosomiasis);Cytokine-cytokine receptor interaction;Graft-versus-host disease;MAPK signaling pathway;Measles;Natural killer cell mediat
Antigen processing and presentation;Phagosome;Protein processing in endoplasmic reticulum
Antigen processing and presentation;Phagosome;Protein processing in endoplasmic reticulum
Calcium signaling pathway;Inositol phosphate metabolism;Phosphatidylinositol signaling system
Calcium signaling pathway;Inositol phosphate metabolism;Phosphatidylinositol signaling system
Cell cycle;TGF-beta signaling pathway
Cell cycle;TGF-beta signaling pathway

Polito et al. Universal ready-to-use immunotherapeutic approach for the treatment of cancer: expanded and activated polyclonal γδ memory T cells.

Phosphorylation sites

Acute myeloid leukemia;Endocytosis;Pathways in cancer;Ubiquitin mediated proteolysis
Acute myeloid leukemia;Endocytosis;Pathways in cancer;Ubiquitin mediated proteolysis
Acute myeloid leukemia;Endocytosis;Pathways in cancer;Ubiquitin mediated proteolysis
Acute myeloid leukemia;Endocytosis;Pathways in cancer;Ubiquitin mediated proteolysis
Acute myeloid leukemia;Endocytosis;Pathways in cancer;Ubiquitin mediated proteolysis
Acute myeloid leukemia;Endocytosis;Pathways in cancer;Ubiquitin mediated proteolysis
Acute myeloid leukemia;Endocytosis;Pathways in cancer;Ubiquitin mediated proteolysis
Chemokine signaling pathway;Cytokine-cytokine receptor interaction
Chemokine signaling pathway;Cytokine-cytokine receptor interaction
Chemokine signaling pathway;Endocytosis
Neuroactive ligand-receptor interaction
Neuroactive ligand-receptor interaction
DNA replication;Mismatch repair;Nucleotide excision repair
DNA replication;Mismatch repair;Nucleotide excision repair
Basal transcription factors

Polito et al. Universal ready-to-use immunotherapeutic approach for the treatment of cancer: expanded and activated polyclonal γδ memory T cells.
Phosphorylation sites

Spliceosome
Dorso-ventral axis formation;MAPK signaling pathway - fly
Huntington's disease
Huntington's disease
Huntington's disease
Huntington's disease
Chemokine signaling pathway;Endocytosis
Proteasome
Calcium signaling pathway;Insulin signaling pathway

Polito et al. Universal ready-to-use immunotherapeutic approach for the treatment of cancer: expanded and activated polyclonal γδ memory T cells.
Phosphorylation sites

Glycerophospholipid metabolism;Phosphonate and phosphinate metabolism
Glycerophospholipid metabolism;Phosphonate and phosphinate metabolism
Glycerophospholipid metabolism;Phosphonate and phosphinate metabolism
Glycerophospholipid metabolism;Phosphonate and phosphinate metabolism
Glycerophospholipid metabolism;Phosphonate and phosphinate metabolism
Glycerophospholipid metabolism;Phosphonate and phosphinate metabolism
Cell cycle;Cell cycle - yeast;DNA replication;Meiosis - yeast
Cell cycle;Cell cycle - yeast;DNA replication;Meiosis - yeast
Axon guidance
Axon guidance
Basal transcription factors;Cell cycle;Nucleotide excision repair

Polito et al. Universal ready-to-use immunotherapeutic approach for the treatment of cancer: expanded and activated polyclonal γδ memory T cells.
Phosphorylation sites

Spliceosome
Spliceosome
Chemokine signaling pathway;Jak-STAT signaling pathway;ko05152;Measles
Chemokine signaling pathway;Jak-STAT signaling pathway;ko05152;Measles
RNA transport
RNA transport
RNA transport
Axon guidance;Chemokine signaling pathway;Cytokine-cytokine receptor interaction;Endocytosis;Intestinal immune network for IgA production;Leukocyte transendothelial migration
Axon guidance;Chemokine signaling pathway;Cytokine-cytokine receptor interaction;Endocytosis;Intestinal immune network for IgA production;Leukocyte transendothelial migration
Axon guidance;Chemokine signaling pathway;Cytokine-cytokine receptor interaction;Endocytosis;Intestinal immune network for IgA production;Leukocyte transendothelial migration
Axon guidance;Chemokine signaling pathway;Cytokine-cytokine receptor interaction;Endocytosis;Intestinal immune network for IgA production;Leukocyte transendothelial migration
Axon guidance;Chemokine signaling pathway;Cytokine-cytokine receptor interaction;Endocytosis;Intestinal immune network for IgA production;Leukocyte transendothelial migration
Insulin signaling pathway;mTOR signaling pathway;Ribosome
Insulin signaling pathway;mTOR signaling pathway;Ribosome
Spliceosome
Spliceosome
Spliceosome
Spliceosome
Spliceosome
Spliceosome
Spliceosome

Polito et al. Universal ready-to-use immunotherapeutic approach for the treatment of cancer: expanded and activated polyclonal γδ memory T cells.
Phosphorylation sites

Cell cycle;Non-homologous end-joining
Spliceosome
Spliceosome
Spliceosome
Spliceosome
Spliceosome
Spliceosome
Acute myeloid leukemia;Chronic myeloid leukemia;Pathways in cancer
Acute myeloid leukemia;Chronic myeloid leukemia;Pathways in cancer
Acute myeloid leukemia;Chronic myeloid leukemia;Pathways in cancer

Nucleotide excision repair
Nucleotide excision repair
Nucleotide excision repair
Nucleotide excision repair
Tight junction;Vascular smooth muscle contraction
Tight junction;Vascular smooth muscle contraction
Tight junction;Vascular smooth muscle contraction
Cell cycle;TGF-beta signaling pathway
Cell cycle;TGF-beta signaling pathway
Cell cycle;TGF-beta signaling pathway
Cell cycle;TGF-beta signaling pathway

Focal adhesion;Leukocyte transendothelial migration
Focal adhesion;Leukocyte transendothelial migration

Polito et al. Universal ready-to-use immunotherapeutic approach for the treatment of cancer: expanded and activated polyclonal γδ memory T cells.
Phosphorylation sites

Fatty acid biosynthesis;Insulin signaling pathway;Propanoate metabolism;Pyruvate metabolism
Fatty acid biosynthesis;Insulin signaling pathway;Propanoate metabolism;Pyruvate metabolism
Adipocytokine signaling pathway;Hypertrophic cardiomyopathy (HCM);Insulin signaling pathway;mTOR signaling pathway;Regulation of autophagy
Axon guidance;ErbB signaling pathway;Focal adhesion;MAPK signaling pathway;Regulation of actin cytoskeleton;Renal cell carcinoma;T cell receptor signaling pathway
Axon guidance;ErbB signaling pathway;Focal adhesion;MAPK signaling pathway;Regulation of actin cytoskeleton;Renal cell carcinoma;T cell receptor signaling pathway
ithelial cells;Chronic myeloid leukemia;Endocytosis;ErbB signaling pathway;Insulin signaling pathway;Jak-STAT signaling pathway;Pathways in cancer;T cell receptor signaling pathway;Ubiquiti
ithelial cells;Chronic myeloid leukemia;Endocytosis;ErbB signaling pathway;Insulin signaling pathway;Jak-STAT signaling pathway;Pathways in cancer;T cell receptor signaling pathway;Ubiquiti
Spliceosome
Spliceosome
Spliceosome
Spliceosome
Axon guidance;B cell receptor signaling pathway;Natural killer cell mediated cytotoxicity;T cell receptor signaling pathway;VEGF signaling pathway;Wnt signaling pathway
Axon guidance;B cell receptor signaling pathway;Natural killer cell mediated cytotoxicity;T cell receptor signaling pathway;VEGF signaling pathway;Wnt signaling pathway
Axon guidance;B cell receptor signaling pathway;Natural killer cell mediated cytotoxicity;T cell receptor signaling pathway;VEGF signaling pathway;Wnt signaling pathway
Axon guidance;B cell receptor signaling pathway;Natural killer cell mediated cytotoxicity;T cell receptor signaling pathway;VEGF signaling pathway;Wnt signaling pathway
Axon guidance;B cell receptor signaling pathway;Natural killer cell mediated cytotoxicity;T cell receptor signaling pathway;VEGF signaling pathway;Wnt signaling pathway
Axon guidance;B cell receptor signaling pathway;Natural killer cell mediated cytotoxicity;T cell receptor signaling pathway;VEGF signaling pathway;Wnt signaling pathway
Axon guidance;B cell receptor signaling pathway;Natural killer cell mediated cytotoxicity;T cell receptor signaling pathway;VEGF signaling pathway;Wnt signaling pathway
Axon guidance;B cell receptor signaling pathway;Natural killer cell mediated cytotoxicity;T cell receptor signaling pathway;VEGF signaling pathway;Wnt signaling pathway
Osteoclast differentiation

Polito et al. Universal ready-to-use immunotherapeutic approach for the treatment of cancer: expanded and activated polyclonal γδ memory T cells.
Phosphorylation sites

pathway;Gastric acid secretion;Glioma;GnRH signaling pathway;ko05152;Long-term potentiation;Melanogenesis;Neurotrophin signaling pathway;Olfactory transduction;Oocyte meiosis;Photo
pathway;Gastric acid secretion;Glioma;GnRH signaling pathway;ko05152;Long-term potentiation;Melanogenesis;Neurotrophin signaling pathway;Olfactory transduction;Oocyte meiosis;Phot
Notch signaling pathway;Spliceosome
Notch signaling pathway;Spliceosome
Spliceosome
Spliceosome
Spliceosome
Spliceosome

Endocytosis
Endocytosis
Endocytosis
Bacterial invasion of epithelial cells;Pathogenic Escherichia coli infection;Shigellosis;Tight junction
Meiosis - yeast;mRNA surveillance pathway;Oocyte meiosis;Wnt signaling pathway
Meiosis - yeast;mRNA surveillance pathway;Oocyte meiosis;Wnt signaling pathway
Jak-STAT signaling pathway
Jak-STAT signaling pathway
Jak-STAT signaling pathway
Jak-STAT signaling pathway

Polito et al. Universal ready-to-use immunotherapeutic approach for the treatment of cancer: expanded and activated polyclonal γδ memory T cells.
Phosphorylation sites

Endocytosis
Endocytosis
Pancreatic cancer;Pathways in cancer
Pancreatic cancer;Pathways in cancer
Insulin signaling pathway
Neuroactive ligand-receptor interaction
Ubiquitin mediated proteolysis
Cell adhesion molecules (CAMs)
Pathways in cancer;Thyroid cancer
Pathways in cancer;Thyroid cancer
Calcium signaling pathway;Long-term potentiation;Neurotrophin signaling pathway;Osteoclast differentiation
Calcium signaling pathway;Long-term potentiation;Neurotrophin signaling pathway;Osteoclast differentiation

Phosphorylation sites

Spliceosome
Spliceosome
Spliceosome
Spliceosome
Spliceosome
Spliceosome
Spliceosome
Spliceosome
Spliceosome
Spliceosome

Parkinson's disease
RNA transport
RNA transport

Polito et al. Universal ready-to-use immunotherapeutic approach for the treatment of cancer: expanded and activated polyclonal γδ memory T cells.

Phosphorylation sites

Spliceosome
Spliceosome
Spliceosome
Spliceosome
Spliceosome
Spliceosome
Spliceosome
Basal transcription factors
Basal transcription factors

Polito et al. Universal ready-to-use immunotherapeutic approach for the treatment of cancer: expanded and activated polyclonal γδ memory T cells.
Phosphorylation sites

Cell cycle - yeast
Cell cycle - yeast
RNA degradation
RNA degradation
RNA degradation
RNA degradation
RNA degradation

Polito et al. Universal ready-to-use immunotherapeutic approach for the treatment of cancer: expanded and activated polyclonal γδ memory T cells.
Phosphorylation sites

mRNA surveillance pathway
mRNA surveillance pathway
mRNA surveillance pathway
Endocytosis

Polito et al. Universal ready-to-use immunotherapeutic approach for the treatment of cancer: expanded and activated polyclonal γδ memory T cells.

Phosphorylation sites

Chemokine signaling pathway;MAPK signaling pathway
Chemokine signaling pathway;MAPK signaling pathway

Polito et al. Universal ready-to-use immunotherapeutic approach for the treatment of cancer: expanded and activated polyclonal γδ memory T cells.
Phosphorylation sites

Ubiquitin mediated proteolysis
RNA degradation
RNA degradation
Neuroactive ligand-receptor interaction
Neuroactive ligand-receptor interaction
Neuroactive ligand-receptor interaction

Neuroactive ligand-receptor interaction
Spliceosome
Spliceosome
Spliceosome
Spliceosome

Insulin signaling pathway;mTOR signaling pathway
Insulin signaling pathway;mTOR signaling pathway
Insulin signaling pathway;mTOR signaling pathway
Insulin signaling pathway;mTOR signaling pathway
Cell cycle;Cell cycle - yeast;Meiosis - yeast
Cell cycle;Cell cycle - yeast;Meiosis - yeast
Cell cycle;Cell cycle - yeast;Meiosis - yeast

Phosphorylation sites

Endocytosis
Endocytosis
Endocytosis
Endocytosis
Endocytosis
Endocytosis
Endocytosis
N-Glycan biosynthesis;Protein processing in endoplasmic reticulum;Various types of N-glycan biosynthesis
N-Glycan biosynthesis;Protein processing in endoplasmic reticulum;Various types of N-glycan biosynthesis

Polito et al. Universal ready-to-use immunotherapeutic approach for the treatment of cancer: expanded and activated polyclonal γδ memory T cells.
Phosphorylation sites

Purine metabolism;Pyrimidine metabolism;RNA degradation
Lysine degradation
Lysine degradation
Lysine degradation
RNA transport
RNA transport
Cell cycle;Cell cycle - yeast;Meiosis - yeast
Regulation of actin cytoskeleton
Regulation of actin cytoskeleton
Nucleotide excision repair;p53 signaling pathway;Ubiquitin mediated proteolysis
Nucleotide excision repair;p53 signaling pathway;Ubiquitin mediated proteolysis

Polito et al. Universal ready-to-use immunotherapeutic approach for the treatment of cancer: expanded and activated polyclonal γδ memory T cells.

Phosphorylation sites

Chemokine signaling pathway;Fc gamma R-mediated phagocytosis
Chemokine signaling pathway;Fc gamma R-mediated phagocytosis
RNA transport
RNA transport
mRNA surveillance pathway;Tight junction
B cell receptor signaling pathway;Fc epsilon RI signaling pathway;Fc gamma R-mediated phagocytosis;Insulin signaling pathway;Phosphatidylinositol signaling system
B cell receptor signaling pathway;Fc epsilon RI signaling pathway;Fc gamma R-mediated phagocytosis;Insulin signaling pathway;Phosphatidylinositol signaling system
RIG-I-like receptor signaling pathway
RIG-I-like receptor signaling pathway
mRNA surveillance pathway;RNA transport
mRNA surveillance pathway;RNA transport
Pathogenic Escherichia coli infection
Pathogenic Escherichia coli infection
Pathogenic Escherichia coli infection

Pathogenic Escherichia coli infection
Pathogenic Escherichia coli infection
Calcium signaling pathway;Insulin signaling pathway
Homologous recombination
Homologous recombination
RNA degradation
Leukocyte transendothelial migration
Leukocyte transendothelial migration
Leukocyte transendothelial migration
Leukocyte transendothelial migration

Protein processing in endoplasmic reticulum
Protein processing in endoplasmic reticulum

Polito et al. Universal ready-to-use immunotherapeutic approach for the treatment of cancer: expanded and activated polyclonal γδ memory T cells.
Phosphorylation sites

Amyotrophic lateral sclerosis (ALS)
Amyotrophic lateral sclerosis (ALS)
RNA transport
RNA transport
NOD-like receptor signaling pathway
NOD-like receptor signaling pathway

Polito et al. Universal ready-to-use immunotherapeutic approach for the treatment of cancer: expanded and activated polyclonal γδ memory T cells.
Phosphorylation sites

Endocytosis
Amyotrophic lateral sclerosis (ALS);MAPK signaling pathway;Neurotrophin signaling pathway;Protein processing in endoplasmic reticulum
Amyotrophic lateral sclerosis (ALS);MAPK signaling pathway;Neurotrophin signaling pathway;Protein processing in endoplasmic reticulum
GnRH signaling pathway;MAPK signaling pathway;Neurotrophin signaling pathway
GnRH signaling pathway;MAPK signaling pathway;Neurotrophin signaling pathway
GnRH signaling pathway;MAPK signaling pathway;Neurotrophin signaling pathway
GnRH signaling pathway;MAPK signaling pathway;Neurotrophin signaling pathway

Polito et al. Universal ready-to-use immunotherapeutic approach for the treatment of cancer: expanded and activated polyclonal γδ memory T cells.

Phosphorylation sites

Spliceosome
Inositol phosphate metabolism;Phosphatidylinositol signaling system
Inositol phosphate metabolism;Phosphatidylinositol signaling system
Ribosome biogenesis in eukaryotes
Ribosome biogenesis in eukaryotes

Polito et al. Universal ready-to-use immunotherapeutic approach for the treatment of cancer: expanded and activated polyclonal γδ memory T cells.
Phosphorylation sites

Lysine degradation
Lysine degradation
Lysine degradation
Lysine degradation
Lysine degradation
Lysine degradation
Ubiquitin mediated proteolysis

Polito et al. Universal ready-to-use immunotherapeutic approach for the treatment of cancer: expanded and activated polyclonal γδ memory T cells.
Phosphorylation sites

Ubiquitin mediated proteolysis
Ribosome biogenesis in eukaryotes;RNA degradation
Ribosome biogenesis in eukaryotes;RNA degradation
Cell cycle;Cell cycle - yeast;Meiosis - yeast;Oocyte meiosis;Progesterone-mediated oocyte maturation;Ubiquitin mediated proteolysis
Cell cycle;Cell cycle - yeast;Meiosis - yeast;Oocyte meiosis;Progesterone-mediated oocyte maturation;Ubiquitin mediated proteolysis
Oocyte meiosis
Oocyte meiosis
Oocyte meiosis
Oocyte meiosis
Protein processing in endoplasmic reticulum
Protein processing in endoplasmic reticulum
Protein processing in endoplasmic reticulum

Polito et al. Universal ready-to-use immunotherapeutic approach for the treatment of cancer: expanded and activated polyclonal γδ memory T cells.
Phosphorylation sites

Wnt signaling pathway
Wnt signaling pathway
Apoptosis;Cytokine-cytokine receptor interaction
Apoptosis;Cytokine-cytokine receptor interaction
RNA degradation
RNA degradation
RNA degradation
RNA degradation
RNA degradation
Osteoclast differentiation;RIG-I-like receptor signaling pathway
Osteoclast differentiation;RIG-I-like receptor signaling pathway
Osteoclast differentiation;RIG-I-like receptor signaling pathway
Focal adhesion;Leukocyte transendothelial migration;Regulation of actin cytoskeleton
Focal adhesion;Leukocyte transendothelial migration;Regulation of actin cytoskeleton
Focal adhesion;Leukocyte transendothelial migration;Regulation of actin cytoskeleton

Cell cycle - yeast
Cell cycle - yeast
p53 signaling pathway
p53 signaling pathway

Polito et al. Universal ready-to-use immunotherapeutic approach for the treatment of cancer: expanded and activated polyclonal γδ memory T cells.

Phosphorylation sites

Tight junction;Vibrio cholerae infection
Tight junction;Vibrio cholerae infection
Amyotrophic lateral sclerosis (ALS);MAPK signaling pathway
Amyotrophic lateral sclerosis (ALS);MAPK signaling pathway
Amyotrophic lateral sclerosis (ALS);MAPK signaling pathway
Amyotrophic lateral sclerosis (ALS);MAPK signaling pathway

Cell cycle;Cell cycle - yeast;Meiosis - yeast;Oocyte meiosis;Progesterone-mediated oocyte maturation;Ubiquitin mediated proteolysis

Base excision repair

Protein processing in endoplasmic reticulum;Ubiquitin mediated proteolysis
Protein processing in endoplasmic reticulum;Ubiquitin mediated proteolysis

Phosphorylation sites

Polito et al. Universal ready-to-use immunotherapeutic approach for the treatment of cancer: expanded and activated polyclonal γδ memory T cells.
Phosphorylation sites

Glycerophospholipid metabolism
Glycerophospholipid metabolism
Calcium signaling pathway;Neuroactive ligand-receptor interaction
Ribosome biogenesis in eukaryotes
Ribosome biogenesis in eukaryotes
Endocytosis;Epithelial cell signaling in Helicobacter pylori infection;Regulation of actin cytoskeleton
Endocytosis;Epithelial cell signaling in Helicobacter pylori infection;Regulation of actin cytoskeleton

Polito et al. Universal ready-to-use immunotherapeutic approach for the treatment of cancer: expanded and activated polyclonal γδ memory T cells.

Phosphorylation sites

Cell adhesion molecules (CAMs);Epithelial cell signaling in Helicobacter pylori infection;Leukocyte transendothelial migration;Tight junction
Cell adhesion molecules (CAMs);Epithelial cell signaling in Helicobacter pylori infection;Leukocyte transendothelial migration;Tight junction
Phagosome;Vasopressin-regulated water reabsorption
Phagosome;Vasopressin-regulated water reabsorption

Polito et al. Universal ready-to-use immunotherapeutic approach for the treatment of cancer: expanded and activated polyclonal γδ memory T cells.
Phosphorylation sites

Nucleotide excision repair
Nucleotide excision repair
Nucleotide excision repair
Nucleotide excision repair
Nucleotide excision repair
Nucleotide excision repair
onic myeloid leukemia;Colorectal cancer;Endometrial cancer;ErbB signaling pathway;Jak-STAT signaling pathway;MAPK signaling pathway;Pathways in cancer;Small cell lung cancer;TGF-beta sig
Proteasome
Cell adhesion molecules (CAMs);Staphylococcus aureus infection
Cell adhesion molecules (CAMs);Staphylococcus aureus infection

RNA transport
RNA transport
mRNA surveillance pathway;RNA transport
mRNA surveillance pathway;RNA transport
mRNA surveillance pathway;RNA transport
MAPK signaling pathway
MAPK signaling pathway
mRNA surveillance pathway;RNA transport;Spliceosome
Ribosome biogenesis in eukaryotes
Spliceosome
RNA transport;Viral myocarditis
Gap junction;Pathogenic Escherichia coli infection;Phagosome
Base excision repair
Cell cycle;Measles;Oocyte meiosis;p53 signaling pathway;Pathways in cancer;Progesterone-mediated oocyte maturation;Prostate cancer;Small cell lung cancer

Polito et al. Universal ready-to-use immunotherapeutic approach for the treatment of cancer: expanded and activated polyclonal γδ memory T cells.
Phosphorylation sites

Focal adhesion;Leukocyte transendothelial migration;Regulation of actin cytoskeleton;Tight junction;Vascular smooth muscle contraction
Spliceosome
Spliceosome
Spliceosome
Spliceosome
Spliceosome
Spliceosome
Spliceosome
Spliceosome
RNA transport
Protein export
Hematopoietic cell lineage
Bladder cancer;Cell cycle;Chronic myeloid leukemia;Glioma;Melanoma;Non-small cell lung cancer;Pancreatic cancer;Pathways in cancer;Prostate cancer;Small cell lung cancer
Cell cycle;Gap junction;Oocyte meiosis;p53 signaling pathway;Progesterone-mediated oocyte maturation
Gap junction;Pathogenic Escherichia coli infection;Phagosome
Apoptosis;Insulin signaling pathway
Dorso-ventral axis formation;MAPK signaling pathway - fly

Polito et al. Universal ready-to-use immunotherapeutic approach for the treatment of cancer: expanded and activated polyclonal γδ memory T cells.
Phosphorylation sites

Osteoclast differentiation
Base excision repair;DNA replication;Mismatch repair;Nucleotide excision repair
RNA transport
Huntington's disease;Purine metabolism;Pyrimidine metabolism;RNA polymerase
Cell cycle;Cell cycle - yeast;DNA replication;Meiosis - yeast
cal adhesion;Gap junction;Glioma;GnRH signaling pathway;Hepatitis C;Insulin signaling pathway;ko05152;Leishmaniasis;Long-term depression;Long-term potentiation;MAPK signaling pathway
Chemokine signaling pathway;Endocytosis
Basal transcription factors
RNA transport
Dorso-ventral axis formation;MAPK signaling pathway - fly
Chemokine signaling pathway;Endocytosis
Proteasome
Basal transcription factors;Cell cycle;Nucleotide excision repair
Axon guidance;Chemokine signaling pathway;Cytokine-cytokine receptor interaction;Endocytosis;Intestinal immune network for IgA production;Leukocyte transendothelial migration
Spliceosome

Polito et al. Universal ready-to-use immunotherapeutic approach for the treatment of cancer: expanded and activated polyclonal γδ memory T cells.
Phosphorylation sites

Acute myeloid leukemia;Chronic myeloid leukemia;Pathways in cancer
Cell cycle;TGF-beta signaling pathway
Cell cycle;TGF-beta signaling pathway
Cell cycle;TGF-beta signaling pathway
Adipocytokine signaling pathway;Hypertrophic cardiomyopathy (HCM);Insulin signaling pathway;mTOR signaling pathway;Regulation of autophagy
Insulin signaling pathway;Progesterone-mediated oocyte maturation;Purine metabolism
Insulin signaling pathway;Progesterone-mediated oocyte maturation;Purine metabolism
Osteoclast differentiation
Acute myeloid leukemia;ErbB signaling pathway;Insulin signaling pathway;mTOR signaling pathway;RNA transport
Acute myeloid leukemia;ErbB signaling pathway;Insulin signaling pathway;mTOR signaling pathway;RNA transport
Spliceosome
Bacterial invasion of epithelial cells;Pathogenic Escherichia coli infection;Shigellosis;Tight junction
Cell adhesion molecules (CAMs)
ishmaniasis;Leukocyte transendothelial migration;MAPK signaling pathway;MAPK signaling pathway - yeast;Neurotrophin signaling pathway;NOD-like receptor signaling pathway;Osteoclast di

RNA degradation

mRNA surveillance pathway

mRNA surveillance pathway;Tight junction
B cell receptor signaling pathway;Fc epsilon RI signaling pathway;Fc gamma R-mediated phagocytosis;Insulin signaling pathway;Phosphatidylinositol signaling system
Spliceosome
Spliceosome
Lysosome
Lysosome

Cell cycle;Cell cycle - yeast;Meiosis - yeast;Oocyte meiosis;Progesterone-mediated oocyte maturation;Ubiquitin mediated proteolysis
Base excision repair

Polito et al. Universal ready-to-use immunotherapeutic approach for the treatment of cancer: expanded and activated polyclonal γδ memory T cells.
Phosphorylation sites

Phagosome;Vasopressin-regulated water reabsorption
Cell cycle;Measles;Oocyte meiosis;p53 signaling pathway;Pathways in cancer;Progesterone-mediated oocyte maturation;Prostate cancer;Small cell lung cancer
Cell cycle;Gap junction;Oocyte meiosis;p53 signaling pathway;Progesterone-mediated oocyte maturation
Dorso-ventral axis formation;MAPK signaling pathway - fly
cal adhesion;Gap junction;Glioma;GnRH signaling pathway;Hepatitis C;Insulin signaling pathway;ko05152;Leishmaniasis;Long-term depression;Long-term potentiation;MAPK signaling pathway
Spliceosome
ishmaniasis;Leukocyte transendothelial migration;MAPK signaling pathway;MAPK signaling pathway - yeast;Neurotrophin signaling pathway;NOD-like receptor signaling pathway;Osteoclast di
RNA transport
RNA transport
RNA transport
RNA transport
Alzheimer's disease;MAPK signaling pathway
Alzheimer's disease;MAPK signaling pathway
Alzheimer's disease;MAPK signaling pathway

RNA transport
RNA transport
mRNA surveillance pathway;RNA transport
mRNA surveillance pathway;RNA transport
mRNA surveillance pathway;RNA transport
mRNA surveillance pathway;RNA transport
mRNA surveillance pathway;RNA transport
mRNA surveillance pathway;RNA transport
mRNA surveillance pathway;RNA transport
mRNA surveillance pathway;RNA transport

Spliceosome
Spliceosome
Spliceosome
Spliceosome
Spliceosome
Spliceosome
MAPK signaling pathway
MAPK signaling pathway
MAPK signaling pathway
Ribosome biogenesis in eukaryotes
Ribosome biogenesis in eukaryotes
Ribosome biogenesis in eukaryotes
Ribosome biogenesis in eukaryotes
Ribosome biogenesis in eukaryotes
Ribosome biogenesis in eukaryotes
Ribosome biogenesis in eukaryotes
Ribosome biogenesis in eukaryotes
Ribosome biogenesis in eukaryotes
Spliceosome

Spliceosome
Spliceosome
Spliceosome
Spliceosome
Spliceosome
Spliceosome
Spliceosome
Spliceosome
Spliceosome
Spliceosome
Spliceosome
Axon guidance;B cell receptor signaling pathway;Natural killer cell mediated cytotoxicity;T cell receptor signaling pathway;VEGF signaling pathway;Wnt signaling pathway
Axon guidance;B cell receptor signaling pathway;Natural killer cell mediated cytotoxicity;T cell receptor signaling pathway;VEGF signaling pathway;Wnt signaling pathway
Neuroactive ligand-receptor interaction
Neuroactive ligand-receptor interaction
Neuroactive ligand-receptor interaction
Bile secretion;Gastric acid secretion;Pancreatic secretion;Salivary secretion
Bile secretion;Gastric acid secretion;Pancreatic secretion;Salivary secretion
Bile secretion;Gastric acid secretion;Pancreatic secretion;Salivary secretion
oma;Leukocyte transendothelial migration;Long-term depression;Long-term potentiation;MAPK signaling pathway;MAPK signaling pathway - yeast;Melanogenesis;Natural killer cell mediated c
oma;Leukocyte transendothelial migration;Long-term depression;Long-term potentiation;MAPK signaling pathway;MAPK signaling pathway - yeast;Melanogenesis;Natural killer cell mediated c
oma;Leukocyte transendothelial migration;Long-term depression;Long-term potentiation;MAPK signaling pathway;MAPK signaling pathway - yeast;Melanogenesis;Natural killer cell mediated c
Insulin signaling pathway;Starch and sucrose metabolism
Insulin signaling pathway;Starch and sucrose metabolism
Insulin signaling pathway;Starch and sucrose metabolism
Osteoclast differentiation
Osteoclast differentiation

Polito et al. Universal ready-to-use immunotherapeutic approach for the treatment of cancer: expanded and activated polyclonal γδ memory T cells.
Phosphorylation sites

Parkinson's disease;Ubiquitin mediated proteolysis
Bile secretion;PPAR signaling pathway;Primary bile acid biosynthesis;Steroid hormone biosynthesis
Huntington's disease;Purine metabolism;Pyrimidine metabolism;RNA polymerase
Huntington's disease;Purine metabolism;Pyrimidine metabolism;RNA polymerase
Huntington's disease;Purine metabolism;Pyrimidine metabolism;RNA polymerase
Acute myeloid leukemia;Endocytosis;Pathways in cancer;Ubiquitin mediated proteolysis
Acute myeloid leukemia;Endocytosis;Pathways in cancer;Ubiquitin mediated proteolysis
Acute myeloid leukemia;Endocytosis;Pathways in cancer;Ubiquitin mediated proteolysis
Acute myeloid leukemia;Endocytosis;Pathways in cancer;Ubiquitin mediated proteolysis
Acute myeloid leukemia;Endocytosis;Pathways in cancer;Ubiquitin mediated proteolysis
Acute myeloid leukemia;Endocytosis;Pathways in cancer;Ubiquitin mediated proteolysis
Glycerophospholipid metabolism;Phosphonate and phosphinate metabolism
Glycerophospholipid metabolism;Phosphonate and phosphinate metabolism
Glycerophospholipid metabolism;Phosphonate and phosphinate metabolism

Polito et al. Universal ready-to-use immunotherapeutic approach for the treatment of cancer: expanded and activated polyclonal γδ memory T cells.
Phosphorylation sites

Axon guidance;Chemokine signaling pathway;Cytokine-cytokine receptor interaction;Endocytosis;Intestinal immune network for IgA production;Leukocyte transendothelial migration
Axon guidance;Chemokine signaling pathway;Cytokine-cytokine receptor interaction;Endocytosis;Intestinal immune network for IgA production;Leukocyte transendothelial migration
Axon guidance;Chemokine signaling pathway;Cytokine-cytokine receptor interaction;Endocytosis;Intestinal immune network for IgA production;Leukocyte transendothelial migration
Insulin signaling pathway;mTOR signaling pathway;Ribosome
Insulin signaling pathway;mTOR signaling pathway;Ribosome
Insulin signaling pathway;mTOR signaling pathway;Ribosome
Insulin signaling pathway;mTOR signaling pathway;Ribosome
Insulin signaling pathway;mTOR signaling pathway;Ribosome
Spliceosome
Spliceosome
Spliceosome
Spliceosome
Spliceosome
Spliceosome
Spliceosome
Spliceosome
Cell cycle;TGF-beta signaling pathway
Cell cycle;TGF-beta signaling pathway

pithelial cells;Chronic myeloid leukemia;Endocytosis;ErbB signaling pathway;Insulin signaling pathway;Jak-STAT signaling pathway;Pathways in cancer;T cell receptor signaling pathway;Ubiquiti
pithelial cells;Chronic myeloid leukemia;Endocytosis;ErbB signaling pathway;Insulin signaling pathway;Jak-STAT signaling pathway;Pathways in cancer;T cell receptor signaling pathway;Ubiquiti
pithelial cells;Chronic myeloid leukemia;Endocytosis;ErbB signaling pathway;Insulin signaling pathway;Jak-STAT signaling pathway;Pathways in cancer;T cell receptor signaling pathway;Ubiquiti
Spliceosome
Spliceosome
Spliceosome
Spliceosome
Spliceosome
Bacterial invasion of epithelial cells;Pathogenic Escherichia coli infection;Shigellosis;Tight junction
Jak-STAT signaling pathway
Jak-STAT signaling pathway
Jak-STAT signaling pathway
Meiosis - yeast;mRNA surveillance pathway;Oocyte meiosis;Wnt signaling pathway
Meiosis - yeast;mRNA surveillance pathway;Oocyte meiosis;Wnt signaling pathway
Meiosis - yeast;mRNA surveillance pathway;Oocyte meiosis;Wnt signaling pathway
Spliceosome

Polito et al. Universal ready-to-use immunotherapeutic approach for the treatment of cancer: expanded and activated polyclonal γδ memory T cells.
Phosphorylation sites

Spliceosome
Spliceosome
Spliceosome
Spliceosome
Spliceosome
Endocytosis
Endocytosis
Endocytosis

Polito et al. Universal ready-to-use immunotherapeutic approach for the treatment of cancer: expanded and activated polyclonal γδ memory T cells.
Phosphorylation sites

Ubiquitin mediated proteolysis
RNA degradation
RNA degradation
Spliceosome
Spliceosome
Spliceosome
Spliceosome
Spliceosome
Spliceosome

Phosphorylation sites

Polito et al. Universal ready-to-use immunotherapeutic approach for the treatment of cancer: expanded and activated polyclonal γδ memory T cells.
Phosphorylation sites

RNA degradation
RNA degradation
Tight junction;Vibrio cholerae infection
Tight junction;Vibrio cholerae infection
Tight junction;Vibrio cholerae infection

Polito et al. Universal ready-to-use immunotherapeutic approach for the treatment of cancer: expanded and activated polyclonal γδ memory T cells.
Phosphorylation sites

Alzheimer's disease;MAPK signaling pathway
RNA transport

Polito et al. Universal ready-to-use immunotherapeutic approach for the treatment of cancer: expanded and activated polyclonal γδ memory T cells.
Phosphorylation sites

Osteoclast differentiation
Cell cycle;TGF-beta signaling pathway
Spliceosome
Bacterial invasion of epithelial cells;Pathogenic Escherichia coli infection;Shigellosis;Tight junction
Ubiquitin mediated proteolysis
Ubiquitin mediated proteolysis
Spliceosome

MAPK signaling pathway

Polito et al. Universal ready-to-use immunotherapeutic approach for the treatment of cancer: expanded and activated polyclonal γδ memory T cells.

Phosphorylation sites

N: Localization prob	N: PEP	N: Score	N: Delta score	N: Score for localization	N: Mass error [ppm]	N: Intensity	N: Position	N: Score diff
1	2,80E-20	219,22	187,04	170,5	0,17263	299860000	12	122,09
1	1,25E-08	195,1	181,51	170,5	0,17263	96750000	14	75,6136
1	9,99E-29	251,02	220,89	251,02	0,37992	72042000	31	82,5235
1	0,000282451	114,29	55,805	114,29	0,13501	40439000	153	76,2637
0,904285	7,90E-06	133,14	107,01	133,14	0,23248	21125000	516	9,75325
1	0,000835089	171,46	136,53	140,14	0,064852	258100000	125	80,1202
0,99994	1,04E-67	237,98	211,85	237,98	-0,18997	217900000	113	44,0888
1	6,92E-09	129,46	106,49	56,728	0,094583	690400000	958	56,728
1	3,32E-11	160,55	135,99	149,57	0,0047215	458360000	967	108,721
1	2,95E-09	161,22	107,1	161,22	-0,19002	93394000	1111	83,8975
0,995747	0,000303545	112,51	93,775	87,498	0,50812	38104000	729	23,7383
0,999065	5,09E-06	149,59	123,54	149,59	-1,579	34755000	426	31,3964
0,99721	1,20E-11	209,53	164,02	209,53	-0,0028273	70384000	358	25,5315
0,956907	8,58E-08	149,47	125,8	128,14	2,5085	38452000	370	16,4811
0,997644	0,000614479	101,65	91,659	101,65	0,0035697	3251900	205	26,268
1	0,000116531	111,43	90,48	111,43	0,26636	28101000	189	111,433
0,996001	1,65E-06	183,96	158,31	60,518	0,12088	140590000	158	24,1233
0,881485	0,00305131	83,647	61,155	81,915	0,41705	29262000	114	10,2073
0,997152	2,51E-15	197	176,8	119,4	1,8043	84479000	421	25,4425
1	3,43E-76	291,15	276,57	291,15	-0,27458	502100000	221	104,186
0,798231	1,26E-05	93,876	80,947	93,876	-0,014339	10751000	62	5,99527
0,979315	2,54E-06	169,04	141,36	138,21	-0,14577	124800000	24	16,7654
0,997518	0,000417519	117,14	61,695	117,14	-0,10488	9112700	567	26,0404
0,999876	0,000316748	123,95	97,906	123,95	0,22627	27696000	76	39,1278
0,999634	5,18E-95	340,52	273,44	315,05	0,058032	185650000	151	34,3598
0,989489	9,00E-07	141	118,47	141	-0,49836	13917000	640	19,7376
0,999992	1,65E-11	203,74	126,87	144,73	-0,24974	681230000	188	54,1692
1	0,00029744	95,067	61,067	95,067	0,25895	6422500	592	95,0671
1	8,97E-06	84,404	64,649	67,31	-0,2122	29852000	381	67,3099
0,862264	5,89E-22	145,86	125,41	141,07	-0,0076131	315040000	459	12,8184
0,990158	0,000216731	131,69	69,225	121,56	0,45993	12186000	220	20,0261
0,830734	0,000217509	129,37	70,692	129,37	-0,52257	9558000	537	6,90891
1	7,80E-25	203,79	178,63	203,79	-0,084919	439120000	550	182,975

Polito et al. Universal ready-to-use immunotherapeutic approach for the treatment of cancer: expanded and activated polyclonal γδ memory T cells.
Phosphorylation sites

0,999788	0,00208727	69,261	51,825	69,261	-0,16234	13320000	46	36,7382
0,941887	3,01E-08	166,92	134,3	166,92	0,41852	56655000	1255	12,2291
0,985935	3,84E-06	105,54	71,991	83,088	0,15922	68466000	137	21,976
1	0,00112489	89,133	57,847	48,288	-0,47445	56347000	1168	48,2881
1	3,82E-95	328,64	281,69	236,26	0,26292	410230000	107	201,302
1	5,73E-182	411,08	373,09	277,79	0,10636	2385300000	113	147,531
1	7,18E-176	426,29	375,82	336,73	-0,22118	2152300000	164	190,808
1	1,19E-105	325,11	295,39	275,5	-0,026695	6561900000	214	149,767
1	1,95E-53	272,13	256,94	263,08	-0,35186	1743900000	137	94,6642
0,999036	0,00416783	72,325	53,054	51,013	0,24371	6928800	934	30,1561
0,841244	0,00261727	51,978	29,418	51,978	0,18175	8530400	222	12,075
1	1,66E-12	160,92	147,6	160,92	-0,083673	17244000	220	89,2199
0,999981	2,25E-15	182,15	161,77	159,36	-0,28714	749390000	867	50,168
0,999972	1,47E-41	245,82	215,72	164,39	-0,47312	995930000	872	45,5429
0,999984	0,000392082	137,95	56,003	137,95	0,076715	8074600	357	48,0675
0,881393	4,72E-11	166,28	144,05	166,28	-0,15516	12256000	301	11,7209
1	2,11E-19	212,9	174,13	212,9	-1,0077	33787000	74	65,2639
0,997591	7,22E-10	156,47	148,93	156,47	0,017106	201100000	258	26,172
0,98819	2,48E-09	119,97	97,996	119,97	0,15045	378400000	382	19,2262
0,92084	0,0272743	50,155	28,605	50,155	0,33187	3248600	1204	13,1424
0,999998	1,11E-05	159,31	140,07	159,31	0,24445	156060000	498	57,957
0,961004	0,000115432	138,99	90,488	71,219	-0,12975	149860000	75	14,2814
1	5,82E-05	134,49	116,22	65,179	-0,81362	70547000	166	65,1792
0,999791	8,01E-05	150,14	77,853	136,99	-0,019873	108780000	28	36,8005
1	0,00275922	92,943	67,506	53,828	0,12701	171550000	578	53,8285
1	7,90E-05	120,9	99,339	111,77	0,18462	153850000	65	64,8772
1	2,59E-06	183,23	149,44	42,257	0,2421	1227500000	612	42,2569
0,842323	3,76E-08	208,74	166,41	188,96	0,11036	199150000	84	7,27986
1	1,11E-05	150,22	92,869	99,344	0,061895	359450000	656	99,344
1	0,0140308	90,601	7,9296	86,67	-0,31926	286730000	664	86,6702
1	8,86E-06	176,18	142,32	153,74	-0,10101	1995700000	250	69,1337
1	2,87E-22	252,02	209,54	207,52	0,17431	320200000	697	74,3761
1	1,38E-05	155,66	115,17	52,071	-0,0066568	75579000	33	52,0715
0,999413	1,33E-05	155,09	130,24	155,09	0,23992	67772000	2	32,3096

Polito et al. Universal ready-to-use immunotherapeutic approach for the treatment of cancer: expanded and activated polyclonal γδ memory T cells.
Phosphorylation sites

1	3,76E-110	319,96	290,49	218,63	0,093162	425040000	560	215,615
0,975347	8,24E-05	86,357	75,096	86,357	-0,33923	10785000	415	17,2095
0,972457	1,06E-05	113,6	89,587	113,6	-0,017036	40666000	569	16,1563
0,999992	9,81E-06	174,89	135,31	163,33	-0,18762	169900000	115	51,2484
1	1,28E-43	219,16	200,43	202,43	-0,082184	77662000	509	68,5822
0,910728	1,83E-06	132,65	94,761	89,873	-0,38864	71832000	142	10,0902
1	8,93E-06	117,23	106,18	52,483	0,3187	101030000	40	52,4826
0,996856	2,34E-10	188,63	157,67	188,63	-0,21623	180860000	479	25,0109
0,878114	4,51E-05	115,17	103,02	115,17	0,8296	9339400	490	8,59664
0,99228	9,86E-05	145,81	122,93	145,81	0,20189	21254000	203	21,3299
0,999542	8,40E-42	254,58	239,61	162,81	0,56045	112180000	200	33,3893
1	2,98E-81	320,5	251,47	320,5	-0,19215	5842100000	14	112,643
1	7,22E-05	151,26	110,76	151,26	-0,0826	981880000	617	90,0217
1	0,000220617	130,01	61,99	130,01	0,15576	131270000	567	65,2763
1	2,94E-61	282,33	260,28	282,33	0,080876	367720000	299	76,6817
1	0,000360474	125,36	125,36	46,069	-0,06577	833070000	351	46,0691
0,999416	3,40E-05	162,93	118,63	136,93	-0,35344	457610000	134	32,3395
0,999803	7,62E-34	232,84	196,36	232,84	0,574	38917000	119	37,0555
0,988916	0,000267679	117,2	68,635	117,2	0,19838	23709000	150	19,5137
0,999979	1,32E-09	132,34	116,58	132,34	-0,073991	89119000	1124	47,9336
1	0,0140308	90,601	22,377	79,893	0,1004	44263000	9	79,8925
0,999999	1,37E-06	167,93	110,23	142	-0,17403	464080000	1103	62,0095
0,98041	5,10E-19	185,16	160,26	178,03	0,10924	54647000	238	16,9939
0,777021	1,76E-06	146,15	129,12	146,15	-0,14347	46964000	1206	5,82897
0,864463	3,07E-06	141,6	116,45	117,4	0,23986	63356000	1207	8,04817
0,984545	3,69E-55	238,63	207,87	209,67	0,11551	125350000	139	19,0464
0,999911	0,00718348	66,262	39,59	61,194	-0,16966	10622000	8	40,4941
0,999999	0,00690549	76,827	8,8341	76,827	0,33264	48963000	212	62,192
0,944287	8,16E-10	150,62	140,02	124,82	1,6062	58970000	621	12,4291
0,999998	0,000186029	119,69	73,81	80,102	-0,027614	77982000	127	58,4326
1	3,12E-26	202,03	173,07	174,4	0,29505	294640000	258	118,932
1	2,46E-05	160,38	117,54	160,38	-0,057456	44679000	73	68,5125
0,970581	0,00282613	86,67	24,708	65,224	0,099074	2745300	466	15,1841
0,998417	0,0247836	45,74	23,354	42,864	-0,11615	4943700	491	27,9994

0,999999	2,57E-63	226,98	209,97	213,3	-0,39819	3181400000	389	60,9493
0,999181	3,71E-47	225,92	211,33	219,89	0,12978	2276900000	490	31,7453
0,77351	3,83E-21	189,37	162,16	99,569	0,14612	876050000	494	5,3342
1	5,00E-14	175,23	157,89	175,23	0,08318	289240000	498	111,234
0,975895	3,32E-135	346,25	299,59	110,98	0,22697	945820000	468	17,0256
0,998942	2,92E-136	353,27	309,48	278,75	-0,13332	2776800000	476	31,7243
1	0,013975	90,761	10,952	90,761	-0,012418	536260000	438	90,7612
1	2,02E-07	207,83	134,6	126,12	0,17219	689080000	291	126,118
1	0,000352446	126,12	66,965	126,12	0,17219	67744000	295	126,118
0,98408	1,05E-60	274,43	230,47	178,88	0,14788	988160000	305	17,9132
0,99991	1,05E-60	274,43	230,47	258,9	0,16029	926820000	309	42,542
0,956177	8,69E-75	300,25	264,57	258,16	0,24768	837170000	312	13,428
0,904868	0,0353645	43,592	31,95	43,592	-0,11599	29740000	443	9,7963
1	0,000680732	116,19	65,238	103,76	-0,35911	101250000	160	103,76
0,999943	0,000585701	103,91	61,192	103,91	0,13471	19153000	266	43,6543
1	0,0107087	62,709	50,178	54,103	-0,63383	34347000	10	54,1033
0,840343	1,00E-11	116,34	100,41	71,528	2,0784	75815000	664	7,21269
0,99236	0,000352863	101,36	77,608	101,36	0,39495	5005800	85	25,3475
0,987335	4,47E-05	99,044	80,234	99,044	-1,8386	9536000	1163	20,0941
1	3,79E-17	175,75	155,1	175,75	-0,50877	95469000	203	175,748
0,979824	5,83E-07	188,9	134,41	181,97	0,17211	100620000	594	17,3164
0,990865	0,000293833	160,86	96,436	129,74	-0,062055	159120000	357	20,3531
0,995997	4,79E-49	223,71	204,68	223,71	-0,070542	160630000	632	23,9598
0,998804	0,000292583	102,87	74,108	77,062	-0,059139	35312000	496	29,2185
0,988336	1,97E-10	190,8	166,3	190,8	0,23806	51767000	465	19,2807
1	6,82E-49	258,34	242,2	258,34	-0,36532	175600000	450	74,129
1	2,48E-14	173,09	158,84	168,78	-0,35018	50095000	456	64,5965
1	2,50E-06	131,82	108,6	56,339	-0,18934	167590000	249	56,3391
1	1,26E-05	122,94	104,44	42,568	-0,65133	238820000	158	42,5683
1	1,91E-49	235,16	215,39	196,75	-0,27594	564980000	620	85,5504
0,999999	2,16E-06	140,84	119,01	137,19	-0,14952	15929000	498	59,1225
1	2,03E-59	287,54	259,69	41,513	-0,10527	664760000	333	41,5131
1	0	531,41	467,11	531,41	0,27301	398880000	539	172,843
0,998999	4,60E-14	185,33	141,13	178,51	0,9033	27226000	478	29,9912

Polito et al. Universal ready-to-use immunotherapeutic approach for the treatment of cancer: expanded and activated polyclonal γδ memory T cells.
Phosphorylation sites

1	8,40E-141	367,71	320,17	336,3	0,29977	65779000	352	105,092
0,97347	0,000435782	77,554	54,086	77,554	-0,19241	14445000	356	15,684
0,937662	4,43E-21	161,44	149,46	161,44	0,29156	35775000	996	11,773
0,999835	1,93E-21	190,71	135,38	95,205	-0,80461	28494000	1179	37,8388
0,999858	3,24E-51	258,87	203,89	95,205	-0,80461	41332000	1180	38,4865
0,928843	0,000147304	150,01	104,73	150,01	-0,11257	75486000	288	11,2023
1	1,40E-05	170,66	99,417	170,66	0,17304	18894000	802	87,8073
0,999944	4,36E-05	91,128	76,122	81,799	0,11974	17505000	335	42,506
1	0,000718056	117,3	76,447	117,3	0,26312	230860000	285	67,0027
1	0,000109854	137,69	76,523	137,69	-0,15361	156660000	547	64,8716
0,999386	8,64E-06	161,41	133,23	145,81	-1,3564	218810000	294	32,1208
0,973751	0,000303091	124,67	86,101	124,67	0,062889	15926000	501	18,0501
0,926297	2,71E-07	88,087	78,29	88,087	0,43283	26995000	398	11,0609
0,999997	2,90E-141	333,87	317,95	333,87	0,072384	675700000	17	54,9008
0,998426	2,40E-12	172,37	137,33	172,37	-0,030953	65783000	95	28,0657
1	2,19E-17	232,01	181,11	161,14	-0,82254	24889000	862	161,135
1	4,29E-54	282,19	249,86	237,3	-0,1345	486010000	801	79,7576
0,99542	7,06E-05	152,4	125,69	131,06	0,015917	350170000	783	23,3718
1	6,29E-05	165,78	120,12	165,78	0,053252	402820000	788	68,0061
1	0,000436786	94,728	75,333	87,932	-0,2742	4769800	61	87,9322
1	7,02E-117	289,18	274,02	233,44	-0,89598	2630300000	94	173,547
0,999989	0,0169321	84,498	45,2	79,713	0,15817	245940000	510	49,6426
0,999872	1,33E-08	180,74	156,32	180,74	0,16214	9228300	369	38,9196
0,999711	7,24E-61	276,03	233,51	276,03	0,74492	73680000	1201	36,2841
1	0,0242997	63,076	36,403	48,346	0,15378	22001000	813	48,3458
0,991752	0,000276552	136,17	97,979	71,344	0,13528	27102000	1039	21,2421
0,808415	0,000224494	68,361	57,05	68,361	1,7193	18990000	15	6,26037
1	3,82E-70	317,26	299,52	130,72	-0,16912	41005000	492	119,603
1	2,56E-116	330,43	313,19	330,43	0,23598	120790000	1034	91,7112
1	3,65E-60	276,4	252,41	276,4	-0,19279	328600000	710	104,695
0,999992	2,47E-09	182,57	151,57	182,57	-0,35829	313610000	832	51,0378
0,999941	0,000352863	101,36	65,073	74,141	0,38728	14788000	1020	44,0688
0,76694	0,000129686	122,08	93,316	90,397	-0,039485	43725000	534	5,17295
0,999936	5,33E-70	282,17	242,3	191,79	1,4767	35619000	446	41,9546

Polito et al. Universal ready-to-use immunotherapeutic approach for the treatment of cancer: expanded and activated polyclonal γδ memory T cells.
Phosphorylation sites

0,865605	2,38E-24	236,12	178,95	236,12	0,11561	13766000	788	11,7829
1	7,86E-27	210,66	197,07	95,153	-0,1723	272120000	103	95,1532
1	3,00E-05	164,13	129,43	63,408	0,062348	276210000	646	63,4082
0,997922	2,70E-17	223,51	176,42	139,98	0,042951	670700000	527	26,8149
0,999946	3,61E-09	200,19	163,04	173,44	0,87184	570040000	392	45,7293
0,999896	0,000501469	110,53	78,119	101,38	-0,26956	223020000	595	39,8209
0,999997	1,45E-27	244,67	190,32	214,28	0,94908	1109000000	371	55,2371
0,99996	7,41E-08	163,68	163,68	163,68	-0,17954	23129000	229	44,0596
0,998086	0,000245198	103,88	80,649	103,88	-0,27299	15326000	290	27,5175
0,999991	0,000488813	111,53	92,052	111,53	0,10257	134340000	217	50,3643
1	3,09E-07	203,61	163,99	203,61	-0,029153	123050000	220	77,8917
1	0,000146519	146,71	107,13	146,71	0,11255	223120000	226	64,7024
0,999798	2,74E-06	148,44	120,14	135,08	0,088544	387150000	178	38,3033
0,999695	0,000281389	126,09	74,8	107,46	0,083228	106780000	132	35,6589
0,838702	1,02E-29	200,59	178,35	200,59	0,088122	278920000	137	7,35931
0,976605	7,19E-58	261,82	242,28	261,82	-0,1214	61665000	115	17,6053
0,990309	1,23E-32	202,41	162,67	162,59	0,20542	464850000	24	20,1346
0,985216	1,24E-05	118,33	102,08	118,33	-0,18655	30400000	394	18,7687
0,999997	0,00183261	83,045	66,132	83,045	-0,77875	10445000	280	56,2588
0,999958	7,36E-07	130,25	113,04	125,73	-0,13833	84351000	371	44,0642
0,808813	9,26E-06	103,17	85,505	95,094	0,35173	35859000	2531	8,61508
0,999403	0,000137767	108,45	89,613	53,328	-0,55402	85838000	2757	32,235
0,996597	0,00393049	59,728	45,037	59,728	-0,033297	9311300	1600	29,6556
1	8,56E-05	143,14	117,1	124,09	0,2225	187730000	494	118,642
0,999992	0,00209735	109,11	31,187	100,98	-0,028364	51360000	231	51,1507
0,869533	0,000332254	86,539	64,853	86,539	0,29049	17680000	250	13,3202
1	0,00771859	65,305	48,378	65,305	-0,12564	7842400	18	65,3052
1	6,51E-05	138,28	71,047	133,48	0,038814	165000000	141	117,1
1	1,00E-98	309,12	290,71	171,88	0,063885	1501900000	156	82,4907
0,99996	5,92E-20	187,47	187,47	106,94	-0,38524	1901300000	143	44,1795
1	1,23E-51	265,73	242,39	93,667	0,15869	37560000	306	93,6667
0,999059	1,98E-11	203,32	159,01	203,32	-1,4168	19619000	316	32,0617
0,999998	5,10E-80	270,39	254,02	245,19	-0,099295	153510000	216	56,9277
1	6,48E-11	159,56	142,4	114,91	-0,098491	63090000	36	71,4151

Polito et al. Universal ready-to-use immunotherapeutic approach for the treatment of cancer: expanded and activated polyclonal γδ memory T cells.
Phosphorylation sites

1	6,94E-11	190,51	148,47	114,82	-0,90242	357130000	13	114,817
1	0,000161878	133,81	67,752	93,096	-0,080806	20811000	NaN	93,0956
1	1,14E-24	206,74	184,9	206,74	-0,073378	110280000	64	69,2877
0,999571	0,0269347	45,438	19,073	45,438	0,066861	7245300	199	33,6694
0,999982	9,19E-20	158,2	136,74	158,2	0,03684	409230000	1138	47,4124
1	0,00189881	135	111,21	46,408	-0,9768	65306000	833	46,4083
1	1,13E-82	342,42	273,45	295,22	-0,032144	61006000000	814	167,702
1	0,00123165	132,17	132,17	68,786	-0,28608	29156000	504	68,7856
1	8,48E-13	183,73	154,11	183,73	-0,34061	149400000	469	75,6661
0,999559	9,48E-06	113,68	100,6	110,78	-0,19359	161930000	419	33,5573
0,985716	2,31E-12	180,81	141,3	180,81	-0,79161	6296900	540	18,3995
0,998994	7,87E-106	320,86	273,15	216,25	0,225	839970000	695	31,7187
0,998155	0,00550972	96,948	37,703	96,948	0,22474	88372000	525	27,3495
0,999988	1,37E-05	157,24	117,24	143,89	-0,096606	332150000	378	49,1984
1	2,13E-21	189,48	154,57	189,48	-0,6386	60052000	437	66,2891
0,966726	2,21E-05	87,85	70,15	62,765	-0,9791	19859000	158	15,675
1	8,43E-12	168,64	139,28	168,64	0,81041	39827000	126	92,0535
0,964329	0,000176939	105,99	84,442	105,99	-1,3486	28782000	448	14,685
0,945819	0,00996585	61,649	35,637	61,649	0,25967	6483500	402	13,6452
1	0,0189635	49,444	25,489	49,444	0,15014	3254200	217	49,4441
0,998451	2,23E-06	119,02	92,358	86,261	-0,40791	26036000	75	28,0934
0,999997	9,21E-05	151,44	124,23	151,44	0,12636	18907000	86	55,1133
0,995828	0,000457105	87,148	75,356	85,493	0,26012	41221000	367	24,943
0,994604	0,000209114	124,25	102,62	117	0,19814	34352000	42	22,6557
0,996495	0,00107661	92,943	63,626	54,486	0,38083	54944000	82	24,5599
1	3,88E-07	194,48	130,11	194,48	-0,1107	114750000	88	86,9276
1	2,35E-128	377,81	329,35	335,98	-0,24509	226950000	2273	124,527
1	3,76E-125	364,88	343,25	364,88	-0,13325	1545900000	1258	90,7955
1	5,17E-20	189,34	168,55	152,71	-0,13039	96327000	217	82,4715
0,99989	1,63E-36	254,56	221,95	241,64	0,15592	110860000	71	39,6262
1	8,35E-53	273,79	241,09	196,31	-0,093252	342720000	94	87,2706
0,999049	0,00104643	117,86	87,309	101,53	0,22591	20760000	85	31,443
0,999891	5,31E-21	237,05	237,05	184,01	-0,0004177	570530000	155	39,6309
1	0,0107127	76,302	30,286	70,675	0,32085	19308000	347	70,675

Polito et al. Universal ready-to-use immunotherapeutic approach for the treatment of cancer: expanded and activated polyclonal γδ memory T cells.
Phosphorylation sites

0,999999	1,37E-108	280,69	260,65	241,12	0,11687	628050000	238	60,6171
0,999993	1,13E-76	241,12	217,21	241,12	0,11687	397360000	240	51,2646
1	4,19E-70	325,24	284,47	314,52	-0,22013	782650000	359	163,216
0,999999	9,32E-10	205,26	165,32	111,79	0,19933	349290000	384	58,7496
0,980966	7,56E-05	164,63	107,04	164,63	-1,2364	45144000	879	17,1213
1	4,46E-07	211,96	134,29	63,76	0,033402	612840000	21	63,7601
0,999995	0,000165436	123,75	97,073	123,75	0,19501	12464000	462	53,1245
1	5,29E-11	214,14	152,71	205,86	-0,069404	69563000	465	95,7799
0,758469	0,0169141	60,691	38,92	60,691	1,0301	7066000	46	4,96965
0,997205	6,36E-95	321,05	273,43	215,75	0,76302	285210000	887	25,5237
0,976399	1,28E-66	289,86	248,77	247,92	-0,31957	209910000	898	18,7725
1	3,84E-05	154,6	135,35	154,6	0,040304	54873000	2	89,1239
1	1,06E-06	178,68	157,81	118,24	-0,20183	57789000	372	118,24
1	1,17E-05	101,6	85,101	53,037	0,18678	17785000	254	53,0367
0,960588	0,000118269	100,53	75,348	100,53	-0,23341	15769000	467	15,5876
0,999961	0,000165979	123,64	83,05	78,508	0,21959	46694000	644	43,5873
0,999739	2,49E-06	164,68	139,21	164,68	0,2623	2780400	1935	35,8362
0,998096	2,76E-06	134,21	103,38	111,43	-0,097851	444740000	374	29,3368
0,815158	3,00E-05	145,7	105,55	105,14	0,39451	8288600	244	6,46092
1	0,000137178	156,29	107,85	127,18	0,41183	324070000	379	127,175
1	1,23E-10	213,35	194,37	177,57	-0,14395	942730000	399	177,572
1	0,000129488	123,96	99,034	121,36	0,062243	11854000	43	101,364
0,999269	8,83E-15	205,95	176,62	205,95	0,045369	36757000	1154	31,3568
0,999064	9,65E-11	196,64	160,75	196,64	-0,022051	41726000	1124	30,2833
1	1,90E-09	159,04	126,48	159,04	0,063202	717200000	8305	134,26
1	3,67E-13	167,56	167,56	158,97	0,18455	303080000	8277	64,809
1	3,67E-13	167,56	167,56	158,97	0,18455	379510000	8280	84,3875
0,972496	0,00107697	88,507	61,726	88,507	0,093245	6634400	5943	15,4849
1	2,41E-16	151,02	129,25	103,13	0,33741	272700000	8223	103,135
0,999994	0,0162279	57,175	22,425	56,551	-2,1292	93647000	4796	52,4316
0,999372	1,28E-43	219,18	189,54	219,18	-0,38839	87122000	5883	32,0174
0,999999	0,0152555	61,344	25,149	61,344	-0,39986	57605000	21	60,2086
1	1,42E-61	248,98	234,82	248,98	0,037151	1242600000	804	151,592
1	0,00542921	114,86	72,462	61,09	0,25509	22861000	246	61,0899

Polito et al. Universal ready-to-use immunotherapeutic approach for the treatment of cancer: expanded and activated polyclonal γδ memory T cells.
Phosphorylation sites

1	0,00051797	109,24	59,128	109,24	-0,33789	24574000	153	109,236
1	5,00E-48	280,25	244,23	280,25	0,31976	261520000	1301	172,287
0,999882	2,96E-14	173,89	133,27	161,8	0,21569	43940000	1563	39,2763
1	0,000762433	143,4	88,286	119,53	0,47556	110200000	296	119,528
0,939762	3,95E-06	160,11	140,49	160,11	0,5272	113820000	1528	11,9314
1	1,25E-69	276,11	229,02	276,11	0,17621	93586000	1595	91,0162
1	0,0279921	60,398	20,776	60,398	0,13804	8763600	972	60,3978
0,999401	2,58E-12	161,24	142,99	161,24	0,13048	416760000	508	32,2234
1	0,000345509	108,34	82,9	100,41	0,057925	30791000	153	100,41
1	1,41E-14	175,33	147,05	175,33	0,30409	68937000	404	147,031
0,990972	5,07E-12	175,59	149,71	141,91	0,14507	219040000	803	20,4085
0,796676	9,00E-05	81,317	67,655	59,003	0,040143	5080800	310	9,39708
0,99995	0,00419053	77,355	37,257	77,355	0,24849	11750000	59	43,0431
1	0,00406396	142	66,17	50,13	-0,0060016	931640000	183	50,1303
0,999941	0,00107902	87,932	54,703	87,932	0,44522	24983000	326	42,3183
0,956938	5,55E-06	165,34	123,64	132,51	0,13162	64676000	369	13,9584
0,997813	9,13E-05	142,83	111,23	142,83	-0,4352	3249700	194	26,5923
1	0,00640875	66,962	66,962	46,819	0,019231	31314000	594	46,8189
0,980081	8,40E-05	164,15	137,49	107,74	0,025	716330000	90	16,9766
0,999994	8,05E-06	113,6	105,11	113,6	0,21627	27177000	131	52,0806
0,947548	1,74E-11	205,26	175	152,88	-0,018448	24321000	1323	12,5684
0,993379	1,81E-47	240,34	188,85	184,03	-0,23788	162280000	1031	21,7616
1	3,74E-06	181,71	161,04	62,799	0,58448	409240000	1217	62,7994
0,999989	0,00500693	95,741	76,038	65,88	-0,056917	18939000	1190	49,7125
1	3,40E-05	107,63	75,359	107,63	0,45628	28535000	466	74,6376
0,999999	2,17E-12	177,02	158,88	155,06	0,033362	377240000	1991	60,3656
0,998505	0,00944727	79,16	16,602	53,124	-0,31838	38236000	140	28,2479
0,994349	8,39E-38	204,9	172,42	154,68	0,29056	337750000	1066	23,9877
1	5,14E-27	209,68	181,35	189,34	-0,15804	2811600000	2004	68,7624
0,999949	2,44E-31	225,98	198,92	191,09	0,086642	492160000	727	45,9012
1	7,88E-158	371,86	355,56	300,03	0,17969	900090000	2025	88,125
0,999996	7,88E-158	371,86	355,56	340,9	0,16506	887810000	2027	53,8679
0,880241	2,56E-42	257,02	226,53	167,92	-0,01079	48078000	231	8,66295
1	0,000497639	131,11	89,803	87,258	0,22223	41238000	172	87,258

Polito et al. Universal ready-to-use immunotherapeutic approach for the treatment of cancer: expanded and activated polyclonal γδ memory T cells.
Phosphorylation sites

1	2,75E-58	267,5	237,89	63,227	1,9119	206200000	165	63,2275
0,995289	6,88E-15	208,85	195,18	203,99	-0,013577	34954000	1298	23,2541
0,999082	0,000662121	96,604	70,487	93,839	-0,016271	66335000	1518	30,3664
1	0,000361464	106,35	87,279	106,35	-0,14463	17300000	175	106,354
0,958312	1,01E-05	97,063	70,646	53,475	-0,5234	18286000	308	14,0087
1	2,01E-07	183,7	109,16	120,36	-0,074426	2164400000	145	120,36
1	0,000144587	124,16	80,569	77,062	-0,20681	181030000	125	77,0624
0,964219	7,52E-05	119,23	89,139	100,77	-0,073874	55849000	221	14,318
1	7,94E-08	174,39	129,97	134,13	-0,18616	459250000	224	66,7451
1	1,19E-72	232,29	215,49	232,29	0,86799	120190000	54	68,9765
1	2,38E-28	190,77	128,81	86,785	0,058207	396980000	368	86,7847
0,999449	3,74E-33	178,22	168,72	128,06	0,15828	1521500000	432	32,5997
1	3,25E-07	186,05	155,09	163,62	-0,0080807	91657000	195	88,1333
0,999749	3,18E-236	408,94	392,97	408,94	-0,48815	486720000	349	36,0022
0,955922	3,74E-55	238,03	218,97	238,03	-0,16373	103330000	356	13,4627
0,999753	4,46E-43	225,34	206,28	225,34	-0,39313	238140000	358	38,4186
0,985835	0,023158	45,864	20,928	45,864	0,71768	6048900	232	18,5282
0,999999	6,15E-75	261,82	242,24	253,44	0,38058	662670000	304	58,8202
0,998522	6,15E-75	261,82	242,24	210,32	0,38583	565600000	307	28,298
0,999786	4,71E-18	178,63	158,6	119,5	-0,49584	763570000	639	40,0937
0,987426	4,15E-15	192,63	174,54	106,13	0,03139	92365000	503	18,9504
1	7,22E-27	203,78	181,38	203,78	0,080586	277080000	250	65,8818
0,980784	2,18E-05	132,31	116,77	115,12	-1,3891	34002000	1018	17,079
0,997986	0,000195838	70,453	55,363	54,225	-0,080416	7150900	981	26,9513
1	2,71E-31	224,56	199	224,56	-0,70672	27424000	62	178,642
0,98051	0,000399366	137,46	102,01	100,88	0,052035	141840000	410	17,0165
0,995548	1,49E-15	137,19	115,85	99,663	0,028764	145420000	1348	24,154
0,947439	0,00132666	82,202	63,977	81,904	0,5942	7623900	1276	12,5588
1	0,000317948	142,98	72,726	120,99	-0,012215	45454000	1486	120,989
0,999148	1,31E-137	299,55	284,16	239,79	-0,27938	1549700000	1566	29,2129
1	1,41E-06	182,33	79,925	182,33	0,071495	49527000	853	182,325
1	2,82E-05	164,68	134,92	67,952	-0,61453	106740000	1475	67,9517
0,999991	1,35E-08	147,41	128,86	147,41	-1,3496	105450000	835	50,3518
0,999998	6,02E-08	163,9	141,61	148,03	0,22474	134650000	1448	57,5829

0,998896	2,60E-20	189,51	167,75	103,85	0,11601	138070000	504	29,5658
1	1,87E-05	118,16	63,676	87,196	-0,56016	53050000	1378	87,1962
1	0,000268423	110,38	86,424	89,629	0,08604	7749400	306	89,6295
0,991241	6,43E-48	220,74	193,02	44,968	0,18583	1065600000	1237	20,6404
0,992048	1,04E-24	191,04	159,6	44,968	0,18583	630990000	1240	21,0707
0,949491	7,20E-07	143,14	119,29	143,14	-0,42447	9000200	437	12,7486
0,999704	3,08E-66	288,53	288,53	139,04	-0,026775	269890000	440	35,2925
0,987168	1,42E-10	159,75	115,14	118,22	0,12506	396270000	1360	18,9521
0,979758	1,15E-10	157,3	142,68	126,28	-0,082904	370840000	1371	18,3461
1	1,58E-14	164,2	145,79	106,42	-0,2897	791280000	1202	106,424
0,999914	7,77E-07	136,57	97,227	136,57	-0,13273	19867000	1229	40,639
0,999913	3,57E-05	170,74	121,79	91,265	0,59729	323430000	1124	41,8471
1	0,000985294	103,63	35,006	96,551	0,3314	52197000	1142	96,5513
0,99671	1,23E-55	241,23	241,23	69,873	0,61393	941140000	915	24,9044
1	1,26E-14	238,01	161,68	238,01	-0,056945	101180000	814	73,8772
0,995316	0,00126352	91,307	53,694	91,307	0,76073	53686000	1034	23,2735
0,976376	3,92E-05	127,88	103,5	78,285	-0,0171	18024000	1095	16,9101
0,999994	0,000653655	121,36	73,269	121,36	0,10103	68072000	867	52,085
0,999985	4,66E-69	268,88	239,85	268,88	0,12224	338280000	973	48,2086
1	0,000612186	103,08	68,391	68,536	0,014331	163090000	991	68,5357
0,970519	0,00597115	54,234	41,674	54,234	0,14172	3106600	892	17,931
0,999962	0,00356869	94,363	41,637	94,363	0,016114	17672000	28	44,1683
0,976476	2,02E-06	166,14	132,27	166,14	-0,069695	8928500	250	16,1826
0,999999	5,10E-08	201,38	173,61	201,38	0,30242	85902000	709	58,6191
1	1,82E-106	312,78	261,64	270,75	0,05467	186580000	942	117,658
0,9993	0,000225707	84,874	72,513	84,874	-0,36094	7609300	755	31,6217
0,999408	1,30E-14	177,6	160,43	177,6	0,040211	270370000	408	33,428
0,989548	0,000209789	122,64	85,294	122,64	0,56016	7139700	408	19,7662
0,999998	0,00041495	129,34	98,614	129,34	0,055172	39961000	329	58,2583
0,999999	0,000728479	113,35	113,35	109,1	-0,39763	19089000	126	58,6518
1	2,02E-83	295,78	274,16	164,31	-0,25374	14248000000	188	164,305
1	5,77E-15	171,57	160,86	158,53	-0,25516	206090000	206	80,4462
0,999961	1,98E-09	130,46	121	79,82	0,043174	32993000	208	44,1428
0,999781	9,43E-08	116,74	60,723	116,54	0,13152	25302000	211	36,6321

Polito et al. Universal ready-to-use immunotherapeutic approach for the treatment of cancer: expanded and activated polyclonal γδ memory T cells.
Phosphorylation sites

1	3,78E-25	225,86	201,33	225,86	0,3202	29064000	109	126,043
0,999969	7,30E-07	139,11	118,27	139,11	0,084667	7049600	118	45,097
0,999966	7,06E-16	229,4	183,24	174,87	0,45533	540410000	596	43,317
1	5,26E-32	264,84	243,96	233,28	-0,47206	4804300000	598	81,1707
0,999449	5,26E-32	264,84	243,96	198,14	0,3818	1381900000	604	32,6089
0,985934	0,00287953	92,112	68,589	92,112	-0,28368	53683000	164	18,4569
0,968109	1,84E-07	189,13	119,1	168,3	-0,04742	229910000	9	16,233
0,997544	3,03E-07	186,76	129,59	186,76	0,37198	12752000	157	26,0878
1	0,00449993	99,305	24,819	99,305	-0,012786	12125000	471	99,3048
1	4,92E-41	313,52	272,41	194,24	0,11868	269910000	31	65,8783
0,750926	0,000240461	124,25	87,269	124,25	0,25978	8806100	368	4,79268
0,999377	1,99E-24	247,4	194,61	194,37	0,15511	367070000	370	32,0548
0,972785	4,17E-05	116,73	116,73	88,548	-0,093391	86179000	327	17,5618
0,999851	8,58E-05	150,05	102,49	144,73	-0,41149	54352000	37	38,3064
1	0,000153443	130,72	91,137	89,231	-0,31546	16184000	345	89,2306
1	5,55E-05	154,23	133,39	154,23	-0,55335	40769000	302	74,3585
1	0,00614383	82,261	35,442	82,261	-1,4414	7603100	154	82,2607
1	3,57E-15	178,11	165,05	178,11	0,0082934	356480000	536	75,9513
1	2,52E-15	158,31	138,99	140,34	0,20345	146490000	563	117,937
0,980272	5,45E-11	140,34	121,72	140,34	0,20345	35066000	572	17,9867
1	0,000538586	128,31	70,964	68,044	0,046881	417190000	835	68,0445
1	0,00204377	100,71	46,818	50,805	0,28794	28037000	1377	50,8046
0,999988	6,36E-06	143,28	115,95	104,44	0,0071336	41903000	432	49,2514
0,988401	0,000174173	84,573	59,064	84,573	0,084066	189740000	187	20,2778
1	6,95E-12	203,27	143,11	181,51	-0,070295	124620000	163	82,4435
0,999462	4,49E-59	292,76	265,1	292,76	-0,061357	365410000	168	32,6869
1	2,93E-49	264,97	241,06	151,13	0,42751	405090000	433	116,941
0,982935	2,05E-25	201,61	179,08	185,87	0,6766	39031000	155	17,6053
0,99746	3,37E-15	142,18	132,03	127	-0,21732	191030000	16	26,9064
0,999981	1,76E-80	282,05	262,26	161,01	-0,014833	7233000000	605	47,3083
0,988442	1,31E-43	231,41	219,72	231,41	-0,35412	433710000	359	19,3208
1	0,00294069	84,615	13,706	84,615	-0,085172	16727000	461	84,6146
0,994587	0,00295594	80,455	47,456	80,455	-0,046684	72320000	182	24,0208
1	8,53E-12	211,45	197,73	191,57	-0,06016	376290000	9	70,0973

0,998012	0,0060514	79,633	56,257	79,633	0,0096416	27646000	307	27,078
0,833509	6,16E-09	178,23	157,39	140,2	0,104	339720000	793	6,9952
0,992004	4,70E-17	227,42	63,642	184,21	-0,60599	33173000	272	20,9365
0,999755	4,00E-54	282,49	224,51	282,49	-0,39575	1386000000	85	36,2752
0,798321	4,94E-16	110,76	97,611	110,76	0,11597	99853000	244	5,98087
0,997846	4,12E-116	326,25	291,85	326,25	-0,084647	166270000	137	27,438
0,995732	2,07E-63	252,69	232,65	247,26	-0,47589	2322800000	812	23,7017
0,982459	9,95E-19	208,49	177,1	201,18	-0,02453	105160000	106	17,8628
0,996409	3,21E-66	287,91	262,35	274,6	0,048133	97666000	924	24,4324
0,873505	0,00550354	79,633	63,743	79,633	0,30085	17079000	1262	8,39208
0,999753	9,51E-57	250,52	224,8	250,52	-0,21661	469580000	1030	36,745
0,999997	0,00173085	89,171	43,17	89,171	0,072957	7103300	1277	54,9651
0,999997	3,79E-10	193,64	152,92	193,64	0,44121	36062000	417	54,916
1	5,34E-24	186,17	169,09	186,17	0,38103	36868000	555	76,755
1	0,00947576	68,277	52,738	68,277	-0,20035	10658000	721	68,2774
1	6,37E-06	166,52	137,57	65,775	-0,036278	444690000	1319	65,7754
0,999132	1,16E-36	259	218,06	242,91	0,13122	527480000	797	35,3819
0,999997	0,00119081	97,456	53,139	97,456	0,27389	7663500	83	54,8577
0,99986	9,59E-07	147,17	110,01	147,17	-0,83133	343620000	721	38,5345
0,985407	8,60E-06	125,89	88,737	125,89	-0,054679	8879300	863	18,8188
0,999979	4,84E-06	98,74	72,593	98,74	-0,45623	38335000	2	46,7142
1	0,000199846	143,98	118,76	143,98	-0,20228	7221600	308	111,681
1	4,98E-05	163,84	102,28	163,84	-0,0335	44469000	1190	95,9973
0,998094	0,000303802	121,21	93,581	84,297	-0,2806	189850000	1217	27,1909
0,999983	4,14E-49	255,6	229,23	237,24	0,15853	52052000	88	47,76
0,994245	9,48E-15	195,89	148,35	195,89	-0,2922	70970000	298	22,3783
0,951582	0,000112835	124,81	106,03	124,81	0,076847	10788000	25	12,9344
0,999992	1,48E-24	195,03	151,04	195,03	0,5702	154170000	147	51,0544
1	0,000565987	98,156	60,429	76,255	-0,32109	26333000	998	76,2551
0,986336	5,43E-07	137,42	109,52	137,42	0,1256	40335000	25	18,8466
0,9997	2,19E-06	173,76	148,98	156,58	-1,027	393280000	651	35,2207
0,839629	3,86E-05	149,17	101,56	129,89	-0,088058	10396000	653	7,18962
0,960735	1,02E-05	158,01	127,45	58,268	-0,15029	417110000	676	13,8626
0,999773	0,00056805	126,71	108,67	126,71	0,13812	93145000	679	36,4448

Polito et al. Universal ready-to-use immunotherapeutic approach for the treatment of cancer: expanded and activated polyclonal γδ memory T cells.
Phosphorylation sites

1	2,13E-40	273,33	239,15	192,16	0,04698	404570000	461	140,951
1	2,52E-41	259,55	228,23	259,55	-0,36325	1437700000	661	133,391
0,998517	1,23E-15	221,13	199,88	221,13	0,062201	551440000	923	28,2827
1	1,96E-15	184,53	171,87	184,53	0,26163	15112000	996	69,6124
0,998683	1,35E-33	217,29	188,71	213,2	-0,32934	127360000	84	31,3215
0,998147	8,43E-33	236,31	208,34	188,38	-0,084603	365660000	642	27,3342
0,981901	9,46E-33	238,83	215,35	197,15	-0,39622	120400000	643	17,344
1	0,00273175	88,364	75,979	88,364	-0,0055041	8376400	298	88,3637
0,93758	4,90E-10	161,49	136,34	161,49	0,26935	29000000	1825	11,7668
1	2,38E-58	311,24	252,09	228,26	0,16059	4007000000	100	93,1868
0,976865	0,000766103	108,71	83,859	108,71	0,035276	9294900	57	16,2559
0,999911	0,0177826	80,098	30,449	80,098	-0,046616	6623300	659	40,7999
0,999938	0,000470362	146,57	72,374	136,49	-0,29137	2269300000	599	42,0794
0,999857	0,0033759	78,287	46,487	74,533	0,10672	162700000	601	38,8812
0,996232	2,60E-20	159,22	134,76	159,22	-0,48969	219600000	64	27,2325
0,99706	1,50E-06	133,47	111,92	133,47	-0,70873	15749000	489	25,3041
0,9999	2,60E-17	230,45	191,78	171,56	0,15749	203320000	461	40,0054
1	5,55E-15	191,99	154,56	168,32	-0,50668	33735000	261	68,1335
0,999982	5,09E-05	139,67	120,12	139,67	-0,0036827	252270000	719	47,3623
0,999776	5,54E-09	154,84	139,59	154,84	-0,64237	72724000	680	36,4883
0,996338	0,000470259	100,04	73,919	90,614	-0,21679	128530000	523	24,3469
0,999997	0,000574072	104,82	88,313	78,488	0,099475	58446000	661	56,1596
1	6,43E-125	360,52	317,89	293,67	0,17924	1347000000	28	74,0061
1	1,48E-73	298,1	274,25	298,1	0,062887	1085800000	31	140,884
0,996824	3,18E-11	196,18	171,83	196,18	0,66393	37430000	244	24,9676
0,989856	0,00611854	67,081	39,777	67,081	0,44218	7903700	175	19,8938
1	0,00961609	66,457	7,7779	66,457	-0,25246	7729100	396	66,4567
1	1,19E-07	196,15	62,258	196,15	0,04399	17334000	71	87,8027
1	3,25E-07	181,66	140,38	181,66	0,22027	19539000	737	145,954
0,99999	7,34E-06	158,01	96,668	158,01	0,072452	15490000	951	50,0451
0,999973	4,74E-39	241,52	195,65	241,52	0,31417	109870000	36	45,7341
1	0,00108096	92,866	65,094	92,866	-0,078336	4629900	301	76,9847
1	0,00095144	85,457	76,594	85,457	-0,12836	10538000	446	67,0002
1	7,20E-05	148,28	118,97	148,28	-0,15576	65386000	76	82,0902

Polito et al. Universal ready-to-use immunotherapeutic approach for the treatment of cancer: expanded and activated polyclonal γδ memory T cells.
Phosphorylation sites

0,985636	0,000362905	84,653	60,095	84,653	-0,42422	29031000	1025	18,3644
0,999999	7,89E-37	262,42	231,1	244,36	0,1062	79901000	984	59,5984
0,988512	0,00021102	122,45	102,46	122,45	0,58801	14743000	584	19,786
1	0,00267294	92,65	58,78	92,65	-0,15	24711000	192	66,758
1	9,34E-16	231,03	180,76	208,6	0,48565	137080000	454	110,79
1	0,015956	66,595	13,268	47,712	-0,463	21915000	467	47,7121
1	1,48E-17	232,84	177,63	200,6	0,60933	76138000	311	200,602
0,998577	0,00206709	116,05	41,007	72,23	0,083159	73301000	859	28,4607
1	5,48E-10	164,27	133,84	164,27	0,60993	74533000	792	65,7315
0,999939	0,00121965	150,17	112,45	149,82	0,0974	56862000	212	42,1226
1	9,36E-05	142,91	122,9	64,224	0,17292	44722000	33	64,2245
0,979538	0,0368641	40,085	17,282	40,085	0,16645	7983600	368	16,8585
0,999933	1,28E-07	157,12	131	147,92	0,1716	223380000	239	41,7413
0,999986	1,97E-07	186,77	128,67	186,77	-0,028762	62364000	1061	48,558
0,999972	1,43E-24	222,39	181,7	176,6	0,004451	251390000	1379	45,4825
1	2,07E-61	250,02	226,96	50,479	-1,2303	159860000	514	50,4787
0,999994	1,23E-09	153,57	139,23	116,74	0,15751	236940000	1475	52,5128
0,997602	2,45E-286	451,75	419,36	451,75	0,13197	234470000	1345	26,1913
0,891764	1,61E-38	237,15	186,49	104,63	-0,32673	264180000	597	9,15131
1	1,39E-94	294	253,16	294	0,13891	1002200000	436	123,734
0,999998	4,25E-11	213,99	179,99	198,52	-0,09679	139730000	1408	57,175
1	2,00E-142	383,13	366,51	358,02	-0,10757	852210000	6	108,663
0,992226	0,00015859	59,838	49,126	59,838	0,0073335	42907000	1883	21,3266
0,999761	0,000161998	130,21	101,67	130,21	-0,073233	18137000	1224	36,208
0,978018	8,87E-05	128,73	104,66	81,703	-0,55723	80147000	598	16,4845
0,874469	1,83E-37	209,66	191,65	193,64	-0,037825	245340000	411	8,61412
0,999998	2,75E-60	297,55	251,55	211,29	0,28626	253400000	10	57,5663
1	2,67E-25	258,41	195,34	210,37	0,64899	151860000	112	210,372
0,995998	1,01E-05	74,397	65,922	74,397	-0,51131	21053000	304	25,6377
1	1,96E-08	191,06	157,09	154,1	-0,19512	83977000	53	95,8337
0,999995	2,40E-06	169,94	132,38	169,94	0,017055	30096000	81	53,3647
0,978475	5,32E-07	74,631	58,851	74,631	-0,024337	28191000	265	16,7491
0,999311	3,81E-10	185,02	155,04	174,52	-0,26421	73268000	466	31,6146
1	7,42E-07	180,74	159,19	171,15	0,26157	19990000	67	77,9085

Polito et al. Universal ready-to-use immunotherapeutic approach for the treatment of cancer: expanded and activated polyclonal γδ memory T cells.
Phosphorylation sites

0,999994	0,000132717	123,99	99,138	101,43	-0,22516	74790000	1974	52,3503
1	0,00142386	104,17	18,16	104,17	0,48702	10823000	1697	104,175
0,86271	3,81E-05	120,23	90,788	116,43	-0,039173	45335000	293	7,98227
0,999991	0,000223779	124,43	92,908	124,43	0,066902	35970000	12	50,4507
0,999999	3,75E-99	318,32	295,22	318,32	-0,36896	49043000	356	60,4862
1	3,77E-05	141,13	115,01	110,92	0,17164	116150000	2454	102,852
0,964548	0,00102389	100,02	73,575	100,02	0,23983	6850800	1981	14,3469
0,999802	0,00035942	105,13	97,654	105,13	-0,050947	17644000	1955	38,4705
0,986237	0,00234458	95,311	70,057	95,311	0,23508	25752000	788	18,7389
0,999994	1,52E-06	146,97	134,77	128,14	-0,28753	75947000	43	52,5657
0,923964	0,000306866	160,16	87,883	93,598	-0,13697	79262000	132	10,8471
1	8,58E-12	168,69	142,54	168,69	0,35576	66706000	2	160,195
1	1,31E-10	206,69	206,69	206,69	0,024642	401530000	90	104,795
1	0,000191572	128,83	80,3	128,83	-0,1953	24605000	567	128,827
0,999188	0,00167067	67,866	49,328	57,366	-0,40533	6856900	504	32,7724
1	8,36E-12	218,11	186,1	218,11	-0,26924	70349000	28	218,113
0,999996	6,12E-05	146,17	112,53	130,94	0,47621	300730000	582	53,5612
1	1,60E-10	212,93	181,39	48,704	0,204	740320000	738	48,704
0,999133	1,60E-31	273,72	218,94	270,79	0,042097	621470000	696	30,6816
1	5,14E-05	182,92	87,417	51,268	0,031138	174660000	675	51,2675
1	0,00628405	91,265	40,073	56,225	0,090701	77304000	683	56,2251
1	0	546,04	456,43	148,17	0,043152	7982400000	874	148,175
1	0,000313919	141,52	106,7	112,42	0,012382	1941900000	431	82,2407
1	2,66E-50	303,45	247,31	228,33	0,6016	4068800000	463	228,326
1	7,94E-16	228,58	197,05	228,33	0,6016	2456300000	465	228,326
1	1,10E-07	205,04	179,97	90,229	0,32299	2983900000	393	90,2292
1	1,92E-32	243,72	215,17	131,48	0,14978	2207000000	260	131,483
0,863715	0,000378587	157,25	95,653	157,25	0,14356	30289000	646	8,01921
0,996602	0,00740087	67,456	38,116	55,261	0,38014	86805000	715	27,5887
1	0,000125849	150,26	66,096	101,62	-0,84679	602480000	450	101,624
0,998193	0,00691776	108,72	52,169	98,182	0,33173	58873000	655	27,4541
1	0,000528201	108,43	45,247	59,418	1,2717	34402000	562	59,4177
0,99996	0,000293457	160,88	92,031	143,37	0,0032013	848220000	547	43,9319
1	0,000403075	192,38	120,77	192,38	-0,17904	1085200000	597	107,813

0,999999	5,73E-05	151,16	118,07	133,18	-0,14197	1475700000	605	61,5427
0,999527	0,000114867	126,12	93,595	126,12	-0,078532	19314000	797	33,252
0,891434	0,035625	56,547	23,654	56,547	-0,9545	35344000	187	9,14393
0,999992	0,000503331	110,39	82,183	110,39	0,20886	83001000	616	50,9613
0,999658	2,11E-05	176,87	147,4	115,8	0,24474	378790000	414	35,3361
0,999676	3,69E-06	160,91	111,72	160,91	0,17889	201490000	1010	34,8871
1	5,81E-07	145,58	123,46	75,539	-0,49549	293080000	992	75,5393
1	0,000680554	119,68	62,091	115,78	0,013304	43337000	247	100,342
0,802634	7,55E-06	97,771	89,967	79,365	-0,19207	19177000	152	10,2946
0,955043	1,36E-05	106,69	96,312	106,69	-0,20752	30363000	15	13,2722
0,844878	6,55E-06	151,98	80,627	151,98	0,1899	18557000	59	8,00182
0,808915	1,35E-05	72,461	63,433	61,971	0,0002131	135980000	469	8,28592
0,90017	0,000512957	122,64	101,09	111,12	-0,2833	36222000	481	9,81206
0,999964	6,87E-33	255,07	208,62	168,69	0,018911	1290300000	94	44,4355
1	1,41E-06	178,53	150,31	178,53	0,0039031	207210000	553	114,109
1	1,94E-58	314,06	240,39	314,06	0,13135	408260000	540	146,005
0,999611	3,61E-26	170,47	150,74	141,25	0,48171	116950000	374	34,2086
0,876441	0,00319291	79,489	40,713	47,058	0,0013579	148610000	78	6,97161
1	8,86E-05	142,55	30,729	135,43	-0,6886	55072000	927	67,7246
1	0,000170737	128,54	95,484	118,9	-0,64944	142690000	646	65,3361
1	0,00343555	81,525	67,388	48,981	-0,09491	88952000	345	48,9809
0,999997	4,13E-08	166,27	126,36	161,84	-0,0038714	33474000	388	55,3169
0,956812	0,00260428	95,483	72,164	95,483	-0,22163	15183000	998	13,4546
0,999996	0,00741258	78,692	34,267	78,692	0,39169	10868000	71	53,8385
0,999431	0,000158881	176,62	103,77	176,62	-0,48672	45344000	498	32,4436
1	8,56E-19	195,96	148,12	115,53	-0,29034	228130000	619	115,527
1	0,000615371	111,66	83,998	111,66	-0,27427	72886000	516	80,5254
1	5,46E-06	157,24	145,18	152,04	-0,11032	2667000000	340	131,416
1	0,000301067	132,08	81,727	66,246	0,28117	34467000	388	66,2462
0,999756	7,94E-26	217,36	188,38	181,4	-0,24099	27673000	411	36,7187
0,979483	2,87E-89	303,7	255,65	219,87	0,14194	62891000	416	16,8077
0,934326	0,000272448	114,56	79,79	114,56	-0,12295	7334300	319	14,3761
0,916943	0,00355665	50,857	34,587	50,857	-0,26663	8306700	206	10,4297
1	0,000403275	125,57	111,85	125,57	-0,54896	156900000	53	66,9648

Polito et al. Universal ready-to-use immunotherapeutic approach for the treatment of cancer: expanded and activated polyclonal γδ memory T cells.
Phosphorylation sites

0,999995	0,0026313	99,215	66,127	93,345	0,29657	64530000	50	52,9936
0,998456	0,000131319	130,47	99,918	94,287	0,0973	74353000	248	30,9955
1	4,13E-21	143,83	132,82	143,83	0,012462	292500000	174	69,4336
0,983416	1,29E-21	188,26	166,04	188,26	0,4037	44046000	223	17,7364
0,999575	0,00205045	113,66	87,553	103,55	0,10403	39165000	144	33,7218
1	0,000837412	109,29	36,057	92,792	0,16588	29768000	14	92,792
1	0,000387229	103,76	54,313	103,76	-0,27063	31523000	29	103,761
1	1,09E-06	180,28	109,79	81,904	-0,47625	29268000	197	81,9037
0,996269	8,16E-48	239,26	231,9	183,86	0,28473	198270000	38	24,2703
1	0,00203286	101,46	73,636	101,46	0,11789	5311200	87	101,46
0,977186	0,0385957	56,02	6,5761	56,02	0,15556	11312000	472	16,3178
1	1,22E-18	184,88	165,55	182,54	0,028725	178720000	178	131,714
1	7,54E-06	119,4	93,278	119,4	-0,039611	36300000	280	100,11
1	0,00205207	125,57	111,85	125,57	-0,54896	117650000	78	66,9648
1	4,00E-41	284,51	231,64	126,48	0,13706	29295000	82	126,485
1	4,69E-82	327,27	285,08	327,27	-0,52704	616140000	4	128,598
0,999951	2,08E-39	247,91	196,02	247,91	0,64165	88110000	242	43,0964
0,97988	4,81E-05	160,18	143,51	160,18	-0,081908	32098000	375	16,8755
1	1,48E-17	185,27	159,86	85,982	-0,46854	160460000	886	85,9817
0,997538	5,19E-38	268,17	234,76	182,1	-0,1866	203040000	214	26,1212
0,999554	1,27E-06	177,22	157,4	87,932	2,7625	821910000	197	34,6321
0,757394	0,00104749	81,723	66,692	57,173	1,1006	31961000	200	5,37553
1	4,05E-142	355,29	327,88	355,29	0,20004	764960000	710	71,1421
0,999994	3,02E-12	212,24	189,92	190,27	0,23889	76273000	81	52,2505
1	0,0140137	68,846	24,469	54,722	0,086295	12349000	143	54,7223
0,999497	0,00278122	87,476	72,81	85,676	0,027815	101130000	125	34,7098
1	1,75E-60	289,59	251,31	289,59	0,1946	733970000	220	145,66
0,999646	8,04E-82	312,66	287,46	312,66	0,509	408580000	107	34,5034
0,980255	2,67E-12	170,91	140,04	170,91	2,9111	44991000	108	16,9588
1	4,28E-68	261,74	239,64	204,24	-0,082468	1148900000	4	183,49
0,998595	8,42E-06	176,42	152,68	176,42	-0,081796	15951000	101	28,5163
0,776078	0,00132531	97,223	78,955	49,988	-1,6123	91981000	33	5,42396
1	3,68E-20	193,06	157,33	178,88	0,014505	229950000	46	125,329
1	6,94E-82	312,86	283,56	312,86	0,094637	136240000	17	168,572

Polito et al. Universal ready-to-use immunotherapeutic approach for the treatment of cancer: expanded and activated polyclonal γδ memory T cells.
Phosphorylation sites

0,859922	0,000493673	85,974	54,554	85,974	-0,16821	9183700	220	7,88584
0,999432	0,000101065	145	111,91	145	-0,022981	46498000	363	32,5615
0,999003	6,07E-23	200,47	184,96	200,47	-0,18566	185920000	98	30,0108
0,998013	6,30E-37	263,9	241,99	217,2	0,40338	33047000	869	29,0629
0,999996	6,05E-08	191,47	161,13	176,28	-0,24577	190930000	238	54,5585
0,871009	0,000182627	113,65	95,989	92,575	-0,14919	20475000	55	9,03534
0,999931	5,17E-11	241,39	164,57	241,39	-0,2149	1352600000	177	41,5856
0,927946	1,33E-14	175,39	130,14	175,39	-0,42508	10647000	199	11,2458
0,993569	2,36E-05	124,78	106,98	99,451	-0,06423	13723000	940	21,8892
0,996503	5,67E-05	156,02	128,26	138,35	-0,029028	38028000	1128	24,5473
1	0,0366783	44,299	13,301	44,299	2,0851	13223000	59	44,2994
0,792469	0,000399761	89,992	56,789	89,992	-0,059655	5742000	49	5,81899
0,99636	0,00553519	86,592	64,263	69,451	0,25539	18402000	534	24,3732
1	9,20E-16	223,46	169,12	190,87	0,24906	431500000	66	145,593
1	4,75E-08	168,5	129,28	110,56	-0,73949	37112000	564	85,677
1	1,71E-10	192,32	148,93	186,77	0,24586	76072000	567	68,2648
0,99984	4,44E-07	185,14	141,64	144,51	-0,098342	448750000	552	37,9644
0,999998	1,96E-08	176,04	157,85	153,09	0,43471	219240000	370	57,8251
1	1,40E-69	323,28	323,28	323,28	0,037299	3250100000	50	70,621
0,998014	1,87E-27	242,26	172,17	242,26	0,16394	234310000	58	30,0226
1	0,0168348	53,775	8,6138	53,775	-0,060284	47426000	184	53,7748
0,899008	2,75E-07	138,09	124,5	138,09	-0,16342	31555000	291	12,5061
0,999969	1,41E-10	163,31	148,05	147,22	0,25786	542340000	431	47,8163
1	8,66E-31	284,26	225,56	231,66	0,13906	5305900000	296	76,8062
0,999489	7,12E-06	113,54	91,067	75,548	0,028689	24436000	97	32,915
0,994296	2,28E-85	354,01	256,55	198,07	0,35343	463700000	270	22,4137
1	5,17E-89	314,38	272,17	314,38	-0,56203	2085700000	576	113,241
1	2,37E-47	241,23	195,25	157,96	0,23575	552040000	739	70,8006
0,907829	0,0080066	62,035	27,137	62,035	0,90751	19979000	115	9,93412
0,999534	0,000243145	114,64	57,689	101,61	-0,25437	172130000	65	33,437
1	3,72E-05	148,72	89,152	47,568	0,096897	606220000	47	47,5678
1	0,00224467	103,88	54,821	103,88	0,33076	21229000	72	103,88
1	0,00745862	85,161	25,043	85,161	-0,3026	134510000	234	85,1608
1	0,000222086	113,93	85,121	113,93	-0,65249	10495000	421	113,926

Polito et al. Universal ready-to-use immunotherapeutic approach for the treatment of cancer: expanded and activated polyclonal γδ memory T cells.
Phosphorylation sites

1	1,72E-47	269,91	212,65	263,45	-0,3763	4090400000	2	168,526
1	6,35E-12	193,51	176,48	184,21	-0,038468	388310000	2	118,735
0,961309	2,09E-24	236,3	210,86	236,3	0,0071682	94477000	9	14,1395
1	5,71E-18	233,02	213,36	233,02	0,26003	93732000	94	75,8208
1	3,78E-31	220,04	190,72	121,72	-0,92193	62278000	72	121,725
0,99915	1,31E-07	201,33	135,45	201,33	-0,17619	1516900000	192	30,7047
0,996971	4,06E-20	269,04	233,5	269,04	-0,0029824	63444000	128	25,1932
0,988709	8,63E-05	107,25	90,94	92,792	-0,35993	55540000	5	19,8024
1	0,000613722	85,493	73,11	61,444	-0,10275	45697000	12	61,4435
1	0,000117988	94,544	81,777	94,544	0,079622	3241300	81	94,5445
0,99998	0,000315131	103,65	92,418	103,65	0,019399	25743000	70	46,9942
1	4,61E-05	160,69	132,48	159,08	-0,057325	54249000	88	104,476
0,998604	2,04E-20	200,26	185,1	200,26	-0,042169	109870000	60	29,0496
0,999992	5,05E-07	145,69	130,53	132,44	-0,11657	23418000	109	51,1278
0,999998	1,59E-110	318,64	298,02	318,64	0,018715	123770000	62	57,1788
1	0,000144491	128,67	90,588	85,536	-0,044931	290060000	278	85,5355
1	5,96E-11	200,9	163,34	131,56	-0,02994	65311000	24	131,556
0,824175	4,13E-31	232,51	212,13	206,62	1,1034	291050000	351	6,70938
0,863937	1,32E-31	232,58	211,96	81,494	-0,14174	753480000	352	8,02757
1	8,55E-08	167,29	127,06	167,29	0,3024	17542000	286	87,8242
0,998939	1,86E-14	210,67	182,31	210,67	-0,12271	89104000	292	31,1172
0,999907	4,53E-70	277,73	242,49	148,04	-0,12589	545320000	301	42,7155
0,99967	1,73E-15	174,72	156,62	134,69	-0,14392	460280000	311	34,8203
0,98724	4,91E-07	138,78	113,11	110,91	0,077815	106340000	327	21,6438
0,999394	2,49E-48	289,37	252,7	289,37	0,13171	17031000	1313	32,1755
1	5,91E-07	189,33	141,06	69,416	0,53905	53689000	938	69,4157
0,996836	3,81E-06	139	104,18	139	-0,27307	52065000	921	24,9841
0,999996	0,00608518	67,704	37,537	67,704	0,052021	12565000	1469	53,5468
0,999916	2,15E-11	145,86	135,18	145,86	-0,27782	79711000	2213	40,9671
1	6,76E-05	120,66	104,77	120,66	0,015341	16062000	60	108,895
0,999694	1,74E-08	151,48	134,08	151,48	0,76412	11718000	12	35,1368
1	4,20E-07	156,69	137,63	156,69	0,11888	17388000	178	112,263
0,999978	1,17E-05	173,19	153,92	169,09	0,12346	51853000	47	46,6301
0,999958	3,42E-27	211,67	190,44	181,6	0,40792	56310000	11	43,751

Polito et al. Universal ready-to-use immunotherapeutic approach for the treatment of cancer: expanded and activated polyclonal γδ memory T cells.
Phosphorylation sites

1	6,93E-12	153,32	136,52	153,32	0,65944	127360000	290	70,6972
1	7,85E-12	204,17	145,06	204,17	-0,10624	74326000	185	126,98
0,999207	1,90E-09	163,7	133,22	46,592	0,097591	77862000	468	31,0002
1	0,00165047	86,114	53,115	65,348	-0,36364	52472000	105	65,3475
1	3,74E-08	194,94	166,85	72,433	-0,27365	22954000	204	72,4328
1	0,000340132	105,38	65,352	65,298	-0,69689	597490000	144	65,2985
0,905878	3,37E-09	140,94	119,33	140,94	-0,37394	47049000	79	9,84101
0,844921	5,00E-05	136,96	113,53	108,56	-0,10243	41200000	9	7,63273
1	3,02E-10	135,72	114,87	135,72	0,10495	131800000	454	99,3705
0,999987	2,43E-06	73,916	62,516	73,916	-0,29354	59594000	1093	49,4305
1	1,07E-05	115,98	77,467	90,714	0,43307	16732000	1083	71,1607
1	8,04E-48	276,38	247,66	255,21	0,068203	269300000	531	73,7029
0,892483	2,17E-16	159,95	147,45	72,22	-0,51666	43783000	364	9,19127
0,999945	7,18E-06	169,04	84,561	160,27	0,43539	78522000	187	42,5687
1	7,88E-241	468,18	420,15	233,28	-0,908	545580000	130	175,747
0,999814	8,91E-96	351,8	305,98	351,8	-1,1615	117000000	139	37,2959
1	1,43E-168	327,5	306,32	269,49	-0,49694	8133600000	111	241,115
0,999999	0,00125911	111,33	59,569	111,33	0,39265	63500000	39	61,7802
1	1,17E-07	184,55	134,2	136,38	0,62528	59609000	175	136,376
0,999974	6,29E-16	227,42	176,41	176,87	0,35315	201040000	70	45,8127
1	9,53E-08	144,5	118,96	71,623	-0,15261	932090000	231	71,6227
1	9,53E-08	144,5	118,96	71,623	-0,15261	926250000	235	71,6227
0,99979	9,14E-31	214,4	195,15	161,19	0,5719	170680000	16	36,9037
1	3,25E-41	281,68	247,68	209,11	0,24144	90724000	175	209,111
1	1,17E-27	250,17	250,17	137,45	-0,16699	257190000	387	96,955
0,935623	1,93E-214	393,88	345,96	386,78	1,0664	62252000	424	11,6994
0,999968	0,000881001	106,37	7,6352	106,37	-0,03128	24944000	233	45,8176
0,998014	4,58E-30	201,61	189,41	197,63	0,24456	378670000	316	27,0124
1	1,42E-05	104,81	90,783	76,262	-0,43233	34475000	272	76,262
1	1,39E-05	105,46	77,598	76,262	-0,43233	19677000	277	76,262
1	2,35E-10	188,59	161,25	188,59	0,11373	210990000	50	99,1577
0,999999	1,32E-12	129,61	118,9	129,61	0,20872	67864000	3	60,3648
0,999074	2,63E-15	176,68	163,43	176,68	-0,72419	267900000	83	30,3347
1	1,85E-16	235,02	212,97	100,41	0,075524	532110000	80	100,41

Polito et al. Universal ready-to-use immunotherapeutic approach for the treatment of cancer: expanded and activated polyclonal γδ memory T cells.
Phosphorylation sites

1	6,77E-07	176,78	132,46	174,77	0,10744	300290000	126	68,1004
1	7,42E-05	89,946	72,029	88,168	0,65023	37194000	26	82,062
0,961447	8,60E-09	172,83	155,25	75,463	-0,0096566	44867000	43	15,6004
0,992722	1,35E-05	155,97	138,46	129,82	-0,037103	54303000	340	21,3489
0,999992	0,0020459	107,49	90,223	99,305	0,098417	220460000	395	51,1473
0,997729	2,07E-21	190,19	166,03	190,19	-0,15893	87169000	756	29,715
0,998673	2,76E-08	175,61	149,99	106,16	-0,10086	203630000	100	28,7654
0,997496	2,22E-21	173,16	155,07	125,54	-0,066178	327910000	192	26,0031
0,999724	6,13E-39	238,19	214,39	238,19	-0,16887	63721000	205	35,5863
1	7,80E-05	106,38	80,94	106,38	0,17491	13202000	157	69,3643
0,983695	0,000621766	65,775	56,492	65,775	-0,36667	17381000	49	17,856
0,863557	5,70E-24	238,88	202,36	227,47	-0,1126	42493000	237	10,3164
0,999999	1,96E-27	241,8	223,94	241,8	0,048311	158430000	12	61,1044
0,999776	4,41E-05	138,55	113,62	136,33	0,040689	260870000	274	36,4971
1	9,97E-05	140,27	123,06	140,27	-0,36678	26151000	718	140,274
0,999926	0,000103032	141,59	88,795	103,91	-0,074075	51925000	746	41,4432
0,808718	0,00584406	68,224	6,1161	68,224	0,12478	11630000	484	6,26123
0,873149	0,00083991	72,898	55,089	72,898	-0,33999	5865900	89	8,37795
0,999873	7,36E-28	239,18	199,15	172,24	-0,13005	94623000	130	38,9594
0,997023	0,000217931	109,03	84,432	109,03	-0,1992	82792000	309	25,2494
0,999999	1,67E-39	223,28	191,91	223,28	-1,2293	68512000	130	59,2304
1	0,029104	41,399	11,487	41,399	-3,112	10996000	8	41,3995
0,99995	1,36E-16	220,4	188,88	220,4	-0,14752	102080000	255	42,9846
0,999995	0,0292656	56,225	11,916	56,225	-1,1964	9025700	243	53,0022
0,999995	1,25E-14	176,42	145,19	168,09	-0,65404	14265000	206	52,7473
0,99781	3,29E-13	220,32	211,37	177,42	-0,068974	301980000	357	28,0082
1	1,94E-32	241,65	203,68	41,164	0,023327	3387600000	799	41,1636
1	2,40E-33	205,89	189,62	115,92	-0,32768	380140000	527	75,2457
0,999595	1,41E-24	222,27	184,72	138,39	0,35569	221260000	1194	37,0026
0,99997	6,81E-34	266,2	222,82	266,2	0,014444	95512000	190	45,2296
0,997595	8,09E-33	271,6	203,6	208,28	-0,017879	304990000	290	26,1776
0,932867	4,71E-05	153,14	109,76	153,14	0,15465	53148000	40	11,4916
0,972874	2,77E-05	160,65	96,923	160,65	-0,57974	21957000	43	15,5512
0,999616	2,84E-66	289,68	194,45	230,07	-0,053046	185270000	575	34,1506

Polito et al. Universal ready-to-use immunotherapeutic approach for the treatment of cancer: expanded and activated polyclonal γδ memory T cells.
Phosphorylation sites

1	0,0032976	82,287	24,697	49,06	0,18324	4447400	72	49,0599
1	0,000143649	132,56	78,025	69,03	-0,17384	69404000	146	69,0301
0,995375	0,00203479	94,09	71,369	90,444	0,0072437	37864000	153	23,3287
1	2,32E-21	188,38	166,85	109,41	0,18798	67621000	160	78,1402
0,999994	8,47E-18	231,58	206,19	217,01	-0,32911	94318000	144	52,0759
1	9,89E-302	429	396,34	307,98	0,55767	20973000000	125	175,808
0,999956	0,00253572	99,815	29,99	99,815	-0,29276	9896600	137	43,5569
0,999855	0,0021023	107,79	43,523	103,7	0,1027	30873000	139	38,3949
0,999988	5,92E-05	123,19	102,46	66,595	-0,053803	31543000	6	49,2099
0,882216	2,59E-20	189,55	168,71	189,55	0,079525	21046000	539	8,7449
0,972072	2,10E-08	169,92	120,59	86,497	-0,043226	14635000	769	15,4167
1	1,11E-05	115,45	102,01	113,6	-0,0040153	76779000	933	106,537
0,999998	0,000148081	121,21	74,856	121,21	-0,38855	107390000	129	56,9839
1	2,41E-10	188,27	156,2	188,27	-0,021932	155150000	844	72,4644
0,996505	1,21E-34	195,07	181,51	195,07	0,17155	39477000	189	25,3756
1	5,61E-68	260,68	223,33	260,68	1,3491	234420000	880	72,1443
1	5,42E-08	194,39	93,841	142,83	0,3856	37159000	2107	142,83
1	0,00793674	75,738	28,656	75,738	-0,12314	6891300	1570	75,7382
0,978796	2,44E-14	189,62	168,27	189,62	0,31856	107410000	926	16,6427
0,999078	8,70E-05	115,74	99,977	115,74	0,018633	34883000	928	31,6289
0,994257	0,000327527	124,21	64,665	112,84	-0,30008	179230000	1546	22,411
1	6,25E-06	120,54	93,542	116,45	-0,1863	68380000	63	101,209
0,999868	4,54E-05	153,81	153,81	131,66	-0,1723	92603000	23	38,8072
1	1,90E-95	337,71	273,98	337,71	-0,39905	4109900000	104	87,6026
0,999998	5,04E-25	249,33	200,76	244,44	0,26273	32113000	120	56,4728
1	2,38E-15	184,32	160,99	111,43	-0,065847	175050000	182	96,8959
1	9,14E-49	254,44	233,35	111,43	-0,065847	215670000	185	97,7423
1	2,35E-74	299,3	283,19	299,3	-0,083025	2945100000	432	175,932
0,999977	0,000126864	157,52	128,75	157,52	0,2612	451450000	503	46,3923
1	1,36E-30	211,06	186,13	193,11	0,035718	462450000	342	98,6497
0,999985	1,06E-99	316,8	261,99	307,04	-0,25695	1029300000	352	49,3097
1	2,64E-80	279,45	260,68	279,45	0,095624	850090000	420	80,9723
0,999995	4,75E-05	139,64	103,86	139,64	0,0001778	87303000	761	53,3729
1	7,70E-161	399,26	358,17	92,866	0,37734	5390500000	158	92,8661

0,970261	0,000703534	79,81	52,147	79,81	-0,23808	14158000	780	15,1357
0,999557	3,09E-13	133,13	121,89	79,467	0,17644	35214000	840	33,5815
1	6,04E-07	182,43	116,5	182,43	0,13505	682770000	767	67,4933
0,999999	0,000680554	121,71	71,516	116,52	0,39645	114410000	599	62,5497
1	4,94E-05	152,4	103,37	64,64	0,43514	1546800000	671	64,6401
1	6,34E-14	264,98	193,95	247,69	-0,25897	5191400000	652	74,9466
0,999734	1,99E-12	174,85	121,4	173,56	-0,27303	180050000	929	35,9434
0,999994	0,00324608	142,08	45,969	126,52	-0,17226	231940000	547	52,3379
1	1,76E-07	198,74	114,79	160,52	0,77476	183170000	270	160,524
1	5,71E-07	181,76	163,49	179,83	-0,13317	1023200000	946	143,834
0,998835	0,000588479	108,97	89,23	108,97	-0,22169	17027000	1103	29,332
0,999997	3,72E-25	252,52	193,9	146,16	0,20634	768340000	307	56,3586
1	5,16E-45	235,34	190,21	170,66	-0,021835	171500000	46	97,9529
1	7,65E-92	316,12	304,18	112,13	-0,018195	92765000	67	77,8932
0,809126	0,00250718	84,759	61,01	58,079	0,11665	36226000	117	6,30785
0,913146	3,38E-05	158,64	158,64	147,09	0,026167	44459000	118	13,2278
0,997491	4,19E-07	177,44	109,42	163,9	-0,17664	2303200000	558	27,3978
1	0,00134586	129,39	58,459	82,954	0,27358	2167000000	892	82,9536
0,999849	1,27E-09	126,56	109	126,56	-0,13974	69554000	6	38,2139
0,766597	0,00162987	120,55	68,004	120,55	-0,23241	84684000	876	5,1646
0,999983	0,000346214	158,05	95,642	82,417	-0,025647	123290000	917	47,7243
1	2,10E-11	202,4	174,72	202,4	-0,06744	49861000	83	73,8582
0,999999	0,000142976	118,21	92,162	118,21	-0,16437	276400000	866	61,6777
0,969388	0,000149102	132,08	67,96	128,76	0,31367	22038000	522	15,0061
0,999994	0,000120559	132,59	109,05	83,087	-0,20545	169390000	449	52,2149
0,941618	2,34E-07	165,34	94,165	109,22	-0,15365	30780000	576	11,95
1	2,01E-68	263,4	214,22	263,4	0,1109	94260000	1722	174,006
0,931079	0,000367276	109,1	59,622	109,1	-0,070359	12746000	384	11,3064
1	8,95E-38	268,93	234,75	177,66	0,41754	403510000	65	65,5392
0,9802	5,54E-15	181,41	146,08	155,03	-0,090615	45095000	816	16,9466
1	2,86E-10	185,63	162,71	185,63	-0,34272	150240000	93	71,2777
1	1,85E-06	173,54	162,71	167,55	0,23971	49163000	16	69,8608
0,983436	0,0121611	62,162	32,158	62,162	0,04309	11626000	179	17,7359
1	5,25E-06	179,93	141,06	179,93	0,15586	7700700	301	64,3574

Polito et al. Universal ready-to-use immunotherapeutic approach for the treatment of cancer: expanded and activated polyclonal γδ memory T cells.
Phosphorylation sites

1	3,33E-05	171,36	145,55	70,451	-0,46492	315410000	906	70,4511
1	3,87E-95	341,1	317,25	138,97	0,45594	226500000	381	70,9705
1	4,44E-14	211,26	158,44	103,16	-0,54874	162320000	1258	103,158
0,999995	5,40E-147	398,31	341,37	373,1	-0,029178	816110000	1379	53,2837
1	6,34E-14	264,98	202,98	119,34	-0,28304	1274400000	1351	119,335
0,909079	2,67E-15	152,32	120,24	92,387	0,20256	137180000	1110	11,4031
0,999965	8,56E-183	370,23	370,23	367,19	0,25084	1540300000	171	44,6241
0,999635	0,000155533	123,02	96,482	75,669	-0,41728	39467000	1228	35,0798
0,999957	1,46E-17	228,38	168,22	228,38	-0,13835	1005300000	1229	44,4899
0,999866	0,000110154	158,93	104,32	142,94	-0,13229	178740000	156	38,7342
1	6,80E-06	178,22	143,72	120,87	0,13326	431380000	422	120,872
1	6,21E-09	155,49	121,22	155,49	-0,10407	147730000	376	96,6646
0,907812	5,60E-09	154,33	123,78	93,407	-1,2728	26170000	388	12,7047
0,99577	8,94E-15	205,78	172,63	205,78	-0,042556	18462000	357	24,0623
1	9,19E-07	179,19	133,62	179,19	-0,33094	628810000	452	79,8677
0,99907	2,28E-07	145,88	113,55	145,88	-0,40844	23808000	489	30,3121
0,981471	2,74E-06	131,04	99,628	83,992	-0,36358	79033000	491	17,3791
1	0,000435781	153,25	96,702	153,25	0,21328	16780000	84	153,249
1	1,80E-17	232,15	187,72	232,15	-0,055285	190430000	653	125,725
1	5,77E-38	258,6	221,46	211,39	-0,55168	303570000	524	82,4442
1	1,13E-14	204,17	175,72	180,47	-0,7487	623280000	375	73,8523
0,944251	0,000253869	102,52	77,046	65,895	0,23491	16045000	2296	12,3267
0,957174	1,34E-21	165,61	151,69	165,61	-0,051561	172480000	641	13,6475
0,999958	1,27E-07	199,31	142,88	178,15	-0,29532	113310000	2145	43,7574
0,999825	1,89E-13	232,6	162,55	183	0,029521	484880000	2073	37,5683
0,954576	0,000325496	100,57	40,972	99,442	-0,29346	39182000	1970	16,2356
0,999999	3,77E-07	178,67	70,878	153,95	0,075308	10840000	1679	59,2542
1	4,25E-81	305,55	263,58	182,17	-0,0031944	560310000	2218	182,168
1	0,000338168	104,09	82,861	104,09	0,26751	17613000	723	104,091
0,997389	2,34E-110	318,32	281,68	264,41	0,26214	605430000	444	27,8135
1	3,95E-13	228,78	187,09	228,78	0,6128	20118000	307	91,2353
0,99023	2,69E-94	295,4	264,86	295,4	-0,44893	317570000	304	20,1969
1	1,20E-14	194,19	159,15	194,19	-0,0004786	47781000	488	184,527
0,999991	0,000622792	123,29	38,134	123,29	0,41197	26077000	562	50,6503

0,955649	1,32E-27	245,4	177,23	222,55	-0,13063	48395000	412	13,334
0,999939	1,10E-47	275,66	176,71	263,98	0,52613	115210000	414	42,1359
1	1,28E-37	268,56	228,07	268,56	-0,99439	44632000	421	195,884
0,892391	1,23E-10	213,06	160,36	213,06	0,40332	19162000	411	9,18708
0,922316	2,85E-07	195,72	124,78	195,72	0,064166	15964000	412	10,7455
0,999999	0,00445523	71,085	38,866	71,085	-0,21777	8067400	330	61,727
1	0,00030418	88,755	62,889	88,755	-0,18718	23132000	359	72,0361
1	0,00175635	117,28	9,4945	117,28	0,065964	268710000	283	73,4745
0,999547	6,78E-74	292,44	258,79	292,44	-0,066995	195640000	464	33,5728
1	2,37E-06	188,91	161,78	130,98	-0,29057	2305500000	406	130,982
0,999966	1,43E-33	230,06	209,89	184,8	0,0015564	151590000	93	44,64
1	4,93E-50	268,93	258,77	268,93	0,11861	137260000	602	182,129
0,951138	0,000279559	67,217	48,919	65,213	0,28947	12460000	550	12,8927
1	5,74E-05	155,86	132,01	117,93	0,16217	31961000	474	117,932
1	1,61E-214	395,67	340,13	324,72	0,15943	558710000	871	168,49
0,999968	3,07E-77	251,21	227,44	236,29	0,021666	997930000	87	46,7532
0,814587	2,84E-05	160,38	118,27	141,13	-0,084804	15242000	89	6,44297
0,814201	1,77E-05	164,53	119,96	164,53	0,62753	15585000	90	6,41687
1	7,15E-06	177,83	147,77	138,02	-0,016717	52810000	58	138,02
0,999405	2,48E-66	301,57	266,45	193,06	-0,060856	293770000	49	32,5147
0,983491	8,11E-10	157,42	128,46	157,42	-0,65204	36049000	475	17,7504
0,999998	1,76E-10	202,32	149,08	202,32	-0,075007	112110000	307	57,7483
1	0,000927345	103,26	49,504	103,26	-0,12563	7707000	741	103,255
0,82872	0,000206107	74,776	55,452	74,776	1,2546	17765000	385	6,8475
1	3,51E-247	449,23	412,29	390,17	-0,25593	2226500000	214	137,469
0,999995	7,78E-42	240,2	197,39	212,24	-0,052078	26239000	2408	53,4238
0,798962	0,0248441	46,462	16,795	46,462	-0,40447	6947600	391	6,11033
0,986114	3,05E-05	118,96	107,34	118,96	0,067942	26403000	441	18,5136
0,990203	0,000752403	101,36	68,424	101,36	0,42172	16619000	280	20,0465
0,953259	3,44E-07	131,56	115,16	110,47	0,35021	54834000	346	13,1207
0,999981	4,04E-27	209,16	187,95	209,16	-0,07315	51283000	905	47,3219
1	1,09E-18	206,59	206,59	195,77	0,012837	114320000	850	149,181
1	4,04E-16	150,69	130,6	150,69	0,49531	45858000	1307	92,1284
1	5,45E-15	217,35	197,52	217,35	0,14906	79059000	2260	90,1549

Polito et al. Universal ready-to-use immunotherapeutic approach for the treatment of cancer: expanded and activated polyclonal γδ memory T cells.
Phosphorylation sites

0,999684	1,37E-21	181,89	162,64	181,89	0,1461	48655000	2687	35,008
0,999999	3,78E-12	154,1	142,58	154,1	0,19902	42680000	1234	60,6053
1	1,10E-05	175,01	119,75	175,01	0,285	23777000	1838	175,009
0,960893	1,85E-20	193,39	166,55	173,89	0,31242	20623000	2902	13,9044
0,999999	4,60E-06	145,82	111,86	145,82	0,075247	28571000	2056	59,3232
1	3,14E-06	184,31	149,56	184,1	0,44213	43883000	1152	184,095
0,999997	1,47E-10	248,6	175,27	248,21	0,3141	2365000000	1123	54,99
1	3,40E-08	147,83	106,6	119	0,48807	410230000	1145	118,999
0,999989	9,90E-38	211,87	194,53	211,87	-0,012565	253260000	227	51,4133
1	9,88E-07	194,39	75,12	103,83	0,2809	374220000	1099	103,834
1	2,04E-05	162,23	39,718	103,83	0,2809	765840000	1101	103,834
0,999993	0,00535351	112,01	43,058	112,01	-0,26296	283930000	1061	51,9628
0,998241	0,000927345	103,26	28,335	94,804	0,23842	32055000	76	28,8714
0,99999	0,00188026	86,879	21,344	86,879	0,86388	6393200	668	49,8044
0,999851	4,56E-07	184,51	158	76,548	-0,24053	97083000	739	38,9934
0,999861	1,63E-32	245,48	219,65	245,48	-0,35307	131140000	598	38,5562
0,842427	0,033932	55,064	15,442	53,756	-0,17929	13062000	87	7,29443
0,972682	7,25E-11	211,38	135,24	139,46	1,226	9064000	10	15,5152
0,999999	5,35E-17	226,24	196,47	226,24	0,16175	44264000	539	59,3371
1	1,28E-08	219,97	163,95	217,2	0,16107	449990000	14	82,0692
0,999997	0,00018756	126,1	97,141	111,95	1,4165	330620000	36	55,8275
0,999505	1,18E-10	204,85	192,58	147,87	-0,88606	47358000	28	33,0494
1	6,38E-39	240,13	201,52	214,35	-0,074293	1550400000	36	103,784
0,855738	0,00055342	98,592	67,378	98,592	-0,055258	10561000	55	7,73189
1	0,000966278	91,694	75,496	91,694	-0,026685	11450000	431	91,6939
0,994164	0,0126312	69,352	31,671	69,352	0,48706	15948000	916	22,3132
1	9,64E-07	135,09	98,804	135,09	0,65679	20221000	654	135,088
1	2,13E-111	369,66	335,39	77,776	1,0551	7756500000	175	77,7756
0,996817	8,38E-09	146,03	130,27	141,98	0,16057	78956000	181	27,6317
0,999998	1,15E-108	331,4	296,39	121,27	-0,17792	1587200000	383	58,1753
1	0,000248241	187,5	141,53	42,629	0,064847	623800000	646	42,629
1	1,05E-22	256,96	211,08	209,23	-0,018943	4822200000	510	147,574
0,999881	2,88E-37	260,96	227,69	144,75	-0,028289	180590000	196	39,2285
1	2,69E-06	177,76	151,8	150,35	-0,33407	5952800000	529	150,351

Polito et al. Universal ready-to-use immunotherapeutic approach for the treatment of cancer: expanded and activated polyclonal γδ memory T cells.
Phosphorylation sites

1	1,01E-41	317,65	241,49	305,17	0,18328	392890000	494	98,0453
1	1,23E-09	245,08	121,21	243,82	-0,33188	8708500000	656	86,6787
1	0,00108382	116,71	49,956	73,082	-0,40398	80582000	688	73,082
1	1,57E-111	367,28	329,51	367,28	-0,17086	2668300000	266	104,2
0,999712	6,42E-28	186,39	173,93	129,51	-0,10392	998850000	283	35,5854
0,999992	6,42E-28	186,39	173,93	129,51	-0,10392	1410500000	288	52,469
0,999849	3,82E-31	219,87	156,83	219,87	-0,36744	403650000	220	39,3921
1	0,0337313	56,434	28,352	49,85	-0,048499	108200000	441	49,8503
1	0,0242565	52,112	36,573	52,112	0,32943	10375000	533	52,1123
1	9,47E-257	463,49	417,34	92,211	0,29268	1362500000	571	92,2108
1	0,000721354	145,46	114,89	123,42	0,37814	106340000	447	123,42
1	0,0151291	67,897	15,415	53,493	-0,38727	427250000	96	53,4935
0,991095	3,59E-24	193,4	153,75	193,4	1,3355	122340000	106	20,4958
0,999998	0	519,08	494,21	265,07	-0,014708	19589000000	17	56,4879
1	5,98E-08	188,27	138,06	170,49	-0,12904	60666000	7	170,487
0,999999	5,39E-61	278,95	255,83	278,95	0,044457	559390000	363	61,2477
1	7,46E-06	173,28	147,55	105,39	0,28833	260700000	303	105,391
1	0,000319187	135,23	78,279	131,4	-0,025784	164800000	1102	76,2005
0,99683	0,00158525	87,088	62,706	87,088	0,22181	25897000	155	24,984
0,999219	7,68E-08	162,43	146,2	139,44	0,024064	133260000	1085	31,0701
0,999904	3,71E-06	167,32	89,001	155,09	0,21191	42165000	1185	40,2079
0,969927	8,72E-06	140,46	109,28	140,46	0,26244	43838000	1015	17,0992
1	2,96E-41	236,77	221,72	214,16	-0,048691	334890000	146	96,7555
1	5,00E-41	283,98	249,08	233,92	-0,040615	4419100000	133	95,0448
0,998329	2,76E-05	159,77	159,77	125,97	0,14438	57588000	624	28,1569
1	0,000390263	132,55	72,036	132,55	-0,16283	419470000	494	103,245
1	1,58E-47	270,95	238,82	270,95	0,21663	226980000	170	81,8423
0,999518	5,79E-16	165,48	153,74	165,48	0,35897	53574000	202	33,2014
1	1,46E-06	173,04	108,66	158,56	0,047673	158540000	247	158,563
0,99968	0,00380463	74,944	40,984	74,944	-0,035315	7465300	329	34,9427
0,997523	0,000826149	87,148	61,827	87,148	-0,65554	5037600	75	26,0512
0,999994	1,44E-33	250,47	217,77	165,45	-0,16433	229810000	5	51,9806
0,999953	9,98E-33	236,16	205,59	188,27	-0,044734	154010000	7	43,2916
0,999316	9,13E-33	237,59	188,77	224,86	-0,063563	97123000	12	32,0123

Polito et al. Universal ready-to-use immunotherapeutic approach for the treatment of cancer: expanded and activated polyclonal γδ memory T cells.
Phosphorylation sites

1	6,39E-147	342,48	285,41	217,1	0,082919	11045000000	125	82,4773
0,999786	0,00158696	84,054	50,864	70,942	-0,3727	9967700	194	36,6888
0,999928	8,12E-05	86,625	64,507	86,625	0,86934	8449000	290	41,6716
0,997893	7,03E-11	167,53	127,51	167,53	-0,16379	278230000	2083	28,7802
1	5,33E-12	168,42	131,78	99,531	0,17039	224410000	1069	99,5307
1	1,28E-39	228,71	201,08	221,6	0,094662	484680000	1327	68,7138
1	1,59E-14	178,43	136,78	178,43	0,26118	35512000	1964	65,14
0,999999	3,14E-05	163,99	113,43	162,31	0,41276	161500000	2032	59,3358
0,999999	1,60E-14	191,8	164,65	170,37	0,080365	433770000	2058	63,6798
0,999601	9,89E-33	171,63	154,33	171,63	-0,014914	473120000	620	35,3129
1	2,20E-37	203,02	155,92	203,02	-0,40696	23118000	464	101,656
0,999583	2,41E-14	186,05	157,03	168,68	0,22225	200860000	521	33,8544
0,997188	0,00625509	53,909	38,318	53,909	0,33689	14028000	25	25,4971
0,999929	7,44E-12	170,61	146,65	170,61	-0,17797	12010000	49	41,4981
0,996731	3,54E-12	161,68	106,92	161,68	-0,14478	267960000	1781	25,0454
0,999995	4,80E-25	223,11	183,48	223,11	-0,15465	396740000	1784	52,9435
1	1,74E-99	314,48	292,49	100,95	0,31182	650640000	1730	100,949
0,981825	1,53E-12	156,4	143,97	140,52	0,056622	798250000	1764	17,3258
0,999996	0,000272371	110,08	77,471	110,08	-0,29356	21985000	1014	54,0341
0,99999	0,000159799	124,86	75,356	111,39	0,098411	53920000	2124	50,0128
1	2,71E-83	337,52	288,04	290,37	0,58807	689030000	193	106,376
0,999994	6,83E-45	217,37	191,51	217,37	-0,41465	618810000	157	52,5443
0,979755	0,000917545	92,063	68,212	64,394	0,41189	15556000	26	16,8619
0,977009	0,000842778	93,51	75,809	93,51	0,024791	11313000	28	16,2874
1	0,000345472	158,94	124,14	68,915	-1,2891	79022000	135	68,9149
0,999531	6,70E-15	188,24	188,24	188,24	-0,035747	50439000	445	33,287
1	4,19E-31	216,65	193,23	210,43	-0,15983	98796000	226	196,014
0,999384	0,000311384	109,07	81,424	82,349	-0,3023	9085800	1180	32,2714
0,999827	1,21E-05	90,306	71,576	90,306	0,059493	29369000	1896	37,9156
0,957723	1,94E-108	327,06	224,43	283,75	0,34539	52555000	1258	13,9024
0,999995	9,92E-08	196,7	151,39	190,66	-0,059314	71309000	574	53,2645
1	4,71E-12	176,04	158,79	176,04	-0,084232	64638000	1970	158,961
1	0,00182761	115,33	67,355	115,33	0,45167	59476000	490	115,327
0,986411	7,60E-05	150,01	129,59	150,01	-0,17962	9574300	870	18,6163

Polito et al. Universal ready-to-use immunotherapeutic approach for the treatment of cancer: expanded and activated polyclonal γδ memory T cells.
Phosphorylation sites

0,781392	0,000933187	101,71	44,363	101,71	0,05801	8377200	129	7,24622
0,999984	2,74E-19	216,19	166,71	144,3	-0,36078	74008000	69	49,0664
0,988342	6,80E-17	221,47	148	221,47	0,28102	56232000	514	19,2855
0,983373	1,60E-10	203,87	149,22	193,81	0,24434	140130000	1244	18,9101
0,998303	3,57E-07	158,98	144,72	158,98	0,20688	200980000	1198	27,7277
1	5,12E-70	276,3	255,86	276,3	0,35105	214990000	499	73,0351
1	0,00254004	99,788	49,754	99,788	-0,0020288	102010000	158	72,3013
1	1,11E-177	389,15	340,74	293,13	0,24126	466730000	48	90,9721
0,989655	1,37E-20	196,89	179,61	196,89	0,12775	58418000	54	19,8152
0,998742	1,21E-98	361,22	328,95	308,68	-0,49175	977250000	371	29,0008
1	2,16E-68	252,53	240,83	128,85	0,29588	150770000	381	111,027
1	2,99E-07	195,71	159,29	195,71	-0,1121	92378000	460	90,7756
0,969663	0,0143206	70,028	60,558	70,028	-0,025185	16694000	384	15,0466
0,970694	8,03E-48	237,33	213,54	237,33	-0,10804	125390000	169	15,2021
1	1,17E-11	215,48	179,95	215,48	0,19089	36746000	210	66,8605
0,877635	0,0109744	70,994	57,386	46,702	0,082705	26859000	235	8,70355
0,994491	0,000114135	135,91	102,37	135,91	-0,22528	38752000	327	22,5651
0,903644	1,25E-06	147,24	124	147,24	0,29571	24542000	5	9,72117
0,809272	1,35E-14	180,45	163,3	180,45	0,16846	74528000	103	6,44547
0,805782	1,35E-14	179,46	167,1	102,97	0,064958	56766000	127	6,17927
1	2,21E-15	246,51	231,57	151,54	-0,18765	197540000	60	151,542
0,999974	0,000634747	113,8	63,523	113,8	-0,15579	33079000	187	45,8463
0,999728	0,0388345	65,465	30,265	65,465	0,30517	12814000	446	35,6586
0,996383	1,06E-24	245,61	225,26	185,99	0,1391	236430000	196	25,3503
0,999667	7,08E-05	151,78	93,411	151,78	-0,11438	30747000	60	36,6442
0,999999	7,10E-48	287,04	152,96	287,04	0,088507	235560000	28	60,4486
1	2,61E-27	238,07	194,3	231,58	0,018936	878820000	329	93,028
0,999794	8,45E-19	210,33	184,74	102,58	0,5313	342950000	202	36,8615
0,842453	0,00063028	103,76	84	103,76	-0,36615	12948000	693	7,29726
0,993213	0,000256852	95,414	85,249	92,492	-0,30979	44662000	785	22,7287
0,992521	4,31E-15	194,27	177,02	130,27	-0,30603	165920000	1367	21,2291
0,999884	2,88E-06	129,88	107,07	120,24	0,035741	47487000	466	39,5039
0,95622	5,82E-15	182,82	151,18	182,82	-0,0438	39219000	479	13,3928
1	0,000324577	124,38	93,808	56,551	0,32973	18436000	1740	56,5506

Polito et al. Universal ready-to-use immunotherapeutic approach for the treatment of cancer: expanded and activated polyclonal γδ memory T cells.
Phosphorylation sites

0,909592	0,000127362	109,6	90,819	109,6	-0,52121	5484500	1154	10,0766
0,930258	8,94E-07	173,76	136,56	144,57	-0,071299	92638000	1753	11,2511
0,998792	9,05E-05	128,44	85,596	111,43	0,27325	10024000	148	30,8992
0,999269	0,000235057	105,46	85,354	105,46	-0,24036	9059200	156	31,5734
0,999908	3,84E-25	225,69	197,6	225,69	-0,59836	56369000	328	40,3584
1	0,0284874	59,306	38,353	48,625	-0,23762	12376000	423	48,6251
0,997272	8,93E-06	120,93	94,991	120,93	-0,17447	31675000	76	25,9657
0,999799	5,26E-27	219,01	196,2	219,01	-1,0228	45081000	1395	37,5954
0,998561	0,00181774	73,781	22,336	73,781	0,17692	142920000	49	28,4133
0,998494	3,32E-07	205,97	124,87	86,463	-0,080197	324240000	52	28,2907
0,999947	1,22E-10	162,76	149,03	80,3	0,099564	79060000	149	42,7496
1	9,20E-54	277,1	230,78	277,1	-0,15492	3243500000	347	163,624
1	3,47E-21	241,98	209,22	241,98	0,081706	1091900000	302	68,9211
0,999999	1,14E-48	295,93	265,02	124,09	-1,5544	1559800000	283	61,7174
0,999986	1,43E-25	254,18	225,9	136,6	0,24805	588620000	604	51,068
1	0,000389212	121,61	84,587	111,63	-0,15906	17571000	930	76,0886
0,999348	0,000163963	145,81	84,215	118,88	0,13505	239330000	771	31,8559
0,968102	1,23E-39	232,54	202,1	232,54	-0,0039164	108930000	213	16,5614
1	3,79E-99	307,85	274,31	140,78	0,14897	203330000	416	132,779
1	0,00204539	118,06	83,252	118,06	0,039612	407190000	495	118,059
0,950144	3,54E-10	131,94	115,93	131,94	-0,40172	72123000	123	12,8011
1	0,00327656	79,148	21,29	79,148	-0,20194	5525000	27	79,1482
1	6,58E-05	132,44	94,082	58,079	-0,26713	35668000	111	58,0786
0,995199	3,68E-70	310,46	286,62	176,94	-0,94677	96723000	173	23,1918
1	2,70E-19	211,16	185,84	211,16	1,0788	32308000	34	74,9022
1	1,23E-05	166,6	138,92	150,22	0,21988	26670000	18	67,013
1	7,40E-08	187,37	122,32	187,37	0,35083	23284000	305	82,4128
1	6,64E-16	218,72	200,35	218,72	0,063815	2261000000	96	135,466
0,796694	3,07E-10	162,85	133,95	162,85	-0,15003	34340000	14	5,93143
0,99986	9,09E-05	149,14	129,02	123,29	0,22858	83022000	110	38,5375
1	5,72E-11	212,39	183,05	212,39	-0,028137	1411200000	151	100,562
1	0,0104881	114,89	39,851	114,89	0,30386	224500000	139	79,6939
1	3,25E-06	160,53	104,41	160,53	-0,15118	205070000	128	71,5826
0,999994	0,00119248	113,38	70,313	95,417	0,10605	152890000	190	52,1205

Polito et al. Universal ready-to-use immunotherapeutic approach for the treatment of cancer: expanded and activated polyclonal γδ memory T cells.
Phosphorylation sites

0,999805	0,00165552	82,543	69,139	82,543	0,69045	26447000	181	41,1424
0,999382	0,00826221	61,657	37,467	52,5	0,10991	9277200	185	32,2214
1	0,00424323	102,51	48,959	102,51	0,21873	60828000	173	65,8127
0,999995	3,55E-05	109,03	84,726	95,5	-0,092199	18721000	176	52,8595
0,99999	0,000399243	63,033	57,252	63,033	-0,36498	10878000	254	50,6868
0,997784	5,78E-09	153,86	130,94	143,67	0,10968	45246000	170	27,3248
0,87835	5,56E-11	159,18	119,26	159,18	0,60111	15676000	318	8,58762
1	0,000243538	118,52	73,906	118,52	-0,37562	74778000	544	69,2215
0,991441	5,18E-16	163	145,07	67,43	-0,19848	27060000	43	20,7279
0,999672	1,13E-69	311,12	282,33	311,12	0,0042743	456700000	661	34,876
0,969983	5,05E-09	135,62	123,65	73,649	-0,36246	25982000	190	15,3035
0,953089	0,013441	57,434	42,755	57,434	0,43415	17914000	19	14,4041
1	0,00382352	74,92	62,679	68,846	-0,264	18049000	2	68,8461
1	9,07E-27	235,26	216,82	206,8	-0,10203	685390000	383	103,639
0,95054	7,60E-39	251,85	167,25	135,02	0,21043	288440000	401	13,4593
0,952756	4,88E-21	237,65	144,03	135,02	0,21043	269760000	402	15,632
0,996876	5,10E-15	238,2	185,49	219,43	0,094157	135330000	181	25,0397
1	0,00279894	78,615	54,19	44,391	0,39393	20050000	57	44,3907
1	0,000346693	102,5	79,387	102,5	0,058172	19413000	131	76,0344
1	9,52E-06	175,21	150,44	120,84	0,32583	32173000	185	120,838
1	9,59E-06	141,86	103,86	141,86	-0,028698	31488000	466	97,9644
0,98361	4,84E-37	254,94	232,94	133,26	-0,31172	442030000	261	17,7953
0,995222	5,83E-28	242,01	218,08	124,2	-0,28242	257600000	262	23,1866
0,999114	9,78E-199	415,38	390,91	284,65	-0,12284	2857200000	306	31,9821
0,976468	4,14E-10	161,41	146,02	70,091	-0,97377	138660000	308	19,304
1	3,88E-11	217,2	55,337	195,03	0,082026	437490000	154	195,027
1	2,40E-08	180,06	140,64	78,78	-0,13039	34165000	132	78,7803
0,995916	1,07E-81	316,38	273,35	199,76	-0,18776	940680000	248	24,9777
0,997926	8,91E-19	209,6	161,83	201,3	0,20843	207520000	1650	29,8336
0,995496	3,73E-07	125,68	115,35	124,32	0,04331	93102000	85	23,4442
1	3,51E-17	171,31	153,4	116,47	1,3114	140520000	283	84,2101
1	9,91E-07	117,22	110,56	116,47	1,3114	135340000	284	84,2101
0,99955	6,01E-31	223,89	193,23	196,61	0,27066	328260000	147	35,0141
0,999999	1,05E-15	162,11	128,48	133,1	0,57359	181430000	252	59,2939

Polito et al. Universal ready-to-use immunotherapeutic approach for the treatment of cancer: expanded and activated polyclonal γδ memory T cells.
Phosphorylation sites

1	6,54E-40	250,05	223,33	178,85	0,11629	542950000	39	178,853
0,95188	7,50E-16	211,45	160,31	211,45	0,3583	68590000	244	12,9626
0,999999	1,60E-17	227,63	203,24	176,87	0,60157	278880000	1071	60,6842
0,999661	6,49E-06	124,35	101,7	74,786	-0,17604	178020000	1131	33,3733
1	0,0146425	56,548	10,546	51,346	0,18553	11120000	1170	51,3458
0,99999	0,0132872	69,812	6,3928	69,812	1,1818	40000000	710	50,1531
0,999991	8,89E-59	312,04	202,97	312,04	-0,12863	40671000	674	50,2985
1	1,77E-48	261,46	218,28	167,51	-0,05969	1212200000	279	85,2113
1	4,83E-22	249,2	189,29	216,03	0,80413	19519000	20	216,029
1	0,000624094	94,673	73,833	94,673	-0,56274	56128000	1078	72,6273
0,942927	8,02E-14	134,37	97,094	83,998	0,02441	101560000	196	12,5283
0,873778	7,69E-11	169,37	128,82	140,84	0,50452	23993000	129	8,40265
1	1,40E-74	298,4	274,25	143,54	-0,24309	896610000	244	143,542
1	6,65E-59	269,11	234,93	205,02	0,044385	972580000	256	178,657
1	1,67E-81	327,7	269,38	307,52	0,041693	444270000	380	164,794
0,963688	5,57E-26	156,1	138,23	156,1	1,1122	582090000	173	14,4195
0,829435	0,000143413	132,97	120,92	132,97	-0,71945	8964200	1317	6,86893
1	3,06E-06	182,35	143,47	182,35	-0,069716	63127000	1163	182,348
1	5,92E-110	314,93	299,86	262,26	0,1891	294110000	1260	89,2038
1	1,85E-124	360,06	296,88	267,6	0,035679	159900000	1082	98,5794
0,99782	0,00459241	86,911	42,047	86,911	-0,086883	39723000	401	26,6056
0,999988	0,000107705	135,6	86,248	135,6	0,20582	242880000	1119	49,754
1	3,21E-20	186,92	186,92	147,26	-0,17651	290460000	1195	72,7039
0,999973	0,00223266	107,03	63,068	93,258	-0,032076	173840000	55	45,6986
1	9,42E-67	298,93	269,86	298,93	0,07123	182870000	310	160,482
0,999738	2,34E-05	162,29	142,09	162,29	-0,32579	34574000	272	35,8083
1	1,90E-95	337,71	273,98	337,71	-0,39905	4105500000	104	87,6026
1	4,11E-07	146,81	122,97	146,81	-0,082198	10274000	161	92,5683
1	0,0137713	93,166	13,453	89,401	-0,023771	19765000	823	89,4007
1	0,0017749	88,496	57,883	87,258	-0,21567	5065600	191	64,7663
0,999983	1,27E-06	182,98	164,59	59,248	0,064563	57216000	1016	47,6116
0,999981	4,63E-07	215,14	148,38	215,14	0,13825	210770000	993	47,3031
1	0,000242501	141,37	116,41	58,073	0,11279	180060000	765	58,0727
0,776967	0,00528478	51,265	37,602	51,265	-1,096	11384000	530	6,69774

Polito et al. Universal ready-to-use immunotherapeutic approach for the treatment of cancer: expanded and activated polyclonal γδ memory T cells.
Phosphorylation sites

0,987744	0,000177073	87,16	87,16	85,937	0,3331	61248000	773	19,0629
0,984867	0,00539709	59,57	38,792	59,57	0,16881	13894000	908	18,1919
1	9,03E-10	162,71	126,96	162,71	0,64603	28985000	545	162,709
1	1,73E-11	213,49	179,9	213,49	0,064718	108180000	668	66,5298
1	6,57E-08	198,57	140,74	198,57	-0,076756	96423000	364	74,6243
0,999392	0,000481178	112,13	81,517	112,13	-0,021696	45683000	348	32,1555
0,882533	0,00138043	55,532	32,644	51,844	0,26583	36381000	2327	8,77599
0,9964	0,000885026	60,379	52,667	60,379	-0,1489	20343000	245	24,4236
1	0,0029177	76,172	43,852	76,172	-0,038741	14602000	176	68,3885
0,99999	4,91E-07	179,35	140,35	147,26	0,38467	74124000	389	50,0959
1	3,45E-34	166,51	155,39	166,51	1,1227	511620000	5	130,723
1	1,08E-21	195,77	167,45	195,77	0,1016	163390000	4034	97,1992
1	1,76E-07	155,76	133,22	155,76	-0,31002	54020000	102	105,707
1	0,00085773	79,568	61,768	77,681	-0,19562	52940000	469	75,7924
0,996232	4,21E-12	209,53	167,1	136,88	0,3467	260610000	478	24,5999
0,999999	0,000453967	133,83	76,917	133,83	0,33505	189480000	203	58,8925
1	2,38E-69	312,78	269,97	196,22	-0,35954	1218000000	288	90,7584
1	2,97E-10	159,8	133,85	57,945	0,23201	173230000	218	57,9454
0,999999	0,000250973	113,7	9,8942	113,7	0,25478	242050000	11	59,9439
1	0,00431303	76,679	62,522	68,224	-0,03638	2433200	197	68,2235
0,994252	8,97E-10	163,91	145,77	161,75	0,64378	106550000	238	25,3898
1	0,0154675	59,975	20,344	58,04	0,34571	74187000	3029	58,0403
0,926817	5,77E-06	130,19	110,37	130,19	0,0095041	10972000	2954	11,0347
0,974763	2,57E-27	238,32	169,29	238,32	0,094429	12831000	2964	15,8686
0,999587	6,28E-05	173,24	77,593	173,24	0,54113	67179000	3019	33,842
0,919001	0,00133315	107,09	67,307	62,528	0,73972	37790000	3021	10,5496
0,995768	0,0396847	54,539	21,162	54,539	0,58619	12662000	146	23,7422
0,997593	9,70E-05	161,76	82,291	161,76	0,10546	13131000	248	26,1741
1	6,95E-19	264,97	227,73	238,55	0,13583	940890000	176	87,9902
0,980534	8,41E-39	212,94	205,32	212,94	-0,27125	365280000	362	17,0218
0,965962	0,000356424	87,554	65,357	54,511	-0,21774	13934000	343	15,147
0,999994	5,25E-25	209,66	183,12	174,2	-0,23834	214060000	307	54,5842
0,999999	7,09E-178	399,9	369,96	383,92	-0,22778	535500000	309	59,6275
0,999501	5,40E-05	113,01	87,284	97,836	0,32368	17449000	15	33,3161

Polito et al. Universal ready-to-use immunotherapeutic approach for the treatment of cancer: expanded and activated polyclonal γδ memory T cells.
Phosphorylation sites

0,998107	0,000280263	122,64	102,31	122,64	-0,30587	14227000	48	27,2213
0,955699	5,59E-05	112,5	100,98	103,39	0,21651	28973000	182	15,8681
1	1,26E-07	186,29	120,1	95,631	-0,23604	145000000	150	95,6312
1	1,75E-68	268,45	239,32	262,97	-0,15057	2186700000	161	127,999
1	4,22E-142	361,38	323,69	121,24	-1,4837	7233600000	170	121,239
1	1,05E-06	157,03	131,82	94,463	0,47169	31593000	2	94,4634
0,99132	1,21E-08	176,56	158,22	176,56	-0,1255	60651000	99	20,579
0,999992	5,01E-20	225,7	157,71	225,7	1,0391	51164000	909	51,114
0,999118	0,0213425	56,205	34,833	56,205	0,22537	5806800	929	34,1495
1	1,06E-05	85	74,852	85	-0,56518	23391000	7	84,2409
0,862815	6,61E-06	151,79	111,94	99,215	-0,46311	17097000	162	8,56213
0,834886	1,17E-06	169,89	140,12	168,98	-0,078948	69968000	163	8,30927
0,94418	5,05E-06	121,26	106,32	121,26	-0,28418	14606000	162	12,5121
0,999765	2,70E-05	122,96	86,014	122,96	1,0881	9892500	398	36,3798
0,999863	0,0036524	66,285	49,259	66,285	-0,32399	9874100	303	38,6361
0,995574	2,96E-63	282,37	256,08	251,12	-0,36376	1218100000	542	23,5203
0,786974	4,61E-15	177,09	153,87	177,09	0,01017	224740000	543	5,67528
0,998772	2,56E-14	174,19	150,77	151,41	0,070675	170400000	291	29,1205
1	2,53E-07	188,44	120,16	174,87	0,15393	86120000	222	68,275
0,99996	6,46E-06	143,14	120,93	143,14	-0,37379	45777000	1081	44,0725
0,922014	0,000120418	110,77	84,234	89,624	0,13885	13841000	282	13,8597
0,999497	0,0156316	61,593	11,399	55,718	0,25136	8976000	202	32,9781
1	0,000387712	138,25	93,277	109,42	0,069359	246070000	78	81,406
0,999951	3,91E-06	176,56	119,38	175,15	0,02469	26159000	27	43,0922
0,999999	0,000759831	114,5	85,023	114,5	0,19221	25137000	281	58,9468
1	7,16E-07	136,62	112,34	46,606	-0,12298	94418000	327	46,6059
1	0,00506781	114,4	33,614	51,436	-0,16028	22844000	10	51,4359
1	0,0078332	169,09	55,588	143,45	0,085076	578500000	251	143,451
1	1,91E-82	261,79	226	45,347	0,15402	344980000	1355	45,3467
0,999999	1,23E-05	158,47	125,84	147,3	-0,34179	108730000	1099	60,18
0,997495	8,86E-63	273,87	226,79	273,87	-0,040114	70449000	1364	26,1308
0,9966	5,57E-124	337,66	312,15	337,66	0,06152	691960000	1331	26,5008
0,999927	0,000648156	127,92	100,44	110,84	-0,14344	29130000	950	41,4924
0,99501	1,06E-05	106,94	92,383	106,94	0,55261	14474000	675	26,3066

0,994269	1,46E-06	131,2	108,17	131,2	-0,18857	24892000	1308	22,4477
1	3,43E-20	192,58	170,72	192,58	-0,10671	45280000	804	97,9106
1	6,03E-11	211,83	183,13	168,64	-0,42642	99060000	4516	168,64
0,891899	8,44E-08	104,03	70,341	104,03	0,73545	11034000	3293	9,17028
0,988983	7,18E-06	113,08	79,135	113,08	-0,14821	56916000	3326	19,5313
0,998376	4,72E-48	271,19	224,23	268,2	-0,98915	80459000	1371	27,9585
0,999322	0,000775431	84,94	70,9	63,76	0,21236	21048000	3922	31,6924
0,999743	4,75E-06	180,48	131,18	180,48	-0,10715	38025000	440	35,9068
1	2,96E-168	414,61	373,39	300,56	-0,00078002	542990000	331	183,684
1	0,00320814	79,427	61,959	79,427	0,084249	129890000	121	65,2698
0,999399	0,00231608	80,318	55,08	61,11	-1,2024	37654000	52	32,207
0,999971	0,00104375	90,614	66,864	75,479	-0,24743	30379000	94	45,7351
0,999836	1,42E-26	165,34	151,17	165,34	1,1751	64846000	50	37,8397
0,998424	0,0016107	93,269	74,974	93,269	-0,61976	22341000	579	28,0196
0,999981	0,000183062	113,59	94,375	113,59	0,35059	5122900	490	47,3012
0,999974	1,43E-142	379,59	322,64	314,1	0,51472	706290000	67	45,8866
0,982051	2,24E-05	109,84	89,732	109,84	0,32193	11501000	1186	17,381
0,96841	0,000662121	96,604	73,918	96,604	0,0071203	15594000	1178	17,4029
1	0,00290516	77,776	68,288	77,776	-0,11504	39269000	439	74,738
0,921322	8,60E-05	146,27	132,85	117,48	-0,043574	44034000	416	10,8815
0,999988	2,84E-89	307,23	278,6	246,42	-0,060403	960160000	721	49,3274
1	2,84E-89	307,23	278,6	307,23	0,27713	885930000	724	100,128
0,98937	4,39E-55	225,45	211,78	117,32	0,5227	284510000	654	22,4918
0,998094	2,71E-26	172,67	154,11	90,515	-0,078431	174370000	656	30,985
0,997915	6,17E-48	280,5	239,75	280,5	0,020614	336320000	701	30,5326
0,988249	4,74E-18	190,44	159,05	190,44	-0,59134	109250000	702	19,2249
0,999869	0,000514597	64,701	46,794	64,701	0,96701	6032300	1768	39,4259
0,775817	7,77E-06	162,94	126,53	162,94	0,12249	44490000	382	6,34396
1	1,72E-59	293,42	259,84	287,03	0,048034	271760000	1286	123,688
0,999999	3,53E-59	287,03	238,57	287,03	0,048034	345790000	1289	62,9592
0,984788	2,13E-07	156,5	141,81	156,5	0,25074	9223600	1235	18,1116
0,973419	4,16E-32	212,7	212,7	212,7	0,040166	490600000	1226	15,7528
0,947282	4,16E-32	179,5	166,05	179,5	0,16843	598420000	1230	11,8604
0,999997	2,10E-24	273,02	227,11	273,02	0,20551	184160000	1251	55,8209

Polito et al. Universal ready-to-use immunotherapeutic approach for the treatment of cancer: expanded and activated polyclonal γδ memory T cells.
Phosphorylation sites

0,999607	4,52E-06	180,84	145,25	180,84	0,38876	27774000	12	34,0528
0,921539	0,00146218	86,114	55,55	86,114	-0,0059277	54396000	275	10,6986
0,999393	2,68E-39	218,71	205,8	160,26	-0,23289	129240000	573	32,4941
0,977234	7,41E-07	135,75	120,15	118,08	0,15449	131010000	637	16,3268
0,999997	0,00137131	146,79	65,595	146,79	0,15264	35479000	513	56,0766
0,999997	5,59E-05	134,98	120,2	134,98	-0,15197	21064000	303	56,0742
1	1,30E-24	204,94	196,21	161,08	-0,1482	147000000	597	70
0,999869	4,25E-25	223,07	196,73	162,11	-0,00195	176160000	1002	38,9641
1	0,000775112	193,62	75,388	79,986	0,47827	310130000	590	79,9855
0,998981	0,000619188	123,51	64,731	123,51	0,18798	17211000	912	29,9157
1	1,50E-21	241,42	194,69	230,36	-0,043112	784910000	119	206,423
0,999854	0,000778735	109,44	59,335	109,44	0,65643	11077000	421	38,3576
0,999899	0,00363142	76,255	32,575	76,255	0,98306	7786200	11	39,9517
0,996596	3,03E-14	179,95	147,56	179,95	-0,030797	251420000	446	24,6656
0,87224	1,88E-05	86,805	78,311	83,09	0,38139	269170000	447	8,34241
0,999988	0,000554037	106,4	71,679	106,4	0,041904	79116000	729	50,751
0,998688	6,24E-10	162,39	147,92	110,57	0,0071631	277600000	513	29,8887
0,972839	3,57E-20	198,77	177,98	198,77	0,15567	224280000	521	15,5454
1	0,00166346	75,682	59,188	75,682	-0,090135	30264000	589	68,4456
1	2,13E-20	221,59	200,53	221,59	0,12538	103310000	1857	121,662
1	0,00023007	98,408	76,535	98,408	0,38332	12598000	1359	98,4084
0,999999	2,74E-06	148,44	84,048	90,367	0,31057	29423000	369	58,9357
0,992986	5,09E-05	151,66	132,69	151,66	0,1115	36526000	420	21,5096
0,999969	3,15E-83	265,7	241,81	99,846	0,12565	1240700000	126	45,2156
0,998684	2,52E-08	151,06	124,91	99,021	-0,31594	132720000	21	29,0425
0,999998	0,00298771	83,998	46,187	83,998	0,1575	6836100	2295	56,861
0,870851	5,37E-08	149,36	124,95	149,36	0,66227	79833000	1811	8,28854
0,901319	1,54E-15	180,86	157,18	155,49	0,09564	78516000	1813	9,60647
1	0,000245613	112,13	78,897	112,13	0,14288	199400000	768	78,8966
0,92442	5,12E-18	178,78	159,66	132,73	1,1697	103110000	2288	10,9107
0,99997	0,000712003	117,71	69,746	117,71	0,27677	47009000	664	45,4624
1	0,00194425	130,56	74,052	130,56	-0,049601	84472000	1405	130,562
0,99987	6,52E-06	163,46	115,42	143,97	-0,048729	160380000	1082	39,7528
1	0,00213603	113,7	97,817	113,7	0,14678	26454000	734	78,2602

0,999999	1,96E-12	174,78	142,96	174,78	-0,45369	45942000	919	61,4946
0,97525	2,52E-17	223,18	204,94	107,99	-0,035853	139420000	839	15,9553
1	1,24E-82	343,34	300,71	40,724	-0,12715	70139000	675	40,7239
0,997145	0,00294428	62,589	38,302	62,589	-0,19722	22229000	541	27,0516
1	1,08E-09	174,23	151,31	155,1	0,63911	204930000	11	126,291
0,976687	2,13E-60	239,31	225,36	160,53	-0,24158	607370000	701	16,7155
0,964466	2,80E-29	252,36	209,17	252,36	0,18568	20072000	118	14,3365
1	1,08E-26	201,4	169,56	201,4	0,021661	58345000	142	148,009
0,8802	1,48E-05	89,982	78,063	89,982	-0,13118	14649000	1703	8,66491
0,959904	0,000188607	146	126,93	112,3	-0,0056914	39871000	1245	13,7916
0,961704	1,05E-16	151,13	136,84	128,39	-0,23723	152760000	1206	14,9261
0,99994	0,000108968	135,04	86,602	135,04	0,76858	13592000	1806	42,2501
0,999995	1,24E-24	205,68	172,87	178,32	-0,24597	67856000	1786	54,1215
1	2,20E-11	216,77	161,36	182,71	0,16536	291130000	1282	182,714
0,999996	4,61E-06	158,06	116,52	121,02	-0,24538	138250000	216	53,6362
0,999978	1,47E-10	205,09	187,46	185,25	0,50929	164090000	1090	46,624
0,810191	9,39E-06	107,75	93,873	107,75	0,73765	14263000	721	8,94895
0,999453	9,39E-11	165,74	144,18	165,74	0,014128	35386000	106	35,6311
1	9,37E-176	422,55	324,14	422,55	0,046867	703660000	235	67,1677
1	1,75E-68	268,45	239,32	168,67	-0,048764	1707700000	216	108,698
1	7,54E-05	153,51	122,01	47,894	0,48654	156990000	6	47,8938
1	0,00650431	66,273	41,061	66,273	-0,21843	6925100	248	66,2734
0,999999	2,07E-28	186,23	171,41	123,52	0,22248	521940000	39	60,9181
1	6,33E-67	295,03	271,2	295,03	-0,21504	468310000	41	249,77
0,895764	0,000125457	136,92	106,35	60,434	-0,84838	24880000	87	9,34176
0,760558	0,00351557	69,345	47,714	69,345	0,050676	114530000	791	6,05451
1	0,00264598	89,903	55,01	80,684	-0,1023	25297000	333	80,6835
1	0,00194151	44,75	35,122	44,75	-0,54936	11854000	11	44,7499
0,999945	4,90E-05	152,89	128,11	133,9	-0,049313	83776000	548	42,5946
0,999977	0,000179293	117,37	83,536	117,37	0,19053	136520000	510	47,2783
0,999949	1,20E-11	217,68	164,74	217,68	0,068626	61060000	72	42,9589
1	0,000105123	127,56	105,41	112,5	-0,28128	82718000	205	64,4075
0,999981	5,77E-08	186,74	186,74	186,24	0,16427	313630000	137	48,4613
0,842367	0,00140207	84,658	43,375	84,658	-0,074502	17523000	138	7,27935

Polito et al. Universal ready-to-use immunotherapeutic approach for the treatment of cancer: expanded and activated polyclonal γδ memory T cells.
Phosphorylation sites

1	4,37E-07	220,81	148,57	220,81	0,2368	137910000	237	118,386
0,999997	7,56E-07	180,62	111,25	139,39	0,1048	87984000	211	55,9377
1	5,66E-18	175,97	151,05	175,97	0,51825	142600000	461	63,0965
1	0,00534379	96,009	12,553	91,516	-0,057425	154010000	100	91,5161
1	0,000344163	107,17	78,401	107,17	0,96925	11121000	250	107,166
0,994258	0,000609323	116,96	52,921	116,96	0,40287	16599000	619	24,5041
1	5,16E-82	327,53	294,67	247,5	-0,40252	275620000	224	181,212
1	1,25E-10	212,95	156,02	62,445	-0,35793	605830000	125	62,4454
0,90449	1,56E-05	91,079	71,701	91,079	-0,5174	17891000	191	9,76356
0,829904	3,15E-09	184,76	124,36	184,76	-0,31018	28374000	144	6,88335
1	2,05E-24	205,06	175,46	201,6	-0,26612	69190000	62	93,5955
0,875099	3,57E-23	172,39	154,67	155,34	-0,59628	124070000	112	8,45787
1	0,000102696	115,76	88,981	69,672	-0,04262	295730000	96	69,6716
1	0,000196464	118,21	26,944	54,784	0,078261	118800000	36	54,7838
1	2,18E-124	352,85	322,44	319,47	0,56205	1907800000	103	68,6376
0,998236	3,51E-07	142,78	125,29	102,39	-0,97669	78806000	92	27,8851
1	0,00234022	72,321	56,431	72,321	0,24039	8946800	124	65,9143
1	2,99E-32	236,78	202,84	236,78	-0,10991	44923000	458	70,4306
1	0,00236695	91,858	46,78	91,307	0,38678	41472000	507	91,3069
0,999995	0,000437916	134,84	105,1	108,98	-0,16412	31272000	432	53,428
1	1,08E-14	180,62	121,79	180,62	0,25301	29190000	205	116,102
1	0,000405423	99,927	87,366	55,755	-0,19035	32570000	442	55,7548
0,992431	1,44E-05	169,79	133,41	169,79	0,14007	8431200	72	21,1768
0,966819	0,00299687	83,948	18,723	71,692	-0,14676	5844700	10	14,6446
0,999991	0,00025411	113,69	94,964	113,69	-0,19248	78304000	186	52,2714
1	5,29E-07	191,69	125,43	147,73	-0,37738	11526000000	199	73,4952
0,999972	0,00341945	93,477	26,27	71,342	-0,17972	412110000	227	46,4483
0,996434	1,22E-11	209,41	144,86	203,11	-0,11925	18060000	130	24,5328
1	4,47E-33	261,61	179,98	261,61	0,31922	79496000	132	162,449
1	7,19E-06	142,1	115,66	136,17	-0,32958	40140000	26	83,4755
1	3,33E-14	203,99	177,32	203,99	0,04781	68304000	35	115,393
1	8,38E-42	254,62	216,28	254,62	0,59438	104880000	219	164,195
1	2,45E-95	331,02	291,39	331,02	-0,092513	357550000	523	151,212
1	7,28E-92	273,19	255,86	273,19	-0,021438	302400000	560	87,6584

Polito et al. Universal ready-to-use immunotherapeutic approach for the treatment of cancer: expanded and activated polyclonal γδ memory T cells.
Phosphorylation sites

1	0,000296676	132,51	119,55	132,51	0,05154	65509000	146	84,2321
1	6,90E-07	169,58	169,58	138,76	0,092585	412220000	308	138,763
0,99988	6,87E-22	187,38	162,73	173,92	-0,4364	878030000	424	39,4623
0,839655	4,94E-07	192,4	152,82	192,4	0,17425	34050000	119	7,19578
0,832106	0,000307495	106,79	53,772	90,22	-0,28754	25862000	120	7,86091
0,995456	0,0260232	72,2	33,62	72,2	-0,60856	59070000	545	23,4063
1	5,82E-95	322,43	300,36	190,8	-0,25661	7670000000	25	155,275
0,956126	3,27E-06	120,4	75,474	120,4	0,24986	36557000	1318	15,5626
1	0,0102182	65,252	6,5913	65,252	0,069216	4683400	17	65,2521
0,985735	0,00581944	68,787	7,8149	68,787	0,2736	5368500	1860	19,4284
1	0,000103278	144,27	111,64	144,27	0,10051	29106000	1467	98,4758
1	0,000256695	116,19	100,93	116,19	0,10864	40433000	1370	110,407
0,999921	0,000893537	105,52	90,316	96,143	0,084999	35650000	2520	41,0792
0,975923	4,72E-06	104,28	85,682	78,234	-0,81186	17660000	1822	16,1926
1	0,00289905	88,282	45,493	88,282	-0,10307	12190000	13	88,2818
0,982966	1,46E-05	90,884	61,766	61,577	0,089553	22035000	28	17,6197
0,999999	5,57E-111	357,71	331,04	357,71	-0,16826	37155000	103	59,9948
0,968812	6,29E-05	134,04	112,81	98,105	-0,20144	94053000	58	14,9227
0,966445	9,24E-10	160,55	134,43	160,55	0,16067	61617000	128	14,6334
1	2,43E-21	234,36	210,61	234,36	0,18373	36558000	193	234,36
1	0,0191308	76,478	27,036	76,478	0,28126	34452000	420	76,4776
0,998748	5,83E-25	220,34	185,3	191,95	0,31277	75839000	281	29,6241
0,999222	3,31E-60	301,57	278,69	225,21	0,22627	174770000	452	31,7593
0,993842	0,000155667	131,06	107,83	104,17	-0,66244	47942000	427	22,6093
1	2,81E-06	132,59	106,72	132,59	0,12299	6613300	2	69,2509
1	1,38E-14	191,78	164,14	175,59	-0,89663	22794000	9	96,7449
0,991209	0,0052812	67,519	54,542	62,732	-0,12024	21463000	52	20,522
1	0,0002086	150,22	124,74	150,22	-0,20095	19537000	60	109,223
1	6,82E-06	127,5	107,95	127,5	0,81311	35188000	311	74,1994
0,997676	8,38E-26	217,26	161,38	217,26	0,47372	99605000	335	27,0339
0,999998	5,83E-10	159,77	134,99	157,22	-0,52724	79836000	343	56,4468
1	0,00196666	131,11	86,024	115,71	0,13592	41829000	127	108,59
0,999044	4,25E-15	240,65	184,29	219,43	-0,56736	213340000	31	30,1916
0,999997	0,00380037	74,962	50,108	74,962	0,053798	11717000	135	55,7188

Polito et al. Universal ready-to-use immunotherapeutic approach for the treatment of cancer: expanded and activated polyclonal γδ memory T cells.
Phosphorylation sites

0,999849	1,24E-230	403,68	360,97	88,634	0,01267	11510000000	150	38,2594
1	0,000338932	103,13	89,995	96,208	0,0090214	79983000	582	96,2077
0,879113	5,12E-33	208,37	187,24	165,04	0,045088	182990000	403	8,61678
1	5,19E-43	234,74	216,02	234,74	-0,61457	288480000	417	116,799
0,999796	3,87E-07	157,75	118,09	151,54	0,91846	7965600	77	36,9785
0,97885	0,00148085	94,454	74,271	86,772	-0,15386	24849000	180	16,6542
0,999998	2,23E-60	272,45	242,73	155,97	0,37373	214150000	201	57,2624
0,999894	0,000111091	60,659	54,364	59,282	-0,37615	27405000	86	40,4974
0,999752	4,69E-05	160,48	129,61	160,48	-0,14379	36373000	192	36,0629
1	0,00196051	131,5	42,685	59,153	0,0034955	119560000	481	59,1529
1	0,00062126	123,38	39,885	68,893	0,21887	162130000	575	68,8931
1	0,00209887	117,84	75,55	43,282	0,092269	619960000	528	43,2819
0,815566	1,26E-09	133,16	103,82	94,632	0,36319	10601000	467	6,45618
0,823403	1,41E-10	194,05	164,14	186,56	-0,1259	30169000	468	6,68628
1	0,00209357	119,75	72,665	40,728	-0,039463	78165000	452	40,7277
1	0,00535791	70,908	23,334	52,549	-0,24892	77790000	513	52,549
1	0,00182865	136,57	65,116	120,99	0,48124	74027000	432	120,989
1	7,38E-06	102,65	88,296	70,249	-0,45952	213050000	919	70,2491
0,95124	0,000302018	86,467	67,687	52,329	-0,13831	47459000	842	14,0958
0,997496	8,18E-05	147,02	124,28	147,02	0,098209	38777000	687	26,0022
0,999997	8,90E-06	166,11	114,66	166,11	0,0043053	1947300000	384	55,1753
1	0,00071323	109,83	83,723	109,83	-0,1479	486690000	388	88,9947
0,999531	1,18E-123	340,53	271,59	243,18	-0,011453	2726100000	410	33,2888
0,999136	0,00192078	98,737	71,408	98,737	-0,17227	15275000	136	30,6299
0,999991	0,000176452	85,619	58,59	85,619	0,04999	13027000	29	50,4156
1	7,34E-07	190,66	149,58	57,106	-0,46678	136780000	86	57,1063
1	0,000192121	153,2	89,26	112,42	0,012382	1953900000	392	82,2407
0,999972	0,00341945	93,477	26,27	71,342	-0,17972	412110000	310	46,4483
1	0,000617693	108,72	87,321	108,72	-0,35832	45039000	142	108,716
0,999795	4,49E-16	201,31	169,15	201,31	-0,15535	166710000	717	37,3994
1	2,01E-182	367,78	339,66	256	-0,36931	761980000	1992	146,972
0,999722	9,47E-16	179,54	168,7	168,69	0,36141	162750000	1405	35,5617
1	0,00364009	127,02	21,886	120,46	0,042787	8555100	1033	120,457
0,997171	2,13E-91	320,27	284,07	231,42	-0,10914	188510000	1331	25,7934

Polito et al. Universal ready-to-use immunotherapeutic approach for the treatment of cancer: expanded and activated polyclonal γδ memory T cells.
Phosphorylation sites

0,992402	1,85E-12	222,07	152,73	222,07	0,17728	783320000	1354	22,2041
0,947632	2,76E-05	173,25	110,85	65,179	0,075698	217120000	1356	13,3382
0,963041	0,000251038	68,243	46,011	68,129	-0,31882	24252000	2141	15,8636
0,99983	1,45E-34	252,8	220,91	52,359	0,076641	145850000	1115	37,7054
1	5,24E-82	333,41	285,41	201,12	0,085255	1114200000	1290	63,889
1	2,86E-05	122,08	108,96	122,08	-0,1034	9947800	1261	68,0987
1	4,41E-14	204,37	172,98	163,56	0,39263	209110000	1267	86,7934
1	0,00526885	71,03	47,711	47,302	-0,25948	8457500	637	47,3018
0,999988	0,000293056	112,44	82,502	112,44	-0,068413	22068000	116	49,1541
0,998648	2,77E-17	229,63	146,72	124,35	-0,029544	940110000	124	28,6848
1	0,000144678	119,65	104,96	119,65	0,15443	9657200	224	119,65
1	0,00074068	115,78	76,477	99,568	-0,16412	36876000	315	99,5681
0,845685	3,11E-05	74,274	61,597	74,274	-0,041504	25387000	448	7,38807
0,878771	2,36E-06	132,35	120,83	132,35	-0,15298	43077000	351	8,82255
0,999613	4,16E-06	123,43	106,35	118,37	0,044047	46242000	65	34,1191
0,998849	0,000820752	73,252	47,662	73,252	0,55177	16043000	589	29,3914
1	7,46E-07	185,95	121,72	185,95	0,19813	85159000	100	73,8627
0,771416	1,03E-05	102,28	75,308	100,45	0,11532	20256000	426	7,51911
0,999999	0,00010014	138,94	138,94	138,94	-0,1082	16289000	90	62,2808
0,999913	7,69E-33	209,96	188,39	207,17	-0,46336	573630000	1001	40,5993
1	4,15E-222	429,16	387,07	345,56	-0,095075	328270000	161	133,246
0,999556	1,77E-21	237,65	200,47	237,65	-0,15615	29782000	5	33,5204
1	7,24E-42	256,67	233,75	256,67	-0,11004	221950000	110	71,5282
1	4,70E-15	216,99	189,66	216,99	0,11052	27709000	43	63,6085
0,997851	1,28E-08	170,49	126,67	170,49	0,48788	11267000	69	27,1095
0,825048	3,49E-99	308,54	260,78	243,87	-0,35051	1071300000	24	6,73559
0,825077	4,84E-99	303,94	255,06	253,49	-0,50837	374170000	25	6,71982
0,989653	1,69E-05	90,22	70,124	90,22	0,30936	14334000	512	19,807
0,902532	0,000185754	119,74	92,313	119,74	0,032834	9972800	323	9,66657
1	8,92E-39	282,63	252,57	282,63	0,065634	2207700000	227	149,783
1	1,51E-05	155,99	83,141	155,99	-0,54636	29097000	14	69,6444
0,99345	0,000999993	76,82	41,347	76,82	-0,5512	9443300	415	21,8093
0,989954	3,76E-08	172,37	172,37	79,652	0,61043	61661000	291	19,9428
1	7,21E-140	359,91	278,48	283,45	0,032575	9757900000	181	79,7522

Polito et al. Universal ready-to-use immunotherapeutic approach for the treatment of cancer: expanded and activated polyclonal γδ memory T cells.
Phosphorylation sites

1	9,26E-125	351,41	335,89	347,14	-0,38309	926720000	57	76,645
1	2,14E-37	263,22	239,39	263,22	-0,09596	994910000	662	104,047
0,995945	0,00198347	76,82	51,856	76,82	-0,1994	20068000	667	24,2974
1	0,00711683	85,154	16,497	48,561	-0,052846	24751000	660	48,5609
1	7,97E-15	200,16	176,93	59,116	-0,42525	62015000	315	59,1163
0,894854	1,71E-39	231,21	207,18	231,21	-0,47848	49012000	221	9,2996
0,999994	0,00181404	87,895	71,692	87,895	0,44748	14045000	211	52,2767
1	0,00206709	111,06	80,395	111,06	0,011934	86607000	258	88,1344
1	7,06E-10	159,04	126,36	94,781	-0,18364	77874000	18	94,781
0,999977	3,31E-11	195,92	173,04	195,92	-0,16683	8305100	313	46,3539
0,995717	0,00115274	98,04	17,875	98,04	-0,066848	19368000	529	23,664
1	4,05E-05	165,24	138,89	159,22	0,030493	264500000	590	122,272
0,995717	0,00111634	98,04	17,875	98,04	-0,066848	35683000	529	23,664
0,999995	0,00206712	105,52	56,08	105,52	0,42309	28036000	338	52,9452
0,969781	5,03E-06	125,73	101,5	125,73	0,3365	41286000	19	15,0641
1	0,00014073	156,6	86,879	156,6	0,55764	19552000	80	71,7078
1	5,10E-05	119,96	92,163	112,67	-0,17163	28333000	327	65,6283
1	1,89E-74	301,03	278,81	154,19	-0,09538	2722100000	265	124,584
1	2,53E-128	377,05	331,69	154,19	-0,09538	2659300000	267	97,4682
0,999994	1,87E-37	264,03	232,32	264,03	-0,025552	124180000	272	52,2719
0,999108	4,00E-56	249,1	249,1	232,53	0,37199	1721000000	300	31,7389
1	5,62E-07	142,01	94,474	43,024	0,43262	33777000	56	43,0241
1	1,10E-66	288,75	264,34	43,024	0,43262	667830000	60	43,0241
0,979555	9,69E-08	160,47	144,29	75,479	-0,17063	32762000	687	16,8045
0,999073	0,000160373	154,28	134,49	154,28	0,22912	12142000	276	30,3331
1	1,84E-08	116,35	98,256	46,898	-0,54958	52720000	593	46,8984
0,991206	0,000137171	65,943	52,226	65,943	-0,080082	12360000	300	20,8033
0,999995	6,82E-20	188,04	165,99	113,88	0,16934	175960000	519	52,7723
0,999995	1,31E-157	372,59	326,19	113,88	0,16934	5858200000	520	52,7723
1	4,70E-12	180,85	149,37	58,024	-0,34097	467320000	569	58,0243
1	2,55E-25	234,22	234,22	58,024	-0,34097	1573700000	570	58,0243
1	2,55E-25	234,22	234,22	58,024	-0,34097	2631400000	563	58,0243
0,999278	6,16E-25	222,06	196,5	163,18	0,1951	365620000	537	31,4138
0,999368	7,88E-17	221,62	174,26	208,39	0,069504	77799000	314	31,9897

0,999999	9,37E-05	86,258	69,316	82,593	0,2819	78370000	60	60,6061
0,9991	1,84E-05	83,499	70,873	83,499	-0,5796	24848000	197	30,4984
0,993674	0,00743728	53,779	42,339	53,779	-0,15869	7341100	79	21,9609
0,999996	4,03E-125	368,64	323,43	240,61	0,0054027	886780000	696	54,4453
0,942612	7,43E-32	234,47	204,26	182,76	0,1258	184180000	772	12,1551
0,999997	3,31E-74	289,45	272,08	211,06	-0,15912	831070000	363	54,8326
0,999972	8,07E-40	248,94	222,46	176,97	-0,12278	252950000	1184	45,5525
1	0,000123116	137,62	79,351	137,62	0,83948	95719000	1751	125,128
0,997354	1,12E-08	200	178,26	200	-0,16719	55222000	382	25,7621
0,932617	0,000144505	133,96	118,82	85,496	-0,39308	266780000	1600	11,7358
0,999023	2,57E-49	260,97	229,17	233,65	0,023321	1002200000	1755	30,0985
0,961827	4,23E-27	208,69	185,09	208,69	0,3561	84352000	660	14,0135
0,801406	8,20E-17	221,03	182,01	141,75	0,20563	241640000	326	8,79921
0,82726	2,99E-17	230,55	214,92	174,59	0,18356	321470000	327	6,9785
0,820326	4,05E-07	142,32	108,61	142,32	0,05228	41214000	278	6,69777
0,999147	4,45E-49	296,27	234,71	246,96	-0,016225	1676900000	415	30,6884
1	5,89E-05	136,21	98,656	86,467	0,22591	99900000	2013	86,4671
0,999969	6,71E-06	170,11	139,93	170,11	0,83969	43601000	440	45,0819
0,999948	0,00229191	92,856	21,681	77,072	-0,11873	50126000	45	42,8189
1	0,000256844	127,57	48,668	127,57	0,091509	81226000	99	127,57
1	7,44E-05	146,28	123,81	111,65	0,0072682	81583000	658	111,645
0,999558	7,15E-42	258,31	246,99	160,88	-0,0060684	207470000	829	33,5797
0,999759	7,78E-05	143,97	94,616	143,97	-1,1462	27235000	302	36,1865
1	0,00168063	96,707	68,502	96,707	0,21457	7006000	1622	81,1789
0,999759	1,60E-11	199,28	178,54	73,327	-0,4891	55095000	1689	36,1808
0,962195	4,06E-11	194,7	176,17	137,61	-0,030543	88515000	1687	14,0596
1	7,50E-05	119,27	100,81	119,27	-0,066346	36268000	1728	87,3937
0,947801	0,0193957	51,03	37,021	51,03	1,0173	5553500	1801	12,6546
0,999994	0,000750425	95,755	74,455	76,01	-0,10345	14295000	1807	52,1782
1	5,69E-05	155,99	155,99	140,78	-0,066769	451190000	4738	140,78
1	4,70E-08	168,69	149,11	168,69	0,090078	95724000	2274	78,241
1	0,000103351	96,666	79,16	43,024	-0,12147	26547000	1834	43,0241
1	1,33E-08	141,08	124,2	141,08	-0,25505	125390000	1151	119,549
1	6,56E-27	206,46	158,96	44,571	0,98636	410030000	1671	44,5709

Polito et al. Universal ready-to-use immunotherapeutic approach for the treatment of cancer: expanded and activated polyclonal γδ memory T cells.
Phosphorylation sites

0,999985	0,00153097	84,896	66,861	84,896	-0,1533	24426000	1606	48,3819
1	1,50E-11	146,58	133,04	146,58	0,065963	34124000	1180	84,2041
0,999245	0,000174238	132,56	84,377	132,56	0,10921	19259000	2640	31,2156
0,999992	1,12E-09	153,59	114,3	142,32	0,85587	48708000	3130	51,4062
1	9,28E-06	139,08	123,7	139,08	-0,22892	17155000	4359	113,475
1	6,19E-10	207,42	197,88	207,42	-0,35935	1155800000	119	172,007
1	7,16E-95	334,68	303,09	219,58	0,055146	2610800000	280	219,583
1	1,98E-08	127,21	107,89	78,758	0,011345	305780000	269	69,0275
1	0,000154926	128,08	118,49	128,08	-0,41446	16789000	2	128,081
0,999968	0	558,59	507,78	446,74	-0,97035	4591300000	290	46,3434
0,999999	4,30E-143	385,91	335,26	305,87	0,065608	134960000	302	61,0099
0,999913	1,09E-08	181,58	160,85	137,89	0,22497	27416000	280	40,6246
0,765096	0,000191658	100,31	70,125	62,861	-0,47374	9111400	286	6,34694
0,992717	0,00563418	96,034	12,757	95,642	-0,049334	63693000	105	21,4772
1	2,45E-140	322	299,56	322	0,042103	664780000	32	201,2
0,99999	0,00122919	130,79	68,587	130,79	0,19862	154660000	76	50,0299
0,948379	2,74E-33	255,45	231,11	255,45	0,04048	43503000	31	12,6654
0,999532	5,08E-05	135,14	114,67	135,14	0,0078125	18246000	672	35,1465
1	0,00334142	138,06	28,584	133,6	-0,16943	393040000	361	86,5546
1	1,70E-09	157,59	125,13	128,69	-0,23955	152400000	299	87,2818
1	1,38E-50	303,29	275,32	300,71	0,34766	1220600000	445	67,1835
0,998983	8,13E-08	195,87	165,87	164,9	0,0060136	158990000	507	29,9248
1	0,000287331	125,73	37,54	86,738	0,71569	27306000	936	86,7382
1	1,80E-08	218,54	183,37	218,54	-0,10556	680230000	851	68,4565
0,999918	1,17E-09	162,65	148,54	162,65	-0,19059	22498000	844	41,018
0,801846	1,44E-30	215,68	196,19	215,68	-0,59816	31889000	153	8,13021
0,999967	4,30E-15	212,7	182,94	176,08	-0,049117	79829000	122	45,7833
0,998723	1,08E-05	124,66	78,535	122,34	-0,45352	43748000	758	31,1748
0,999986	9,38E-06	160,98	75,213	147,91	0,29851	86819000	1055	48,626
0,99986	9,92E-05	145,6	135,65	145,6	-0,18653	25146000	358	39,2783
0,862038	8,13E-07	137,69	104,5	84,718	0,22127	7304100	804	8,19507
0,999999	1,31E-10	203,29	163,22	166,03	0,24341	170920000	576	63,2949
0,999933	2,09E-08	178,02	167,66	178,02	0,15086	64305000	467	41,9438
1	2,41E-15	184,34	139,66	184,34	-0,42015	93781000	67	64,0088

Polito et al. Universal ready-to-use immunotherapeutic approach for the treatment of cancer: expanded and activated polyclonal γδ memory T cells.
Phosphorylation sites

1	0,000296654	108,06	87,97	108,06	-0,1219	18692000	810	63,6232
0,986824	3,52E-17	168,98	152,58	168,98	0,0014999	399510000	470	18,7449
1	9,54E-42	240,73	198,65	240,73	0,12621	43001000	1191	130,219
0,999989	1,15E-21	188,22	172,11	188,22	-0,35536	127450000	1124	50,1106
0,995234	0,000190981	72,421	59,654	72,421	0,55819	11645000	1049	23,1974
0,999927	0,00015564	120,09	80,172	120,09	0,66672	5845200	429	41,4351
0,983223	0,0289819	54,023	26,542	54,023	0,0019359	13760000	371	20,2624
0,999859	2,60E-07	188,96	159,49	188,96	0,065584	12534000	1011	38,5043
1	2,71E-16	137,97	127,81	137,97	-0,2029	54885000	1155	88,0286
0,997342	0,000208354	117,4	84,175	117,4	-0,47207	13965000	635	25,7433
1	0,000993841	94,409	65,453	75,509	-0,015356	97541000	225	75,509
1	0,000365587	104,44	64,791	104,44	0,022959	433800000	558	71,0793
1	7,62E-09	182,82	152,08	182,82	-0,31817	59960000	329	155,852
1	1,09E-31	231,42	202,08	231,42	-0,026949	166000000	46	80,719
1	6,14E-16	227,63	201,11	164,53	0,14004	205400000	573	84,6553
0,829068	2,90E-12	216,81	216,81	73,877	0,055049	23777000	319	6,85766
1	0,000605559	103,34	85,283	103,34	0,31861	15025000	3	98,3535
0,998147	8,43E-33	236,31	208,34	188,38	-0,084603	365660000	310	27,3342
0,981901	9,46E-33	238,83	215,35	197,15	-0,39622	120400000	311	17,344
0,999976	0,00298985	78,548	60,489	78,548	0,0086914	7232200	226	46,3891
0,998931	7,60E-23	166,49	155,03	132,48	0,2646	220060000	811	30,5397
1	0,00020931	150,35	66,568	100,38	-0,17856	58371000	104	100,376
0,995223	1,42E-39	249,47	216,32	78,451	-0,8104	32052000	90	24,5462
0,816096	0,000739608	52,483	30,262	52,483	0,51597	8852400	761	7,08716
0,99874	0,00034996	106,93	84,313	100,41	0,003068	9721900	228	30,6813
1	1,10E-30	212,77	176,1	131,4	-0,037654	227650000	363	109,565
1	1,80E-47	240,4	207,38	226,07	-0,25448	387310000	148	73,7206
1	2,68E-06	149,34	118,8	147,06	0,016069	337950000	265	64,4728
1	0,00205835	104,48	80,464	104,48	-0,19	40355000	103	84,5625
0,987884	3,42E-11	137,52	103,08	137,52	-0,96283	26382000	325	19,1813
0,981303	4,19E-12	171,09	139,26	143,39	0,3939	36917000	527	17,2012
0,999483	1,04E-06	128,41	104,11	70,98	0,0801	40222000	183	36,6335
0,995529	1,13E-06	124,98	103,68	67,364	0,40661	19855000	498	23,4762
0,999655	8,61E-09	168,1	116,83	168,1	0,11972	18495000	45	34,6219

Polito et al. Universal ready-to-use immunotherapeutic approach for the treatment of cancer: expanded and activated polyclonal γδ memory T cells.
Phosphorylation sites

1	8,05E-05	159,22	135,75	103,88	-0,17606	126590000	436	103,881
0,989433	0,0143104	62,714	19,714	62,714	0,2511	11068000	416	19,7141
1	3,91E-60	270,68	237,32	223,89	-0,38161	239470000	6	148,347
1	5,52E-126	338,06	296,03	302,49	-0,21329	1467100000	253	156,892
0,981458	0,000982152	94,616	27,535	94,616	0,050281	34205000	297	17,2372
0,826462	8,63E-10	154,13	107,71	79,177	1,6732	136430000	2444	6,77828
0,859856	2,14E-11	144,22	121,74	87,932	-0,0013628	248000000	1518	8,91047
1	1,70E-07	152,89	129,97	152,89	-0,072885	5507200	701	152,887
1	9,52E-06	175,21	147,04	175,21	-0,0071348	15471000	459	102,894
1	0,000533627	128,86	90,554	109,66	-0,072247	17803000	223	79,0998
0,996917	1,93E-14	210,37	169,14	210,37	0,38133	11552000	323	25,102
1	9,79E-06	99,508	68,397	99,508	0,54522	7701300	1582	88,8497
0,985447	5,17E-22	189,2	172,18	189,2	0,24227	43244000	208	18,3848
0,999508	7,87E-28	238,22	208,24	155,06	0,61612	40272000	255	33,078
0,999998	2,61E-06	182,89	154,68	182,89	0,11704	62364000	1412	56,7892
0,999958	4,55E-34	268,93	230,63	255,85	0,087604	183820000	1528	43,7423
0,999883	3,76E-05	169,94	122,99	157,23	-0,060639	40752000	288	40,1523
1	1,60E-21	240,65	193,22	222,73	0,15657	245990000	1564	70,3276
0,999999	0,000149344	121,02	96,678	121,02	0,10182	4917800	184	61,2215
1	3,89E-08	171,56	112,97	171,56	-0,91037	9043100	430	171,561
1	1,94E-57	253	228,93	246,55	0,22755	1292700000	448	217,398
0,954934	0,000162235	124,38	107,54	111,12	-0,08808	44949000	370	13,2628
0,858354	6,25E-30	209,43	172,35	209,43	1,0303	82289000	417	7,82841
1	0,000138968	157,91	90,387	157,91	0,17029	136120000	389	74,2729
1	8,25E-54	278,08	247,19	237	-0,31223	286710000	439	93,6147
0,999999	7,71E-06	151,26	107,97	151,26	0,047971	19243000	394	59,9435
0,942546	1,40E-06	134,02	119,09	134,02	0,10319	64002000	1144	12,1499
0,85946	0,00145383	78,809	60,945	78,486	-0,006668	35283000	33	7,86426
0,999957	3,38E-107	335,46	302,05	317	0,12515	235590000	1228	43,6681
0,894394	0,0051705	74,793	43,212	51,546	0,64207	12737000	49	12,3985
0,915702	8,42E-10	163,62	119,39	163,62	-0,4013	13170000	23	12,9336
1	0,00115222	98,048	73,05	98,048	0,12608	30042000	252	98,0483
1	2,35E-135	339,2	302,08	254,45	-0,045884	878830000	619	254,448
0,899874	0,000792151	75,819	53,133	75,819	0,31321	20561000	113	9,5345

0,999969	6,82E-49	258,34	222,99	258,34	-0,29832	189110000	21	45,6531
1	4,07E-24	276,31	200,84	276,31	-0,5517	2828300000	166	69,2711
1	1,85E-10	190,11	146,83	190,11	0,18288	12982000	12	152,08
0,829639	0,000361414	97,45	73,42	97,45	-1,1548	11022000	152	6,88905
1	7,30E-15	215,81	194,34	189,56	-0,18389	73103000	391	162,855
0,999998	5,99E-05	155,08	137,26	155,08	0,30211	26044000	255	56,9786
1	1,20E-05	112,8	102,4	85,185	-0,73828	44054000	276	85,1854
1	1,82E-05	147,72	66,624	142,89	0,10321	188700000	234	93,7059
0,999989	3,66E-60	296,46	264,39	252,37	0,51492	1080300000	340	49,4397
0,999935	0,00131267	76,588	63,175	76,588	-0,51329	34880000	214	41,8956
0,842739	4,52E-06	126,19	75,905	126,19	0,50563	23465000	284	7,95027
0,995024	0,000818882	110,53	95,383	91,658	0,073814	54345000	294	23,9543
0,999975	0,000107639	76,061	56,737	76,061	-0,11331	17003000	4	46,4247
1	3,03E-95	346,18	308,56	280,62	0,32012	1793500000	224	210,129
0,999899	6,75E-70	305,9	271,88	270,95	0,22807	273390000	43	39,9946
0,999971	4,13E-10	153,36	135,4	58,577	0,33539	86372000	101	47,633
1	7,89E-07	175,21	107,08	151,7	0,3082	814010000	84	119,572
1	9,13E-48	237,24	207,14	237,24	0,53013	219740000	20	88,7993
0,999953	4,22E-05	115,58	83,329	94,454	0,39797	29571000	281	44,6606
0,847599	0,00220507	79,337	52,188	61,212	-0,7875	7100500	187	7,45817
0,999907	2,34E-18	182,02	138,32	123,62	0,22002	153290000	73	40,3972
0,992549	3,47E-40	252,05	219,71	155,2	0,77719	154330000	298	21,3374
1	0,000100429	131,56	77,335	82,01	0,24051	265870000	312	82,0101
1	5,73E-08	187,62	103,76	82,01	0,24051	146280000	318	82,0101
1	1,33E-39	249,69	216,39	249,69	0,20269	138720000	260	94,474
0,965401	1,09E-106	331,47	293,79	240,13	-0,85804	165860000	330	14,5896
0,99995	4,65E-41	238,8	211,59	238,8	-0,14541	447180000	90	43,0246
1	0,00529778	105,52	39,02	46,73	0,15265	1034300000	10	46,7296
1	7,89E-09	176,21	153,64	80,534	-0,7859	24324000	41	80,5339
1	3,40E-05	143,64	108,12	139,58	0,97892	23930000	211	80,99
1	4,64E-14	261,22	233,96	206,45	0,048357	1047000000	403	91,6883
0,999184	2,14E-14	265,82	265,82	197,68	0,65209	114660000	409	30,9097
1	1,84E-42	266,18	214,38	101,71	2,3169	787000000	579	101,707
1	7,50E-42	255,19	229,63	255,19	-0,33134	570400000	529	89,1536

Polito et al. Universal ready-to-use immunotherapeutic approach for the treatment of cancer: expanded and activated polyclonal γδ memory T cells.
Phosphorylation sites

1	8,73E-142	380,71	339,02	380,71	-0,056599	1188200000	498	128,204
0,998764	1,39E-59	300,33	253,67	165,34	-0,089887	1152500000	453	29,0732
0,996284	2,07E-07	150,28	126,09	71,268	-0,1407	208180000	481	24,2826
1	7,51E-08	123,79	50,544	106,42	0,11075	68946000	721	106,42
1	1,06E-15	248,77	220,86	106,42	0,11075	249440000	713	106,42
1	1,45E-24	203,71	177,51	106,42	0,11075	224900000	714	106,42
1	2,11E-62	274,8	274,8	274,8	-0,23759	1582400000	702	169,421
1	1,25E-285	438,89	397,22	276,19	0,2183	5081600000	676	276,191
0,99999	1,37E-25	290,31	186,44	290,31	0,1371	622000000	387	50,0011
1	8,26E-257	469,3	437,98	342,02	0,010193	2864900000	642	215,574
0,993568	5,38E-14	185,12	164,39	185,12	-0,47686	162380000	184	21,892
1	2,75E-07	136,49	116,42	84,173	-0,23438	322730000	77	84,1734
1	0,033299	45,879	7,4768	45,879	-1,3582	2993300	88	45,8792
1	1,52E-58	266,28	234,21	266,28	0,0060173	357710000	245	124,909
1	7,34E-07	139,04	123,89	70,942	-0,12264	40896000	328	70,9421
0,998689	0,000516281	82,406	63,575	82,406	-0,38611	21532000	915	32,1806
1	1,10E-98	304,86	270,48	136,49	1,131	54768000	153	136,489
1	4,49E-128	383,24	329,76	323,02	0,080176	602210000	103	161,074
1	3,40E-08	194,67	168,31	194,23	0,10355	848280000	160	82,0752
0,876344	1,28E-06	147,1	101,23	125,5	-1,5018	12421000	2	8,5046
0,99998	8,91E-07	136,34	98,787	136,34	-0,22876	10409000	1112	46,9835
1	2,43E-15	222,45	165,51	183,6	0,17682	124270000	617	150,372
1	3,65E-182	368,07	344,09	46,39	-0,36586	765410000	227	46,3899
0,99634	0,000286531	125,74	62,524	125,74	-0,61807	16462000	171	24,3595
1	6,87E-238	376,6	376,6	376,6	-0,61355	1265300000	66	164,391
1	0,00016404	103,39	80,375	87,287	-0,051874	42316000	825	87,2873
1	9,83E-05	128,57	103,87	128,57	0,0099441	21476000	137	82,1033
0,99457	0,00203653	124,92	18,011	122,33	-0,081847	147130000	116	22,6287
0,929172	0,0241232	54,524	33,747	54,524	-0,29272	2639000	1544	11,7812
0,946529	0,0106279	46,069	31,716	46,069	0,40285	4638600	28	12,4802
0,996747	7,57E-07	186,42	99,507	168,67	-0,11163	115290000	352	25,0374
0,968301	2,22E-06	99,866	80,361	99,866	0,020401	41919000	348	14,9033
0,994302	4,82E-07	143,38	129,22	143,38	0,23921	32207000	793	22,4176
0,999973	4,17E-43	269,16	251,36	269,16	0,057902	104640000	774	45,6588

Polito et al. Universal ready-to-use immunotherapeutic approach for the treatment of cancer: expanded and activated polyclonal γδ memory T cells.
Phosphorylation sites

0,845085	0,000165216	108,59	86,964	99,86	0,15821	54017000	134	7,36806
1	1,05E-05	137,69	107,61	137,69	0,22738	36992000	146	128,632
1	0,00278414	152,54	24,156	107,21	-0,054997	243220000	747	107,205
0,999295	0,00197235	93,909	57,337	85,006	-0,21863	75531000	728	31,5159
0,997771	0,00260818	67,605	56,751	64,152	0,42519	21038000	1203	26,51
0,888804	5,48E-31	224,67	194,47	157,84	0,40469	158800000	710	8,86894
0,988415	2,18E-11	165,49	141,29	121,45	0,042414	108840000	527	20,1806
0,887059	1,95E-17	177,32	158,96	133,26	-0,048409	332330000	1645	8,95102
0,99091	0,00135166	85,554	56,596	85,554	-3,8983	15366000	1440	20,3749
0,999998	5,15E-06	164,65	86,095	131,12	0,13206	62299000	1043	56,5826
0,999842	0,00155883	81,606	58,689	71,692	-0,21693	22899000	699	38,0066
0,963343	1,07E-25	124,59	116,27	52,667	0,45216	249810000	69	14,4502
0,99999	0,00147921	85,813	56,664	85,813	0,35753	8634900	588	50,1482
1	4,40E-25	231,71	216,19	231,71	-0,2187	38839000	1719	226,227
0,99998	0,00102419	117,03	89,062	98,902	0,15843	26183000	72	46,9526
1	1,10E-17	180,86	160,78	180,86	-0,47661	24270000	177	151,517
1	3,08E-157	369,64	340,47	333,14	-0,2964	1088600000	364	175,06
1	0,000534175	115,92	102,41	55,98	0,16275	57912000	6	55,9796
0,998179	1,98E-07	187,33	148,11	171,87	0,067119	240500000	203	27,3987
0,974021	3,48E-12	216,34	168,78	198,74	-0,19909	331100000	188	15,7395
0,962523	6,32E-18	181,26	162,18	181,26	0,36669	19210000	159	14,0978
1	1,23E-27	216,44	190,32	216,44	-0,51476	169540000	375	125,087
1	5,77E-05	134,57	89,198	134,57	0,61202	125830000	38	108,182
0,999721	6,45E-06	128,75	109,03	110,52	-0,55598	56704000	340	35,673
0,999998	0,00984719	62,408	14,849	62,408	0,4563	8447900	670	56,1138
1	2,30E-10	157,96	134,05	157,96	0,20649	44127000	13	71,8338
0,808727	3,80E-06	177,04	161,89	177,04	0,2567	33667000	226	6,26148
0,938822	6,36E-14	207,85	163,55	156	0,053059	38253000	472	12,0307
1	1,08E-12	218,28	218,28	167,61	-0,28494	96845000	6	78,1068
1	0,000134234	136,5	111,65	117,7	-0,37522	31705000	267	117,704
0,982407	0,000125108	131,69	100,62	115,57	-0,065472	15553000	188	17,4695
0,999999	4,18E-30	207,48	190,45	207,48	-0,021738	631090000	19	62,2886
0,990672	9,34E-06	115,06	104,05	115,06	0,19664	29932000	23	20,2908
0,999938	2,55E-06	164,48	110,73	140,24	-0,28238	166680000	119	42,0875

Polito et al. Universal ready-to-use immunotherapeutic approach for the treatment of cancer: expanded and activated polyclonal γδ memory T cells.
Phosphorylation sites

0,756311	0,000798647	91,28	73,586	41,644	0,021308	45328000	279	7,89937
0,999956	4,58E-11	213,59	213,59	173,19	-0,071017	582030000	66	44,5126
0,999997	1,60E-08	220,37	174,49	220,37	0,085512	1587500000	9	55,4756
1	1,45E-214	368,62	355,47	46,449	4,6729	401590000	942	46,449
0,937943	5,02E-07	131,65	116,5	124,2	0,45446	104840000	430	12,2978
0,999648	0,00105931	97,69	42,228	84,892	-0,079372	60308000	322	34,5382
1	2,84E-12	169,98	132,43	169,98	0,1234	148900000	485	101,725
0,990067	1,11E-105	325,35	295,62	250,86	-0,056841	2729800000	494	20,8992
1	1,48E-06	203,02	177,79	99,755	0,035178	922840000	513	99,7553
1	1,35E-140	369,95	319,12	223,93	-0,085886	2407100000	451	131,495
1	1,35E-140	369,95	319,12	223,93	-0,085886	2370800000	453	96,3483
0,999998	3,12E-31	229,78	197,43	223,93	-0,085886	406070000	455	57,6589
1	7,96E-32	218,67	202,89	196,15	0,25103	362050000	21	184,917
0,999985	0,00963362	67,563	37,753	67,563	-0,1641	10588000	123	48,2918
1	0,000823522	173,48	87,38	80,787	-0,54551	332990000	1117	80,7875
0,867123	1,49E-47	242,26	219,94	242,26	-0,0019228	115270000	469	8,1463
0,847793	5,40E-70	282,1	262	147,12	0,65099	243670000	470	7,45881
0,949323	0,000142021	78,758	53,552	78,758	-0,02636	79916000	1126	12,7413
0,998524	2,98E-06	178	135,74	178	0,3041	37491000	601	28,3926
0,999864	2,16E-15	176,87	157	136,89	-0,18973	101700000	76	38,6531
0,995791	3,67E-21	233,59	182,76	233,59	-0,0014118	216690000	432	23,7399
0,973029	1,60E-05	117,4	97,561	117,4	-0,2358	18136000	343	16,0144
0,998305	1,23E-05	156,58	70,774	156,58	-0,17376	45901000	1215	28,5465
1	4,05E-37	257,35	229,26	182,57	0,8412	160770000	1149	161,256
0,999999	5,97E-07	172,27	86,742	172,27	0,13039	42416000	1167	59,9175
0,999998	0,00204746	125,47	46,699	123,21	0,19983	112800000	1197	57,7427
1	7,96E-96	334,73	304,08	41,348	1,2196	200510000	300	41,3476
0,999923	0,000155674	200,06	108,97	200,06	-0,0093807	543380000	288	41,1193
0,861616	1,26E-07	165,02	113,93	165,02	-0,32622	124030000	243	8,51946
0,919364	3,51E-05	134,37	105,42	134,37	0,2764	15416000	624	10,6157
0,992201	0,0085836	71,491	19,828	71,491	0,0098263	12699000	1229	21,6577
1	2,62E-05	161,21	140,78	161,21	0,21918	34227000	681	89,0052
1	1,47E-05	163,9	96,172	87,083	0,35365	49789000	705	87,0829
1	1,42E-49	267,38	244,05	267,38	-0,16106	938600000	882	82,3899

0,999951	5,53E-05	93,754	70,952	93,754	0,26498	21749000	474	43,1461
0,999506	4,65E-25	221,86	187,25	195,13	-0,27192	395930000	539	33,4469
1	6,40E-05	161,19	132,46	161,19	0,029379	165480000	899	119,568
0,987046	6,40E-05	161,19	132,46	161,19	0,029379	50058000	905	20,0613
1	0,000336177	111,6	63,069	111,6	0,20189	38856000	625	111,595
1	9,37E-06	134,88	109,32	134,88	-0,0077428	343680000	290	74,5372
1	9,66E-08	196,84	125,69	182,89	0,17973	72663000	132	74,1701
0,984856	2,61E-39	241,57	193,89	91,175	0,081311	1175400000	758	18,7498
1	3,54E-08	172,82	115,02	172,82	0,15281	224120000	108	79,3416
0,977525	8,50E-06	106,73	77,929	106,73	-1,1105	14068000	664	17,0664
0,876191	0,000366867	84,41	49,522	84,41	-0,67503	7567000	262	8,49903
0,999984	6,18E-06	118,15	99,848	67,358	-1,3194	76955000	2150	49,4414
1	7,71E-15	197,72	185,77	58,672	-0,26051	325970000	277	58,6723
0,958565	6,62E-06	72,292	62,748	72,292	-0,65582	42084000	385	14,5117
1	4,00E-34	217,4	198,49	217,4	-0,033037	1669500000	102	64,0095
0,999975	5,15E-06	173,72	120,15	173,72	0,082534	33403000	407	46,003
1	7,14E-58	264,21	239,73	264,21	0,042289	457890000	169	106,592
0,999824	1,13E-195	400,22	372,23	396,05	-0,02717	763250000	172	37,5592
0,976669	0,00532946	123,79	40,586	123,79	0,22153	27558000	2143	16,218
1	6,41E-12	204,52	181,38	204,52	0,21989	229300000	1472	138,301
0,999786	1,57E-84	303,23	287,98	125,09	0,16838	146270000	2436	36,6949
0,998511	0,000707191	91,812	54,386	85,42	0,069061	35557000	1450	28,2843
0,958766	8,78E-15	215,85	194,39	139,69	-0,11519	147590000	1196	13,6698
0,999999	2,10E-83	341,27	313,31	341,27	-0,4383	558880000	2184	59,7652
1	0,000167872	133,42	77,06	63,864	-0,031775	170430000	2120	63,8636
1	4,96E-147	338,12	321,35	89,266	0,60346	189710000	224	89,2663
1	9,19E-99	305,7	284,96	305,7	0,14612	334520000	2151	166,022
1	0,000474654	103,21	81,127	103,21	-0,089352	37192000	1242	71,2242
0,999988	4,22E-99	308,97	258,88	303,23	0,85431	283710000	1977	47,1219
0,977078	0,000282562	113,52	81,512	113,52	0,35114	8101700	17	16,2968
1	1,22E-129	387,38	346,1	119,27	0,1925	2417800000	30	119,269
0,909668	0,000223366	120,53	87,92	76,465	0,21657	28436000	623	10,0304
0,771178	0,0348093	43,68	17,078	43,68	-0,70445	4042500	469	5,71165
0,999078	2,27E-47	237,12	229,1	237,12	-0,073753	18067000	158	30,3473

Polito et al. Universal ready-to-use immunotherapeutic approach for the treatment of cancer: expanded and activated polyclonal γδ memory T cells.
Phosphorylation sites

0,775794	0,000355339	121,29	95,989	54,259	-0,37084	13068000	175	6,95742
0,999996	6,40E-10	159,98	145,3	132,14	0,26728	52959000	168	53,6305
1	1,32E-58	308,04	272,39	308,04	0,11895	540950000	137	100,637
0,976816	9,97E-06	94,856	77,977	86,479	0,48397	74002000	176	16,7422
0,999823	7,08E-22	202,07	178,93	202,07	-0,23057	104640000	911	37,5393
1	0,00190783	110,97	7,2108	110,97	-0,87956	12663000	572	110,972
1	3,01E-25	256,96	220,28	127,49	0,16626	138410000	861	127,494
0,9614	0,000293901	80,609	59,656	51,265	0,48631	7060700	24	18,2701
0,999998	1,68E-17	174,47	152,03	174,47	-0,79077	46987000	406	57,9861
0,999853	0,000551254	106,62	55,182	70,942	-0,14247	109420000	638	38,6122
1	8,28E-06	123,14	102,76	114,99	-0,0039019	42085000	538	114,986
0,997628	1,66E-54	227,22	201,28	173,28	0,2404	946350000	25	26,9825
1	0,000166602	121,28	87,101	121,28	-0,1361	184050000	432	121,28
1	3,71E-10	166,65	132	158,37	-0,36185	49215000	380	140,376
0,939676	0,000542093	83,877	65,281	83,877	0,42408	12403000	315	11,9435
1	5,42E-68	244,62	203,43	185,13	-0,62299	1132900000	131	150,3
1	3,52E-84	333,08	291,77	316,56	0,31408	600010000	129	113,296
0,774847	3,95E-15	154,71	144	44,426	-0,81329	24020000	116	5,65722
0,992863	6,95E-10	156,92	131,7	142,1	-0,36804	82033000	206	21,4354
[truncated: 431,268 more chars]
